# Supplementary material for: A roadmap for ribosome assembly in human mitochondria
Source: Nat Struct Mol Biol. 2024 Jul 11;31(12):1898–908. doi: 10.1038/s41594-024-01356-w (PMC11638073; doi:10.1038/s41594-024-01356-w)
Supplement: Supplementary file 1 — Supplementary Figs. 1–15 and Tables 7–12. [file 41594_2024_1356_MOESM1_ESM.pdf]

nature structural & molecular biology

Supplementary information https://doi.org/10.1038/s41594-024-01356-w

A roadmap for ribosome assembly in human mitochondria

In the format provided by the authors and unedited

Supplementary information

Supplementary Figures

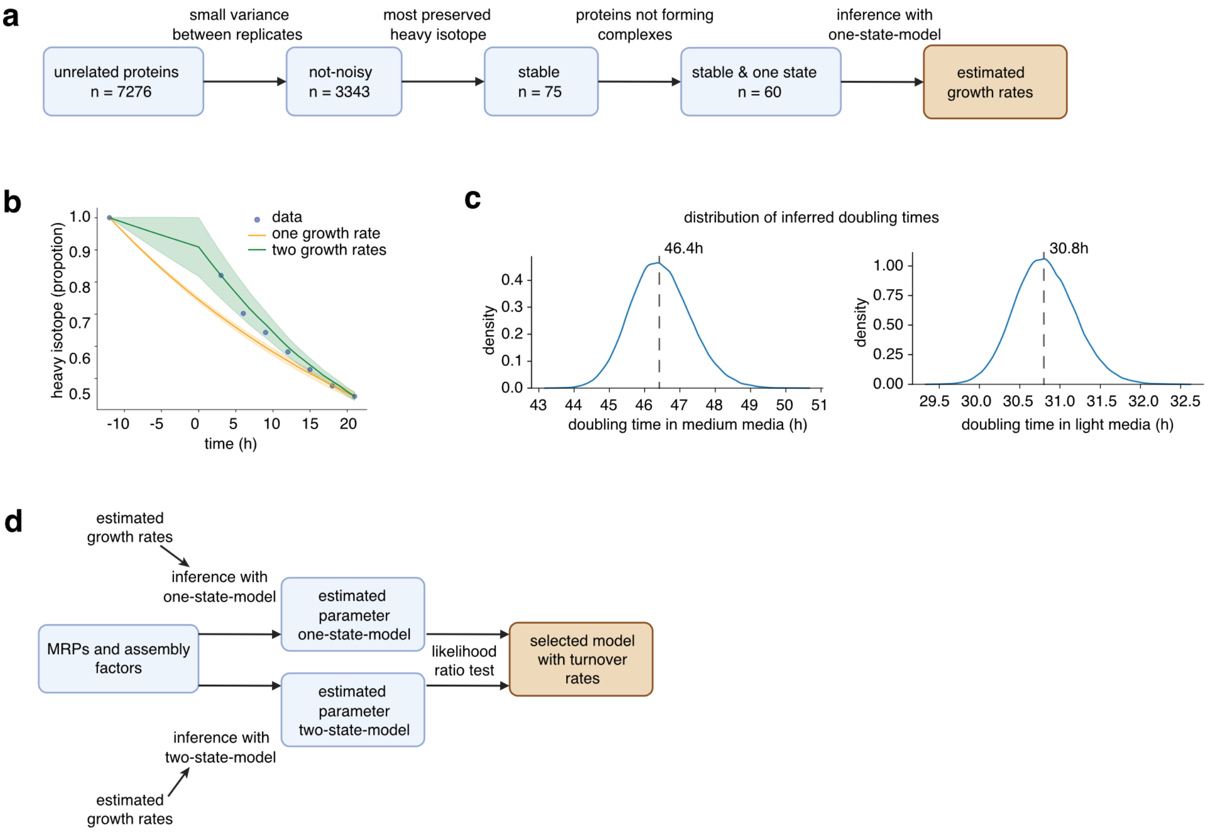

Supplementary Fig.1|Workflow for inference of global intracellular turnover of MRPs and mitoribosome assembly factors. a, Estimation of the cell growth rate. Unrelated proteins, *i.e.* proteins that are not MRPs or their assembly factors, were filtered by variance between replicates, value of "heavy" isotope in the late time-points (18 and 21 h) and complex formation to get stable one state proteins with reliable data. These proteins were model using one-state-model to infer cell growth rate in the "medium" and in the "light" media. **b,** Medians of the "heavy" isotope derived from selected proteins in (a, indicated as dots) compared to inference results using one growth rate (based on 21 h time point) and using two growth rates (based on 3 and 21 h time point). Solid lines indicate median and shaded areas indicate 5%-ile and 95%-ile of model fits. **c,** Distribution of inferred cell doubling times (directly related to the cell growth rates). Vertical line represents the median of the distribution, value used for the subsequent modelling of MRPs and assembly factors. **d,** Schema of modelling of MRPs and assembly factors. Proteins were modelled with both one-state-model and two-state-model using the previously inferred cell growth rates. Likelihood ratio test was used to select the more likely model for every protein.

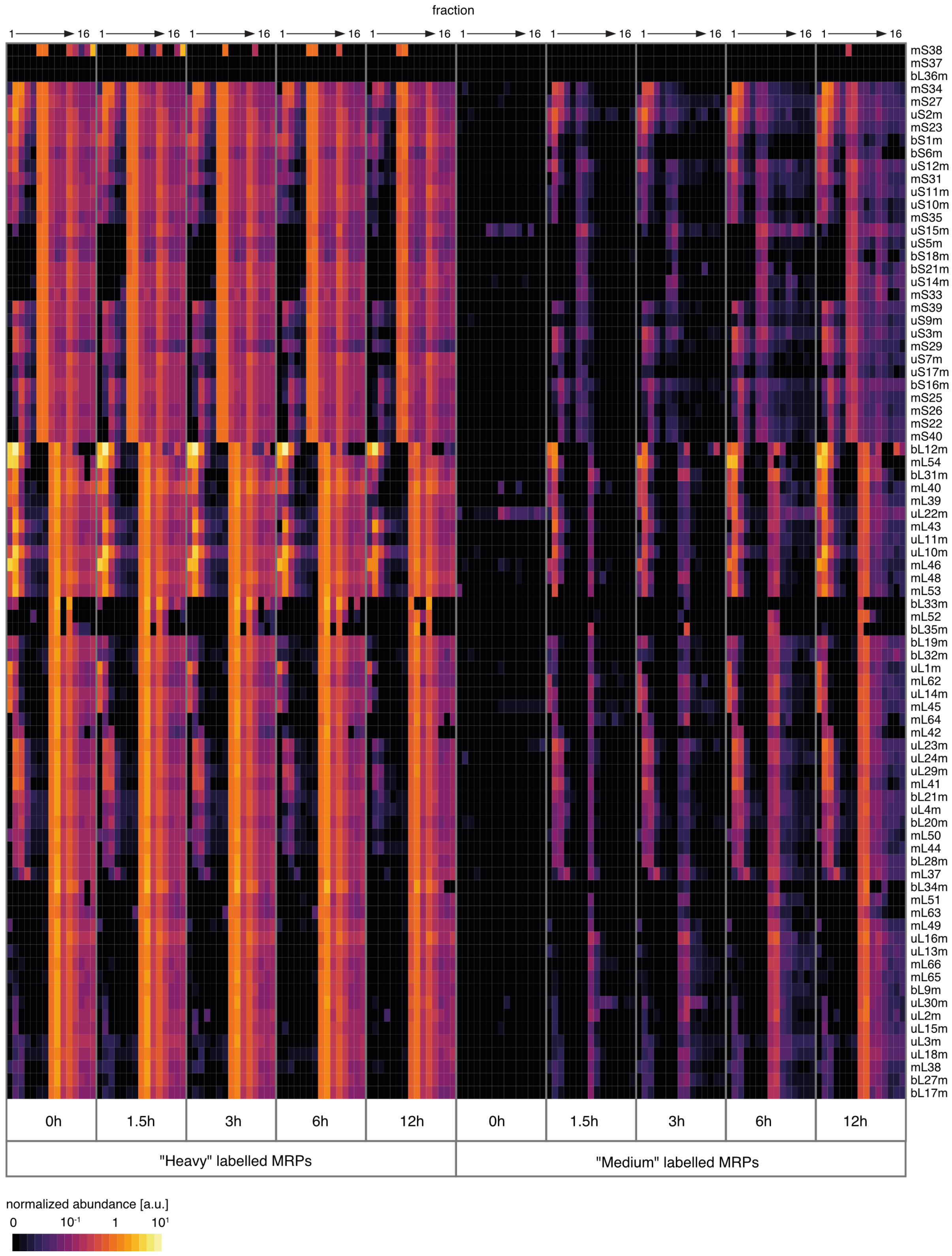

Supplementary Fig.2|Normalized abundance of "heavy" and "medium" labelled MRP across sucrose gradient fractions for five collected time points (0 h-12 h). The abundance of "heavy" and "medium" labelled MRPs is indicated as a range from black (zero) to light yellow (maximal value). MRPs are arranged based on hierarchical clustering of "heavy" and "medium" labelled protein abundances across all sucrose gradient fractions and time points.

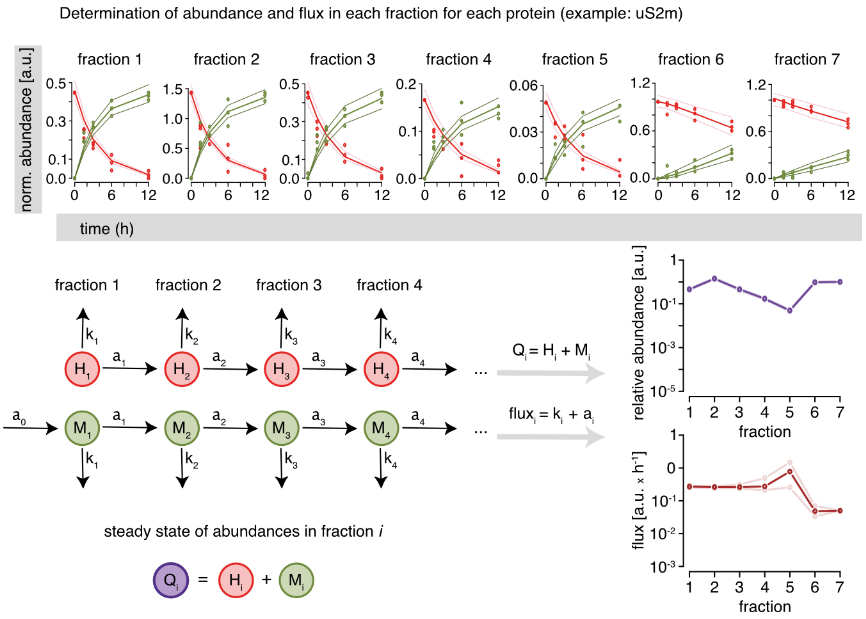

Supplementary Fig.3| Inference of steady state abundance and fluxes. As an example, normalized abundance of uS2m is depicted for fractions 1-7 over time. Dots represent individual data points of the biological replicates and lines represent median and confidence ranges of flux model fits, where "heavy" labelled uS2m is depicted in red and "medium" labelled uS2m is depicted in green. The flux model describes the turnover (*k*) of MRPs in each fraction as well as the MRP transfer across fractions (*a*). H = "heavy", M = "medium", Q = steady state abundance, a = integration rate, k = turnover rate.

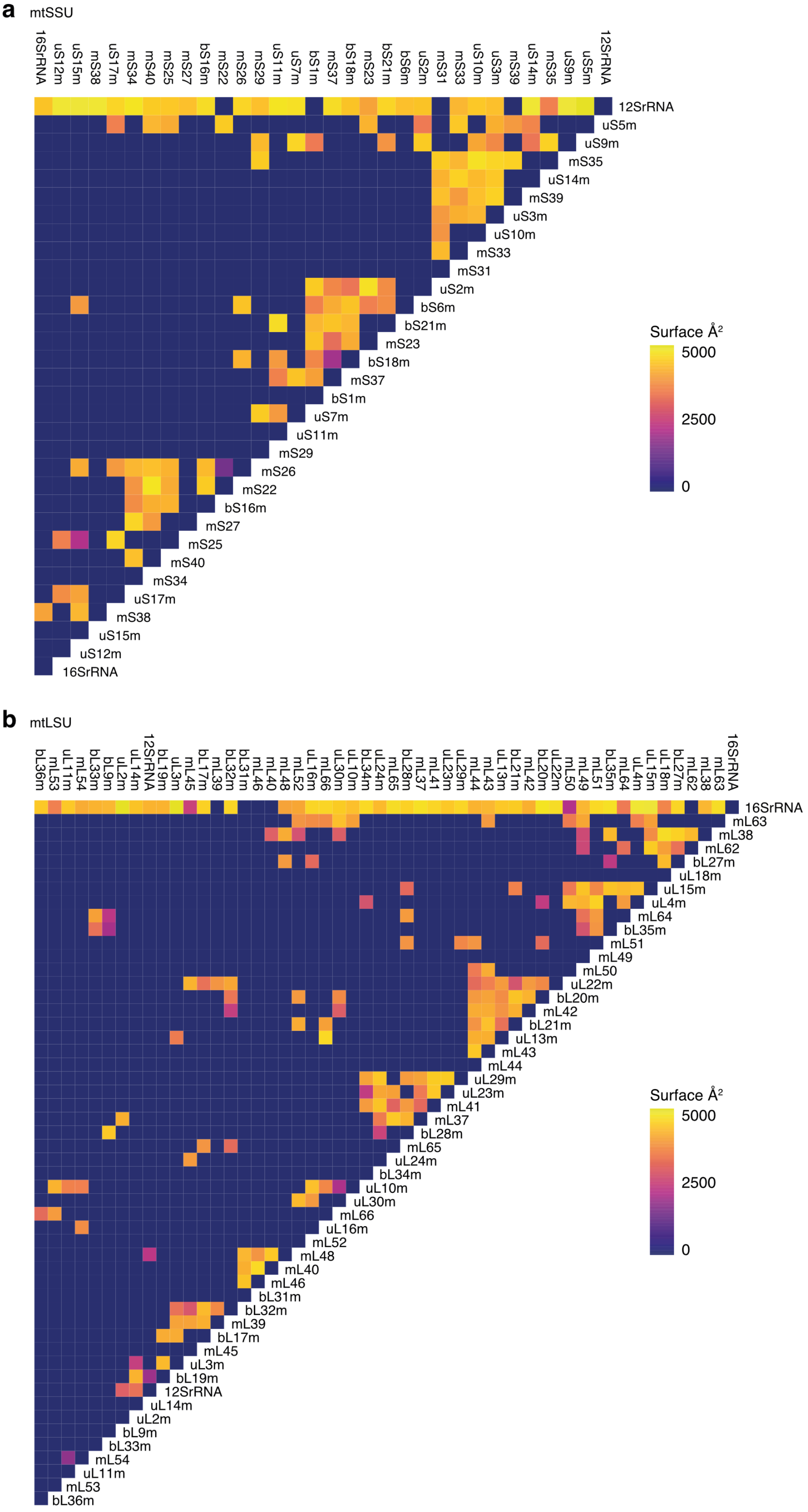

Supplementary Fig.4|Contact matrix of the mtLSU (a) and mtSSU (b). Contact matrix shows the pairwise contact surface area between two MRPs, as well as between MRPs and RNAs, computed based on the structure PDB 6zm6 using the software PDBePISA. The contact surface area of MRPs is indicated as a range from blue (minimal value) to light yellow (maximal value).

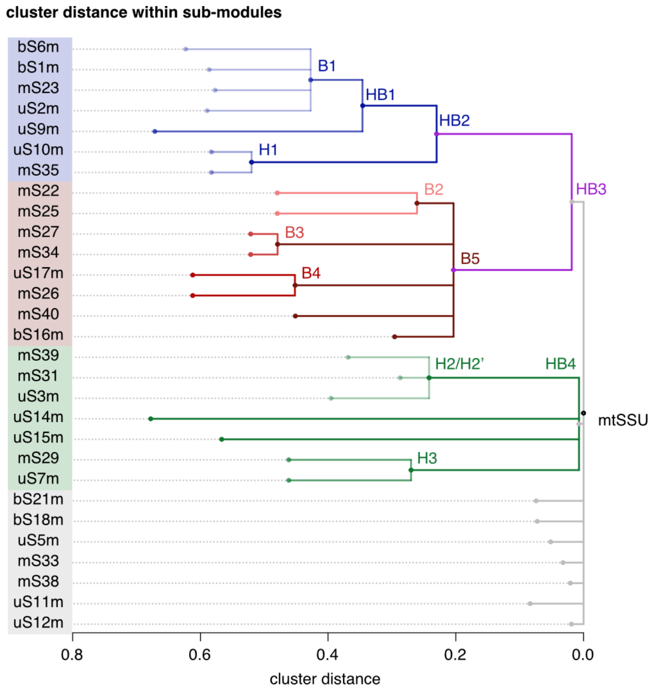

Supplementary Fig.5|Assembly module similarity of mtSSU. Dendrogram illustrates the mtSSU assembly pathway, where the distance between a MRP node and a module node indicates the distance between the flux and abundance of the MRP compared to the mean of the fluxes and abundances of all MRPs assigned to the module. Hence, the larger the distance, *d*, the more divergent was the MRP from its assigned module. Accordingly, the distance between two module nodes, indicates the distance between the average flux and abundance of the smaller module compared to the average of the fluxes and abundances of the larger module.

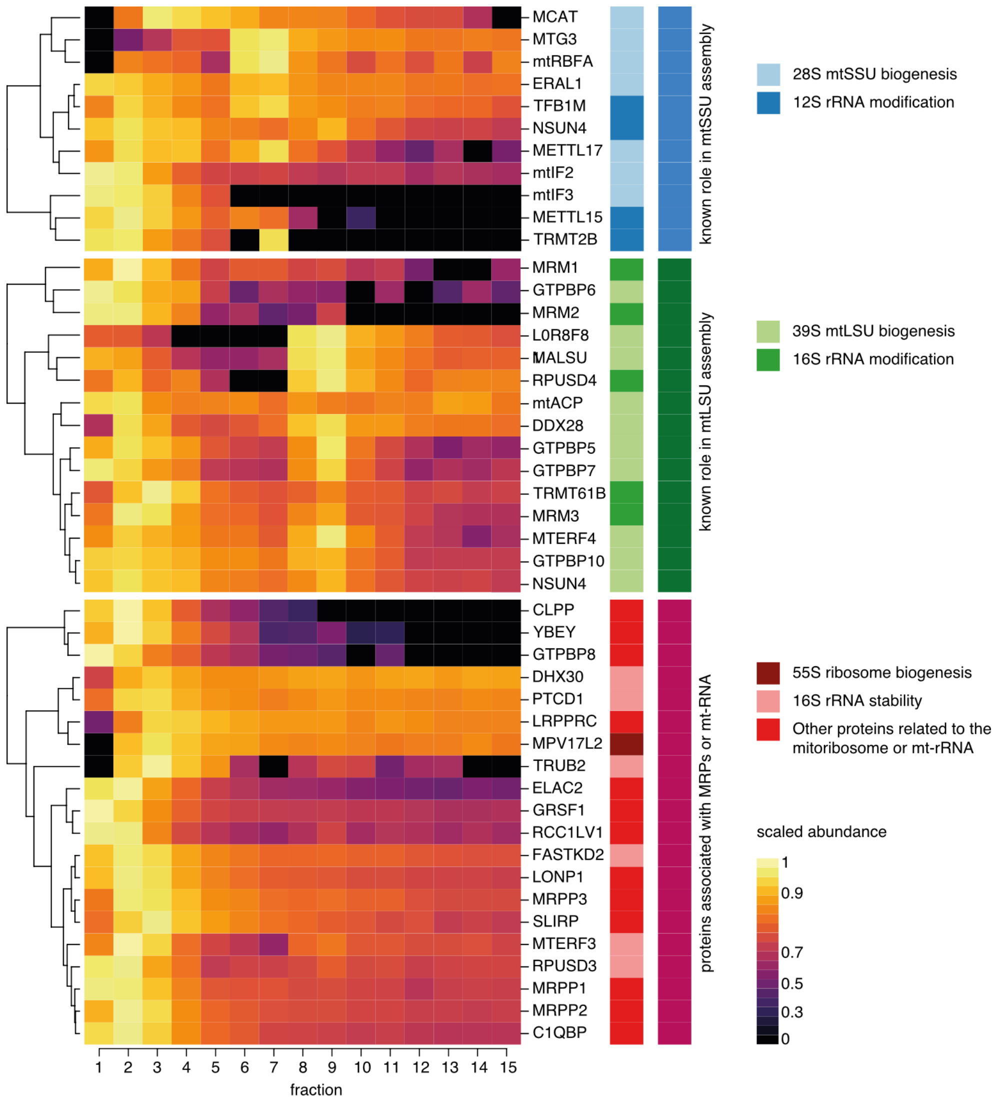

Supplementary Fig.6|Abundance of assembly factors and mitoribosome-associated proteins across gradient fractions. Proteins are grouped based on their known function into “known role in mtSSU assembly”, “known role in mtLSU assembly” and “proteins associated with MRPs or mtRNA”. Within each group, proteins are arranged based on hierarchical clustering of scaled abundances across all sucrose gradient fractions.

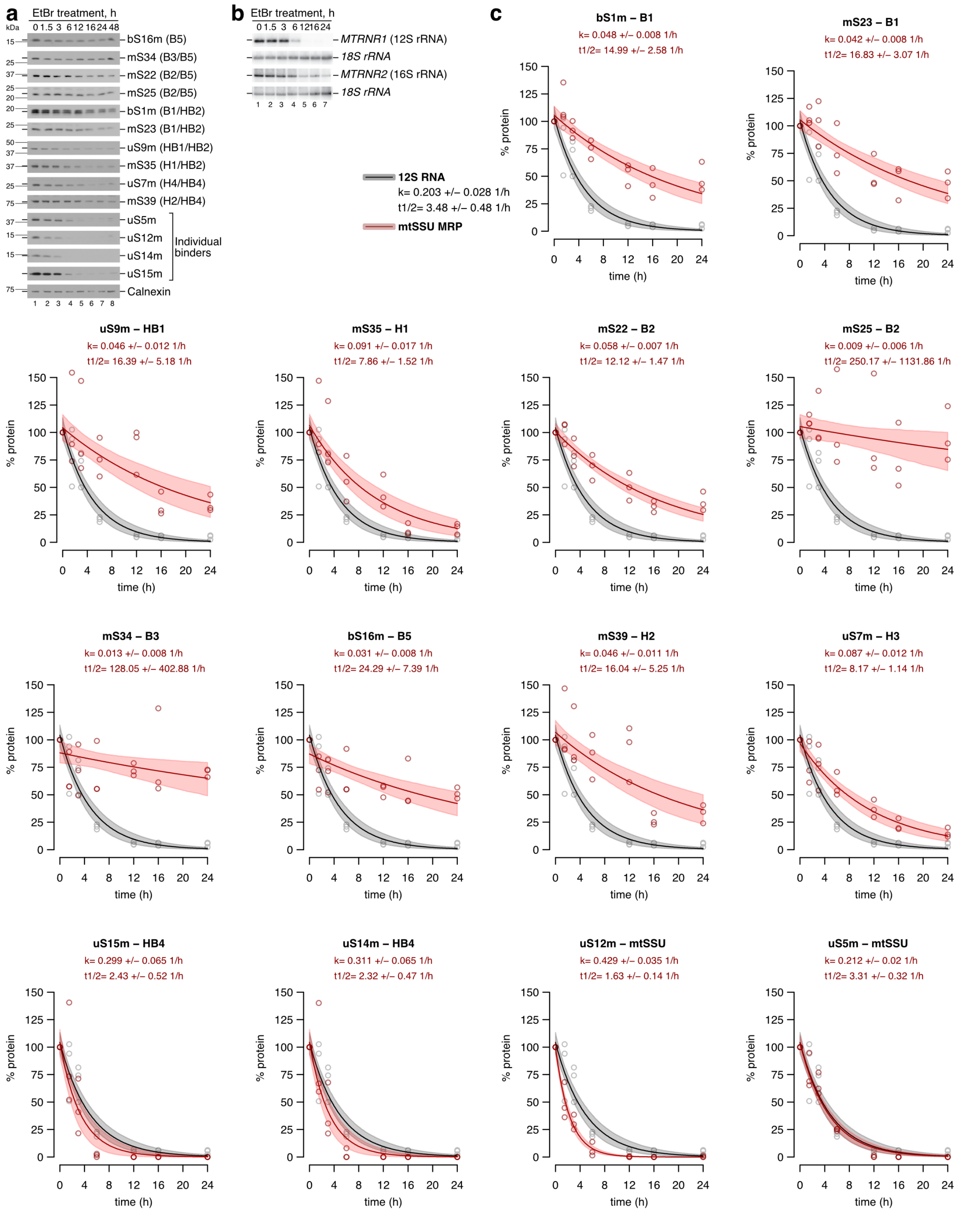

Supplementary Fig.7|Formation of assembly modules is independent of the presence of rRNA – inference of decay rates. **a**, Western blot analysis of the MRPs stability upon mt-rRNA depletion. HEK293 WT cells were induced with 0.25 µg/mL of ethidium bromide and harvested after indicated time intervals. MRPs were detected using indicated antibodies. Calnexin was used as a loading control. **b**, Monitoring of the mt-rRNA decay upon inhibition of mitochondrial transcription. Cells were treated according to the scheme presented in a. Total RNA was isolated and the presence of mt-rRNAs were detected by northern blot with indicated probes. 18S cytosolic ribosomal rRNA was used as a loading control. **c**, MRPs decay rate upon repression of mt-rRNA synthesis by ethidium bromide. Plotted are relative MRP (red) and 12S rRNA (grey) abundance at indicated time points post-treatment as percentage of the starting abundance (time point 0 h). Exponential decay model was employed to

determine decay rates (*k*) and half-life (*t1/2*) of MRPs and 12S RNA. Solid lines and shaded areas indicate the median and confidence ranges of the model fits, respectively. The title of each panel indicates MRP name and its corresponding first mtSSU module it associates with.

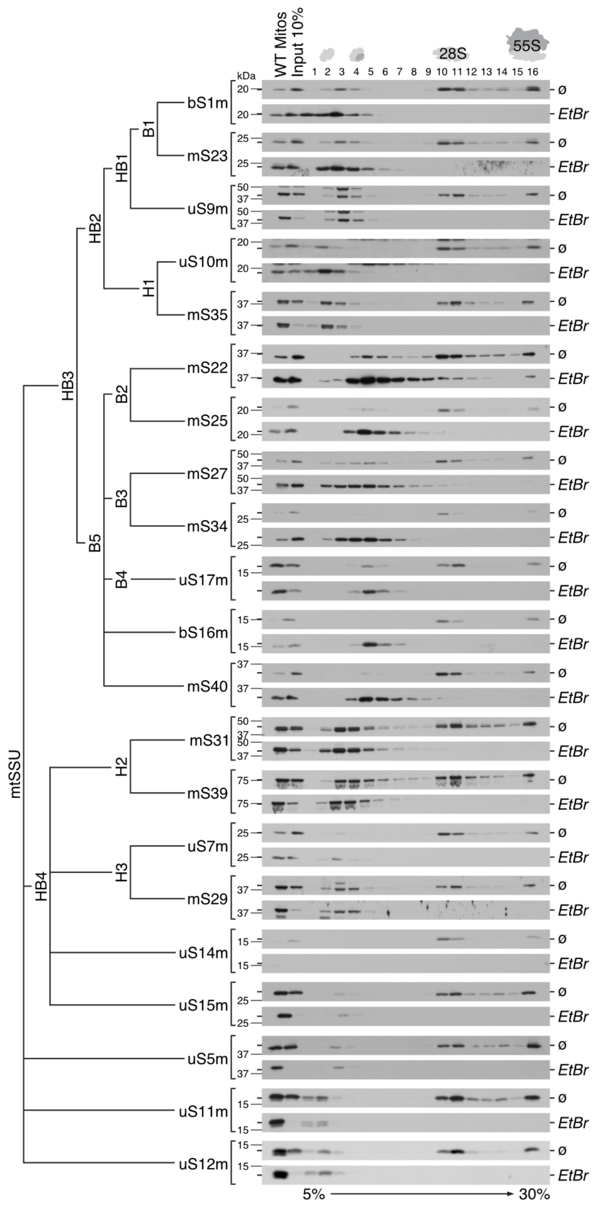

Supplementary Fig.8|Formation of the mtSSU assembly clusters upon 12S mt-rRNA depletion. Mitoribosomal complexes isolated from ethidium bromide-treated (EtBr) or untreated cells (Ø) were separated by sucrose gradient ultracentrifugation. H – mtSSU head; B – mtSSU body; HB – mtSSU head-body assembly module.

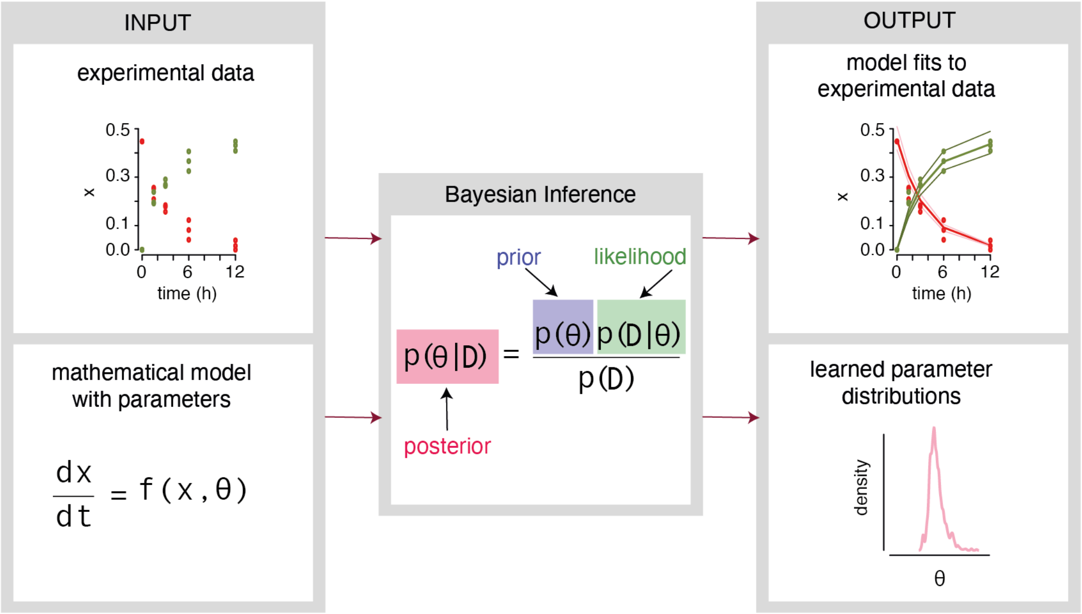

Supplementary Fig.9|Bayesian inference schematic. In order to derive kinetic parameter estimates (posterior distribution) of a mathematical model describing experimental data (input), Bayesian parameter inference can be employed. Here, the initial best guess of the parameters (prior distribution) is updated via the likelihood, which combines the data with the mathematical model, resulting in the posterior parameter distribution. Successful inference results in good agreement between model simulations and experimental data.

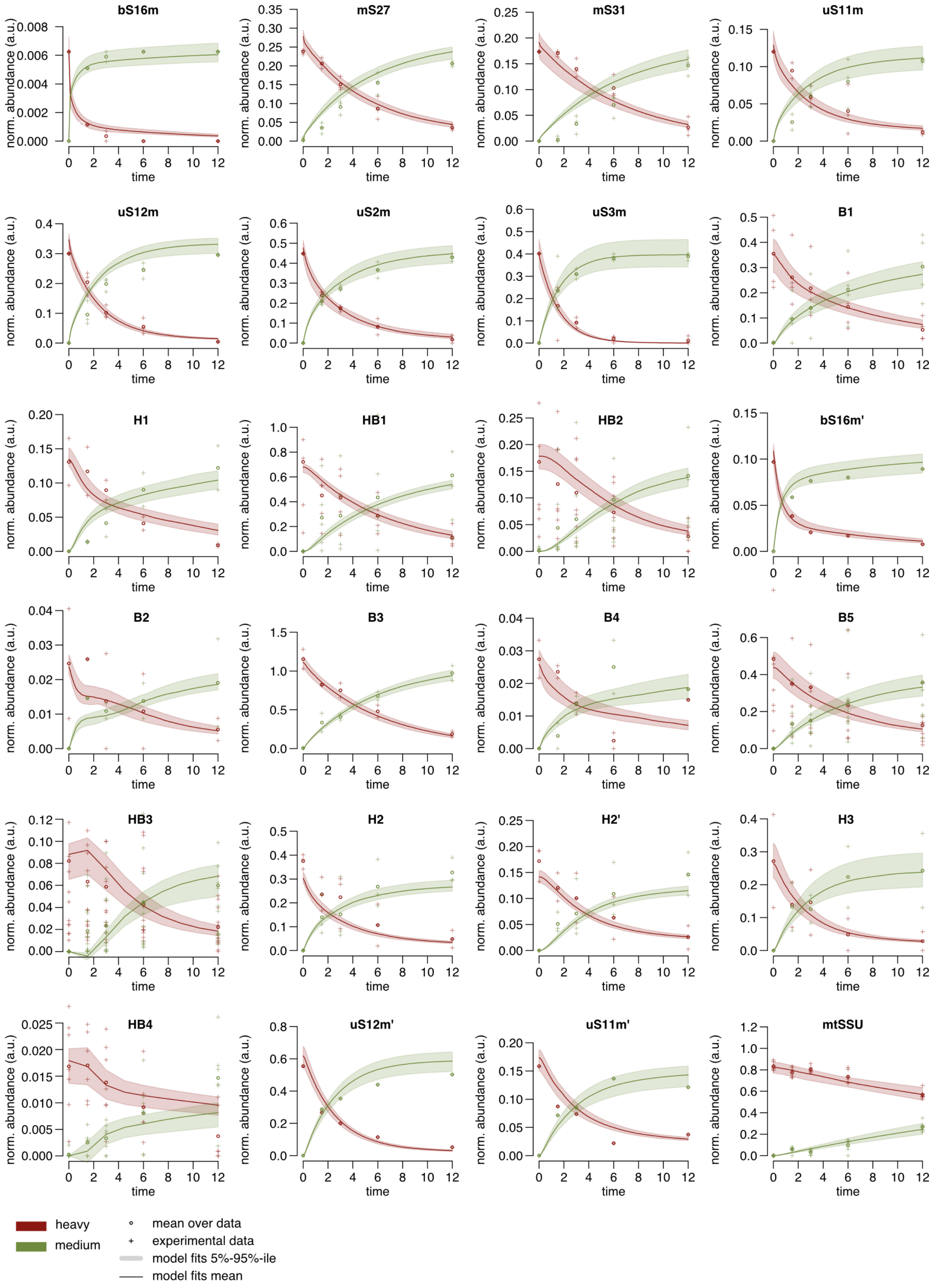

Supplementary Fig.10|Experimental data and model fits for mtSSU assembly. Shown is normalized abundance of "heavy" and "medium" labelled MRPs and mtSSU complexes (n=3 biological replicates). Model fits are obtained from posterior sample upon Bayesian inference. Solid lines indicate median and shaded areas indicate 5%-ile and 95%-ile of model fits.

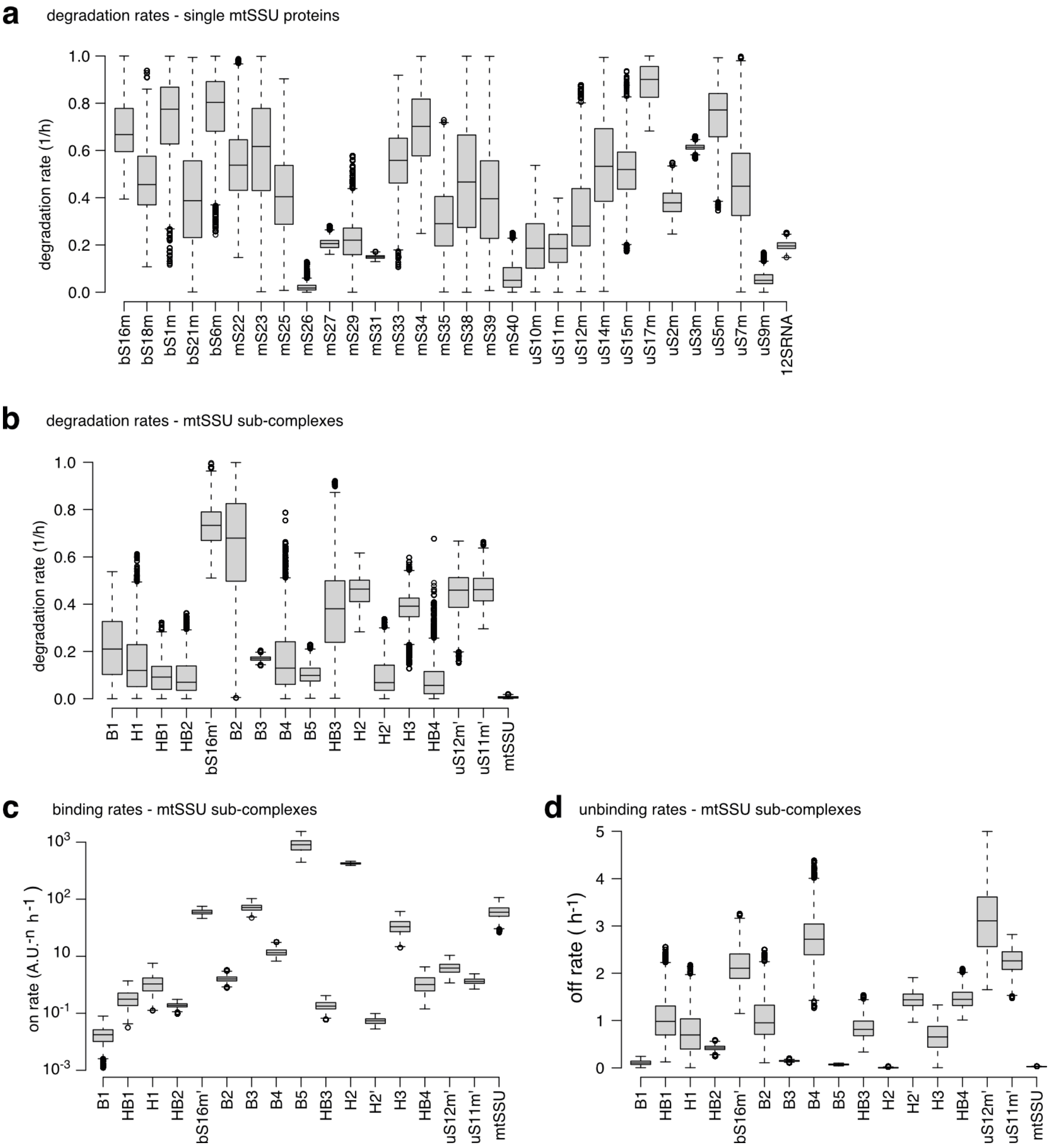

Supplementary Fig.11| Kinetic rates of mtSSU assembly

a-d, Inferred kinetic rates of mtSSU assembly. Boxplots indicate median, 1st quartile, 3rd quartile, as well as minimum and maximum after outlier removal over the respective marginal posterior parameter distribution inferred based on n=3 biological replicates. Outliers are indicated as dots. In (c) n indicates the order of the reaction (i.e. number of binding partners) -1.

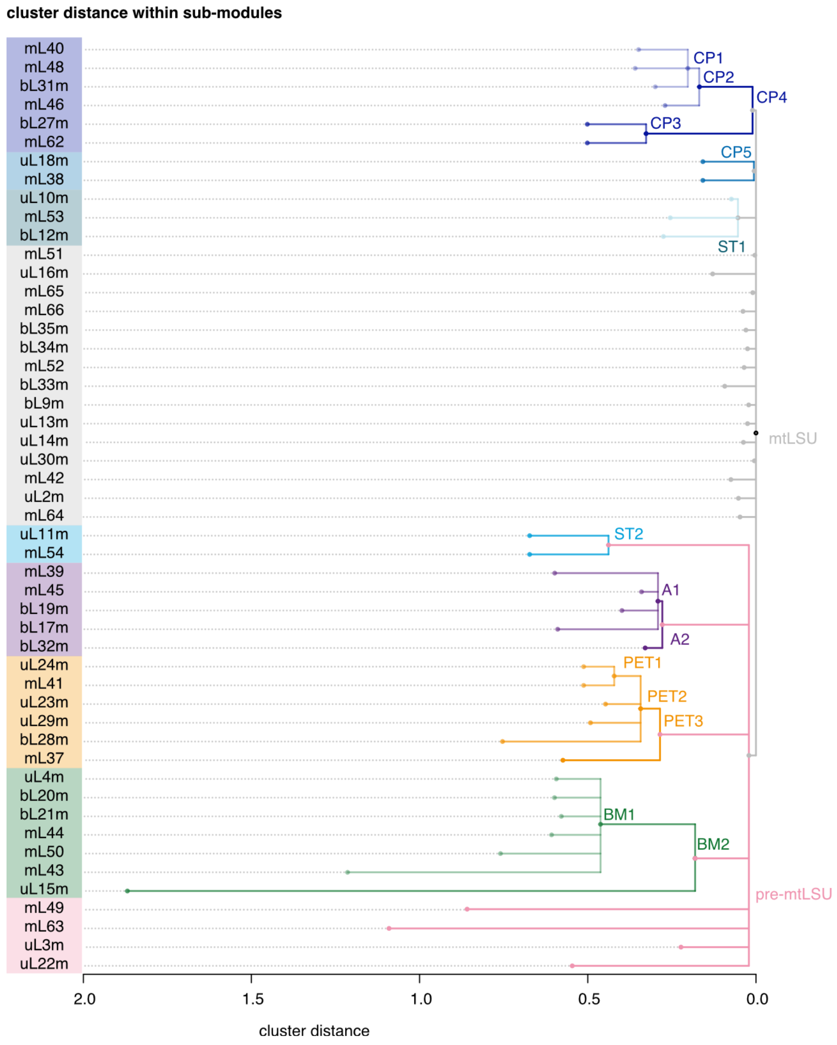

Supplementary Fig.12| Assembly module similarity of mtLSU. Dendrogram illustrates the mtLSU assembly pathway, where the distance between a MRP node and a module node indicates the distance between the flux and abundance of the MRP compared to the mean of the fluxes and abundances of all MRPs assigned to the module. Hence, the larger the distance, *d*, the more divergent was the MRP from its assigned module. Accordingly, the distance between two module nodes, indicates the distance between the average flux and abundance of the smaller module compared to the average of the fluxes and abundances of the larger module.

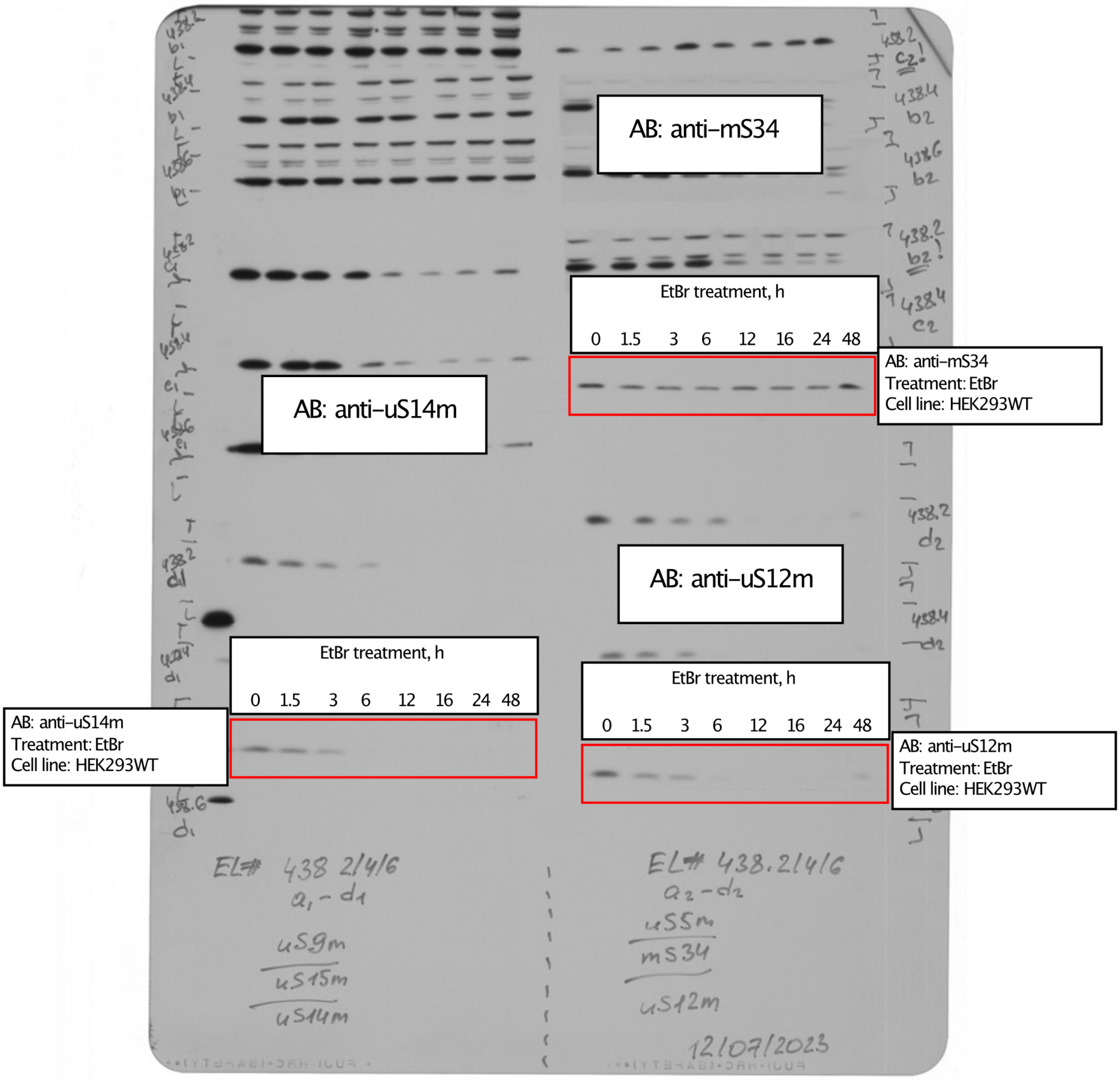

Supplementary Fig.13| Source Data for Supplementary Figure 7a

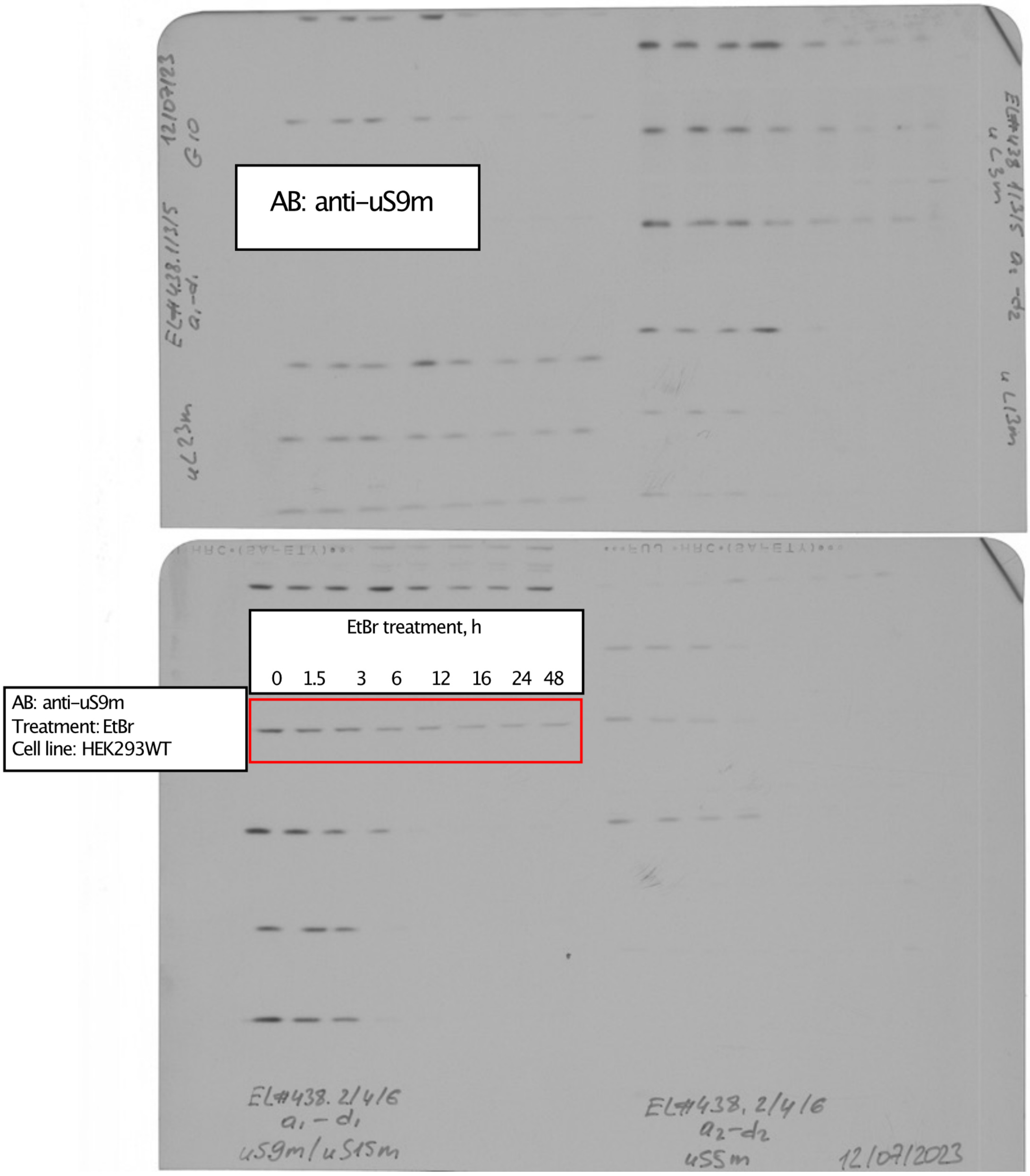

Supplementary Fig.13| Source Data for Supplementary Figure 7a (continuation)

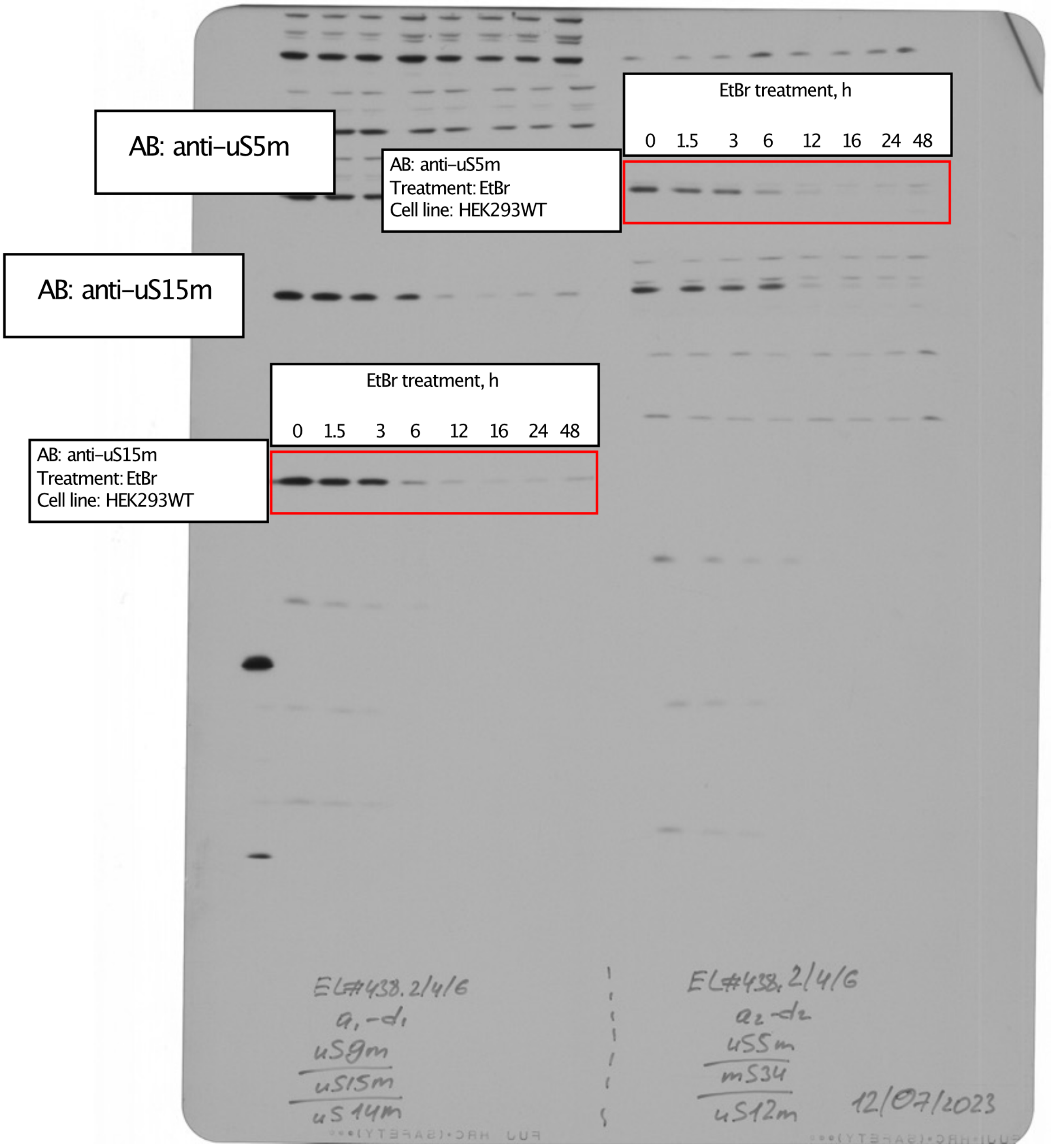

Supplementary Fig.13| Source Data for Supplementary Figure 7a (continuation)

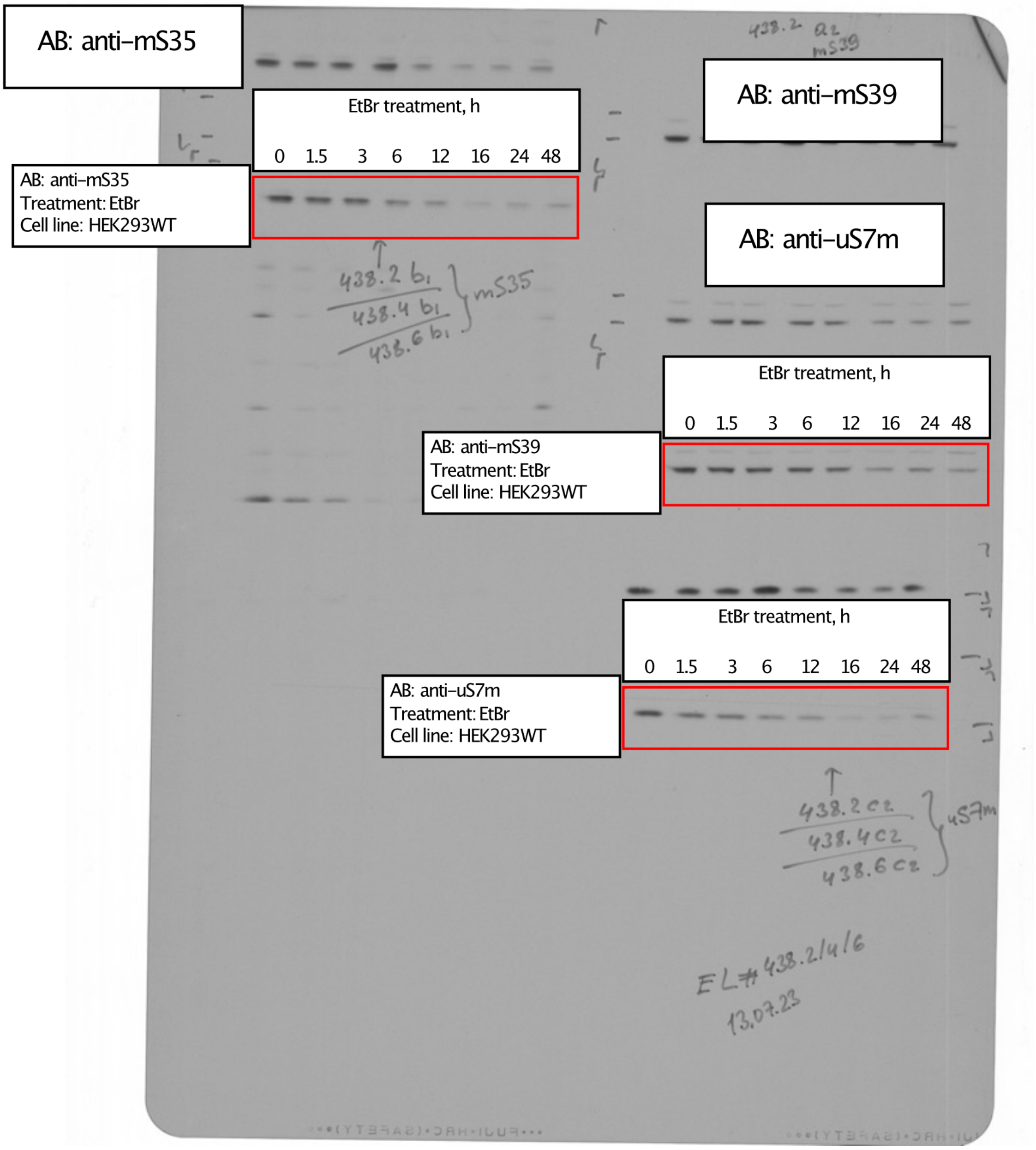

Supplementary Fig.13| Source Data for Supplementary Figure 7a (continuation)

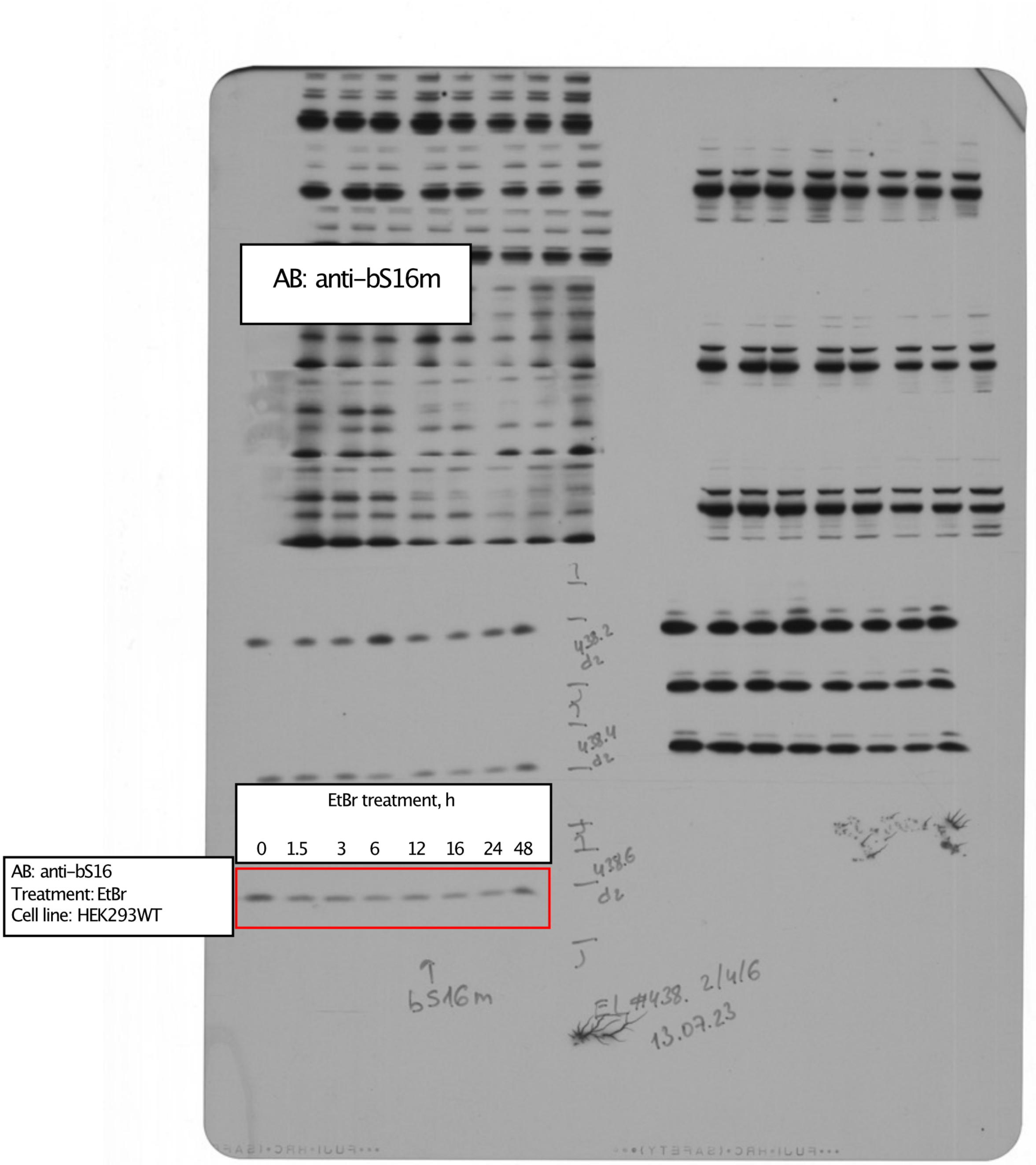

Supplementary Fig.13| Source Data for Supplementary Figure 7a (continuation)

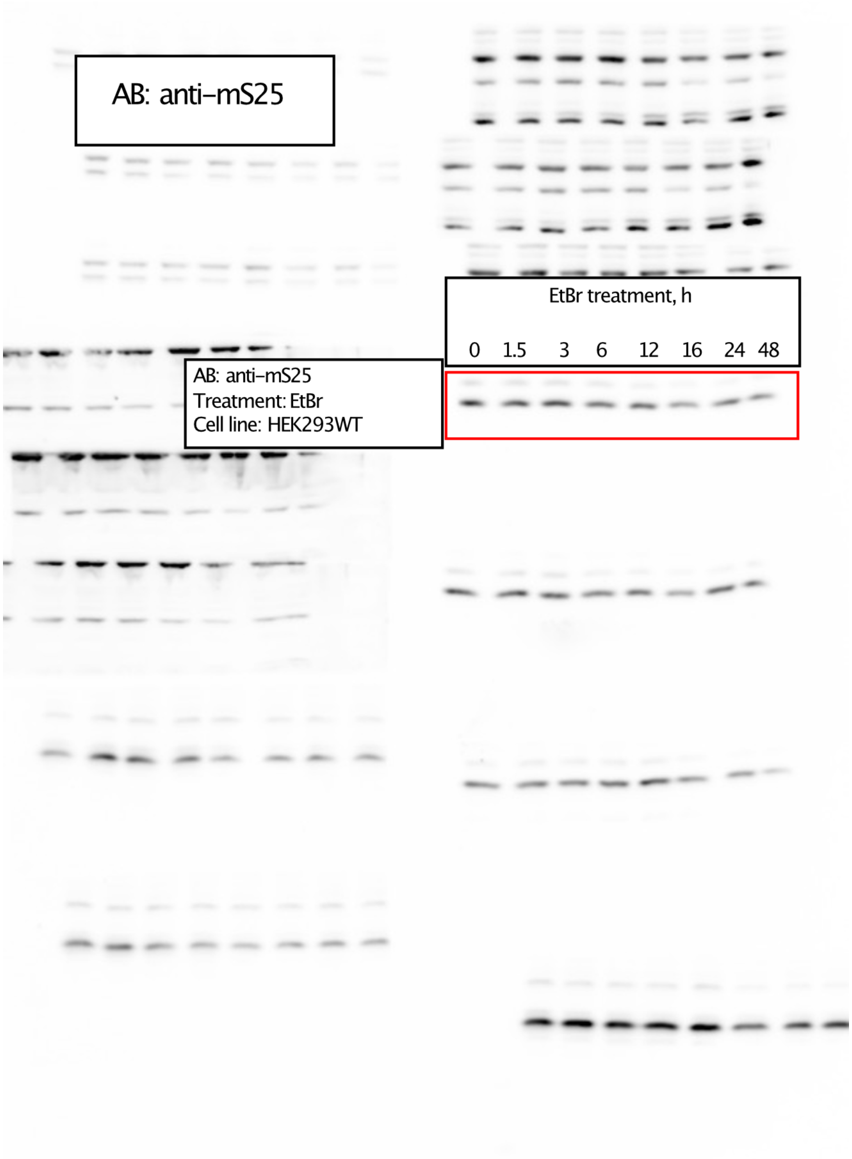

Supplementary Fig.13| Source Data for Supplementary Figure 7a (continuation)

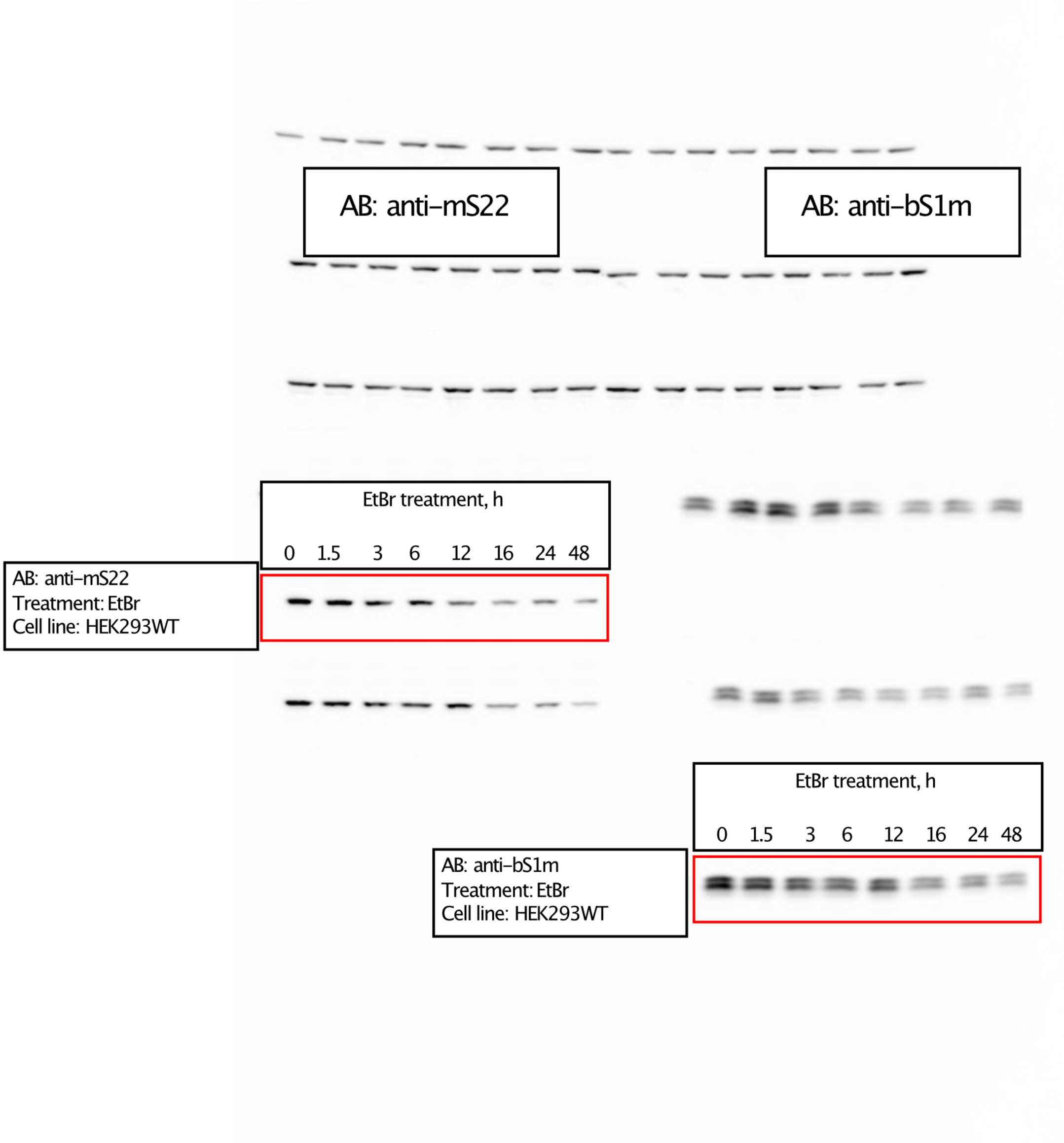

Supplementary Fig.13| Source Data for Supplementary Figure 7a (continuation)

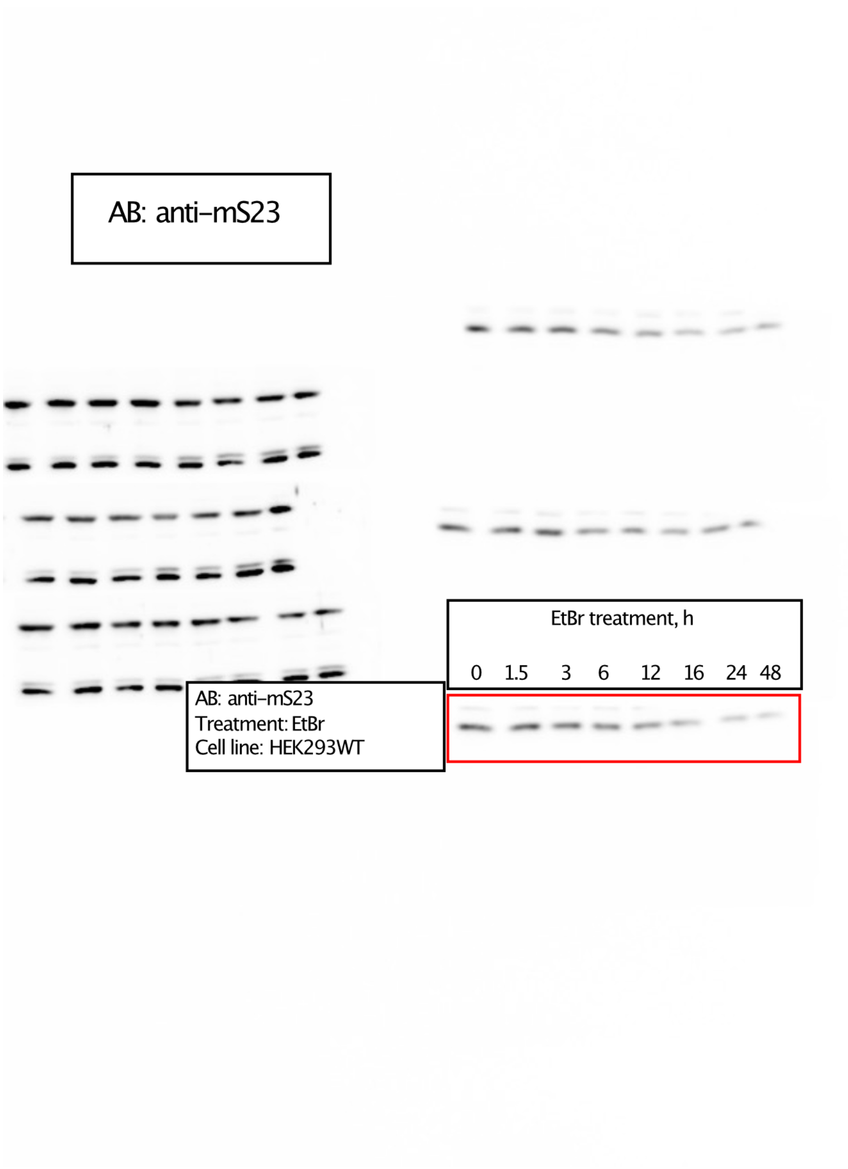

Supplementary Fig.13| Source Data for Supplementary Figure 7a (continuation)

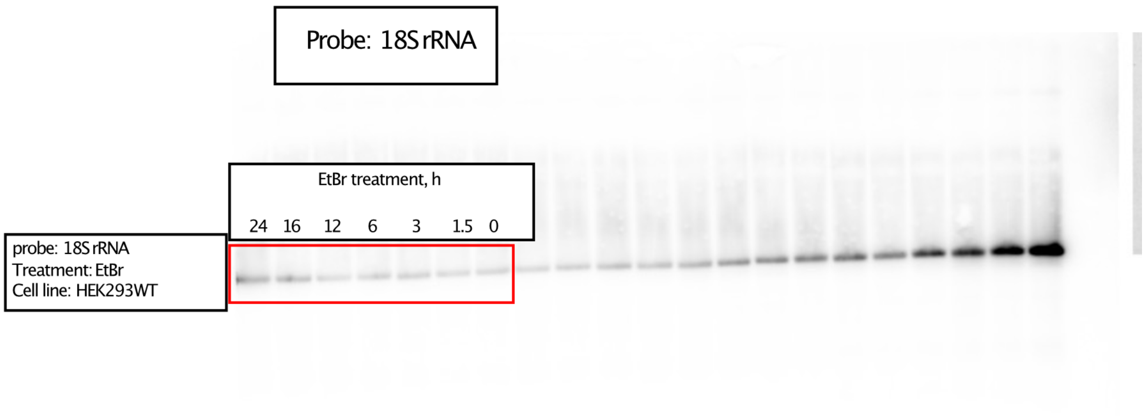

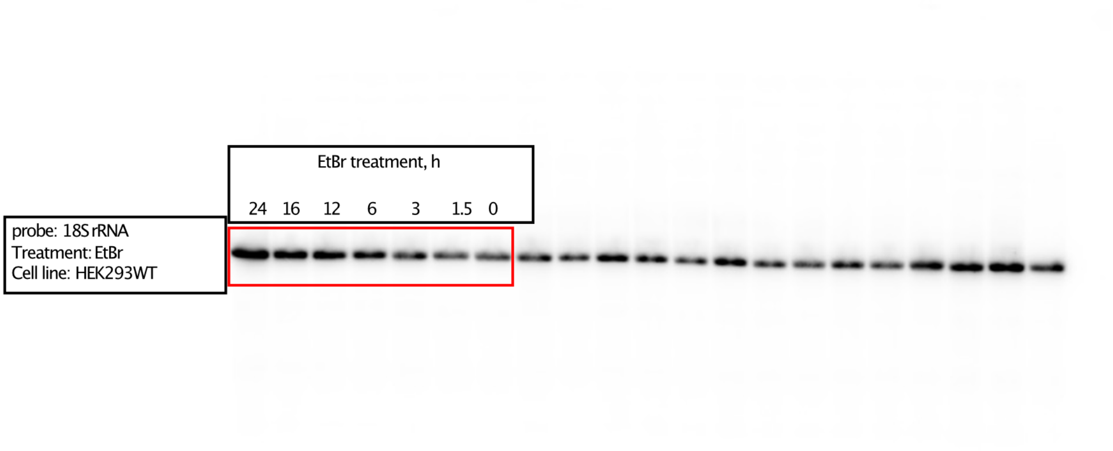

Supplementary Fig.14| Source Data for Supplementary Figure 7b

Probe: MTRNR2 (16S rRNA)

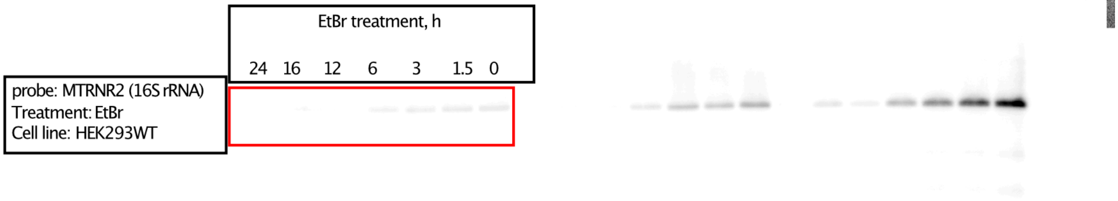

Probe: MTRNR1 (12S rRNA)

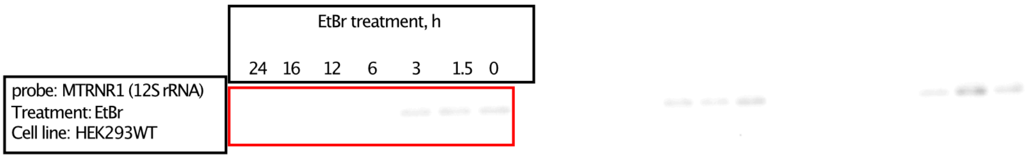

Supplementary Fig.14| Source Data for Supplementary Figure 7b (continuation)

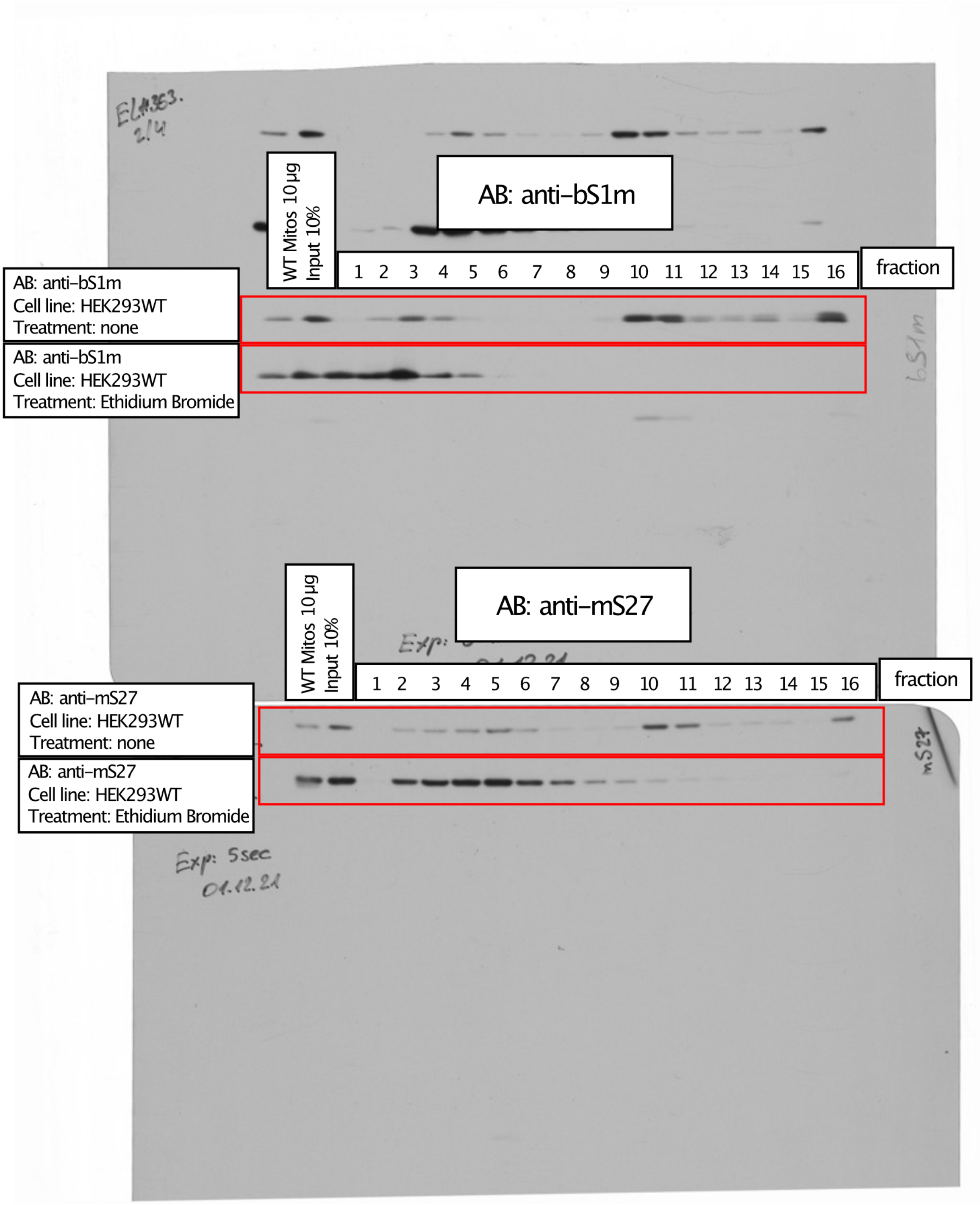

Supplementary Fig.15| Source Data for Supplementary Figure 8

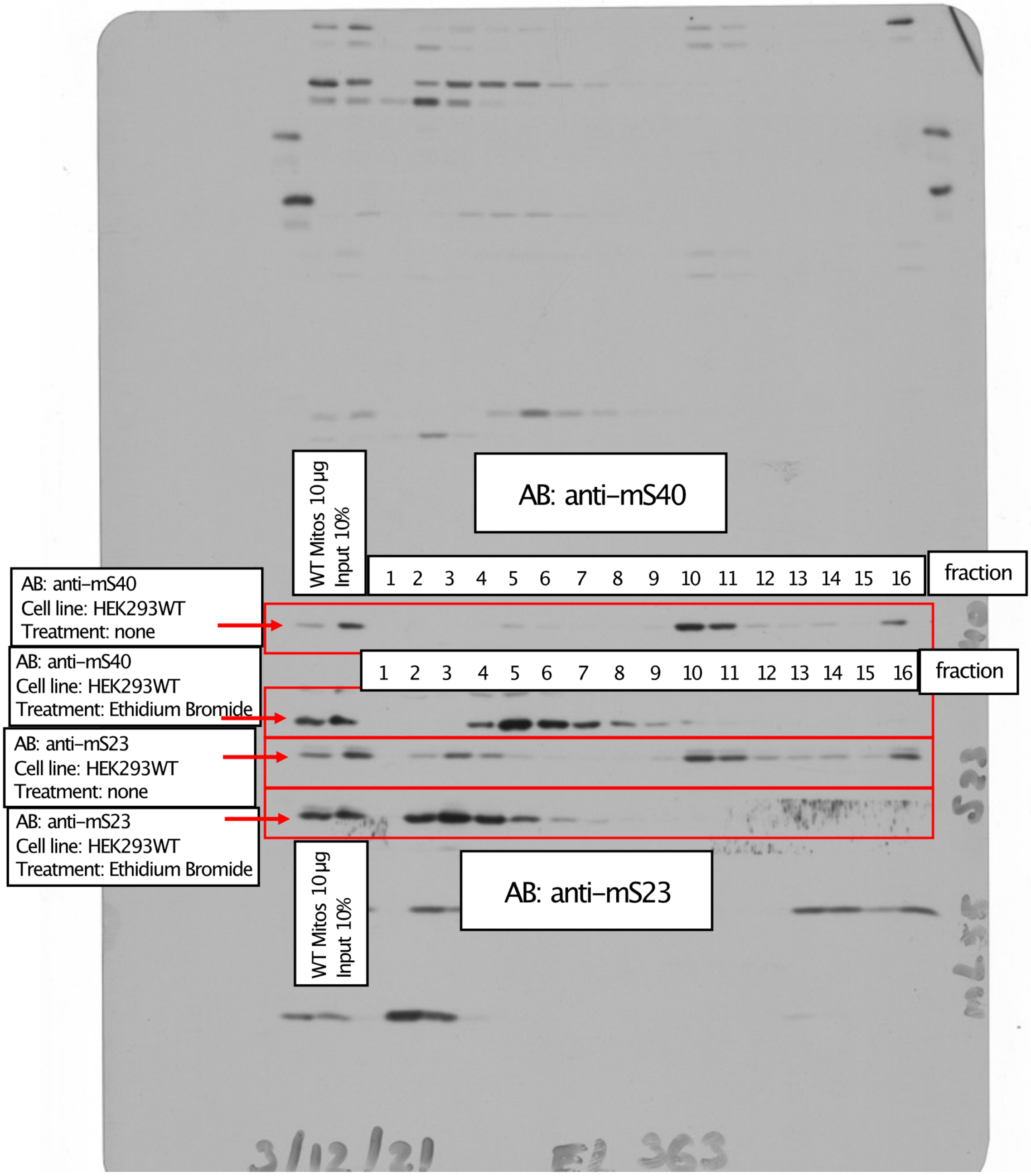

Supplementary Fig.15| Source Data for Supplementary Figure 8 (continuation)

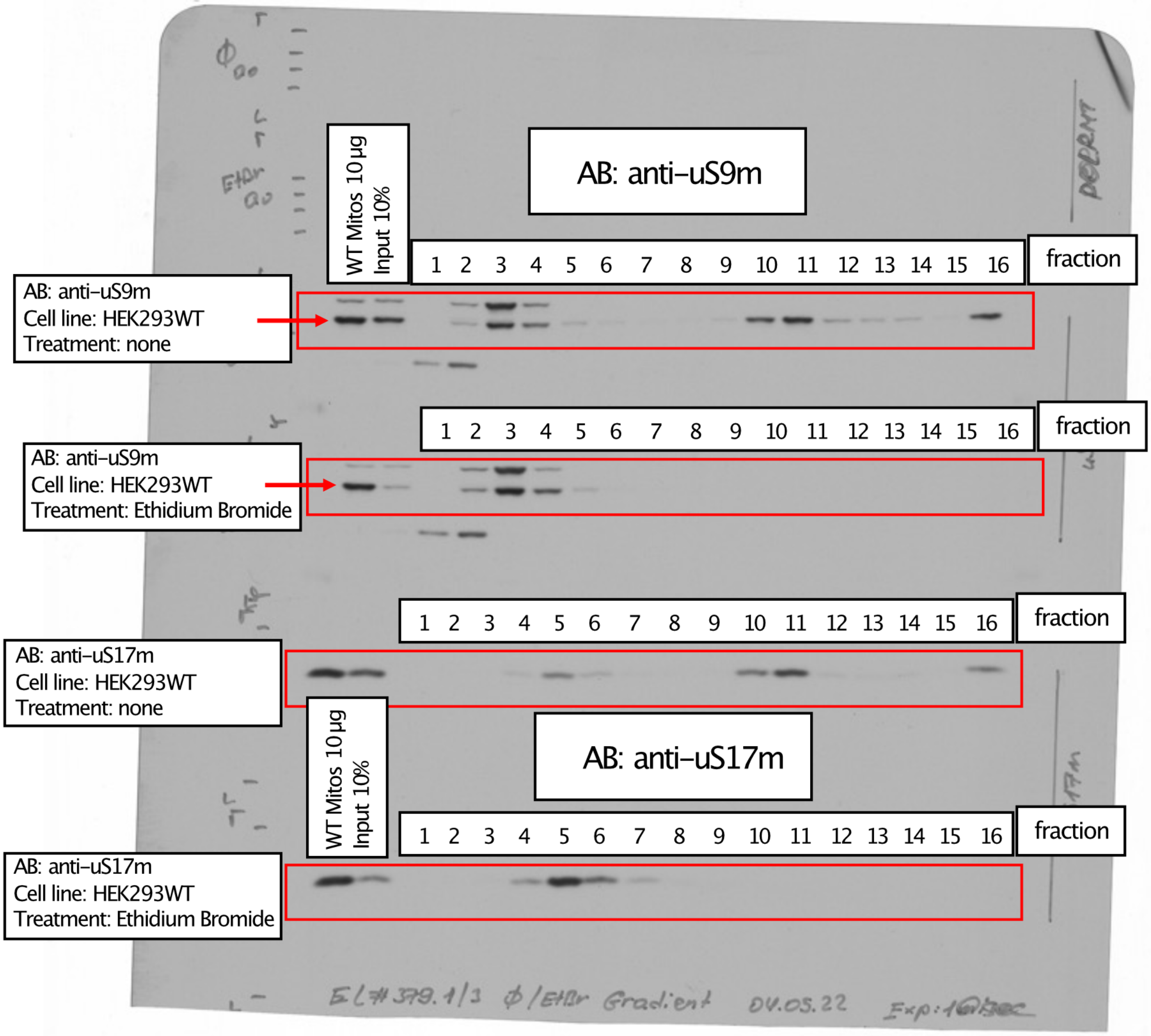

Supplementary Fig.15| Source Data for Supplementary Figure 8 (continuation)

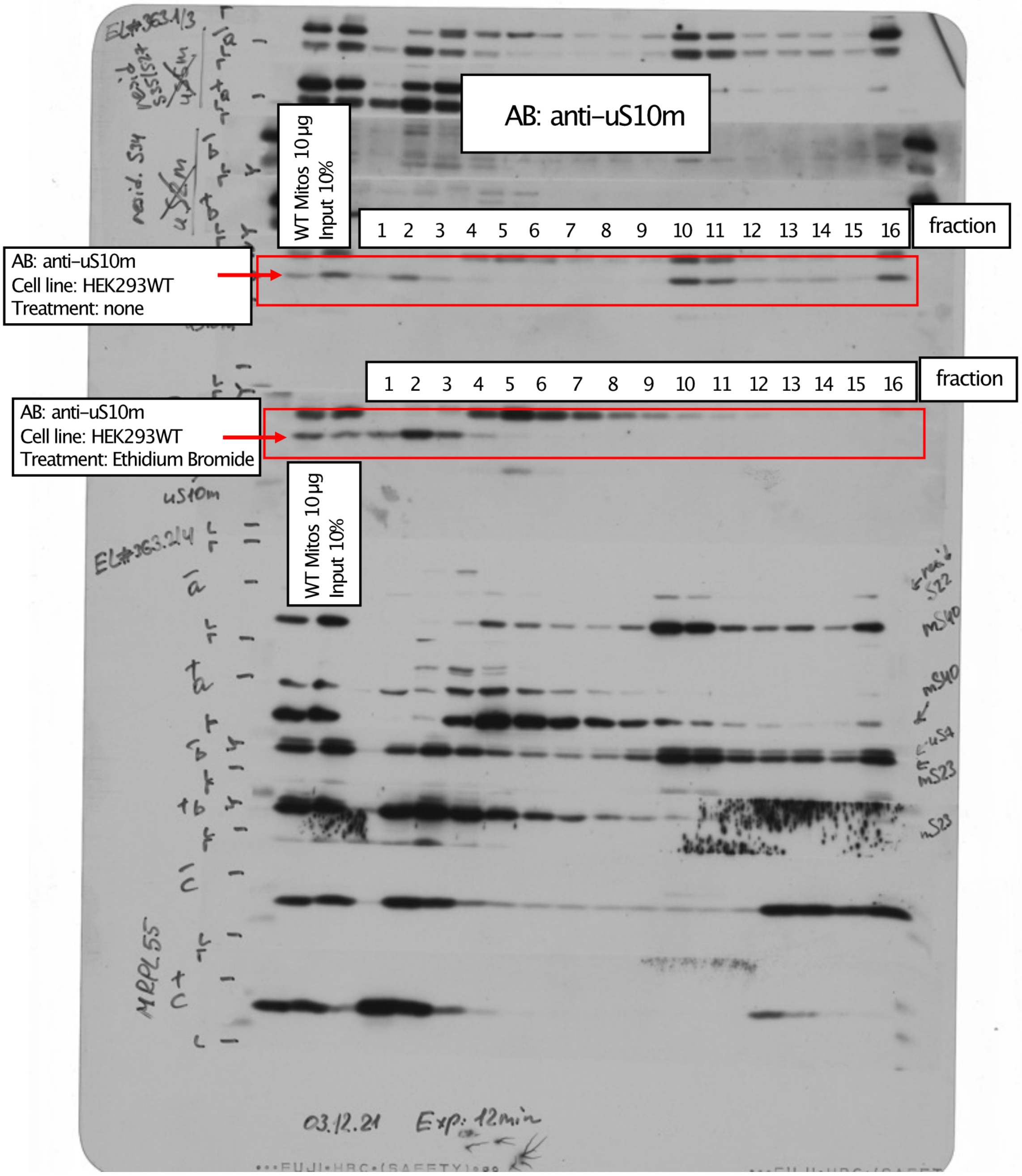

Supplementary Fig.15| Source Data for Supplementary Figure 8 (continuation)

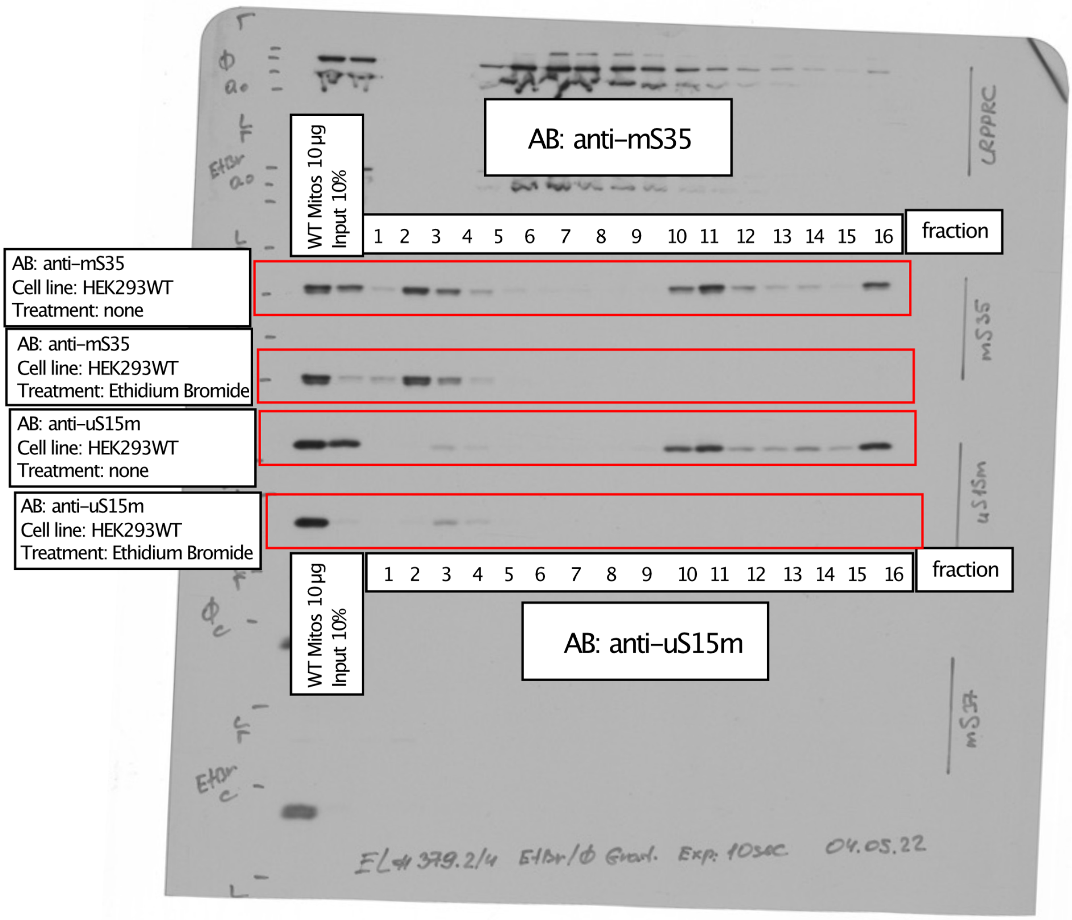

Supplementary Fig.15| Source Data for Supplementary Figure 8 (continuation)

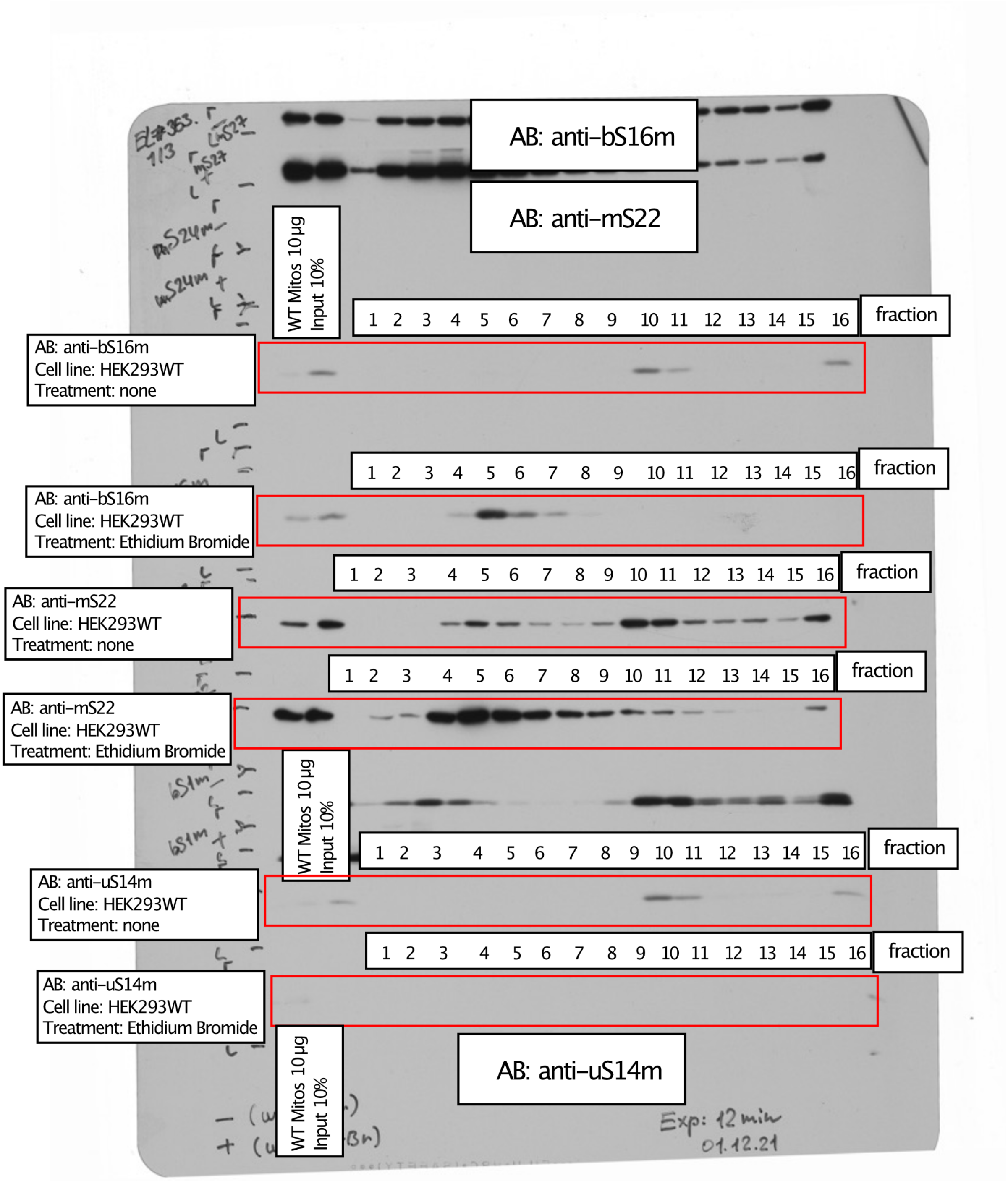

Supplementary Fig.15| Source Data for Supplementary Figure 8 (continuation)

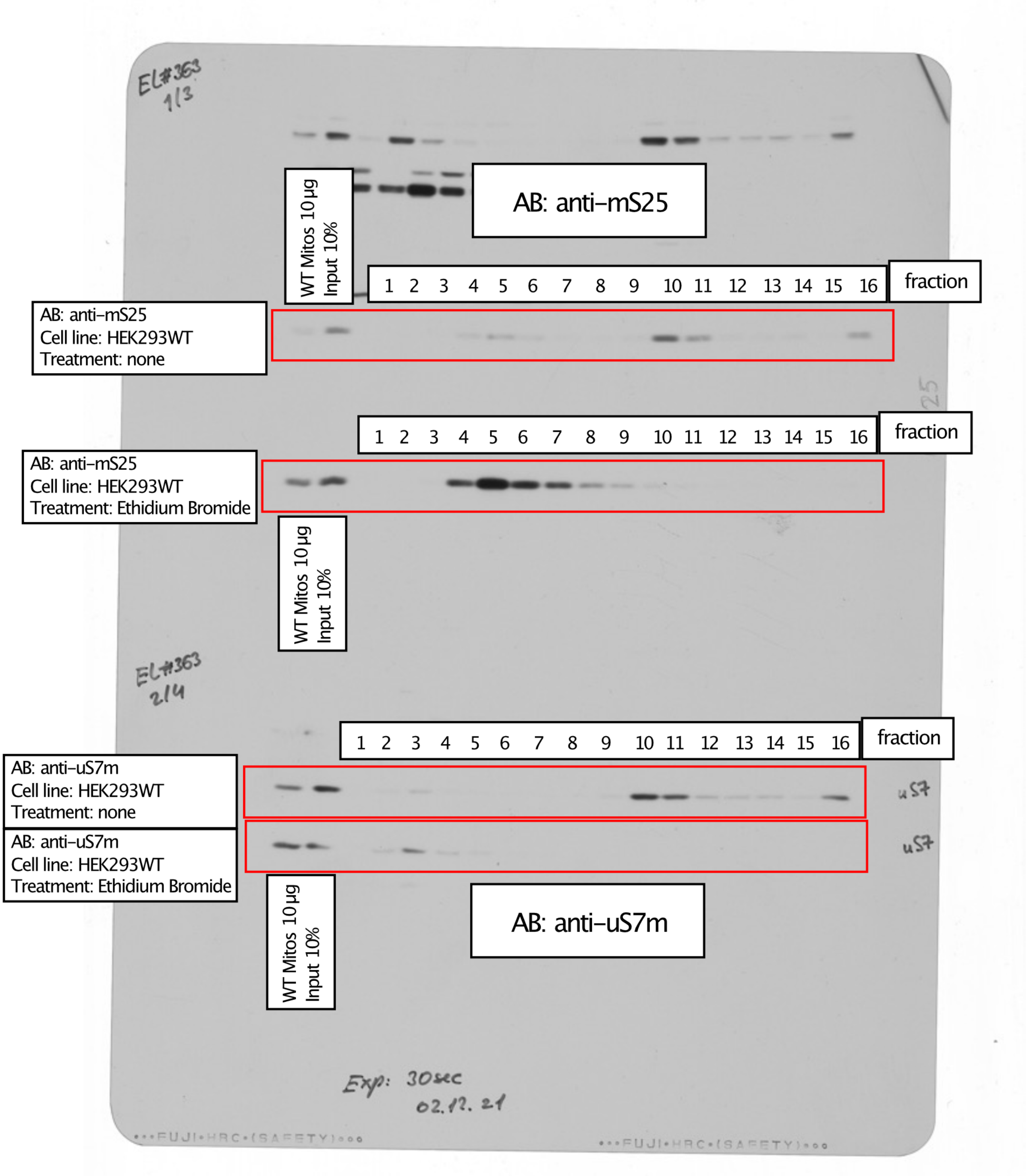

Supplementary Fig.15| Source Data for Supplementary Figure 8 (continuation)

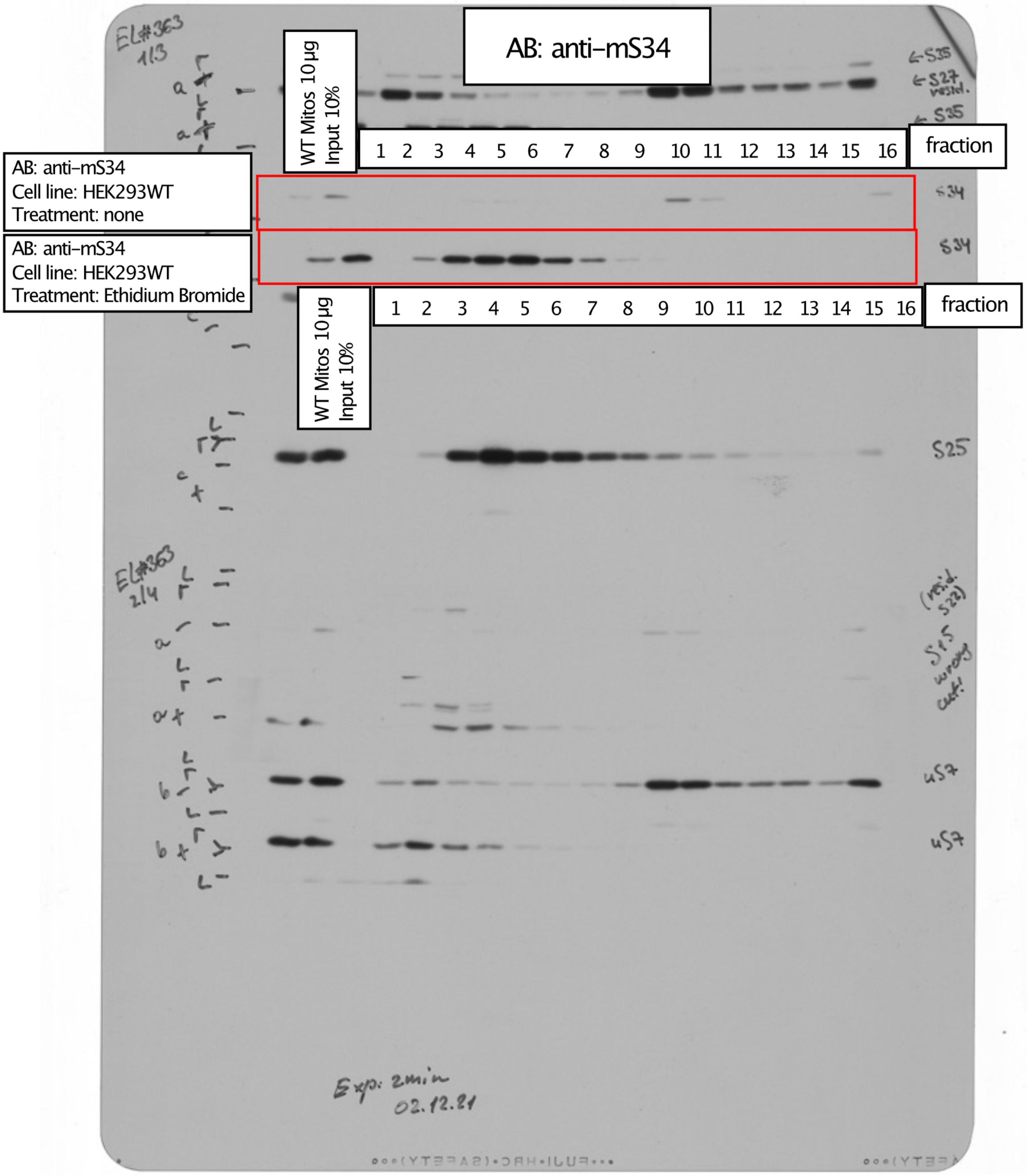

Supplementary Fig.15| Source Data for Supplementary Figure 8 (continuation)

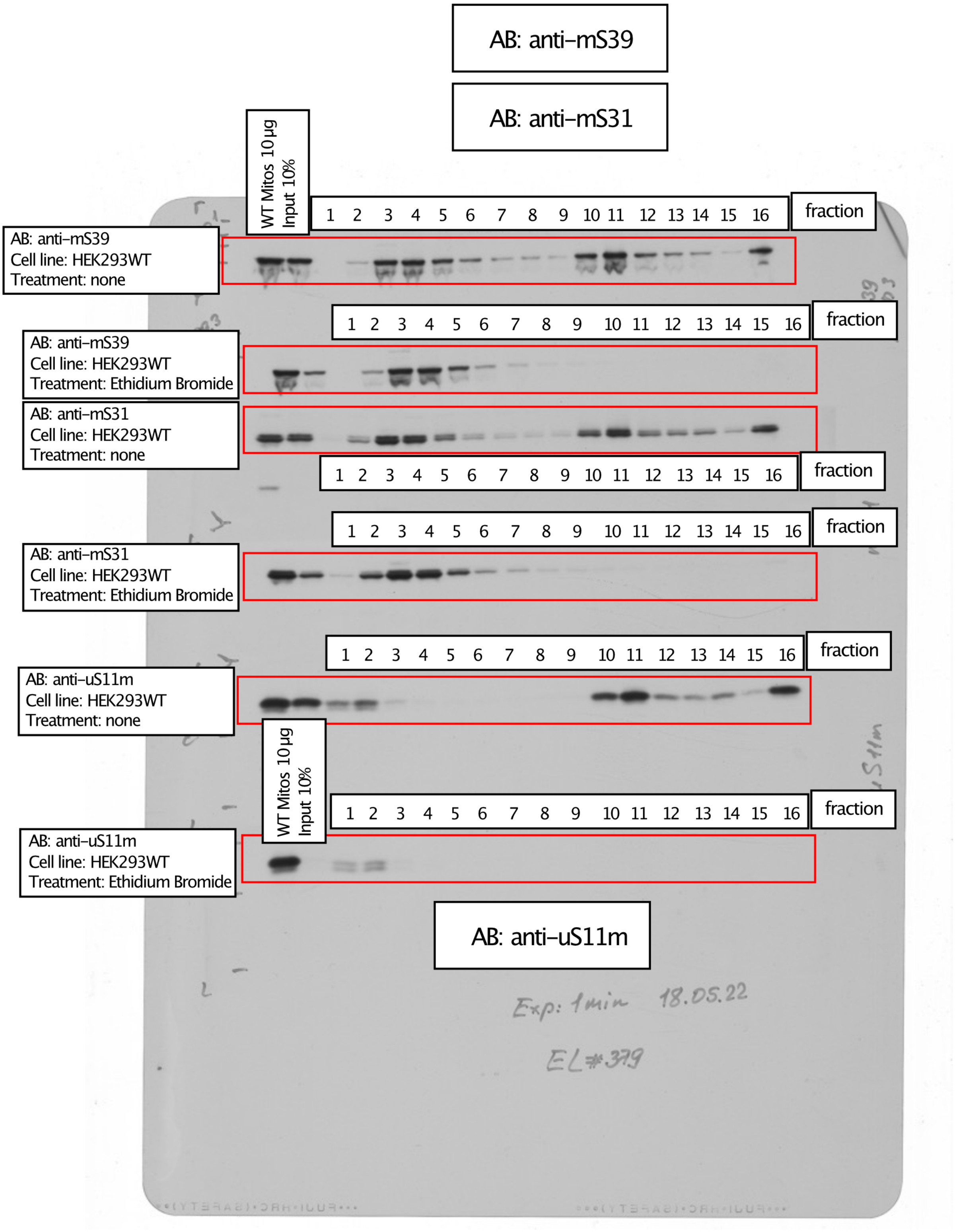

Supplementary Fig.15| Source Data for Supplementary Figure 8 (continuation)

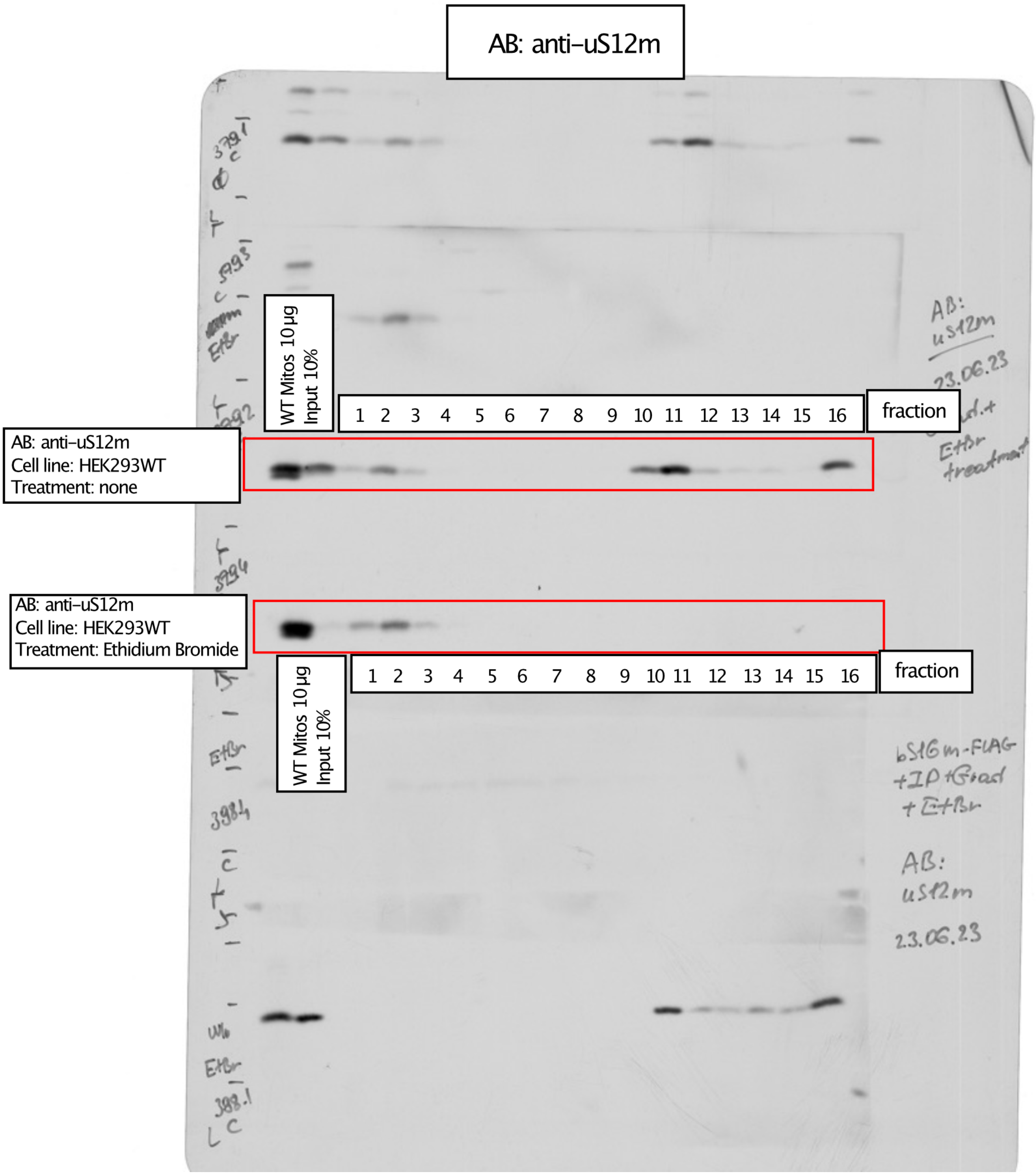

Supplementary Fig.15| Source Data for Supplementary Figure 8 (continuation)

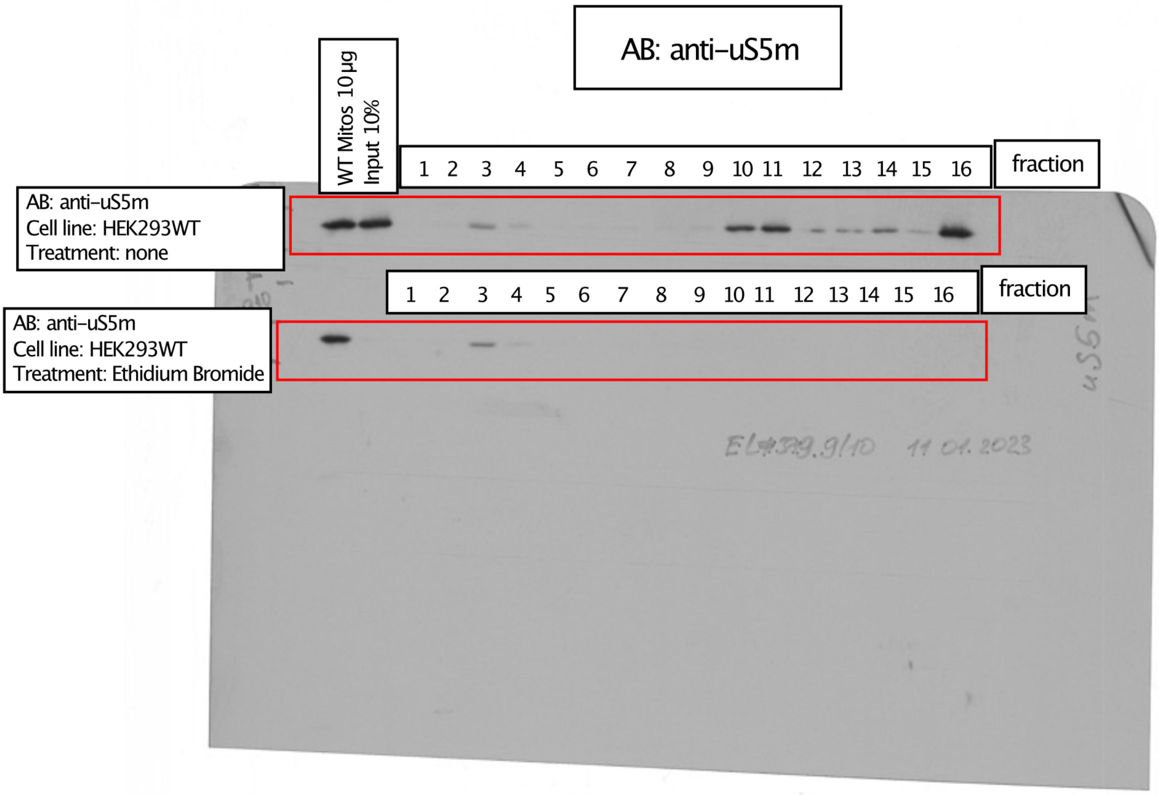

Supplementary Fig.15| Source Data for Supplementary Figure 8 (continuation)

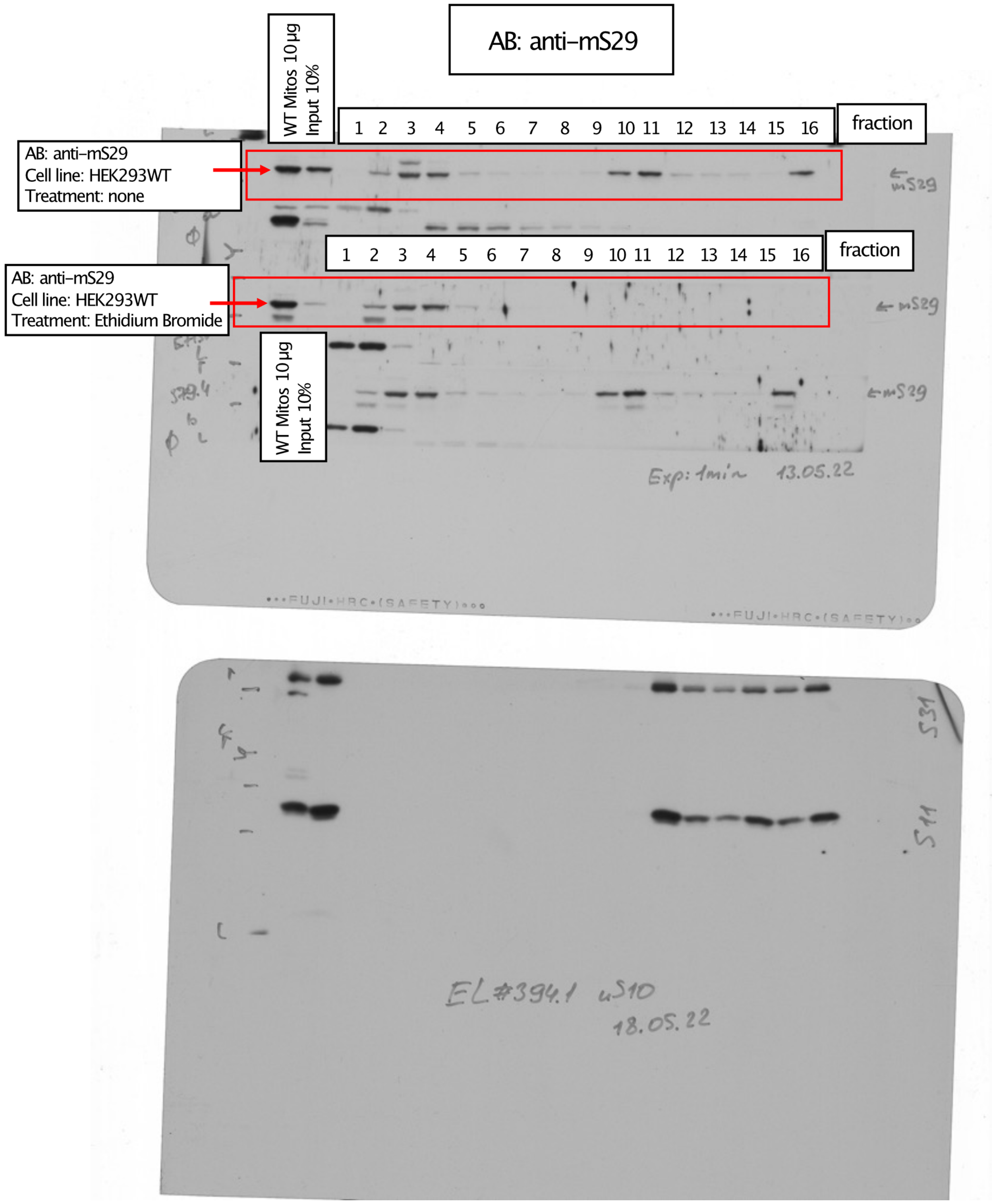

Supplementary Fig.15| Source Data for Supplementary Figure 8 (continuation)

Supplementary Tables

Supplementary Table 7: Prior distributions of kinetic rates for one-state-model and two-state-model

parameter	*parameter name*	*distribution*	*distribution parameters*	*unit*
k_a	degradation rate state A	Uniform	L=0; U=1	h^{-1}
k_b	degradation rate state B	Uniform	L=0; U=1	h^{-1}
k_{ab}	transfer rate from state A to state B	Uniform	L=0; U=1	h^{-1}
t_m	doubling time in medium chase	Gamma	α=5; β=0.7	h
t_l	doubling time in light chase	Gamma	α=5; β=0.7	h
σ	standard deviation of Normal distribution of additive error	HalfNormal	μ=0; σ=0.1	-

Supplementary Table 8: Uniform parameter prior distribution ranges of flux model

parameter	*parameter name*	*min*	*max*	*unit*
ki	turnover rate	0	1	h^{-1}
a_i	transfer rate	0	20	h^{-1}
$H_i(t=0)$	MRP initial condition	0	2	a.u.
sd	standard deviation	0	100	-

Supplementary Table 9: mtSSU MRPs and module assignment

MRP or module	*members*	*sucrose gradient fraction*
mS27	mS27	1
mS31	mS31	1
bS16m	bS16m	1
uS2m	uS2m	1
uS12m	uS12m	1
uS11m	uS11m	1

bS16m‘	bS16m	2
uS11m‘	uS11m	2
uS12m‘	uS12m	2
uS3m	uS3m	2
B1	bS6m, bS1m, mS23, uS2m	1
H1	uS10m, mS35	1
HB1	B1, uS9m	2
B2	mS22, mS25	2
B3	mS27, mS34	2
B4	uS17m, mS26	2
H3	mS29, uS7m	2
H2	mS39, mS31, uS3m	2
H2‘	H2	3
HB2	H1, HB1	3
B5	mS40, bS16m’, B2, B3, B4	3
HB3	B5, HB2	4
HB4	H2’, H3, 12SRNA, uS14m, uS15m	5
mtSSU	all mtSSU MRPs (bS18m, bS21m, HB3, HB4, mS33, uS12m’, uS5m, uS11m’)	6

Supplementary Table 10: List of all reactions that lead to the formation of different mtSSU modules. A graphic representation of these reactions is depicted in **Fig.3**.

educt E	***product P***
bS1m	B1
bS6m	B1
mS23	B1
uS2m	B1
mS22	B2
mS25	B2
mS27	B3
mS34	B3
mS26	B4
uS17m	B4
B2	B5
B3	B5
B4	B5
bS16m'	B5
mS40	B5
bS16m	bS16m'
mS35	H1
uS10m	H1
mS31	H2
mS39	H2
uS3m	H2
H2	H2'

mS29	H3
uS7m	H3
B1	HB1
uS9m	HB1
H1	HB2
HB1	HB2
B5	HB3
HB2	HB3
H2'	HB4
H3	HB4
12SRNA	HB4
uS14m	HB4
uS15m	HB4
bS18m	mtSSU
bS21m	mtSSU
HB3	mtSSU
HB4	mtSSU
mS33	mtSSU
mS38	mtSSU
uS12m'	mtSSU
uS5m	mtSSU
uS11m'	mtSSU
uS11m	uS11m'
uS12m	uS12m'

Supplementary Table 11: Truncated normal prior distribution parameters of mtSSU model.

parameter	**parameter name**	μ	σ	***max***	***unit***
on_j	binding rate	100	100	10^6	a.u.$^{-n}$ h^{-1}
off_j	unbinding rate	1	1	5	h^{-1}
k_i	turnover rate	0.1	0.3	1	h^{-1}
$P_i(t=0)$	MRPs initial condition	0.1	1	5	a.u.
sd	standard deviation	0	1	10	-

Here, *n* indicates the order of the binding reaction (*i.e.* number of binding partners) -1.

Supplementary Table 12. Key reagents and resources

**REAGENT or RESOURCE**	**Supplier**	**Catalog No.**
**Antibodies**		
Rabbit polyclonal anti-bS1m (1:1000)	Proteintech	16378-1-AP
Rabbit polyclonal anti-uS5m (1:5000)	Proteintech	16428-1-AP
Rabbit polyclonal anti-uS7m (1:1000)	Sigma-Aldrich	HPA 023007

Rabbit polyclonal anti-uS9m (1:7500)	ProteinTech	16533-1-AP
Rabbit polyclonal anti-uS10m (1:1000)	ProteinTech	16030-1-AP
Rabbit polyclonal anti-uS11m (1:1000)	ProteinTech	17041-1-AP
Rabbit polyclonal anti-uS12m (1:1000)	ProteinTech	15225-1-AP
Rabbit polyclonal anti-uS14m (1:1000)	ProteinTech	16301-1-AP
Rabbit polyclonal anti-uS15m (1:5000)	ProteinTech	17106-1-AP
Rabbit polyclonal anti-bS16m (1:5000)	ProteinTech	16735-1-AP
Rabbit polyclonal anti-uS17m (1:1000)	ProteinTech	18881-1-AP
Rabbit polyclonal anti-mS22 (1:5000)	ProteinTech	10984-1-AP
Rabbit polyclonal anti-mS23 (1:10000)	ProteinTech	18345-1-AP
Rabbit polyclonal anti-mS25 (1:5000)	ProteinTech	15277-1-AP
Rabbit polyclonal anti-mS27 (1:10000)	ProteinTech	17280-1-AP
Rabbit polyclonal anti-mS29 (1:1000)	ProteinTech	10276-1-AP
Rabbit polyclonal anti-mS31 (1:10000)	ProteinTech	16288-1-AP
Rabbit polyclonal anti-mS34 (1:5000)	ProteinTech	15166-1-AP
Rabbit polyclonal anti-mS35 (1:5000)	ProteinTech	16457-1-AP
Rabbit polyclonal anti-mS37 (1:500)	ProteinTech	11728-1-AP
Rabbit polyclonal anti-mS39 (1:20000)	ProteinTech	25158-1-AP
Rabbit polyclonal anti-mS40 (1:5000)	ProteinTech	16139-1-AP
Rabbit polyclonal anti-uL1m (1:10000)	[1]	PRAB4964
Rabbit polyclonal anti-uL3m (1:500)	Proteintech	16584-1-AP
Rabbit polyclonal anti-uL4m (1:1000)	Proteintech	27484-1-AP
Rabbit polyclonal anti-bL9m (1:5000)	Proteintech	15342-1-AP
Rabbit polyclonal anti-uL10m (1:1000)	Proteintech	16652-1-AP
Rabbit polyclonal anti-uL11m (1:1000)	Proteintech	15543-1-AP
Rabbit polyclonal anti-bL12m (1:5000)	Proteintech	14795-1-AP
Rabbit polyclonal anti-uL13m (1:5000)	Proteintech	16241-1-AP
Rabbit polyclonal anti-uL14m (1:1000)	Proteintech	15040-1-AP
Rabbit polyclonal anti-uL15m (1:1000)	Proteintech	18339-1-AP
Rabbit polyclonal anti-bL17m (1:1000)	Proteintech	17214-1-AP
Rabbit polyclonal anti-uL18m (1:1000)	Proteintech	15178-1-AP
Rabbit polyclonal anti-bL19m (1:1000)	Proteintech	16517-1-AP
Rabbit polyclonal anti-bL20m (1:200)	Proteintech	16969-A-AP
Rabbit polyclonal anti-bL21m (1:1000)	Proteintech	16978-1-AP
Rabbit polyclonal anti-uL22m (1:500)	Proteintech	16299-1-AP
Rabbit polyclonal anti-uL23m (1:10000)	[1]	PRAB1716
Rabbit polyclonal anti-uL24m (1:10000)	Proteintech	16224-1-AP
Rabbit polyclonal anti-uL29m (1:1000)	Proteintech	24728-1-AP
Rabbit polyclonal anti-bL31m (1:1000)	Proteintech	17679-1-AP
Rabbit polyclonal anti-bL32m (1:1000)	[2]	4957/4
Rabbit polyclonal anti-mL38 (1:7500)	Proteintech	15913-1-AP
Rabbit polyclonal anti-mL39 (1:500)	This study	PRAB4962

Rabbit polyclonal anti-mL40 (1:5000)	Novusbio	NBP1-82620
Rabbit polyclonal anti-mL43 (1:5000)	Proteintech	17477-1-AP
Rabbit polyclonal anti-mL44 (1:20000)	Proteintech	16394-1-AP
Rabbit polyclonal anti-mL45 (1:5000)	Proteintech	15682-1-AP
Rabbit polyclonal anti-mL46 (1:10000)	Proteintech	16611-1-AP
Rabbit polyclonal anti-mL48 (1:5000)	Proteintech	14677-1-AP
Rabbit polyclonal anti-mL50 (1:1000)	Invitrogen	PA5-54638
Rabbit polyclonal anti-mL52 (1:1000)	Proteintech	16800-1-AP
Rabbit polyclonal anti-mL53 (1:1000)	Proteintech	16142-1-AP
Rabbit polyclonal anti-mL54 (1:1000)	Proteintech	17683-1-AP
Rabbit polyclonal anti-mL62 (1:1000)	Proteintech	10403-1-AP
Rabbit polyclonal anti-mL64 (1:10000)	Proteintech	16260-1-AP
Mouse monoclonal anti-Calnexin (1:250000) (clone 2A2C6)	Proteintech	66903-1-Ig
Rabbit polyclonal anti-COX1 (1:2000)	[1]	PRAB5121
Rabbit polyclonal anti-POLRMT (1:20000)	Proteintech	17748-1-AP
Mouse monoclonal anti-SDHA (1:20000) (clone 2E3GC12FB2AE2)	ThermoFisher Scientific	459200
**Oligonucleotides**		
Primer: Generation of the FLAG-tagged version of uL10m Forward: 5'-CTCTCCGGATCCCCACCATGGCTGCGGCCGTGGCGG-3'	This study; Microsynth	N/A
Primer: Generation of the FLAG-tagged version of uL10m Reverse: 5'-CTTTCTGATATCCTACTTATCGTCGTCATCCTTGTAATCCGAGTCCGGAACAGTGTCAGGATC-3'	This study; Microsynth	N/A
Primer: Generation of the FLAG-tagged version of uL11m Forward: 5'-CTCTCCAAGCTTCCACCATGTCAAAGCTCGGCCGGGCC-3'	This study; Microsynth	N/A
Primer: Generation of the FLAG-tagged version of uL11m Reverse: 5'-CTTTCTGATATCCTACTTATCGTCGTCATCCTTGTAATCCTTCTTGGCAGCTTCTTCTTGGGCAG-3'	This study; Microsynth	N/A
Primer: Generation of the FLAG-tagged version of bL12m Forward: 5'-CTCTCCGGATCCCCACCATGCTGCCGGCGGCCGCTC-3'	This study; Microsynth	N/A
Primer: Generation of the FLAG-tagged version of bL12m Reverse: 5'-CTTTCTCTCGAGCTACTTATCGTCGTCATCCTTGTAATCCTCCAGAACCACGGTGCCGCC-3'	This study; Microsynth	N/A
Primer: Generation of the FLAG-tagged version of bL31m Forward: 5'-CTCTCCGGATCCCCACCATGGCGGCCGTGGGCAGCCT-3'	This study; Microsynth	N/A
Primer: Generation of the FLAG-tagged version of bL31m Reverse: 5'-CTTTCTGATATCCTACTTATCGTCGTCATCCTTGTAATCCTTCTTGGTCCTGGTCCAGAACTGTC-3'	This study; Microsynth	N/A
Primer: Generation of the FLAG-tagged version of mL39 Forward: 5'-CTCTCCAAGCTTCCACCATGGAGGCGCTGGCCATGGG-3'	This study; Microsynth	N/A
Primer: Generation of the FLAG-tagged version of mL39 Reverse: 5'-	This study; Microsynth	N/A

CTTTCTGATATCCTACTTATCGTCGTCATCCTTGTAATCGG TAGATGTACATTCCTCTGTTGCTTTAC-3’		
Primer: Generation of the FLAG-tagged version of mL44 Forward: 5’- CTCTCCGGATCC*CCACC*ATGGCGTCCGGGCTGGTAAG-3’	This study; Microsynth	N/A
Primer: Generation of the FLAG-tagged version of mL44 Reverse: 5’-CTTTCTGATATCCTACTTATCGTCGTCATCCTTGTAATCGC TGGCAGTGATGCTCTTTTC-3’	This study; Microsynth	N/A
Primer: Generation of the FLAG-tagged version of bS1m Forward: 5’-CTCTCCAAGCTTCCACCATGGCGGCGCTGTGTCGGAC-3’	This study; Microsynth	N/A
Primer: Generation of the FLAG-tagged version of bS1m Reverse: 5’-CTTTCTGATATCCTACTTATCGTCGTCATCCTTGTAATCTT TTTCATGATGTTCTTCTTTCGATCTTGAGTC-3’	This study; Microsynth	N/A
Primer: Generation of the FLAG-tagged version of uS10m Forward: 5’-CTCTCCGGATCCCCACCATGGCGGCGCGGACAGCGTT-3’	This study; Microsynth	N/A
Primer: Generation of the FLAG-tagged version of uS10m Reverse: 5’-CTTTCTGATATCCTACTTATCGTCGTCATCCTTGTAATCTG ACTTGCTTTCTTCTTTTTCTTCTGA-3’	This study; Microsynth	N/A
Primer: Generation of the FLAG-tagged version of mS22 Forward: 5’-CTCTCCGGATCCCCACCATGGCGCCCCTCGGAACAACTG-3’	This study; Microsynth	N/A
Primer: Generation of the FLAG-tagged version of mS22 Reverse: 5’-CTTTCTGATATCCTACTTATCGTCGTCATCCTTGTAATCGG AAGCTGCAGAATGGCGACTG-3’	This study; Microsynth	N/A
Primer: Generation of the FLAG-tagged version of mS25 Forward: 5’-CTCCTAAGCTTCCACCATGCCCATGAAGGGCCG-3’	This study; Microsynth	N/A
Primer: Generation of the FLAG-tagged version of mS25 Reverse: 5’-CTTCTGATATCCTACTTATCGTCGTCATCCTTGTAATCGTC CTGGGCATCGGCTTTC-3’	This study; Microsynth	N/A
Primer: Generation of the FLAG-tagged version of mS27 Forward: 5’-CTTTCTGGATCCCCACCATGGCTGCCTCCATAGTGCGGC-3’	This study; Microsynth	N/A
Primer: Generation of the FLAG-tagged version of mS27 Reverse: 5’-CTCTCCGATATCCTACTTATCGTCGTCATCCTTGTAATCGG CAGATGCCTTTGCTGCTTTCTGAGCCTG-3’	This study; Microsynth	N/A
Guide RNA: targeting the Exon 4 of uL4m: 5’-AGTGGCACTGACCGTCAAGC-3’	This study; IDT	N/A
Guide RNA: targeting the Exon 2 of bL20m: 5’-TTTCACAAAGGCTCGAATCA-3’	This study; IDT	N/A
Guide RNA: targeting the Exon 1 of mL44: 5’-AAGCTGGTCCCTCCGGTTCG-3’	This study; Microsynth	N/A
Guide RNA: targeting the Exon 6 of mL45: 5’-GGGCAACGTGTACGGCCAGA-3’	This study; Microsynth	N/A
Guide RNA: targeting the Exon 5 of mL62: 5’-ATGGAACCTGACTTCTGCCT-3’	This study; IDT	N/A
Guide RNA: targeting the Exon 1 of uS7m: 5’-CACCGTCGGGCAACCTTCACTG-3’	This study; Microsynth	N/A
Guide RNA: targeting the Exon 1 of mS40: 5’-CTGTATTAAACACCGTGCTG-3’	This study; IDT	N/A

Probe for northern blot: targeting MTRNR1 (12S rRNA): 5'-TCGATTACAGAACAGGCTCCTCTAG-3'	[3]	N/A
Probe for northern blot: targeting MTRNR2 (16S rRNA): 5'-GTTTGGCTAAGGTTGTCTGGTAGTA-3'	[2]	N/A
Probe for northern blot: targeting 18S rRNA: 5'-TTTACTTCCTCTAGATAGTCAAGTTCGACC-3'	[4]	N/A
**Experimental models: Cell Lines**		
HEK293-Flp-In T-Rex WT	ThermoFisher Scientific	R78007
HEK293-Flp-In T-Rex uL4m$^{-/-}$	This study	N/A
HEK293-Flp-In T-Rex bL20m$^{-/-}$	This study	N/A
HEK293-Flp-In T-Rex mL44$^{-/-}$	This study	N/A
HEK293-Flp-In T-Rex mL45$^{-/-}$	This study	N/A
HEK293-Flp-In T-Rex mL62$^{-/-}$	This study	N/A
HEK293-Flp-In T-Rex uS7m$^{-/-}$	This study	N/A
HEK293-Flp-In T-Rex mS40$^{-/-}$	This study	N/A
HEK293-Flp-In T-Rex uL10m^{FLAG}	This study	N/A
HEK293-Flp-In T-Rex uL11m^{FLAG}	This study	N/A
HEK293-Flp-In T-Rex bL12m^{FLAG}	This study	N/A
HEK293-Flp-In T-Rex bL31m^{FLAG}	This study	N/A
HEK293-Flp-In T-Rex mL39^{FLAG}	This study	N/A
HEK293-Flp-In T-Rex mL44^{FLAG}	This study	N/A
HEK293-Flp-In T-Rex mL62^{FLAG}	[1]	N/A
HEK293-Flp-In T-Rex bS1m^{FLAG}	This study	N/A
HEK293-Flp-In T-Rex uS10m^{FLAG}	This study	N/A
HEK293-Flp-In T-Rex mS22^{FLAG}	This study	N/A
HEK293-Flp-In T-Rex mS25^{FLAG}	This study	N/A
HEK293-Flp-In T-Rex mS27^{FLAG}	This study	N/A
HEK293-Flp-In T-Rex mS40^{FLAG}	[2]	N/A
**Recombinant DNA**		
pOG44 Flp-Recombinase Expression Vector	ThermoFisher Scientific	V600520
pcDNA5/FRT/TO	ThermoFisher Scientific	V6520-20
pX330-U6-Chimeric_BB-CBh-hSpCas9	Addgene	42230
pEGFP-N1	Clontech	N/A
**Software and Algorithm**		
Affinity Designer	Serif Europe Ltd	N/A
Affinity Photo	Serif Europe Ltd	N/A
Typhoon imaging system	GE Healthcare	N/A
PDBePISA	European Bioinformatics Institute	N/A
UCSF ChimeraX	Resource for Biocomputing, Visualization, and Informatics (RBVI)	N/A

MaxQuant software version 1.6.0.1	Max-Planck-Institute of Biochemistry [5]	N/A
R: A language and environment for statistical computing version 4.1.0	R Core Team, R Foundation for Statistical Computing	https://www.R-project.org/
**Chemicals**		
Anti-FLAG M2 Affinity Gel	Sigma-Aldrich	A2220
cOmplete™ Protease inhibitor cocktail tablets	Roche	11836170001
RiboLock RNase Inhibitor	ThermoFisher Scientific	EO0381
Ethidium Bromide	Roth	2218.1
GlycoBlue coprecipitant	Invitrogen	AM9515
FLAG-peptide	Sigma-Aldrich	F3290-4MG
L-Arginine:HCL ($^{13}C_6$, 99%)	14L-515	Cambridge Isotope Laboratories
L-Arginine:HCL ($^{13}C_6$, 99%; $^{15}N_4$, 99%)	16J-570	Cambridge Isotope Laboratories
L-Lysine:2HCL (4,4,5,5,-D4, 96-98%)	17H-615	Cambridge Isotope Laboratories
L-Lysine:2HCL ($^{13}C_6$, 99%; $^{15}N_2$, 99%)	13L-177	Cambridge Isotope Laboratories
L-[^{35}S]methionine	Hartmann Analytic	SCM-01
Adenosine 5'-triphosphate, [γ-^{32}P]	Hartmann Analytic	SRP-501
Emetine dihydrochloride hydrate	Sigma-Aldrich	219282
Lipofectamine 3000	Invitrogen	L3000-015
GeneJuice	Novagen	70967-3
Alt-R® CRISPR-Cas9 tracrRNA, ATTO™ 550	Integrated DNA technologies	1075927
Alt-R® S.p. Cas9 Nuclease V3	Integrated DNA technologies	1081058

Supplementary References

1. Richter-Dennerlein, R. *et al.* Mitochondrial Protein Synthesis Adapts to Influx of Nuclear-Encoded Protein. *Cell* **167**, 471-483.e10 (2016).

2. Lavdovskaia, E. *et al.* The human Obg protein GTPBP10 is involved in mitoribosomal biogenesis. *Nucleic Acids Res.* **46**, 8471–8482 (2018).

3. Lavdovskaia, E. *et al.* Dual function of GTPBP6 in biogenesis and recycling of human mitochondrial ribosomes. *Nucleic acids Res.* **48**, 12929–12942 (2020).

4. Larburu, N. *et al.* Structure of a human pre-40S particle points to a role for RACK1 in the final steps of 18S rRNA processing. *Nucleic Acids Res.* **44**, 8465–8478 (2016).

5. Cox, J. & Mann, M. MaxQuant enables high peptide identification rates, individualized p.p.b.-range mass accuracies and proteome-wide protein quantification. *Nat. Biotechnol.* **26**, 1367–1372 (2008).
