# Supplementary material for: A roadmap for ribosome assembly in human mitochondria
Source: Nat Struct Mol Biol. 2024 Jul 11;31(12):1898–908. doi: 10.1038/s41594-024-01356-w (PMC11638073; doi:10.1038/s41594-024-01356-w)
Supplement: Supplementary file 2 — Reporting Summary [file 41594_2024_1356_MOESM2_ESM.pdf]

nature portfolio

Corresponding author(s): Ricarda Richter-Dennerlein, Juliane Liepe, Henning Urlaub

Last updated by author(s): Mar 5, 2024

Reporting Summary

Nature Portfolio wishes to improve the reproducibility of the work that we publish. This form provides structure for consistency and transparency in reporting. For further information on Nature Portfolio policies, see our Editorial Policies and the Editorial Policy Checklist.

Statistics

For all statistical analyses, confirm that the following items are present in the figure legend, table legend, main text, or Methods section.

n/a	Confirmed	
☐	☒	The exact sample size (*n*) for each experimental group/condition, given as a discrete number and unit of measurement
☐	☒	A statement on whether measurements were taken from distinct samples or whether the same sample was measured repeatedly
☐	☒	The statistical test(s) used AND whether they are one- or two-sided<br>*Only common tests should be described solely by name; describe more complex techniques in the Methods section.*
☒	☐	A description of all covariates tested
☒	☐	A description of any assumptions or corrections, such as tests of normality and adjustment for multiple comparisons
☐	☒	A full description of the statistical parameters including central tendency (e.g. means) or other basic estimates (e.g. regression coefficient) AND variation (e.g. standard deviation) or associated estimates of uncertainty (e.g. confidence intervals)
☐	☒	For null hypothesis testing, the test statistic (e.g. *F*, *t*, *r*) with confidence intervals, effect sizes, degrees of freedom and *P* value noted<br>*Give P values as exact values whenever suitable.*
☐	☒	For Bayesian analysis, information on the choice of priors and Markov chain Monte Carlo settings
☒	☐	For hierarchical and complex designs, identification of the appropriate level for tests and full reporting of outcomes
☒	☐	Estimates of effect sizes (e.g. Cohen's *d*, Pearson's *r*), indicating how they were calculated

Our web collection on statistics for biologists contains articles on many of the points above.

Software and code

Policy information about availability of computer code

Data collection	for mass spectrometry data acquisition: Thermo Fisher Scientific software: Thermo Xcalibur Instrument Setup, Thermo Scientific Xcalibur Version 4. 4.16.14, Tune Application, Version 4.0.309.28 (for monitoring detection).
Data analysis	- Affinity Designer V2 (Serif Europe Ltd); Affinity Photo V2 (Serif Europe Ltd); Typhoon imaging system v. 8.1 (GE Healthcare); PDBePISA (European Bioinformatics Institute); UCSF ChimeraX (Resource for Biocomputing, Visualization, and Informatics (RBVI)); MaxQuant software version 1. 6.0 .1 (software to analyze mass spectrometry data)<br>- custom scripts: R scripts to process and visualize MaxQuant software outputs, R scripts to model mtSSU kinetics (detailed in methods; used R packages: deSolve v1.35, stringr v1.5.0, dplyr v1.1.1, vroom v1.6.1, tidr v1.1.4), Python (v3.9.17) scripts to analyse unfractionated dataset (used Python packages: PyMC v 5.7.2), Matlab (v 9.13.) to obtain an algebraic solution of the ODE systems of the one-state-model and of the two-state-model<br><br>No custom algorithm or software were utilized in this study. R Scripts and Python scripts for data processing and visualization are provided on Figshare: 10.6084/m9.figshare.25343125

For manuscripts utilizing custom algorithms or software that are central to the research but not yet described in published literature, software must be made available to editors and reviewers. We strongly encourage code deposition in a community repository (e.g. GitHub). See the Nature Portfolio guidelines for submitting code & software for further information.

Data

Policy information about availability of data

All manuscripts must include a data availability statement. This statement should provide the following information, where applicable:

- Accession codes, unique identifiers, or web links for publicly available datasets
- A description of any restrictions on data availability
- For clinical datasets or third party data, please ensure that the statement adheres to our policy

A data availability statement is included in the manuscript.
Material will be available upon reasonable request and source data are provided with this paper. The original data generated in this study are provided in the supplementary information and the source data files.
The mass spectrometry original data, protein sequence databases (downloaded from UniProt Knowledgebase), MaxQuant analysis files, and database search output files have been deposited via the MASSIVE repository and are available using the following identifiers: MSV000091653, MSV000091652.

Research involving human participants, their data, or biological material

Policy information about studies with human participants or human data. See also policy information about sex, gender (identity/presentation), and sexual orientation and race, ethnicity and racism.

Reporting on sex and gender	n.a.
Reporting on race, ethnicity, or other socially relevant groupings	n.a.
Population characteristics	n.a.
Recruitment	n.a.
Ethics oversight	n.a.

Note that full information on the approval of the study protocol must also be provided in the manuscript.

Field-specific reporting

Please select the one below that is the best fit for your research. If you are not sure, read the appropriate sections before making your selection.

☒ Life sciences ☐ Behavioural & social sciences ☐ Ecological, evolutionary & environmental sciences

For a reference copy of the document with all sections, see nature.com/documents/nr-reporting-summary-flat.pdf

Life sciences study design

All studies must disclose on these points even when the disclosure is negative.

Sample size	No sample-size calculation was performed. All experiments were done in vitro. Sample in this study is defined as a biological replicate, which is derived from a HEK293 cell line clone. Hence, biological replicates represent variation in experimentation rather than biological variation. The number of biological replicates (n=3) was chosen based on community standard.
Data exclusions	No data were excluded.
Replication	All experiments were done in vitro with commonly used 3 biological replicates and 2 technical replicates. All replication attempts were successful.
Randomization	In this study, no comparison between experimental sample groups was carried out. Hence, there was no randomization necessary.
Blinding	Blinding is not applicable in this study, as only in vitro experiments without any human or animal subjects were carried out. The knowlegde of the experiment subject by the person carrying out the experiment has no impact on the results in this study.

Reporting for specific materials, systems and methods

We require information from authors about some types of materials, experimental systems and methods used in many studies. Here, indicate whether each material, system or method listed is relevant to your study. If you are not sure if a list item applies to your research, read the appropriate section before selecting a response.

Materials & experimental systems

n/a	Involved in the study
☐	☒ Antibodies
☐	☒ Eukaryotic cell lines
☒	☐ Palaeontology and archaeology
☒	☐ Animals and other organisms
☒	☐ Clinical data
☒	☐ Dual use research of concern
☒	☐ Plants

Methods

n/a	Involved in the study
☒	☐ ChIP-seq
☒	☐ Flow cytometry
☒	☐ MRI-based neuroimaging

Antibodies

Antibodies used

Rabbit polyclonal anti-bS1m (Proteintech; Cat#16378-1-AP); Rabbit polyclonal anti-uS5m (Proteintech; Cat#16428-1-AP); Rabbit polyclonal anti-uS7m (Sigma-Aldrich; Cat# HPA 023007); Rabbit polyclonal anti-uS9m (Proteintech; Cat#16533-1-AP); Rabbit polyclonal anti-uS10m (Proteintech; Cat#16030-1-AP); Rabbit polyclonal anti-uS11m (Proteintech; Cat#17041-1-AP); Rabbit polyclonal anti-uS12m (Proteintech, Cat#15225-1-AP); Rabbit polyclonal anti-uS14m (Proteintech; Cat#16301-1-AP); Rabbit polyclonal anti-uS15m (Proteintech; Cat#17106-1-AP); Rabbit polyclonal anti-bS16m (Proteintech; Cat#16735-1-AP); Rabbit polyclonal anti-uS17m (ProteinTech; Cat#18881-1-AP); Rabbit polyclonal anti-mS22 (ProteinTech; Cat#10984-1-AP); Rabbit polyclonal anti-mS23 (ProteinTech; Cat#18345-1-AP); Rabbit polyclonal anti-mS25 (ProteinTech; Cat#15277-1-AP); Rabbit polyclonal anti-mS27 (ProteinTech; Cat#17280-1-AP); Rabbit polyclonal anti-mS29 (ProteinTech; Cat#10276-1-AP); Rabbit polyclonal anti-mS31 (ProteinTech; Cat#16288-1-AP); Rabbit polyclonal anti-mS34 (ProteinTech; Cat#15166-1-AP); Rabbit polyclonal anti-mS35 (ProteinTech; Cat#16457-1-AP);Rabbit polyclonal anti-mS37 (Proteintech, #11728-1-AP); Rabbit polyclonal anti-mS39 (ProteinTech; Cat#25158-1-AP); Rabbit polyclonal anti-mS40 (ProteinTech; Cat#16139-1-AP); Rabbit polyclonal anti-uL1m (homemade; provided by P. Rehling); Rabbit polyclonal anti-uL3m (Proteintech; Cat#16584-1-AP); Rabbit polyclonal anti-uL4m (Proteintech; Cat#27484-1-AP); Rabbit polyclonal anti-bL9m (Proteintech; Cat#15342-1-AP); Rabbit polyclonal anti-uL10m (Proteintech; Cat#16652-1-AP); Rabbit polyclonal anti-uL11m (Proteintech; Cat#15543-1-AP); Rabbit polyclonal anti-bL12m (Proteintech; Cat#14795-1-AP); Rabbit polyclonal anti-uL13m (Proteintech; Cat#16241-1-AP); Rabbit polyclonal anti-uL14m (Proteintech, Cat#15040-1-AP); Rabbit polyclonal anti-uL15m (Proteintech; Cat#18339-1-AP); Rabbit polyclonal anti-bL17m (Proteintech; Cat#17214-1-AP); Rabbit polyclonal anti-uL18m (Proteintech; Cat#15178-1-AP); Rabbit polyclonal anti-bL19m (Proteintech; Cat#16517-1-AP); Rabbit polyclonal anti-bL20m (Proteintech; Cat#16969-1-AP); Rabbit polyclonal anti-bL21m (Proteintech; Cat#16978-1-AP); Rabbit polyclonal anti-uL22m (Proteintech; Cat#16299-1-AP); Rabbit polyclonal anti-uL23m (homemade; provided by P. Rehling); Rabbit polyclonal anti-uL24m (Proteintech; Cat#16224-1-AP); Rabbit polyclonal anti-uL29m (Proteintech; Cat#24728-1-AP); Rabbit polyclonal anti-bL31m (Proteintech; Cat#17679-1-AP); Rabbit polyclonal anti-bL32m (homemade; provided by P. Rehling); Rabbit polyclonal anti-mL38 (Proteintech; Cat#15913-1-AP); Rabbit polyclonal anti-mL39 (homemade; provided by P. Rehling); Rabbit polyclonal anti-mL40 (Novusbio; Cat#NBP1-82620); Rabbit polyclonal anti-mL43 (Proteintech; Cat#17477-1-AP); Rabbit polyclonal anti-mL44 (Proteintech; Cat#16394-1-AP); Rabbit polyclonal anti-mL45 (Proteintech; Cat#15682-1-AP); Rabbit polyclonal anti-mL46 (Proteintech; Cat#16611-1-AP); Rabbit polyclonal anti-mL48 (Proteintech; Cat#14677-1-AP); Rabbit polyclonal anti-mL50 (Invitrogen; Cat#PA5-54638); Rabbit polyclonal anti-mL52 (Proteintech; Cat#16800-1-AP); Rabbit polyclonal anti-mL53 (Proteintech; Cat#16142-1-AP); Rabbit polyclonal anti-mL54 (Proteintech; Cat#17683-1-AP); Rabbit polyclonal anti-mL62 (Proteintech; Cat#10403-1-AP); Rabbit polyclonal anti-mL64 (Proteintech; Cat#16260-1-AP); Mouse monoclonal anti-Calnexin (Proteintech; Cat#66903-1-Ig, clone 2A2C6); Rabbit polyclonal anti-COX1 (homemade; provided by P. Rehling); Rabbit polyclonal anti-POLRMT (Proteintech; Cat#17748-1-AP). Mouse monoclonal anti-SDHA (ThermoFisher Scientific, Cat#459200, clone clone 2E3GC12FB2AE2)

Validation

Commercially supplied antibodies were validated by manufacturers by subjecting lysates of multiple human cell lines (e.g. HEK, HeLa, HepG2) or human tissue (e.g. liver) to SDS-PAGE followed by immunoblotting using the respective antibodies. Additionally, all antibodies for mitoribosomal proteins used in this study follow the expected behavior, meaning detection of ribosomal proteins i.) co-migrating with the mitoribosomal particles in sucrose gradients, and ii.) co-purifying during FLAG-immunoprecipitation of mitoribosome complexes.
Homemade antibodies were validated in previous studies (Rabbit polyclonal anti-uL1m, Rabbit polyclonal anti-uL23m and Rabbit polyclonal anti-COX: Richter-Dennerlein et al., 2016; Rabbit polyclonal anti-bL32m: Lavdovskaia et al., 2018). Rabbit polyclonal anti-mL39 was validated by western blot using cell line overexpressing FLAG-tagged mL39 (HEK293-Flp-In T-Rex mL39FLAG, see Extended Data Fig.10a).

Eukaryotic cell lines

Policy information about [cell lines and Sex and Gender in Research]

Cell line source(s)

HEK293-Flp-In T-Rex (Thermo Fisher Scientific; R78007); HEK293-Flp-In T-Rex uL4m-/-, HEK293-Flp-In T-Rex bL20m-/-, HEK293-Flp-In T-Rex mL44-/-, HEK293-Flp-In T-Rex mL45-/-, HEK293-Flp-In T-Rex mL62-/-, HEK293-Flp-In T-Rex uS7m-/-, and HEK293-Flp-In T-Rex mS40-/- cell lines were generated using CRISPR/Cas9 technology; HEK293-Flp-In T-Rex uL10m-FLAG, HEK293-Flp-In T-Rex uL11m-FLAG, HEK293-Flp-In T-Rex bL12m-FLAG, HEK293-Flp-In T-Rex bL31m-FLAG, HEK293-Flp-In T-Rex mL39-FLAG, HEK293-Flp-In T-Rex mL44-FLAG, HEK293-Flp-In T-Rex mL62-FLAG, HEK293-Flp-In T-Rex bS1m-FLAG, HEK293-Flp-In T-Rex uS10m-FLAG, HEK293-Flp-In T-Rex mS22-FLAG, HEK293-Flp-In T-Rex mS25-FLAG, HEK293-Flp-In T-Rex mS27-FLAG, and HEK293-Flp-In T-Rex mS40-FLAG cell lines were generated by co-transfection of the maternal HEK293-Flp-In T-Rex WT cell line with pcDNA5/FRT/TO bearing the respective FLAG-tagged MRP nucleotide sequence and pOG44 Flp-Recombinase Expression Vector.

Authentication

HEK293-Flp-In T-Rex cell lines were routinely treated with Blasticidin S to ensure the authentic presence of the Blasticidin S-resistance locus. Stable insertion of the FLAG-tagged MRPs into the T-Rex expression cassette were confirmed by western

	blot using mouse monoclonal anti-FLAG (Sigma-Aldrich) antibody. The cell lines expressing FLAG-tagged MRPs were routinely treated with Hygromycin B to ensure the stability of the insertion. Knockout cell lines were confirmed by western blotting and Sanger gDNA sequencing.
Mycoplasma contamination	Cell Lines used in this study were systematically tested negative for the presence of Mycoplasma by GATC Biotech.
Commonly misidentified lines (See ICLAC register)	No commonly misidentified cell lines were used.

Plants

Seed stocks	*Report on the source of all seed stocks or other plant material used. If applicable, state the seed stock centre and catalogue number. If plant specimens were collected from the field, describe the collection location, date and sampling procedures.*
Novel plant genotypes	*Describe the methods by which all novel plant genotypes were produced. This includes those generated by transgenic approaches, gene editing, chemical/radiation-based mutagenesis and hybridization. For transgenic lines, describe the transformation method, the number of independent lines analyzed and the generation upon which experiments were performed. For gene-edited lines, describe the editor used, the endogenous sequence targeted for editing, the targeting guide RNA sequence (if applicable) and how the editor was applied.*
Authentication	*Describe any authentication procedures for each seed stock used or novel genotype generated. Describe any experiments used to assess the effect of a mutation and, where applicable, how potential secondary effects (e.g. second site T-DNA insertions, mosiacism, off-target gene editing) were examined.*
