# Supplementary material for: A roadmap for ribosome assembly in human mitochondria
Source: Nat Struct Mol Biol. 2024 Jul 11;31(12):1898–908. doi: 10.1038/s41594-024-01356-w (PMC11638073; doi:10.1038/s41594-024-01356-w)
Supplement: Supplementary file 4 — Inference of global MRP and assembly factor turnover. For each MRP, MS1 intensities and normalized MS1 intensities for H, M and L labeled MRPs and assembly factors in mitochondria and cell lysate, respectively, are shown. Raw MS data, model fits of normalized data and posterior distribution of model parameters are shown for all modeled proteins. [file 41594_2024_1356_MOESM4_ESM.pdf]

uL1m
MRPL1
Q9BYD6
39S ribosomal protein L1, mitochondrial

Cell lysate

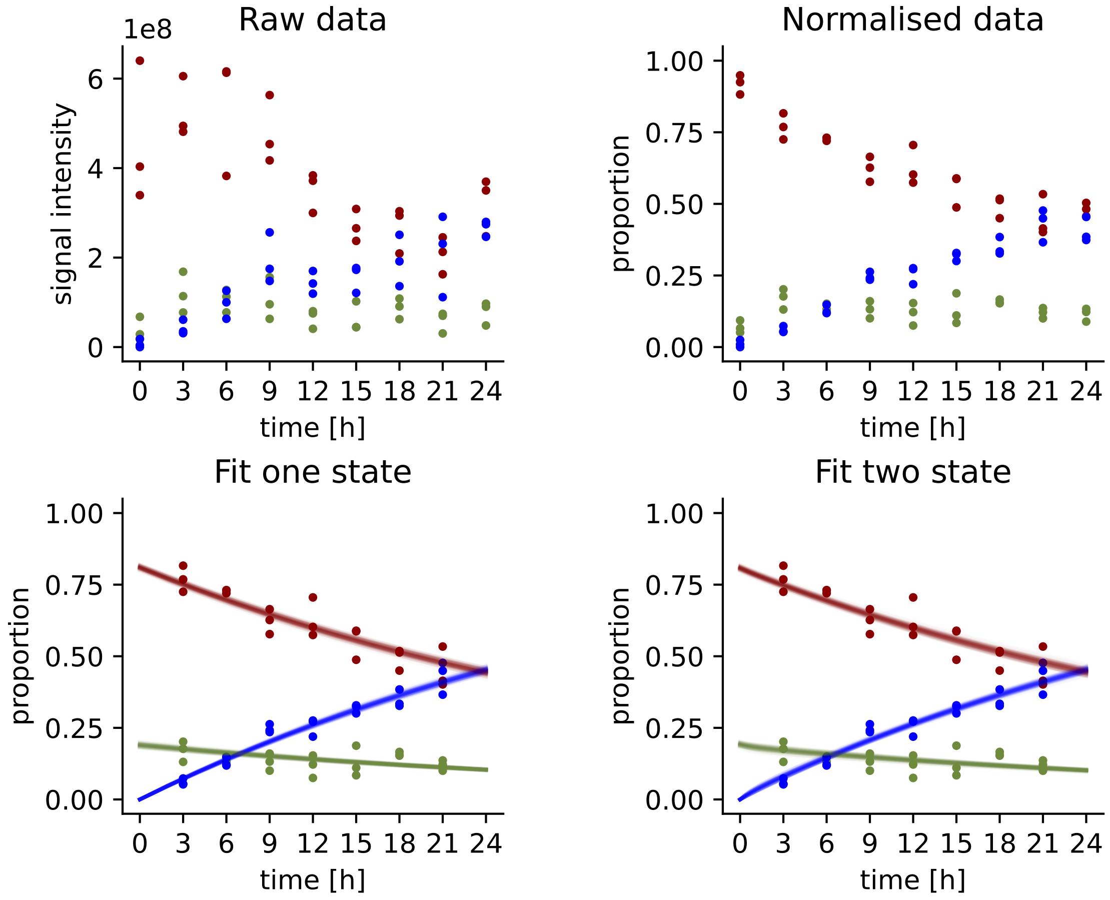

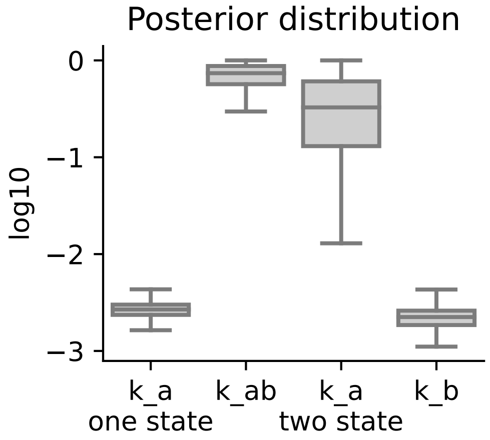

p value	9.95E-01
log likelihood one state	115.884
log likelihood two state	115.889
rmse one state	0.291
rmse two state	0.29
k_a one state	0.003
k_ab	0.738
k_a two state	0.327
k_b	0.002
sigma one state	0.039
sigma two state	0.039

Mitochondria

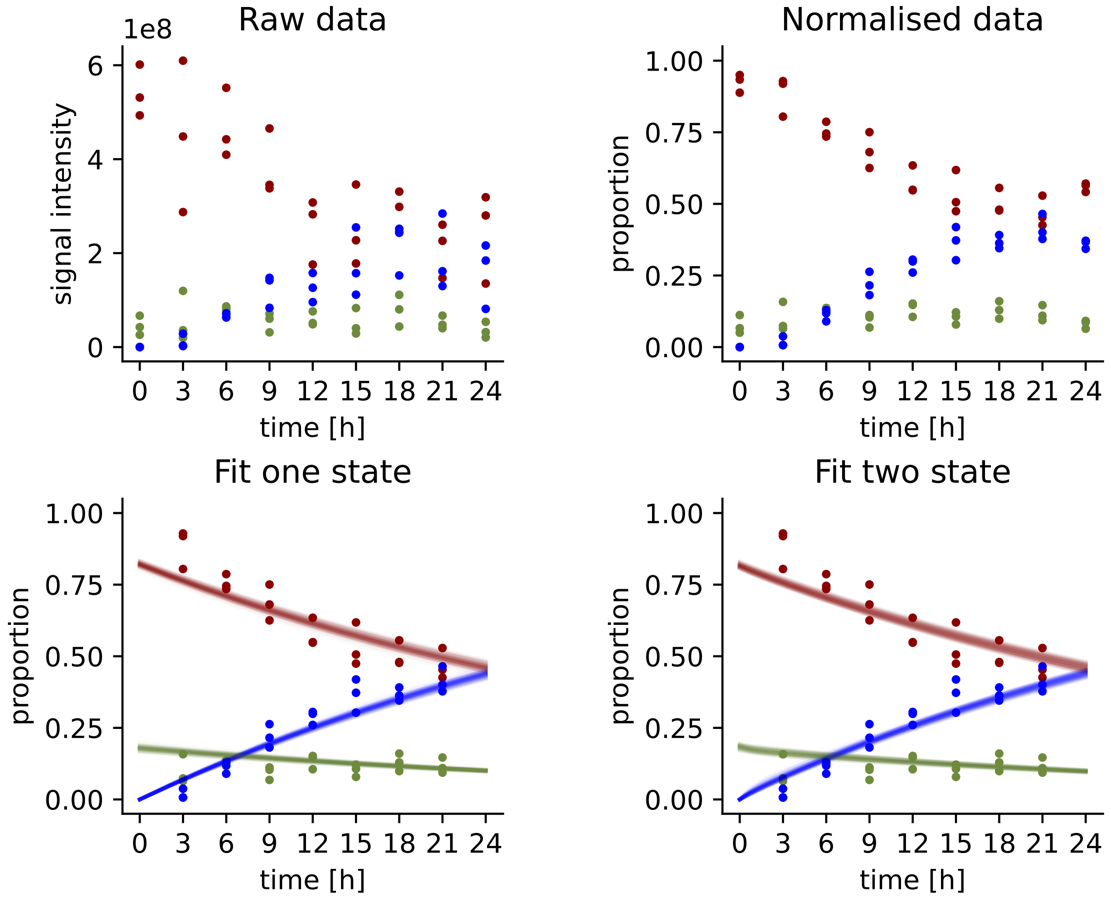

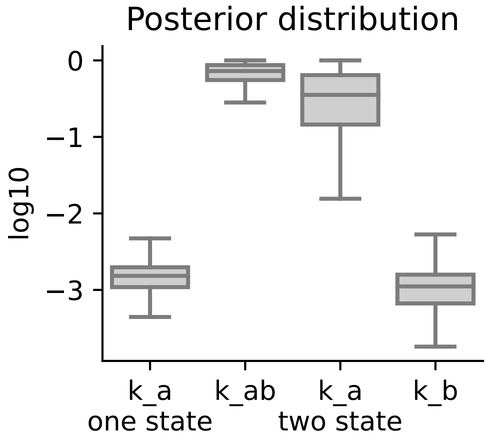

p value	9.81E-01
log likelihood one state	92.671
log likelihood two state	92.69
rmse one state	0.311
rmse two state	0.311
k_a one state	0.002
k_ab	0.725
k_a two state	0.354
k_b	0.001
sigma one state	0.057
sigma two state	0.057

uL2m
MRPL2
Q5T653
39S ribosomal protein L2, mitochondrial

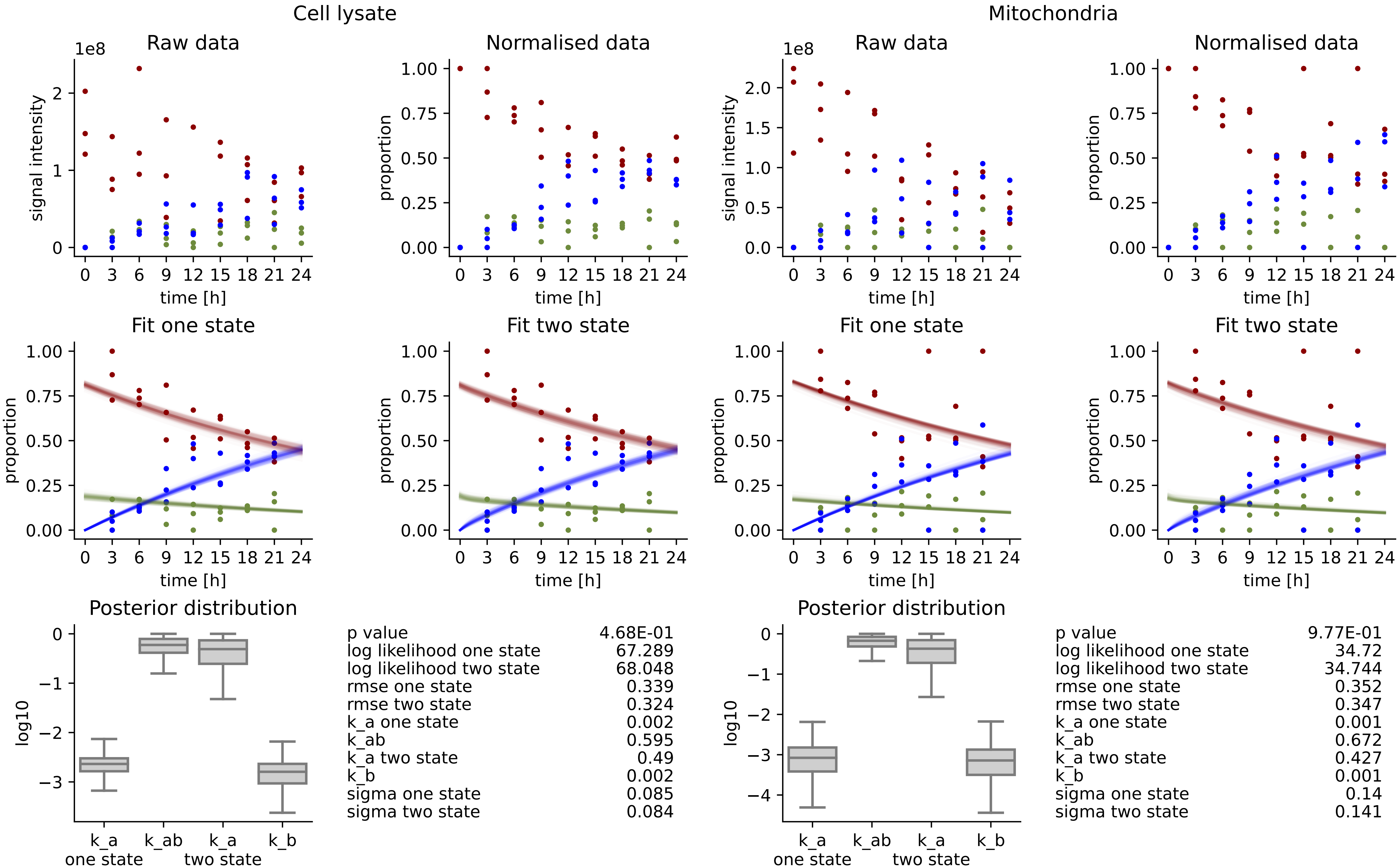

p value	4.68E-01
log likelihood one state	67.289
log likelihood two state	68.048
rmse one state	0.339
rmse two state	0.324
k_a one state	0.002
k_ab	0.595
k_a two state	0.49
k_b	0.002
sigma one state	0.085
sigma two state	0.084

p value	9.77E-01
log likelihood one state	34.72
log likelihood two state	34.744
rmse one state	0.352
rmse two state	0.347
k_a one state	0.001
k_ab	0.672
k_a two state	0.427
k_b	0.001
sigma one state	0.14
sigma two state	0.141

uL3m
MRPL3
P09001
39S ribosomal protein L3, mitochondrial

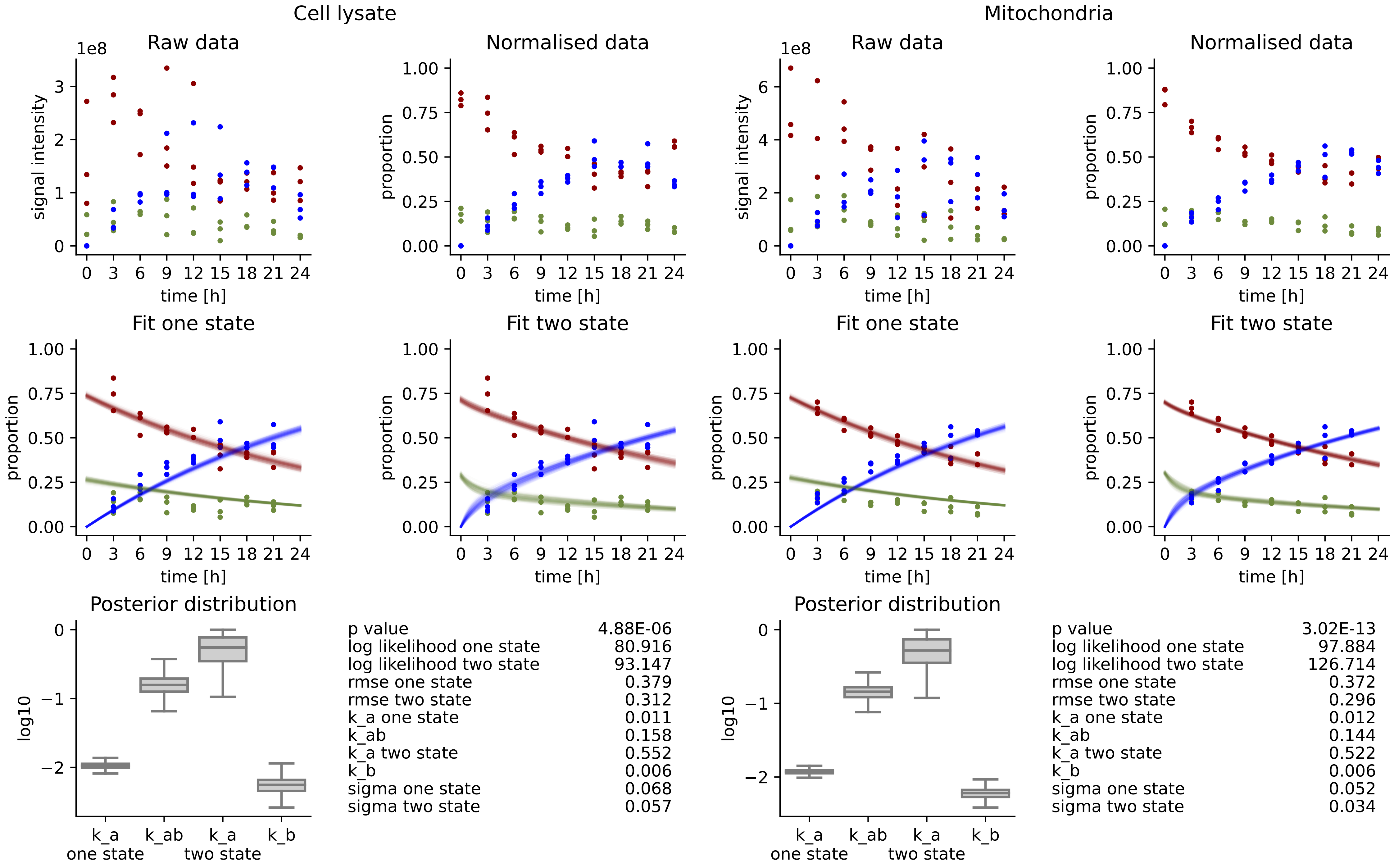

Cell lysate

p value	4.88E-06
log likelihood one state	80.916
log likelihood two state	93.147
rmse one state	0.379
rmse two state	0.312
k_a one state	0.011
k_ab	0.158
k_a two state	0.552
k_b	0.006
sigma one state	0.068
sigma two state	0.057

Mitochondria

p value	3.02E-13
log likelihood one state	97.884
log likelihood two state	126.714
rmse one state	0.372
rmse two state	0.296
k_a one state	0.012
k_ab	0.144
k_a two state	0.522
k_b	0.006
sigma one state	0.052
sigma two state	0.034

uL4m
MRPL4
Q9BYD3
39S ribosomal protein L4, mitochondrial

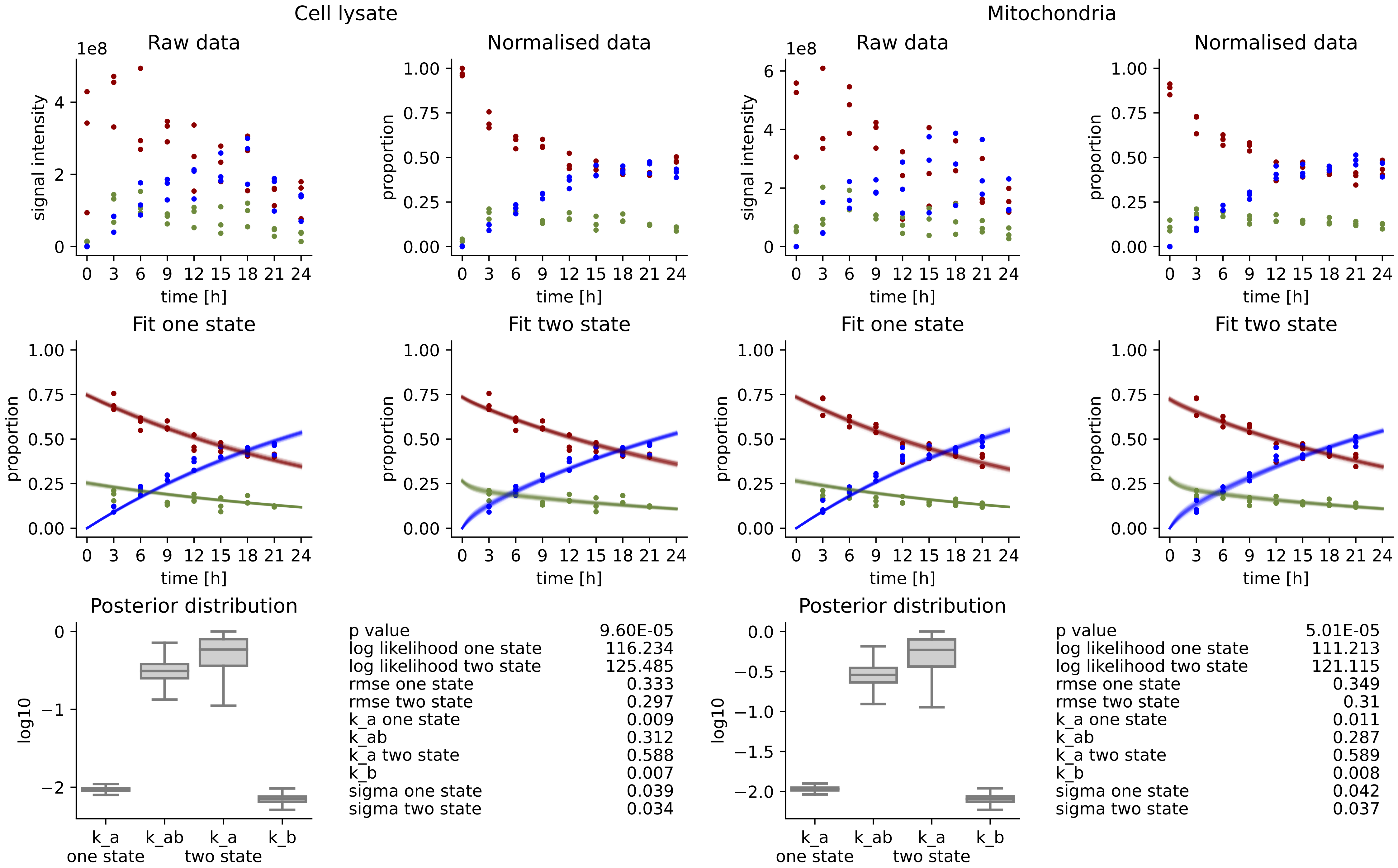

bL9m
MRPL9
Q9BYD2
39S ribosomal protein L9, mitochondrial

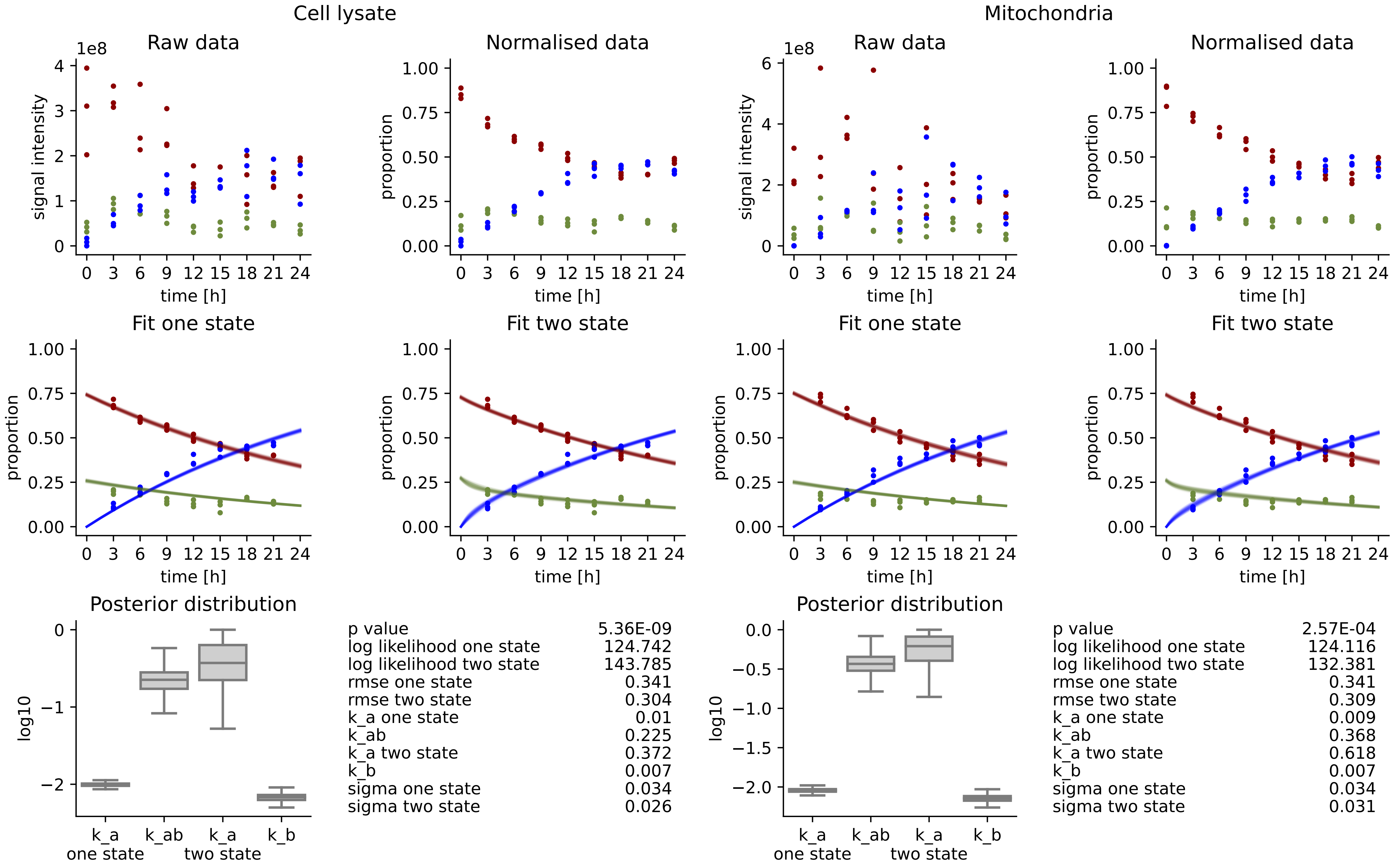

uL10m
MRPL10
Q7Z7H8
39S ribosomal protein L10, mitochondrial

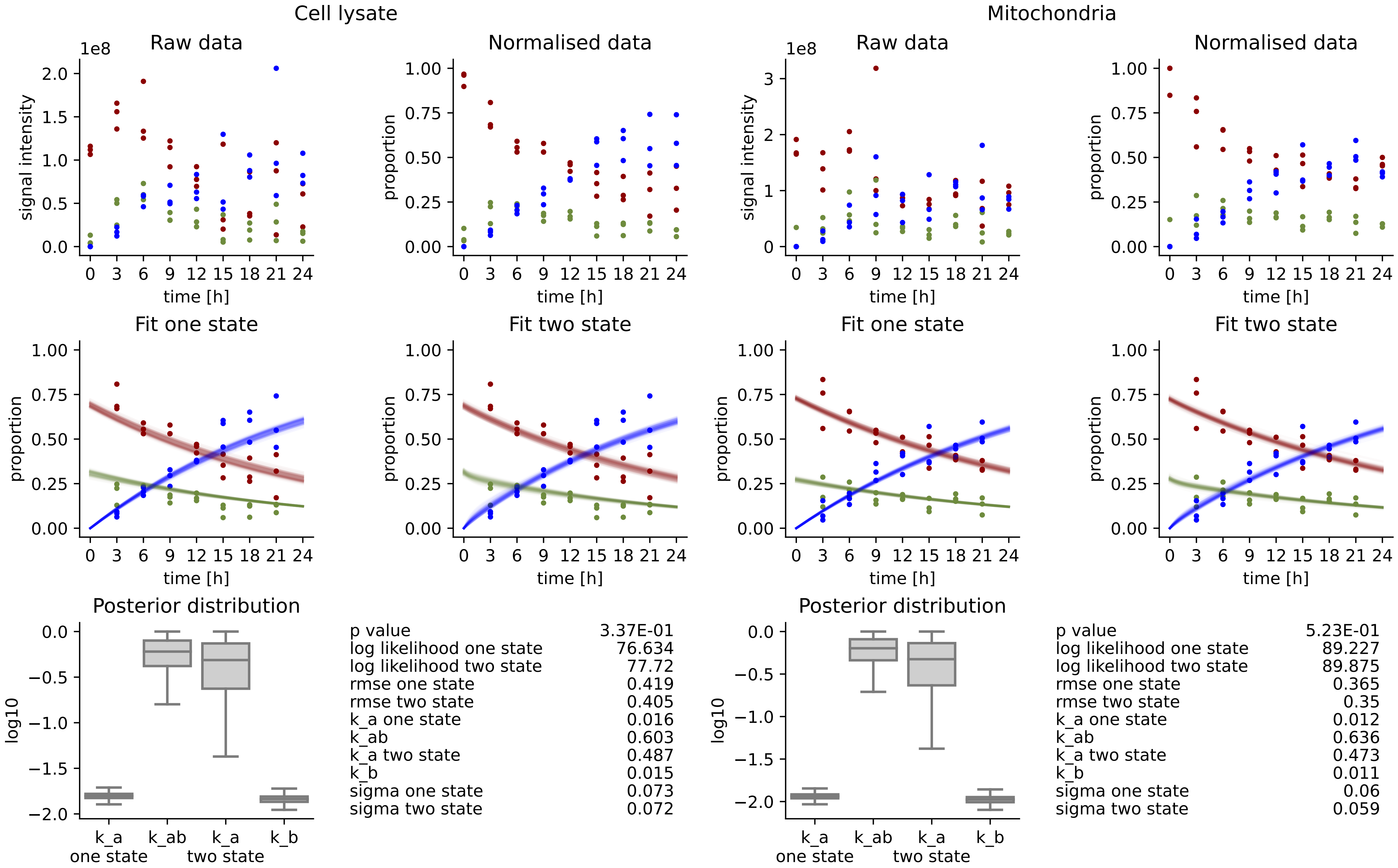

Cell lysate

p value	3.37E-01
log likelihood one state	76.634
log likelihood two state	77.72
rmse one state	0.419
rmse two state	0.405
k_a one state	0.016
k_ab	0.603
k_a two state	0.487
k_b	0.015
sigma one state	0.073
sigma two state	0.072

Mitochondria

p value	5.23E-01
log likelihood one state	89.227
log likelihood two state	89.875
rmse one state	0.365
rmse two state	0.35
k_a one state	0.012
k_ab	0.636
k_a two state	0.473
k_b	0.011
sigma one state	0.06
sigma two state	0.059

uL11m
MRPL11
Q9Y3B7
39S ribosomal protein L11, mitochondrial

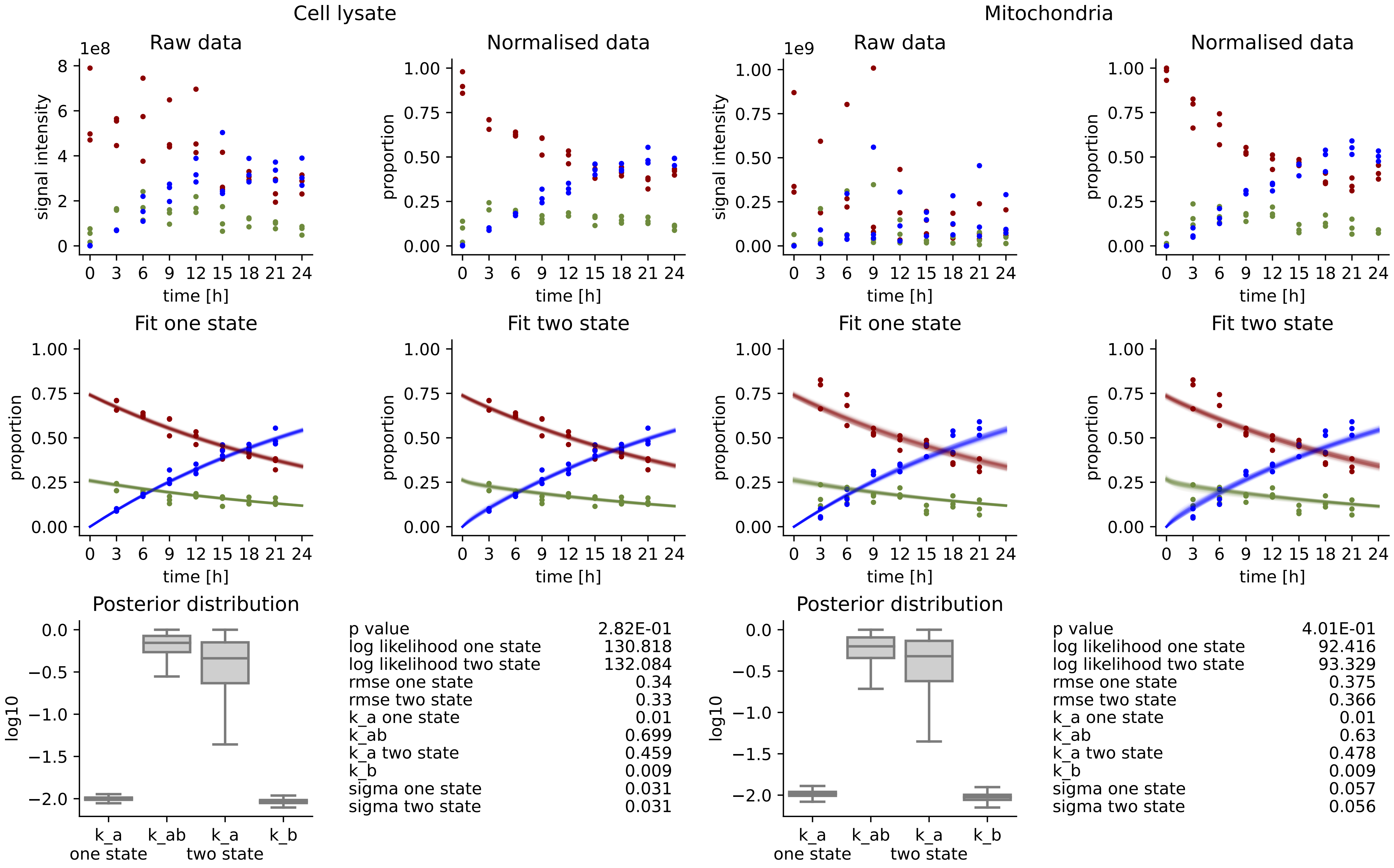

Cell lysate

p value	2.82E-01
log likelihood one state	130.818
log likelihood two state	132.084
rmse one state	0.34
rmse two state	0.33
k_a one state	0.01
k_ab	0.699
k_a two state	0.459
k_b	0.009
sigma one state	0.031
sigma two state	0.031

Mitochondria

p value	4.01E-01
log likelihood one state	92.416
log likelihood two state	93.329
rmse one state	0.375
rmse two state	0.366
k_a one state	0.01
k_ab	0.63
k_a two state	0.478
k_b	0.009
sigma one state	0.057
sigma two state	0.056

bL12m
MRPL12
P52815
39S ribosomal protein L12, mitochondrial

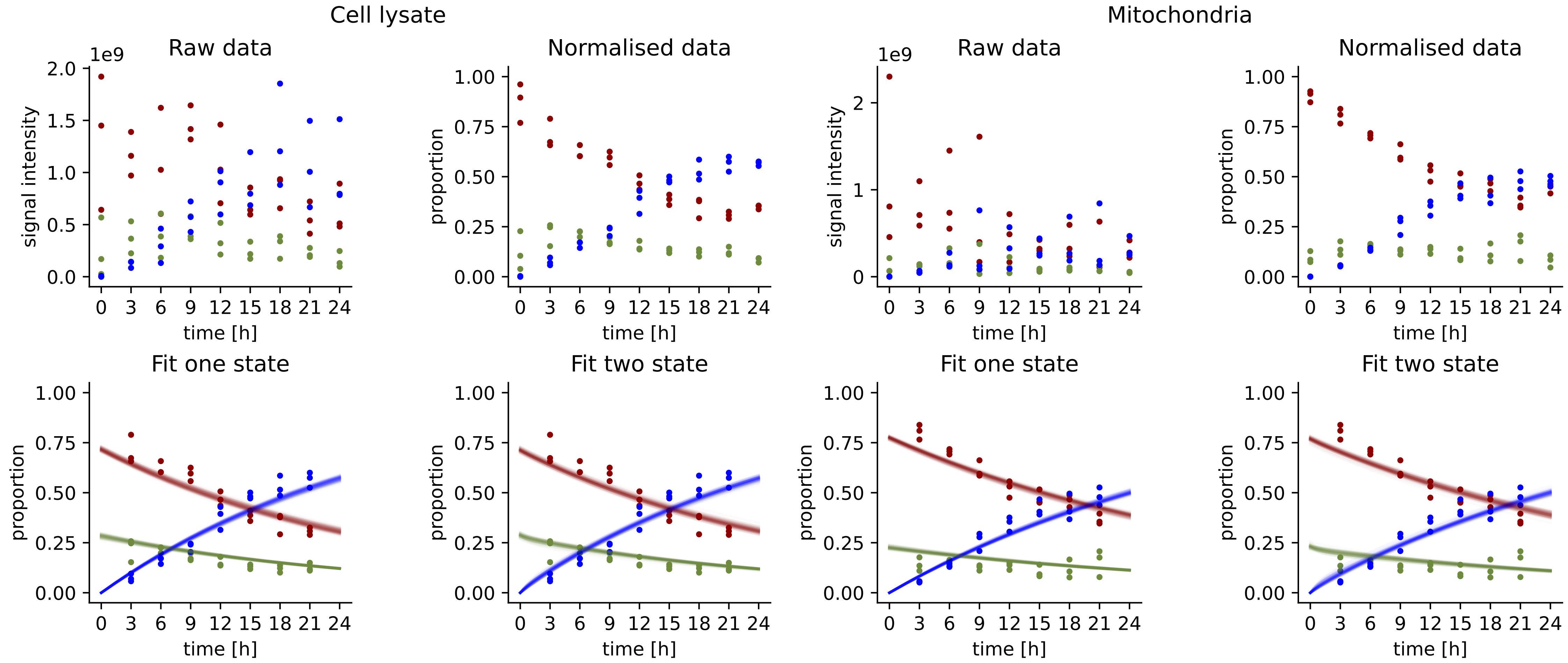

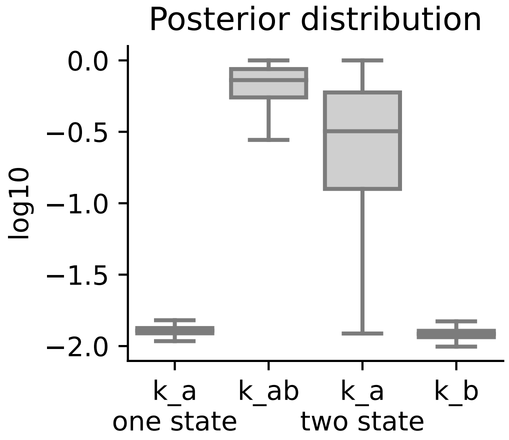

p value	8.94E-01
log likelihood one state	99.442
log likelihood two state	99.555
rmse one state	0.385
rmse two state	0.384
k_a one state	0.013
k_ab	0.728
k_a two state	0.319
k_b	0.012
sigma one state	0.051
sigma two state	0.051

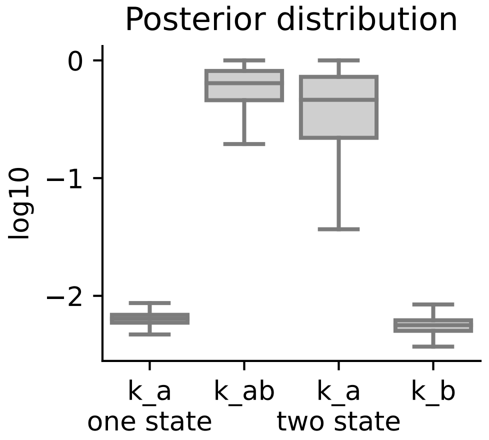

p value	5.11E-01
log likelihood one state	96.123
log likelihood two state	96.794
rmse one state	0.347
rmse two state	0.336
k_a one state	0.006
k_ab	0.64
k_a two state	0.462
k_b	0.006
sigma one state	0.054
sigma two state	0.053

uL13m
MRPL13
Q9BYD1
39S ribosomal protein L13, mitochondrial

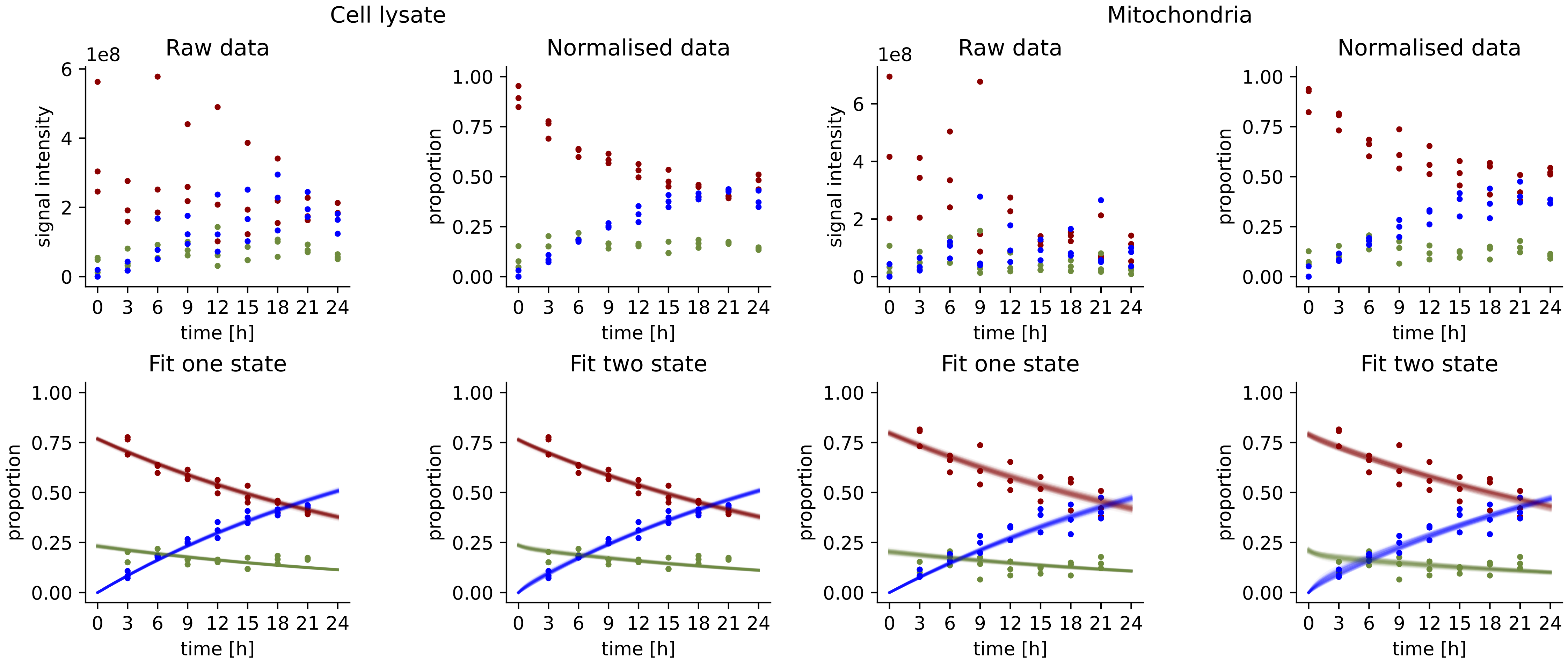

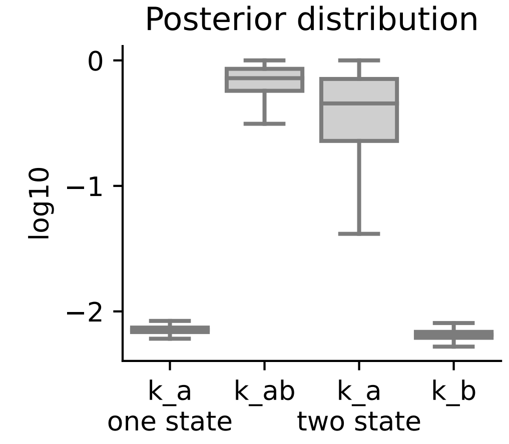

p value	4.45E-01
log likelihood one state	130.478
log likelihood two state	131.286
rmse one state	0.312
rmse two state	0.301
k_a one state	0.007
k_ab	0.721
k_a two state	0.454
k_b	0.007
sigma one state	0.031
sigma two state	0.031

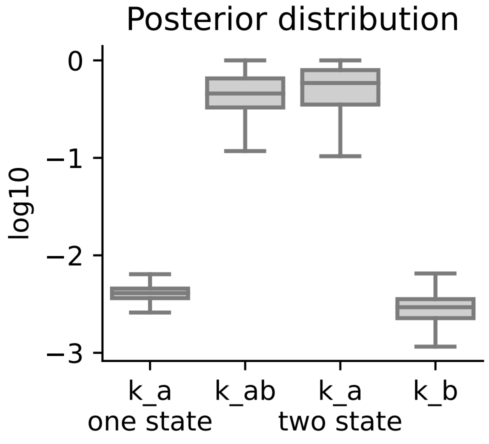

p value	7.45E-02
log likelihood one state	96.916
log likelihood two state	99.514
rmse one state	0.31
rmse two state	0.28
k_a one state	0.004
k_ab	0.457
k_a two state	0.586
k_b	0.003
sigma one state	0.053
sigma two state	0.051

uL14m
MRPL14
Q6P1L8
39S ribosomal protein L14, mitochondrial

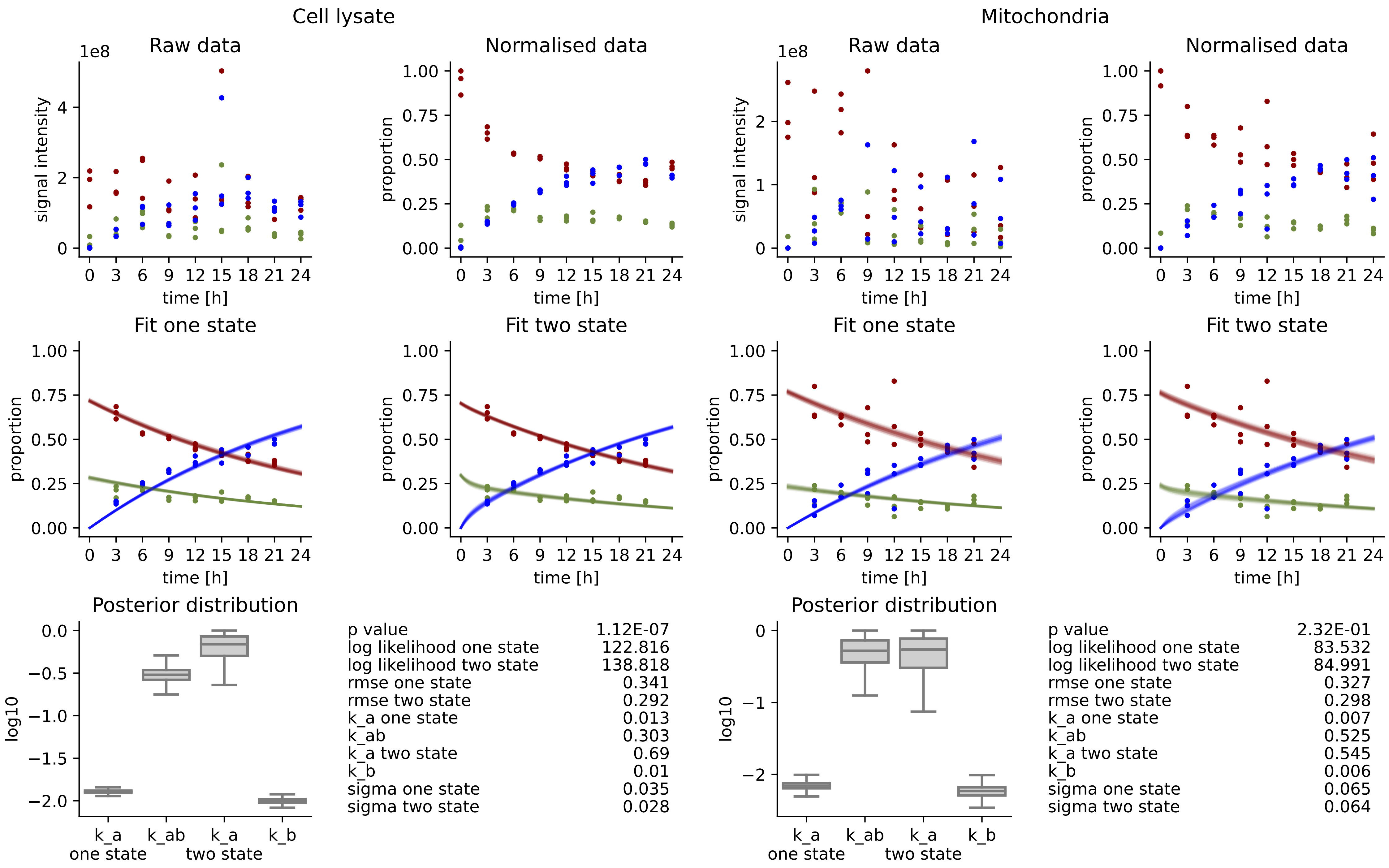

uL15m
MRPL15
Q9P015
39S ribosomal protein L15, mitochondrial

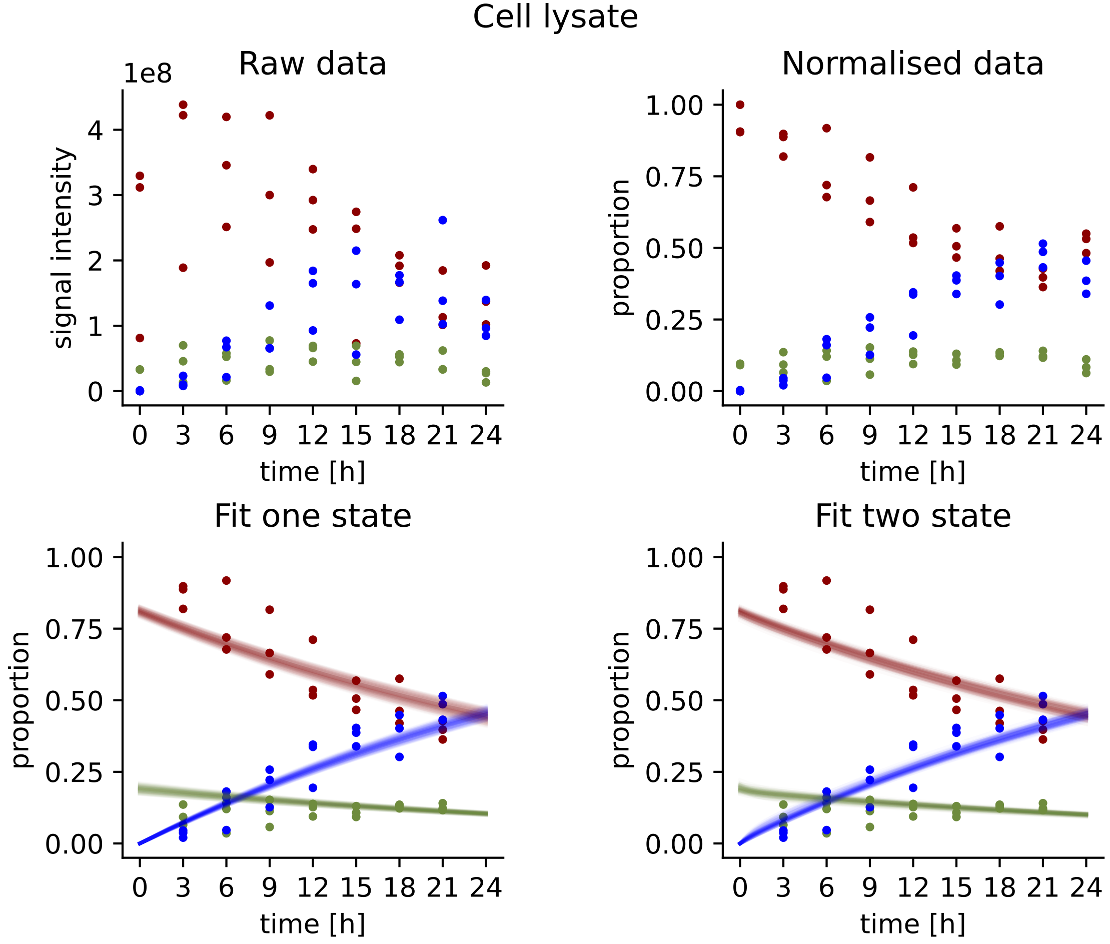

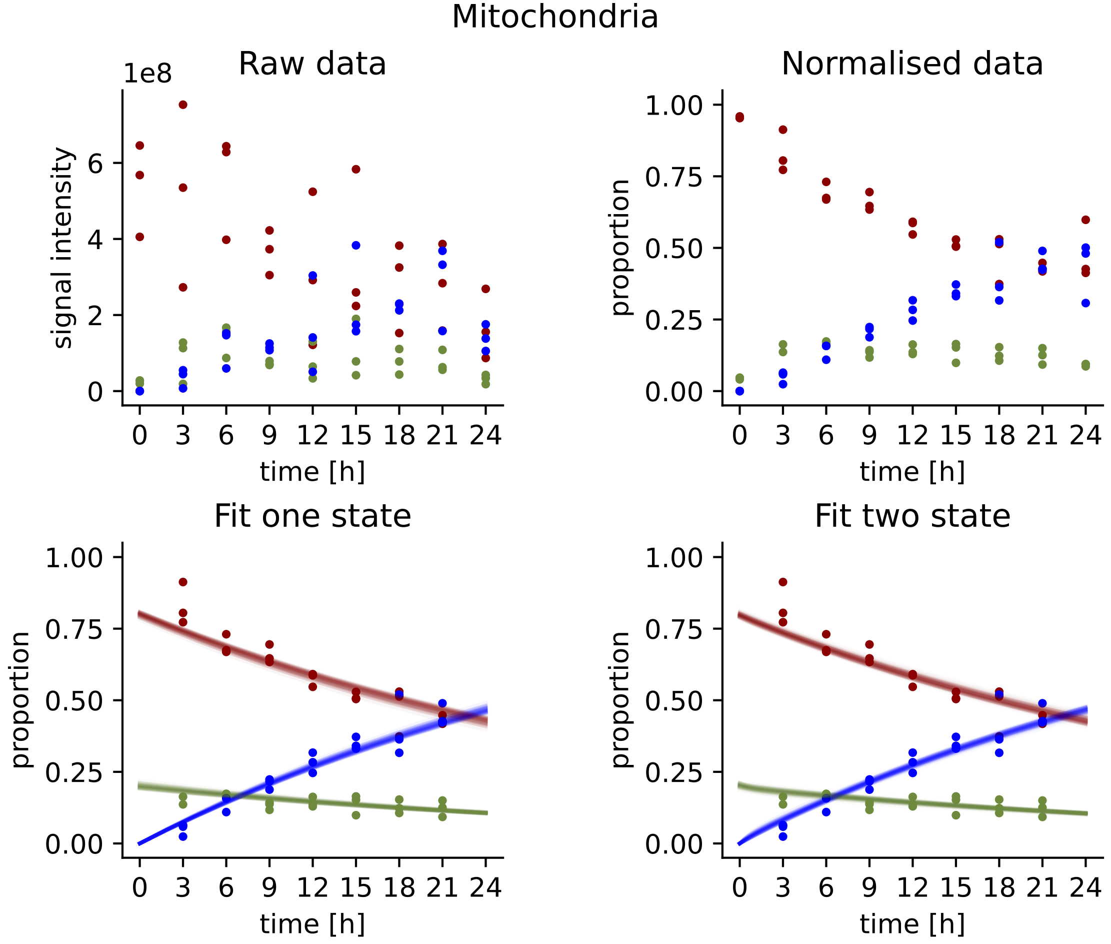

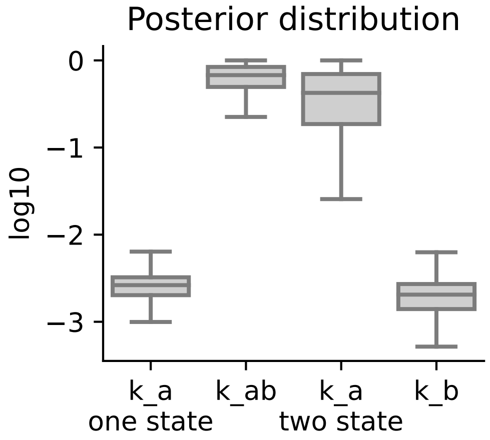

p value	9.89E-01
log likelihood one state	75.09
log likelihood two state	75.101
rmse one state	0.34
rmse two state	0.337
k_a one state	0.003
k_ab	0.677
k_a two state	0.424
k_b	0.002
sigma one state	0.075
sigma two state	0.075

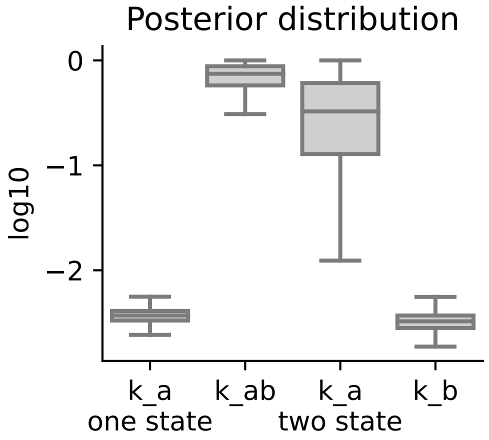

p value	9.35E-01
log likelihood one state	104.791
log likelihood two state	104.858
rmse one state	0.313
rmse two state	0.314
k_a one state	0.004
k_ab	0.745
k_a two state	0.327
k_b	0.003
sigma one state	0.047
sigma two state	0.047

uL16m
MRPL16
Q9NX20
39S ribosomal protein L16, mitochondrial

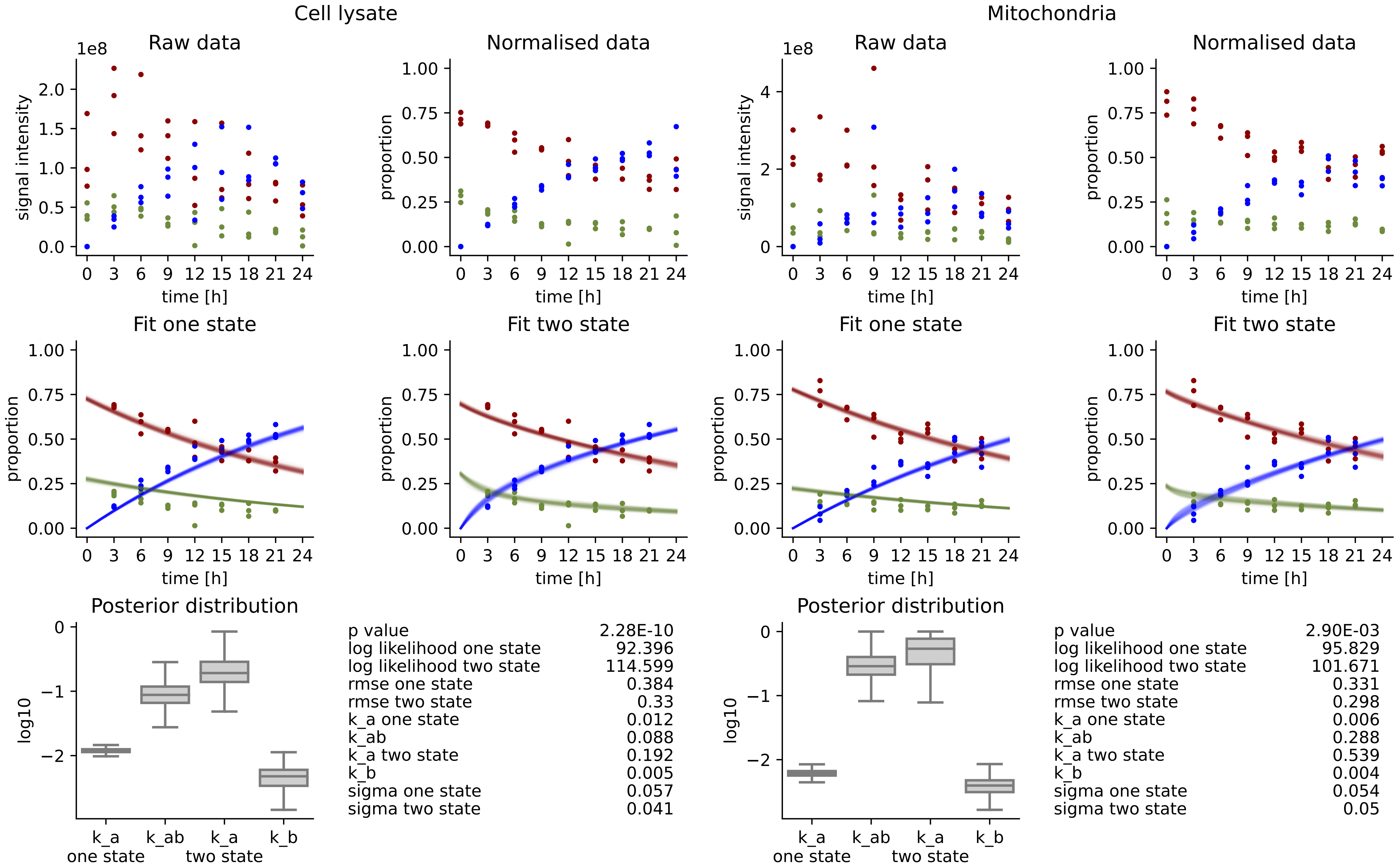

bL17m
MRPL17
Q9NRX2
39S ribosomal protein L17, mitochondrial

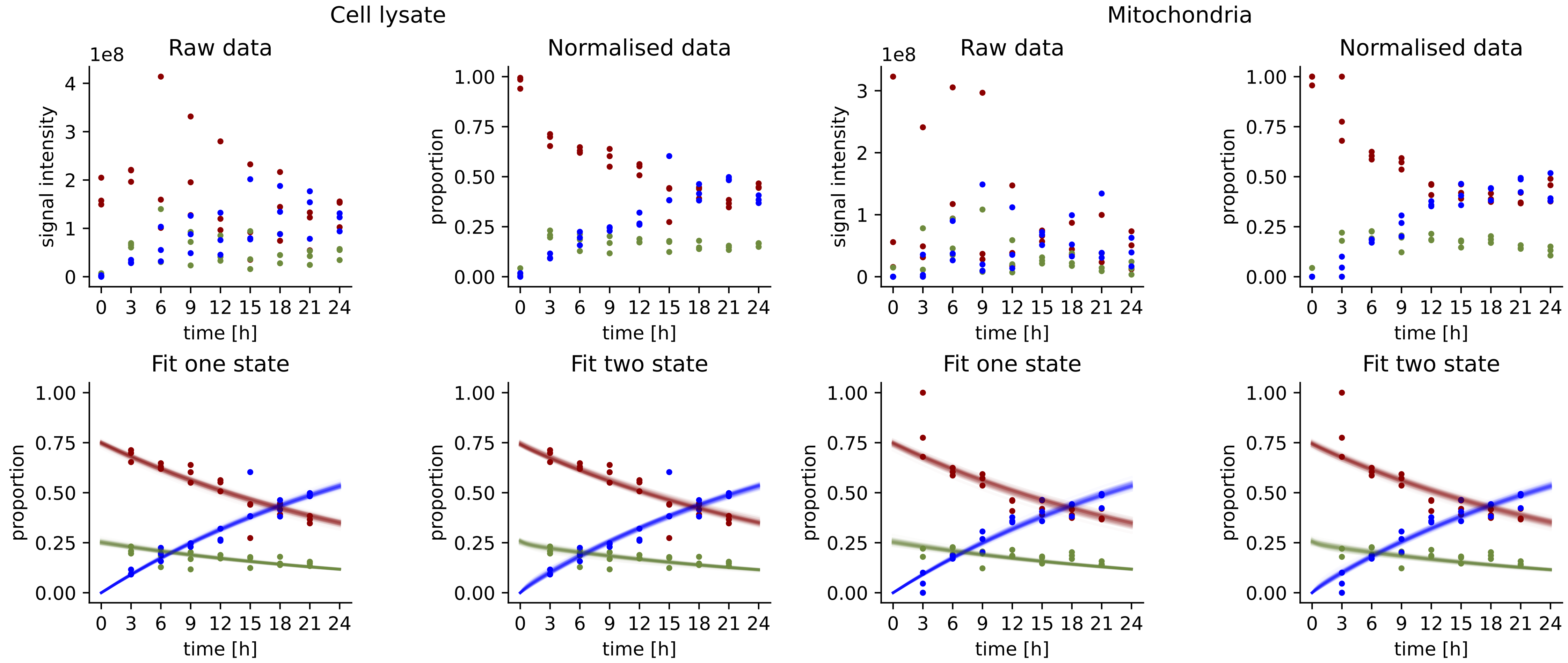

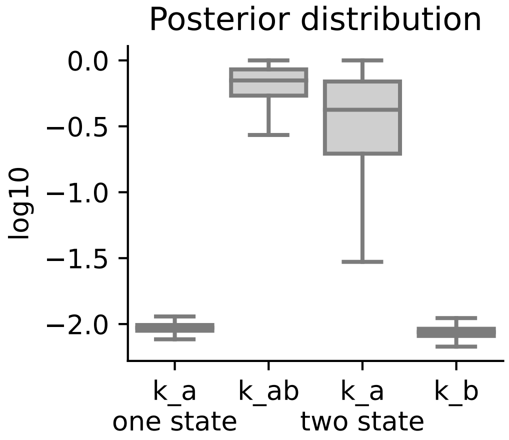

p value	7.88E-01
log likelihood one state	103.064
log likelihood two state	103.303
rmse one state	0.338
rmse two state	0.329
k_a one state	0.009
k_ab	0.704
k_a two state	0.422
k_b	0.009
sigma one state	0.048
sigma two state	0.048

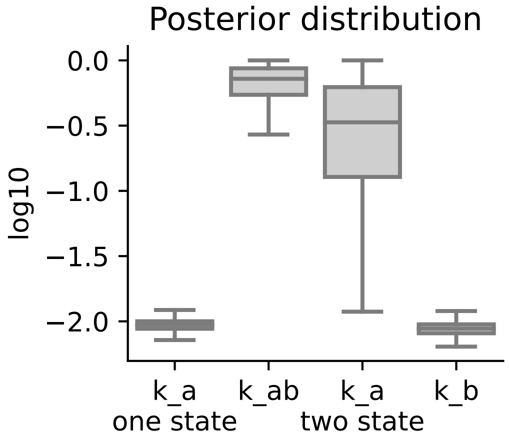

p value	6.36E-01
log likelihood one state	84.429
log likelihood two state	84.881
rmse one state	0.35
rmse two state	0.354
k_a one state	0.009
k_ab	0.723
k_a two state	0.335
k_b	0.009
sigma one state	0.064
sigma two state	0.065

uL18m
MRPL18
Q9H0U6
39S ribosomal protein L18, mitochondrial

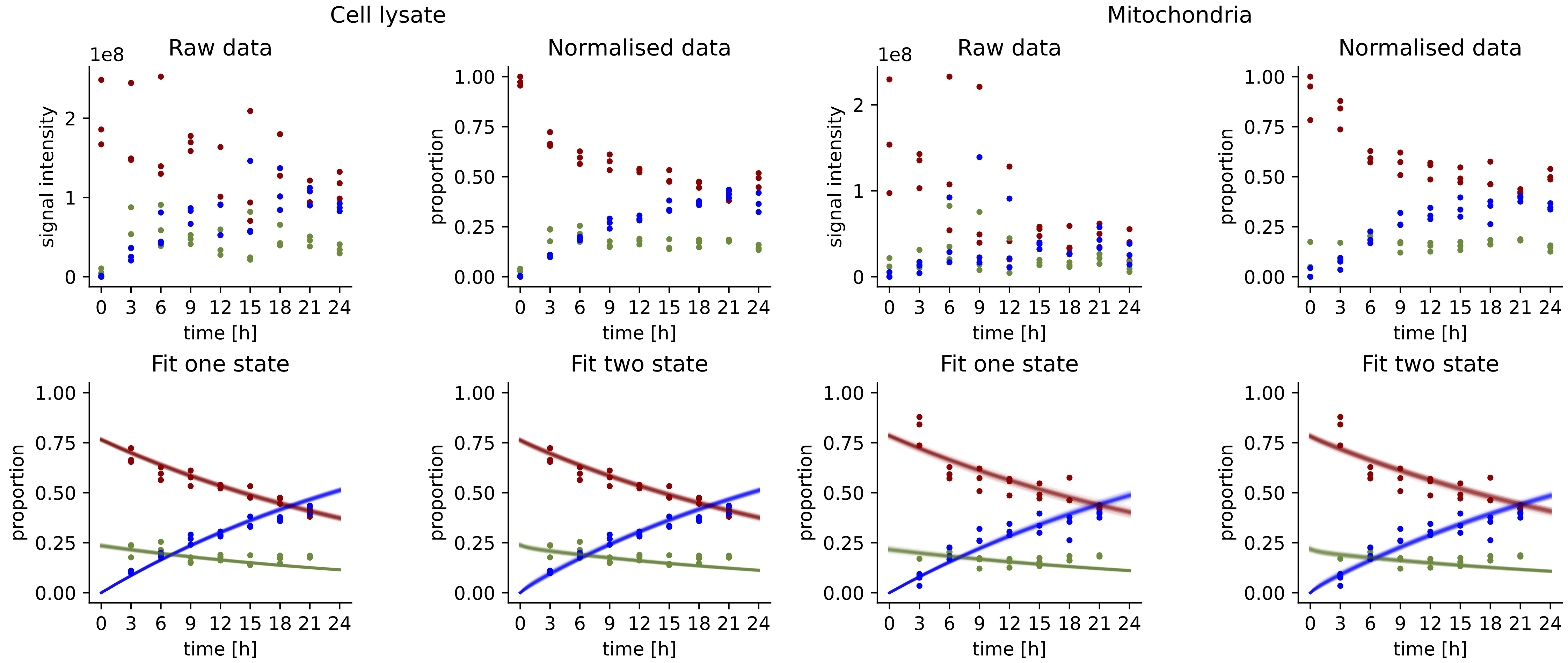

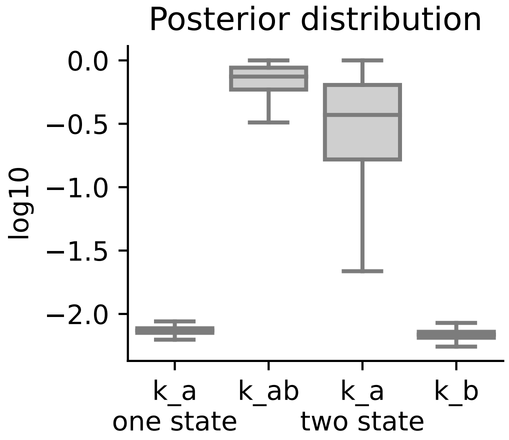

p value	7.86E-01
log likelihood one state	125.155
log likelihood two state	125.395
rmse one state	0.293
rmse two state	0.286
k_a one state	0.007
k_ab	0.745
k_a two state	0.371
k_b	0.007
sigma one state	0.034
sigma two state	0.034

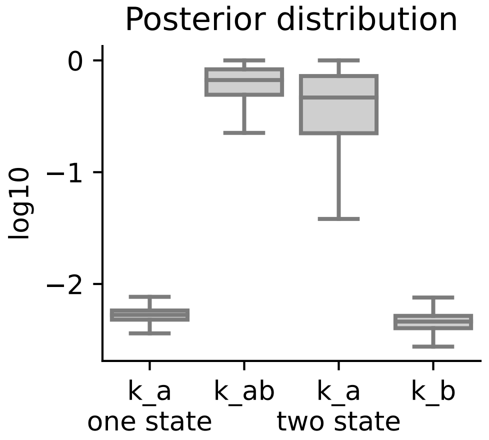

p value	7.23E-01
log likelihood one state	92.685
log likelihood two state	93.009
rmse one state	0.302
rmse two state	0.29
k_a one state	0.005
k_ab	0.667
k_a two state	0.465
k_b	0.005
sigma one state	0.057
sigma two state	0.056

bL19m
MRPL19
P49406
39S ribosomal protein L19, mitochondrial

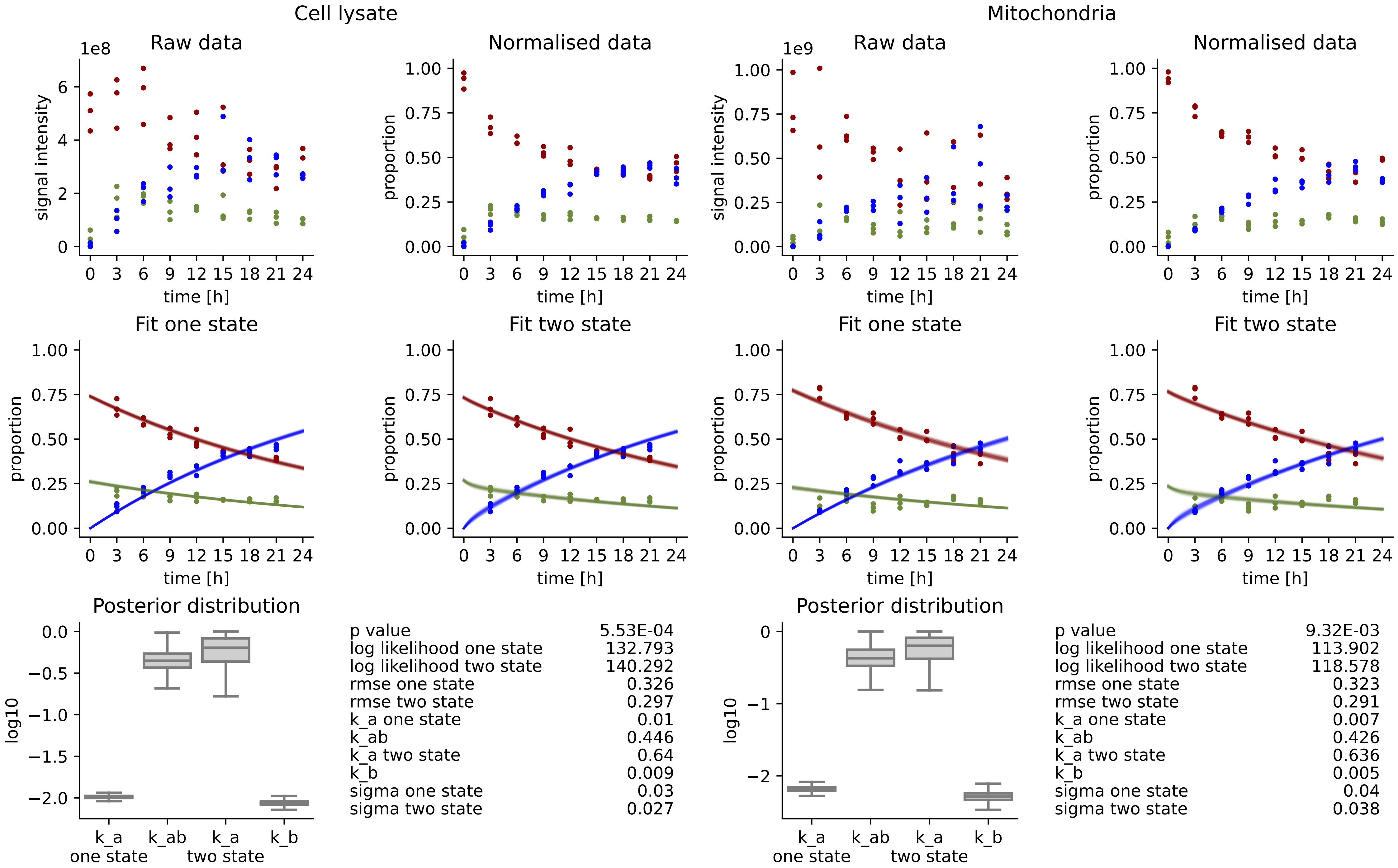

bL20m
MRPL20
Q9BYC9
39S ribosomal protein L20, mitochondrial

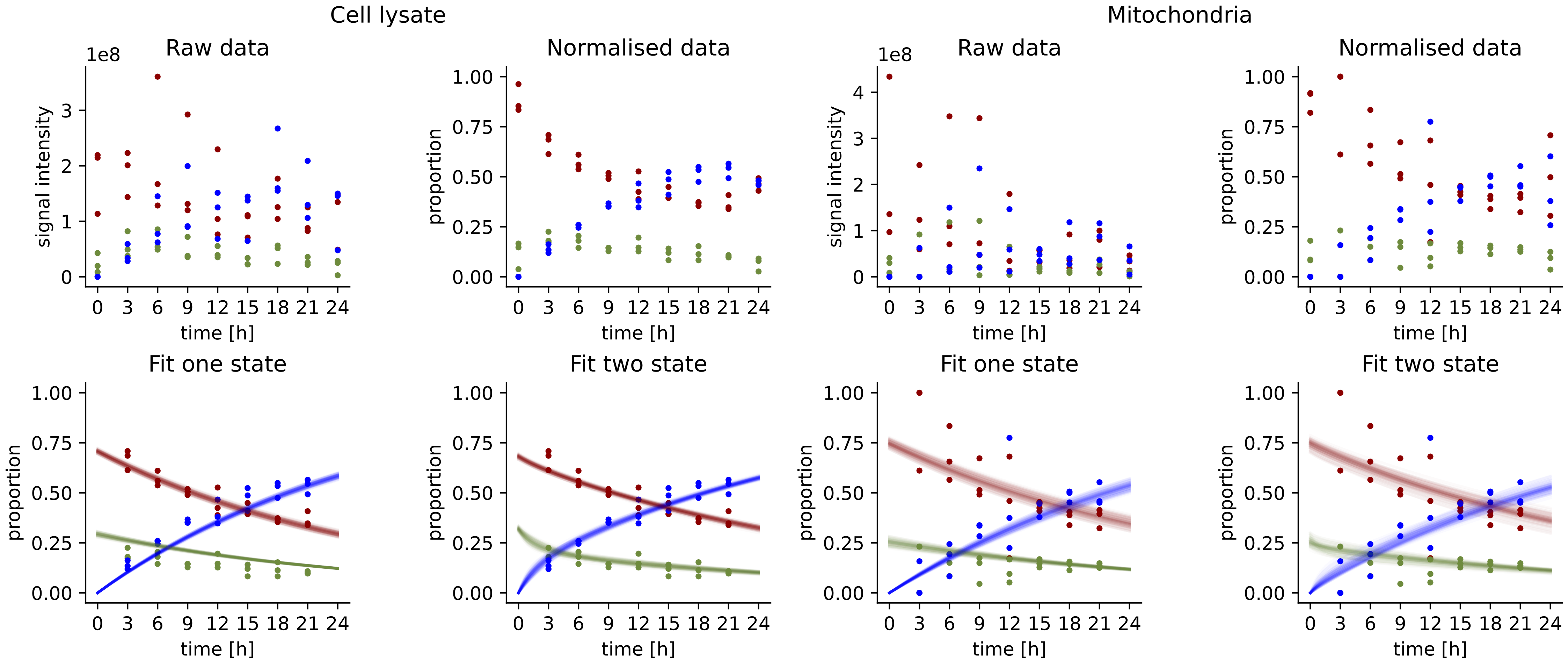

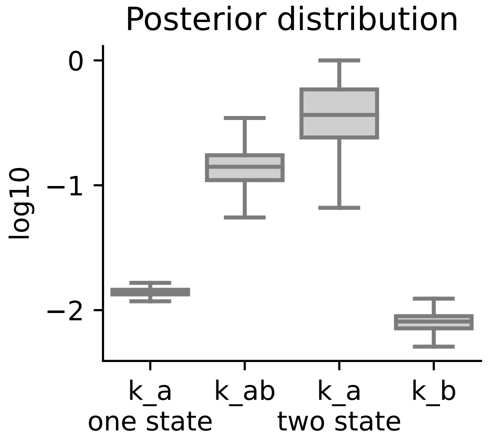

p value	1.50E-10
log likelihood one state	96.204
log likelihood two state	118.828
rmse one state	0.387
rmse two state	0.324
k_a one state	0.014
k_ab	0.141
k_a two state	0.366
k_b	0.008
sigma one state	0.054
sigma two state	0.038

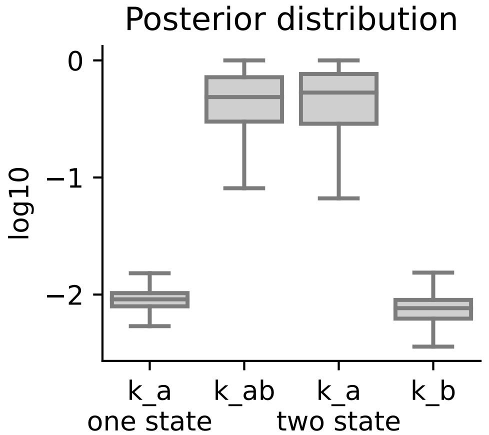

p value	3.63E-01
log likelihood one state	44.038
log likelihood two state	45.052
rmse one state	0.422
rmse two state	0.39
k_a one state	0.009
k_ab	0.486
k_a two state	0.531
k_b	0.008
sigma one state	0.121
sigma two state	0.12

bL21m
MRPL21
Q7Z2W9
39S ribosomal protein L21, mitochondrial

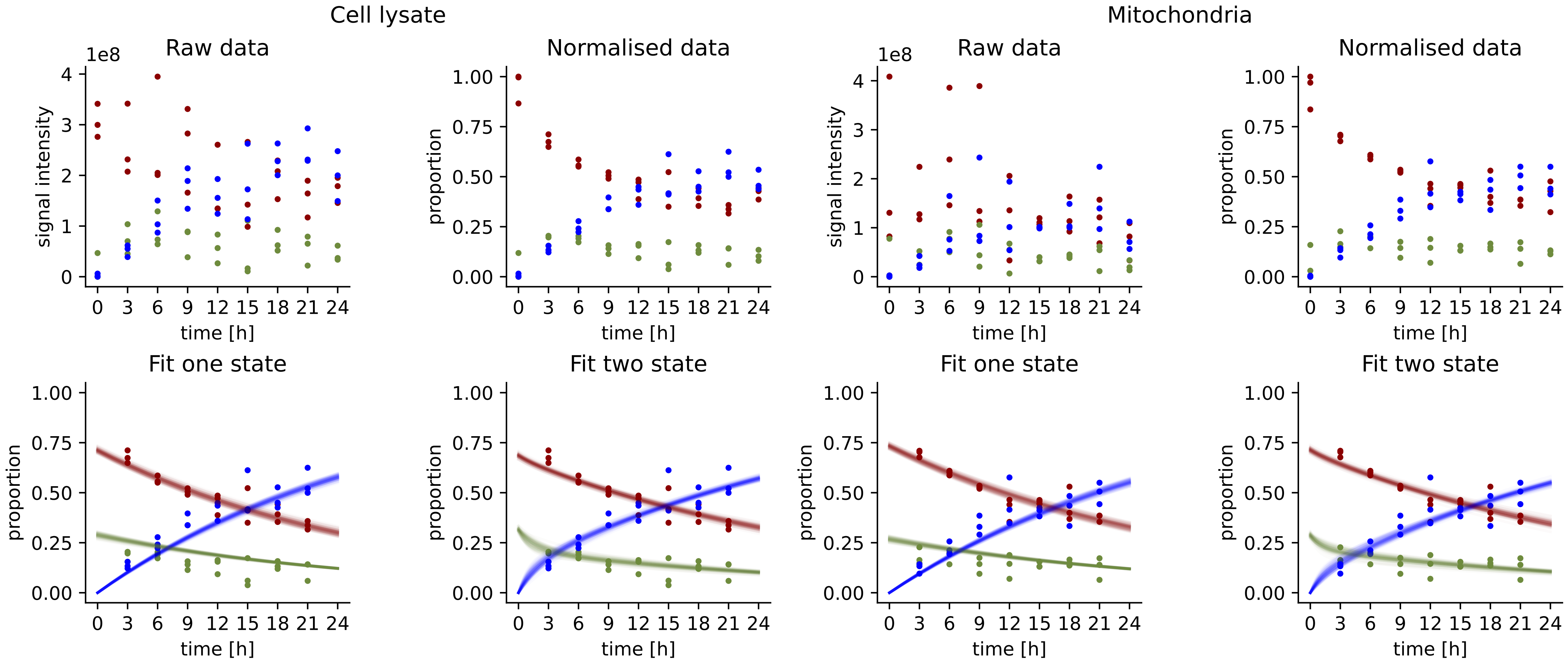

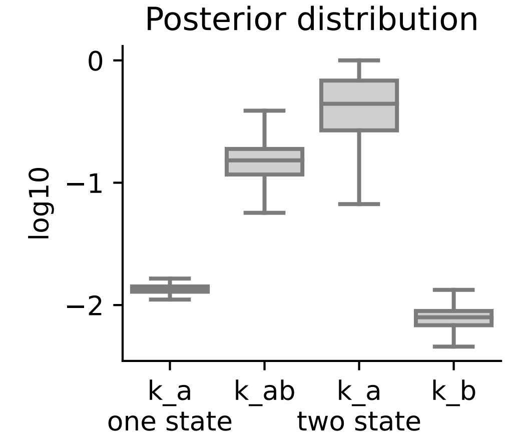

p value	1.59E-07
log likelihood one state	87.264
log likelihood two state	102.918
rmse one state	0.388
rmse two state	0.326
k_a one state	0.014
k_ab	0.152
k_a two state	0.442
k_b	0.008
sigma one state	0.062
sigma two state	0.049

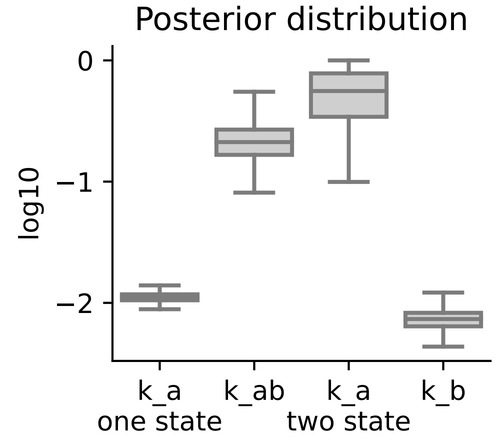

p value	1.03E-04
log likelihood one state	88.008
log likelihood two state	97.19
rmse one state	0.361
rmse two state	0.31
k_a one state	0.011
k_ab	0.212
k_a two state	0.559
k_b	0.007
sigma one state	0.061
sigma two state	0.054

uL22m
MRPL22
Q9NWU5
39S ribosomal protein L22, mitochondrial

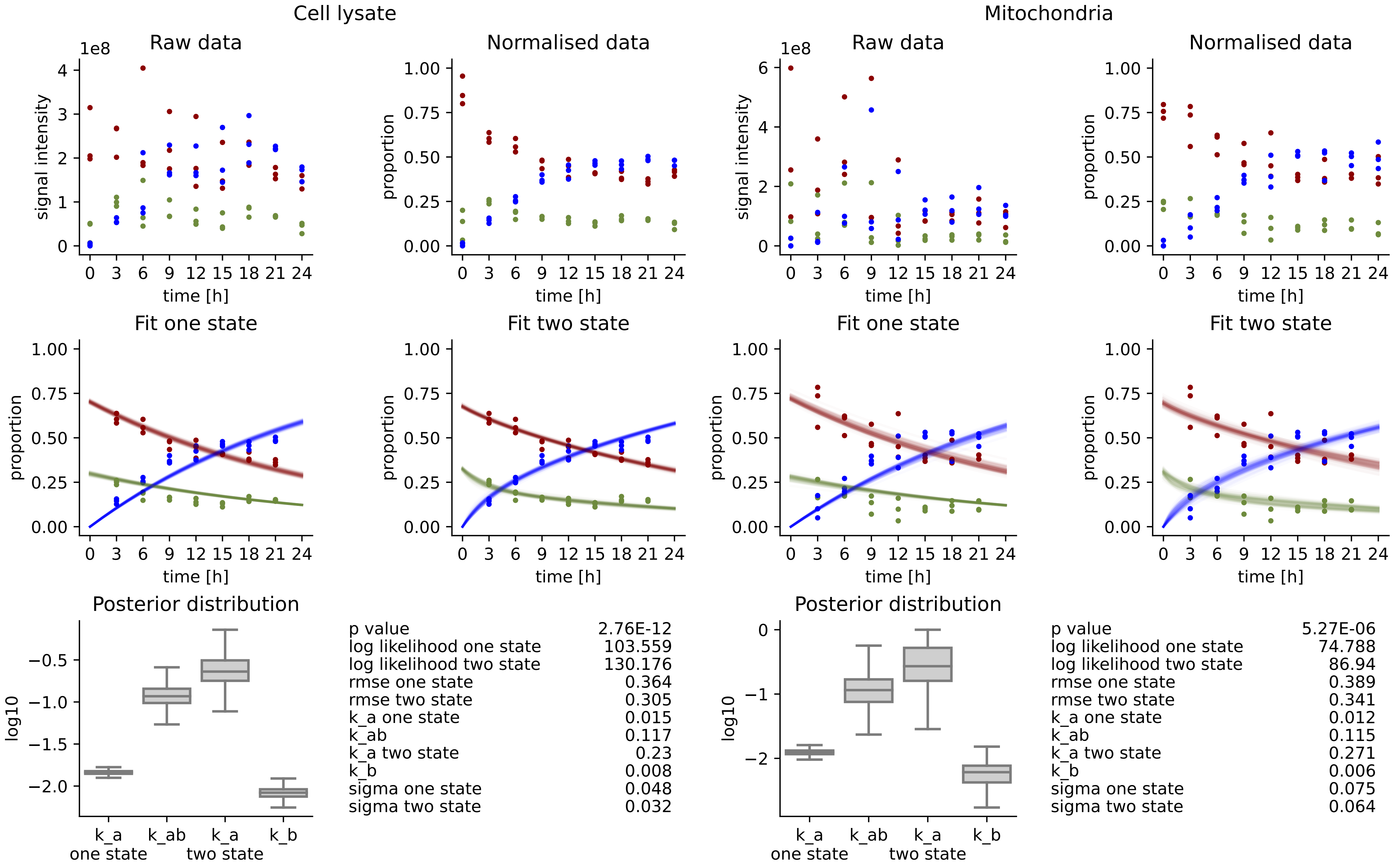

Cell lysate	
p value	2.76E-12
log likelihood one state	103.559
log likelihood two state	130.176
rmse one state	0.364
rmse two state	0.305
k_a one state	0.015
k_ab	0.117
k_a two state	0.23
k_b	0.008
sigma one state	0.048
sigma two state	0.032

Mitochondria	
p value	5.27E-06
log likelihood one state	74.788
log likelihood two state	86.94
rmse one state	0.389
rmse two state	0.341
k_a one state	0.012
k_ab	0.115
k_a two state	0.271
k_b	0.006
sigma one state	0.075
sigma two state	0.064

uL23m
MRPL23
Q16540
39S ribosomal protein L23, mitochondrial

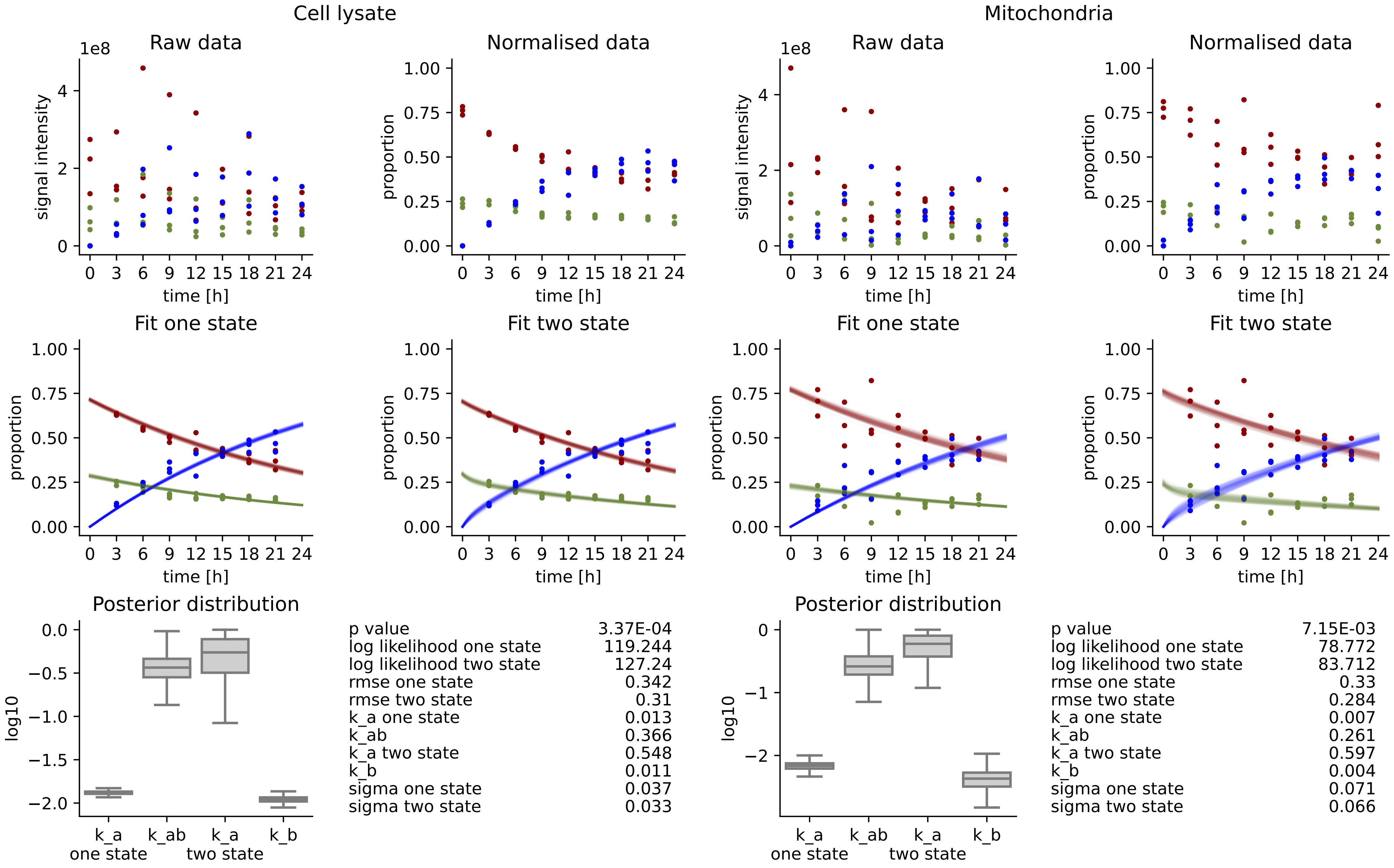

Cell lysate

p value	3.37E-04
log likelihood one state	119.244
log likelihood two state	127.24
rmse one state	0.342
rmse two state	0.31
k_a one state	0.013
k_ab	0.366
k_a two state	0.548
k_b	0.011
sigma one state	0.037
sigma two state	0.033

Mitochondria

p value	7.15E-03
log likelihood one state	78.772
log likelihood two state	83.712
rmse one state	0.33
rmse two state	0.284
k_a one state	0.007
k_ab	0.261
k_a two state	0.597
k_b	0.004
sigma one state	0.071
sigma two state	0.066

uL24m
MRPL24
Q96A35
39S ribosomal protein L24, mitochondrial

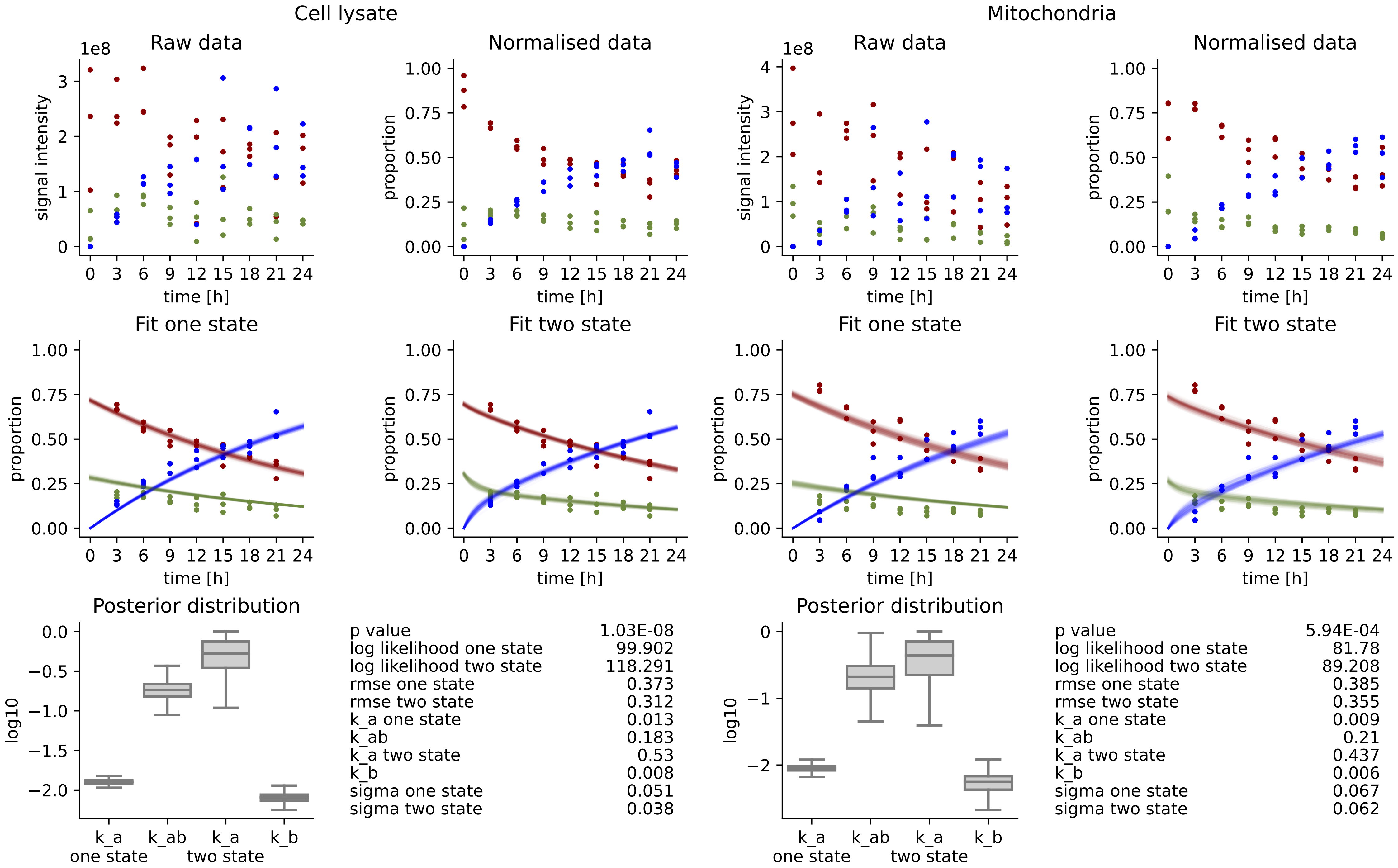

Cell lysate

p value	1.03E-08
log likelihood one state	99.902
log likelihood two state	118.291
rmse one state	0.373
rmse two state	0.312
k_a one state	0.013
k_ab	0.183
k_a two state	0.53
k_b	0.008
sigma one state	0.051
sigma two state	0.038

Mitochondria

p value	5.94E-04
log likelihood one state	81.78
log likelihood two state	89.208
rmse one state	0.385
rmse two state	0.355
k_a one state	0.009
k_ab	0.21
k_a two state	0.437
k_b	0.006
sigma one state	0.067
sigma two state	0.062

bL27m
MRPL27
Q9P0M9
39S ribosomal protein L27, mitochondrial

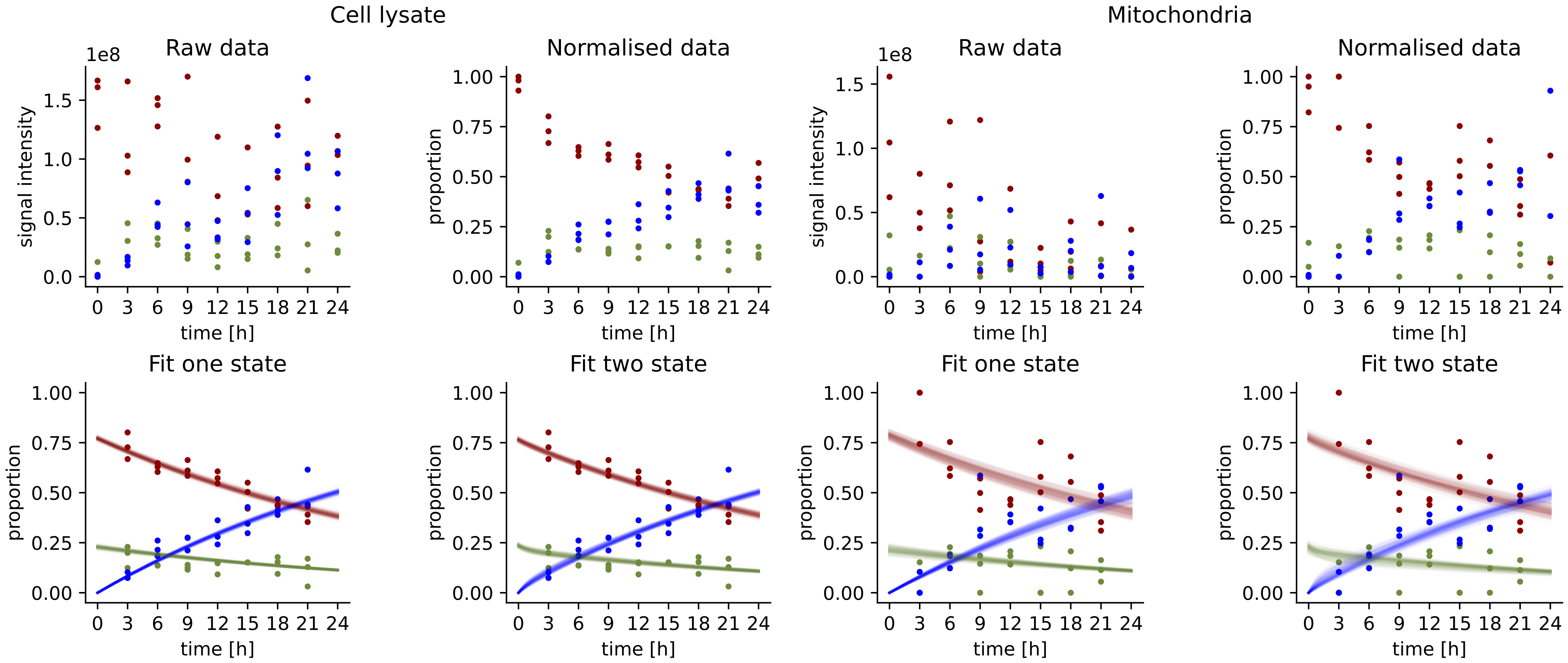

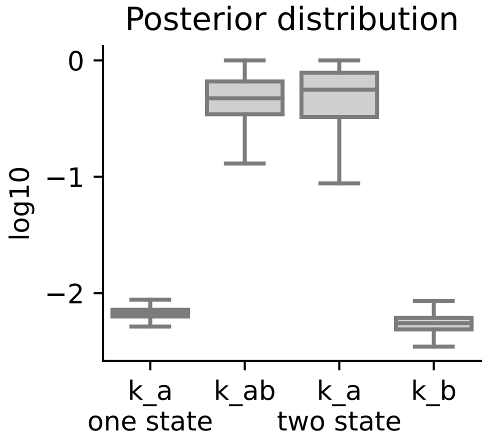

p value	6.10E-02
log likelihood one state	102.176
log likelihood two state	104.973
rmse one state	0.331
rmse two state	0.305
k_a one state	0.007
k_ab	0.473
k_a two state	0.56
k_b	0.006
sigma one state	0.049
sigma two state	0.047

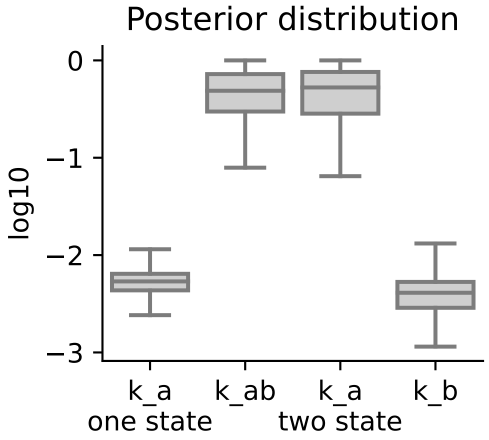

p value	3.30E-01
log likelihood one state	46.329
log likelihood two state	47.438
rmse one state	0.384
rmse two state	0.352
k_a one state	0.005
k_ab	0.488
k_a two state	0.527
k_b	0.004
sigma one state	0.117
sigma two state	0.116

bL28m
MRPL28
Q13084
39S ribosomal protein L28, mitochondrial

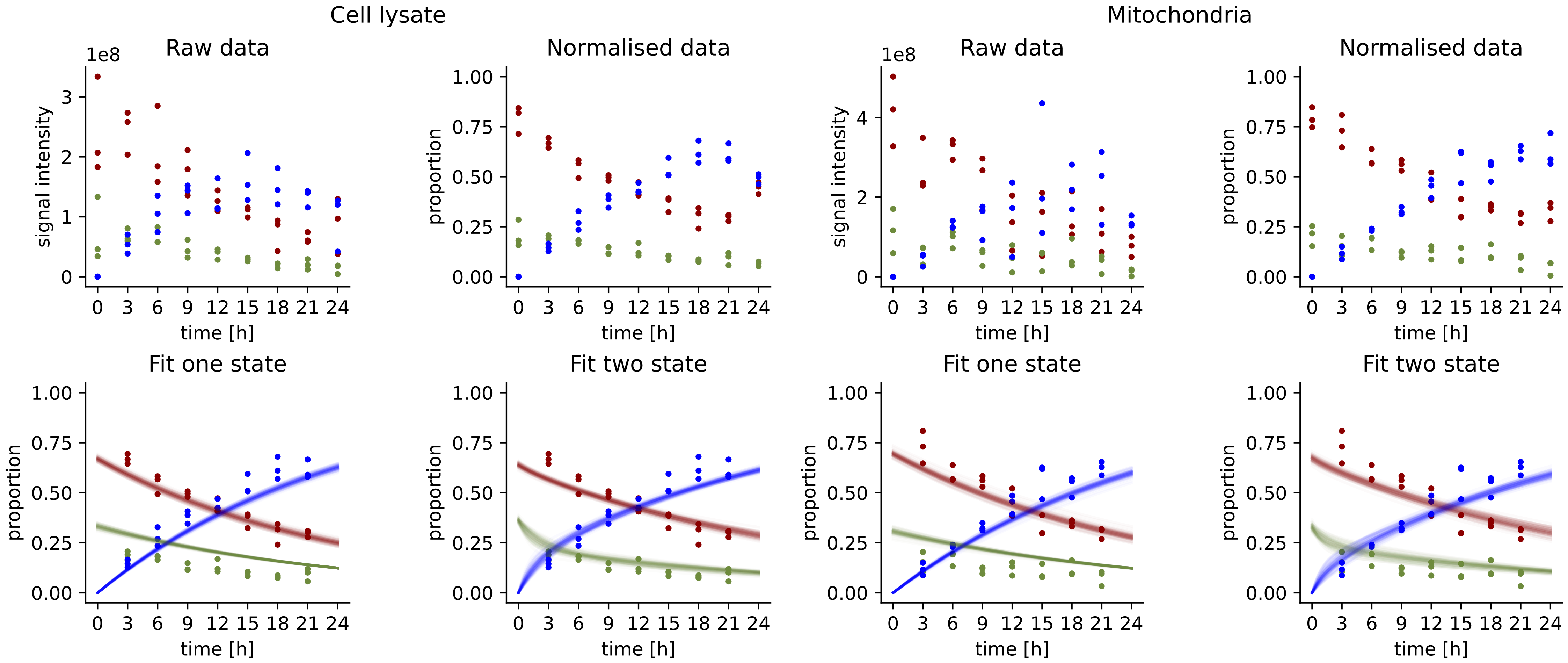

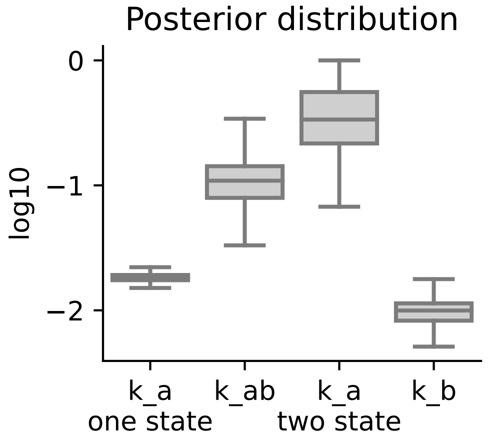

p value	2.86E-09
log likelihood one state	78.374
log likelihood two state	98.047
rmse one state	0.442
rmse two state	0.371
k_a one state	0.018
k_ab	0.109
k_a two state	0.336
k_b	0.01
sigma one state	0.071
sigma two state	0.053

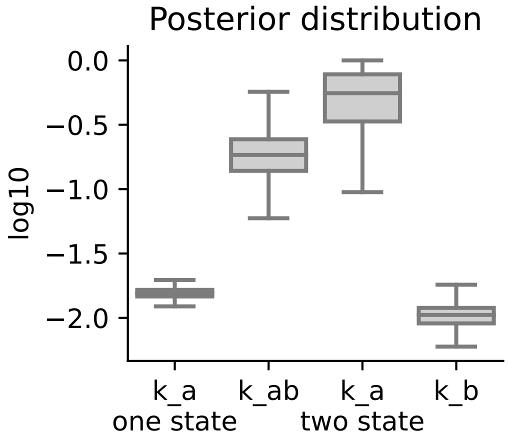

p value	2.54E-04
log likelihood one state	70.718
log likelihood two state	78.996
rmse one state	0.438
rmse two state	0.38
k_a one state	0.016
k_ab	0.184
k_a two state	0.556
k_b	0.011
sigma one state	0.08
sigma two state	0.072

uL29m
MRPL47
Q9HD33
39S ribosomal protein L47, mitochondrial

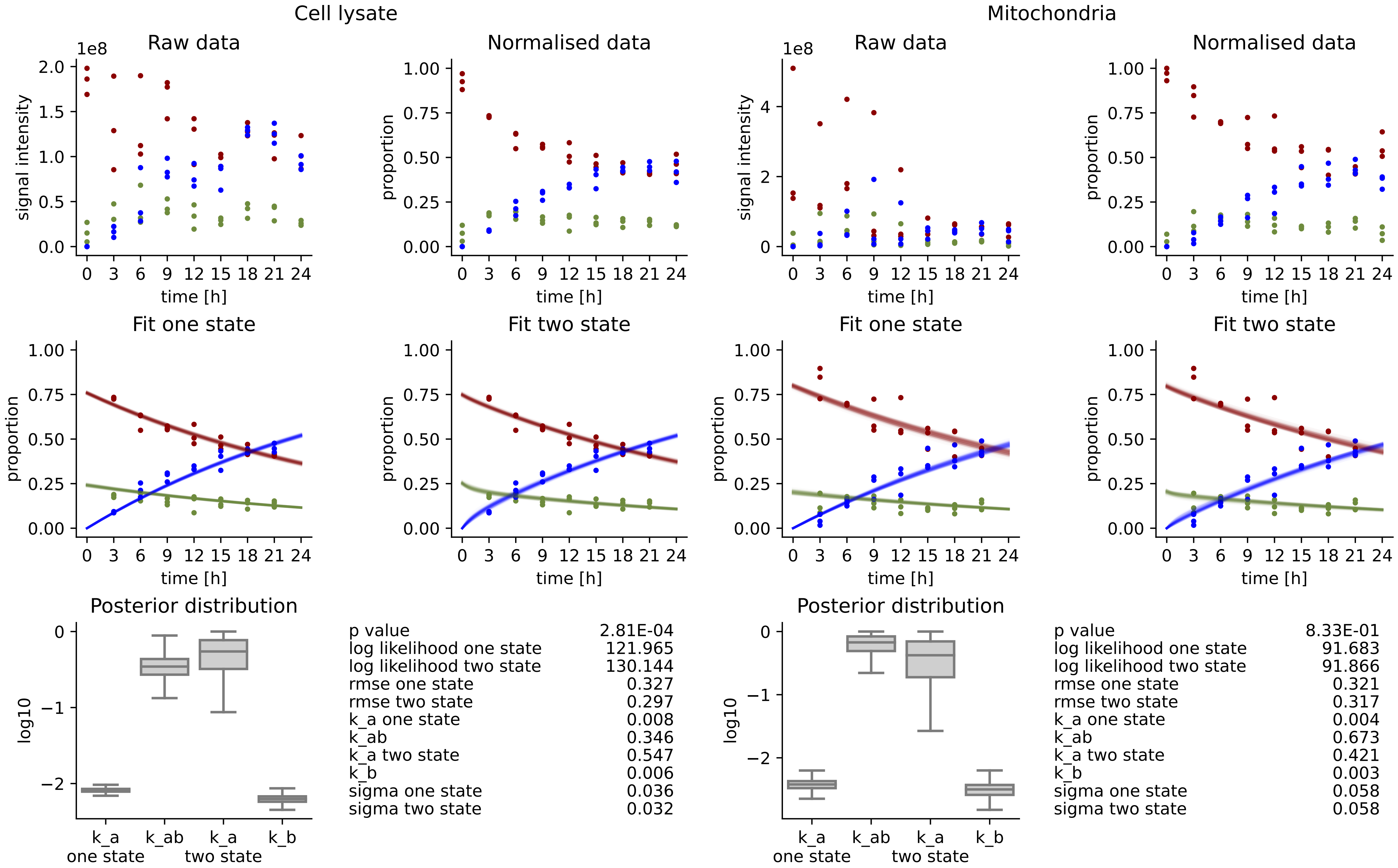

uL30m
MRPL30
Q8TCC3
39S ribosomal protein L30, mitochondrial

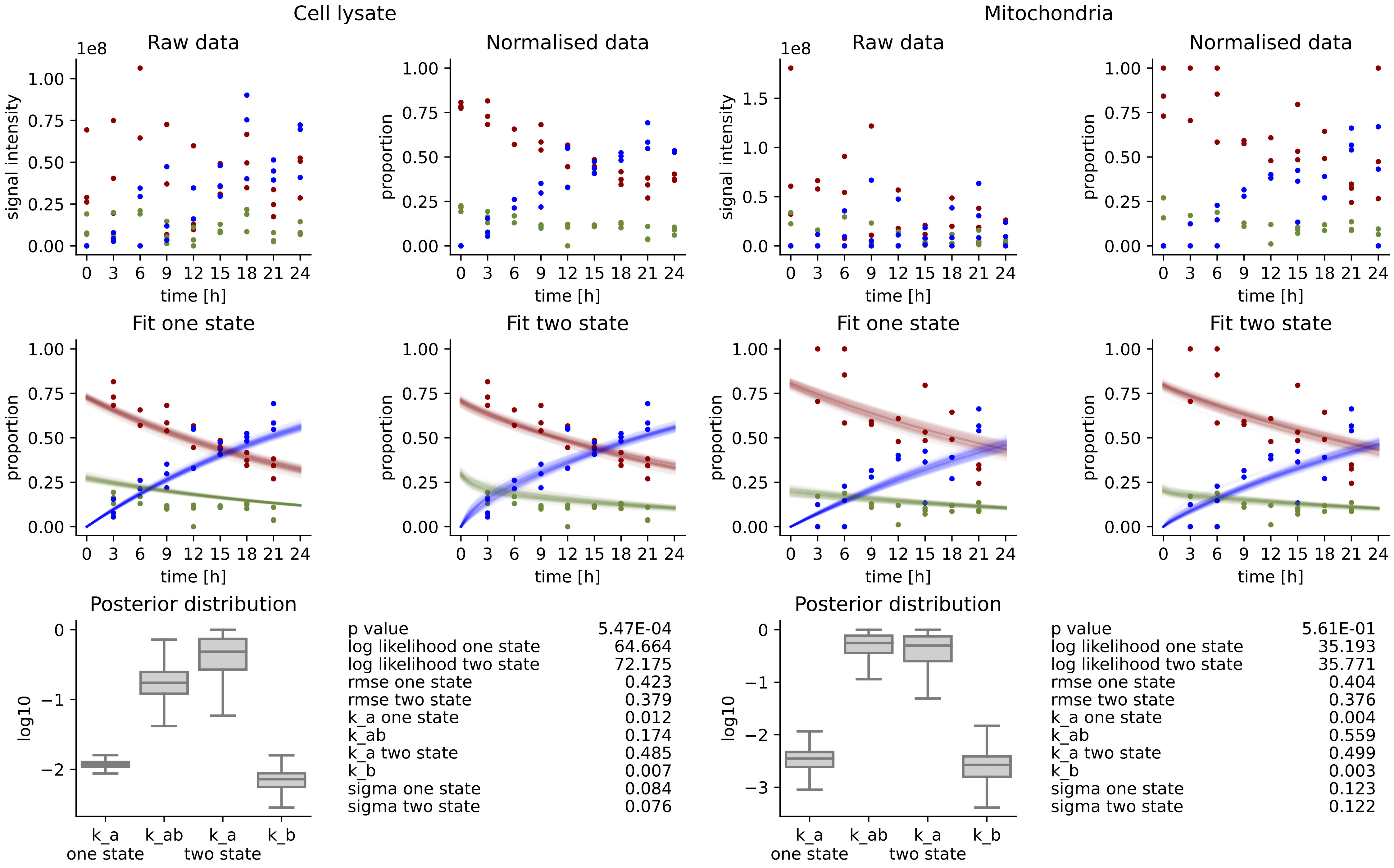

bL31m
MRPL55
Q7Z7F7
39S ribosomal protein L55, mitochondrial

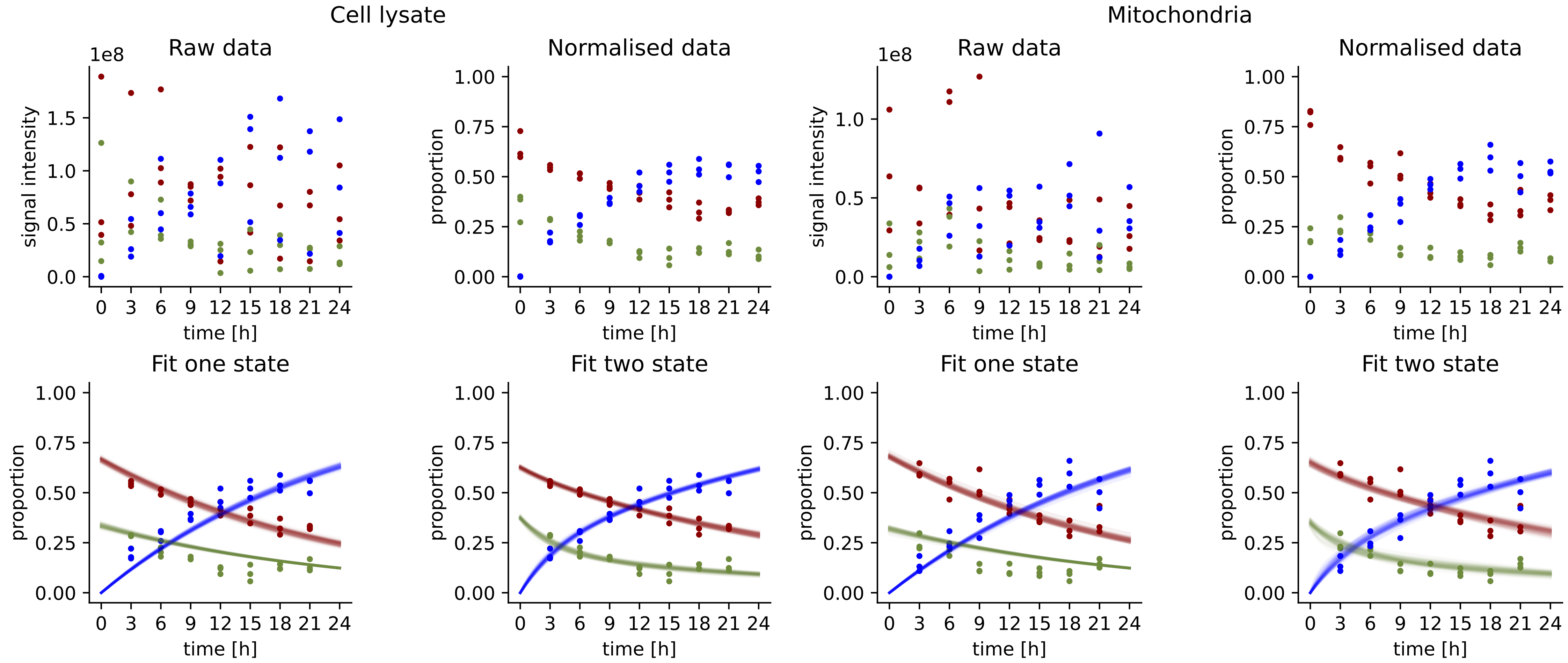

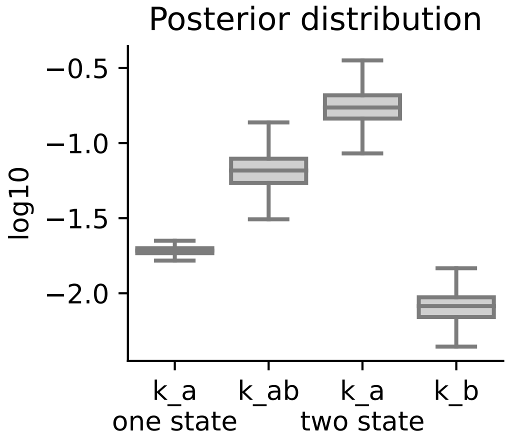

p value	9.90E-18
log likelihood one state	91.998
log likelihood two state	131.152
rmse one state	0.403
rmse two state	0.331
k_a one state	0.019
k_ab	0.066
k_a two state	0.172
k_b	0.008
sigma one state	0.057
sigma two state	0.031

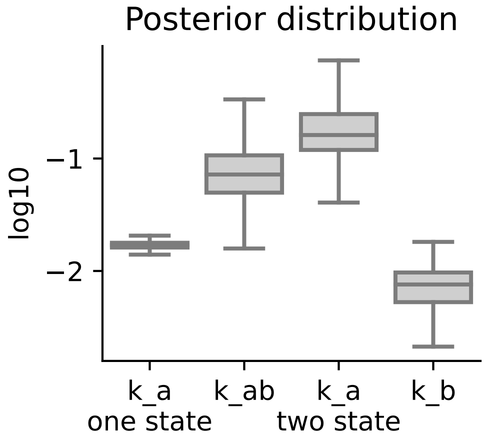

p value	3.45E-08
log likelihood one state	80.087
log likelihood two state	97.269
rmse one state	0.411
rmse two state	0.355
k_a one state	0.017
k_ab	0.072
k_a two state	0.162
k_b	0.008
sigma one state	0.069
sigma two state	0.054

bL32m
MRPL32
Q9BYC8
39S ribosomal protein L32, mitochondrial

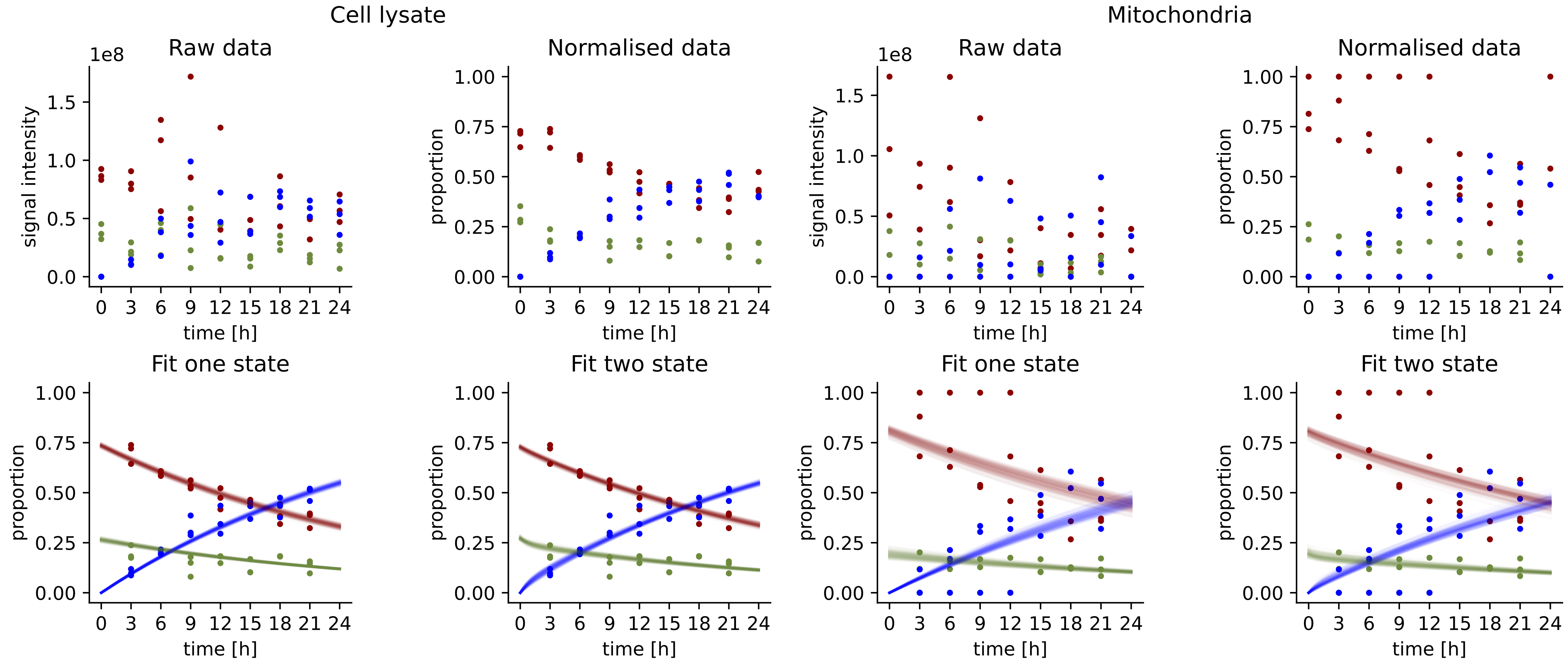

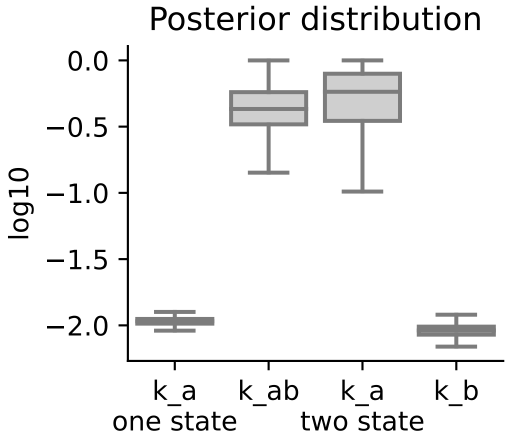

p value	1.11E-02
log likelihood one state	109.846
log likelihood two state	114.343
rmse one state	0.347
rmse two state	0.317
k_a one state	0.011
k_ab	0.43
k_a two state	0.579
k_b	0.009
sigma one state	0.043
sigma two state	0.041

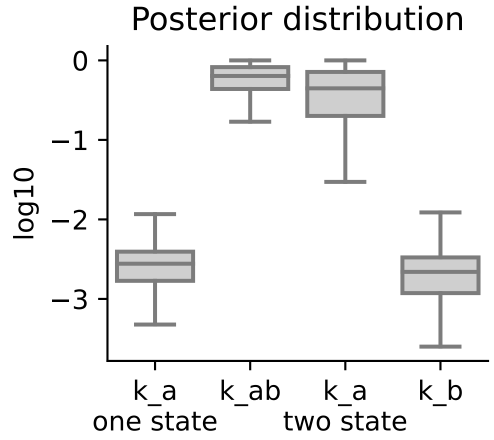

p value	9.77E-01
log likelihood one state	33.509
log likelihood two state	33.532
rmse one state	0.4
rmse two state	0.395
k_a one state	0.003
k_ab	0.637
k_a two state	0.445
k_b	0.002
sigma one state	0.139
sigma two state	0.139

bL33m
MRPL33
O75394
39S ribosomal protein L33, mitochondrial

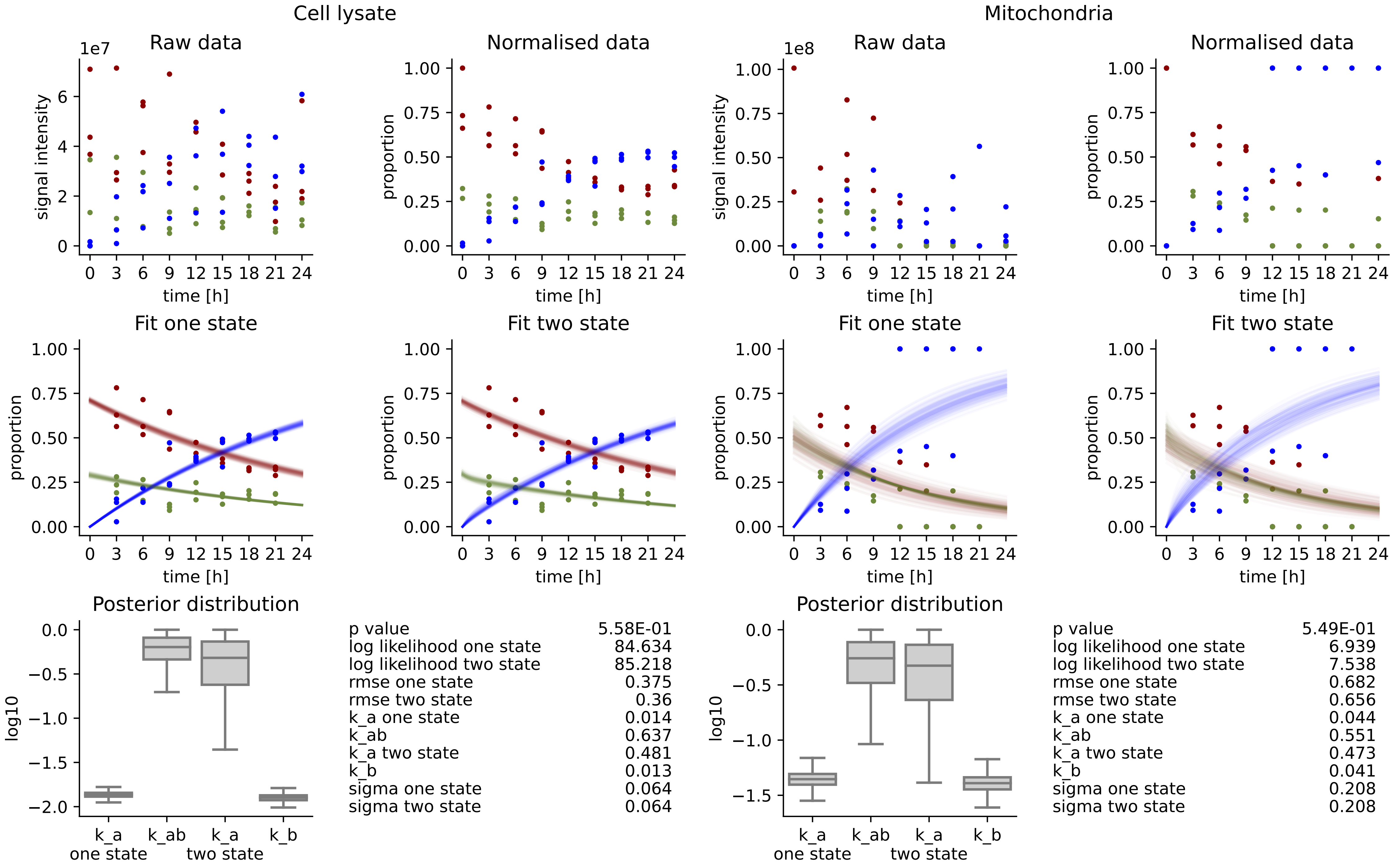

bL34m
MRPL34
Q9BQ48
39S ribosomal protein L34, mitochondrial

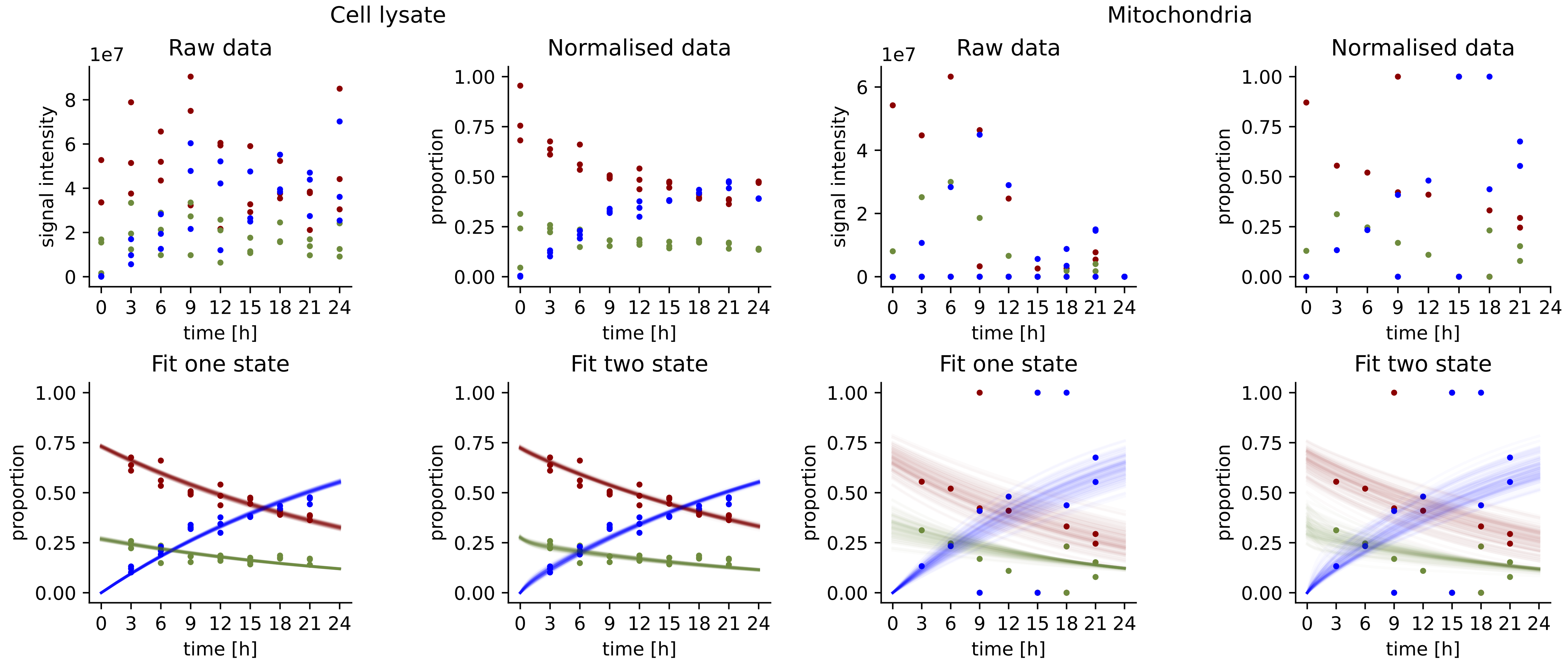

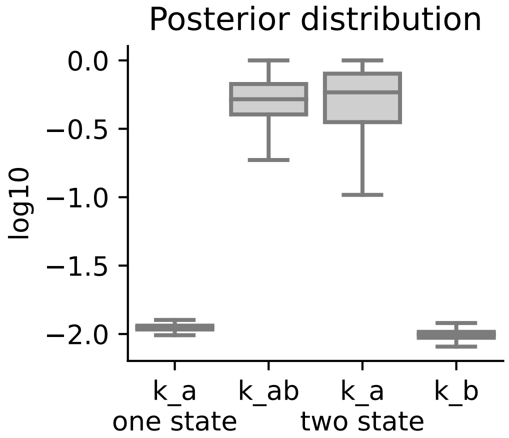

p value	1.67E-02
log likelihood one state	123.138
log likelihood two state	127.23
rmse one state	0.325
rmse two state	0.302
k_a one state	0.011
k_ab	0.52
k_a two state	0.584
k_b	0.01
sigma one state	0.035
sigma two state	0.033

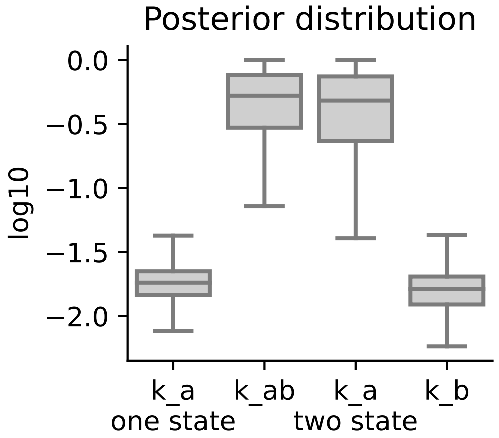

p value	7.12E-01
log likelihood one state	-0.224
log likelihood two state	0.115
rmse one state	0.625
rmse two state	0.606
k_a one state	0.018
k_ab	0.528
k_a two state	0.483
k_b	0.016
sigma one state	0.233
sigma two state	0.233

bL35m
MRPL35
Q9NZE8
39S ribosomal protein L35, mitochondrial

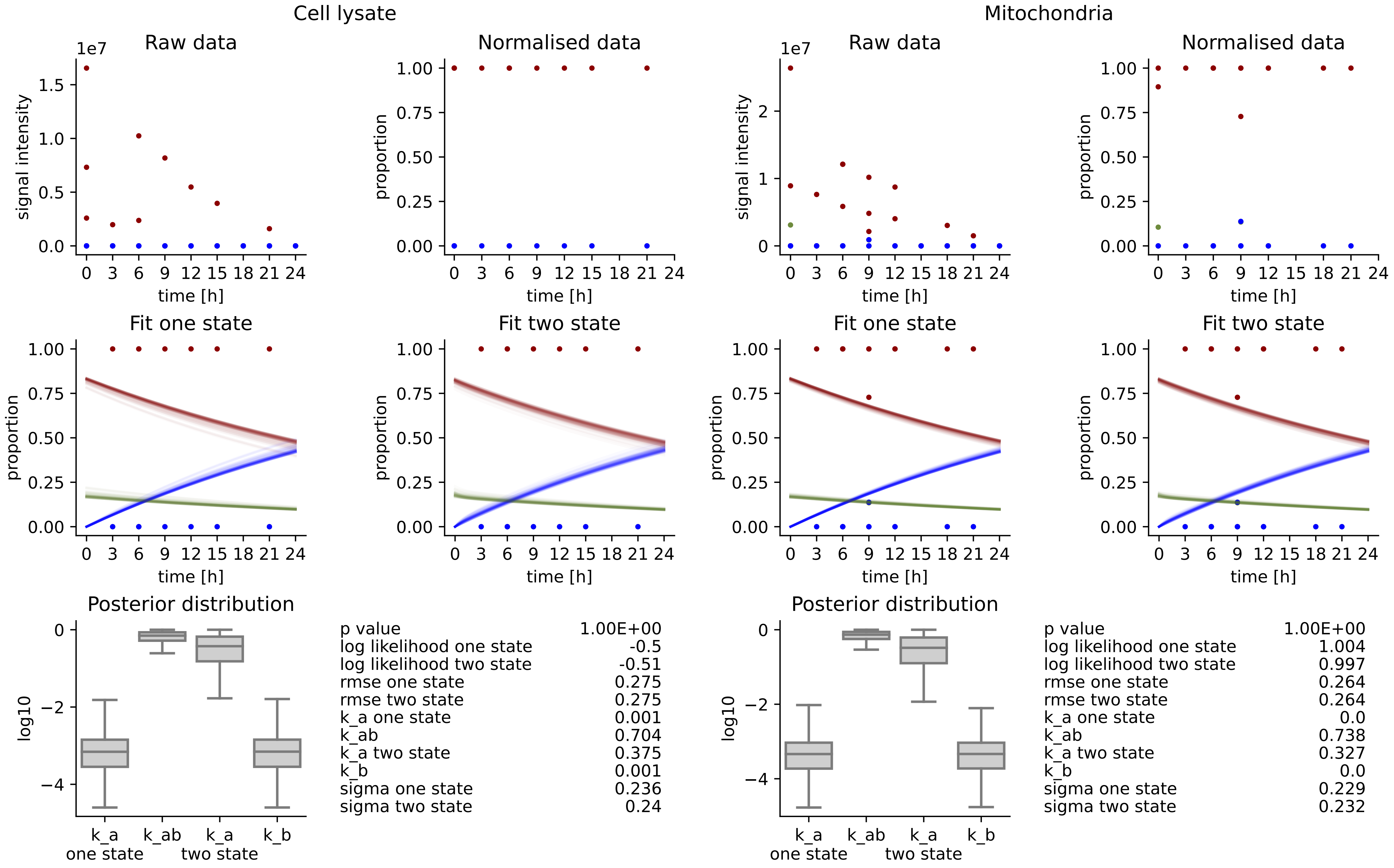

p value	1.00E+00
log likelihood one state	-0.5
log likelihood two state	-0.51
rmse one state	0.275
rmse two state	0.275
k_a one state	0.001
k_ab	0.704
k_a two state	0.375
k_b	0.001
sigma one state	0.236
sigma two state	0.24

p value	1.00E+00
log likelihood one state	1.004
log likelihood two state	0.997
rmse one state	0.264
rmse two state	0.264
k_a one state	0.0
k_ab	0.738
k_a two state	0.327
k_b	0.0
sigma one state	0.229
sigma two state	0.232

mL37
MRPL37
Q9BZE1
39S ribosomal protein L37, mitochondrial

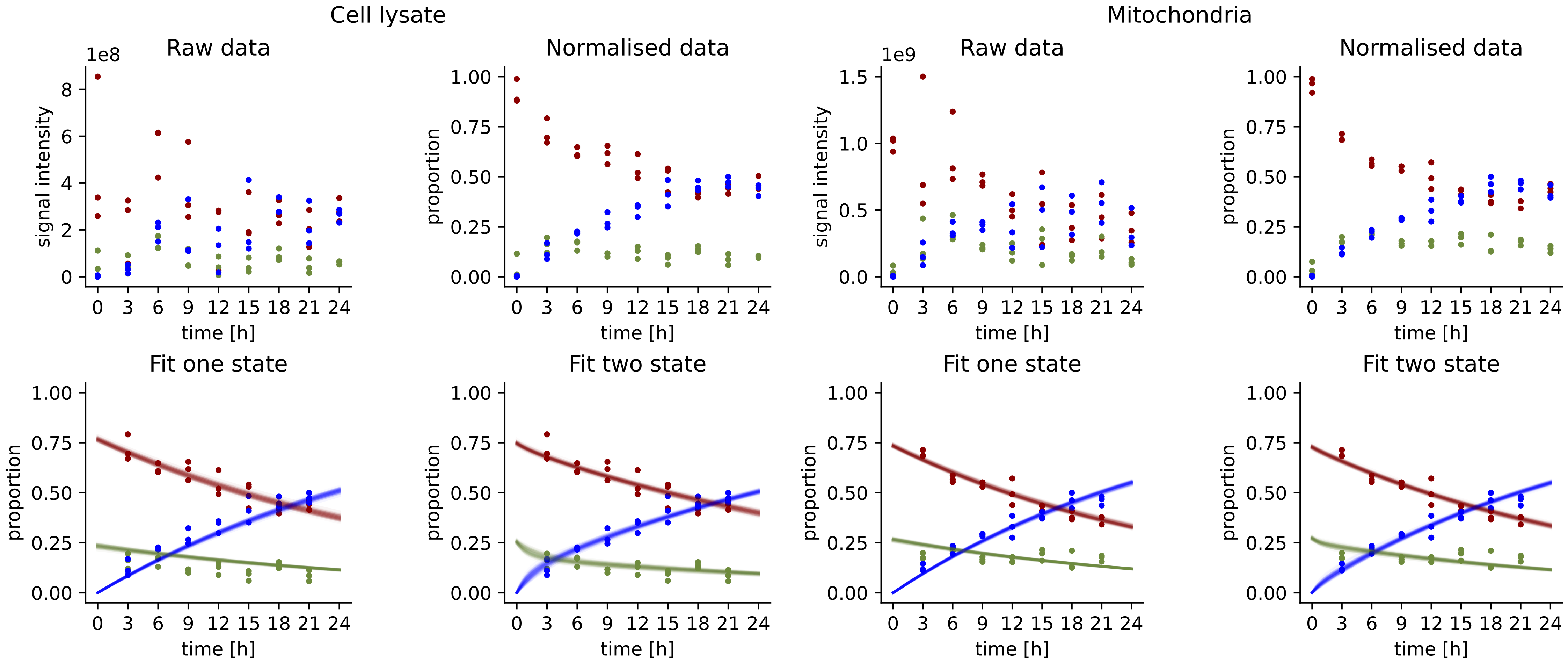

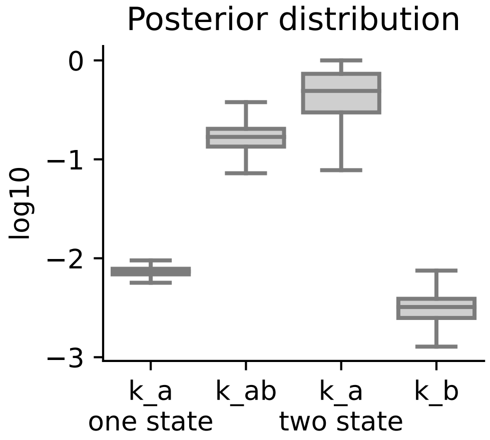

p value	9.52E-08
log likelihood one state	99.281
log likelihood two state	115.448
rmse one state	0.344
rmse two state	0.29
k_a one state	0.007
k_ab	0.169
k_a two state	0.492
k_b	0.003
sigma one state	0.051
sigma two state	0.04

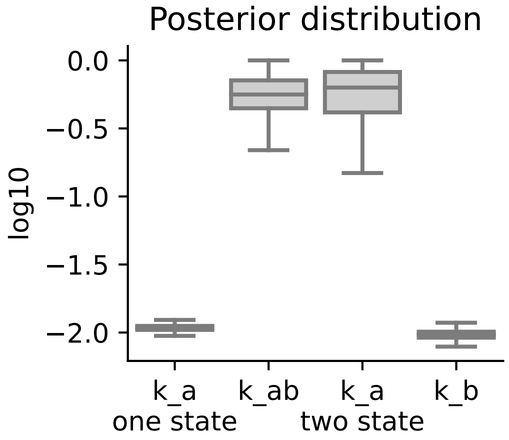

p value	2.80E-02
log likelihood one state	121.543
log likelihood two state	125.119
rmse one state	0.333
rmse two state	0.308
k_a one state	0.011
k_ab	0.562
k_a two state	0.631
k_b	0.01
sigma one state	0.036
sigma two state	0.034

mL38
MRPL38
Q96DV4
39S ribosomal protein L38, mitochondrial

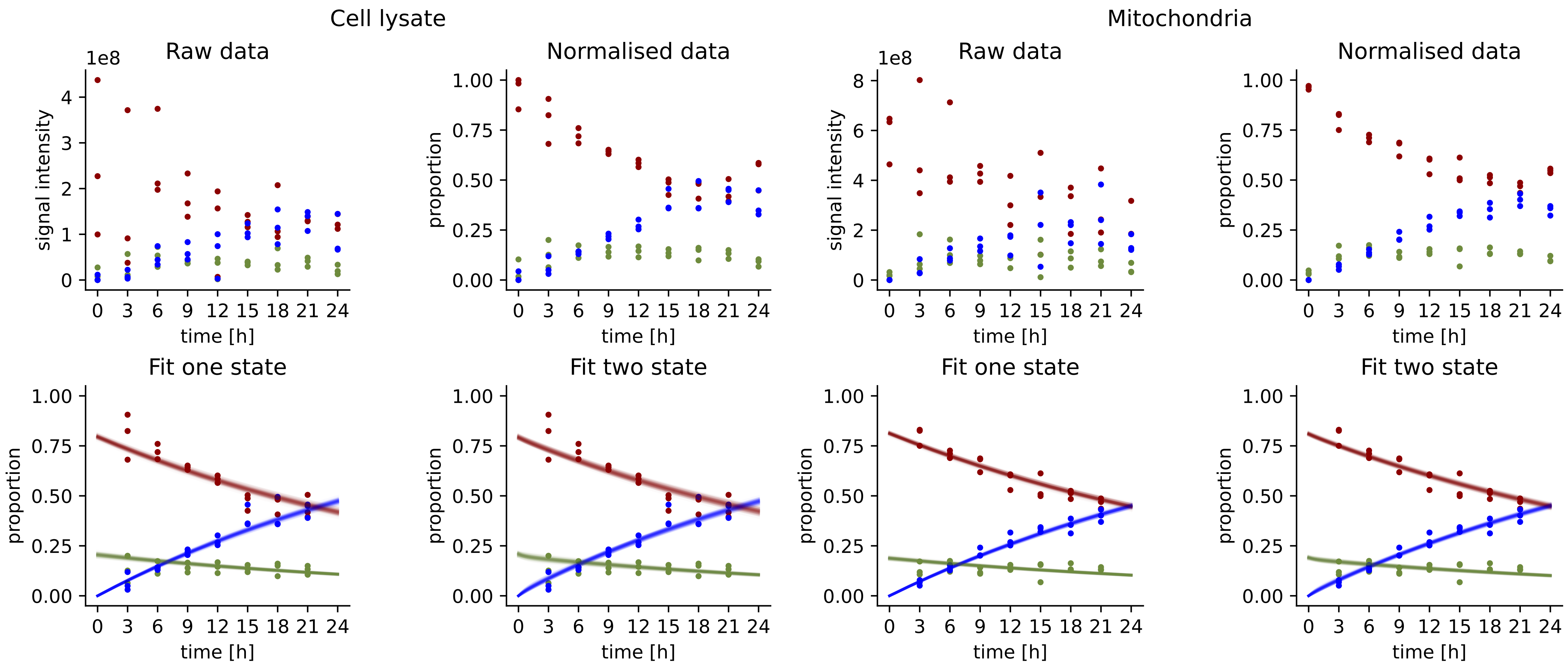

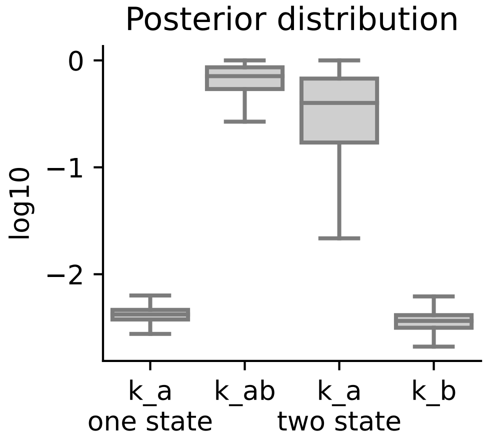

p value	9.58E-01
log likelihood one state	99.785
log likelihood two state	99.828
rmse one state	0.319
rmse two state	0.315
k_a one state	0.004
k_ab	0.712
k_a two state	0.4
k_b	0.004
sigma one state	0.051
sigma two state	0.051

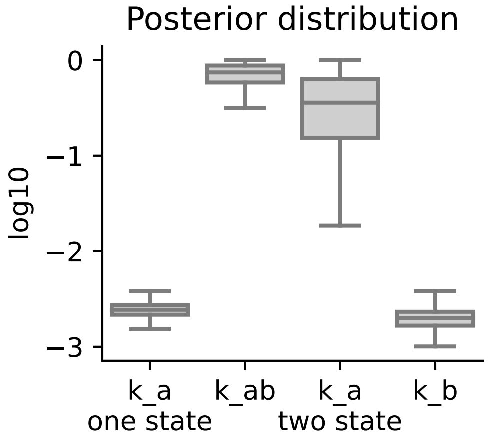

p value	9.19E-01
log likelihood one state	125.777
log likelihood two state	125.861
rmse one state	0.288
rmse two state	0.284
k_a one state	0.002
k_ab	0.744
k_a two state	0.359
k_b	0.002
sigma one state	0.034
sigma two state	0.034

mL39
MRPL39
Q9NYK5
39S ribosomal protein L39, mitochondrial

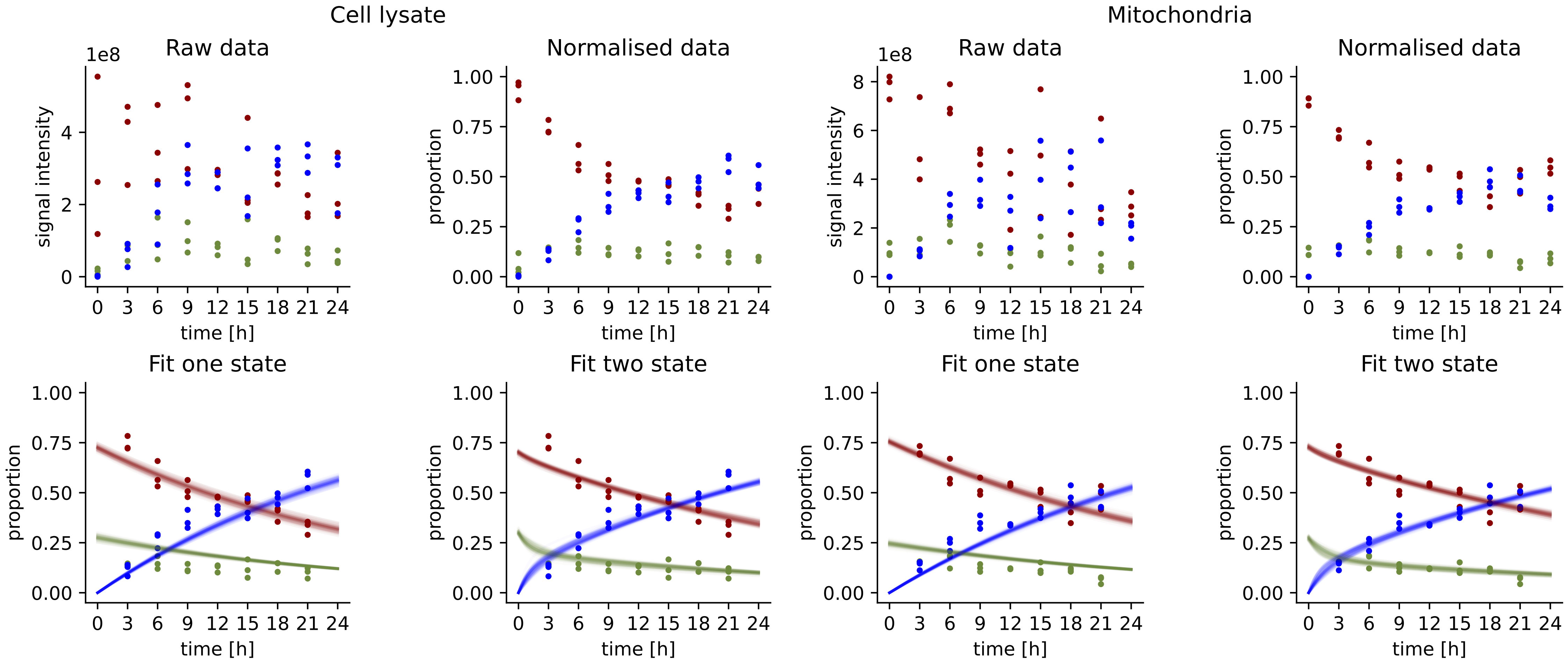

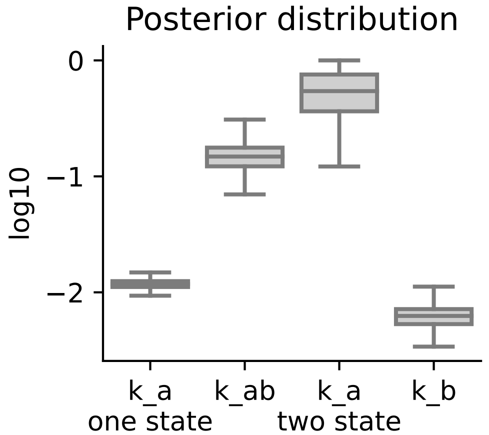

p value	8.26E-08
log likelihood one state	84.428
log likelihood two state	100.738
rmse one state	0.39
rmse two state	0.315
k_a one state	0.012
k_ab	0.148
k_a two state	0.543
k_b	0.006
sigma one state	0.065
sigma two state	0.051

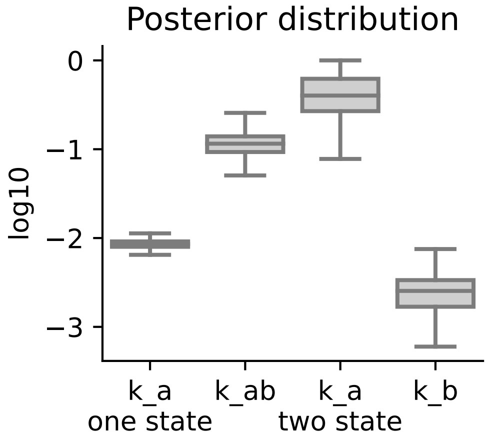

p value	1.21E-10
log likelihood one state	88.841
log likelihood two state	111.673
rmse one state	0.351
rmse two state	0.282
k_a one state	0.009
k_ab	0.115
k_a two state	0.402
k_b	0.003
sigma one state	0.06
sigma two state	0.043

mL40
MRPL40
Q9NQ50
39S ribosomal protein L40, mitochondrial

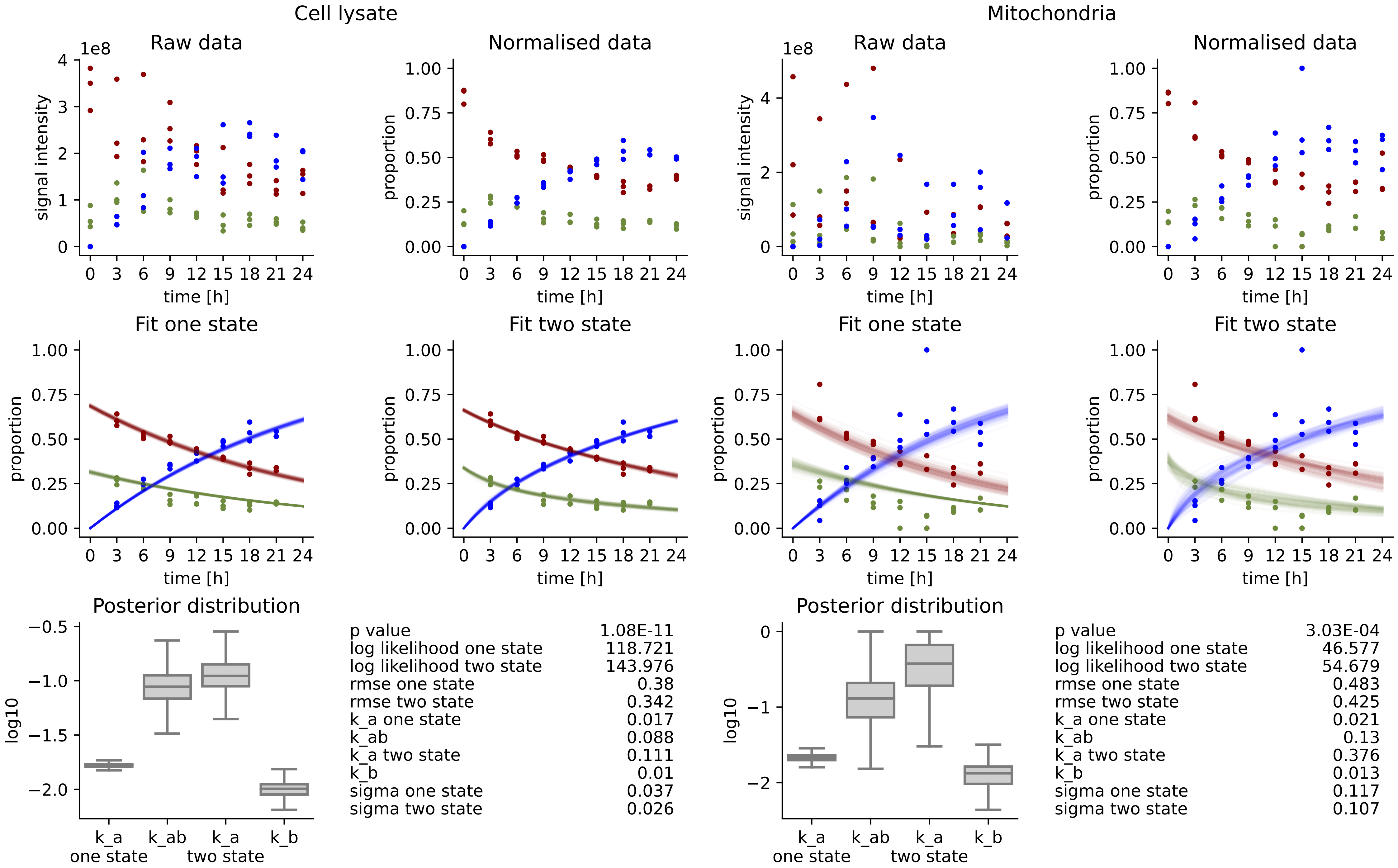

Cell lysate

p value	1.08E-11
log likelihood one state	118.721
log likelihood two state	143.976
rmse one state	0.38
rmse two state	0.342
k_a one state	0.017
k_ab	0.088
k_a two state	0.111
k_b	0.01
sigma one state	0.037
sigma two state	0.026

Mitochondria

p value	3.03E-04
log likelihood one state	46.577
log likelihood two state	54.679
rmse one state	0.483
rmse two state	0.425
k_a one state	0.021
k_ab	0.13
k_a two state	0.376
k_b	0.013
sigma one state	0.117
sigma two state	0.107

mL41
MRPL41
Q8IXM3
39S ribosomal protein L41, mitochondrial

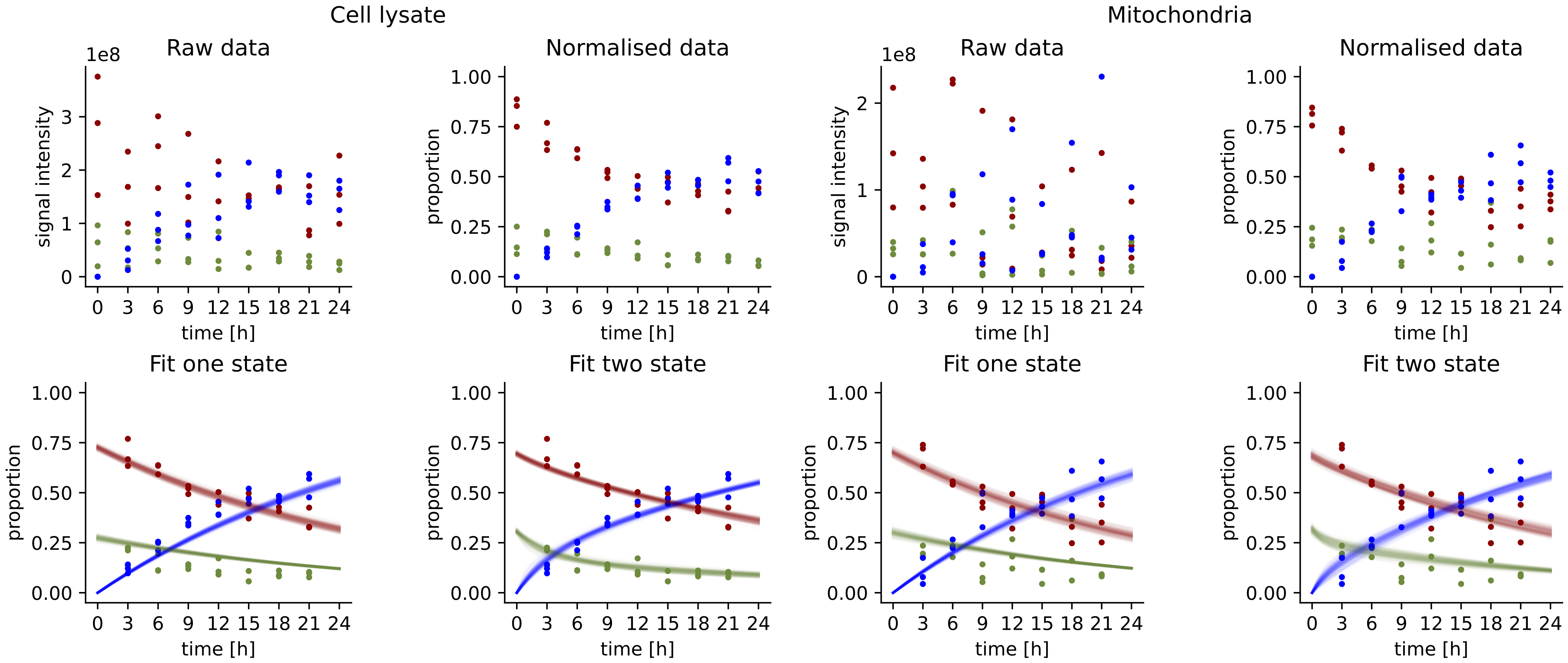

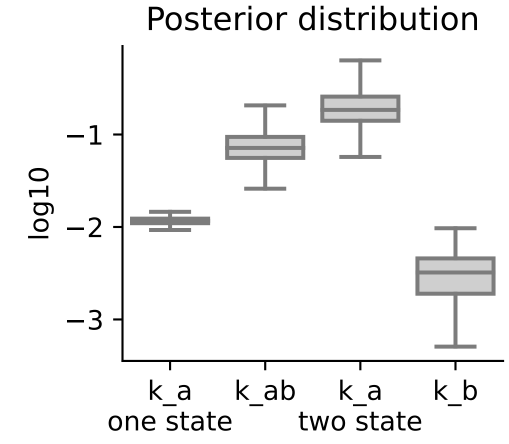

p value	5.35E-11
log likelihood one state	85.642
log likelihood two state	109.293
rmse one state	0.388
rmse two state	0.331
k_a one state	0.012
k_ab	0.072
k_a two state	0.185
k_b	0.003
sigma one state	0.063
sigma two state	0.045

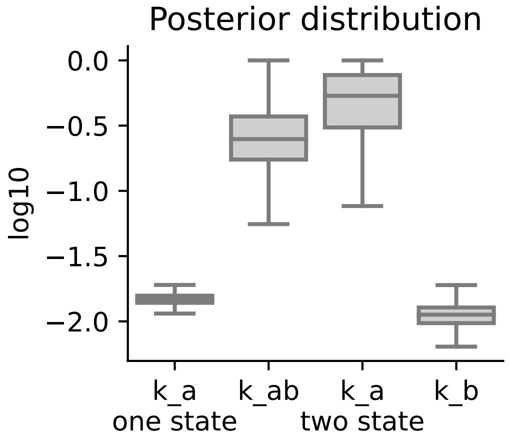

p value	4.98E-03
log likelihood one state	67.994
log likelihood two state	73.297
rmse one state	0.403
rmse two state	0.355
k_a one state	0.015
k_ab	0.249
k_a two state	0.535
k_b	0.011
sigma one state	0.083
sigma two state	0.079

mL42
MRPL42
Q9Y6G3
39S ribosomal protein L42, mitochondrial

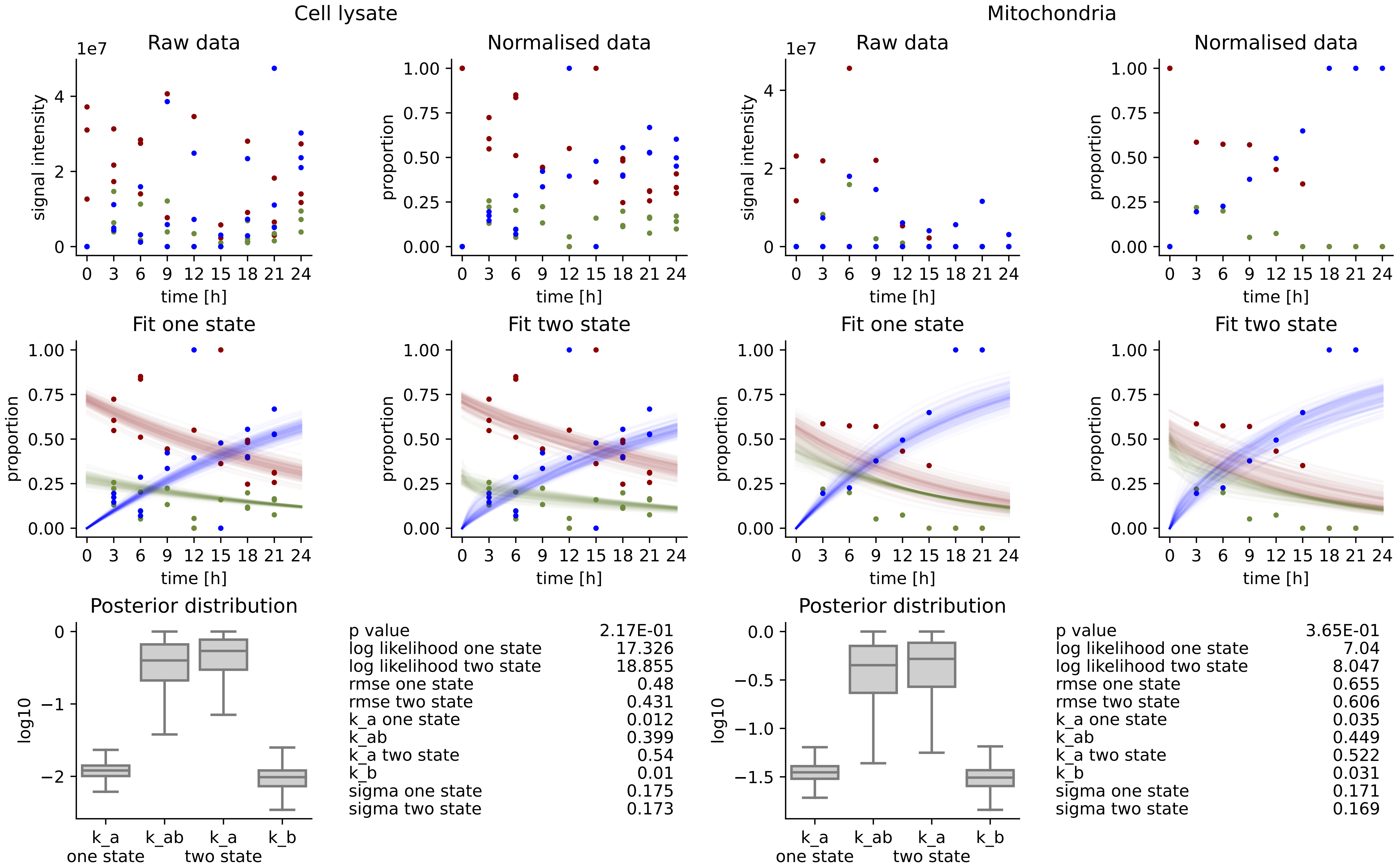

p value	2.17E-01
log likelihood one state	17.326
log likelihood two state	18.855
rmse one state	0.48
rmse two state	0.431
k_a one state	0.012
k_ab	0.399
k_a two state	0.54
k_b	0.01
sigma one state	0.175
sigma two state	0.173

p value	3.65E-01
log likelihood one state	7.04
log likelihood two state	8.047
rmse one state	0.655
rmse two state	0.606
k_a one state	0.035
k_ab	0.449
k_a two state	0.522
k_b	0.031
sigma one state	0.171
sigma two state	0.169

mL43
MRPL43
Q8N983
39S ribosomal protein L43, mitochondrial

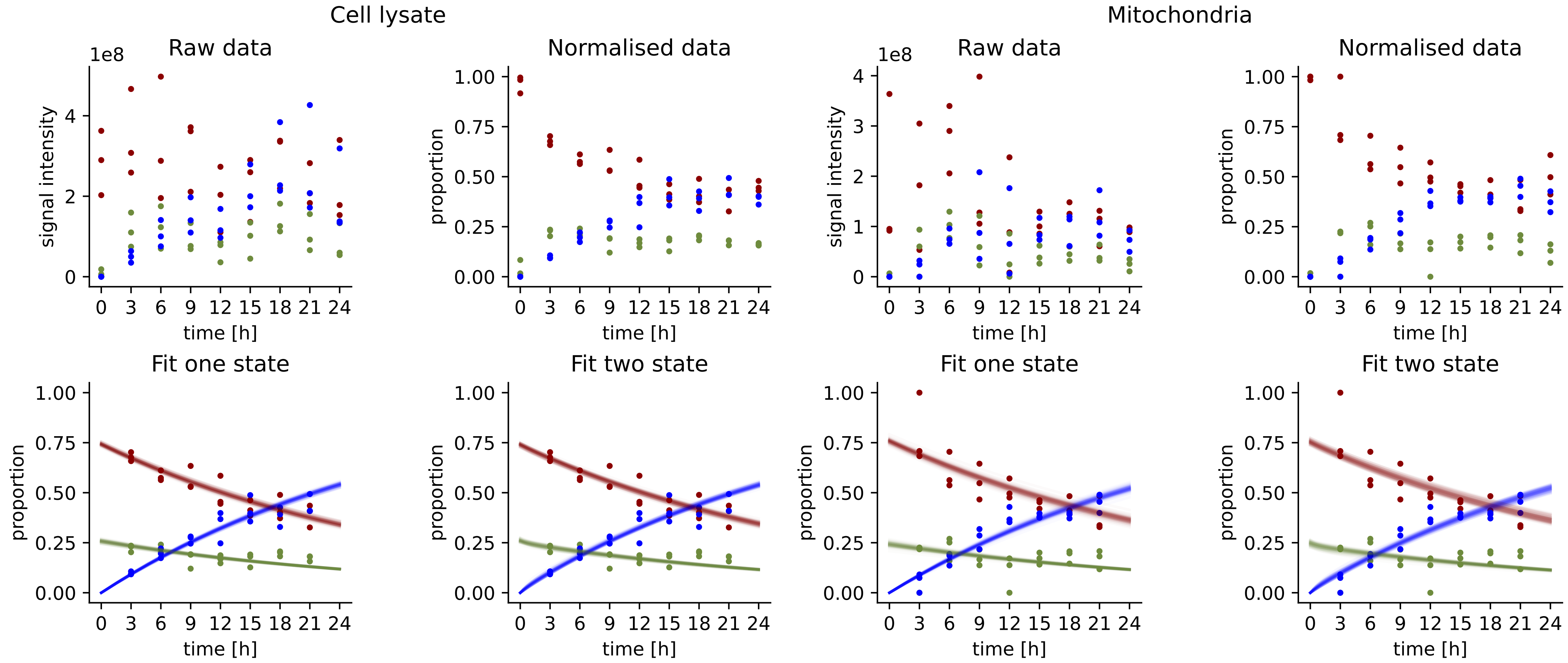

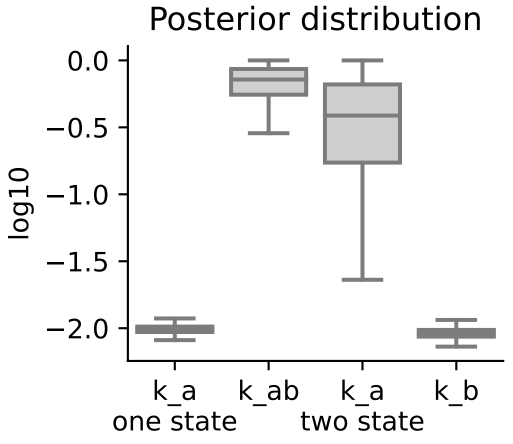

p value	8.85E-01
log likelihood one state	105.273
log likelihood two state	105.395
rmse one state	0.32
rmse two state	0.313
k_a one state	0.01
k_ab	0.72
k_a two state	0.387
k_b	0.009
sigma one state	0.046
sigma two state	0.046

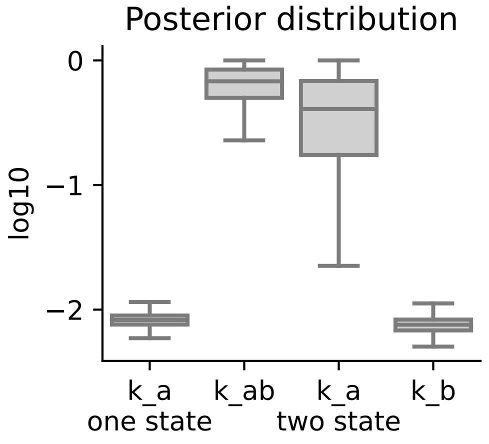

p value	9.81E-01
log likelihood one state	76.735
log likelihood two state	76.755
rmse one state	0.344
rmse two state	0.347
k_a one state	0.008
k_ab	0.68
k_a two state	0.407
k_b	0.008
sigma one state	0.073
sigma two state	0.073

mL44
MRPL44
Q9H9J2
39S ribosomal protein L44, mitochondrial

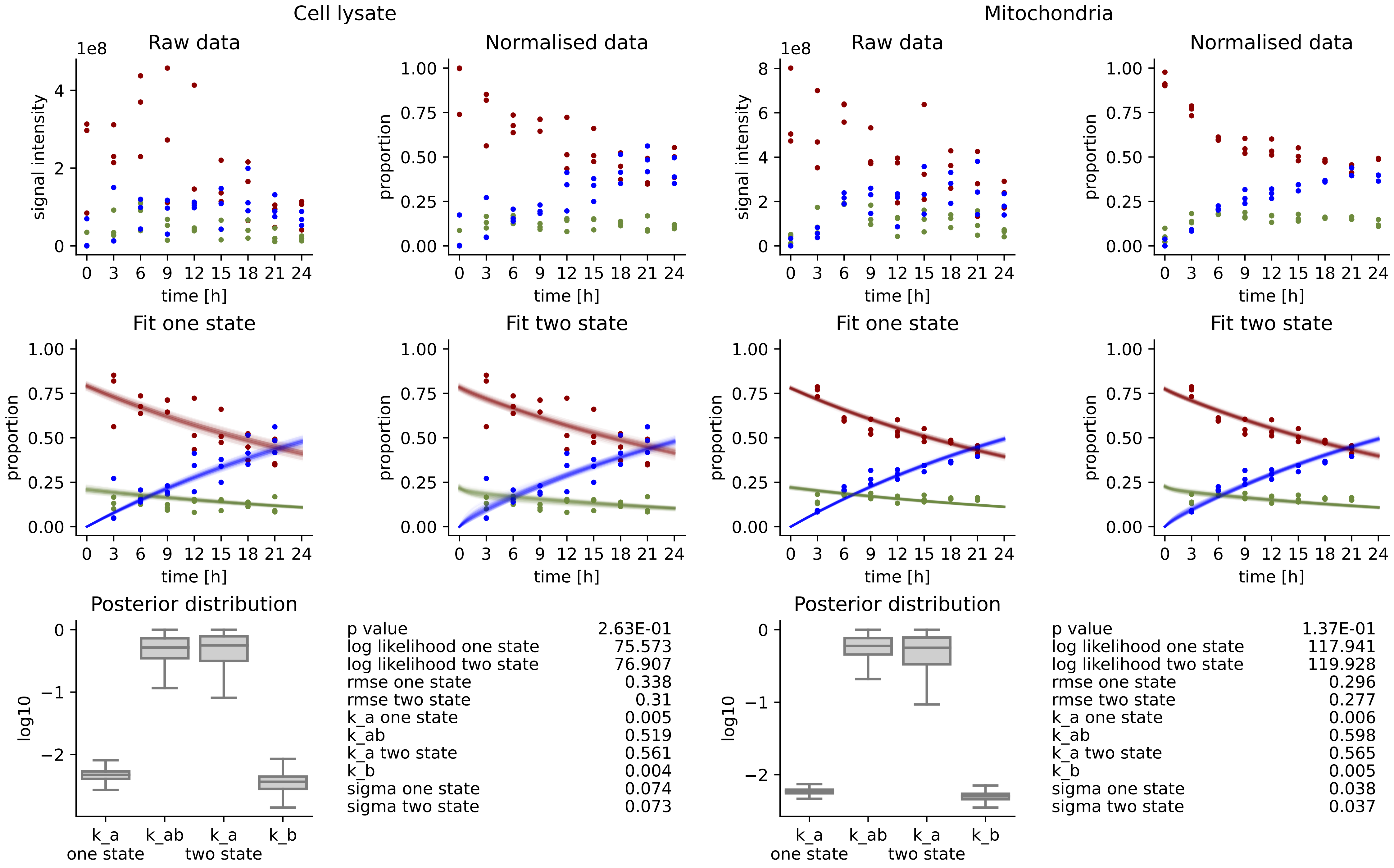

mL45
MRPL45
Q9BRJ2
39S ribosomal protein L45, mitochondrial

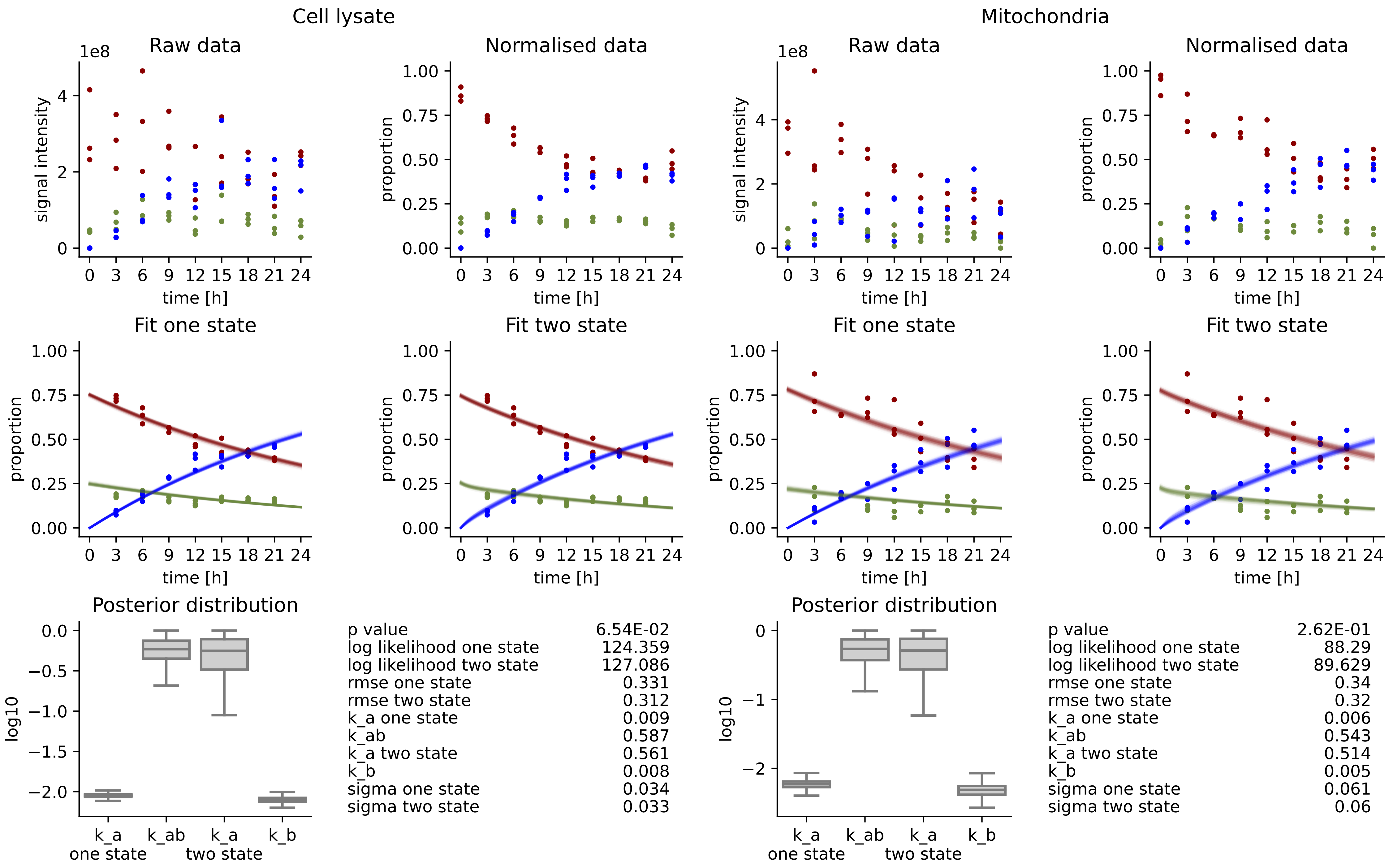

mL46
MRPL46
Q9H2W6
39S ribosomal protein L46, mitochondrial

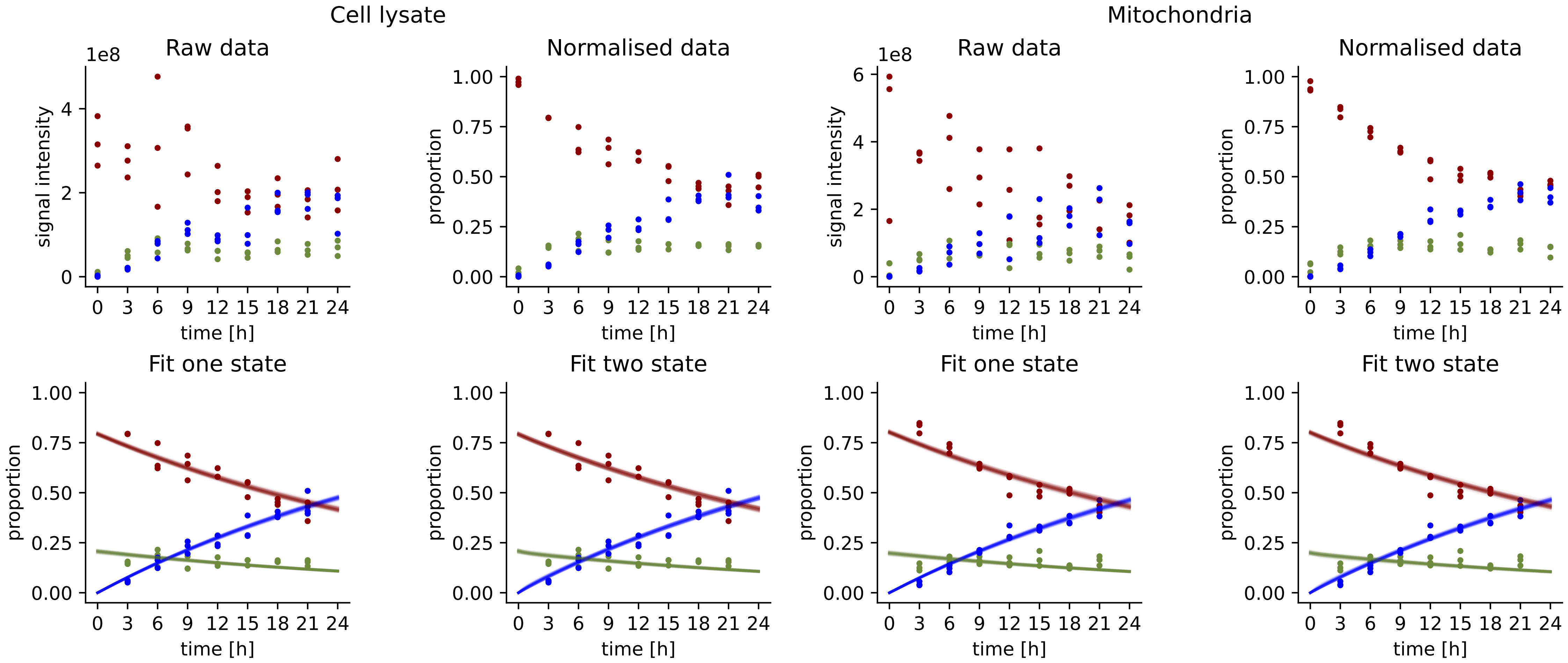

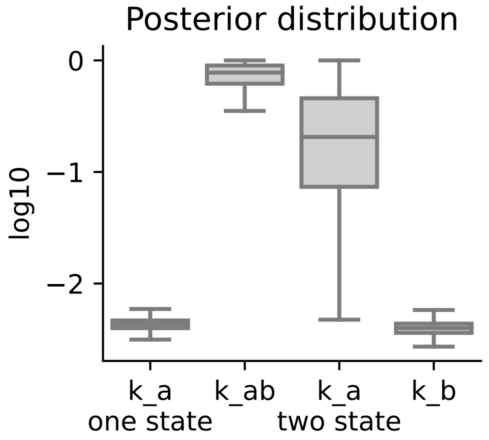

p value	4.36E-01
log likelihood one state	115.534
log likelihood two state	116.364
rmse one state	0.301
rmse two state	0.303
k_a one state	0.004
k_ab	0.778
k_a two state	0.206
k_b	0.004
sigma one state	0.039
sigma two state	0.04

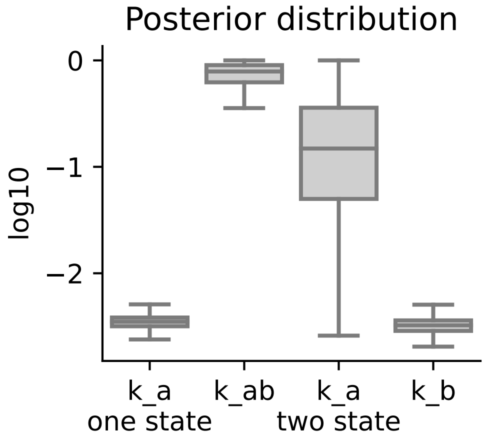

p value	2.83E-01
log likelihood one state	115.425
log likelihood two state	116.686
rmse one state	0.298
rmse two state	0.299
k_a one state	0.004
k_ab	0.787
k_a two state	0.149
k_b	0.003
sigma one state	0.04
sigma two state	0.04

mL48
MRPL48
Q96GC5
39S ribosomal protein L48, mitochondrial

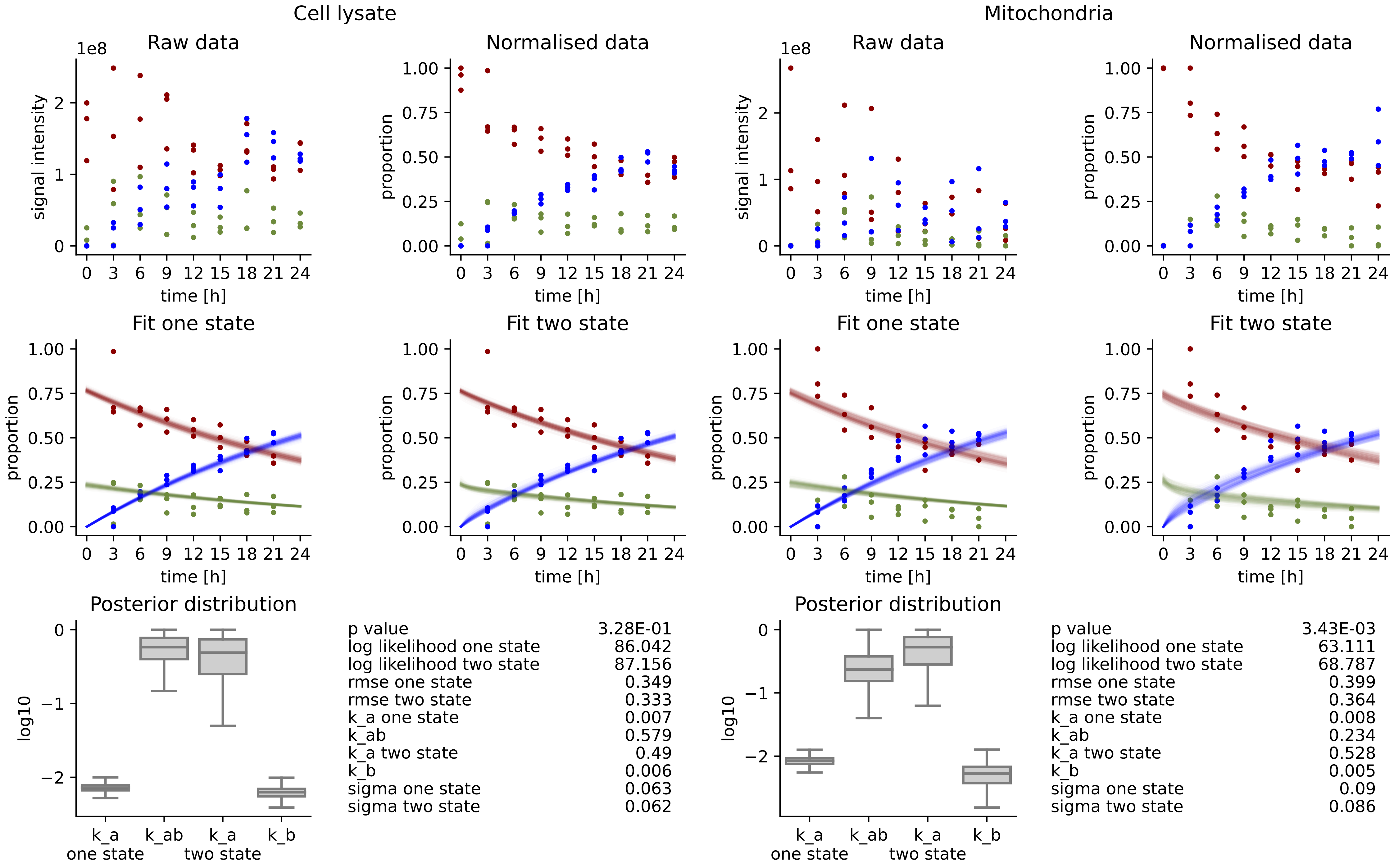

Cell lysate	
p value	3.28E-01
log likelihood one state	86.042
log likelihood two state	87.156
rmse one state	0.349
rmse two state	0.333
k_a one state	0.007
k_ab	0.579
k_a two state	0.49
k_b	0.006
sigma one state	0.063
sigma two state	0.062

Mitochondria	
p value	3.43E-03
log likelihood one state	63.111
log likelihood two state	68.787
rmse one state	0.399
rmse two state	0.364
k_a one state	0.008
k_ab	0.234
k_a two state	0.528
k_b	0.005
sigma one state	0.09
sigma two state	0.086

mL49
MRPL49
Q13405
39S ribosomal protein L49, mitochondrial

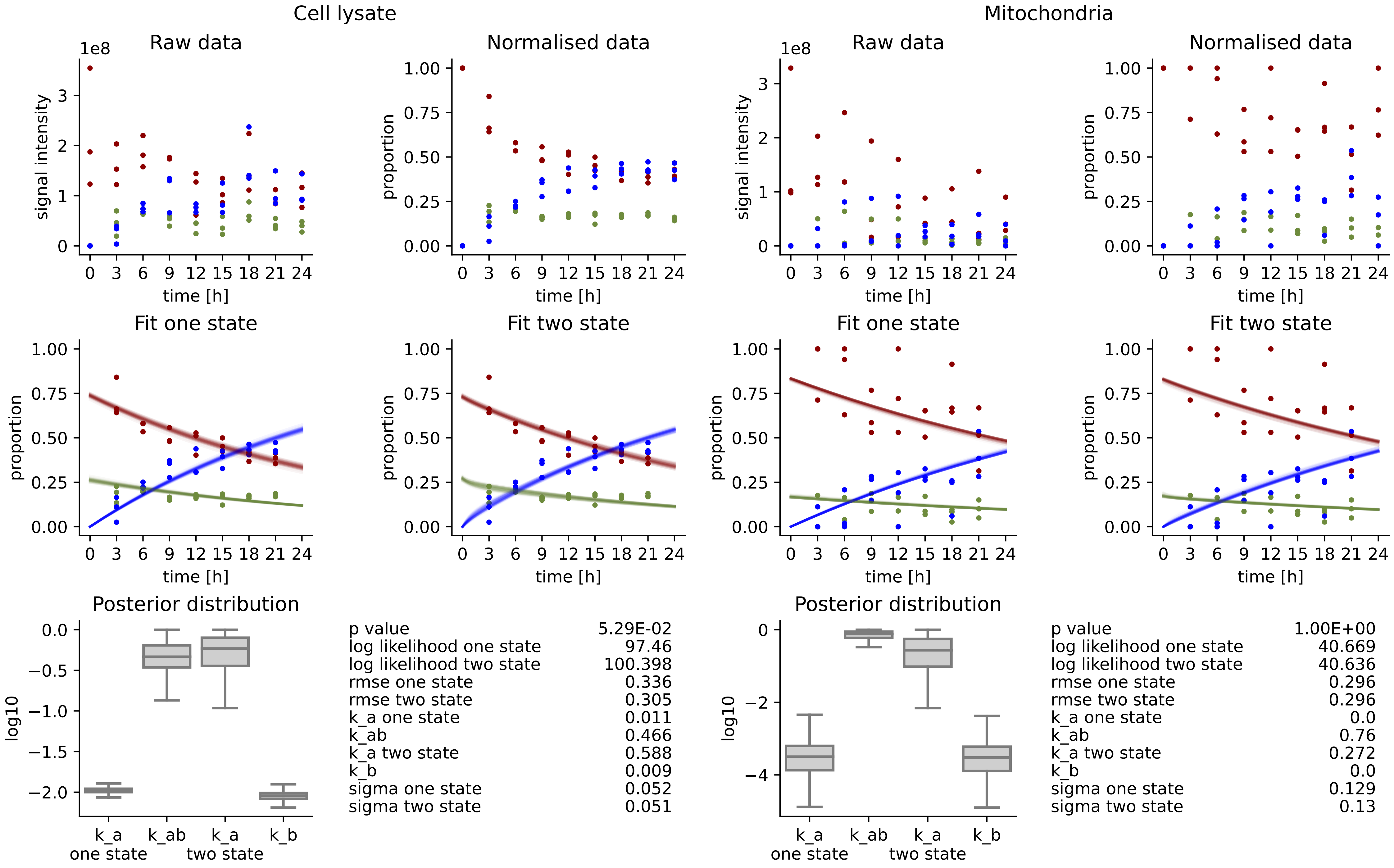

p value	5.29E-02
log likelihood one state	97.46
log likelihood two state	100.398
rmse one state	0.336
rmse two state	0.305
k_a one state	0.011
k_ab	0.466
k_a two state	0.588
k_b	0.009
sigma one state	0.052
sigma two state	0.051

p value	1.00E+00
log likelihood one state	40.669
log likelihood two state	40.636
rmse one state	0.296
rmse two state	0.296
k_a one state	0.0
k_ab	0.76
k_a two state	0.272
k_b	0.0
sigma one state	0.129
sigma two state	0.13

mL50
MRPL50
Q8N5N7
39S ribosomal protein L50, mitochondrial

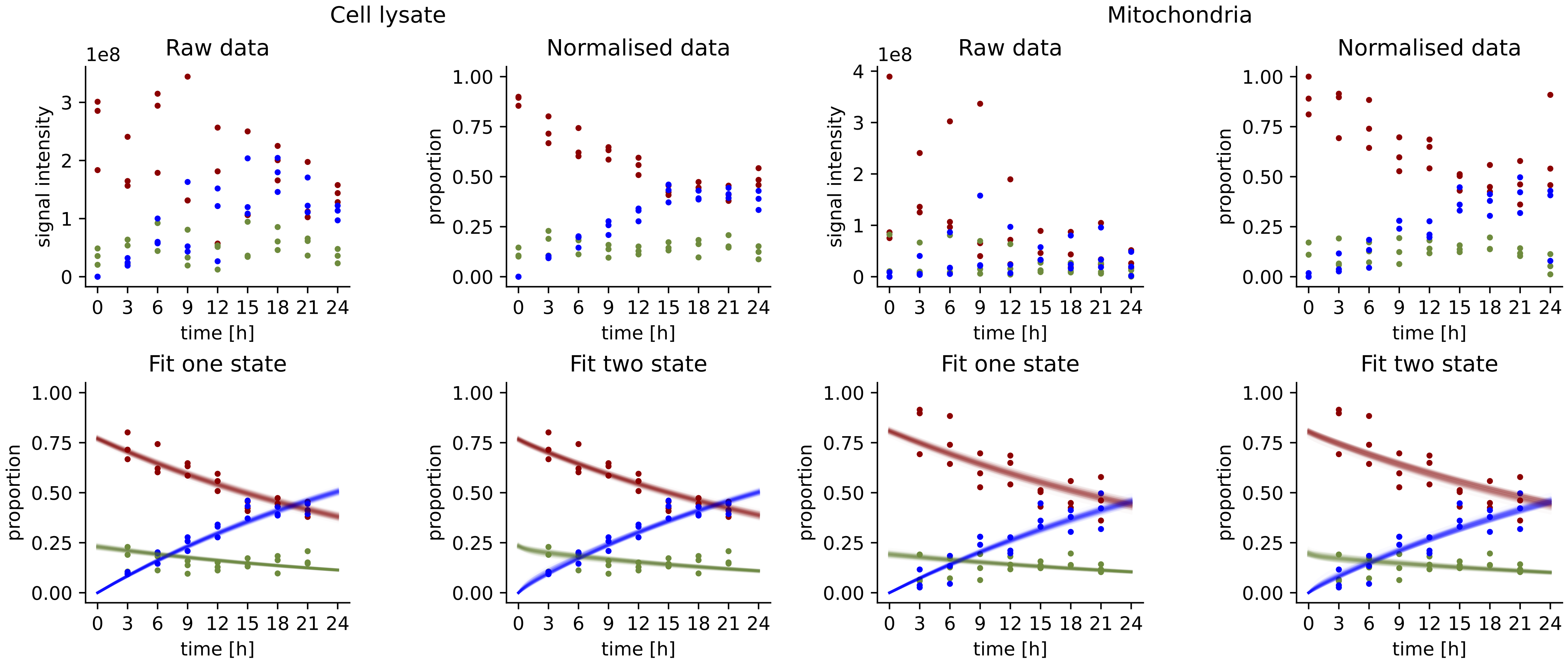

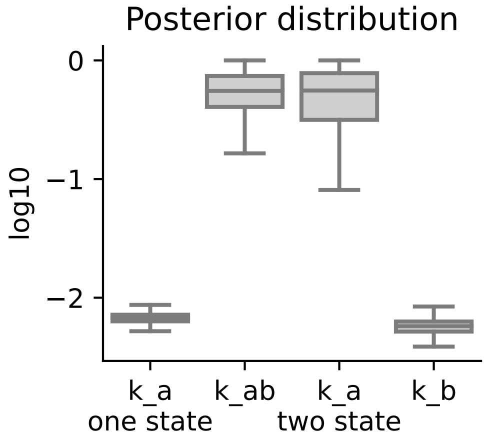

p value	1.48E-01
log likelihood one state	105.281
log likelihood two state	107.19
rmse one state	0.322
rmse two state	0.3
k_a one state	0.007
k_ab	0.553
k_a two state	0.557
k_b	0.006
sigma one state	0.046
sigma two state	0.045

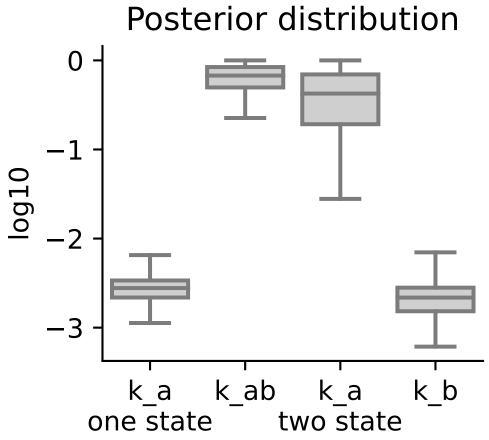

p value	9.80E-01
log likelihood one state	77.265
log likelihood two state	77.284
rmse one state	0.319
rmse two state	0.315
k_a one state	0.003
k_ab	0.675
k_a two state	0.424
k_b	0.002
sigma one state	0.072
sigma two state	0.072

mL51
MRPL51
Q4U2R6
39S ribosomal protein L51, mitochondrial

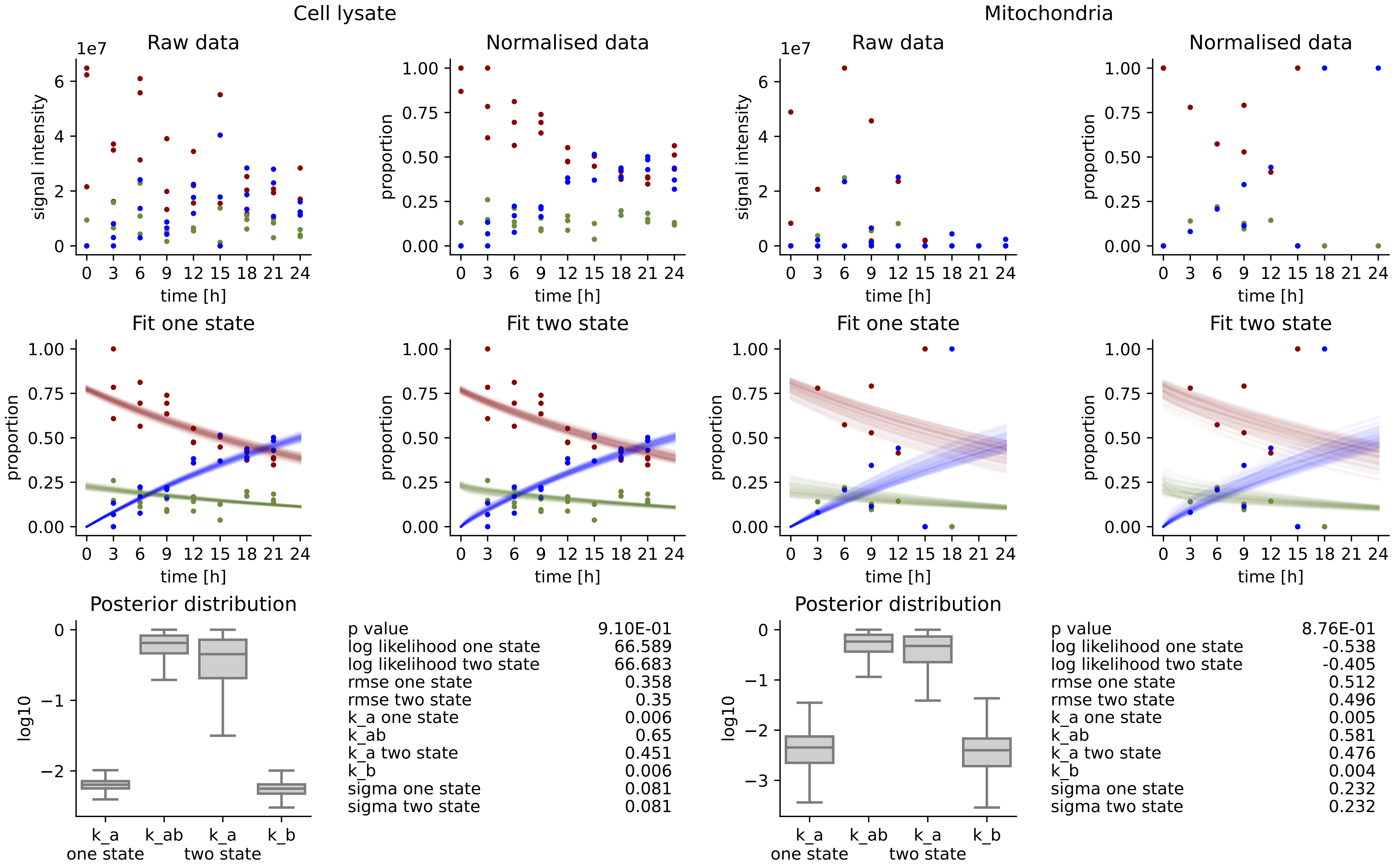

Cell lysate

p value	9.10E-01
log likelihood one state	66.589
log likelihood two state	66.683
rmse one state	0.358
rmse two state	0.35
k_a one state	0.006
k_ab	0.65
k_a two state	0.451
k_b	0.006
sigma one state	0.081
sigma two state	0.081

Mitochondria

p value	8.76E-01
log likelihood one state	-0.538
log likelihood two state	-0.405
rmse one state	0.512
rmse two state	0.496
k_a one state	0.005
k_ab	0.581
k_a two state	0.476
k_b	0.004
sigma one state	0.232
sigma two state	0.232

mL52
MRPL52
Q86TS9
39S ribosomal protein L52, mitochondrial

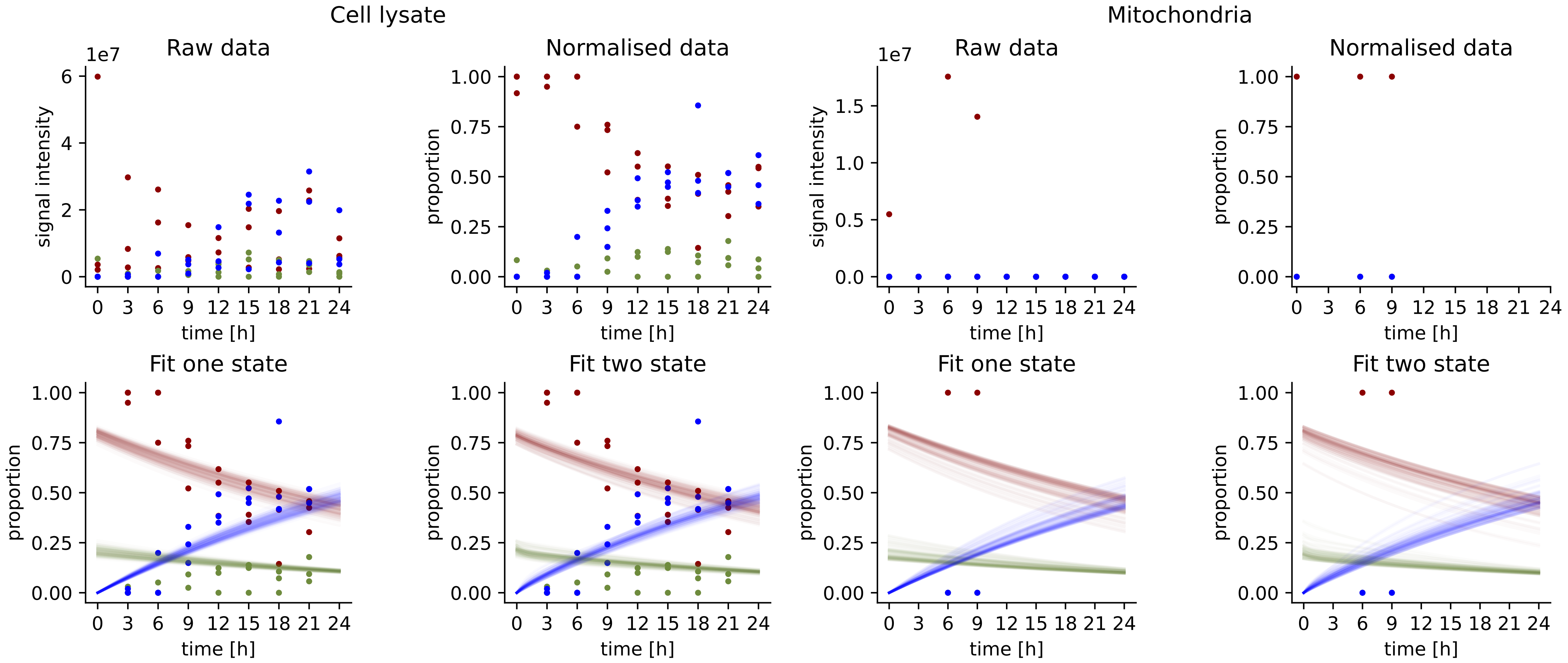

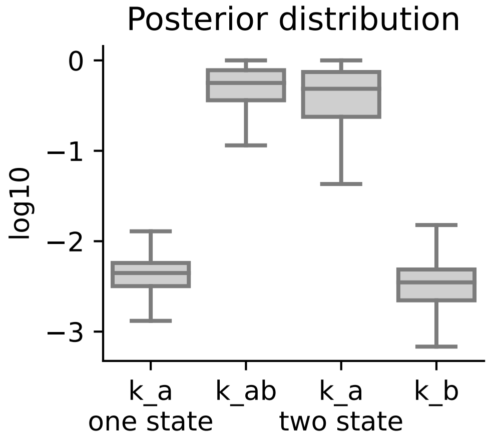

p value	6.55E-01
log likelihood one state	30.016
log likelihood two state	30.439
rmse one state	0.462
rmse two state	0.444
k_a one state	0.004
k_ab	0.563
k_a two state	0.485
k_b	0.004
sigma one state	0.15
sigma two state	0.15

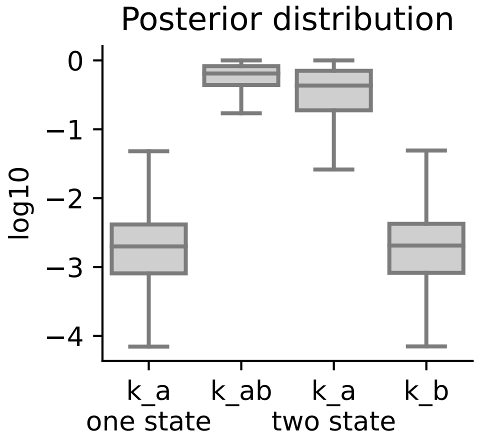

p value	1.00E+00
log likelihood one state	0.885
log likelihood two state	0.874
rmse one state	0.275
rmse two state	0.275
k_a one state	0.002
k_ab	0.645
k_a two state	0.431
k_b	0.002
sigma one state	0.197
sigma two state	0.202

mL53
MRPL53
Q96EL3
39S ribosomal protein L53, mitochondrial

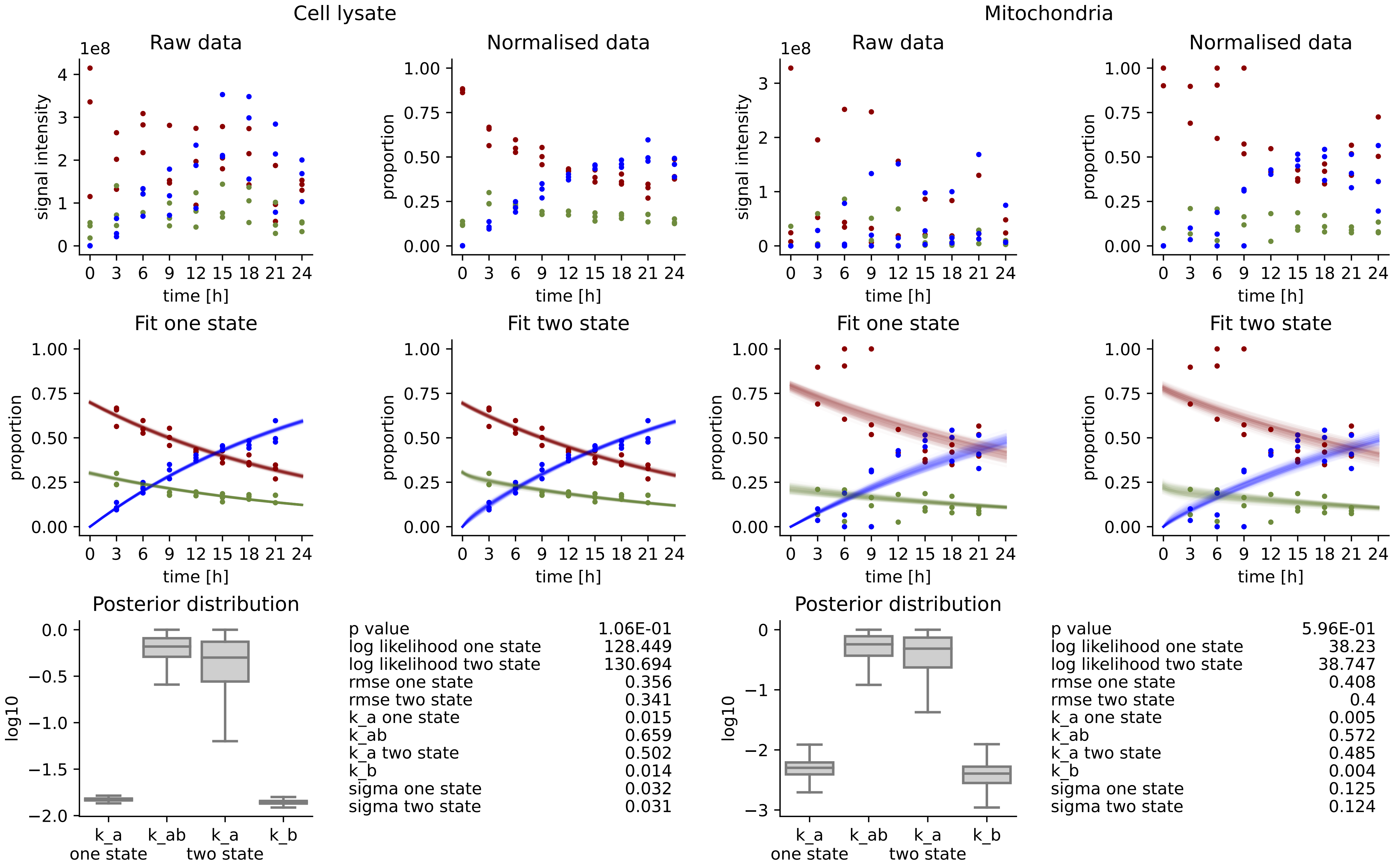

Cell lysate

p value	1.06E-01
log likelihood one state	128.449
log likelihood two state	130.694
rmse one state	0.356
rmse two state	0.341
k_a one state	0.015
k_ab	0.659
k_a two state	0.502
k_b	0.014
sigma one state	0.032
sigma two state	0.031

Mitochondria

p value	5.96E-01
log likelihood one state	38.23
log likelihood two state	38.747
rmse one state	0.408
rmse two state	0.4
k_a one state	0.005
k_ab	0.572
k_a two state	0.485
k_b	0.004
sigma one state	0.125
sigma two state	0.124

mL54
MRPL54
Q6P161
39S ribosomal protein L54, mitochondrial

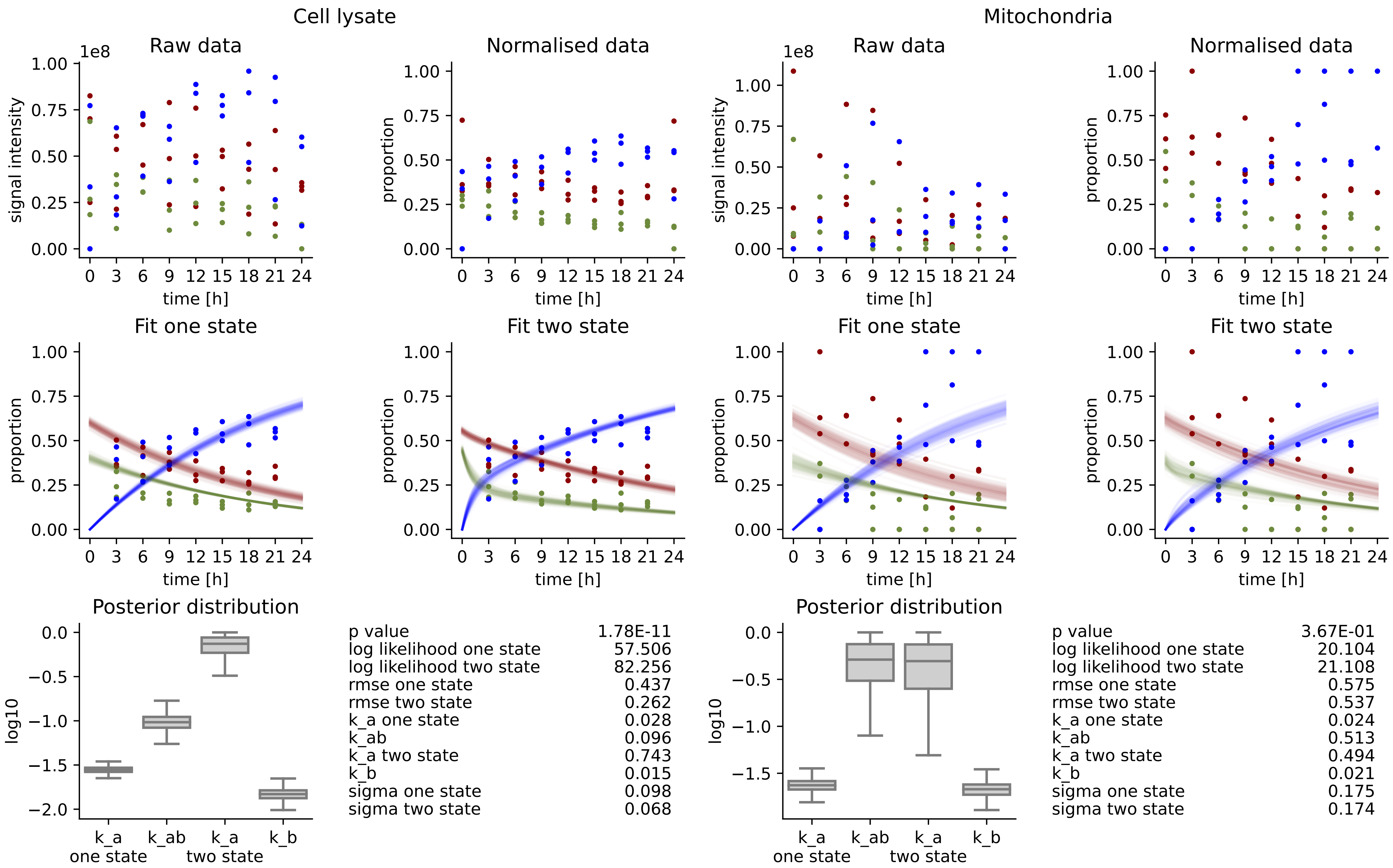

p value	1.78E-11
log likelihood one state	57.506
log likelihood two state	82.256
rmse one state	0.437
rmse two state	0.262
k_a one state	0.028
k_ab	0.096
k_a two state	0.743
k_b	0.015
sigma one state	0.098
sigma two state	0.068

p value	3.67E-01
log likelihood one state	20.104
log likelihood two state	21.108
rmse one state	0.575
rmse two state	0.537
k_a one state	0.024
k_ab	0.513
k_a two state	0.494
k_b	0.021
sigma one state	0.175
sigma two state	0.174

mL62
ICT1
Q14197
Peptidyl-tRNA hydrolase ICT1, mitochondrial

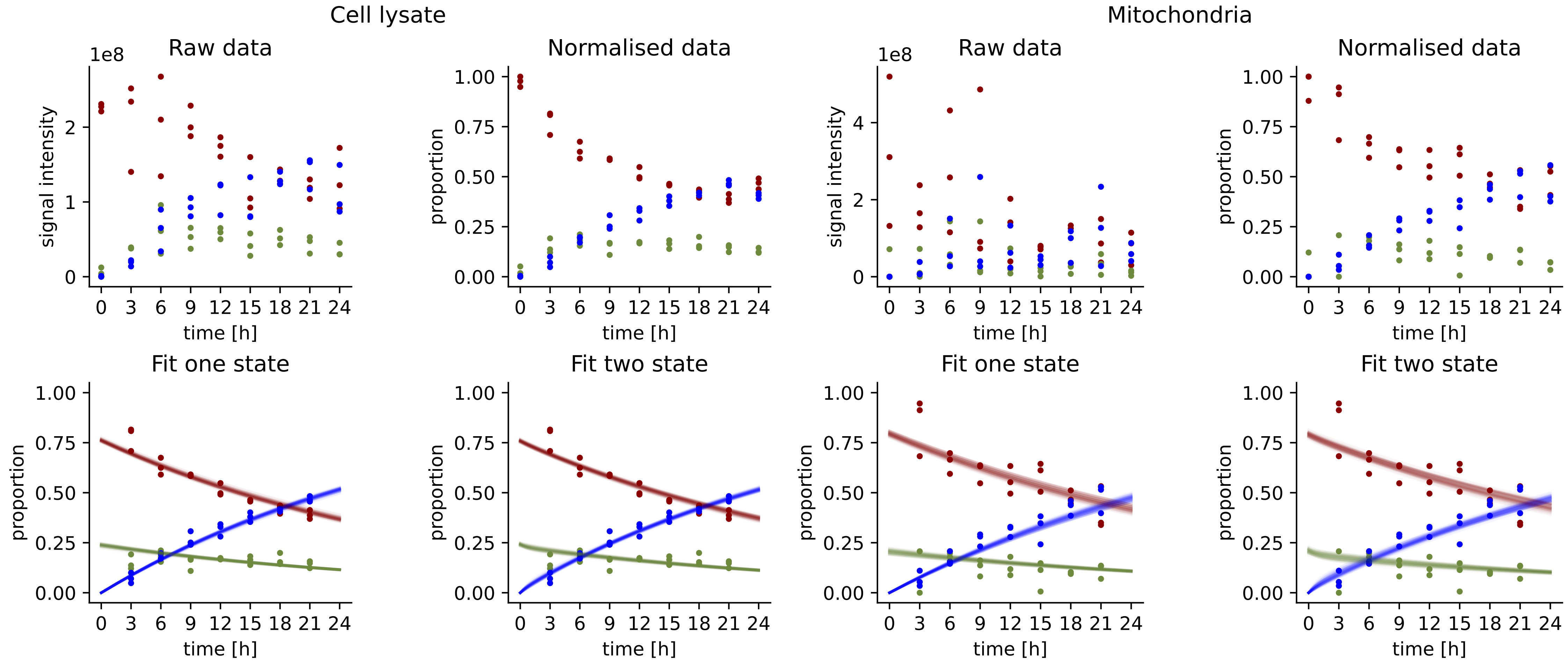

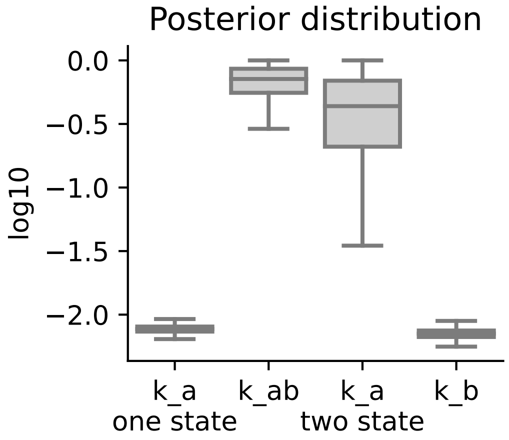

p value	6.00E-01
log likelihood one state	118.914
log likelihood two state	119.425
rmse one state	0.328
rmse two state	0.318
k_a one state	0.008
k_ab	0.714
k_a two state	0.437
k_b	0.007
sigma one state	0.037
sigma two state	0.037

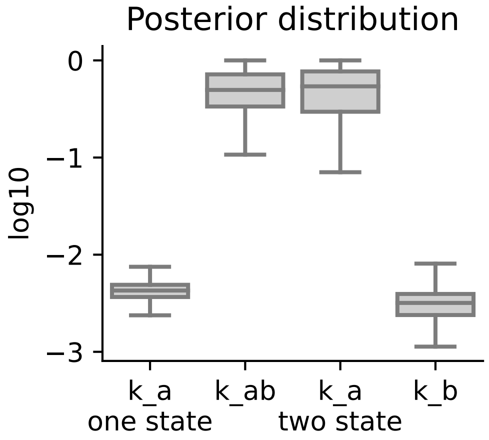

p value	2.28E-01
log likelihood one state	78.025
log likelihood two state	79.503
rmse one state	0.34
rmse two state	0.313
k_a one state	0.004
k_ab	0.496
k_a two state	0.541
k_b	0.003
sigma one state	0.071
sigma two state	0.07

mL63
MRPL57
Q9BQC6
Ribosomal protein 63, mitochondrial

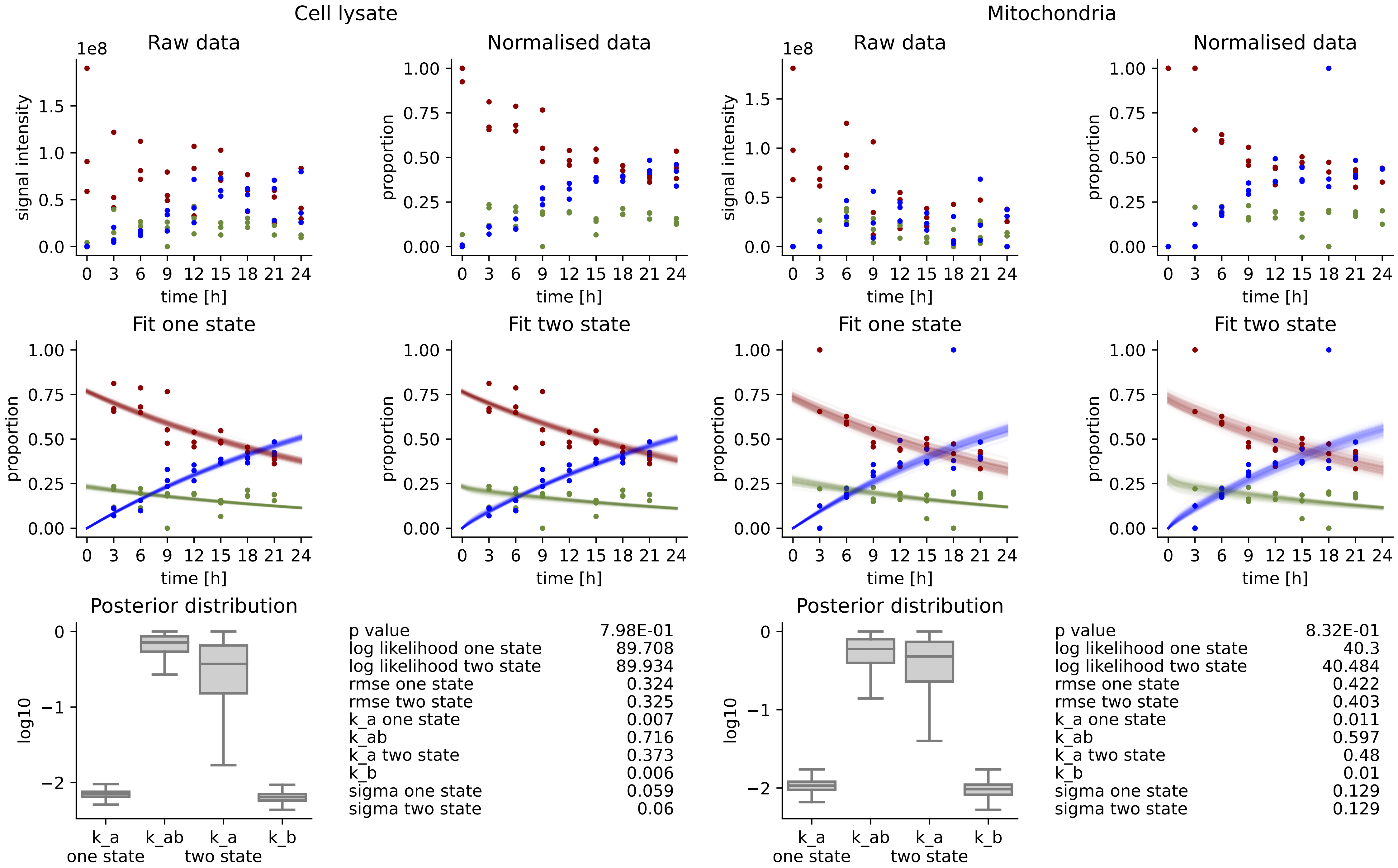

mL64
GADD45GIP1
Q8TAE8
Growth arrest and DNA damage-inducible proteins-interacting protein 1

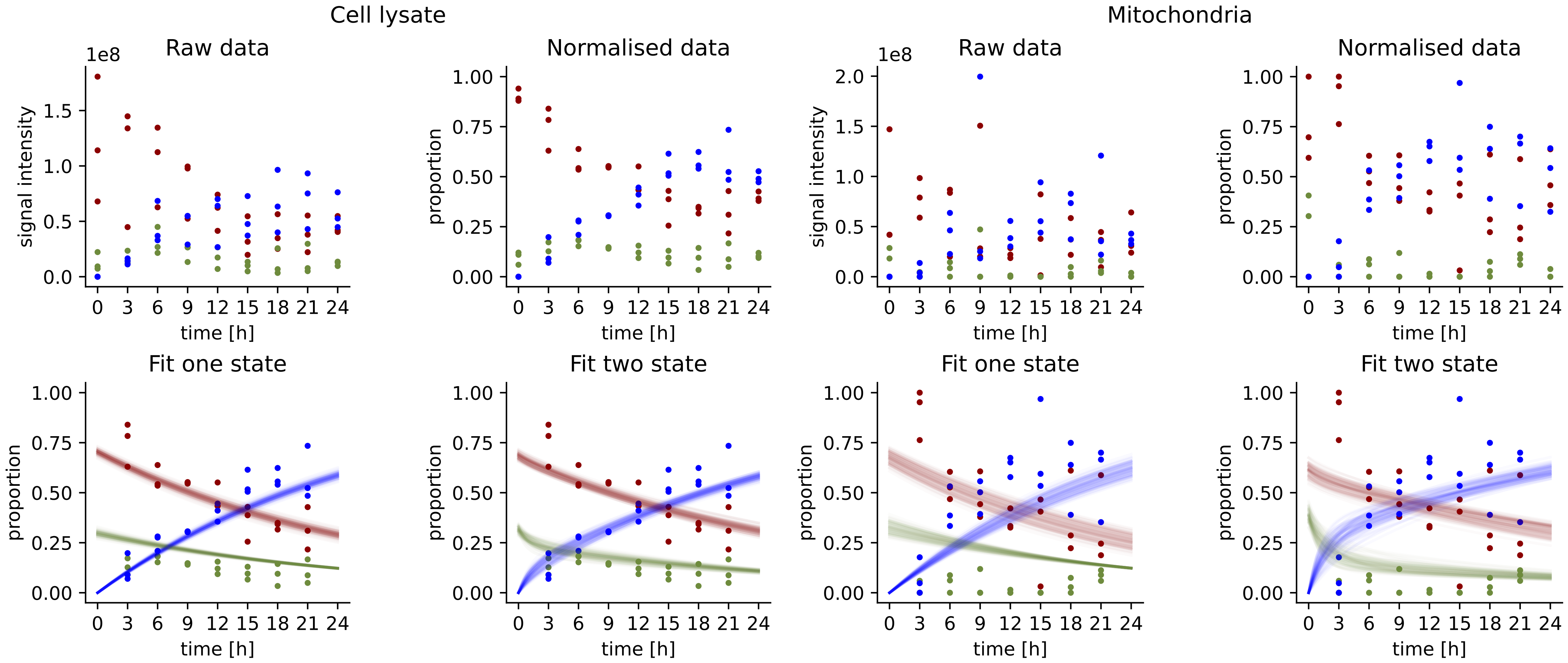

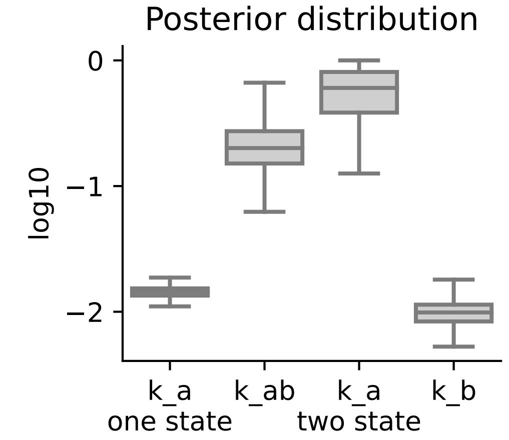

p value	1.34E-03
log likelihood one state	66.797
log likelihood two state	73.409
rmse one state	0.432
rmse two state	0.364
k_a one state	0.014
k_ab	0.201
k_a two state	0.604
k_b	0.01
sigma one state	0.085
sigma two state	0.078

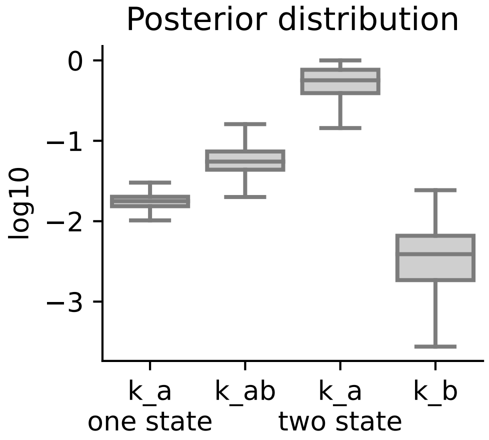

p value	1.12E-05
log likelihood one state	14.415
log likelihood two state	25.811
rmse one state	0.58
rmse two state	0.424
k_a one state	0.018
k_ab	0.055
k_a two state	0.566
k_b	0.004
sigma one state	0.191
sigma two state	0.165

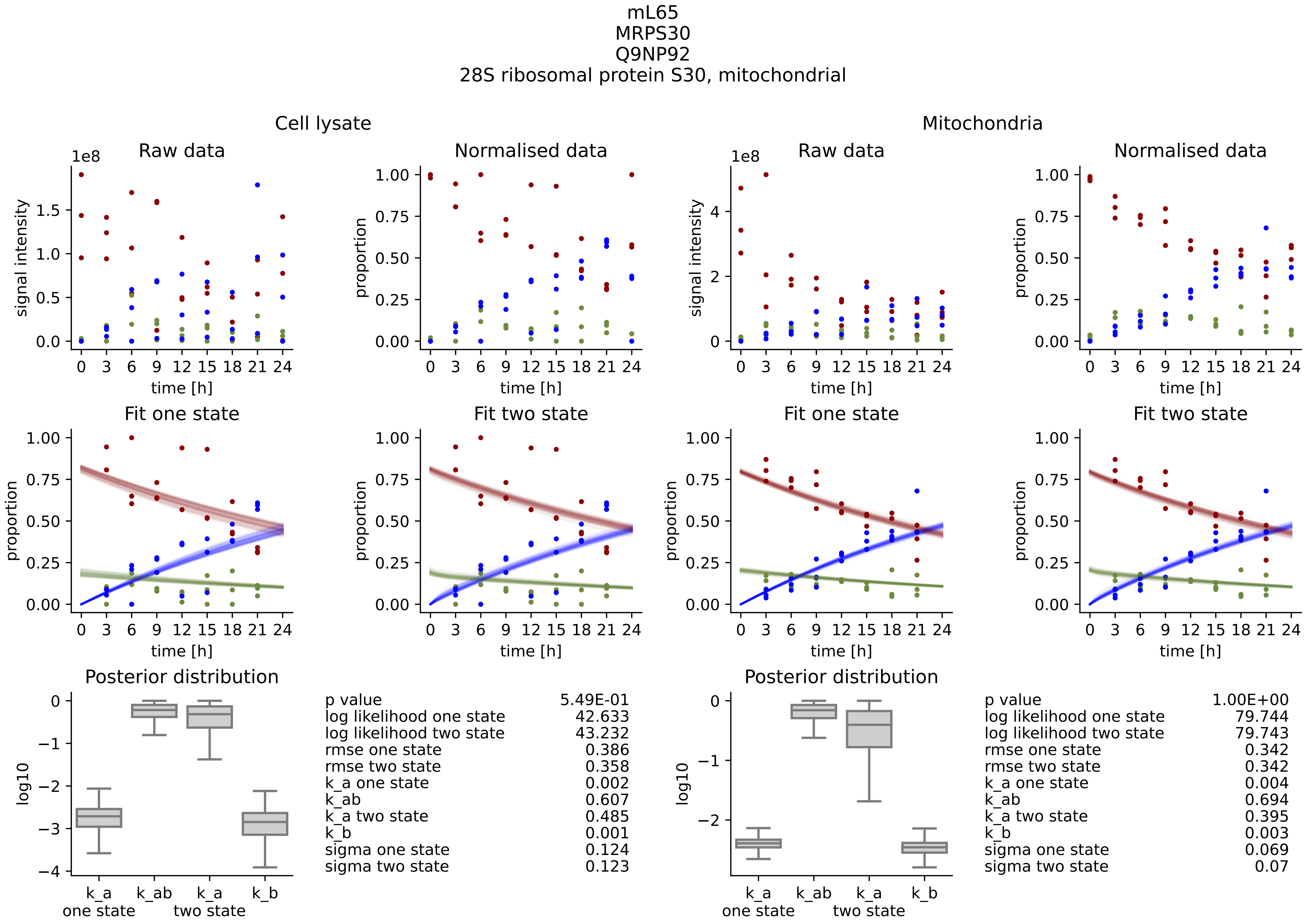
mL65
MRPS30
Q9NP92
28S ribosomal protein S30, mitochondrial
Cell lysate
Mitochondria
Raw data
Normalised data
Raw data
Normalised data
1e8
1e8
signal intensity
proportion
time [h]
Fit one state
Fit two state
Fit one state
Fit two state
Posterior distribution
Posterior distribution
log10
k_a one state
k_ab
k_a two state
k_b
p value 5.49E-01
log likelihood one state 42.633
log likelihood two state 43.232
rmse one state 0.386
rmse two state 0.358
k_a one state 0.002
k_ab 0.607
k_a two state 0.485
k_b 0.001
sigma one state 0.124
sigma two state 0.123
p value 1.00E+00
log likelihood one state 79.744
log likelihood two state 79.743
rmse one state 0.342
rmse two state 0.342
k_a one state 0.004
k_ab 0.694
k_a two state 0.395
k_b 0.003
sigma one state 0.069
sigma two state 0.07

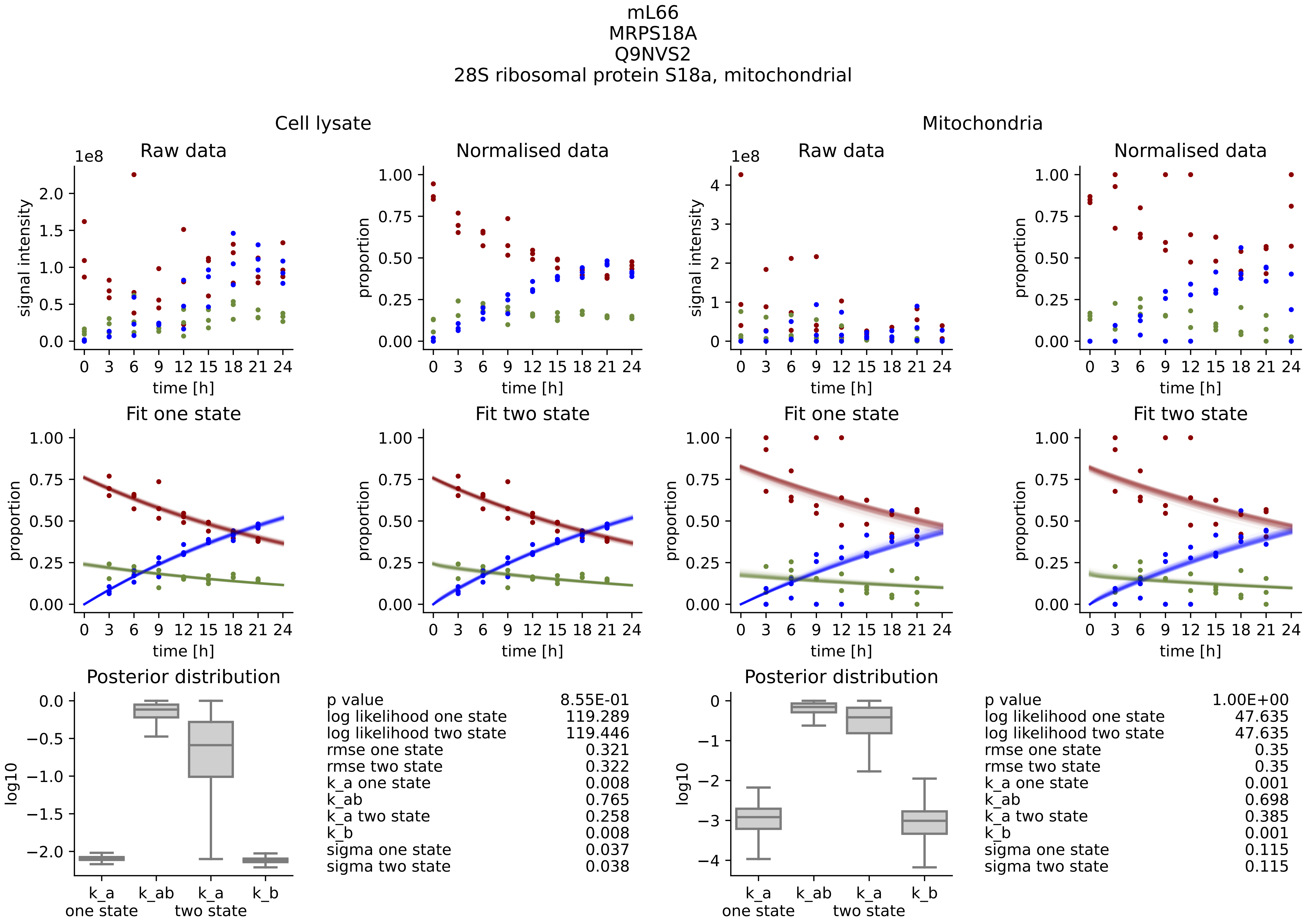
mL66
MRPS18A
Q9NVS2
28S ribosomal protein S18a, mitochondrial
Cell lysate
Mitochondria
Raw data
Normalised data
Raw data
Normalised data
1e8
signal intensity
proportion
time [h]
Fit one state
Fit two state
Fit one state
Fit two state
Posterior distribution
Posterior distribution
log10
k_a
one state
k_ab
k_a
two state
k_b
p value 8.55E-01
log likelihood one state 119.289
log likelihood two state 119.446
rmse one state 0.321
rmse two state 0.322
k_a one state 0.008
k_ab 0.765
k_a two state 0.258
k_b 0.008
sigma one state 0.037
sigma two state 0.038
p value 1.00E+00
log likelihood one state 47.635
log likelihood two state 47.635
rmse one state 0.35
rmse two state 0.35
k_a one state 0.001
k_ab 0.698
k_a two state 0.385
k_b 0.001
sigma one state 0.115
sigma two state 0.115

bS1m
MRPS28
Q9Y2Q9
28S ribosomal protein S28, mitochondrial

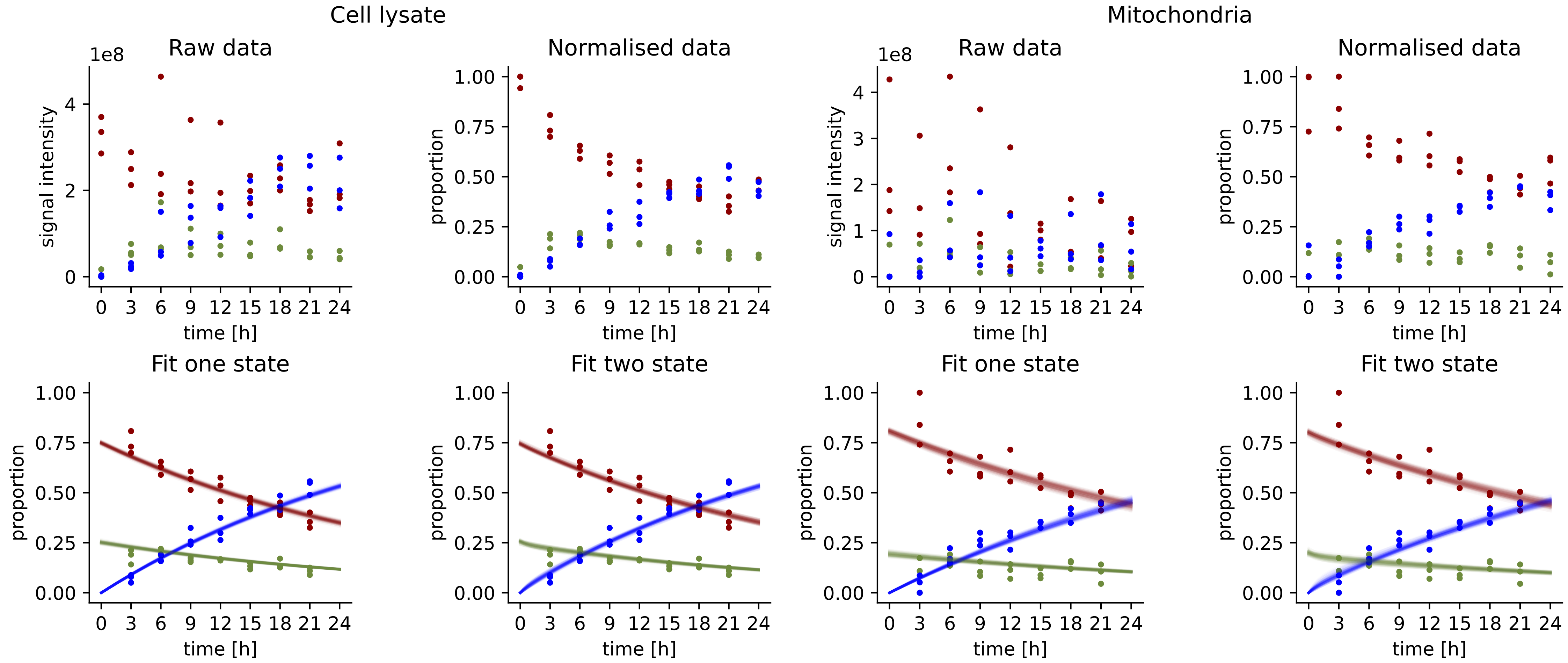

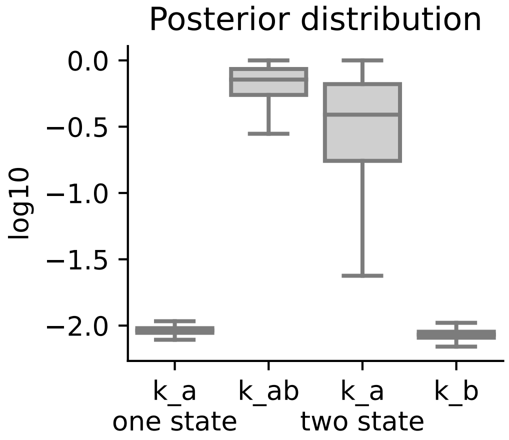

p value	7.16E-01
log likelihood one state	117.13
log likelihood two state	117.464
rmse one state	0.346
rmse two state	0.339
k_a one state	0.009
k_ab	0.717
k_a two state	0.39
k_b	0.009
sigma one state	0.038
sigma two state	0.038

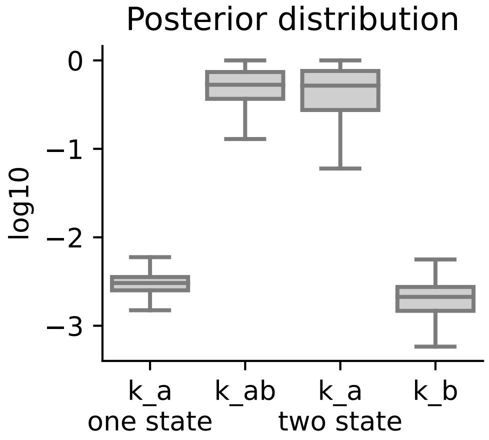

p value	2.36E-01
log likelihood one state	87.709
log likelihood two state	89.151
rmse one state	0.321
rmse two state	0.301
k_a one state	0.003
k_ab	0.53
k_a two state	0.519
k_b	0.002
sigma one state	0.061
sigma two state	0.06

uS2m
MRPS2
Q9Y399
28S ribosomal protein S2, mitochondrial

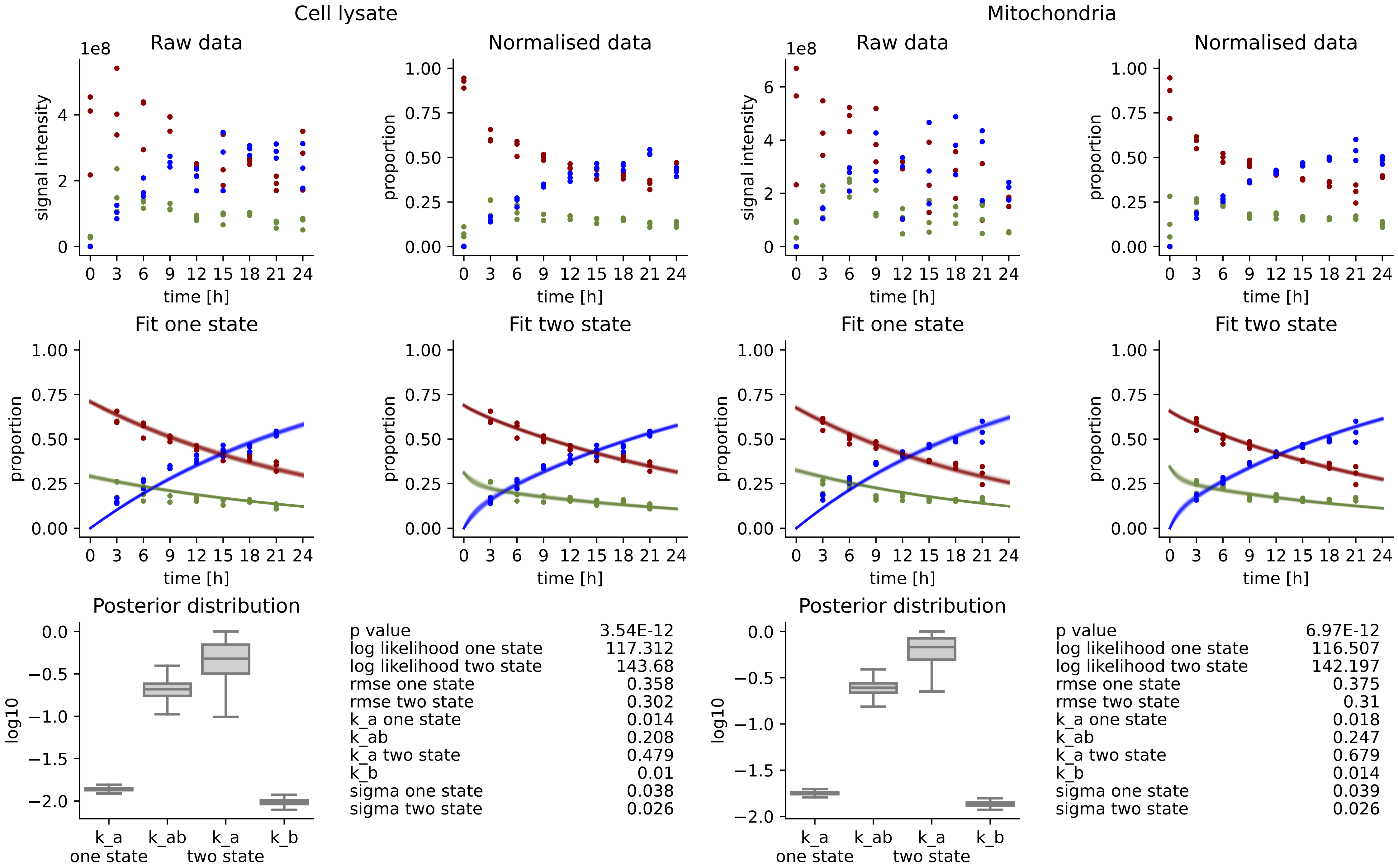

Cell lysate

p value	3.54E-12
log likelihood one state	117.312
log likelihood two state	143.68
rmse one state	0.358
rmse two state	0.302
k_a one state	0.014
k_ab	0.208
k_a two state	0.479
k_b	0.01
sigma one state	0.038
sigma two state	0.026

Mitochondria

p value	6.97E-12
log likelihood one state	116.507
log likelihood two state	142.197
rmse one state	0.375
rmse two state	0.31
k_a one state	0.018
k_ab	0.247
k_a two state	0.679
k_b	0.014
sigma one state	0.039
sigma two state	0.026

uS3m
MRPS24
Q96EL2
28S ribosomal protein S24, mitochondrial

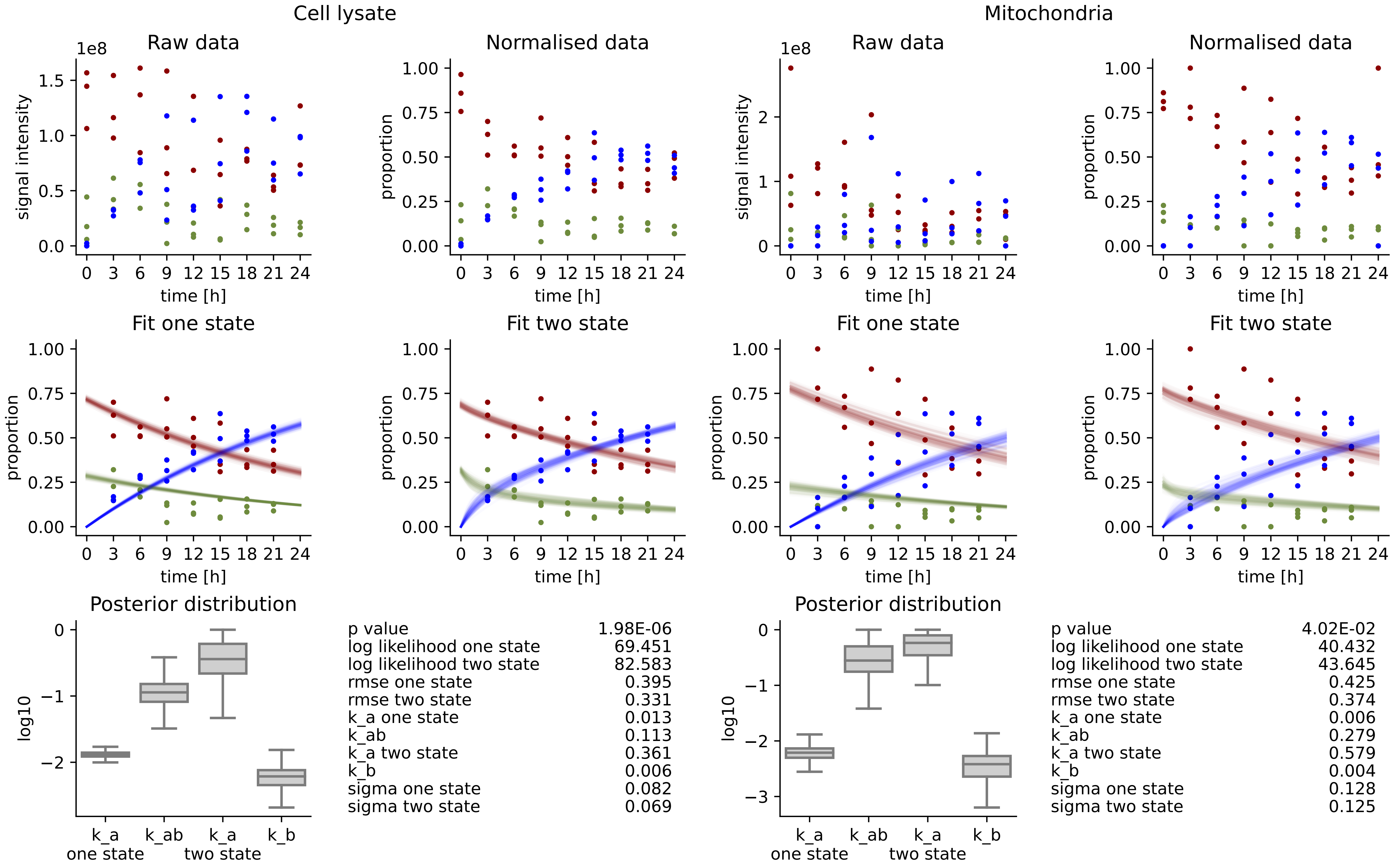

uS5m
MRPS5
P82675
28S ribosomal protein S5, mitochondrial

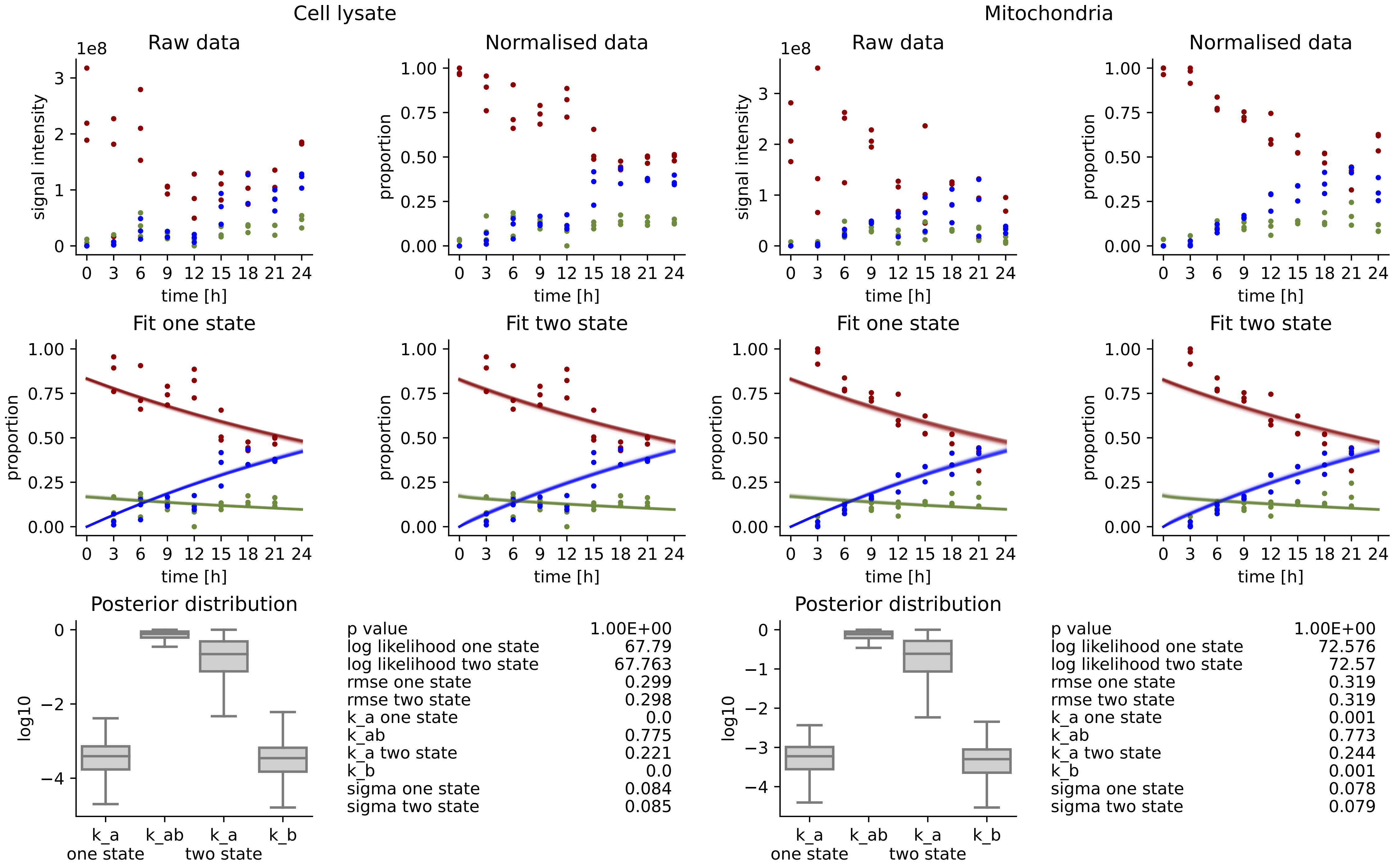

bS6m
MRPS6
P82932
28S ribosomal protein S6, mitochondrial

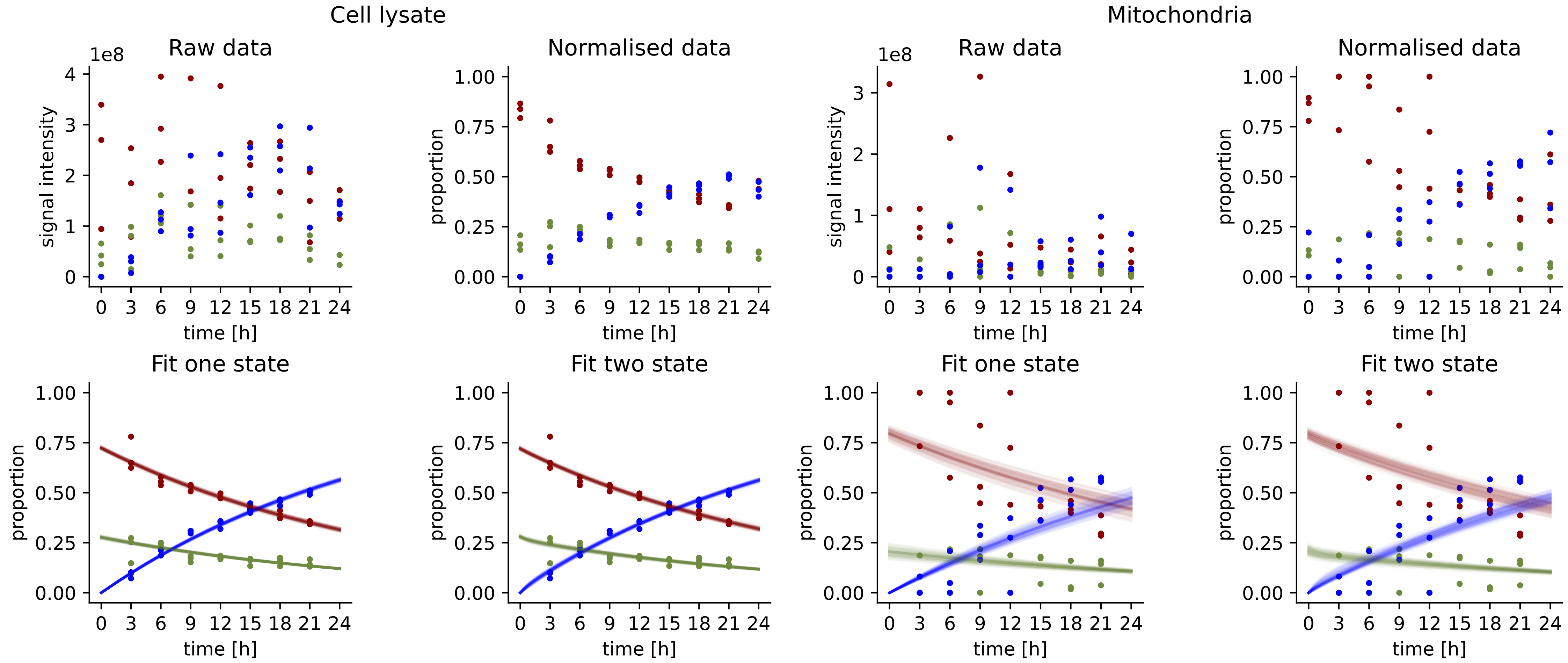

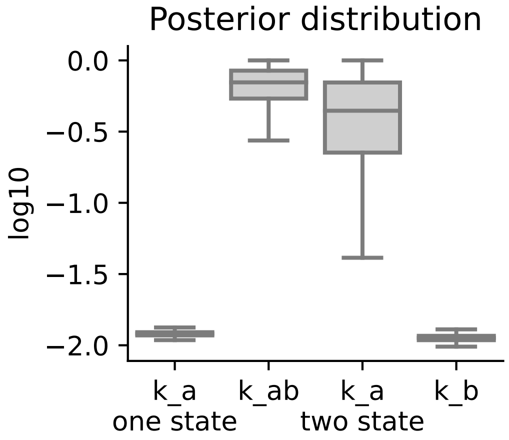

p value	2.13E-01
log likelihood one state	133.572
log likelihood two state	135.117
rmse one state	0.343
rmse two state	0.332
k_a one state	0.012
k_ab	0.701
k_a two state	0.443
k_b	0.011
sigma one state	0.03
sigma two state	0.029

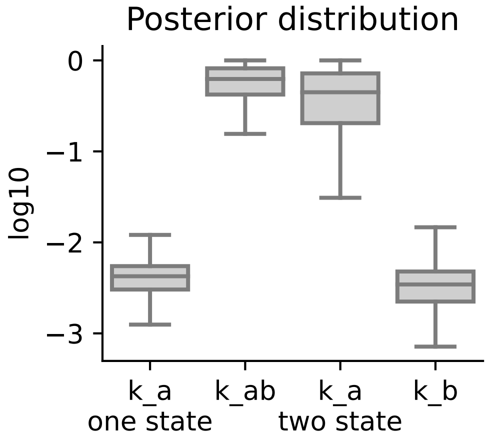

p value	9.74E-01
log likelihood one state	32.168
log likelihood two state	32.195
rmse one state	0.438
rmse two state	0.436
k_a one state	0.004
k_ab	0.625
k_a two state	0.447
k_b	0.003
sigma one state	0.146
sigma two state	0.146

uS7m
MRPS7
Q9Y2R9
28S ribosomal protein S7, mitochondrial

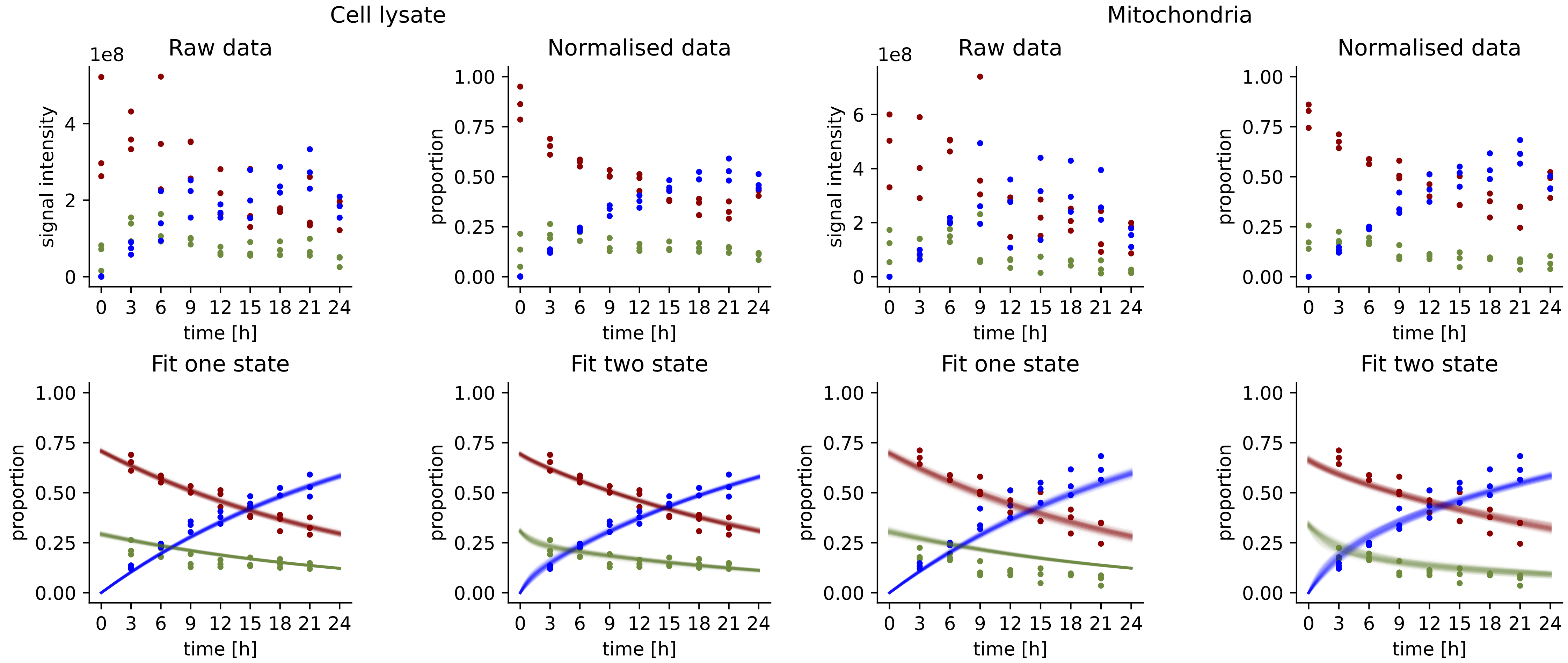

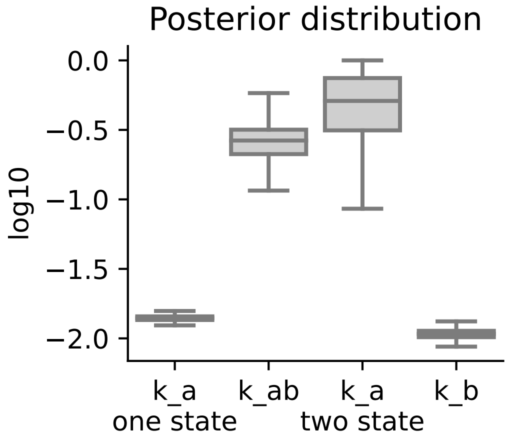

p value	2.94E-07
log likelihood one state	117.211
log likelihood two state	132.25
rmse one state	0.371
rmse two state	0.329
k_a one state	0.014
k_ab	0.265
k_a two state	0.511
k_b	0.011
sigma one state	0.038
sigma two state	0.031

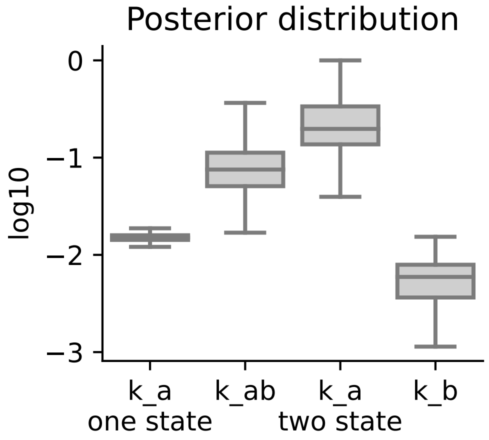

p value	5.33E-09
log likelihood one state	76.935
log likelihood two state	95.984
rmse one state	0.426
rmse two state	0.371
k_a one state	0.015
k_ab	0.075
k_a two state	0.197
k_b	0.006
sigma one state	0.073
sigma two state	0.056

uS9m
MRPS9
P82933
28S ribosomal protein S9, mitochondrial

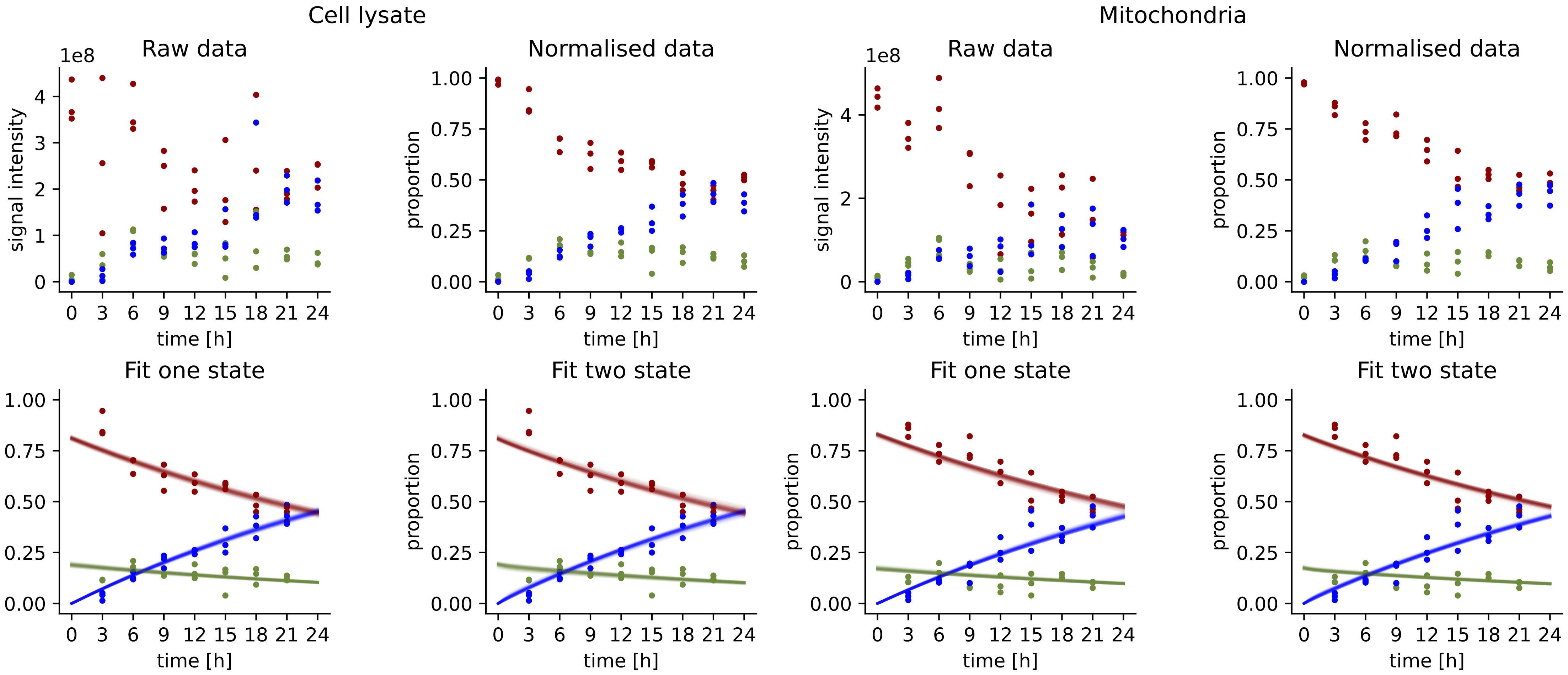

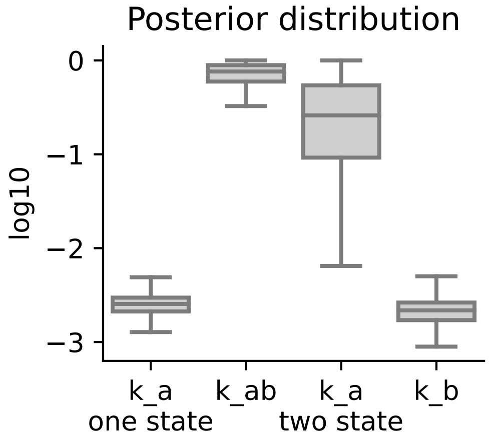

p value	7.62E-01
log likelihood one state	97.693
log likelihood two state	97.965
rmse one state	0.301
rmse two state	0.301
k_a one state	0.003
k_ab	0.762
k_a two state	0.26
k_b	0.002
sigma one state	0.052
sigma two state	0.053

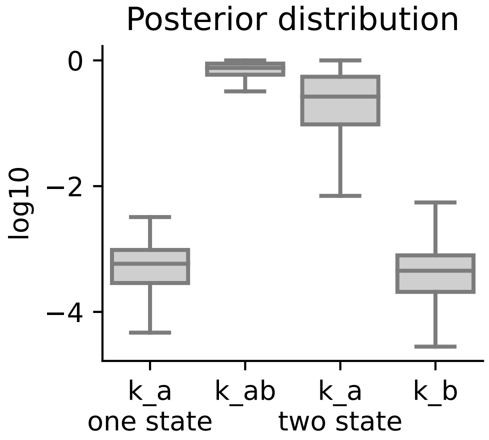

p value	1.00E+00
log likelihood one state	92.261
log likelihood two state	92.26
rmse one state	0.3
rmse two state	0.3
k_a one state	0.001
k_ab	0.759
k_a two state	0.265
k_b	0.0
sigma one state	0.057
sigma two state	0.057

uS10m
MRPS10
P82664
28S ribosomal protein S10, mitochondrial

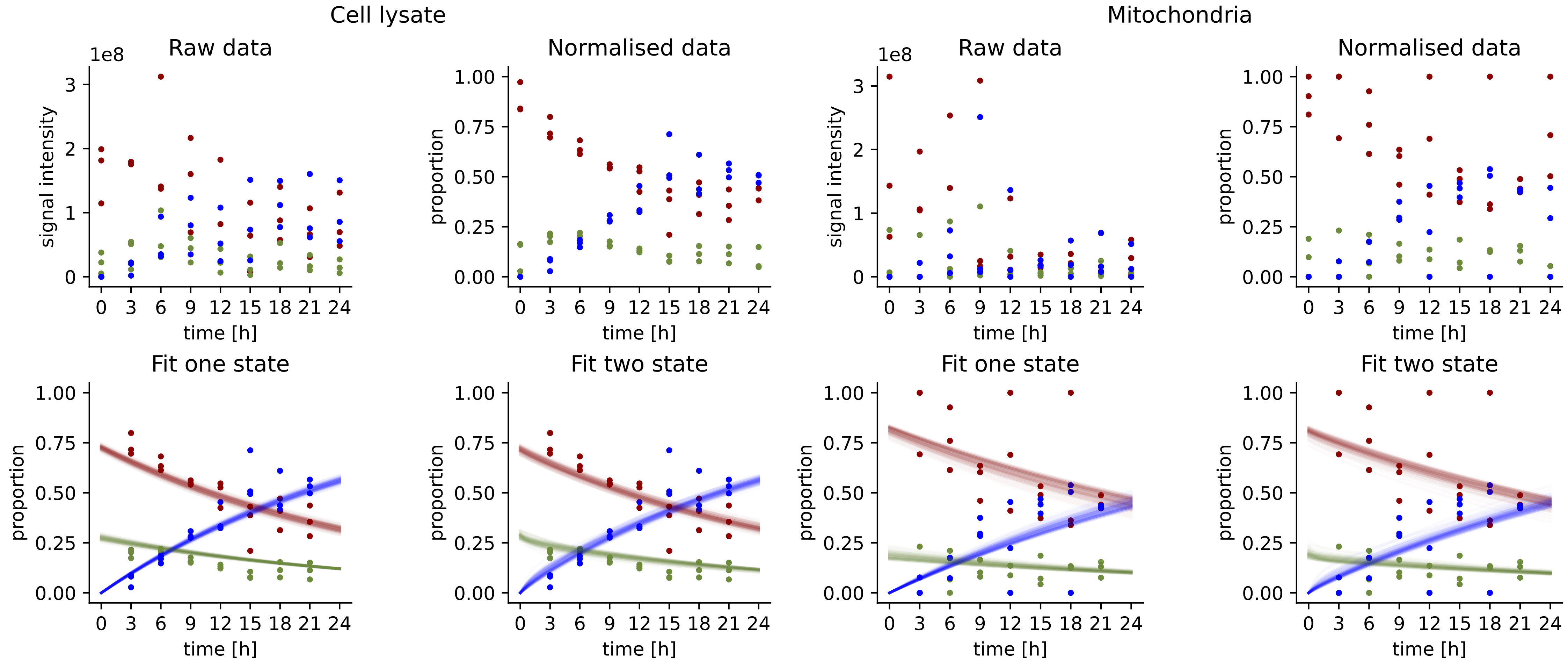

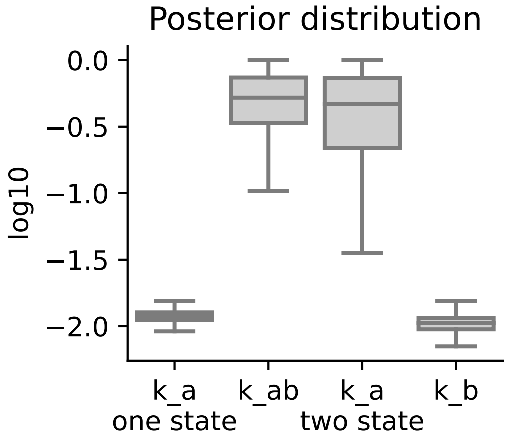

p value	1.01E-01
log likelihood one state	74.27
log likelihood two state	76.564
rmse one state	0.398
rmse two state	0.38
k_a one state	0.012
k_ab	0.522
k_a two state	0.467
k_b	0.011
sigma one state	0.076
sigma two state	0.075

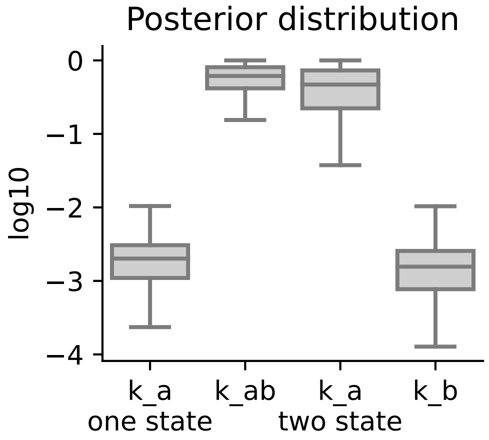

p value	6.64E-01
log likelihood one state	32.3
log likelihood two state	32.71
rmse one state	0.395
rmse two state	0.377
k_a one state	0.002
k_ab	0.614
k_a two state	0.469
k_b	0.002
sigma one state	0.145
sigma two state	0.145

uS11m
MRPS11
P82912
28S ribosomal protein S11, mitochondrial

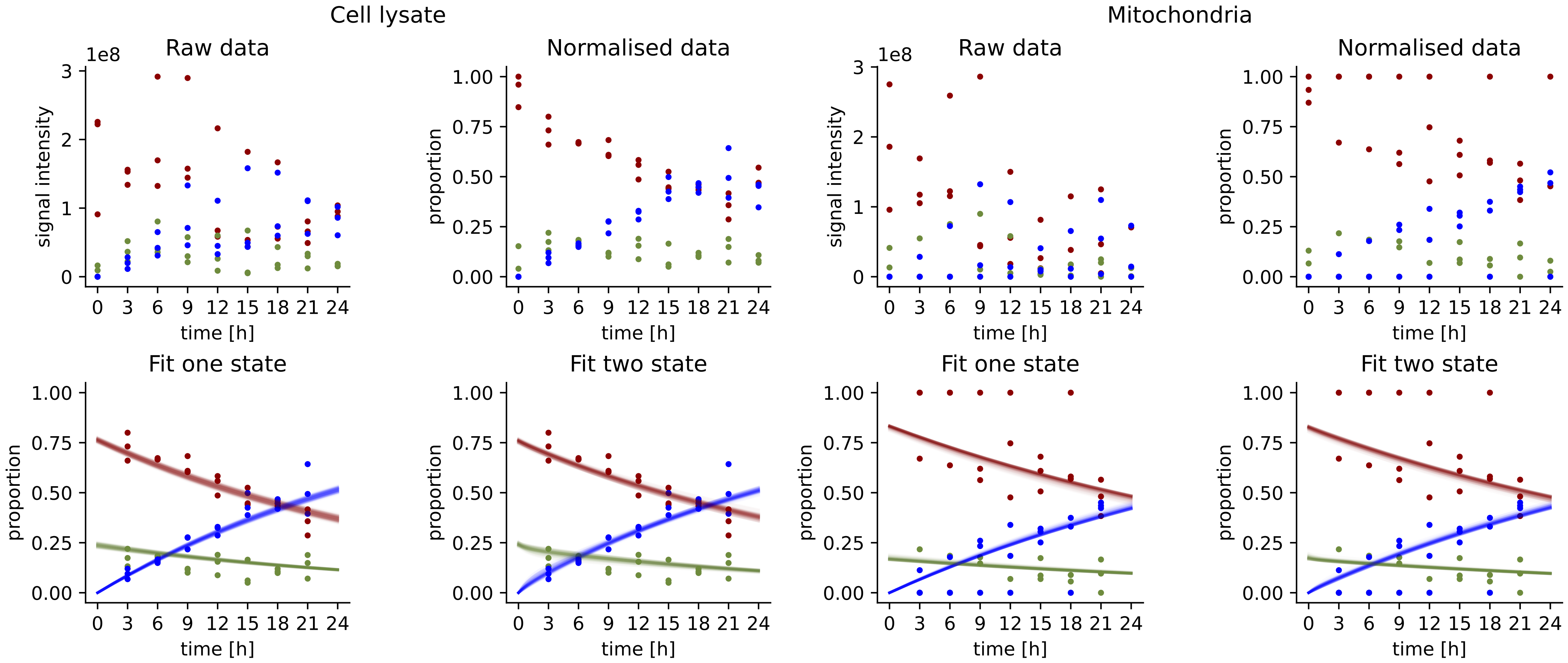

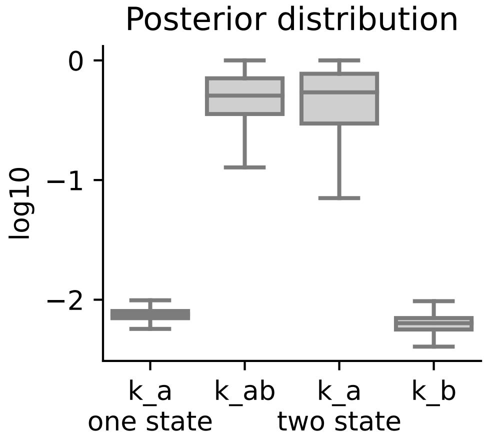

p value	1.18E-01
log likelihood one state	94.265
log likelihood two state	96.401
rmse one state	0.352
rmse two state	0.334
k_a one state	0.008
k_ab	0.508
k_a two state	0.541
k_b	0.006
sigma one state	0.055
sigma two state	0.054

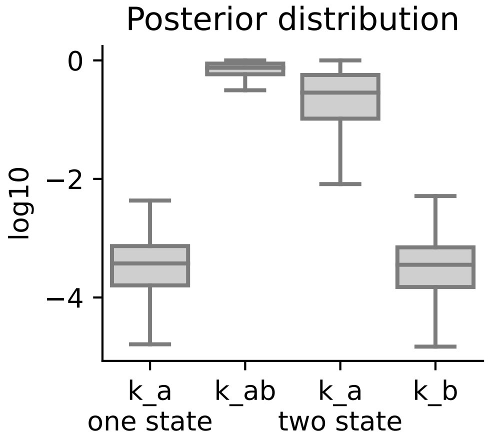

p value	1.00E+00
log likelihood one state	34.34
log likelihood two state	34.329
rmse one state	0.324
rmse two state	0.324
k_a one state	0.0
k_ab	0.751
k_a two state	0.286
k_b	0.0
sigma one state	0.142
sigma two state	0.144

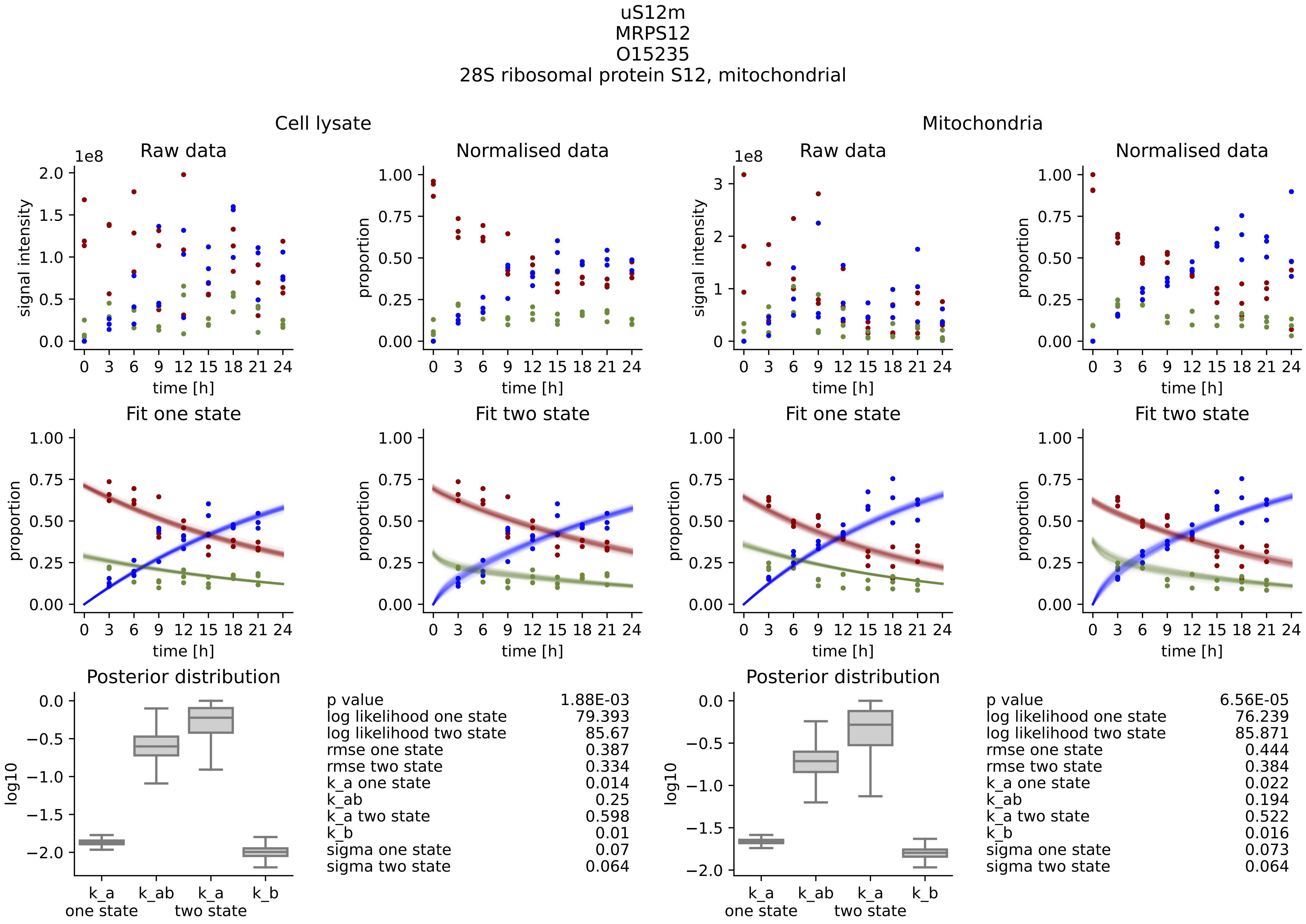
uS12m
MRPS12
O15235
28S ribosomal protein S12, mitochondrial
Cell lysate
Mitochondria
Raw data
Normalised data
Raw data
Normalised data
1e8
1e8
signal intensity
proportion
time [h]
Fit one state
Fit two state
Fit one state
Fit two state
Posterior distribution
Posterior distribution
log10
k_a
one state
k_ab
k_a
two state
k_b
p value 1.88E-03
log likelihood one state 79.393
log likelihood two state 85.67
rmse one state 0.387
rmse two state 0.334
k_a one state 0.014
k_ab 0.25
k_a two state 0.598
k_b 0.01
sigma one state 0.07
sigma two state 0.064
p value 6.56E-05
log likelihood one state 76.239
log likelihood two state 85.871
rmse one state 0.444
rmse two state 0.384
k_a one state 0.022
k_ab 0.194
k_a two state 0.522
k_b 0.016
sigma one state 0.073
sigma two state 0.064

uS14m
MRPS14
O60783
28S ribosomal protein S14, mitochondrial

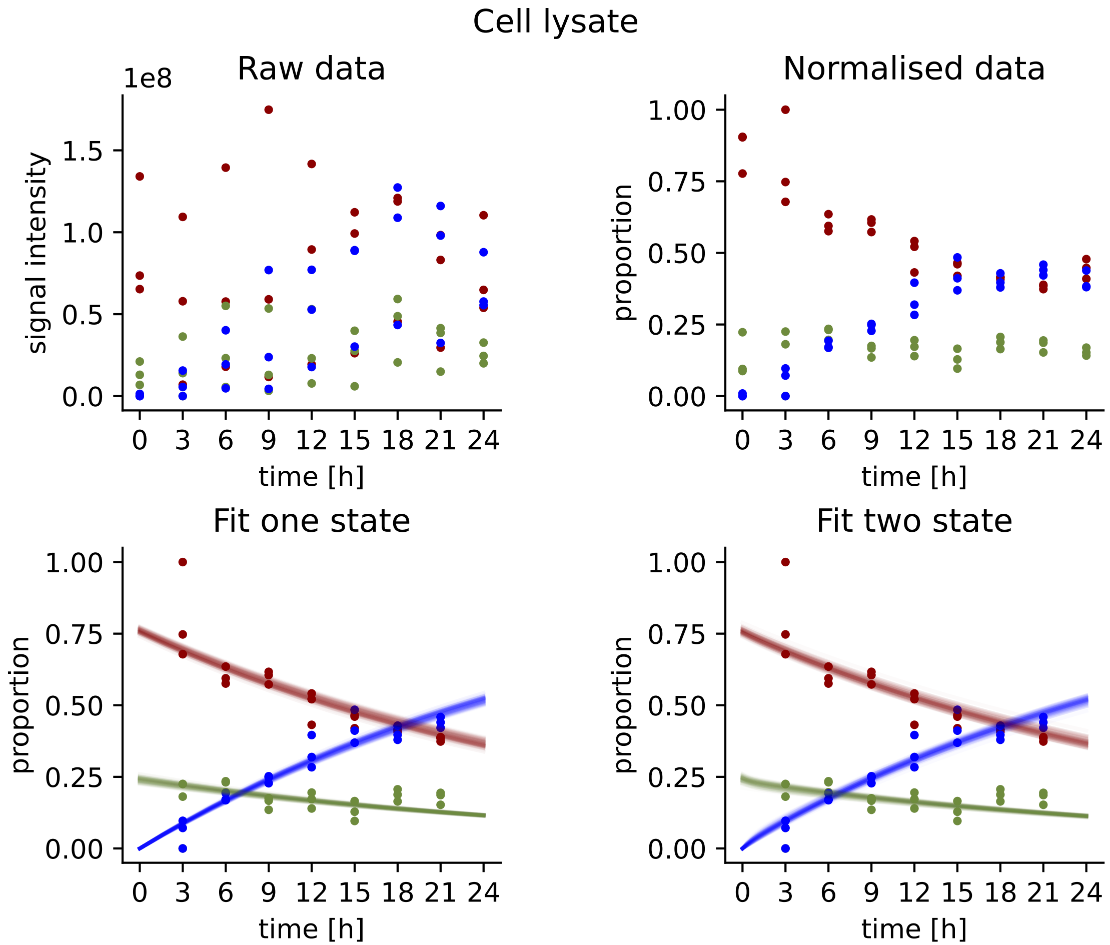

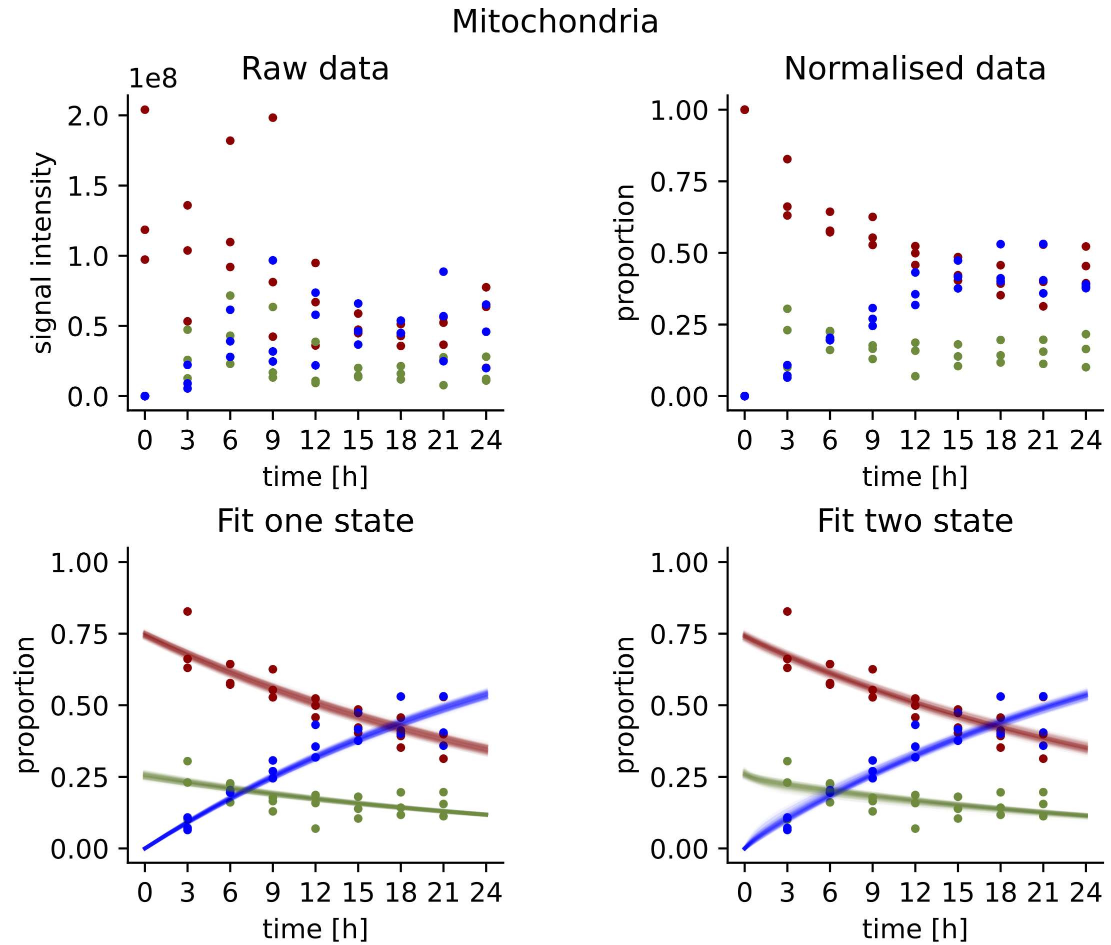

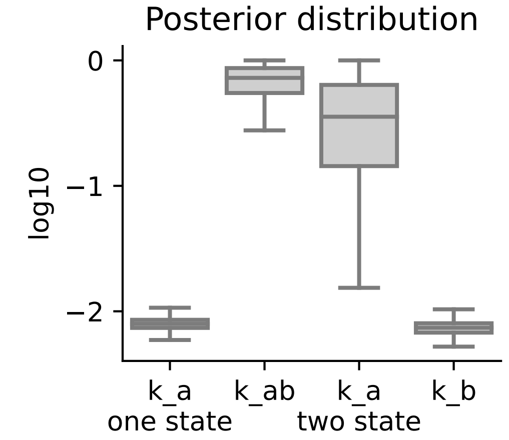

p value	6.69E-01
log likelihood one state	86.405
log likelihood two state	86.807
rmse one state	0.338
rmse two state	0.341
k_a one state	0.008
k_ab	0.726
k_a two state	0.356
k_b	0.007
sigma one state	0.063
sigma two state	0.063

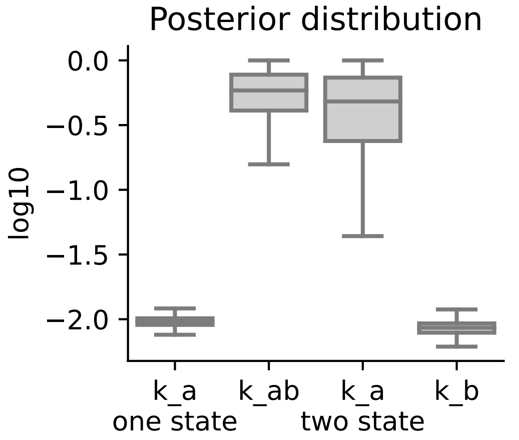

p value	2.61E-01
log likelihood one state	92.302
log likelihood two state	93.645
rmse one state	0.341
rmse two state	0.324
k_a one state	0.01
k_ab	0.586
k_a two state	0.482
k_b	0.009
sigma one state	0.057
sigma two state	0.056

uS15m
MRPS15
P82914
28S ribosomal protein S15, mitochondrial

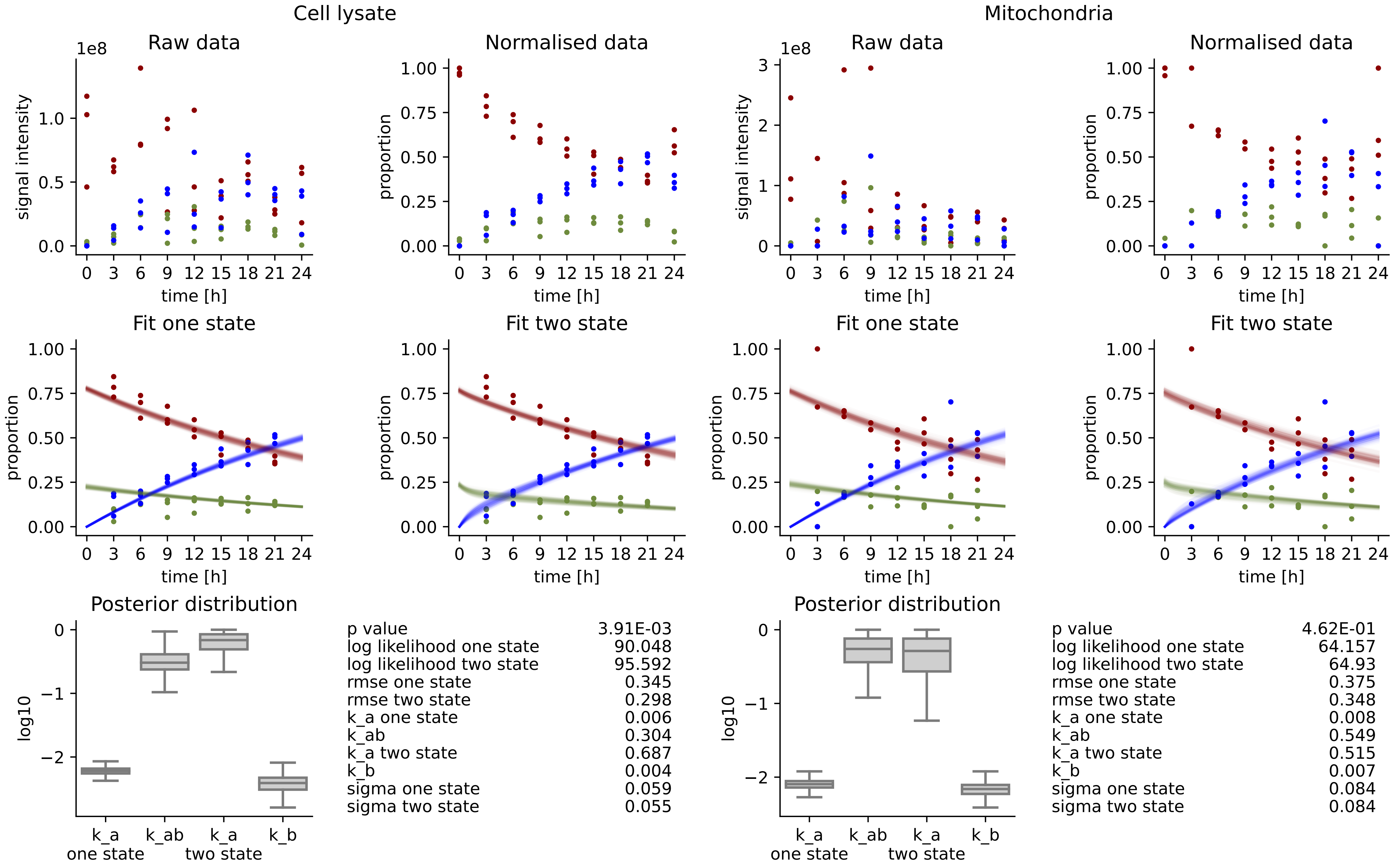

bS16m
MRPS16
Q9Y3D3
28S ribosomal protein S16, mitochondrial

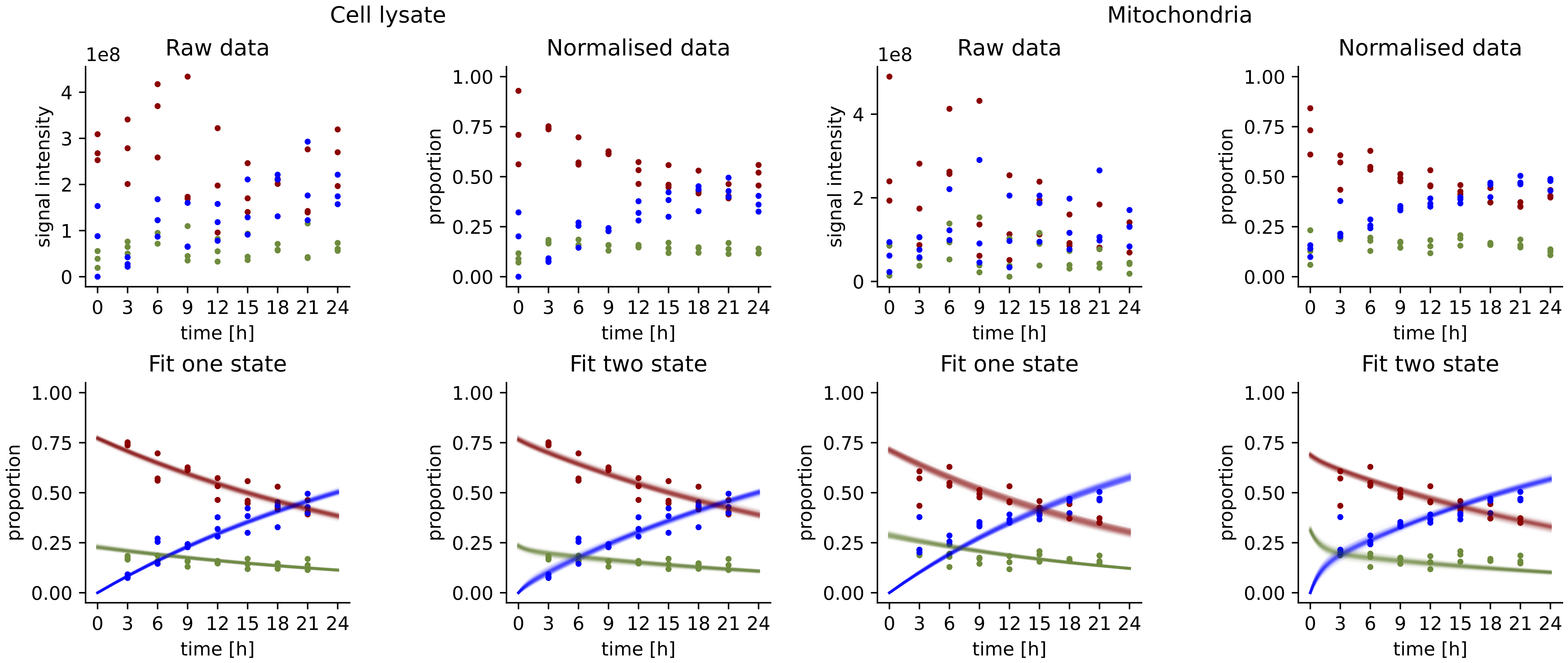

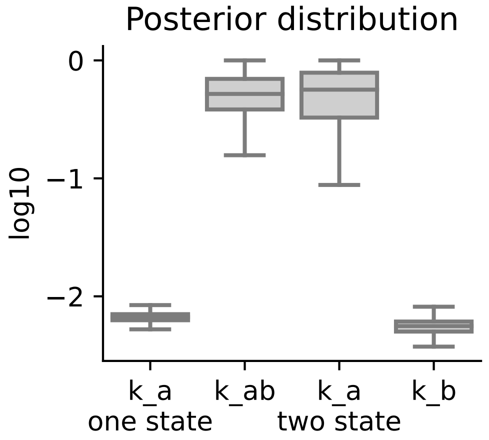

p value	7.59E-02
log likelihood one state	109.624
log likelihood two state	112.202
rmse one state	0.318
rmse two state	0.295
k_a one state	0.007
k_ab	0.52
k_a two state	0.565
k_b	0.006
sigma one state	0.043
sigma two state	0.042

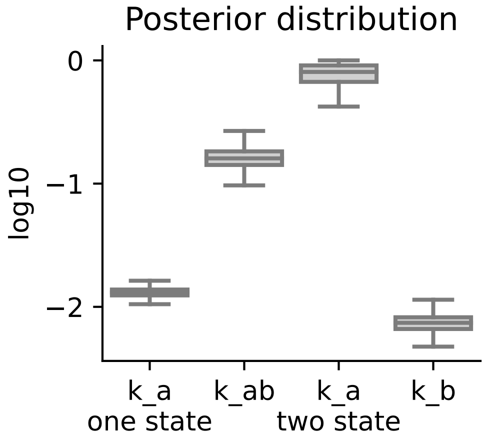

p value	4.49E-09
log likelihood one state	83.437
log likelihood two state	102.658
rmse one state	0.348
rmse two state	0.25
k_a one state	0.013
k_ab	0.16
k_a two state	0.806
k_b	0.007
sigma one state	0.066
sigma two state	0.049

uS17m
MRPS17
Q9Y2R5
28S ribosomal protein S17, mitochondrial

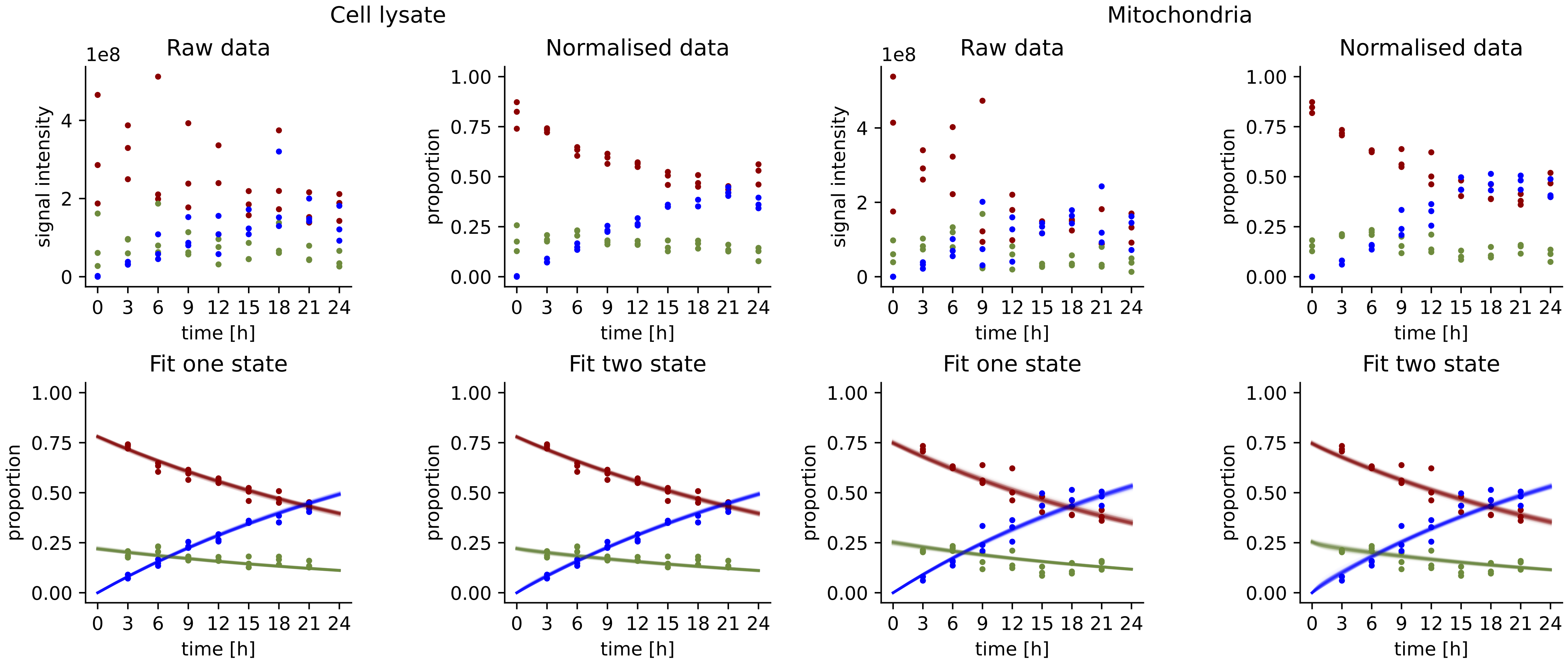

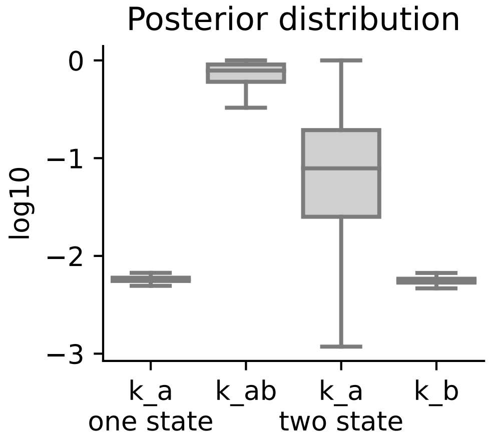

p value	1.80E-01
log likelihood one state	145.692
log likelihood two state	147.405
rmse one state	0.289
rmse two state	0.292
k_a one state	0.006
k_ab	0.787
k_a two state	0.079
k_b	0.006
sigma one state	0.024
sigma two state	0.025

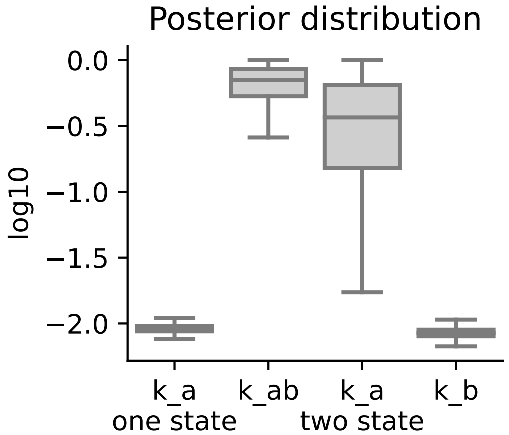

p value	4.83E-01
log likelihood one state	109.926
log likelihood two state	110.654
rmse one state	0.344
rmse two state	0.337
k_a one state	0.009
k_ab	0.708
k_a two state	0.367
k_b	0.008
sigma one state	0.043
sigma two state	0.043

bS18m
MRPS18C
Q9Y3D5
28S ribosomal protein S18c, mitochondrial

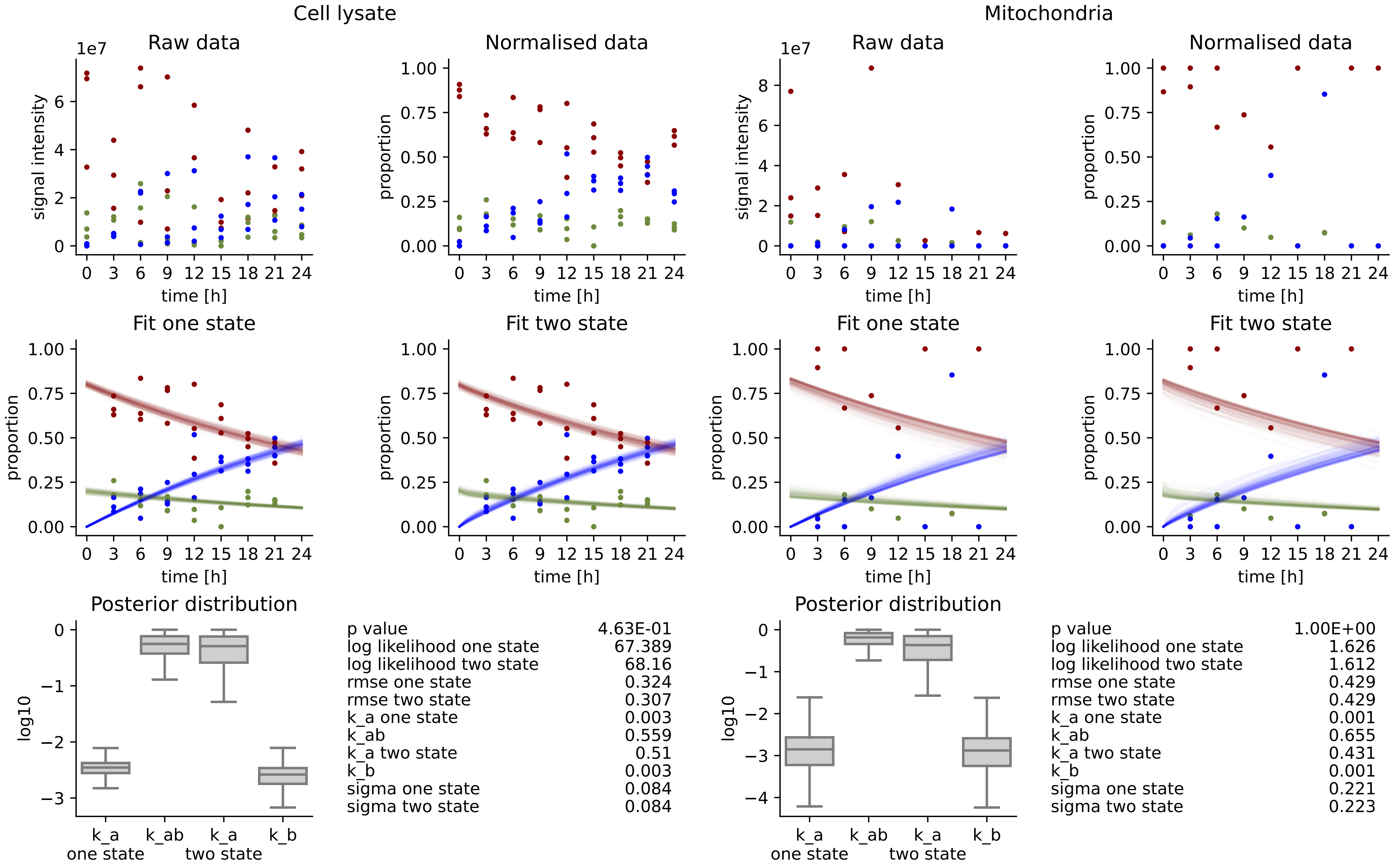

bS21m
MRPS21
P82921
28S ribosomal protein S21, mitochondrial

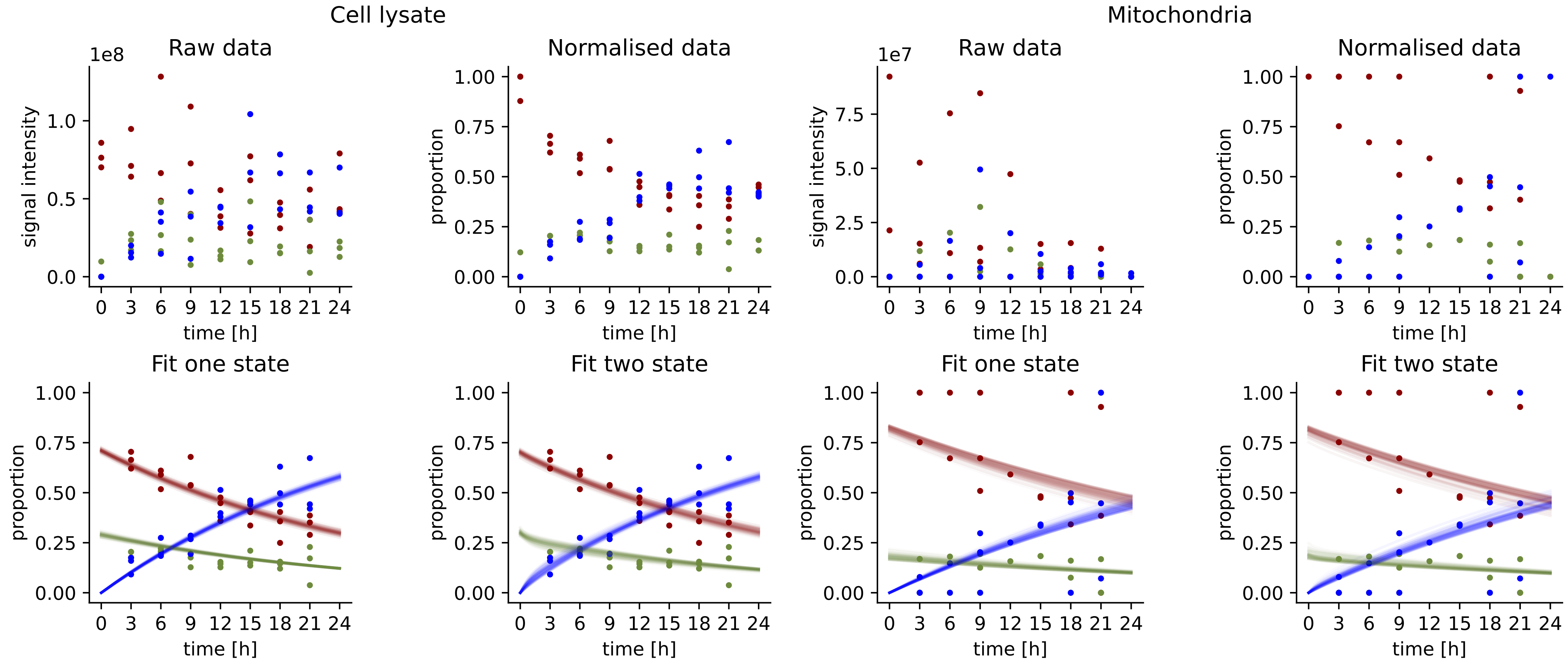

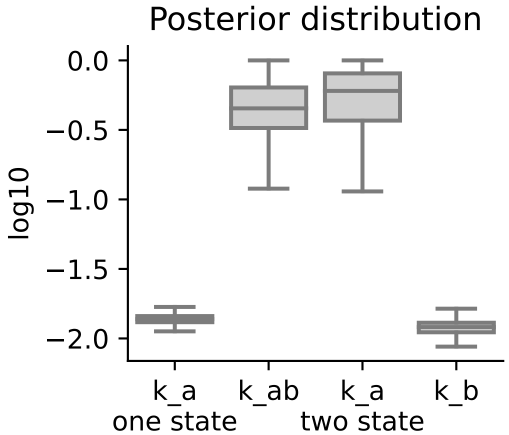

p value	6.58E-02
log likelihood one state	84.694
log likelihood two state	87.415
rmse one state	0.381
rmse two state	0.342
k_a one state	0.014
k_ab	0.452
k_a two state	0.603
k_b	0.012
sigma one state	0.064
sigma two state	0.062

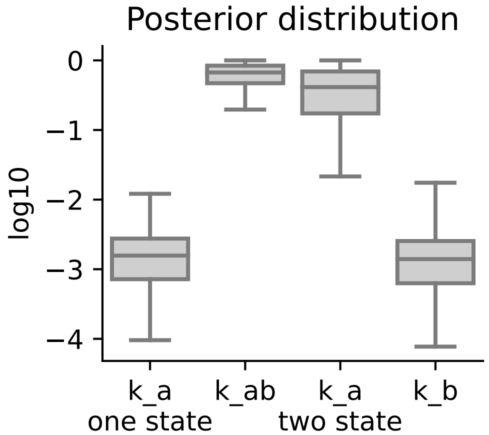

p value	1.00E+00
log likelihood one state	11.559
log likelihood two state	11.552
rmse one state	0.451
rmse two state	0.45
k_a one state	0.002
k_ab	0.67
k_a two state	0.413
k_b	0.001
sigma one state	0.191
sigma two state	0.192

mS22
MRPS22
P82650
28S ribosomal protein S22, mitochondrial

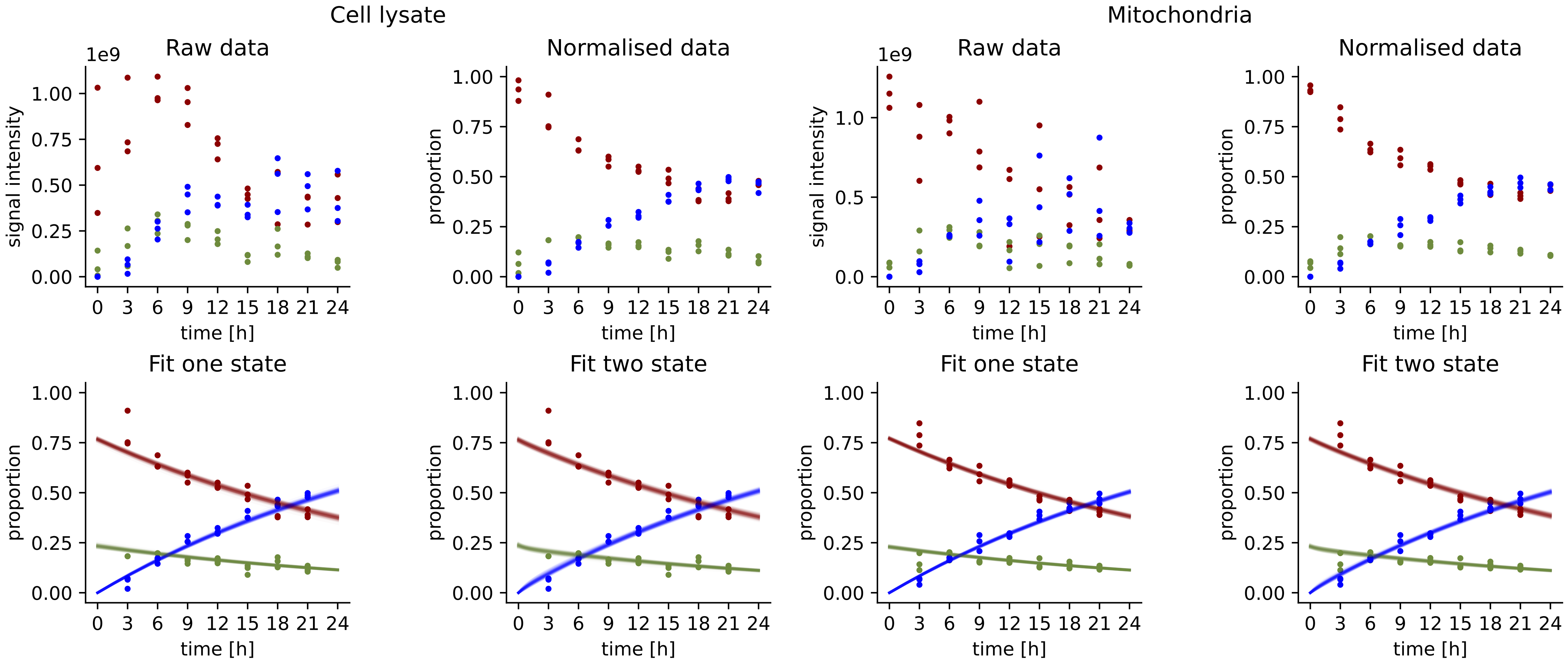

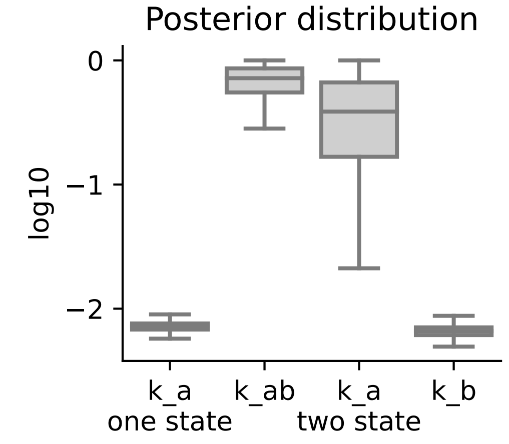

p value	8.95E-01
log likelihood one state	108.94
log likelihood two state	109.051
rmse one state	0.338
rmse two state	0.334
k_a one state	0.007
k_ab	0.719
k_a two state	0.387
k_b	0.007
sigma one state	0.044
sigma two state	0.044

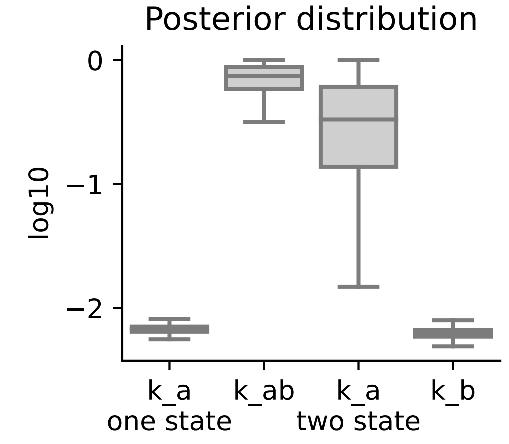

p value	9.19E-01
log likelihood one state	123.734
log likelihood two state	123.818
rmse one state	0.326
rmse two state	0.322
k_a one state	0.007
k_ab	0.748
k_a two state	0.332
k_b	0.006
sigma one state	0.035
sigma two state	0.035

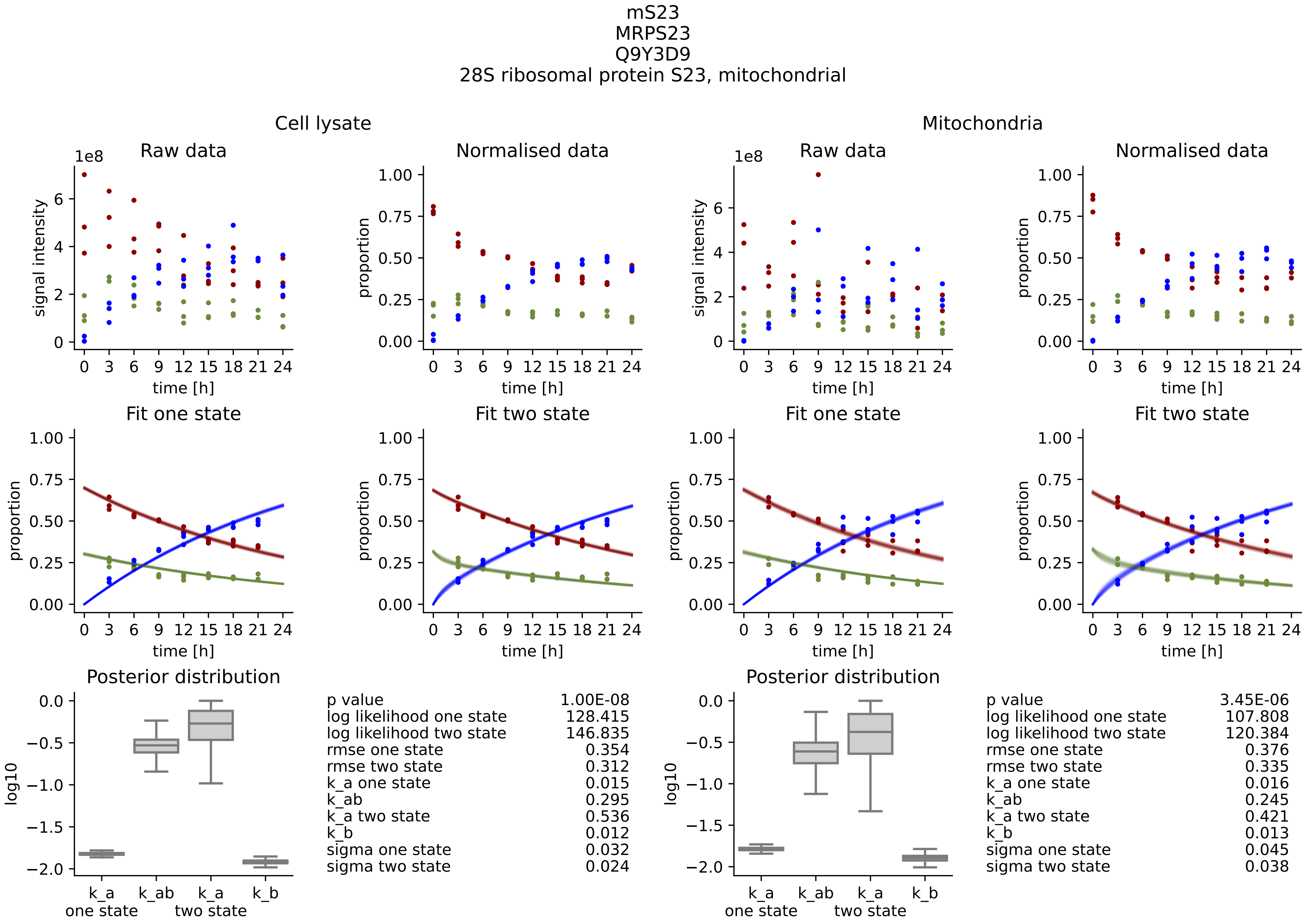
mS23
MRPS23
Q9Y3D9
28S ribosomal protein S23, mitochondrial
Cell lysate
Mitochondria
Raw data
Normalised data
Fit one state
Fit two state
Posterior distribution
1e8
signal intensity
proportion
time [h]
log10
k_a one state
k_ab
k_a two state
k_b
p value 1.00E-08
log likelihood one state 128.415
log likelihood two state 146.835
rmse one state 0.354
rmse two state 0.312
k_a one state 0.015
k_ab 0.295
k_a two state 0.536
k_b 0.012
sigma one state 0.032
sigma two state 0.024
p value 3.45E-06
log likelihood one state 107.808
log likelihood two state 120.384
rmse one state 0.376
rmse two state 0.335
k_a one state 0.016
k_ab 0.245
k_a two state 0.421
k_b 0.013
sigma one state 0.045
sigma two state 0.038

mS25
MRPS25
P82663
28S ribosomal protein S25, mitochondrial

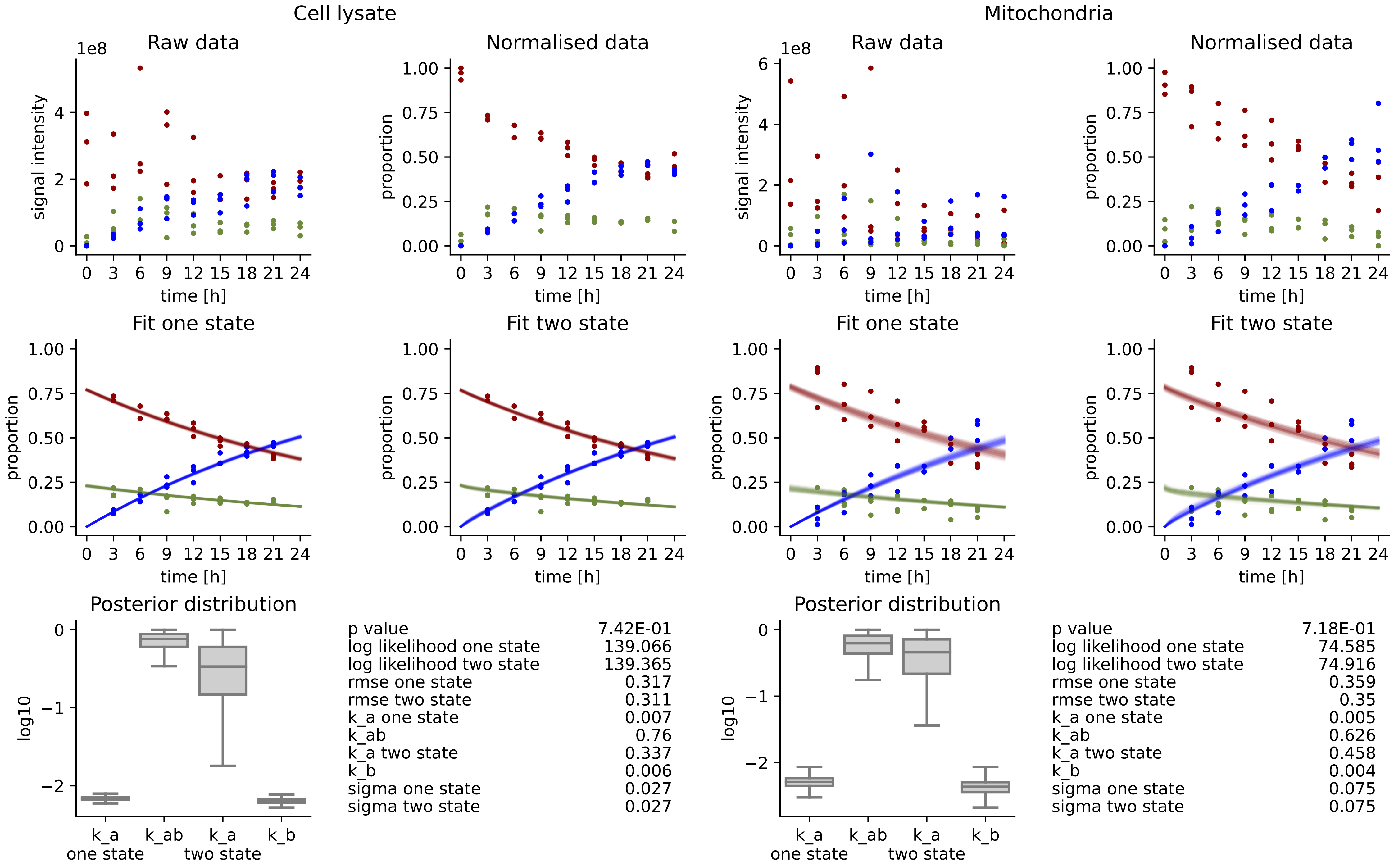

mS26
MRPS26
Q9BYN8
28S ribosomal protein S26, mitochondrial

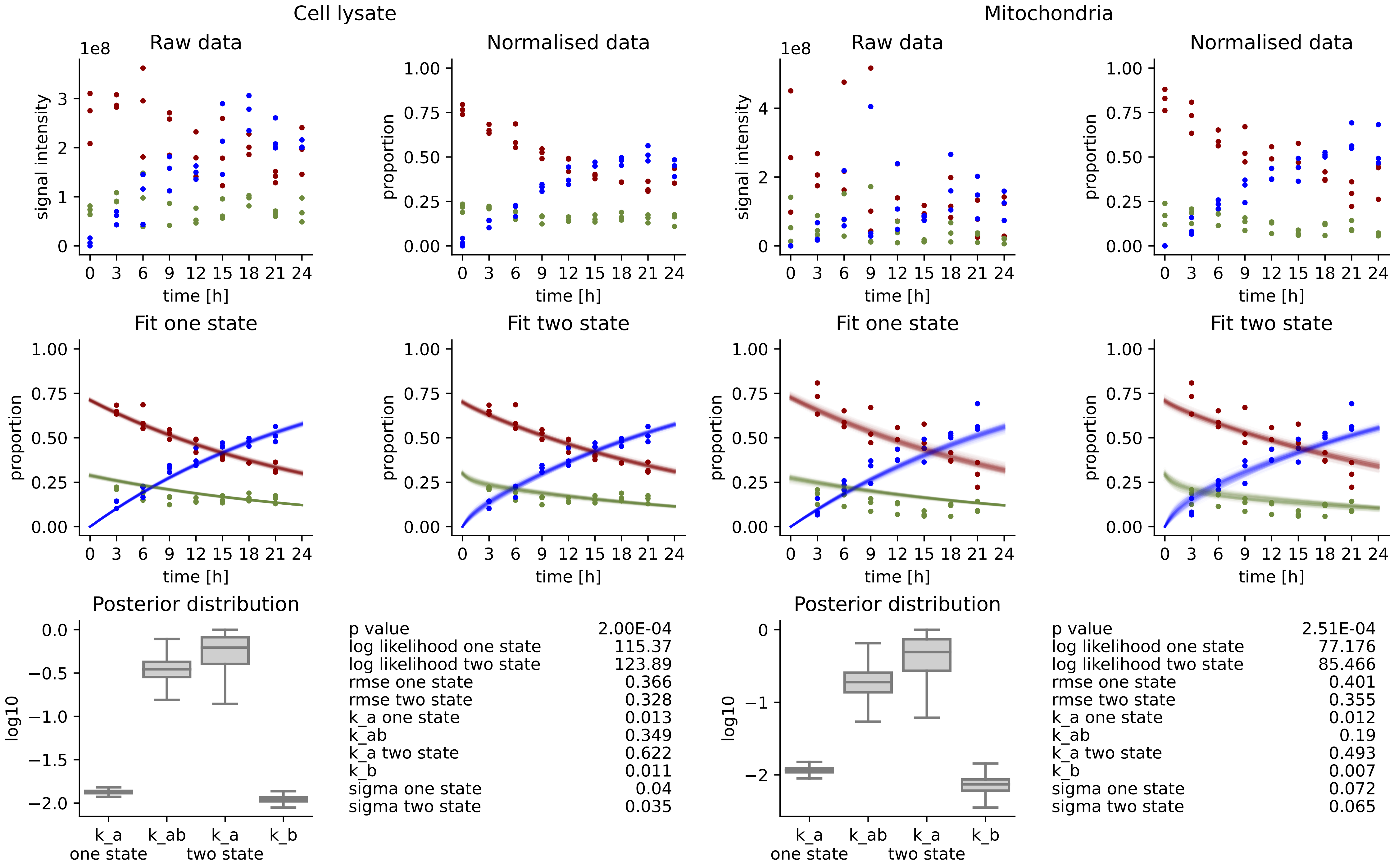

mS27
MRPS27
Q92552
28S ribosomal protein S27, mitochondrial

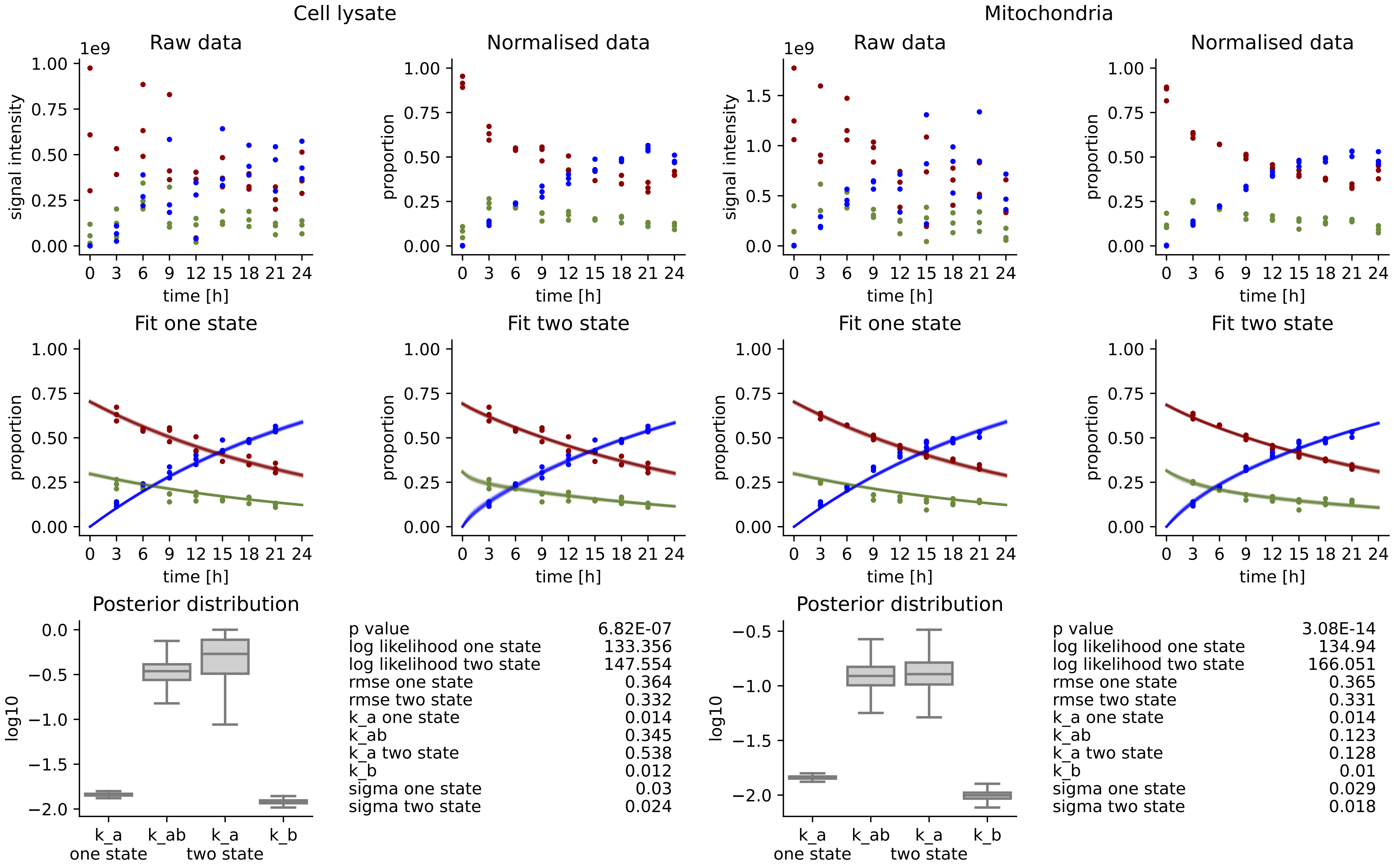

Cell lysate	
p value	6.82E-07
log likelihood one state	133.356
log likelihood two state	147.554
rmse one state	0.364
rmse two state	0.332
k_a one state	0.014
k_ab	0.345
k_a two state	0.538
k_b	0.012
sigma one state	0.03
sigma two state	0.024

Mitochondria	
p value	3.08E-14
log likelihood one state	134.94
log likelihood two state	166.051
rmse one state	0.365
rmse two state	0.331
k_a one state	0.014
k_ab	0.123
k_a two state	0.128
k_b	0.01
sigma one state	0.029
sigma two state	0.018

mS29
DAP3
P51398
28S ribosomal protein S29, mitochondrial

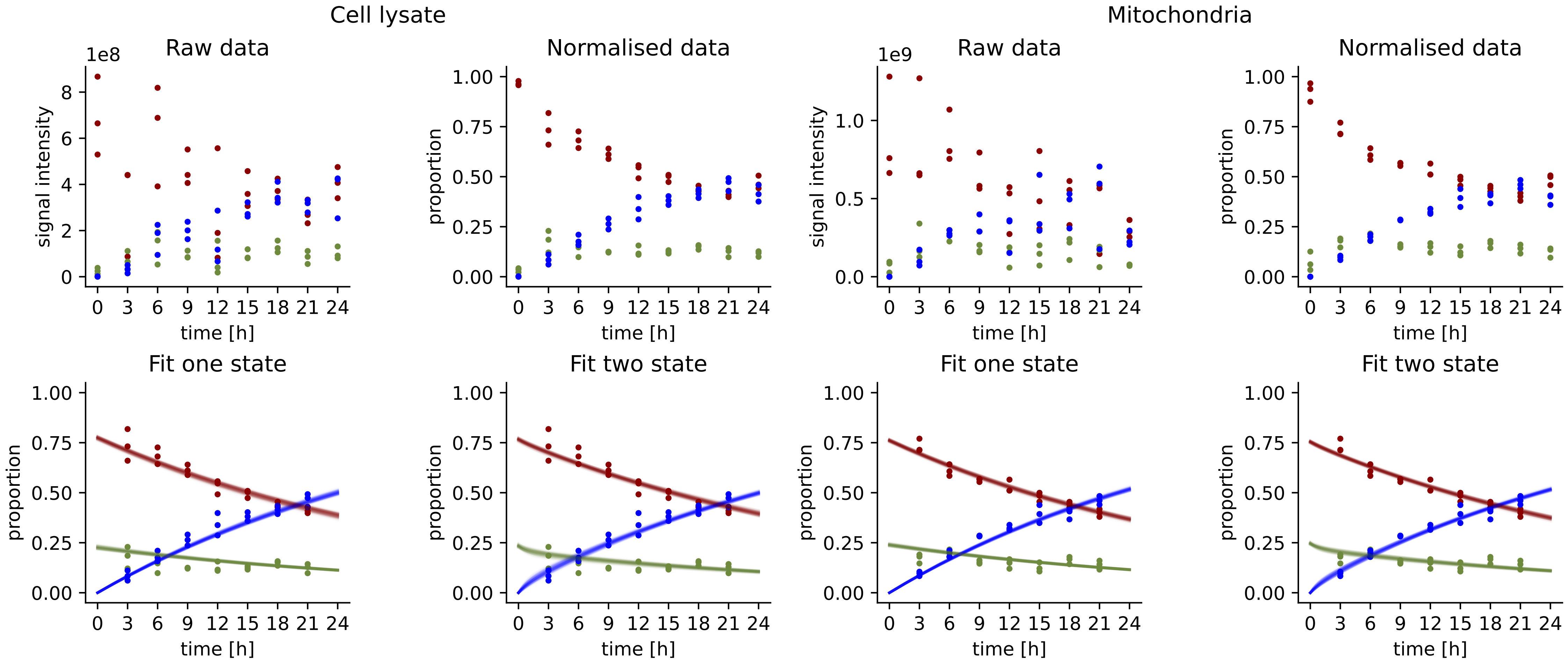

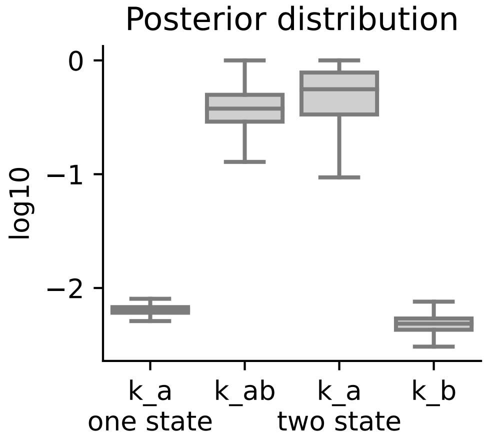

p value	3.21E-03
log likelihood one state	115.19
log likelihood two state	120.931
rmse one state	0.329
rmse two state	0.303
k_a one state	0.006
k_ab	0.377
k_a two state	0.558
k_b	0.005
sigma one state	0.04
sigma two state	0.037

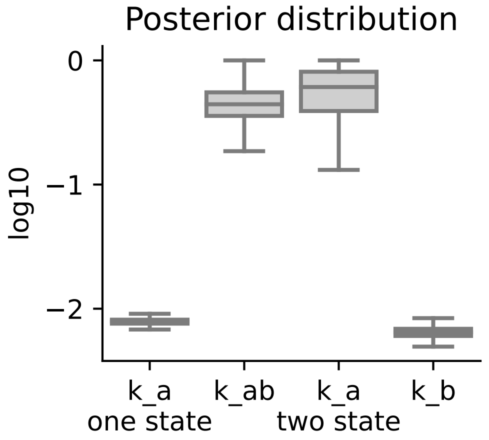

p value	1.68E-03
log likelihood one state	131.244
log likelihood two state	137.632
rmse one state	0.322
rmse two state	0.296
k_a one state	0.008
k_ab	0.443
k_a two state	0.61
k_b	0.006
sigma one state	0.031
sigma two state	0.028

mS31
MRPS31
Q92665
28S ribosomal protein S31, mitochondrial

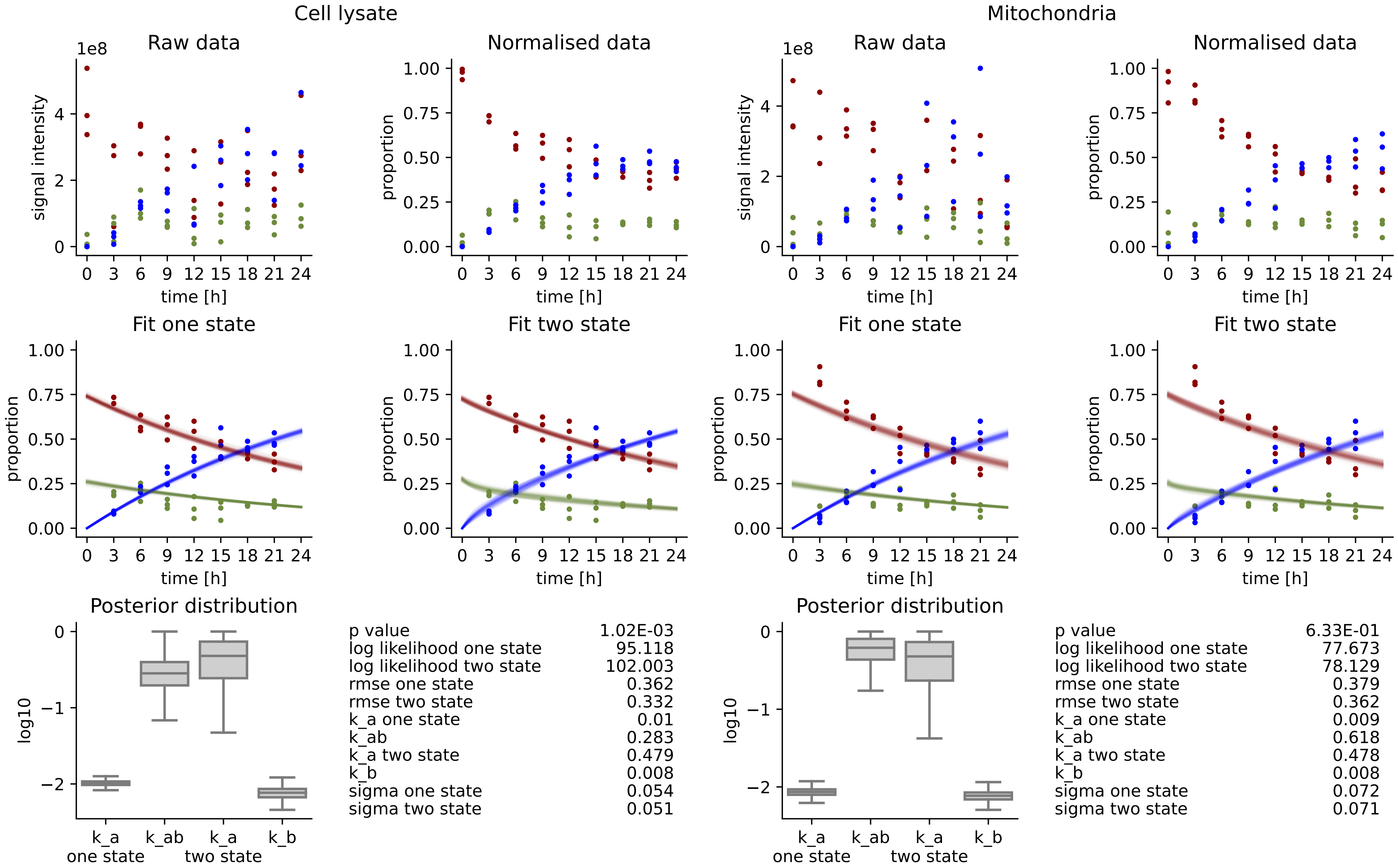

mS33
MRPS33
Q9Y291
28S ribosomal protein S33, mitochondrial

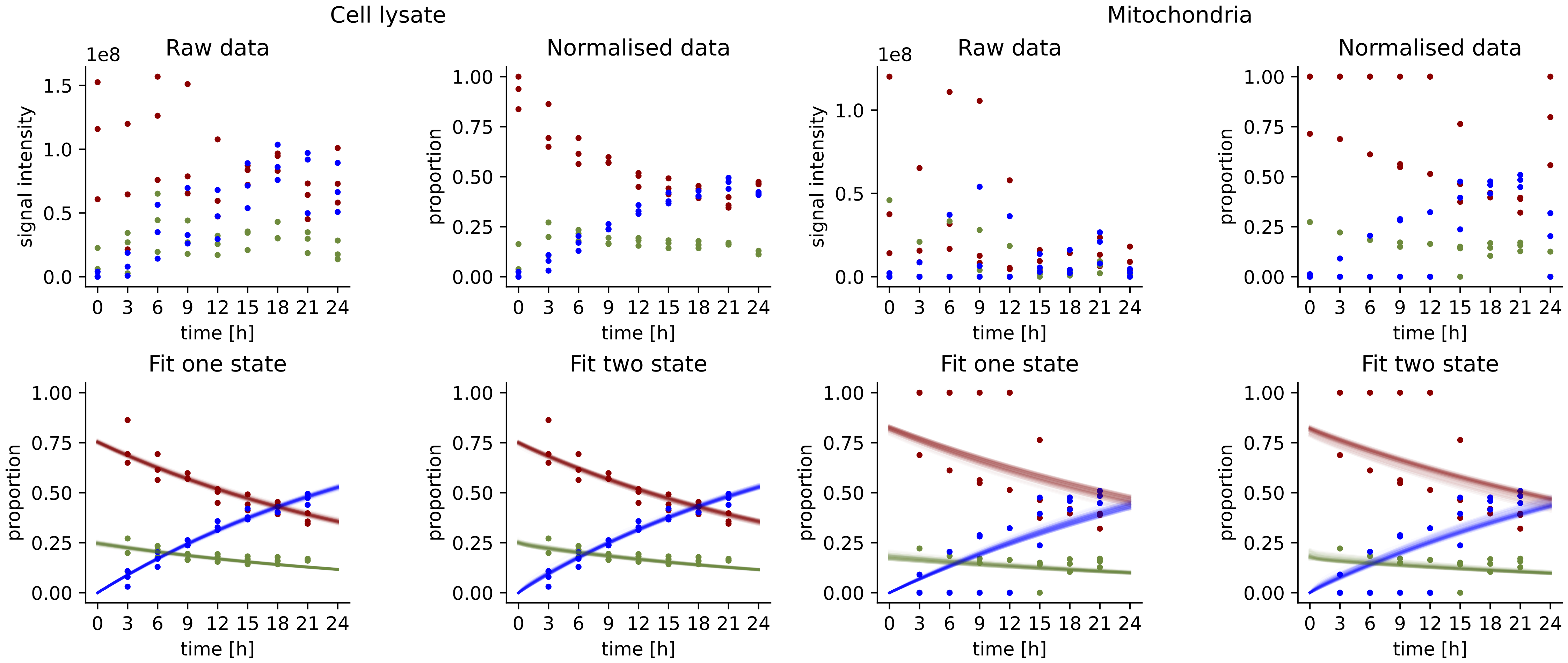

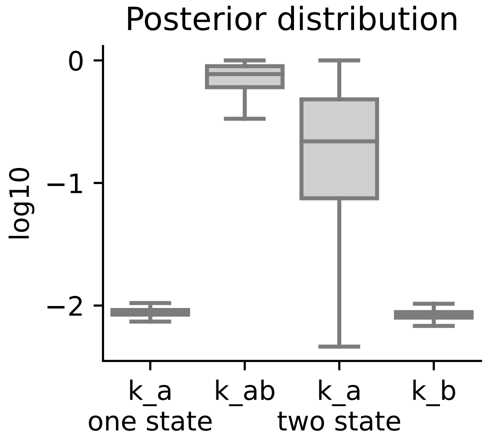

p value	4.23E-01
log likelihood one state	113.822
log likelihood two state	114.682
rmse one state	0.327
rmse two state	0.331
k_a one state	0.009
k_ab	0.773
k_a two state	0.219
k_b	0.008
sigma one state	0.041
sigma two state	0.041

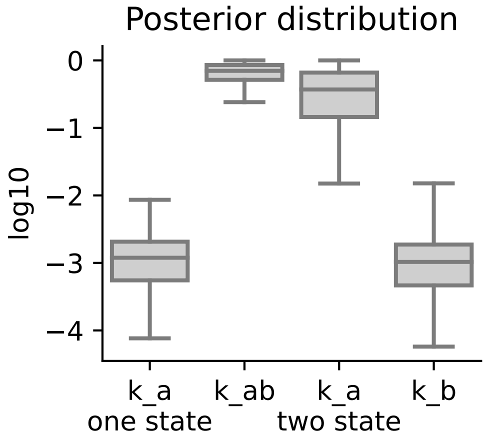

p value	1.00E+00
log likelihood one state	29.812
log likelihood two state	29.811
rmse one state	0.404
rmse two state	0.404
k_a one state	0.001
k_ab	0.7
k_a two state	0.371
k_b	0.001
sigma one state	0.151
sigma two state	0.152

mS34
MRPS34
P82930
28S ribosomal protein S34, mitochondrial

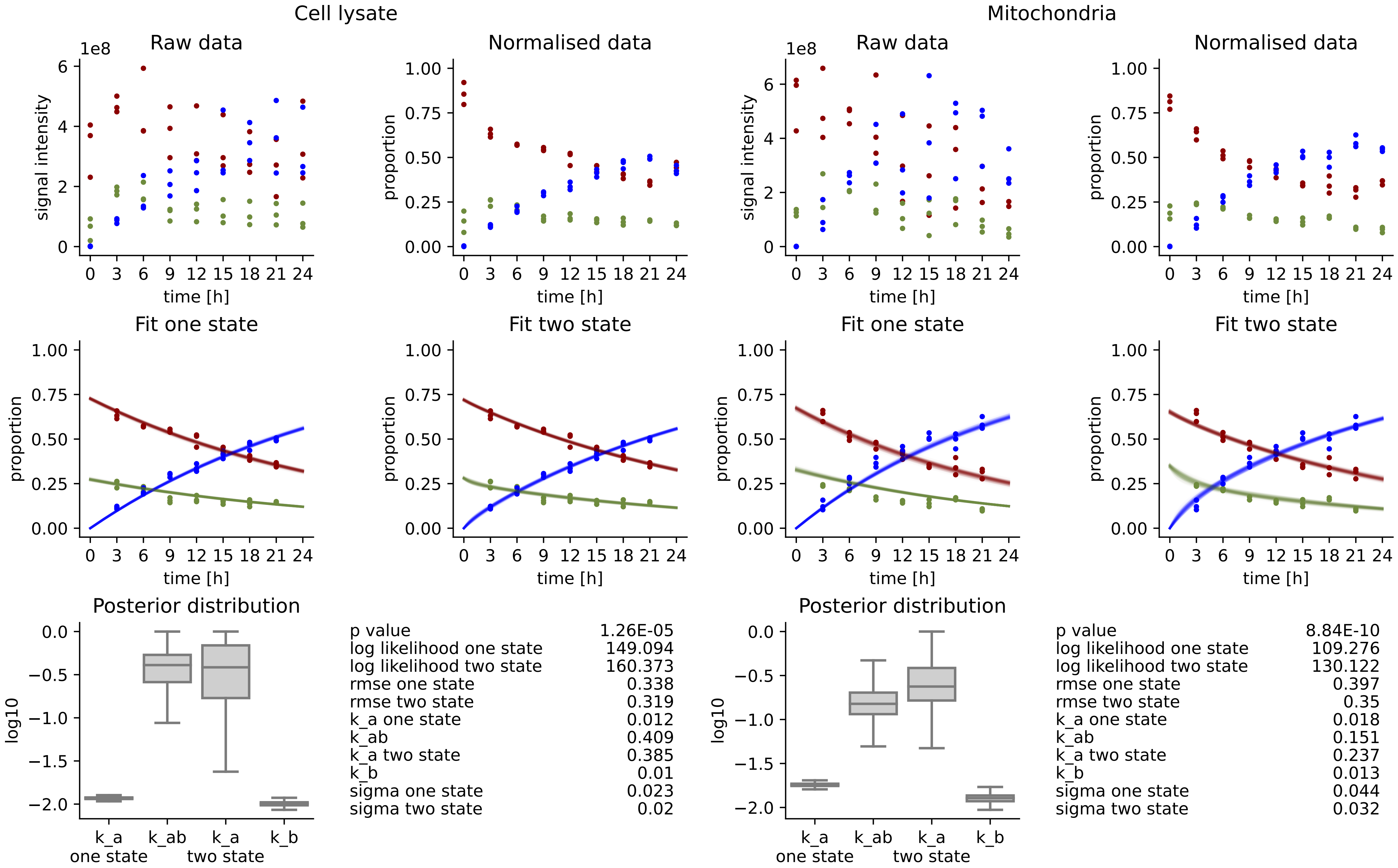

Cell lysate

p value	1.26E-05
log likelihood one state	149.094
log likelihood two state	160.373
rmse one state	0.338
rmse two state	0.319
k_a one state	0.012
k_ab	0.409
k_a two state	0.385
k_b	0.01
sigma one state	0.023
sigma two state	0.02

Mitochondria

p value	8.84E-10
log likelihood one state	109.276
log likelihood two state	130.122
rmse one state	0.397
rmse two state	0.35
k_a one state	0.018
k_ab	0.151
k_a two state	0.237
k_b	0.013
sigma one state	0.044
sigma two state	0.032

mS35
MRPS35
P82673
28S ribosomal protein S35, mitochondrial

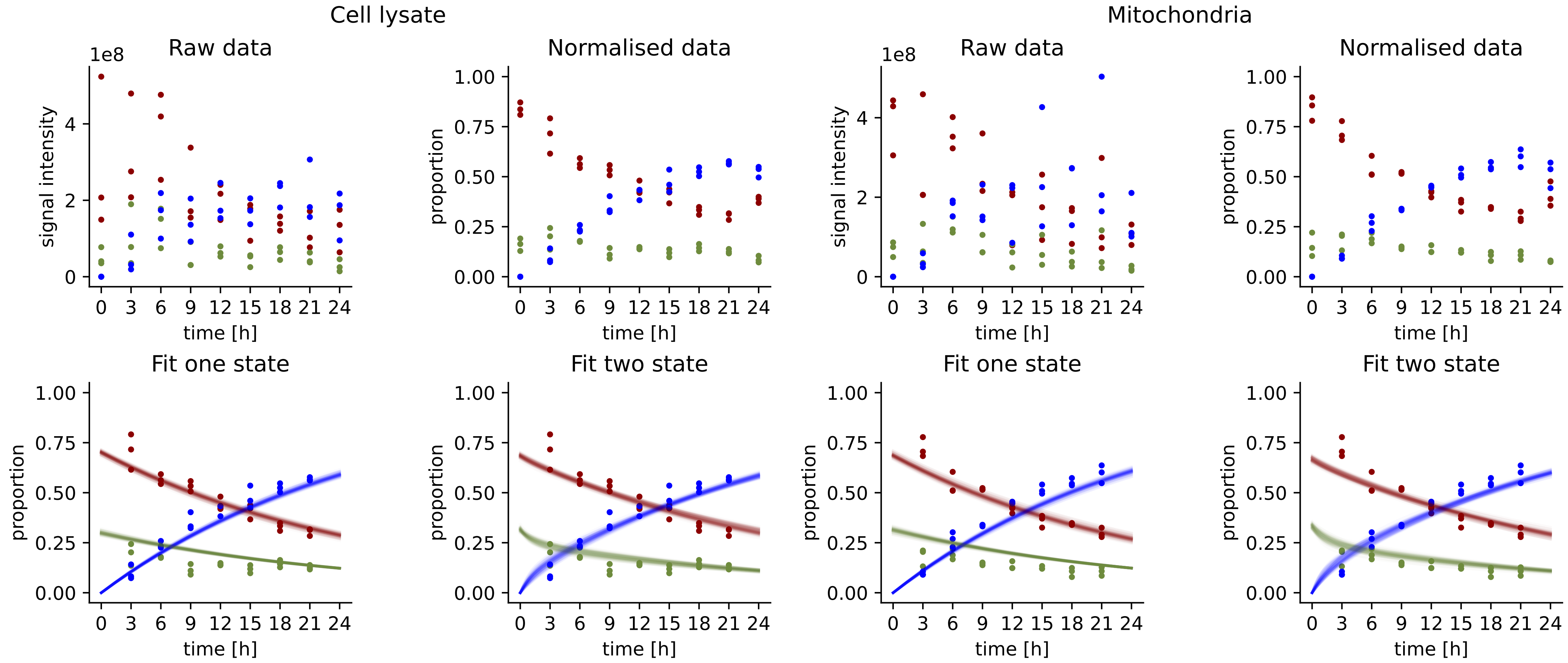

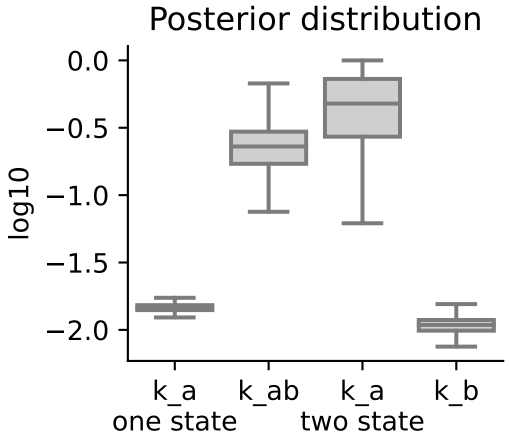

p value	6.00E-05
log likelihood one state	93.926
log likelihood two state	103.648
rmse one state	0.4
rmse two state	0.353
k_a one state	0.015
k_ab	0.23
k_a two state	0.478
k_b	0.011
sigma one state	0.056
sigma two state	0.049

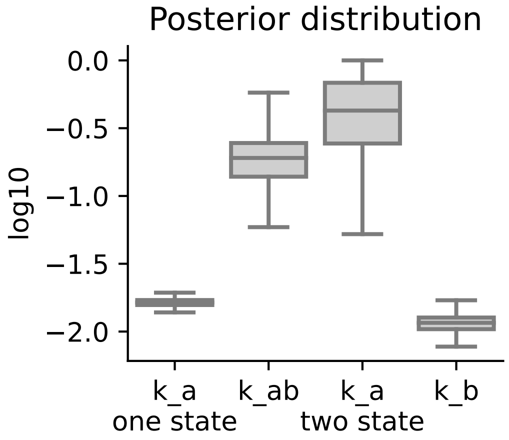

p value	9.79E-06
log likelihood one state	88.768
log likelihood two state	100.302
rmse one state	0.42
rmse two state	0.371
k_a one state	0.016
k_ab	0.191
k_a two state	0.426
k_b	0.012
sigma one state	0.06
sigma two state	0.052

mS37
CHCHD1
Q96BP2
Coiled-coil-helix-coiled-coil-helix domain-containing protein 1

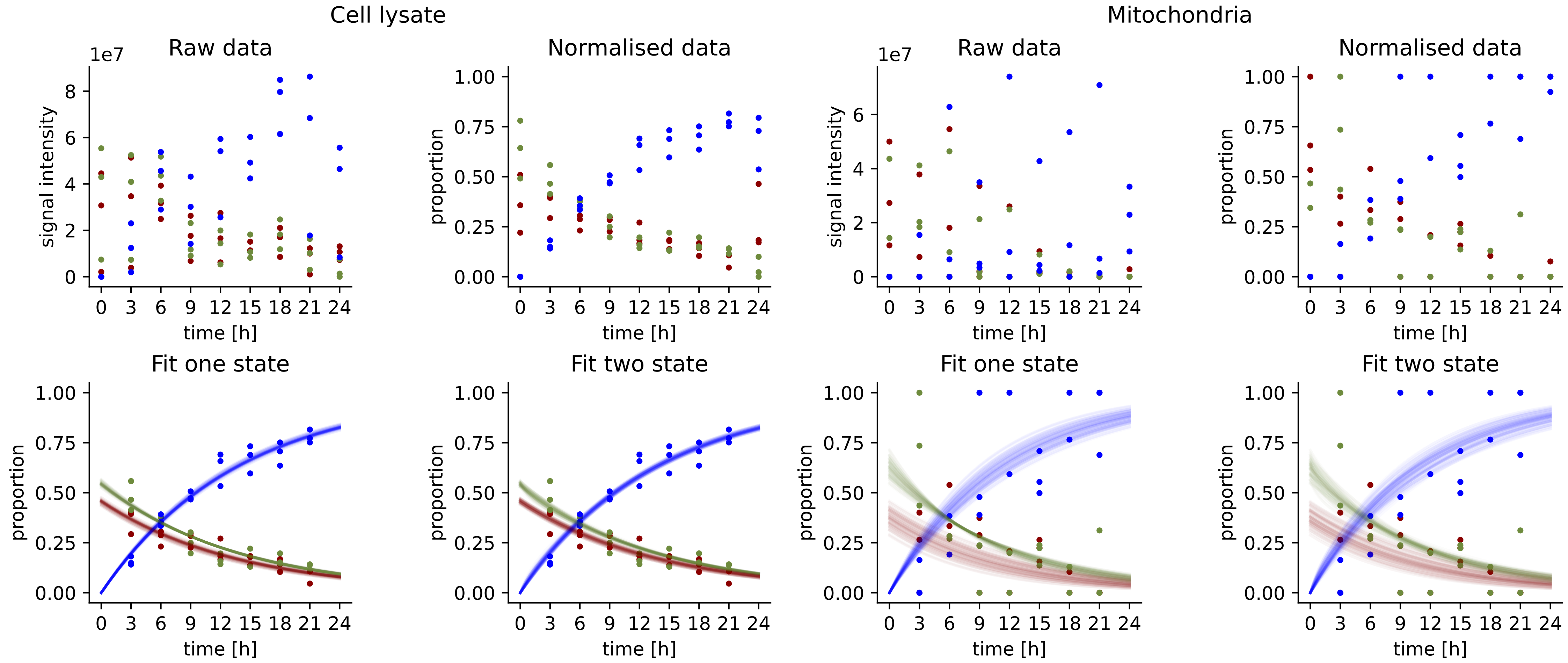

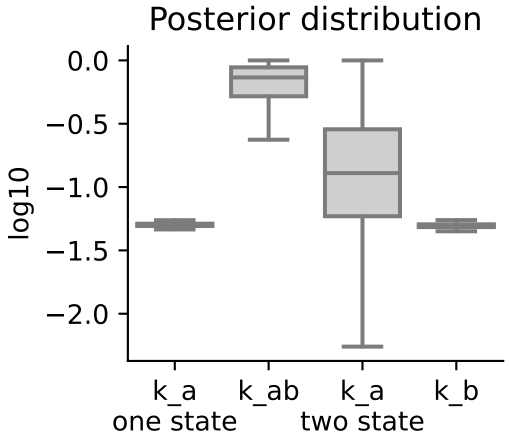

p value	7.49E-01
log likelihood one state	104.999
log likelihood two state	105.288
rmse one state	0.495
rmse two state	0.487
k_a one state	0.05
k_ab	0.733
k_a two state	0.129
k_b	0.05
sigma one state	0.047
sigma two state	0.047

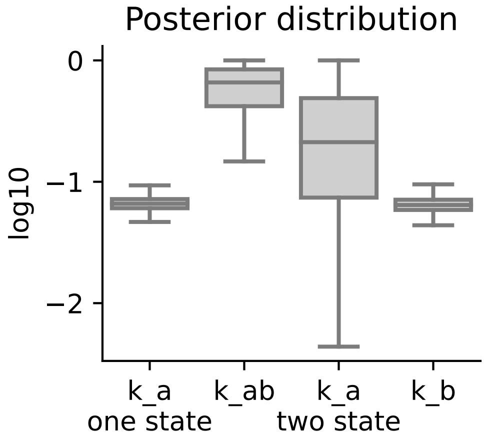

p value	7.55E-01
log likelihood one state	17.056
log likelihood two state	17.338
rmse one state	0.627
rmse two state	0.639
k_a one state	0.066
k_ab	0.658
k_a two state	0.212
k_b	0.064
sigma one state	0.176
sigma two state	0.177

mS39
PTCD3
Q96EY7
Pentatricopeptide repeat domain-containing protein 3, mitochondrial

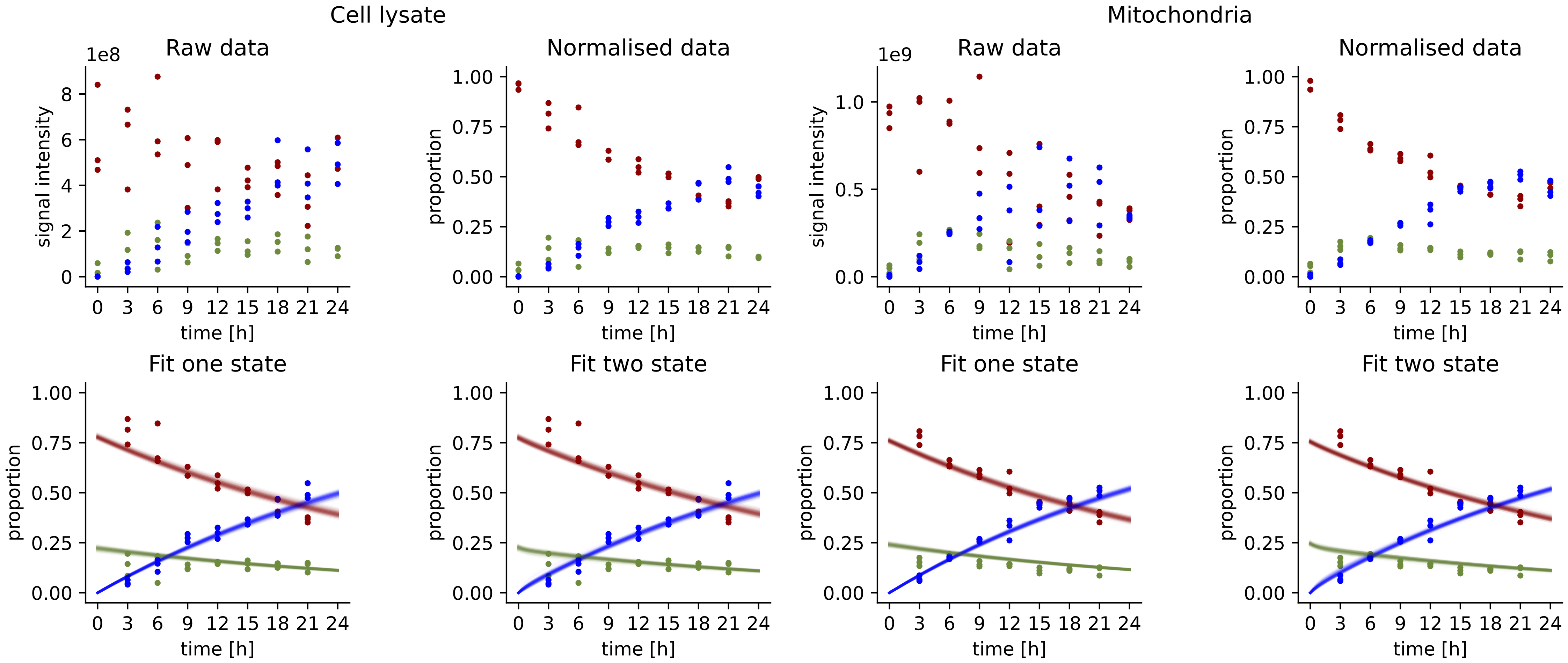

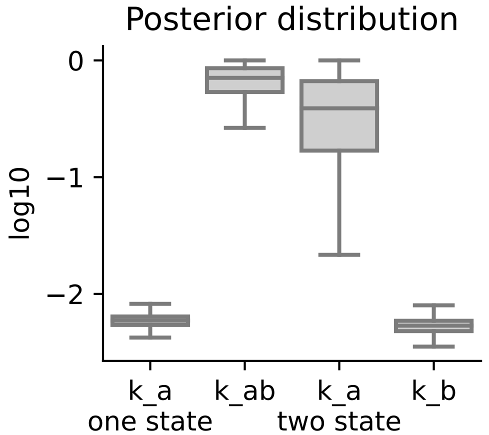

p value	9.67E-01
log likelihood one state	95.26
log likelihood two state	95.293
rmse one state	0.342
rmse two state	0.339
k_a one state	0.006
k_ab	0.708
k_a two state	0.389
k_b	0.005
sigma one state	0.054
sigma two state	0.055

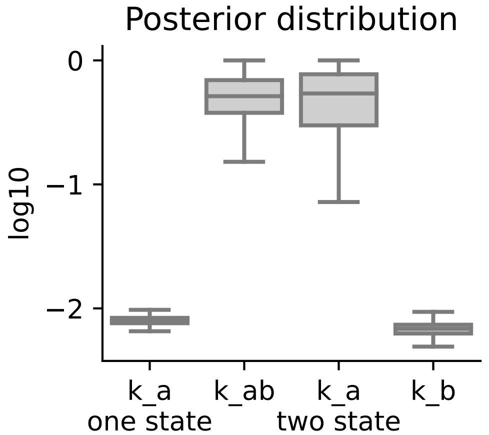

p value	4.20E-02
log likelihood one state	112.078
log likelihood two state	115.247
rmse one state	0.351
rmse two state	0.338
k_a one state	0.008
k_ab	0.514
k_a two state	0.541
k_b	0.007
sigma one state	0.042
sigma two state	0.04

mS40
MRPS18B
Q9Y676
28S ribosomal protein S18b, mitochondrial

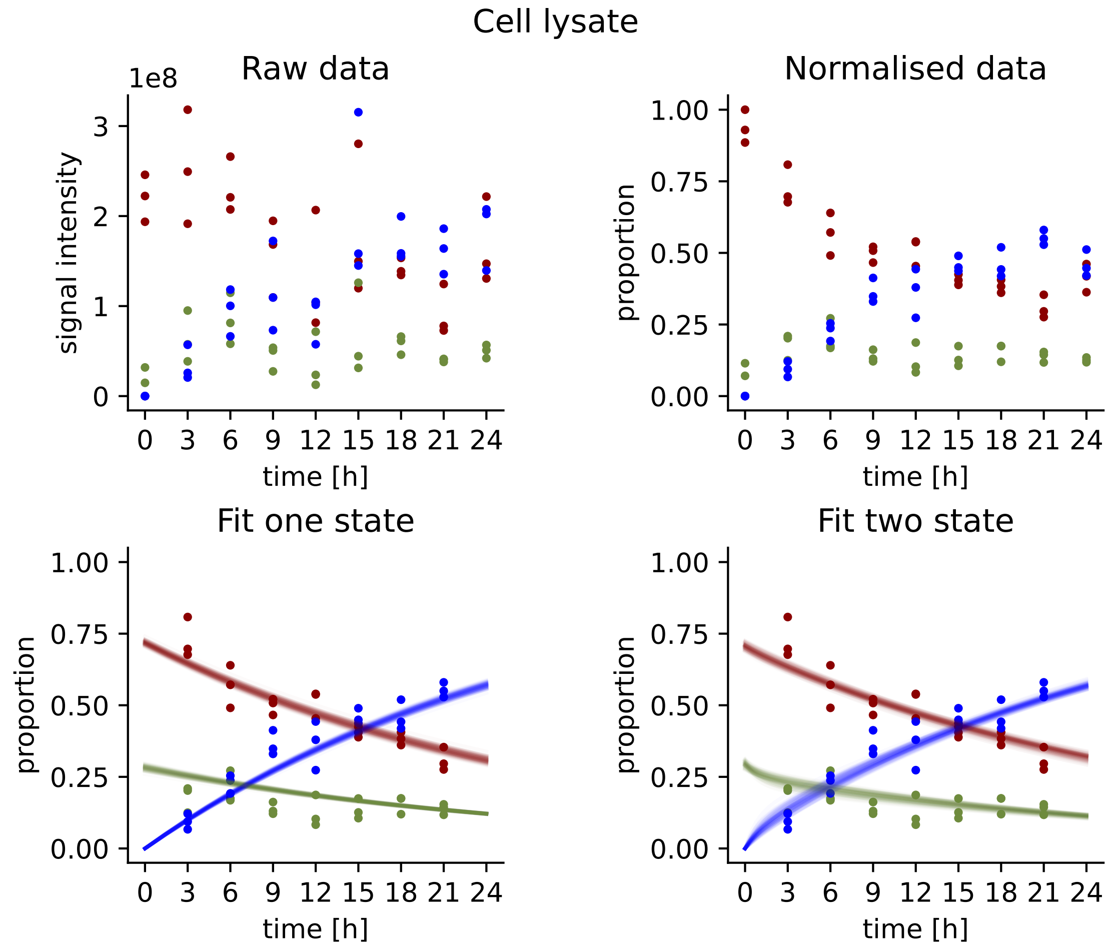

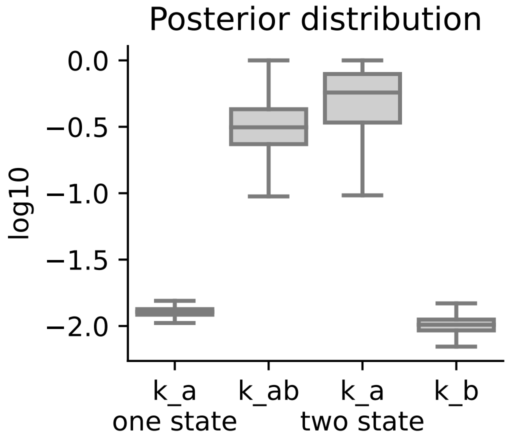

p value	4.00E-03
log likelihood one state	91.0
log likelihood two state	96.52
rmse one state	0.381
rmse two state	0.342
k_a one state	0.013
k_ab	0.313
k_a two state	0.573
k_b	0.01
sigma one state	0.058
sigma two state	0.054

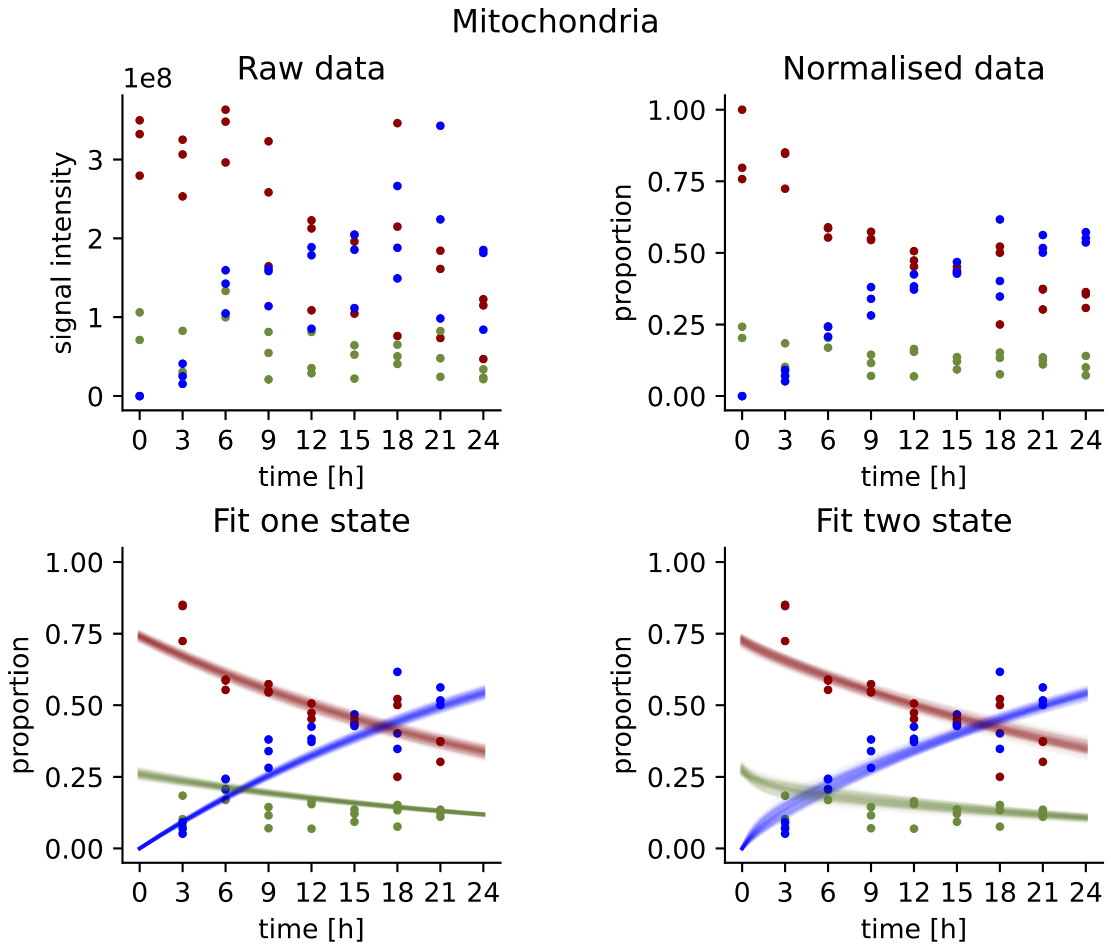

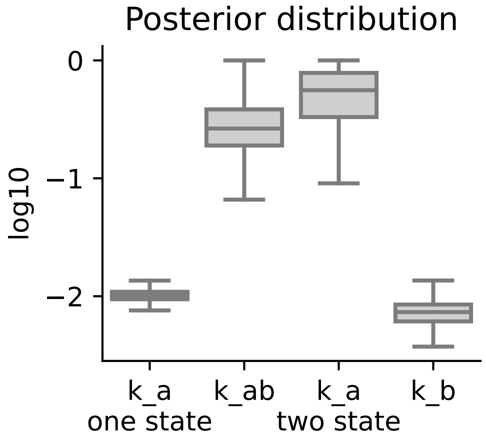

p value	6.50E-03
log likelihood one state	76.371
log likelihood two state	81.406
rmse one state	0.384
rmse two state	0.337
k_a one state	0.01
k_ab	0.264
k_a two state	0.559
k_b	0.007
sigma one state	0.073
sigma two state	0.069

TFB1M
TFB1M
Q8WVM0
Dimethyladenosine transferase 1, mitochondrial

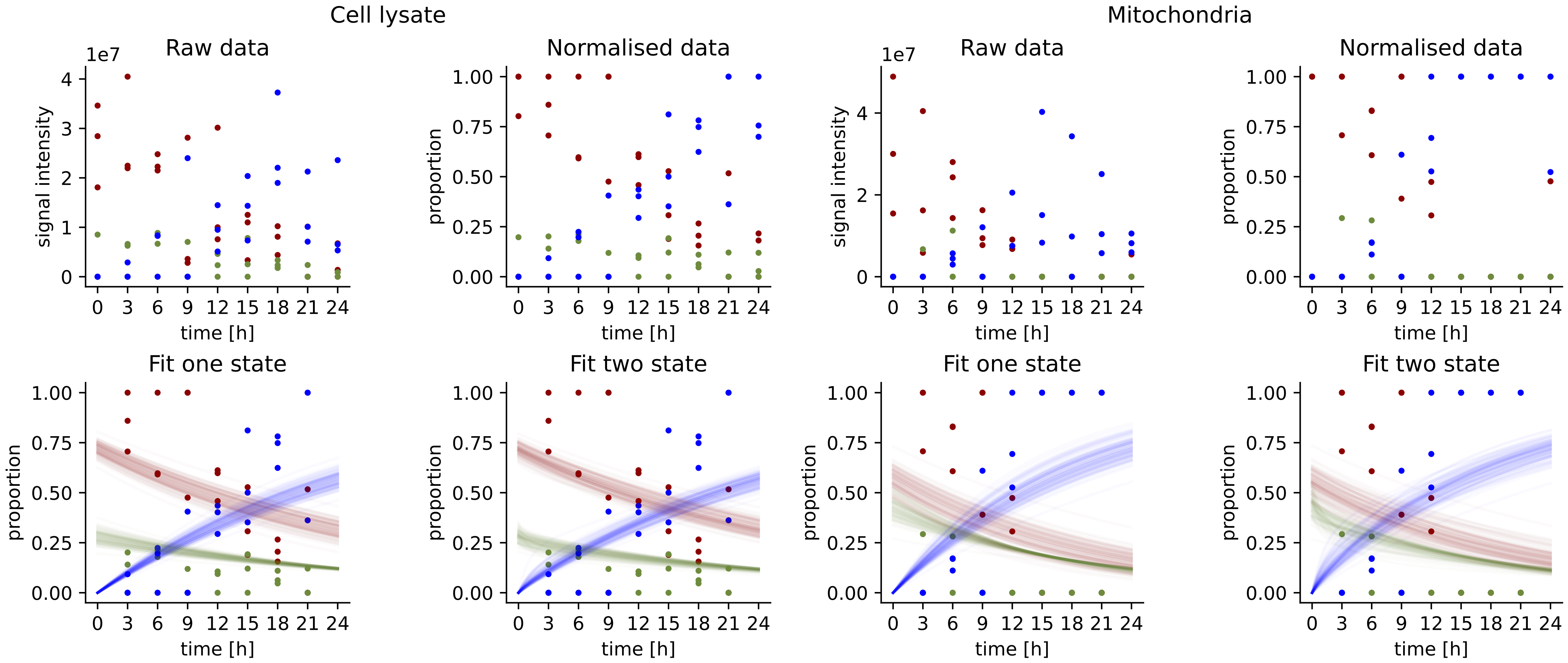

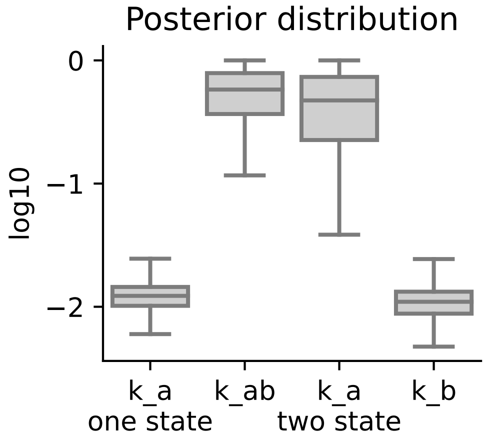

p value	8.88E-01
log likelihood one state	9.424
log likelihood two state	9.543
rmse one state	0.583
rmse two state	0.569
k_a one state	0.012
k_ab	0.58
k_a two state	0.473
k_b	0.011
sigma one state	0.206
sigma two state	0.206

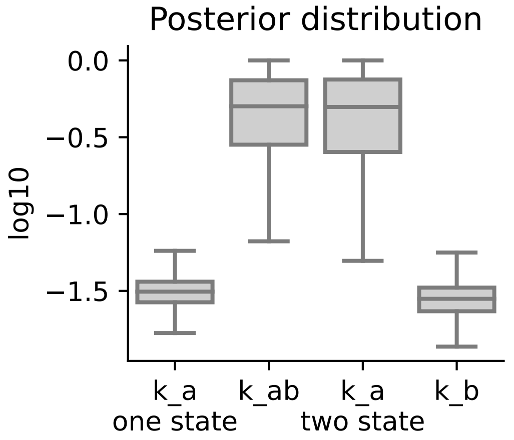

p value	5.71E-01
log likelihood one state	-12.435
log likelihood two state	-11.873
rmse one state	0.812
rmse two state	0.76
k_a one state	0.031
k_ab	0.503
k_a two state	0.497
k_b	0.028
sigma one state	0.285
sigma two state	0.284

METTL15
METTL15
A6NJ78
Probable methyltransferase-like protein 15

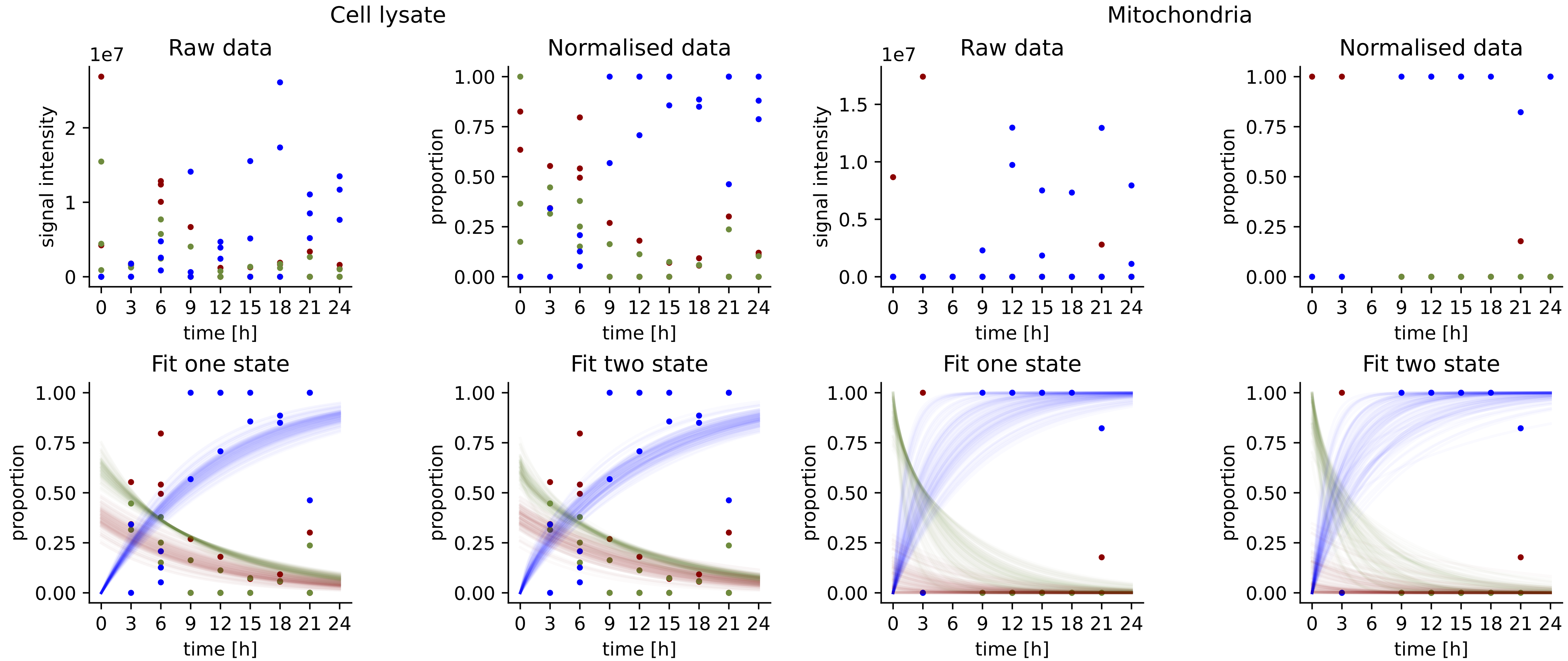

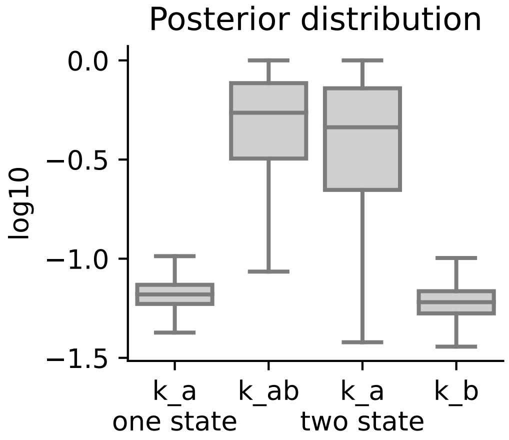

p value	3.10E-01
log likelihood one state	8.417
log likelihood two state	9.587
rmse one state	0.716
rmse two state	0.664
k_a one state	0.066
k_ab	0.544
k_a two state	0.46
k_b	0.06
sigma one state	0.202
sigma two state	0.202

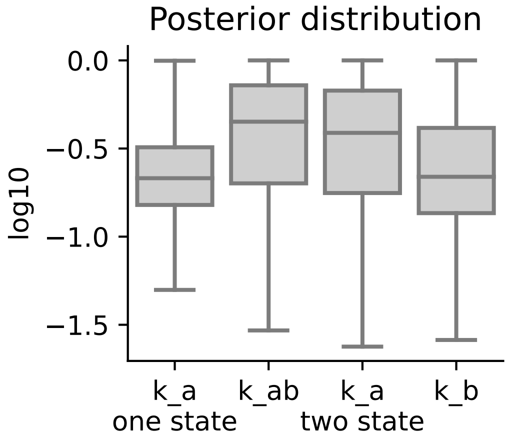

p value	3.33E-01
log likelihood one state	-0.632
log likelihood two state	0.468
rmse one state	0.83
rmse two state	0.674
k_a one state	0.215
k_ab	0.449
k_a two state	0.388
k_b	0.219
sigma one state	0.236
sigma two state	0.235

NSUN4
NSUN4
Q96CB9
5-methylcytosine rRNA methyltransferase NSUN4

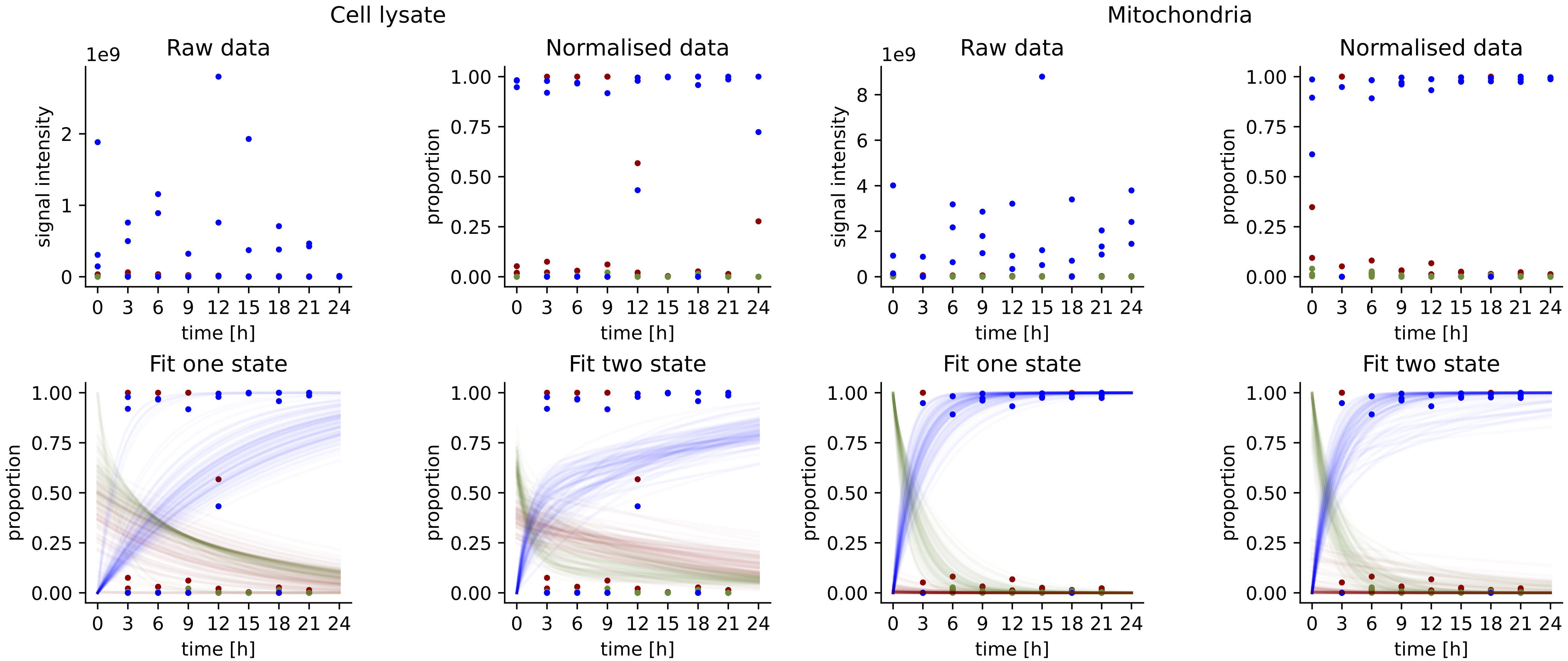

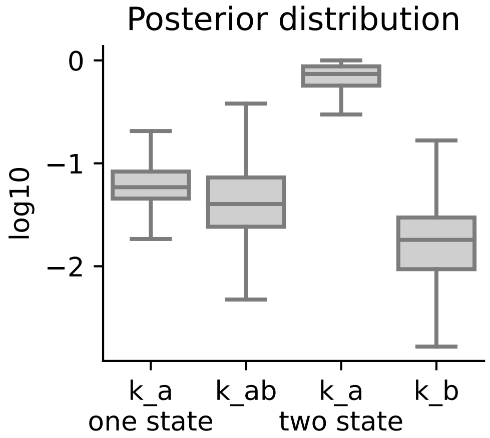

p value	5.05E-04
log likelihood one state	-28.233
log likelihood two state	-20.641
rmse one state	0.898
rmse two state	0.623
k_a one state	0.059
k_ab	0.04
k_a two state	0.738
k_b	0.018
sigma one state	0.367
sigma two state	0.334

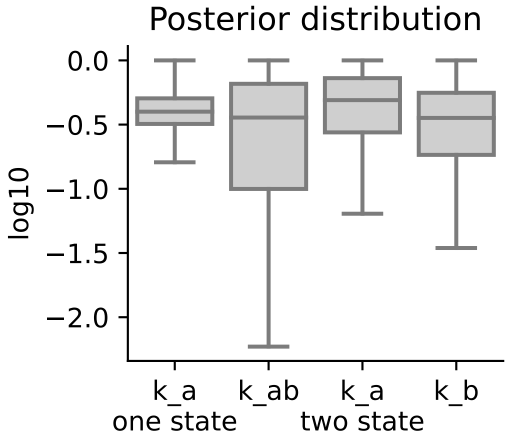

p value	5.98E-03
log likelihood one state	-11.687
log likelihood two state	-6.567
rmse one state	0.621
rmse two state	0.542
k_a one state	0.399
k_ab	0.359
k_a two state	0.49
k_b	0.356
sigma one state	0.28
sigma two state	0.277

TRMT2B
TRMT2B
Q96GJ1
tRNA (uracil(54)-C(5))-methyltransferase homolog

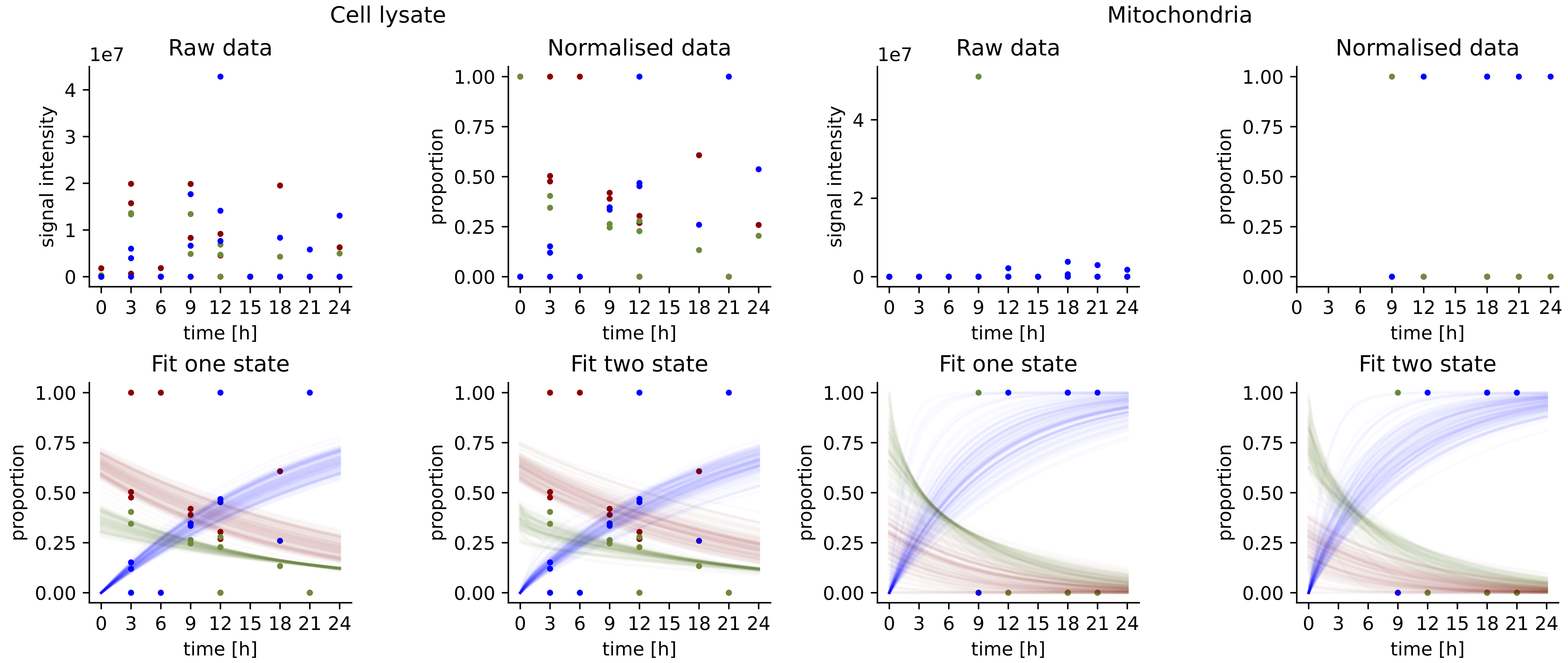

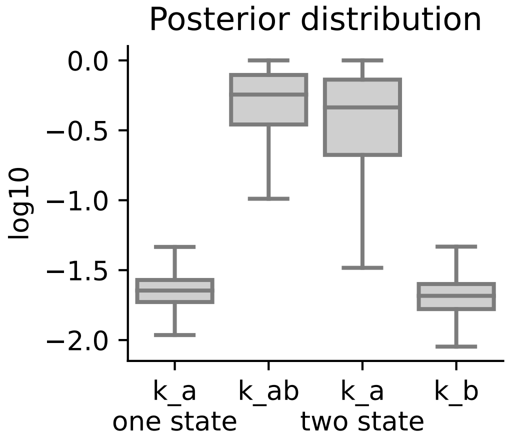

p value	9.58E-01
log likelihood one state	2.125
log likelihood two state	2.168
rmse one state	0.571
rmse two state	0.558
k_a one state	0.023
k_ab	0.57
k_a two state	0.461
k_b	0.021
sigma one state	0.219
sigma two state	0.22

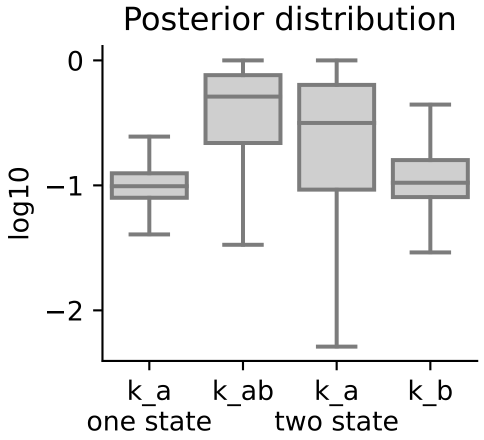

p value	6.21E-01
log likelihood one state	-1.952
log likelihood two state	-1.476
rmse one state	0.854
rmse two state	0.886
k_a one state	0.098
k_ab	0.513
k_a two state	0.316
k_b	0.105
sigma one state	0.249
sigma two state	0.251

METTL17
METTL17
Q9H7H0
Methyltransferase-like protein 17, mitochondrial

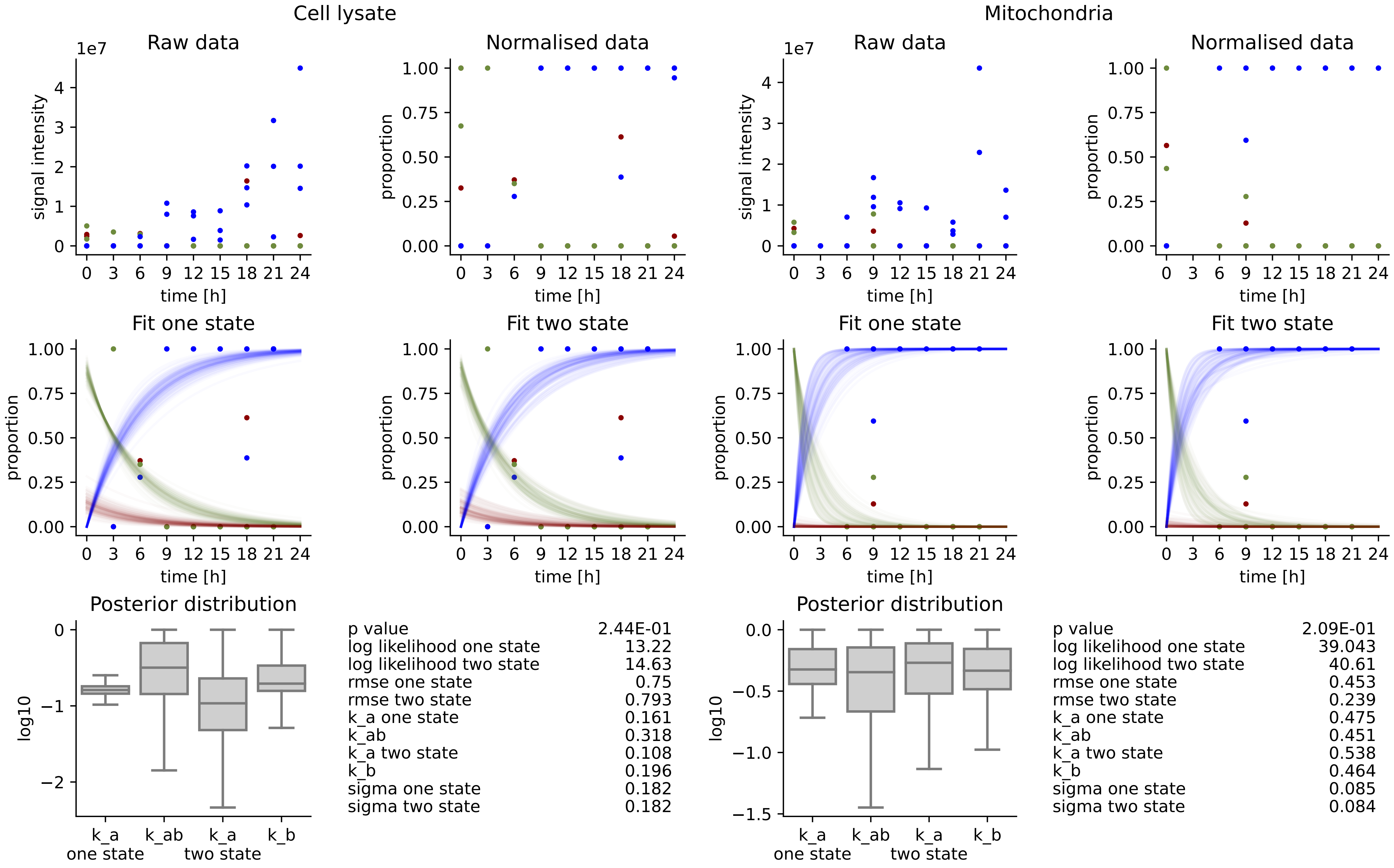

Cell lysate

p value	2.44E-01
log likelihood one state	13.22
log likelihood two state	14.63
rmse one state	0.75
rmse two state	0.793
k_a one state	0.161
k_ab	0.318
k_a two state	0.108
k_b	0.196
sigma one state	0.182
sigma two state	0.182

Mitochondria

p value	2.09E-01
log likelihood one state	39.043
log likelihood two state	40.61
rmse one state	0.453
rmse two state	0.239
k_a one state	0.475
k_ab	0.451
k_a two state	0.538
k_b	0.464
sigma one state	0.085
sigma two state	0.084

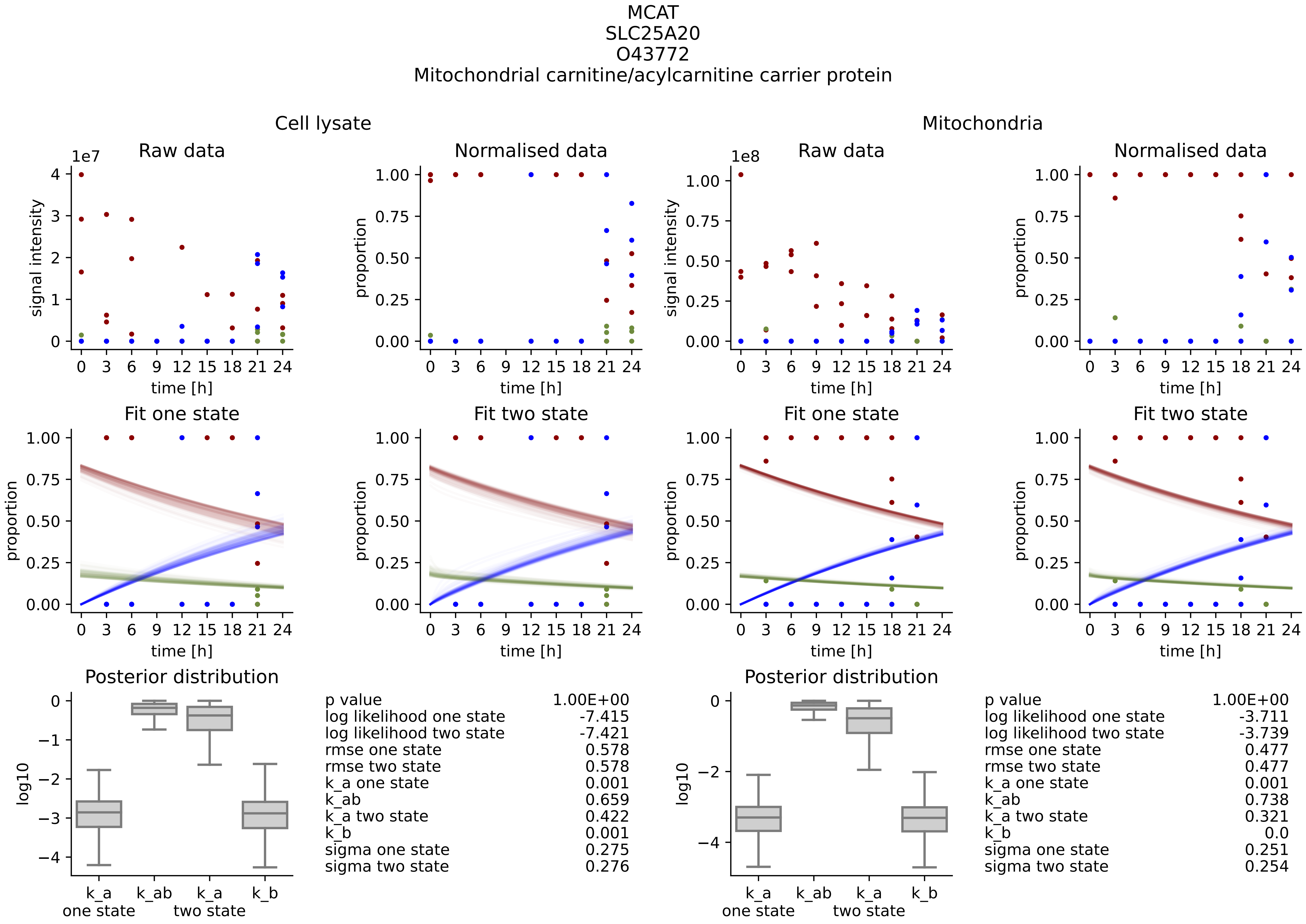
MCAT
SLC25A20
O43772
Mitochondrial carnitine/acylcarnitine carrier protein
Cell lysate
Mitochondria
Raw data
Normalised data
Fit one state
Fit two state
Posterior distribution
signal intensity
proportion
time [h]
log10
1e7
1e8
k_a one state
k_ab
k_a two state
k_b
p value 1.00E+00
log likelihood one state -7.415
log likelihood two state -7.421
rmse one state 0.578
rmse two state 0.578
k_a one state 0.001
k_ab 0.659
k_a two state 0.422
k_b 0.001
sigma one state 0.275
sigma two state 0.276
p value 1.00E+00
log likelihood one state -3.711
log likelihood two state -3.739
rmse one state 0.477
rmse two state 0.477
k_a one state 0.001
k_ab 0.738
k_a two state 0.321
k_b 0.0
sigma one state 0.251
sigma two state 0.254

ERAL1
ERAL1
O75616
GTPase Era, mitochondrial

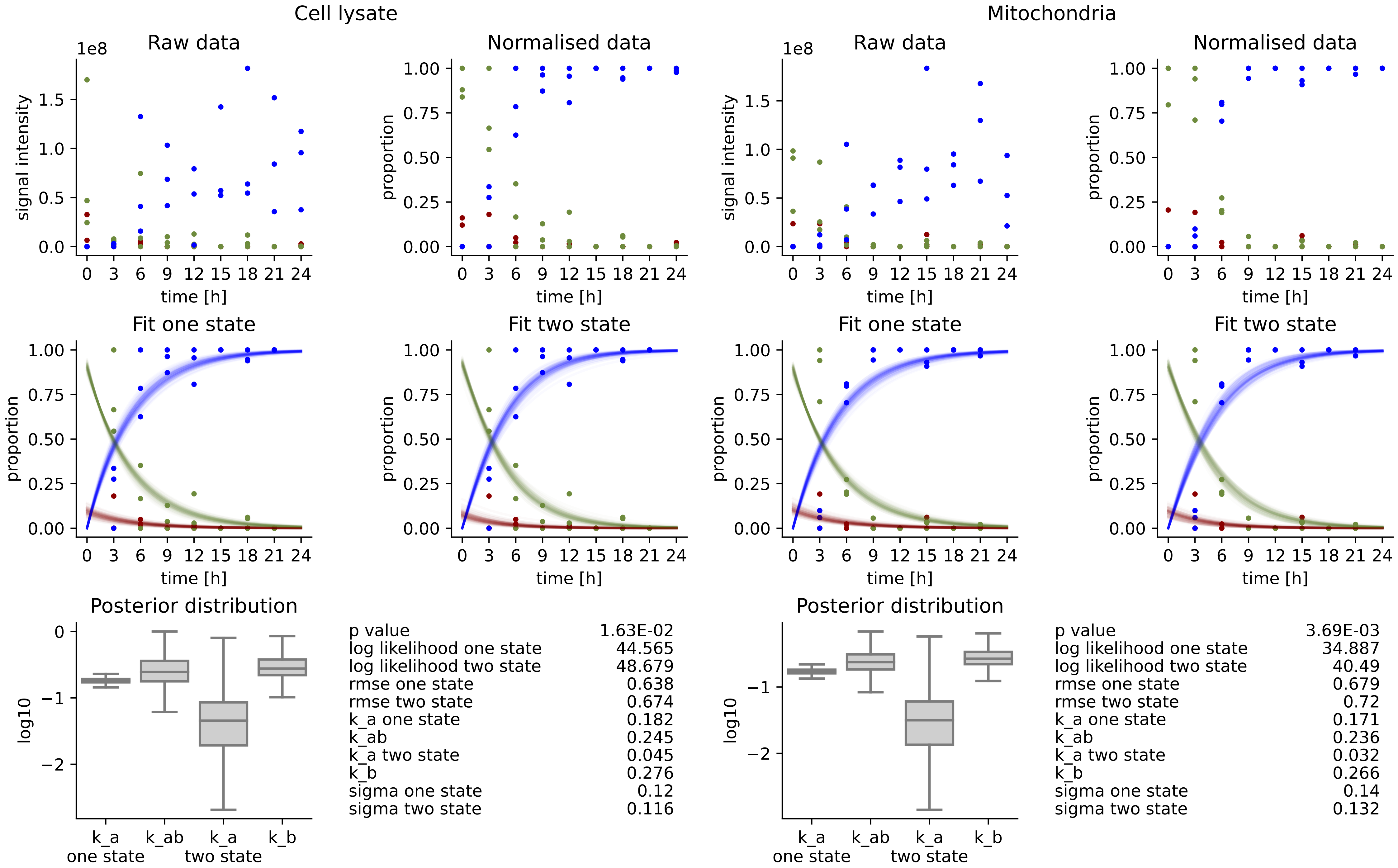

mtRBFA
RBFA
Q8N0V3
Putative ribosome-binding factor A, mitochondrial

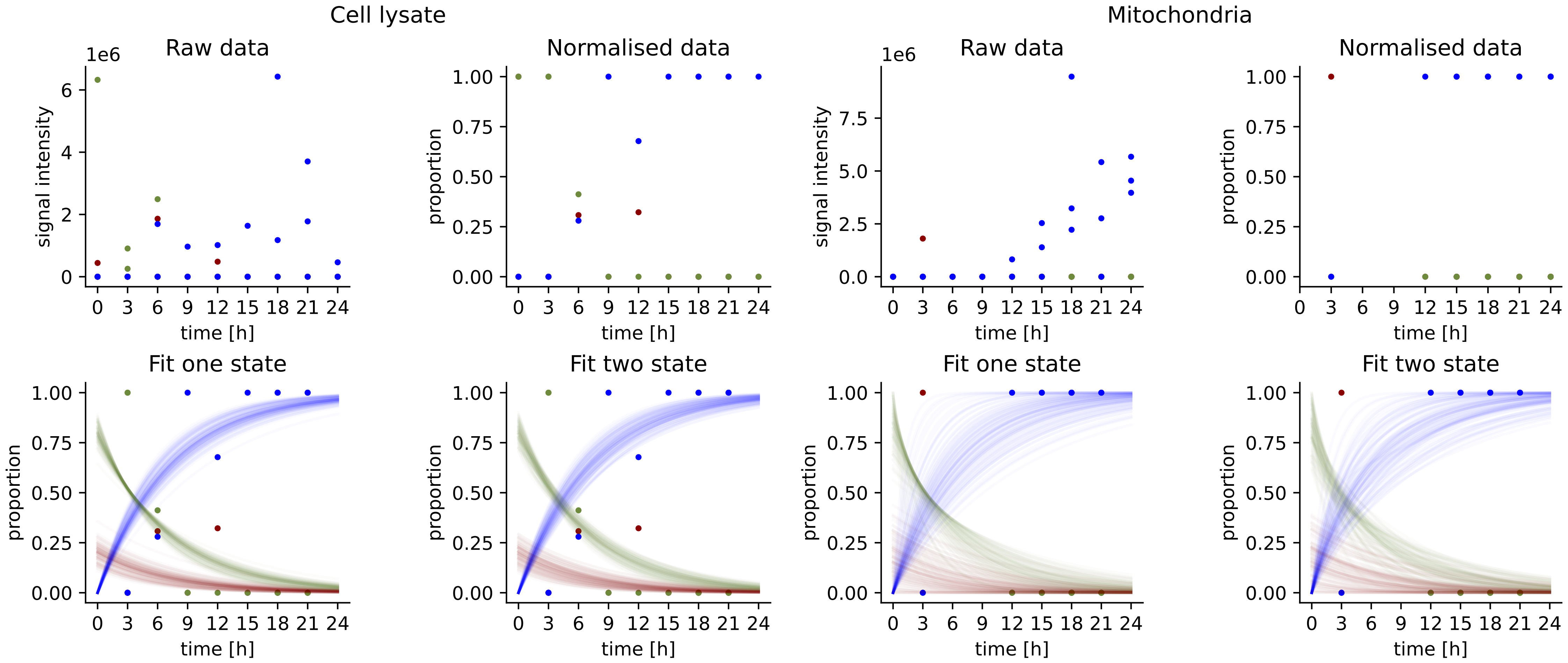

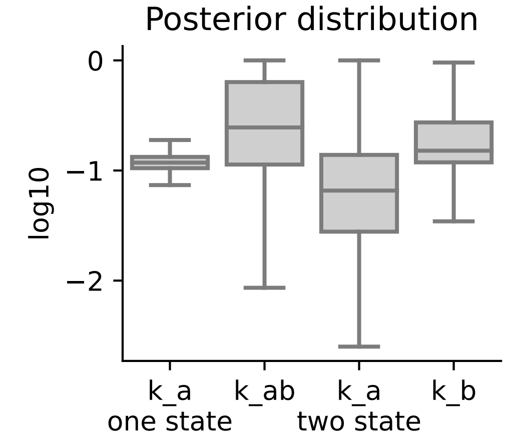

p value	1.37E-01
log likelihood one state	6.047
log likelihood two state	8.036
rmse one state	0.747
rmse two state	0.777
k_a one state	0.118
k_ab	0.246
k_a two state	0.066
k_b	0.151
sigma one state	0.194
sigma two state	0.192

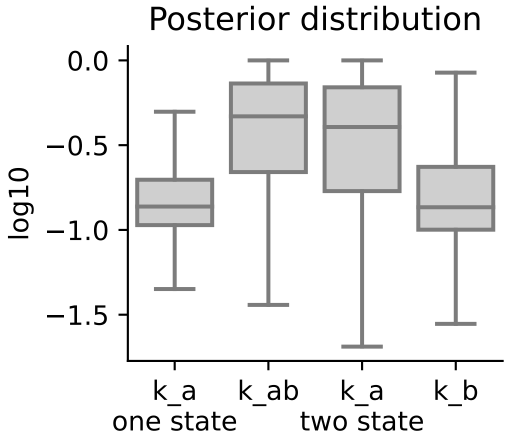

p value	7.70E-01
log likelihood one state	2.768
log likelihood two state	3.03
rmse one state	0.889
rmse two state	0.766
k_a one state	0.137
k_ab	0.468
k_a two state	0.405
k_b	0.136
sigma one state	0.213
sigma two state	0.213

MTG3
NOA1
Q8NC60
Nitric oxide-associated protein 1

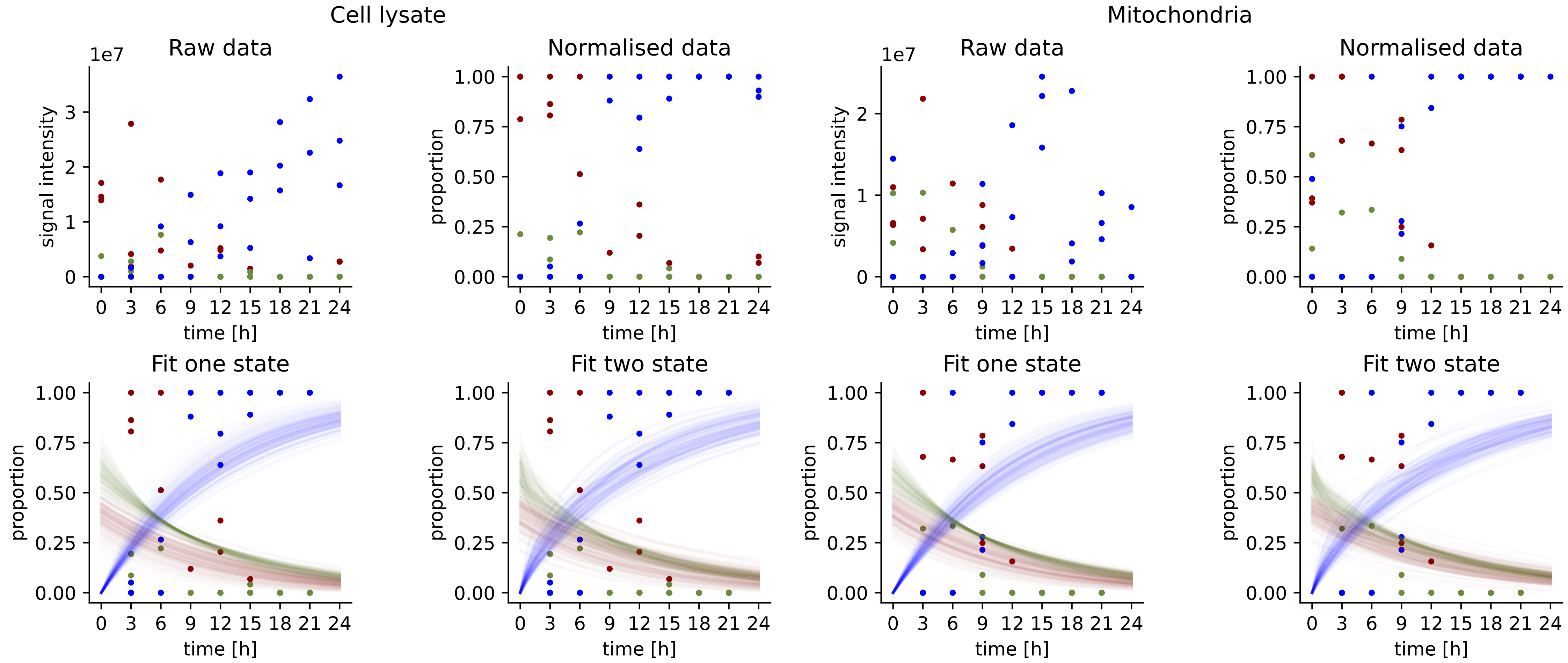

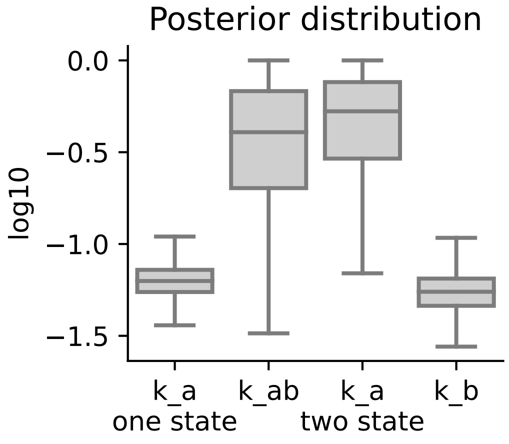

p value	1.82E-01
log likelihood one state	-6.065
log likelihood two state	-4.362
rmse one state	0.857
rmse two state	0.751
k_a one state	0.063
k_ab	0.406
k_a two state	0.528
k_b	0.055
sigma one state	0.26
sigma two state	0.257

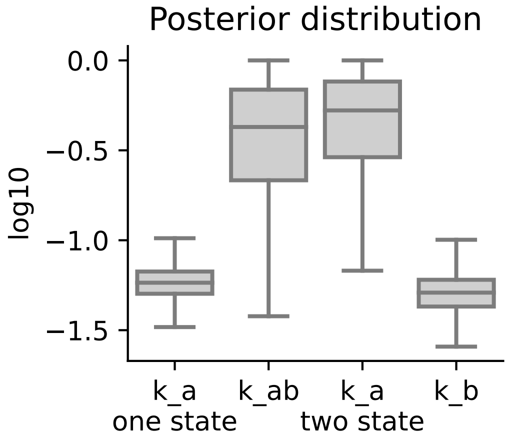

p value	2.54E-01
log likelihood one state	-8.829
log likelihood two state	-7.459
rmse one state	0.855
rmse two state	0.76
k_a one state	0.058
k_ab	0.426
k_a two state	0.527
k_b	0.051
sigma one state	0.271
sigma two state	0.269

mtIF3
MTIF3
Q9H2K0
Translation initiation factor IF-3, mitochondrial

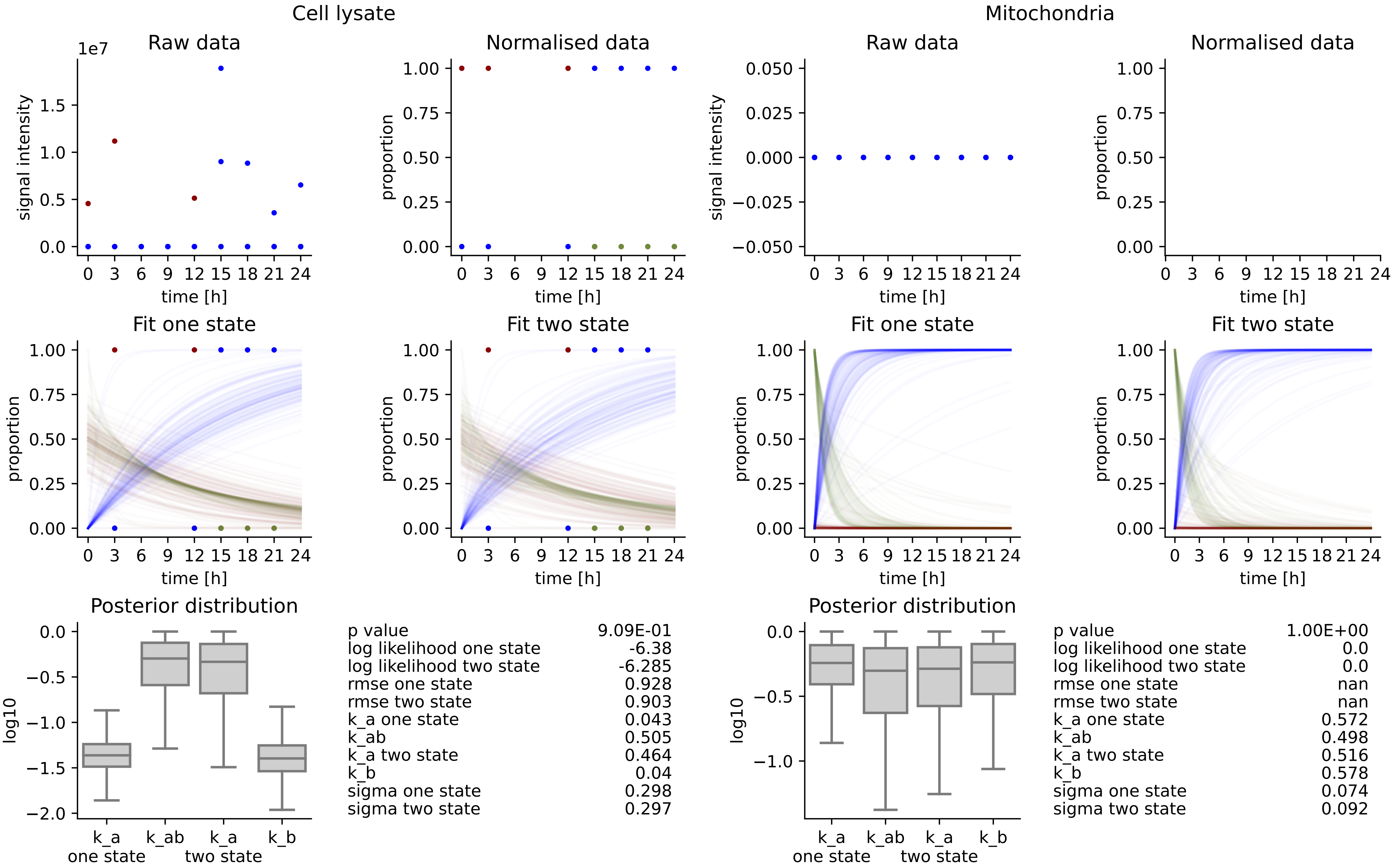

mtIF2
MTIF2
P46199
Translation initiation factor IF-2, mitochondrial

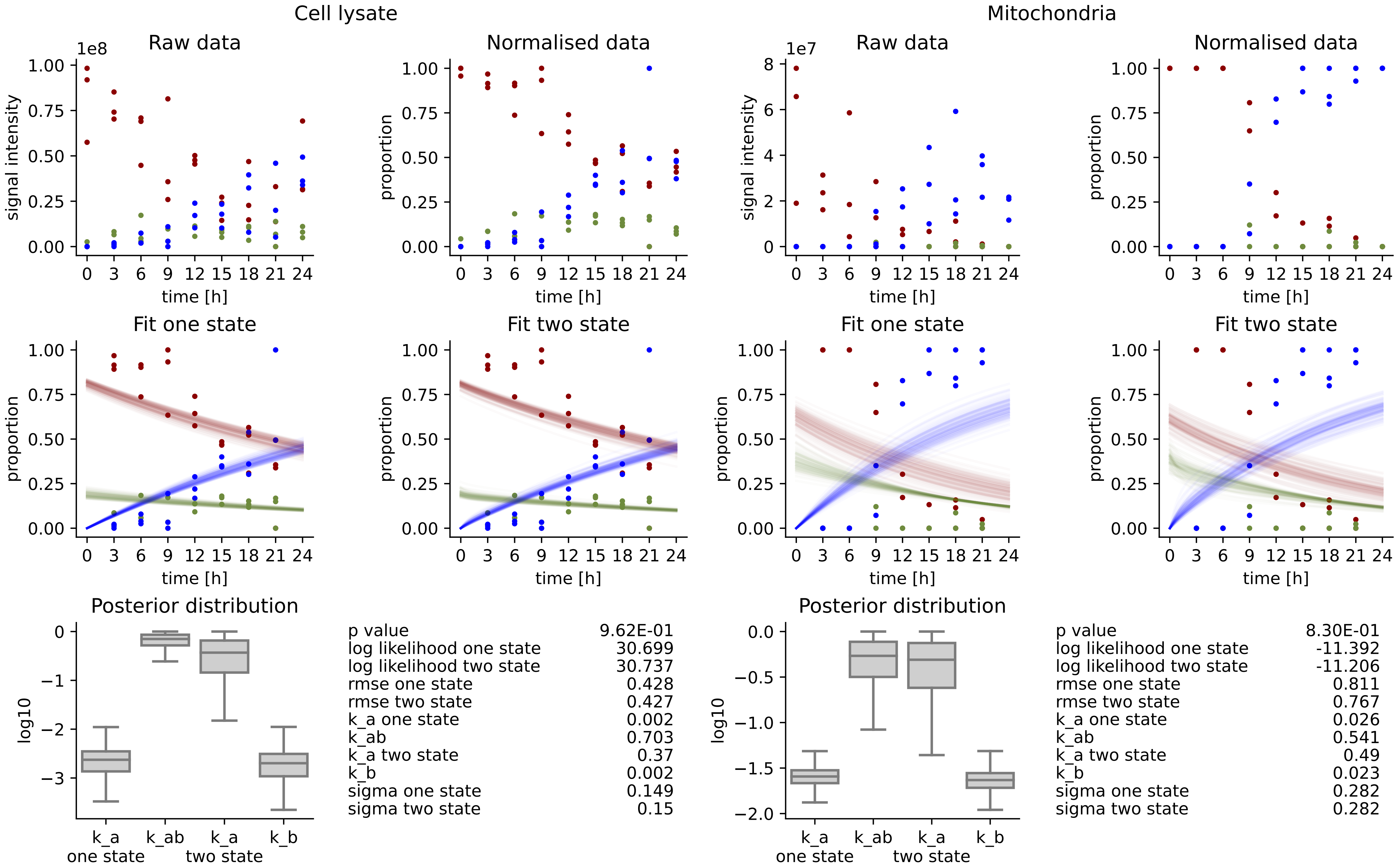

MRM2
FTSJ2
Q9UI43
rRNA methyltransferase 2, mitochondrial

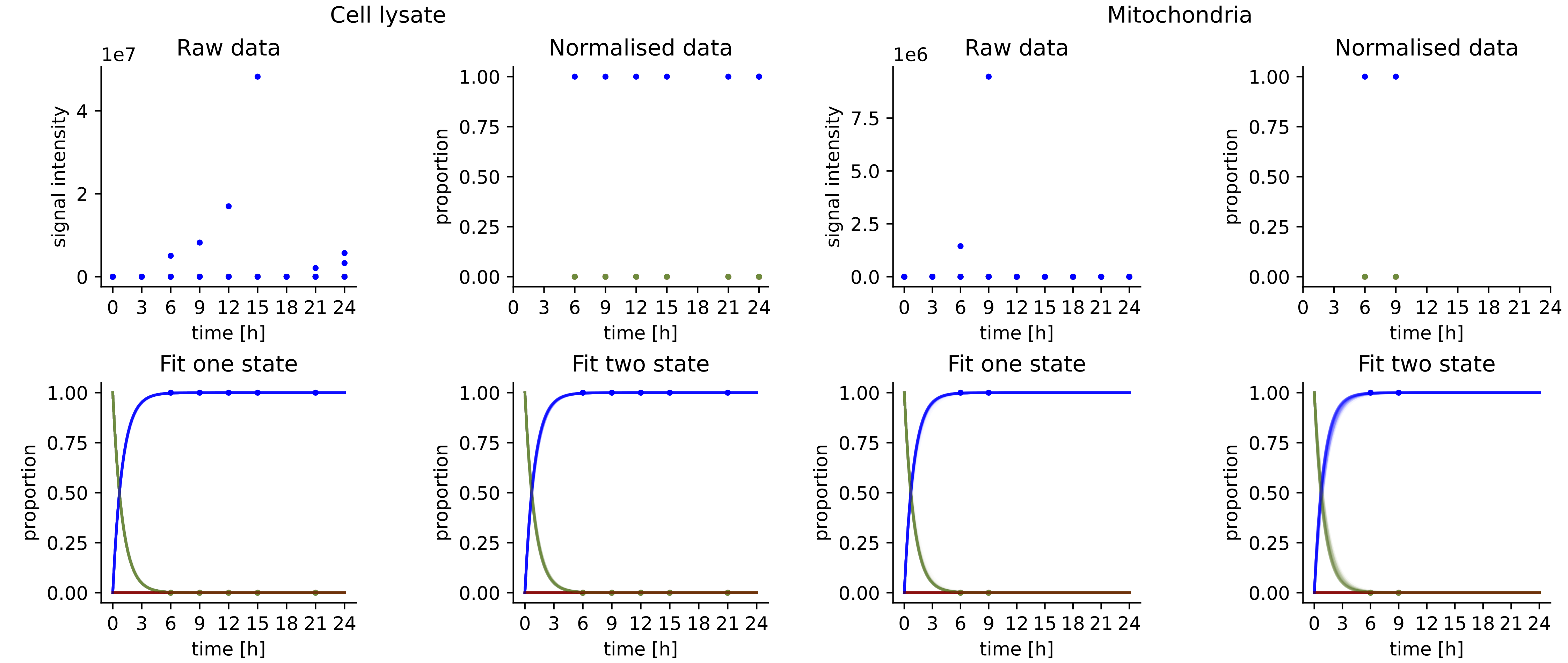

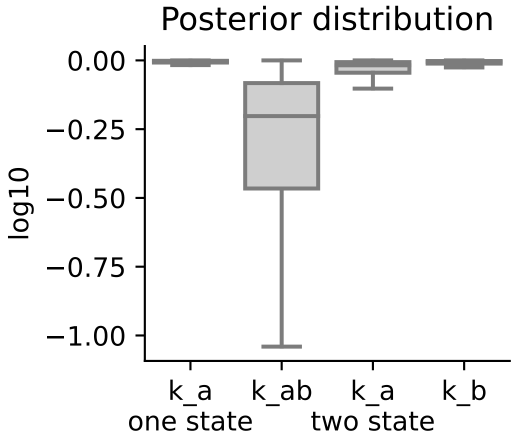

p value	1.00E+00
log likelihood one state	85.836
log likelihood two state	85.808
rmse one state	0.066
rmse two state	0.066
k_a one state	0.991
k_ab	0.627
k_a two state	0.96
k_b	0.987
sigma one state	0.001
sigma two state	0.001

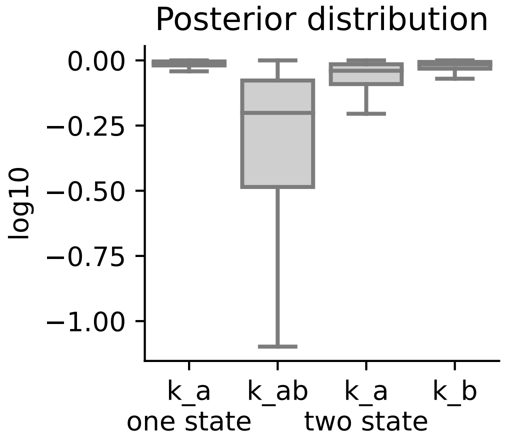

p value	1.00E+00
log likelihood one state	31.585
log likelihood two state	31.561
rmse one state	0.066
rmse two state	0.066
k_a one state	0.978
k_ab	0.629
k_a two state	0.913
k_b	0.966
sigma one state	0.002
sigma two state	0.002

MRM3
RNMTL1
Q9HC36
rRNA methyltransferase 3, mitochondrial

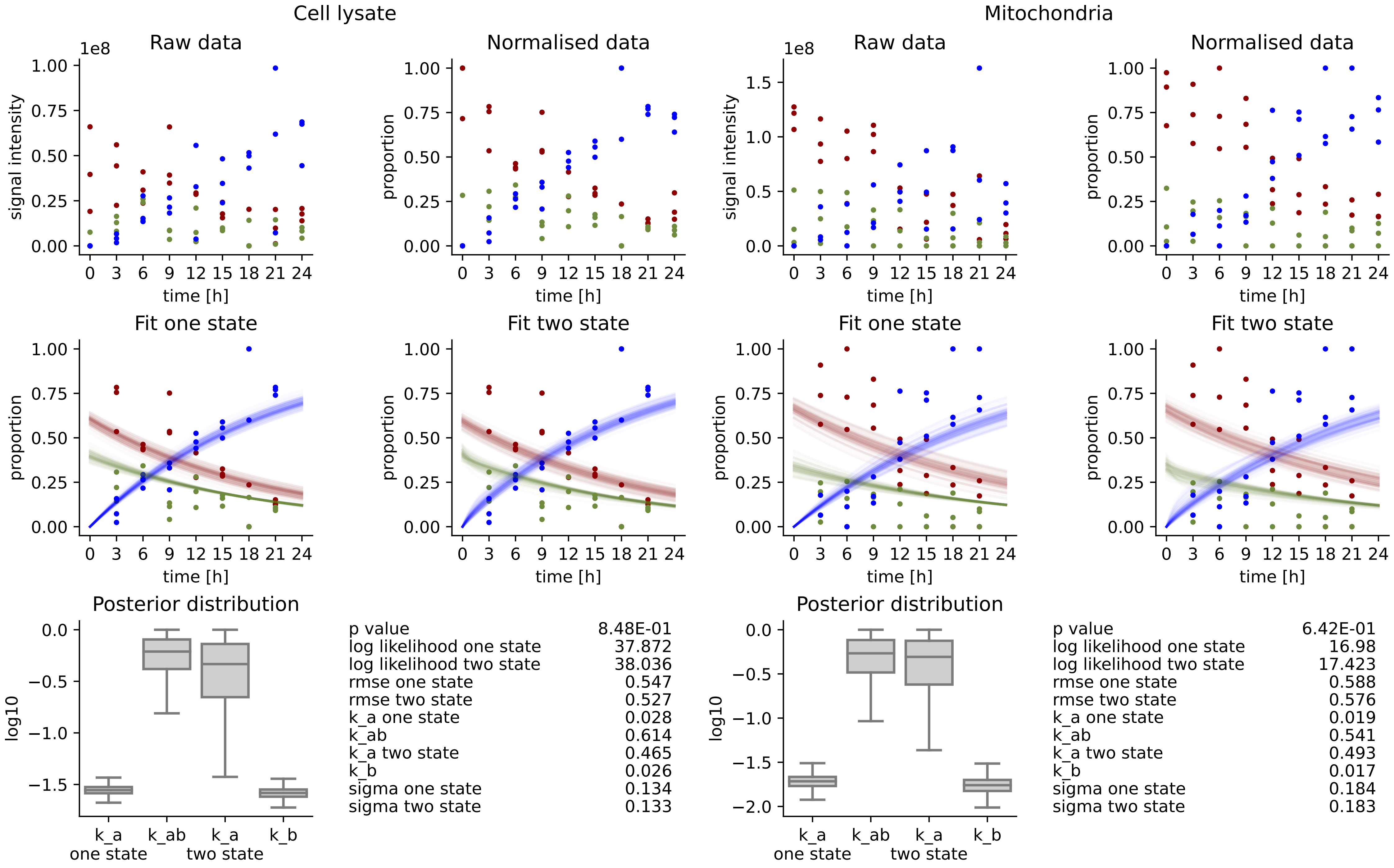

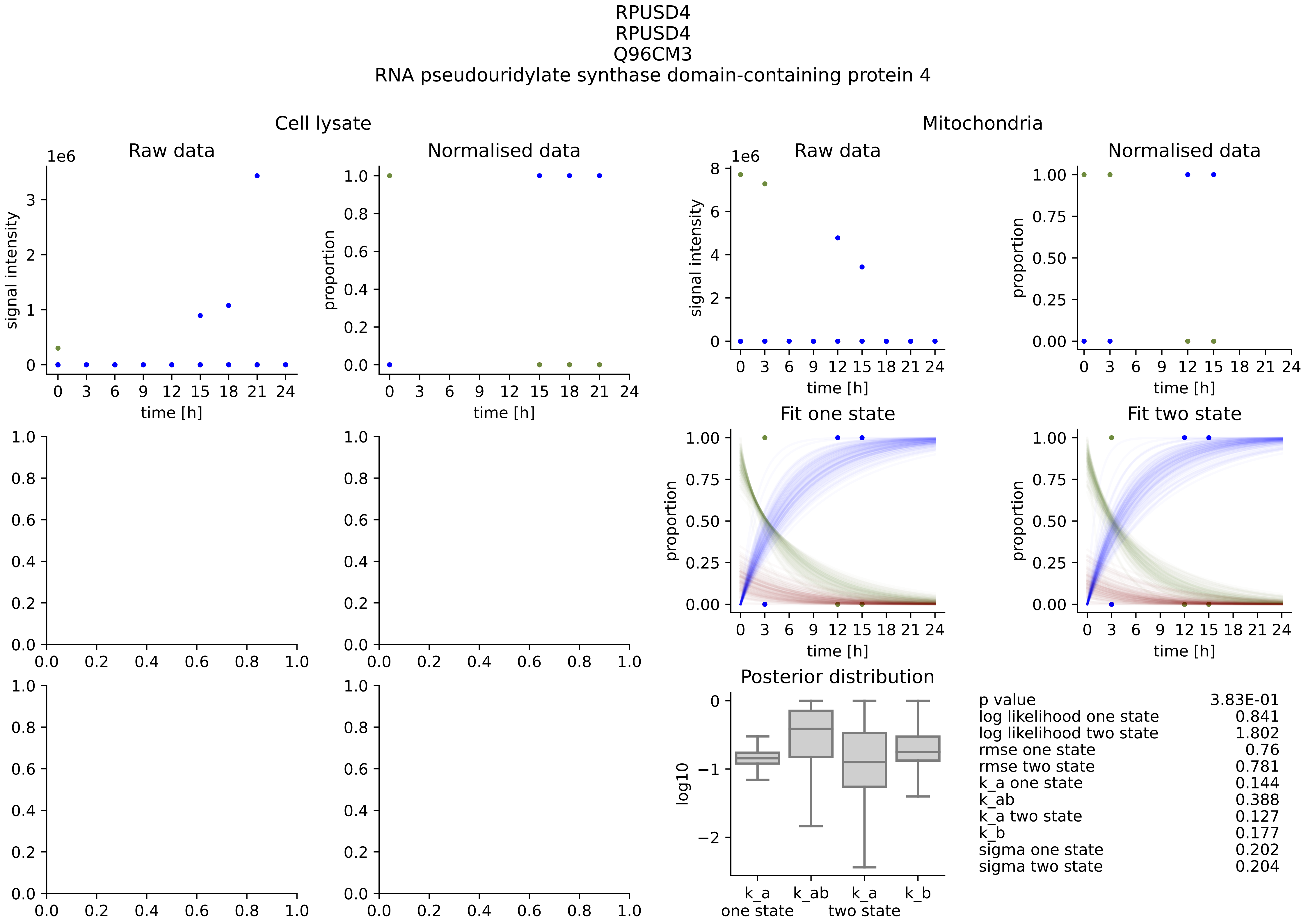
RPUSD4
RPUSD4
Q96CM3
RNA pseudouridylate synthase domain-containing protein 4
Cell lysate
Mitochondria
Raw data
Normalised data
Raw data
Normalised data
1e6
1e6
signal intensity
proportion
time [h]
Fit one state
Fit two state
Posterior distribution
log10
k_a
k_ab
k_a
k_b
one state
two state
p value 3.83E-01
log likelihood one state 0.841
log likelihood two state 1.802
rmse one state 0.76
rmse two state 0.781
k_a one state 0.144
k_ab 0.388
k_a two state 0.127
k_b 0.177
sigma one state 0.202
sigma two state 0.204

MALSU1
MALSU1
Q96EH3
Mitochondrial assembly of ribosomal large subunit protein 1

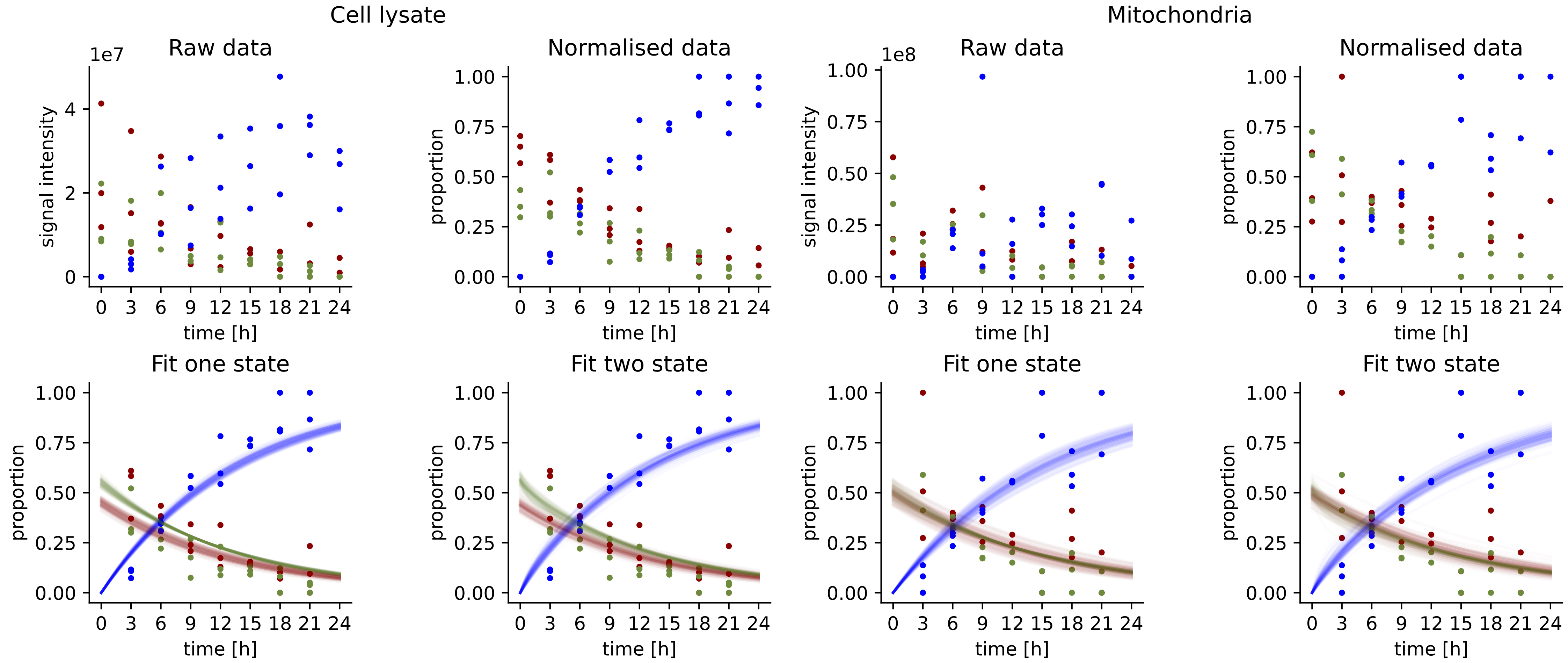

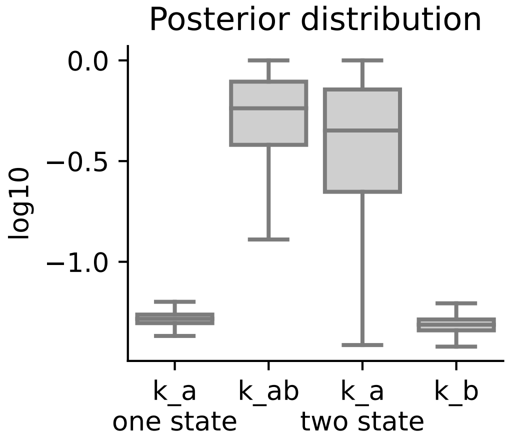

p value	1.16E-01
log likelihood one state	52.385
log likelihood two state	54.538
rmse one state	0.598
rmse two state	0.552
k_a one state	0.052
k_ab	0.578
k_a two state	0.449
k_b	0.049
sigma one state	0.107
sigma two state	0.106

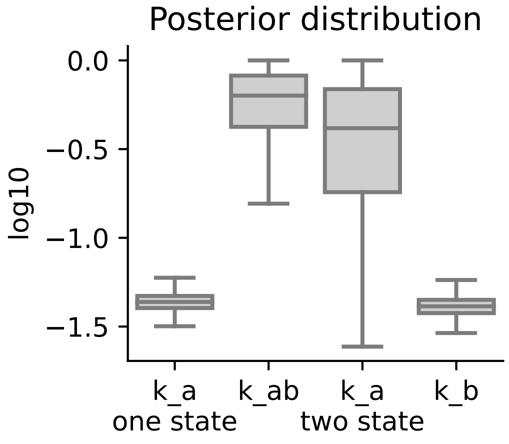

p value	6.56E-01
log likelihood one state	24.983
log likelihood two state	25.404
rmse one state	0.612
rmse two state	0.596
k_a one state	0.044
k_ab	0.633
k_a two state	0.415
k_b	0.041
sigma one state	0.16
sigma two state	0.16

L0R8F8
SMCR7L
L0R8F8
nan

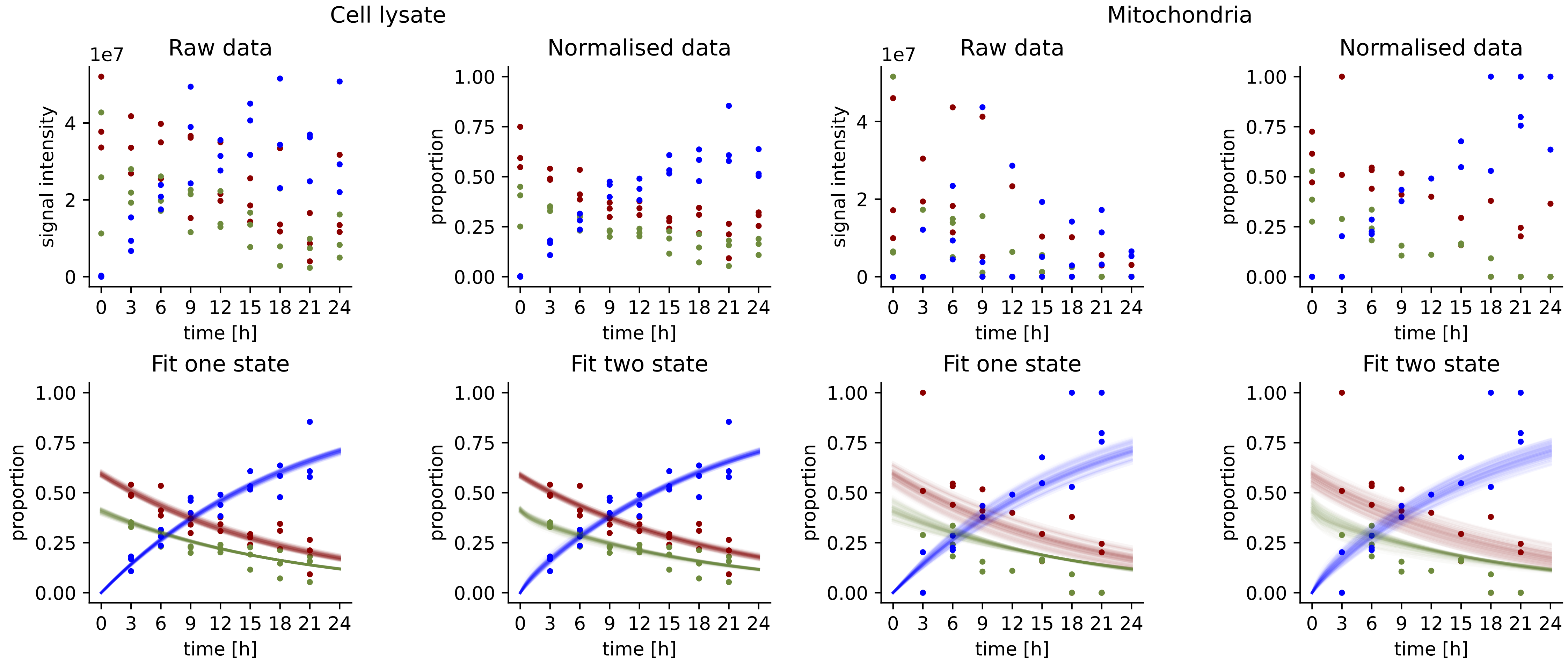

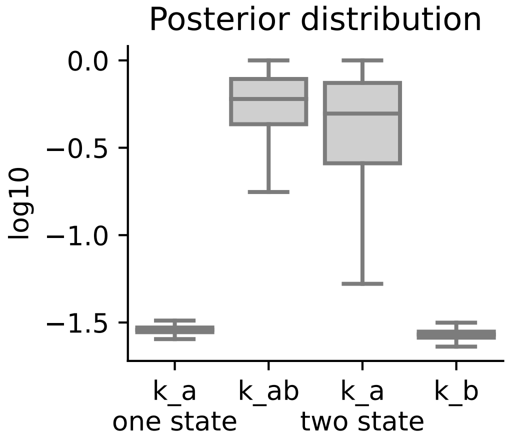

p value	1.57E-01
log likelihood one state	91.33
log likelihood two state	93.181
rmse one state	0.433
rmse two state	0.407
k_a one state	0.029
k_ab	0.601
k_a two state	0.496
k_b	0.027
sigma one state	0.058
sigma two state	0.057

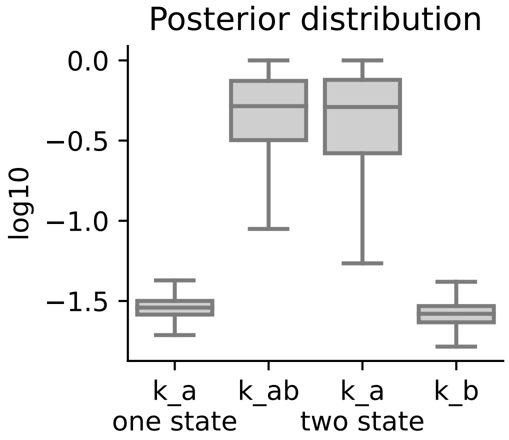

p value	5.41E-01
log likelihood one state	20.32
log likelihood two state	20.935
rmse one state	0.593
rmse two state	0.539
k_a one state	0.029
k_ab	0.518
k_a two state	0.512
k_b	0.026
sigma one state	0.155
sigma two state	0.153

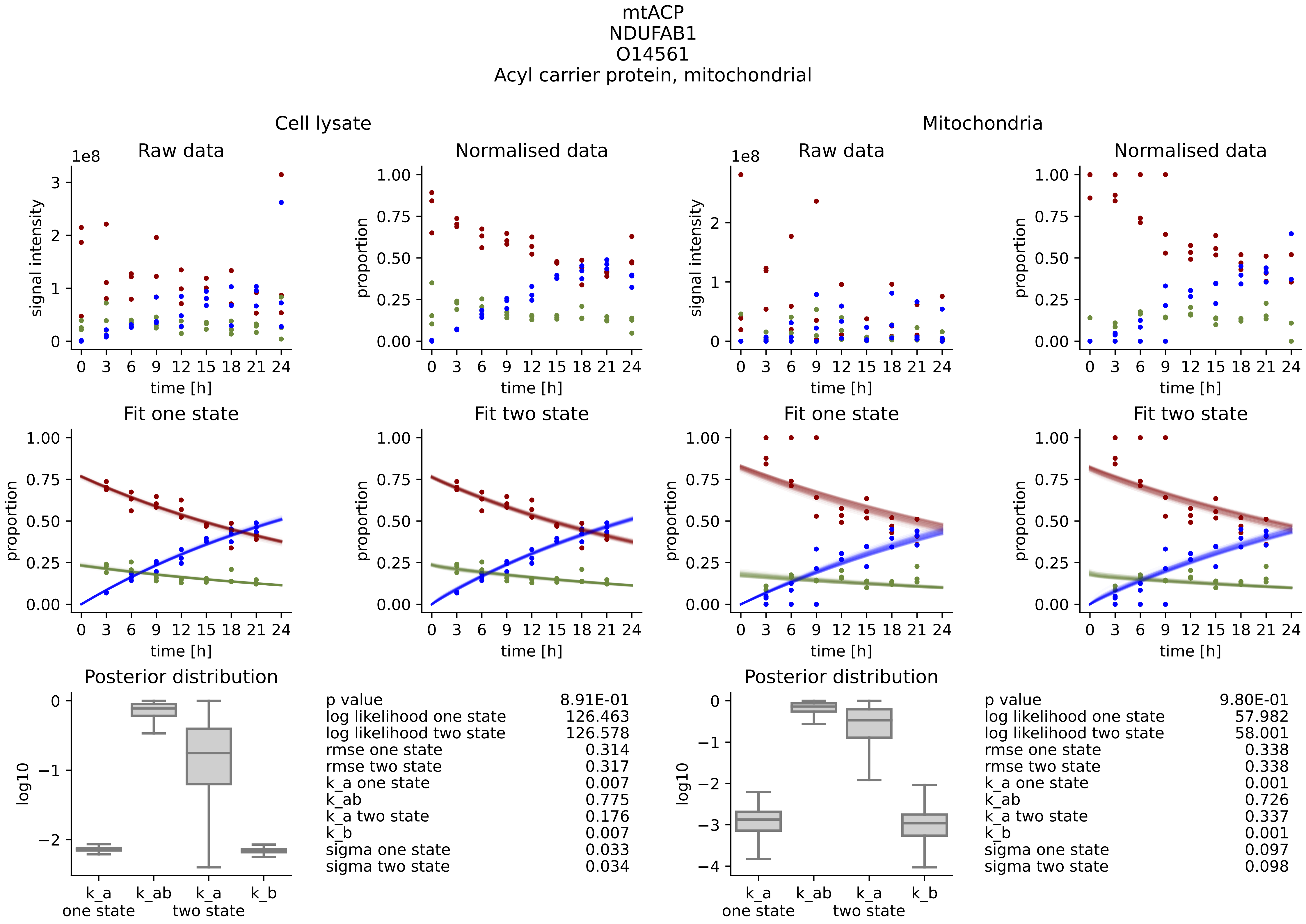
mtACP
NDUFAB1
O14561
Acyl carrier protein, mitochondrial
Cell lysate
Mitochondria
Raw data
Normalised data
Raw data
Normalised data
1e8
signal intensity
proportion
time [h]
Fit one state
Fit two state
Fit one state
Fit two state
Posterior distribution
log10
k_a one state
k_ab
k_a two state
k_b
p value 8.91E-01
log likelihood one state 126.463
log likelihood two state 126.578
rmse one state 0.314
rmse two state 0.317
k_a one state 0.007
k_ab 0.775
k_a two state 0.176
k_b 0.007
sigma one state 0.033
sigma two state 0.034
Posterior distribution
p value 9.80E-01
log likelihood one state 57.982
log likelihood two state 58.001
rmse one state 0.338
rmse two state 0.338
k_a one state 0.001
k_ab 0.726
k_a two state 0.337
k_b 0.001
sigma one state 0.097
sigma two state 0.098

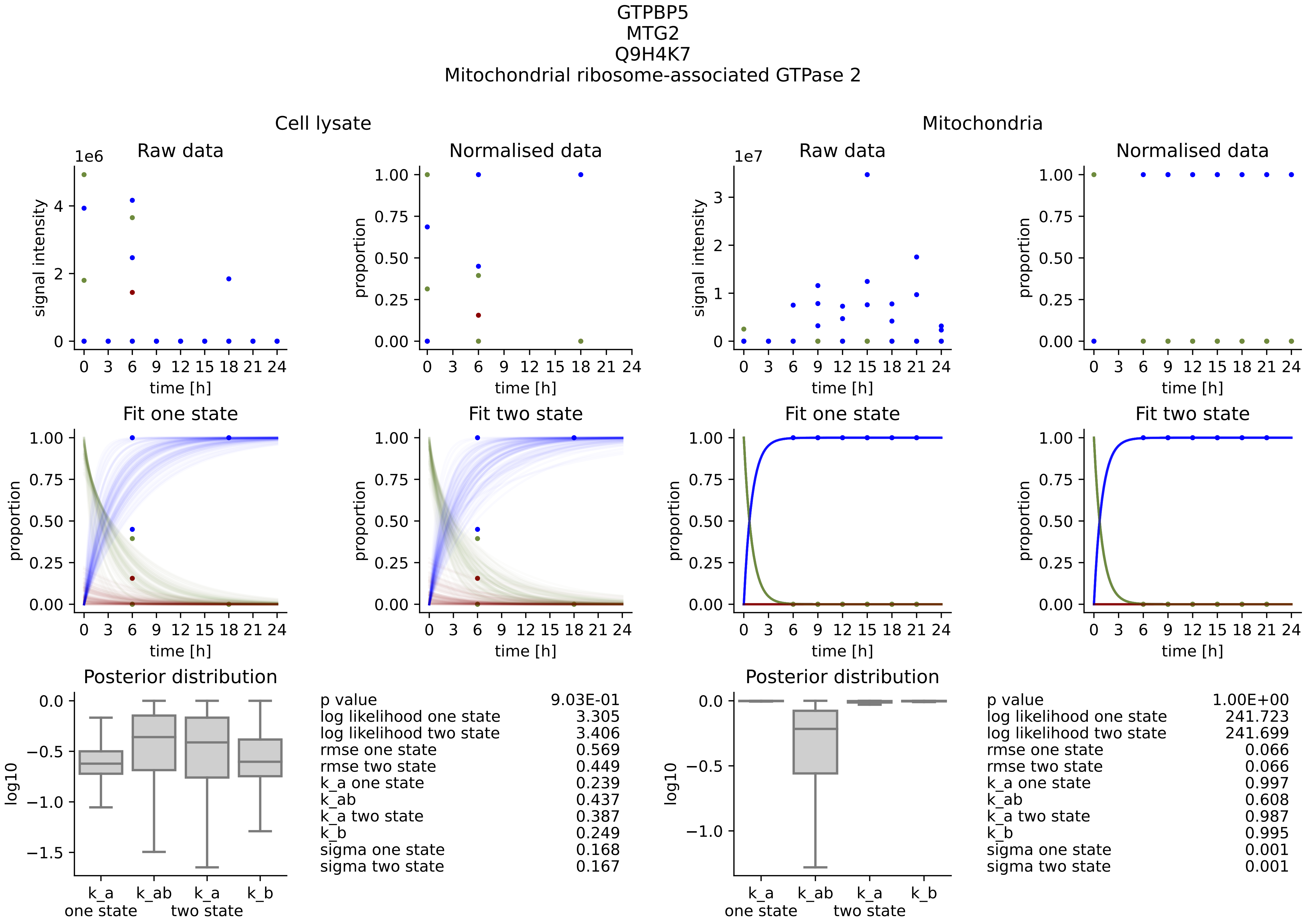
GTPBP5
MTG2
Q9H4K7
Mitochondrial ribosome-associated GTPase 2
Cell lysate
Mitochondria
Raw data
Normalised data
Fit one state
Fit two state
Posterior distribution
1e6
1e7
signal intensity
proportion
time [h]
log10
k_a
k_ab
k_b
one state
two state
p value 9.03E-01
log likelihood one state 3.305
log likelihood two state 3.406
rmse one state 0.569
rmse two state 0.449
k_a one state 0.239
k_ab 0.437
k_a two state 0.387
k_b 0.249
sigma one state 0.168
sigma two state 0.167
p value 1.00E+00
log likelihood one state 241.723
log likelihood two state 241.699
rmse one state 0.066
rmse two state 0.066
k_a one state 0.997
k_ab 0.608
k_a two state 0.987
k_b 0.995
sigma one state 0.001
sigma two state 0.001

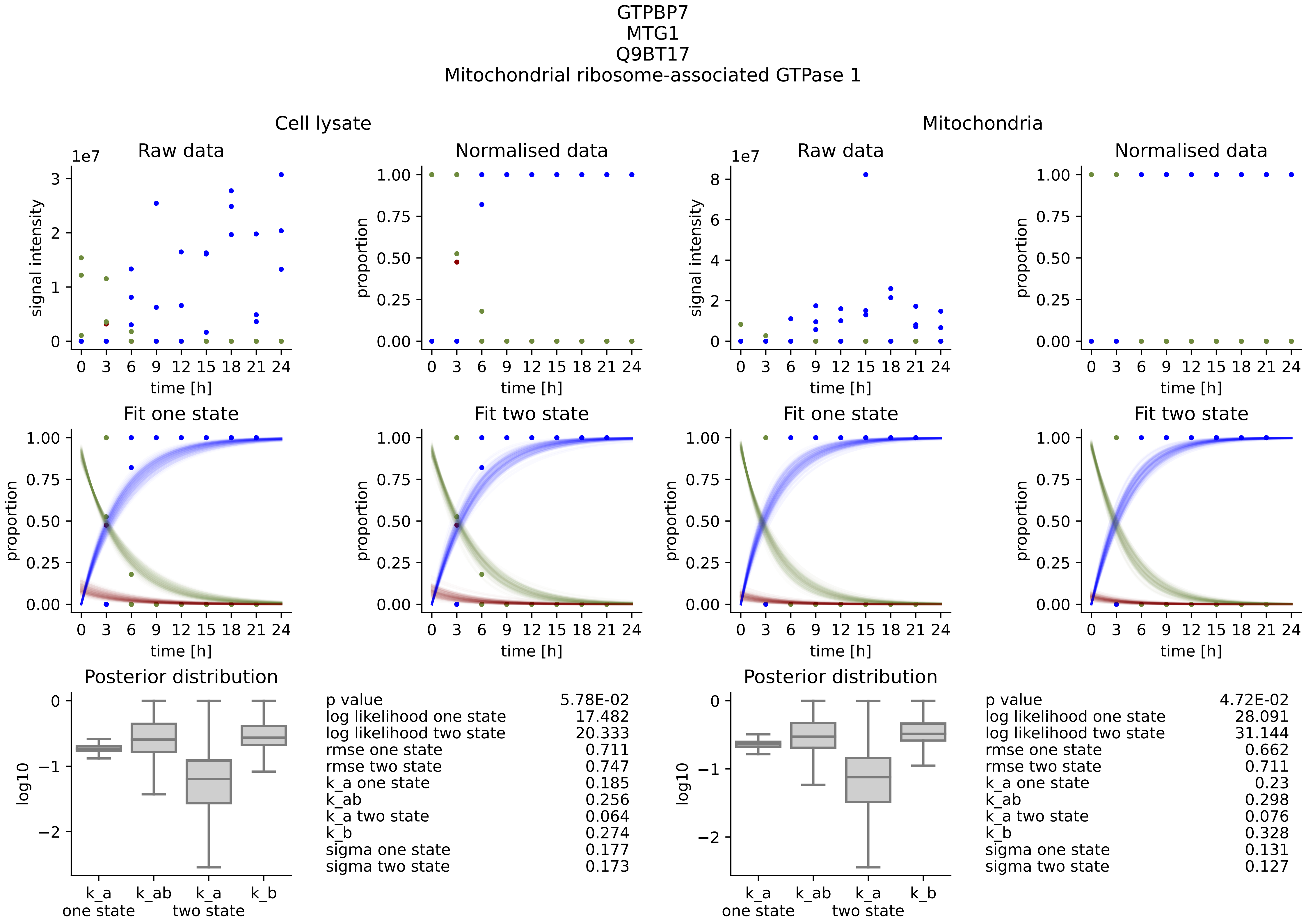
GTPBP7
MTG1
Q9BT17
Mitochondrial ribosome-associated GTPase 1
Cell lysate
Mitochondria
Raw data
Normalised data
1e7
signal intensity
proportion
time [h]
Fit one state
Fit two state
Posterior distribution
log10
k_a
one state
k_ab
k_a
two state
k_b
p value 5.78E-02
log likelihood one state 17.482
log likelihood two state 20.333
rmse one state 0.711
rmse two state 0.747
k_a one state 0.185
k_ab 0.256
k_a two state 0.064
k_b 0.274
sigma one state 0.177
sigma two state 0.173
p value 4.72E-02
log likelihood one state 28.091
log likelihood two state 31.144
rmse one state 0.662
rmse two state 0.711
k_a one state 0.23
k_ab 0.298
k_a two state 0.076
k_b 0.328
sigma one state 0.131
sigma two state 0.127

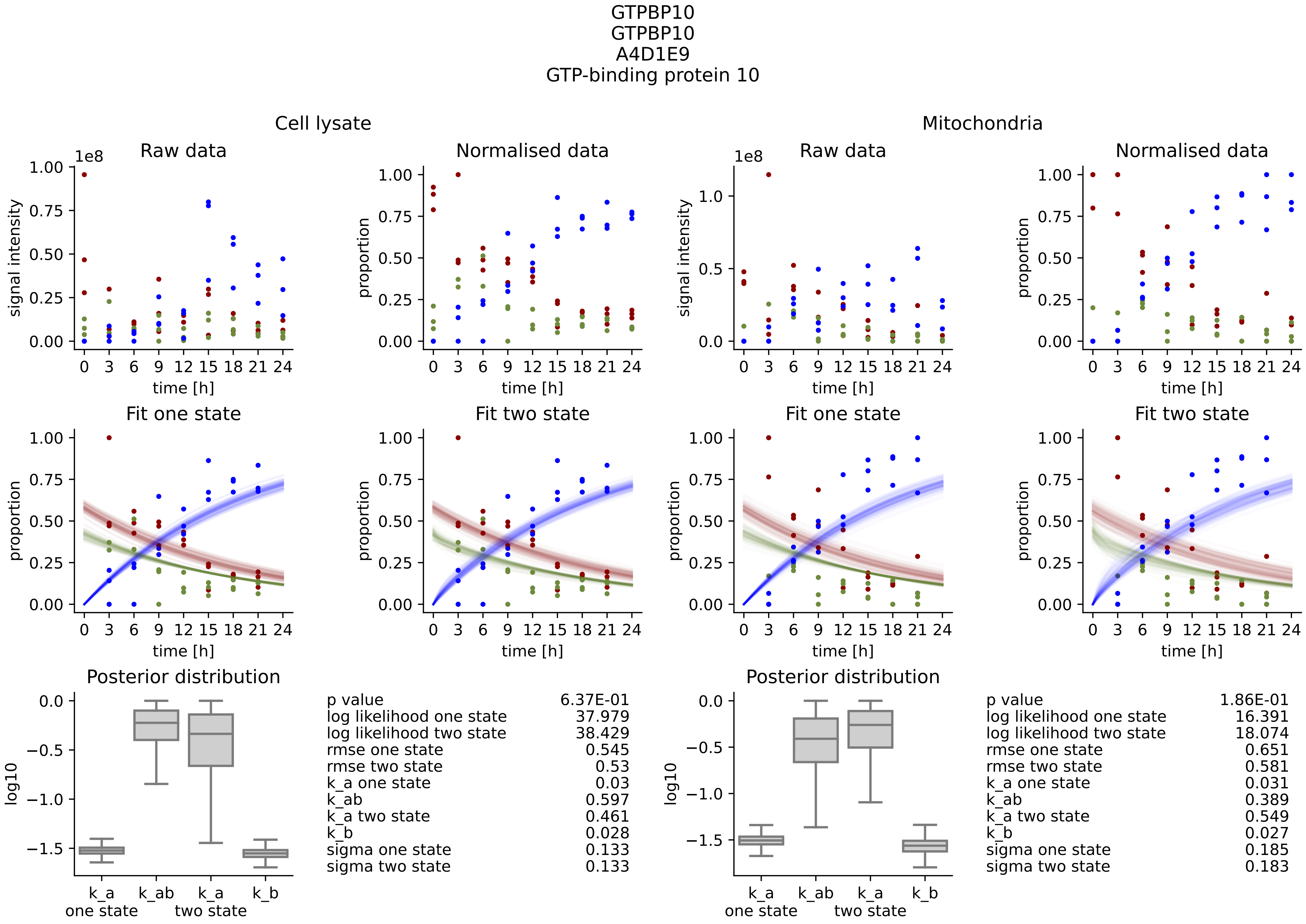
GTPBP10
GTPBP10
A4D1E9
GTP-binding protein 10
Cell lysate
Mitochondria
Raw data
Normalised data
Fit one state
Fit two state
Posterior distribution
signal intensity
proportion
log10
time [h]
1e8
k_a one state
k_ab
k_a two state
k_b
p value 6.37E-01
log likelihood one state 37.979
log likelihood two state 38.429
rmse one state 0.545
rmse two state 0.53
k_a one state 0.03
k_ab 0.597
k_a two state 0.461
k_b 0.028
sigma one state 0.133
sigma two state 0.133
p value 1.86E-01
log likelihood one state 16.391
log likelihood two state 18.074
rmse one state 0.651
rmse two state 0.581
k_a one state 0.031
k_ab 0.389
k_a two state 0.549
k_b 0.027
sigma one state 0.185
sigma two state 0.183

MTERF4
MTERF4
Q7Z6M4
Transcription termination factor 4, mitochondrial;mTERF domain-containing protein 2 processed

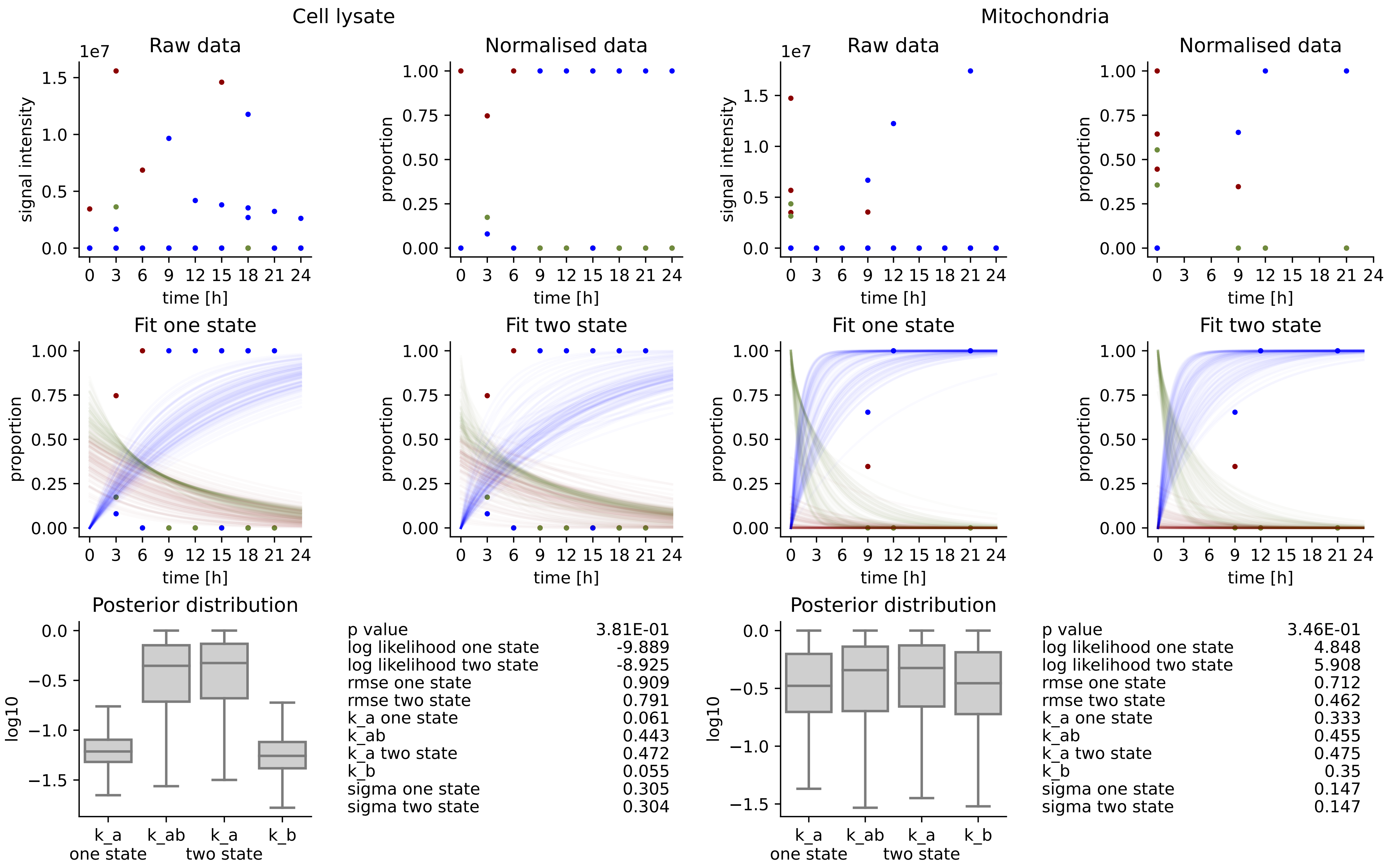

DDX28
DDX28
Q9NUL7
Probable ATP-dependent RNA helicase DDX28

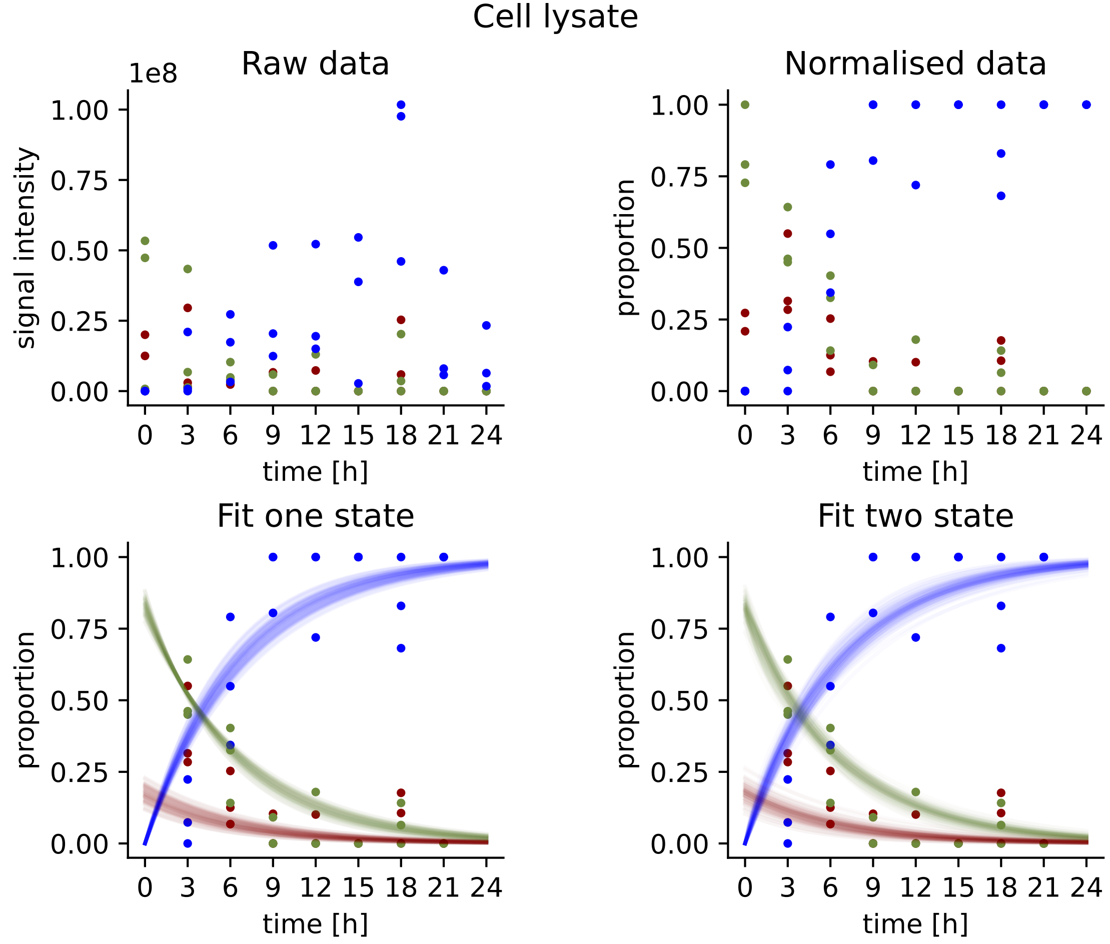

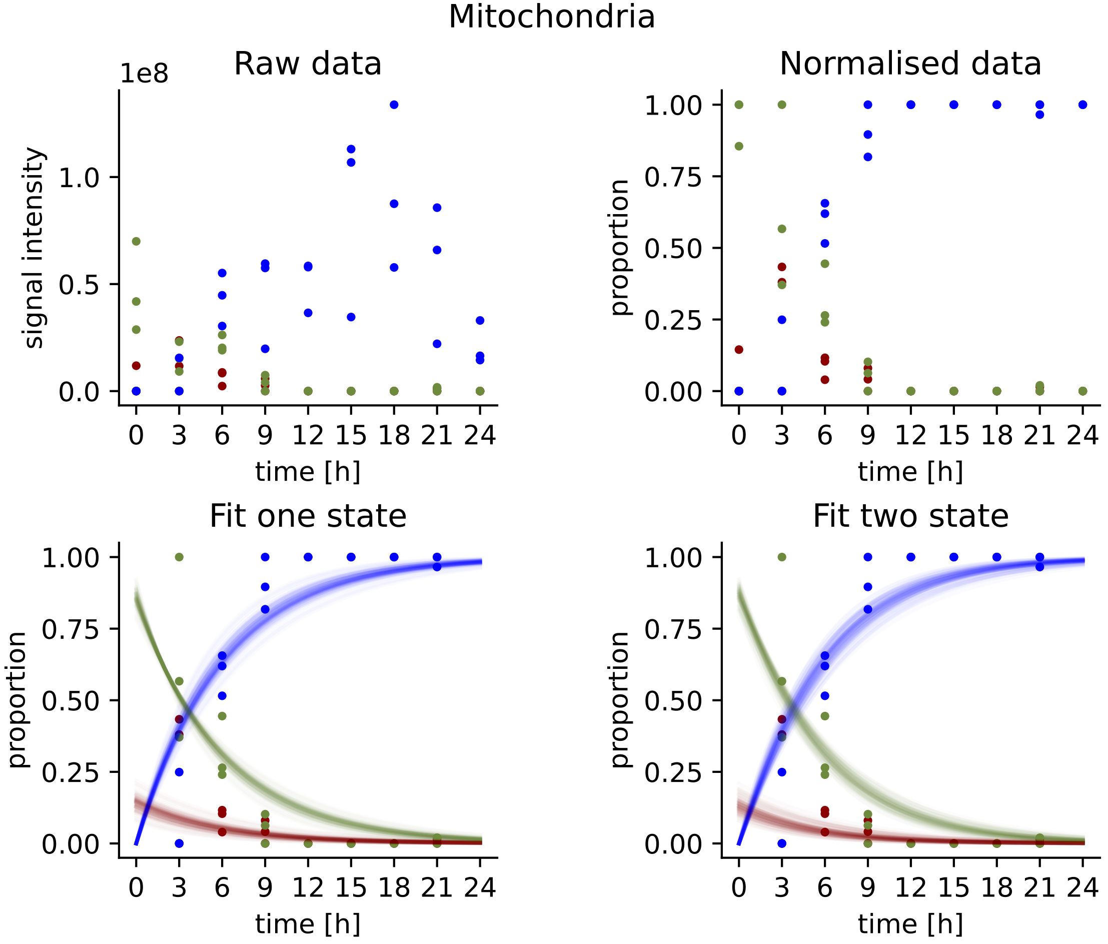

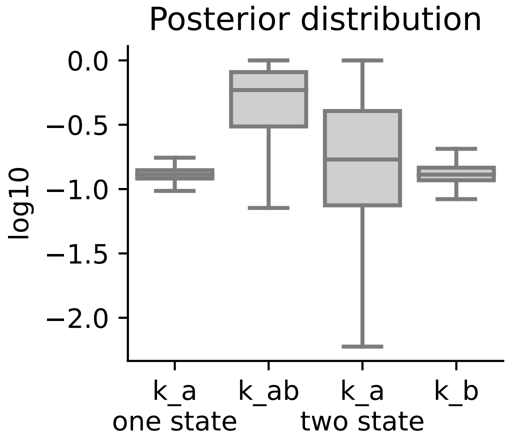

p value	7.22E-01
log likelihood one state	34.094
log likelihood two state	34.419
rmse one state	0.685
rmse two state	0.708
k_a one state	0.13
k_ab	0.588
k_a two state	0.17
k_b	0.13
sigma one state	0.142
sigma two state	0.143

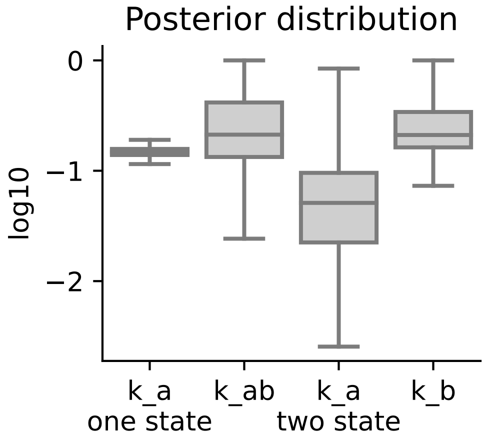

p value	4.78E-02
log likelihood one state	37.535
log likelihood two state	40.576
rmse one state	0.694
rmse two state	0.736
k_a one state	0.148
k_ab	0.212
k_a two state	0.051
k_b	0.211
sigma one state	0.134
sigma two state	0.131

MTERF3
MTERF3
Q96E29
Transcription termination factor 3, mitochondrial

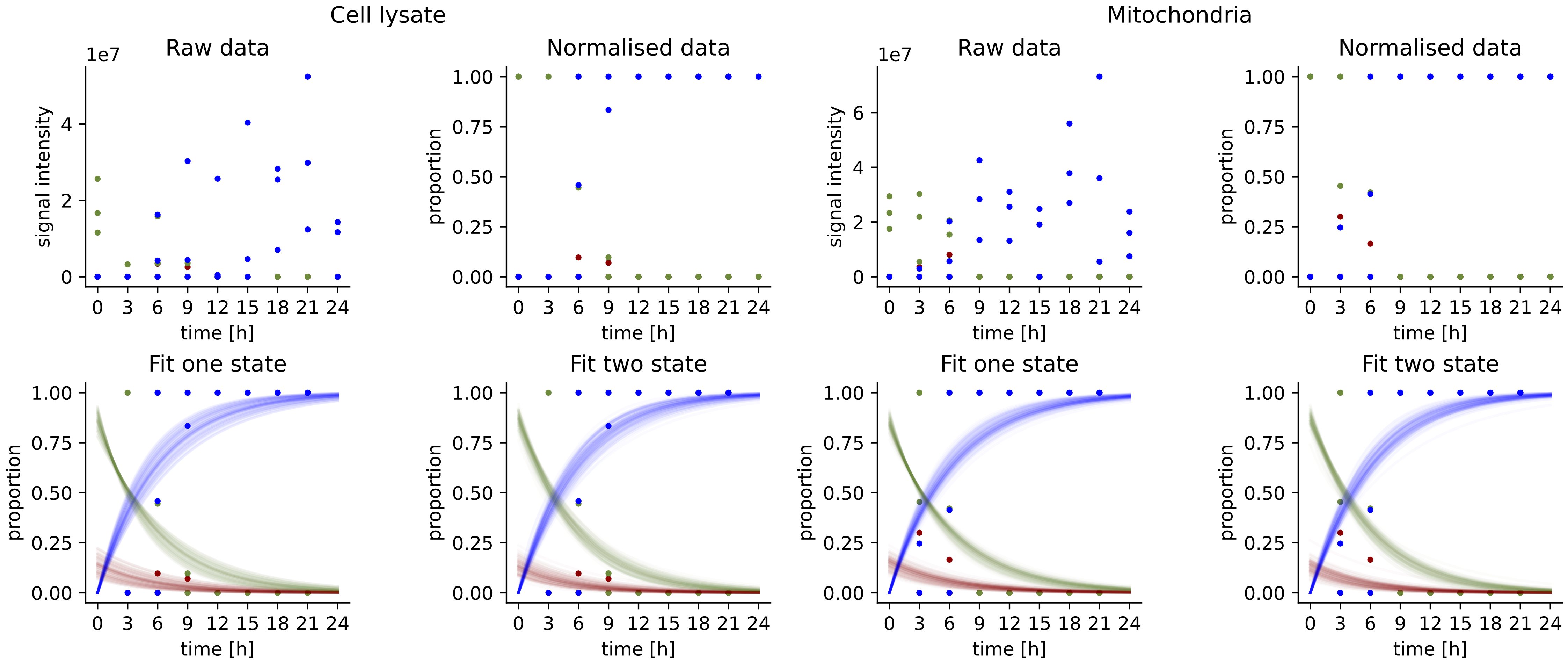

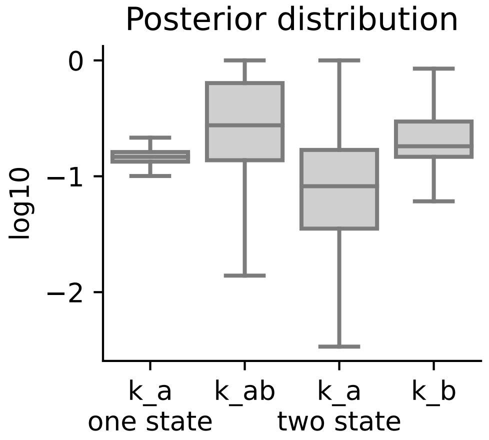

p value	1.34E-01
log likelihood one state	11.204
log likelihood two state	13.212
rmse one state	0.745
rmse two state	0.789
k_a one state	0.147
k_ab	0.275
k_a two state	0.082
k_b	0.182
sigma one state	0.19
sigma two state	0.188

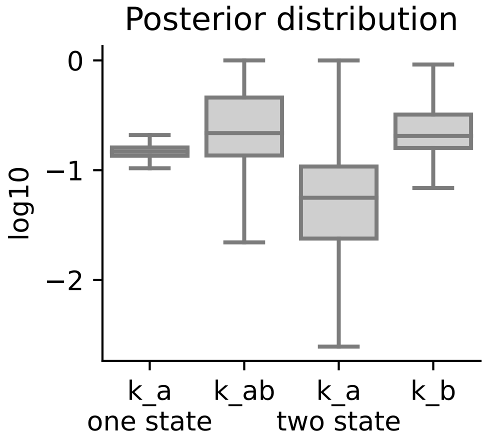

p value	6.21E-02
log likelihood one state	10.893
log likelihood two state	13.673
rmse one state	0.738
rmse two state	0.779
k_a one state	0.147
k_ab	0.218
k_a two state	0.056
k_b	0.205
sigma one state	0.199
sigma two state	0.197

FASTKD2
FASTKD2
Q9NYY8
FAST kinase domain-containing protein 2

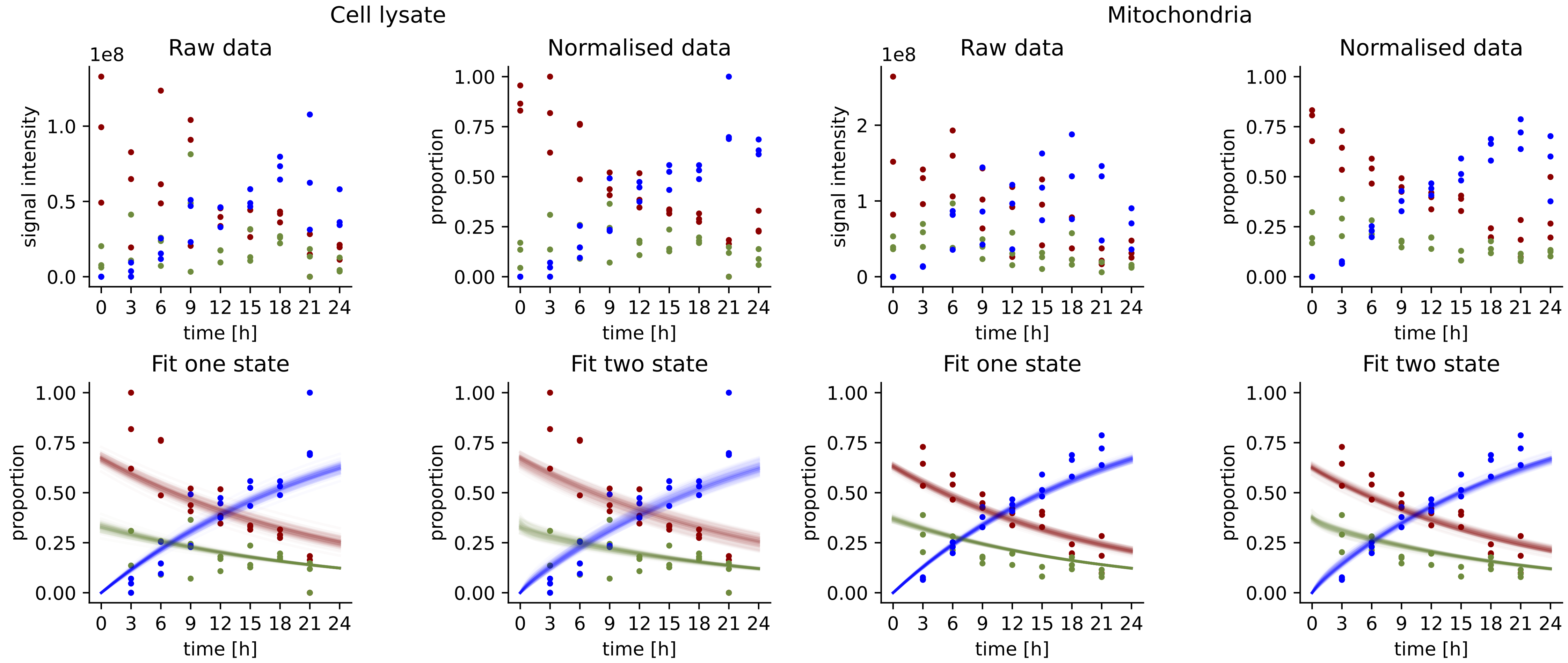

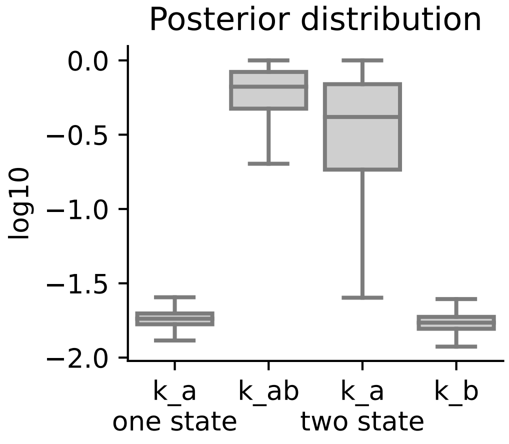

p value	8.63E-01
log likelihood one state	40.4
log likelihood two state	40.547
rmse one state	0.493
rmse two state	0.498
k_a one state	0.018
k_ab	0.665
k_a two state	0.416
k_b	0.017
sigma one state	0.128
sigma two state	0.129

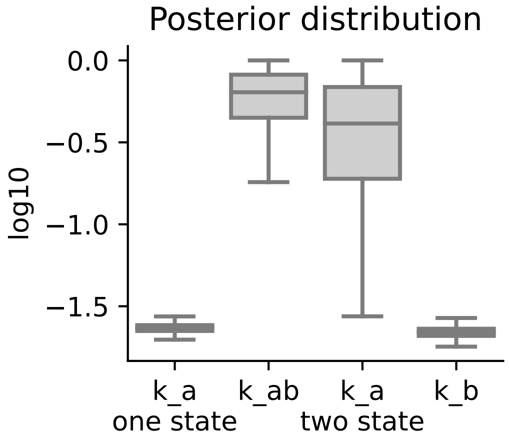

p value	3.06E-01
log likelihood one state	78.388
log likelihood two state	79.571
rmse one state	0.461
rmse two state	0.443
k_a one state	0.023
k_ab	0.639
k_a two state	0.412
k_b	0.022
sigma one state	0.071
sigma two state	0.071

RPUSD3
RPUSD3
Q6P087
RNA pseudouridylate synthase domain-containing protein 3

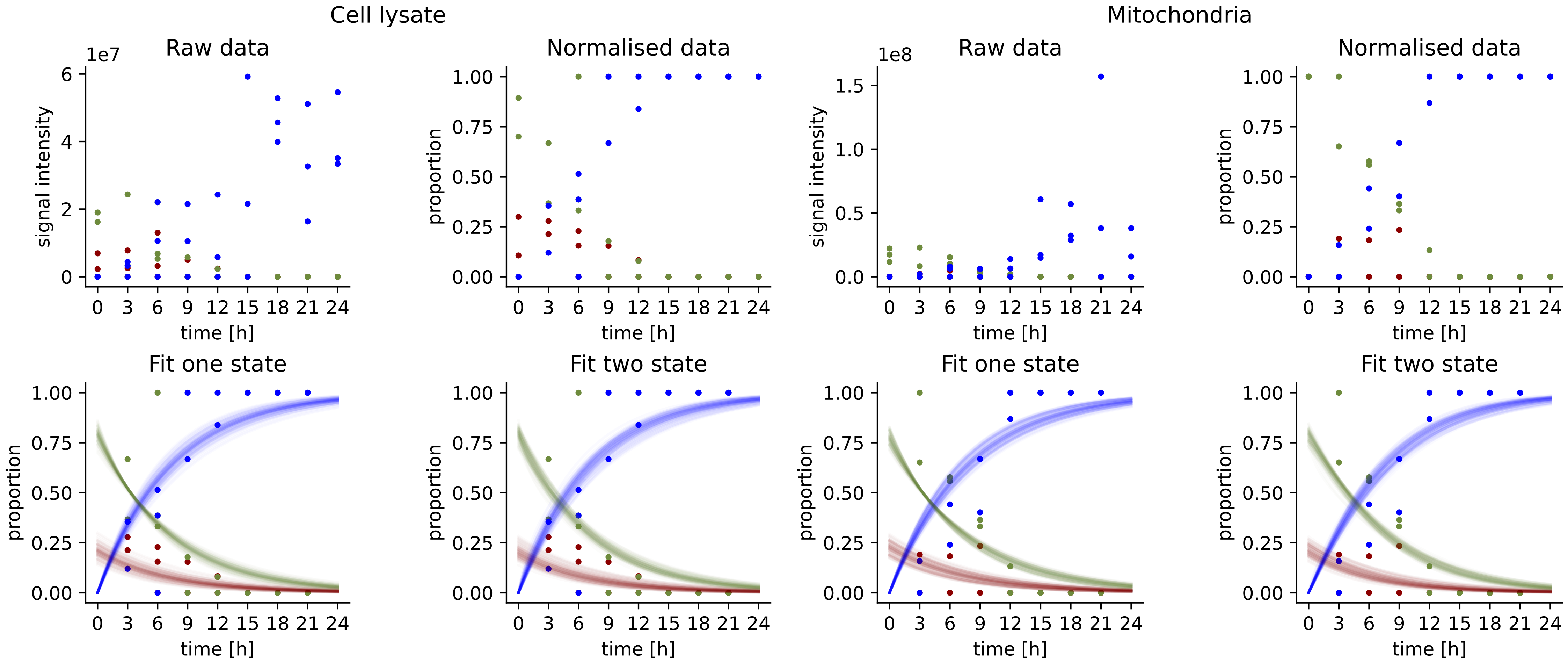

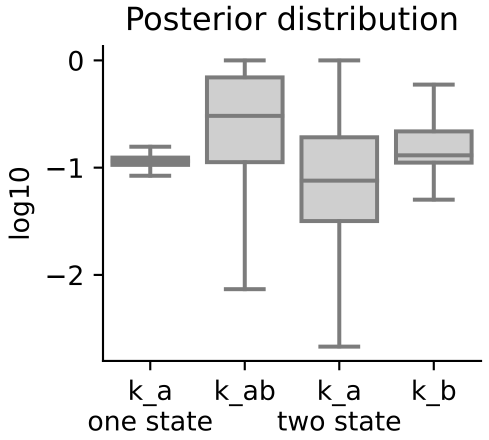

p value	1.62E-01
log likelihood one state	22.118
log likelihood two state	23.94
rmse one state	0.712
rmse two state	0.759
k_a one state	0.115
k_ab	0.304
k_a two state	0.076
k_b	0.13
sigma one state	0.157
sigma two state	0.157

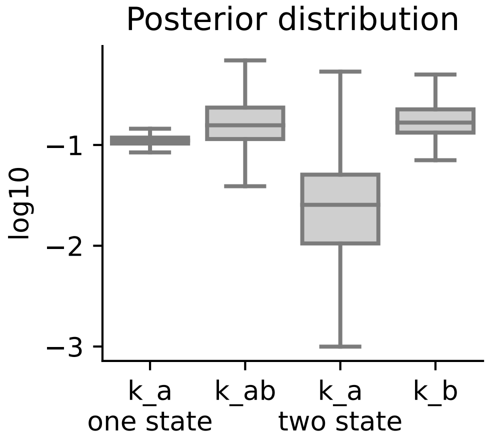

p value	7.55E-03
log likelihood one state	24.95
log likelihood two state	29.837
rmse one state	0.701
rmse two state	0.741
k_a one state	0.111
k_ab	0.157
k_a two state	0.025
k_b	0.167
sigma one state	0.145
sigma two state	0.137

PTCD1
PTCD1
O75127
Pentatricopeptide repeat-containing protein 1, mitochondrial

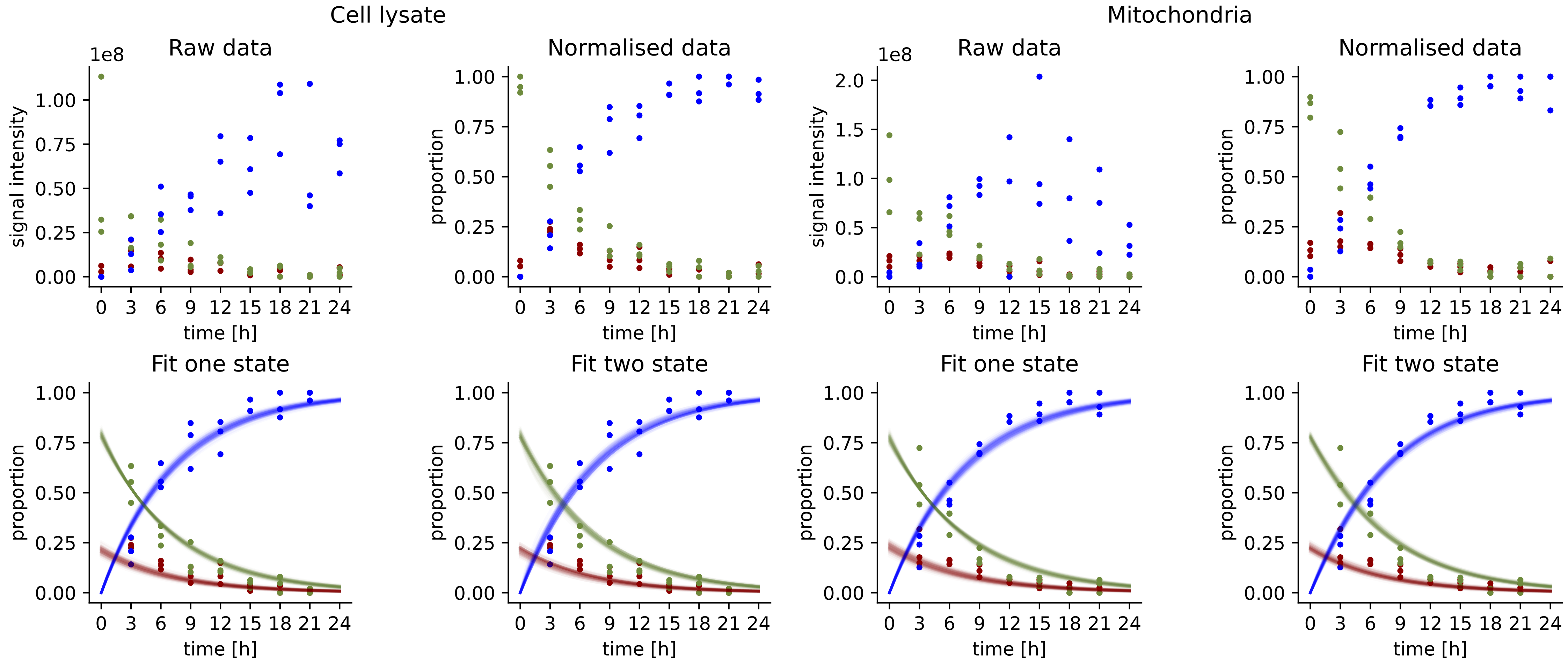

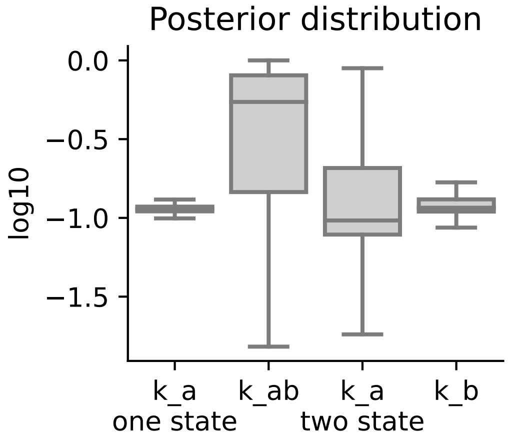

p value	7.03E-01
log likelihood one state	80.077
log likelihood two state	80.43
rmse one state	0.627
rmse two state	0.64
k_a one state	0.114
k_ab	0.545
k_a two state	0.096
k_b	0.116
sigma one state	0.069
sigma two state	0.068

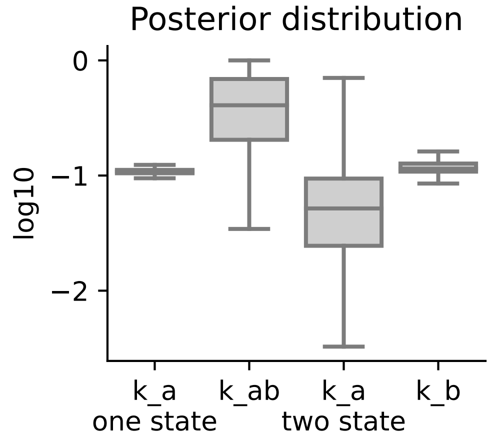

p value	1.52E-01
log likelihood one state	77.618
log likelihood two state	79.5
rmse one state	0.624
rmse two state	0.651
k_a one state	0.108
k_ab	0.408
k_a two state	0.052
k_b	0.115
sigma one state	0.068
sigma two state	0.067

TRUB2
TRUB2
O95900
Probable tRNA pseudouridine synthase 2

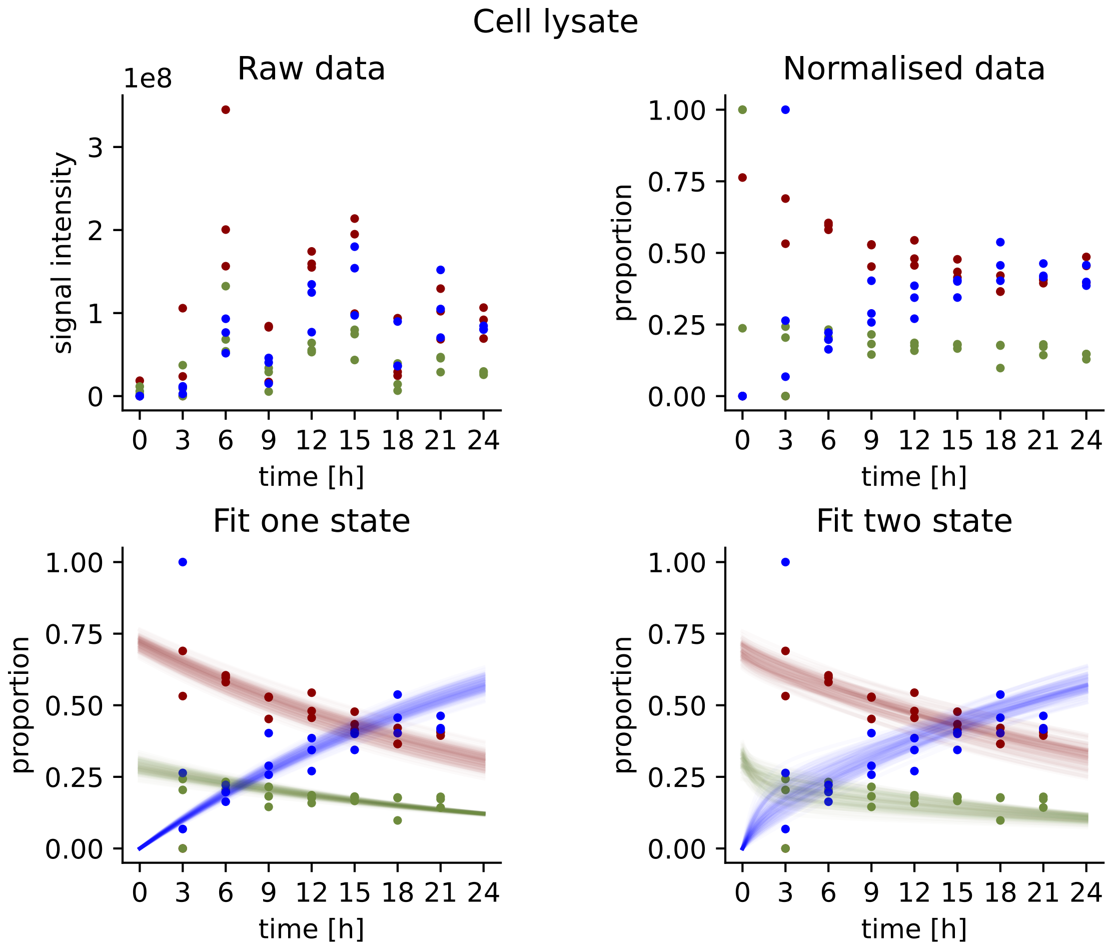

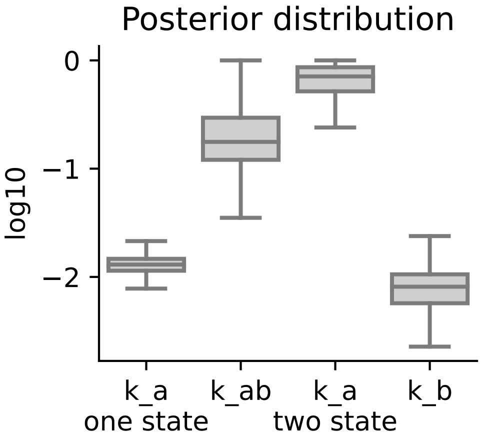

p value	6.65E-03
log likelihood one state	29.534
log likelihood two state	34.546
rmse one state	0.405
rmse two state	0.301
k_a one state	0.013
k_ab	0.177
k_a two state	0.711
k_b	0.008
sigma one state	0.152
sigma two state	0.144

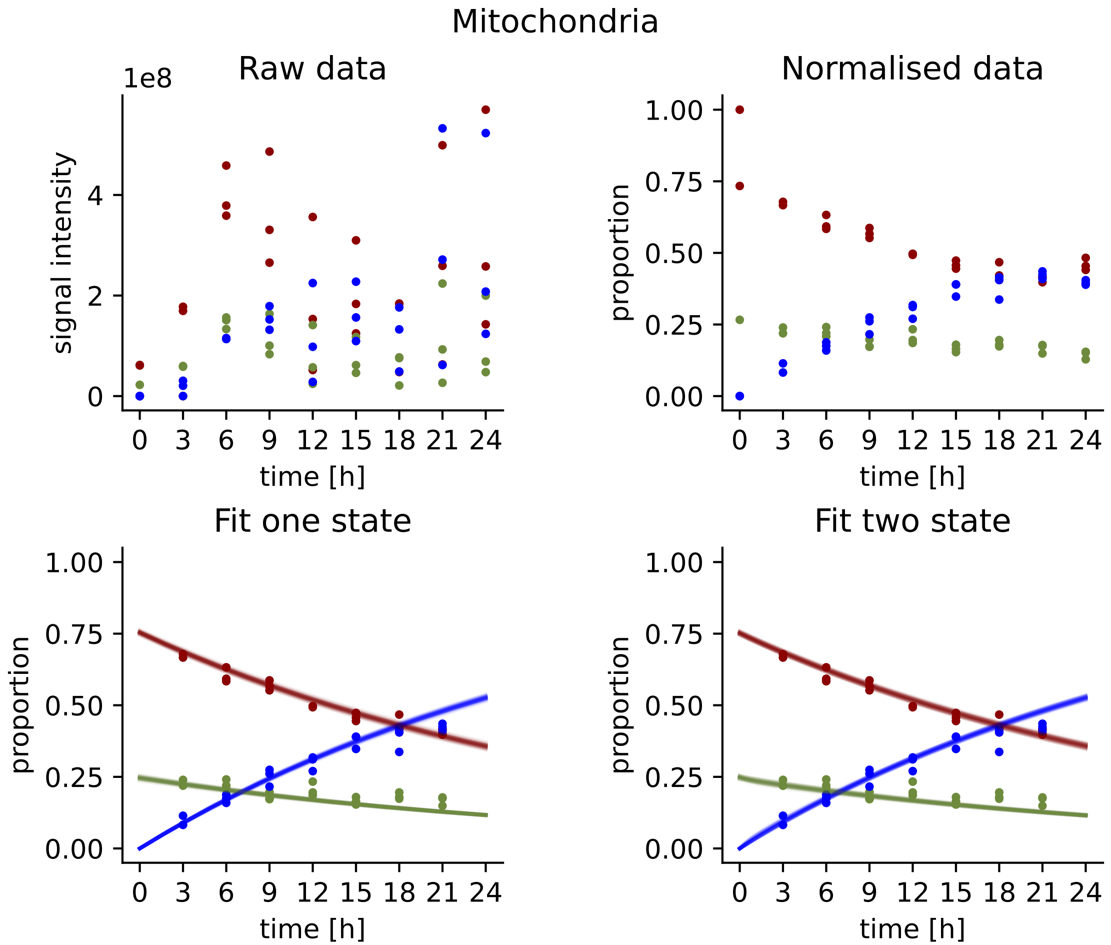

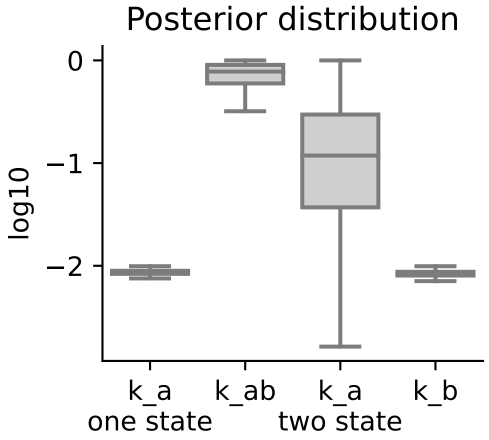

p value	8.88E-02
log likelihood one state	125.212
log likelihood two state	127.633
rmse one state	0.307
rmse two state	0.311
k_a one state	0.009
k_ab	0.778
k_a two state	0.118
k_b	0.008
sigma one state	0.031
sigma two state	0.031

DHX30
DHX30
Q7L2E3
Putative ATP-dependent RNA helicase DHX30

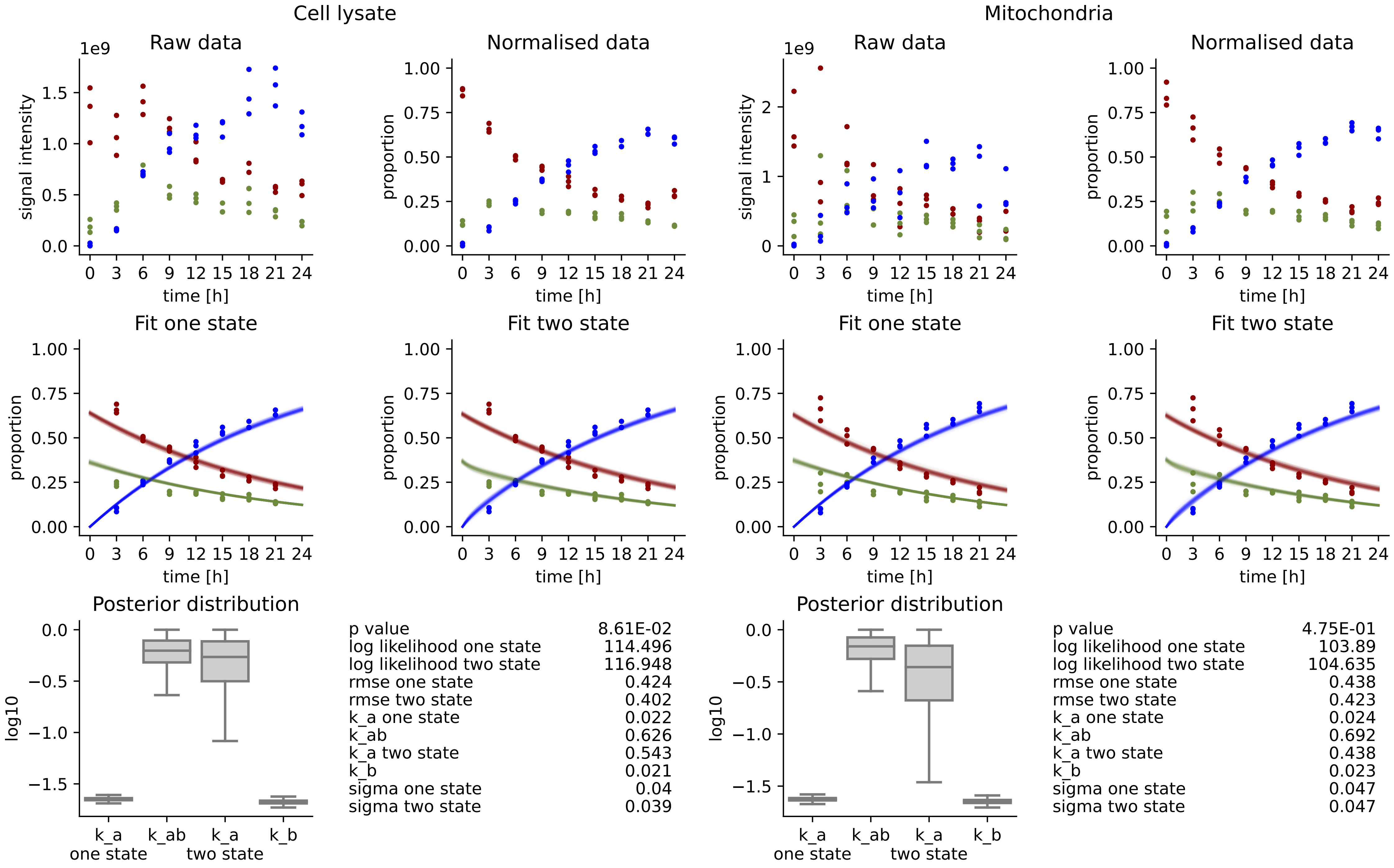

MPV17L2
MPV17L2
Q567V2
Mpv17-like protein 2

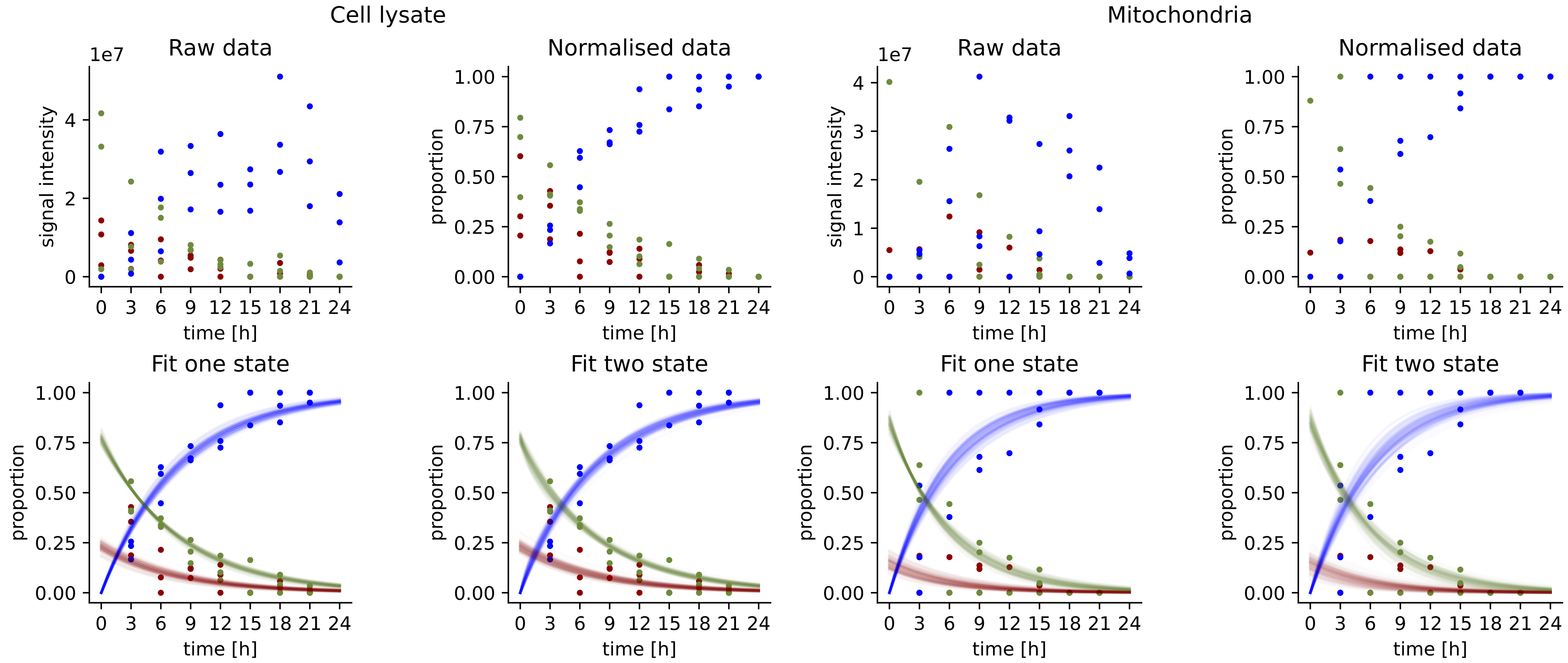

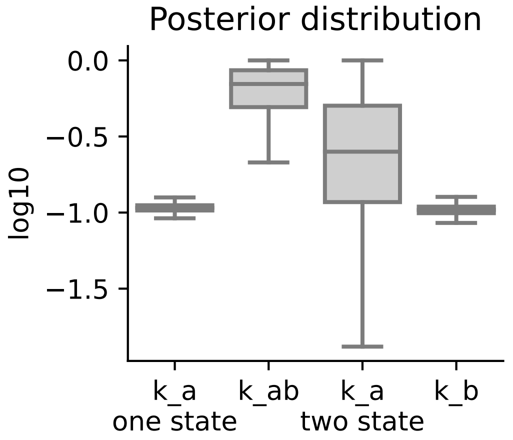

p value	9.63E-01
log likelihood one state	69.904
log likelihood two state	69.941
rmse one state	0.633
rmse two state	0.624
k_a one state	0.107
k_ab	0.7
k_a two state	0.251
k_b	0.104
sigma one state	0.081
sigma two state	0.082

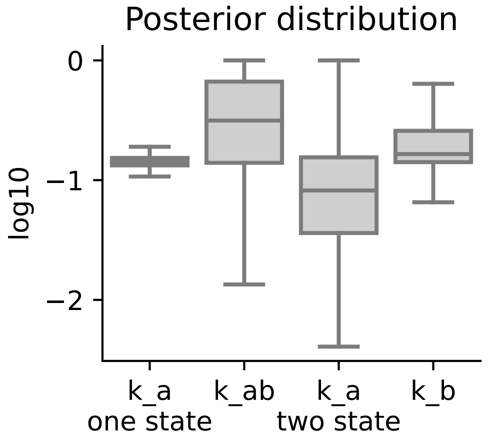

p value	2.09E-01
log likelihood one state	31.906
log likelihood two state	33.472
rmse one state	0.666
rmse two state	0.713
k_a one state	0.143
k_ab	0.314
k_a two state	0.082
k_b	0.165
sigma one state	0.139
sigma two state	0.139

SLIRP
SLIRP
Q9GZT3
SRA stem-loop-interacting RNA-binding protein, mitochondrial

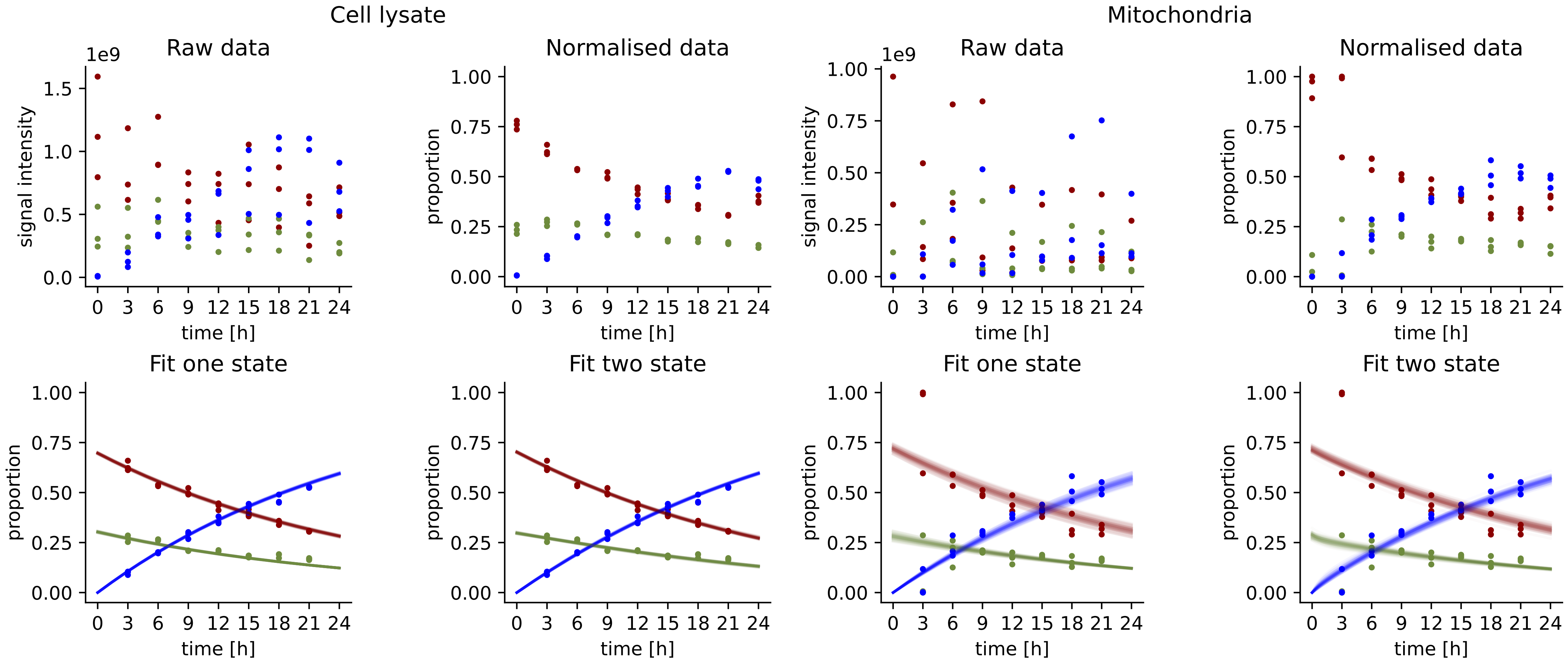

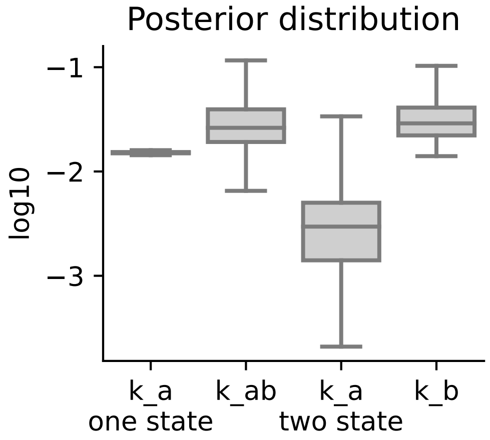

p value	2.04E-04
log likelihood one state	160.555
log likelihood two state	169.054
rmse one state	0.344
rmse two state	0.353
k_a one state	0.015
k_ab	0.026
k_a two state	0.003
k_b	0.029
sigma one state	0.019
sigma two state	0.017

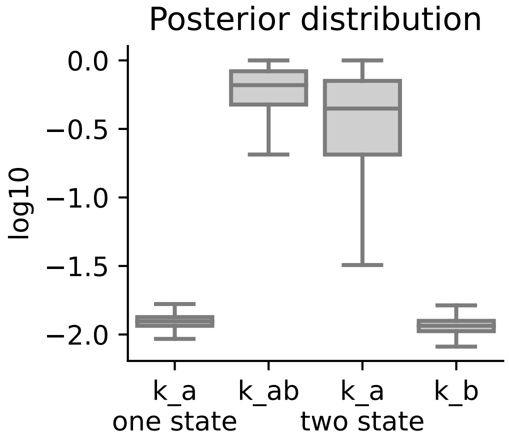

p value	9.15E-01
log likelihood one state	64.456
log likelihood two state	64.544
rmse one state	0.399
rmse two state	0.401
k_a one state	0.012
k_ab	0.66
k_a two state	0.445
k_b	0.012
sigma one state	0.088
sigma two state	0.088

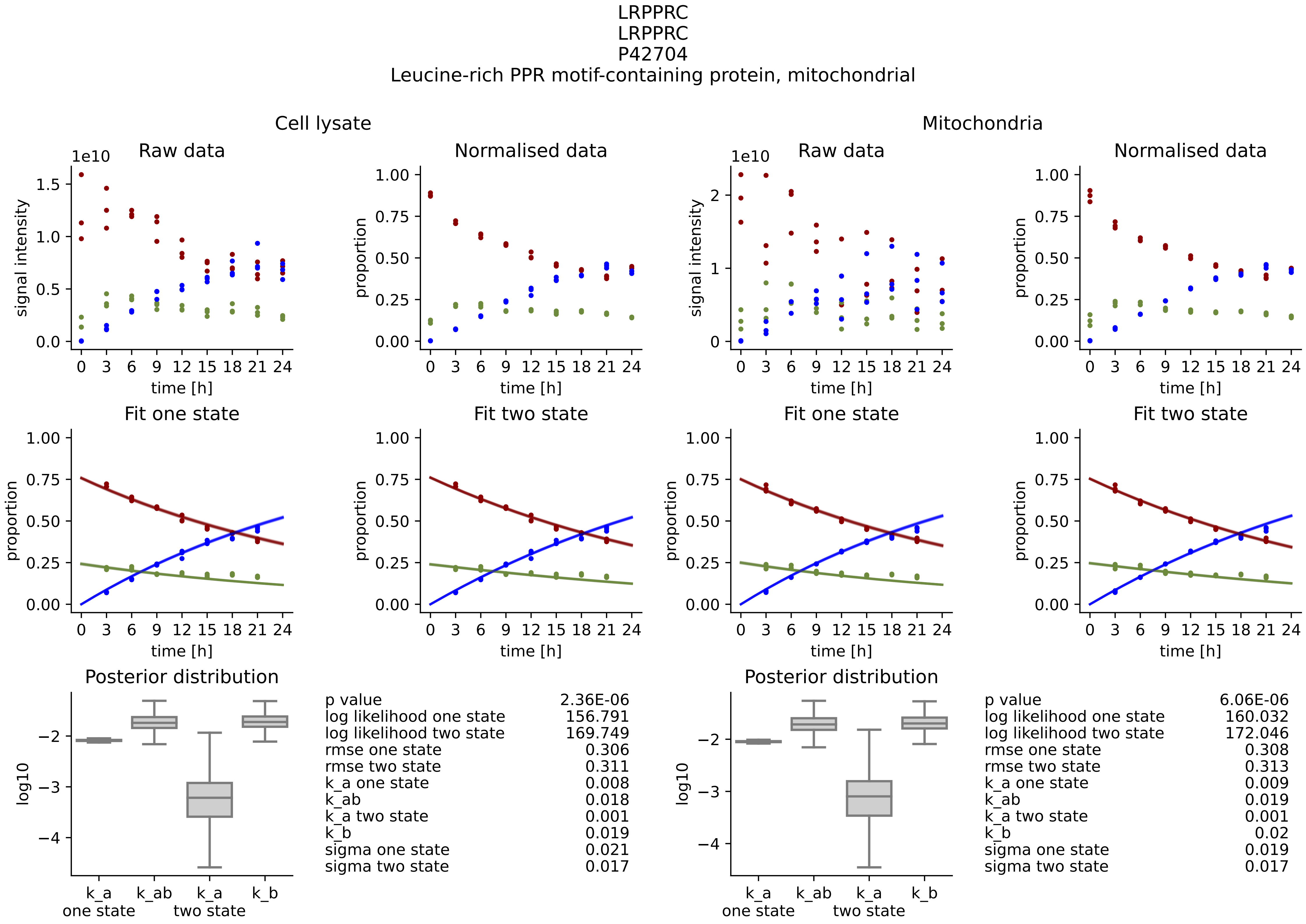
LRPPRC
LRPPRC
P42704
Leucine-rich PPR motif-containing protein, mitochondrial
Cell lysate
Mitochondria
Raw data
Normalised data
Raw data
Normalised data
1e10
signal intensity
proportion
time [h]
Fit one state
Fit two state
Fit one state
Fit two state
Posterior distribution
log10
k_a
one state
k_ab
k_a
two state
k_b
p value 2.36E-06
log likelihood one state 156.791
log likelihood two state 169.749
rmse one state 0.306
rmse two state 0.311
k_a one state 0.008
k_ab 0.018
k_a two state 0.001
k_b 0.019
sigma one state 0.021
sigma two state 0.017
Posterior distribution
p value 6.06E-06
log likelihood one state 160.032
log likelihood two state 172.046
rmse one state 0.308
rmse two state 0.313
k_a one state 0.009
k_ab 0.019
k_a two state 0.001
k_b 0.02
sigma one state 0.019
sigma two state 0.017

GRSF1
GRSF1
Q12849
G-rich sequence factor 1

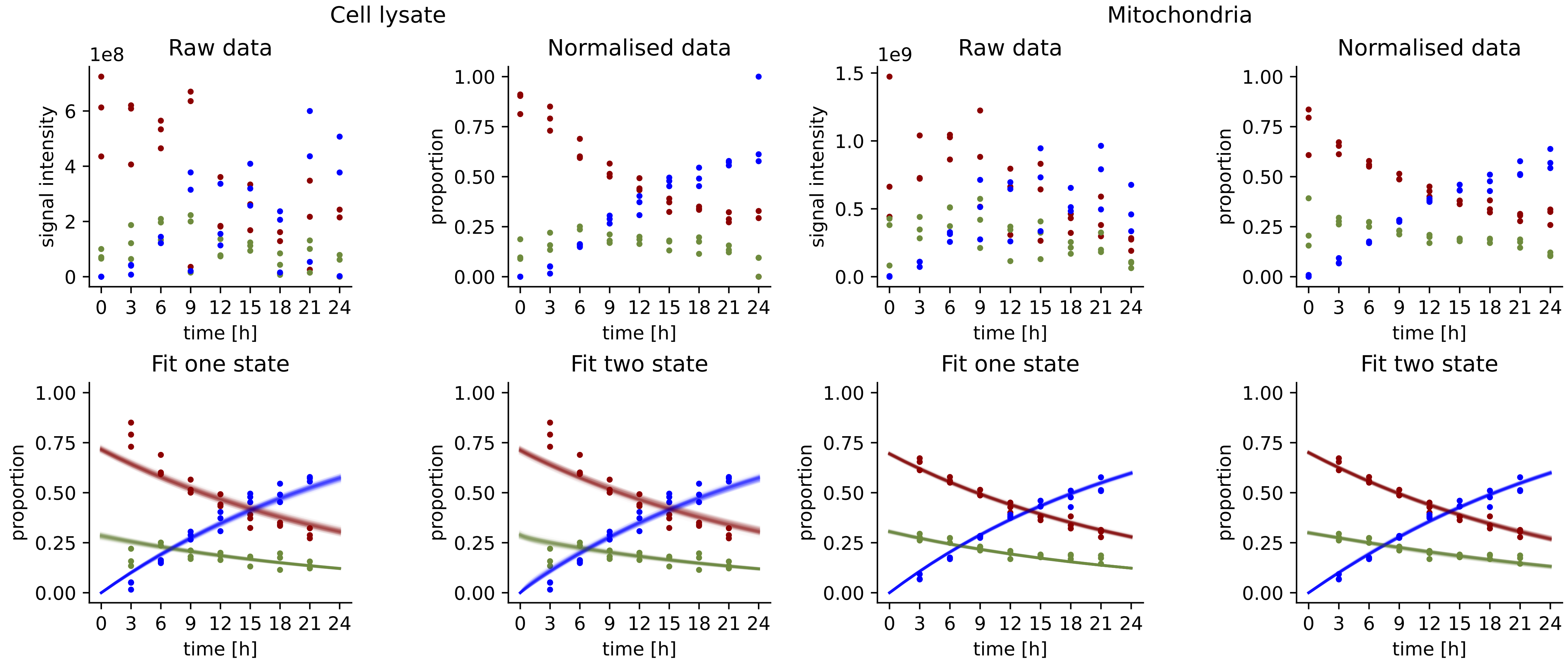

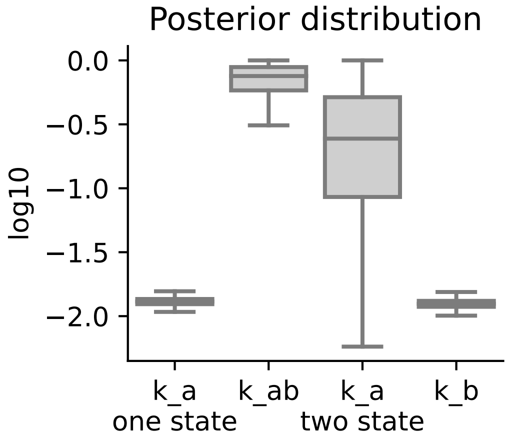

p value	5.86E-01
log likelihood one state	91.516
log likelihood two state	92.051
rmse one state	0.388
rmse two state	0.392
k_a one state	0.013
k_ab	0.754
k_a two state	0.244
k_b	0.012
sigma one state	0.058
sigma two state	0.058

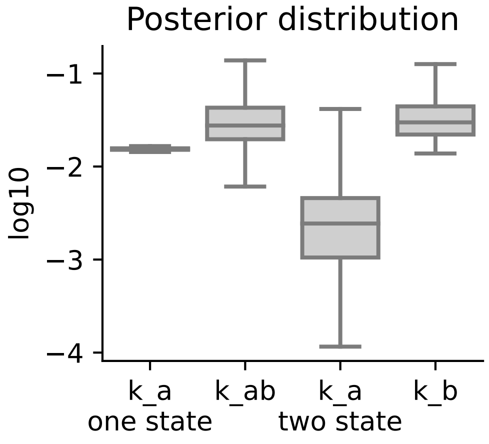

p value	3.08E-04
log likelihood one state	143.201
log likelihood two state	151.286
rmse one state	0.353
rmse two state	0.362
k_a one state	0.015
k_ab	0.028
k_a two state	0.002
k_b	0.03
sigma one state	0.025
sigma two state	0.023

ELAC2
ELAC2
Q9BQ52
Zinc phosphodiesterase ELAC protein 2

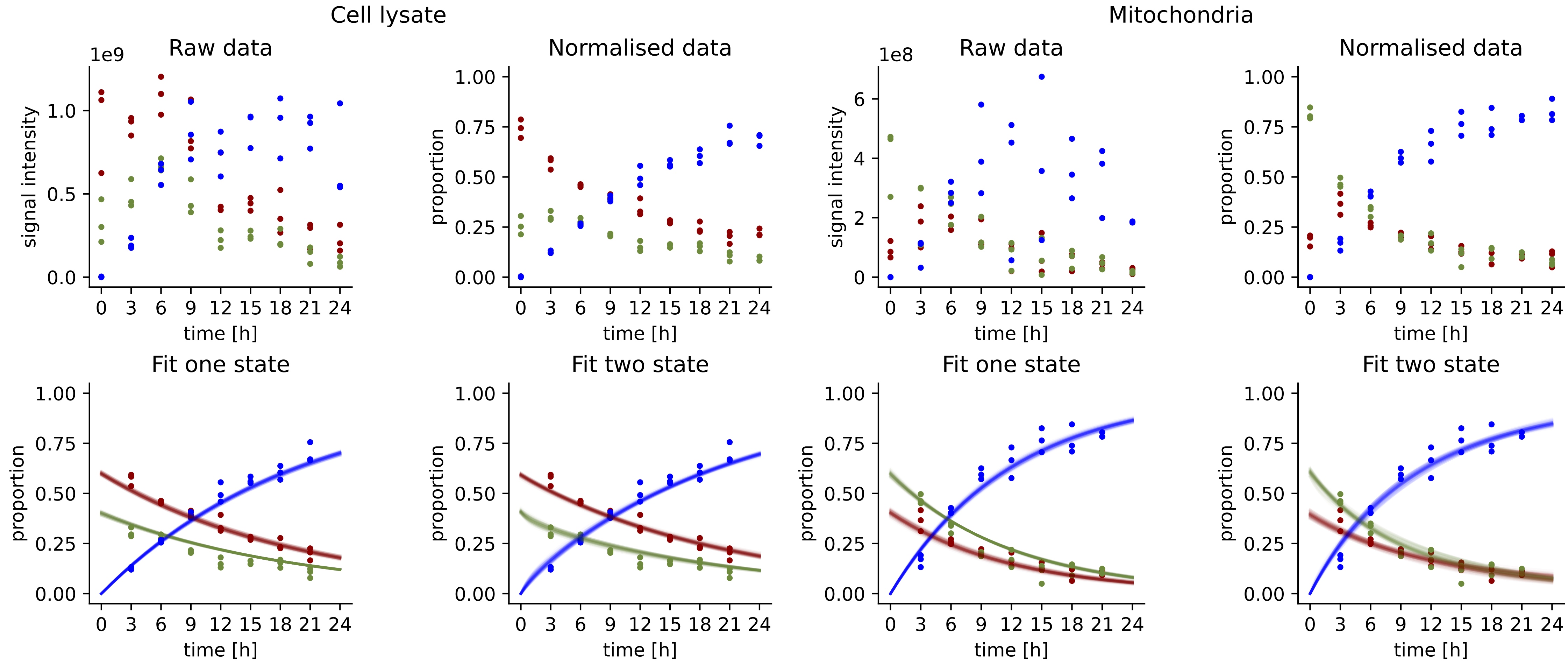

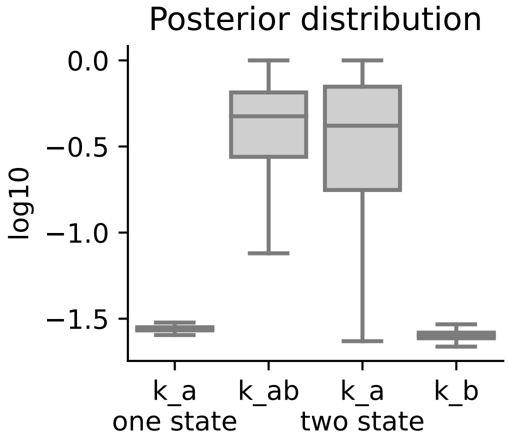

p value	1.32E-03
log likelihood one state	116.182
log likelihood two state	122.809
rmse one state	0.447
rmse two state	0.428
k_a one state	0.028
k_ab	0.474
k_a two state	0.417
k_b	0.025
sigma one state	0.039
sigma two state	0.037

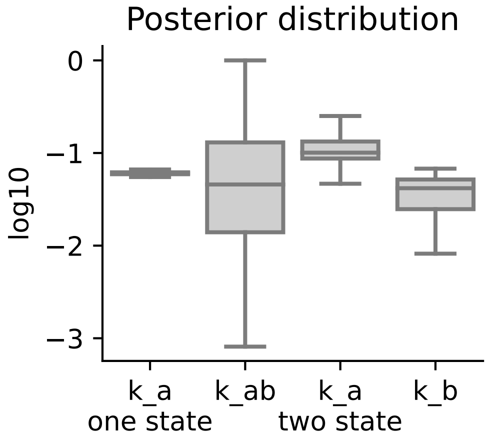

p value	1.26E-04
log likelihood one state	99.426
log likelihood two state	108.405
rmse one state	0.537
rmse two state	0.51
k_a one state	0.06
k_ab	0.046
k_a two state	0.101
k_b	0.042
sigma one state	0.051
sigma two state	0.047

MRPP1
TRMT10C
Q7L0Y3
Mitochondrial ribonuclease P protein 1

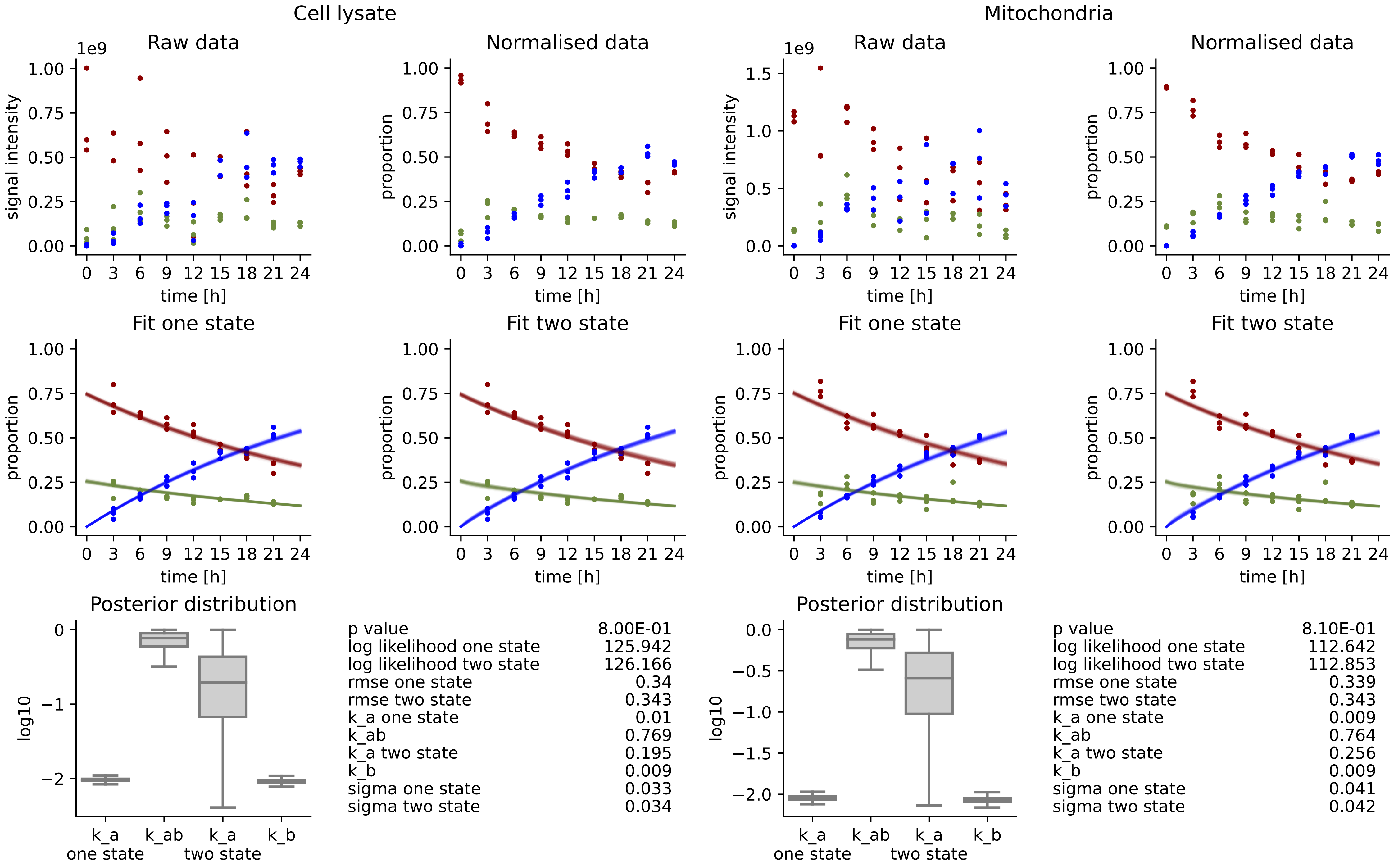

MRPP2
HSD17B10
Q99714
3-hydroxyacyl-CoA dehydrogenase type-2

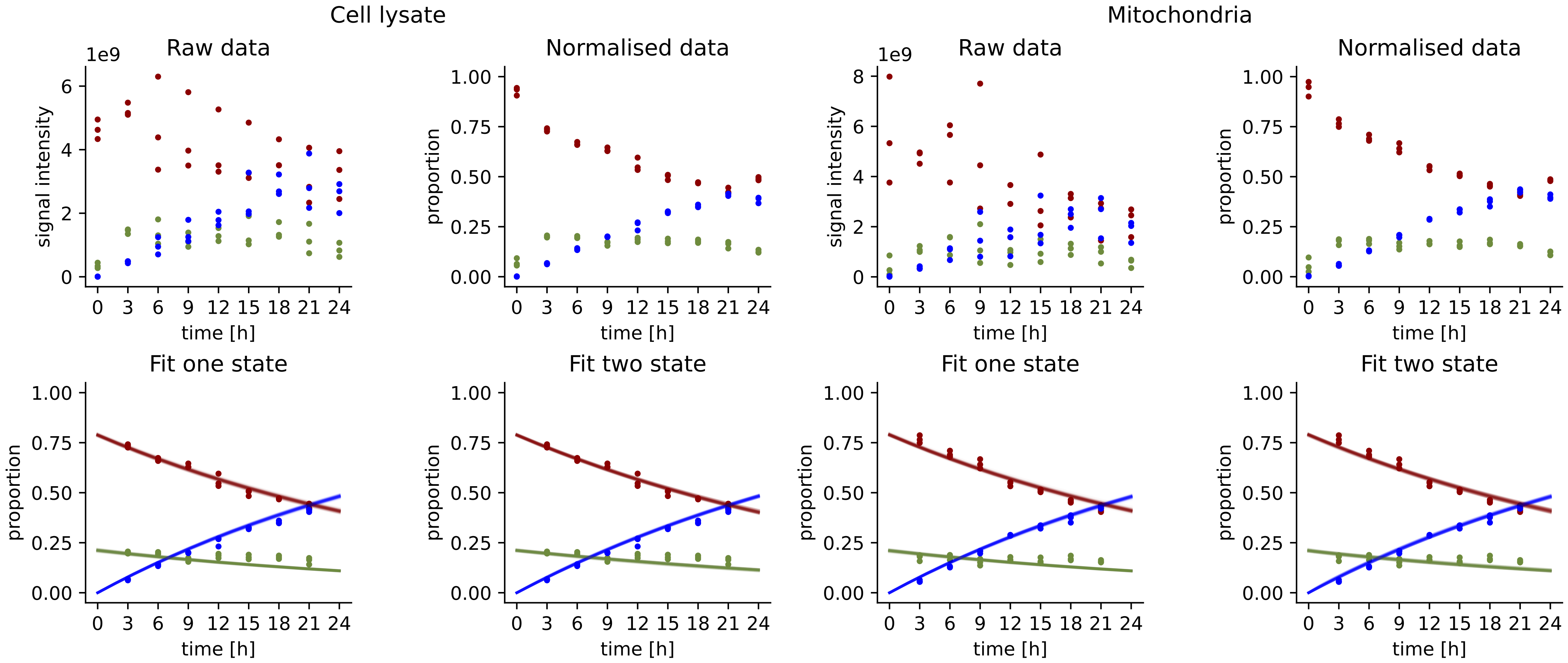

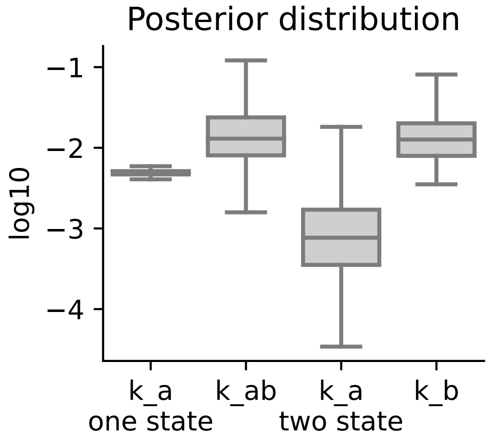

p value	2.47E-04
log likelihood one state	141.625
log likelihood two state	149.931
rmse one state	0.279
rmse two state	0.281
k_a one state	0.005
k_ab	0.013
k_a two state	0.001
k_b	0.013
sigma one state	0.026
sigma two state	0.025

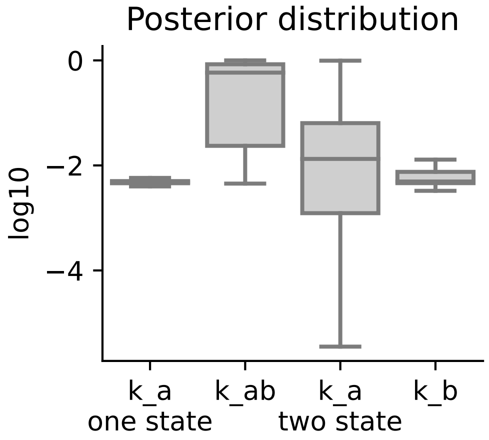

p value	2.76E-03
log likelihood one state	142.388
log likelihood two state	148.281
rmse one state	0.294
rmse two state	0.297
k_a one state	0.005
k_ab	0.585
k_a two state	0.013
k_b	0.005
sigma one state	0.026
sigma two state	0.025

MRPP3
KIAA0391
O15091
Mitochondrial ribonuclease P protein 3

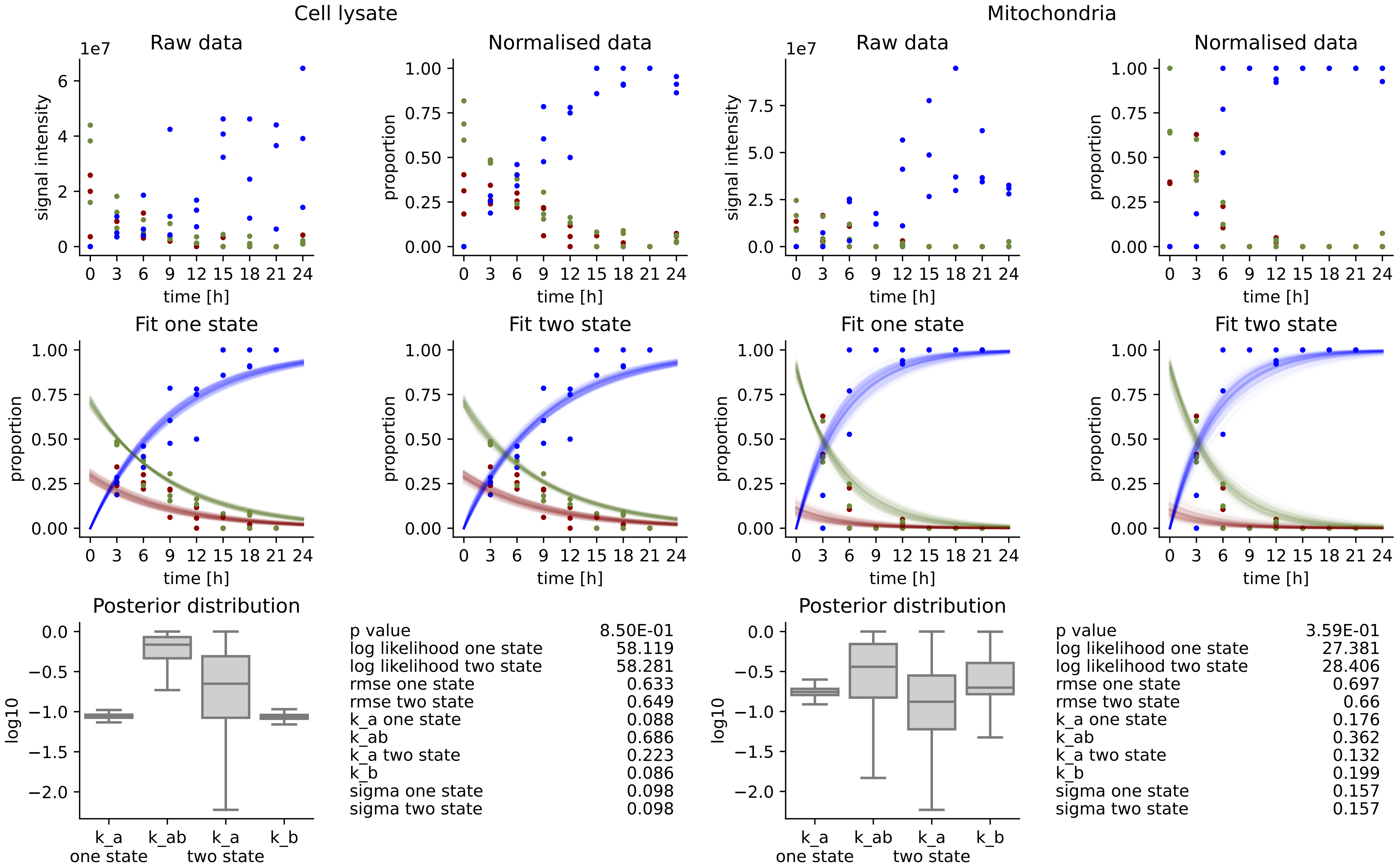

LONP1
LONP1
P36776
Lon protease homolog, mitochondrial

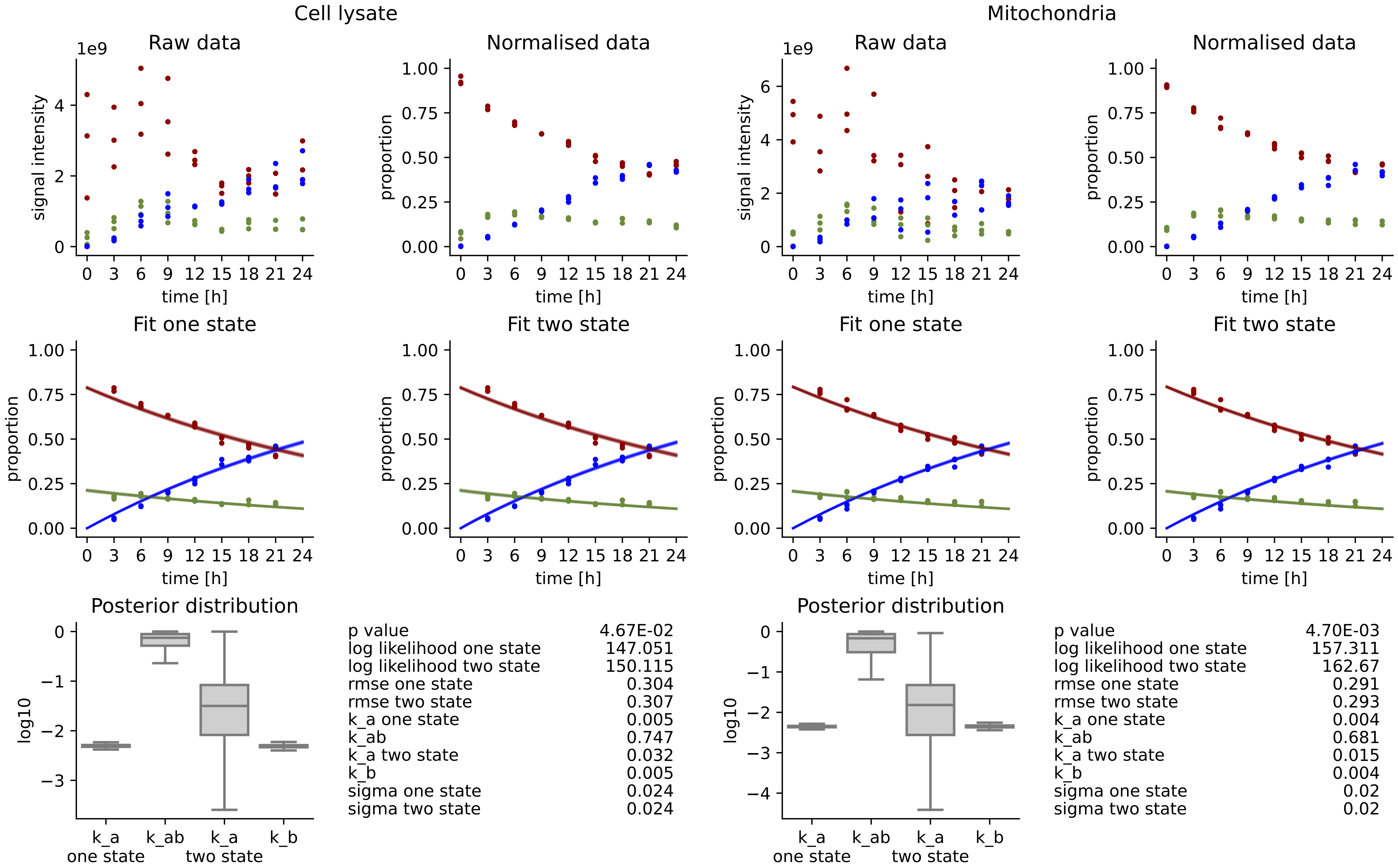

Cell lysate

p value	4.67E-02
log likelihood one state	147.051
log likelihood two state	150.115
rmse one state	0.304
rmse two state	0.307
k_a one state	0.005
k_ab	0.747
k_a two state	0.032
k_b	0.005
sigma one state	0.024
sigma two state	0.024

Mitochondria

p value	4.70E-03
log likelihood one state	157.311
log likelihood two state	162.67
rmse one state	0.291
rmse two state	0.293
k_a one state	0.004
k_ab	0.681
k_a two state	0.015
k_b	0.004
sigma one state	0.02
sigma two state	0.02

C1QBP
C1QBP
Q07021
Complement component 1 Q subcomponent-binding protein, mitochondrial

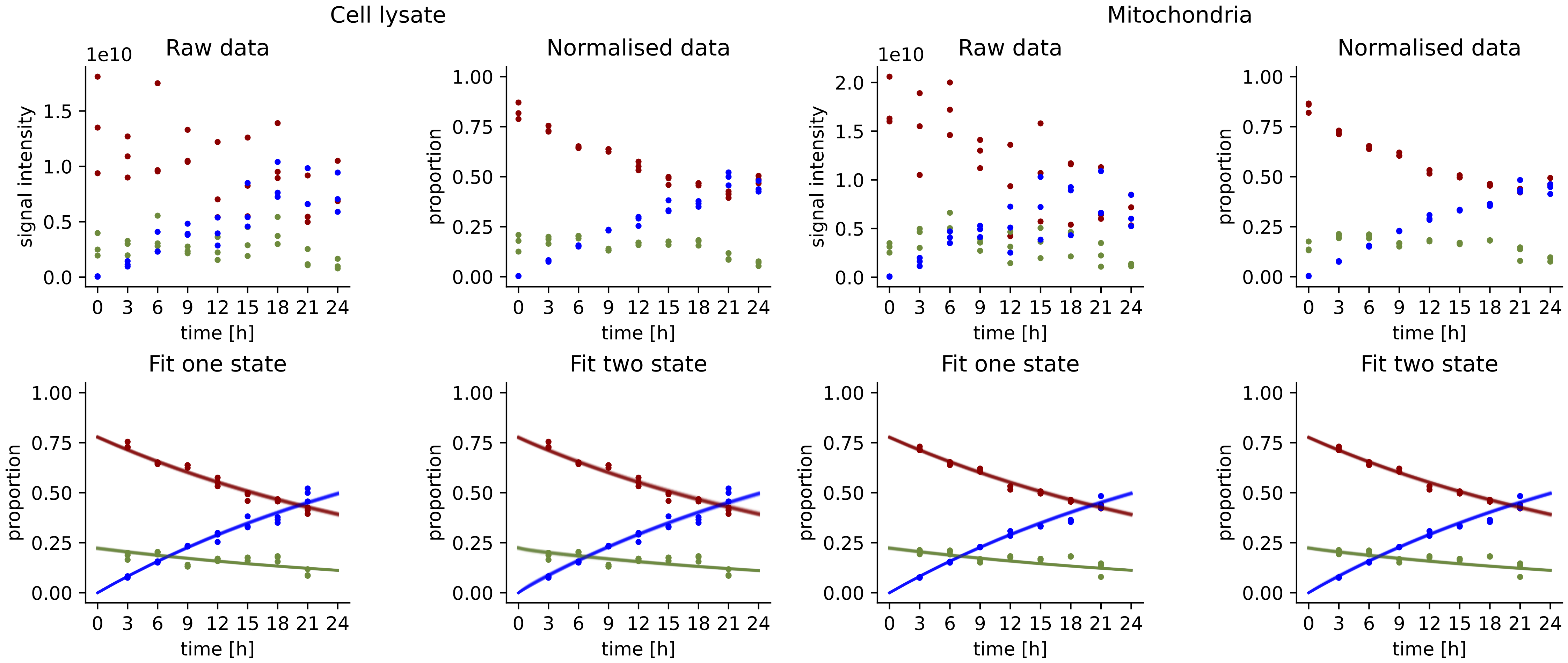

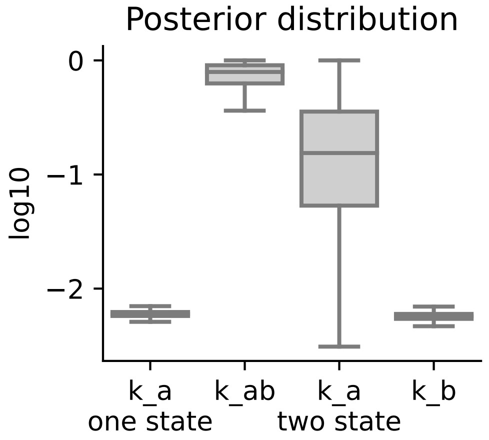

p value	4.02E-01
log likelihood one state	140.717
log likelihood two state	141.627
rmse one state	0.306
rmse two state	0.31
k_a one state	0.006
k_ab	0.792
k_a two state	0.154
k_b	0.006
sigma one state	0.026
sigma two state	0.027

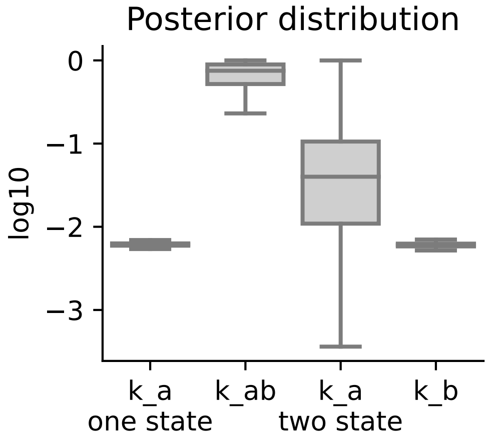

p value	3.64E-02
log likelihood one state	155.046
log likelihood two state	158.36
rmse one state	0.292
rmse two state	0.295
k_a one state	0.006
k_ab	0.751
k_a two state	0.04
k_b	0.006
sigma one state	0.021
sigma two state	0.021

CLPP
CLPP
Q16740
ATP-dependent Clp protease proteolytic subunit, mitochondrial

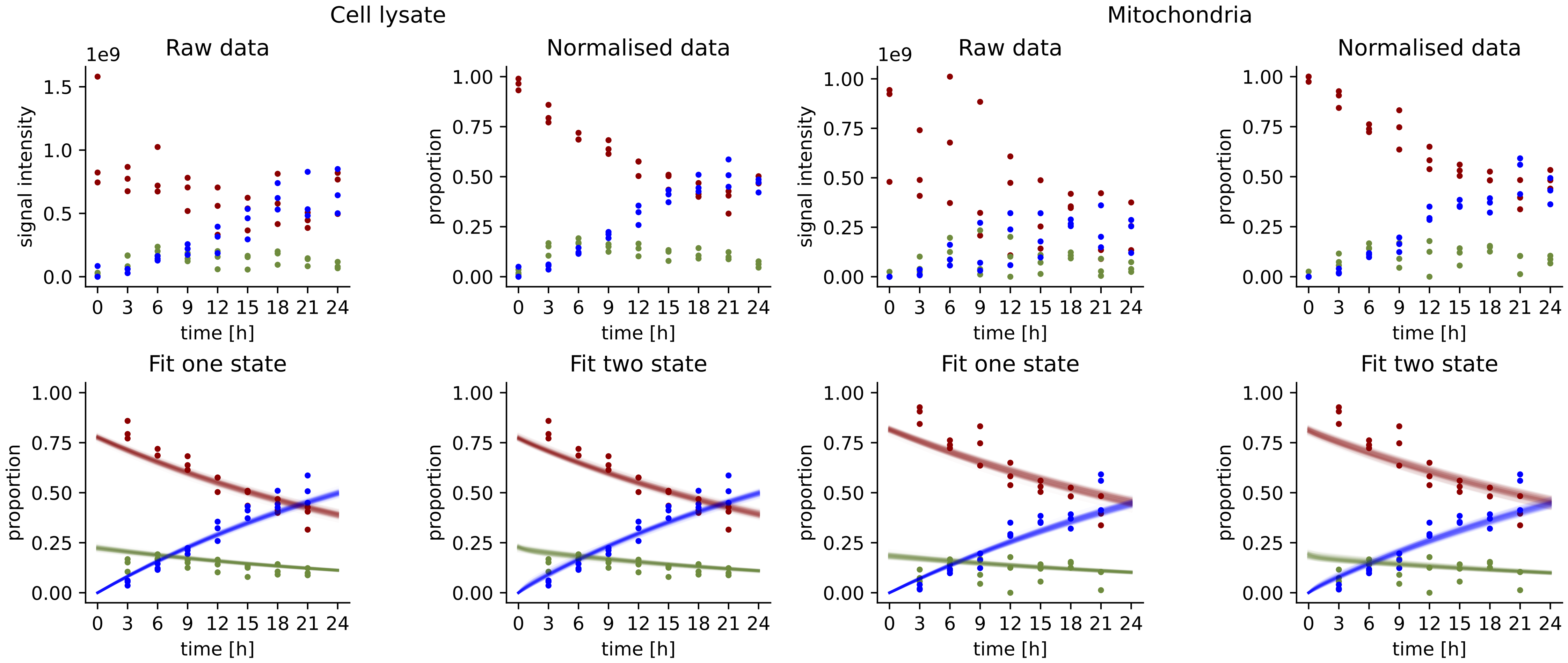

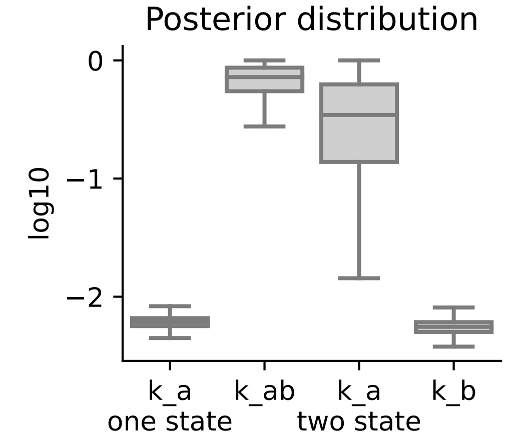

p value	9.32E-01
log likelihood one state	97.873
log likelihood two state	97.943
rmse one state	0.348
rmse two state	0.347
k_a one state	0.006
k_ab	0.722
k_a two state	0.345
k_b	0.006
sigma one state	0.052
sigma two state	0.052

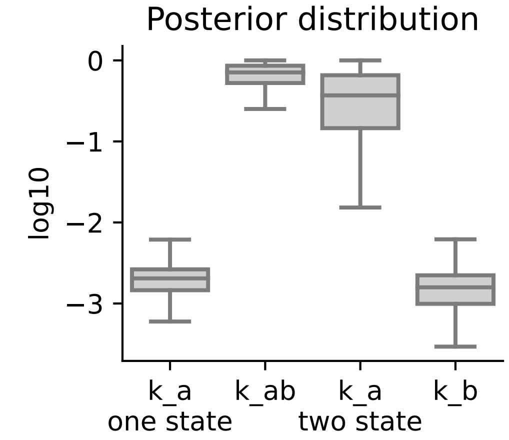

p value	9.13E-01
log likelihood one state	76.136
log likelihood two state	76.227
rmse one state	0.338
rmse two state	0.337
k_a one state	0.002
k_ab	0.71
k_a two state	0.37
k_b	0.002
sigma one state	0.074
sigma two state	0.074

RCC1LV1
WBSCR16
Q96I51
Williams-Beuren syndrome chromosomal region 16 protein

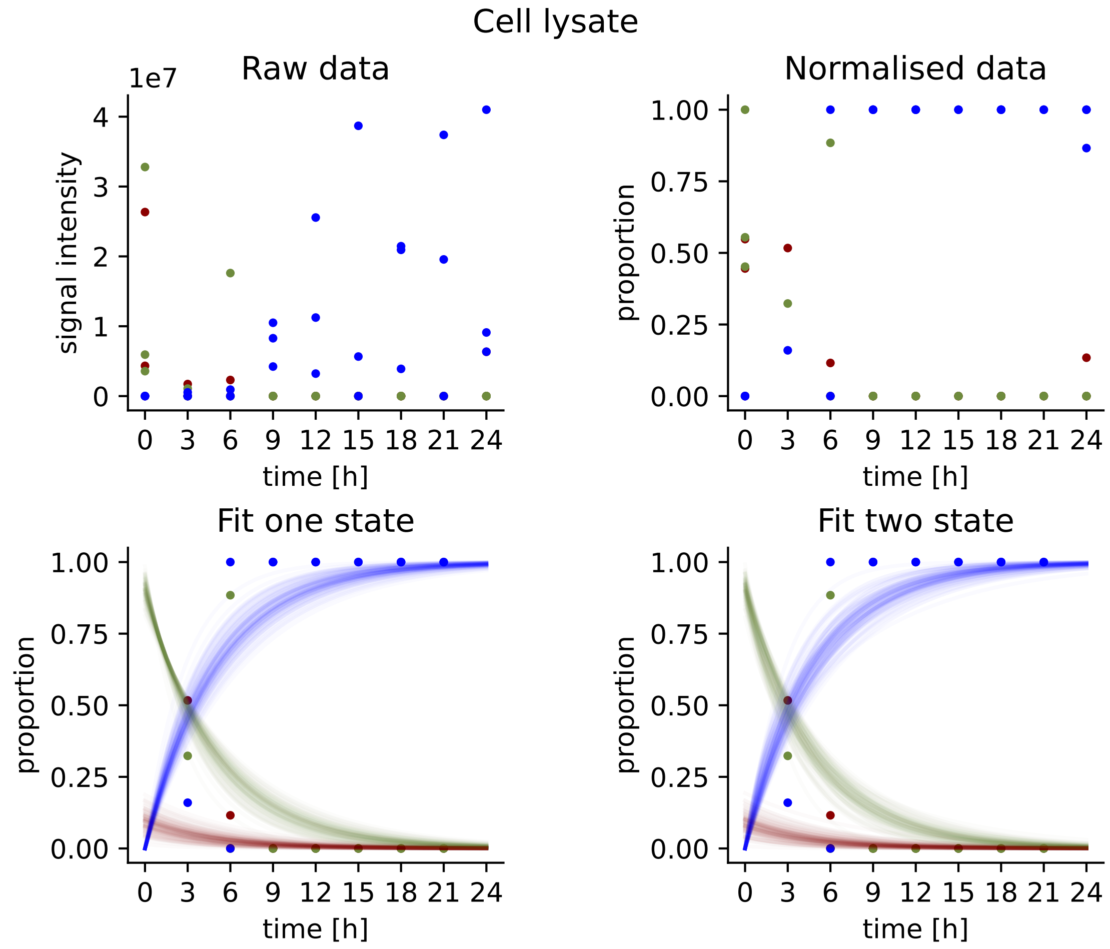

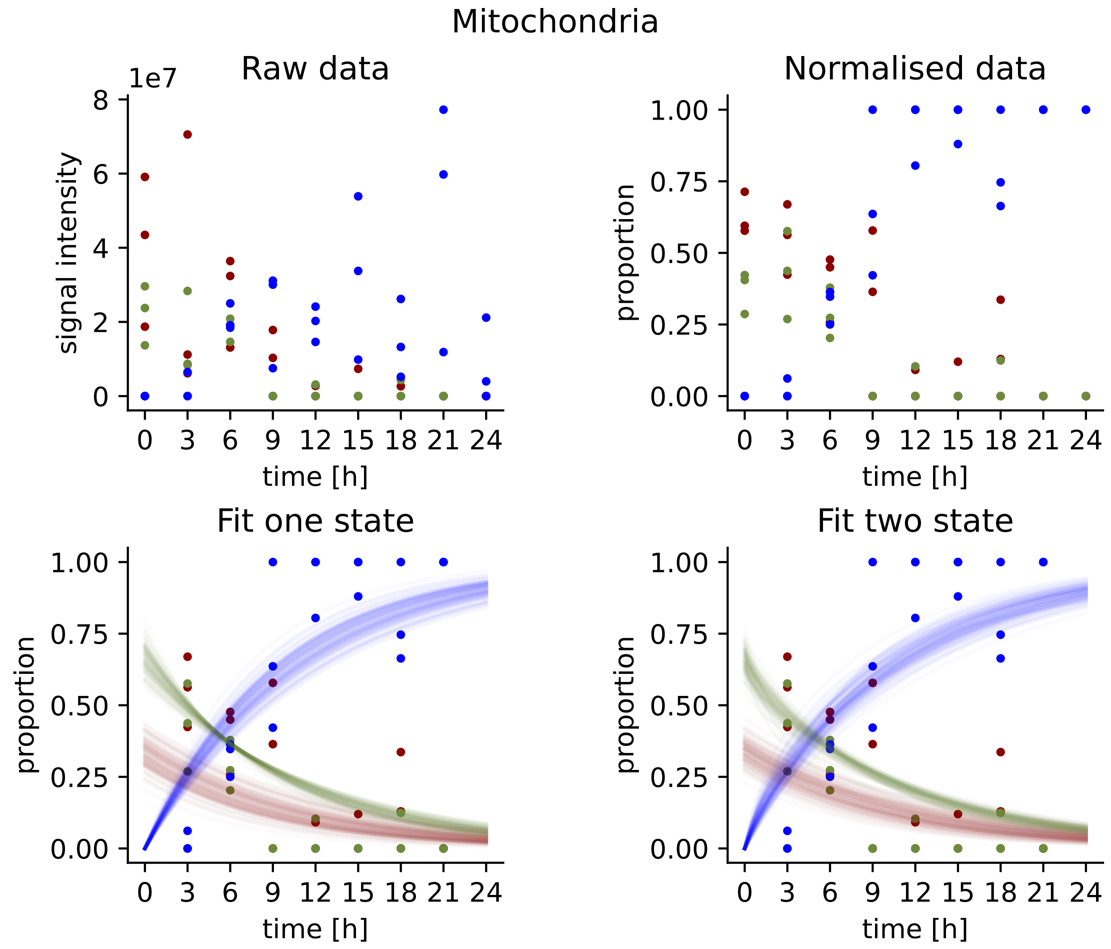

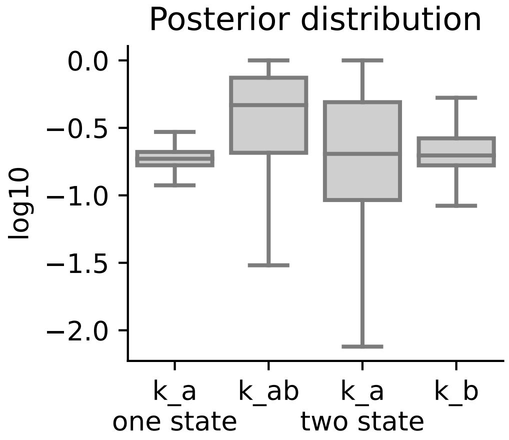

p value	5.97E-01
log likelihood one state	13.934
log likelihood two state	14.45
rmse one state	0.719
rmse two state	0.771
k_a one state	0.187
k_ab	0.466
k_a two state	0.203
k_b	0.198
sigma one state	0.18
sigma two state	0.181

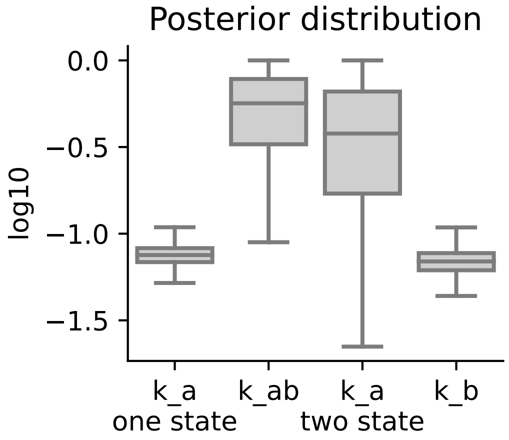

p value	1.32E-01
log likelihood one state	15.8
log likelihood two state	17.825
rmse one state	0.734
rmse two state	0.679
k_a one state	0.075
k_ab	0.565
k_a two state	0.378
k_b	0.069
sigma one state	0.187
sigma two state	0.187

YBEY
YBEY
P58557
Putative ribonuclease

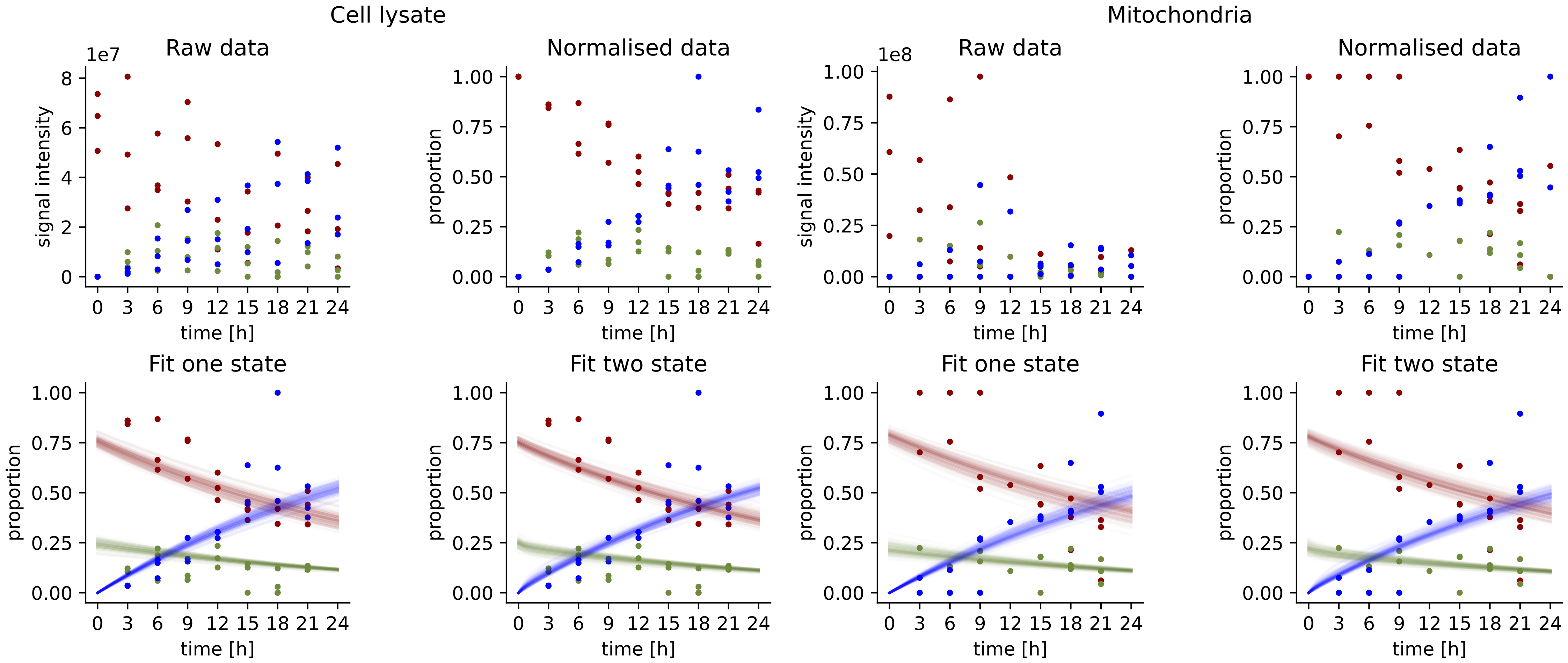

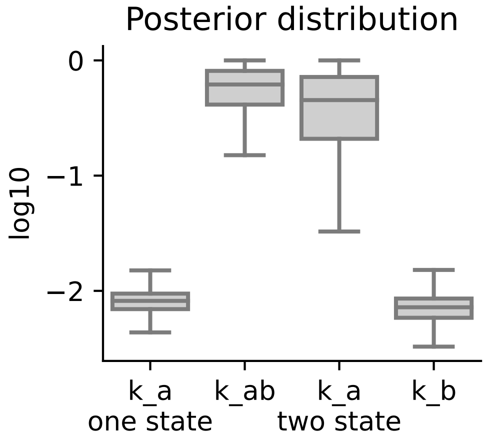

p value	8.52E-01
log likelihood one state	37.972
log likelihood two state	38.132
rmse one state	0.442
rmse two state	0.436
k_a one state	0.008
k_ab	0.618
k_a two state	0.452
k_b	0.007
sigma one state	0.133
sigma two state	0.133

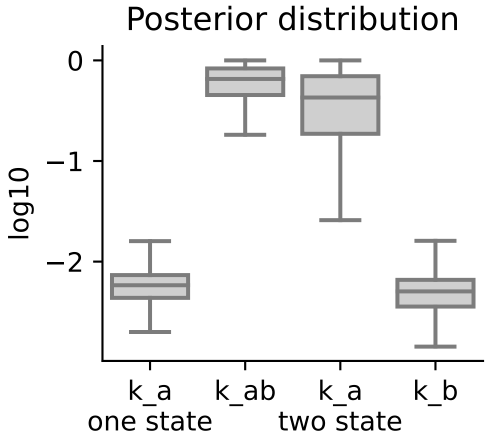

p value	9.67E-01
log likelihood one state	23.704
log likelihood two state	23.738
rmse one state	0.47
rmse two state	0.471
k_a one state	0.006
k_ab	0.654
k_a two state	0.427
k_b	0.005
sigma one state	0.156
sigma two state	0.157

GTPBP8
GTPBP8
Q8N3Z3
GTP-binding protein 8

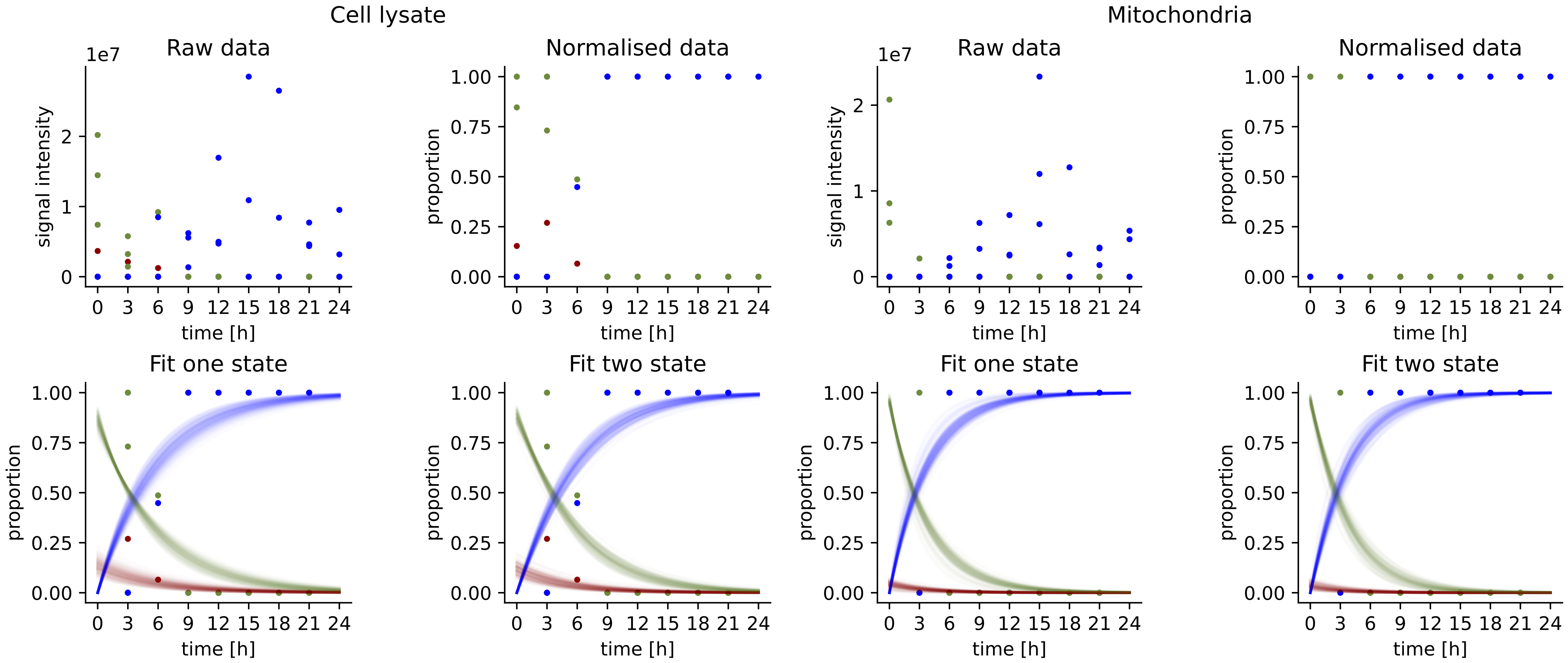

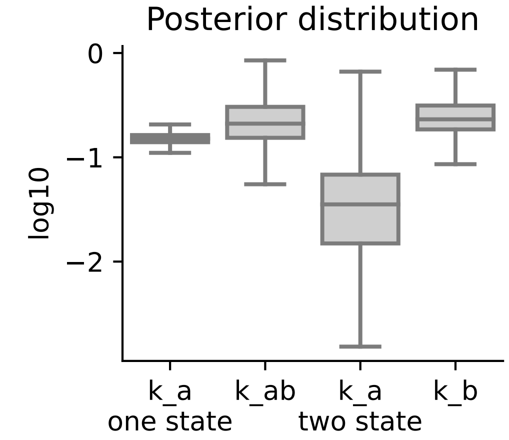

p value	1.13E-02
log likelihood one state	17.872
log likelihood two state	22.356
rmse one state	0.737
rmse two state	0.774
k_a one state	0.151
k_ab	0.21
k_a two state	0.035
k_b	0.231
sigma one state	0.17
sigma two state	0.162

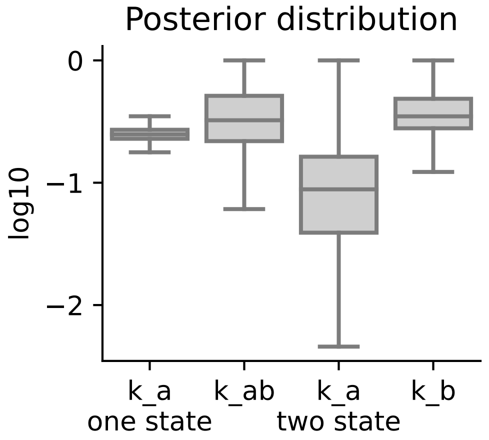

p value	6.23E-02
log likelihood one state	29.307
log likelihood two state	32.083
rmse one state	0.635
rmse two state	0.682
k_a one state	0.248
k_ab	0.324
k_a two state	0.088
k_b	0.349
sigma one state	0.133
sigma two state	0.129
