# Supplementary material for: A roadmap for ribosome assembly in human mitochondria
Source: Nat Struct Mol Biol. 2024 Jul 11;31(12):1898–908. doi: 10.1038/s41594-024-01356-w (PMC11638073; doi:10.1038/s41594-024-01356-w)
Supplement: Supplementary file 5 — Visualization of mtSSU MRP’s MS RAW data across sucrose gradient fractions. MS1 raw intensities before normalization for all H (red), M (green) and L (blue) labeled MRPs of the mtSSU over the chase time of 12 h for all collected 16 sucrose gradient fractions. [file 41594_2024_1356_MOESM5_ESM.pdf]

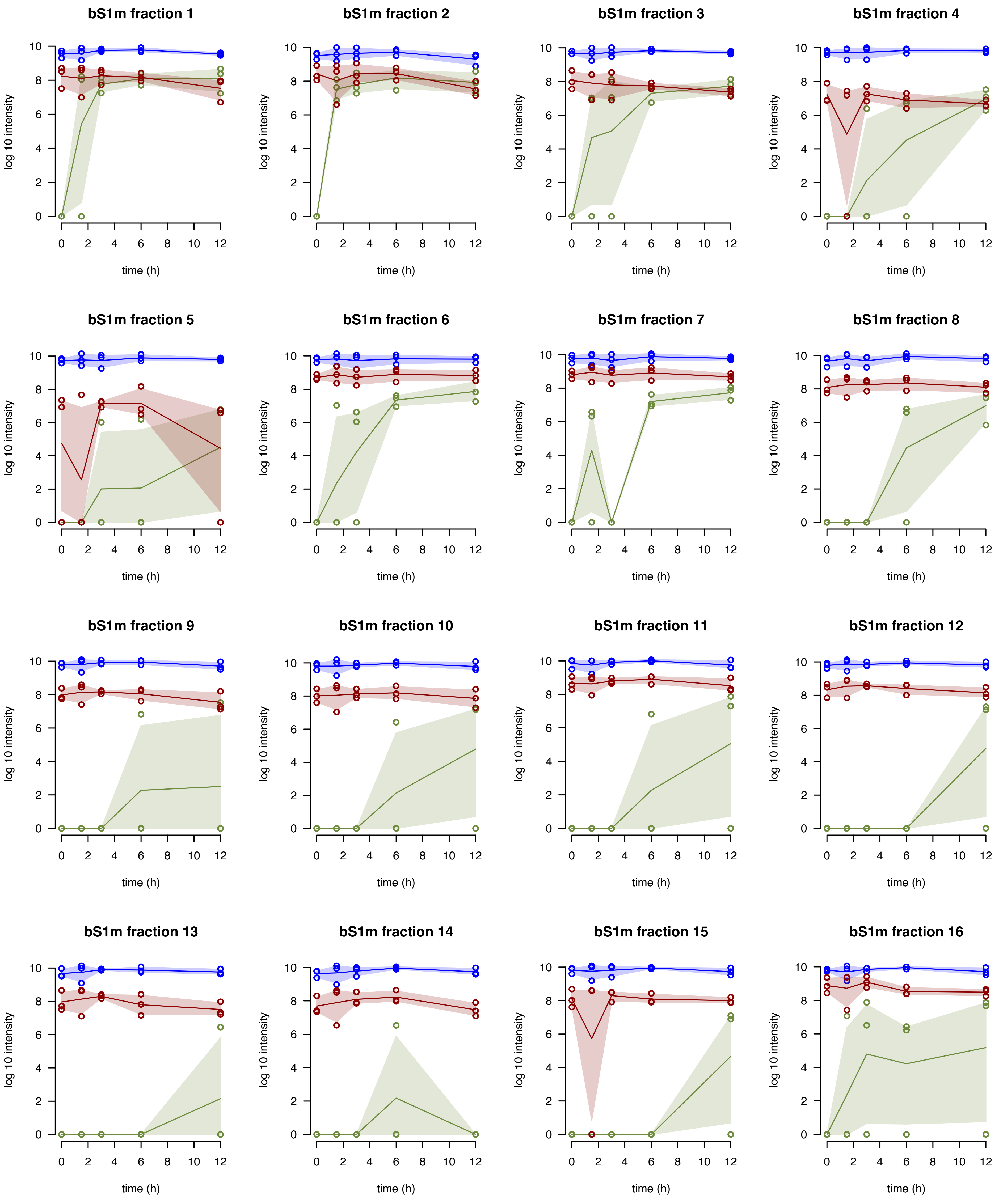
bS1m fraction 1
bS1m fraction 2
bS1m fraction 3
bS1m fraction 4
bS1m fraction 5
bS1m fraction 6
bS1m fraction 7
bS1m fraction 8
bS1m fraction 9
bS1m fraction 10
bS1m fraction 11
bS1m fraction 12
bS1m fraction 13
bS1m fraction 14
bS1m fraction 15
bS1m fraction 16
log 10 intensity
time (h)
0
2
4
6
8
10
12

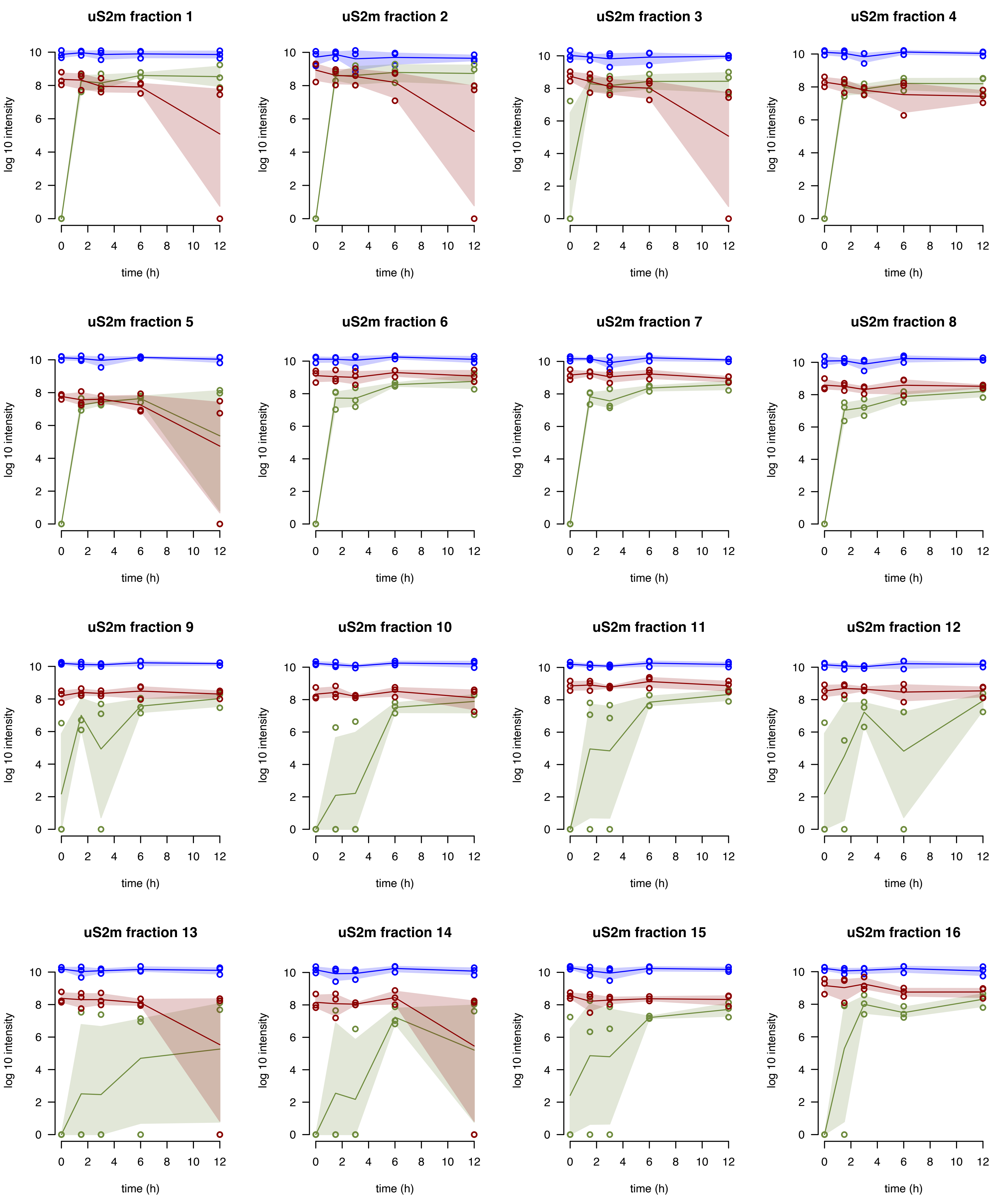
uS2m fraction 1
uS2m fraction 2
uS2m fraction 3
uS2m fraction 4
uS2m fraction 5
uS2m fraction 6
uS2m fraction 7
uS2m fraction 8
uS2m fraction 9
uS2m fraction 10
uS2m fraction 11
uS2m fraction 12
uS2m fraction 13
uS2m fraction 14
uS2m fraction 15
uS2m fraction 16
log 10 intensity
time (h)
0 2 4 6 8 10
0 2 4 6 8 10 12

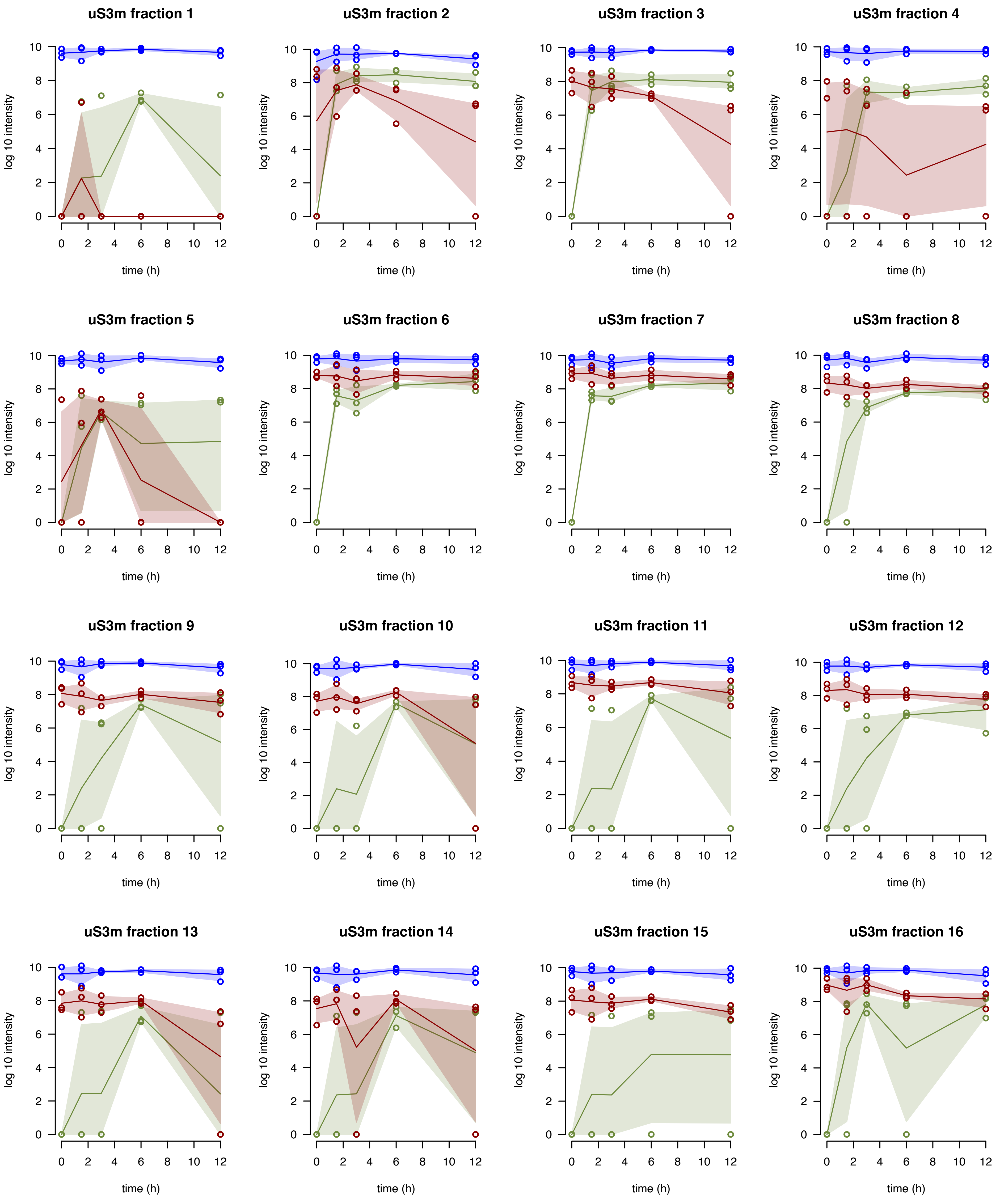
uS3m fraction 1
uS3m fraction 2
uS3m fraction 3
uS3m fraction 4
uS3m fraction 5
uS3m fraction 6
uS3m fraction 7
uS3m fraction 8
uS3m fraction 9
uS3m fraction 10
uS3m fraction 11
uS3m fraction 12
uS3m fraction 13
uS3m fraction 14
uS3m fraction 15
uS3m fraction 16
log 10 intensity
time (h)
0 2 4 6 8 10
0 2 4 6 8 10 12

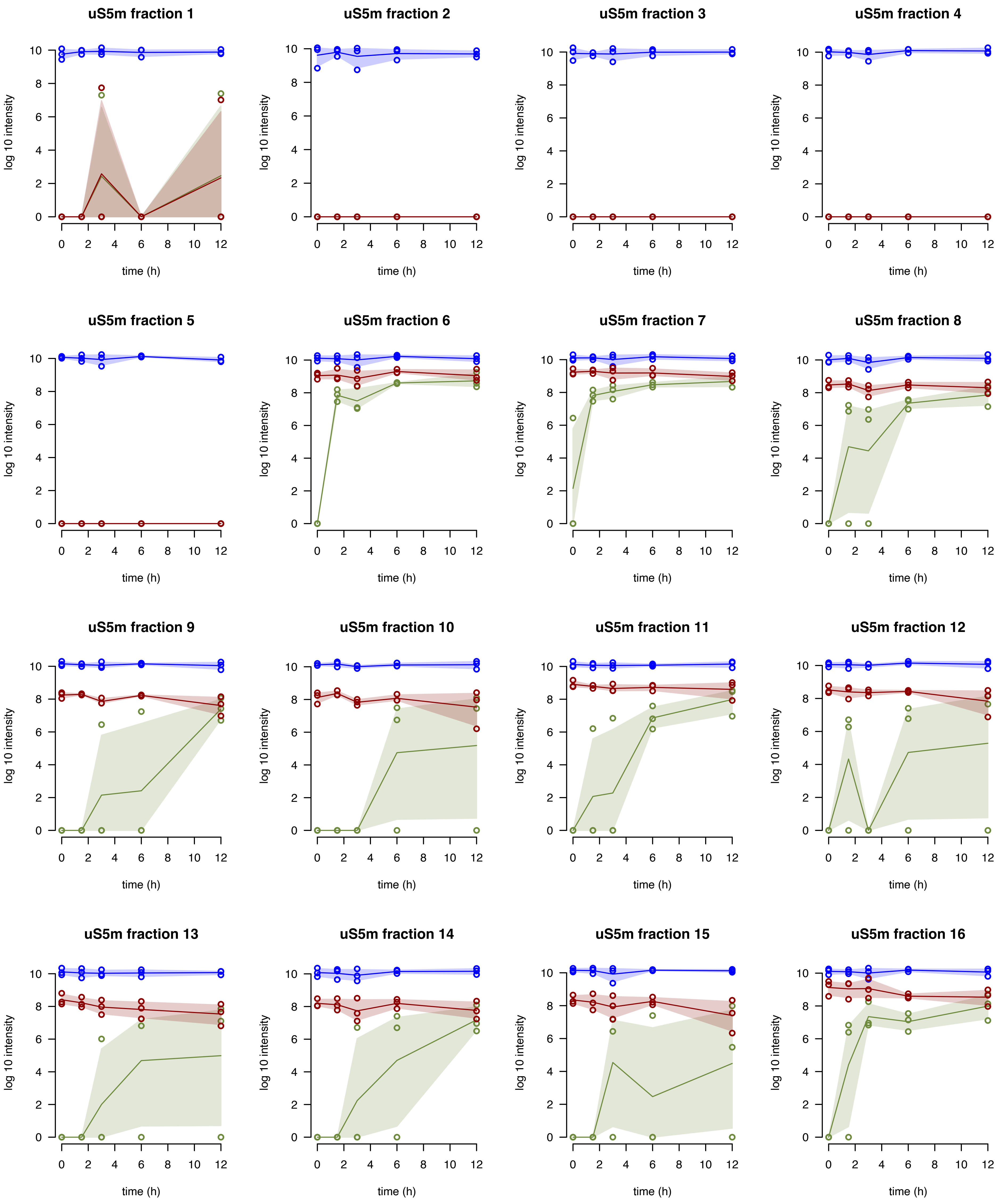
uS5m fraction 1
uS5m fraction 2
uS5m fraction 3
uS5m fraction 4
uS5m fraction 5
uS5m fraction 6
uS5m fraction 7
uS5m fraction 8
uS5m fraction 9
uS5m fraction 10
uS5m fraction 11
uS5m fraction 12
uS5m fraction 13
uS5m fraction 14
uS5m fraction 15
uS5m fraction 16
log 10 intensity
time (h)

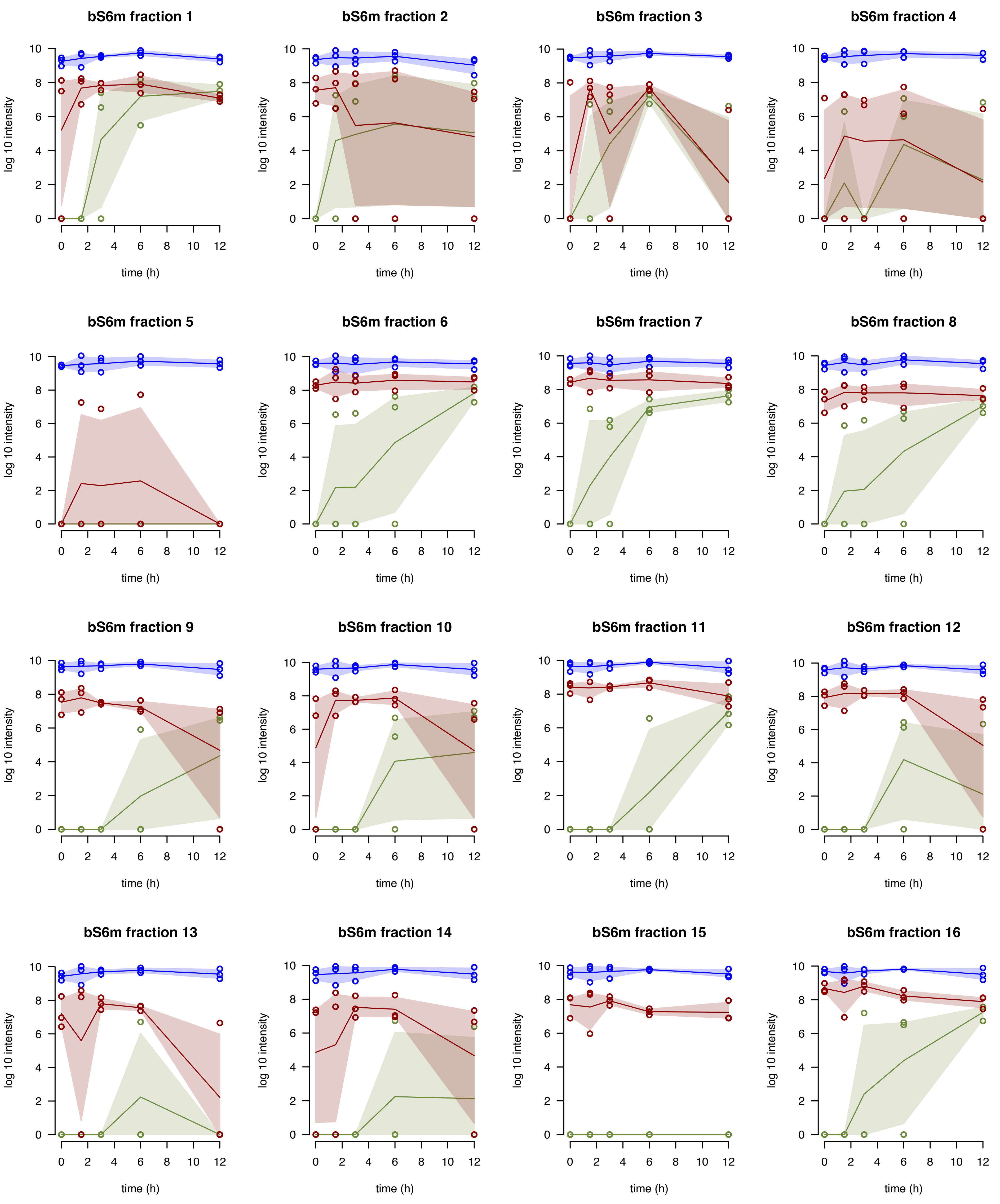
bS6m fraction 1
bS6m fraction 2
bS6m fraction 3
bS6m fraction 4
bS6m fraction 5
bS6m fraction 6
bS6m fraction 7
bS6m fraction 8
bS6m fraction 9
bS6m fraction 10
bS6m fraction 11
bS6m fraction 12
bS6m fraction 13
bS6m fraction 14
bS6m fraction 15
bS6m fraction 16
log 10 intensity
time (h)

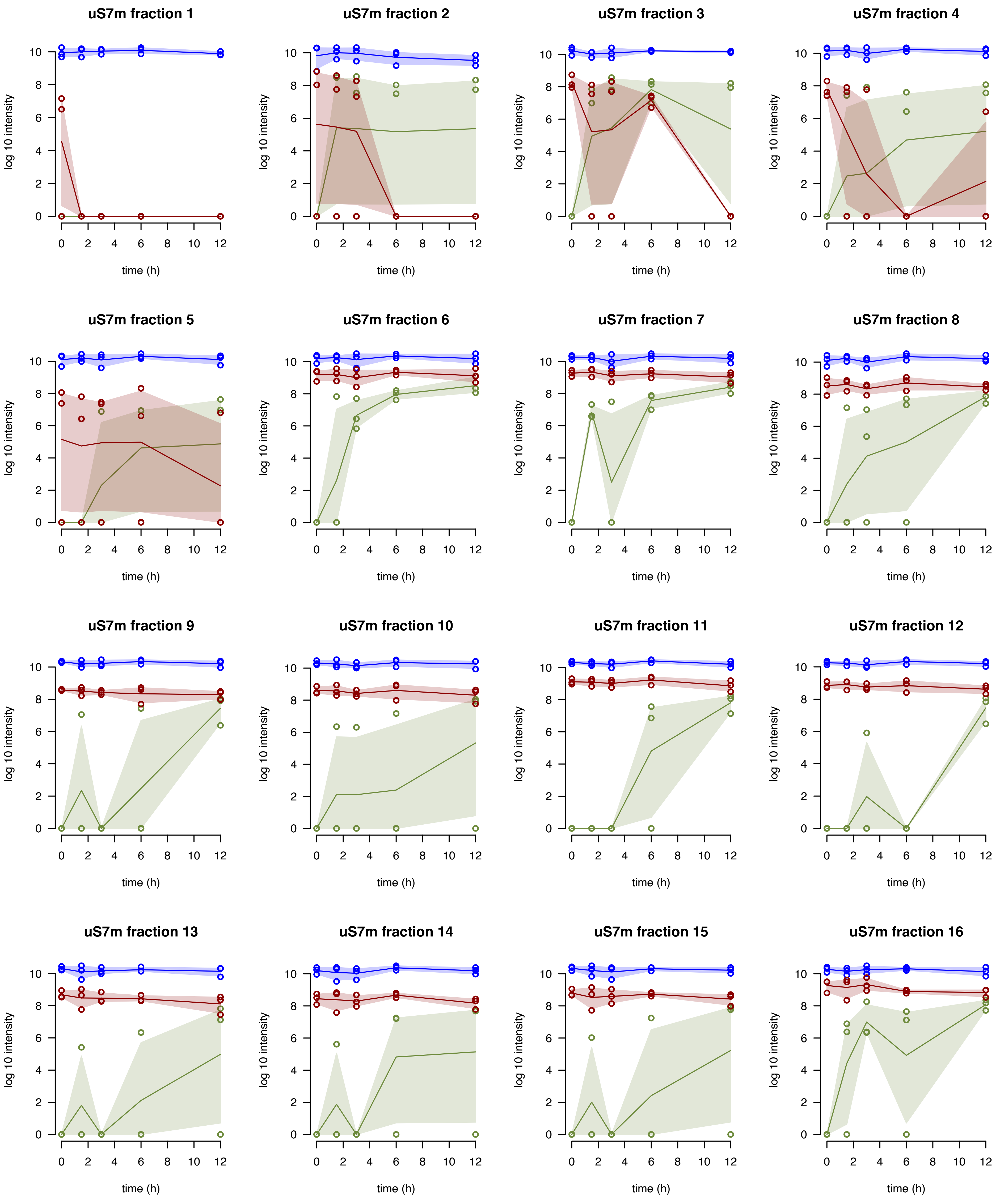
uS7m fraction 1
uS7m fraction 2
uS7m fraction 3
uS7m fraction 4
uS7m fraction 5
uS7m fraction 6
uS7m fraction 7
uS7m fraction 8
uS7m fraction 9
uS7m fraction 10
uS7m fraction 11
uS7m fraction 12
uS7m fraction 13
uS7m fraction 14
uS7m fraction 15
uS7m fraction 16
log 10 intensity
time (h)
0 2 4 6 8 10
0 2 4 6 8 10 12

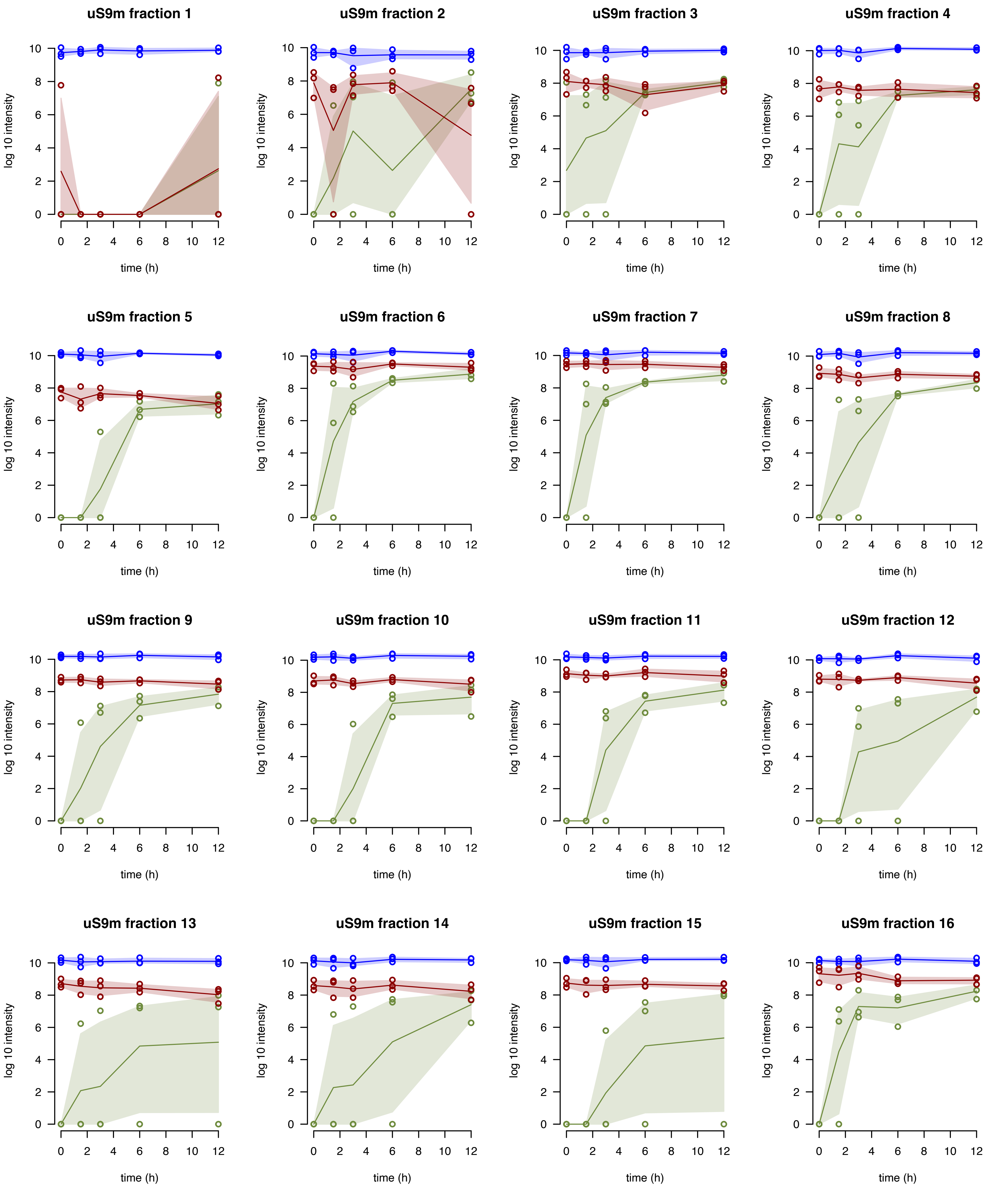
uS9m fraction 1
uS9m fraction 2
uS9m fraction 3
uS9m fraction 4
uS9m fraction 5
uS9m fraction 6
uS9m fraction 7
uS9m fraction 8
uS9m fraction 9
uS9m fraction 10
uS9m fraction 11
uS9m fraction 12
uS9m fraction 13
uS9m fraction 14
uS9m fraction 15
uS9m fraction 16
log 10 intensity
time (h)
0
2
4
6
8
10
12

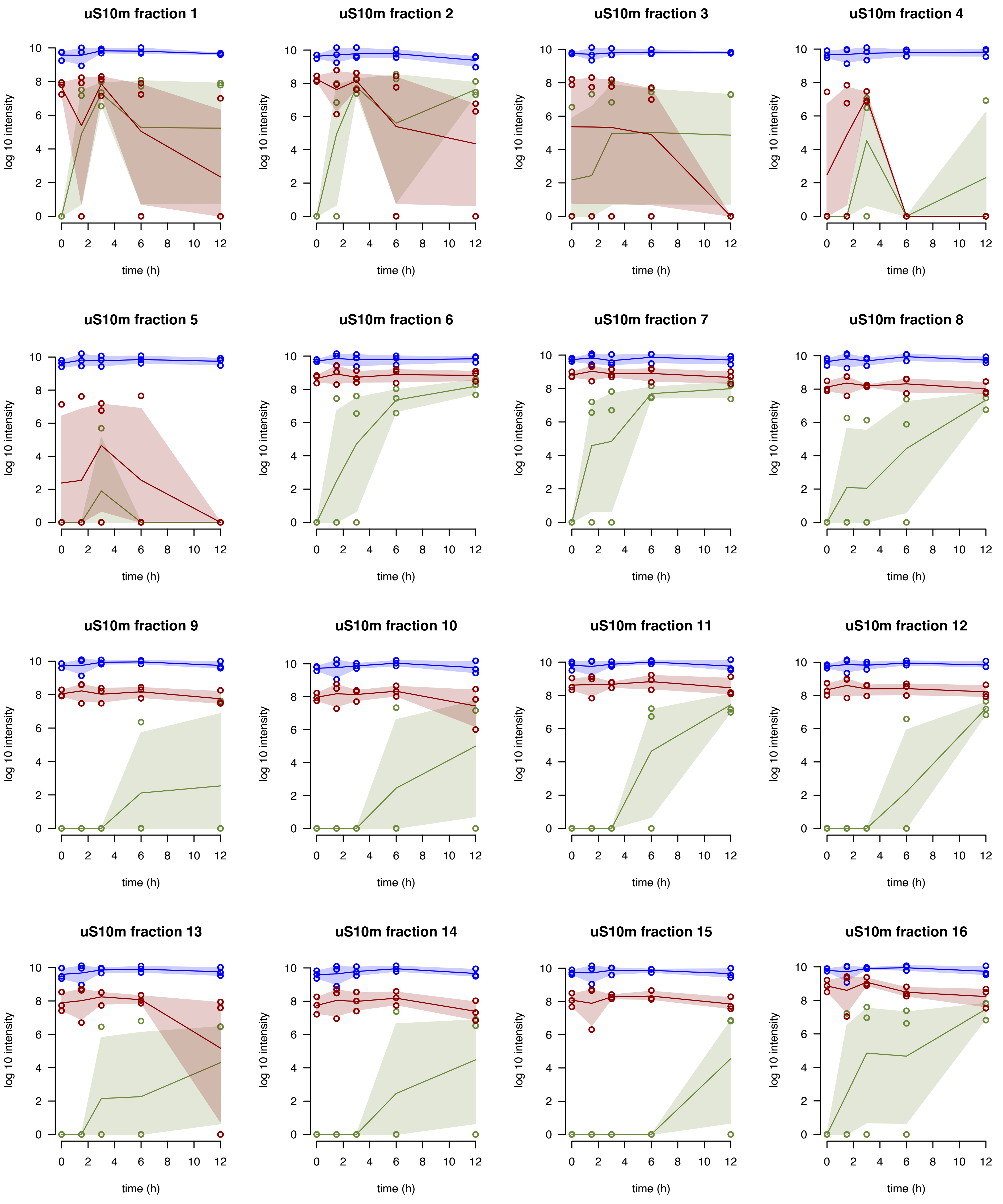

uS10m fraction 1
uS10m fraction 2
uS10m fraction 3
uS10m fraction 4
uS10m fraction 5
uS10m fraction 6
uS10m fraction 7
uS10m fraction 8
uS10m fraction 9
uS10m fraction 10
uS10m fraction 11
uS10m fraction 12
uS10m fraction 13
uS10m fraction 14
uS10m fraction 15
uS10m fraction 16
log 10 intensity
time (h)

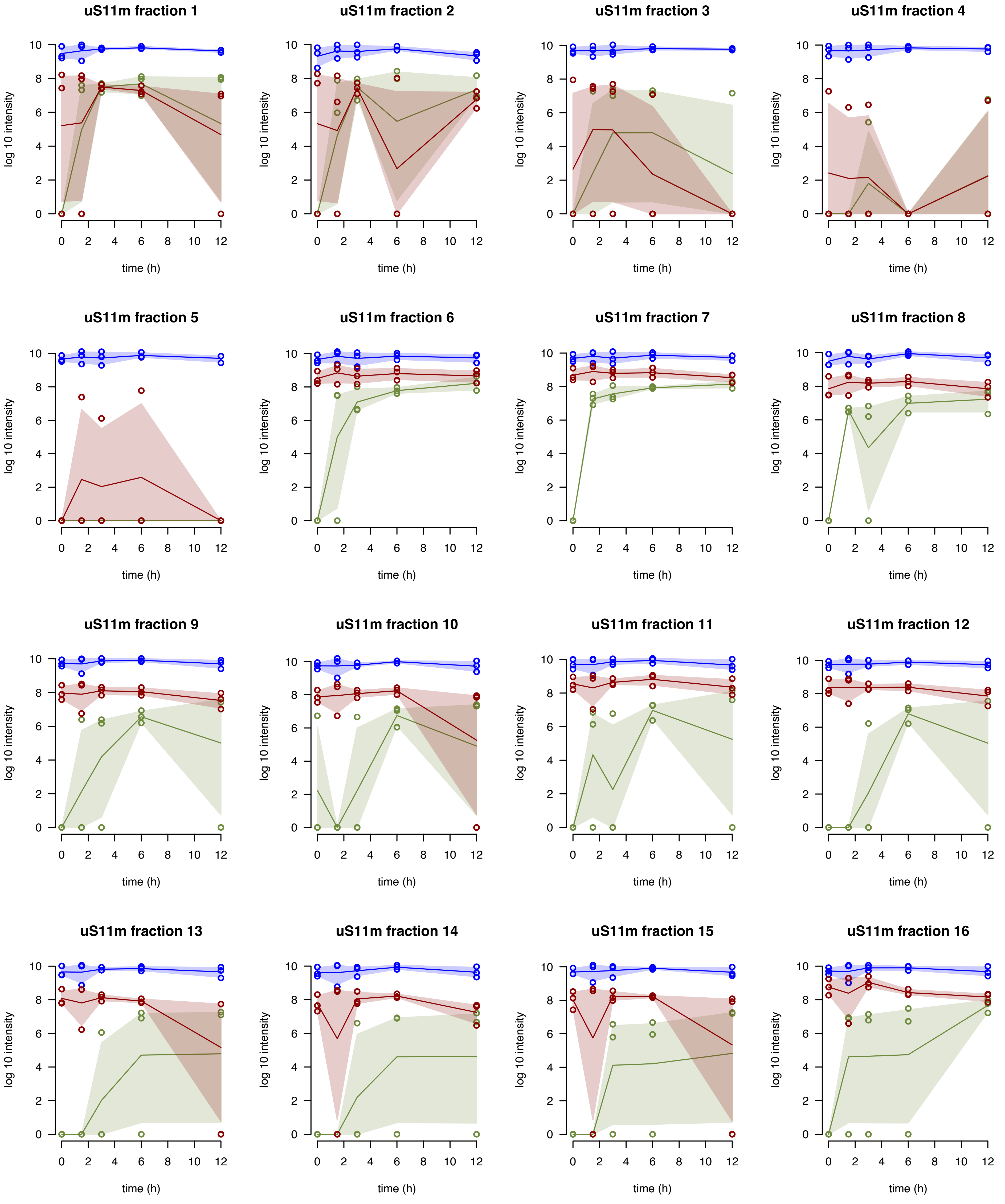
uS11m fraction 1
uS11m fraction 2
uS11m fraction 3
uS11m fraction 4
uS11m fraction 5
uS11m fraction 6
uS11m fraction 7
uS11m fraction 8
uS11m fraction 9
uS11m fraction 10
uS11m fraction 11
uS11m fraction 12
uS11m fraction 13
uS11m fraction 14
uS11m fraction 15
uS11m fraction 16
log 10 intensity
time (h)

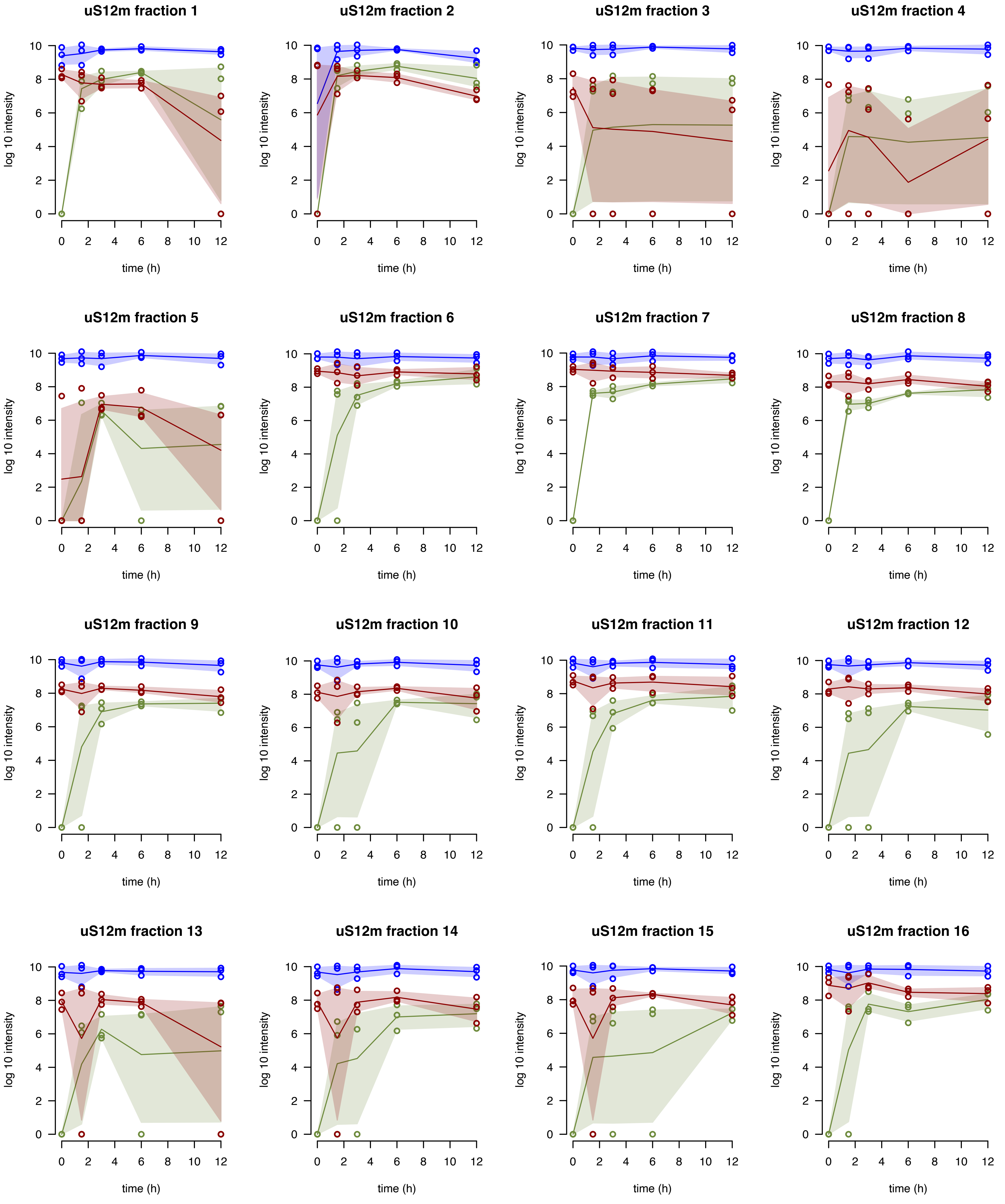
uS12m fraction 1
uS12m fraction 2
uS12m fraction 3
uS12m fraction 4
uS12m fraction 5
uS12m fraction 6
uS12m fraction 7
uS12m fraction 8
uS12m fraction 9
uS12m fraction 10
uS12m fraction 11
uS12m fraction 12
uS12m fraction 13
uS12m fraction 14
uS12m fraction 15
uS12m fraction 16
log 10 intensity
time (h)

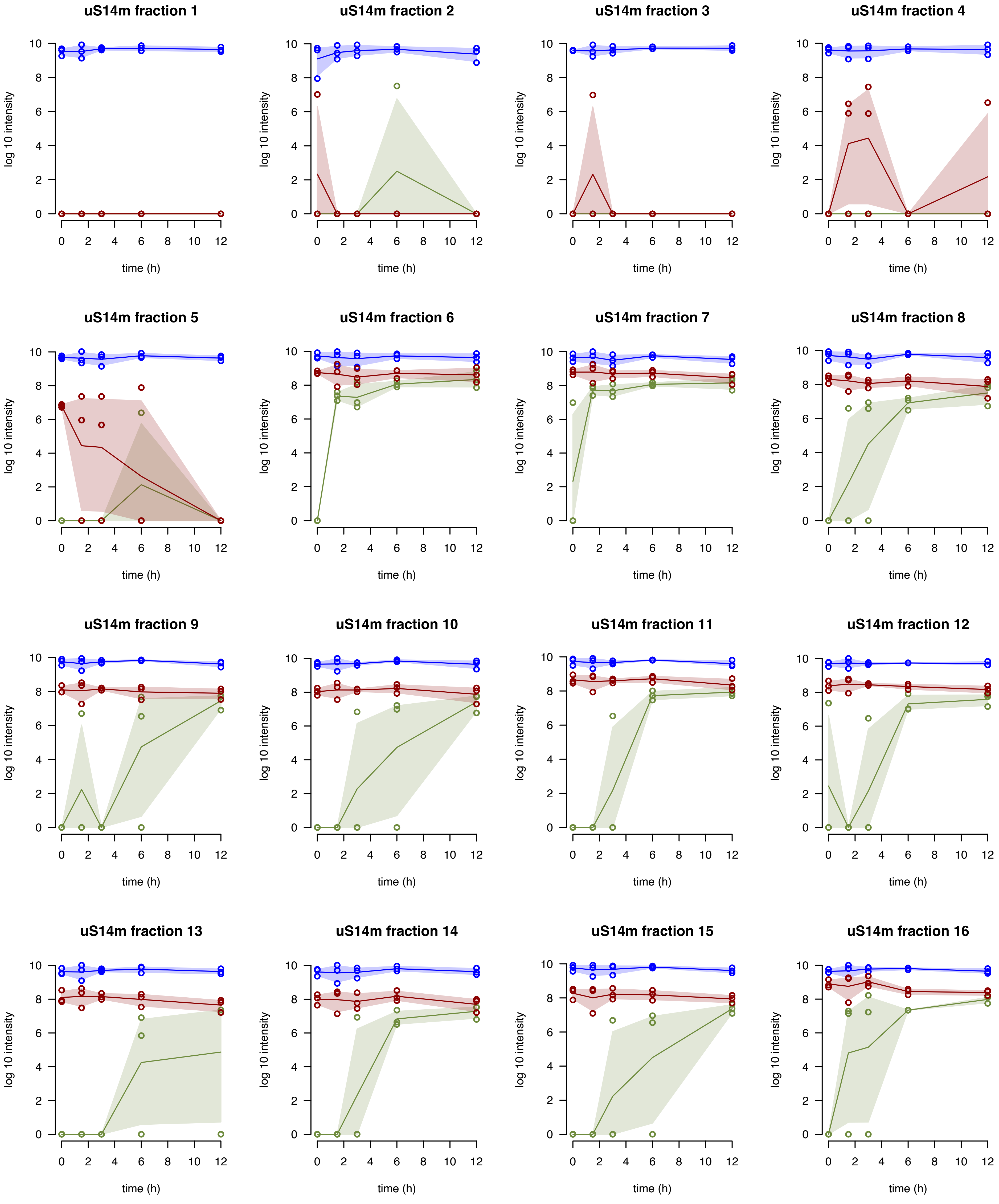
uS14m fraction 1
uS14m fraction 2
uS14m fraction 3
uS14m fraction 4
uS14m fraction 5
uS14m fraction 6
uS14m fraction 7
uS14m fraction 8
uS14m fraction 9
uS14m fraction 10
uS14m fraction 11
uS14m fraction 12
uS14m fraction 13
uS14m fraction 14
uS14m fraction 15
uS14m fraction 16
log 10 intensity
time (h)

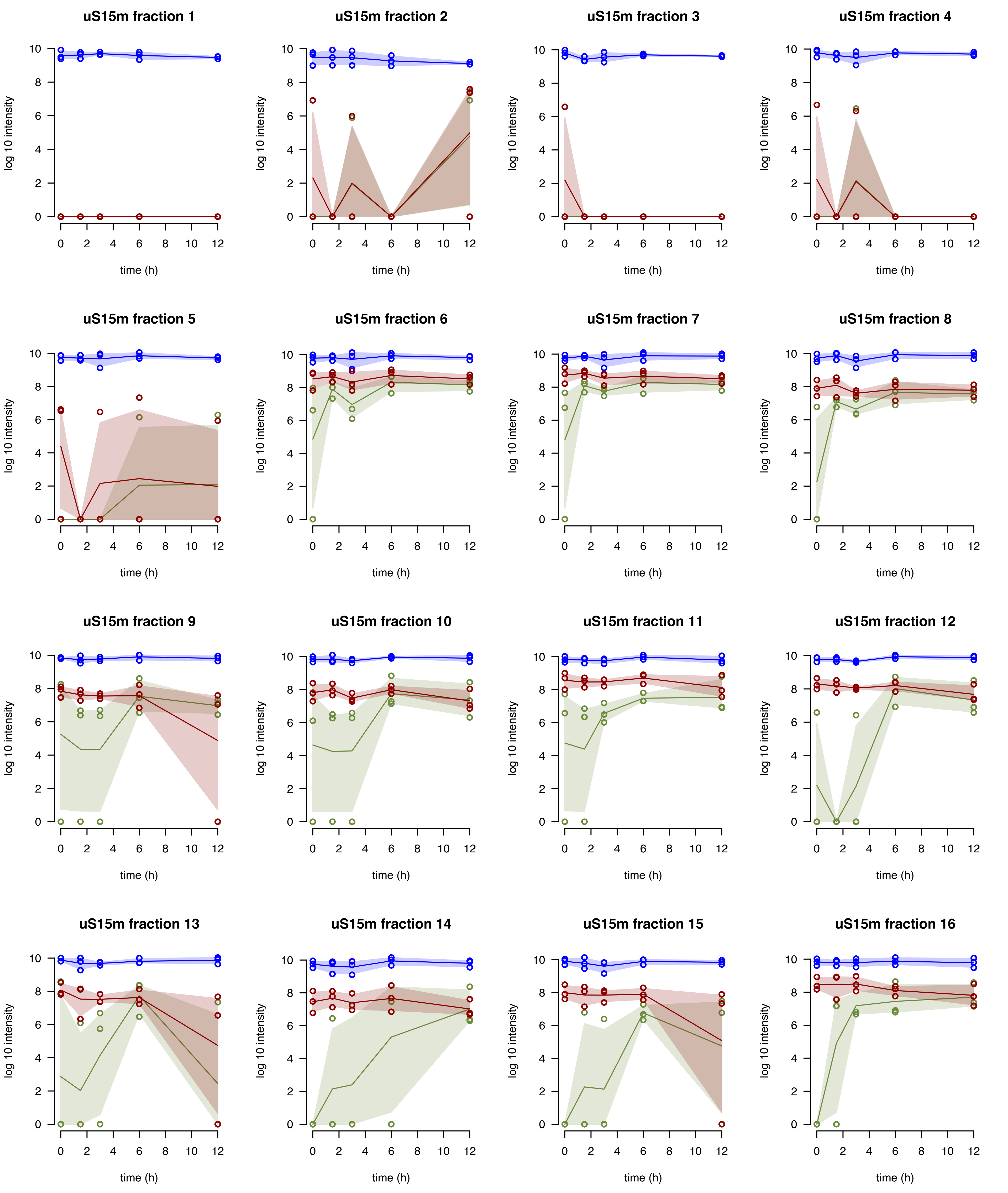
uS15m fraction 1
uS15m fraction 2
uS15m fraction 3
uS15m fraction 4
uS15m fraction 5
uS15m fraction 6
uS15m fraction 7
uS15m fraction 8
uS15m fraction 9
uS15m fraction 10
uS15m fraction 11
uS15m fraction 12
uS15m fraction 13
uS15m fraction 14
uS15m fraction 15
uS15m fraction 16
log 10 intensity
time (h)

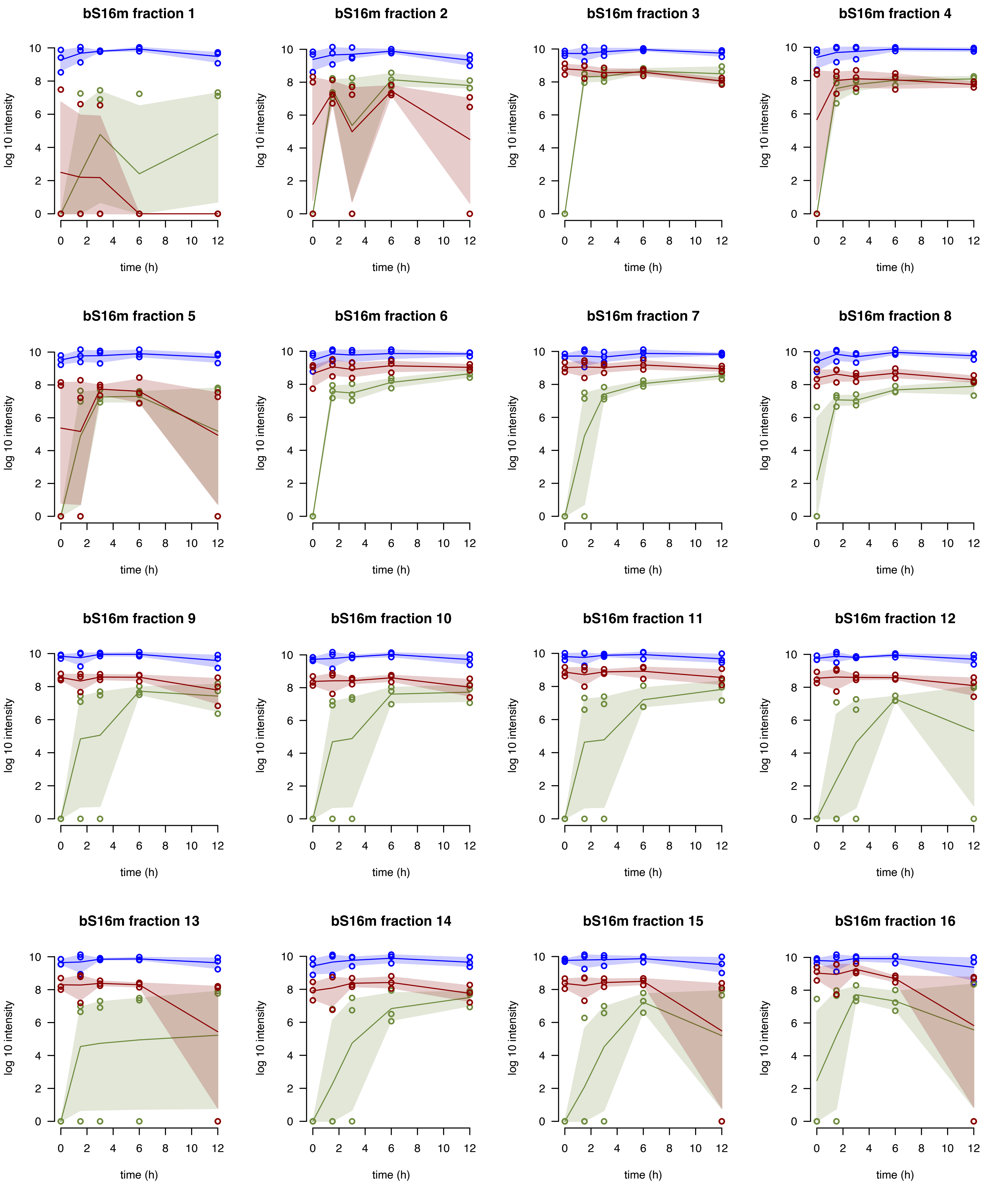

bS16m fraction 1
bS16m fraction 2
bS16m fraction 3
bS16m fraction 4
bS16m fraction 5
bS16m fraction 6
bS16m fraction 7
bS16m fraction 8
bS16m fraction 9
bS16m fraction 10
bS16m fraction 11
bS16m fraction 12
bS16m fraction 13
bS16m fraction 14
bS16m fraction 15
bS16m fraction 16
log 10 intensity
time (h)

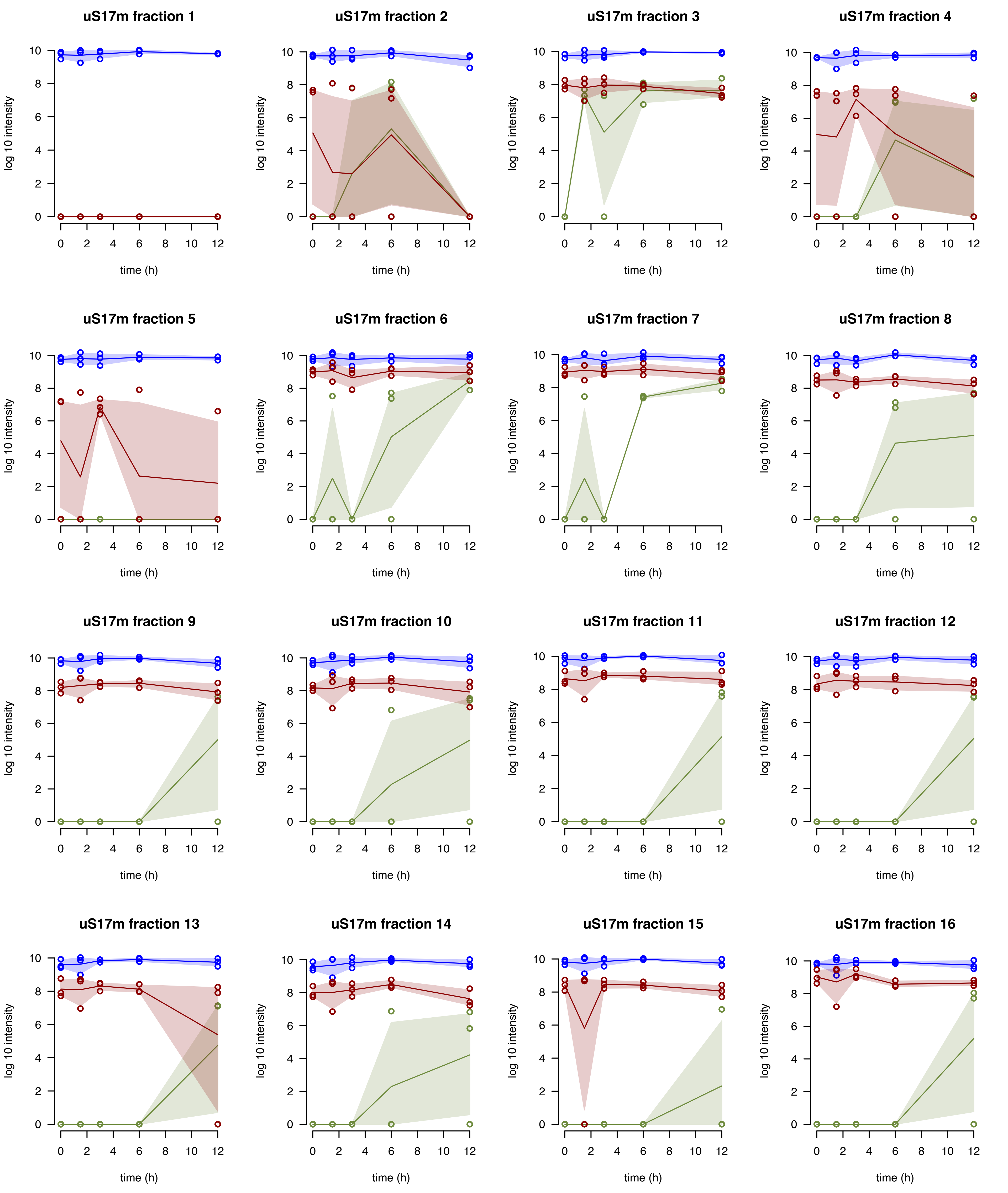
uS17m fraction 1
uS17m fraction 2
uS17m fraction 3
uS17m fraction 4
uS17m fraction 5
uS17m fraction 6
uS17m fraction 7
uS17m fraction 8
uS17m fraction 9
uS17m fraction 10
uS17m fraction 11
uS17m fraction 12
uS17m fraction 13
uS17m fraction 14
uS17m fraction 15
uS17m fraction 16
log 10 intensity
time (h)

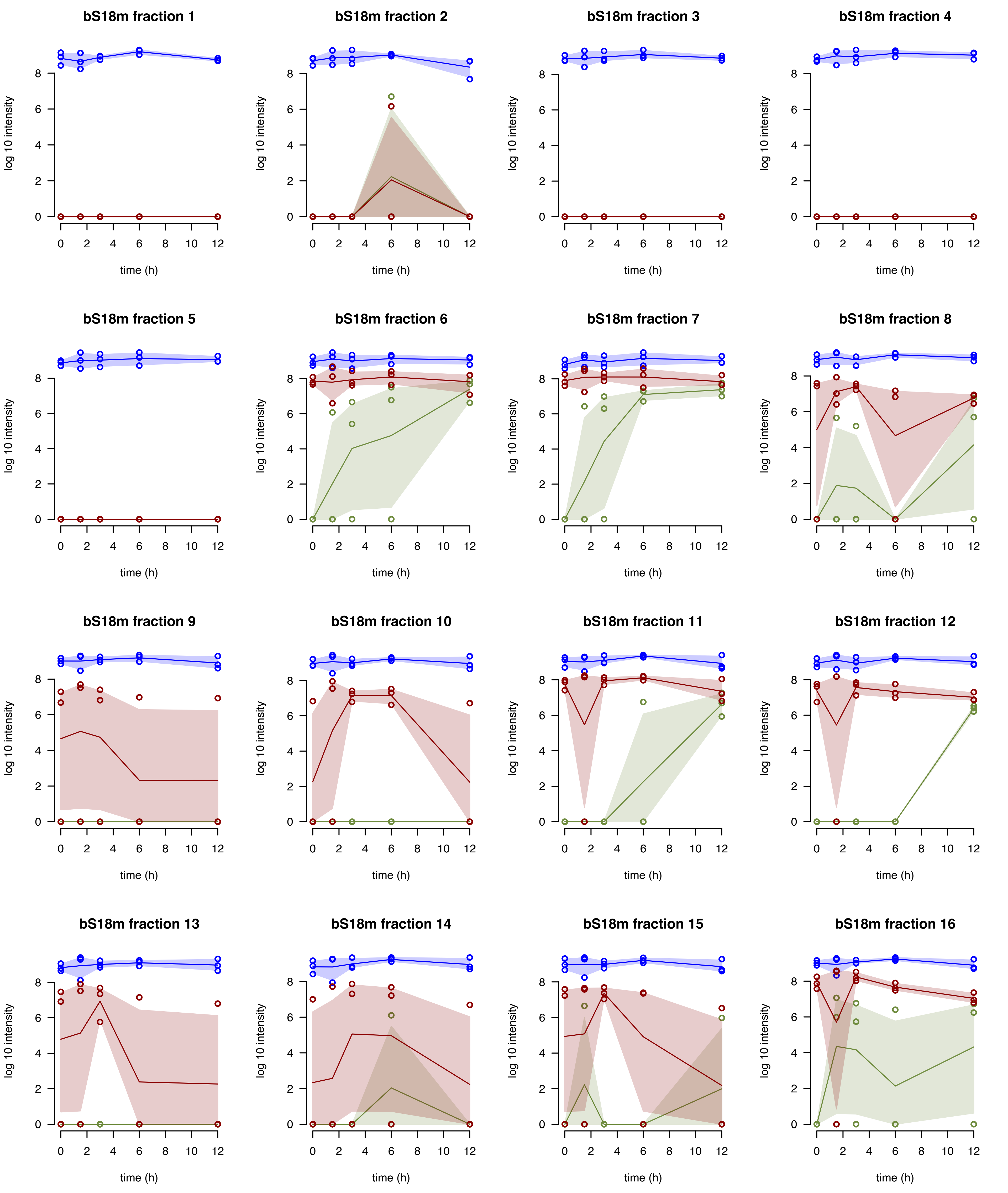
bS18m fraction 1
bS18m fraction 2
bS18m fraction 3
bS18m fraction 4
bS18m fraction 5
bS18m fraction 6
bS18m fraction 7
bS18m fraction 8
bS18m fraction 9
bS18m fraction 10
bS18m fraction 11
bS18m fraction 12
bS18m fraction 13
bS18m fraction 14
bS18m fraction 15
bS18m fraction 16
log 10 intensity
time (h)

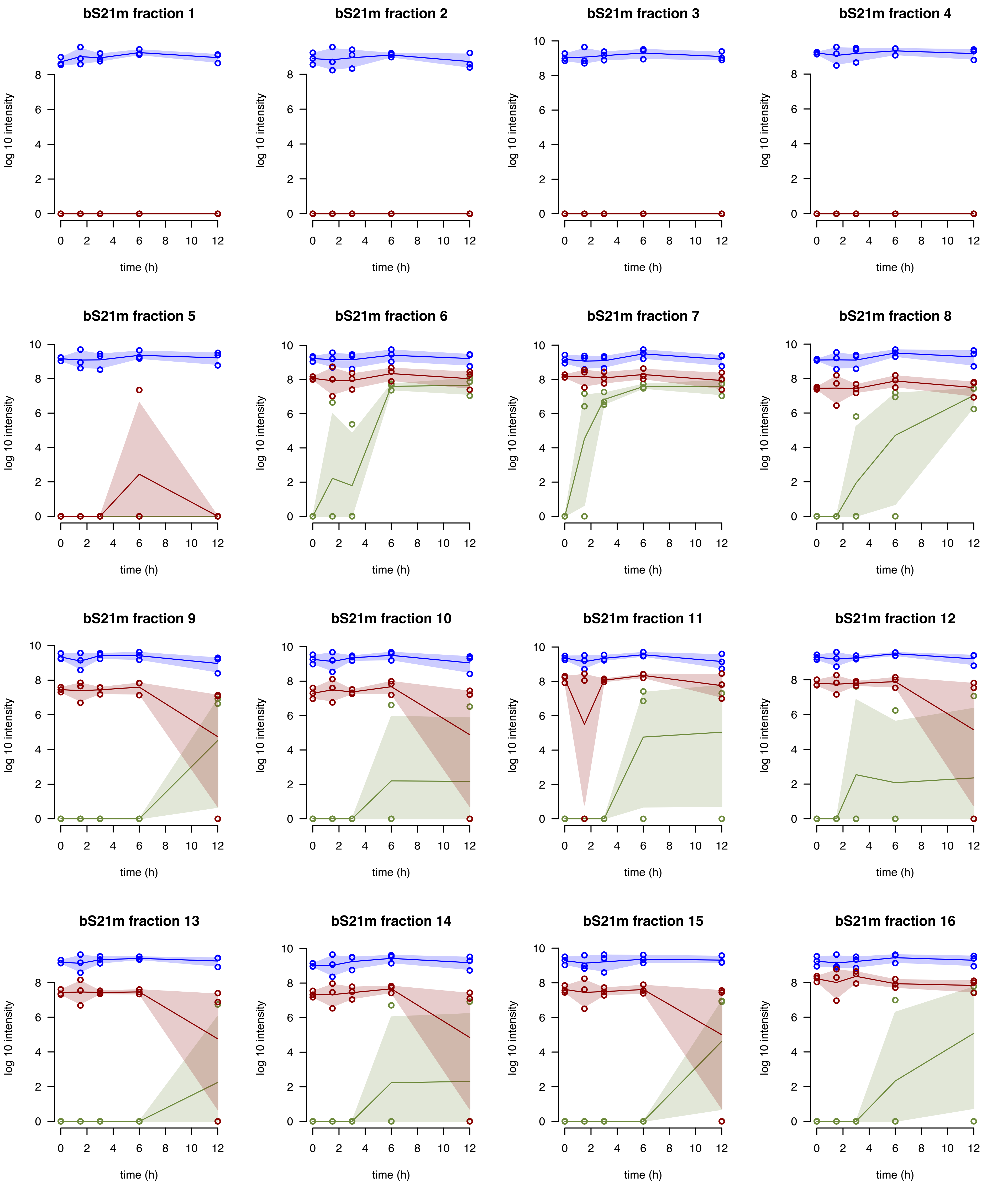
bS21m fraction 1
bS21m fraction 2
bS21m fraction 3
bS21m fraction 4
bS21m fraction 5
bS21m fraction 6
bS21m fraction 7
bS21m fraction 8
bS21m fraction 9
bS21m fraction 10
bS21m fraction 11
bS21m fraction 12
bS21m fraction 13
bS21m fraction 14
bS21m fraction 15
bS21m fraction 16
log 10 intensity
time (h)

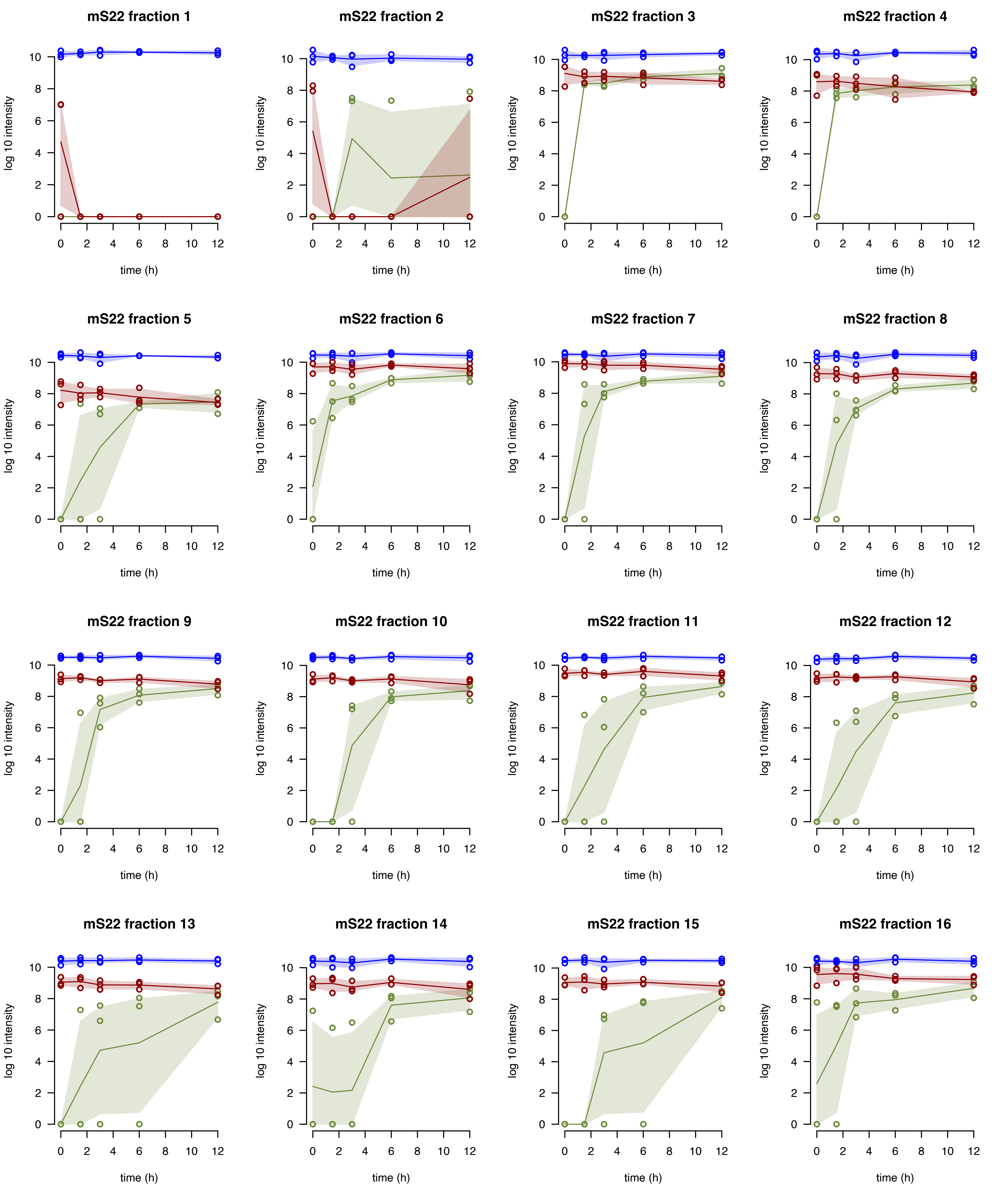
mS22 fraction 1
mS22 fraction 2
mS22 fraction 3
mS22 fraction 4
mS22 fraction 5
mS22 fraction 6
mS22 fraction 7
mS22 fraction 8
mS22 fraction 9
mS22 fraction 10
mS22 fraction 11
mS22 fraction 12
mS22 fraction 13
mS22 fraction 14
mS22 fraction 15
mS22 fraction 16
log 10 intensity
time (h)

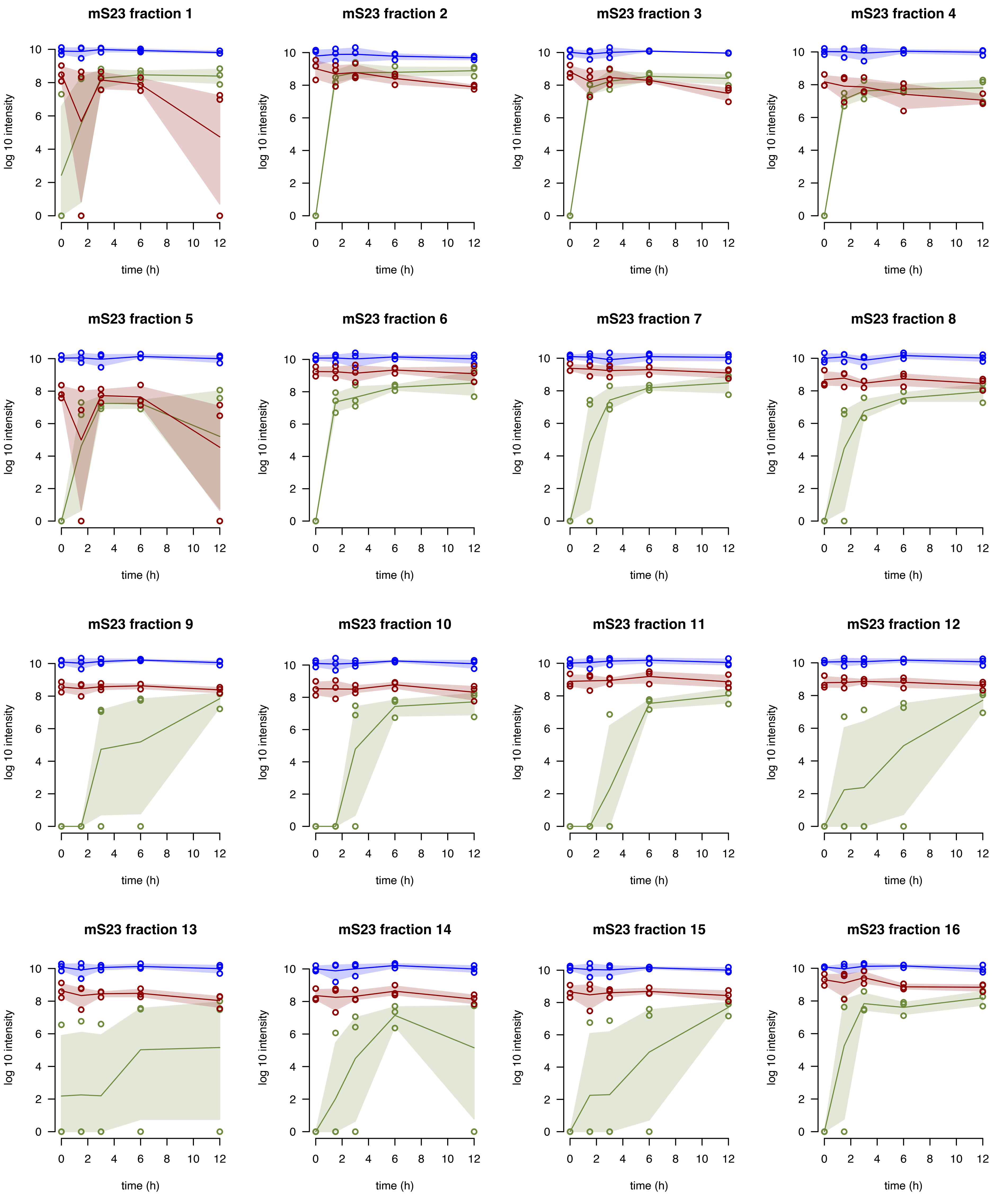
mS23 fraction 1
mS23 fraction 2
mS23 fraction 3
mS23 fraction 4
mS23 fraction 5
mS23 fraction 6
mS23 fraction 7
mS23 fraction 8
mS23 fraction 9
mS23 fraction 10
mS23 fraction 11
mS23 fraction 12
mS23 fraction 13
mS23 fraction 14
mS23 fraction 15
mS23 fraction 16
log 10 intensity
time (h)
0 2 4 6 8 10
0 2 4 6 8 10 12

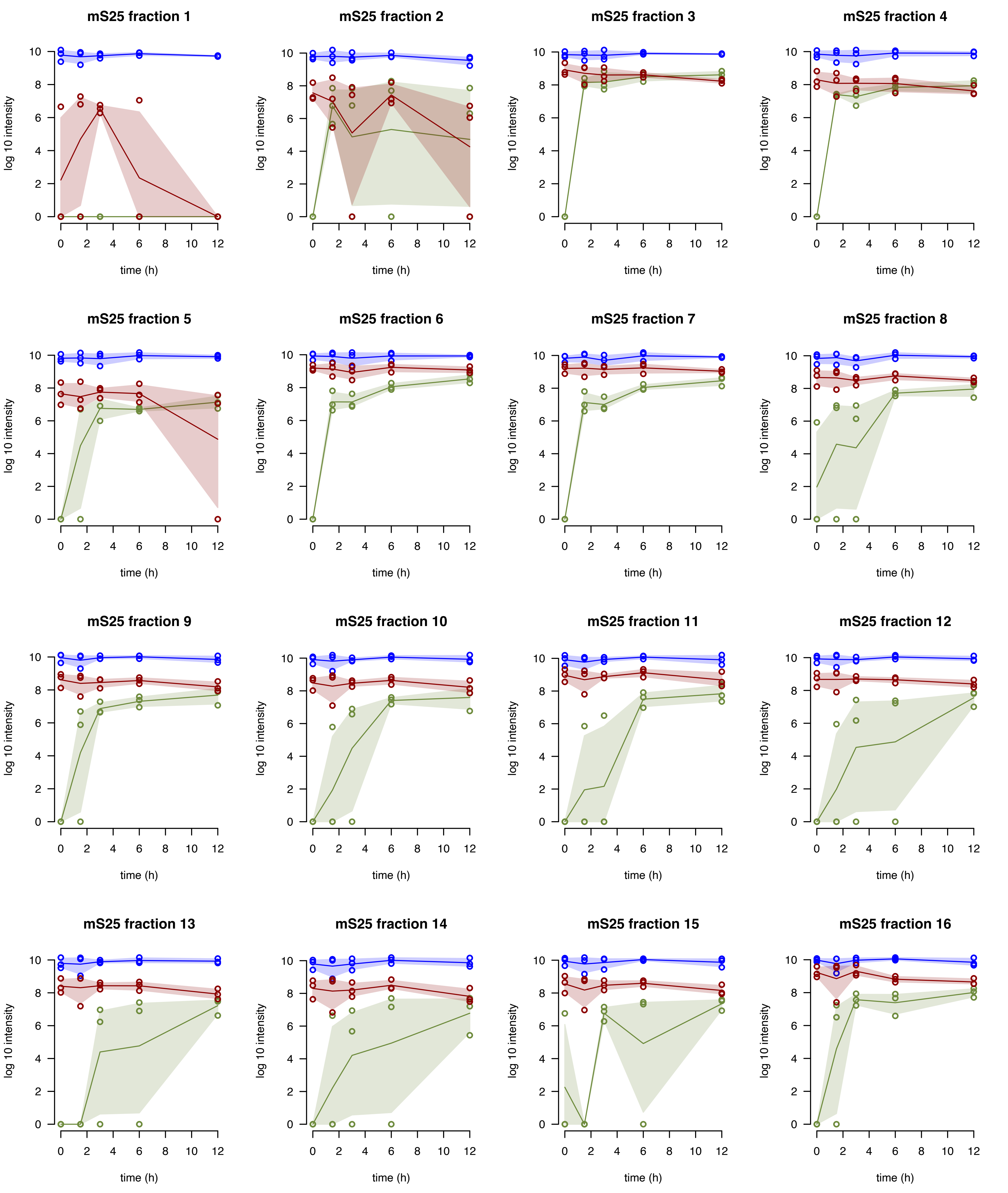

mS25 fraction 1
mS25 fraction 2
mS25 fraction 3
mS25 fraction 4
mS25 fraction 5
mS25 fraction 6
mS25 fraction 7
mS25 fraction 8
mS25 fraction 9
mS25 fraction 10
mS25 fraction 11
mS25 fraction 12
mS25 fraction 13
mS25 fraction 14
mS25 fraction 15
mS25 fraction 16
log 10 intensity
time (h)

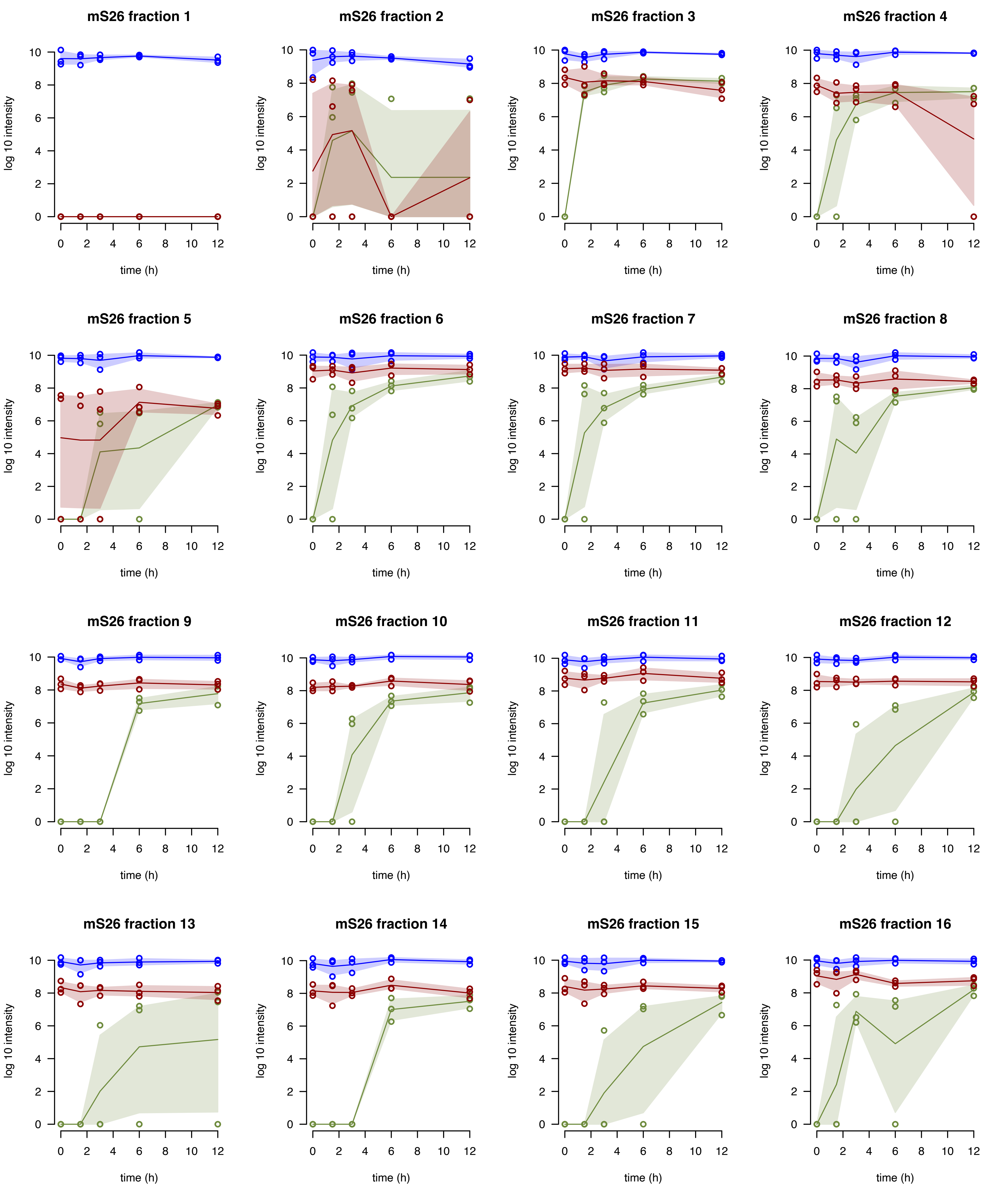
mS26 fraction 1
mS26 fraction 2
mS26 fraction 3
mS26 fraction 4
mS26 fraction 5
mS26 fraction 6
mS26 fraction 7
mS26 fraction 8
mS26 fraction 9
mS26 fraction 10
mS26 fraction 11
mS26 fraction 12
mS26 fraction 13
mS26 fraction 14
mS26 fraction 15
mS26 fraction 16
log 10 intensity
time (h)

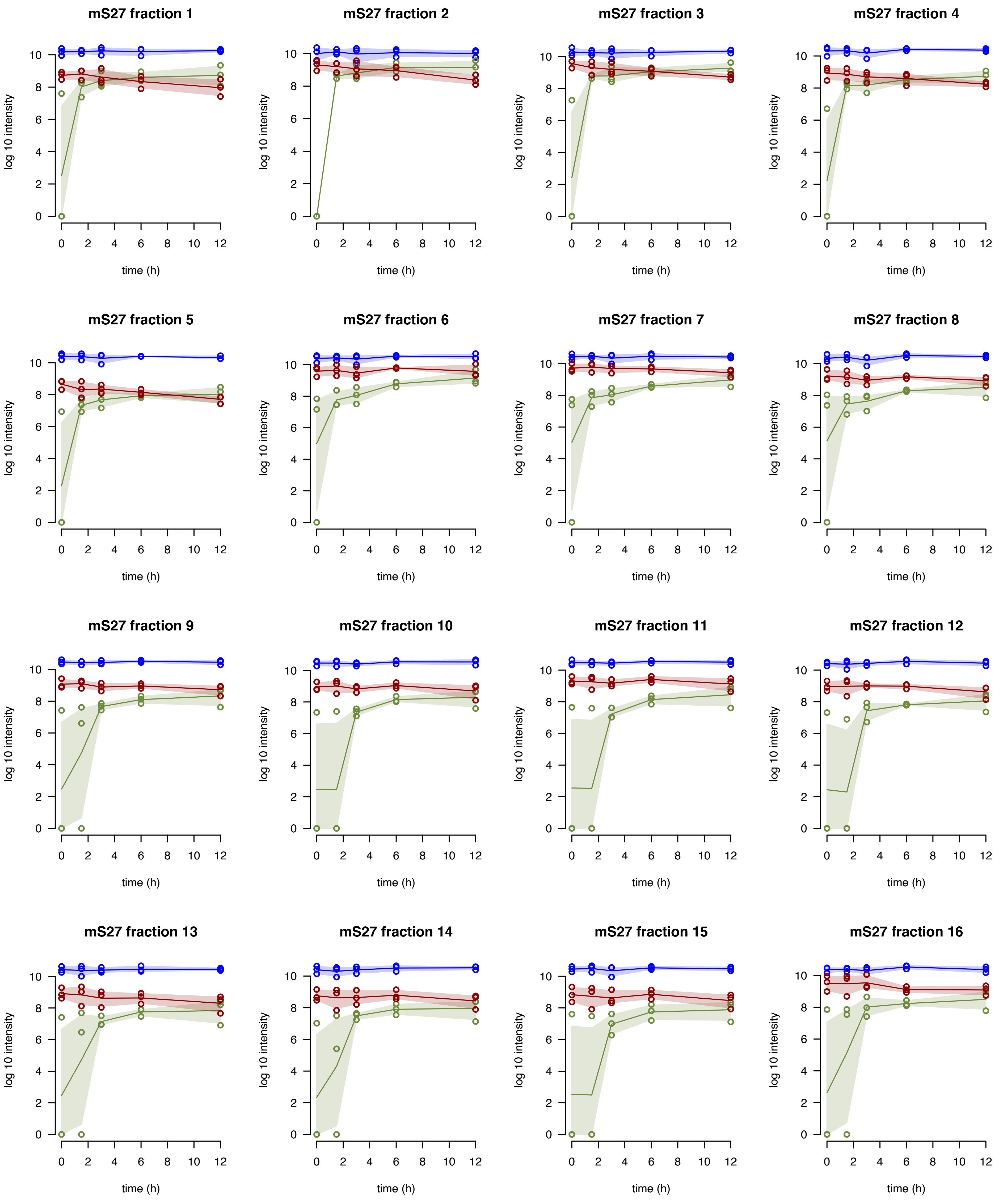
mS27 fraction 1
mS27 fraction 2
mS27 fraction 3
mS27 fraction 4
mS27 fraction 5
mS27 fraction 6
mS27 fraction 7
mS27 fraction 8
mS27 fraction 9
mS27 fraction 10
mS27 fraction 11
mS27 fraction 12
mS27 fraction 13
mS27 fraction 14
mS27 fraction 15
mS27 fraction 16
log 10 intensity
time (h)

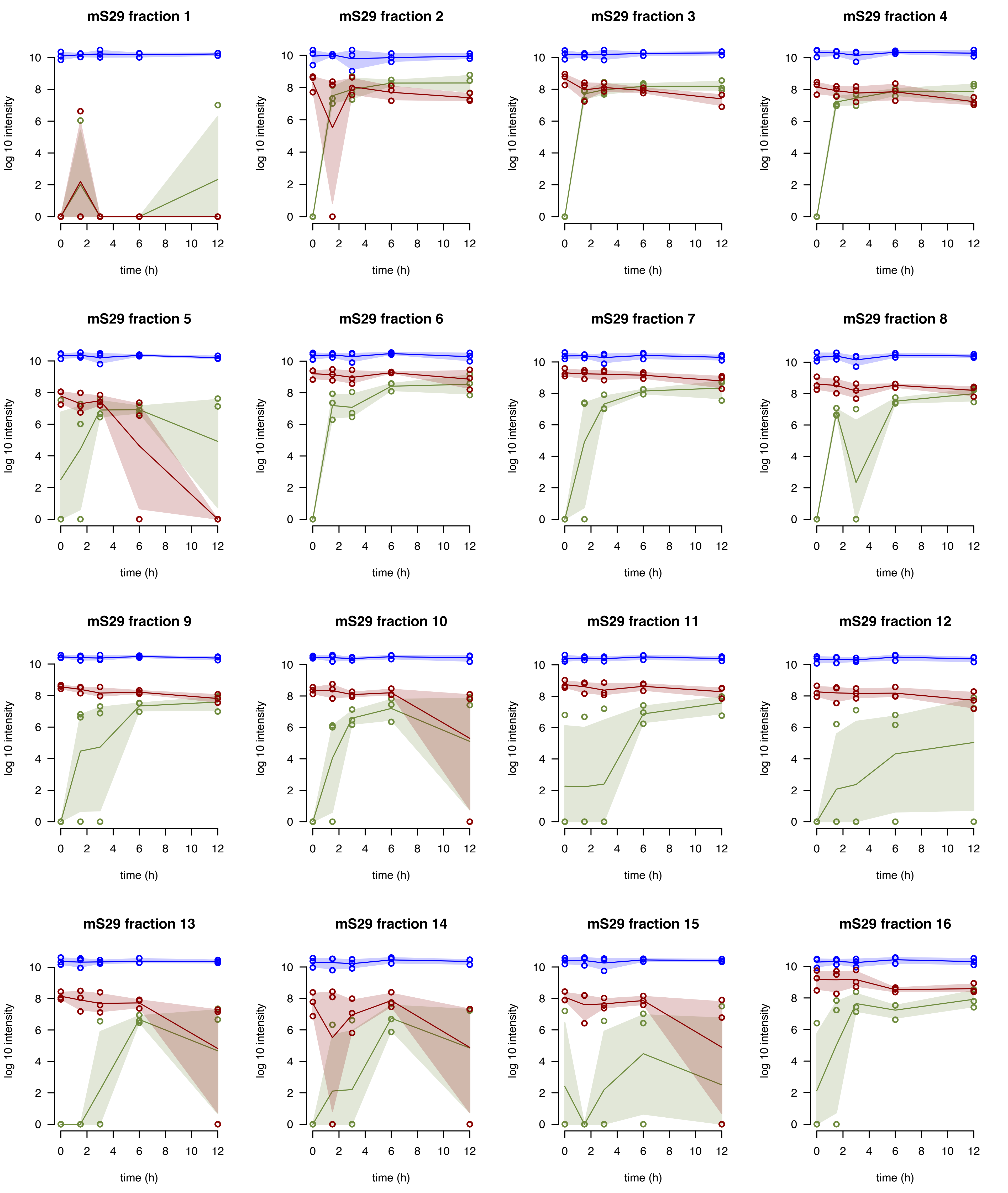
mS29 fraction 1
mS29 fraction 2
mS29 fraction 3
mS29 fraction 4
mS29 fraction 5
mS29 fraction 6
mS29 fraction 7
mS29 fraction 8
mS29 fraction 9
mS29 fraction 10
mS29 fraction 11
mS29 fraction 12
mS29 fraction 13
mS29 fraction 14
mS29 fraction 15
mS29 fraction 16
log 10 intensity
time (h)

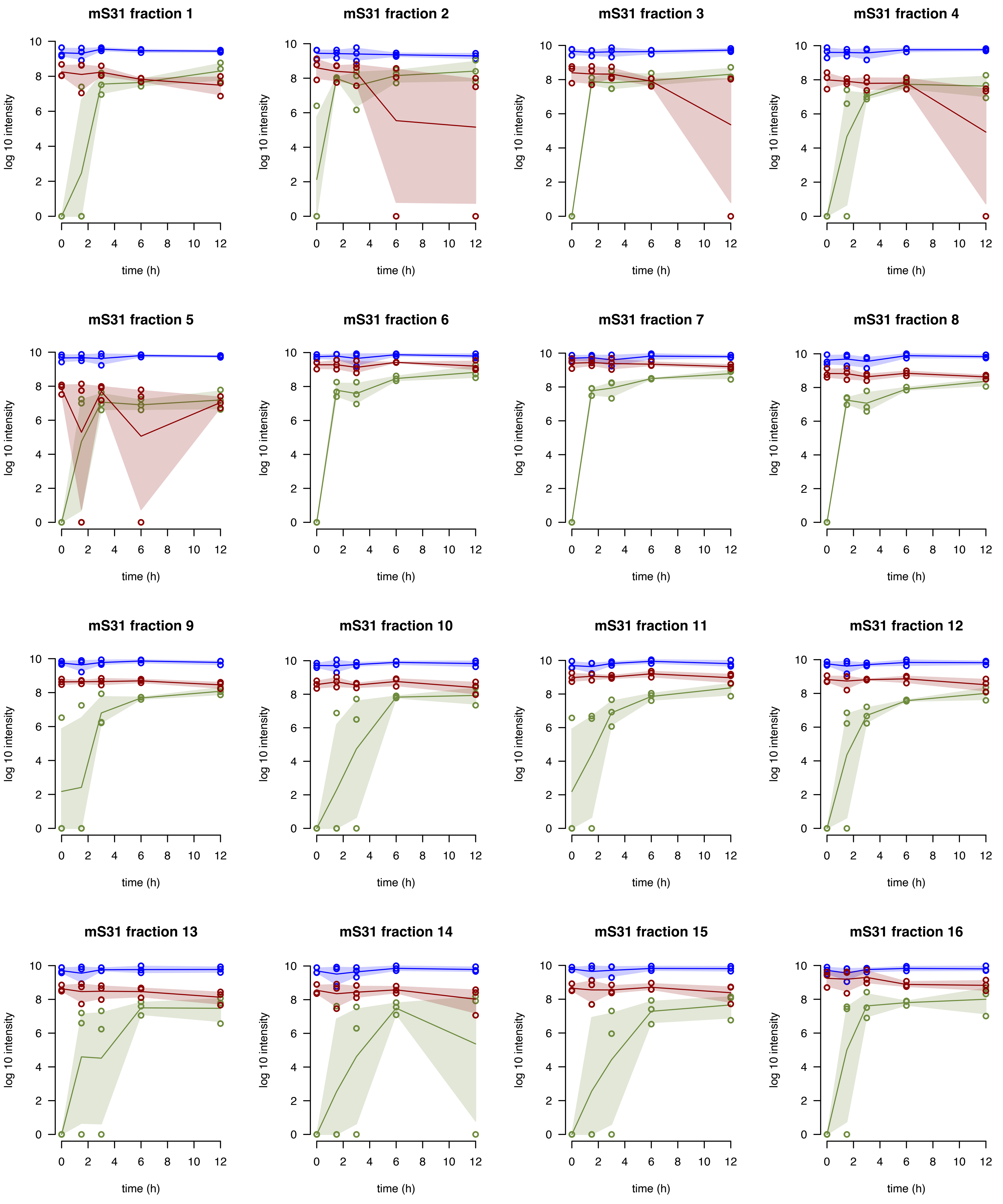
mS31 fraction 1
mS31 fraction 2
mS31 fraction 3
mS31 fraction 4
mS31 fraction 5
mS31 fraction 6
mS31 fraction 7
mS31 fraction 8
mS31 fraction 9
mS31 fraction 10
mS31 fraction 11
mS31 fraction 12
mS31 fraction 13
mS31 fraction 14
mS31 fraction 15
mS31 fraction 16
log 10 intensity
time (h)
0 2 4 6 8 10
0 2 4 6 8 10 12

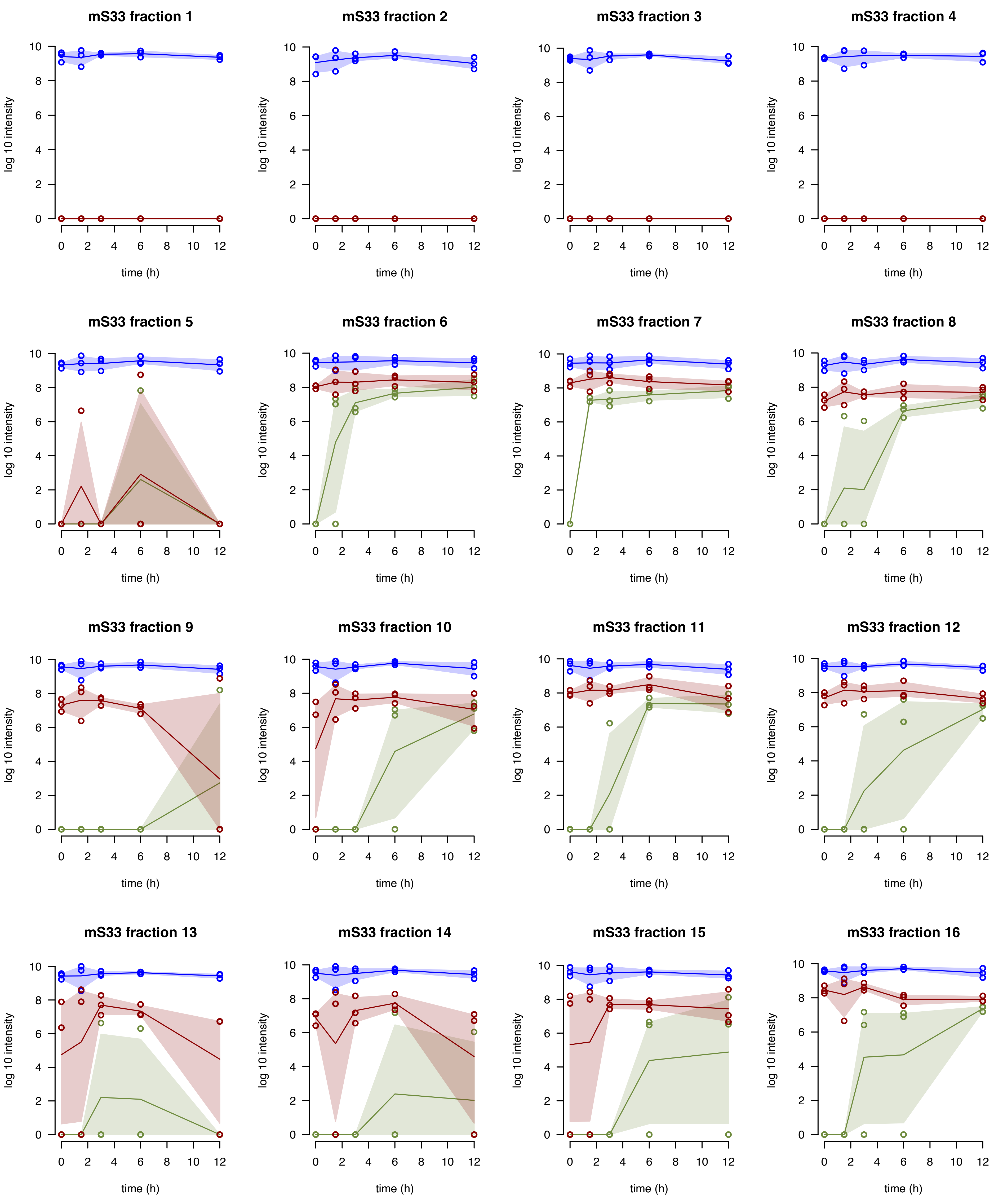

mS33 fraction 1
mS33 fraction 2
mS33 fraction 3
mS33 fraction 4
mS33 fraction 5
mS33 fraction 6
mS33 fraction 7
mS33 fraction 8
mS33 fraction 9
mS33 fraction 10
mS33 fraction 11
mS33 fraction 12
mS33 fraction 13
mS33 fraction 14
mS33 fraction 15
mS33 fraction 16
log 10 intensity
time (h)
0
2
4
6
8
10
12

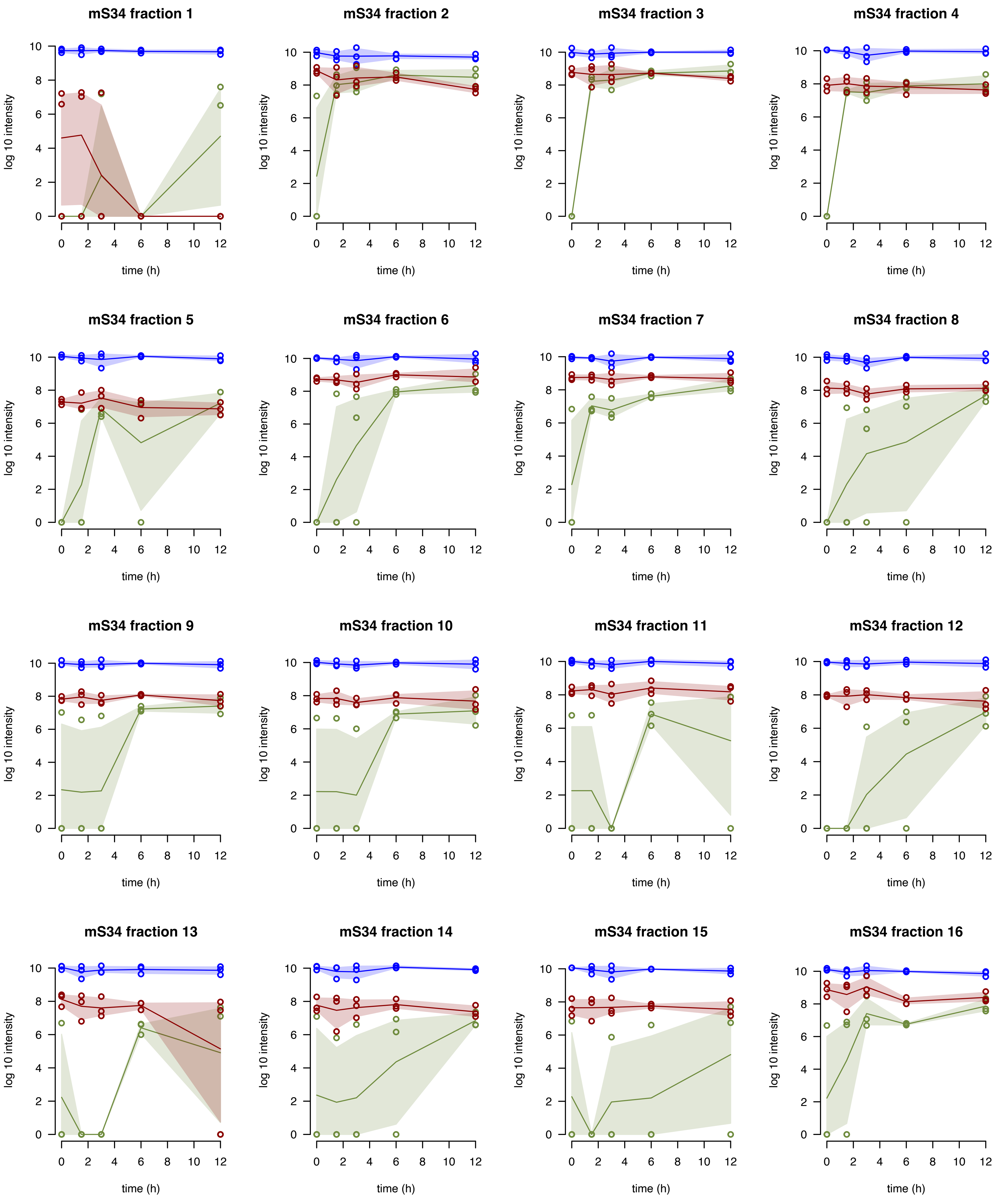

mS34 fraction 1
log 10 intensity
time (h)
mS34 fraction 2
log 10 intensity
time (h)
mS34 fraction 3
log 10 intensity
time (h)
mS34 fraction 4
log 10 intensity
time (h)
mS34 fraction 5
log 10 intensity
time (h)
mS34 fraction 6
log 10 intensity
time (h)
mS34 fraction 7
log 10 intensity
time (h)
mS34 fraction 8
log 10 intensity
time (h)
mS34 fraction 9
log 10 intensity
time (h)
mS34 fraction 10
log 10 intensity
time (h)
mS34 fraction 11
log 10 intensity
time (h)
mS34 fraction 12
log 10 intensity
time (h)
mS34 fraction 13
log 10 intensity
time (h)
mS34 fraction 14
log 10 intensity
time (h)
mS34 fraction 15
log 10 intensity
time (h)
mS34 fraction 16
log 10 intensity
time (h)

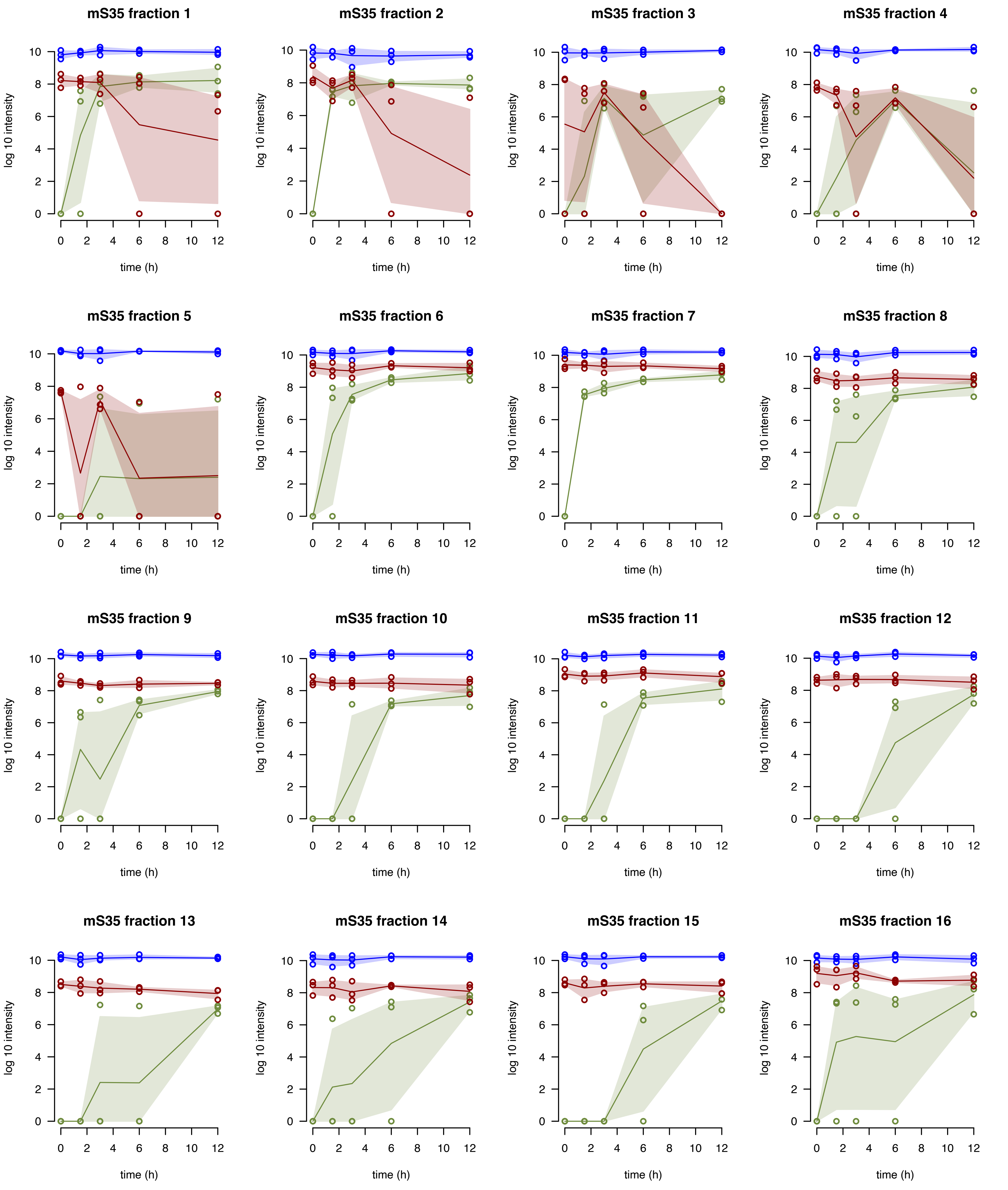
mS35 fraction 1
mS35 fraction 2
mS35 fraction 3
mS35 fraction 4
mS35 fraction 5
mS35 fraction 6
mS35 fraction 7
mS35 fraction 8
mS35 fraction 9
mS35 fraction 10
mS35 fraction 11
mS35 fraction 12
mS35 fraction 13
mS35 fraction 14
mS35 fraction 15
mS35 fraction 16
log 10 intensity
time (h)
0 2 4 6 8 10 12
0 2 4 6 8 10

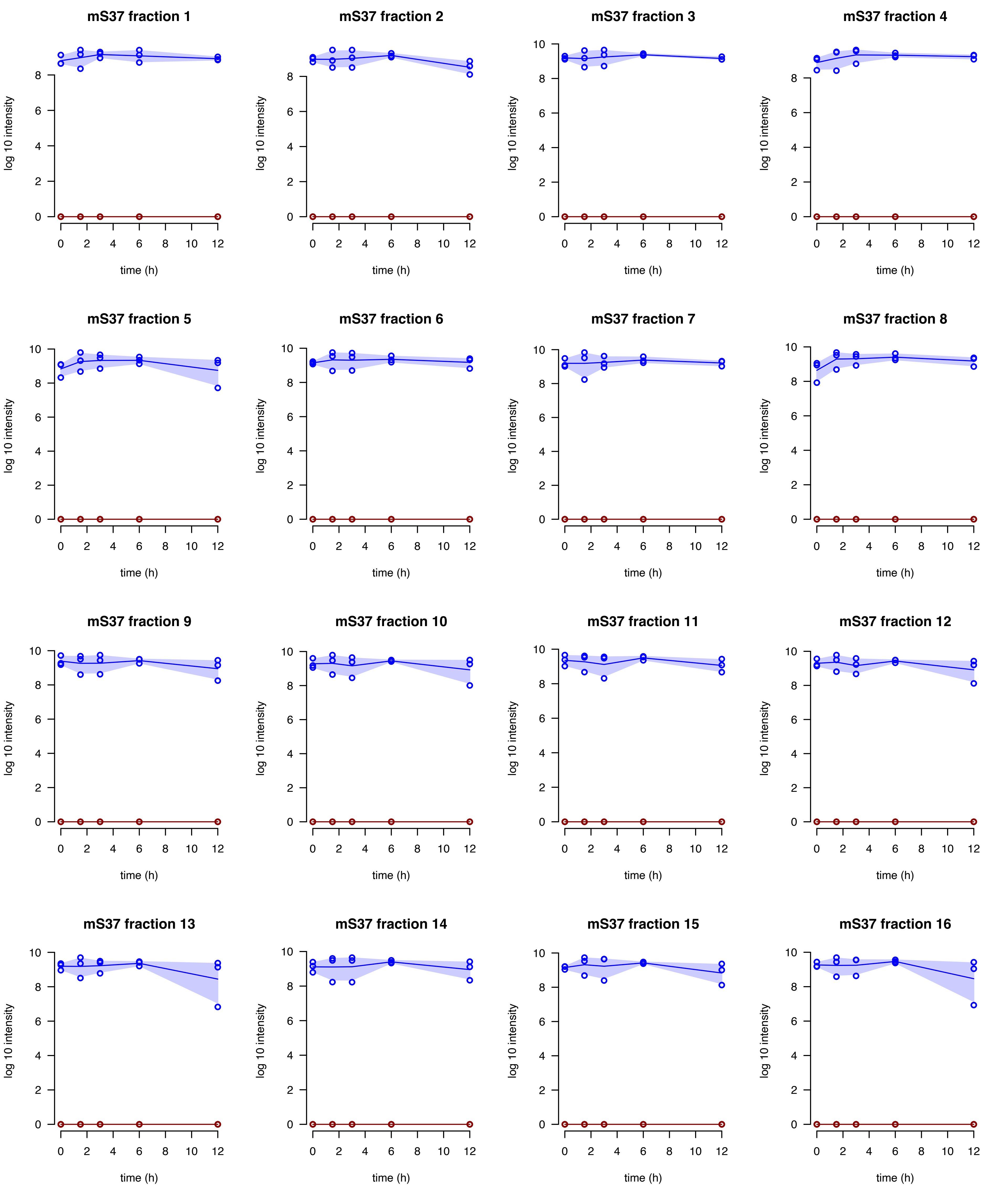

mS37 fraction 1
mS37 fraction 2
mS37 fraction 3
mS37 fraction 4
mS37 fraction 5
mS37 fraction 6
mS37 fraction 7
mS37 fraction 8
mS37 fraction 9
mS37 fraction 10
mS37 fraction 11
mS37 fraction 12
mS37 fraction 13
mS37 fraction 14
mS37 fraction 15
mS37 fraction 16
log 10 intensity
time (h)
0
2
4
6
8
10
12

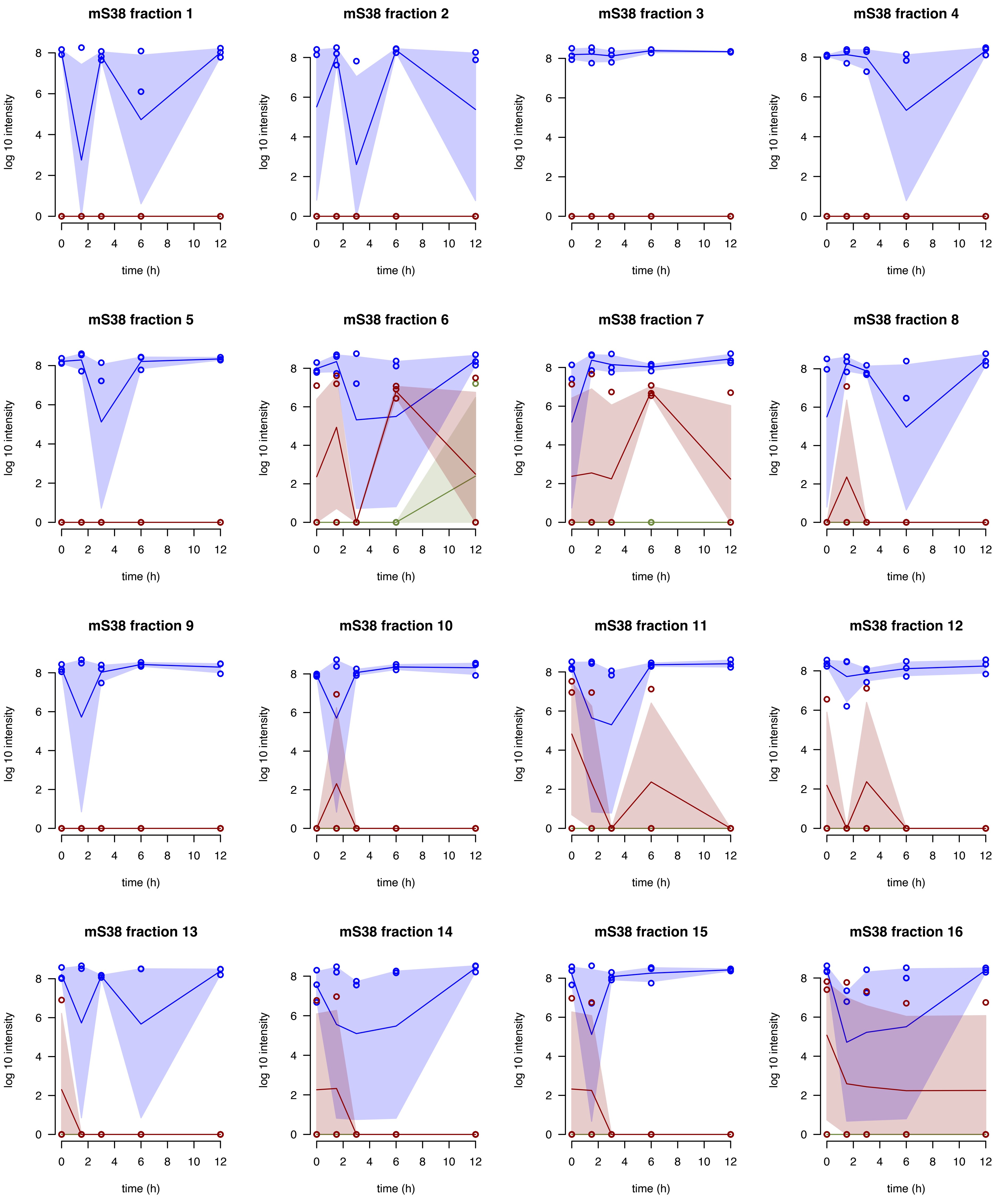
mS38 fraction 1
mS38 fraction 2
mS38 fraction 3
mS38 fraction 4
mS38 fraction 5
mS38 fraction 6
mS38 fraction 7
mS38 fraction 8
mS38 fraction 9
mS38 fraction 10
mS38 fraction 11
mS38 fraction 12
mS38 fraction 13
mS38 fraction 14
mS38 fraction 15
mS38 fraction 16
log 10 intensity
time (h)
0
2
4
6
8
10
12

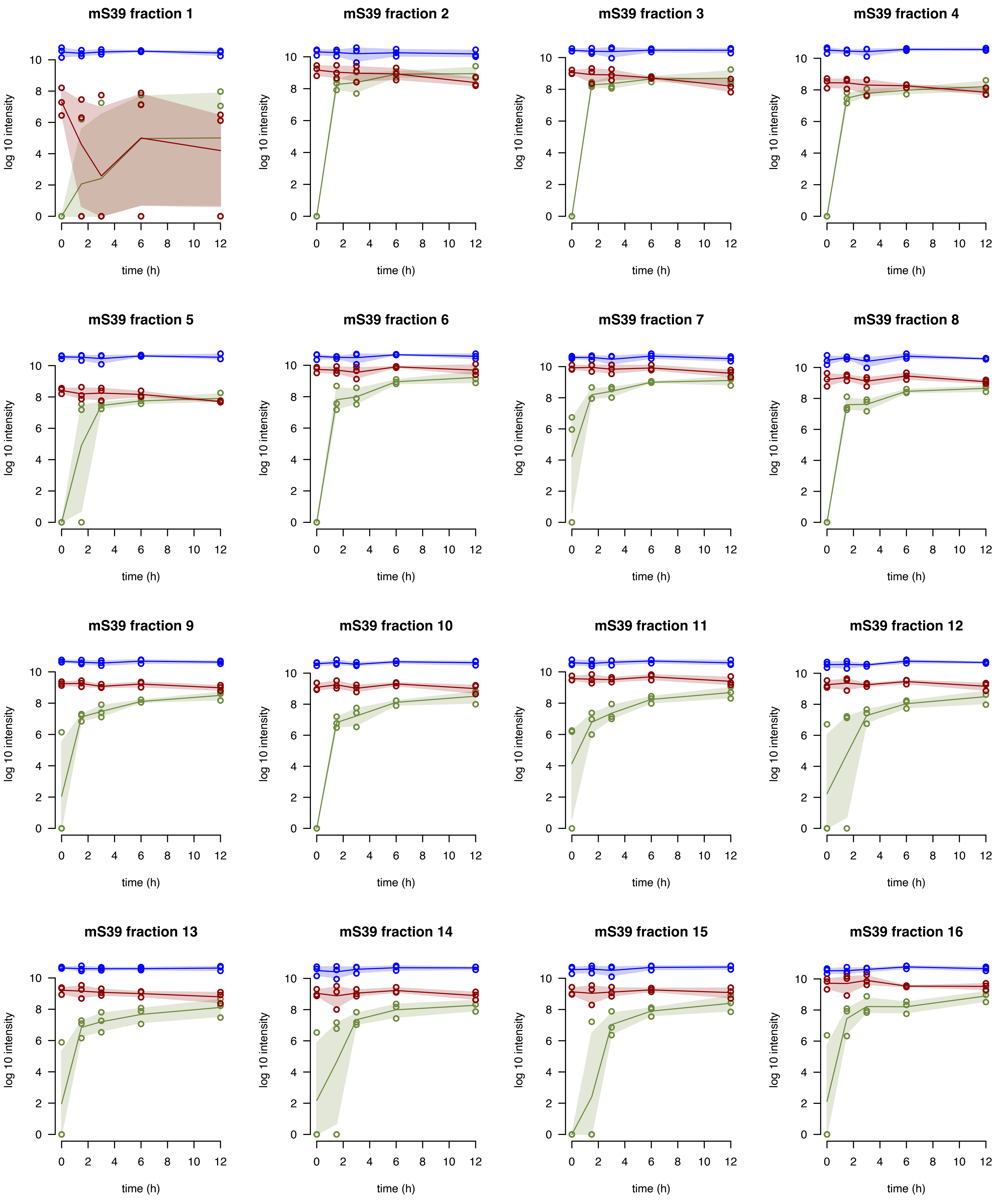

mS39 fraction 1
mS39 fraction 2
mS39 fraction 3
mS39 fraction 4
mS39 fraction 5
mS39 fraction 6
mS39 fraction 7
mS39 fraction 8
mS39 fraction 9
mS39 fraction 10
mS39 fraction 11
mS39 fraction 12
mS39 fraction 13
mS39 fraction 14
mS39 fraction 15
mS39 fraction 16
log 10 intensity
time (h)

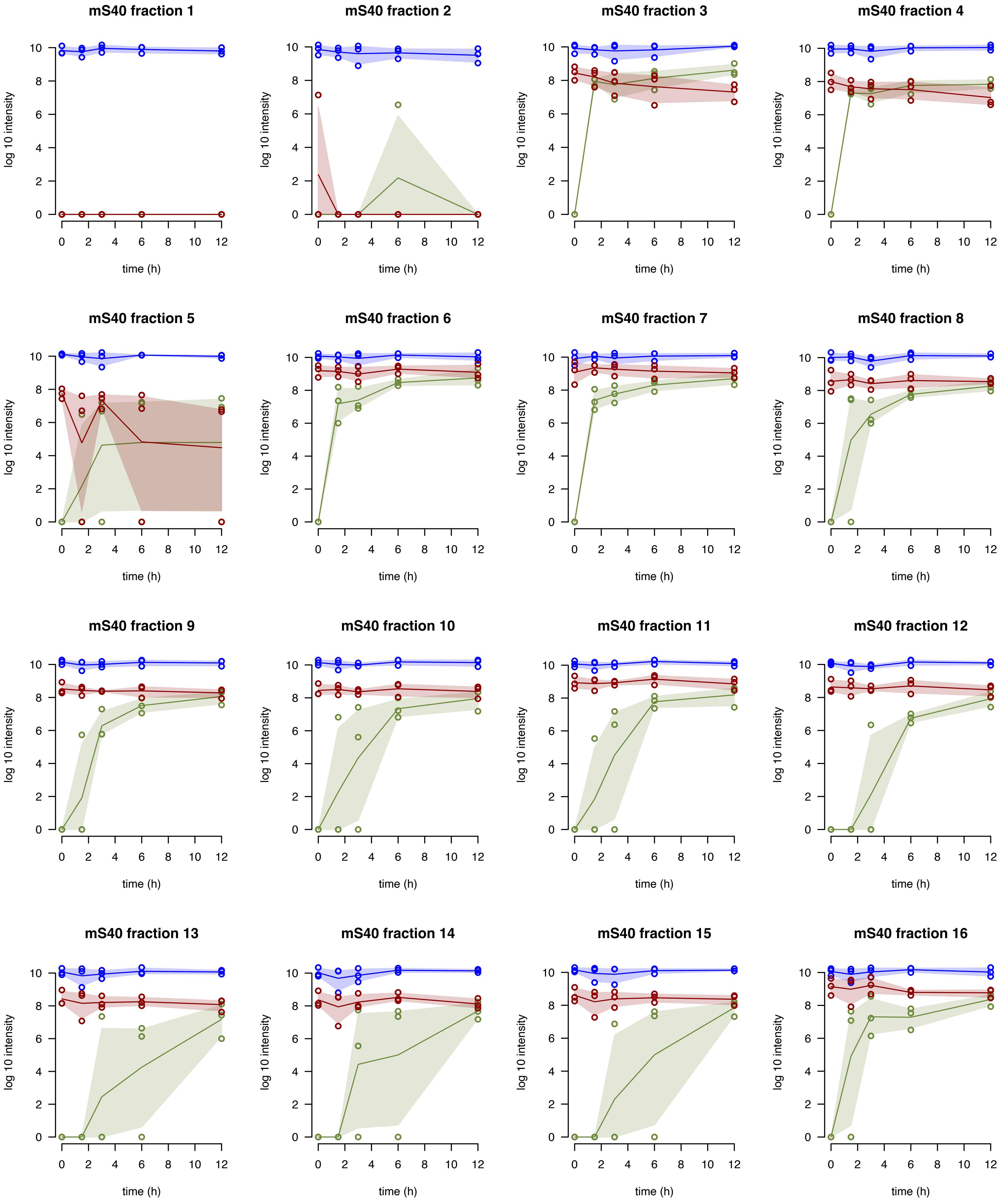
mS40 fraction 1
mS40 fraction 2
mS40 fraction 3
mS40 fraction 4
mS40 fraction 5
mS40 fraction 6
mS40 fraction 7
mS40 fraction 8
mS40 fraction 9
mS40 fraction 10
mS40 fraction 11
mS40 fraction 12
mS40 fraction 13
mS40 fraction 14
mS40 fraction 15
mS40 fraction 16
log 10 intensity
time (h)
