# Supplementary material for: A roadmap for ribosome assembly in human mitochondria
Source: Nat Struct Mol Biol. 2024 Jul 11;31(12):1898–908. doi: 10.1038/s41594-024-01356-w (PMC11638073; doi:10.1038/s41594-024-01356-w)
Supplement: Supplementary file 6 — Visualization of mtLSU MRP’s MS RAW data across sucrose gradient fractions. MS1 raw intensities before normalization for all H (red), M (green) and L (blue) labeled MRPs of the mtLSU over the chase time of 12 h for all collected 16 sucrose gradient fractions. [file 41594_2024_1356_MOESM6_ESM.pdf]

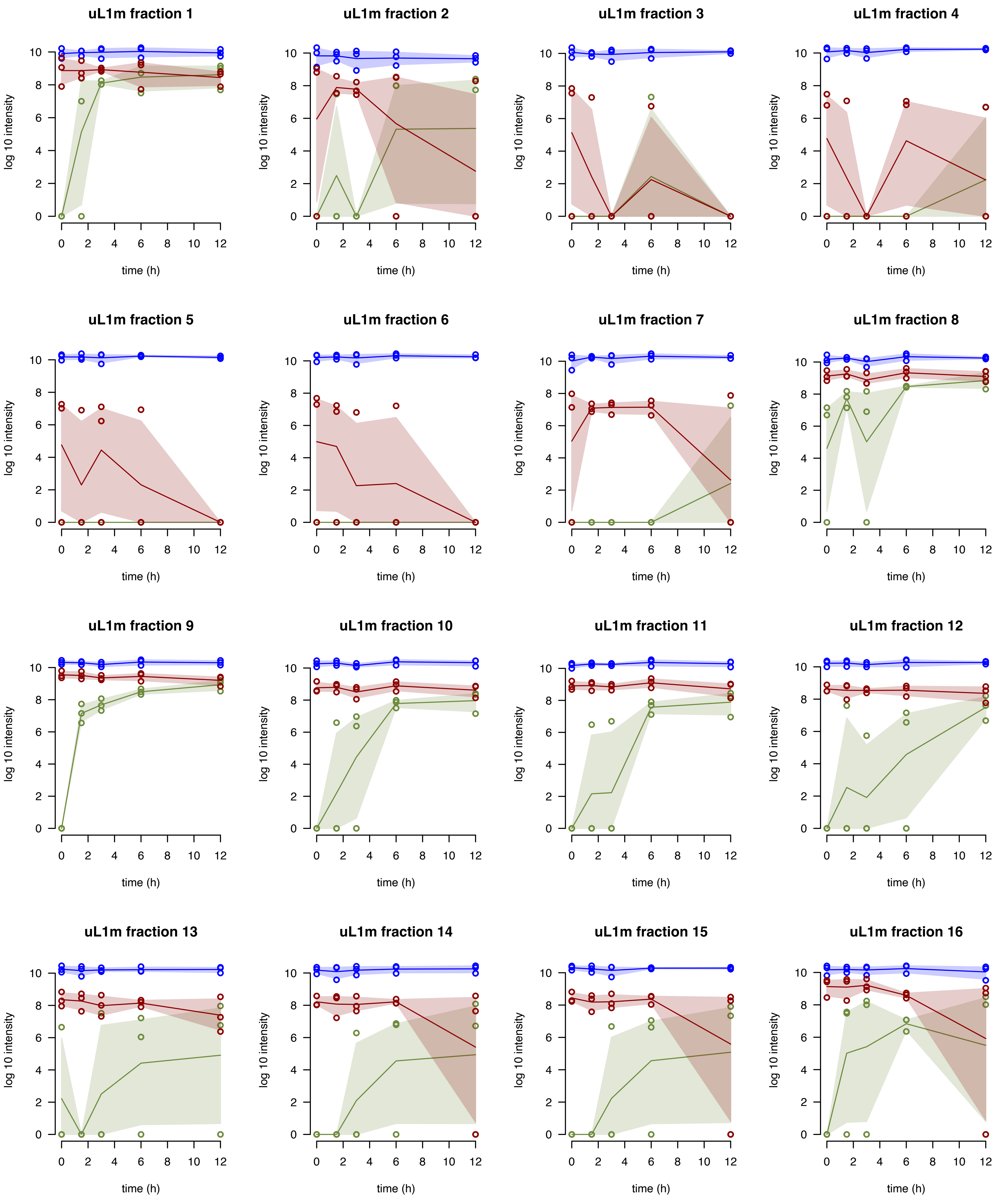
uL1m fraction 1
uL1m fraction 2
uL1m fraction 3
uL1m fraction 4
uL1m fraction 5
uL1m fraction 6
uL1m fraction 7
uL1m fraction 8
uL1m fraction 9
uL1m fraction 10
uL1m fraction 11
uL1m fraction 12
uL1m fraction 13
uL1m fraction 14
uL1m fraction 15
uL1m fraction 16
log 10 intensity
time (h)

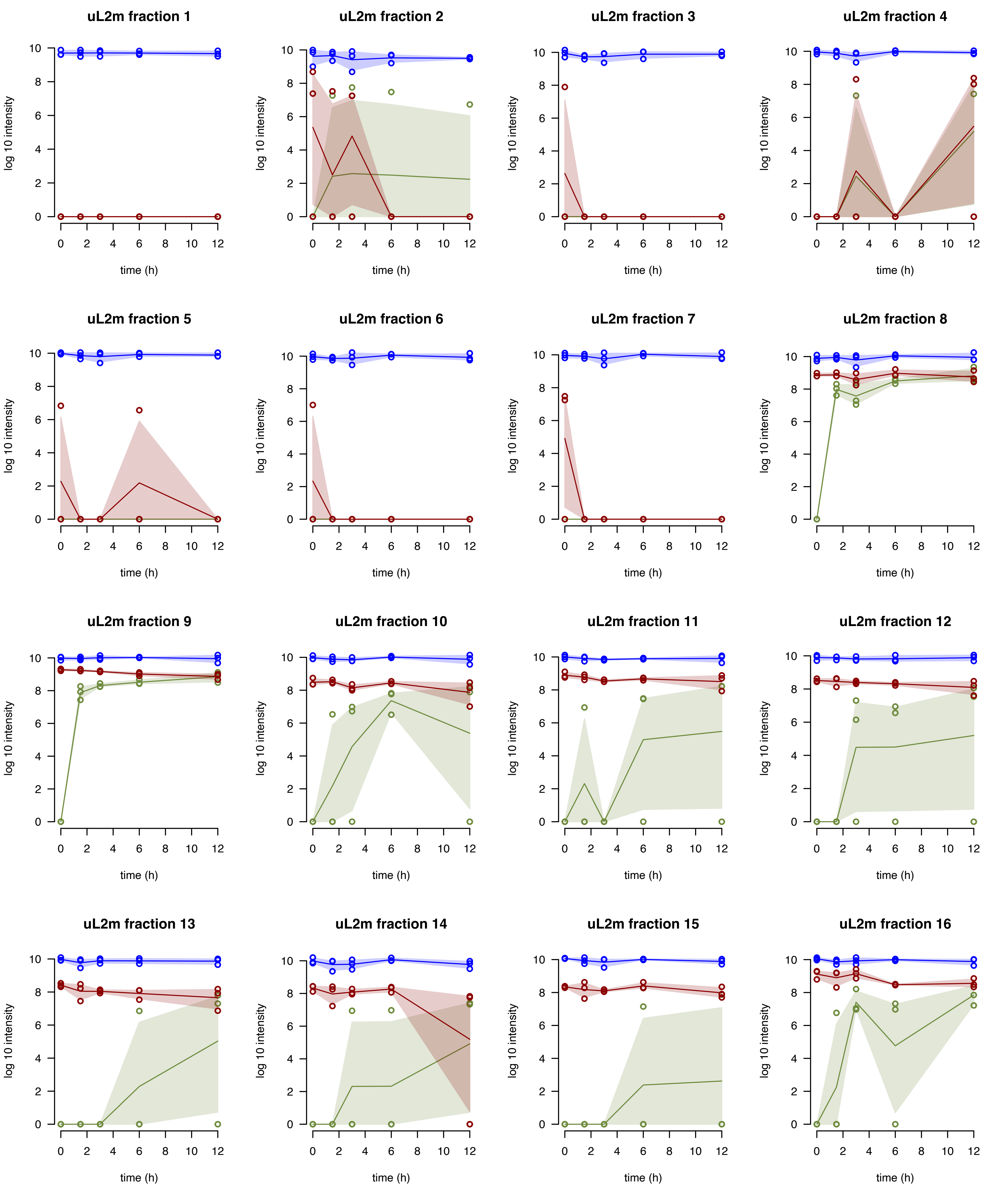
uL2m fraction 1
uL2m fraction 2
uL2m fraction 3
uL2m fraction 4
uL2m fraction 5
uL2m fraction 6
uL2m fraction 7
uL2m fraction 8
uL2m fraction 9
uL2m fraction 10
uL2m fraction 11
uL2m fraction 12
uL2m fraction 13
uL2m fraction 14
uL2m fraction 15
uL2m fraction 16
log 10 intensity
time (h)
0
2
4
6
8
10
12

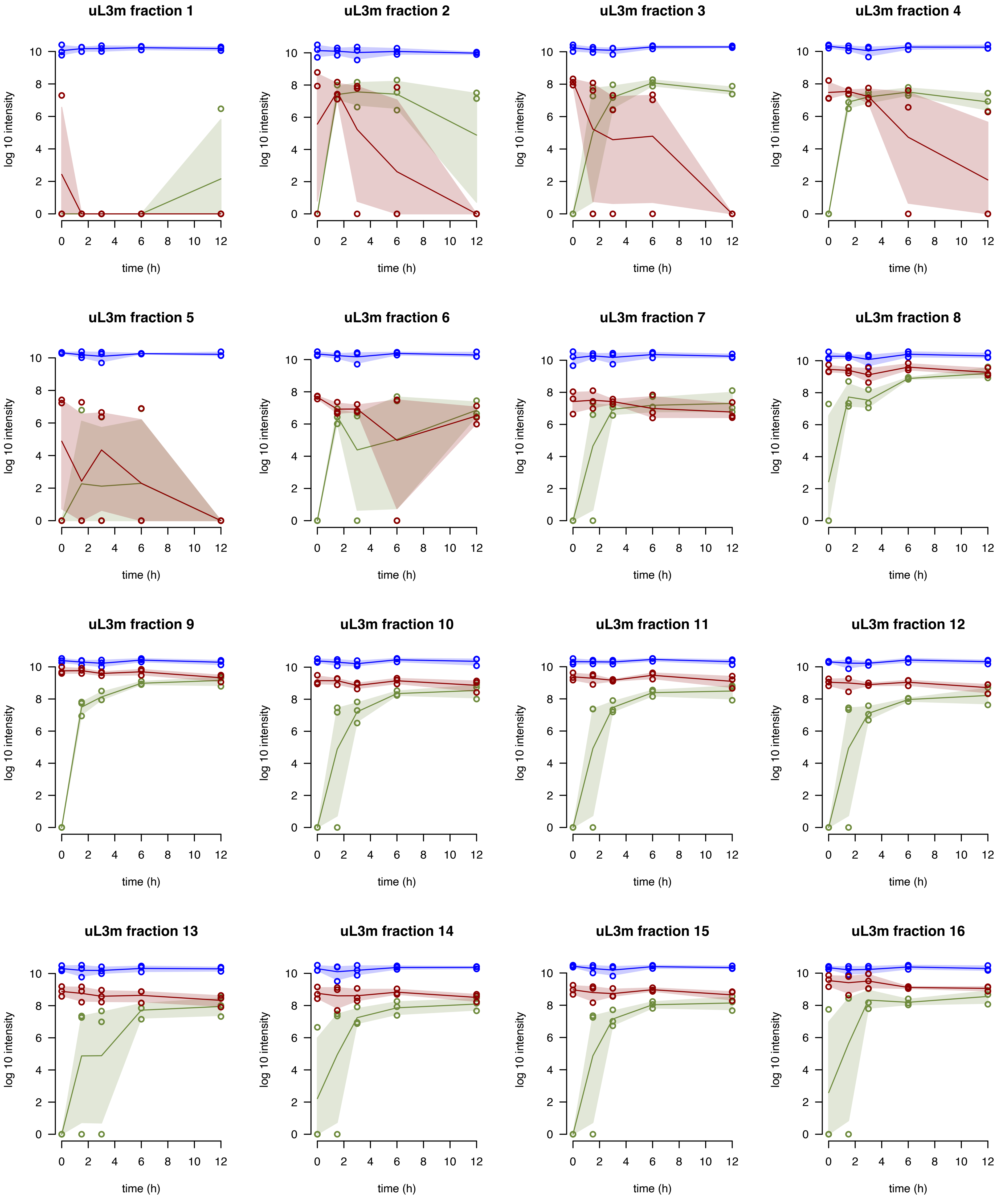
uL3m fraction 1
uL3m fraction 2
uL3m fraction 3
uL3m fraction 4
uL3m fraction 5
uL3m fraction 6
uL3m fraction 7
uL3m fraction 8
uL3m fraction 9
uL3m fraction 10
uL3m fraction 11
uL3m fraction 12
uL3m fraction 13
uL3m fraction 14
uL3m fraction 15
uL3m fraction 16
log 10 intensity
time (h)

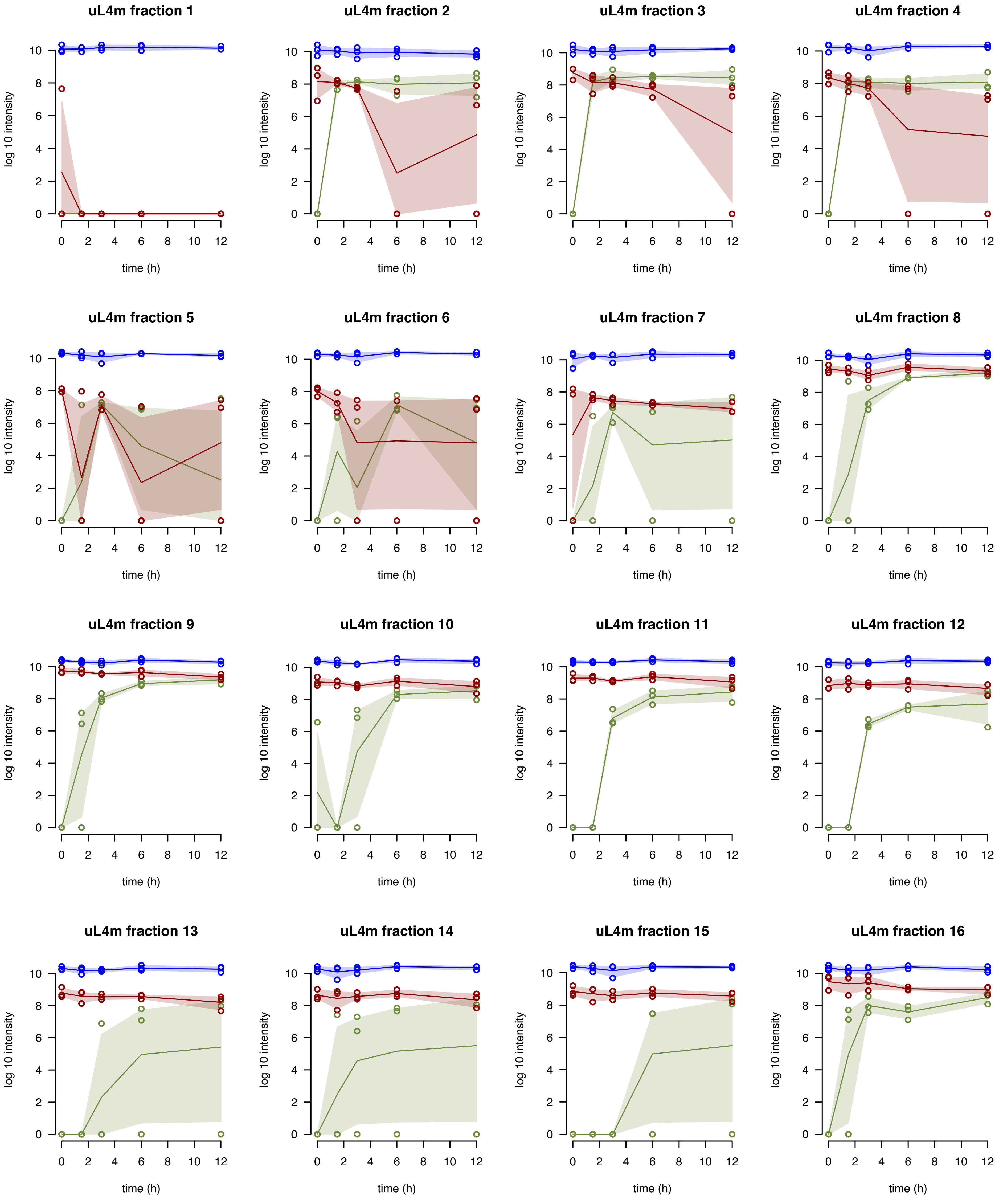
uL4m fraction 1
uL4m fraction 2
uL4m fraction 3
uL4m fraction 4
uL4m fraction 5
uL4m fraction 6
uL4m fraction 7
uL4m fraction 8
uL4m fraction 9
uL4m fraction 10
uL4m fraction 11
uL4m fraction 12
uL4m fraction 13
uL4m fraction 14
uL4m fraction 15
uL4m fraction 16
log 10 intensity
time (h)
0
2
4
6
8
10
12

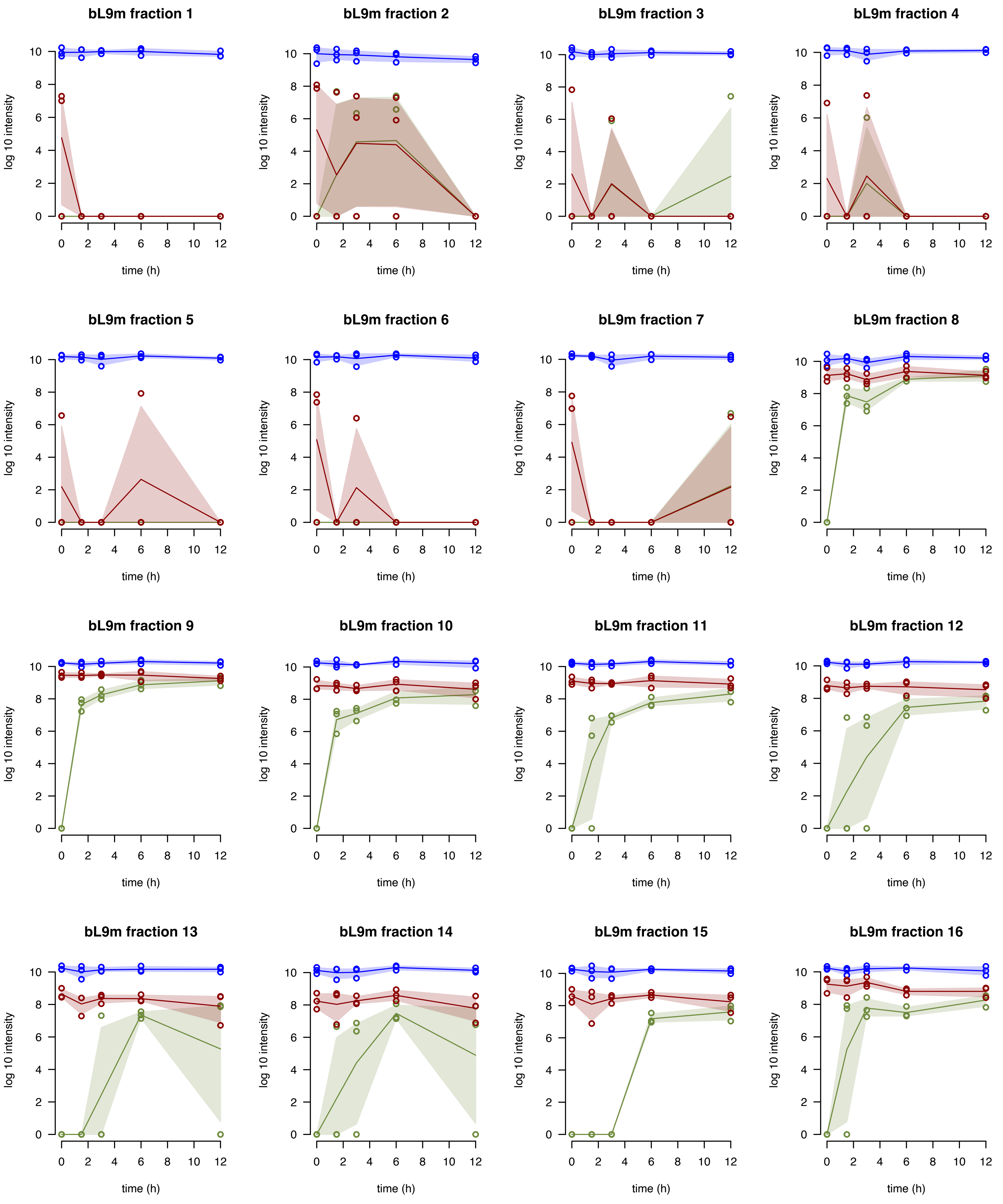
bL9m fraction 1
bL9m fraction 2
bL9m fraction 3
bL9m fraction 4
bL9m fraction 5
bL9m fraction 6
bL9m fraction 7
bL9m fraction 8
bL9m fraction 9
bL9m fraction 10
bL9m fraction 11
bL9m fraction 12
bL9m fraction 13
bL9m fraction 14
bL9m fraction 15
bL9m fraction 16
log 10 intensity
time (h)
0
2
4
6
8
10
12

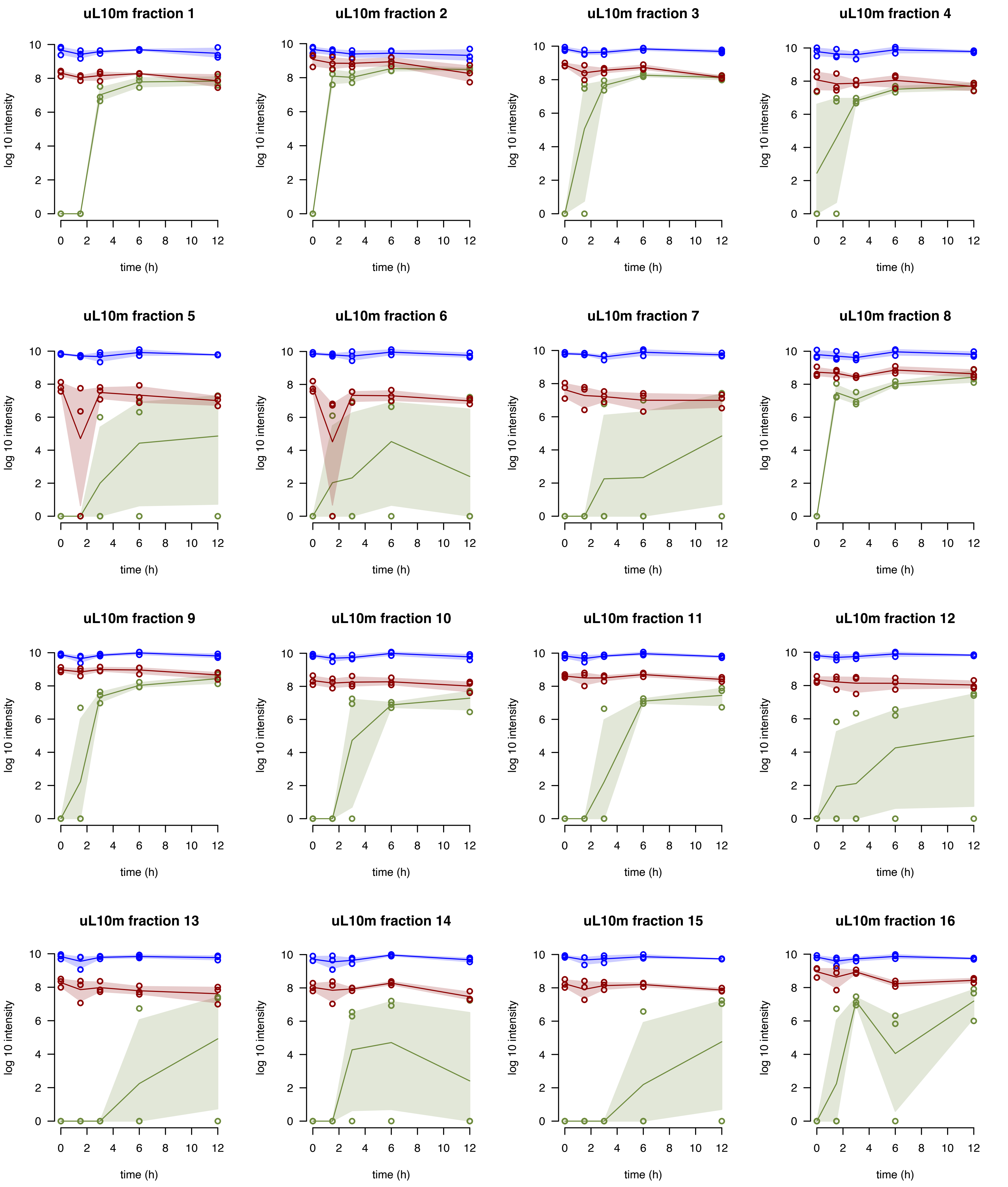

uL10m fraction 1
uL10m fraction 2
uL10m fraction 3
uL10m fraction 4
uL10m fraction 5
uL10m fraction 6
uL10m fraction 7
uL10m fraction 8
uL10m fraction 9
uL10m fraction 10
uL10m fraction 11
uL10m fraction 12
uL10m fraction 13
uL10m fraction 14
uL10m fraction 15
uL10m fraction 16
log 10 intensity
time (h)
0 2 4 6 8 10 12
0 2 4 6 8 10

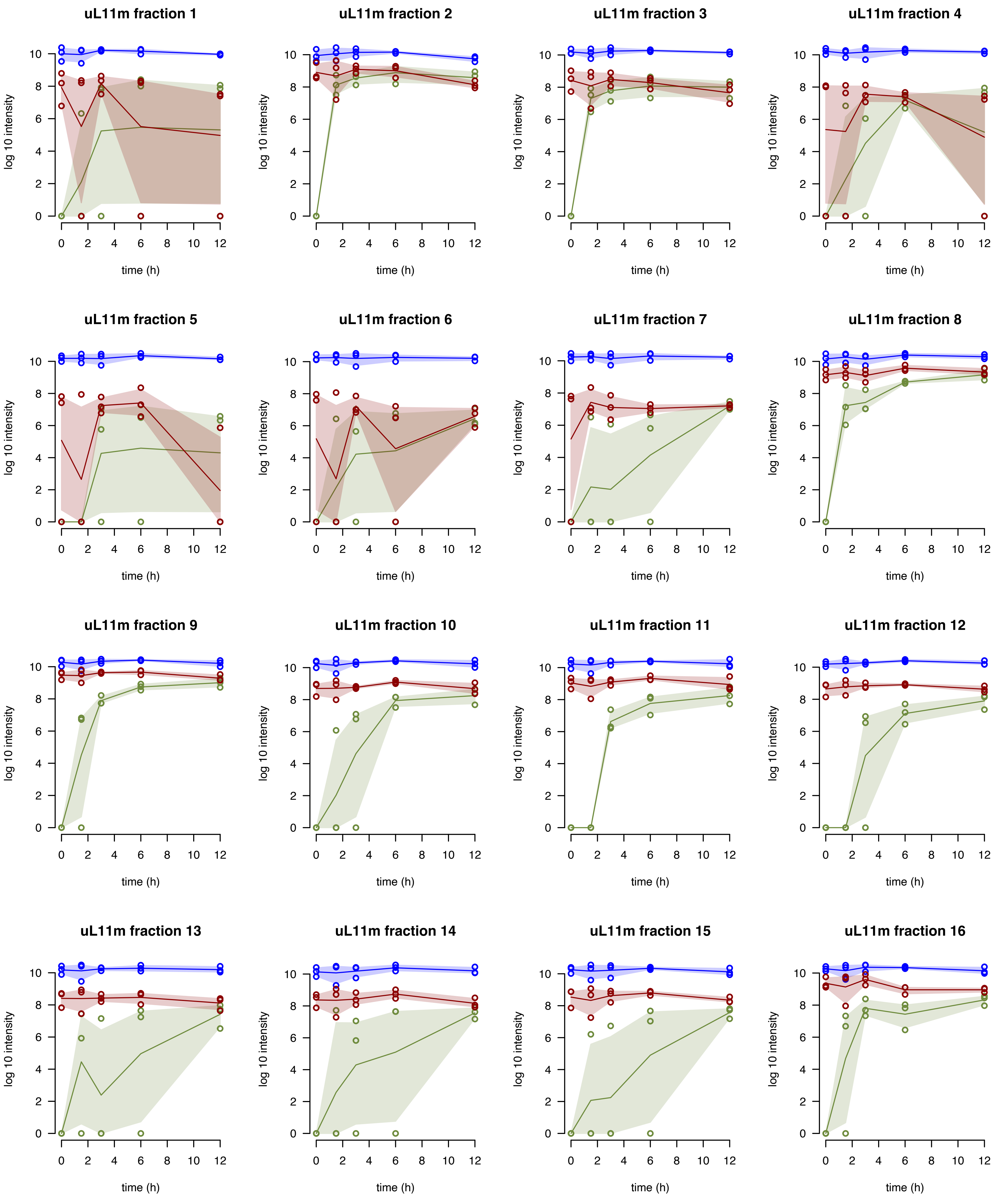
uL11m fraction 1
uL11m fraction 2
uL11m fraction 3
uL11m fraction 4
uL11m fraction 5
uL11m fraction 6
uL11m fraction 7
uL11m fraction 8
uL11m fraction 9
uL11m fraction 10
uL11m fraction 11
uL11m fraction 12
uL11m fraction 13
uL11m fraction 14
uL11m fraction 15
uL11m fraction 16
log 10 intensity
0
2
4
6
8
10
time (h)
0
2
4
6
8
10
12

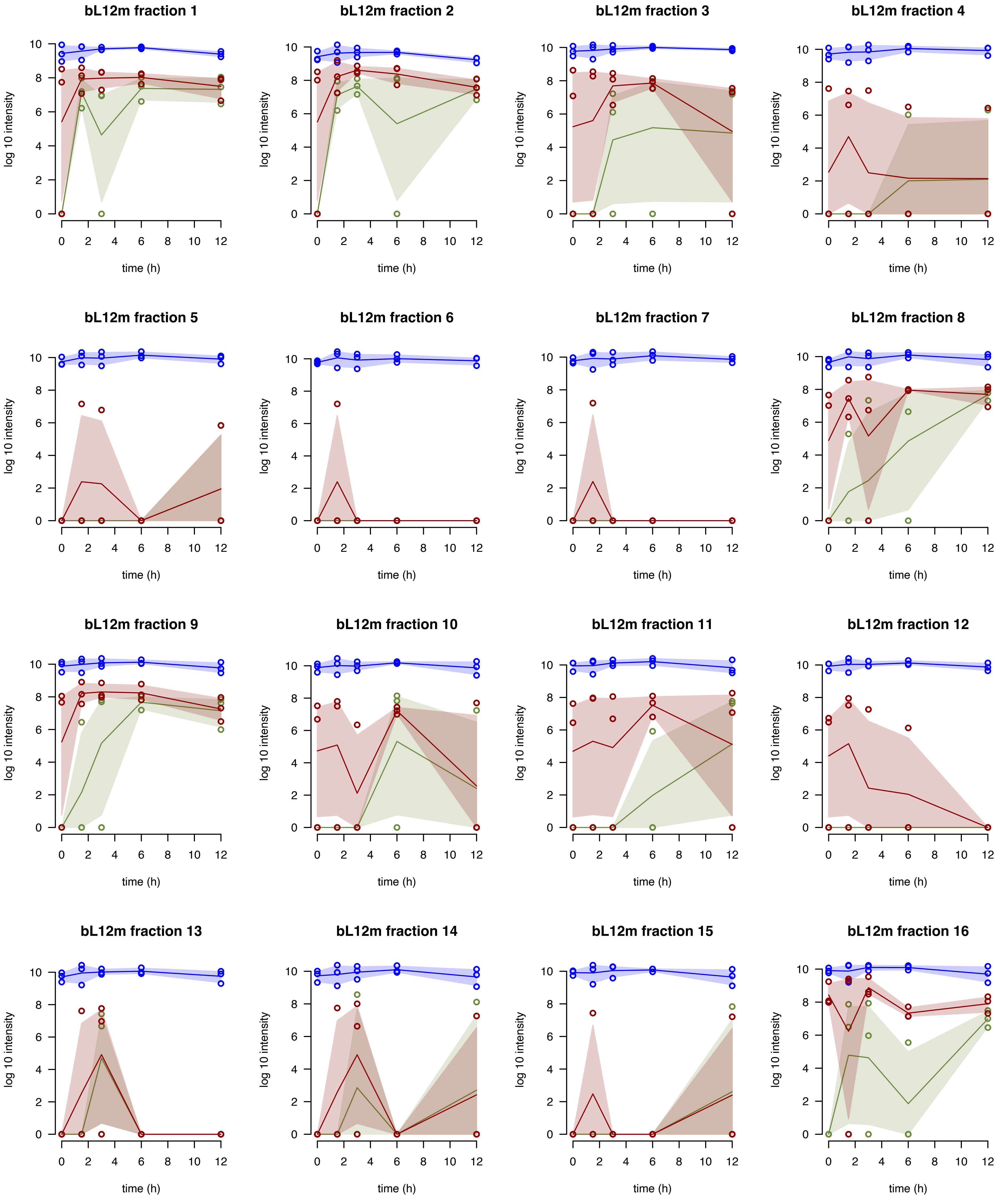
bL12m fraction 1
bL12m fraction 2
bL12m fraction 3
bL12m fraction 4
bL12m fraction 5
bL12m fraction 6
bL12m fraction 7
bL12m fraction 8
bL12m fraction 9
bL12m fraction 10
bL12m fraction 11
bL12m fraction 12
bL12m fraction 13
bL12m fraction 14
bL12m fraction 15
bL12m fraction 16
log 10 intensity
time (h)
0 2 4 6 8 10 12
0 2 4 6 8 10

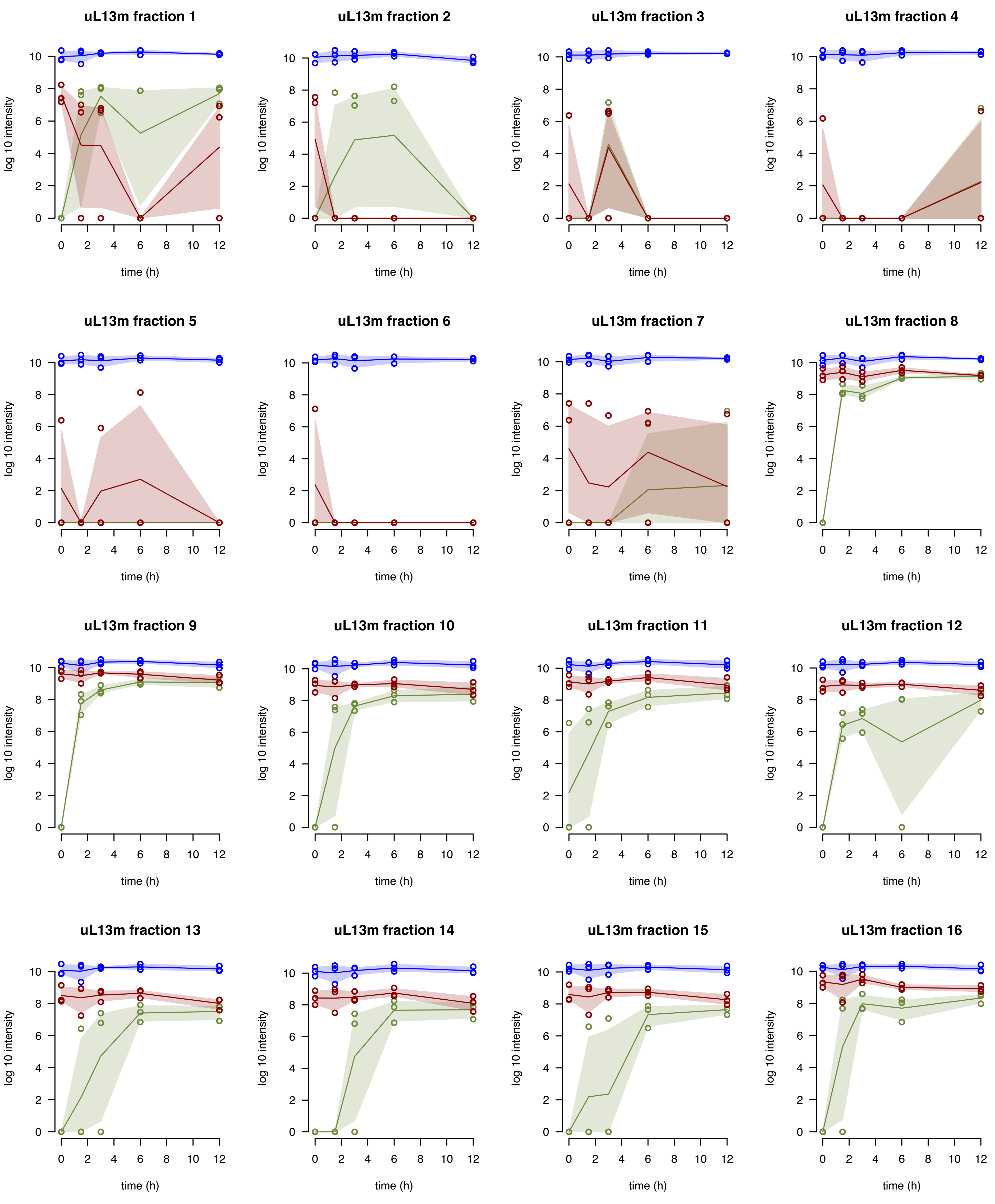
uL13m fraction 1
uL13m fraction 2
uL13m fraction 3
uL13m fraction 4
uL13m fraction 5
uL13m fraction 6
uL13m fraction 7
uL13m fraction 8
uL13m fraction 9
uL13m fraction 10
uL13m fraction 11
uL13m fraction 12
uL13m fraction 13
uL13m fraction 14
uL13m fraction 15
uL13m fraction 16
log 10 intensity
time (h)
0
2
4
6
8
10
12

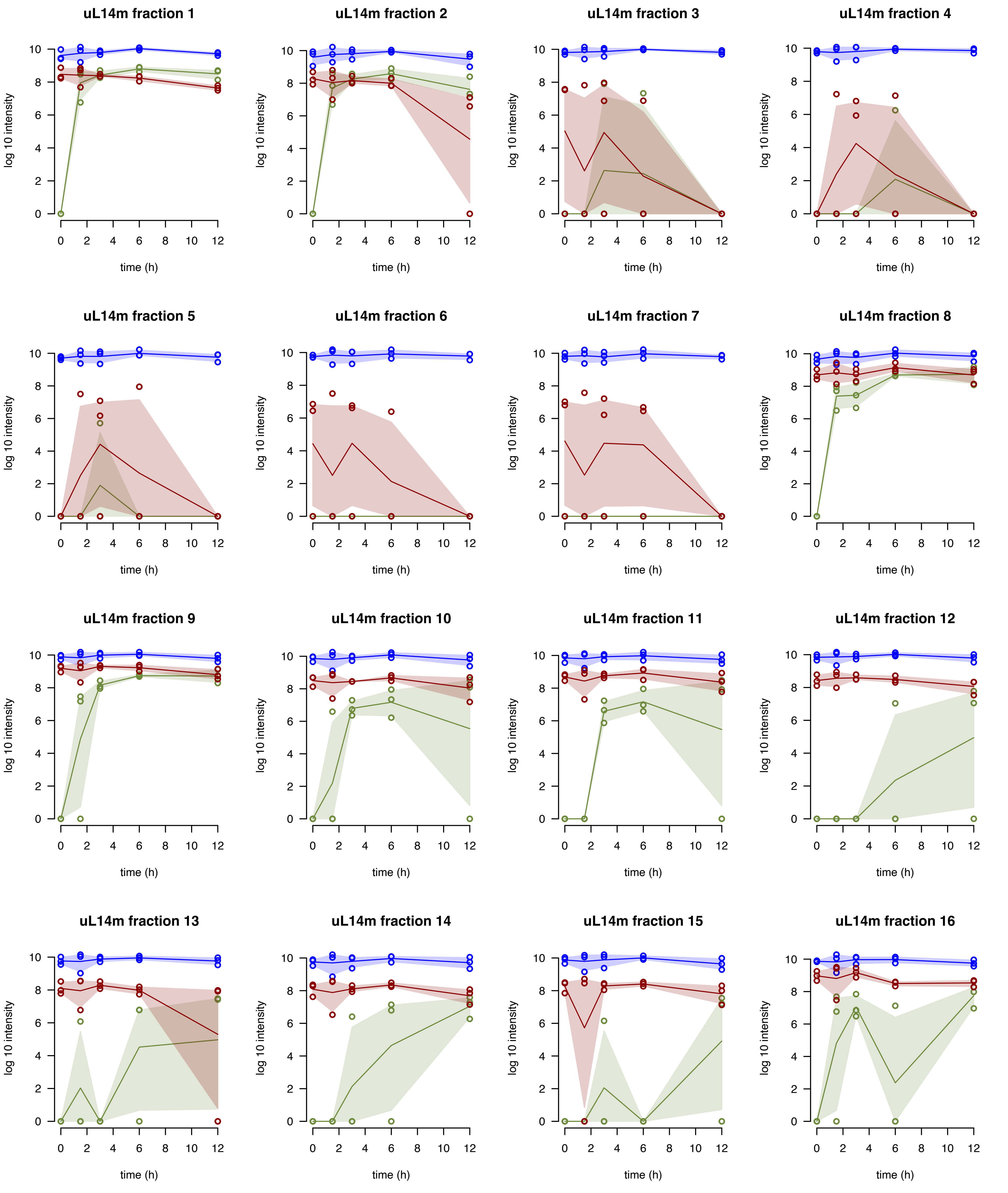
uL14m fraction 1
uL14m fraction 2
uL14m fraction 3
uL14m fraction 4
uL14m fraction 5
uL14m fraction 6
uL14m fraction 7
uL14m fraction 8
uL14m fraction 9
uL14m fraction 10
uL14m fraction 11
uL14m fraction 12
uL14m fraction 13
uL14m fraction 14
uL14m fraction 15
uL14m fraction 16
log 10 intensity
time (h)

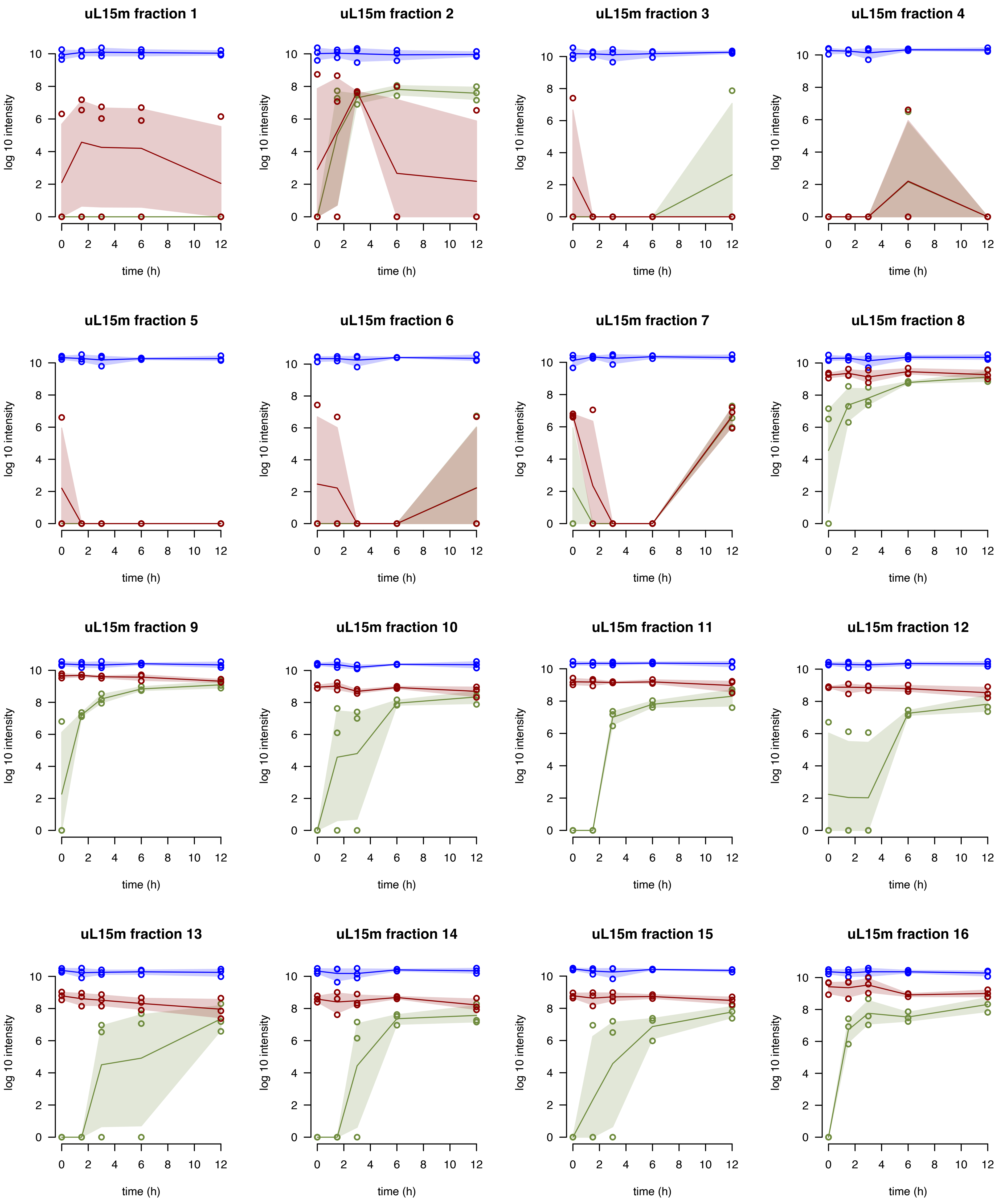

uL15m fraction 1
uL15m fraction 2
uL15m fraction 3
uL15m fraction 4
uL15m fraction 5
uL15m fraction 6
uL15m fraction 7
uL15m fraction 8
uL15m fraction 9
uL15m fraction 10
uL15m fraction 11
uL15m fraction 12
uL15m fraction 13
uL15m fraction 14
uL15m fraction 15
uL15m fraction 16
log 10 intensity
time (h)

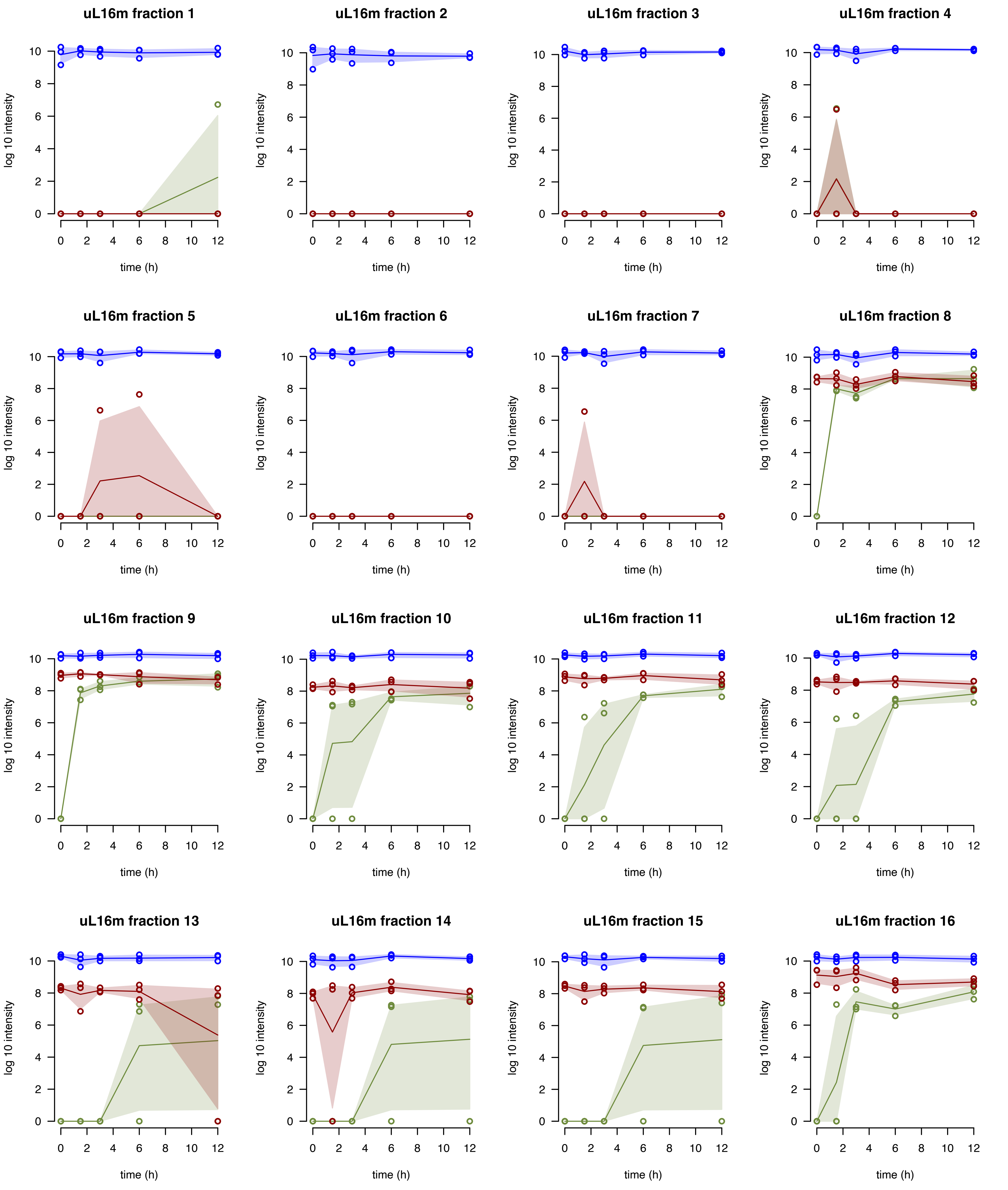
uL16m fraction 1
uL16m fraction 2
uL16m fraction 3
uL16m fraction 4
uL16m fraction 5
uL16m fraction 6
uL16m fraction 7
uL16m fraction 8
uL16m fraction 9
uL16m fraction 10
uL16m fraction 11
uL16m fraction 12
uL16m fraction 13
uL16m fraction 14
uL16m fraction 15
uL16m fraction 16
log 10 intensity
time (h)
0
2
4
6
8
10
12

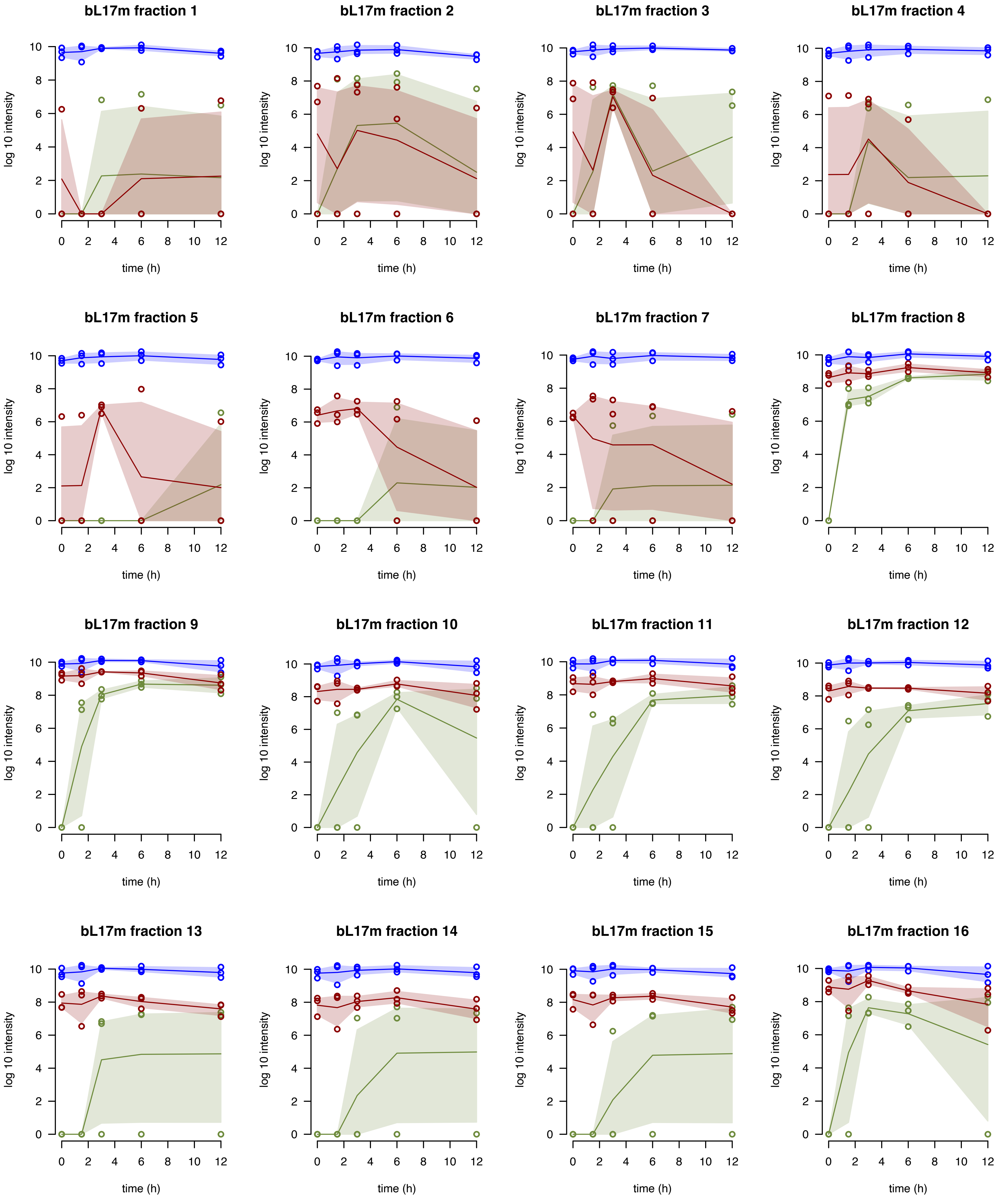
bL17m fraction 1
bL17m fraction 2
bL17m fraction 3
bL17m fraction 4
bL17m fraction 5
bL17m fraction 6
bL17m fraction 7
bL17m fraction 8
bL17m fraction 9
bL17m fraction 10
bL17m fraction 11
bL17m fraction 12
bL17m fraction 13
bL17m fraction 14
bL17m fraction 15
bL17m fraction 16
log 10 intensity
time (h)

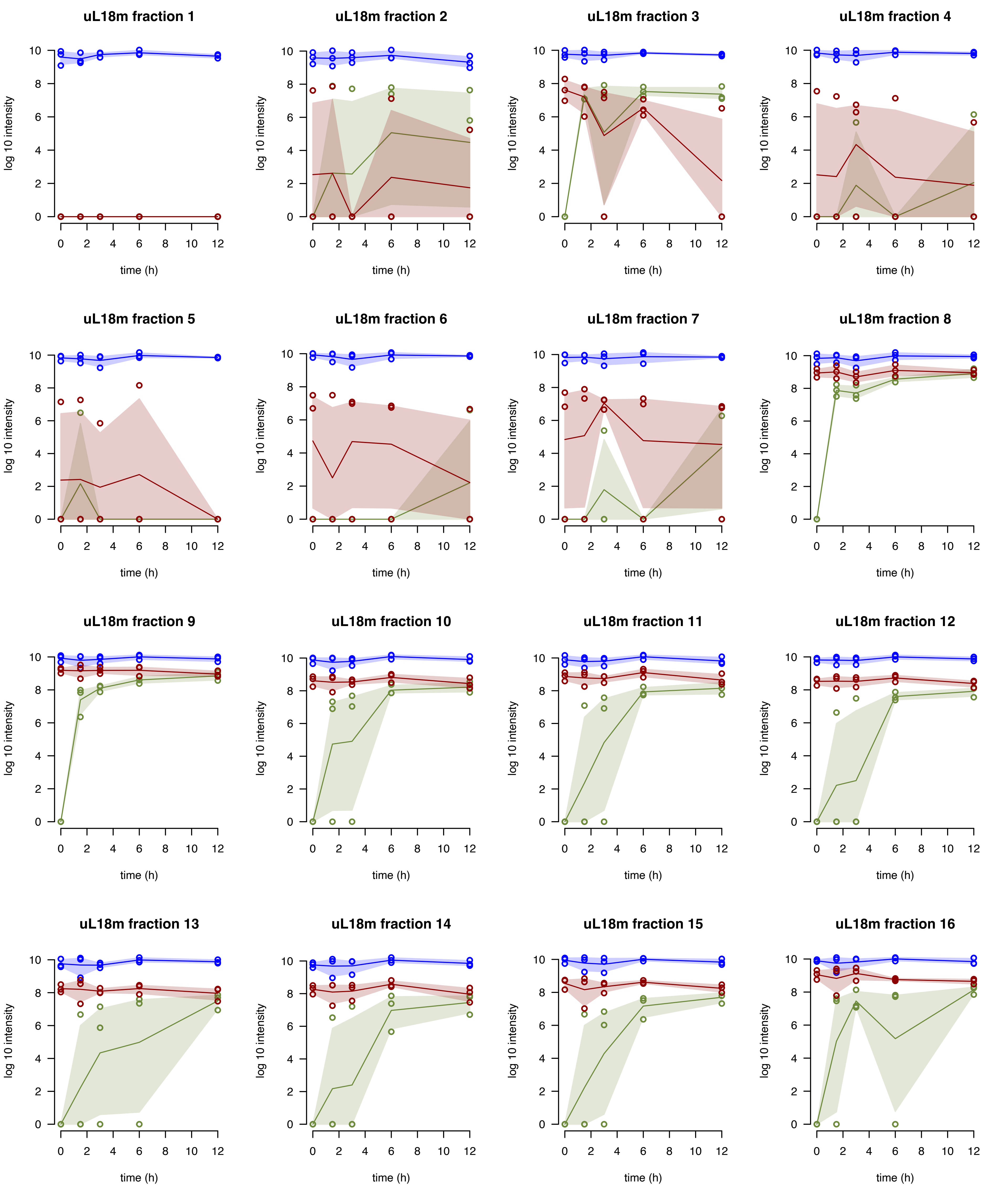

uL18m fraction 1
uL18m fraction 2
uL18m fraction 3
uL18m fraction 4
uL18m fraction 5
uL18m fraction 6
uL18m fraction 7
uL18m fraction 8
uL18m fraction 9
uL18m fraction 10
uL18m fraction 11
uL18m fraction 12
uL18m fraction 13
uL18m fraction 14
uL18m fraction 15
uL18m fraction 16
log 10 intensity
time (h)

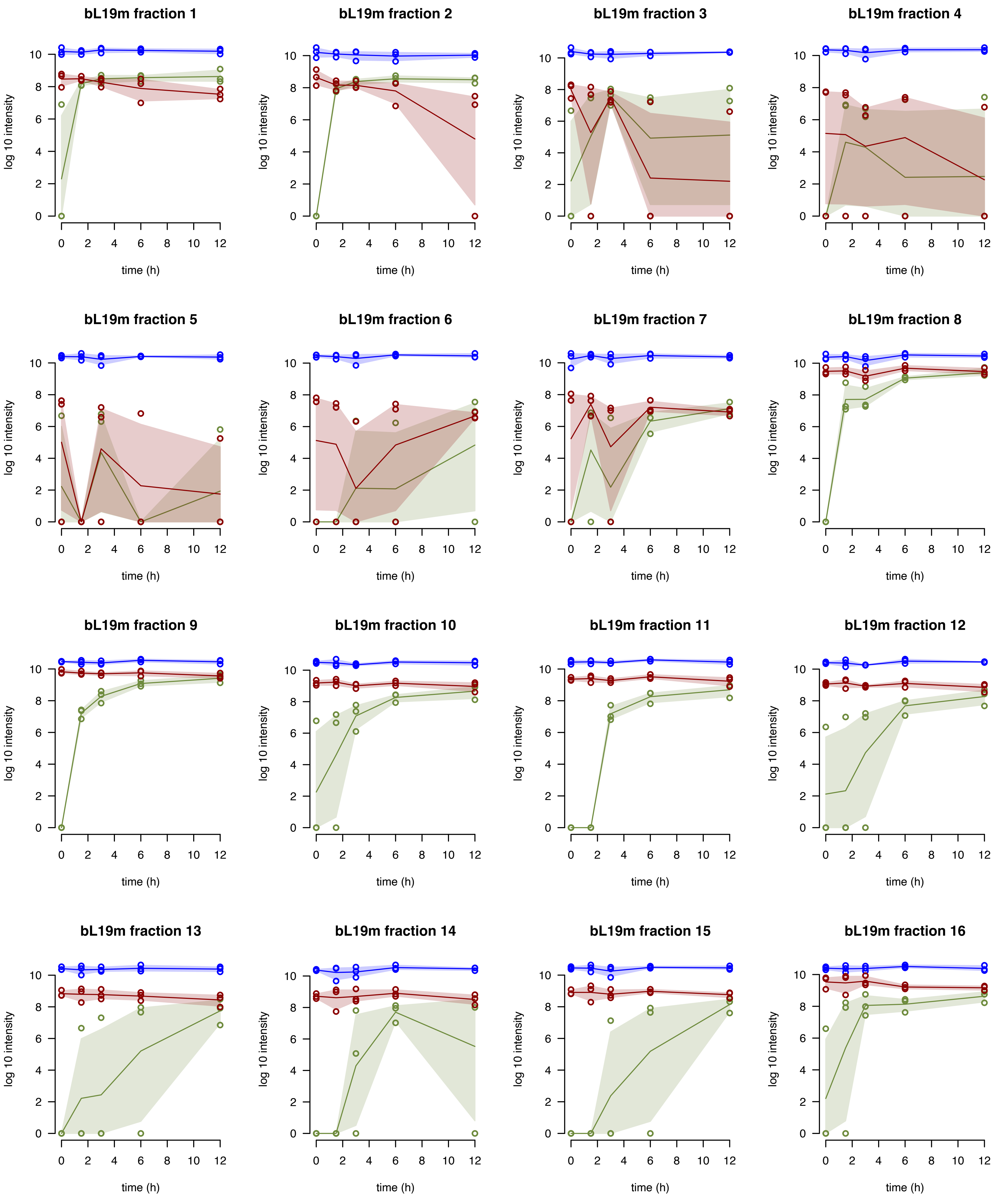
bL19m fraction 1
bL19m fraction 2
bL19m fraction 3
bL19m fraction 4
bL19m fraction 5
bL19m fraction 6
bL19m fraction 7
bL19m fraction 8
bL19m fraction 9
bL19m fraction 10
bL19m fraction 11
bL19m fraction 12
bL19m fraction 13
bL19m fraction 14
bL19m fraction 15
bL19m fraction 16
log 10 intensity
time (h)
0
2
4
6
8
10
12

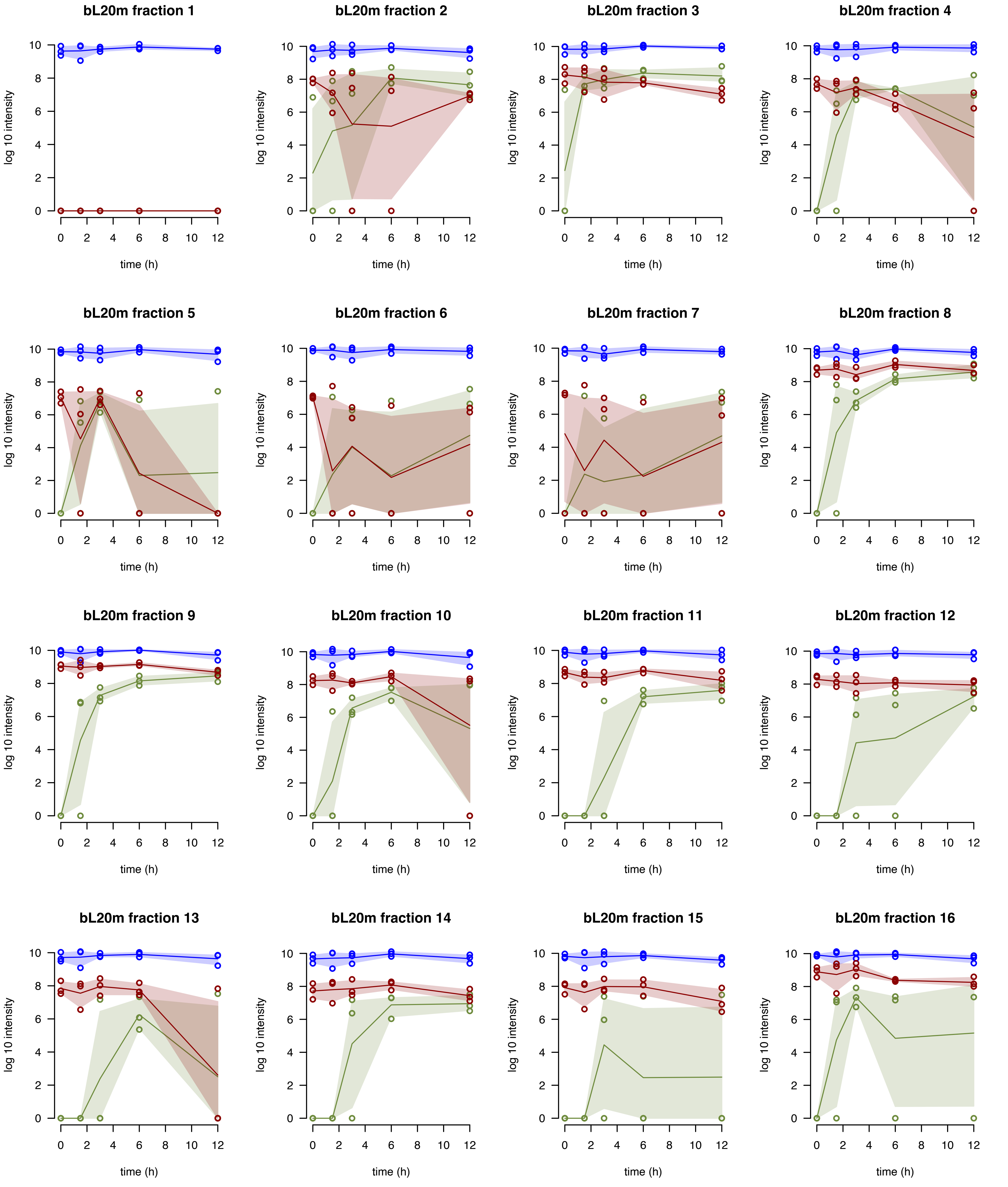
bL20m fraction 1
bL20m fraction 2
bL20m fraction 3
bL20m fraction 4
bL20m fraction 5
bL20m fraction 6
bL20m fraction 7
bL20m fraction 8
bL20m fraction 9
bL20m fraction 10
bL20m fraction 11
bL20m fraction 12
bL20m fraction 13
bL20m fraction 14
bL20m fraction 15
bL20m fraction 16
log 10 intensity
time (h)
0
2
4
6
8
10
12

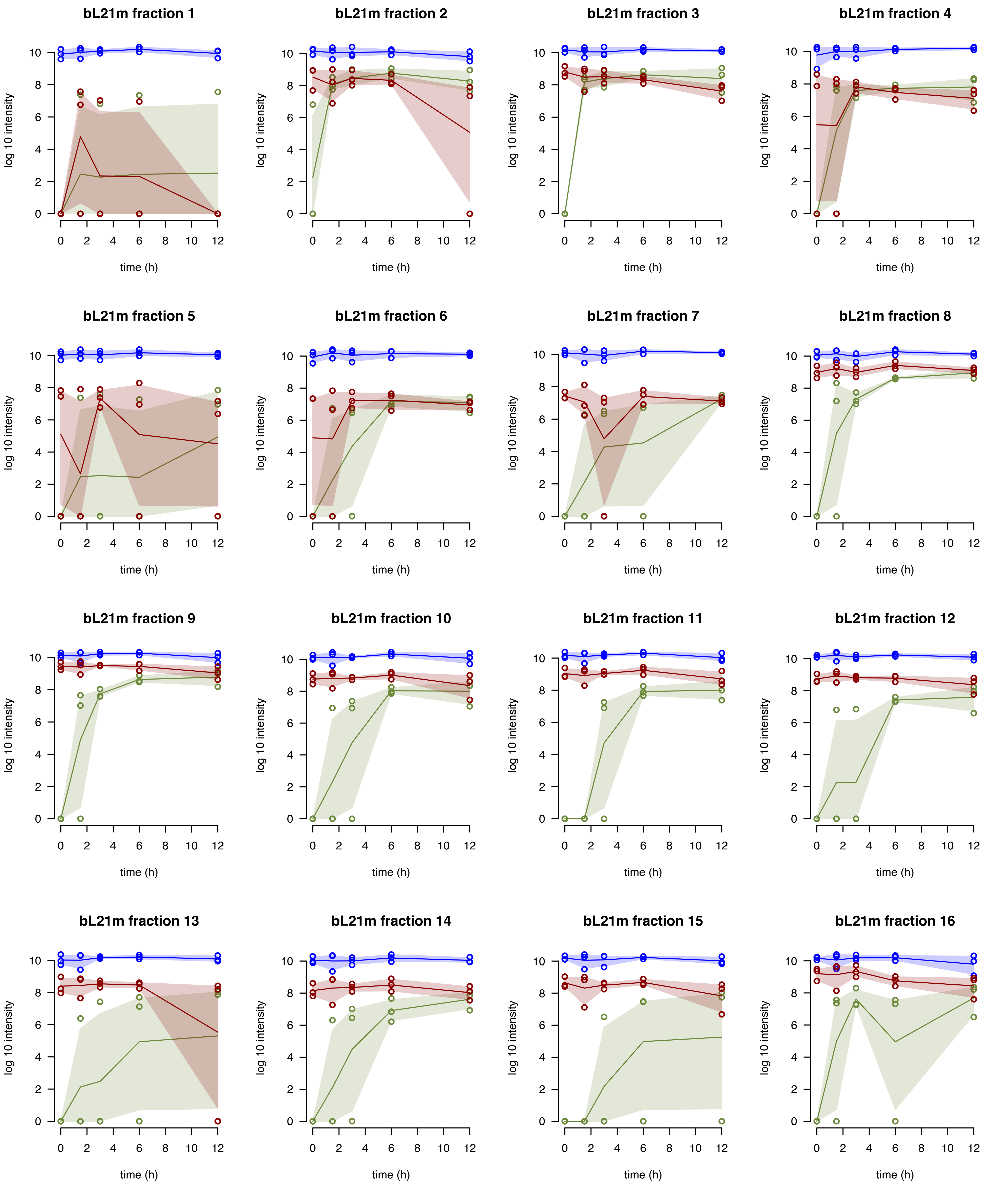
bL21m fraction 1
bL21m fraction 2
bL21m fraction 3
bL21m fraction 4
bL21m fraction 5
bL21m fraction 6
bL21m fraction 7
bL21m fraction 8
bL21m fraction 9
bL21m fraction 10
bL21m fraction 11
bL21m fraction 12
bL21m fraction 13
bL21m fraction 14
bL21m fraction 15
bL21m fraction 16
log 10 intensity
time (h)

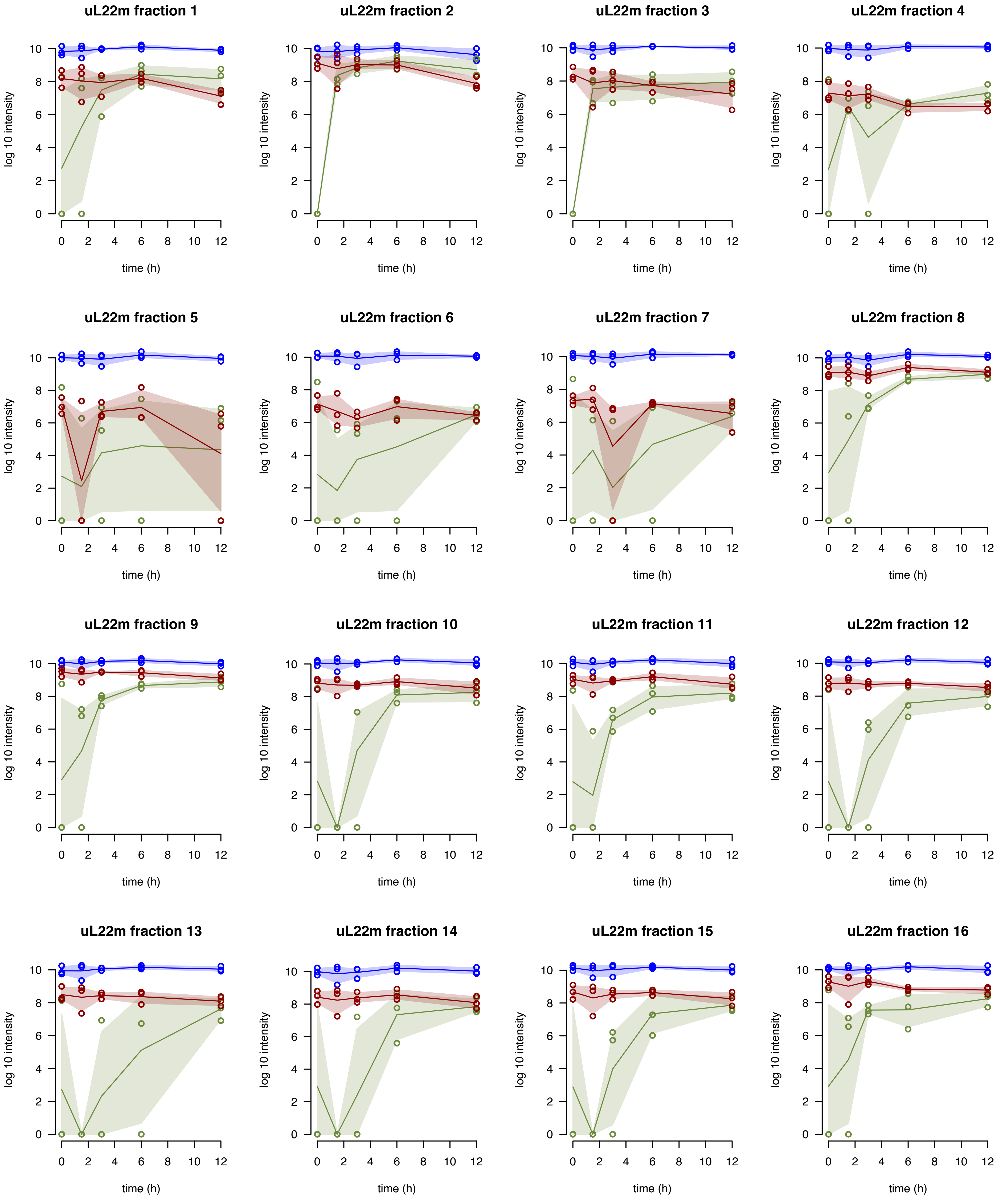
uL22m fraction 1
uL22m fraction 2
uL22m fraction 3
uL22m fraction 4
uL22m fraction 5
uL22m fraction 6
uL22m fraction 7
uL22m fraction 8
uL22m fraction 9
uL22m fraction 10
uL22m fraction 11
uL22m fraction 12
uL22m fraction 13
uL22m fraction 14
uL22m fraction 15
uL22m fraction 16
log 10 intensity
time (h)
0
2
4
6
8
10
12

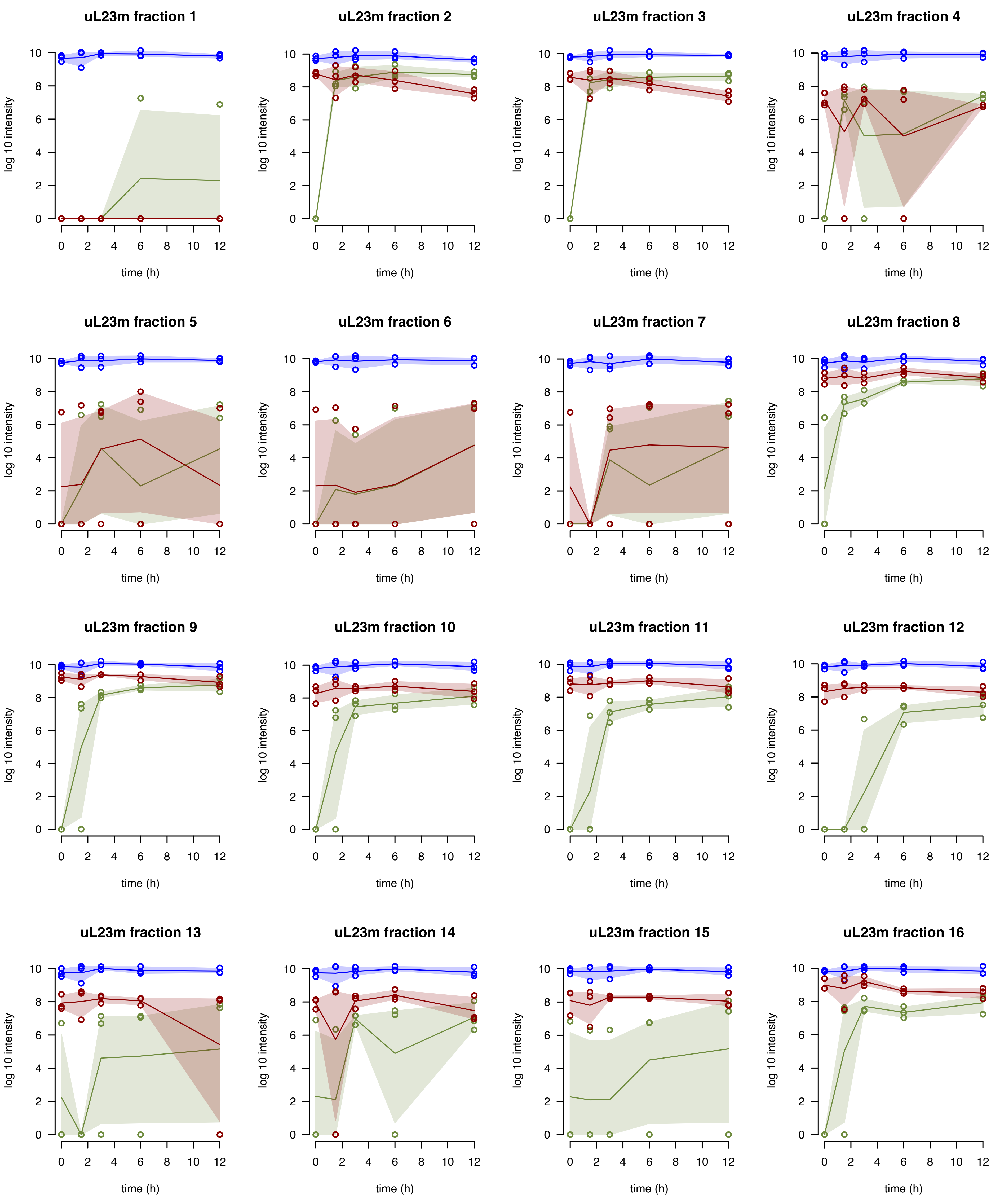
uL23m fraction 1
uL23m fraction 2
uL23m fraction 3
uL23m fraction 4
uL23m fraction 5
uL23m fraction 6
uL23m fraction 7
uL23m fraction 8
uL23m fraction 9
uL23m fraction 10
uL23m fraction 11
uL23m fraction 12
uL23m fraction 13
uL23m fraction 14
uL23m fraction 15
uL23m fraction 16
log 10 intensity
time (h)
0 2 4 6 8 10
0 2 4 6 8 10 12

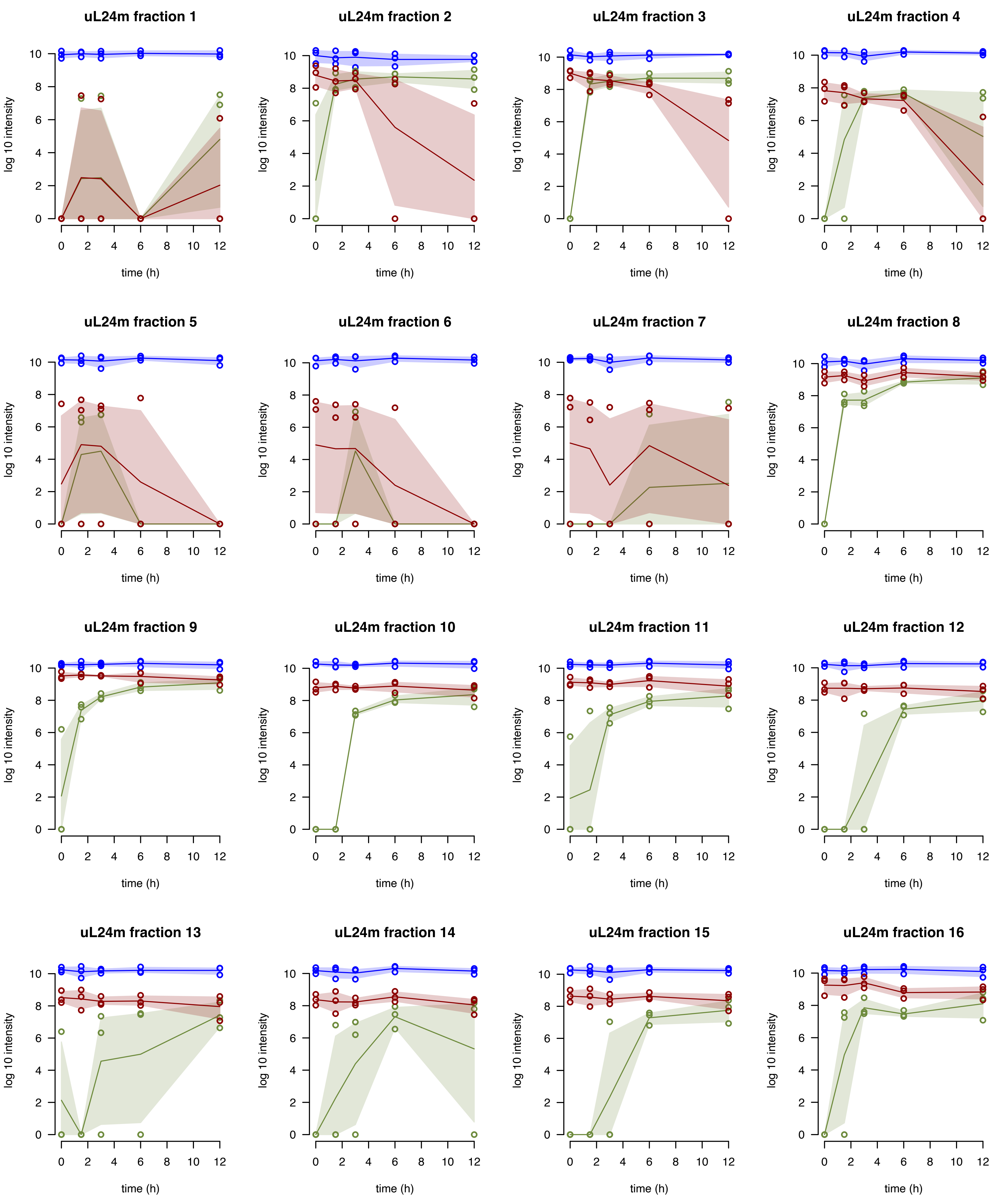

uL24m fraction 1
uL24m fraction 2
uL24m fraction 3
uL24m fraction 4
uL24m fraction 5
uL24m fraction 6
uL24m fraction 7
uL24m fraction 8
uL24m fraction 9
uL24m fraction 10
uL24m fraction 11
uL24m fraction 12
uL24m fraction 13
uL24m fraction 14
uL24m fraction 15
uL24m fraction 16
log 10 intensity
time (h)

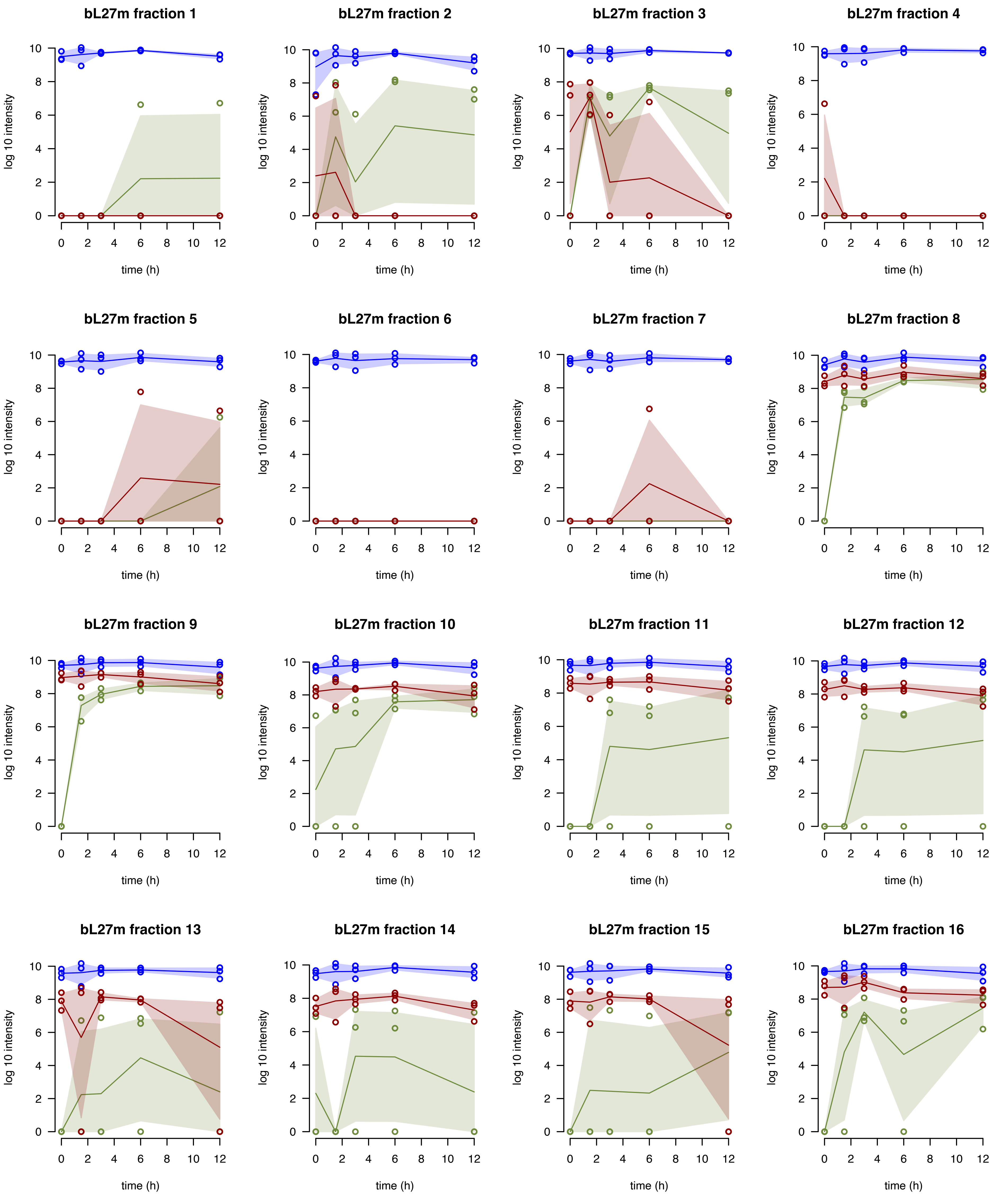

bL27m fraction 1
bL27m fraction 2
bL27m fraction 3
bL27m fraction 4
bL27m fraction 5
bL27m fraction 6
bL27m fraction 7
bL27m fraction 8
bL27m fraction 9
bL27m fraction 10
bL27m fraction 11
bL27m fraction 12
bL27m fraction 13
bL27m fraction 14
bL27m fraction 15
bL27m fraction 16
log 10 intensity
time (h)

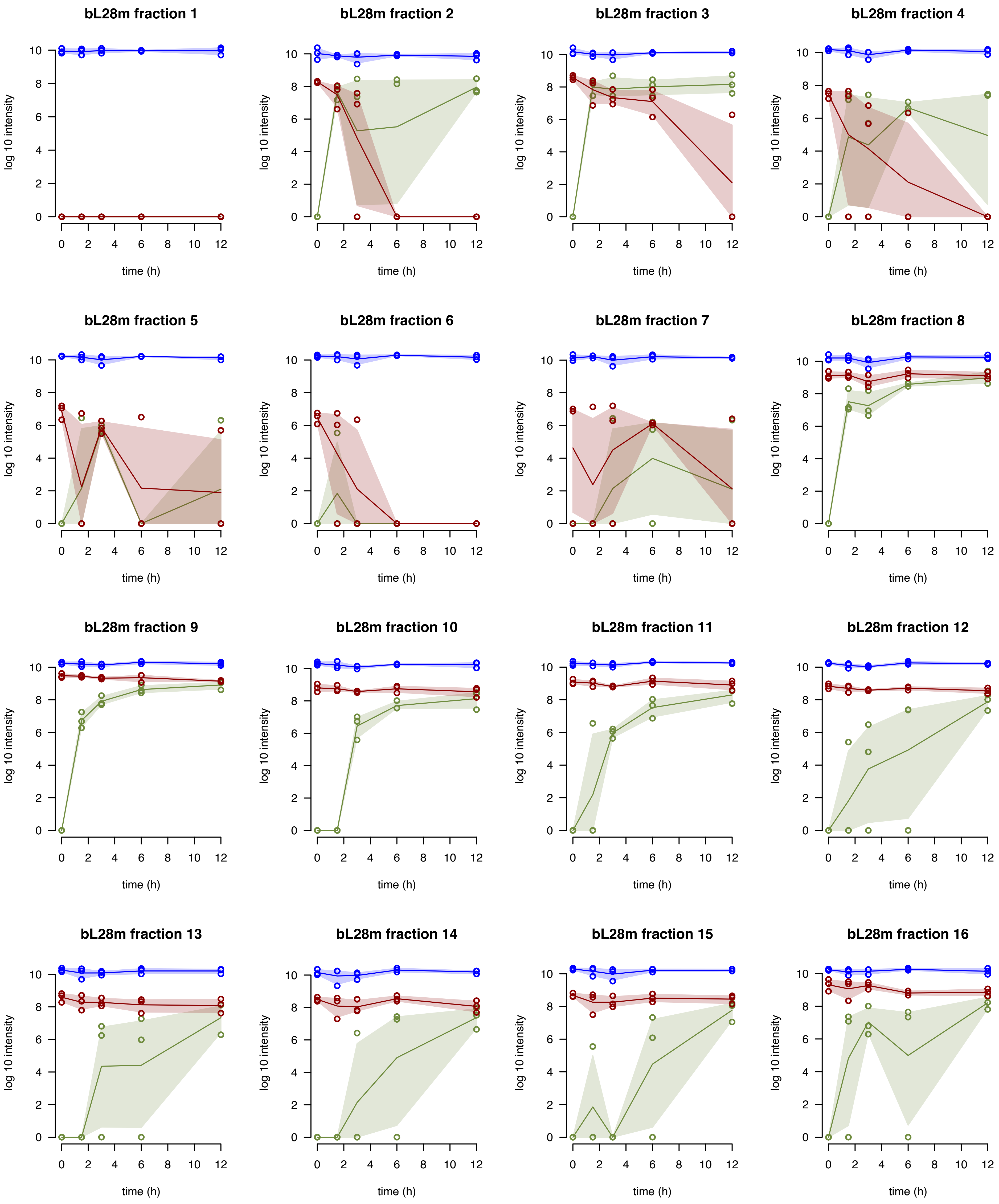
bL28m fraction 1
bL28m fraction 2
bL28m fraction 3
bL28m fraction 4
bL28m fraction 5
bL28m fraction 6
bL28m fraction 7
bL28m fraction 8
bL28m fraction 9
bL28m fraction 10
bL28m fraction 11
bL28m fraction 12
bL28m fraction 13
bL28m fraction 14
bL28m fraction 15
bL28m fraction 16
log 10 intensity
time (h)

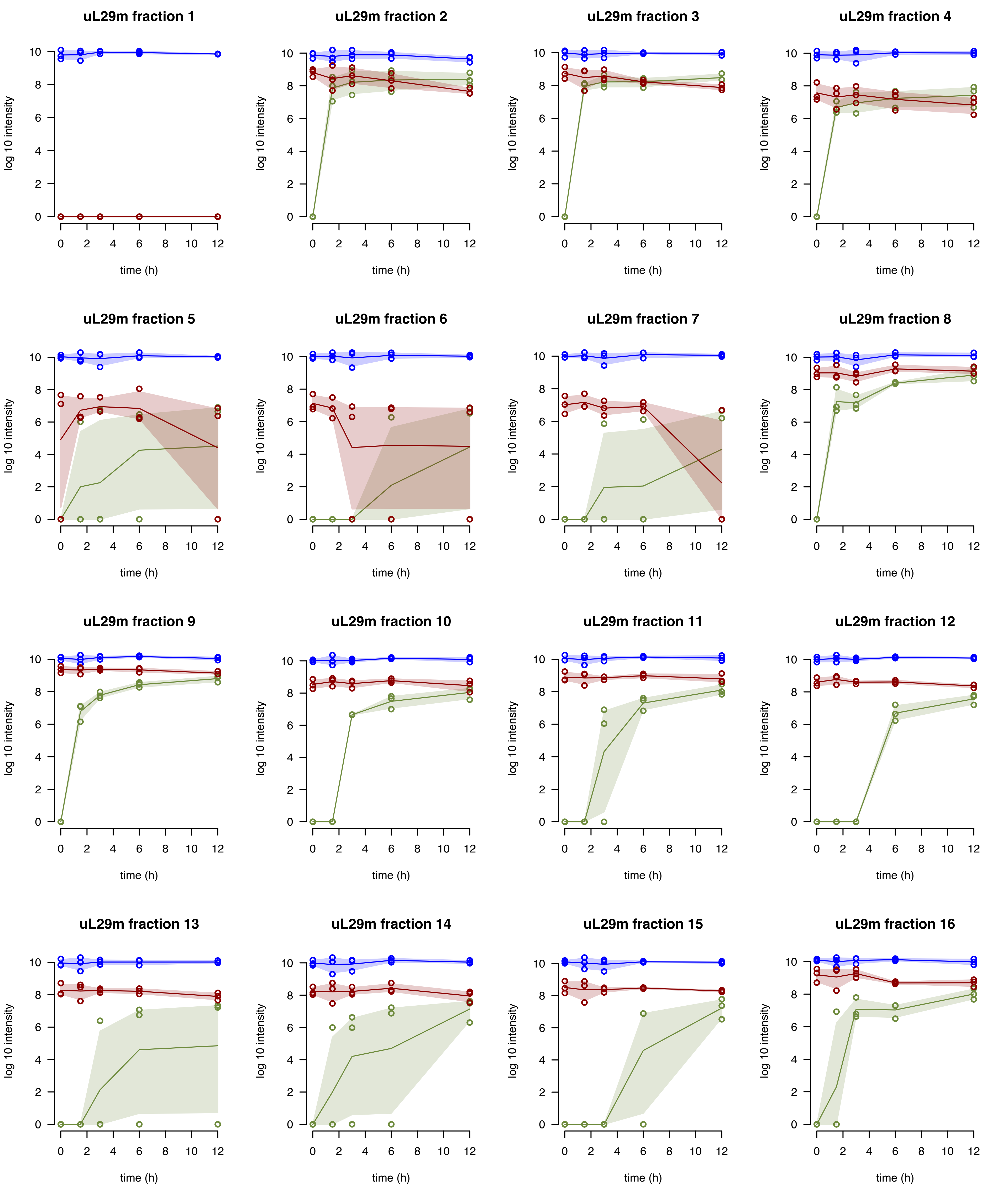
uL29m fraction 1
uL29m fraction 2
uL29m fraction 3
uL29m fraction 4
uL29m fraction 5
uL29m fraction 6
uL29m fraction 7
uL29m fraction 8
uL29m fraction 9
uL29m fraction 10
uL29m fraction 11
uL29m fraction 12
uL29m fraction 13
uL29m fraction 14
uL29m fraction 15
uL29m fraction 16
log 10 intensity
time (h)

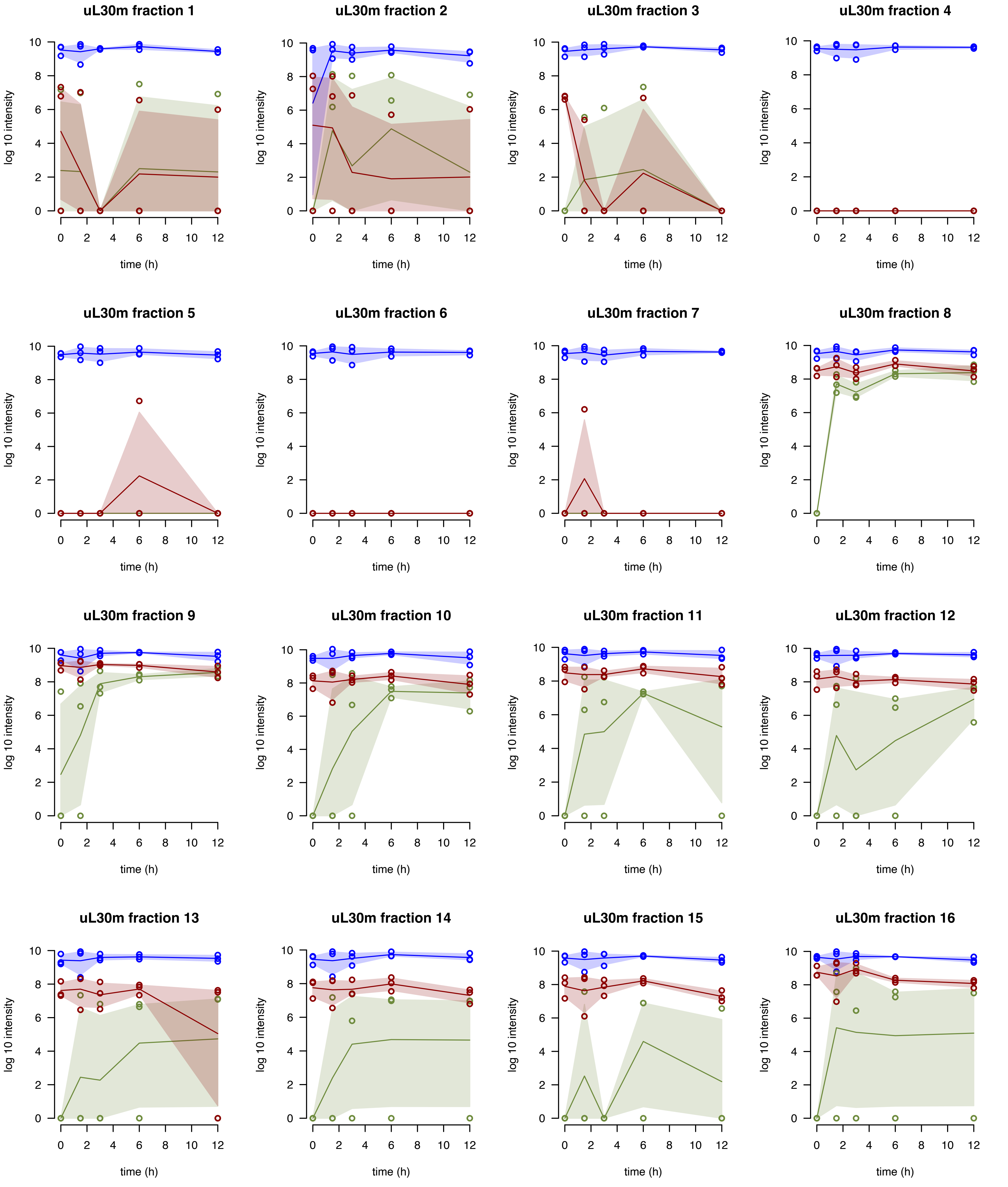
uL30m fraction 1
uL30m fraction 2
uL30m fraction 3
uL30m fraction 4
uL30m fraction 5
uL30m fraction 6
uL30m fraction 7
uL30m fraction 8
uL30m fraction 9
uL30m fraction 10
uL30m fraction 11
uL30m fraction 12
uL30m fraction 13
uL30m fraction 14
uL30m fraction 15
uL30m fraction 16
log 10 intensity
time (h)
0
2
4
6
8
10
12

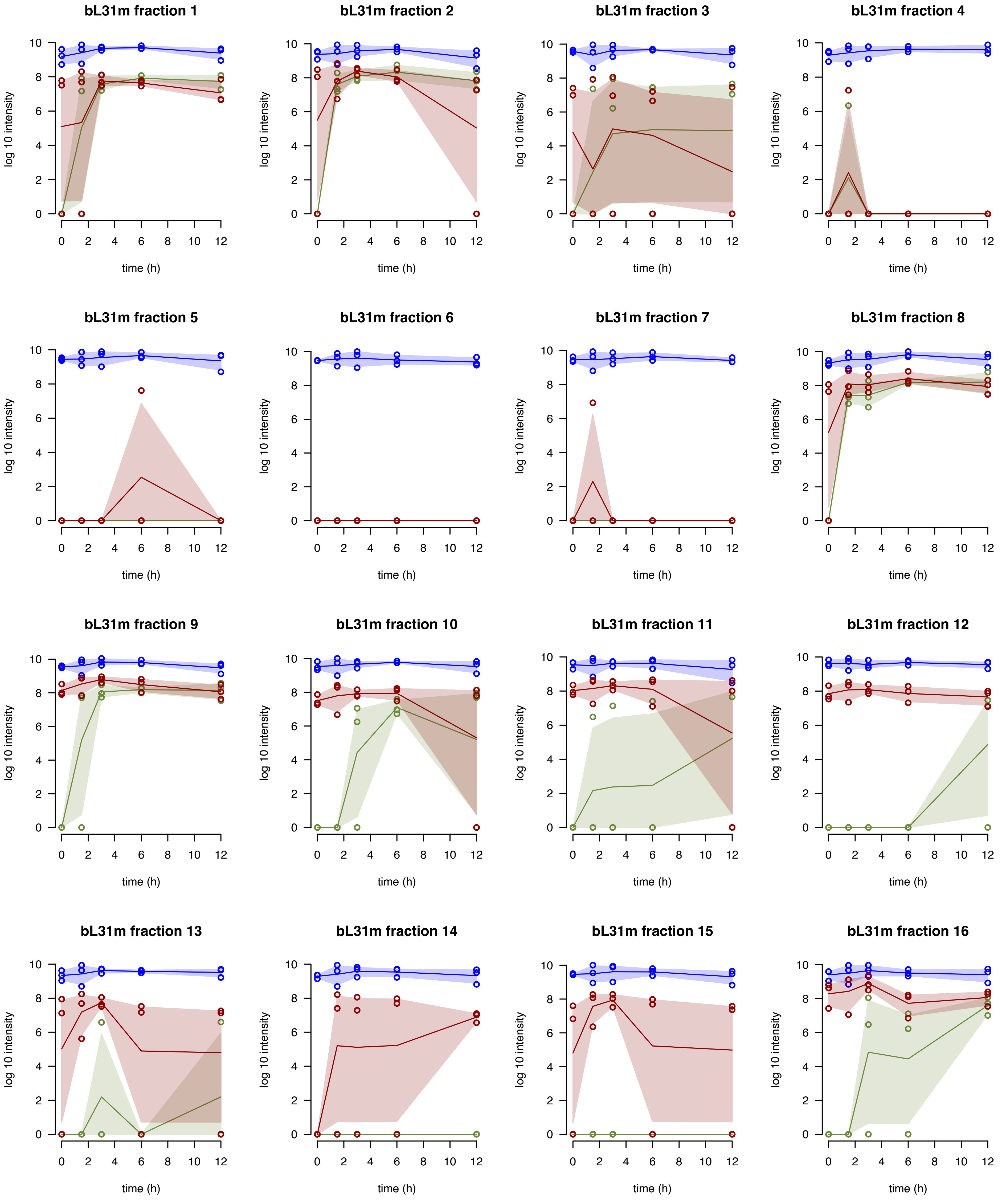
bL31m fraction 1
bL31m fraction 2
bL31m fraction 3
bL31m fraction 4
bL31m fraction 5
bL31m fraction 6
bL31m fraction 7
bL31m fraction 8
bL31m fraction 9
bL31m fraction 10
bL31m fraction 11
bL31m fraction 12
bL31m fraction 13
bL31m fraction 14
bL31m fraction 15
bL31m fraction 16
log 10 intensity
time (h)

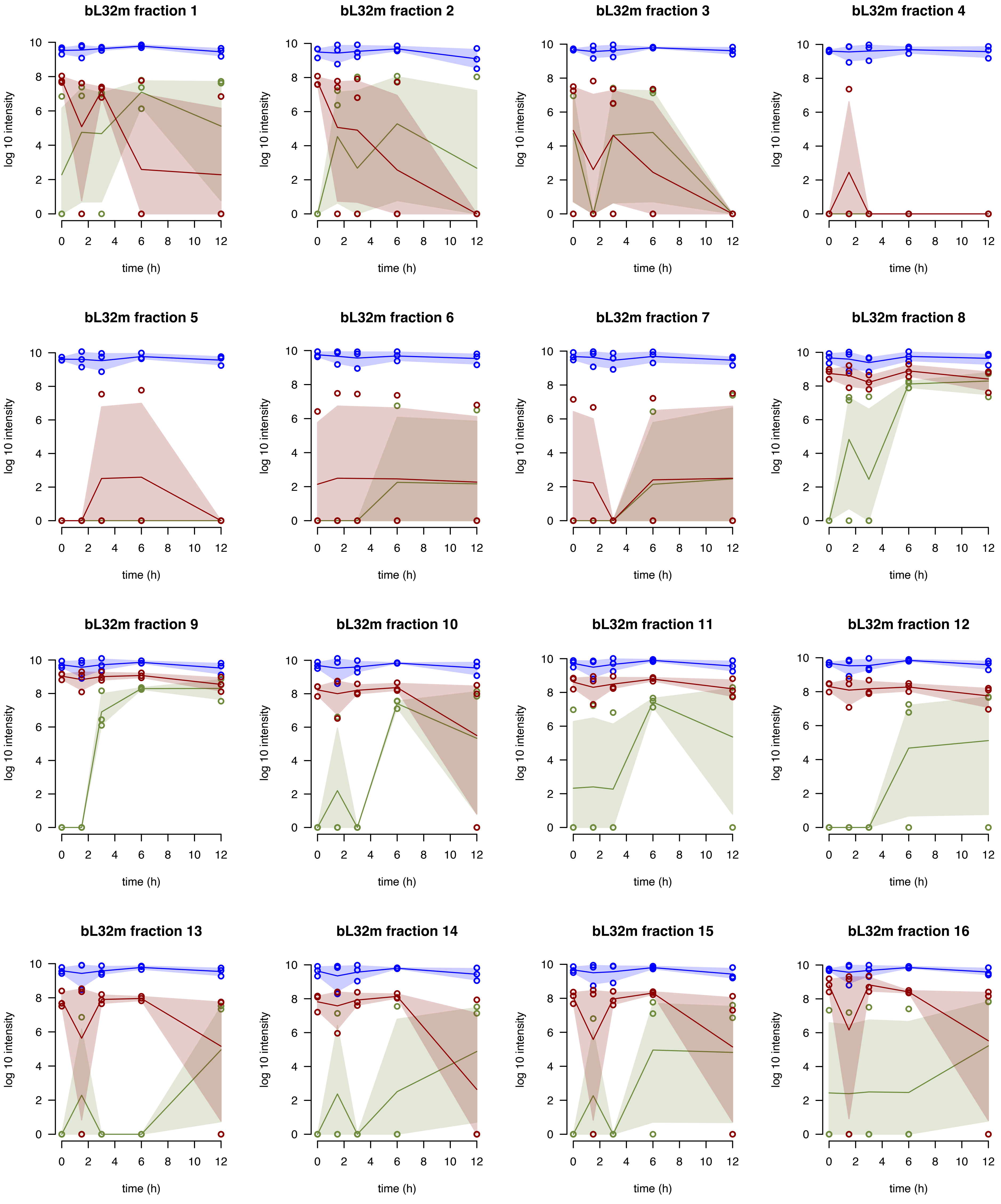
bL32m fraction 1
bL32m fraction 2
bL32m fraction 3
bL32m fraction 4
bL32m fraction 5
bL32m fraction 6
bL32m fraction 7
bL32m fraction 8
bL32m fraction 9
bL32m fraction 10
bL32m fraction 11
bL32m fraction 12
bL32m fraction 13
bL32m fraction 14
bL32m fraction 15
bL32m fraction 16
log 10 intensity
0 2 4 6 8 10
time (h)
0 2 4 6 8 10 12

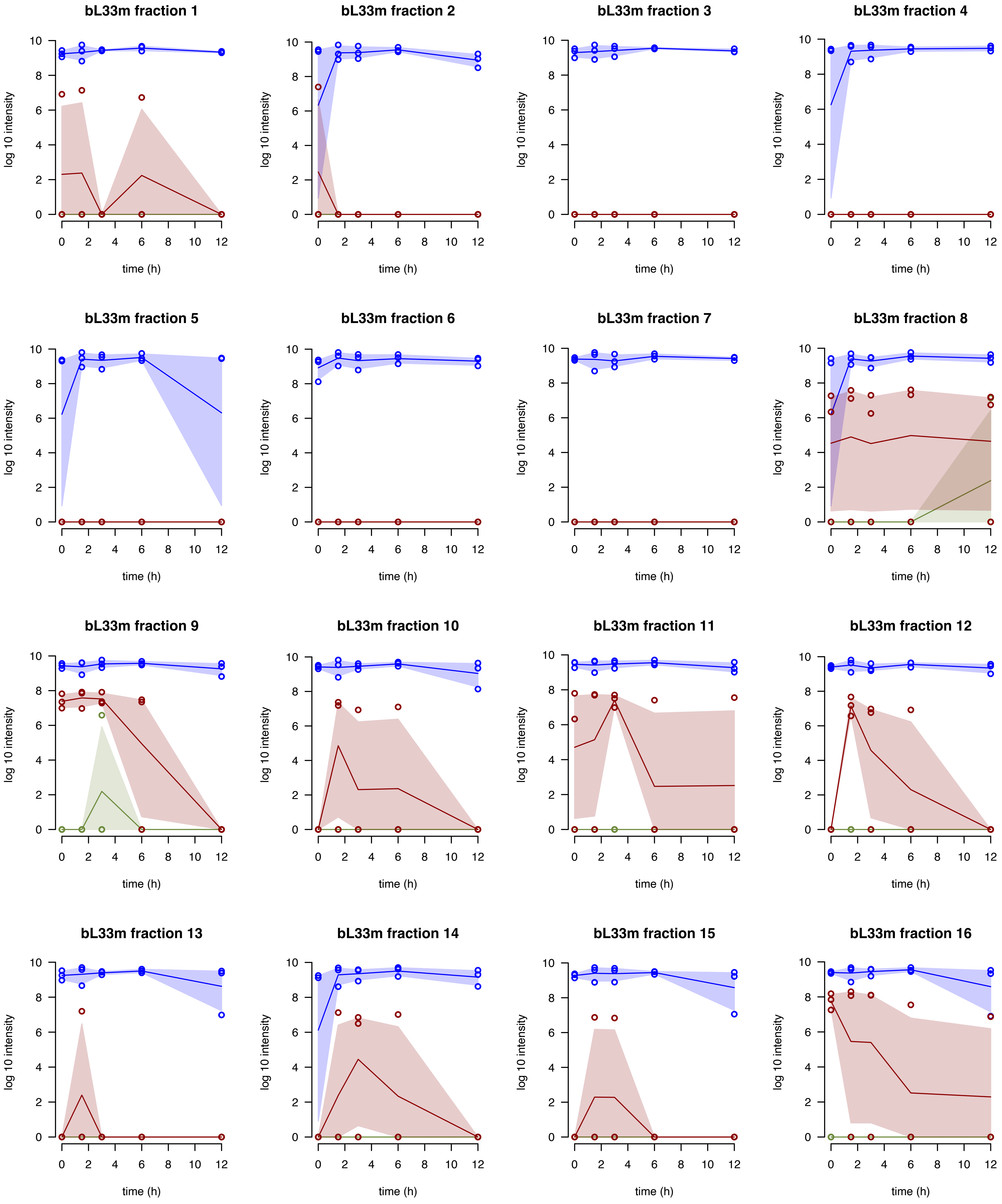
bL33m fraction 1
bL33m fraction 2
bL33m fraction 3
bL33m fraction 4
bL33m fraction 5
bL33m fraction 6
bL33m fraction 7
bL33m fraction 8
bL33m fraction 9
bL33m fraction 10
bL33m fraction 11
bL33m fraction 12
bL33m fraction 13
bL33m fraction 14
bL33m fraction 15
bL33m fraction 16
log 10 intensity
time (h)

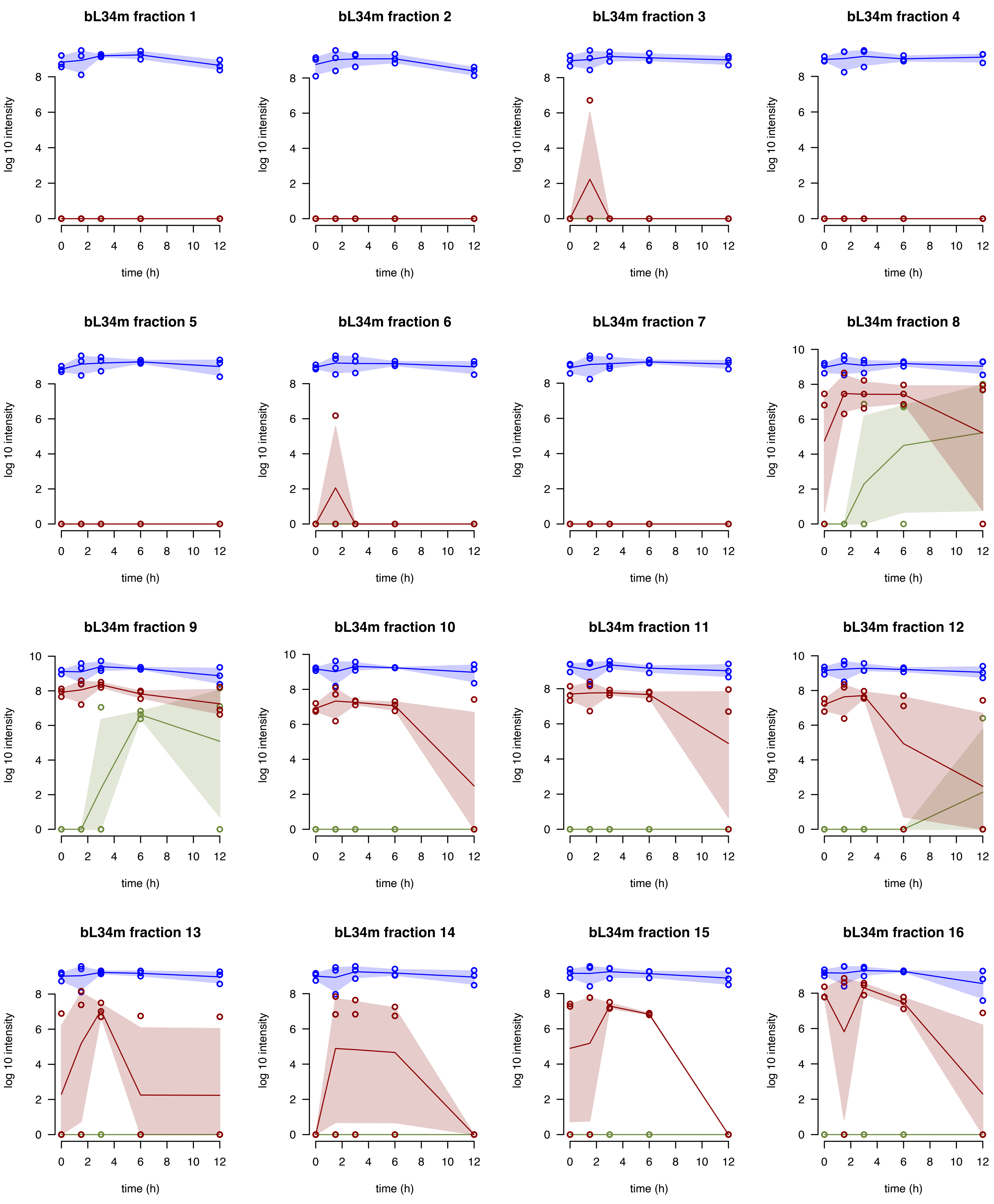
bL34m fraction 1
bL34m fraction 2
bL34m fraction 3
bL34m fraction 4
bL34m fraction 5
bL34m fraction 6
bL34m fraction 7
bL34m fraction 8
bL34m fraction 9
bL34m fraction 10
bL34m fraction 11
bL34m fraction 12
bL34m fraction 13
bL34m fraction 14
bL34m fraction 15
bL34m fraction 16
log 10 intensity
time (h)

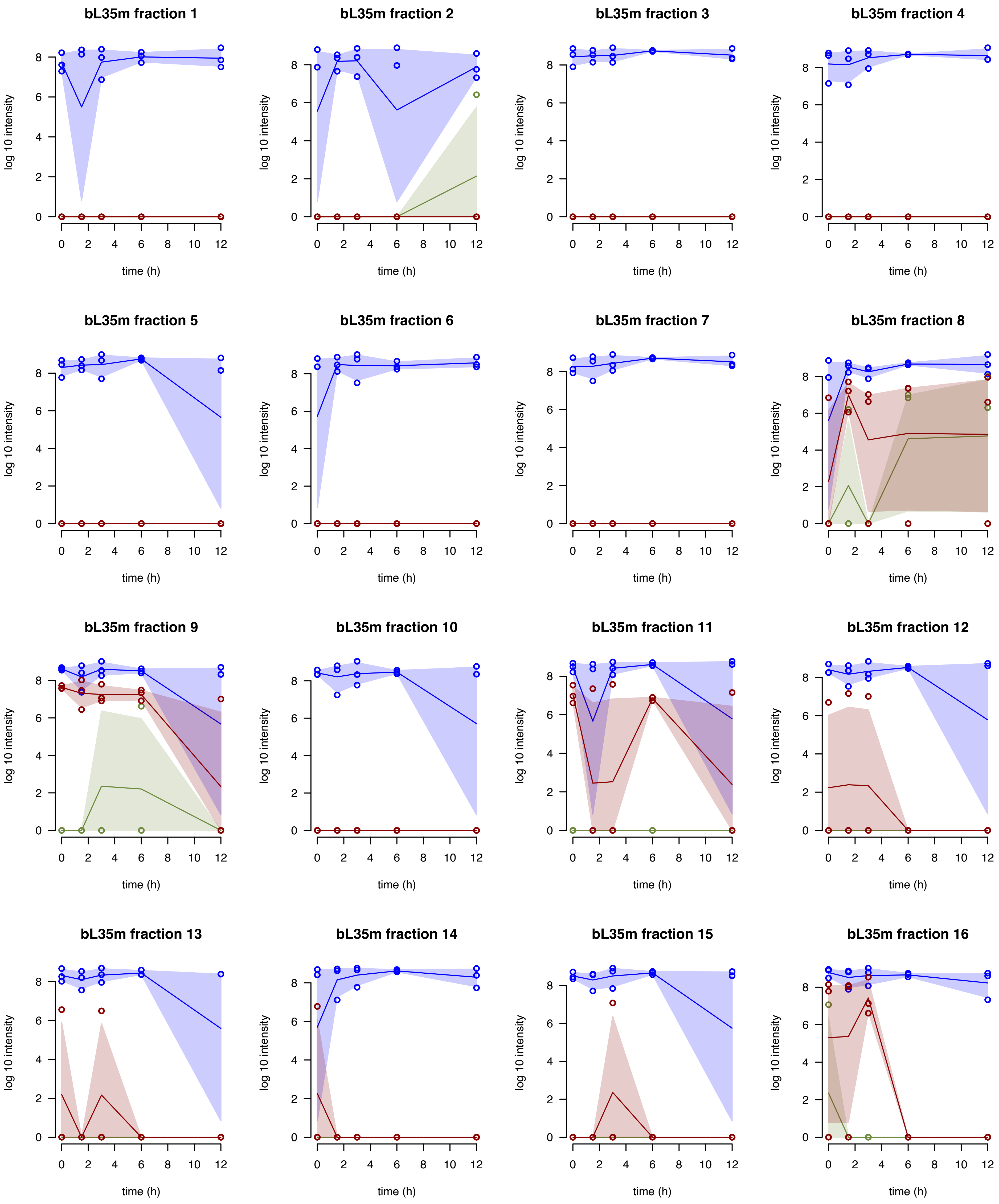
bL35m fraction 1
bL35m fraction 2
bL35m fraction 3
bL35m fraction 4
bL35m fraction 5
bL35m fraction 6
bL35m fraction 7
bL35m fraction 8
bL35m fraction 9
bL35m fraction 10
bL35m fraction 11
bL35m fraction 12
bL35m fraction 13
bL35m fraction 14
bL35m fraction 15
bL35m fraction 16
log 10 intensity
0 2 4 6 8
time (h)
0 2 4 6 8 10 12

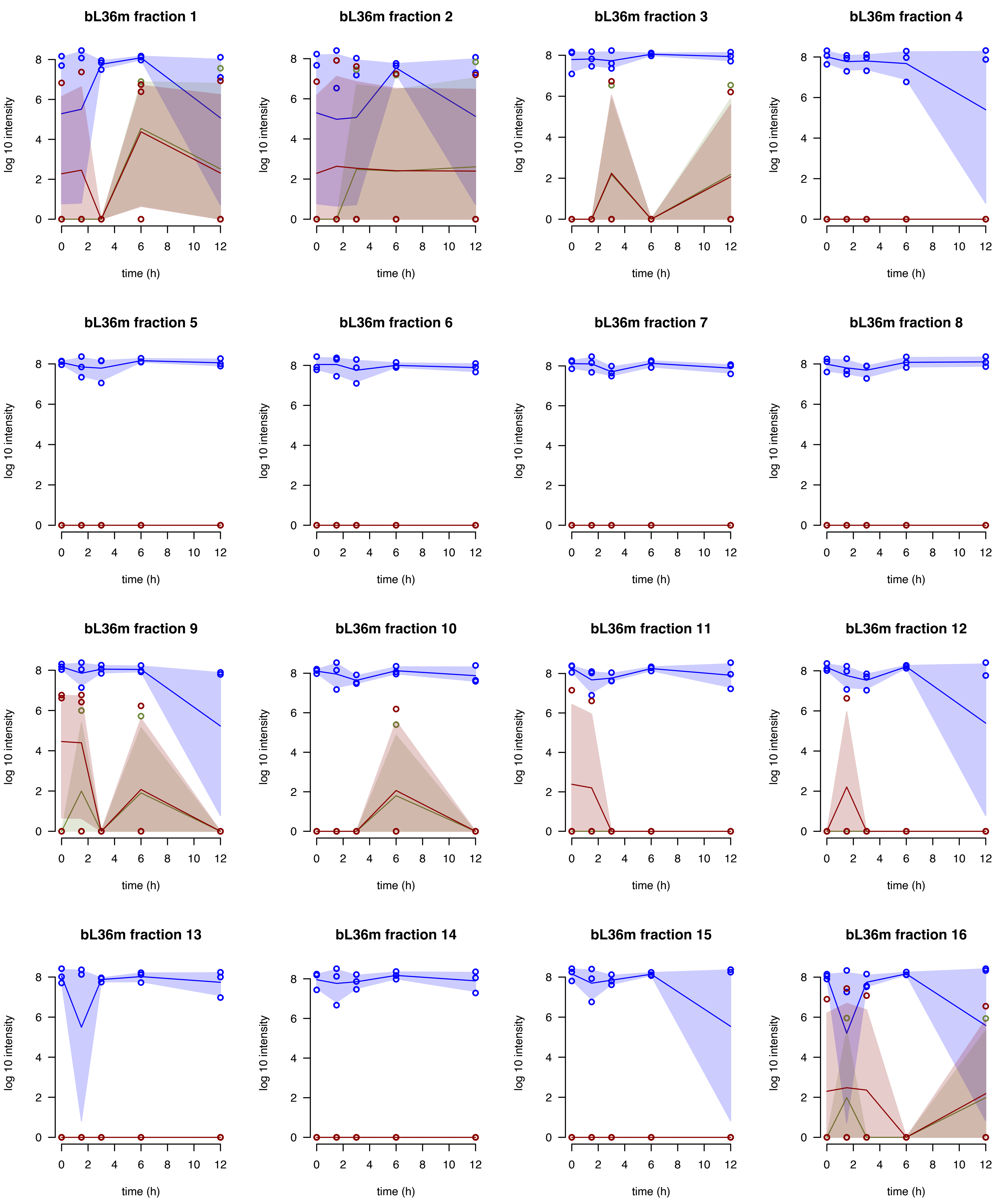
bL36m fraction 1
bL36m fraction 2
bL36m fraction 3
bL36m fraction 4
bL36m fraction 5
bL36m fraction 6
bL36m fraction 7
bL36m fraction 8
bL36m fraction 9
bL36m fraction 10
bL36m fraction 11
bL36m fraction 12
bL36m fraction 13
bL36m fraction 14
bL36m fraction 15
bL36m fraction 16
log 10 intensity
time (h)

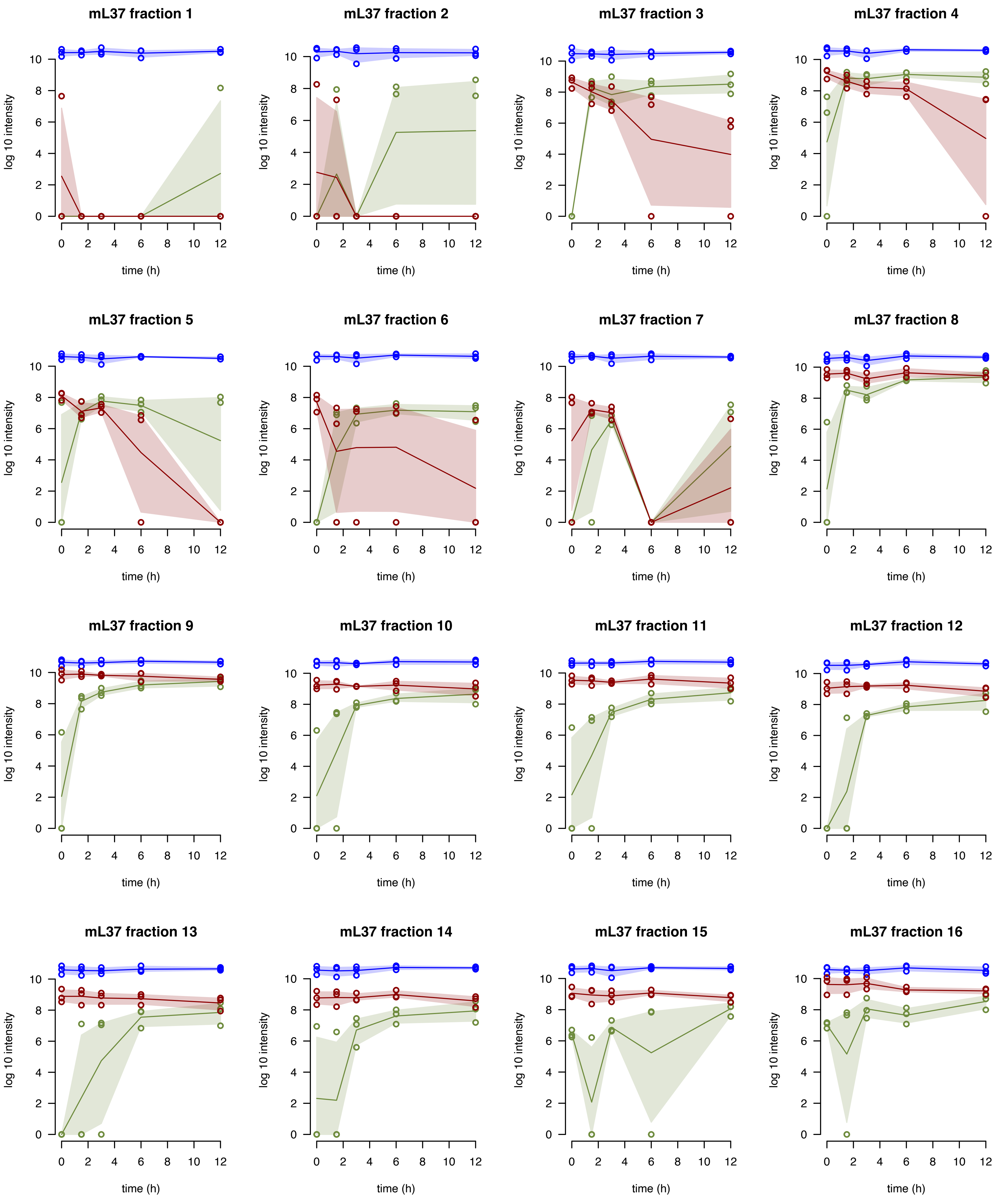
mL37 fraction 1
mL37 fraction 2
mL37 fraction 3
mL37 fraction 4
mL37 fraction 5
mL37 fraction 6
mL37 fraction 7
mL37 fraction 8
mL37 fraction 9
mL37 fraction 10
mL37 fraction 11
mL37 fraction 12
mL37 fraction 13
mL37 fraction 14
mL37 fraction 15
mL37 fraction 16
log 10 intensity
time (h)

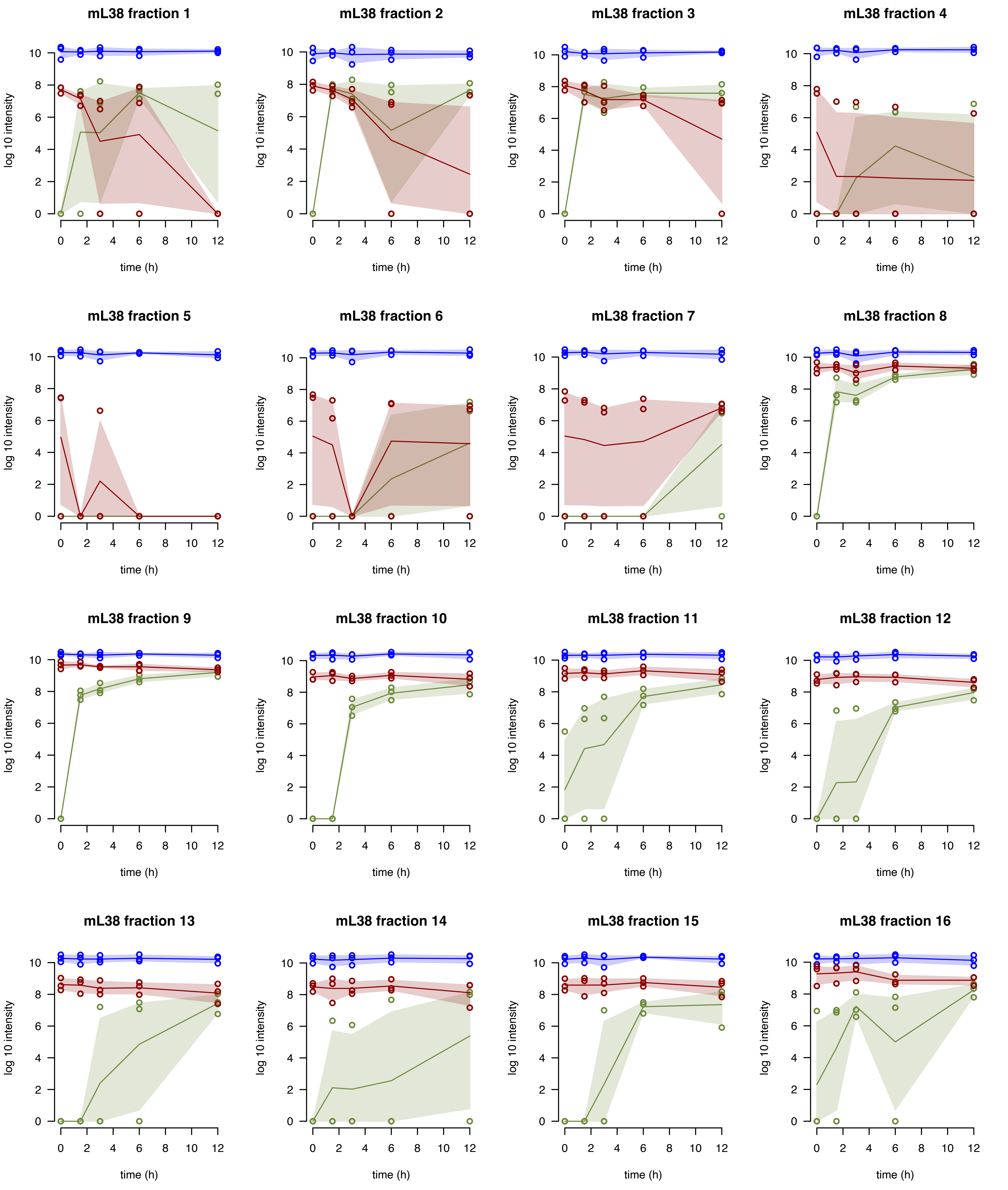
mL38 fraction 1
mL38 fraction 2
mL38 fraction 3
mL38 fraction 4
mL38 fraction 5
mL38 fraction 6
mL38 fraction 7
mL38 fraction 8
mL38 fraction 9
mL38 fraction 10
mL38 fraction 11
mL38 fraction 12
mL38 fraction 13
mL38 fraction 14
mL38 fraction 15
mL38 fraction 16
log 10 intensity
time (h)
0 2 4 6 8 10 12
0 2 4 6 8 10

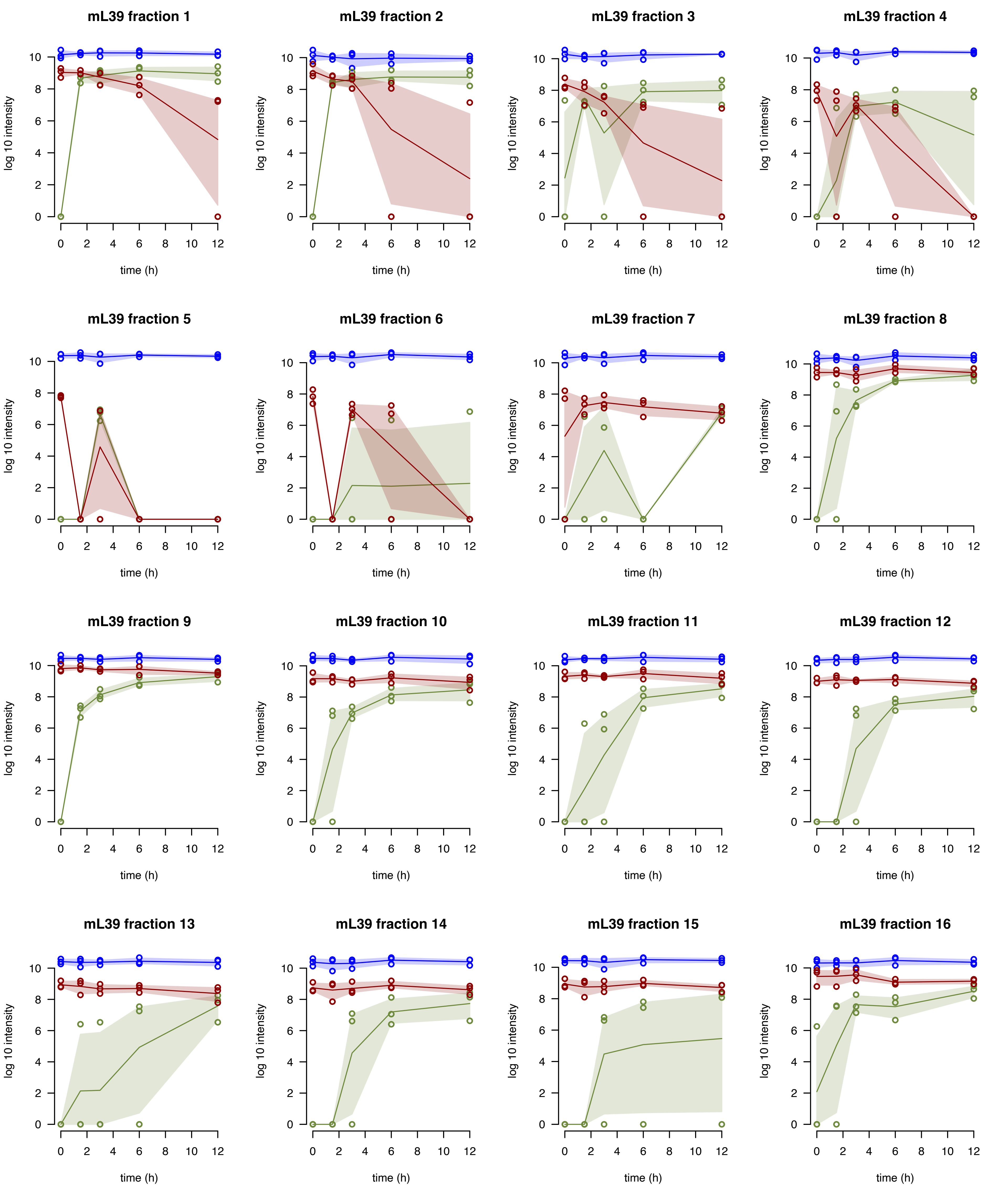
mL39 fraction 1
mL39 fraction 2
mL39 fraction 3
mL39 fraction 4
mL39 fraction 5
mL39 fraction 6
mL39 fraction 7
mL39 fraction 8
mL39 fraction 9
mL39 fraction 10
mL39 fraction 11
mL39 fraction 12
mL39 fraction 13
mL39 fraction 14
mL39 fraction 15
mL39 fraction 16
log 10 intensity
time (h)
0 2 4 6 8 10 12
0 2 4 6 8 10

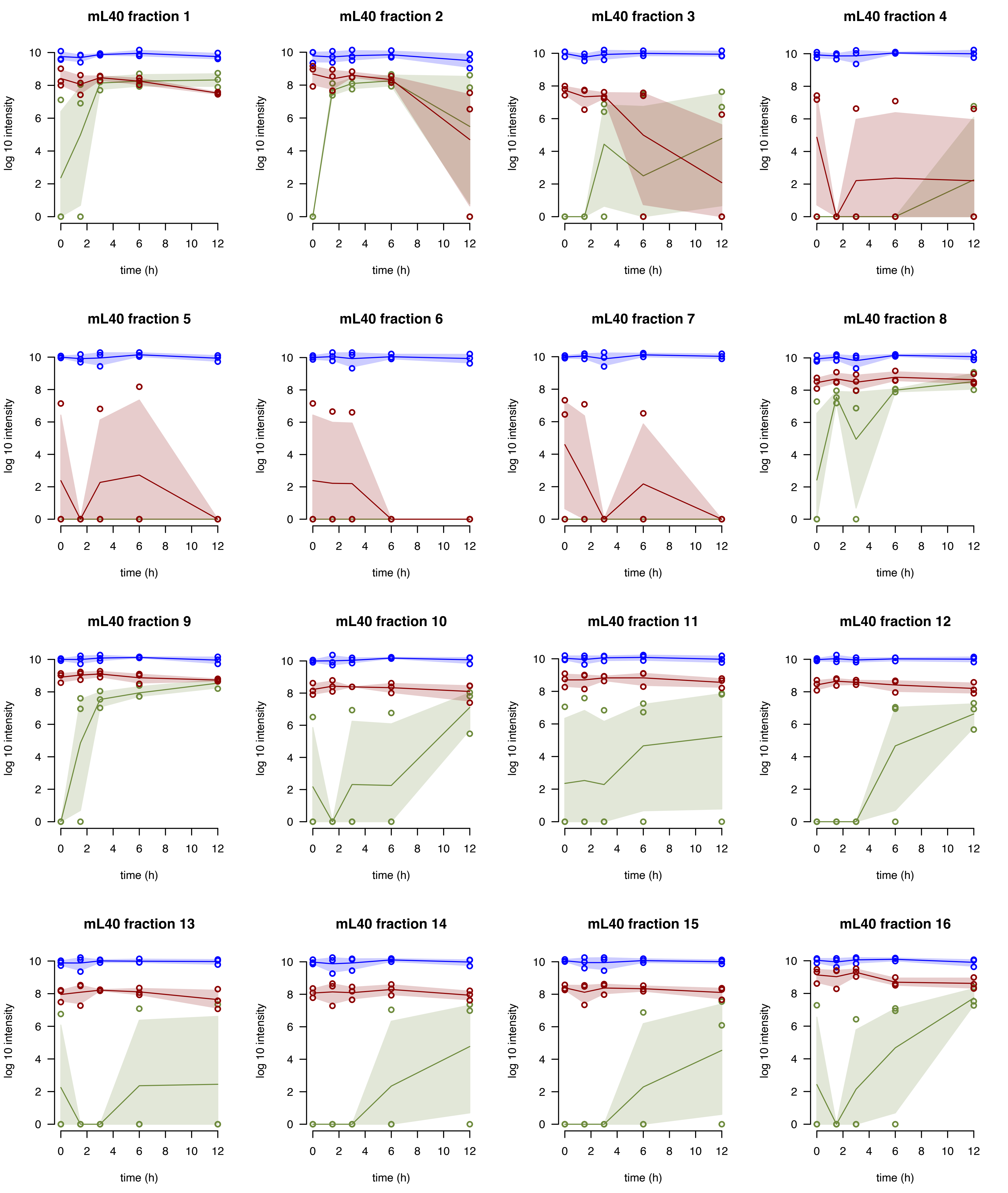

mL40 fraction 1
mL40 fraction 2
mL40 fraction 3
mL40 fraction 4
mL40 fraction 5
mL40 fraction 6
mL40 fraction 7
mL40 fraction 8
mL40 fraction 9
mL40 fraction 10
mL40 fraction 11
mL40 fraction 12
mL40 fraction 13
mL40 fraction 14
mL40 fraction 15
mL40 fraction 16
log 10 intensity
time (h)

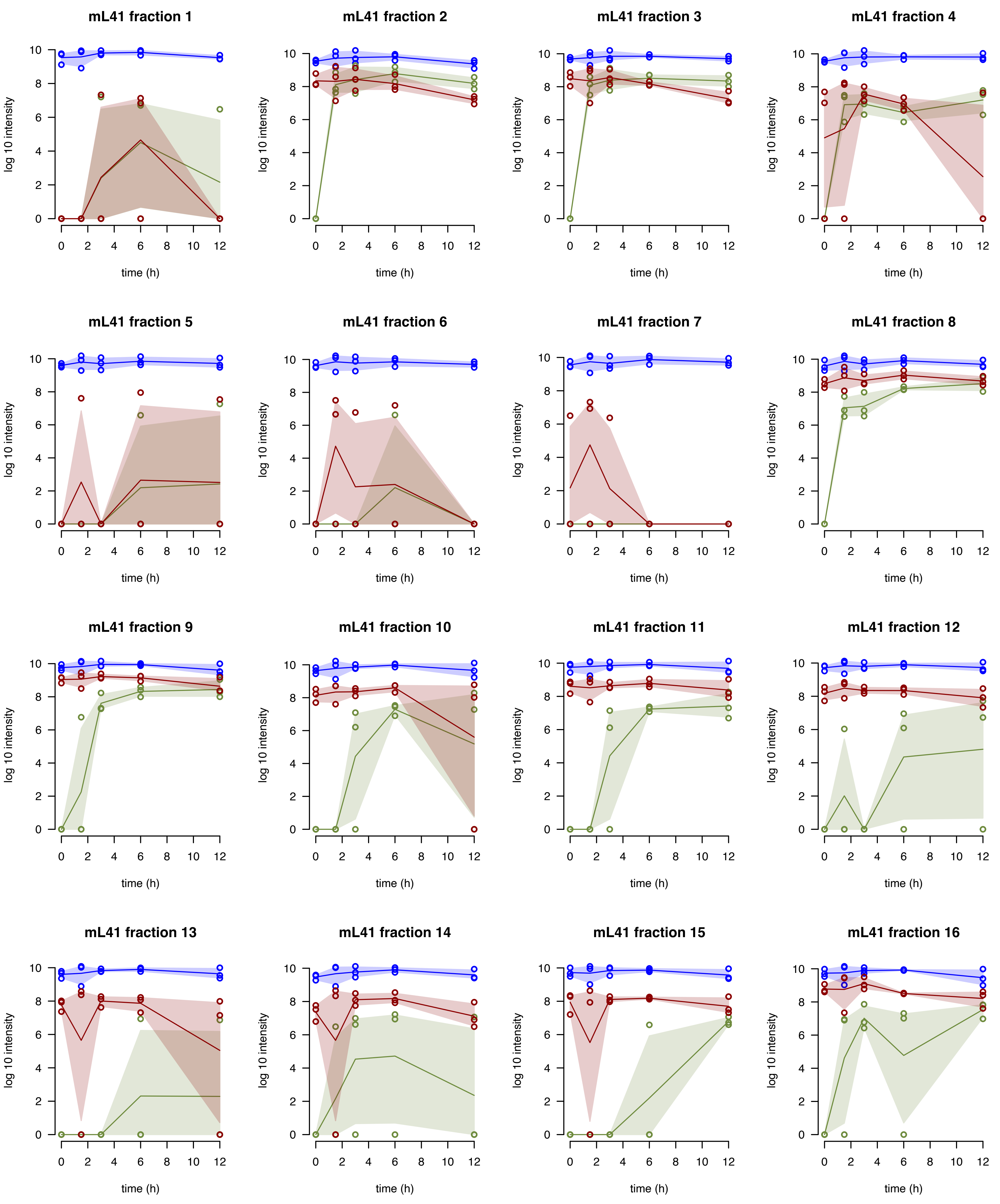
mL41 fraction 1
mL41 fraction 2
mL41 fraction 3
mL41 fraction 4
mL41 fraction 5
mL41 fraction 6
mL41 fraction 7
mL41 fraction 8
mL41 fraction 9
mL41 fraction 10
mL41 fraction 11
mL41 fraction 12
mL41 fraction 13
mL41 fraction 14
mL41 fraction 15
mL41 fraction 16
log 10 intensity
time (h)
0 2 4 6 8 10 12
0 2 4 6 8 10

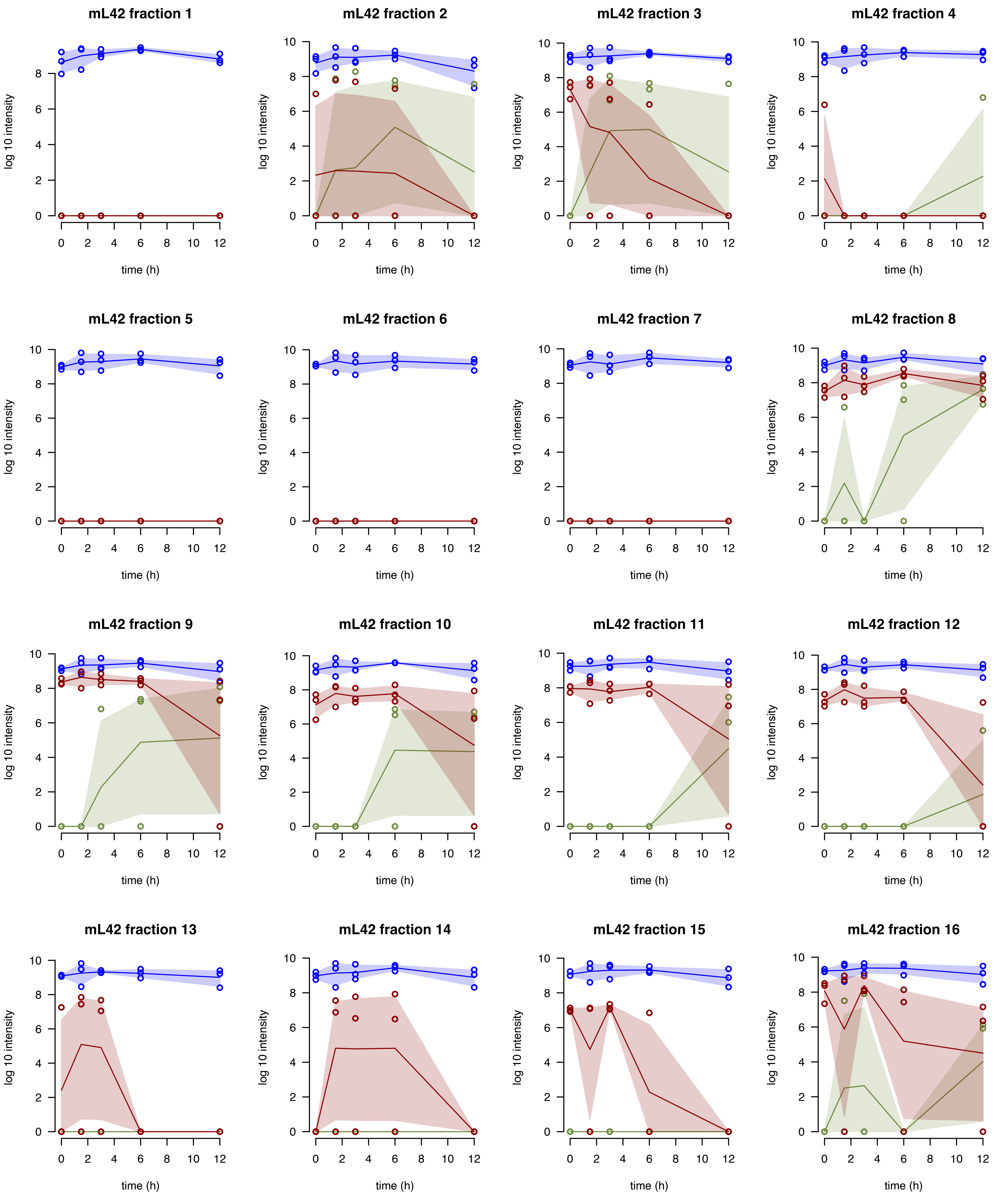
mL42 fraction 1
mL42 fraction 2
mL42 fraction 3
mL42 fraction 4
mL42 fraction 5
mL42 fraction 6
mL42 fraction 7
mL42 fraction 8
mL42 fraction 9
mL42 fraction 10
mL42 fraction 11
mL42 fraction 12
mL42 fraction 13
mL42 fraction 14
mL42 fraction 15
mL42 fraction 16
log 10 intensity
time (h)

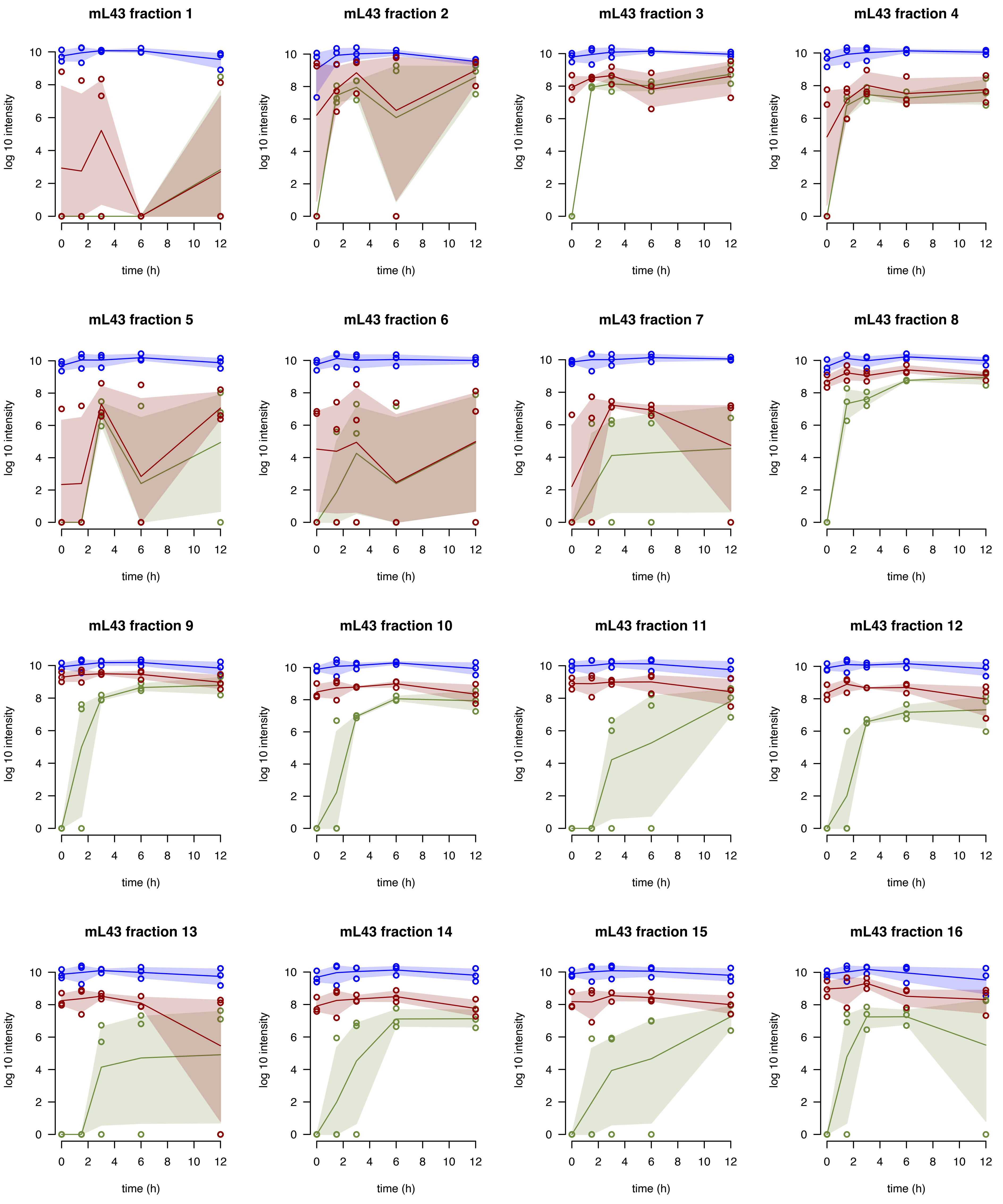
mL43 fraction 1
mL43 fraction 2
mL43 fraction 3
mL43 fraction 4
mL43 fraction 5
mL43 fraction 6
mL43 fraction 7
mL43 fraction 8
mL43 fraction 9
mL43 fraction 10
mL43 fraction 11
mL43 fraction 12
mL43 fraction 13
mL43 fraction 14
mL43 fraction 15
mL43 fraction 16
log 10 intensity
time (h)

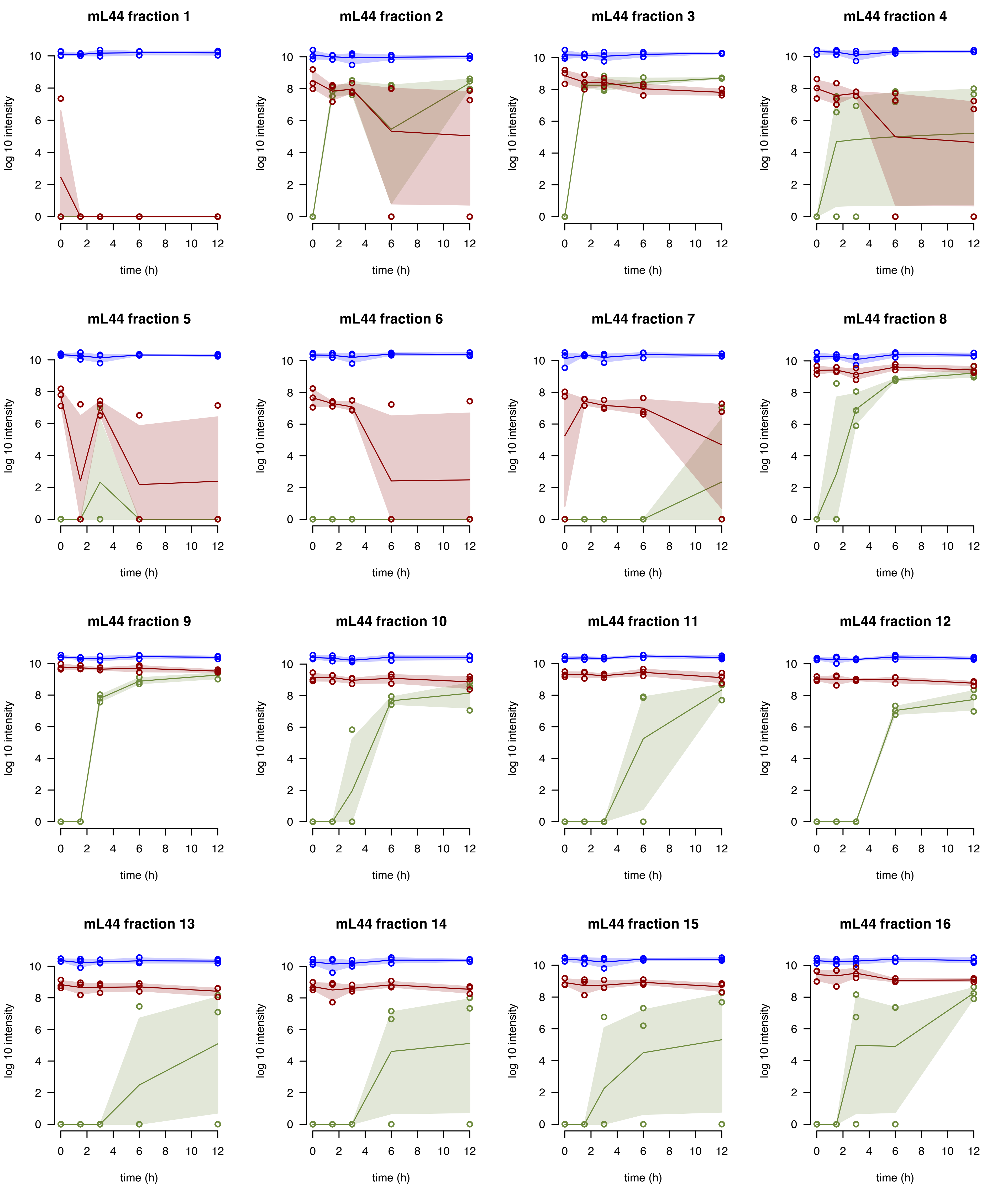
mL44 fraction 1
mL44 fraction 2
mL44 fraction 3
mL44 fraction 4
mL44 fraction 5
mL44 fraction 6
mL44 fraction 7
mL44 fraction 8
mL44 fraction 9
mL44 fraction 10
mL44 fraction 11
mL44 fraction 12
mL44 fraction 13
mL44 fraction 14
mL44 fraction 15
mL44 fraction 16
log 10 intensity
time (h)
0
2
4
6
8
10
12

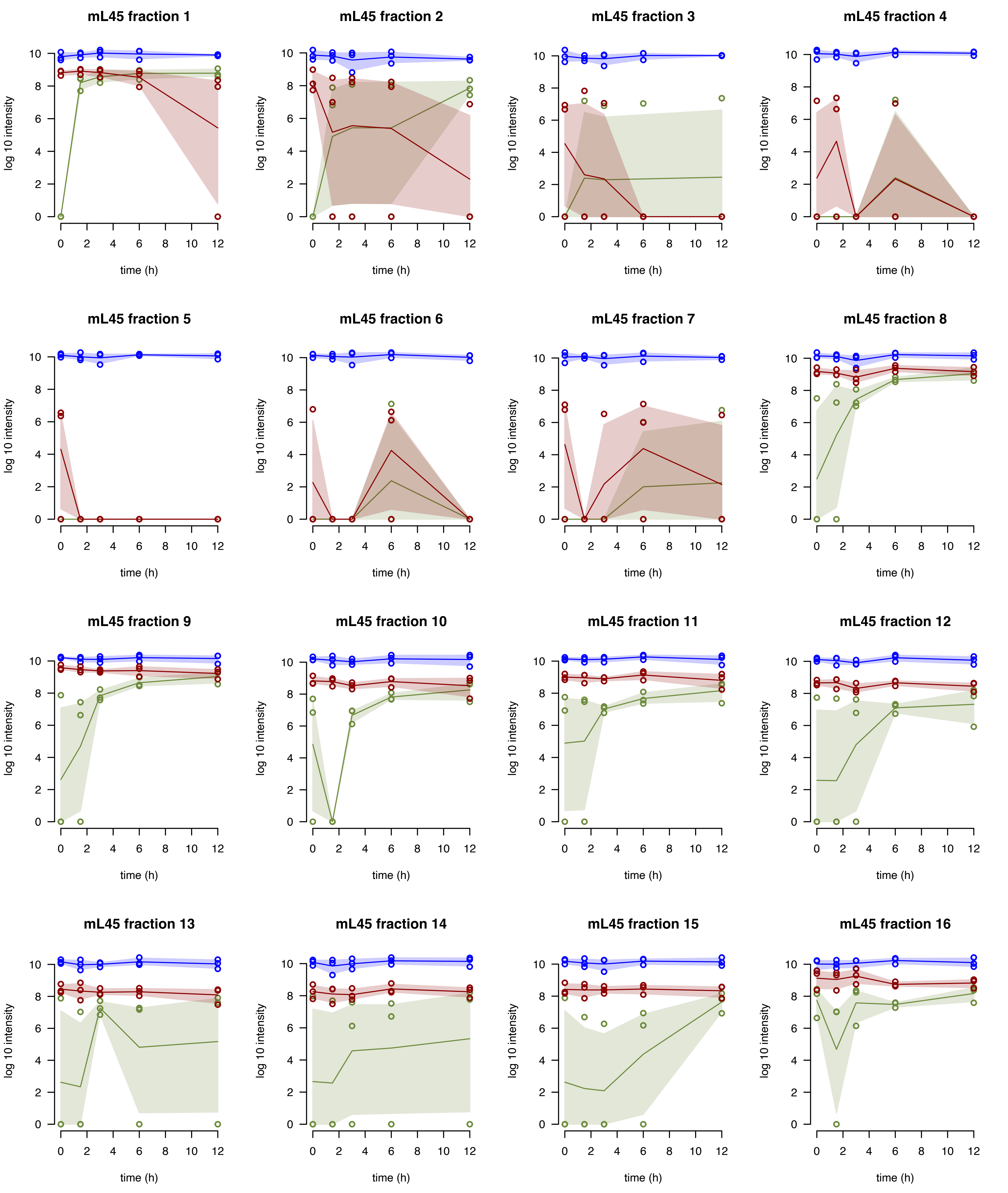
mL45 fraction 1
mL45 fraction 2
mL45 fraction 3
mL45 fraction 4
mL45 fraction 5
mL45 fraction 6
mL45 fraction 7
mL45 fraction 8
mL45 fraction 9
mL45 fraction 10
mL45 fraction 11
mL45 fraction 12
mL45 fraction 13
mL45 fraction 14
mL45 fraction 15
mL45 fraction 16
log 10 intensity
time (h)

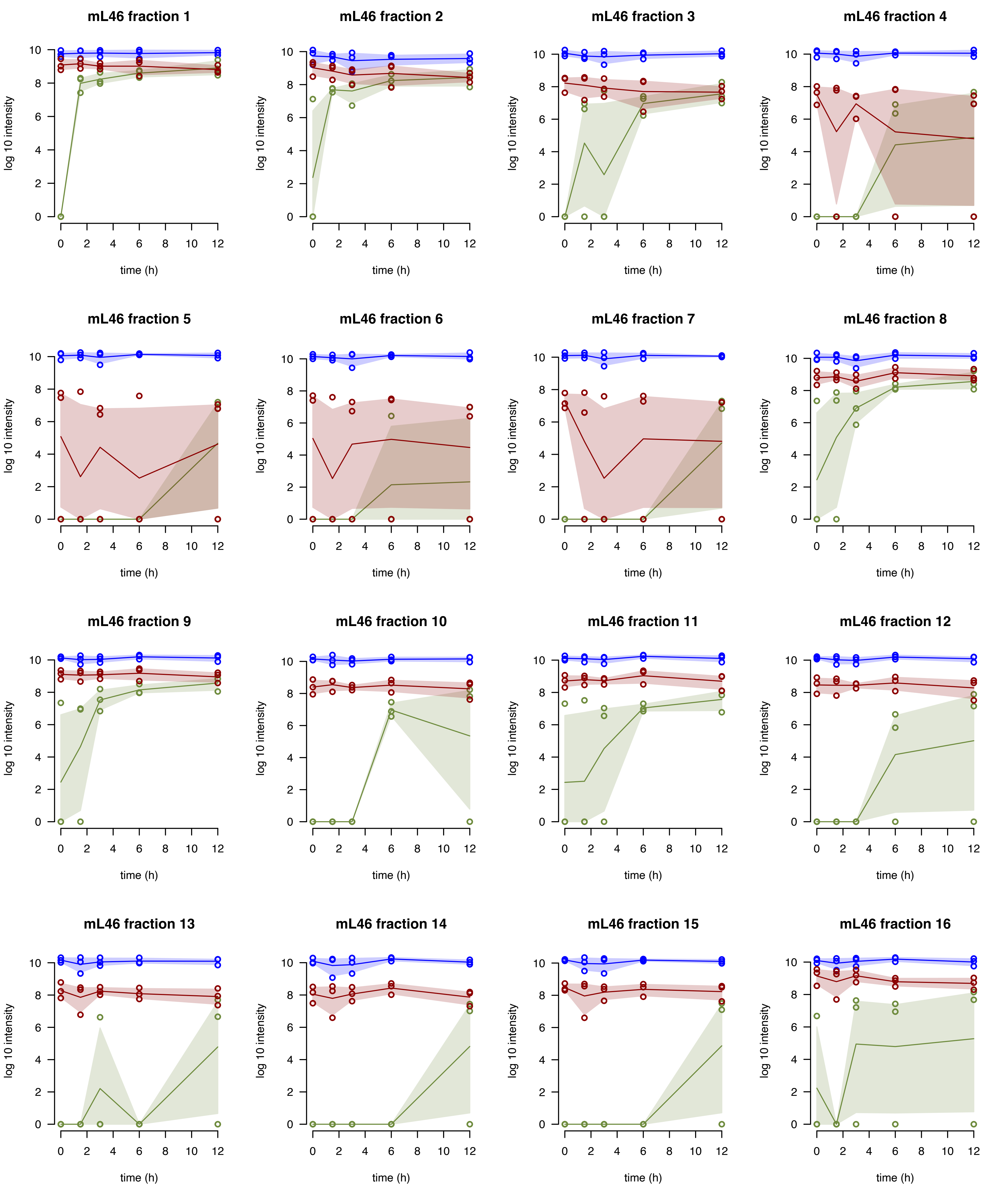
mL46 fraction 1
mL46 fraction 2
mL46 fraction 3
mL46 fraction 4
mL46 fraction 5
mL46 fraction 6
mL46 fraction 7
mL46 fraction 8
mL46 fraction 9
mL46 fraction 10
mL46 fraction 11
mL46 fraction 12
mL46 fraction 13
mL46 fraction 14
mL46 fraction 15
mL46 fraction 16
log 10 intensity
time (h)

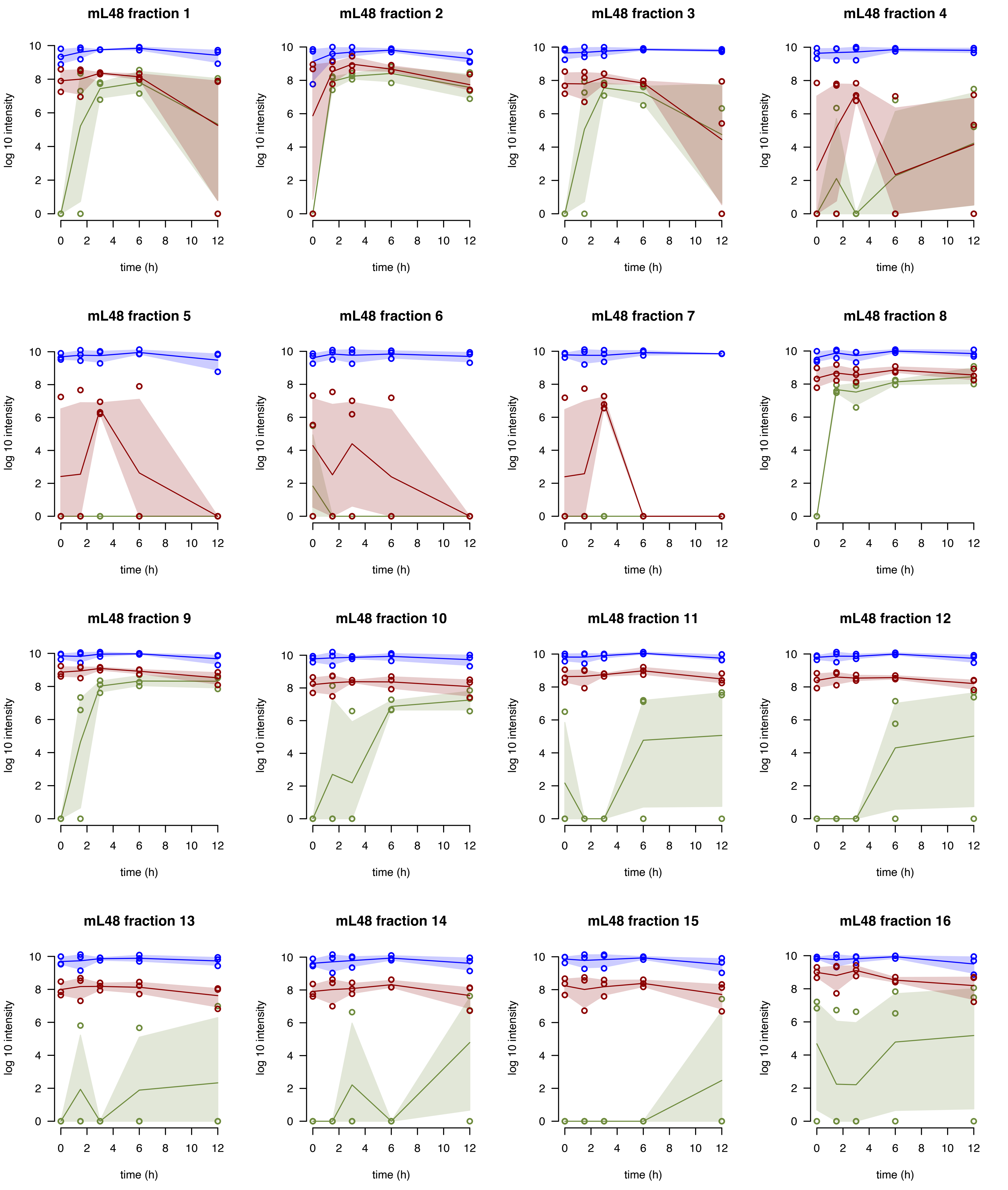
mL48 fraction 1
mL48 fraction 2
mL48 fraction 3
mL48 fraction 4
mL48 fraction 5
mL48 fraction 6
mL48 fraction 7
mL48 fraction 8
mL48 fraction 9
mL48 fraction 10
mL48 fraction 11
mL48 fraction 12
mL48 fraction 13
mL48 fraction 14
mL48 fraction 15
mL48 fraction 16
log 10 intensity
time (h)

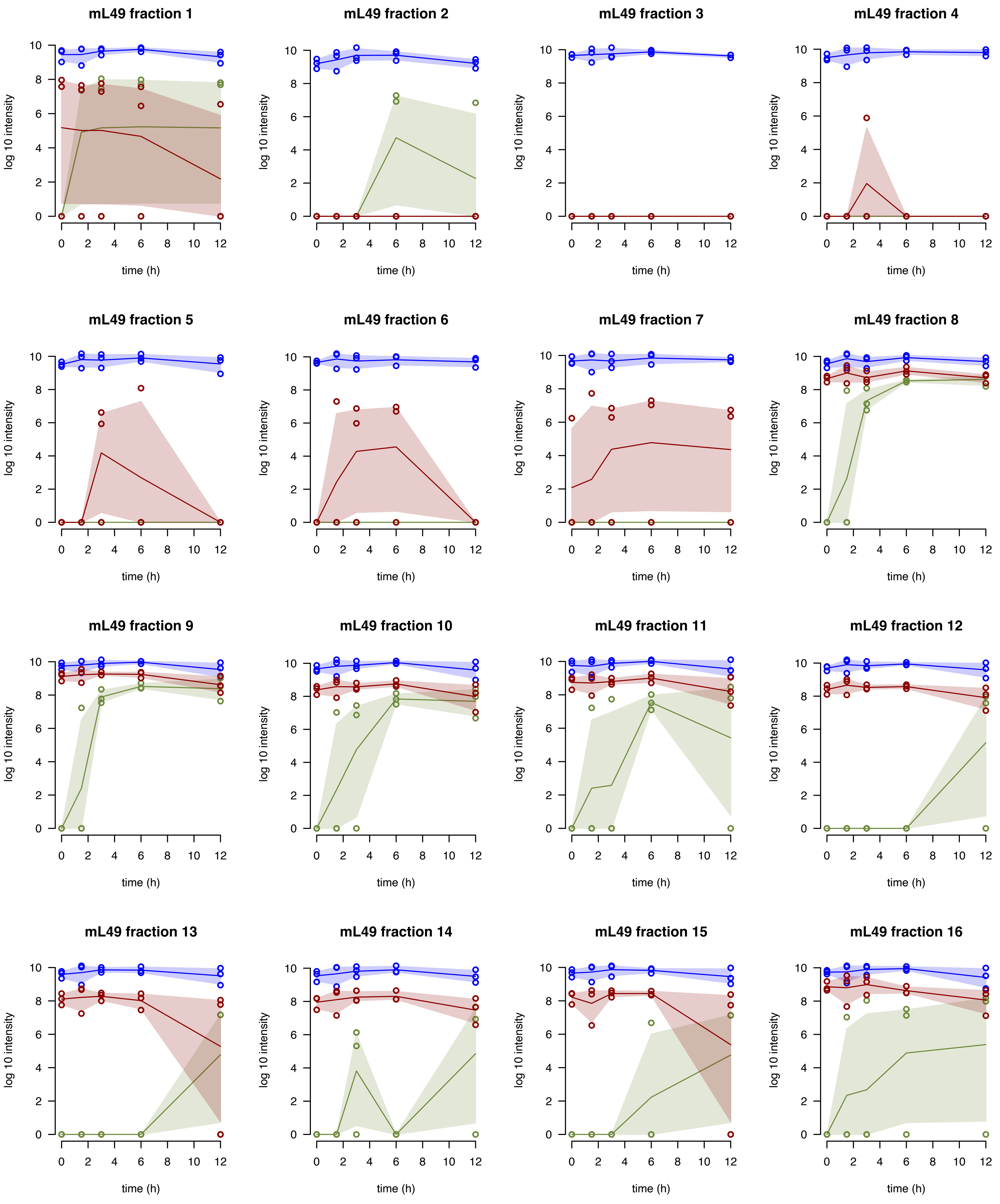
mL49 fraction 1
mL49 fraction 2
mL49 fraction 3
mL49 fraction 4
mL49 fraction 5
mL49 fraction 6
mL49 fraction 7
mL49 fraction 8
mL49 fraction 9
mL49 fraction 10
mL49 fraction 11
mL49 fraction 12
mL49 fraction 13
mL49 fraction 14
mL49 fraction 15
mL49 fraction 16
log 10 intensity
time (h)

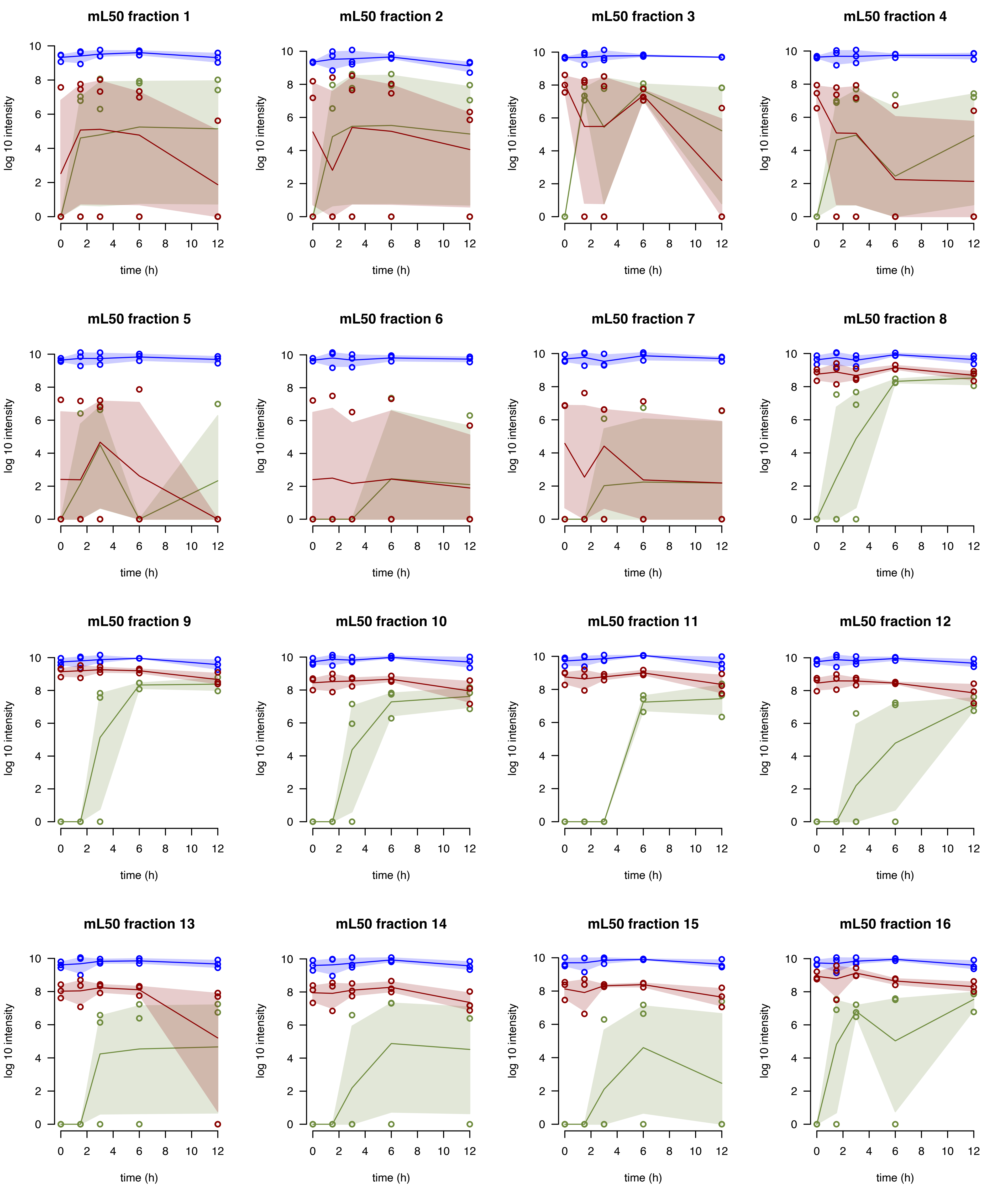
mL50 fraction 1
mL50 fraction 2
mL50 fraction 3
mL50 fraction 4
mL50 fraction 5
mL50 fraction 6
mL50 fraction 7
mL50 fraction 8
mL50 fraction 9
mL50 fraction 10
mL50 fraction 11
mL50 fraction 12
mL50 fraction 13
mL50 fraction 14
mL50 fraction 15
mL50 fraction 16
log 10 intensity
time (h)

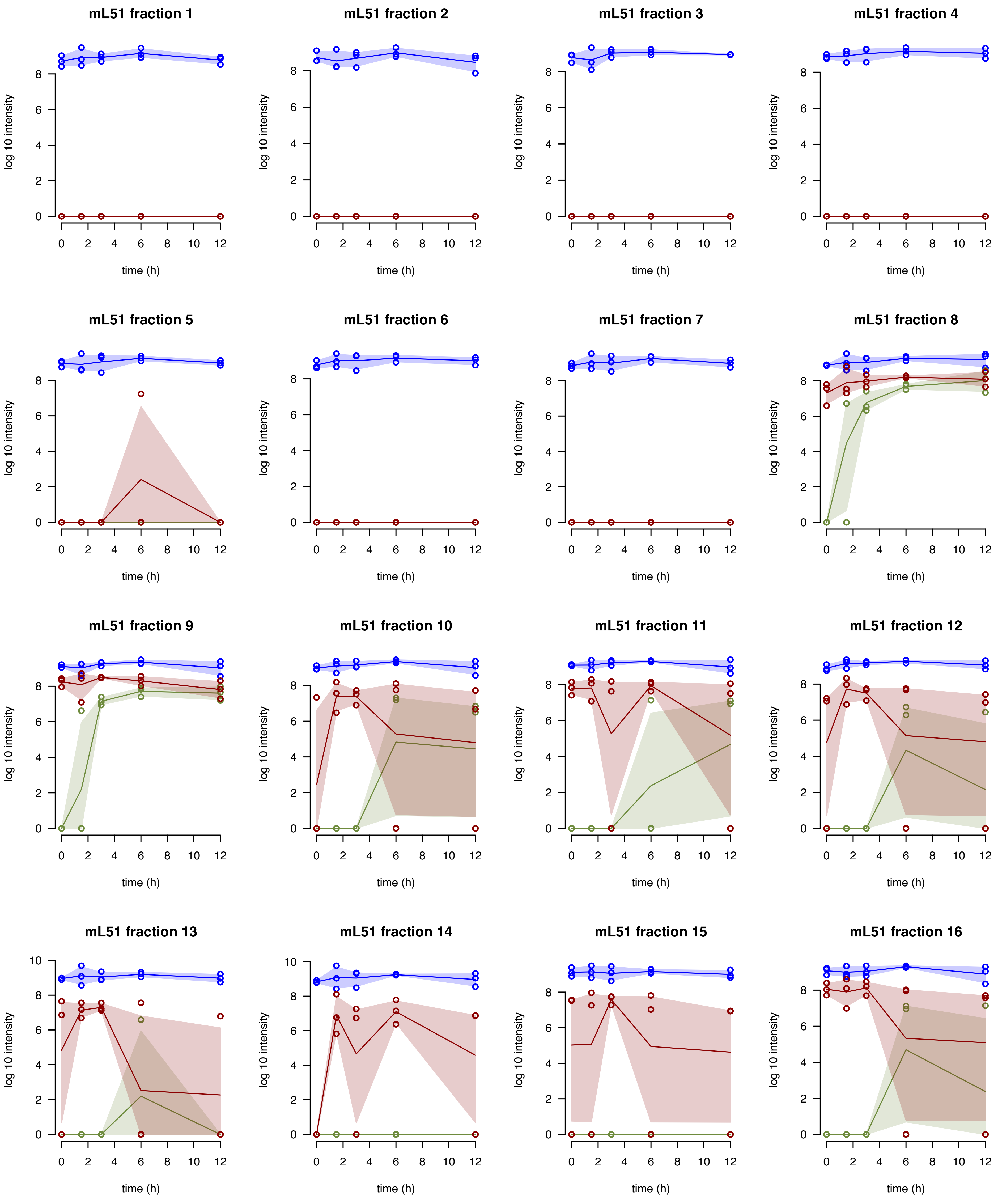
mL51 fraction 1
mL51 fraction 2
mL51 fraction 3
mL51 fraction 4
mL51 fraction 5
mL51 fraction 6
mL51 fraction 7
mL51 fraction 8
mL51 fraction 9
mL51 fraction 10
mL51 fraction 11
mL51 fraction 12
mL51 fraction 13
mL51 fraction 14
mL51 fraction 15
mL51 fraction 16
log 10 intensity
time (h)

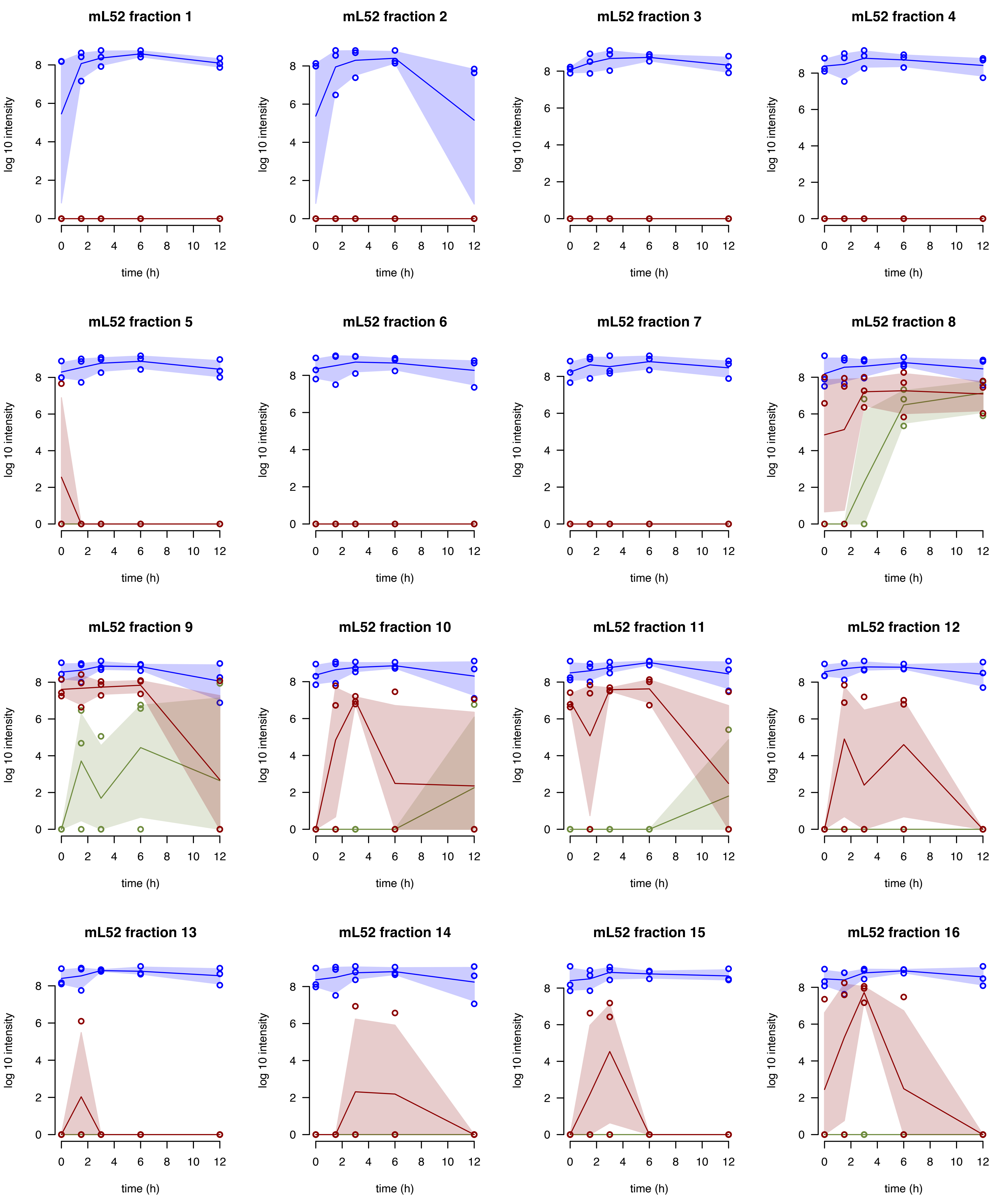
mL52 fraction 1
mL52 fraction 2
mL52 fraction 3
mL52 fraction 4
mL52 fraction 5
mL52 fraction 6
mL52 fraction 7
mL52 fraction 8
mL52 fraction 9
mL52 fraction 10
mL52 fraction 11
mL52 fraction 12
mL52 fraction 13
mL52 fraction 14
mL52 fraction 15
mL52 fraction 16
log 10 intensity
time (h)
0
2
4
6
8
10
12

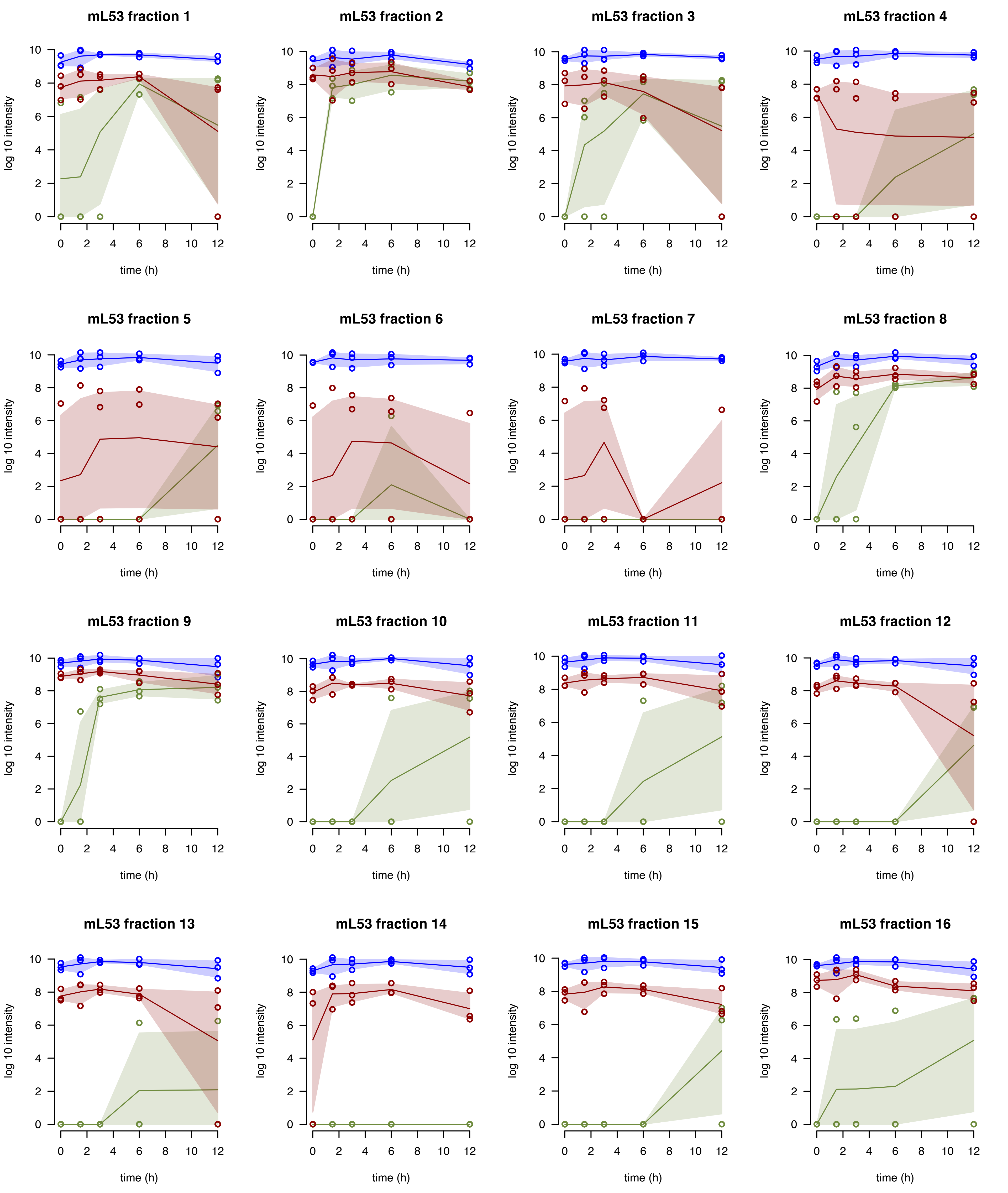
mL53 fraction 1
mL53 fraction 2
mL53 fraction 3
mL53 fraction 4
mL53 fraction 5
mL53 fraction 6
mL53 fraction 7
mL53 fraction 8
mL53 fraction 9
mL53 fraction 10
mL53 fraction 11
mL53 fraction 12
mL53 fraction 13
mL53 fraction 14
mL53 fraction 15
mL53 fraction 16
log 10 intensity
time (h)

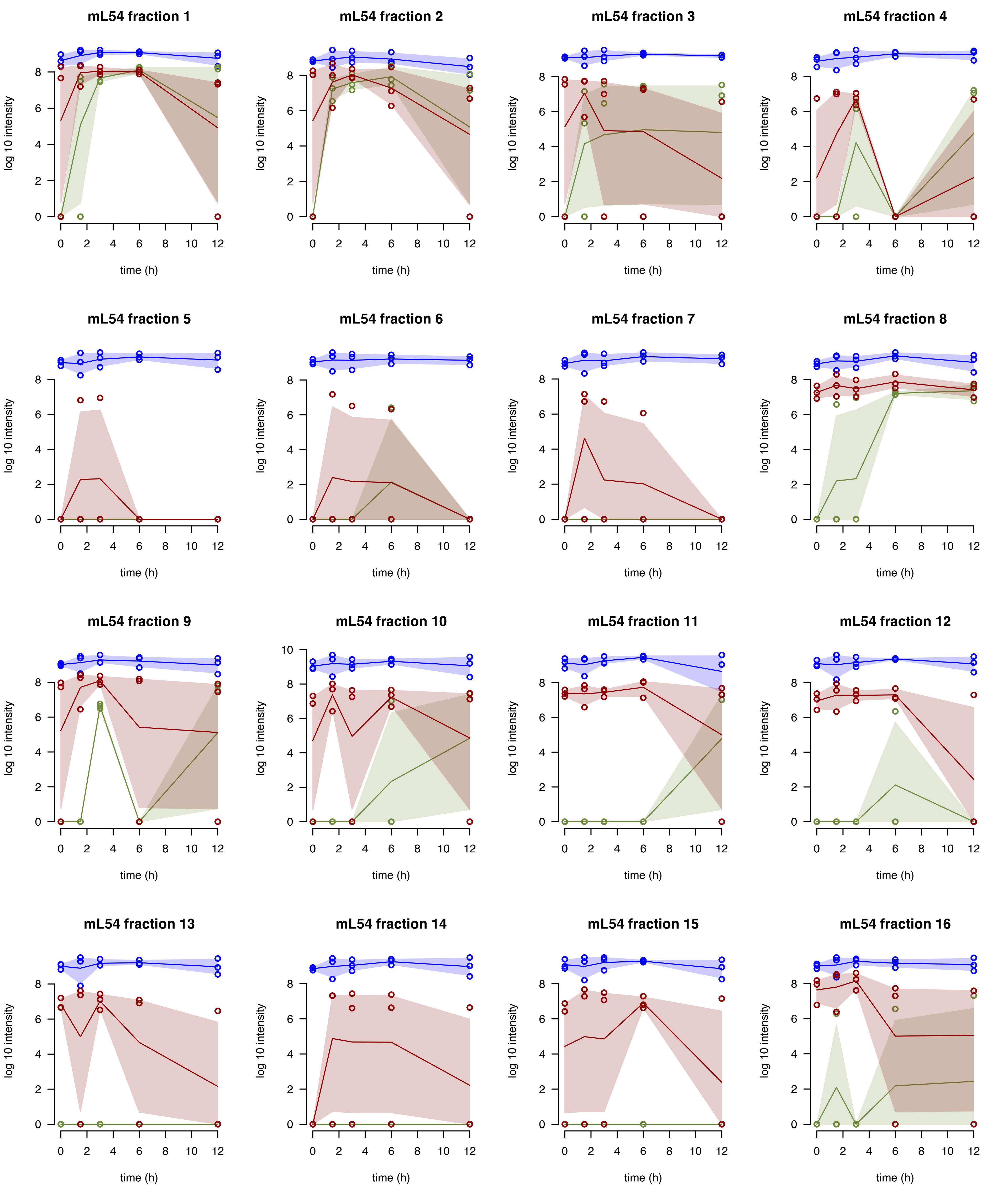
mL54 fraction 1
mL54 fraction 2
mL54 fraction 3
mL54 fraction 4
mL54 fraction 5
mL54 fraction 6
mL54 fraction 7
mL54 fraction 8
mL54 fraction 9
mL54 fraction 10
mL54 fraction 11
mL54 fraction 12
mL54 fraction 13
mL54 fraction 14
mL54 fraction 15
mL54 fraction 16
log 10 intensity
time (h)
0 2 4 6 8 10 12
0 2 4 6 8 10

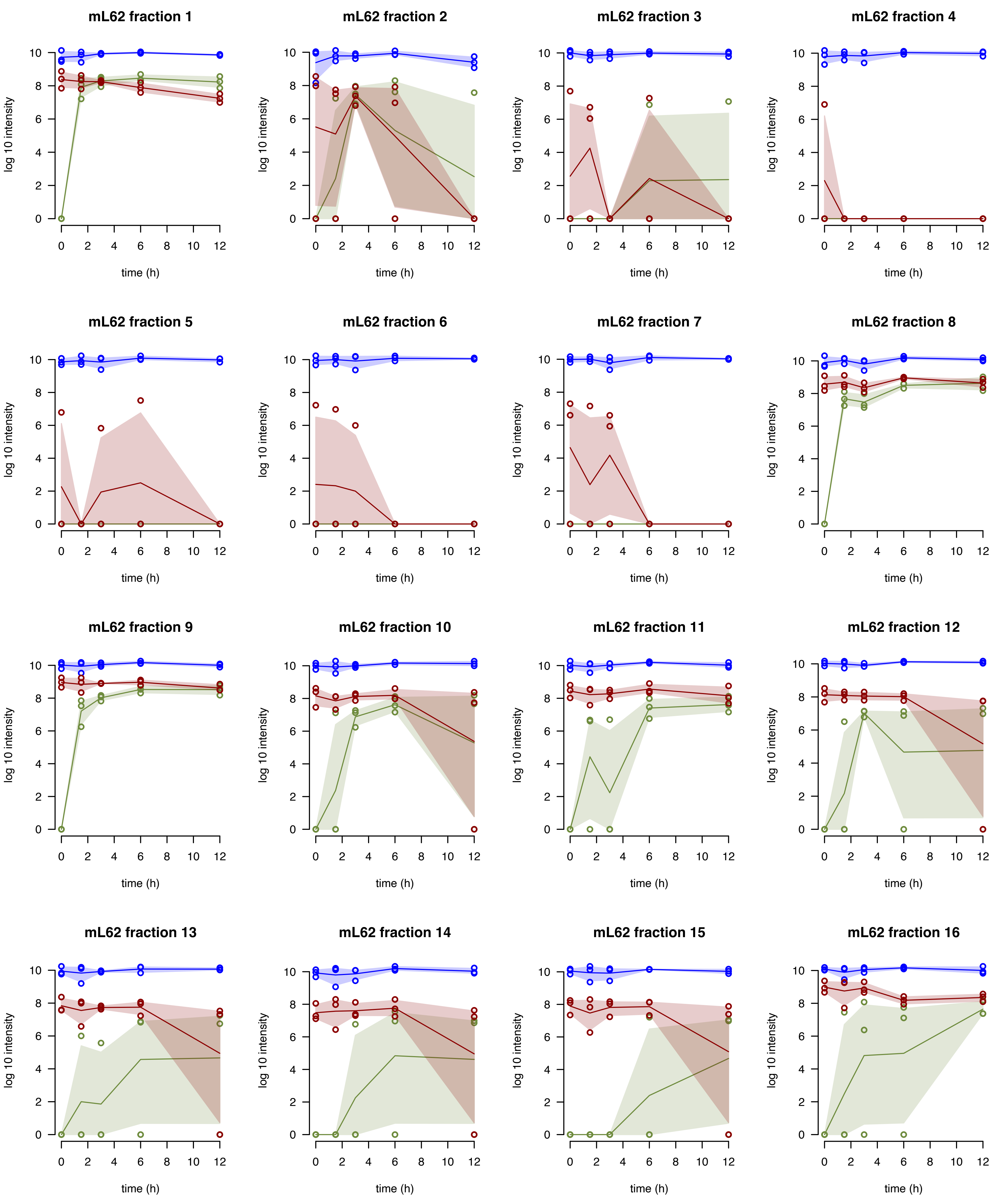

mL62 fraction 1
mL62 fraction 2
mL62 fraction 3
mL62 fraction 4
mL62 fraction 5
mL62 fraction 6
mL62 fraction 7
mL62 fraction 8
mL62 fraction 9
mL62 fraction 10
mL62 fraction 11
mL62 fraction 12
mL62 fraction 13
mL62 fraction 14
mL62 fraction 15
mL62 fraction 16
log 10 intensity
time (h)

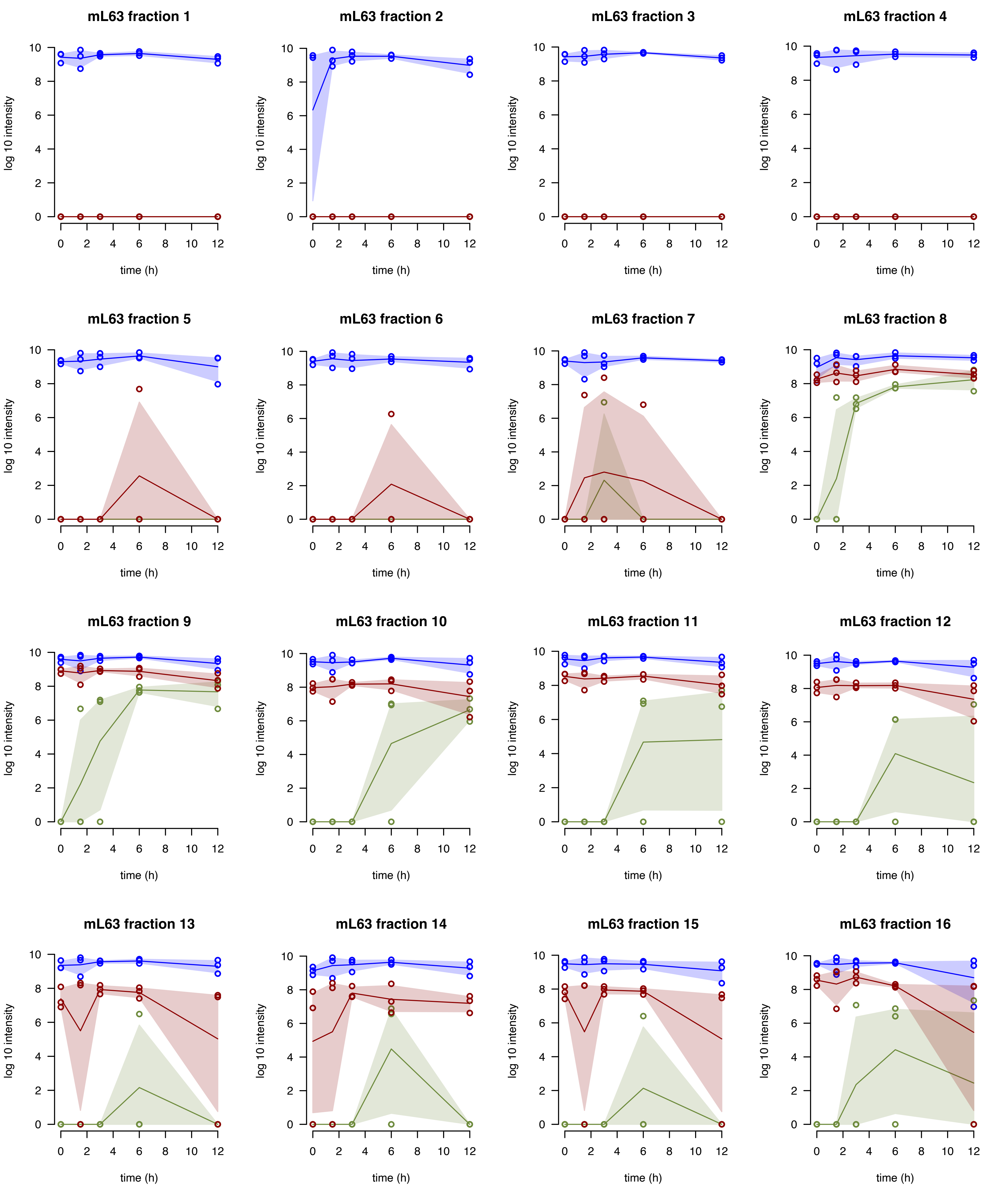

mL63 fraction 1
mL63 fraction 2
mL63 fraction 3
mL63 fraction 4
mL63 fraction 5
mL63 fraction 6
mL63 fraction 7
mL63 fraction 8
mL63 fraction 9
mL63 fraction 10
mL63 fraction 11
mL63 fraction 12
mL63 fraction 13
mL63 fraction 14
mL63 fraction 15
mL63 fraction 16
log 10 intensity
time (h)
0
2
4
6
8
10
12

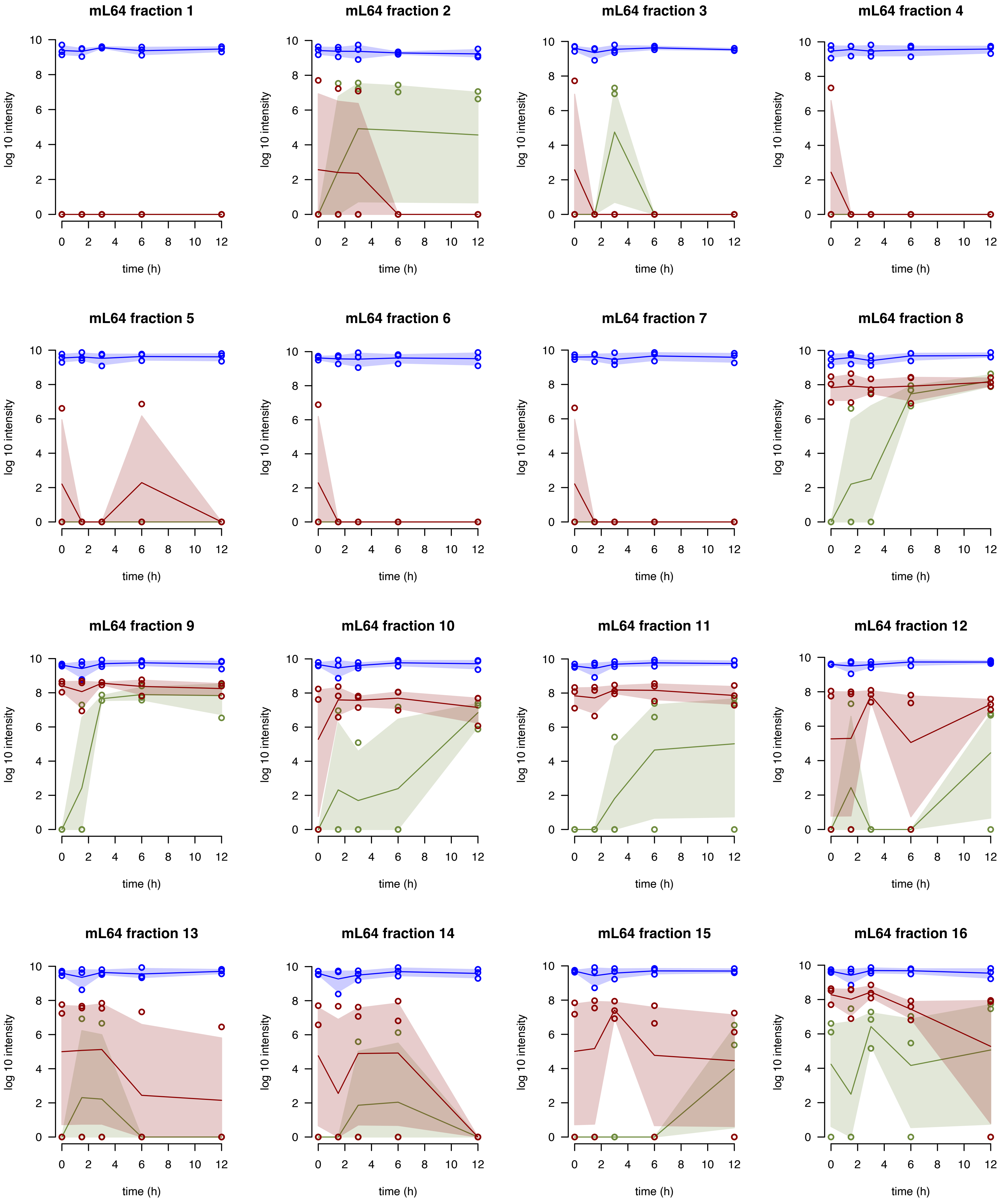

mL64 fraction 1
mL64 fraction 2
mL64 fraction 3
mL64 fraction 4
mL64 fraction 5
mL64 fraction 6
mL64 fraction 7
mL64 fraction 8
mL64 fraction 9
mL64 fraction 10
mL64 fraction 11
mL64 fraction 12
mL64 fraction 13
mL64 fraction 14
mL64 fraction 15
mL64 fraction 16
log 10 intensity
time (h)
0
2
4
6
8
10
12

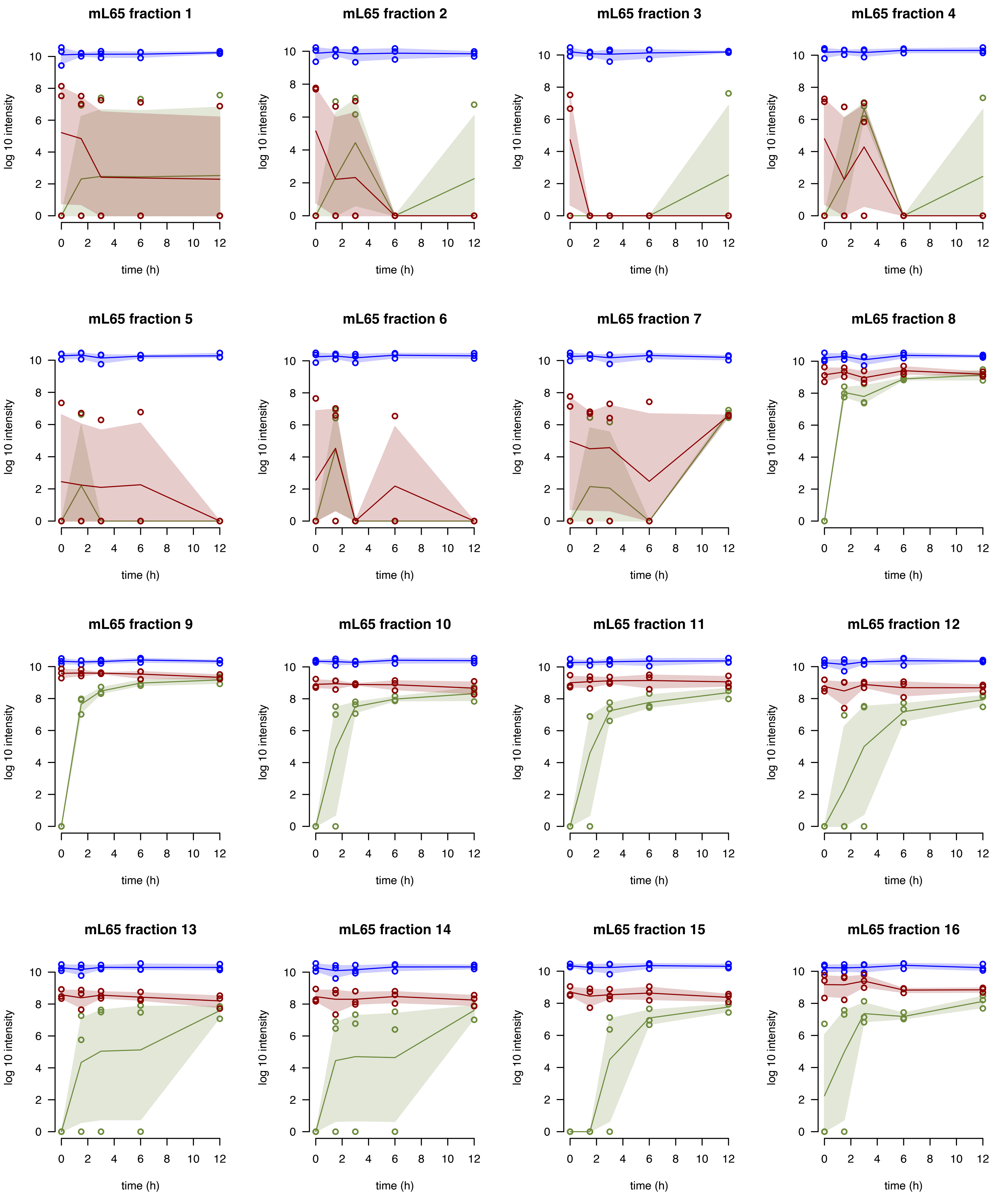
mL65 fraction 1
mL65 fraction 2
mL65 fraction 3
mL65 fraction 4
mL65 fraction 5
mL65 fraction 6
mL65 fraction 7
mL65 fraction 8
mL65 fraction 9
mL65 fraction 10
mL65 fraction 11
mL65 fraction 12
mL65 fraction 13
mL65 fraction 14
mL65 fraction 15
mL65 fraction 16
log 10 intensity
time (h)
0
2
4
6
8
10
12

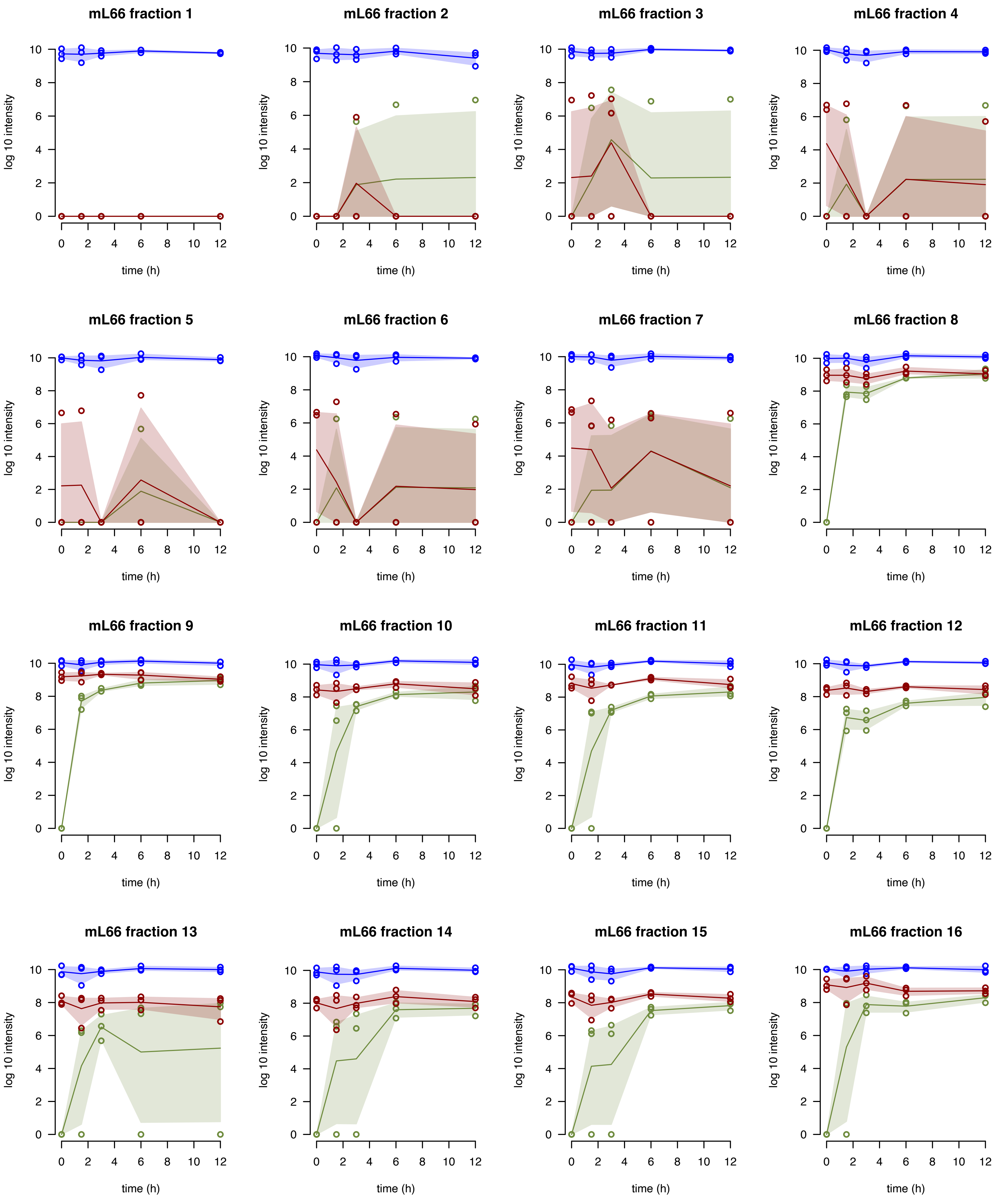
mL66 fraction 1
mL66 fraction 2
mL66 fraction 3
mL66 fraction 4
mL66 fraction 5
mL66 fraction 6
mL66 fraction 7
mL66 fraction 8
mL66 fraction 9
mL66 fraction 10
mL66 fraction 11
mL66 fraction 12
mL66 fraction 13
mL66 fraction 14
mL66 fraction 15
mL66 fraction 16
log 10 intensity
time (h)
0
2
4
6
8
10
12
