# Supplementary material for: A roadmap for ribosome assembly in human mitochondria
Source: Nat Struct Mol Biol. 2024 Jul 11;31(12):1898–908. doi: 10.1038/s41594-024-01356-w (PMC11638073; doi:10.1038/s41594-024-01356-w)
Supplement: Supplementary file 9 — Inference of steady-state abundances and fluxes for mtSSU MRPs. Inference of fluxes for all MRPs of the mtSSU. Shown are normalized abundances derived from experimental data (dots) and model fits (median, bold line; 5th and 95th percentiles, thin lines) for sucrose gradient fractions 1–7. Additionally, measured steady-state abundances and inferred fluxes are shown across sucrose gradient fractions. [file 41594_2024_1356_MOESM9_ESM.pdf]

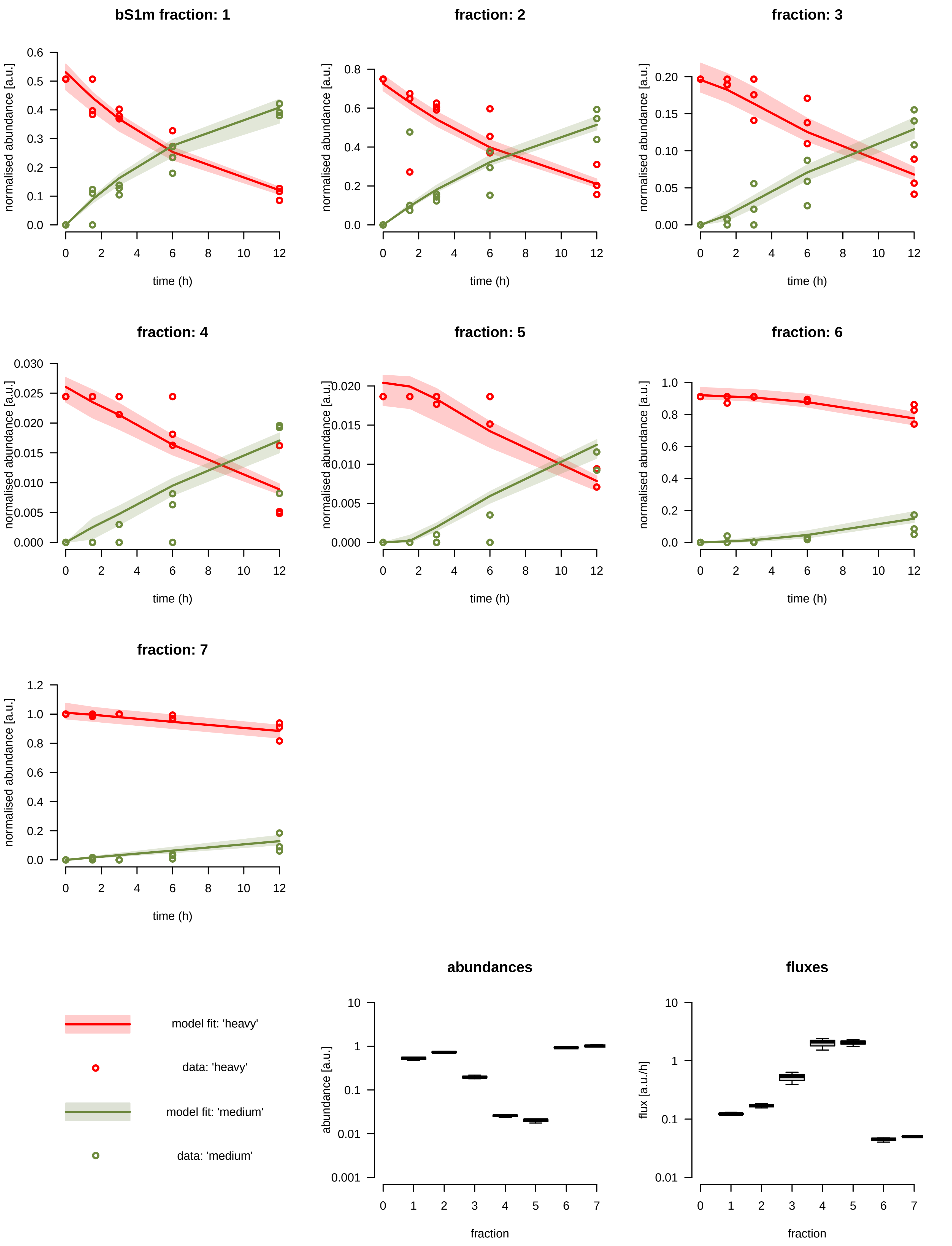
bS1m fraction: 1
fraction: 2
fraction: 3
fraction: 4
fraction: 5
fraction: 6
fraction: 7
normalised abundance [a.u.]
time (h)
model fit: 'heavy'
data: 'heavy'
model fit: 'medium'
data: 'medium'
abundances
abundance [a.u.]
fluxes
flux [a.u./h]
fraction

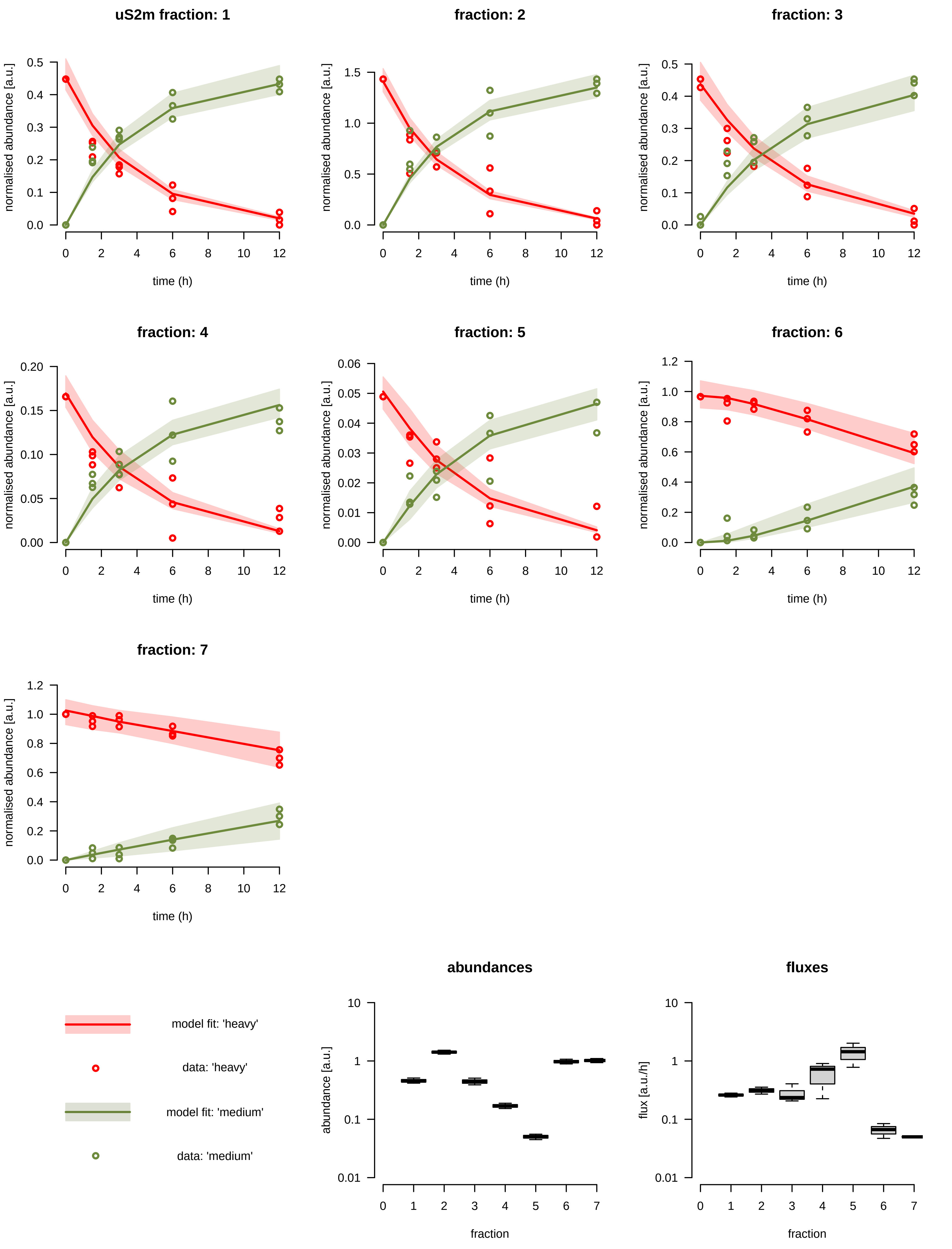

uS2m fraction: 1
fraction: 2
fraction: 3
fraction: 4
fraction: 5
fraction: 6
fraction: 7
normalised abundance [a.u.]
time (h)
model fit: 'heavy'
data: 'heavy'
model fit: 'medium'
data: 'medium'
abundances
abundance [a.u.]
fraction
fluxes
flux [a.u./h]
fraction

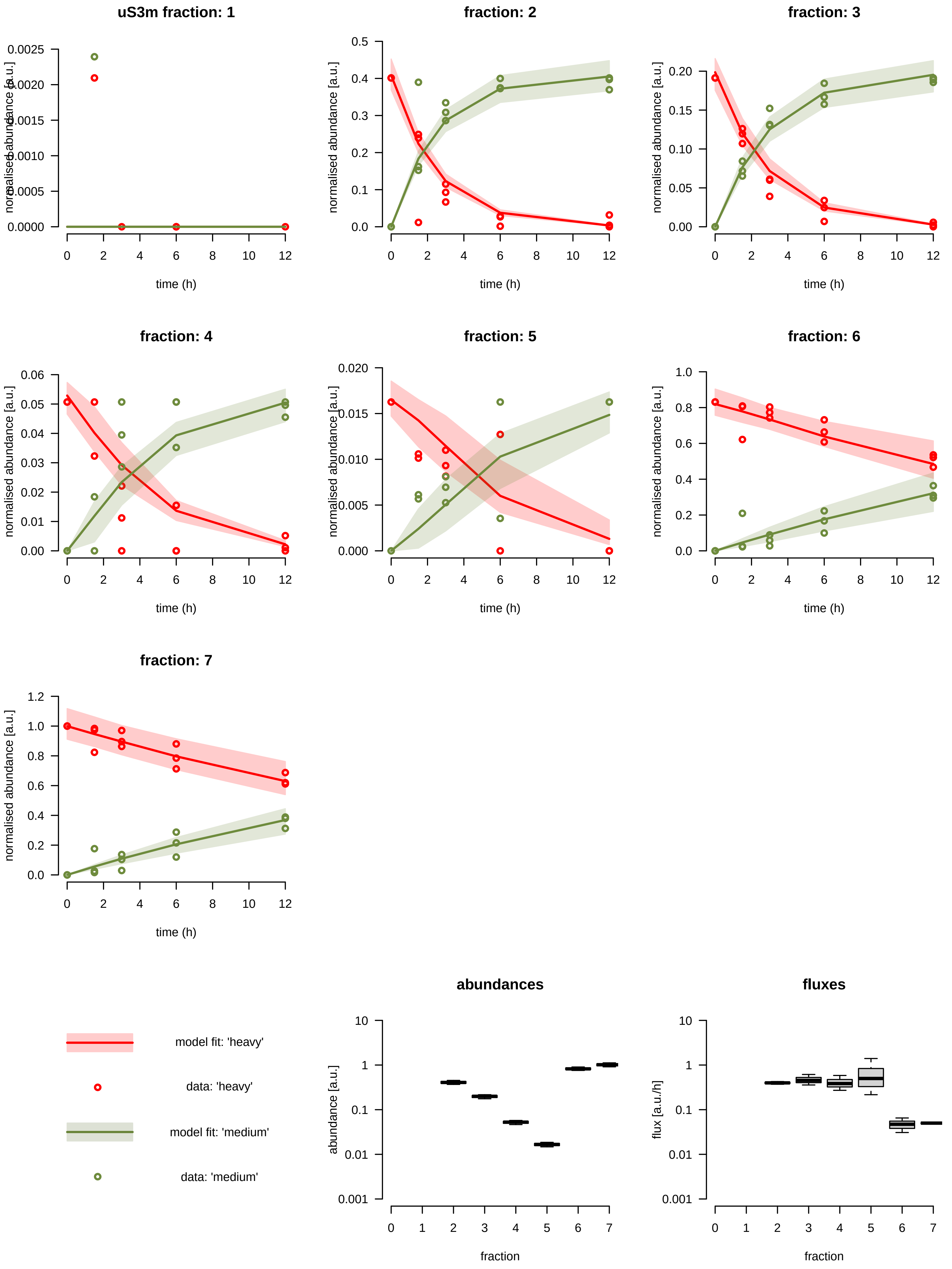

uS3m fraction: 1
fraction: 2
fraction: 3
fraction: 4
fraction: 5
fraction: 6
fraction: 7
normalised abundance [a.u.]
time (h)
model fit: 'heavy'
data: 'heavy'
model fit: 'medium'
data: 'medium'
abundances
abundance [a.u.]
fraction
fluxes
flux [a.u./h]
fraction

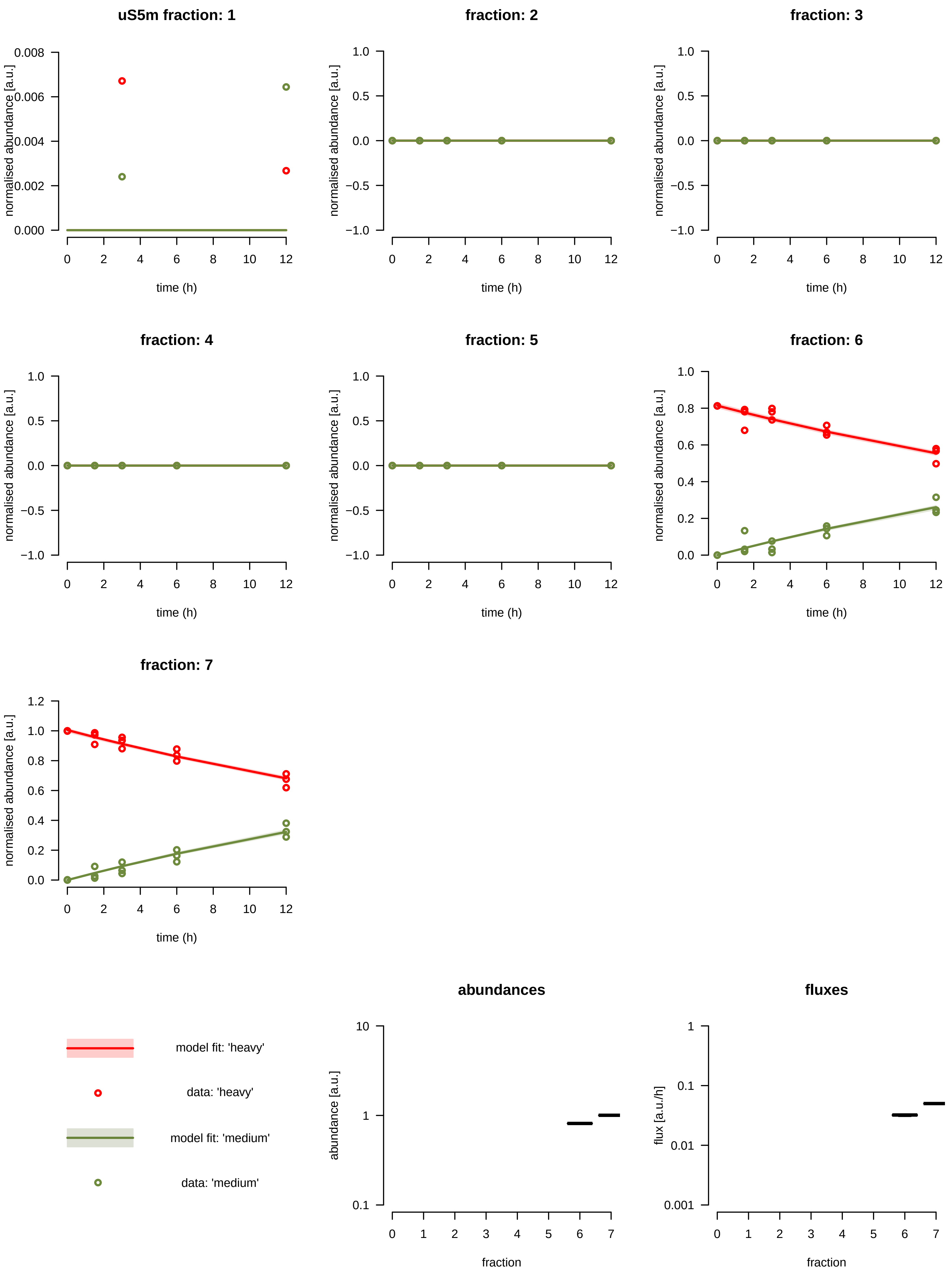
uS5m fraction: 1
fraction: 2
fraction: 3
fraction: 4
fraction: 5
fraction: 6
fraction: 7
normalised abundance [a.u.]
time (h)
model fit: 'heavy'
data: 'heavy'
model fit: 'medium'
data: 'medium'
abundances
abundance [a.u.]
fraction
fluxes
flux [a.u./h]
fraction

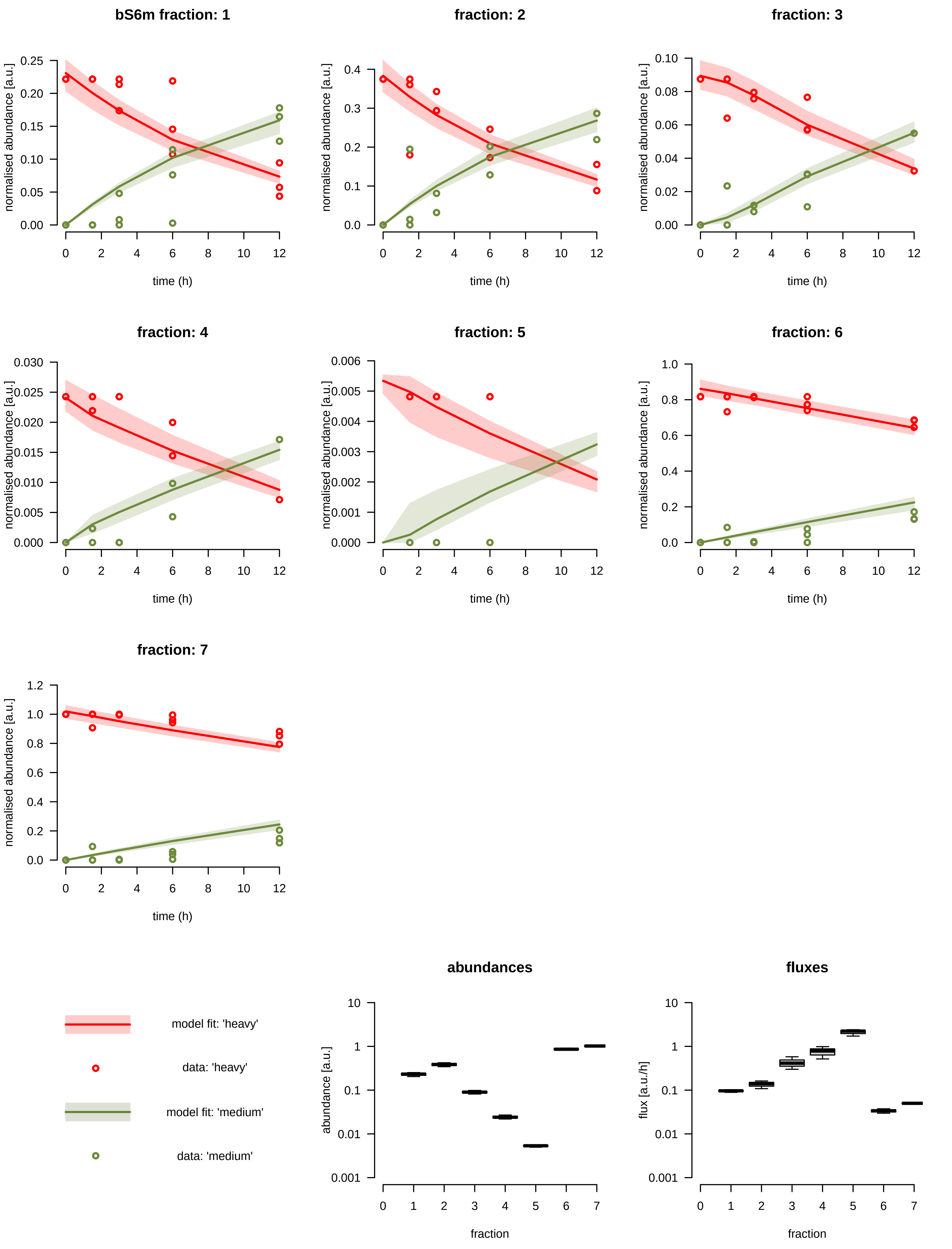

bS6m fraction: 1
fraction: 2
fraction: 3
fraction: 4
fraction: 5
fraction: 6
fraction: 7
normalised abundance [a.u.]
time (h)
model fit: 'heavy'
data: 'heavy'
model fit: 'medium'
data: 'medium'
abundances
abundance [a.u.]
fraction
fluxes
flux [a.u./h]
fraction

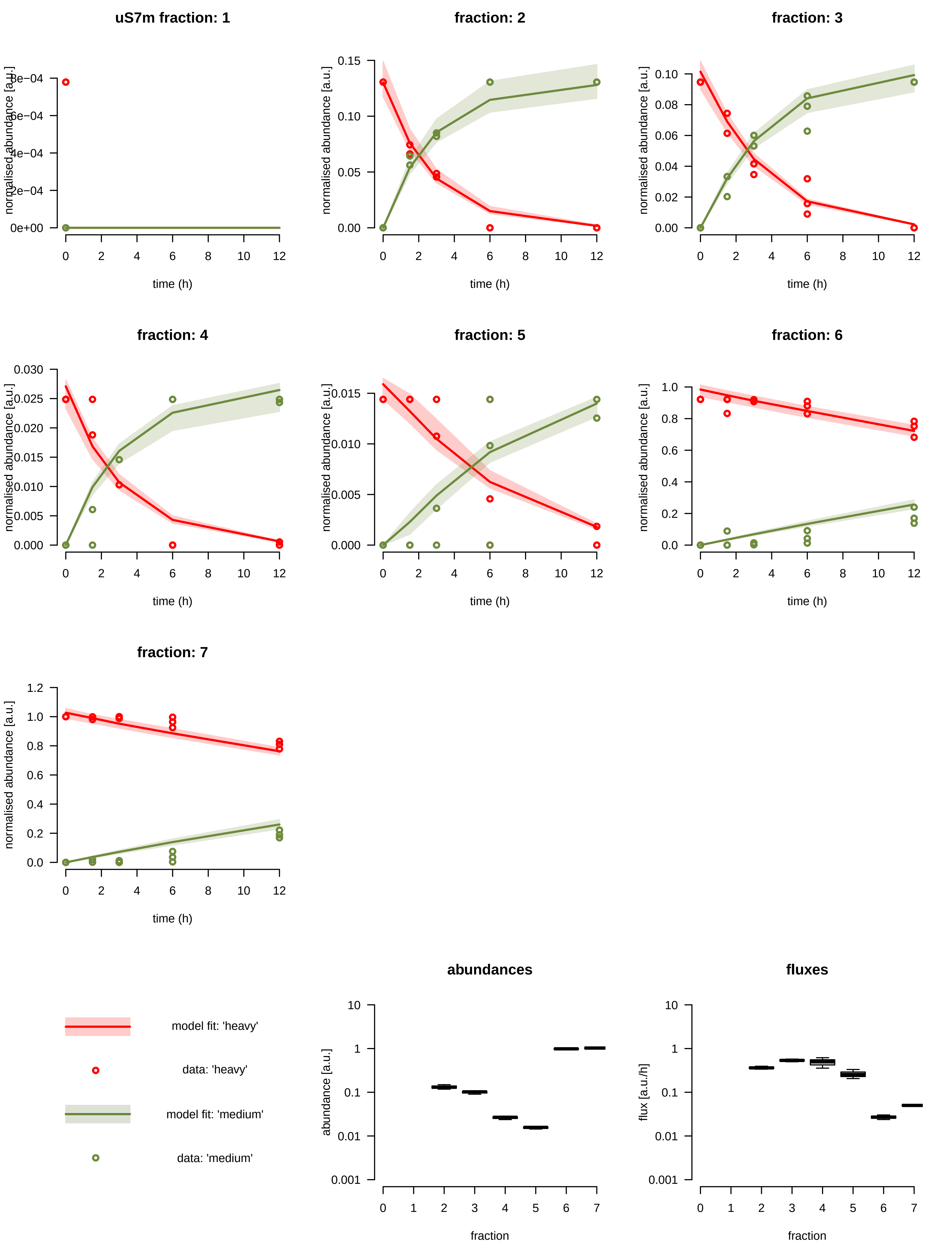
uS7m fraction: 1
fraction: 2
fraction: 3
fraction: 4
fraction: 5
fraction: 6
fraction: 7
normalised abundance [a.u.]
time (h)
model fit: 'heavy'
data: 'heavy'
model fit: 'medium'
data: 'medium'
abundances
abundance [a.u.]
fluxes
flux [a.u./h]
fraction

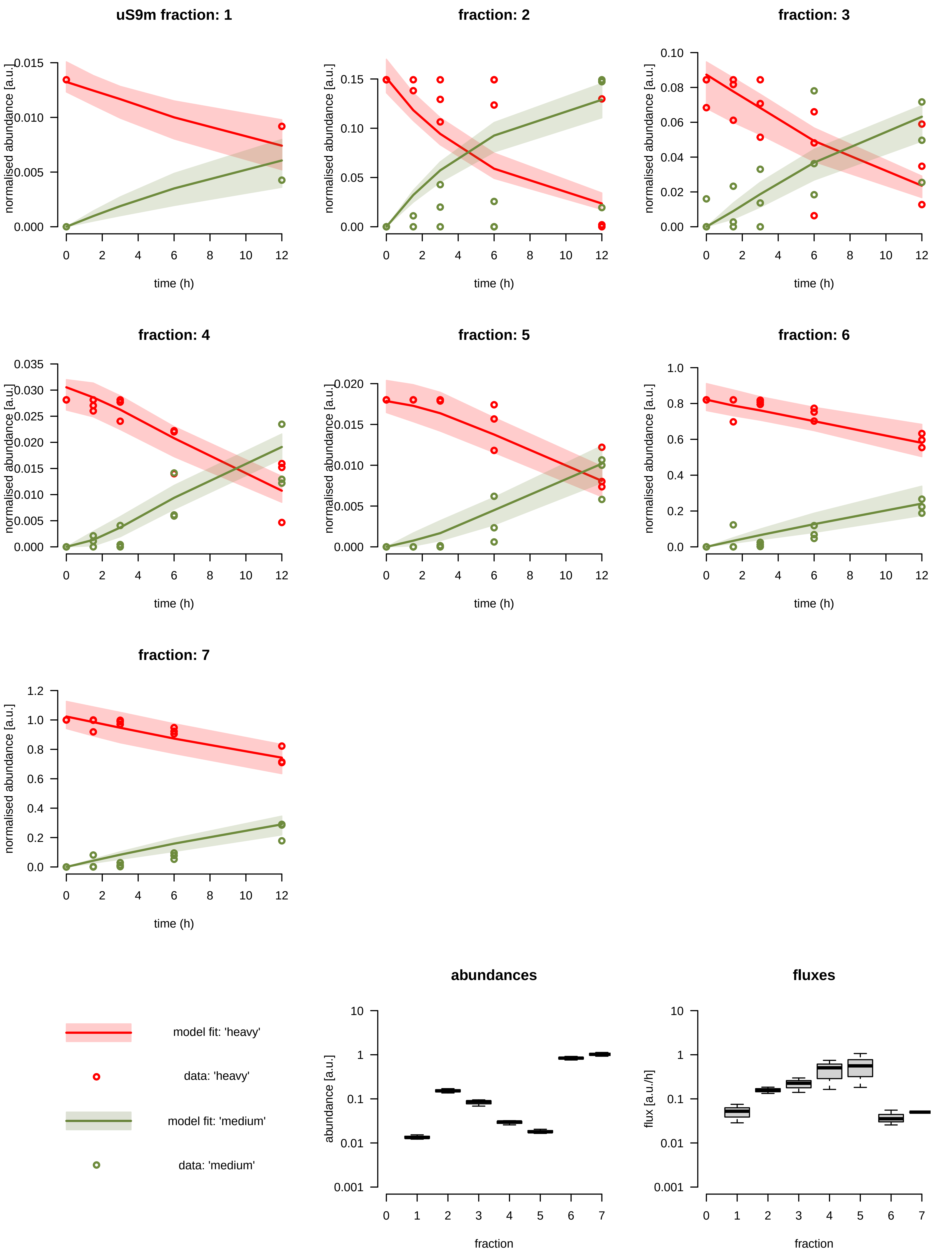
uS9m fraction: 1
fraction: 2
fraction: 3
fraction: 4
fraction: 5
fraction: 6
fraction: 7
normalised abundance [a.u.]
time (h)
model fit: 'heavy'
data: 'heavy'
model fit: 'medium'
data: 'medium'
abundances
abundance [a.u.]
fraction
fluxes
flux [a.u./h]
fraction

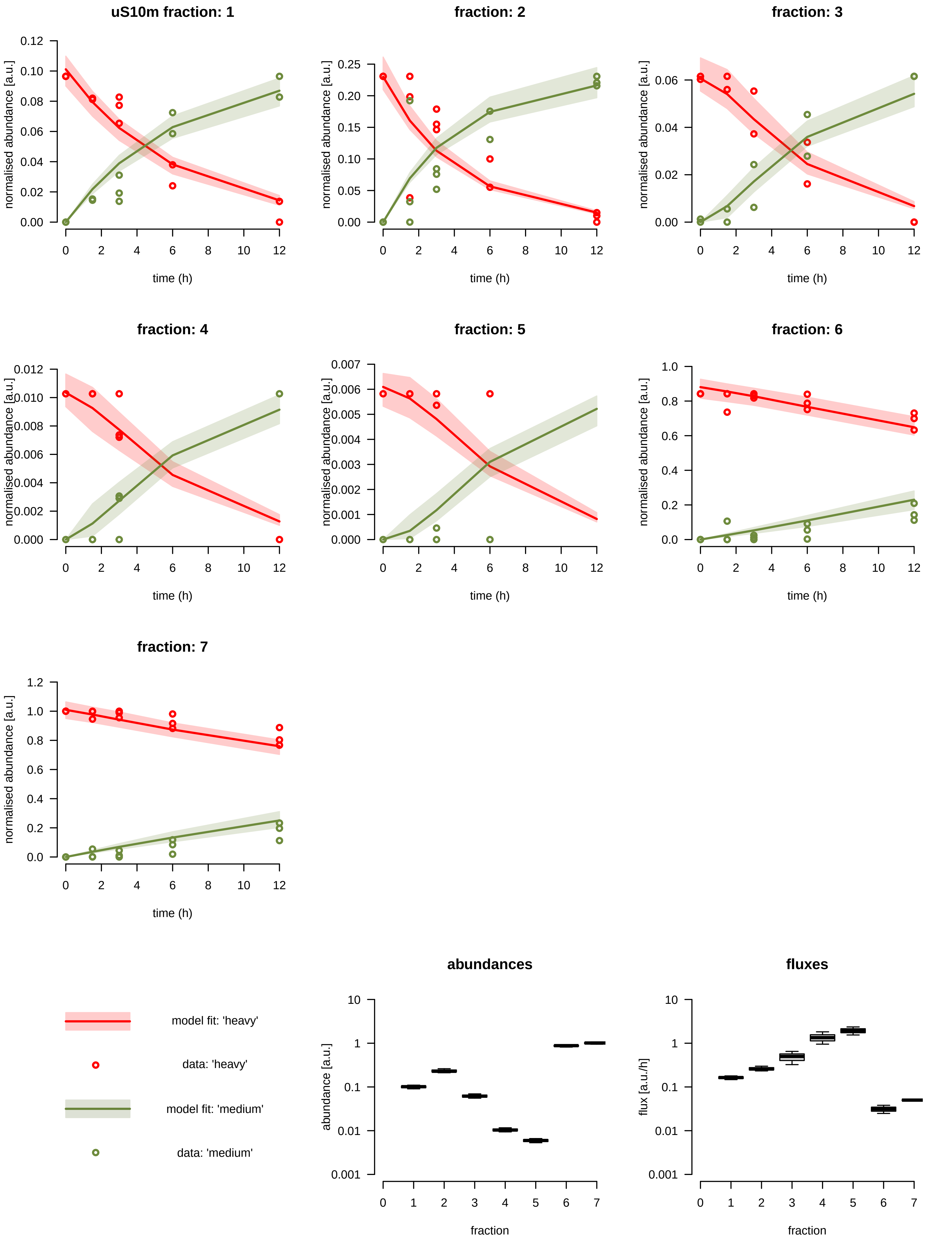
uS10m fraction: 1
fraction: 2
fraction: 3
fraction: 4
fraction: 5
fraction: 6
fraction: 7
normalised abundance [a.u.]
time (h)
model fit: 'heavy'
data: 'heavy'
model fit: 'medium'
data: 'medium'
abundances
abundance [a.u.]
fraction
fluxes
flux [a.u./h]
fraction

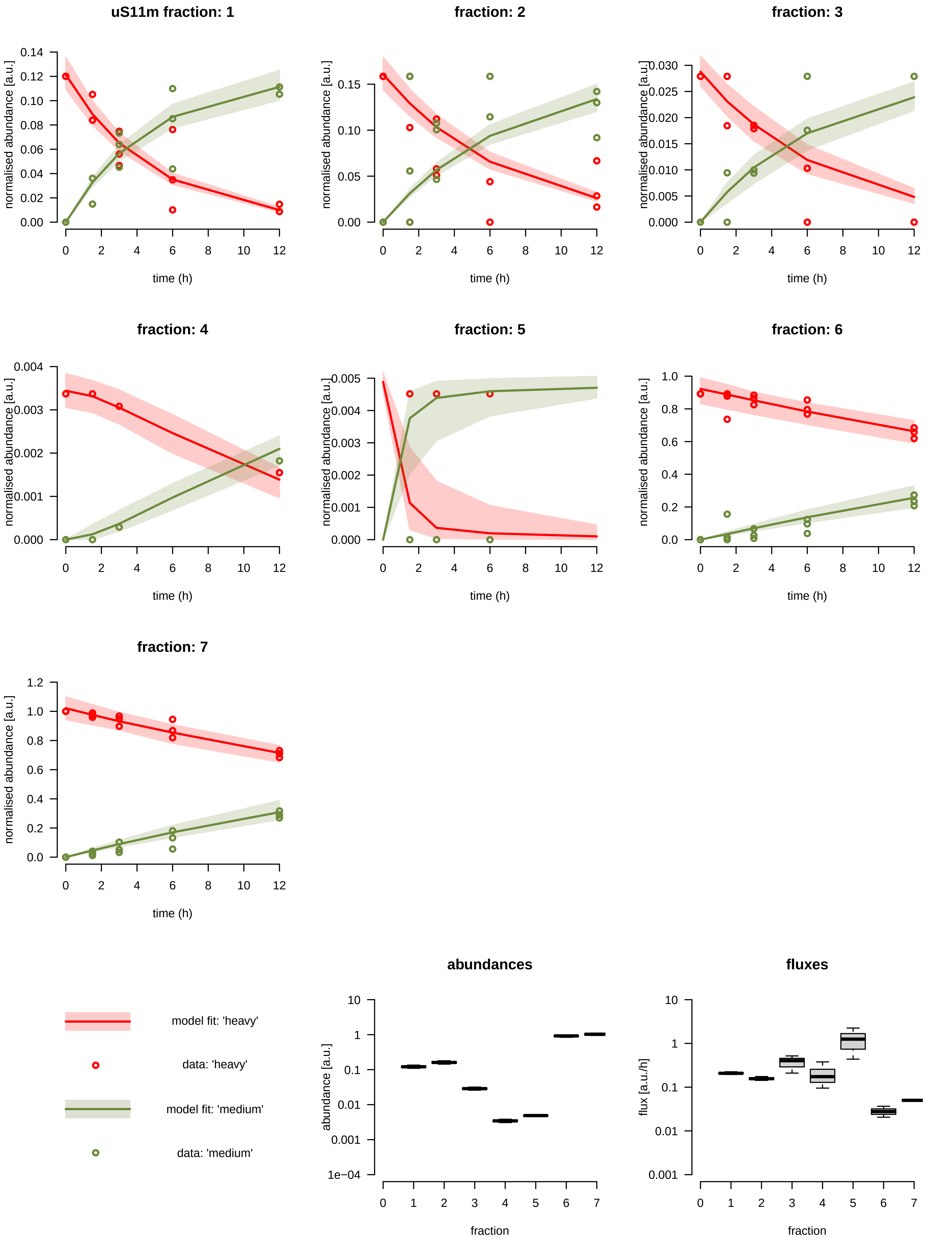
uS11m fraction: 1
fraction: 2
fraction: 3
fraction: 4
fraction: 5
fraction: 6
fraction: 7
normalised abundance [a.u.]
time (h)
model fit: 'heavy'
data: 'heavy'
model fit: 'medium'
data: 'medium'
abundances
abundance [a.u.]
fraction
fluxes
flux [a.u./h]
fraction

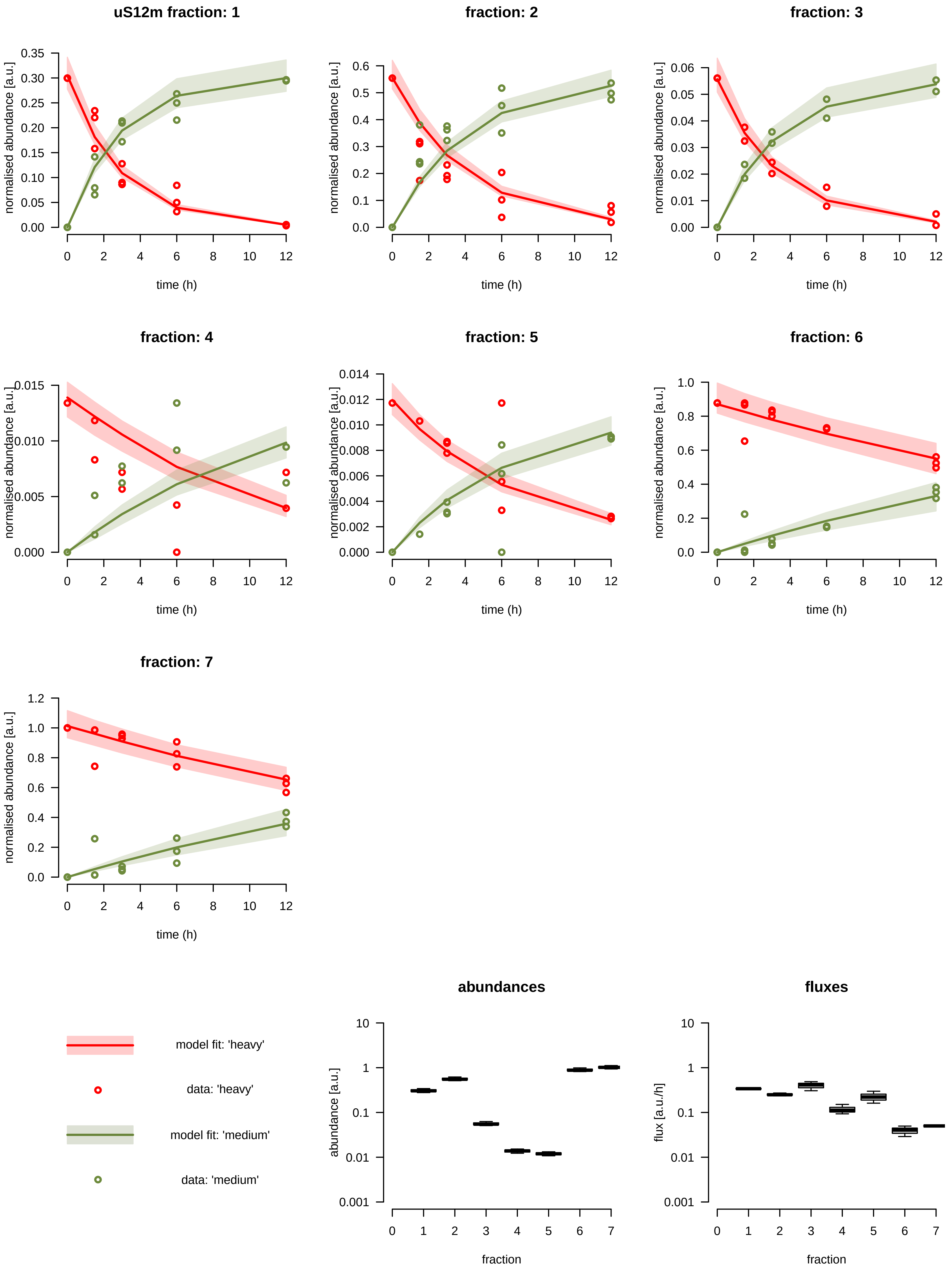

uS12m fraction: 1
fraction: 2
fraction: 3
fraction: 4
fraction: 5
fraction: 6
fraction: 7
normalised abundance [a.u.]
time (h)
model fit: 'heavy'
data: 'heavy'
model fit: 'medium'
data: 'medium'
abundances
abundance [a.u.]
fraction
fluxes
flux [a.u./h]

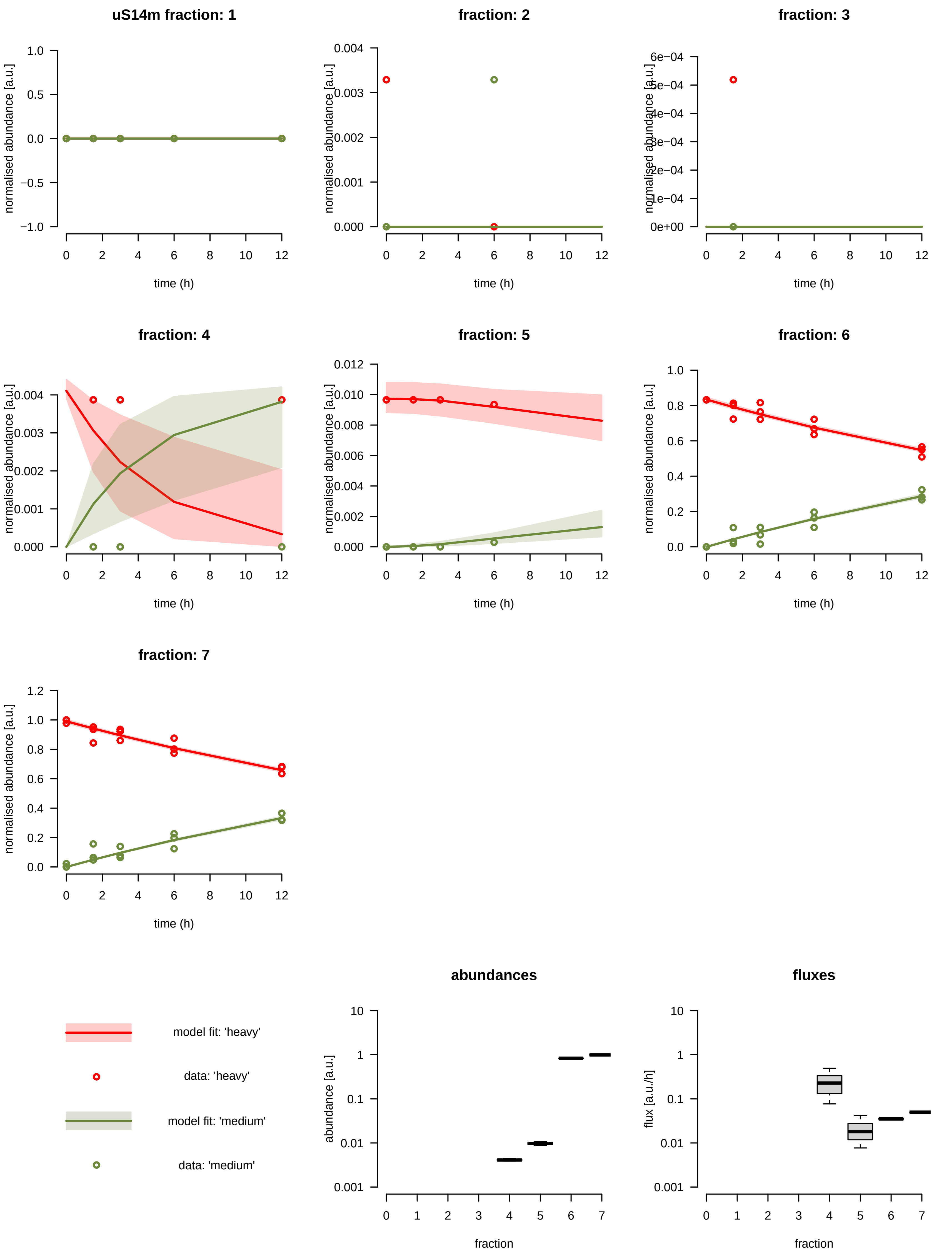
uS14m fraction: 1
fraction: 2
fraction: 3
fraction: 4
fraction: 5
fraction: 6
fraction: 7
normalised abundance [a.u.]
time (h)
model fit: 'heavy'
data: 'heavy'
model fit: 'medium'
data: 'medium'
abundances
abundance [a.u.]
fraction
fluxes
flux [a.u./h]
fraction

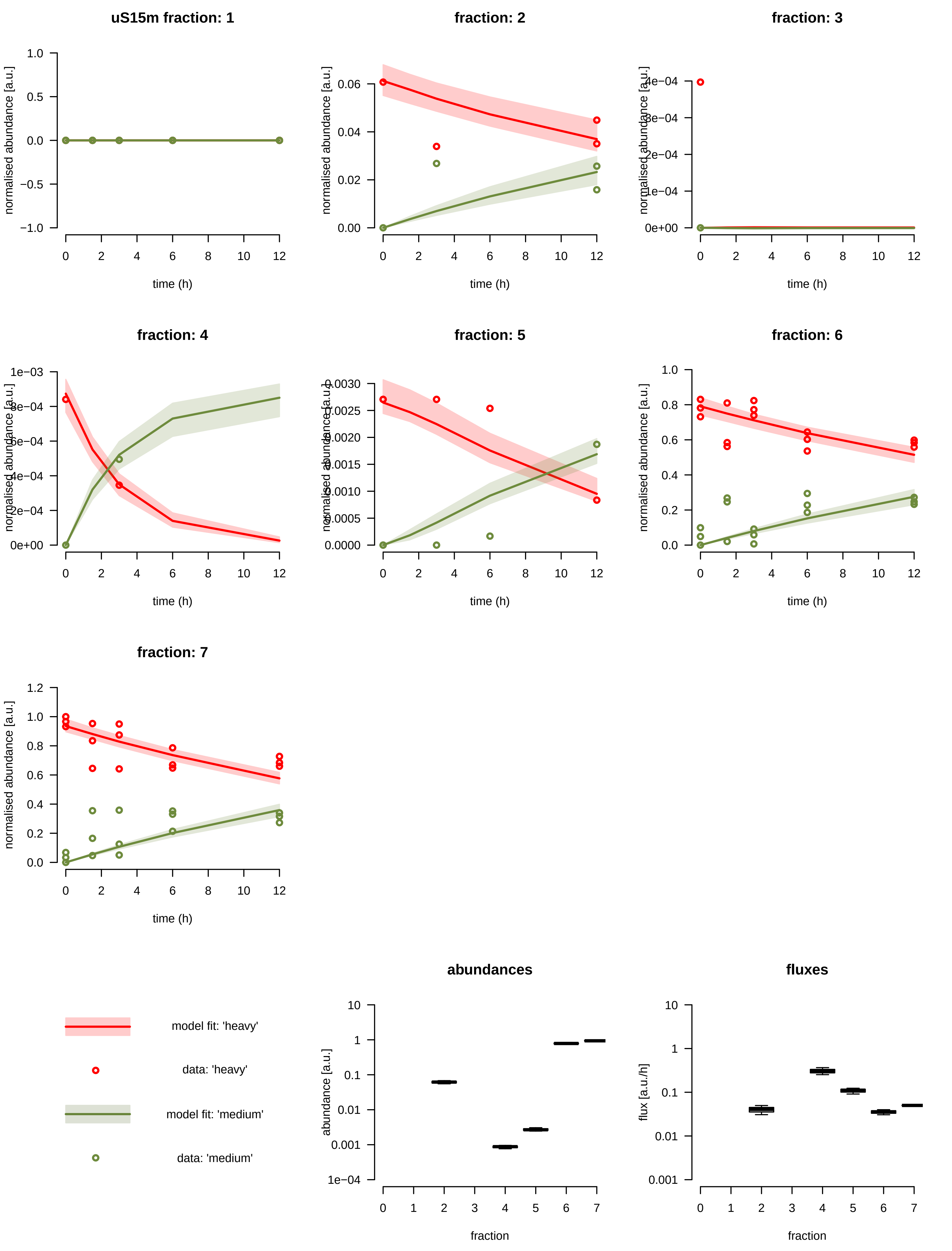
uS15m fraction: 1
fraction: 2
fraction: 3
fraction: 4
fraction: 5
fraction: 6
fraction: 7
abundances
fluxes
normalised abundance [a.u.]
time (h)
abundance [a.u.]
flux [a.u./h]
fraction
model fit: 'heavy'
data: 'heavy'
model fit: 'medium'
data: 'medium'

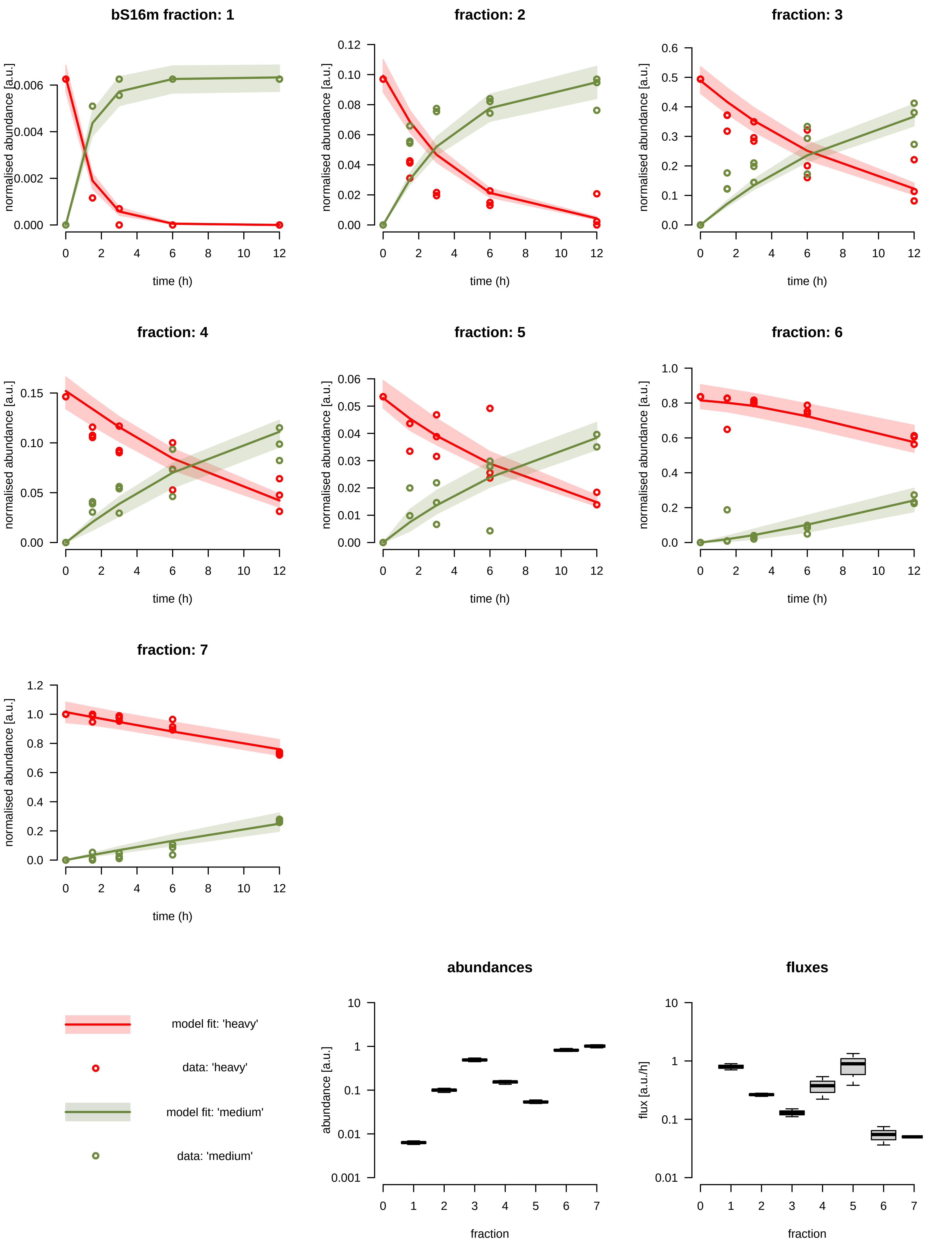

bS16m fraction: 1
fraction: 2
fraction: 3
fraction: 4
fraction: 5
fraction: 6
fraction: 7
normalised abundance [a.u.]
time (h)
model fit: 'heavy'
data: 'heavy'
model fit: 'medium'
data: 'medium'
abundances
abundance [a.u.]
fraction
fluxes
flux [a.u./h]
fraction

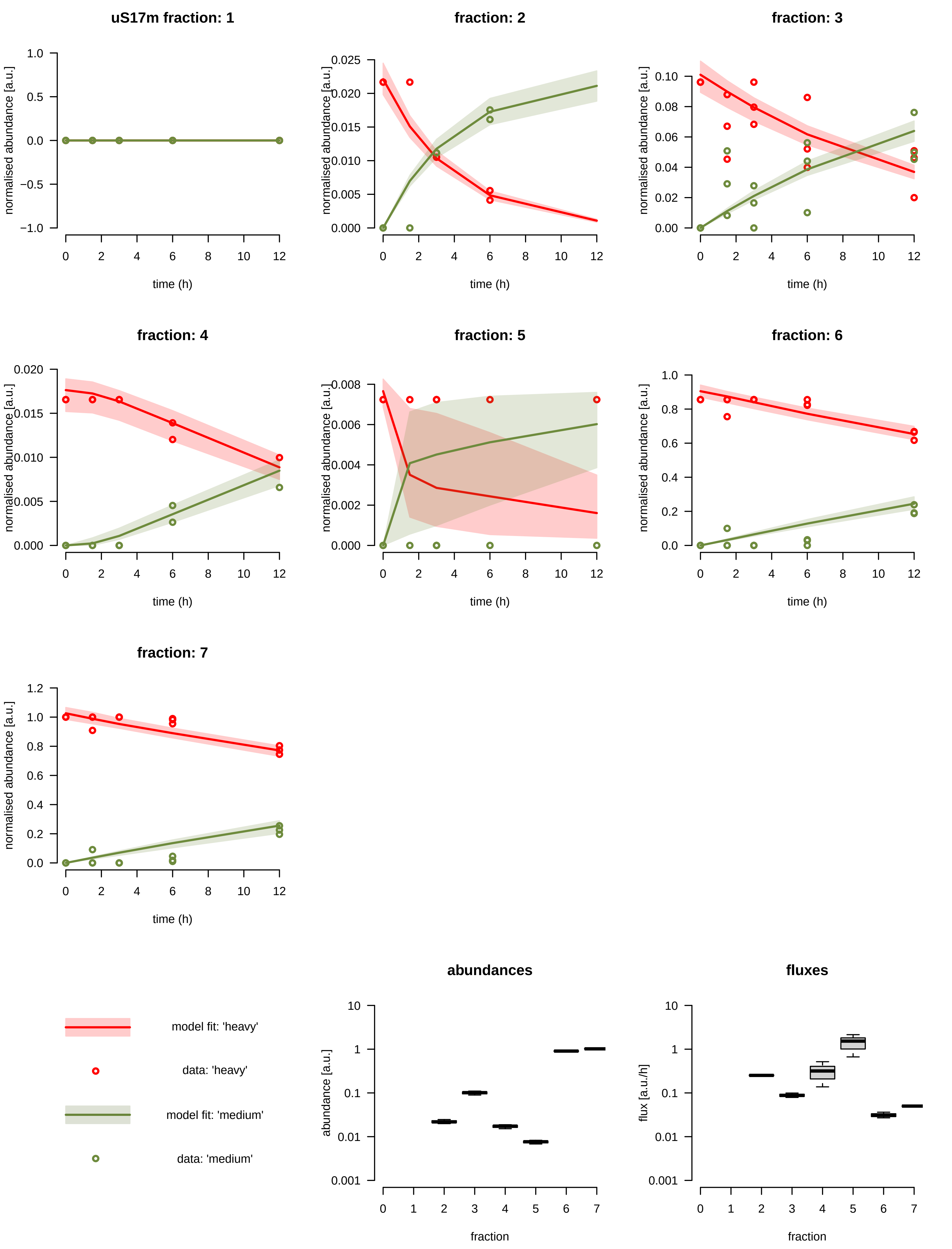
uS17m fraction: 1
fraction: 2
fraction: 3
fraction: 4
fraction: 5
fraction: 6
fraction: 7
normalised abundance [a.u.]
time (h)
model fit: 'heavy'
data: 'heavy'
model fit: 'medium'
data: 'medium'
abundances
abundance [a.u.]
fraction
fluxes
flux [a.u./h]

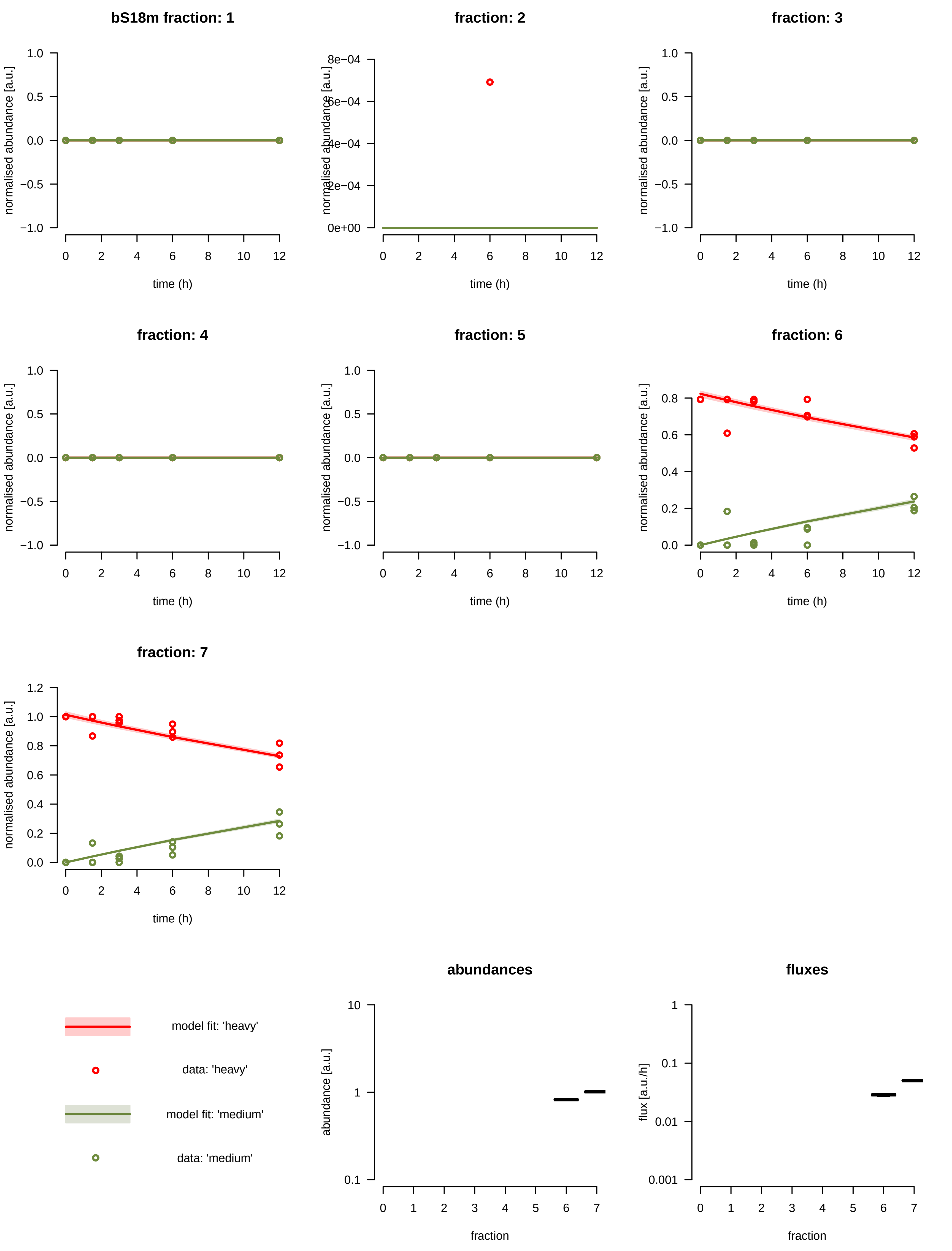
bS18m fraction: 1
fraction: 2
fraction: 3
fraction: 4
fraction: 5
fraction: 6
fraction: 7
normalised abundance [a.u.]
time (h)
model fit: 'heavy'
data: 'heavy'
model fit: 'medium'
data: 'medium'
abundances
abundance [a.u.]
fluxes
flux [a.u./h]
fraction

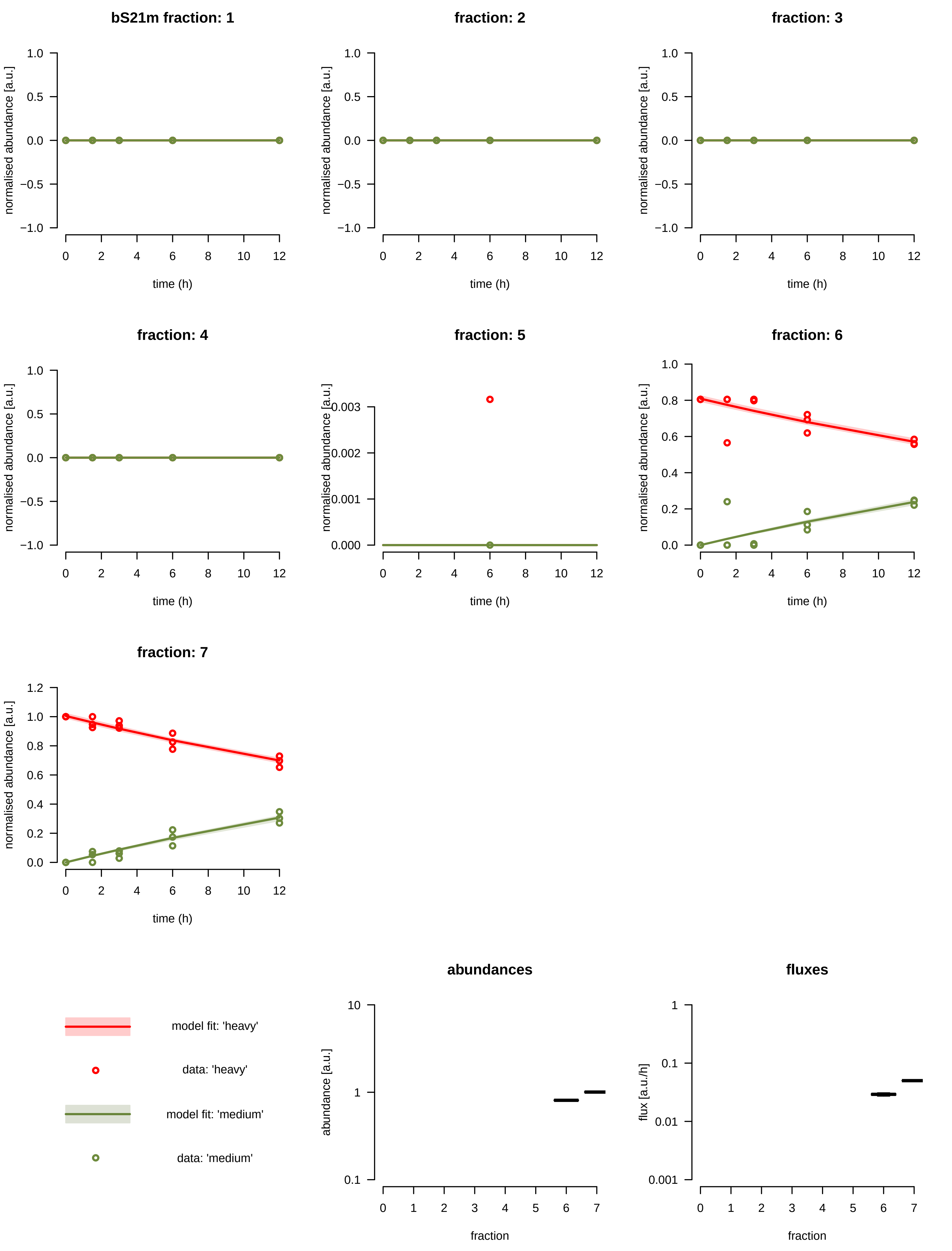
bS21m fraction: 1
fraction: 2
fraction: 3
fraction: 4
fraction: 5
fraction: 6
fraction: 7
normalised abundance [a.u.]
time (h)
model fit: 'heavy'
data: 'heavy'
model fit: 'medium'
data: 'medium'
abundances
abundance [a.u.]
fraction
fluxes
flux [a.u./h]
fraction

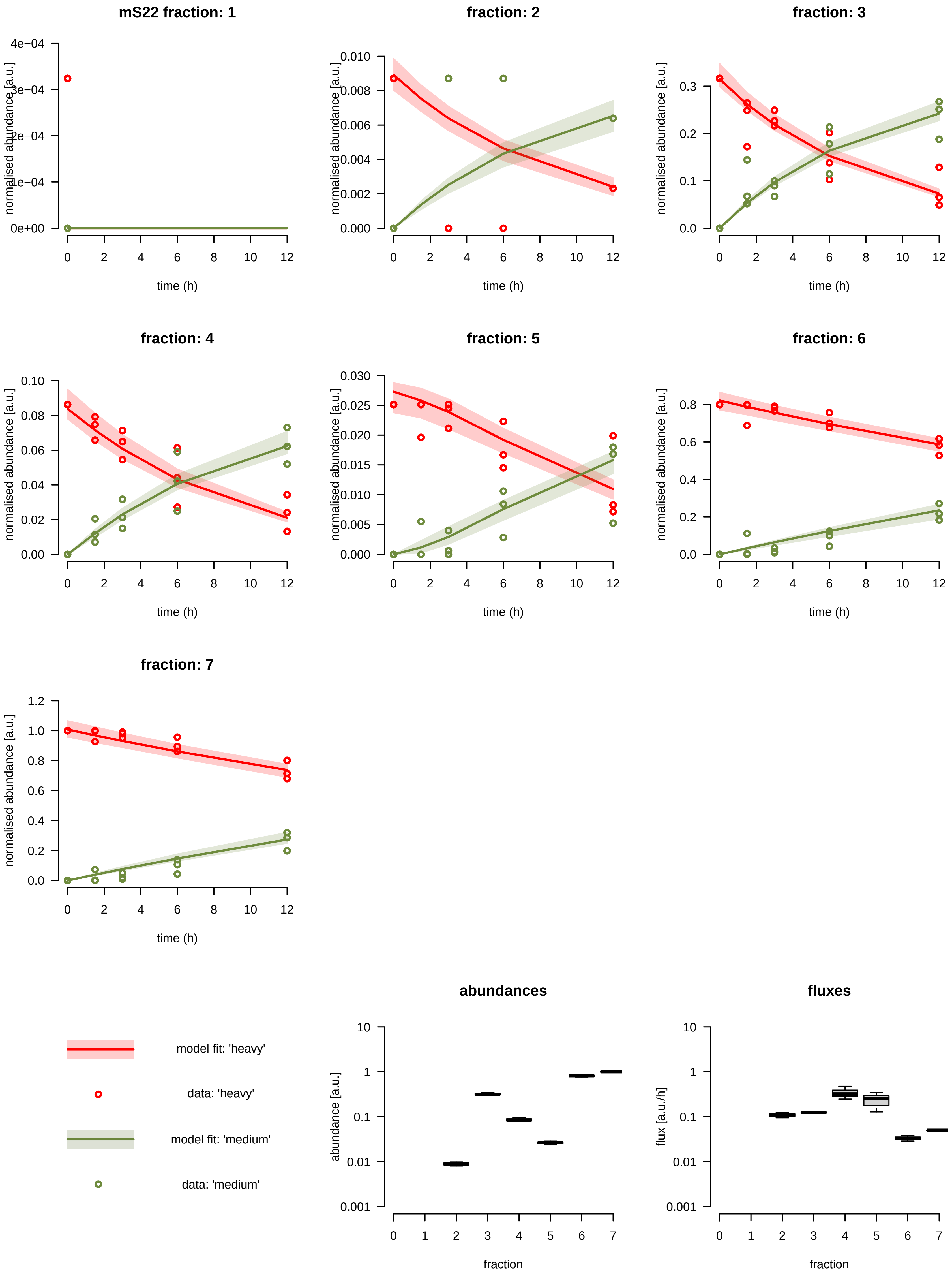
mS22 fraction: 1
fraction: 2
fraction: 3
fraction: 4
fraction: 5
fraction: 6
fraction: 7
normalised abundance [a.u.]
time (h)
model fit: 'heavy'
data: 'heavy'
model fit: 'medium'
data: 'medium'
abundances
abundance [a.u.]
fraction
fluxes
flux [a.u./h]
fraction

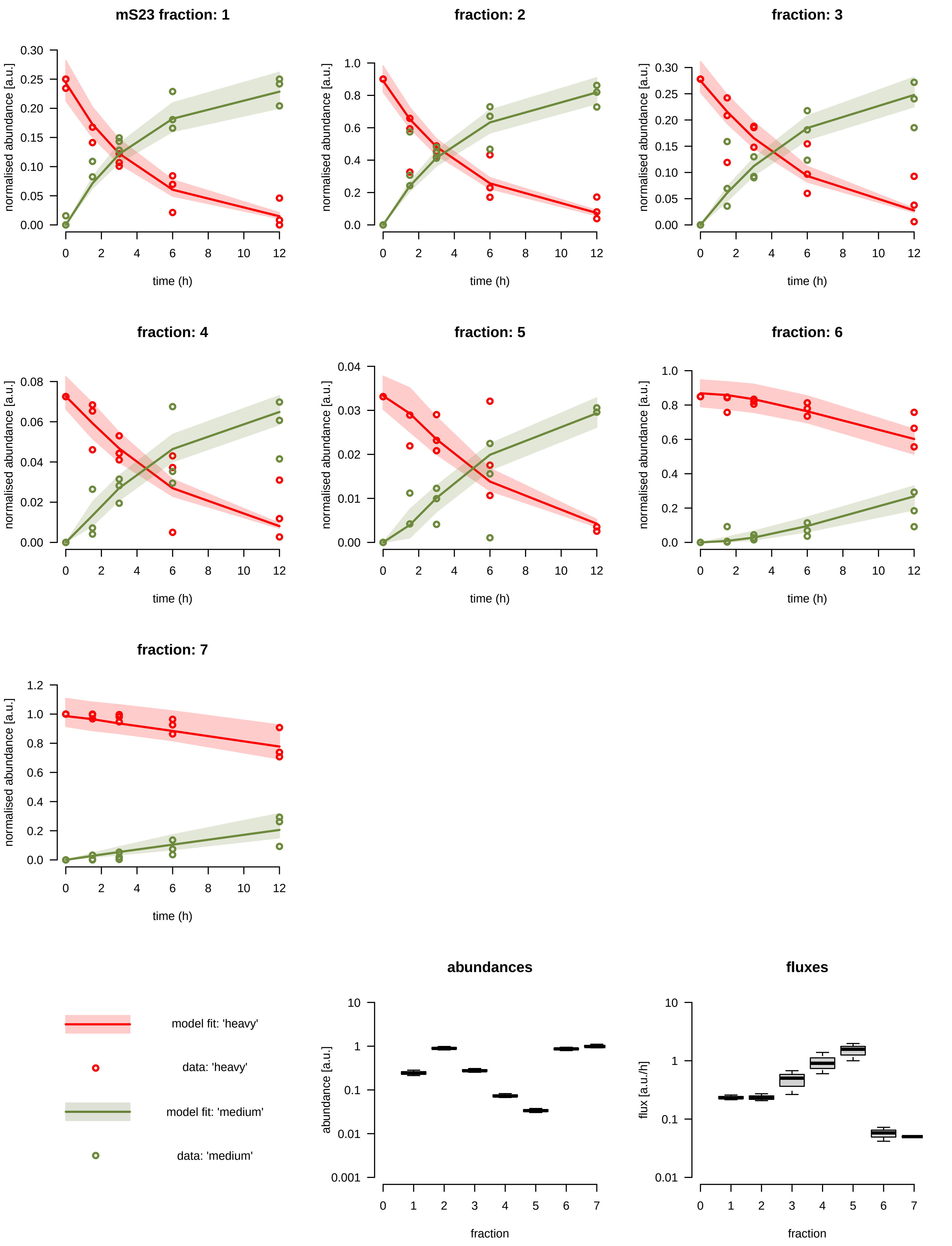
mS23 fraction: 1
fraction: 2
fraction: 3
fraction: 4
fraction: 5
fraction: 6
fraction: 7
normalised abundance [a.u.]
time (h)
model fit: 'heavy'
data: 'heavy'
model fit: 'medium'
data: 'medium'
abundances
abundance [a.u.]
fraction
fluxes
flux [a.u./h]
fraction

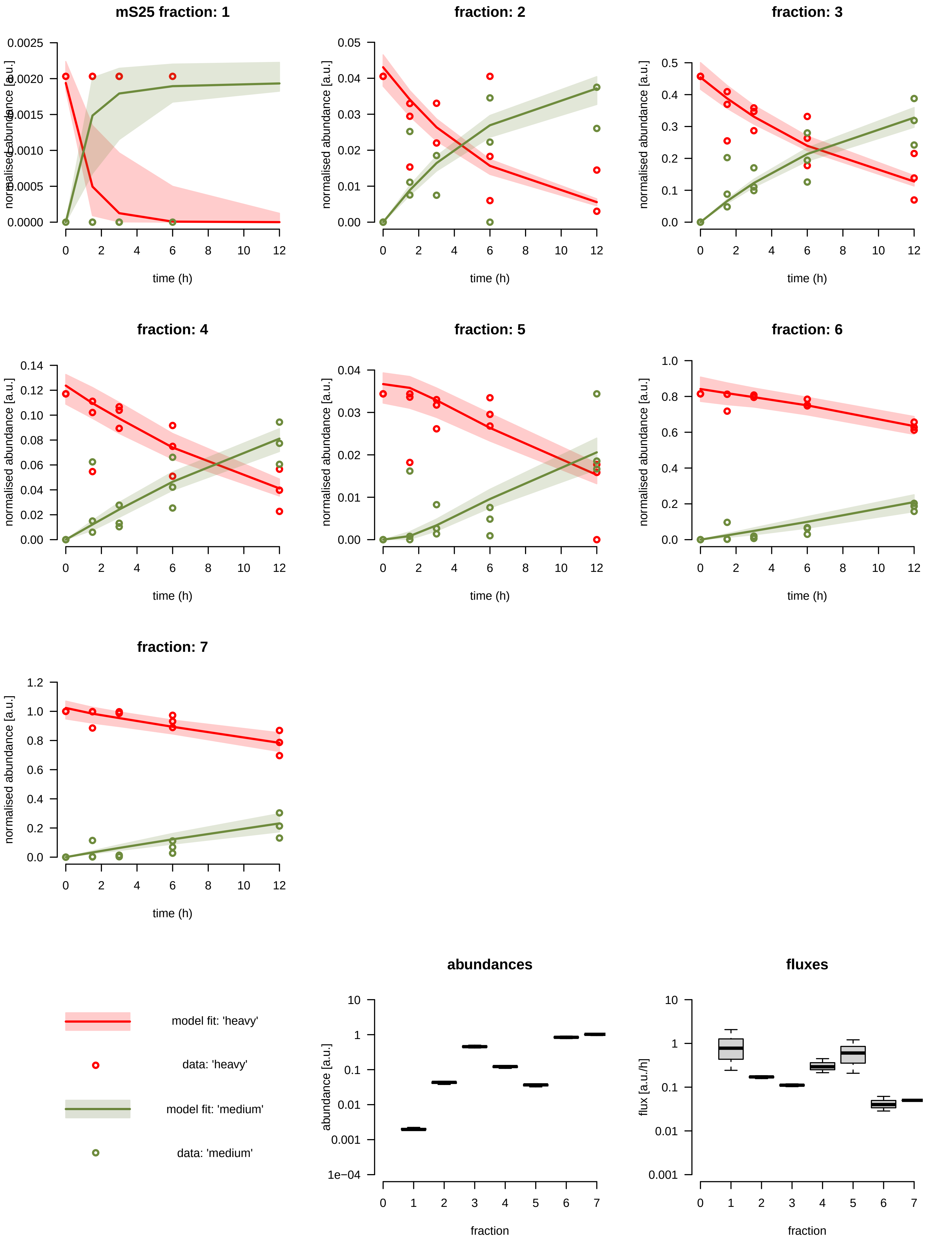
mS25 fraction: 1
fraction: 2
fraction: 3
fraction: 4
fraction: 5
fraction: 6
fraction: 7
normalised abundance [a.u.]
time (h)
model fit: 'heavy'
data: 'heavy'
model fit: 'medium'
data: 'medium'
abundances
abundance [a.u.]
fraction
fluxes
flux [a.u./h]
fraction

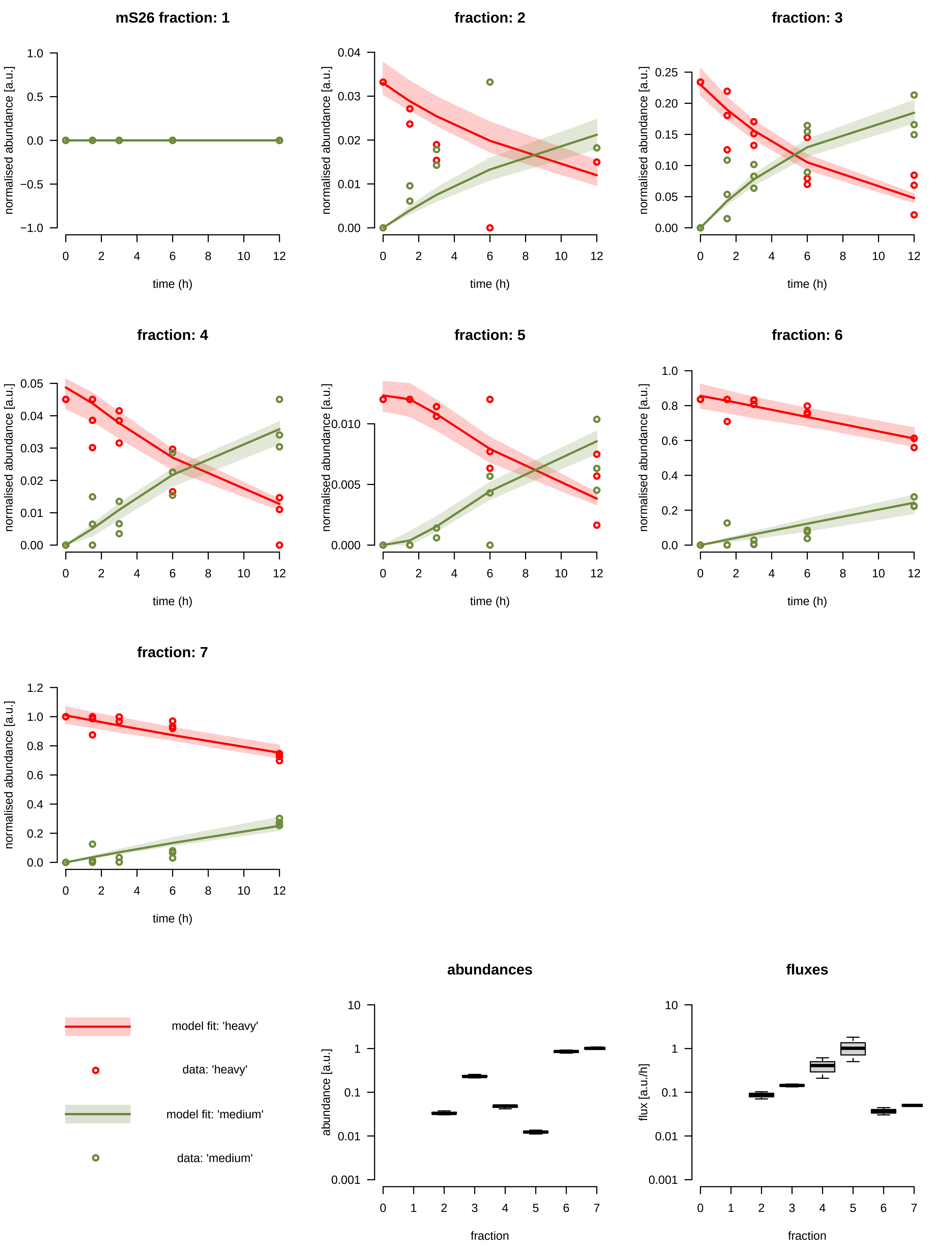

mS26 fraction: 1
fraction: 2
fraction: 3
fraction: 4
fraction: 5
fraction: 6
fraction: 7
normalised abundance [a.u.]
time (h)
model fit: 'heavy'
data: 'heavy'
model fit: 'medium'
data: 'medium'
abundances
abundance [a.u.]
fraction
fluxes
flux [a.u./h]
fraction

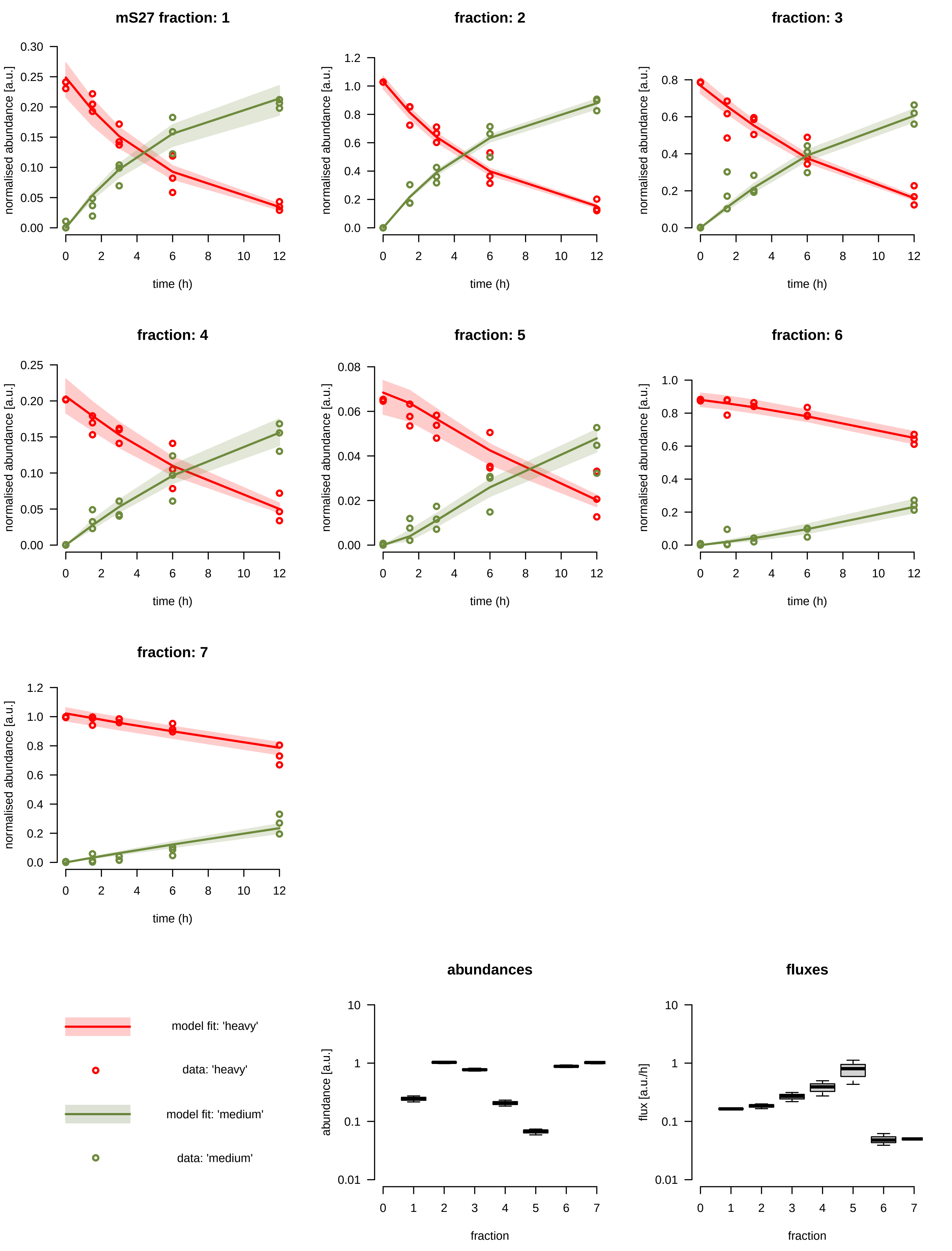
mS27 fraction: 1
fraction: 2
fraction: 3
fraction: 4
fraction: 5
fraction: 6
fraction: 7
normalised abundance [a.u.]
time (h)
model fit: 'heavy'
data: 'heavy'
model fit: 'medium'
data: 'medium'
abundances
abundance [a.u.]
fluxes
flux [a.u./h]
fraction

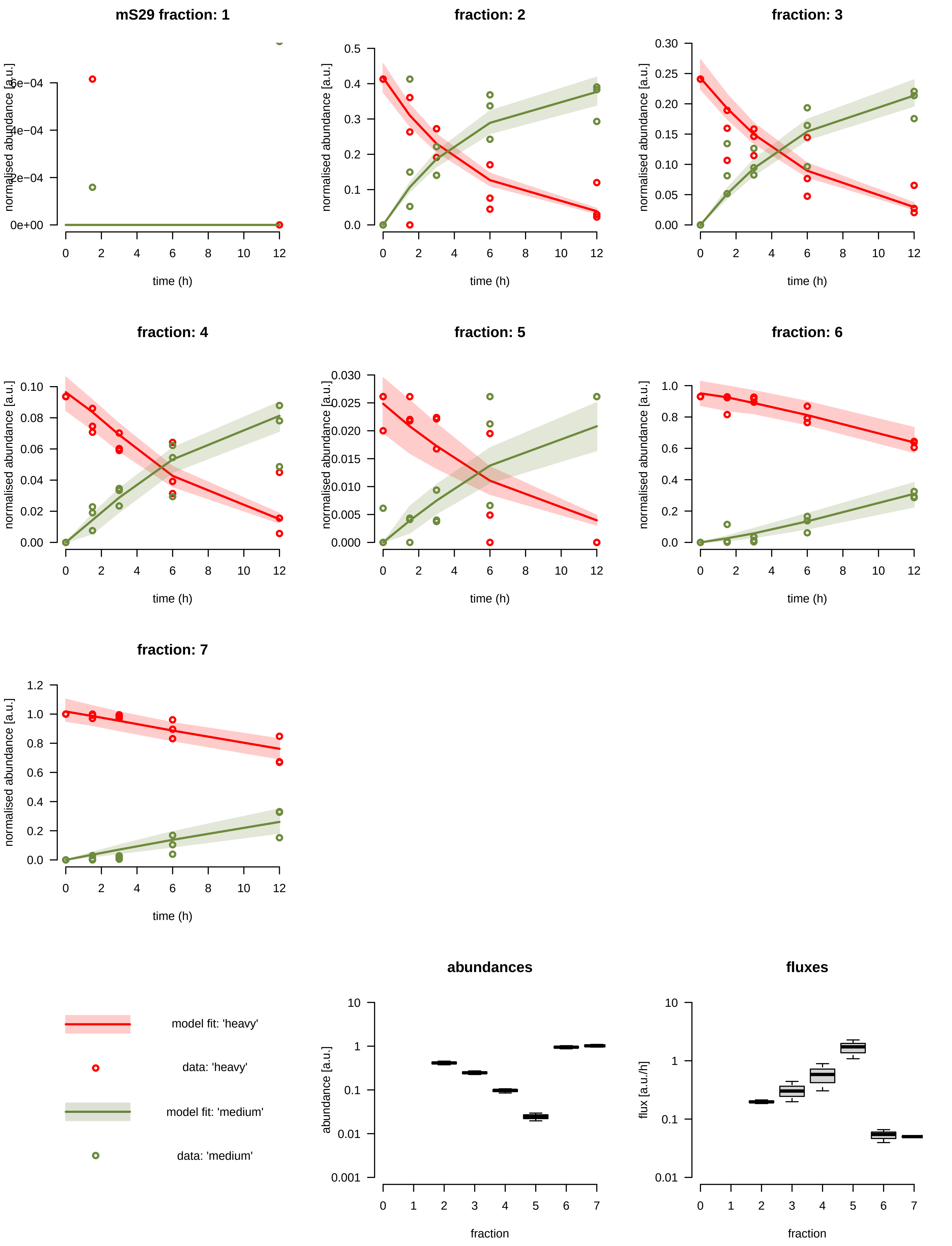
mS29 fraction: 1
fraction: 2
fraction: 3
fraction: 4
fraction: 5
fraction: 6
fraction: 7
normalised abundance [a.u.]
time (h)
model fit: 'heavy'
data: 'heavy'
model fit: 'medium'
data: 'medium'
abundances
abundance [a.u.]
fraction
fluxes
flux [a.u./h]
fraction

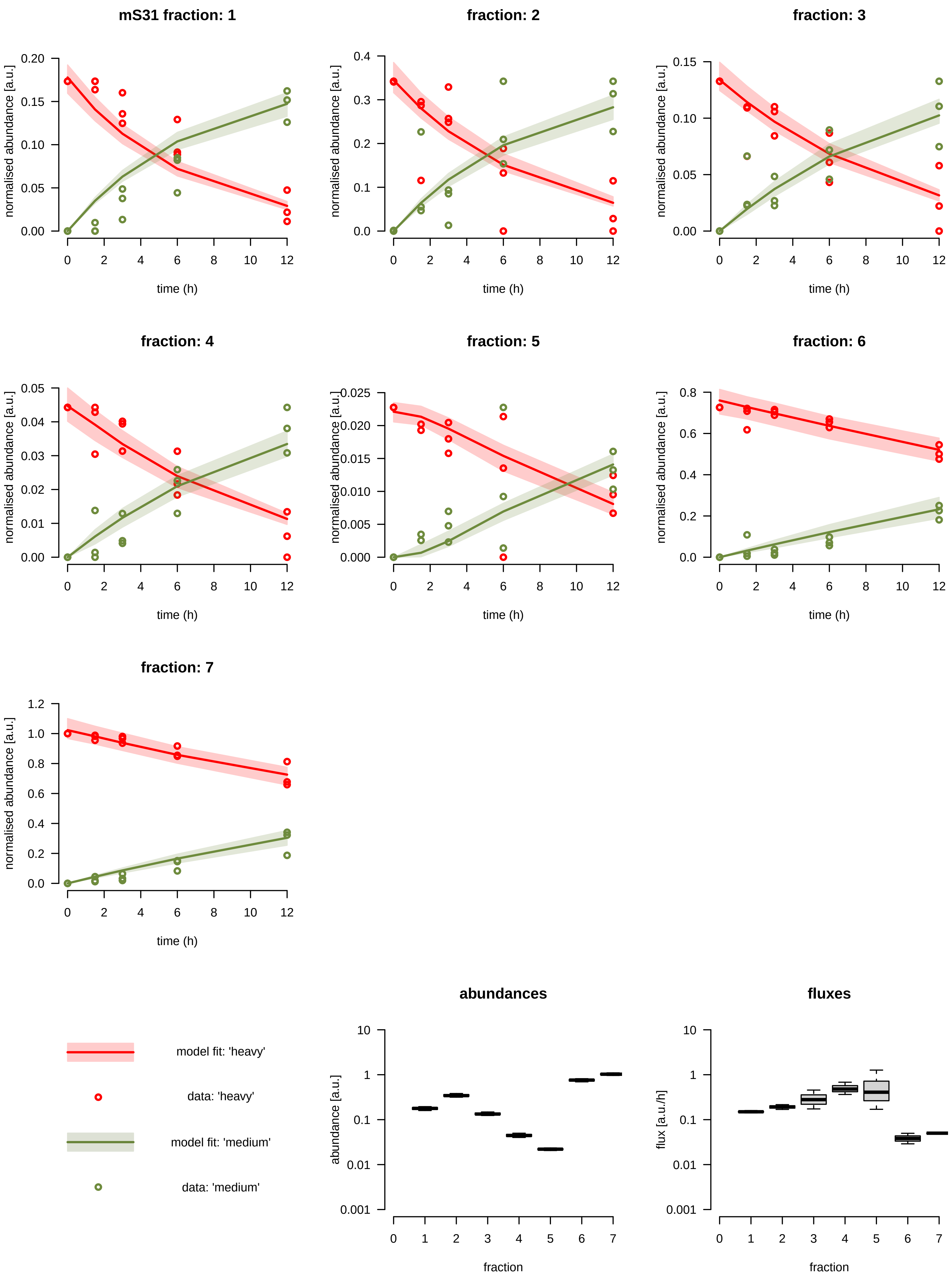
mS31 fraction: 1
fraction: 2
fraction: 3
fraction: 4
fraction: 5
fraction: 6
fraction: 7
normalised abundance [a.u.]
time (h)
model fit: 'heavy'
data: 'heavy'
model fit: 'medium'
data: 'medium'
abundances
abundance [a.u.]
fraction
fluxes
flux [a.u./h]
fraction

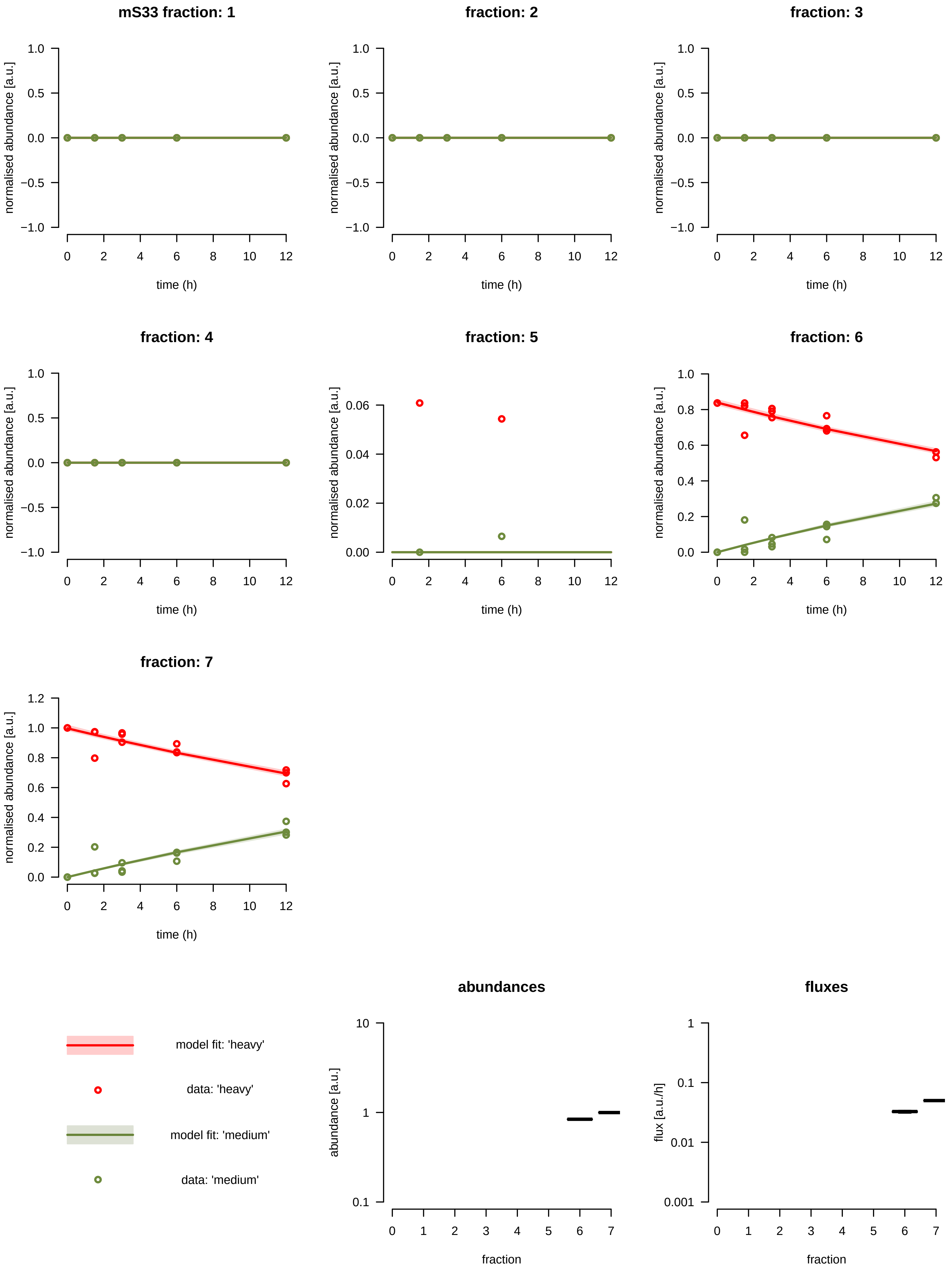
mS33 fraction: 1
fraction: 2
fraction: 3
fraction: 4
fraction: 5
fraction: 6
fraction: 7
normalised abundance [a.u.]
time (h)
model fit: 'heavy'
data: 'heavy'
model fit: 'medium'
data: 'medium'
abundances
abundance [a.u.]
fraction
fluxes
flux [a.u./h]
fraction

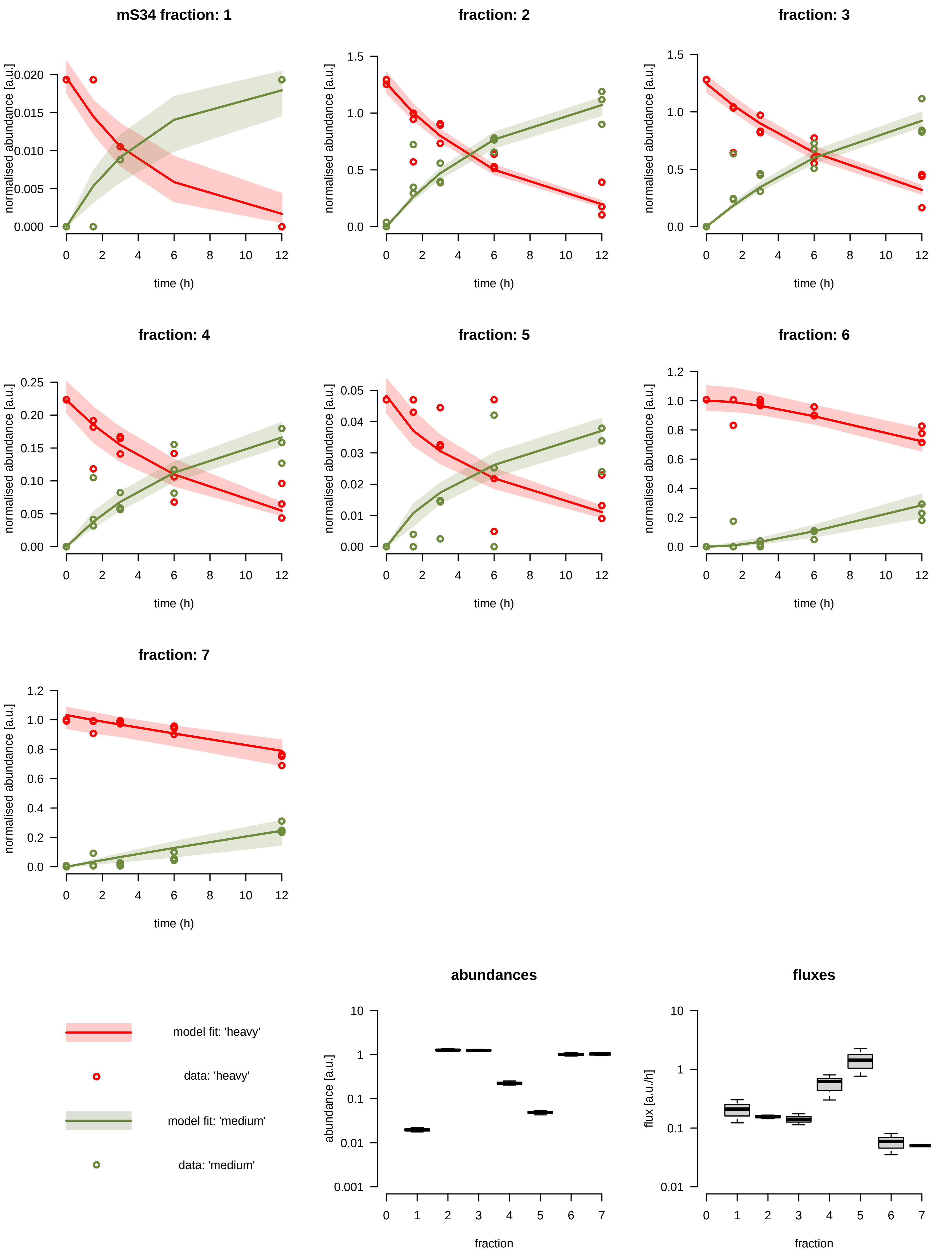
mS34 fraction: 1
fraction: 2
fraction: 3
fraction: 4
fraction: 5
fraction: 6
fraction: 7
normalised abundance [a.u.]
time (h)
model fit: 'heavy'
data: 'heavy'
model fit: 'medium'
data: 'medium'
abundances
abundance [a.u.]
fraction
fluxes
flux [a.u./h]
fraction

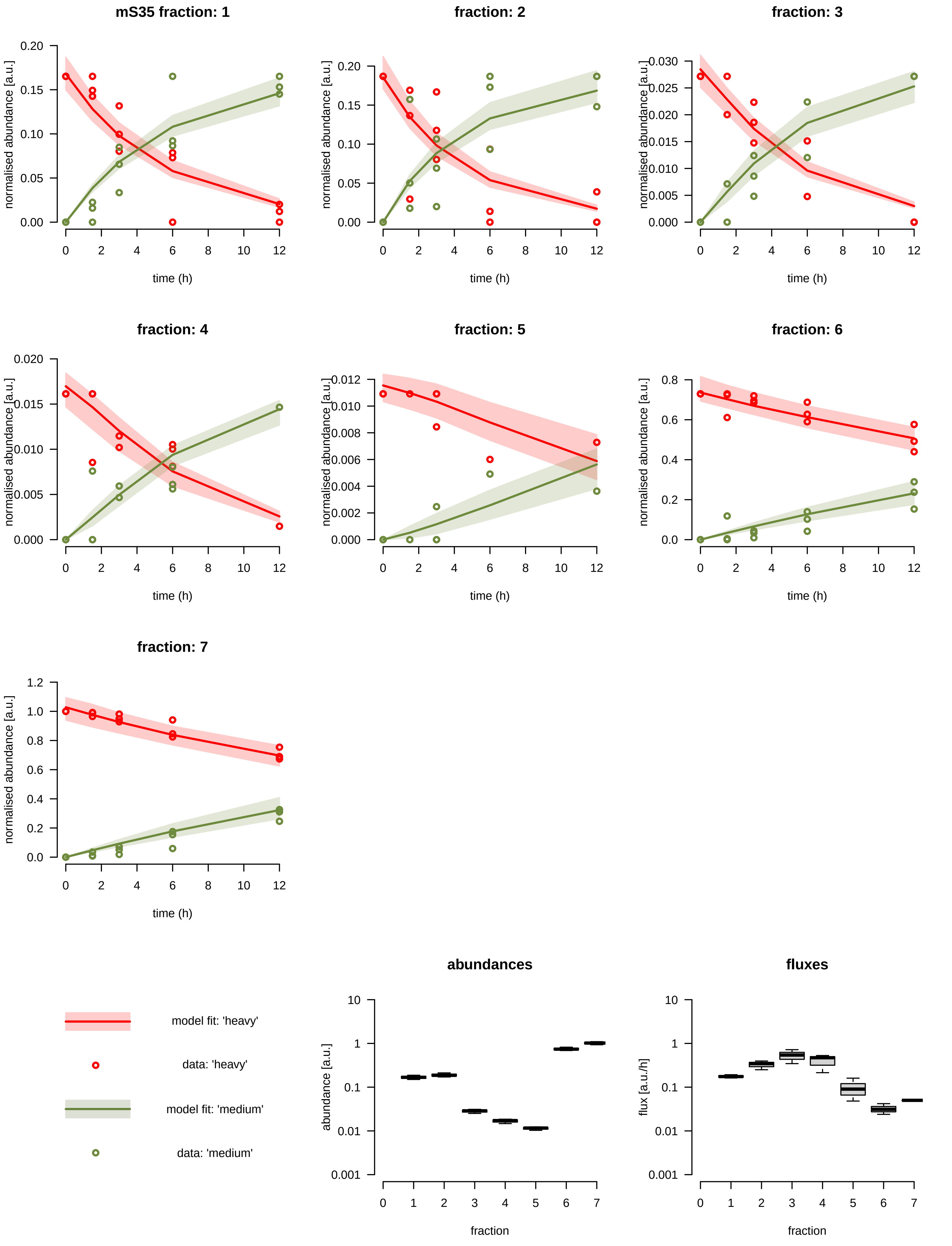

mS35 fraction: 1
fraction: 2
fraction: 3
fraction: 4
fraction: 5
fraction: 6
fraction: 7
normalised abundance [a.u.]
time (h)
model fit: 'heavy'
data: 'heavy'
model fit: 'medium'
data: 'medium'
abundances
abundance [a.u.]
fraction
fluxes
flux [a.u./h]

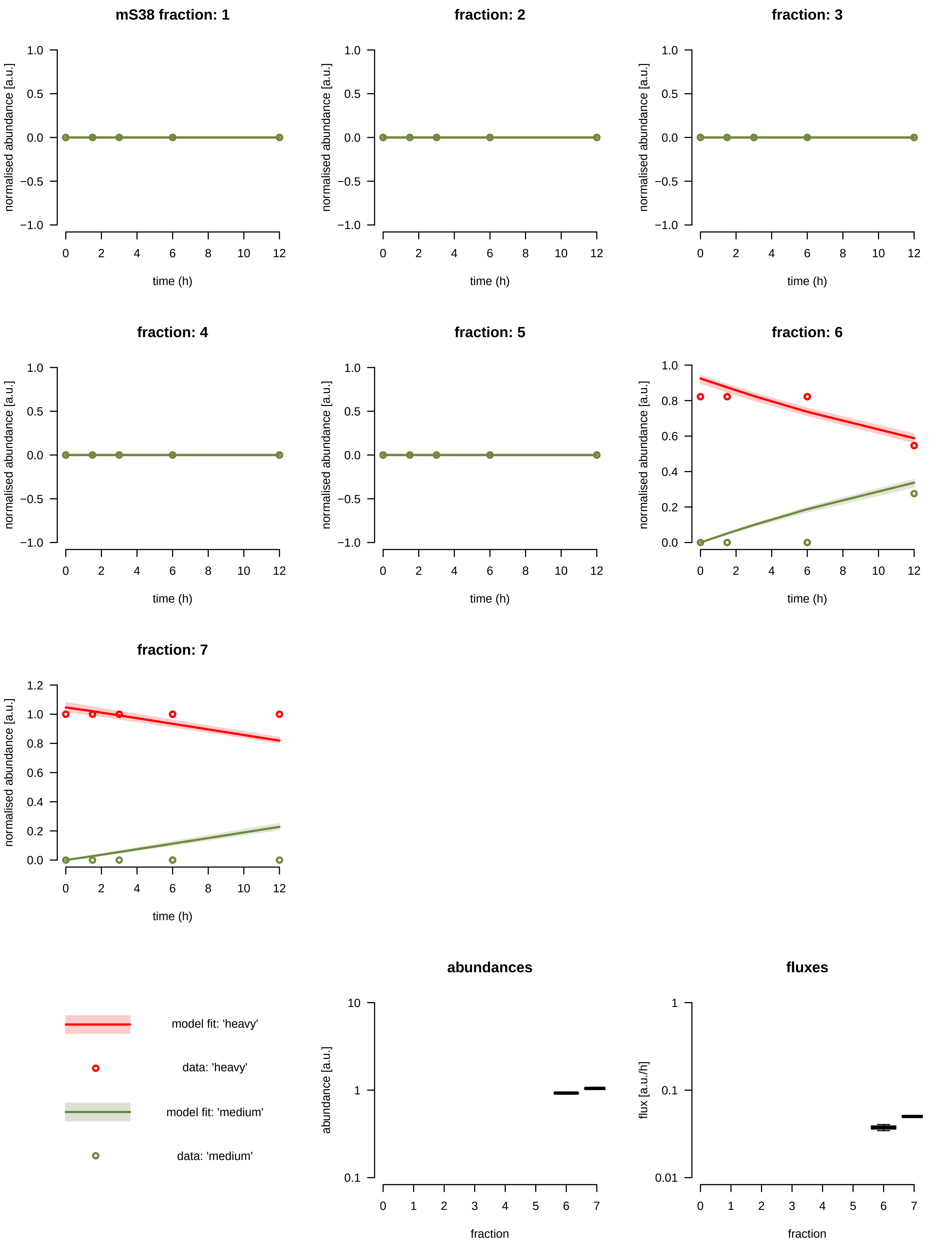
mS38 fraction: 1
fraction: 2
fraction: 3
fraction: 4
fraction: 5
fraction: 6
fraction: 7
normalised abundance [a.u.]
time (h)
model fit: 'heavy'
data: 'heavy'
model fit: 'medium'
data: 'medium'
abundances
abundance [a.u.]
fraction
fluxes
flux [a.u./h]
fraction

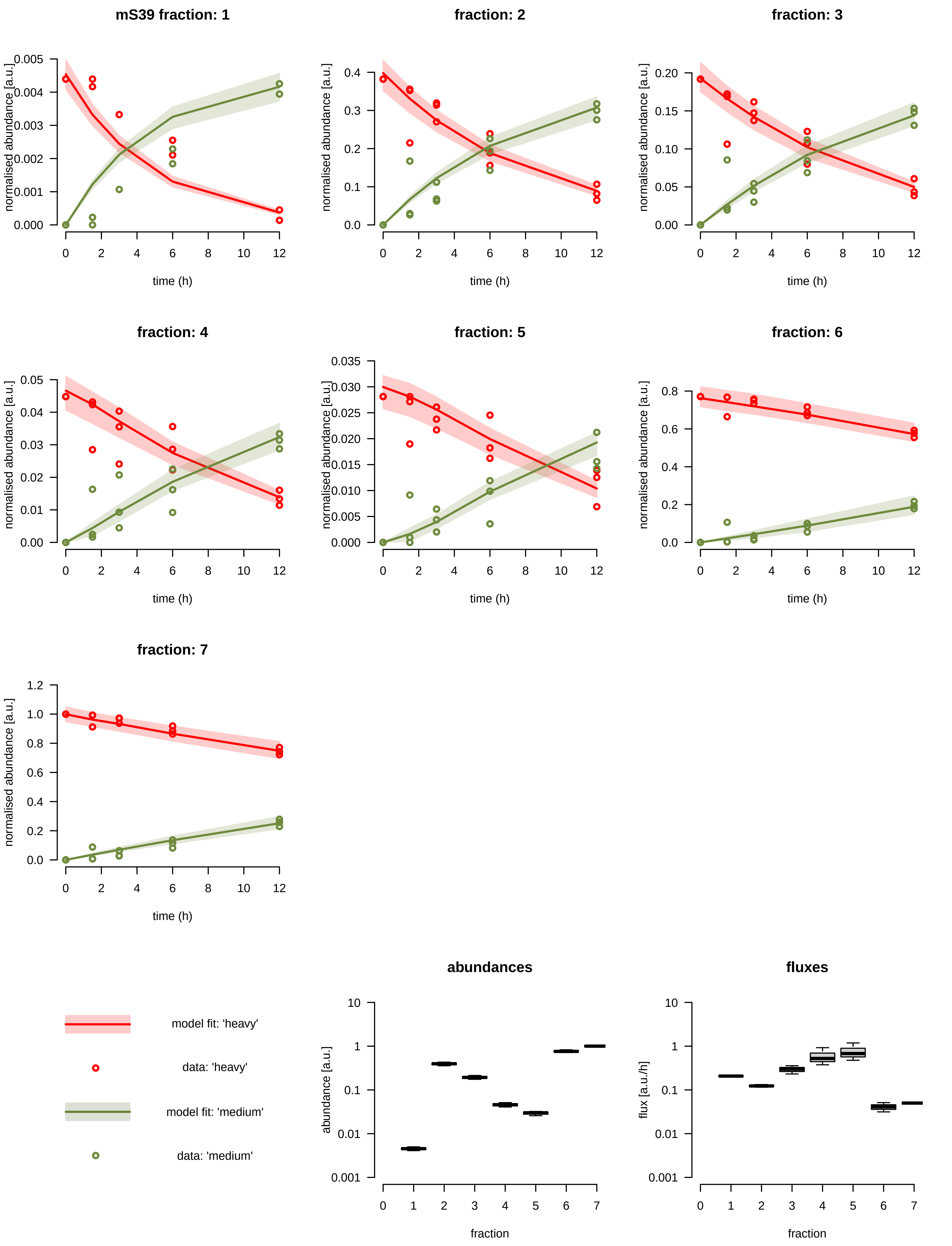
mS39 fraction: 1
fraction: 2
fraction: 3
fraction: 4
fraction: 5
fraction: 6
fraction: 7
normalised abundance [a.u.]
time (h)
model fit: 'heavy'
data: 'heavy'
model fit: 'medium'
data: 'medium'
abundances
abundance [a.u.]
fraction
fluxes
flux [a.u./h]
fraction

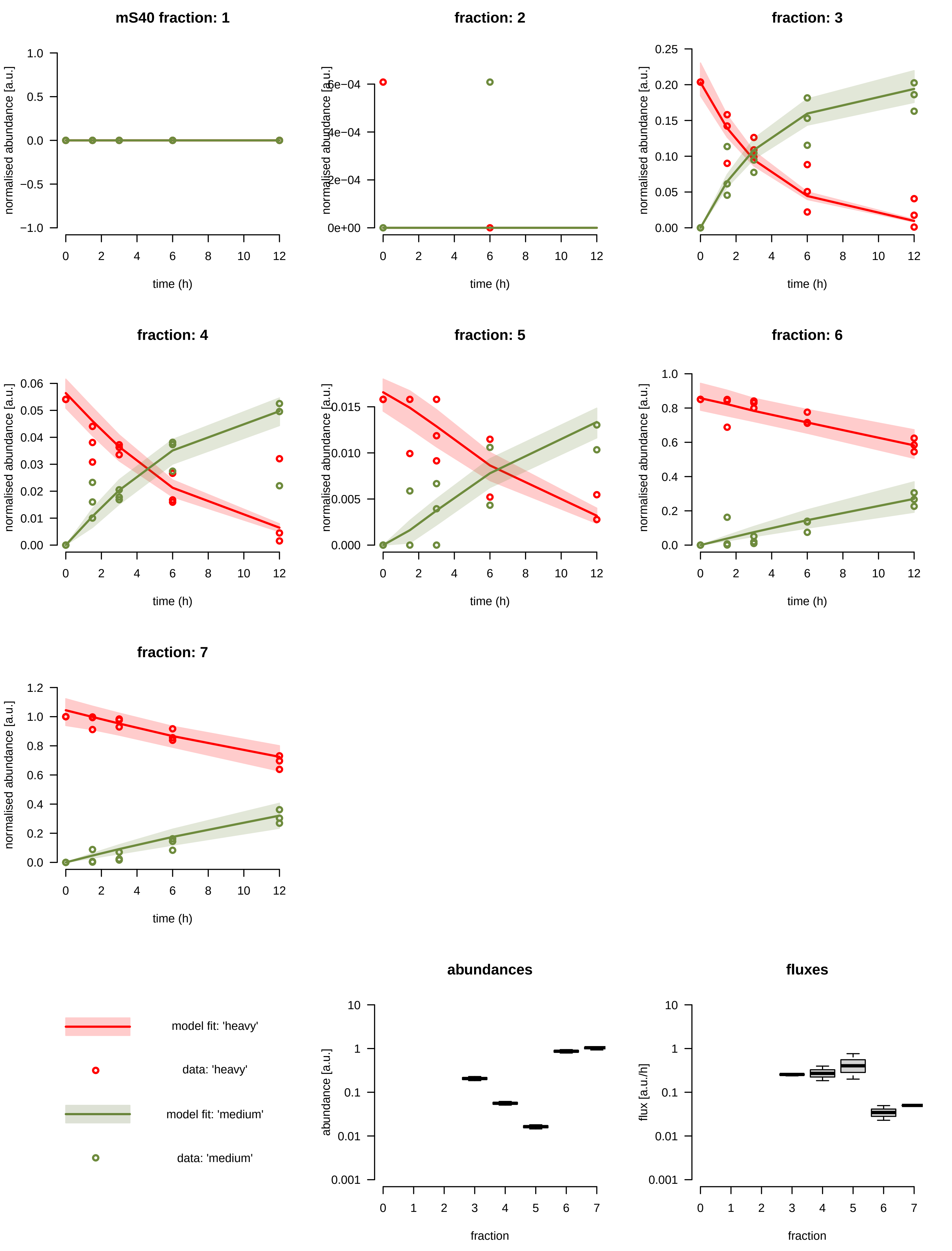
mS40 fraction: 1
fraction: 2
fraction: 3
fraction: 4
fraction: 5
fraction: 6
fraction: 7
normalised abundance [a.u.]
time (h)
model fit: 'heavy'
data: 'heavy'
model fit: 'medium'
data: 'medium'
abundances
abundance [a.u.]
fraction
fluxes
flux [a.u./h]
fraction
