# Supplementary material for: A roadmap for ribosome assembly in human mitochondria
Source: Nat Struct Mol Biol. 2024 Jul 11;31(12):1898–908. doi: 10.1038/s41594-024-01356-w (PMC11638073; doi:10.1038/s41594-024-01356-w)
Supplement: Supplementary file 10 — Inference of steady-state abundances and fluxes for mtLSU MRPs. Inference of fluxes for all MRPs of the mtLSU. Shown are normalized abundances derived from experimental data (dots) and model fits (median, bold line; 5th and 95th percentiles, thin lines) for sucrose gradient fractions 1–9. Additionally, measured steady-state abundances and inferred fluxes are shown across sucrose gradient fractions. [file 41594_2024_1356_MOESM10_ESM.pdf]

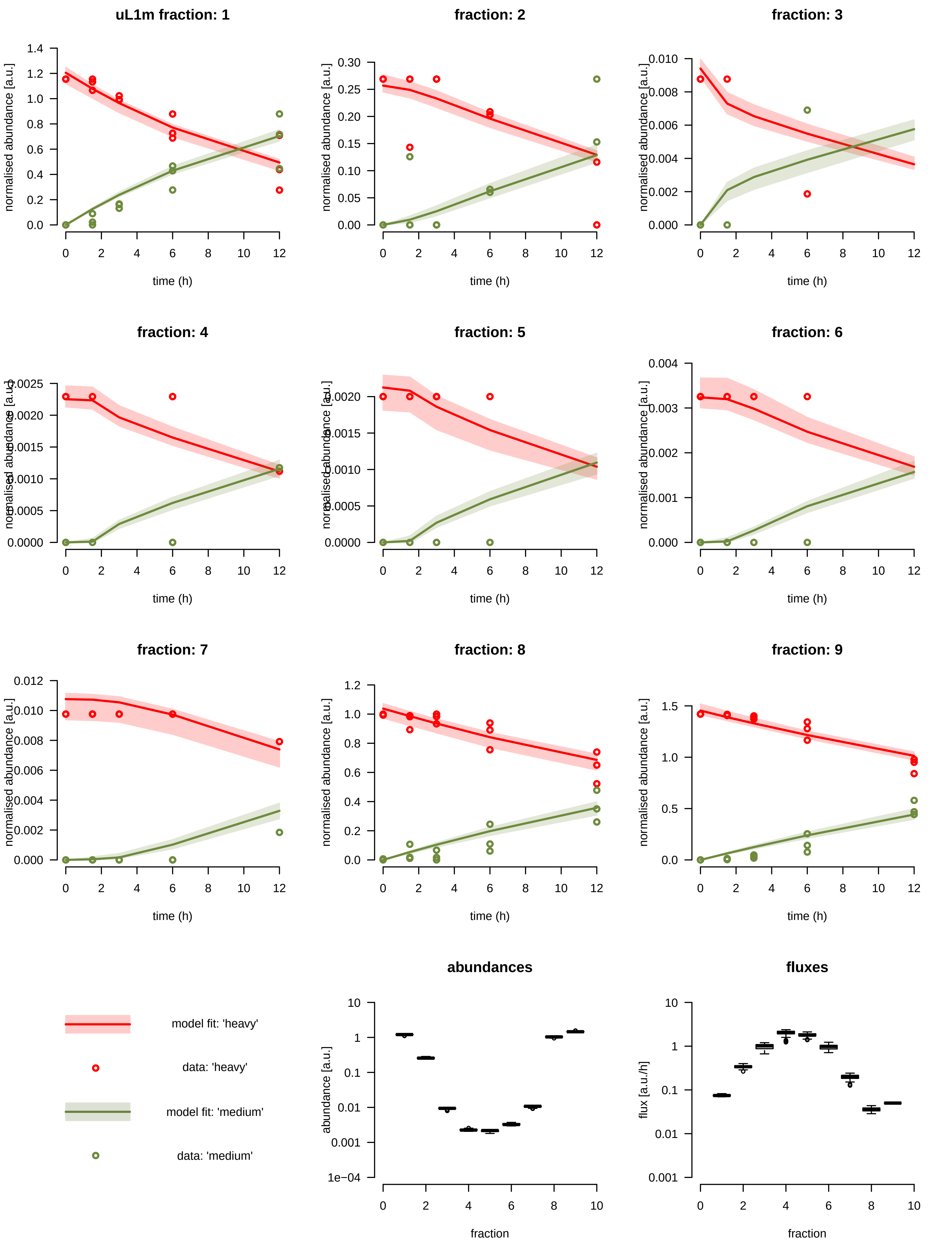
uL1m fraction: 1
fraction: 2
fraction: 3
fraction: 4
fraction: 5
fraction: 6
fraction: 7
fraction: 8
fraction: 9
normalised abundance [a.u.]
time (h)
model fit: 'heavy'
data: 'heavy'
model fit: 'medium'
data: 'medium'
abundances
abundance [a.u.]
fraction
fluxes
flux [a.u./h]
fraction

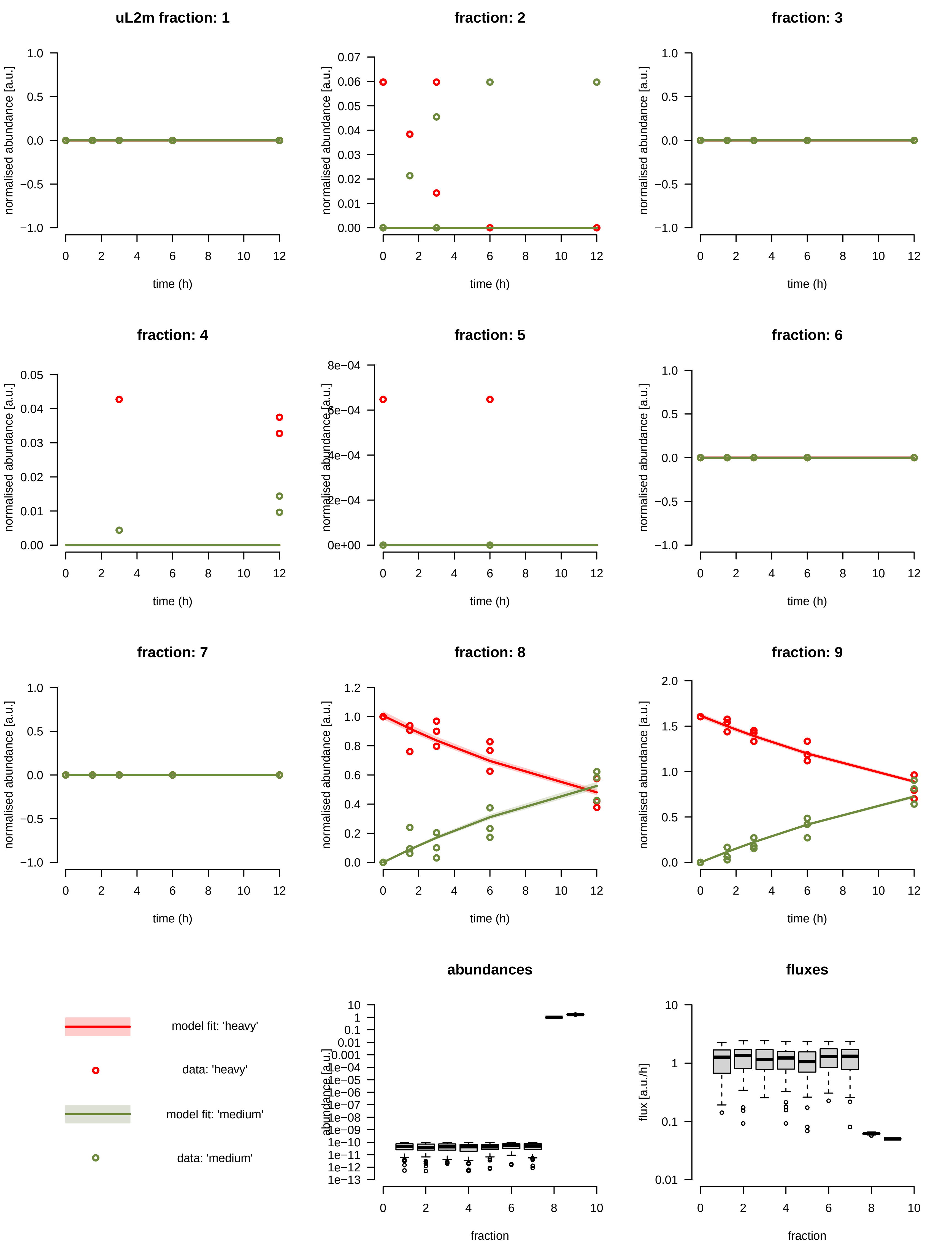
uL2m fraction: 1
fraction: 2
fraction: 3
fraction: 4
fraction: 5
fraction: 6
fraction: 7
fraction: 8
fraction: 9
normalised abundance [a.u.]
time (h)
model fit: 'heavy'
data: 'heavy'
model fit: 'medium'
data: 'medium'
abundances
abundance [a.u.]
fraction
fluxes
flux [a.u./h]
fraction

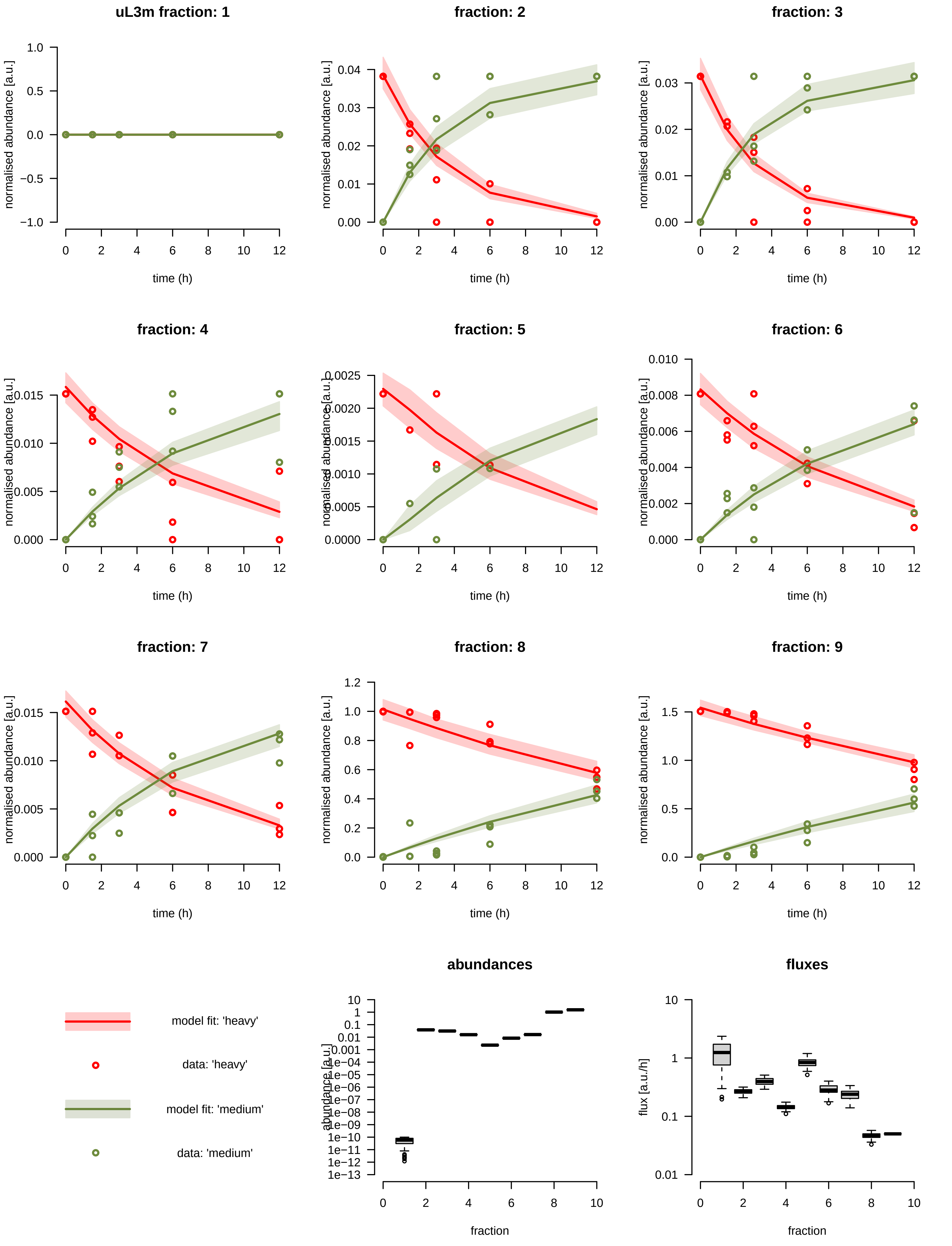
uL3m fraction: 1
fraction: 2
fraction: 3
fraction: 4
fraction: 5
fraction: 6
fraction: 7
fraction: 8
fraction: 9
abundances
fluxes
normalised abundance [a.u.]
time (h)
abundance [a.u.]
flux [a.u./h]
fraction
model fit: 'heavy'
data: 'heavy'
model fit: 'medium'
data: 'medium'

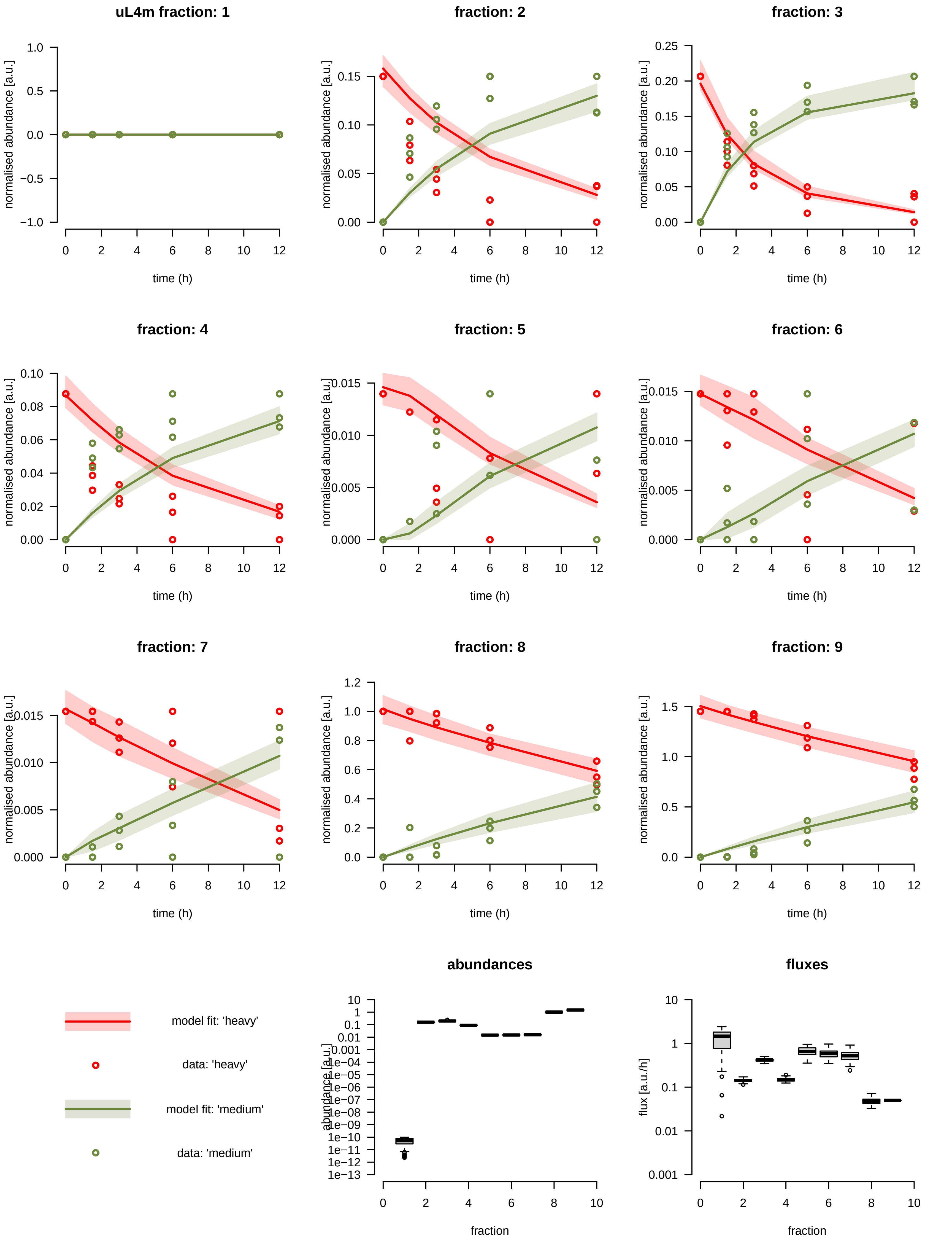

uL4m fraction: 1
fraction: 2
fraction: 3
fraction: 4
fraction: 5
fraction: 6
fraction: 7
fraction: 8
fraction: 9
normalised abundance [a.u.]
time (h)
model fit: 'heavy'
data: 'heavy'
model fit: 'medium'
data: 'medium'
abundances
abundance [a.u.]
fraction
fluxes
flux [a.u./h]
fraction

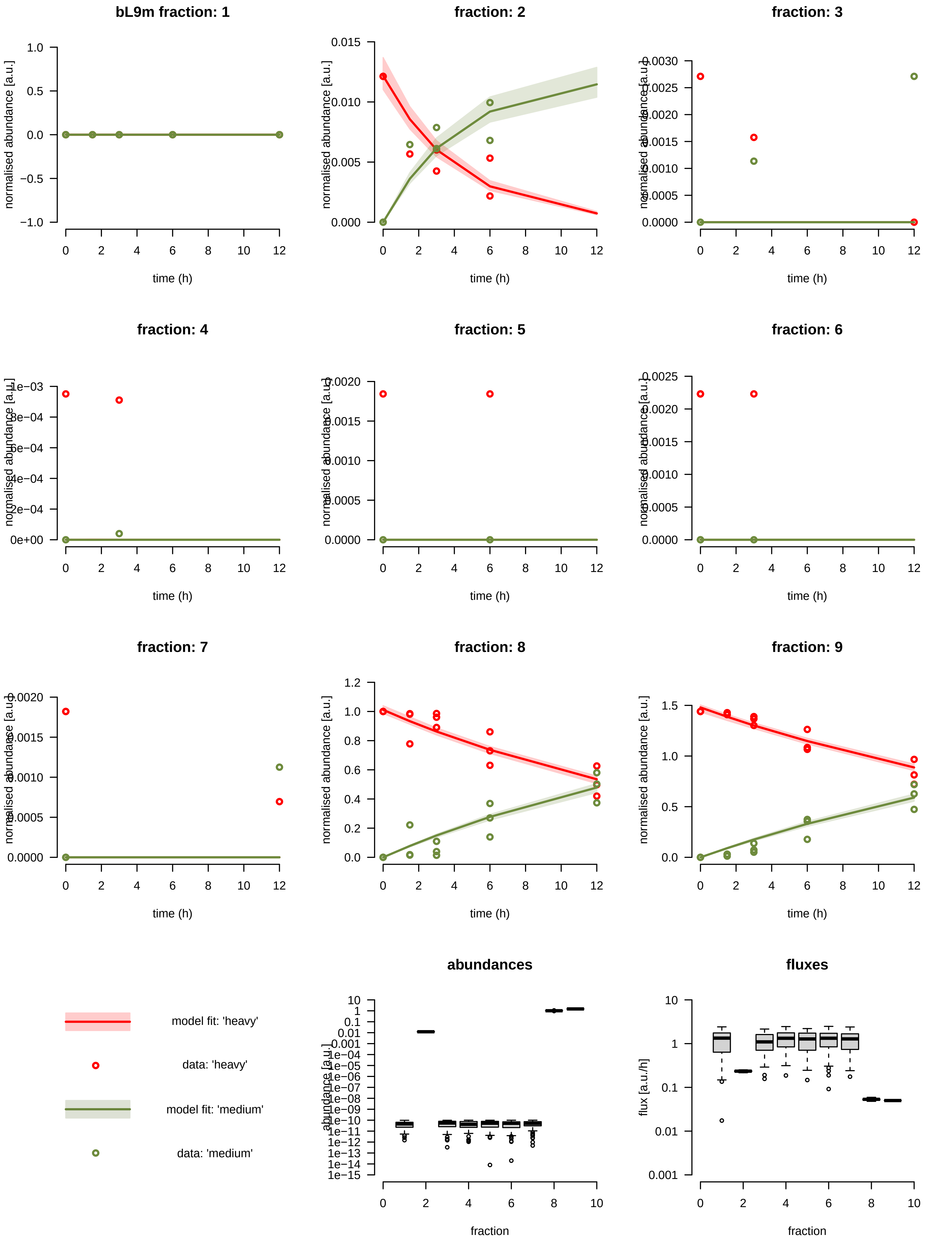

bL9m fraction: 1
fraction: 2
fraction: 3
fraction: 4
fraction: 5
fraction: 6
fraction: 7
fraction: 8
fraction: 9
normalised abundance [a.u.]
time (h)
model fit: 'heavy'
data: 'heavy'
model fit: 'medium'
data: 'medium'
abundances
abundance [a.u.]
fraction
fluxes
flux [a.u./h]
fraction

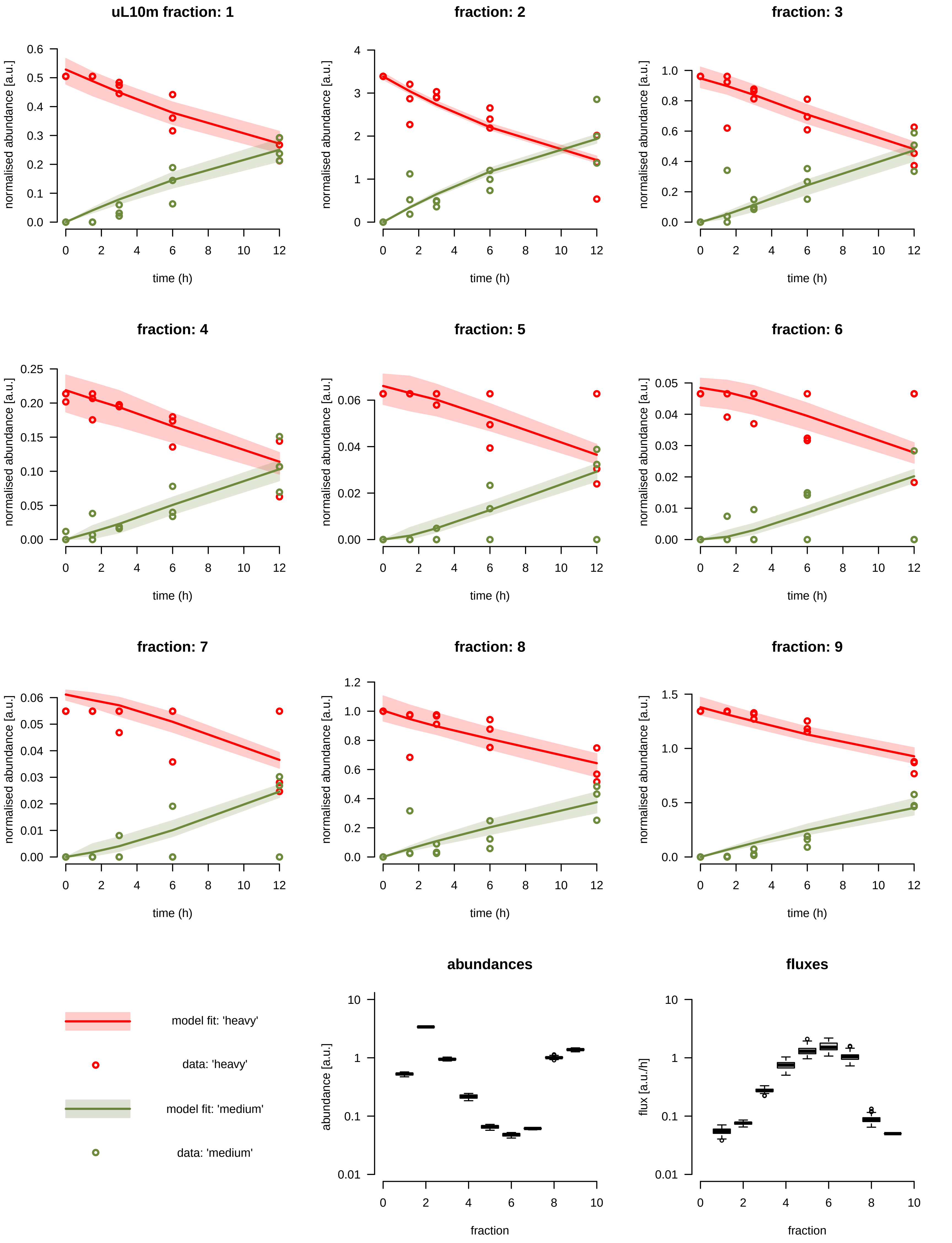

uL10m fraction: 1
fraction: 2
fraction: 3
fraction: 4
fraction: 5
fraction: 6
fraction: 7
fraction: 8
fraction: 9
normalised abundance [a.u.]
time (h)
model fit: 'heavy'
data: 'heavy'
model fit: 'medium'
data: 'medium'
abundances
abundance [a.u.]
fraction
fluxes
flux [a.u./h]
fraction

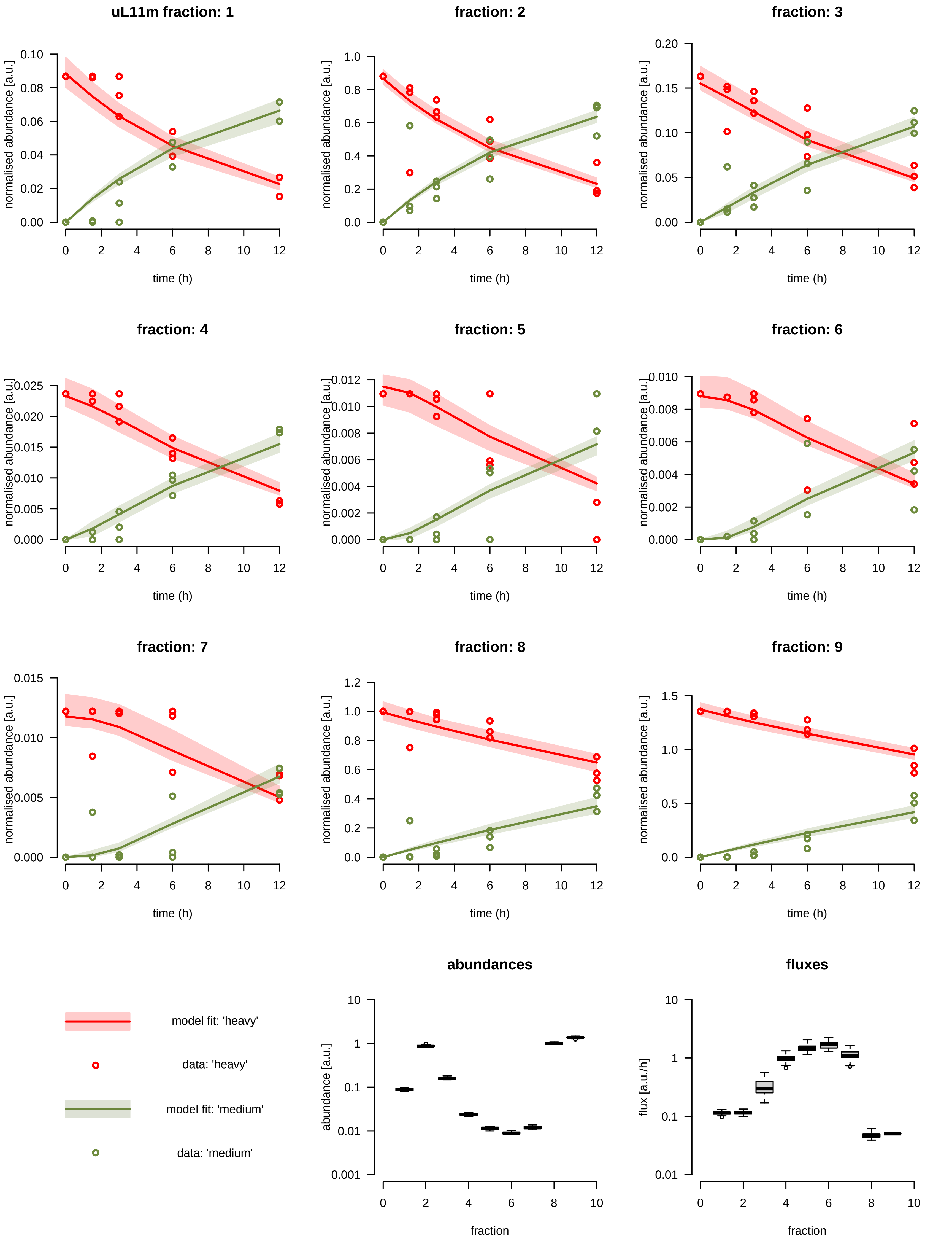

uL11m fraction: 1
fraction: 2
fraction: 3
fraction: 4
fraction: 5
fraction: 6
fraction: 7
fraction: 8
fraction: 9
normalised abundance [a.u.]
time (h)
model fit: 'heavy'
data: 'heavy'
model fit: 'medium'
data: 'medium'
abundances
abundance [a.u.]
fraction
fluxes
flux [a.u./h]

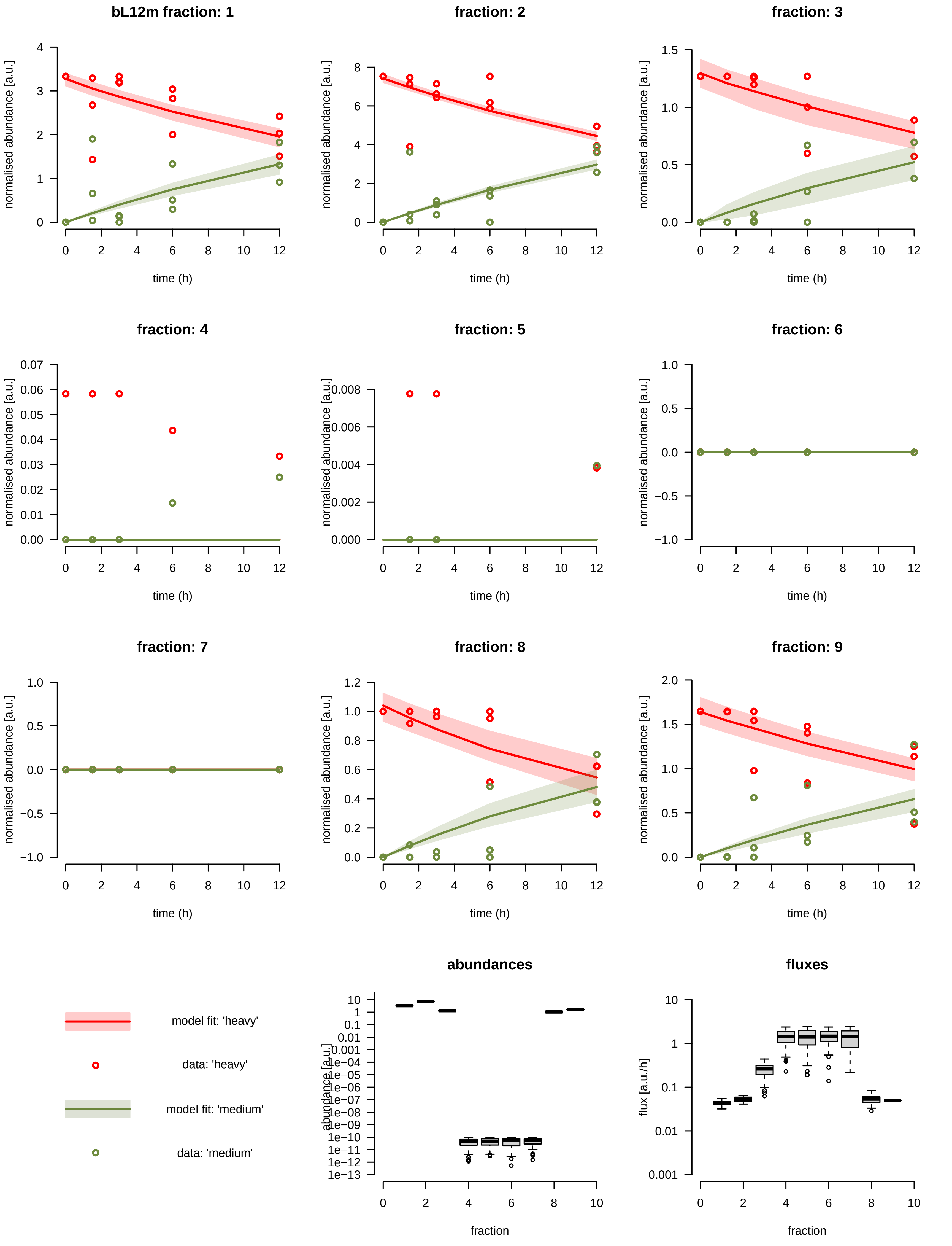
bL12m fraction: 1
fraction: 2
fraction: 3
fraction: 4
fraction: 5
fraction: 6
fraction: 7
fraction: 8
fraction: 9
normalised abundance [a.u.]
time (h)
model fit: 'heavy'
data: 'heavy'
model fit: 'medium'
data: 'medium'
abundances
abundance [a.u.]
fraction
fluxes
flux [a.u./h]
fraction

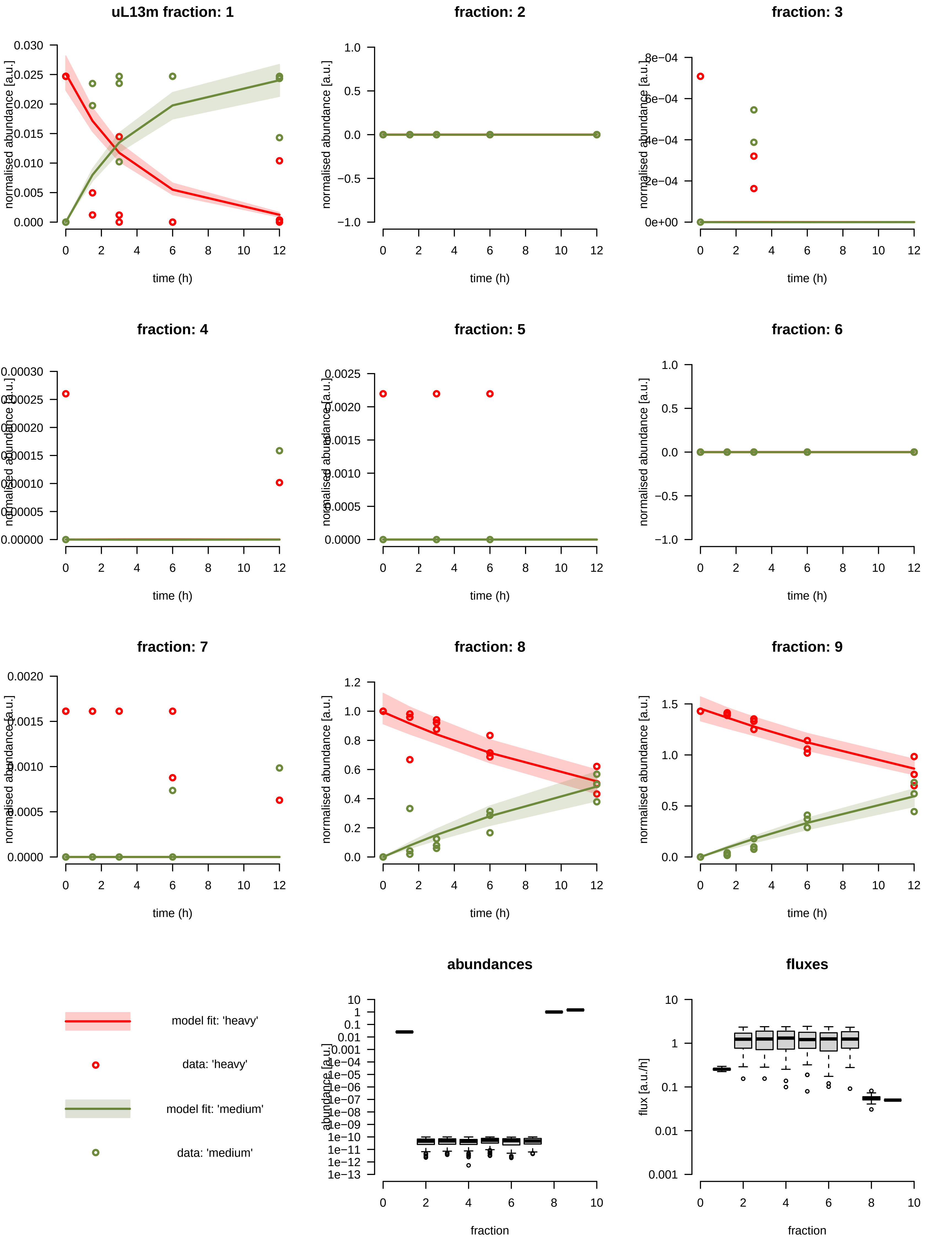

uL13m fraction: 1
fraction: 2
fraction: 3
fraction: 4
fraction: 5
fraction: 6
fraction: 7
fraction: 8
fraction: 9
normalised abundance [a.u.]
time (h)
model fit: 'heavy'
data: 'heavy'
model fit: 'medium'
data: 'medium'
abundances
abundance [a.u.]
fraction
fluxes
flux [a.u./h]
fraction

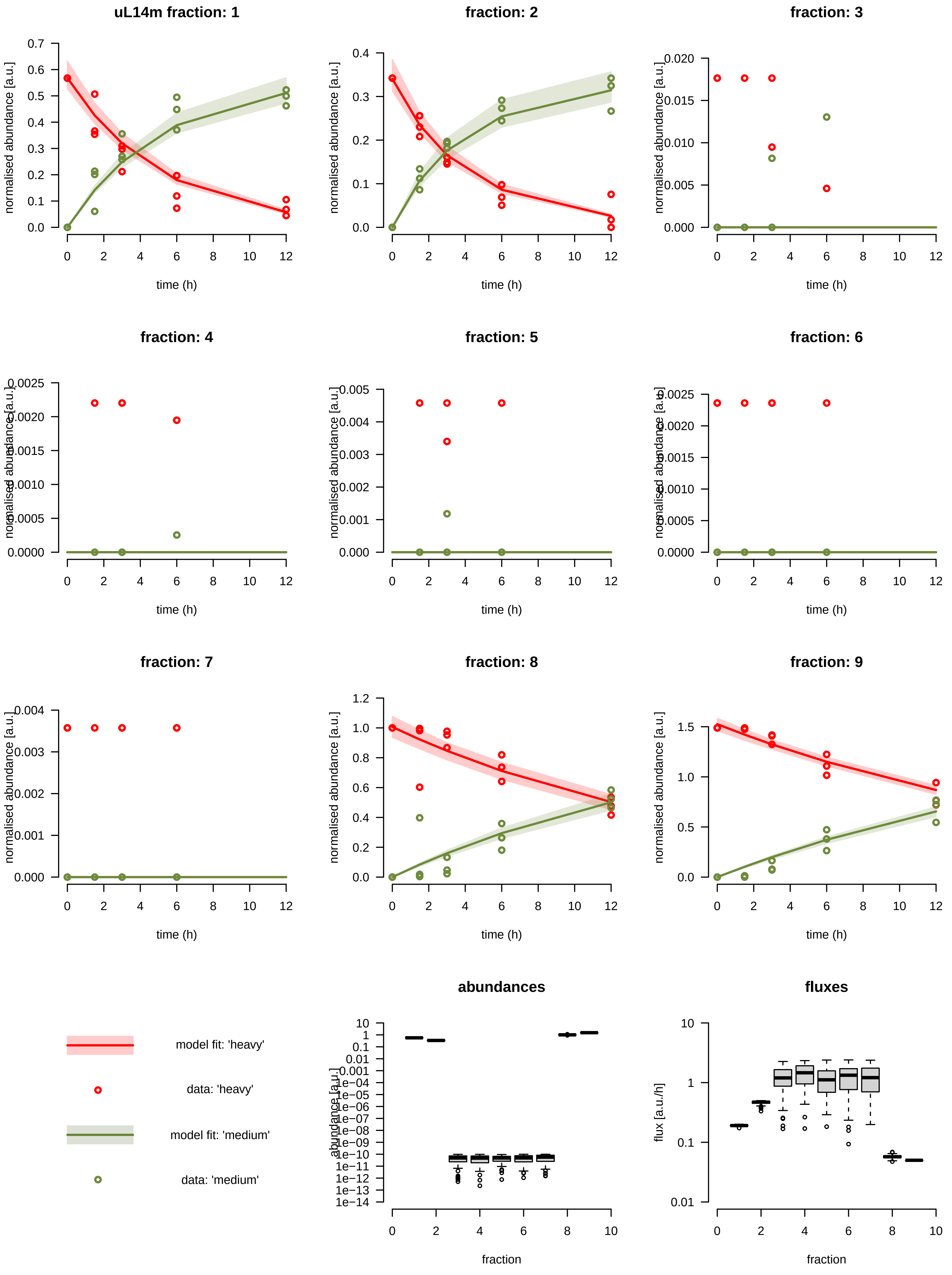

uL14m fraction: 1
fraction: 2
fraction: 3
fraction: 4
fraction: 5
fraction: 6
fraction: 7
fraction: 8
fraction: 9
normalised abundance [a.u.]
time (h)
model fit: 'heavy'
data: 'heavy'
model fit: 'medium'
data: 'medium'
abundances
abundance [a.u.]
fraction
fluxes
flux [a.u./h]
fraction

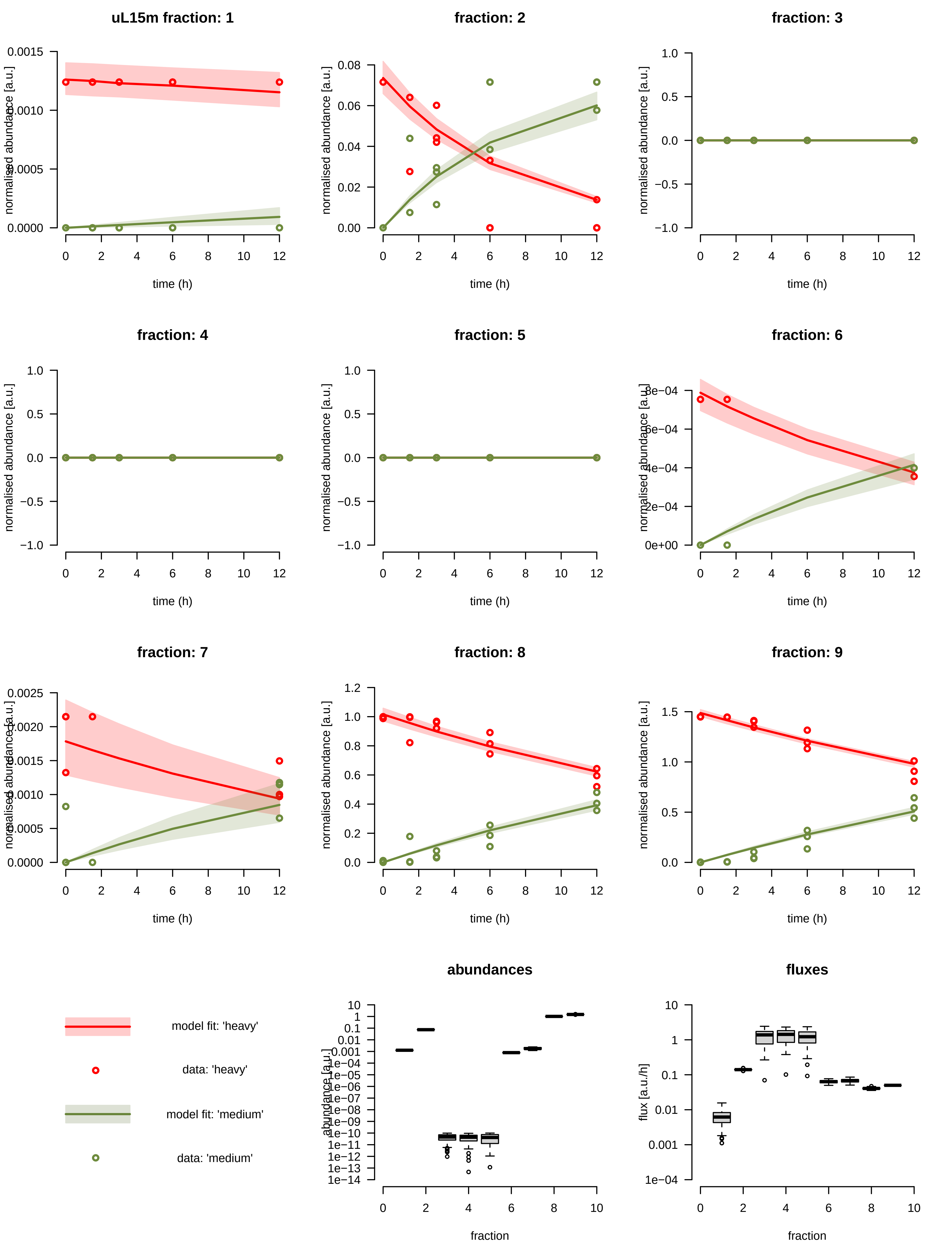

uL15m fraction: 1
fraction: 2
fraction: 3
fraction: 4
fraction: 5
fraction: 6
fraction: 7
fraction: 8
fraction: 9
normalised abundance [a.u.]
time (h)
model fit: 'heavy'
data: 'heavy'
model fit: 'medium'
data: 'medium'
abundances
abundance [a.u.]
fraction
fluxes
flux [a.u./h]
fraction

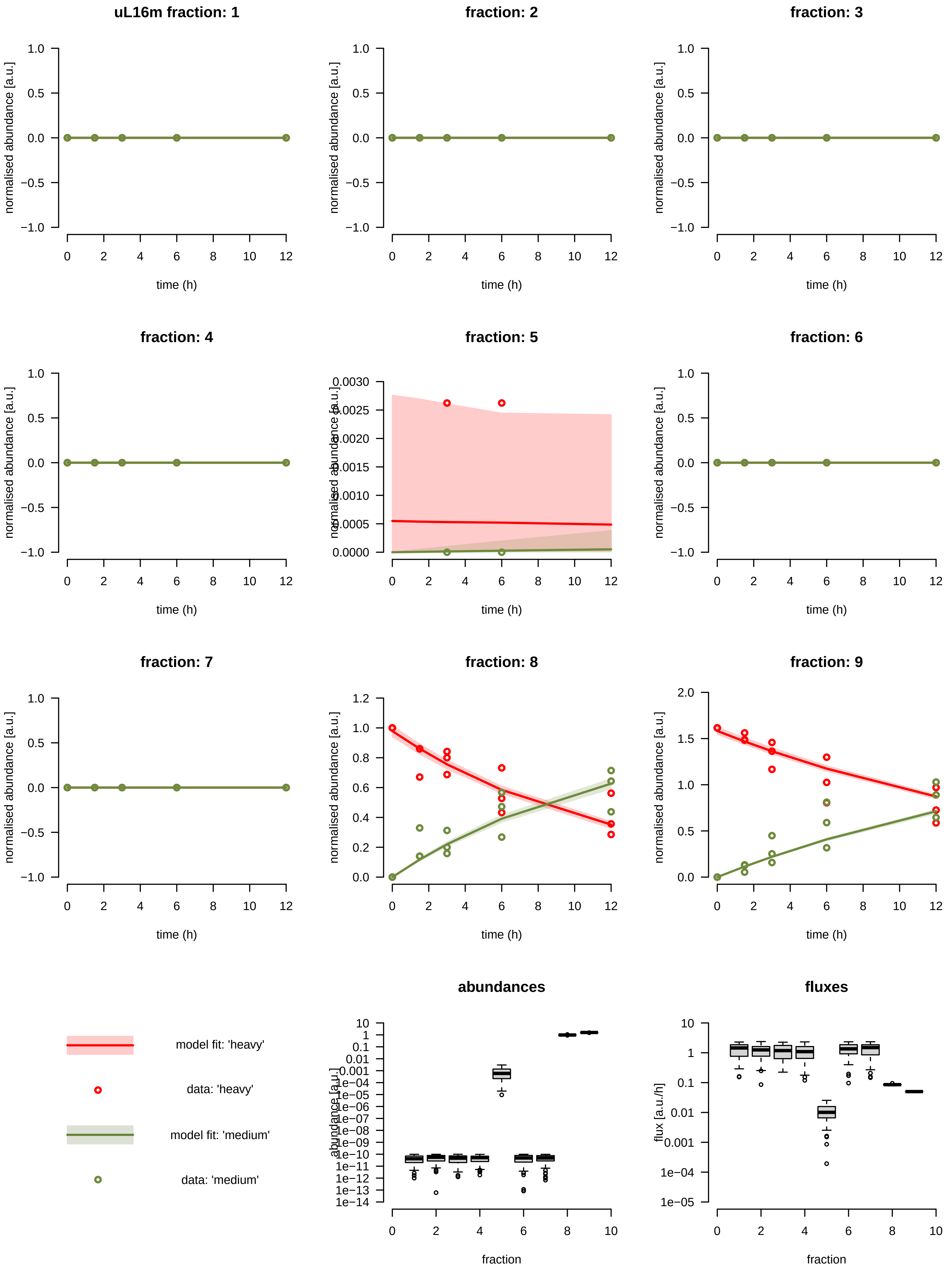
uL16m fraction: 1
fraction: 2
fraction: 3
fraction: 4
fraction: 5
fraction: 6
fraction: 7
fraction: 8
fraction: 9
normalised abundance [a.u.]
time (h)
model fit: 'heavy'
data: 'heavy'
model fit: 'medium'
data: 'medium'
abundances
abundance [a.u.]
fraction
fluxes
flux [a.u./h]
fraction

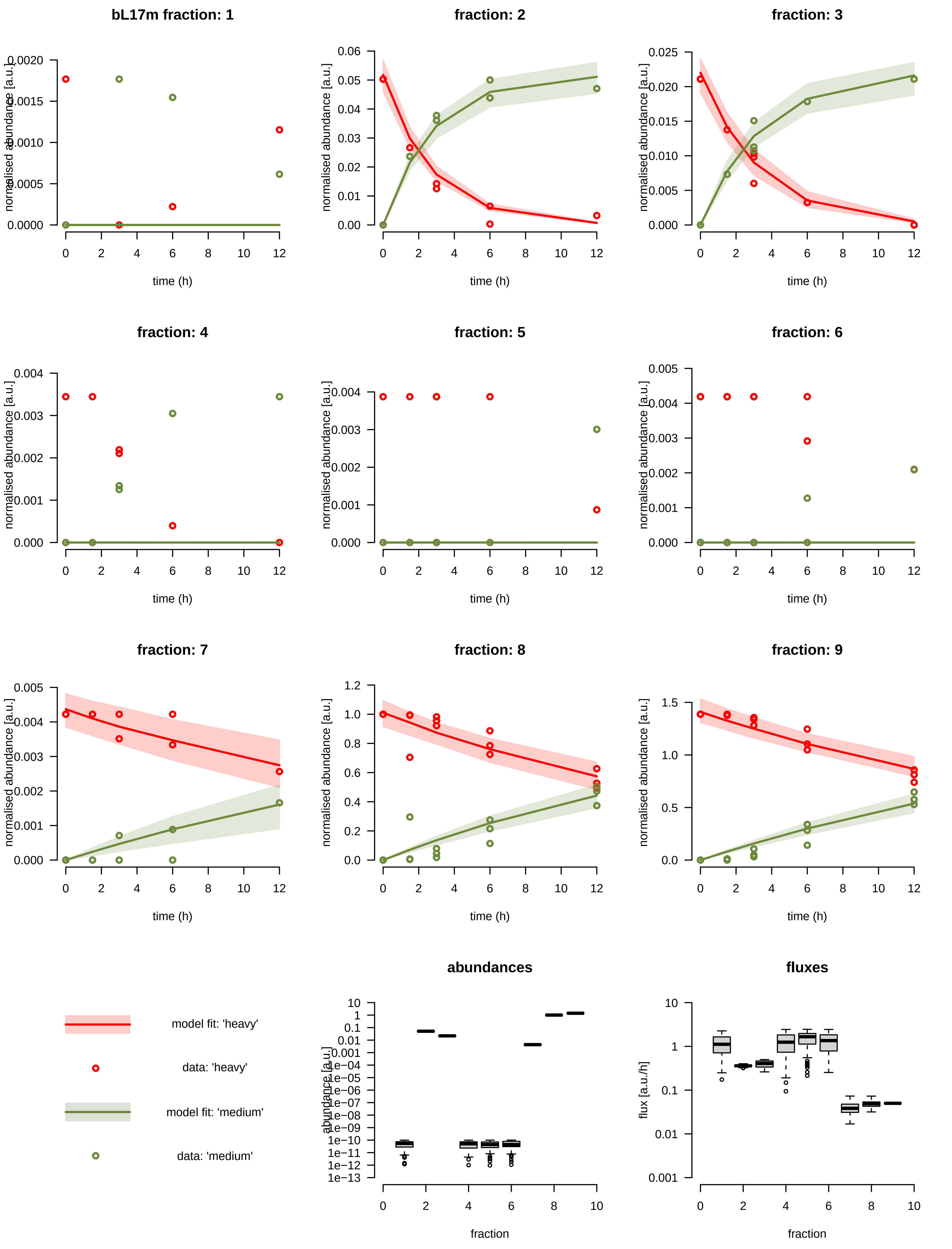
bL17m fraction: 1
fraction: 2
fraction: 3
fraction: 4
fraction: 5
fraction: 6
fraction: 7
fraction: 8
fraction: 9
abundances
fluxes
normalised abundance [a.u.]
time (h)
abundance [a.u.]
flux [a.u./h]
fraction
model fit: 'heavy'
data: 'heavy'
model fit: 'medium'
data: 'medium'

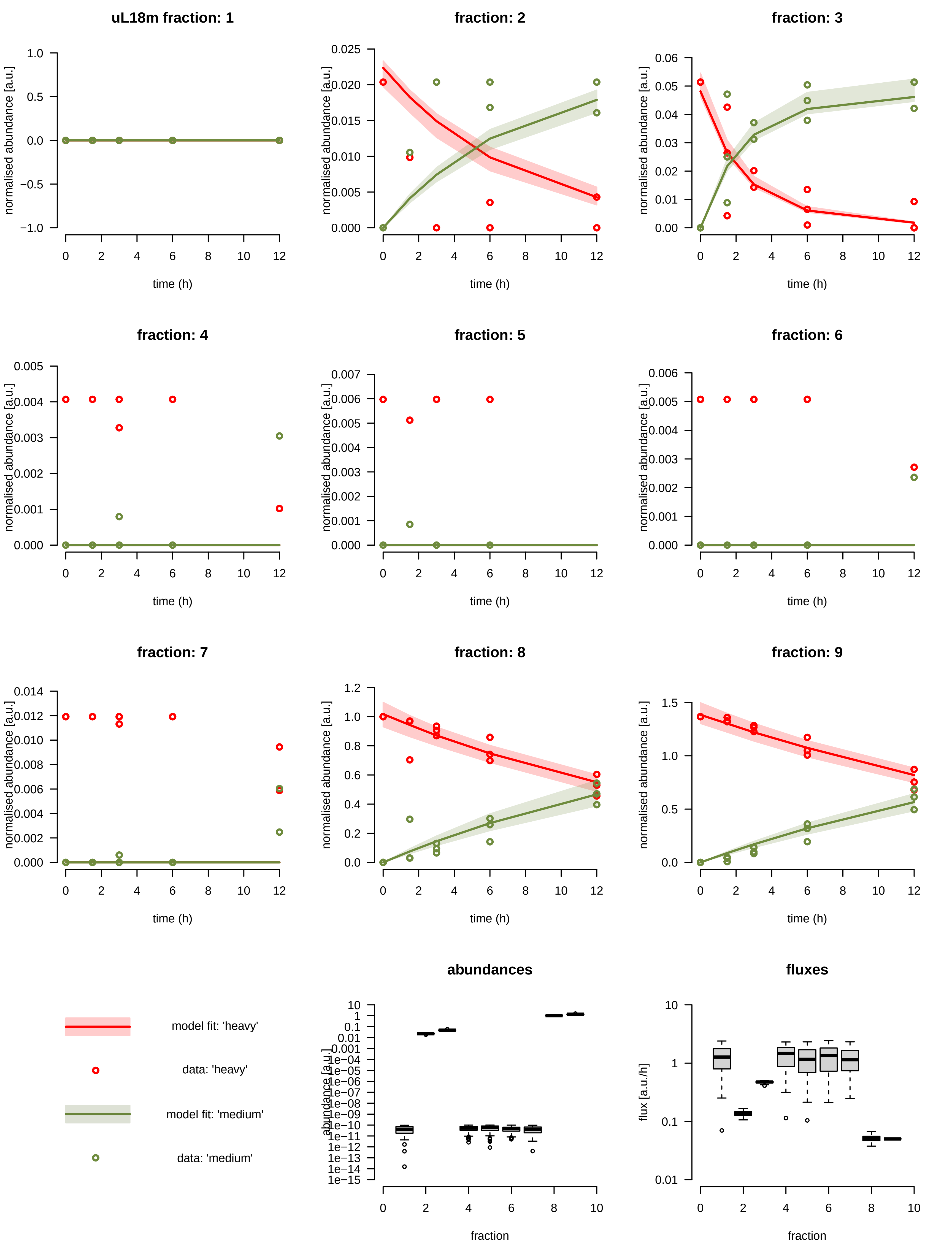
uL18m fraction: 1
fraction: 2
fraction: 3
fraction: 4
fraction: 5
fraction: 6
fraction: 7
fraction: 8
fraction: 9
normalised abundance [a.u.]
time (h)
0 2 4 6 8 10 12
1.0 0.5 0.0 −0.5 −1.0
0.025 0.020 0.015 0.010 0.005 0.000
0.06 0.05 0.04 0.03 0.02 0.01 0.00
0.005 0.004 0.003 0.002 0.001 0.000
0.007 0.006 0.005 0.004 0.003 0.002 0.001 0.000
0.006 0.005 0.004 0.003 0.002 0.001 0.000
0.014 0.012 0.010 0.008 0.006 0.004 0.002 0.000
1.2 1.0 0.8 0.6 0.4 0.2 0.0
1.5 1.0 0.5 0.0
model fit: 'heavy'
data: 'heavy'
model fit: 'medium'
data: 'medium'
abundances
abundance [a.u.]
10 1 0.1 0.01 0.001 1e−04 1e−05 1e−06 1e−07 1e−08 1e−09 1e−10 1e−11 1e−12 1e−13 1e−14 1e−15
fraction
0 2 4 6 8 10
fluxes
flux [a.u./h]
10 1 0.1 0.01

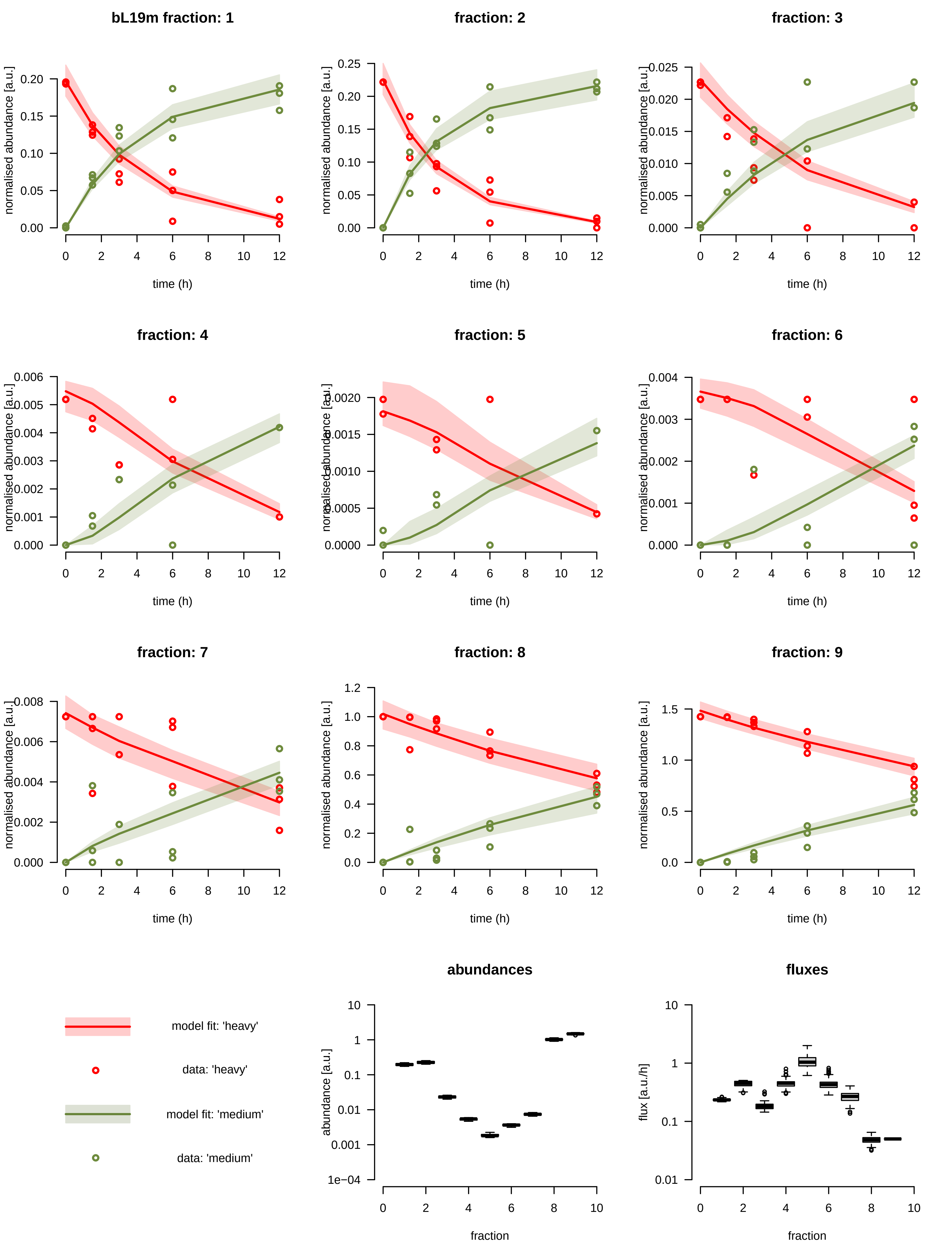

bL19m fraction: 1
fraction: 2
fraction: 3
fraction: 4
fraction: 5
fraction: 6
fraction: 7
fraction: 8
fraction: 9
normalised abundance [a.u.]
time (h)
abundances
abundance [a.u.]
fraction
fluxes
flux [a.u./h]
model fit: 'heavy'
data: 'heavy'
model fit: 'medium'
data: 'medium'

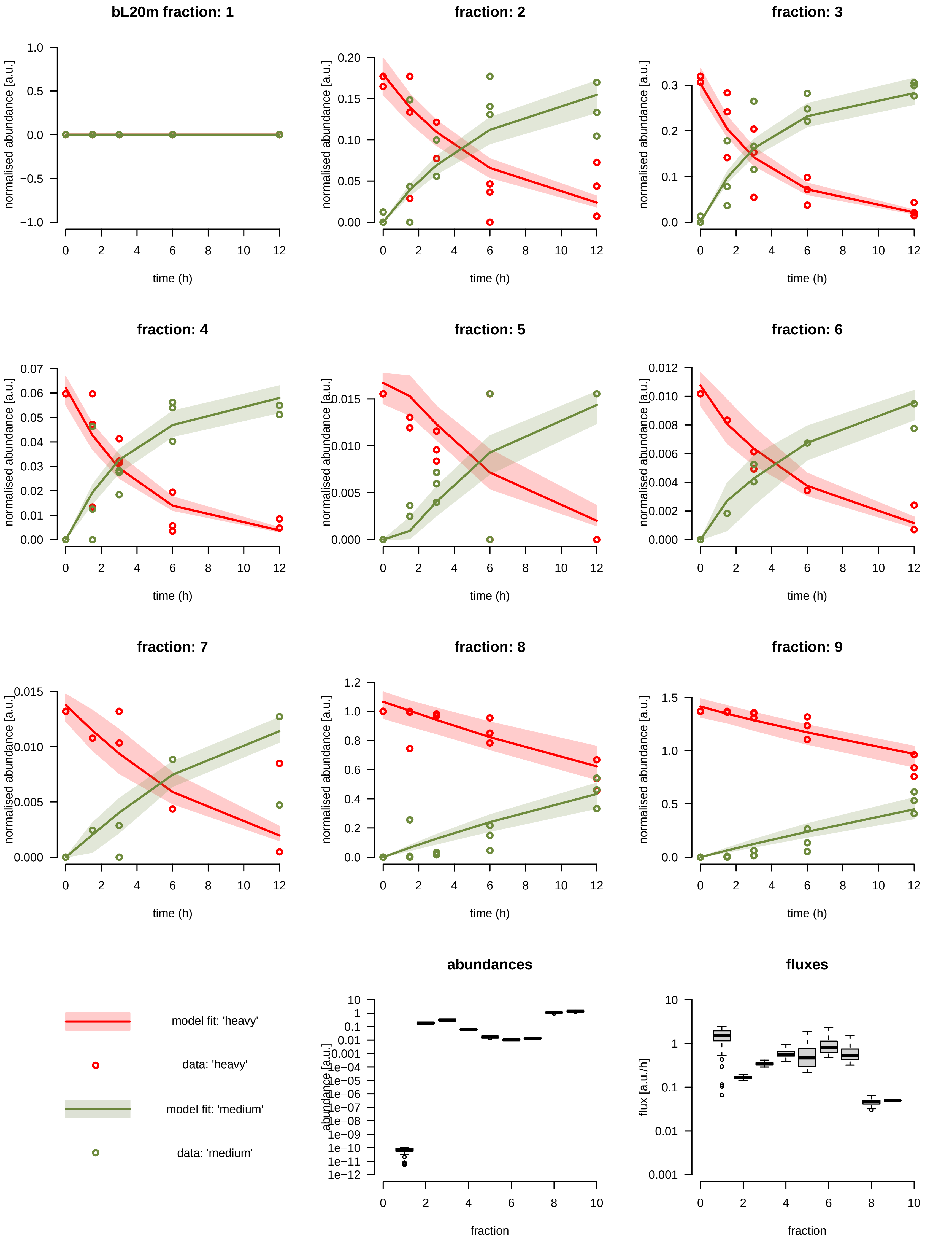
bL20m fraction: 1
fraction: 2
fraction: 3
fraction: 4
fraction: 5
fraction: 6
fraction: 7
fraction: 8
fraction: 9
normalised abundance [a.u.]
time (h)
model fit: 'heavy'
data: 'heavy'
model fit: 'medium'
data: 'medium'
abundances
abundance [a.u.]
fraction
fluxes
flux [a.u./h]
fraction

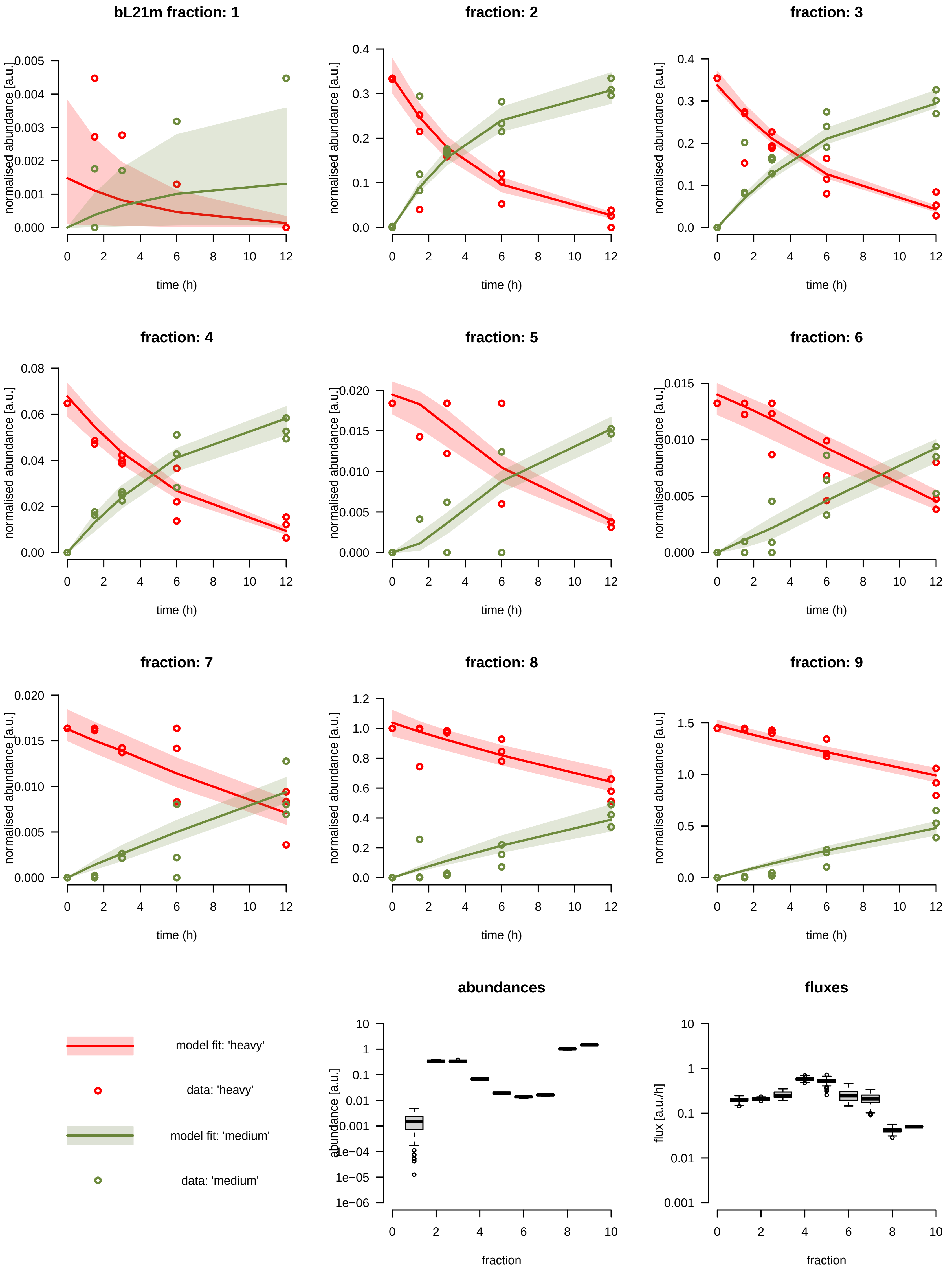
bL21m fraction: 1
fraction: 2
fraction: 3
fraction: 4
fraction: 5
fraction: 6
fraction: 7
fraction: 8
fraction: 9
normalised abundance [a.u.]
time (h)
model fit: 'heavy'
data: 'heavy'
model fit: 'medium'
data: 'medium'
abundances
abundance [a.u.]
fraction
fluxes
flux [a.u./h]
fraction

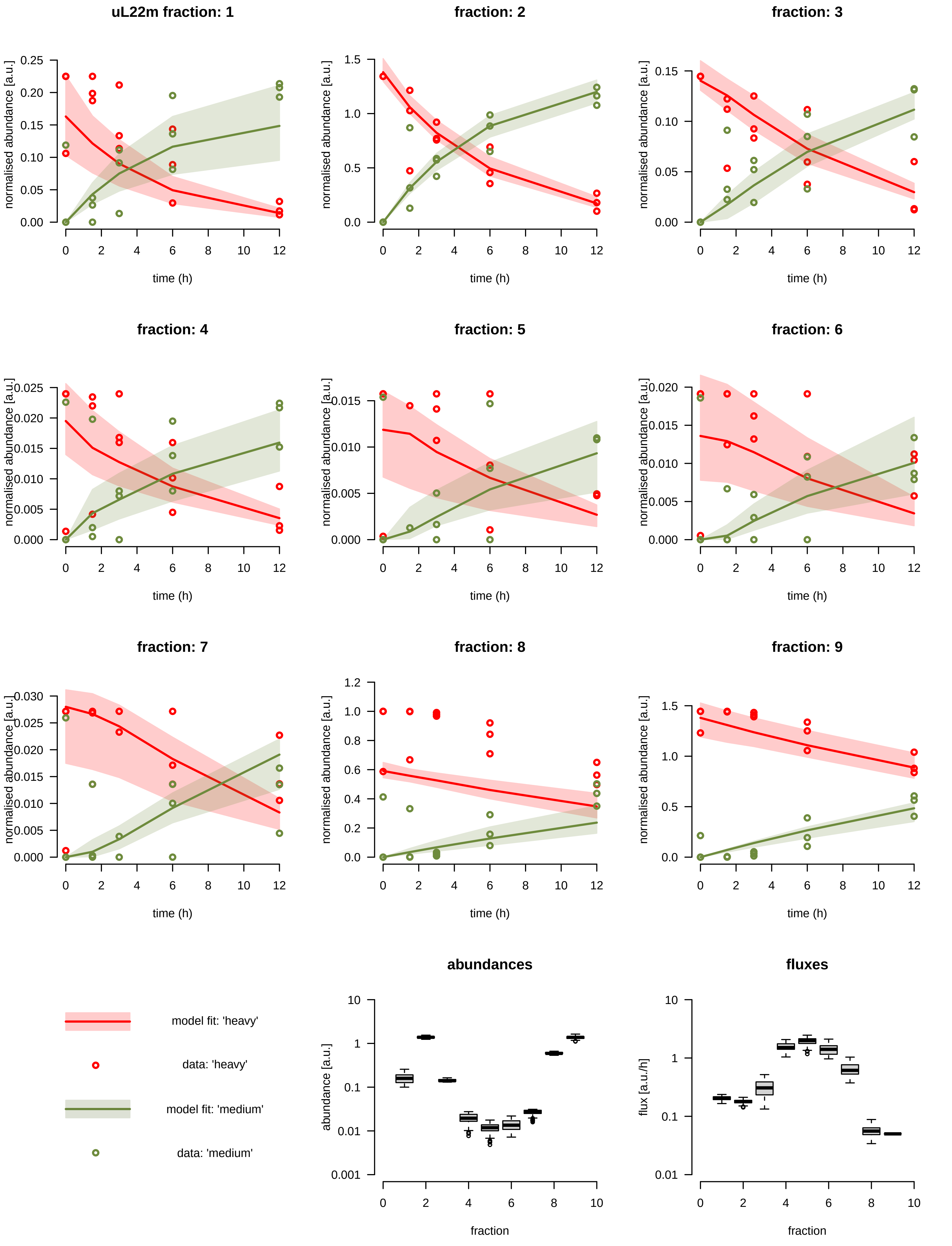
uL22m fraction: 1
fraction: 2
fraction: 3
fraction: 4
fraction: 5
fraction: 6
fraction: 7
fraction: 8
fraction: 9
normalised abundance [a.u.]
time (h)
model fit: 'heavy'
data: 'heavy'
model fit: 'medium'
data: 'medium'
abundances
abundance [a.u.]
fraction
fluxes
flux [a.u./h]
fraction

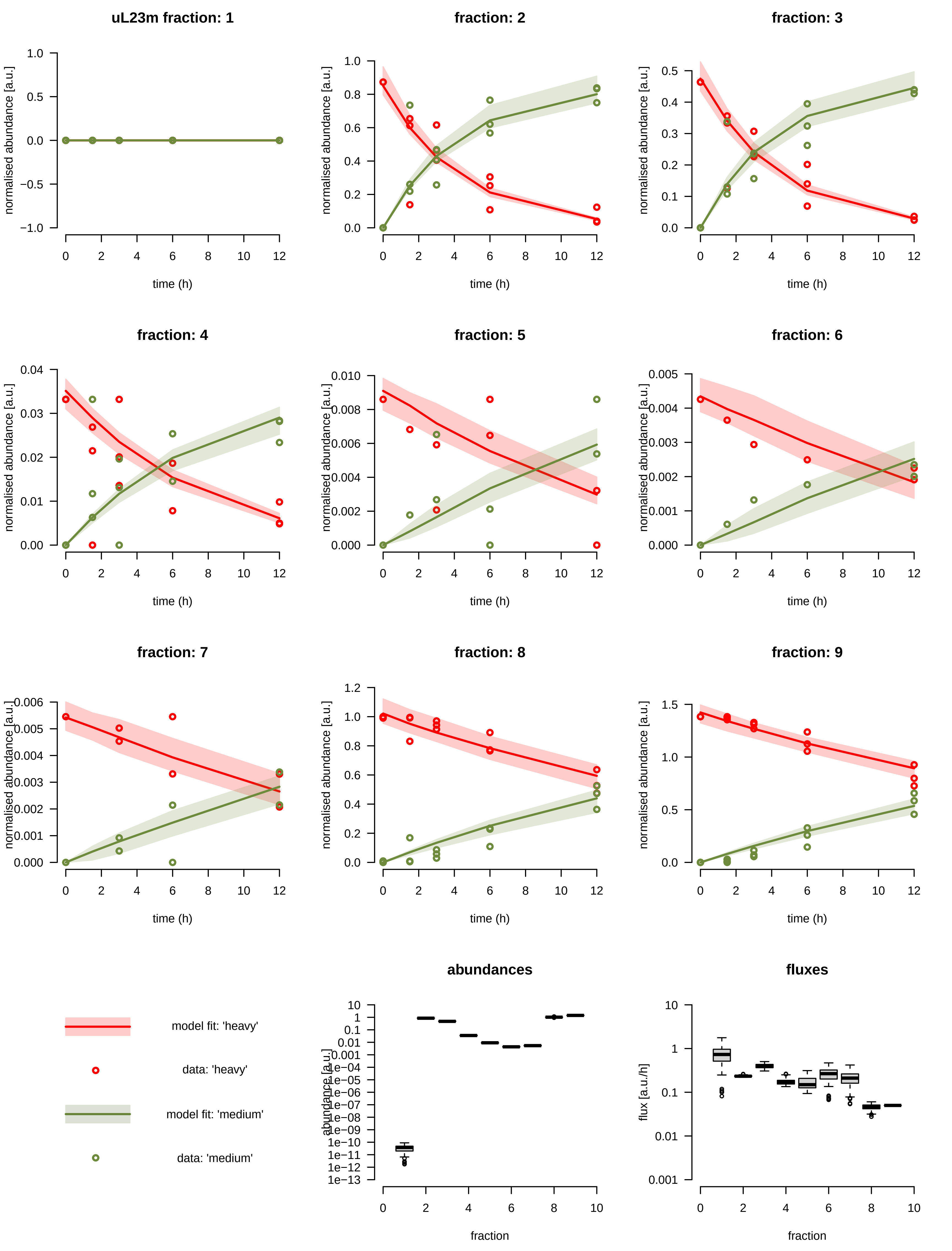
uL23m fraction: 1
fraction: 2
fraction: 3
fraction: 4
fraction: 5
fraction: 6
fraction: 7
fraction: 8
fraction: 9
normalised abundance [a.u.]
time (h)
model fit: 'heavy'
data: 'heavy'
model fit: 'medium'
data: 'medium'
abundances
abundance [a.u.]
fraction
fluxes
flux [a.u./h]
fraction

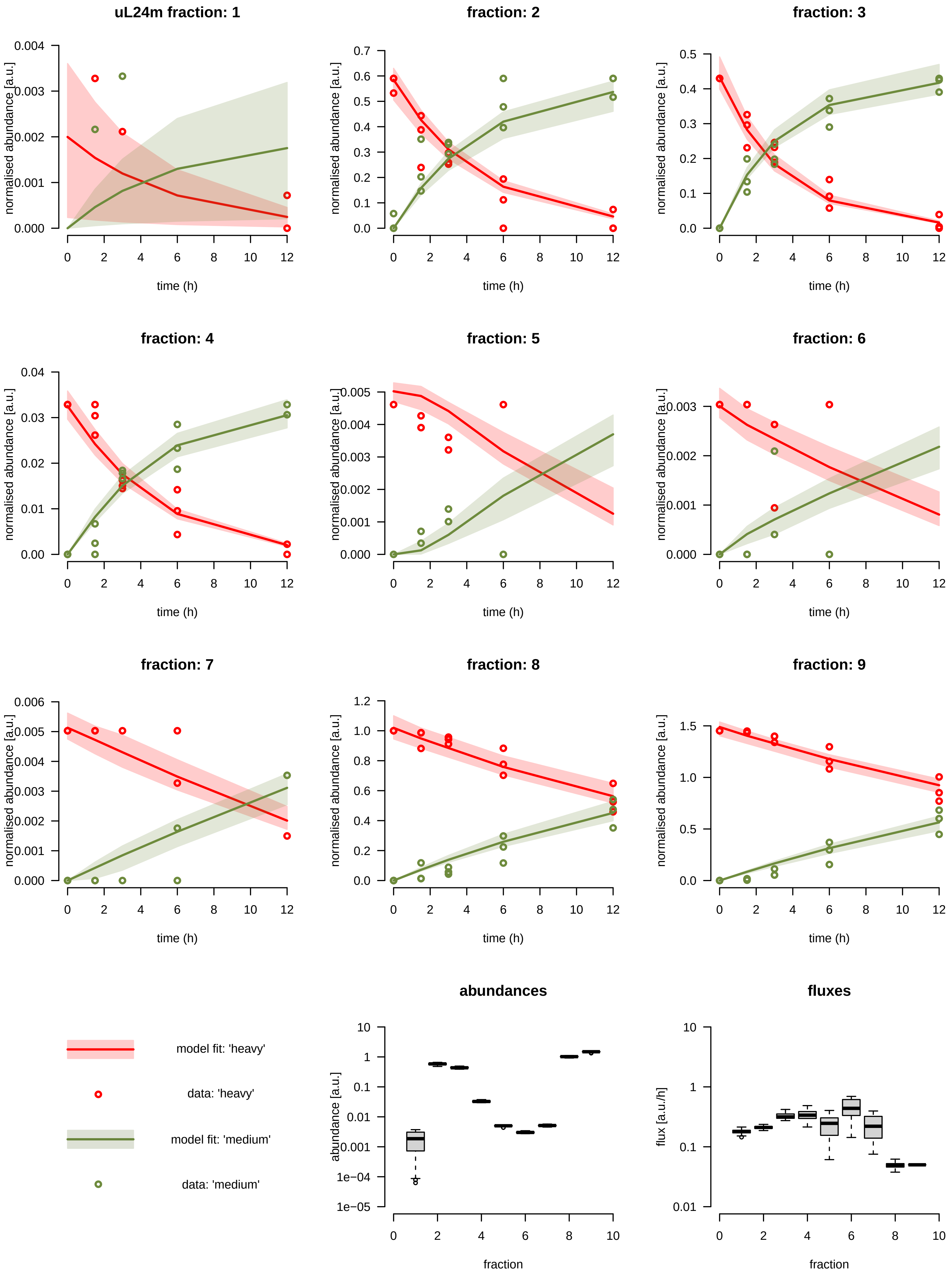
uL24m fraction: 1
fraction: 2
fraction: 3
fraction: 4
fraction: 5
fraction: 6
fraction: 7
fraction: 8
fraction: 9
normalised abundance [a.u.]
time (h)
model fit: 'heavy'
data: 'heavy'
model fit: 'medium'
data: 'medium'
abundances
abundance [a.u.]
fraction
fluxes
flux [a.u./h]
fraction

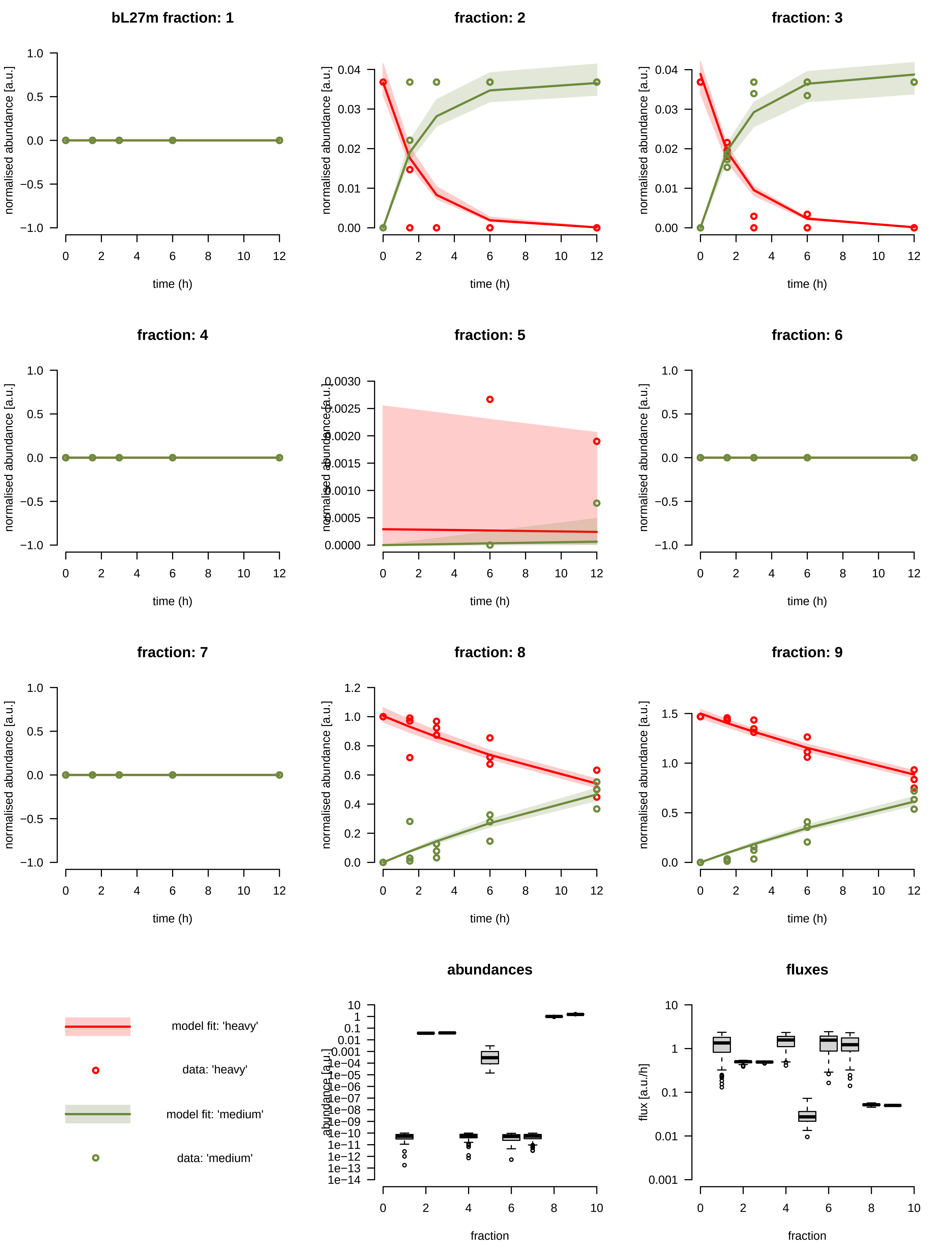
bL27m fraction: 1
fraction: 2
fraction: 3
fraction: 4
fraction: 5
fraction: 6
fraction: 7
fraction: 8
fraction: 9
normalised abundance [a.u.]
time (h)
model fit: 'heavy'
data: 'heavy'
model fit: 'medium'
data: 'medium'
abundances
abundance [a.u.]
fraction
fluxes
flux [a.u./h]

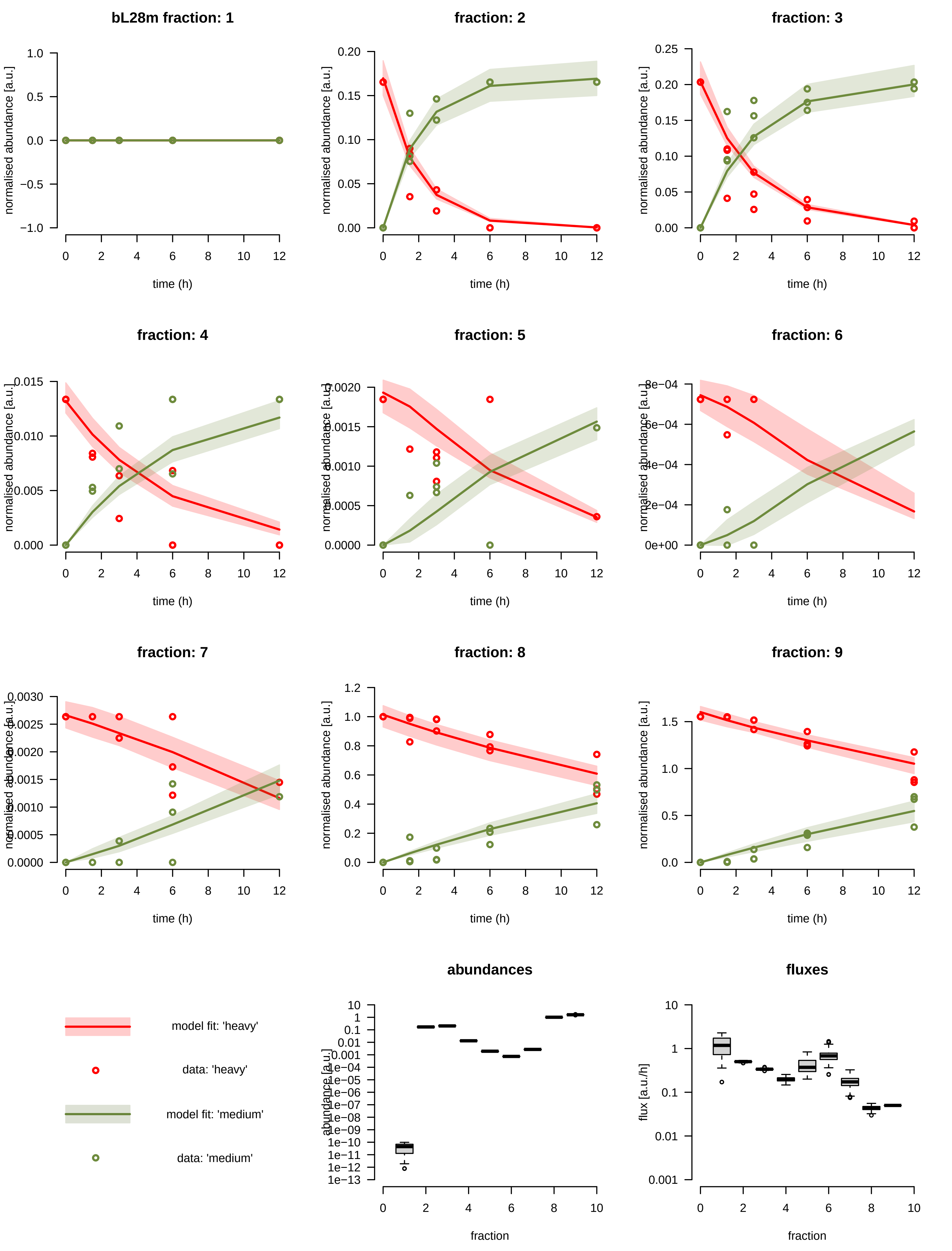

bL28m fraction: 1
fraction: 2
fraction: 3
fraction: 4
fraction: 5
fraction: 6
fraction: 7
fraction: 8
fraction: 9
abundances
fluxes
normalised abundance [a.u.]
time (h)
abundance [a.u.]
flux [a.u./h]
fraction
model fit: 'heavy'
data: 'heavy'
model fit: 'medium'
data: 'medium'

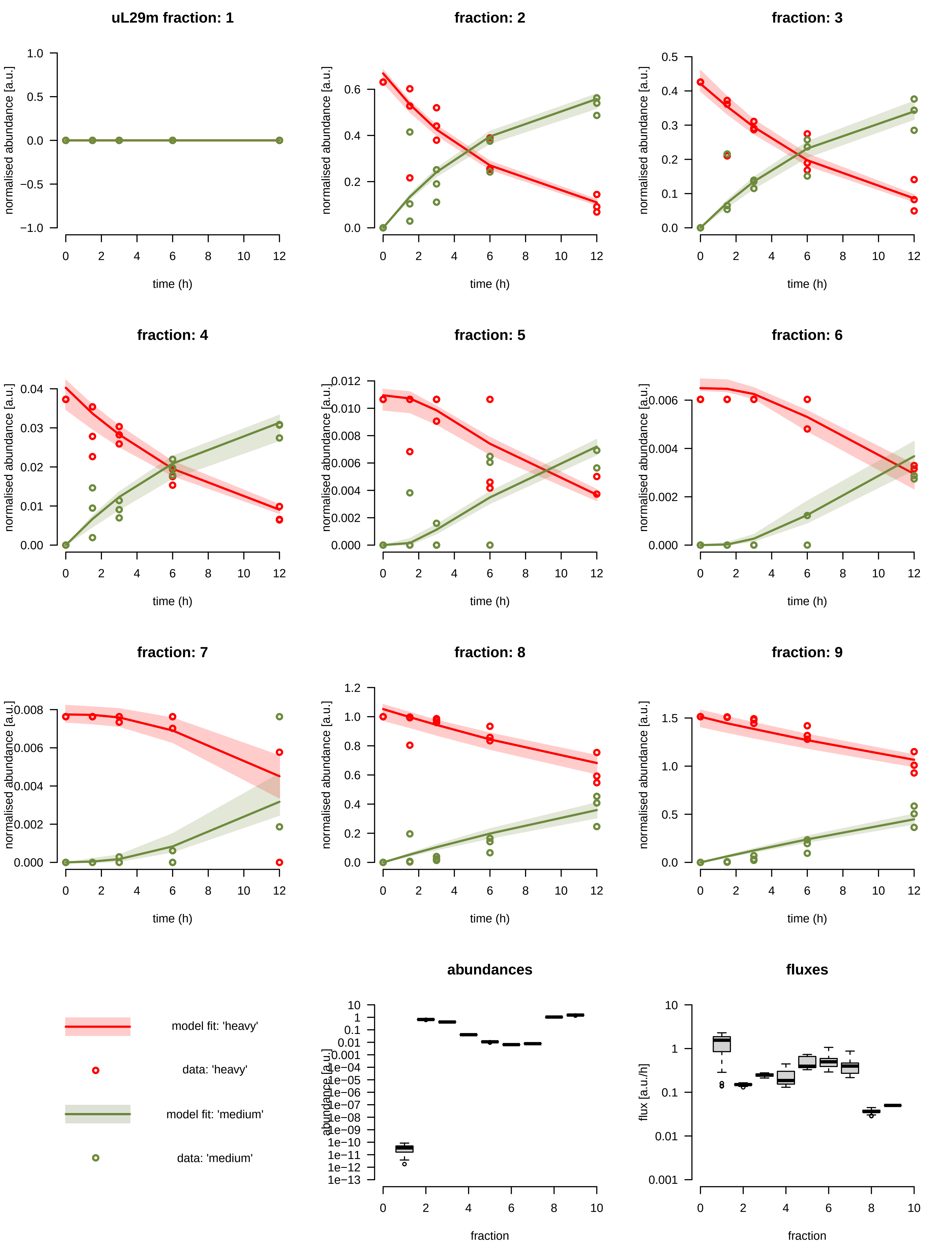

uL29m fraction: 1
fraction: 2
fraction: 3
fraction: 4
fraction: 5
fraction: 6
fraction: 7
fraction: 8
fraction: 9
normalised abundance [a.u.]
time (h)
model fit: 'heavy'
data: 'heavy'
model fit: 'medium'
data: 'medium'
abundances
abundance [a.u.]
fraction
fluxes
flux [a.u./h]
fraction

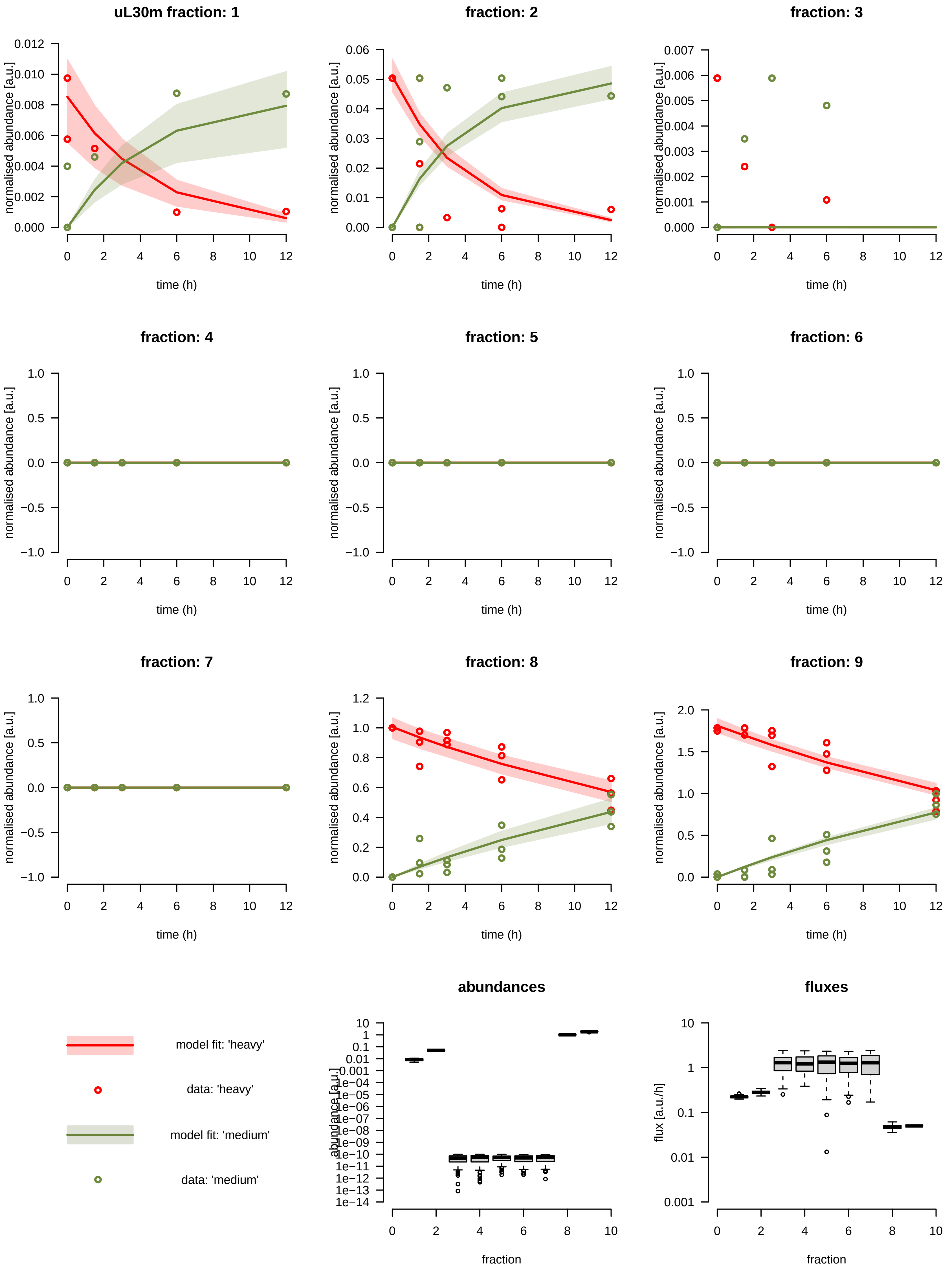

uL30m fraction: 1
fraction: 2
fraction: 3
fraction: 4
fraction: 5
fraction: 6
fraction: 7
fraction: 8
fraction: 9
normalised abundance [a.u.]
time (h)
model fit: 'heavy'
data: 'heavy'
model fit: 'medium'
data: 'medium'
abundances
abundance [a.u.]
fraction
fluxes
flux [a.u./h]

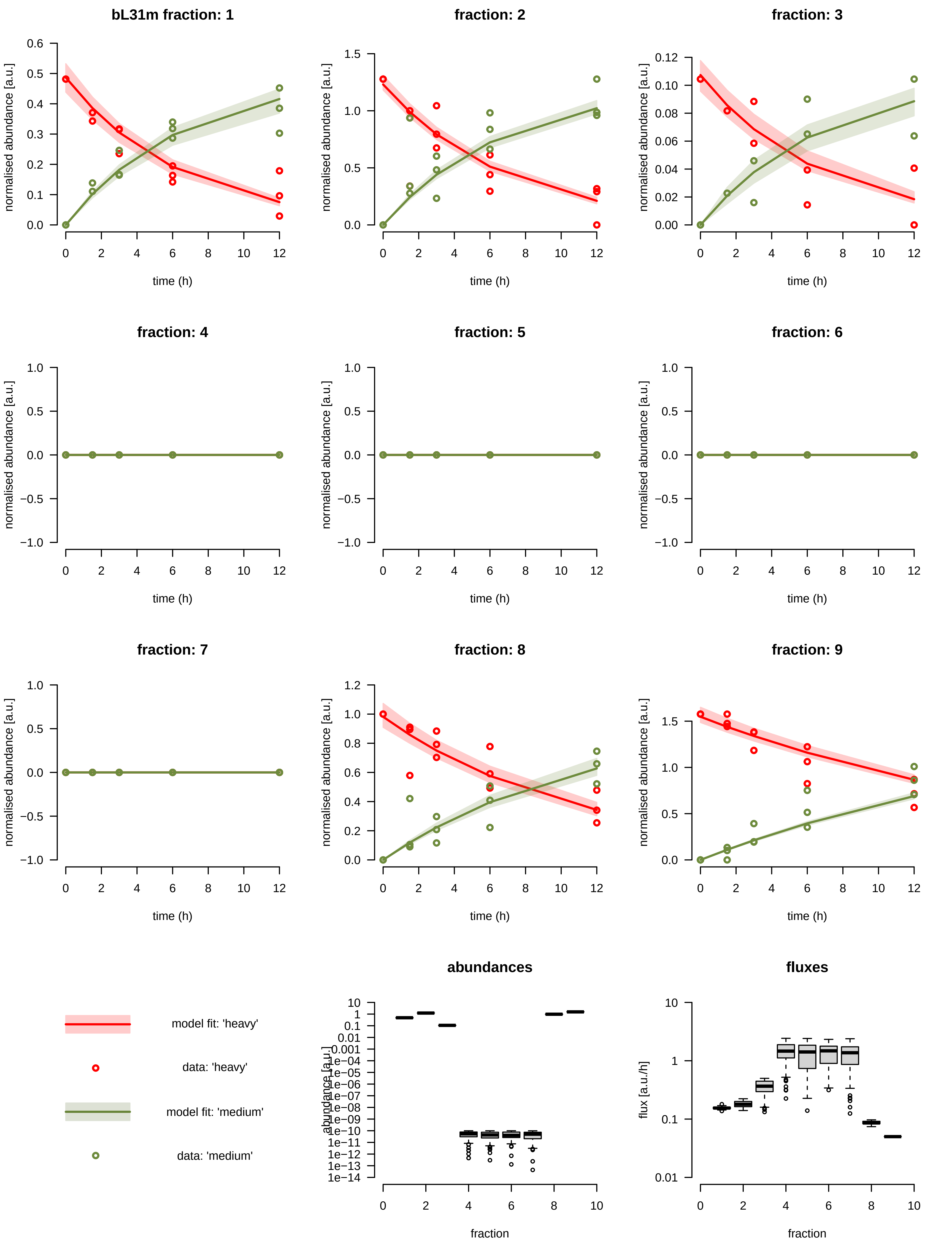
bL31m fraction: 1
fraction: 2
fraction: 3
fraction: 4
fraction: 5
fraction: 6
fraction: 7
fraction: 8
fraction: 9
normalised abundance [a.u.]
time (h)
model fit: 'heavy'
data: 'heavy'
model fit: 'medium'
data: 'medium'
abundances
abundance [a.u.]
fraction
fluxes
flux [a.u./h]
fraction

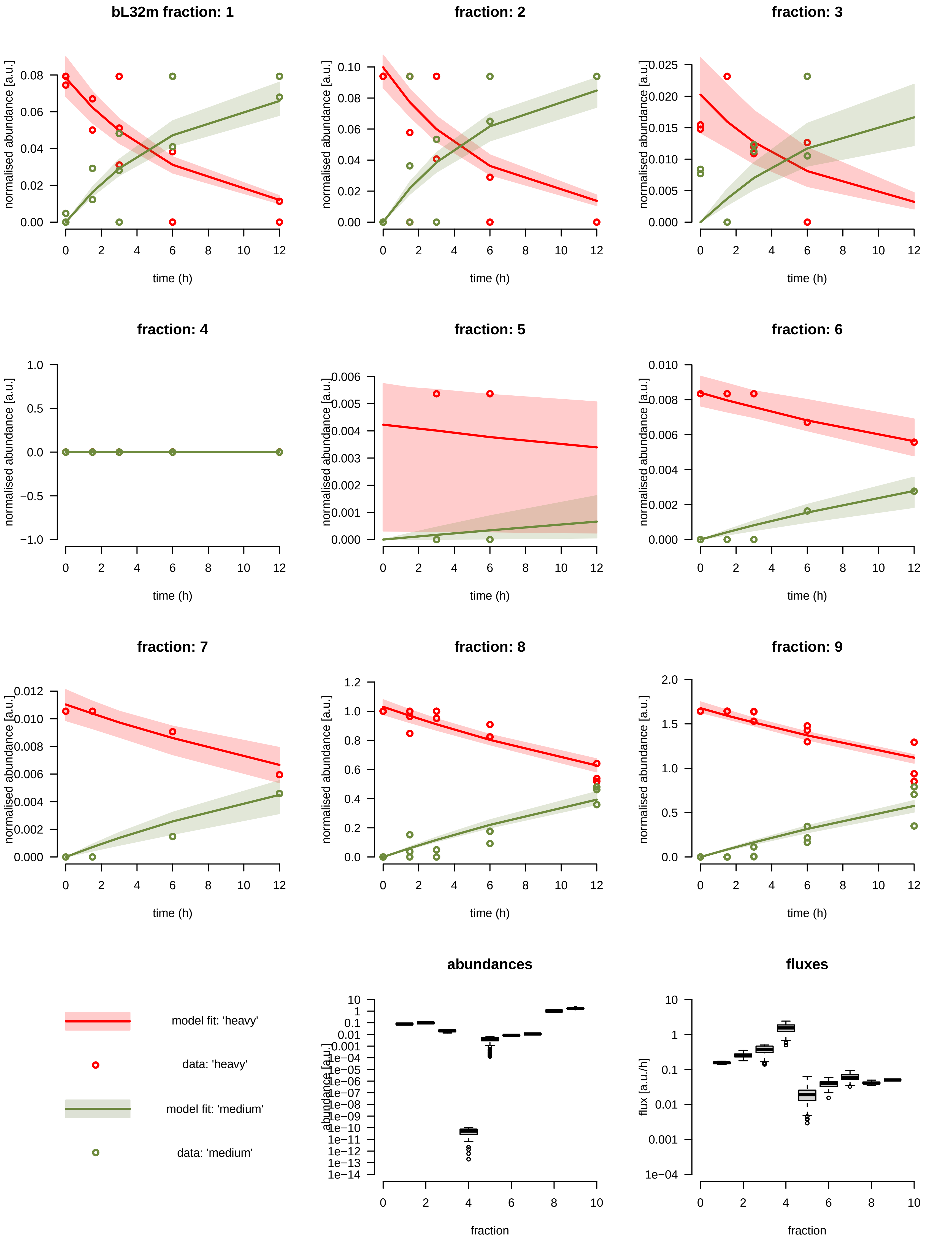
bL32m fraction: 1
fraction: 2
fraction: 3
fraction: 4
fraction: 5
fraction: 6
fraction: 7
fraction: 8
fraction: 9
normalised abundance [a.u.]
time (h)
model fit: 'heavy'
data: 'heavy'
model fit: 'medium'
data: 'medium'
abundances
abundance [a.u.]
fraction
fluxes
flux [a.u./h]
fraction

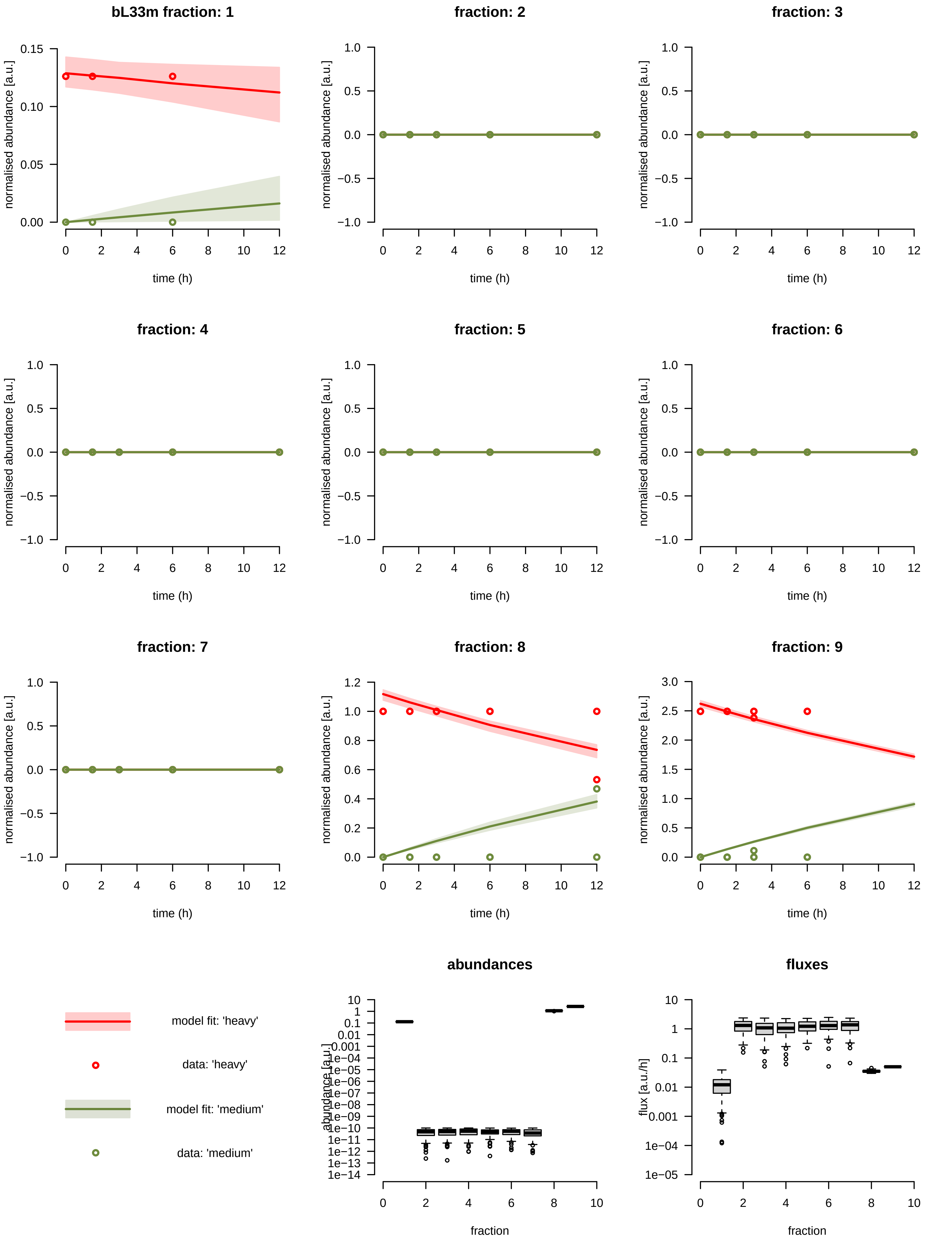

bL33m fraction: 1
fraction: 2
fraction: 3
fraction: 4
fraction: 5
fraction: 6
fraction: 7
fraction: 8
fraction: 9
normalised abundance [a.u.]
time (h)
model fit: 'heavy'
data: 'heavy'
model fit: 'medium'
data: 'medium'
abundances
abundance [a.u.]
fraction
fluxes
flux [a.u./h]

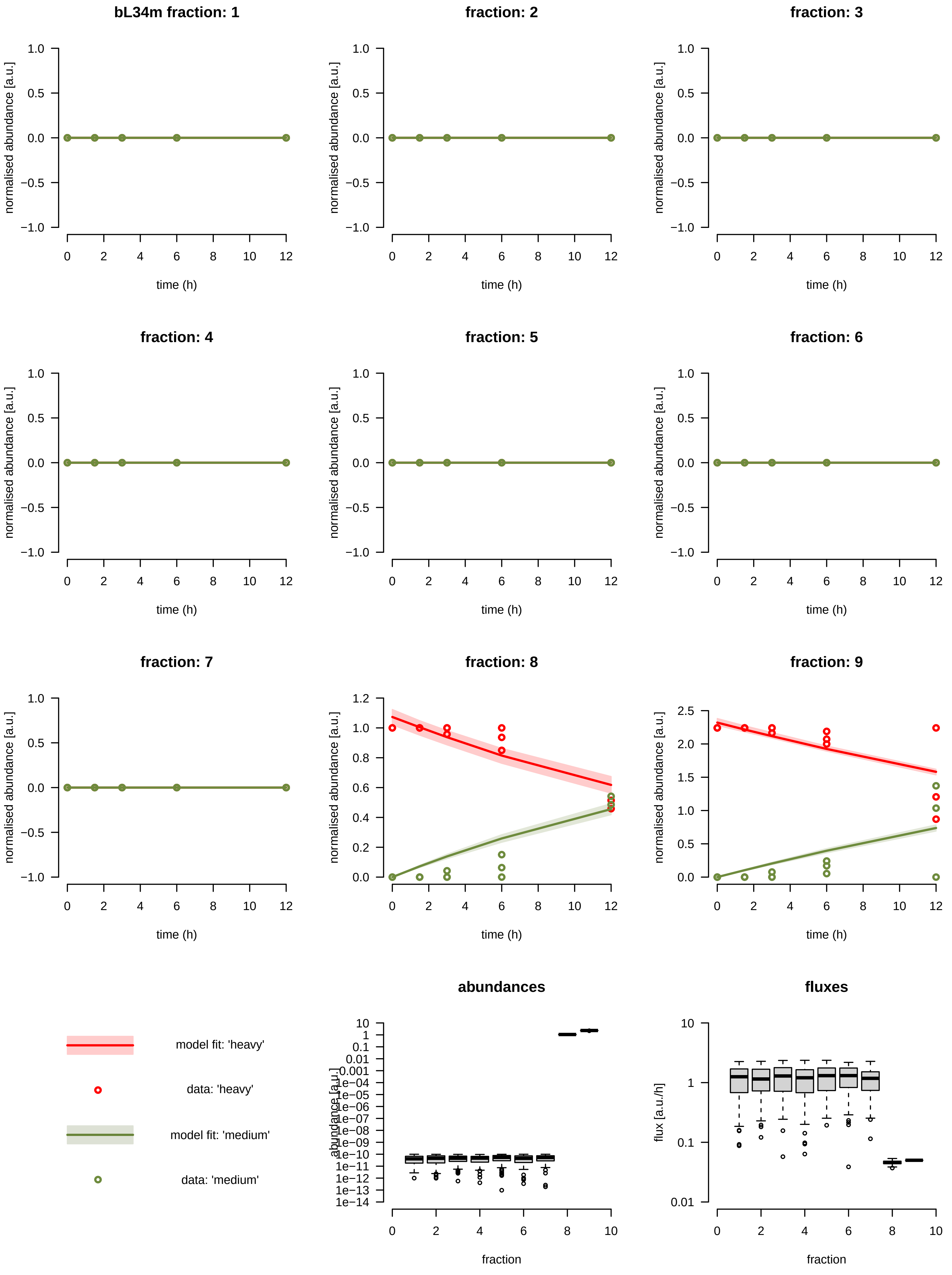
bL34m fraction: 1
fraction: 2
fraction: 3
fraction: 4
fraction: 5
fraction: 6
fraction: 7
fraction: 8
fraction: 9
normalised abundance [a.u.]
time (h)
model fit: 'heavy'
data: 'heavy'
model fit: 'medium'
data: 'medium'
abundances
abundance [a.u.]
fraction
fluxes
flux [a.u./h]
fraction

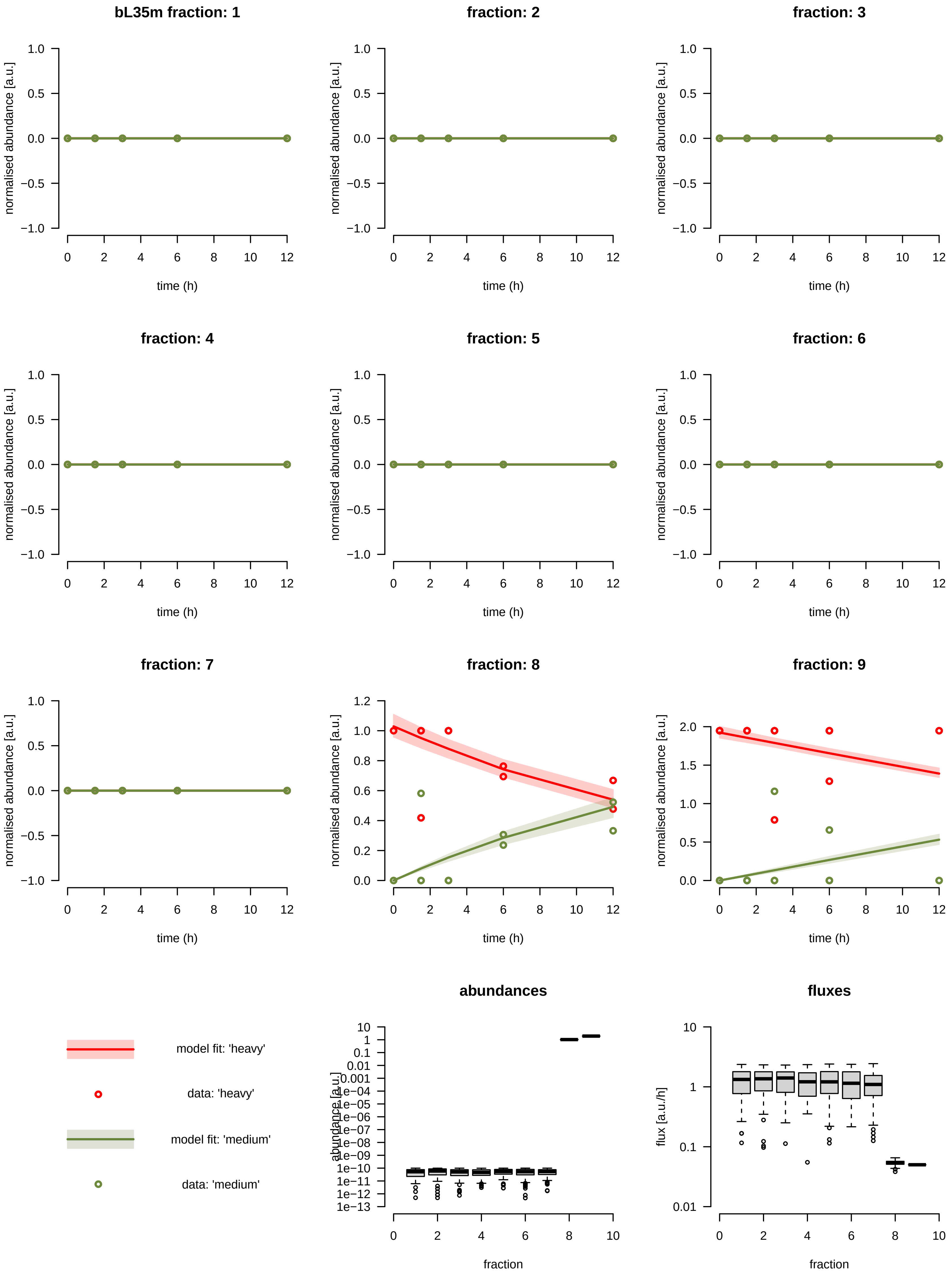

bL35m fraction: 1
fraction: 2
fraction: 3
fraction: 4
fraction: 5
fraction: 6
fraction: 7
fraction: 8
fraction: 9
normalised abundance [a.u.]
time (h)
1.0
0.5
0.0
−0.5
−1.0
1.2
0.8
0.6
0.4
0.2
2.0
1.5
0
2
4
6
8
10
12
model fit: 'heavy'
data: 'heavy'
model fit: 'medium'
data: 'medium'
abundances
abundance [a.u.]
10
1
0.1
0.01
0.001
1e−04
1e−05
1e−06
1e−07
1e−08
1e−09
1e−10
1e−11
1e−12
1e−13
fraction
fluxes
flux [a.u./h]

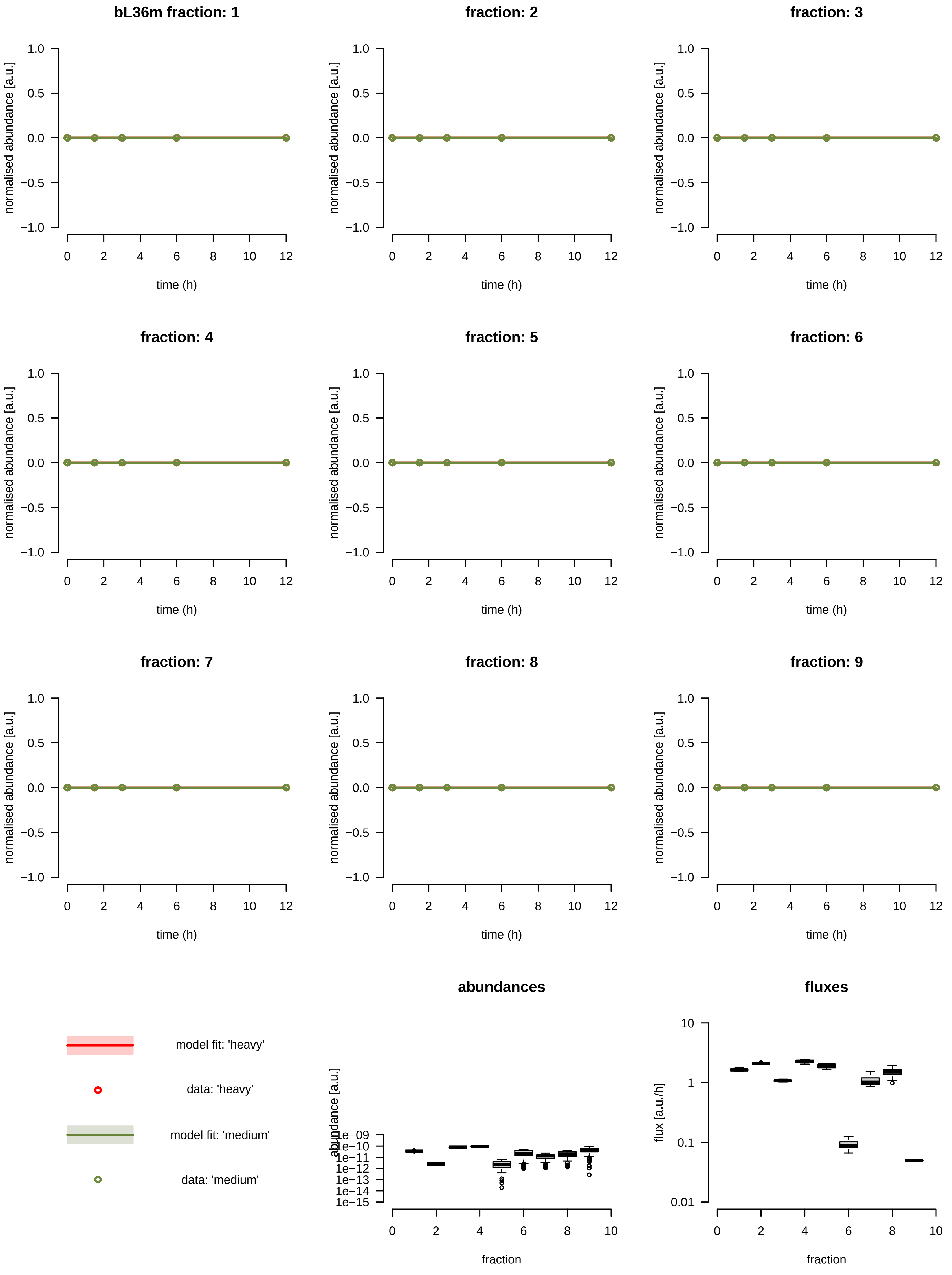

bL36m fraction: 1
fraction: 2
fraction: 3
fraction: 4
fraction: 5
fraction: 6
fraction: 7
fraction: 8
fraction: 9
normalised abundance [a.u.]
1.0
0.5
0.0
−0.5
−1.0
0
2
4
6
8
10
12
time (h)
model fit: 'heavy'
data: 'heavy'
model fit: 'medium'
data: 'medium'
abundances
abundance [a.u.]
1e−09
1e−10
1e−11
1e−12
1e−13
1e−14
1e−15
fraction
fluxes
flux [a.u./h]
10
1
0.1
0.01
fraction

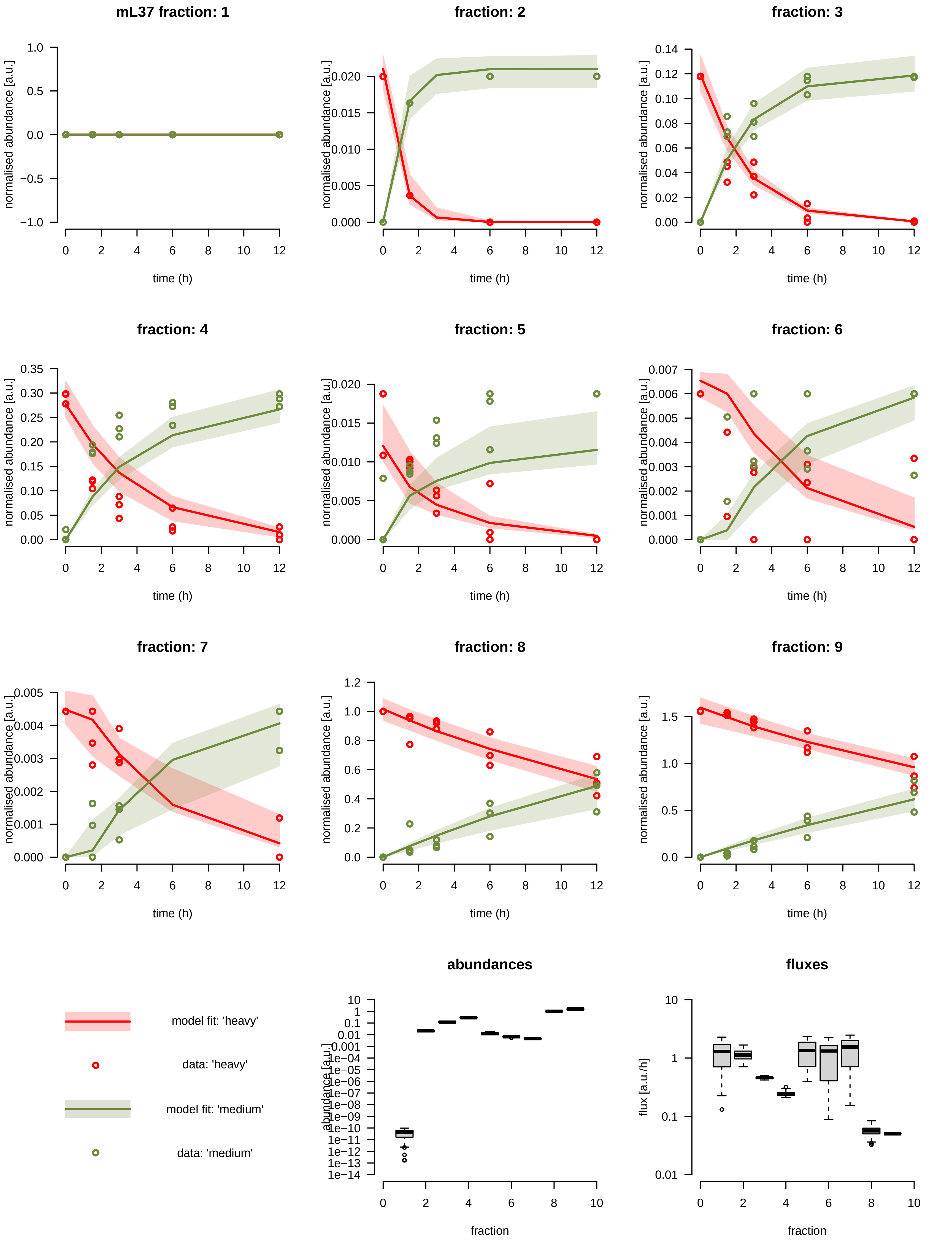
mL37 fraction: 1
fraction: 2
fraction: 3
fraction: 4
fraction: 5
fraction: 6
fraction: 7
fraction: 8
fraction: 9
normalised abundance [a.u.]
time (h)
model fit: 'heavy'
data: 'heavy'
model fit: 'medium'
data: 'medium'
abundances
abundance [a.u.]
fraction
fluxes
flux [a.u./h]
fraction

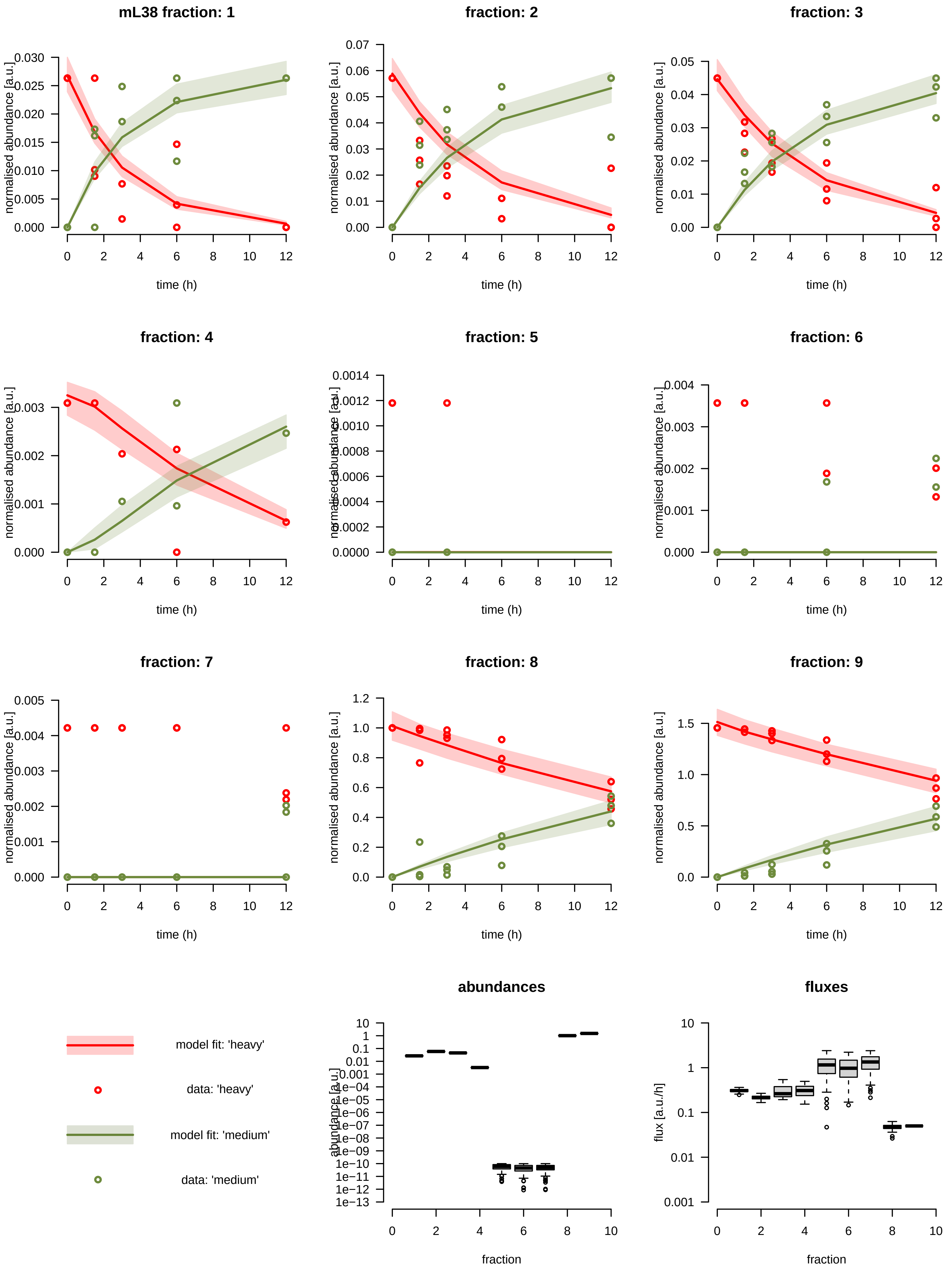
mL38 fraction: 1
fraction: 2
fraction: 3
fraction: 4
fraction: 5
fraction: 6
fraction: 7
fraction: 8
fraction: 9
normalised abundance [a.u.]
time (h)
model fit: 'heavy'
data: 'heavy'
model fit: 'medium'
data: 'medium'
abundances
abundance [a.u.]
fraction
fluxes
flux [a.u./h]
fraction

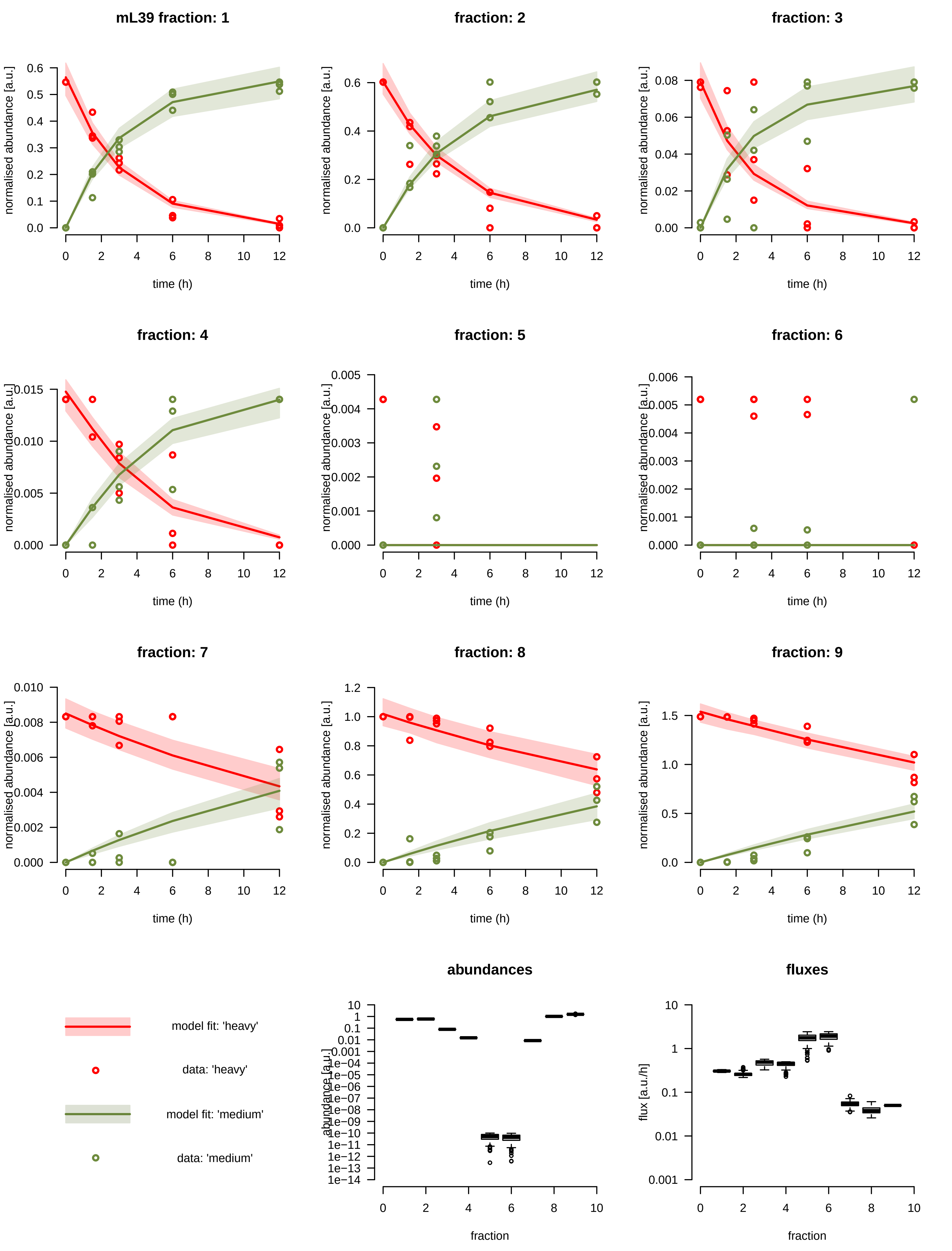

mL39 fraction: 1
fraction: 2
fraction: 3
fraction: 4
fraction: 5
fraction: 6
fraction: 7
fraction: 8
fraction: 9
normalised abundance [a.u.]
time (h)
model fit: 'heavy'
data: 'heavy'
model fit: 'medium'
data: 'medium'
abundances
abundance [a.u.]
fraction
fluxes
flux [a.u./h]

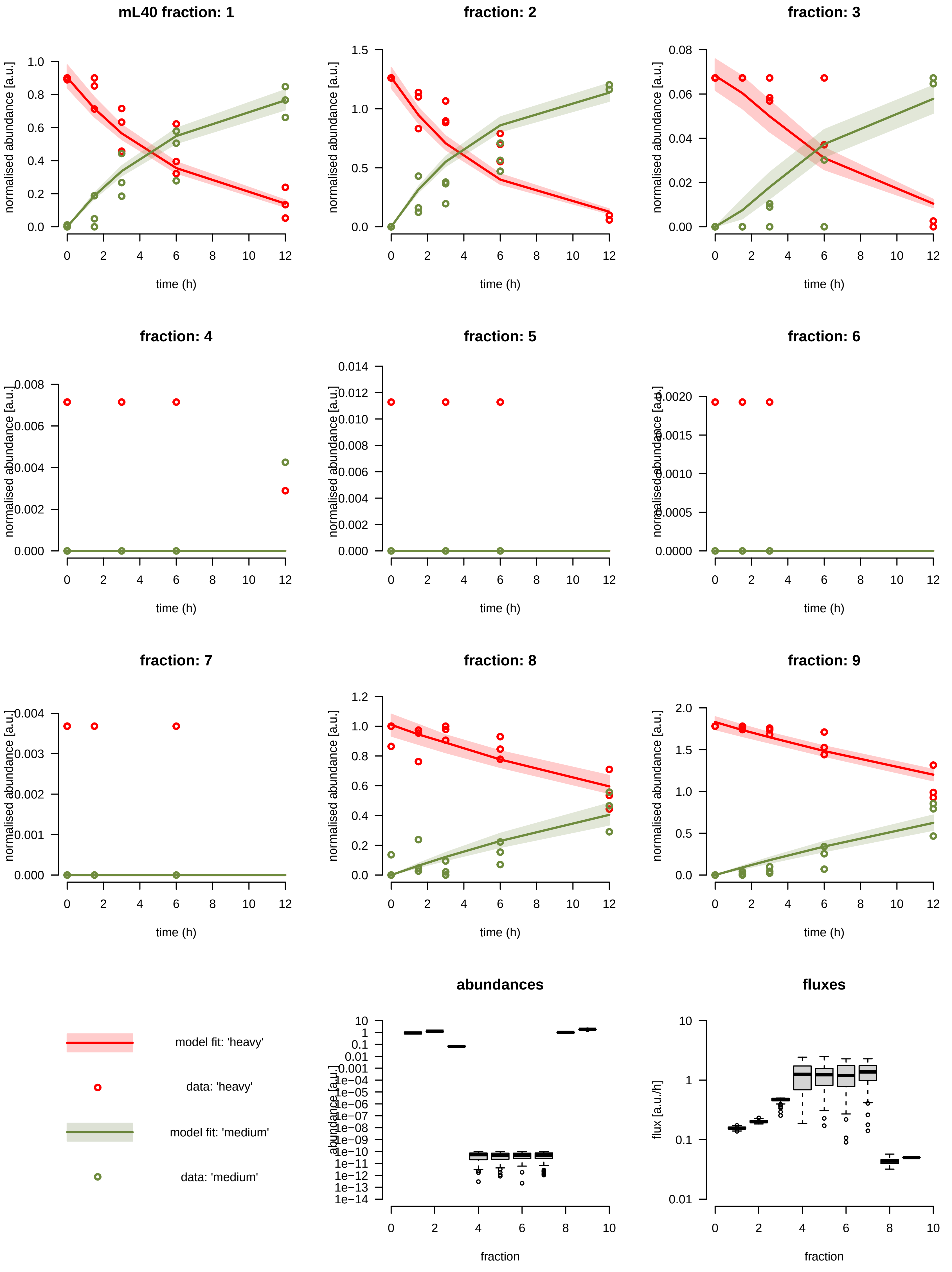
mL40 fraction: 1
fraction: 2
fraction: 3
fraction: 4
fraction: 5
fraction: 6
fraction: 7
fraction: 8
fraction: 9
normalised abundance [a.u.]
time (h)
model fit: 'heavy'
data: 'heavy'
model fit: 'medium'
data: 'medium'
abundances
abundance [a.u.]
fraction
fluxes
flux [a.u./h]
fraction

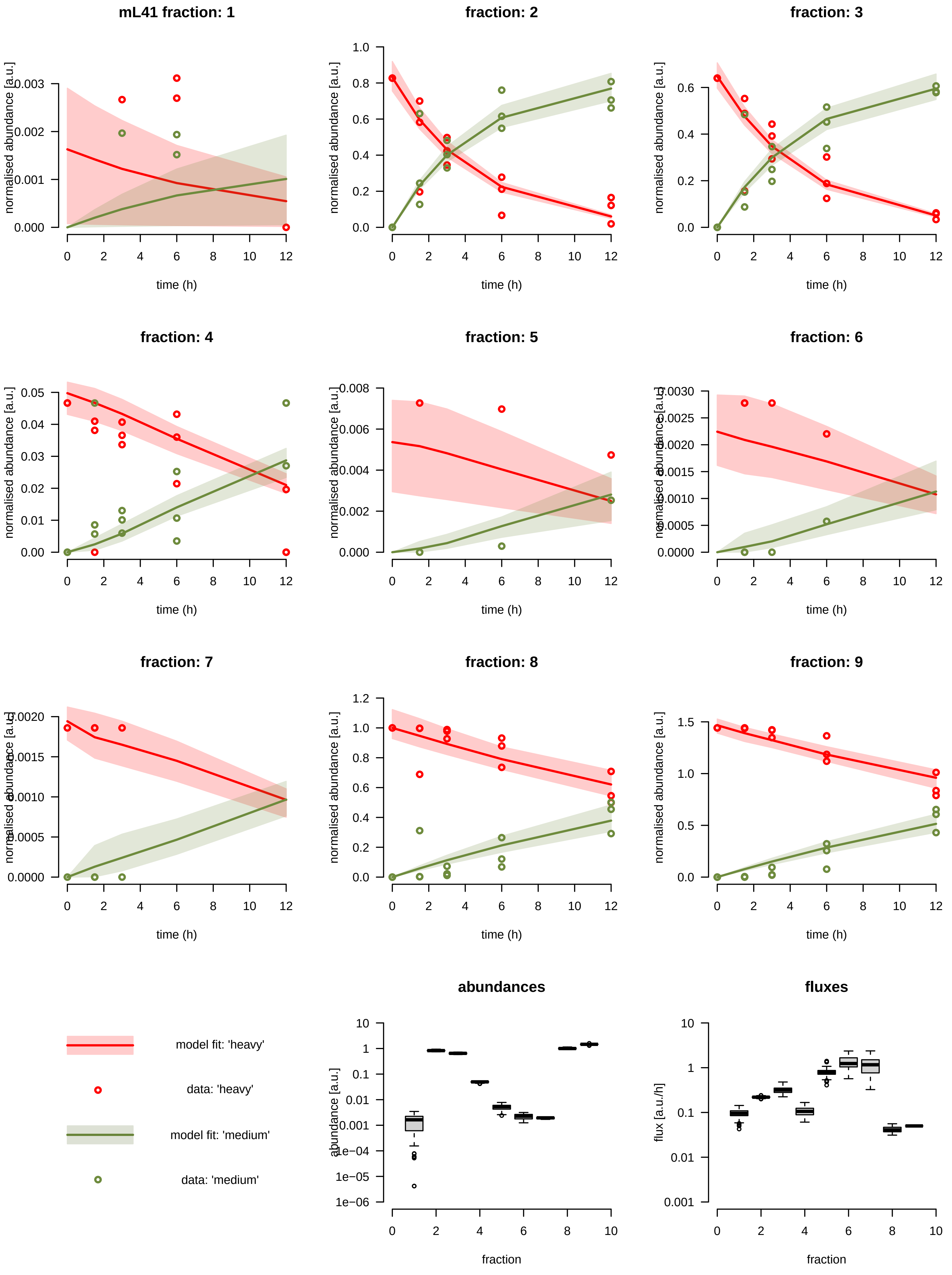
mL41 fraction: 1
fraction: 2
fraction: 3
fraction: 4
fraction: 5
fraction: 6
fraction: 7
fraction: 8
fraction: 9
normalised abundance [a.u.]
time (h)
model fit: 'heavy'
data: 'heavy'
model fit: 'medium'
data: 'medium'
abundances
abundance [a.u.]
fraction
fluxes
flux [a.u./h]
fraction

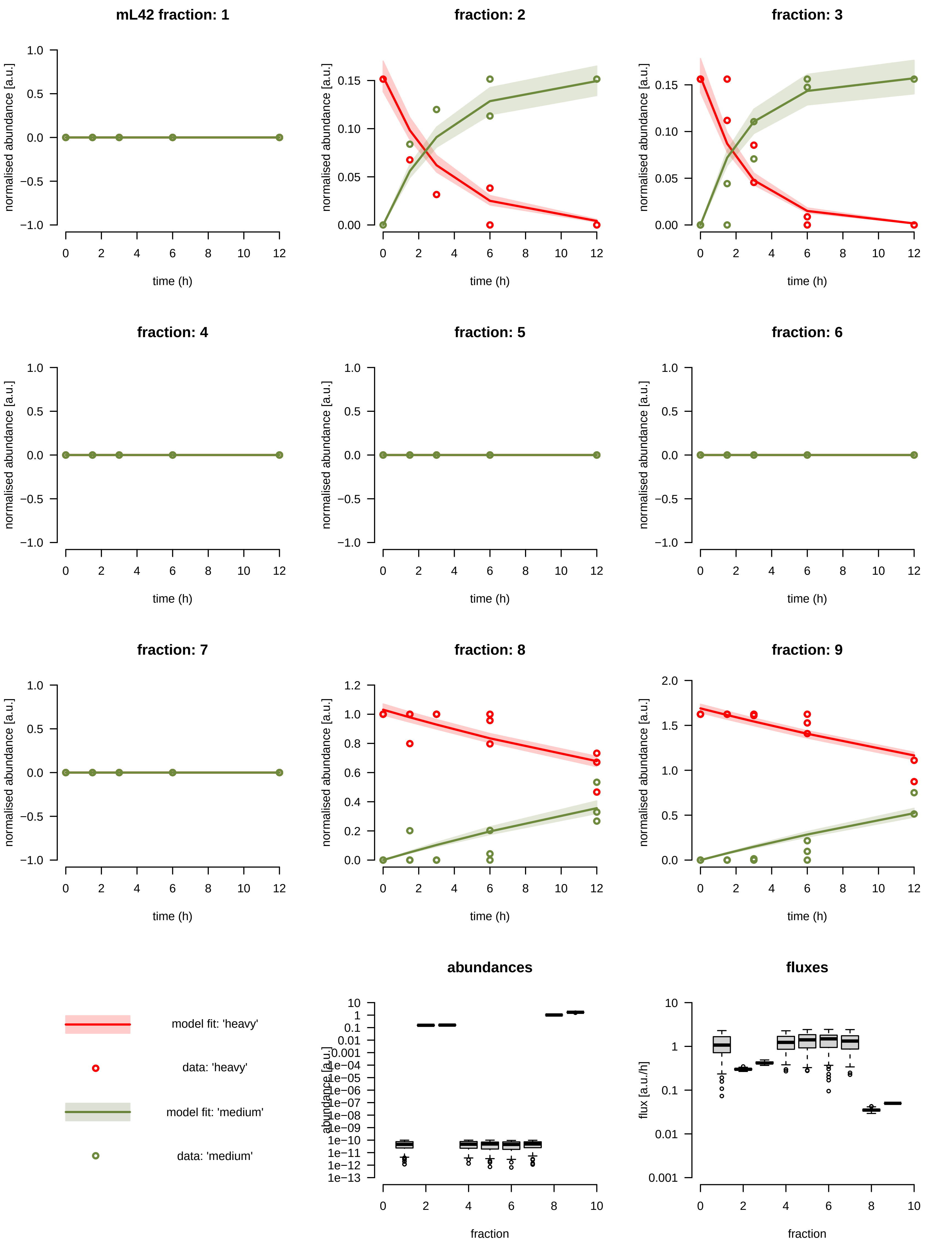
mL42 fraction: 1
fraction: 2
fraction: 3
fraction: 4
fraction: 5
fraction: 6
fraction: 7
fraction: 8
fraction: 9
normalised abundance [a.u.]
time (h)
model fit: 'heavy'
data: 'heavy'
model fit: 'medium'
data: 'medium'
abundances
abundance [a.u.]
fraction
fluxes
flux [a.u./h]
fraction

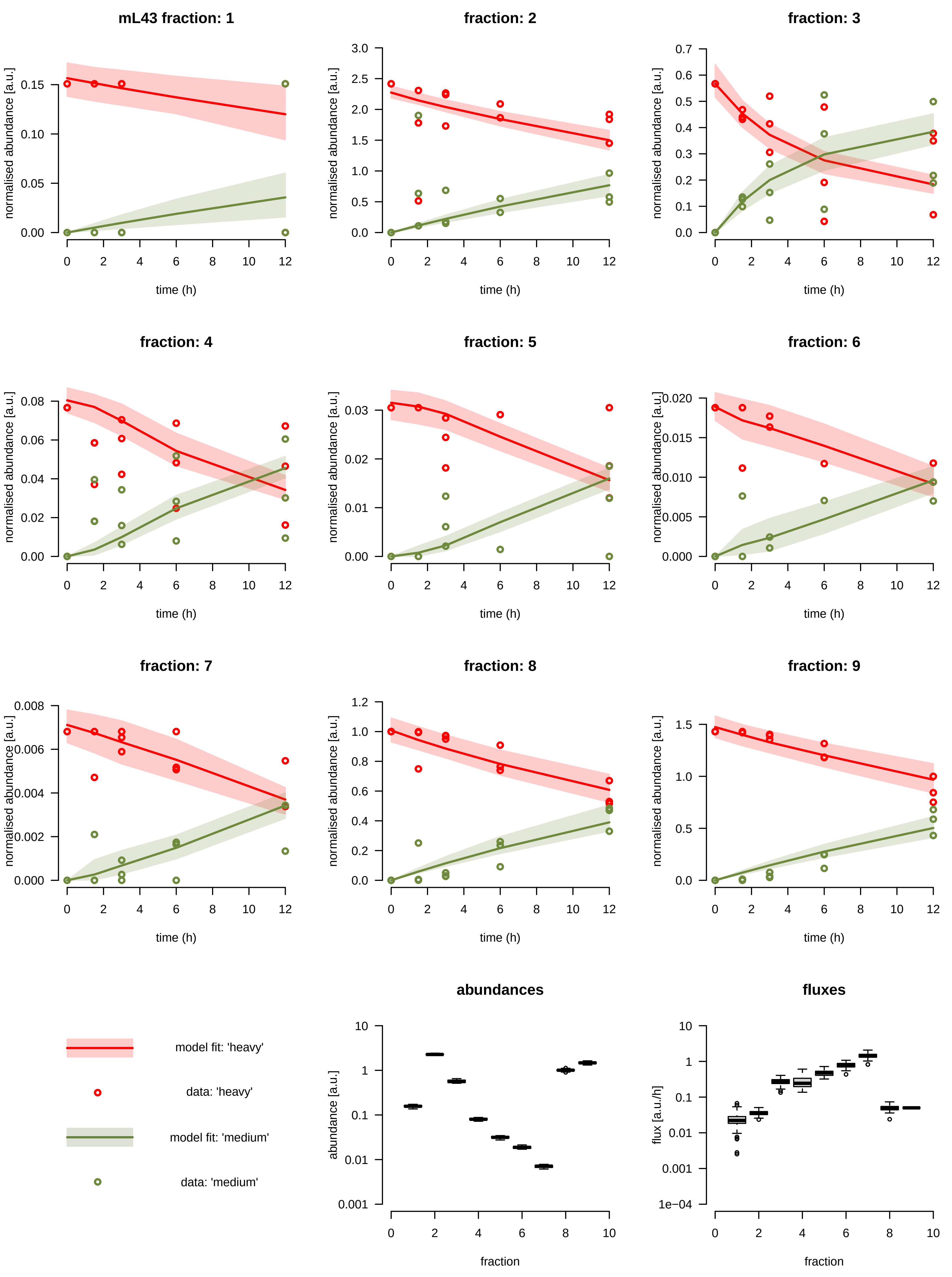
mL43 fraction: 1
fraction: 2
fraction: 3
fraction: 4
fraction: 5
fraction: 6
fraction: 7
fraction: 8
fraction: 9
normalised abundance [a.u.]
time (h)
model fit: 'heavy'
data: 'heavy'
model fit: 'medium'
data: 'medium'
abundances
abundance [a.u.]
fraction
fluxes
flux [a.u./h]
fraction

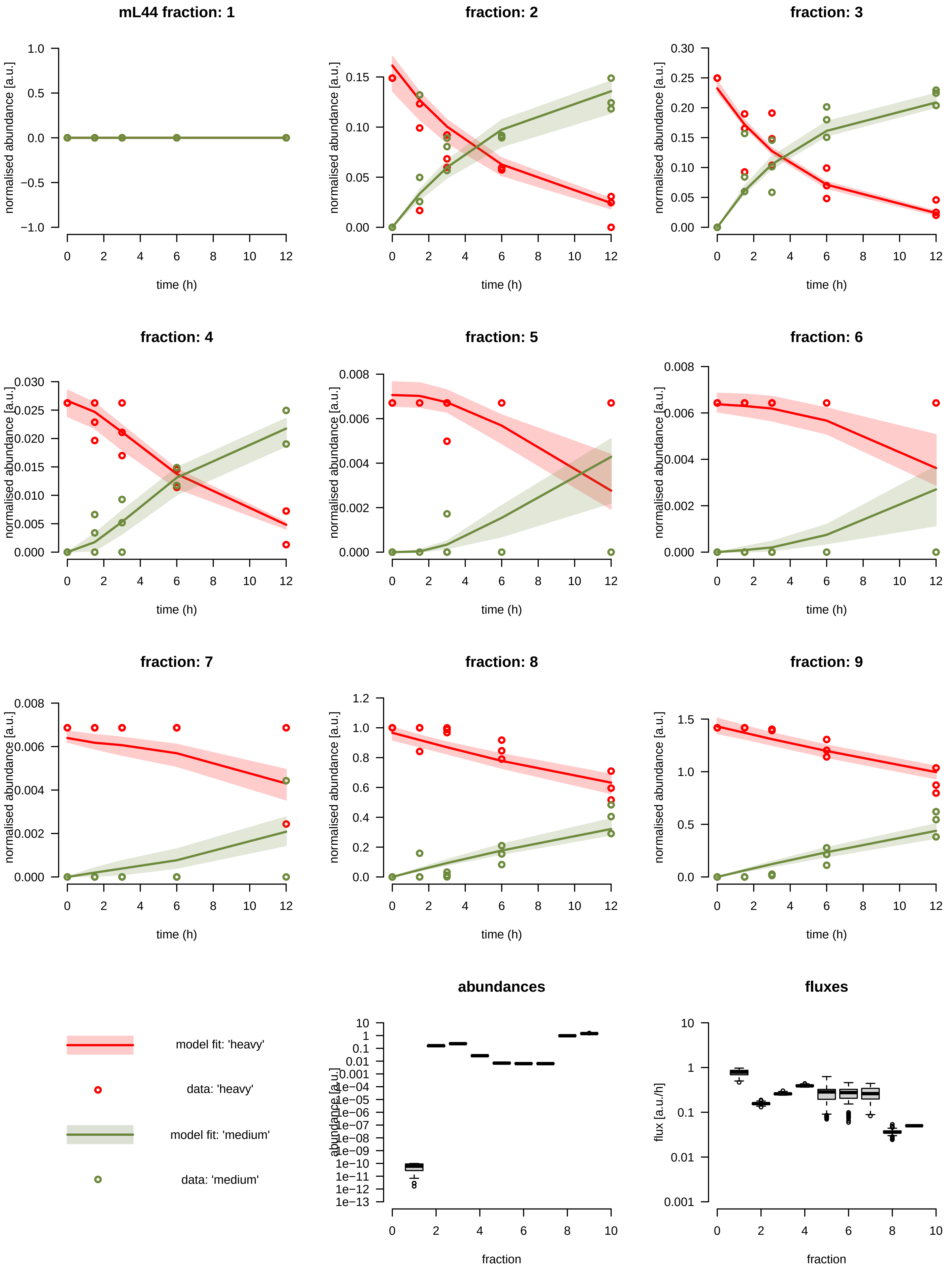
mL44 fraction: 1
fraction: 2
fraction: 3
fraction: 4
fraction: 5
fraction: 6
fraction: 7
fraction: 8
fraction: 9
normalised abundance [a.u.]
time (h)
model fit: 'heavy'
data: 'heavy'
model fit: 'medium'
data: 'medium'
abundances
abundance [a.u.]
fraction
fluxes
flux [a.u./h]
fraction

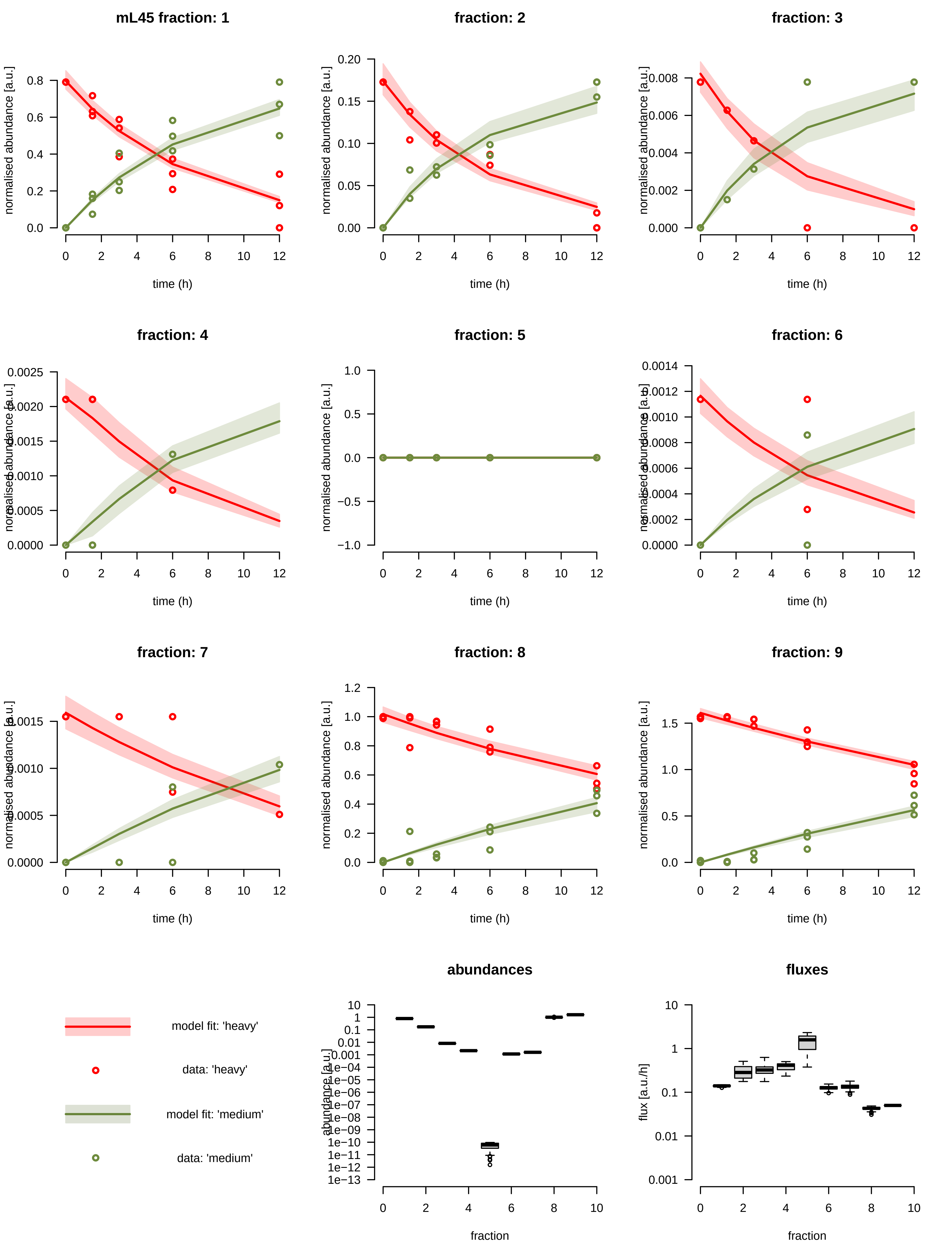

mL45 fraction: 1
fraction: 2
fraction: 3
fraction: 4
fraction: 5
fraction: 6
fraction: 7
fraction: 8
fraction: 9
abundances
fluxes
normalised abundance [a.u.]
time (h)
model fit: 'heavy'
data: 'heavy'
model fit: 'medium'
data: 'medium'
abundance [a.u.]
flux [a.u./h]
fraction

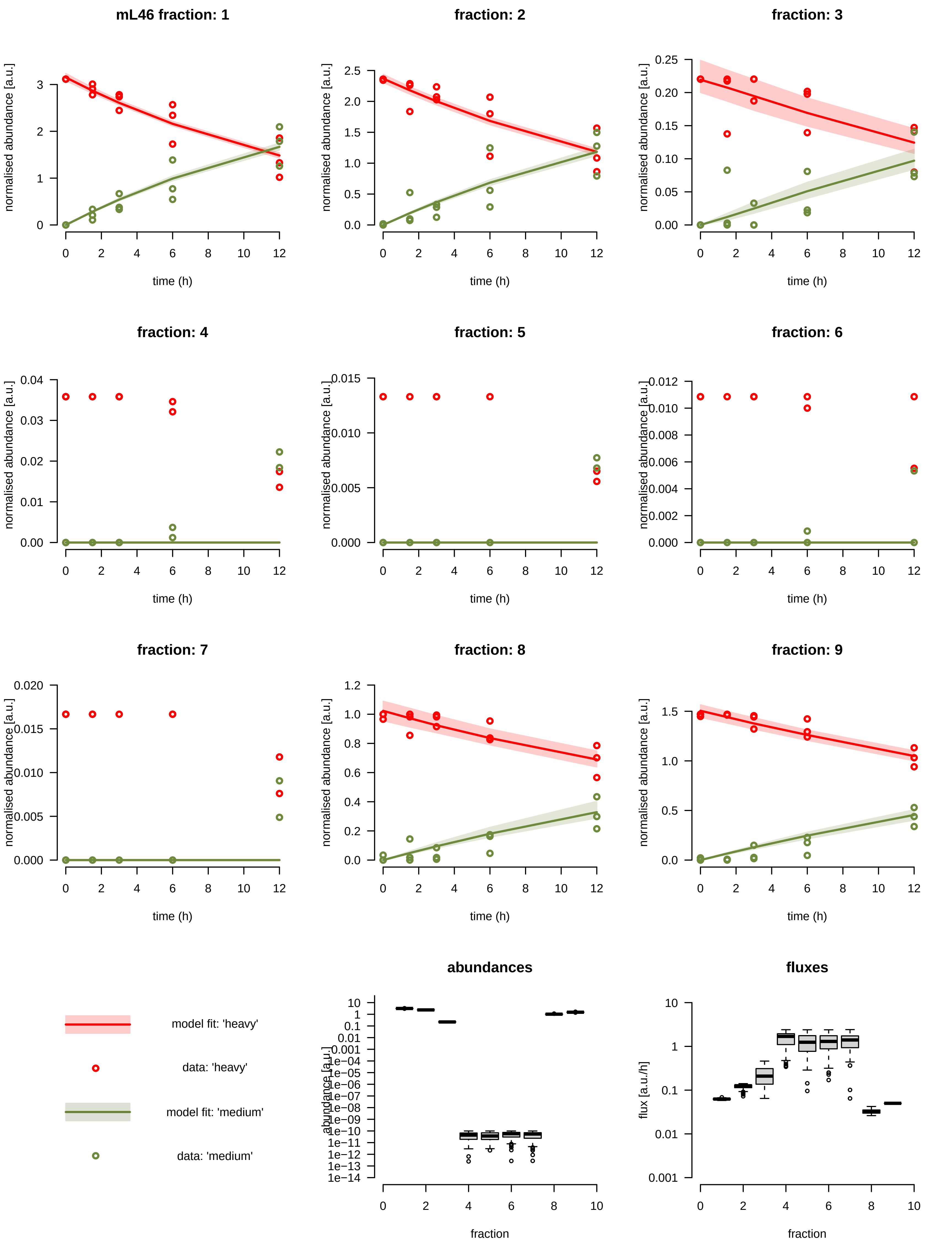

mL46 fraction: 1
fraction: 2
fraction: 3
fraction: 4
fraction: 5
fraction: 6
fraction: 7
fraction: 8
fraction: 9
normalised abundance [a.u.]
time (h)
model fit: 'heavy'
data: 'heavy'
model fit: 'medium'
data: 'medium'
abundances
abundance [a.u.]
fraction
fluxes
flux [a.u./h]
fraction

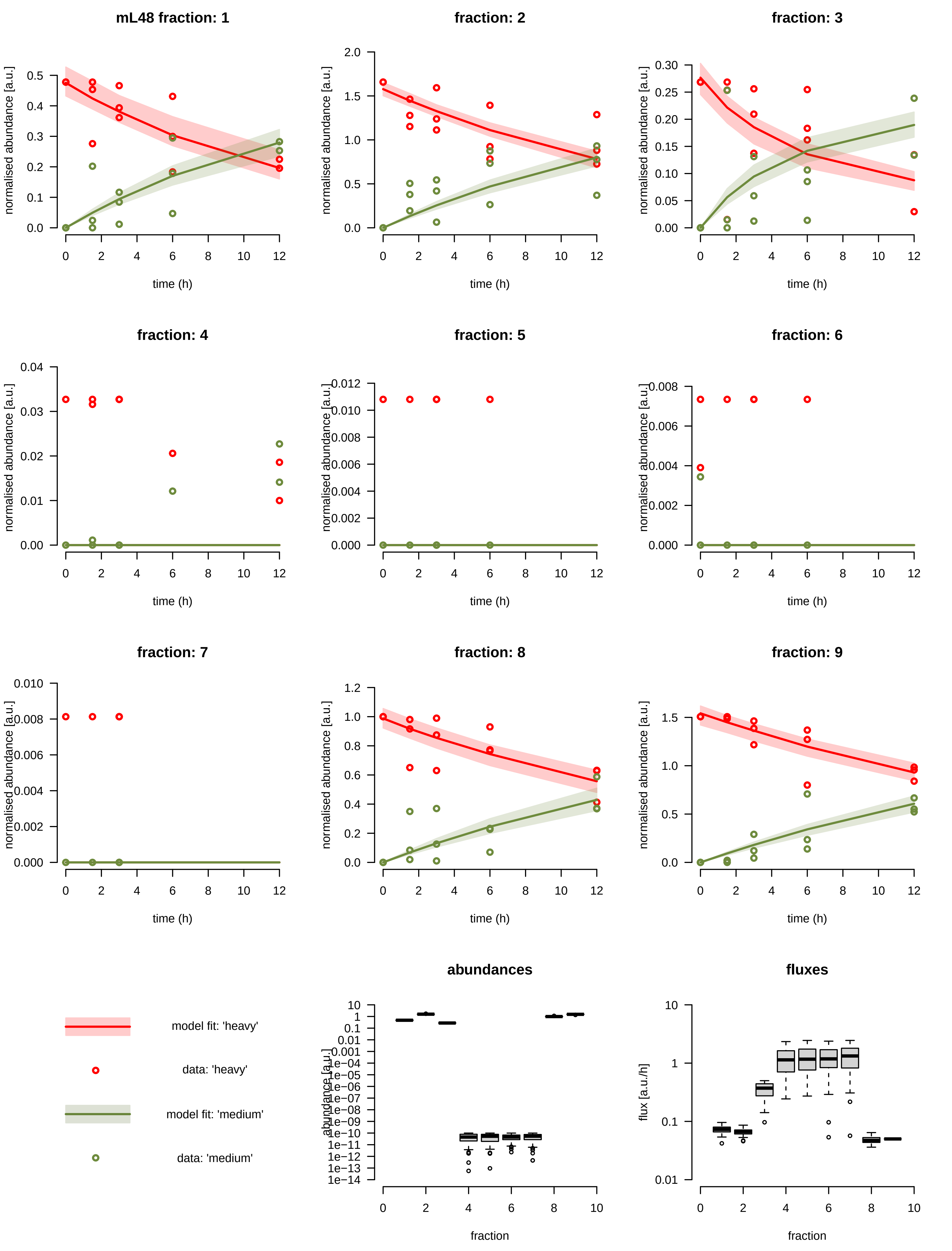

mL48 fraction: 1
fraction: 2
fraction: 3
fraction: 4
fraction: 5
fraction: 6
fraction: 7
fraction: 8
fraction: 9
normalised abundance [a.u.]
time (h)
model fit: 'heavy'
data: 'heavy'
model fit: 'medium'
data: 'medium'
abundances
abundance [a.u.]
fraction
fluxes
flux [a.u./h]
fraction

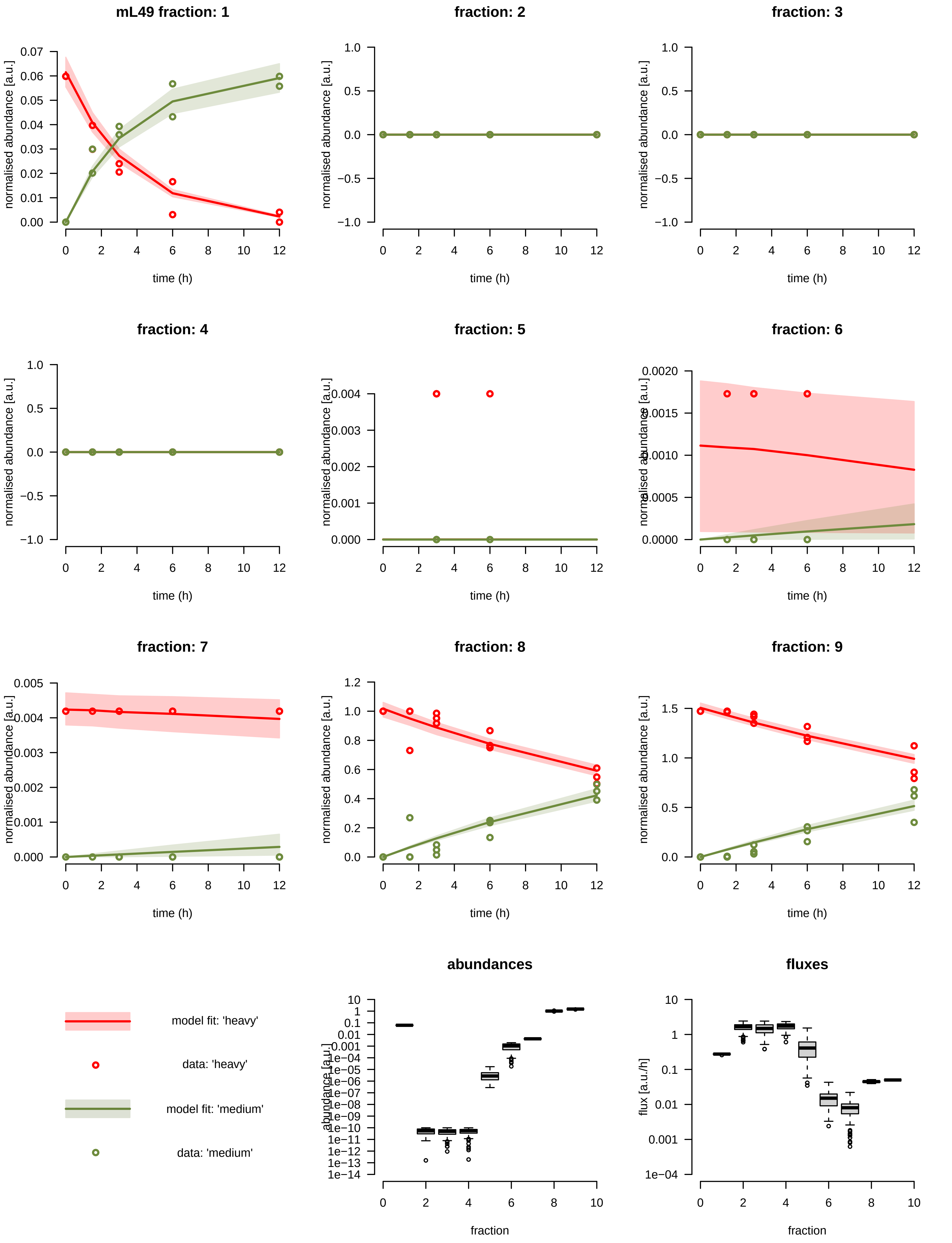

mL49 fraction: 1
fraction: 2
fraction: 3
fraction: 4
fraction: 5
fraction: 6
fraction: 7
fraction: 8
fraction: 9
normalised abundance [a.u.]
time (h)
model fit: 'heavy'
data: 'heavy'
model fit: 'medium'
data: 'medium'
abundances
abundance [a.u.]
fraction
fluxes
flux [a.u./h]
fraction

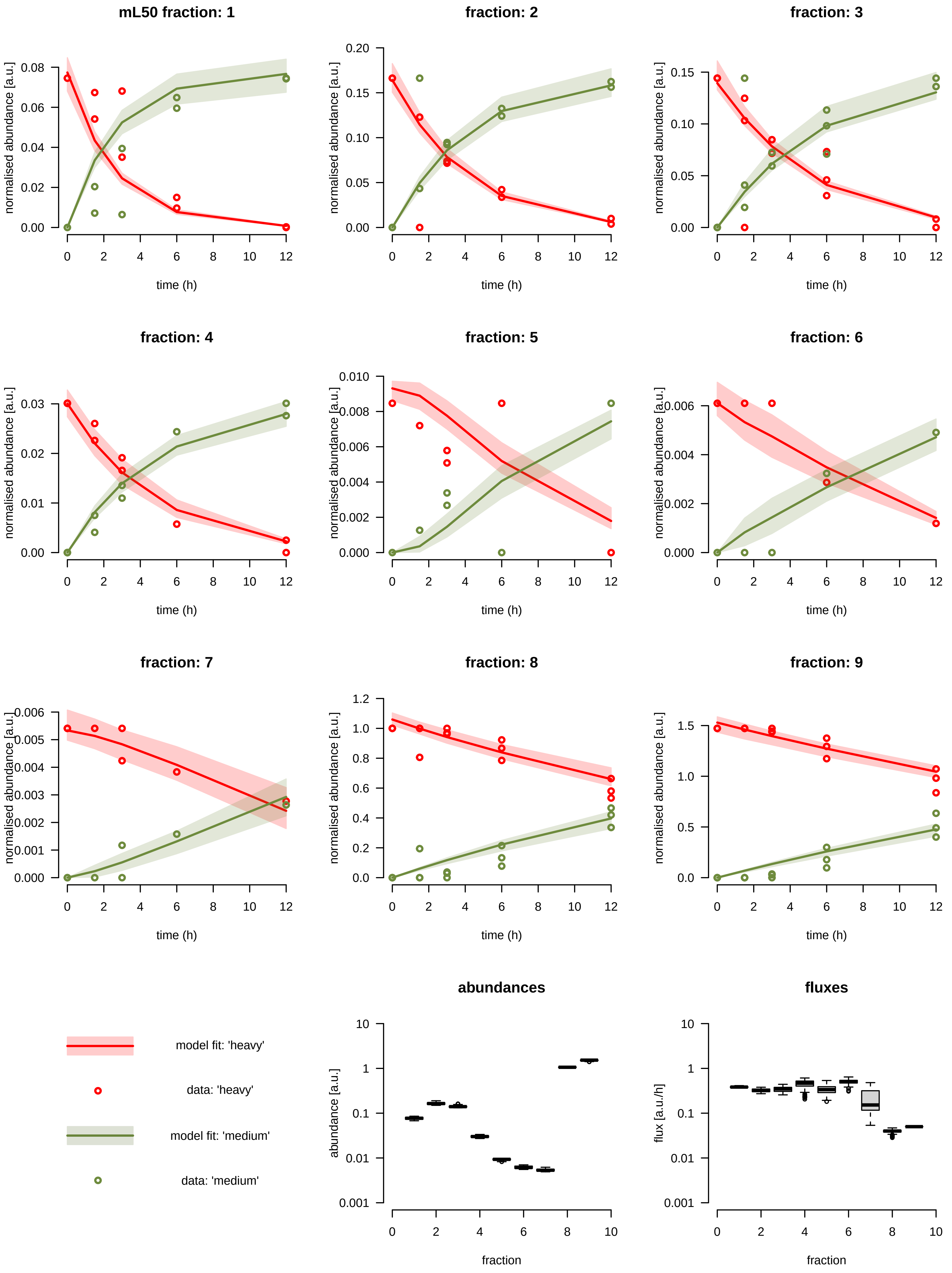

mL50 fraction: 1
fraction: 2
fraction: 3
fraction: 4
fraction: 5
fraction: 6
fraction: 7
fraction: 8
fraction: 9
normalised abundance [a.u.]
time (h)
model fit: 'heavy'
data: 'heavy'
model fit: 'medium'
data: 'medium'
abundances
abundance [a.u.]
fraction
fluxes
flux [a.u./h]
fraction

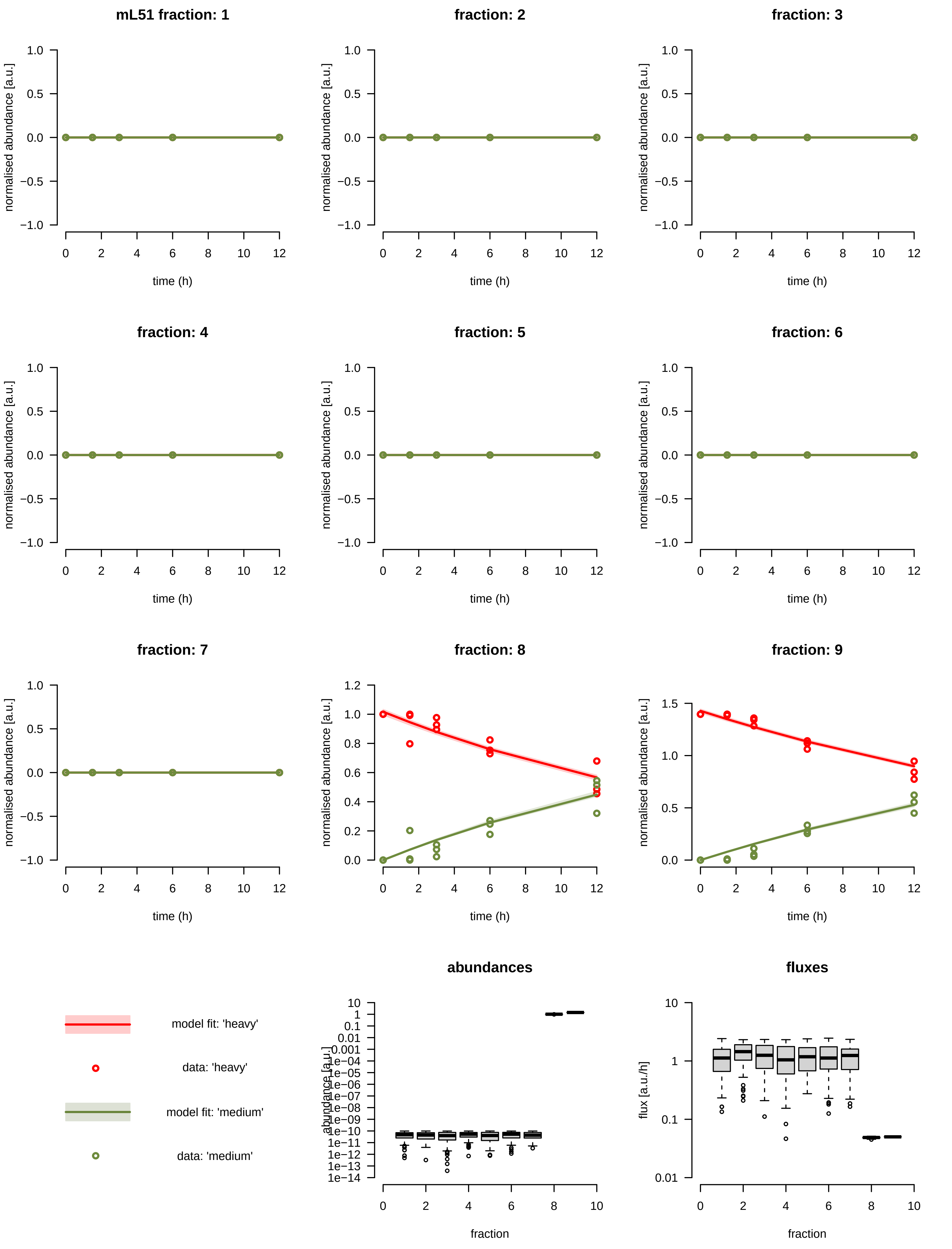
mL51 fraction: 1
fraction: 2
fraction: 3
fraction: 4
fraction: 5
fraction: 6
fraction: 7
fraction: 8
fraction: 9
normalised abundance [a.u.]
time (h)
model fit: 'heavy'
data: 'heavy'
model fit: 'medium'
data: 'medium'
abundances
abundance [a.u.]
fraction
fluxes
flux [a.u./h]
fraction

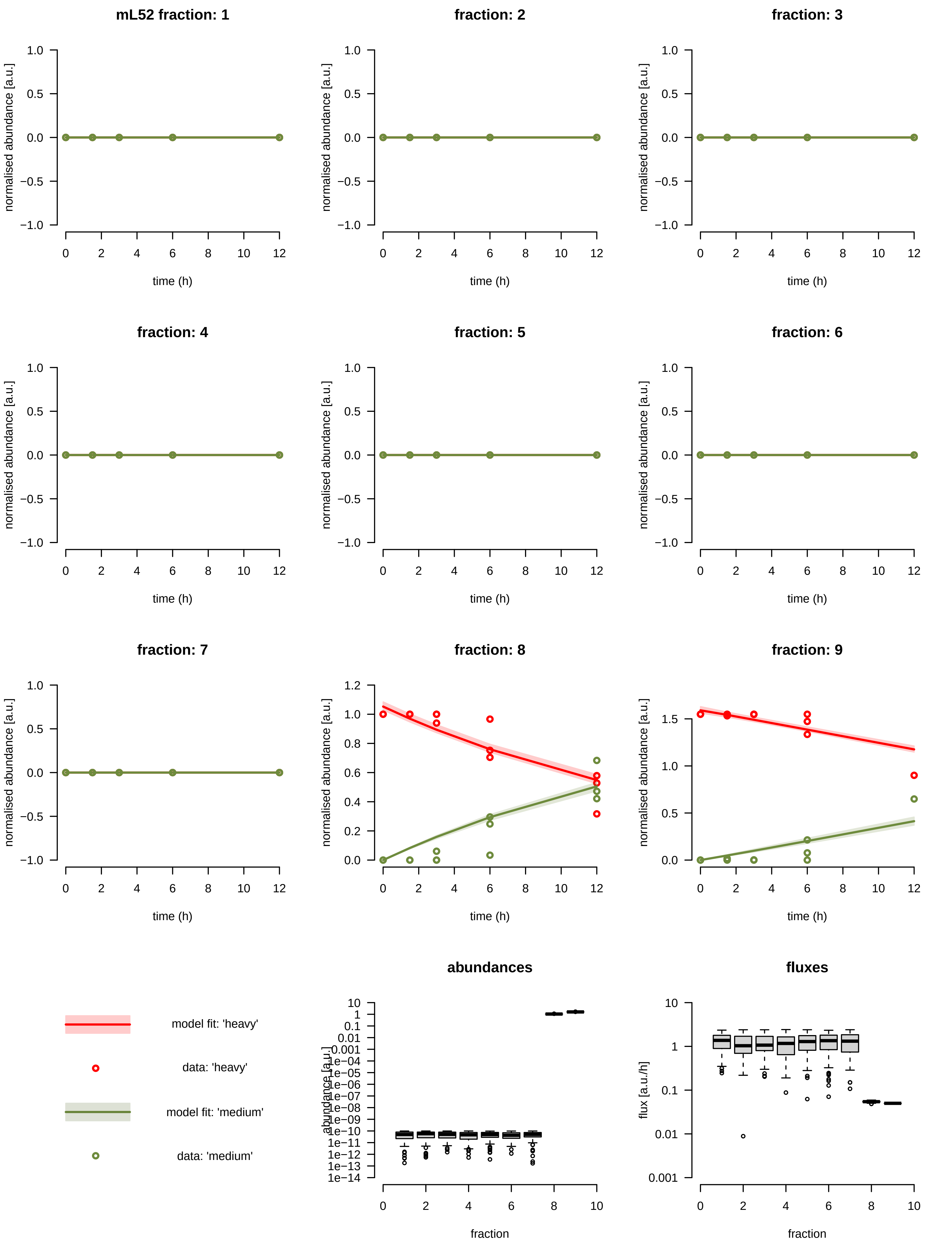
mL52 fraction: 1
fraction: 2
fraction: 3
fraction: 4
fraction: 5
fraction: 6
fraction: 7
fraction: 8
fraction: 9
normalised abundance [a.u.]
time (h)
model fit: 'heavy'
data: 'heavy'
model fit: 'medium'
data: 'medium'
abundances
abundance [a.u.]
fraction
fluxes
flux [a.u./h]
fraction

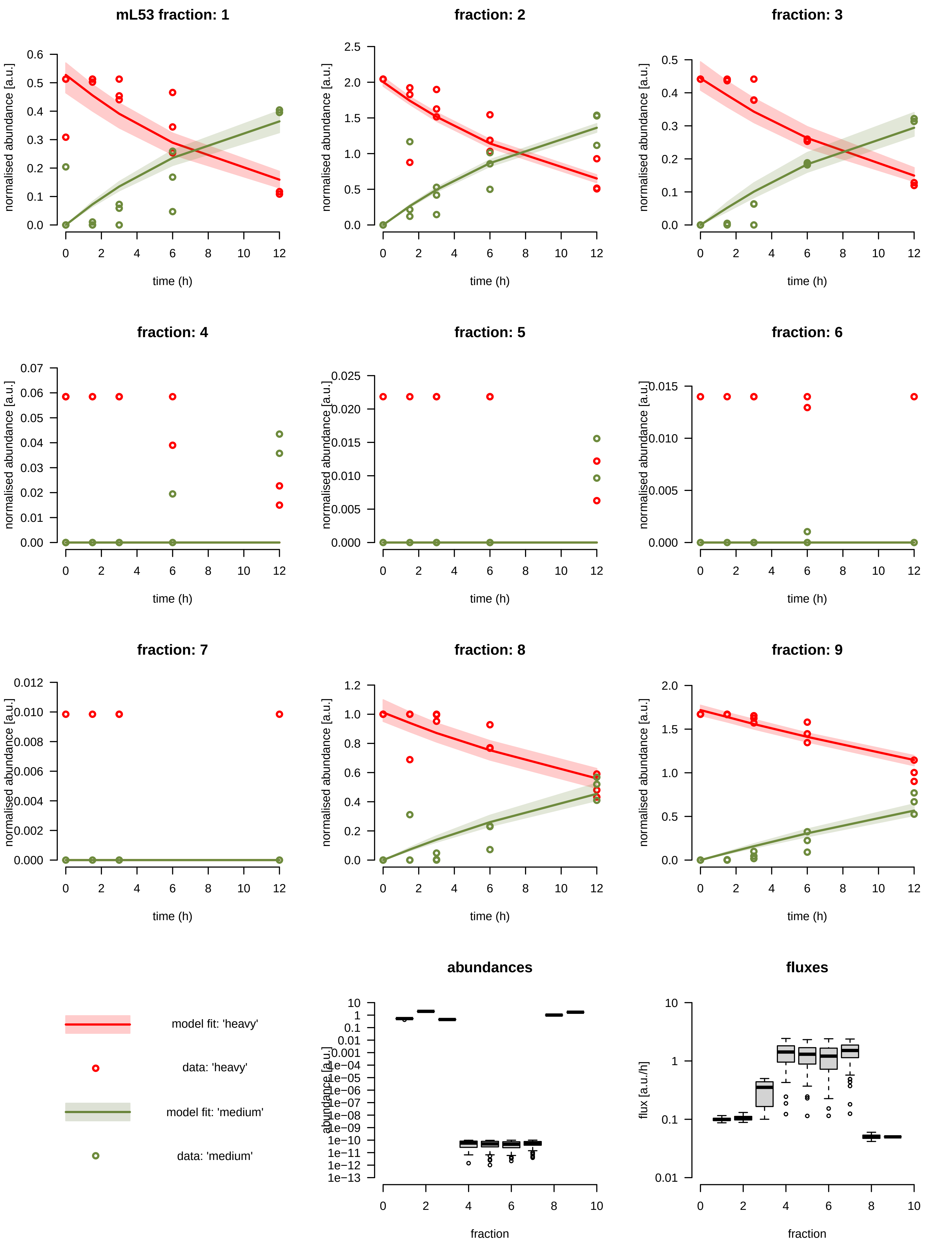
mL53 fraction: 1
fraction: 2
fraction: 3
fraction: 4
fraction: 5
fraction: 6
fraction: 7
fraction: 8
fraction: 9
abundances
fluxes
normalised abundance [a.u.]
time (h)
abundance [a.u.]
flux [a.u./h]
fraction
model fit: 'heavy'
data: 'heavy'
model fit: 'medium'
data: 'medium'

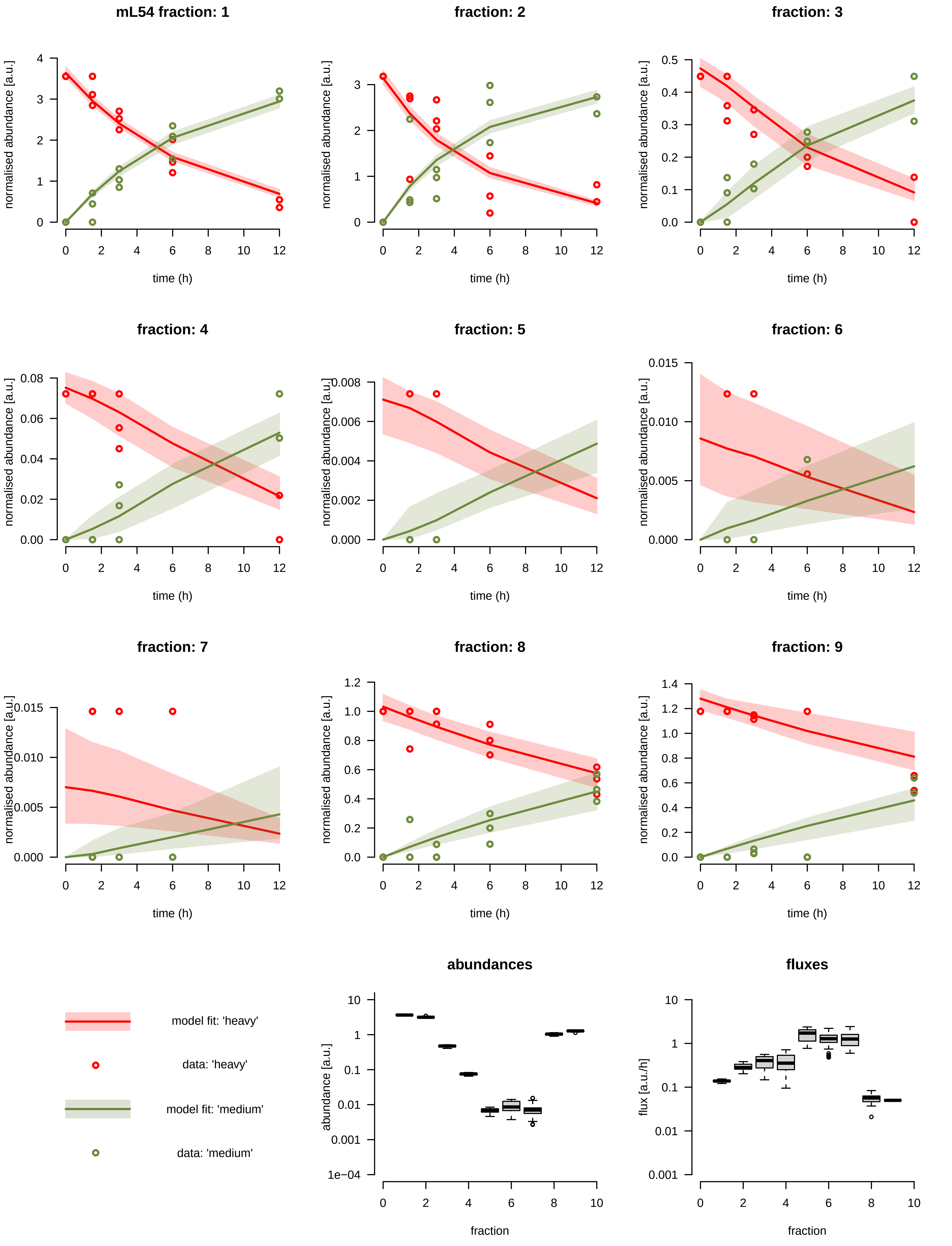
mL54 fraction: 1
fraction: 2
fraction: 3
fraction: 4
fraction: 5
fraction: 6
fraction: 7
fraction: 8
fraction: 9
normalised abundance [a.u.]
time (h)
model fit: 'heavy'
data: 'heavy'
model fit: 'medium'
data: 'medium'
abundances
abundance [a.u.]
fraction
fluxes
flux [a.u./h]
fraction

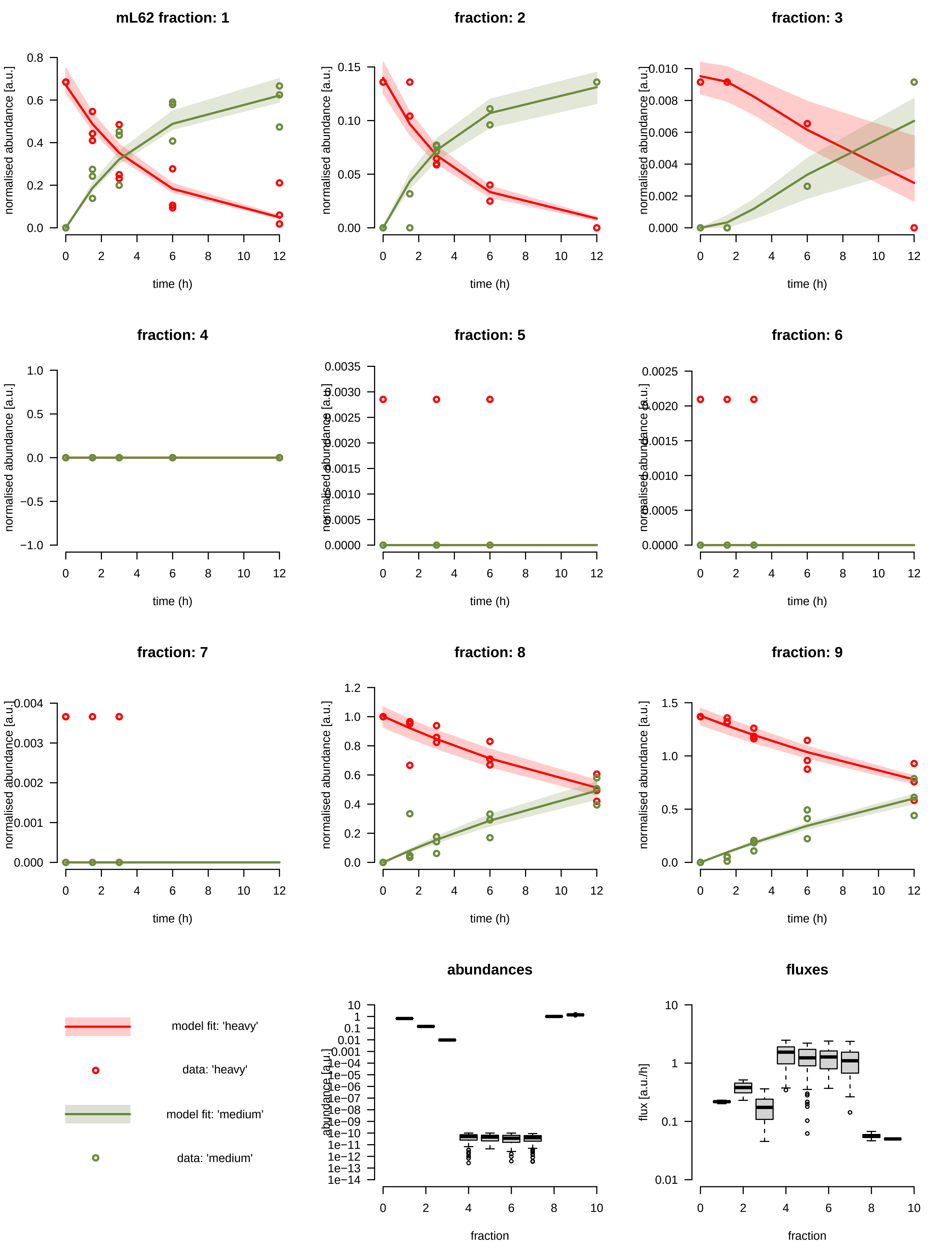
mL62 fraction: 1
fraction: 2
fraction: 3
fraction: 4
fraction: 5
fraction: 6
fraction: 7
fraction: 8
fraction: 9
normalised abundance [a.u.]
time (h)
model fit: 'heavy'
data: 'heavy'
model fit: 'medium'
data: 'medium'
abundances
abundance [a.u.]
fraction
fluxes
flux [a.u./h]
fraction

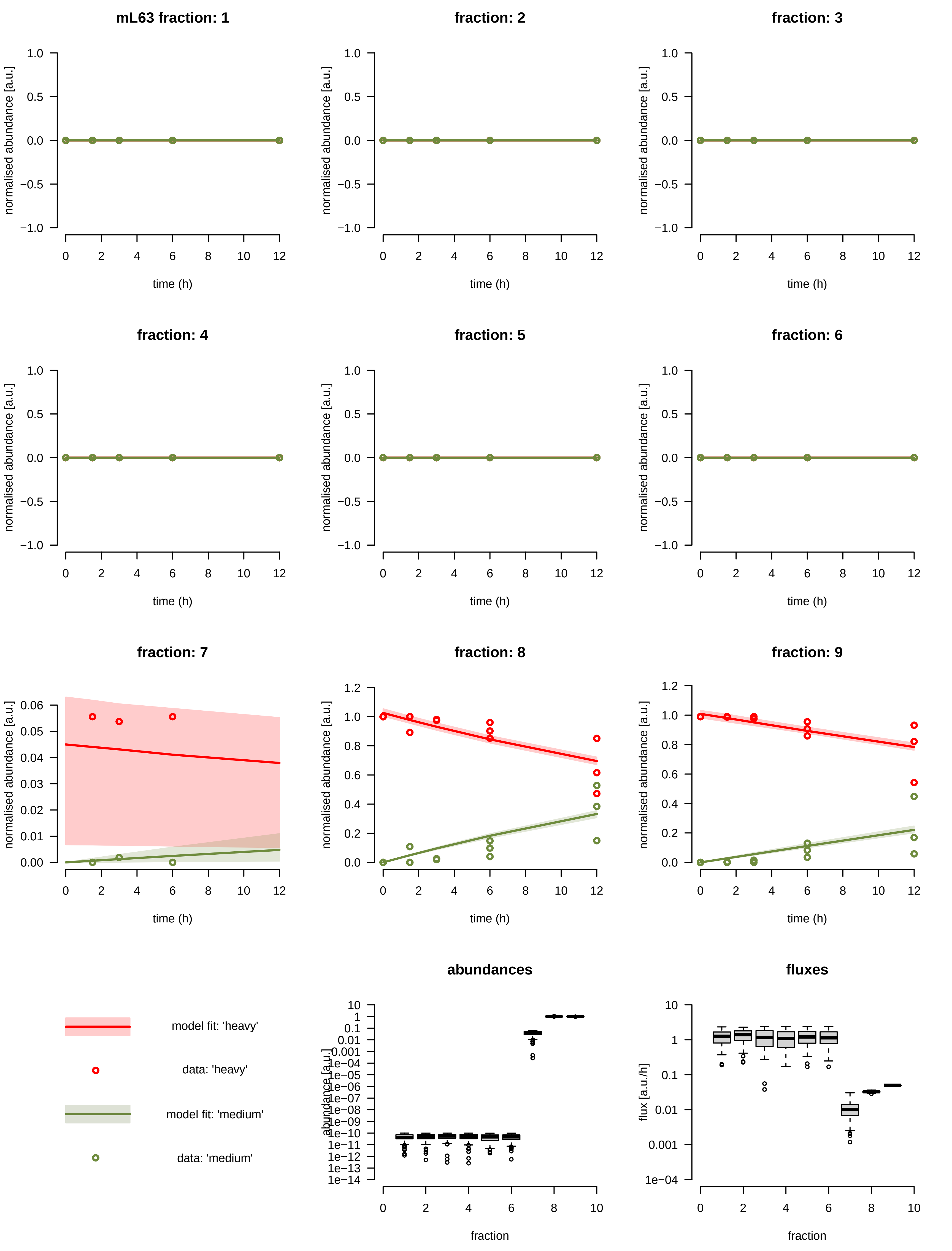

mL63 fraction: 1
fraction: 2
fraction: 3
fraction: 4
fraction: 5
fraction: 6
fraction: 7
fraction: 8
fraction: 9
normalised abundance [a.u.]
time (h)
model fit: 'heavy'
data: 'heavy'
model fit: 'medium'
data: 'medium'
abundances
abundance [a.u.]
fraction
fluxes
flux [a.u./h]
fraction

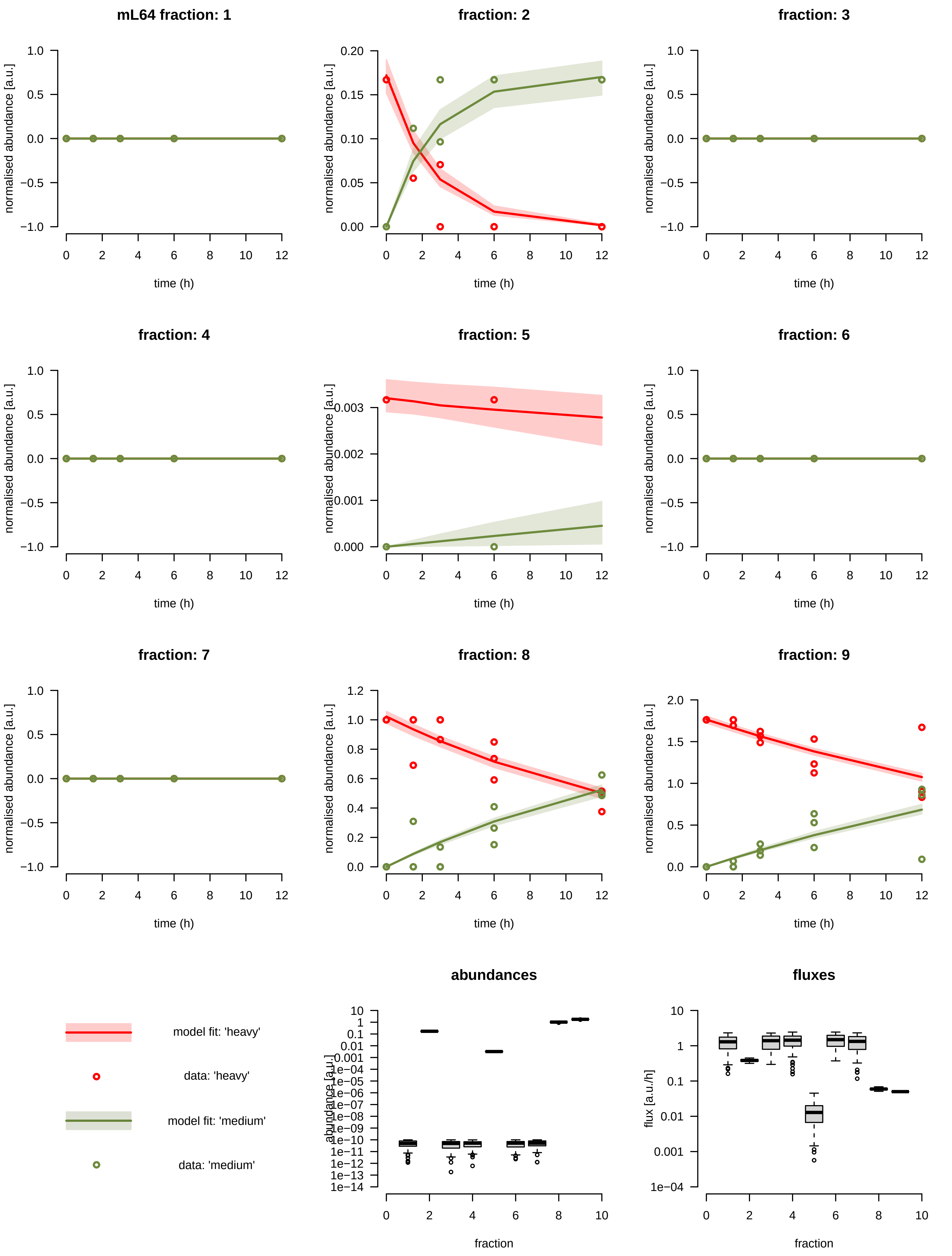
mL64 fraction: 1
fraction: 2
fraction: 3
fraction: 4
fraction: 5
fraction: 6
fraction: 7
fraction: 8
fraction: 9
normalised abundance [a.u.]
time (h)
model fit: 'heavy'
data: 'heavy'
model fit: 'medium'
data: 'medium'
abundances
abundance [a.u.]
fraction
fluxes
flux [a.u./h]
fraction

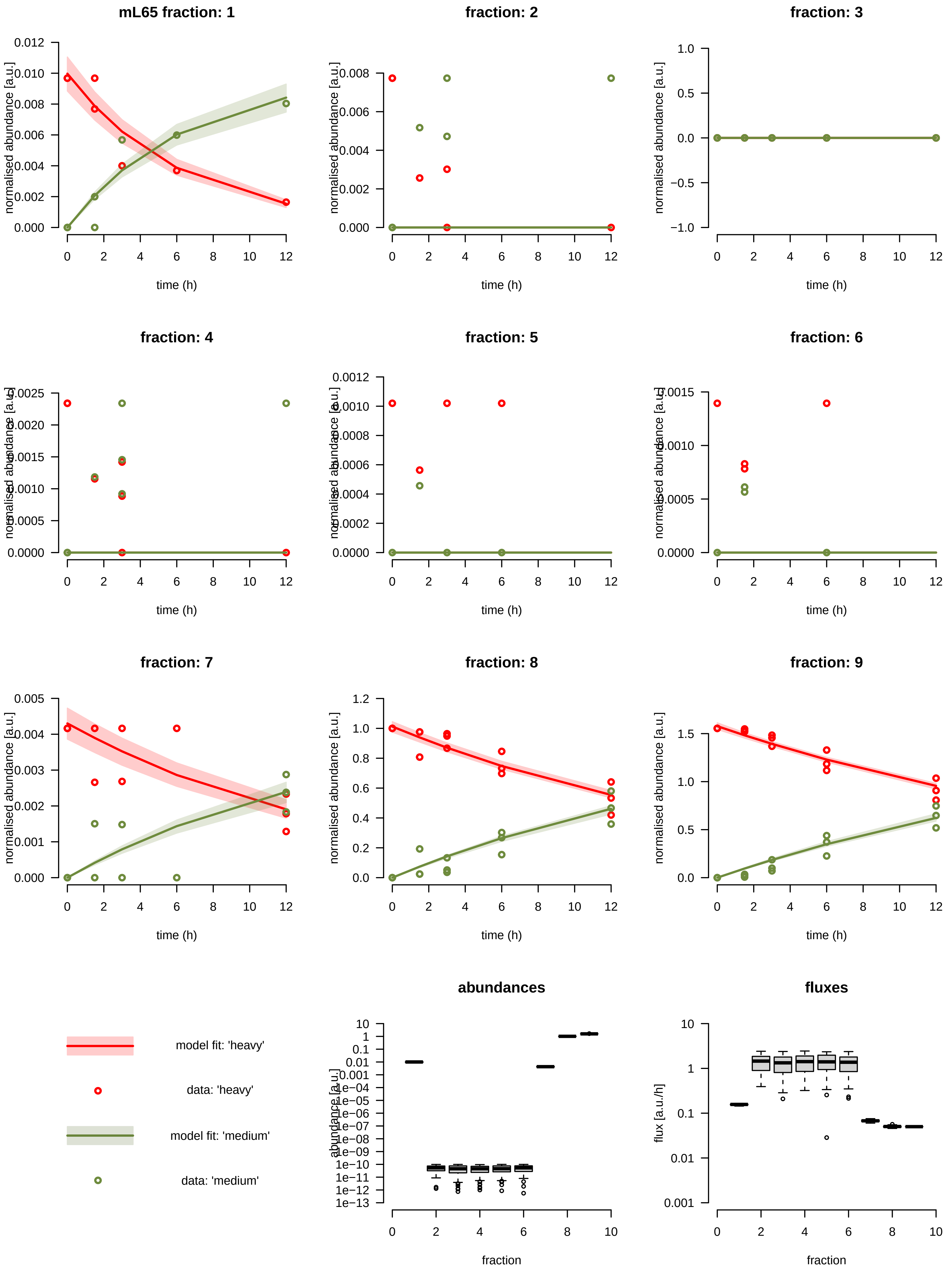
mL65 fraction: 1
fraction: 2
fraction: 3
fraction: 4
fraction: 5
fraction: 6
fraction: 7
fraction: 8
fraction: 9
normalised abundance [a.u.]
time (h)
model fit: 'heavy'
data: 'heavy'
model fit: 'medium'
data: 'medium'
abundances
abundance [a.u.]
fraction
fluxes
flux [a.u./h]
fraction

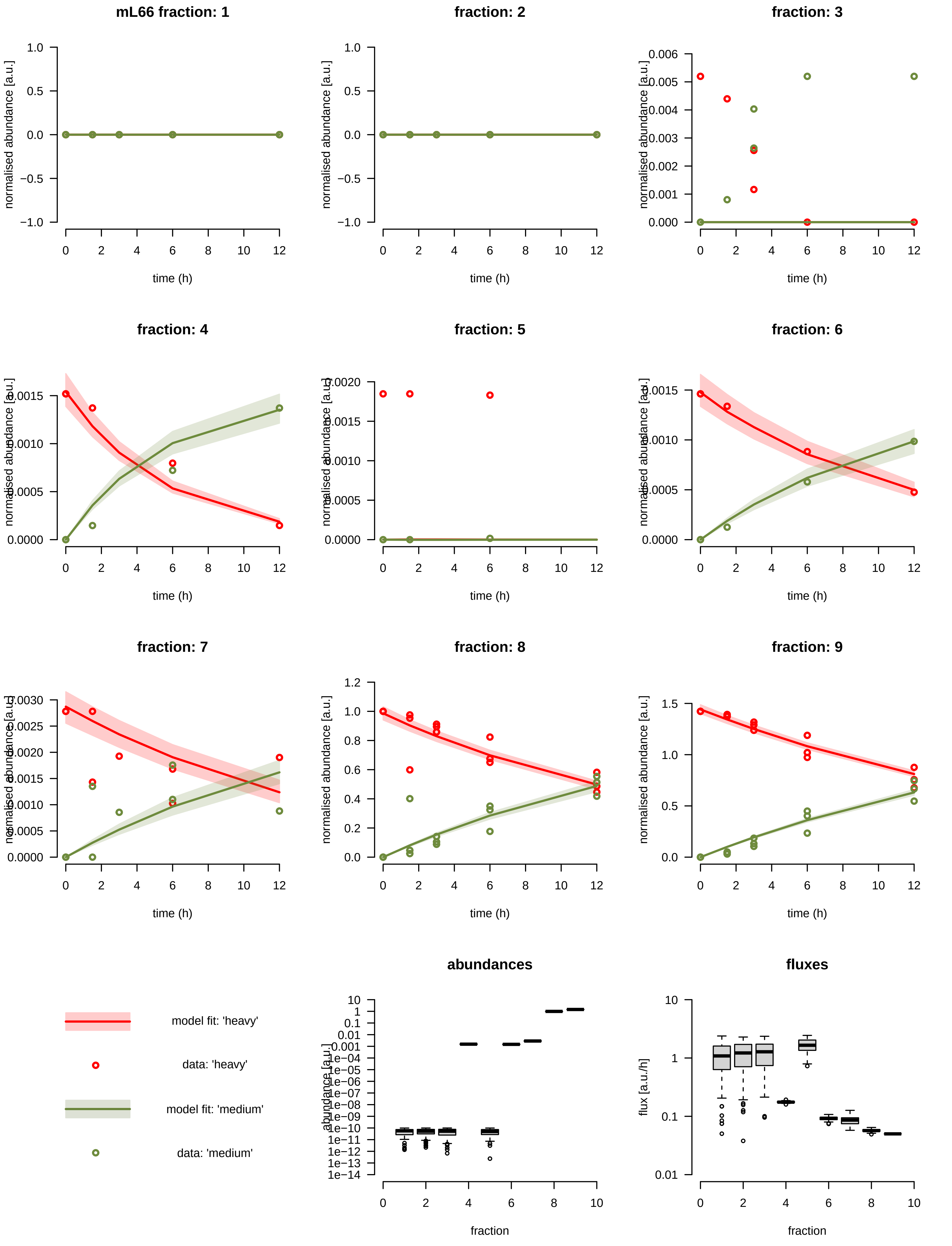
mL66 fraction: 1
fraction: 2
fraction: 3
fraction: 4
fraction: 5
fraction: 6
fraction: 7
fraction: 8
fraction: 9
normalised abundance [a.u.]
time (h)
model fit: 'heavy'
data: 'heavy'
model fit: 'medium'
data: 'medium'
abundances
abundance [a.u.]
fraction
fluxes
flux [a.u./h]
fraction
