# Supplementary material for: A roadmap for ribosome assembly in human mitochondria
Source: Nat Struct Mol Biol. 2024 Jul 11;31(12):1898–908. doi: 10.1038/s41594-024-01356-w (PMC11638073; doi:10.1038/s41594-024-01356-w)
Supplement: Supplementary file 11 — mtSSU MRP clustering based on fluxes and steady-state abundances using contact matrix constraints. Each row displays the fluxes and steady-state abundances of one mtSSU MRP (dark blue lines denoted as ‘*’). Fluxes and abundances of all MRPs that have common surface area with the target MRP (dark blue) in the final assembly structure of the mtSSU are shown in gray, if they were not clustered with the target MRP, and in brighter colors, if they were clustered with the target MRP. Red triangles above the plot indicate the sucrose gradient fraction in which the target MRP was clustered into mtSSU modules. The first fraction, in which the target MRP was clustered, is indicated as vertical dashed line. Cluster heterogeneity is denoted as ‘cluster within ss’. The heterogeneity of the cluster containing all displayed MRPs is denoted as ‘all within ss’. [file 41594_2024_1356_MOESM11_ESM.pdf]

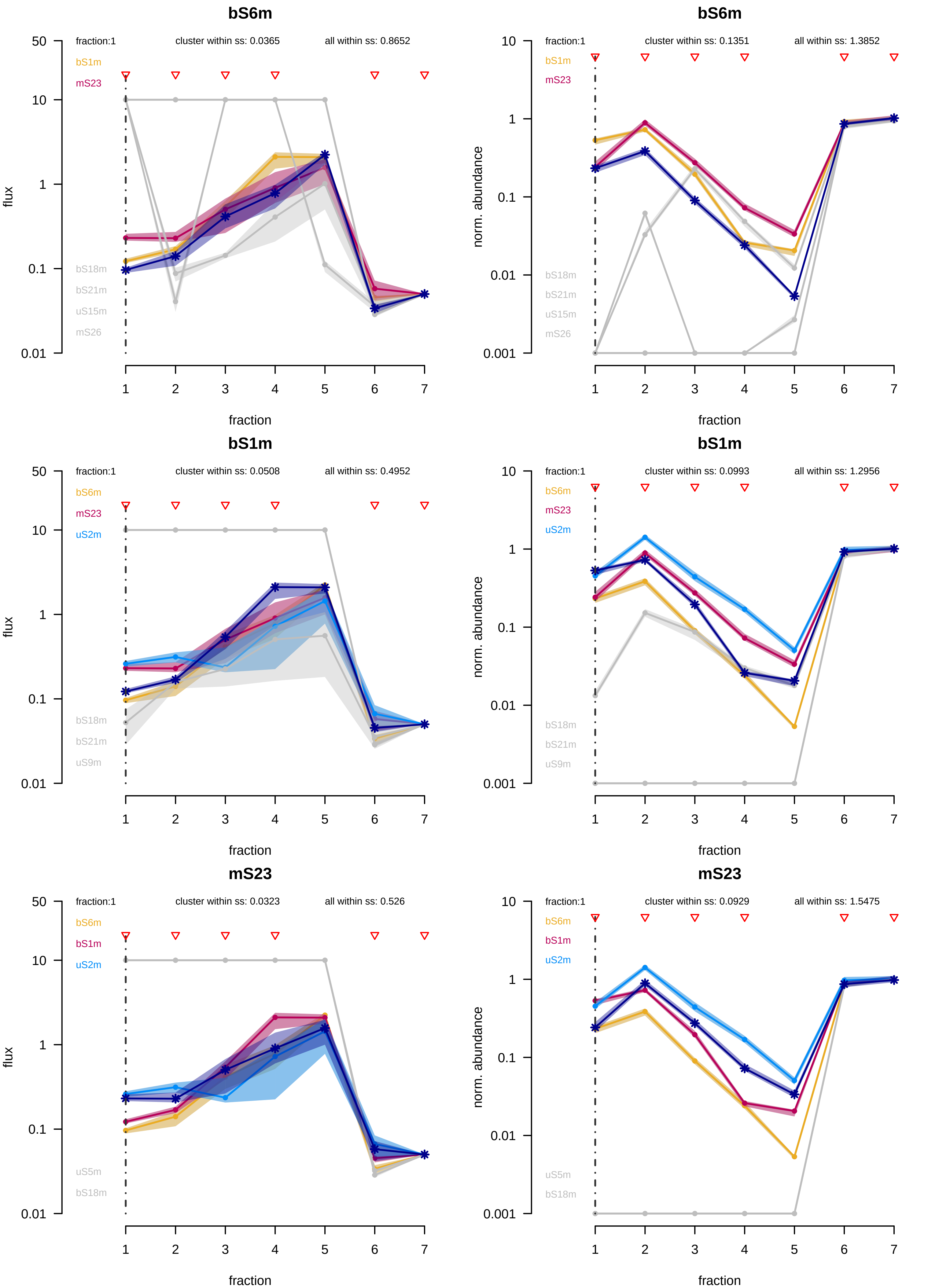

bS6m
fraction:1
cluster within ss: 0.0365
all within ss: 0.8652
bS1m
mS23
bS18m
bS21m
uS15m
mS26
flux
fraction
bS6m
fraction:1
cluster within ss: 0.1351
all within ss: 1.3852
bS1m
mS23
bS18m
bS21m
uS15m
mS26
norm. abundance
fraction
bS1m
fraction:1
cluster within ss: 0.0508
all within ss: 0.4952
bS6m
mS23
uS2m
bS18m
bS21m
uS9m
flux
fraction
bS1m
fraction:1
cluster within ss: 0.0993
all within ss: 1.2956
bS6m
mS23
uS2m
bS18m
bS21m
uS9m
norm. abundance
fraction
mS23
fraction:1
cluster within ss: 0.0323
all within ss: 0.526
bS6m
bS1m
uS2m
uS5m
bS18m
flux
fraction
mS23
fraction:1
cluster within ss: 0.0929
all within ss: 1.5475
bS6m
bS1m
uS2m
uS5m
bS18m
norm. abundance
fraction

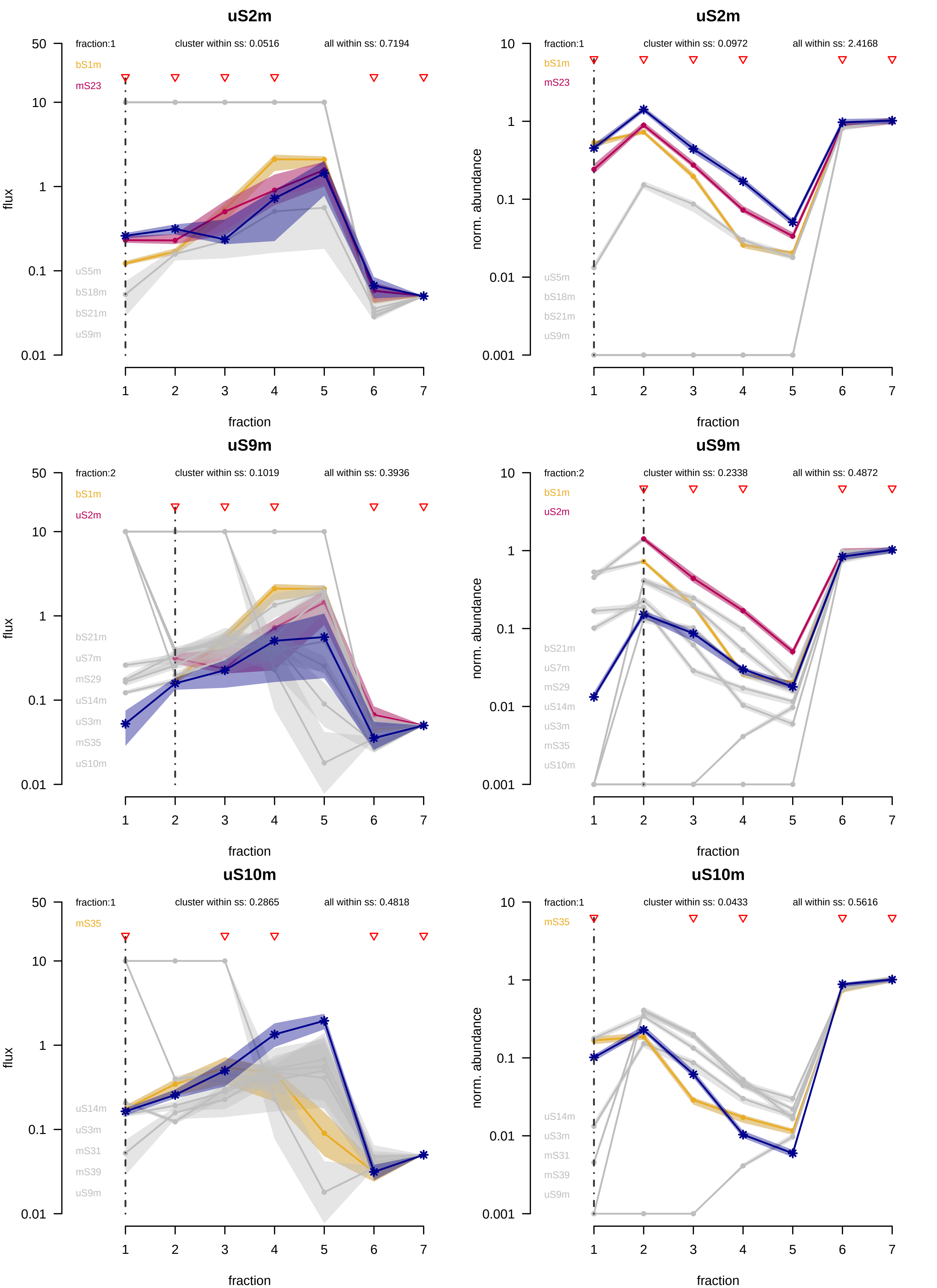

uS2m
fraction:1
cluster within ss: 0.0516
all within ss: 0.7194
bS1m
mS23
uS5m
bS18m
bS21m
uS9m
flux
50
10
1
0.1
0.01
1 2 3 4 5 6 7
fraction
uS2m
fraction:1
cluster within ss: 0.0972
all within ss: 2.4168
bS1m
mS23
uS5m
bS18m
bS21m
uS9m
norm. abundance
10
1
0.1
0.01
0.001
1 2 3 4 5 6 7
fraction
uS9m
fraction:2
cluster within ss: 0.1019
all within ss: 0.3936
bS1m
uS2m
bS21m
uS7m
mS29
uS14m
uS3m
mS35
uS10m
flux
50
10
1
0.1
0.01
1 2 3 4 5 6 7
fraction
uS9m
fraction:2
cluster within ss: 0.2338
all within ss: 0.4872
bS1m
uS2m
bS21m
uS7m
mS29
uS14m
uS3m
mS35
uS10m
norm. abundance
10
1
0.1
0.01
0.001
1 2 3 4 5 6 7
fraction
uS10m
fraction:1
cluster within ss: 0.2865
all within ss: 0.4818
mS35
uS14m
uS3m
mS31
mS39
uS9m
flux
50
10
1
0.1
0.01
1 2 3 4 5 6 7
fraction
uS10m
fraction:1
cluster within ss: 0.0433
all within ss: 0.5616
mS35
uS14m
uS3m
mS31
mS39
uS9m
norm. abundance
10
1
0.1
0.01
0.001
1 2 3 4 5 6 7
fraction

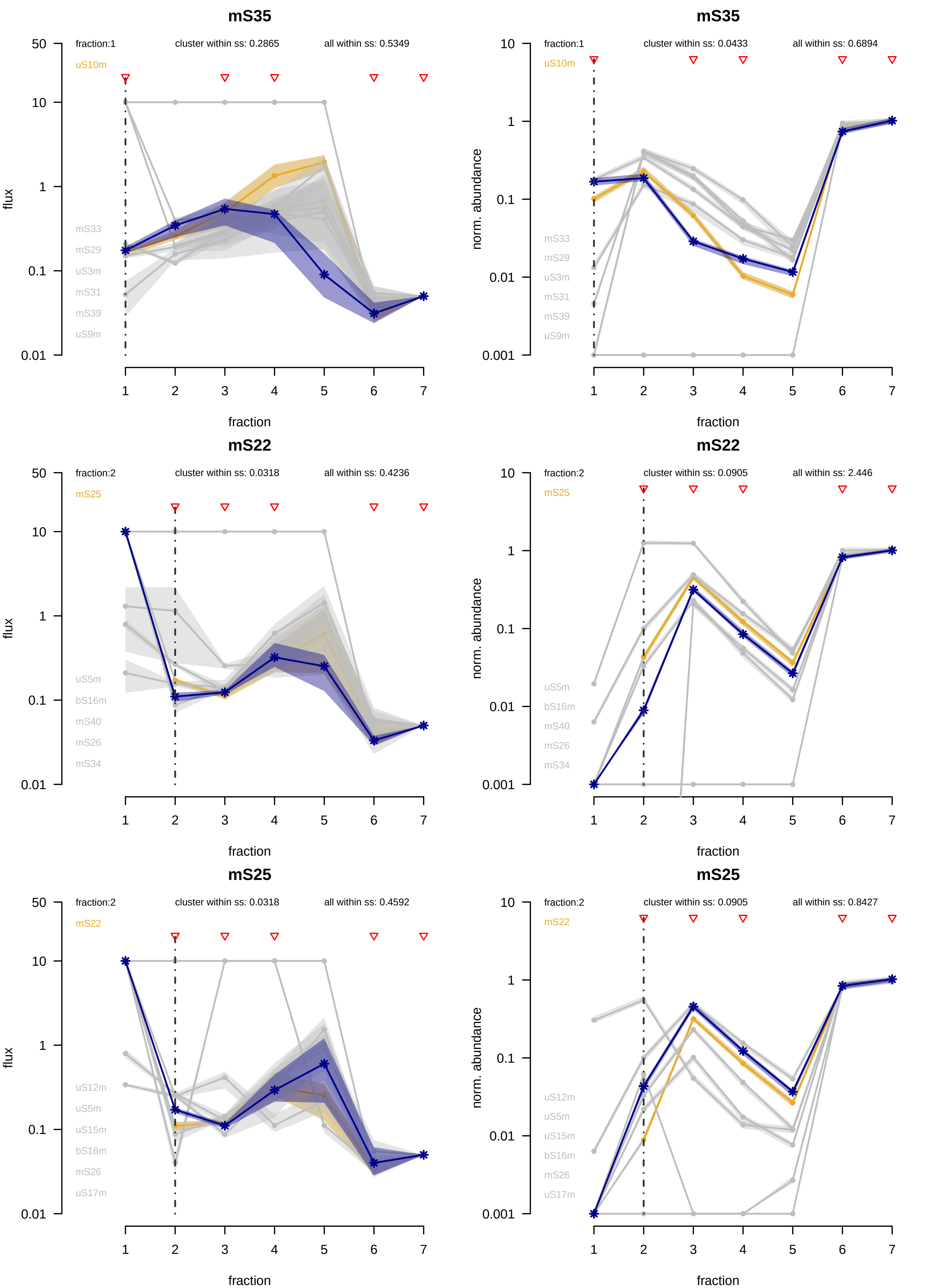
mS35
fraction:1
cluster within ss: 0.2865
all within ss: 0.5349
uS10m
mS33
mS29
uS3m
mS31
mS39
uS9m
flux
fraction
mS35
fraction:1
cluster within ss: 0.0433
all within ss: 0.6894
uS10m
mS33
mS29
uS3m
mS31
mS39
uS9m
norm. abundance
fraction
mS22
fraction:2
cluster within ss: 0.0318
all within ss: 0.4236
mS25
uS5m
bS16m
mS40
mS26
mS34
flux
fraction
mS22
fraction:2
cluster within ss: 0.0905
all within ss: 2.446
mS25
uS5m
bS16m
mS40
mS26
mS34
norm. abundance
fraction
mS25
fraction:2
cluster within ss: 0.0318
all within ss: 0.4592
mS22
uS12m
uS5m
uS15m
bS16m
mS26
uS17m
flux
fraction
mS25
fraction:2
cluster within ss: 0.0905
all within ss: 0.8427
mS22
uS12m
uS5m
uS15m
bS16m
mS26
uS17m
norm. abundance
fraction

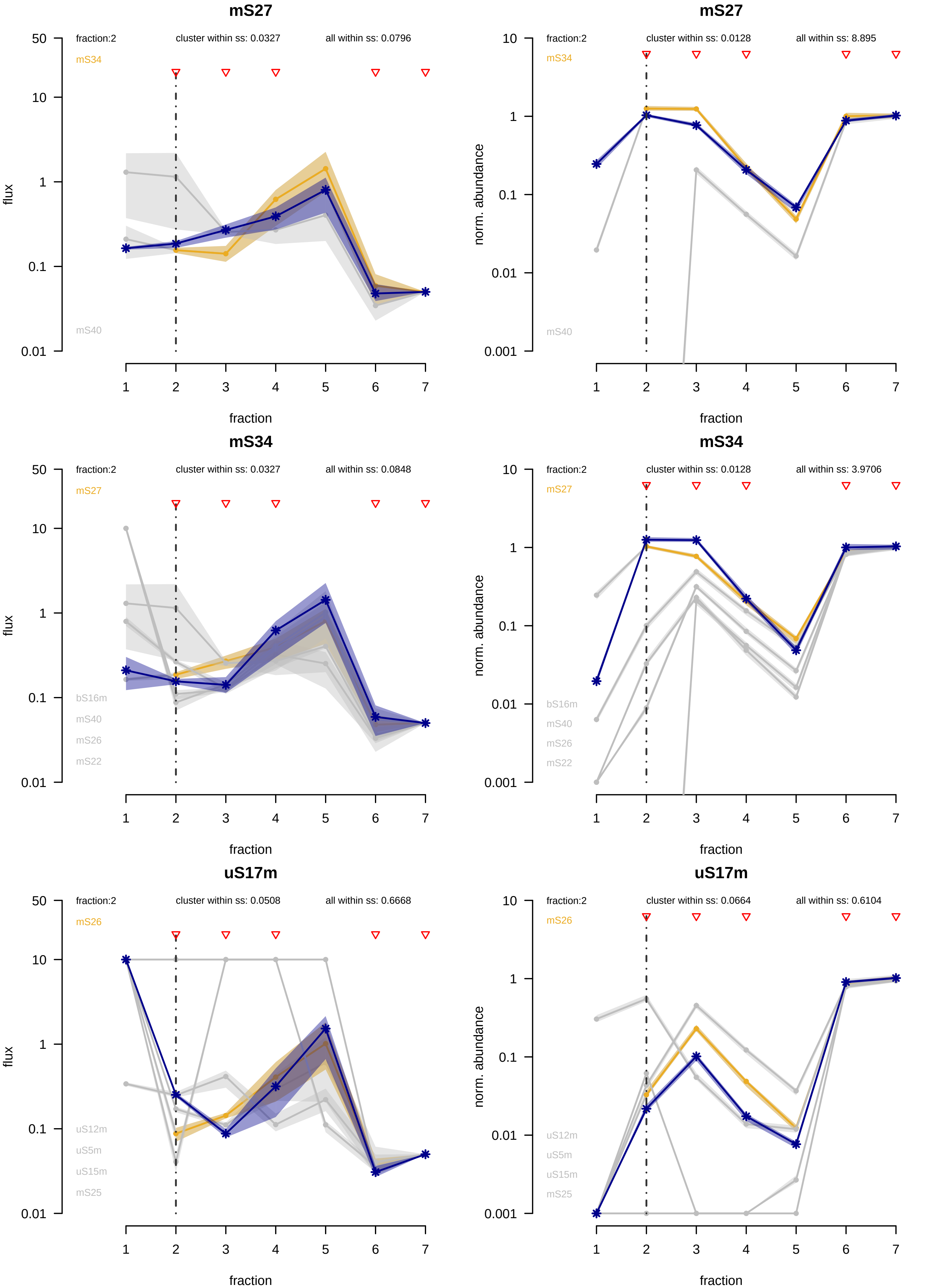
mS27
fraction:2
cluster within ss: 0.0327
all within ss: 0.0796
mS34
mS40
flux
fraction
mS27
fraction:2
cluster within ss: 0.0128
all within ss: 8.895
mS34
mS40
norm. abundance
fraction
mS34
fraction:2
cluster within ss: 0.0327
all within ss: 0.0848
mS27
bS16m
mS40
mS26
mS22
flux
fraction
mS34
fraction:2
cluster within ss: 0.0128
all within ss: 3.9706
mS27
bS16m
mS40
mS26
mS22
norm. abundance
fraction
uS17m
fraction:2
cluster within ss: 0.0508
all within ss: 0.6668
mS26
uS12m
uS5m
uS15m
mS25
flux
fraction
uS17m
fraction:2
cluster within ss: 0.0664
all within ss: 0.6104
mS26
uS12m
uS5m
uS15m
mS25
norm. abundance
fraction

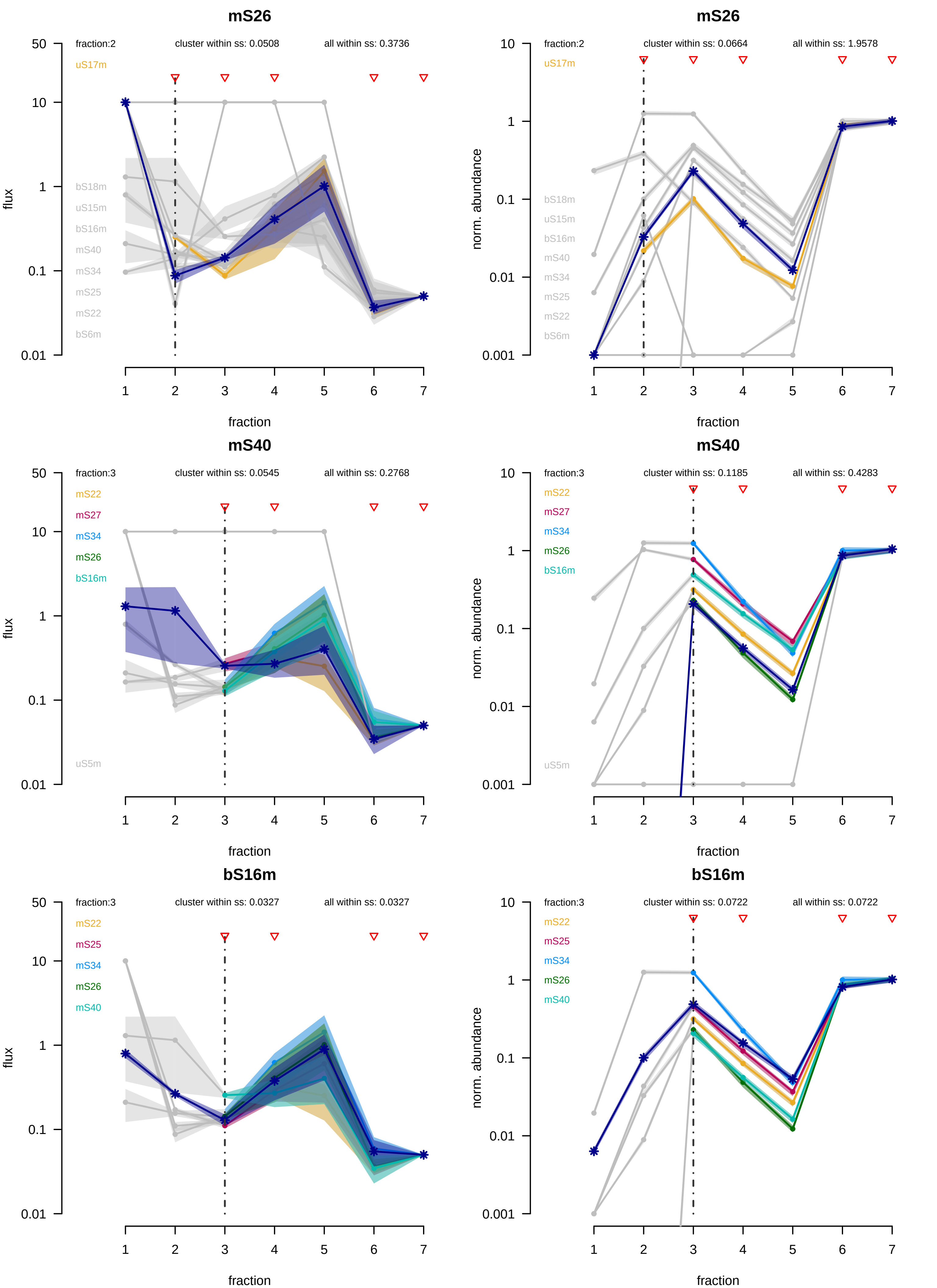

mS26
fraction:2
cluster within ss: 0.0508
all within ss: 0.3736
uS17m
bS18m
uS15m
bS16m
mS40
mS34
mS25
mS22
bS6m
flux
50
10
1
0.1
0.01
1 2 3 4 5 6 7
fraction
mS26
fraction:2
cluster within ss: 0.0664
all within ss: 1.9578
uS17m
bS18m
uS15m
bS16m
mS40
mS34
mS25
mS22
bS6m
norm. abundance
10
1
0.1
0.01
0.001
1 2 3 4 5 6 7
fraction
mS40
fraction:3
cluster within ss: 0.0545
all within ss: 0.2768
mS22
mS27
mS34
mS26
bS16m
uS5m
flux
50
10
1
0.1
0.01
1 2 3 4 5 6 7
fraction
mS40
fraction:3
cluster within ss: 0.1185
all within ss: 0.4283
mS22
mS27
mS34
mS26
bS16m
uS5m
norm. abundance
10
1
0.1
0.01
0.001
1 2 3 4 5 6 7
fraction
bS16m
fraction:3
cluster within ss: 0.0327
all within ss: 0.0327
mS22
mS25
mS34
mS26
mS40
flux
50
10
1
0.1
0.01
1 2 3 4 5 6 7
fraction
bS16m
fraction:3
cluster within ss: 0.0722
all within ss: 0.0722
mS22
mS25
mS34
mS26
mS40
norm. abundance
10
1
0.1
0.01
0.001
1 2 3 4 5 6 7
fraction

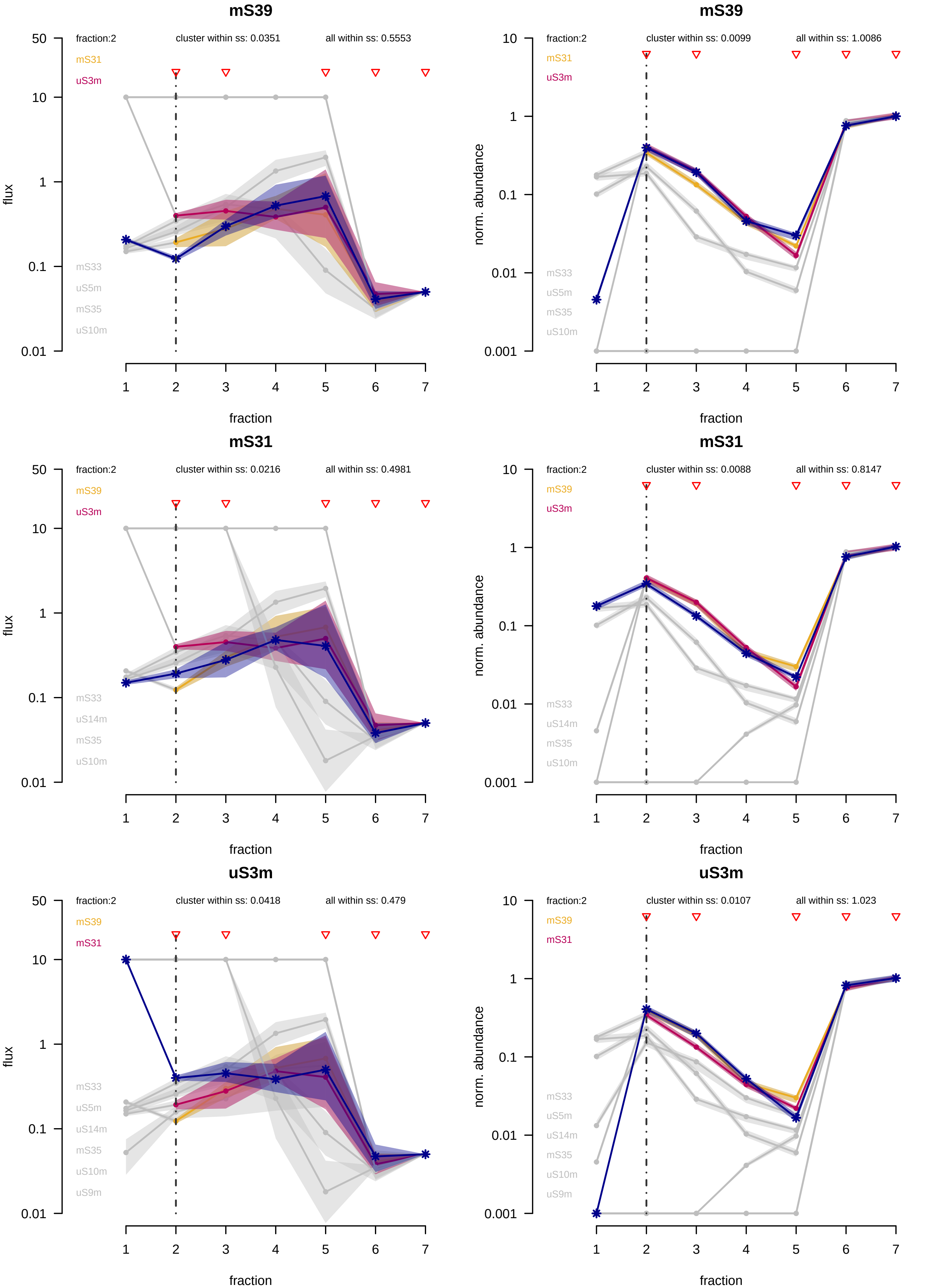

mS39
fraction:2
cluster within ss: 0.0351
all within ss: 0.5553
mS31
uS3m
mS33
uS5m
mS35
uS10m
flux
fraction
mS39
fraction:2
cluster within ss: 0.0099
all within ss: 1.0086
mS31
uS3m
mS33
uS5m
mS35
uS10m
norm. abundance
fraction
mS31
fraction:2
cluster within ss: 0.0216
all within ss: 0.4981
mS39
uS3m
mS33
uS14m
mS35
uS10m
flux
fraction
mS31
fraction:2
cluster within ss: 0.0088
all within ss: 0.8147
mS39
uS3m
mS33
uS14m
mS35
uS10m
norm. abundance
fraction
uS3m
fraction:2
cluster within ss: 0.0418
all within ss: 0.479
mS39
mS31
mS33
uS5m
uS14m
mS35
uS10m
uS9m
flux
fraction
uS3m
fraction:2
cluster within ss: 0.0107
all within ss: 1.023
mS39
mS31
mS33
uS5m
uS14m
mS35
uS10m
uS9m
norm. abundance
fraction

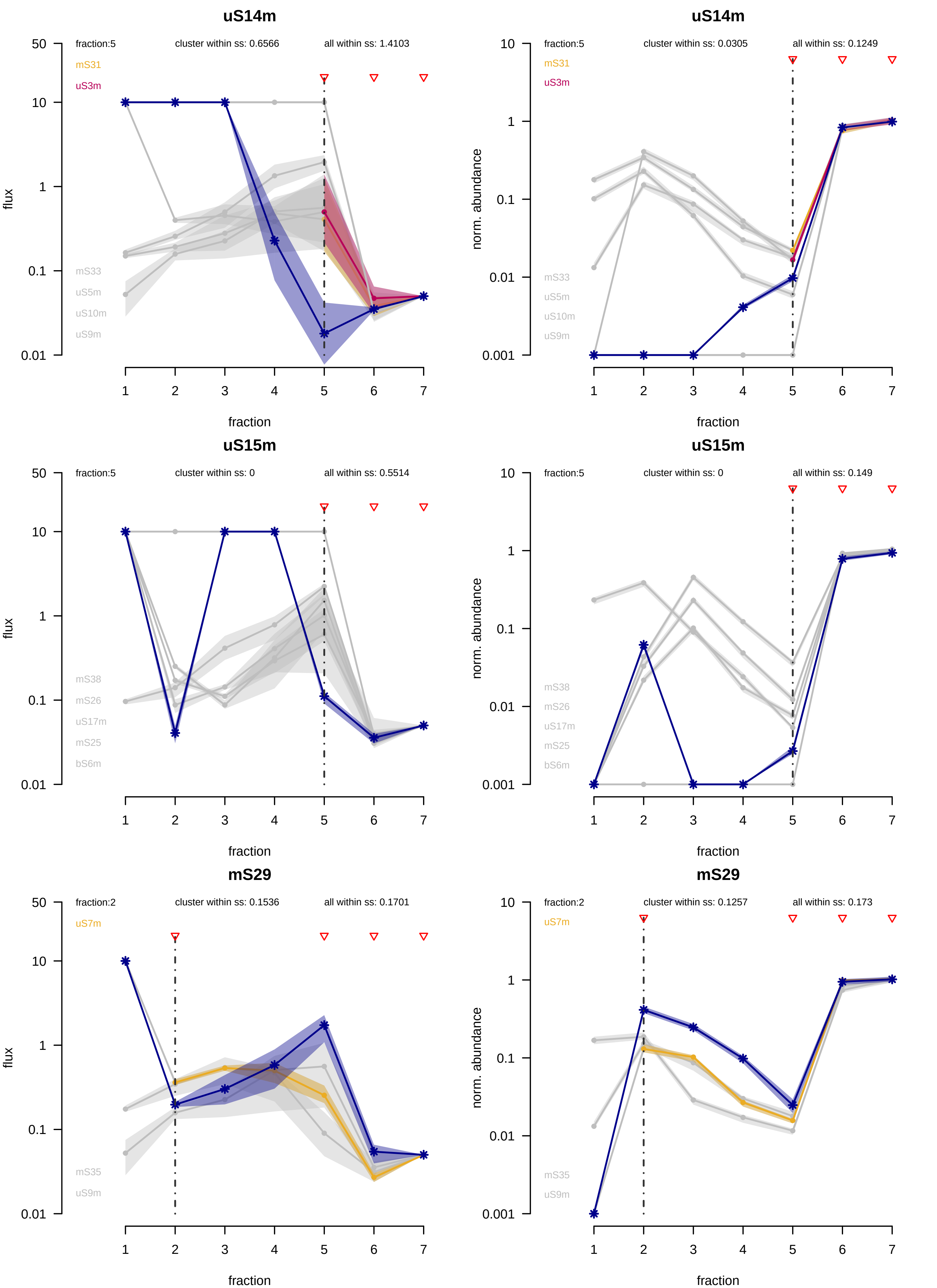
uS14m
fraction:5
cluster within ss: 0.6566
all within ss: 1.4103
mS31
uS3m
mS33
uS5m
uS10m
uS9m
flux
fraction
uS14m
fraction:5
cluster within ss: 0.0305
all within ss: 0.1249
mS31
uS3m
mS33
uS5m
uS10m
uS9m
norm. abundance
fraction
uS15m
fraction:5
cluster within ss: 0
all within ss: 0.5514
mS38
mS26
uS17m
mS25
bS6m
flux
fraction
uS15m
fraction:5
cluster within ss: 0
all within ss: 0.149
mS38
mS26
uS17m
mS25
bS6m
norm. abundance
fraction
mS29
fraction:2
cluster within ss: 0.1536
all within ss: 0.1701
uS7m
mS35
uS9m
flux
fraction
mS29
fraction:2
cluster within ss: 0.1257
all within ss: 0.173
uS7m
mS35
uS9m
norm. abundance
fraction

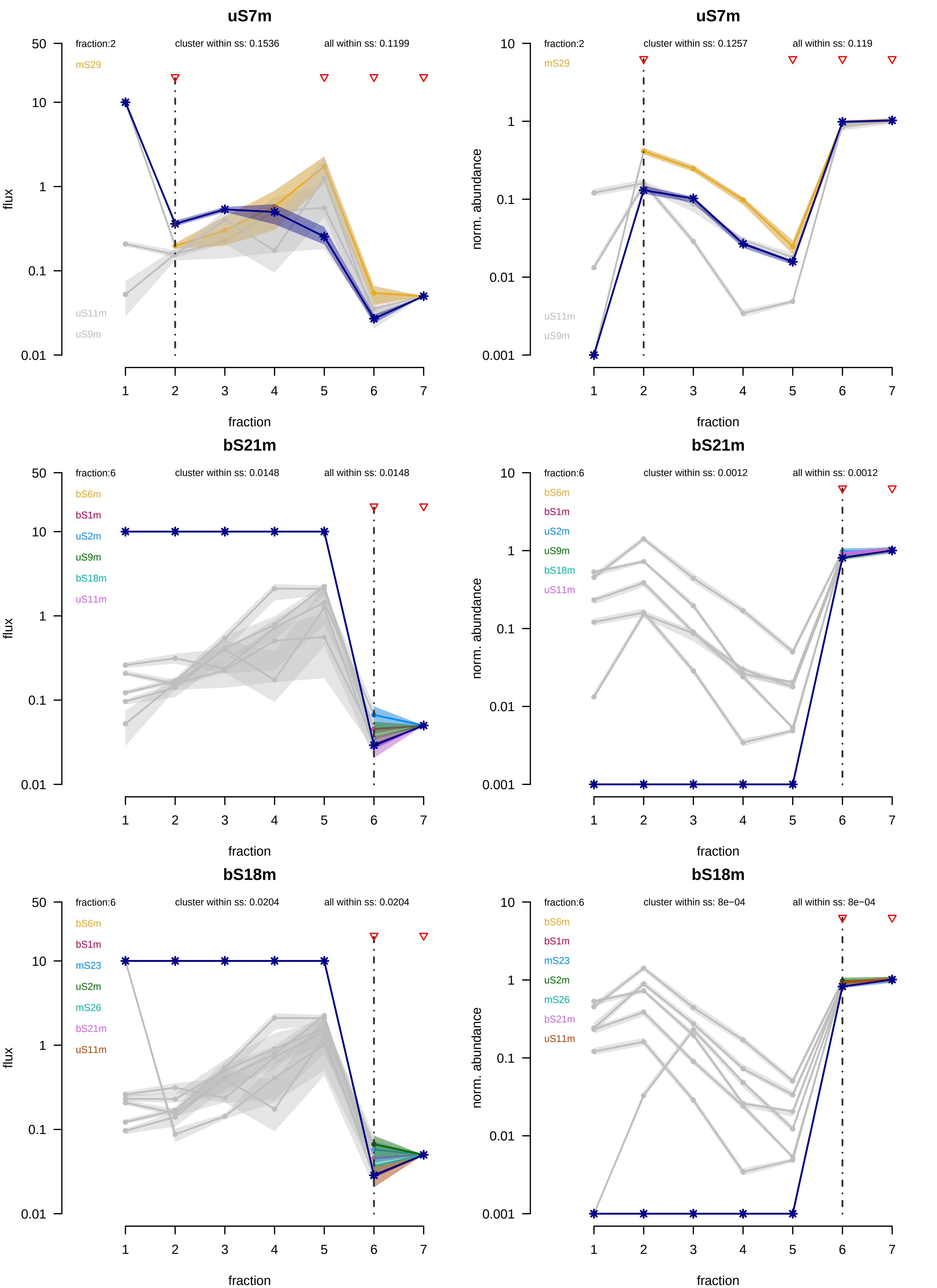

uS7m
fraction:2
cluster within ss: 0.1536
all within ss: 0.1199
mS29
uS11m
uS9m
flux
fraction
uS7m
fraction:2
cluster within ss: 0.1257
all within ss: 0.119
mS29
uS11m
uS9m
norm. abundance
fraction
bS21m
fraction:6
cluster within ss: 0.0148
all within ss: 0.0148
bS6m
bS1m
uS2m
uS9m
bS18m
uS11m
flux
fraction
bS21m
fraction:6
cluster within ss: 0.0012
all within ss: 0.0012
bS6m
bS1m
uS2m
uS9m
bS18m
uS11m
norm. abundance
fraction
bS18m
fraction:6
cluster within ss: 0.0204
all within ss: 0.0204
bS6m
bS1m
mS23
uS2m
mS26
bS21m
uS11m
flux
fraction
bS18m
fraction:6
cluster within ss: 8e−04
all within ss: 8e−04
bS6m
bS1m
mS23
uS2m
mS26
bS21m
uS11m
norm. abundance
fraction

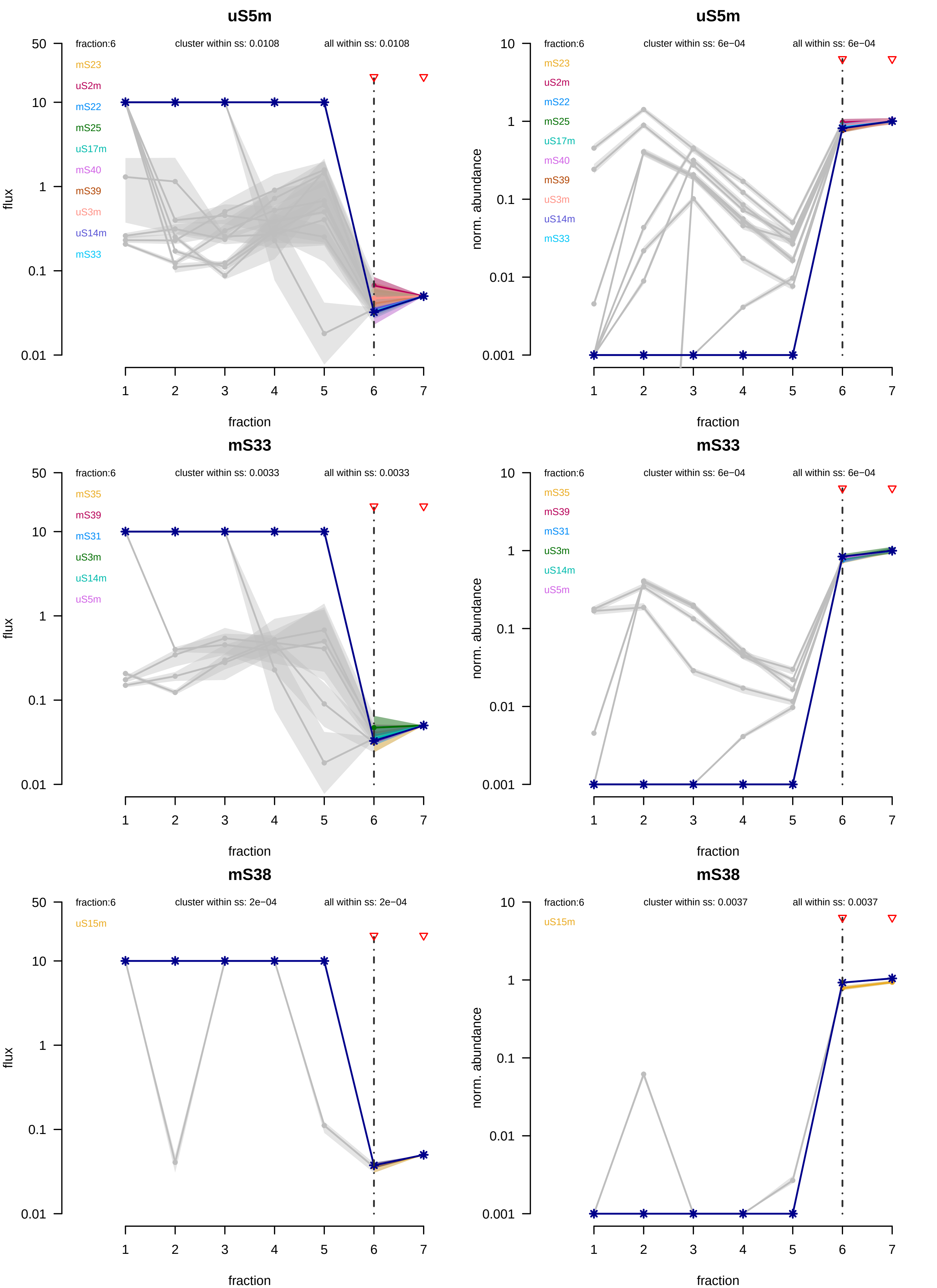
uS5m
fraction:6
cluster within ss: 0.0108
all within ss: 0.0108
mS23
uS2m
mS22
mS25
uS17m
mS40
mS39
uS3m
uS14m
mS33
flux
50
10
1
0.1
0.01
1
2
3
4
5
6
7
fraction
uS5m
fraction:6
cluster within ss: 6e−04
all within ss: 6e−04
mS23
uS2m
mS22
mS25
uS17m
mS40
mS39
uS3m
uS14m
mS33
norm. abundance
10
1
0.1
0.01
0.001
fraction
mS33
fraction:6
cluster within ss: 0.0033
all within ss: 0.0033
mS35
mS39
mS31
uS3m
uS14m
uS5m
flux
fraction
mS33
fraction:6
cluster within ss: 6e−04
all within ss: 6e−04
mS35
mS39
mS31
uS3m
uS14m
uS5m
norm. abundance
fraction
mS38
fraction:6
cluster within ss: 2e−04
all within ss: 2e−04
uS15m
flux
fraction
mS38
fraction:6
cluster within ss: 0.0037
all within ss: 0.0037
uS15m
norm. abundance
fraction

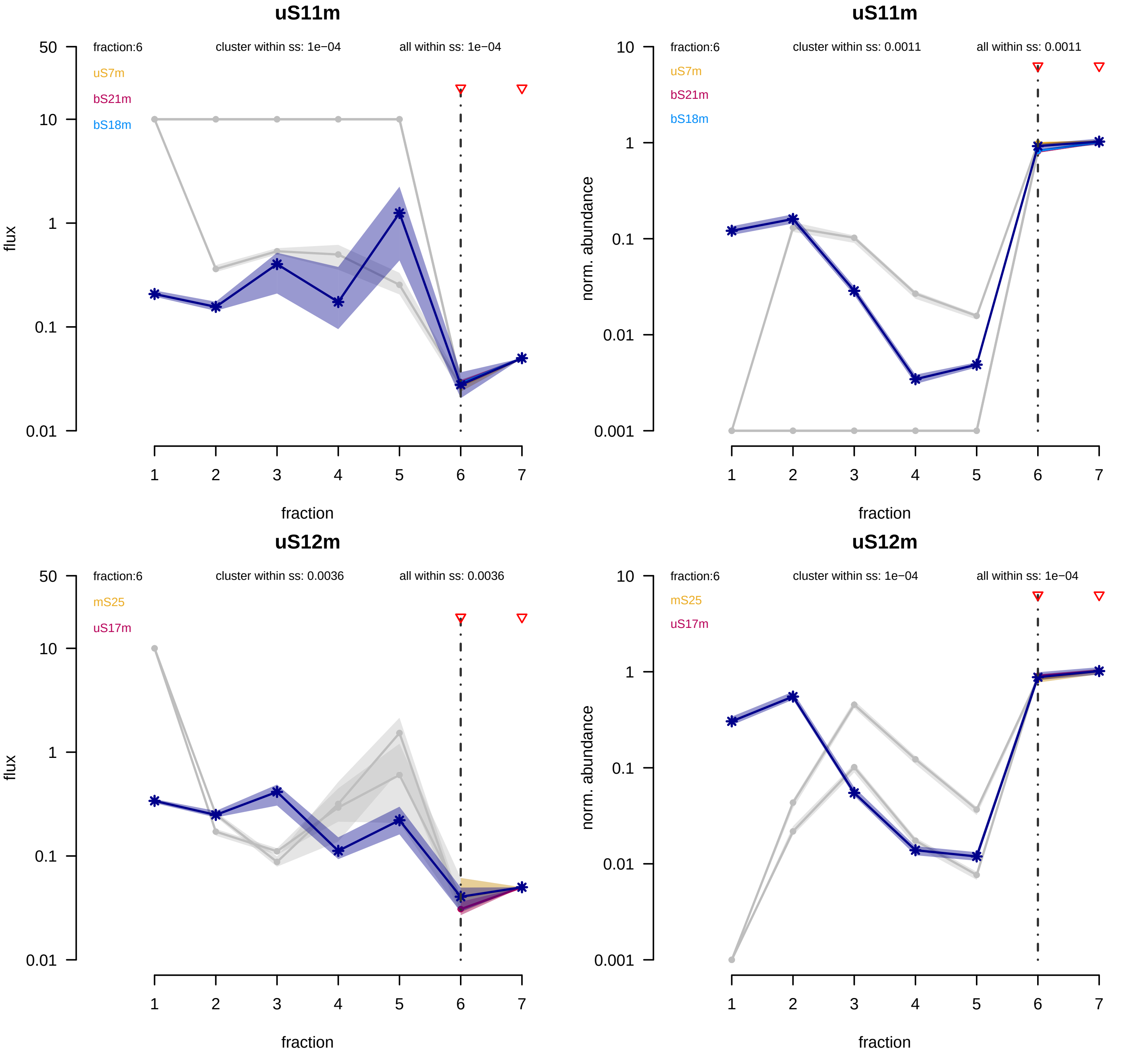
uS11m
fraction:6
cluster within ss: 1e−04
all within ss: 1e−04
uS7m
bS21m
bS18m
flux
50
10
1
0.1
0.01
1 2 3 4 5 6 7
fraction
uS11m
fraction:6
cluster within ss: 0.0011
all within ss: 0.0011
uS7m
bS21m
bS18m
norm. abundance
10
1
0.1
0.01
0.001
1 2 3 4 5 6 7
fraction
uS12m
fraction:6
cluster within ss: 0.0036
all within ss: 0.0036
mS25
uS17m
flux
50
10
1
0.1
0.01
1 2 3 4 5 6 7
fraction
uS12m
fraction:6
cluster within ss: 1e−04
all within ss: 1e−04
mS25
uS17m
norm. abundance
10
1
0.1
0.01
0.001
1 2 3 4 5 6 7
fraction
