# Supplementary material for: A roadmap for ribosome assembly in human mitochondria
Source: Nat Struct Mol Biol. 2024 Jul 11;31(12):1898–908. doi: 10.1038/s41594-024-01356-w (PMC11638073; doi:10.1038/s41594-024-01356-w)
Supplement: Supplementary file 12 — mtLSU MRP clustering based on fluxes and steady-state abundances using contact matrix constraints. Each row displays the fluxes and steady-state abundances of one mtLSU MRP (dark blue lines denoted as ‘*’). Fluxes and abundances of all MRPs that have common surface area with the target MRP (dark blue) in the final assembly structure of the mtLSU are shown in gray, if they were not clustered with the target MRP, and in brighter colors, if they were clustered with the target MRP. Red triangles above the plot indicate the sucrose gradient fraction, in which the target MRP was clustered into mtLSU modules. The first fraction, in which the target MRP was clustered, is indicated as vertical dashed line. Cluster heterogeneity is denoted as ‘cluster within ss’. The heterogeneity of the cluster containing all displayed MRPs is denoted as ‘all within ss’. [file 41594_2024_1356_MOESM12_ESM.pdf]

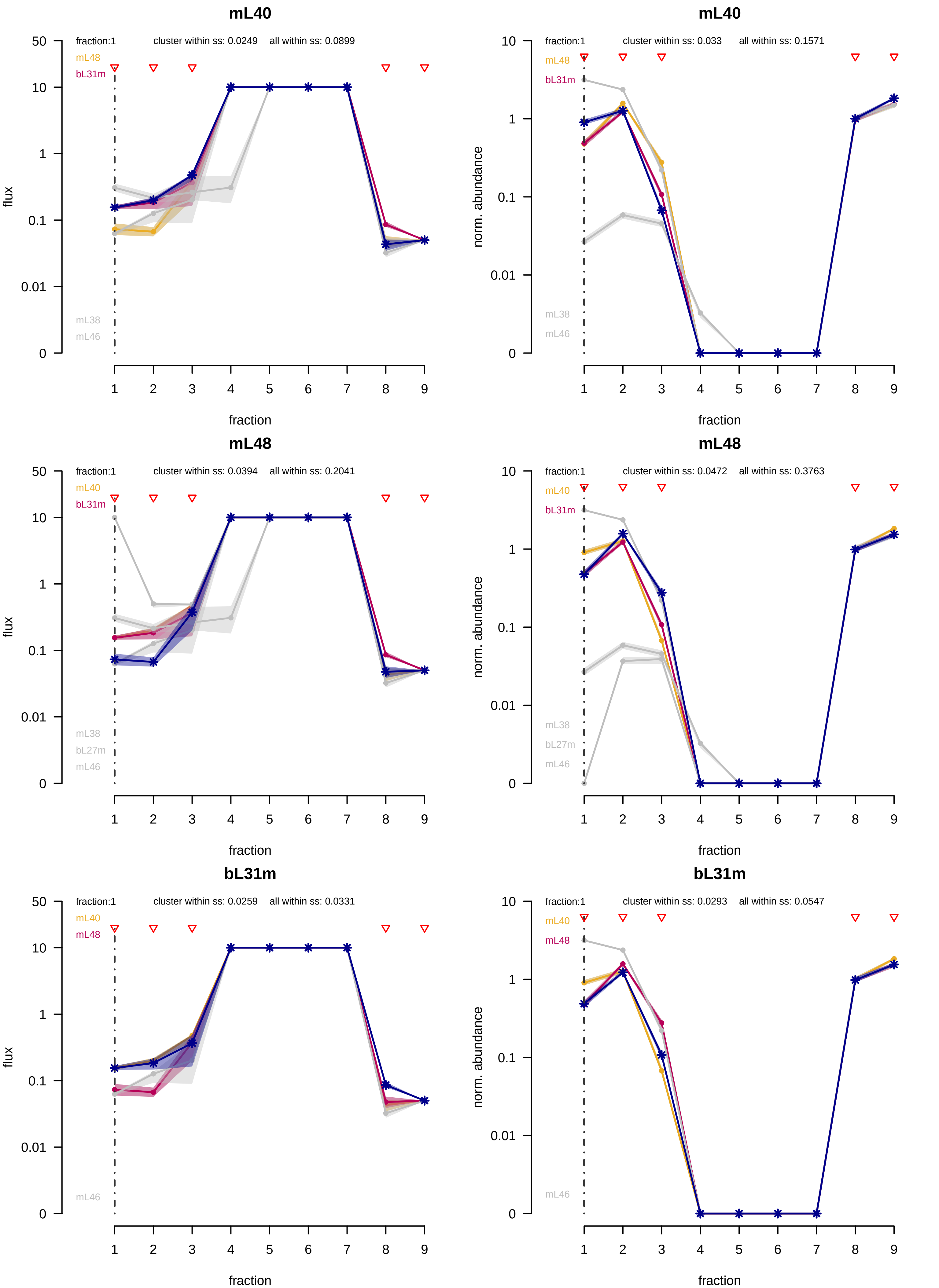
mL40
fraction:1
cluster within ss: 0.0249
all within ss: 0.0899
mL48
bL31m
mL38
mL46
flux
fraction
mL40
fraction:1
cluster within ss: 0.033
all within ss: 0.1571
mL48
bL31m
mL38
mL46
norm. abundance
fraction
mL48
fraction:1
cluster within ss: 0.0394
all within ss: 0.2041
mL40
bL31m
mL38
bL27m
mL46
flux
fraction
mL48
fraction:1
cluster within ss: 0.0472
all within ss: 0.3763
mL40
bL31m
mL38
bL27m
mL46
norm. abundance
fraction
bL31m
fraction:1
cluster within ss: 0.0259
all within ss: 0.0331
mL40
mL48
mL46
flux
fraction
bL31m
fraction:1
cluster within ss: 0.0293
all within ss: 0.0547
mL40
mL48
mL46
norm. abundance
fraction

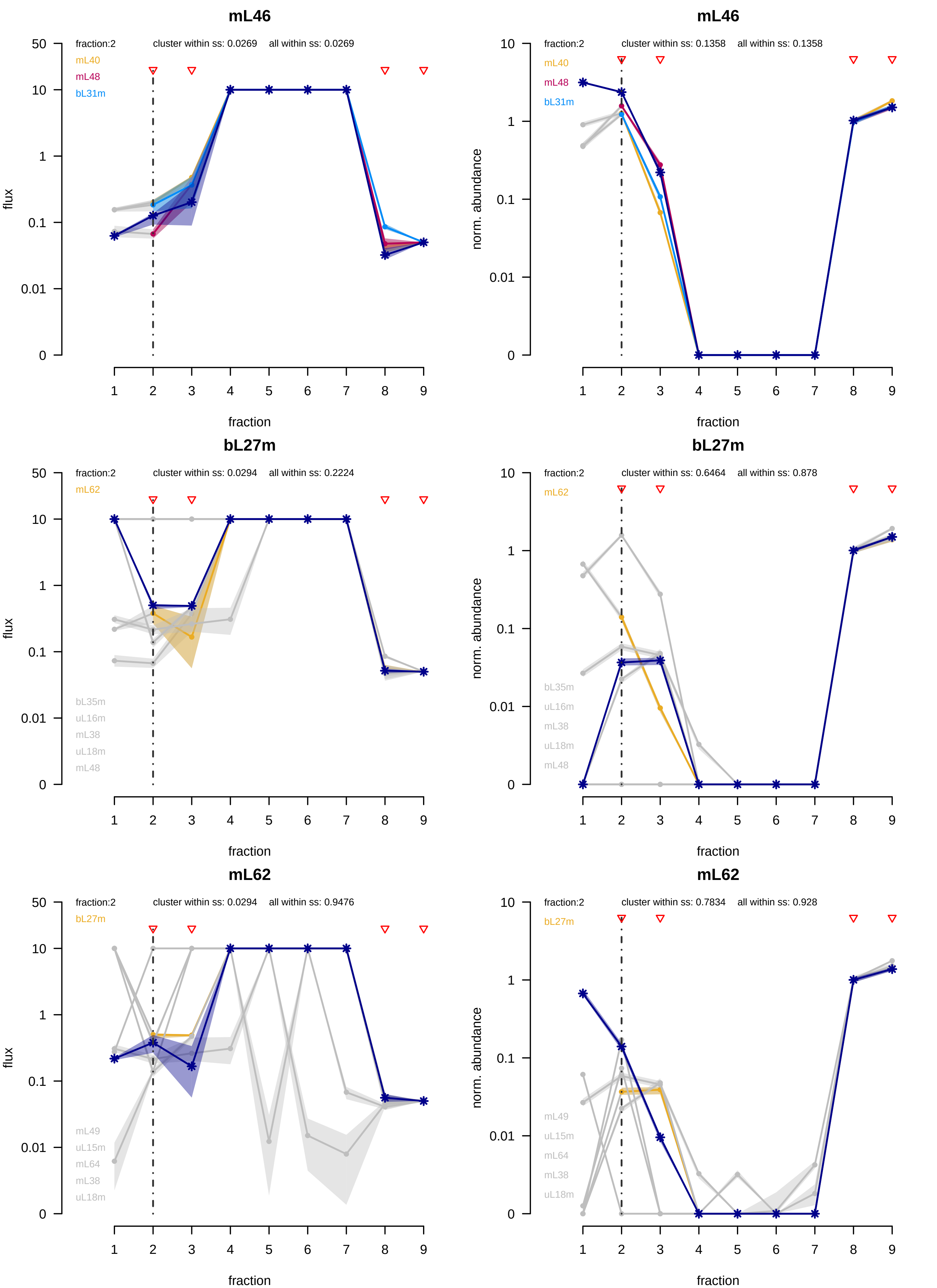
mL46
fraction:2
cluster within ss: 0.0269
all within ss: 0.0269
mL40
mL48
bL31m
flux
fraction
mL46
fraction:2
cluster within ss: 0.1358
all within ss: 0.1358
mL40
mL48
bL31m
norm. abundance
fraction
bL27m
fraction:2
cluster within ss: 0.0294
all within ss: 0.2224
mL62
bL35m
uL16m
mL38
uL18m
mL48
flux
fraction
bL27m
fraction:2
cluster within ss: 0.6464
all within ss: 0.878
mL62
bL35m
uL16m
mL38
uL18m
mL48
norm. abundance
fraction
mL62
fraction:2
cluster within ss: 0.0294
all within ss: 0.9476
bL27m
mL49
uL15m
mL64
mL38
uL18m
flux
fraction
mL62
fraction:2
cluster within ss: 0.7834
all within ss: 0.928
bL27m
mL49
uL15m
mL64
mL38
uL18m
norm. abundance
fraction

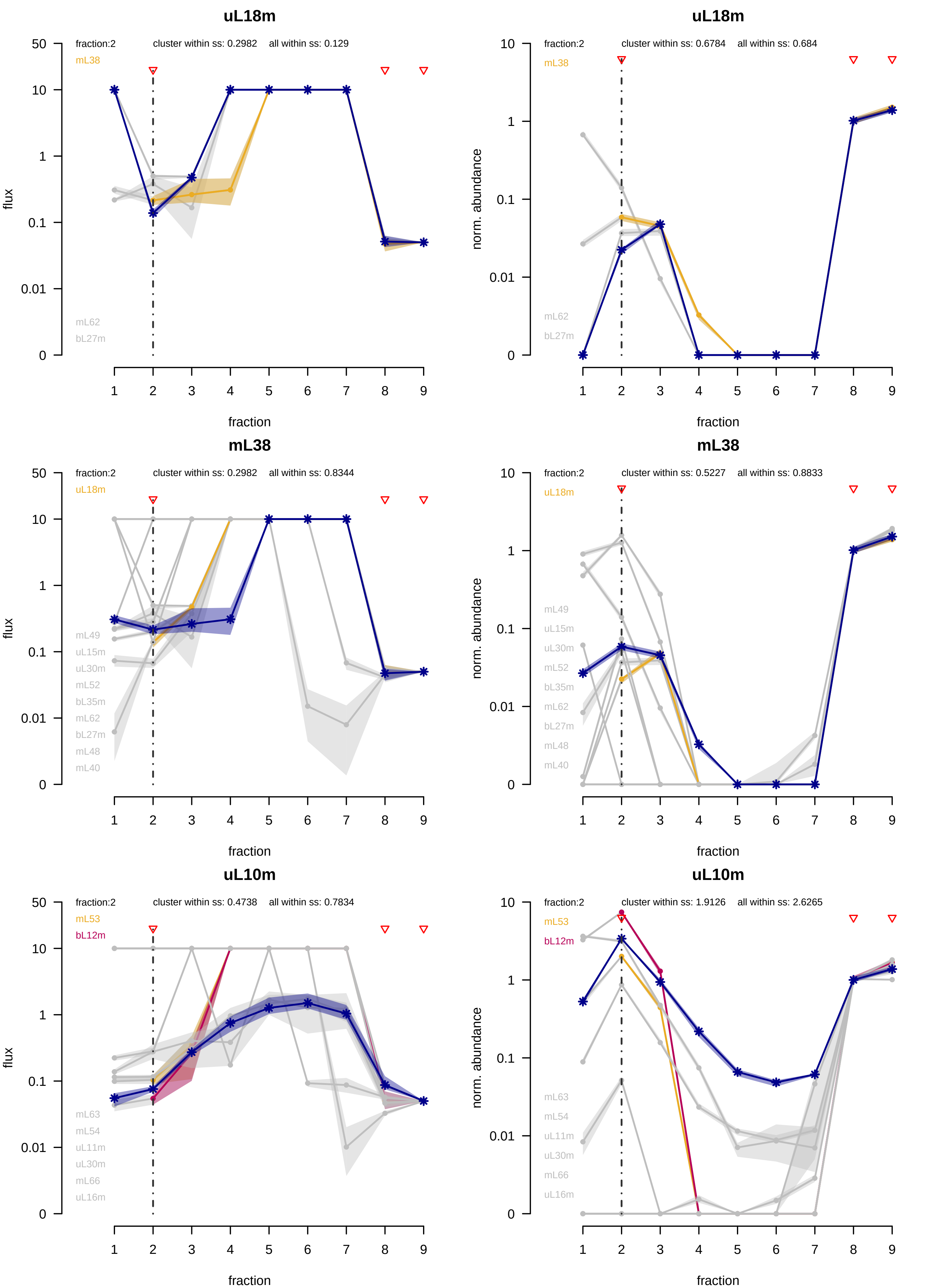

uL18m
fraction:2
cluster within ss: 0.2982
all within ss: 0.129
mL38
mL62
bL27m
flux
fraction
uL18m
fraction:2
cluster within ss: 0.6784
all within ss: 0.684
mL38
mL62
bL27m
norm. abundance
fraction
mL38
fraction:2
cluster within ss: 0.2982
all within ss: 0.8344
uL18m
mL49
uL15m
uL30m
mL52
bL35m
mL62
bL27m
mL48
mL40
flux
fraction
mL38
fraction:2
cluster within ss: 0.5227
all within ss: 0.8833
uL18m
mL49
uL15m
uL30m
mL52
bL35m
mL62
bL27m
mL48
mL40
norm. abundance
fraction
uL10m
fraction:2
cluster within ss: 0.4738
all within ss: 0.7834
mL53
bL12m
mL63
mL54
uL11m
uL30m
mL66
uL16m
flux
fraction
uL10m
fraction:2
cluster within ss: 1.9126
all within ss: 2.6265
mL53
bL12m
mL63
mL54
uL11m
uL30m
mL66
uL16m
norm. abundance
fraction

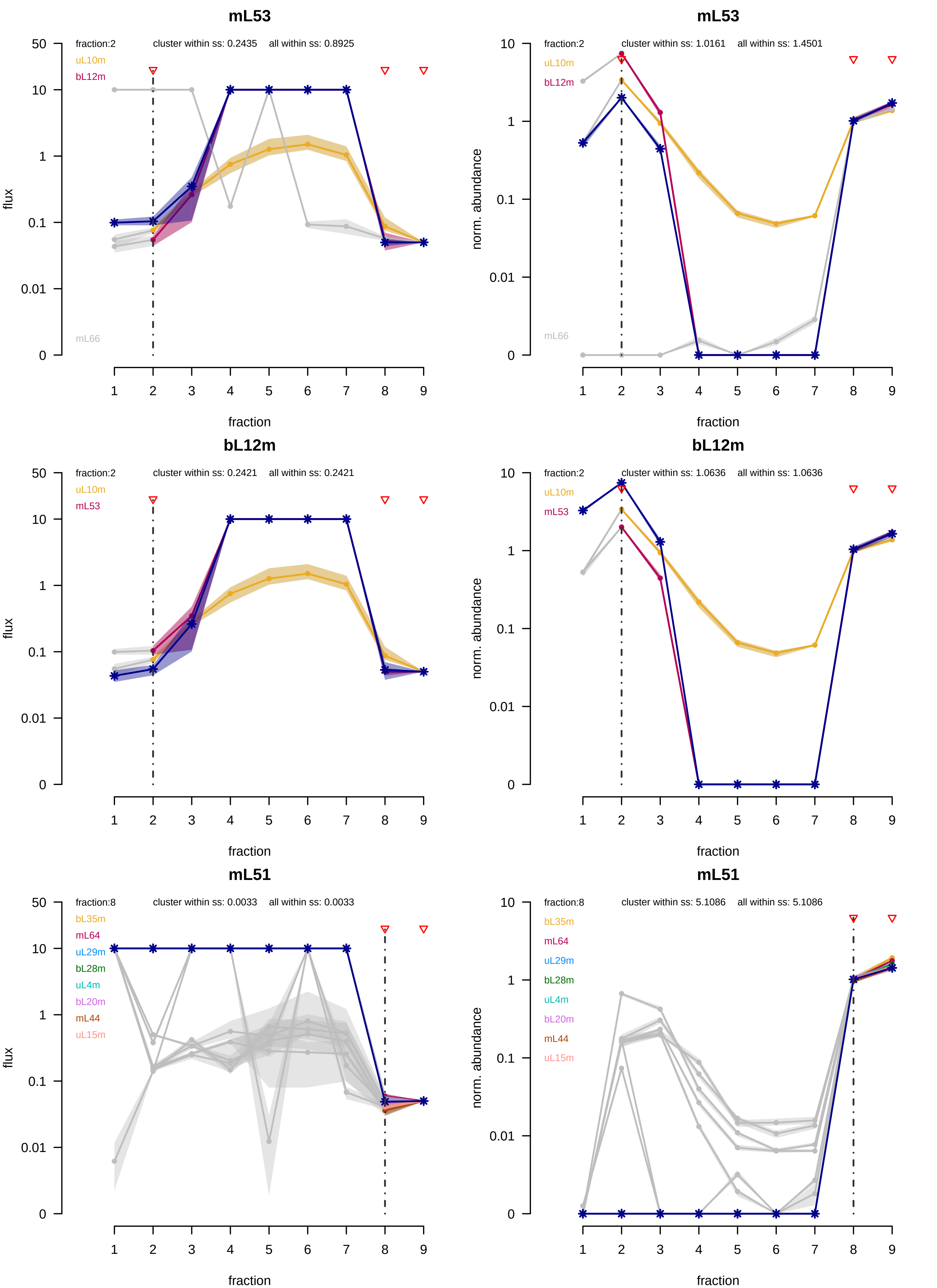

mL53
fraction:2
cluster within ss: 0.2435
all within ss: 0.8925
uL10m
bL12m
mL66
flux
fraction
mL53
fraction:2
cluster within ss: 1.0161
all within ss: 1.4501
uL10m
bL12m
mL66
norm. abundance
fraction
bL12m
fraction:2
cluster within ss: 0.2421
all within ss: 0.2421
uL10m
mL53
flux
fraction
bL12m
fraction:2
cluster within ss: 1.0636
all within ss: 1.0636
uL10m
mL53
norm. abundance
fraction
mL51
fraction:8
cluster within ss: 0.0033
all within ss: 0.0033
bL35m
mL64
uL29m
bL28m
uL4m
bL20m
mL44
uL15m
flux
fraction
mL51
fraction:8
cluster within ss: 5.1086
all within ss: 5.1086
bL35m
mL64
uL29m
bL28m
uL4m
bL20m
mL44
uL15m
norm. abundance
fraction

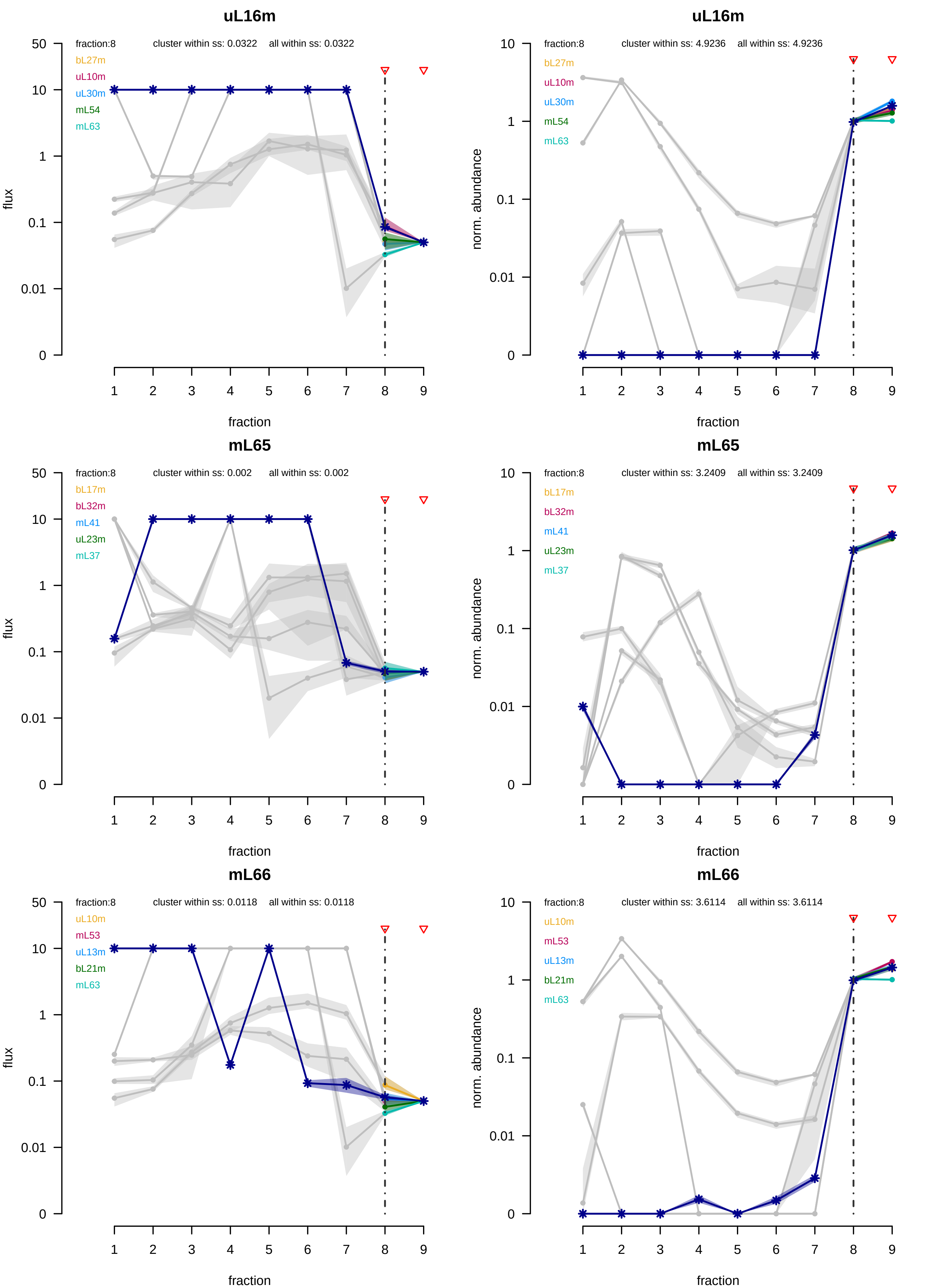
uL16m
fraction:8
cluster within ss: 0.0322
all within ss: 0.0322
bL27m
uL10m
uL30m
mL54
mL63
flux
50
10
1
0.1
0.01
0
1 2 3 4 5 6 7 8 9
fraction
uL16m
fraction:8
cluster within ss: 4.9236
all within ss: 4.9236
bL27m
uL10m
uL30m
mL54
mL63
norm. abundance
10
1
0.1
0.01
0
1 2 3 4 5 6 7 8 9
fraction
mL65
fraction:8
cluster within ss: 0.002
all within ss: 0.002
bL17m
bL32m
mL41
uL23m
mL37
flux
50
10
1
0.1
0.01
0
1 2 3 4 5 6 7 8 9
fraction
mL65
fraction:8
cluster within ss: 3.2409
all within ss: 3.2409
bL17m
bL32m
mL41
uL23m
mL37
norm. abundance
10
1
0.1
0.01
0
1 2 3 4 5 6 7 8 9
fraction
mL66
fraction:8
cluster within ss: 0.0118
all within ss: 0.0118
uL10m
mL53
uL13m
bL21m
mL63
flux
50
10
1
0.1
0.01
0
1 2 3 4 5 6 7 8 9
fraction
mL66
fraction:8
cluster within ss: 3.6114
all within ss: 3.6114
uL10m
mL53
uL13m
bL21m
mL63
norm. abundance
10
1
0.1
0.01
0
1 2 3 4 5 6 7 8 9
fraction

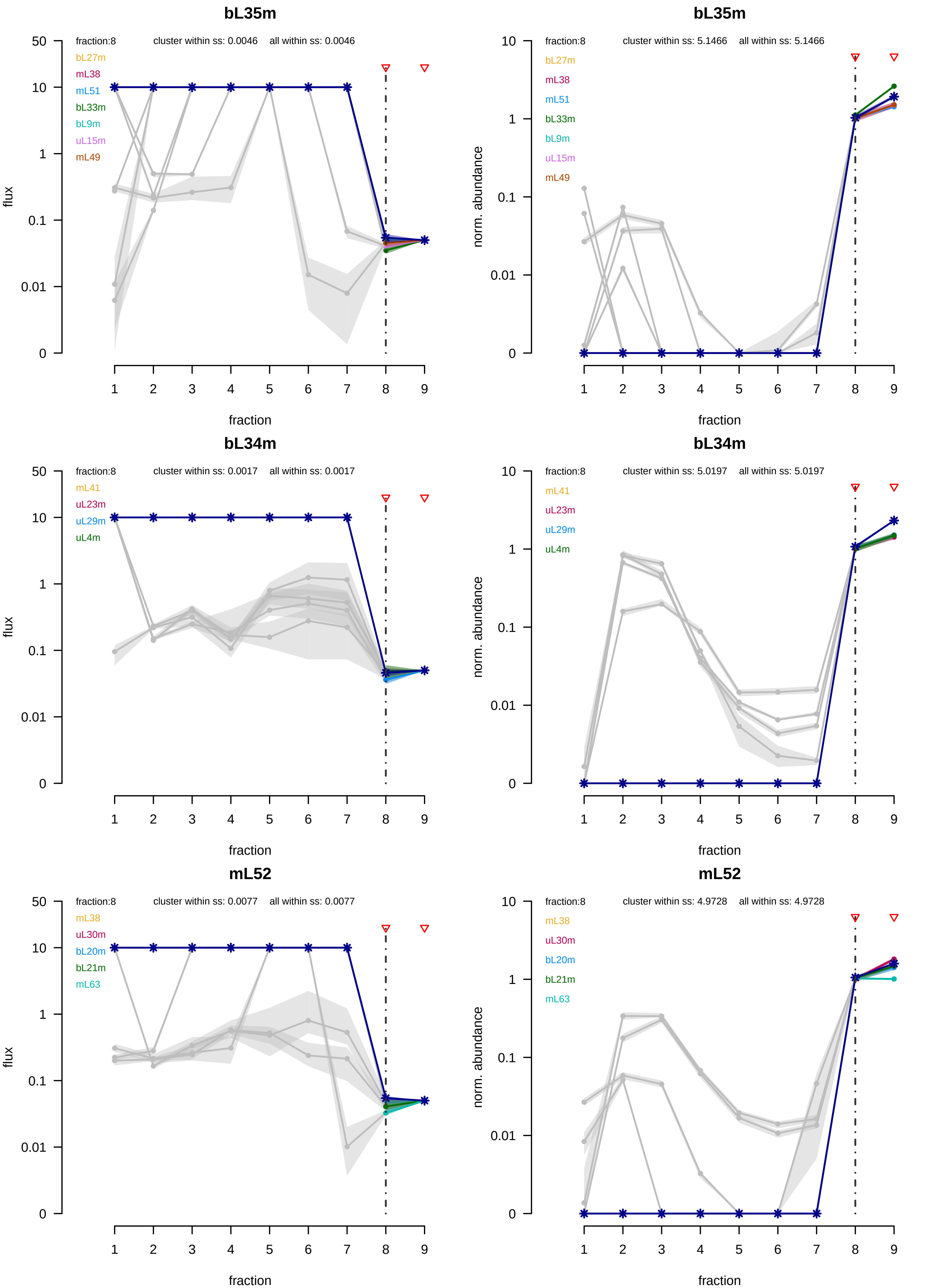
bL35m
fraction:8
cluster within ss: 0.0046
all within ss: 0.0046
bL27m
mL38
mL51
bL33m
bL9m
uL15m
mL49
flux
50
10
1
0.1
0.01
0
1
2
3
4
5
6
7
8
9
fraction
bL35m
fraction:8
cluster within ss: 5.1466
all within ss: 5.1466
bL27m
mL38
mL51
bL33m
bL9m
uL15m
mL49
norm. abundance
10
1
0.1
0.01
0
fraction
bL34m
fraction:8
cluster within ss: 0.0017
all within ss: 0.0017
mL41
uL23m
uL29m
uL4m
flux
fraction
bL34m
fraction:8
cluster within ss: 5.0197
all within ss: 5.0197
mL41
uL23m
uL29m
uL4m
norm. abundance
fraction
mL52
fraction:8
cluster within ss: 0.0077
all within ss: 0.0077
mL38
uL30m
bL20m
bL21m
mL63
flux
fraction
mL52
fraction:8
cluster within ss: 4.9728
all within ss: 4.9728
mL38
uL30m
bL20m
bL21m
mL63
norm. abundance
fraction

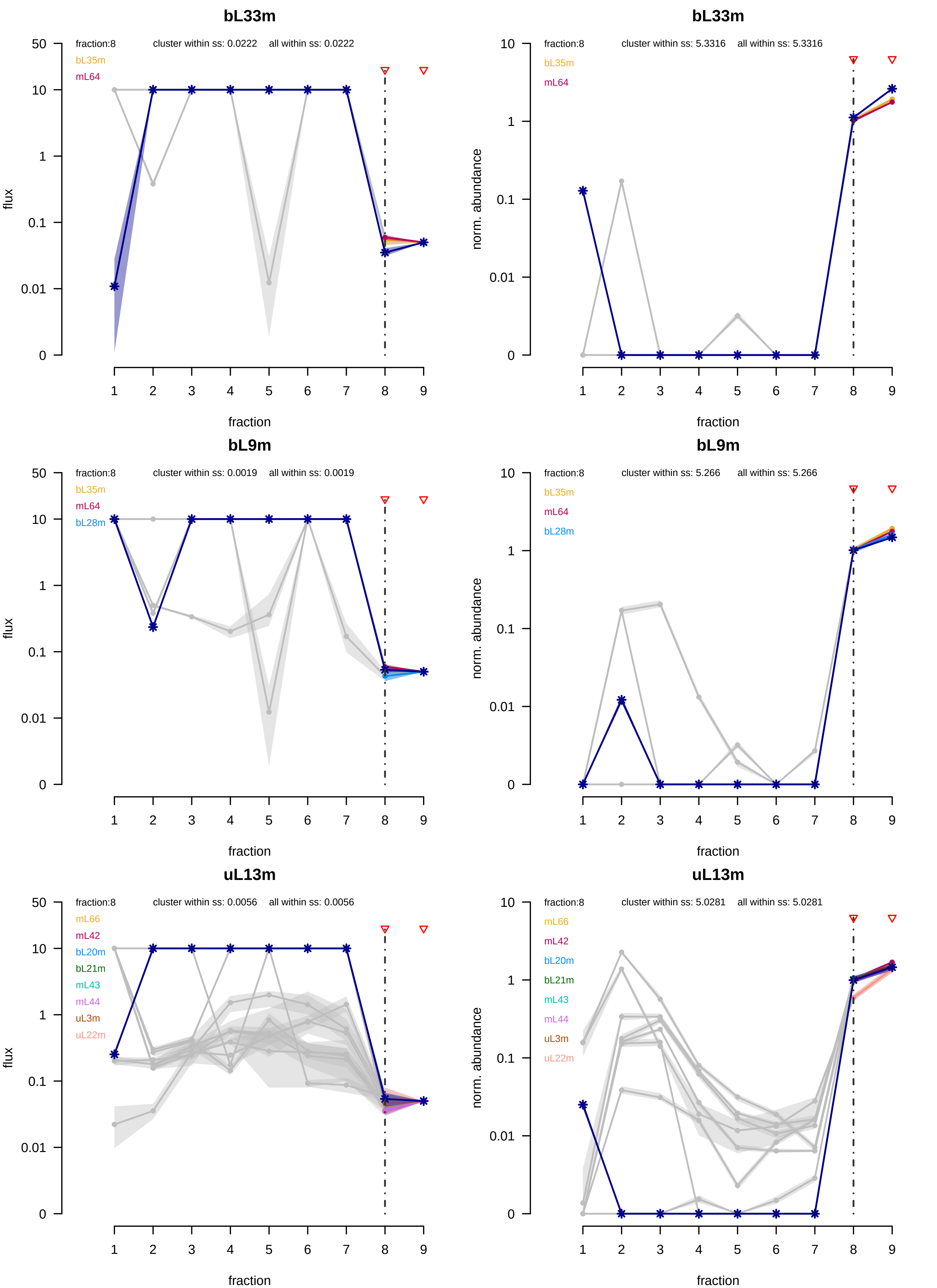

bL33m
fraction:8
cluster within ss: 0.0222
all within ss: 0.0222
bL35m
mL64
flux
50
10
1
0.1
0.01
0
fraction
1 2 3 4 5 6 7 8 9
bL33m
fraction:8
cluster within ss: 5.3316
all within ss: 5.3316
bL35m
mL64
norm. abundance
10
1
0.1
0.01
0
fraction
1 2 3 4 5 6 7 8 9
bL9m
fraction:8
cluster within ss: 0.0019
all within ss: 0.0019
bL35m
mL64
bL28m
flux
50
10
1
0.1
0.01
0
fraction
1 2 3 4 5 6 7 8 9
bL9m
fraction:8
cluster within ss: 5.266
all within ss: 5.266
bL35m
mL64
bL28m
norm. abundance
10
1
0.1
0.01
0
fraction
1 2 3 4 5 6 7 8 9
uL13m
fraction:8
cluster within ss: 0.0056
all within ss: 0.0056
mL66
mL42
bL20m
bL21m
mL43
mL44
uL3m
uL22m
flux
50
10
1
0.1
0.01
0
fraction
1 2 3 4 5 6 7 8 9
uL13m
fraction:8
cluster within ss: 5.0281
all within ss: 5.0281
mL66
mL42
bL20m
bL21m
mL43
mL44
uL3m
uL22m
norm. abundance
10
1
0.1
0.01
0
fraction
1 2 3 4 5 6 7 8 9

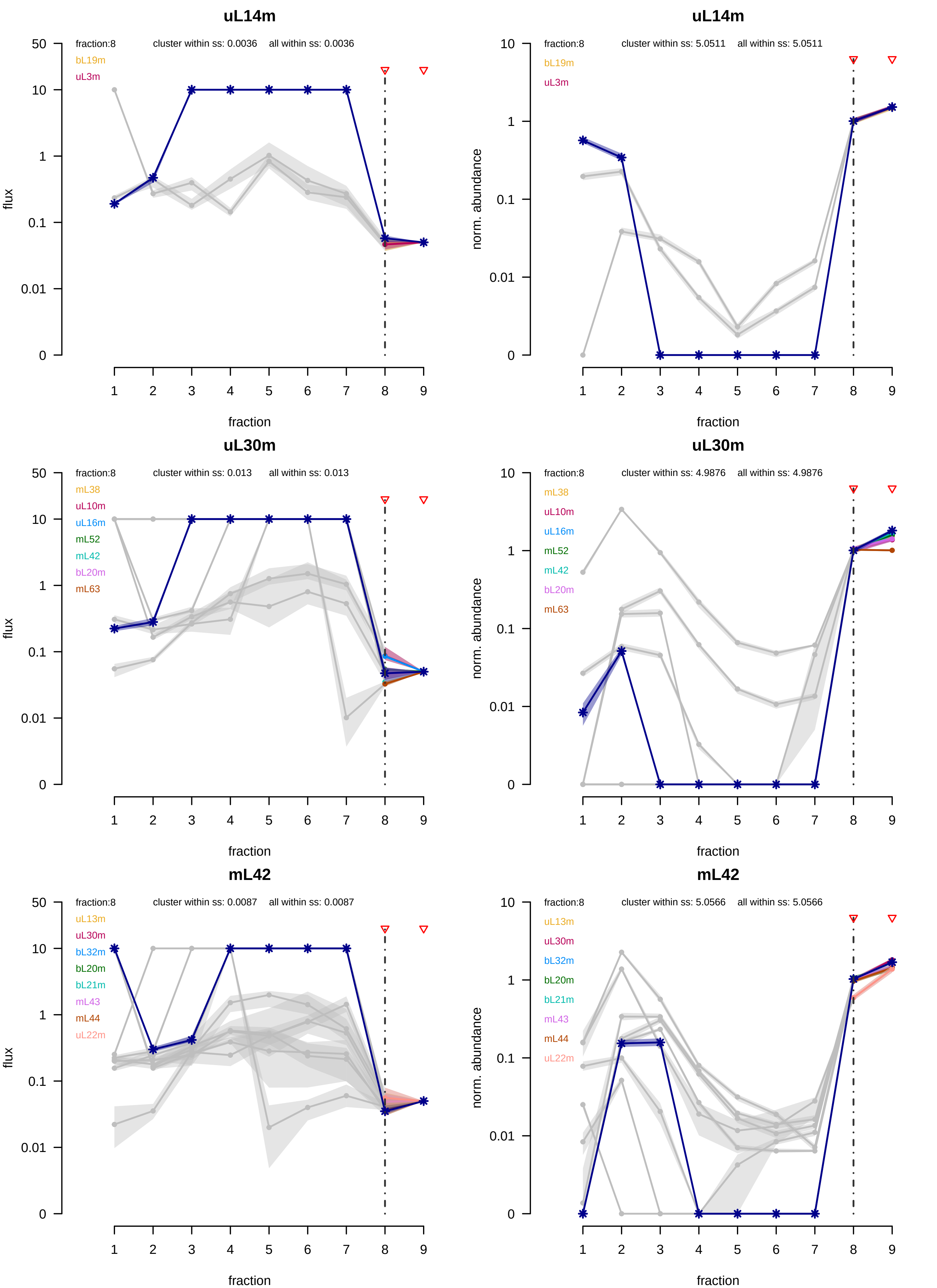
uL14m
fraction:8
cluster within ss: 0.0036
all within ss: 0.0036
bL19m
uL3m
flux
50
10
1
0.1
0.01
0
fraction
1 2 3 4 5 6 7 8 9
uL14m
fraction:8
cluster within ss: 5.0511
all within ss: 5.0511
bL19m
uL3m
norm. abundance
10
1
0.1
0.01
0
fraction
1 2 3 4 5 6 7 8 9
uL30m
fraction:8
cluster within ss: 0.013
all within ss: 0.013
mL38
uL10m
uL16m
mL52
mL42
bL20m
mL63
flux
50
10
1
0.1
0.01
0
fraction
1 2 3 4 5 6 7 8 9
uL30m
fraction:8
cluster within ss: 4.9876
all within ss: 4.9876
mL38
uL10m
uL16m
mL52
mL42
bL20m
mL63
norm. abundance
10
1
0.1
0.01
0
fraction
1 2 3 4 5 6 7 8 9
mL42
fraction:8
cluster within ss: 0.0087
all within ss: 0.0087
uL13m
uL30m
bL32m
bL20m
bL21m
mL43
mL44
uL22m
flux
50
10
1
0.1
0.01
0
fraction
1 2 3 4 5 6 7 8 9
mL42
fraction:8
cluster within ss: 5.0566
all within ss: 5.0566
uL13m
uL30m
bL32m
bL20m
bL21m
mL43
mL44
uL22m
norm. abundance
10
1
0.1
0.01
0
fraction
1 2 3 4 5 6 7 8 9

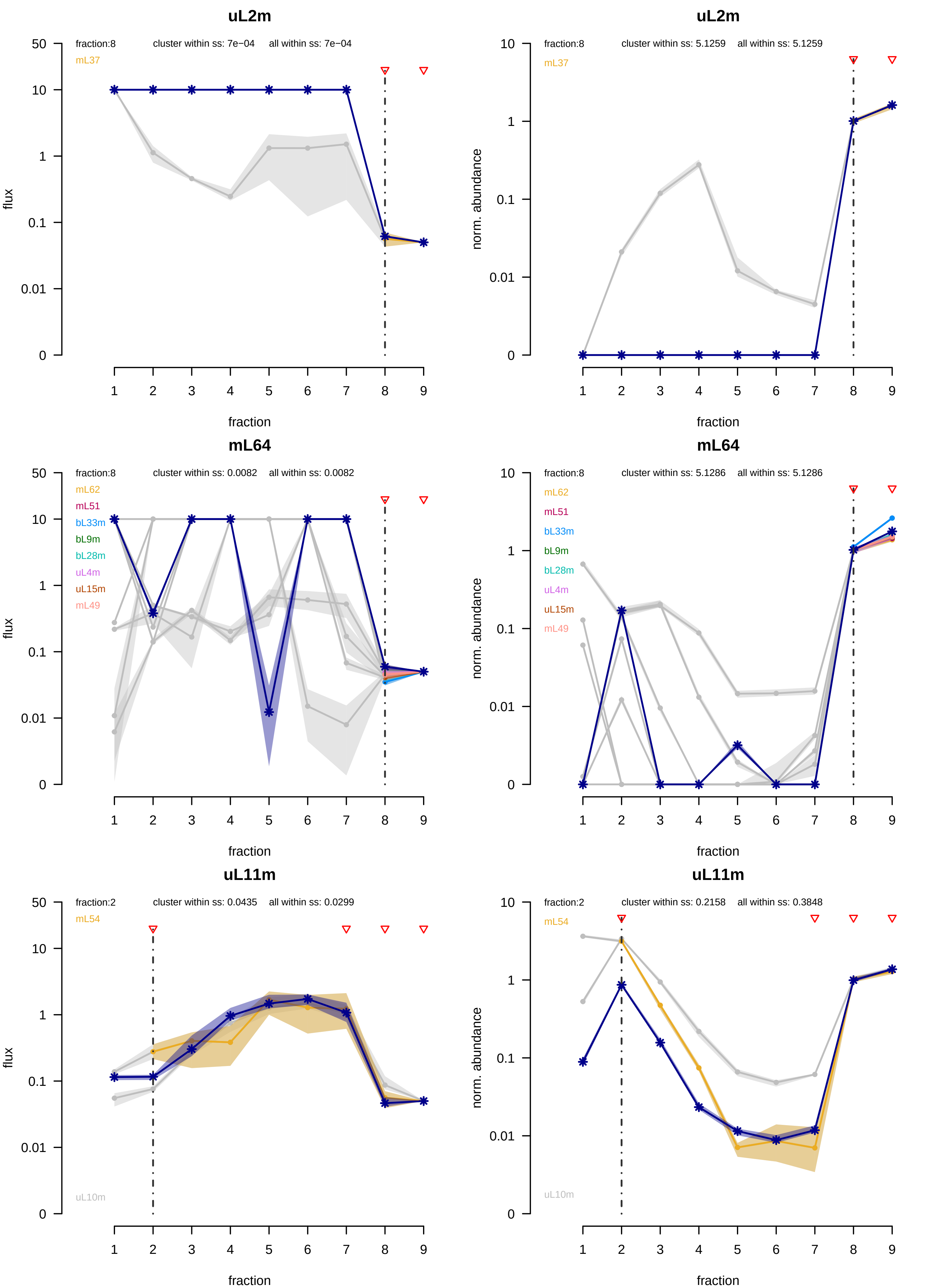

uL2m
fraction:8
cluster within ss: 7e−04
all within ss: 7e−04
mL37
flux
fraction
uL2m
fraction:8
cluster within ss: 5.1259
all within ss: 5.1259
mL37
norm. abundance
fraction
mL64
fraction:8
cluster within ss: 0.0082
all within ss: 0.0082
mL62
mL51
bL33m
bL9m
bL28m
uL4m
uL15m
mL49
flux
fraction
mL64
fraction:8
cluster within ss: 5.1286
all within ss: 5.1286
mL62
mL51
bL33m
bL9m
bL28m
uL4m
uL15m
mL49
norm. abundance
fraction
uL11m
fraction:2
cluster within ss: 0.0435
all within ss: 0.0299
mL54
uL10m
flux
fraction
uL11m
fraction:2
cluster within ss: 0.2158
all within ss: 0.3848
mL54
uL10m
norm. abundance
fraction

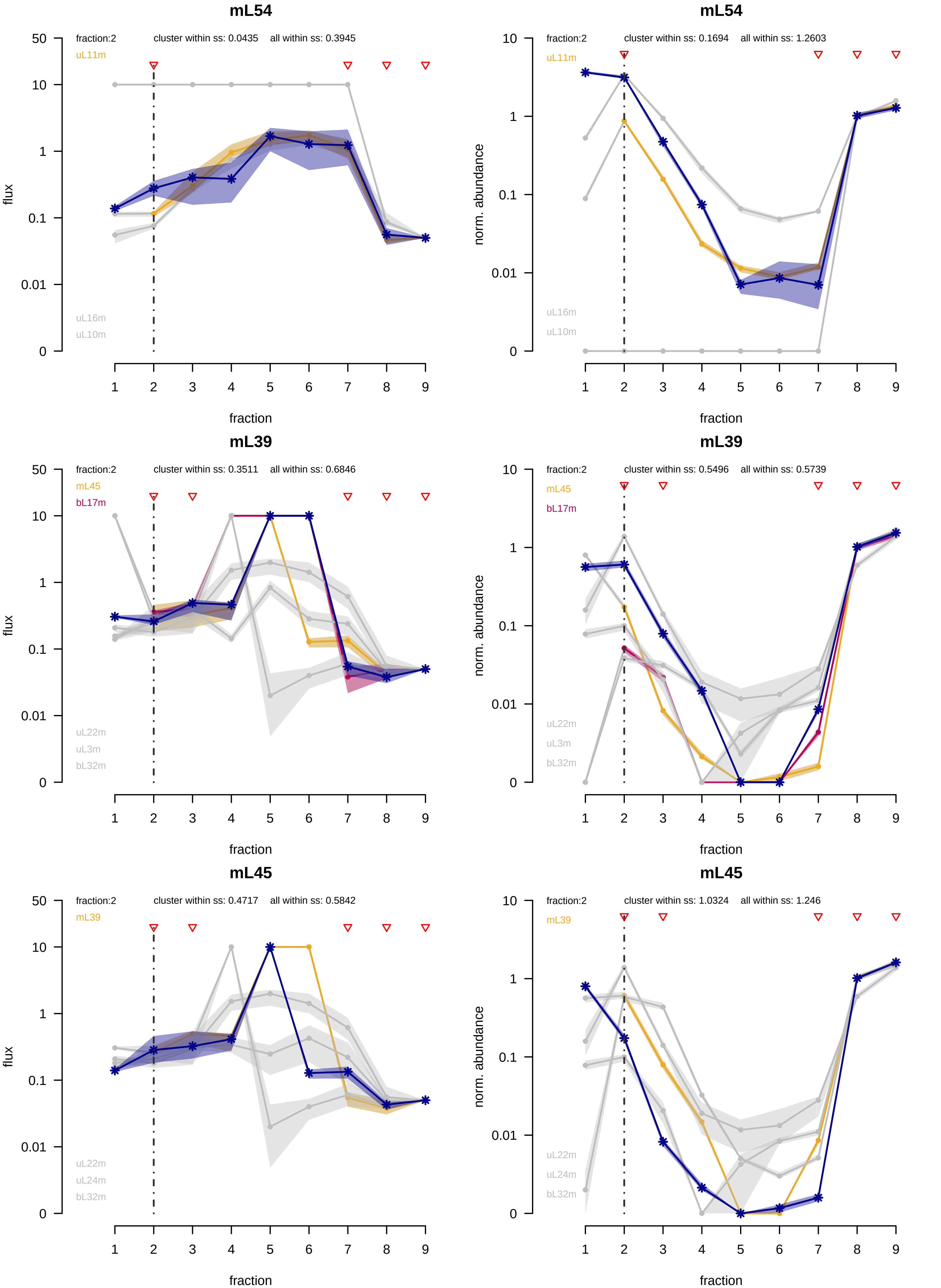

mL54
fraction:2
cluster within ss: 0.0435
all within ss: 0.3945
uL11m
uL16m
uL10m
flux
50
10
1
0.1
0.01
0
1 2 3 4 5 6 7 8 9
fraction
mL54
fraction:2
cluster within ss: 0.1694
all within ss: 1.2603
uL11m
uL16m
uL10m
norm. abundance
10
1
0.1
0.01
0
1 2 3 4 5 6 7 8 9
fraction
mL39
fraction:2
cluster within ss: 0.3511
all within ss: 0.6846
mL45
bL17m
uL22m
uL3m
bL32m
flux
50
10
1
0.1
0.01
0
1 2 3 4 5 6 7 8 9
fraction
mL39
fraction:2
cluster within ss: 0.5496
all within ss: 0.5739
mL45
bL17m
uL22m
uL3m
bL32m
norm. abundance
10
1
0.1
0.01
0
1 2 3 4 5 6 7 8 9
fraction
mL45
fraction:2
cluster within ss: 0.4717
all within ss: 0.5842
mL39
uL22m
uL24m
bL32m
flux
50
10
1
0.1
0.01
0
1 2 3 4 5 6 7 8 9
fraction
mL45
fraction:2
cluster within ss: 1.0324
all within ss: 1.246
mL39
uL22m
uL24m
bL32m
norm. abundance
10
1
0.1
0.01
0
1 2 3 4 5 6 7 8 9
fraction

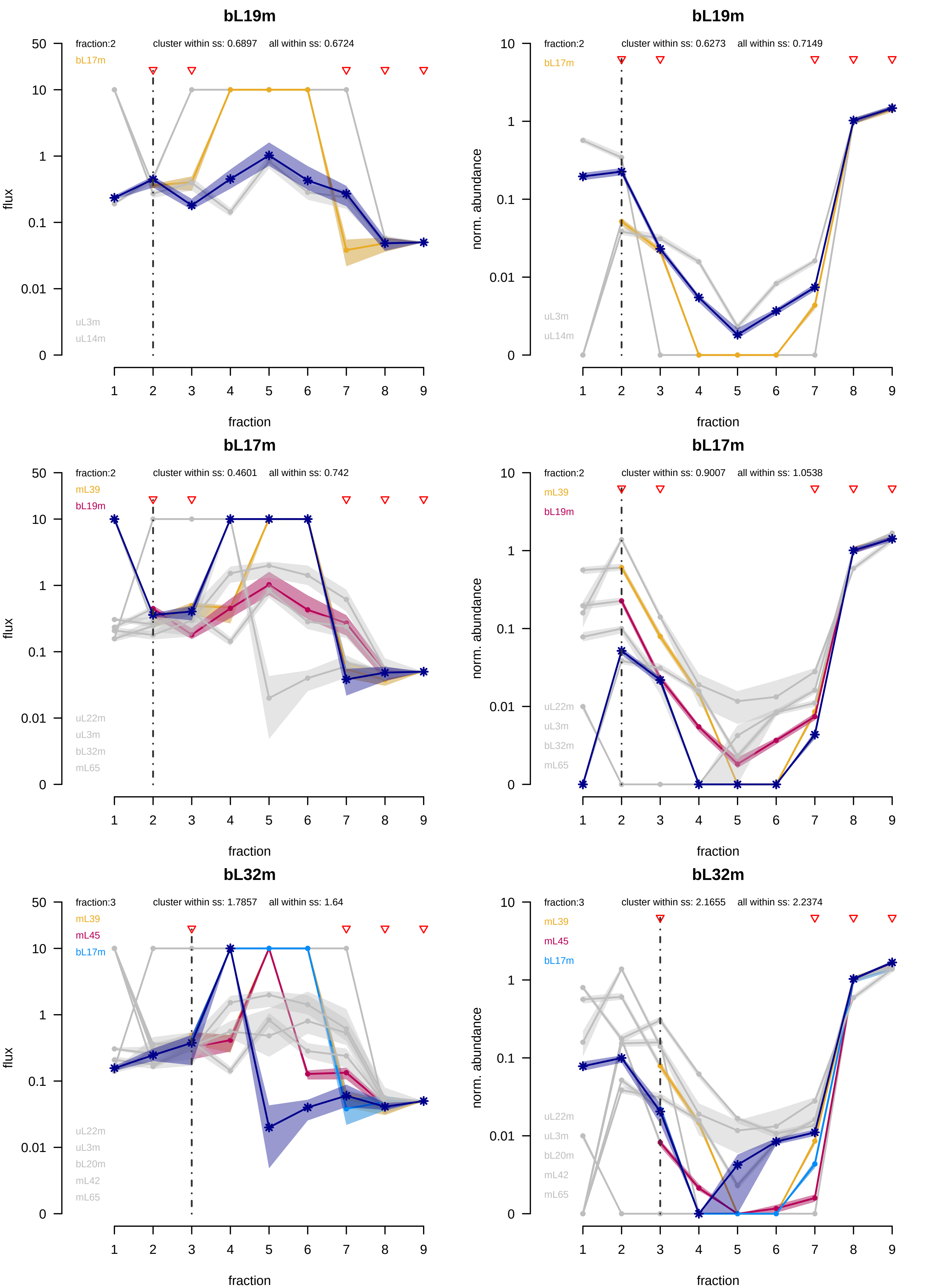

bL19m
fraction:2
cluster within ss: 0.6897
all within ss: 0.6724
bL17m
uL3m
uL14m
flux
fraction
bL19m
fraction:2
cluster within ss: 0.6273
all within ss: 0.7149
bL17m
uL3m
uL14m
norm. abundance
fraction
bL17m
fraction:2
cluster within ss: 0.4601
all within ss: 0.742
mL39
bL19m
uL22m
uL3m
bL32m
mL65
flux
fraction
bL17m
fraction:2
cluster within ss: 0.9007
all within ss: 1.0538
mL39
bL19m
uL22m
uL3m
bL32m
mL65
norm. abundance
fraction
bL32m
fraction:3
cluster within ss: 1.7857
all within ss: 1.64
mL39
mL45
bL17m
uL22m
uL3m
bL20m
mL42
mL65
flux
fraction
bL32m
fraction:3
cluster within ss: 2.1655
all within ss: 2.2374
mL39
mL45
bL17m
uL22m
uL3m
bL20m
mL42
mL65
norm. abundance
fraction

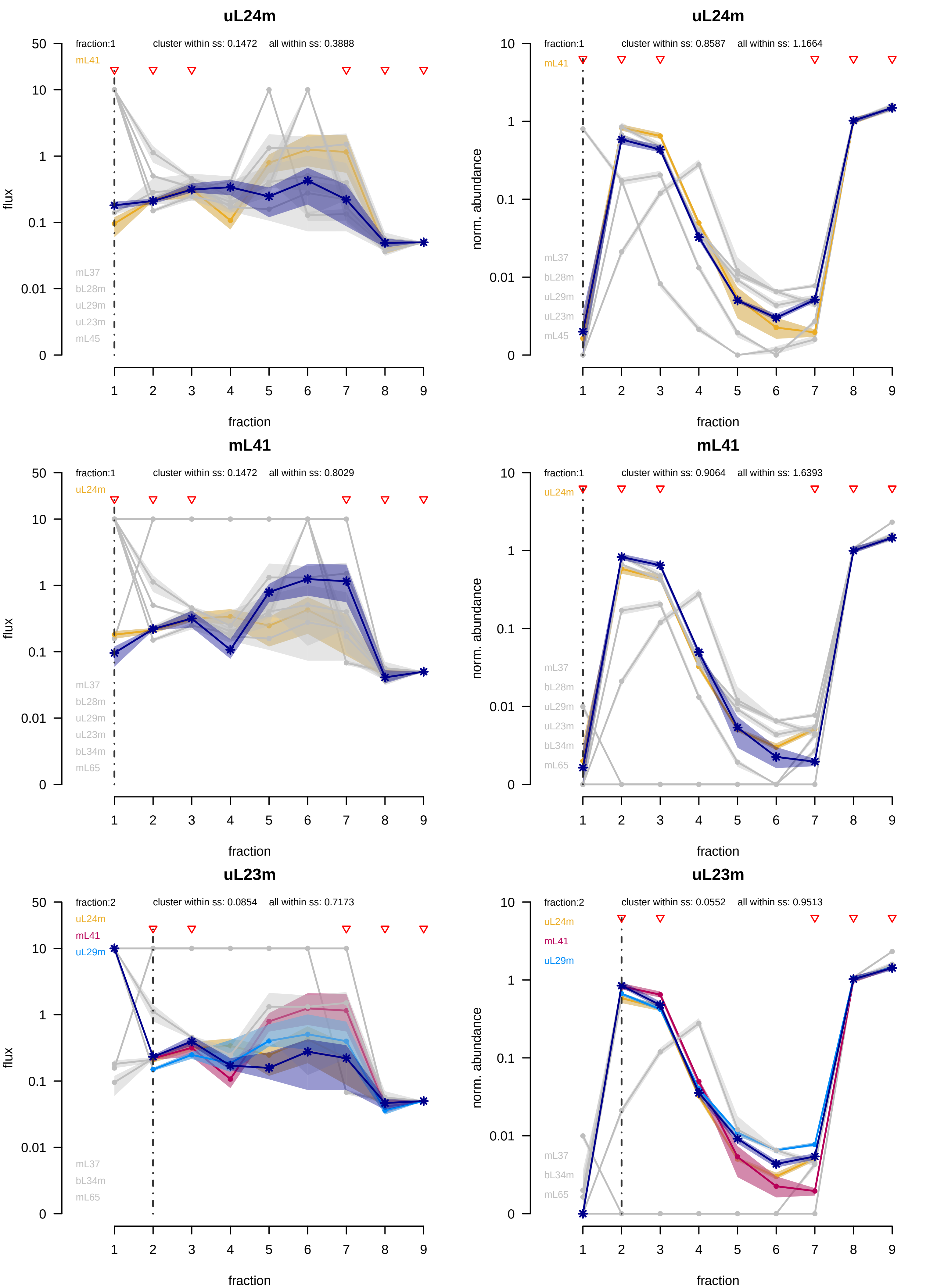
uL24m
fraction:1
cluster within ss: 0.1472
all within ss: 0.3888
mL41
mL37
bL28m
uL29m
uL23m
mL45
flux
fraction
uL24m
fraction:1
cluster within ss: 0.8587
all within ss: 1.1664
mL41
mL37
bL28m
uL29m
uL23m
mL45
norm. abundance
fraction
mL41
fraction:1
cluster within ss: 0.1472
all within ss: 0.8029
uL24m
mL37
bL28m
uL29m
uL23m
bL34m
mL65
flux
fraction
mL41
fraction:1
cluster within ss: 0.9064
all within ss: 1.6393
uL24m
mL37
bL28m
uL29m
uL23m
bL34m
mL65
norm. abundance
fraction
uL23m
fraction:2
cluster within ss: 0.0854
all within ss: 0.7173
uL24m
mL41
uL29m
mL37
bL34m
mL65
flux
fraction
uL23m
fraction:2
cluster within ss: 0.0552
all within ss: 0.9513
uL24m
mL41
uL29m
mL37
bL34m
mL65
norm. abundance
fraction

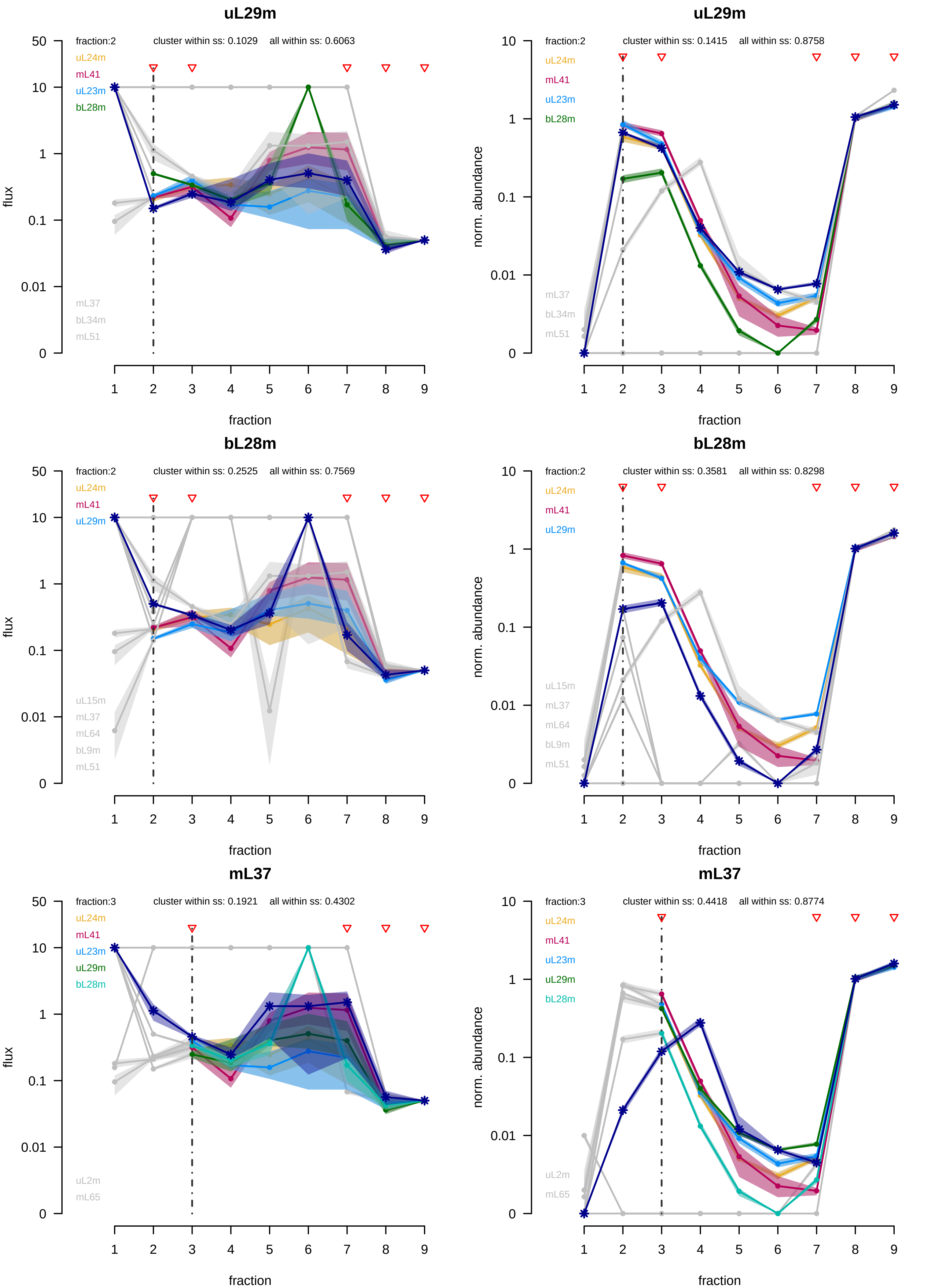
uL29m
fraction:2
cluster within ss: 0.1029
all within ss: 0.6063
uL24m
mL41
uL23m
bL28m
mL37
bL34m
mL51
flux
fraction
uL29m
fraction:2
cluster within ss: 0.1415
all within ss: 0.8758
uL24m
mL41
uL23m
bL28m
mL37
bL34m
mL51
norm. abundance
fraction
bL28m
fraction:2
cluster within ss: 0.2525
all within ss: 0.7569
uL24m
mL41
uL29m
uL15m
mL37
mL64
bL9m
mL51
flux
fraction
bL28m
fraction:2
cluster within ss: 0.3581
all within ss: 0.8298
uL24m
mL41
uL29m
uL15m
mL37
mL64
bL9m
mL51
norm. abundance
fraction
mL37
fraction:3
cluster within ss: 0.1921
all within ss: 0.4302
uL24m
mL41
uL23m
uL29m
bL28m
uL2m
mL65
flux
fraction
mL37
fraction:3
cluster within ss: 0.4418
all within ss: 0.8774
uL24m
mL41
uL23m
uL29m
bL28m
uL2m
mL65
norm. abundance
fraction

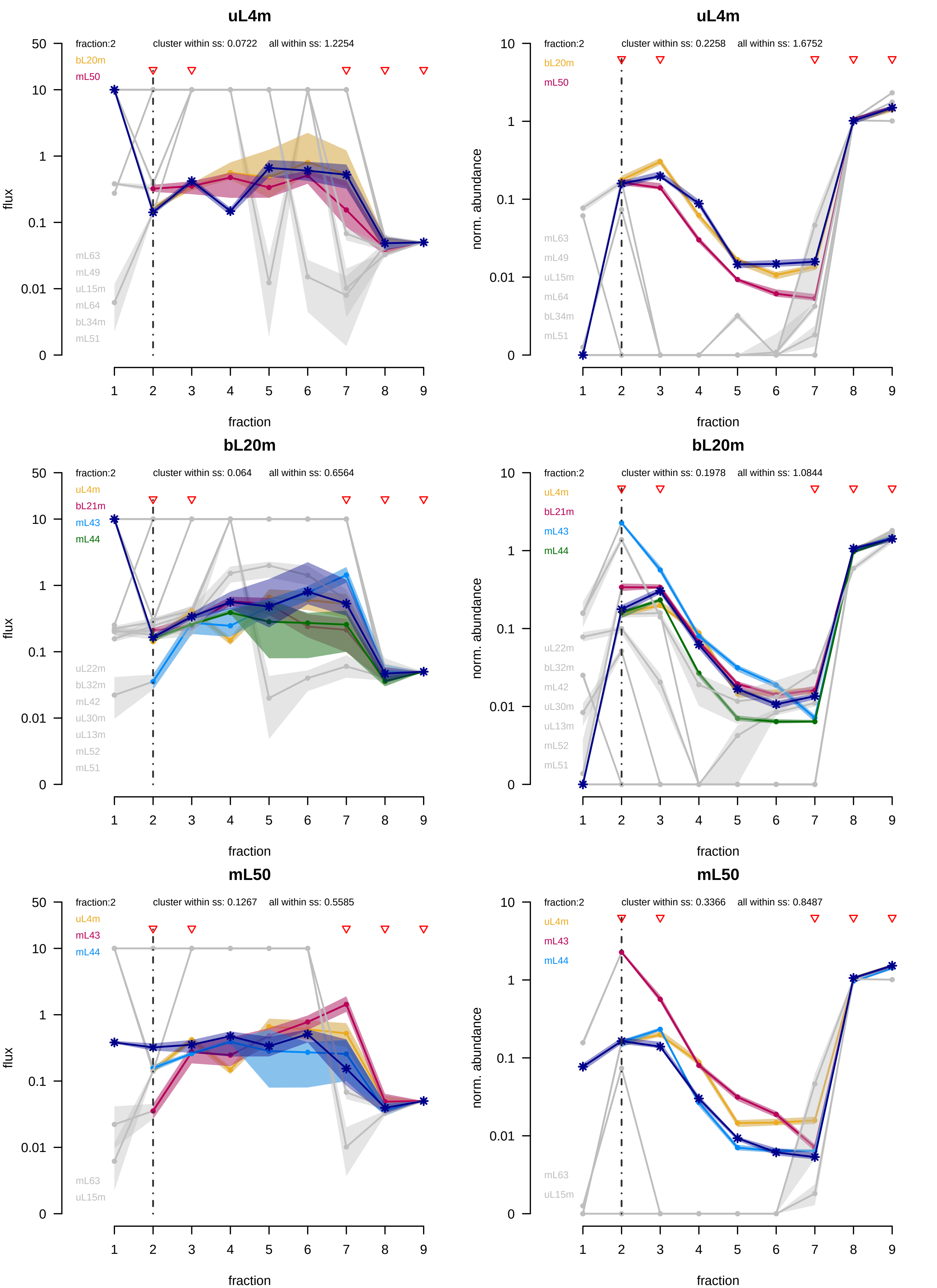
uL4m
fraction:2
cluster within ss: 0.0722
all within ss: 1.2254
bL20m
mL50
mL63
mL49
uL15m
mL64
bL34m
mL51
flux
fraction
uL4m
fraction:2
cluster within ss: 0.2258
all within ss: 1.6752
bL20m
mL50
mL63
mL49
uL15m
mL64
bL34m
mL51
norm. abundance
fraction
bL20m
fraction:2
cluster within ss: 0.064
all within ss: 0.6564
uL4m
bL21m
mL43
mL44
uL22m
bL32m
mL42
uL30m
uL13m
mL52
mL51
flux
fraction
bL20m
fraction:2
cluster within ss: 0.1978
all within ss: 1.0844
uL4m
bL21m
mL43
mL44
uL22m
bL32m
mL42
uL30m
uL13m
mL52
mL51
norm. abundance
fraction
mL50
fraction:2
cluster within ss: 0.1267
all within ss: 0.5585
uL4m
mL43
mL44
mL63
uL15m
flux
fraction
mL50
fraction:2
cluster within ss: 0.3366
all within ss: 0.8487
uL4m
mL43
mL44
mL63
uL15m
norm. abundance
fraction

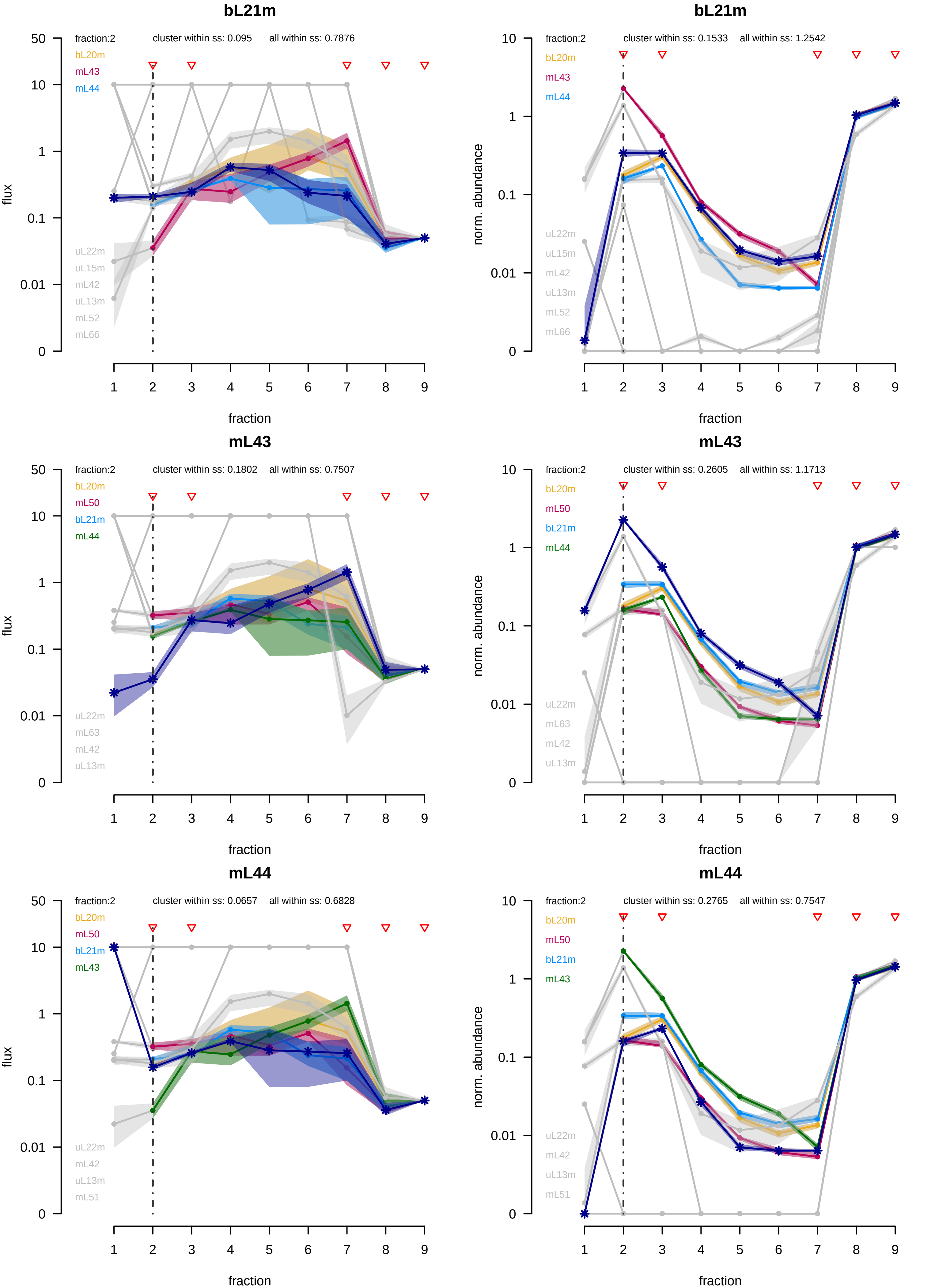
bL21m
fraction:2
cluster within ss: 0.095
all within ss: 0.7876
bL20m
mL43
mL44
uL22m
uL15m
mL42
uL13m
mL52
mL66
flux
fraction
bL21m
fraction:2
cluster within ss: 0.1533
all within ss: 1.2542
bL20m
mL43
mL44
uL22m
uL15m
mL42
uL13m
mL52
mL66
norm. abundance
fraction
mL43
fraction:2
cluster within ss: 0.1802
all within ss: 0.7507
bL20m
mL50
bL21m
mL44
uL22m
mL63
mL42
uL13m
flux
fraction
mL43
fraction:2
cluster within ss: 0.2605
all within ss: 1.1713
bL20m
mL50
bL21m
mL44
uL22m
mL63
mL42
uL13m
norm. abundance
fraction
mL44
fraction:2
cluster within ss: 0.0657
all within ss: 0.6828
bL20m
mL50
bL21m
mL43
uL22m
mL42
uL13m
mL51
flux
fraction
mL44
fraction:2
cluster within ss: 0.2765
all within ss: 0.7547
bL20m
mL50
bL21m
mL43
uL22m
mL42
uL13m
mL51
norm. abundance
fraction

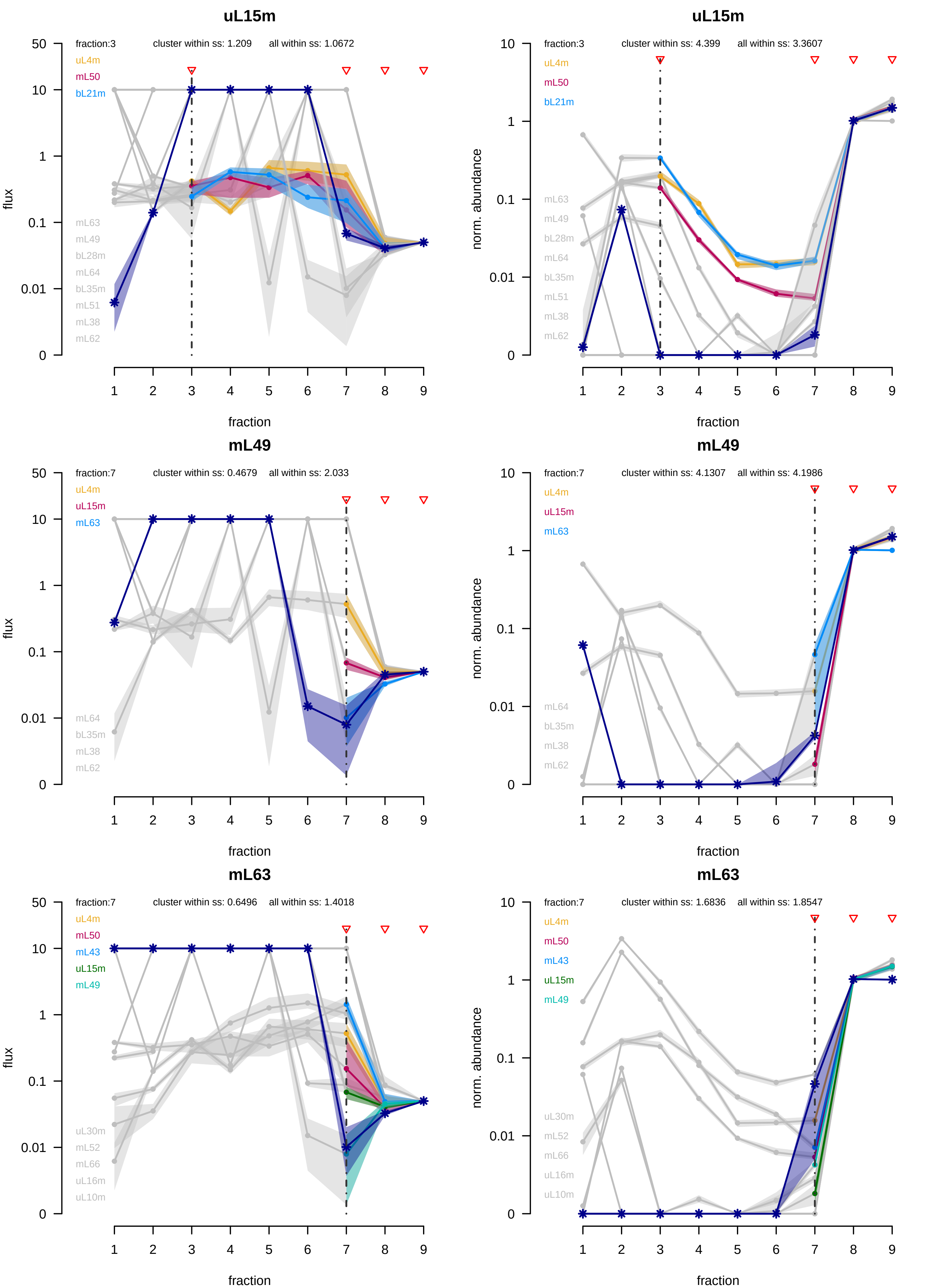
uL15m
fraction:3
cluster within ss: 1.209
all within ss: 1.0672
uL4m
mL50
bL21m
mL63
mL49
bL28m
mL64
bL35m
mL51
mL38
mL62
flux
fraction
uL15m
fraction:3
cluster within ss: 4.399
all within ss: 3.3607
uL4m
mL50
bL21m
mL63
mL49
bL28m
mL64
bL35m
mL51
mL38
mL62
norm. abundance
fraction
mL49
fraction:7
cluster within ss: 0.4679
all within ss: 2.033
uL4m
uL15m
mL63
mL64
bL35m
mL38
mL62
flux
fraction
mL49
fraction:7
cluster within ss: 4.1307
all within ss: 4.1986
uL4m
uL15m
mL63
mL64
bL35m
mL38
mL62
norm. abundance
fraction
mL63
fraction:7
cluster within ss: 0.6496
all within ss: 1.4018
uL4m
mL50
mL43
uL15m
mL49
uL30m
mL52
mL66
uL16m
uL10m
flux
fraction
mL63
fraction:7
cluster within ss: 1.6836
all within ss: 1.8547
uL4m
mL50
mL43
uL15m
mL49
uL30m
mL52
mL66
uL16m
uL10m
norm. abundance
fraction

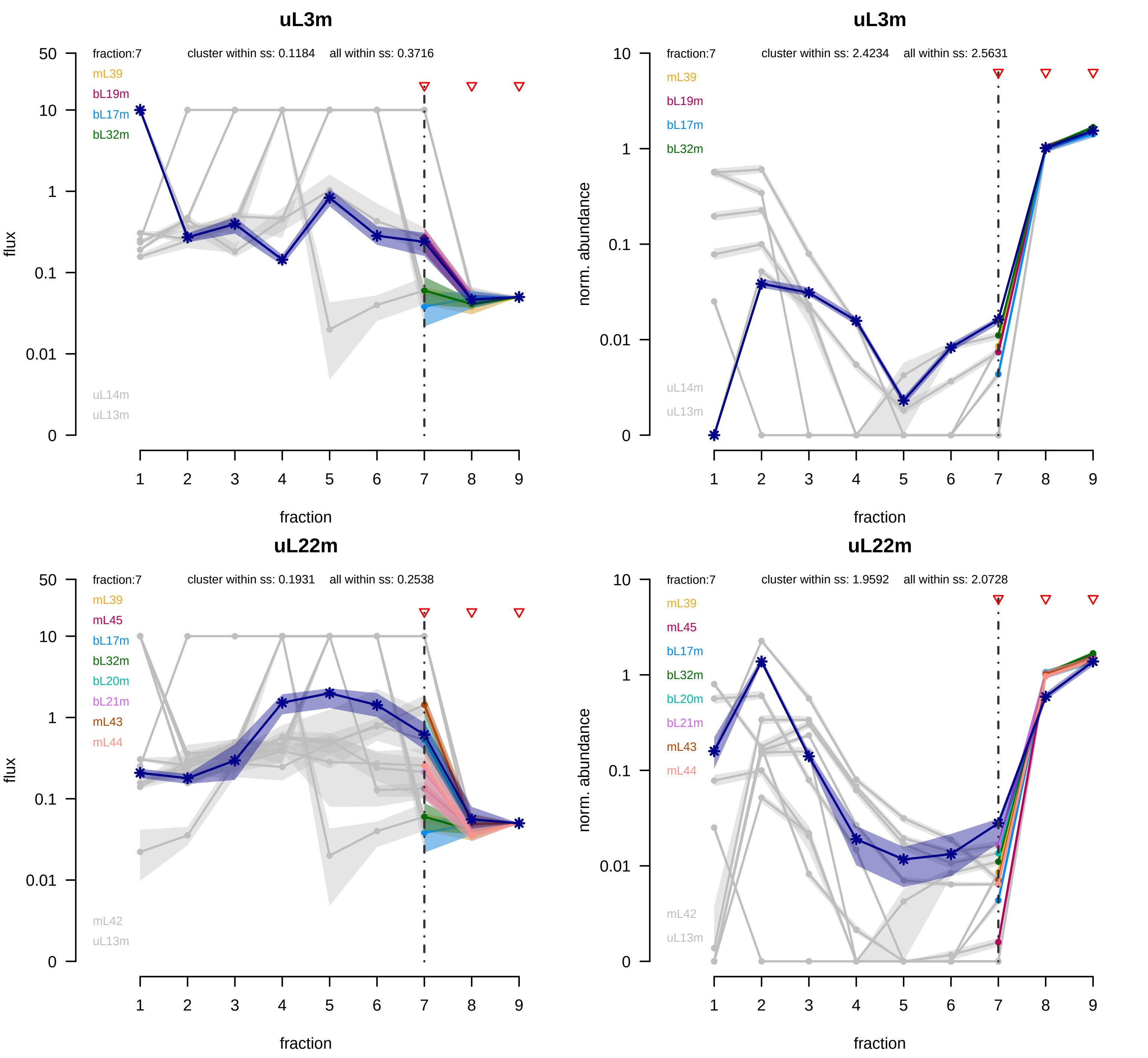

uL3m
fraction:7
cluster within ss: 0.1184
all within ss: 0.3716
mL39
bL19m
bL17m
bL32m
uL14m
uL13m
flux
fraction
uL3m
fraction:7
cluster within ss: 2.4234
all within ss: 2.5631
mL39
bL19m
bL17m
bL32m
uL14m
uL13m
norm. abundance
fraction
uL22m
fraction:7
cluster within ss: 0.1931
all within ss: 0.2538
mL39
mL45
bL17m
bL32m
bL20m
bL21m
mL43
mL44
mL42
uL13m
flux
fraction
uL22m
fraction:7
cluster within ss: 1.9592
all within ss: 2.0728
mL39
mL45
bL17m
bL32m
bL20m
bL21m
mL43
mL44
mL42
uL13m
norm. abundance
fraction
