# Supplementary material for: A roadmap for ribosome assembly in human mitochondria
Source: Nat Struct Mol Biol. 2024 Jul 11;31(12):1898–908. doi: 10.1038/s41594-024-01356-w (PMC11638073; doi:10.1038/s41594-024-01356-w)

Source Data 1_related to Main Fig.4a
EL#398 WT Gradient + RNA isolation
Rotor: SW41 Ti
Gradient: Sucrose 5-30%
Speed: 158.000xg
Time: 15h

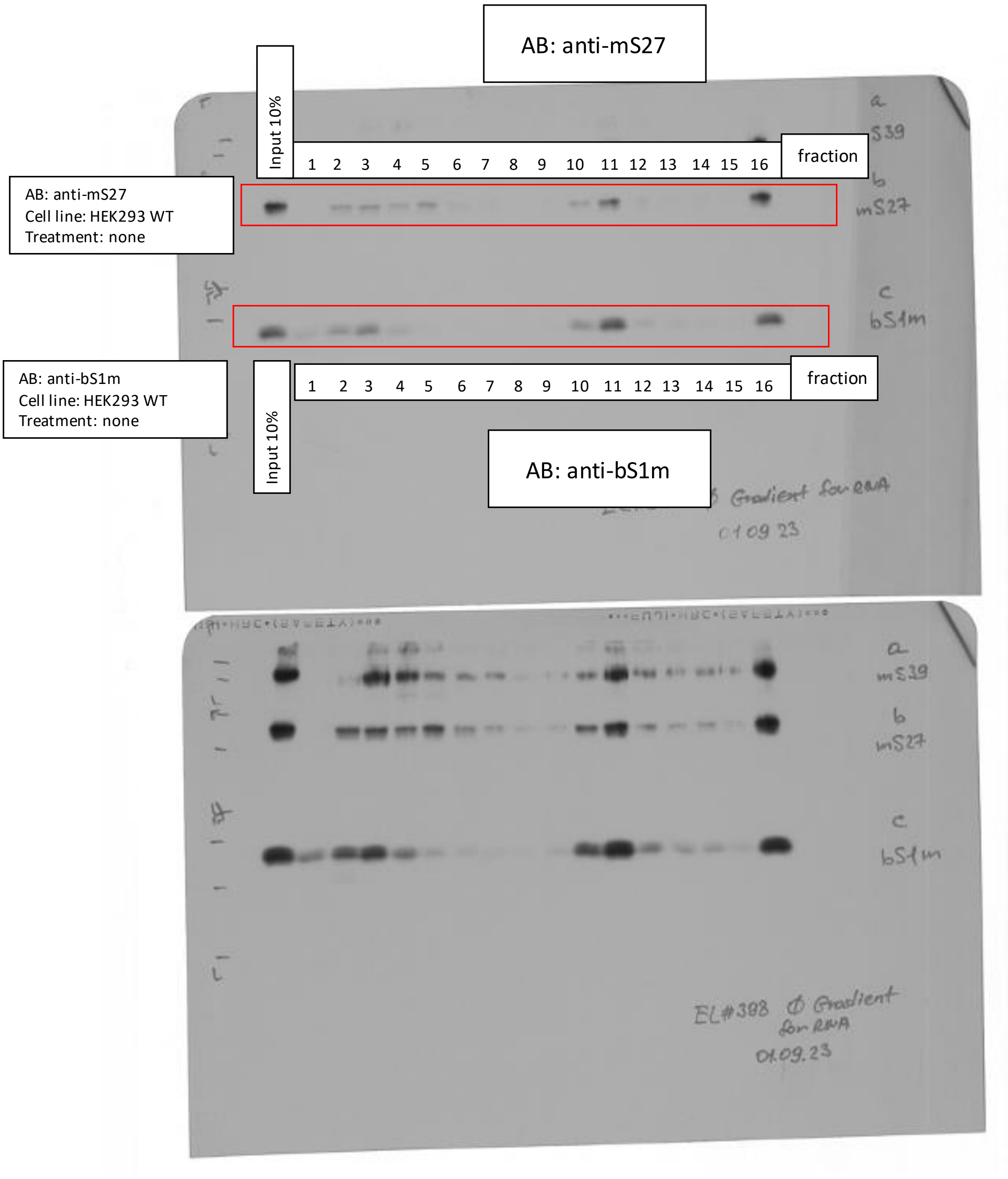

Source Data 1_related to Main Fig.4a
EL#398 WT Gradient + RNA isolation
Rotor: SW41 Ti
Gradient: Sucrose 5-30%
Speed: 158.000xg
Time: 15h

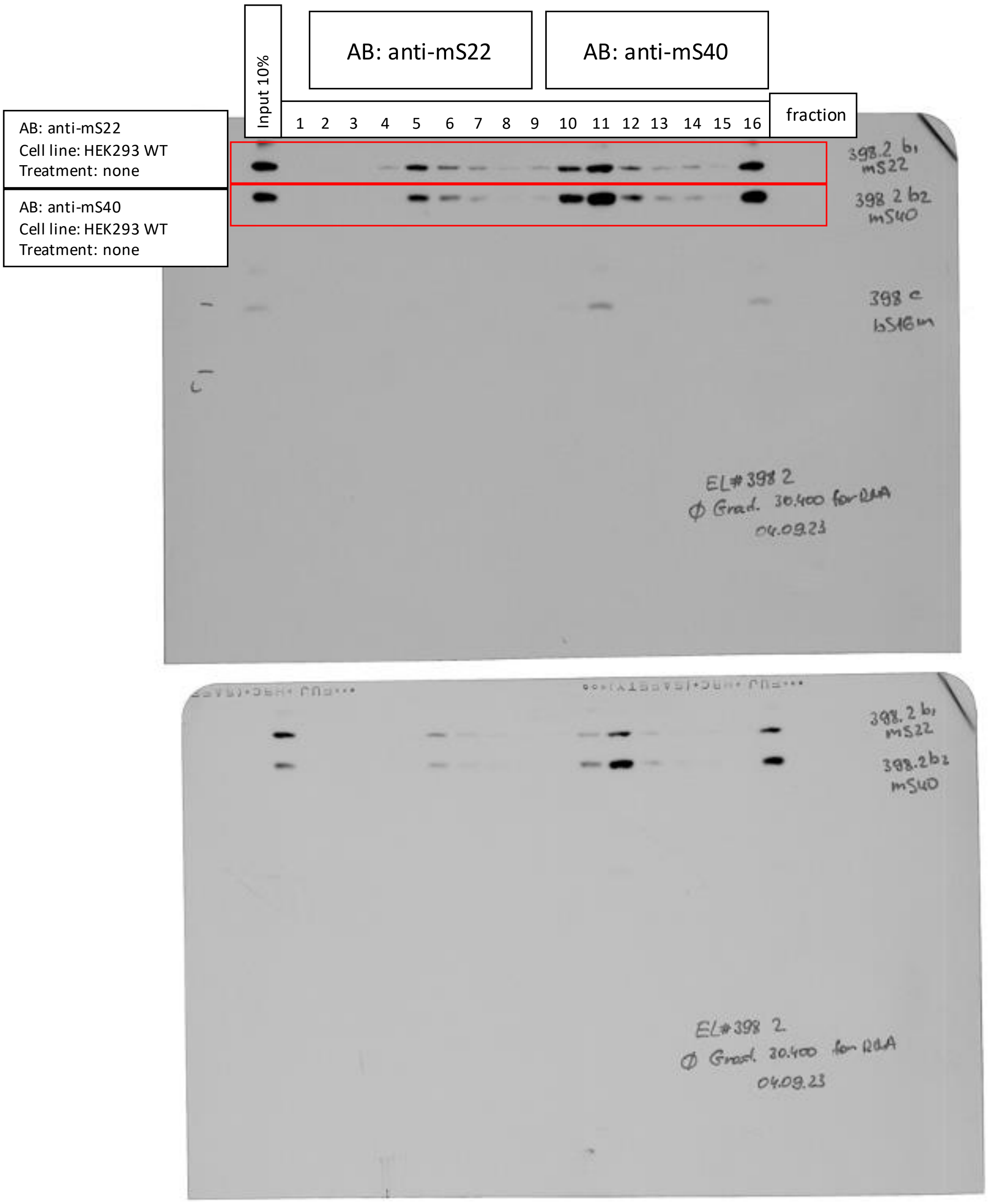

Source Data 1_related to Main Fig.4a
EL#398 WT Gradient + RNA isolation
Rotor: SW41 Ti
Gradient: Sucrose 5-30%
Speed: 158.000xg
Time: 15h

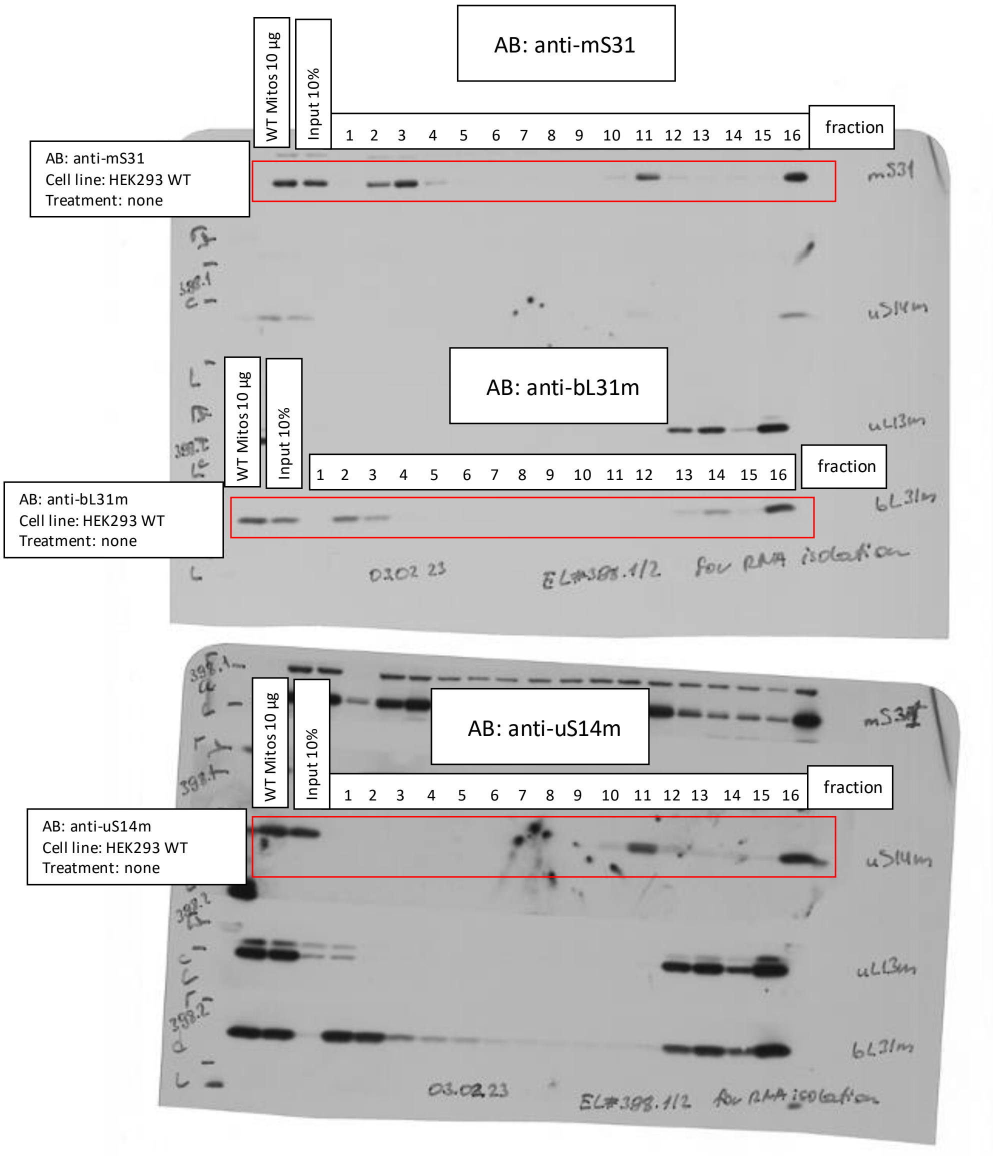

Source Data 1_related to Main Fig.4a
EL#398 WT Gradient + RNA isolation
Rotor: SW41 Ti
Gradient: Sucrose 5-30%
Speed: 158.000xg
Time: 15h

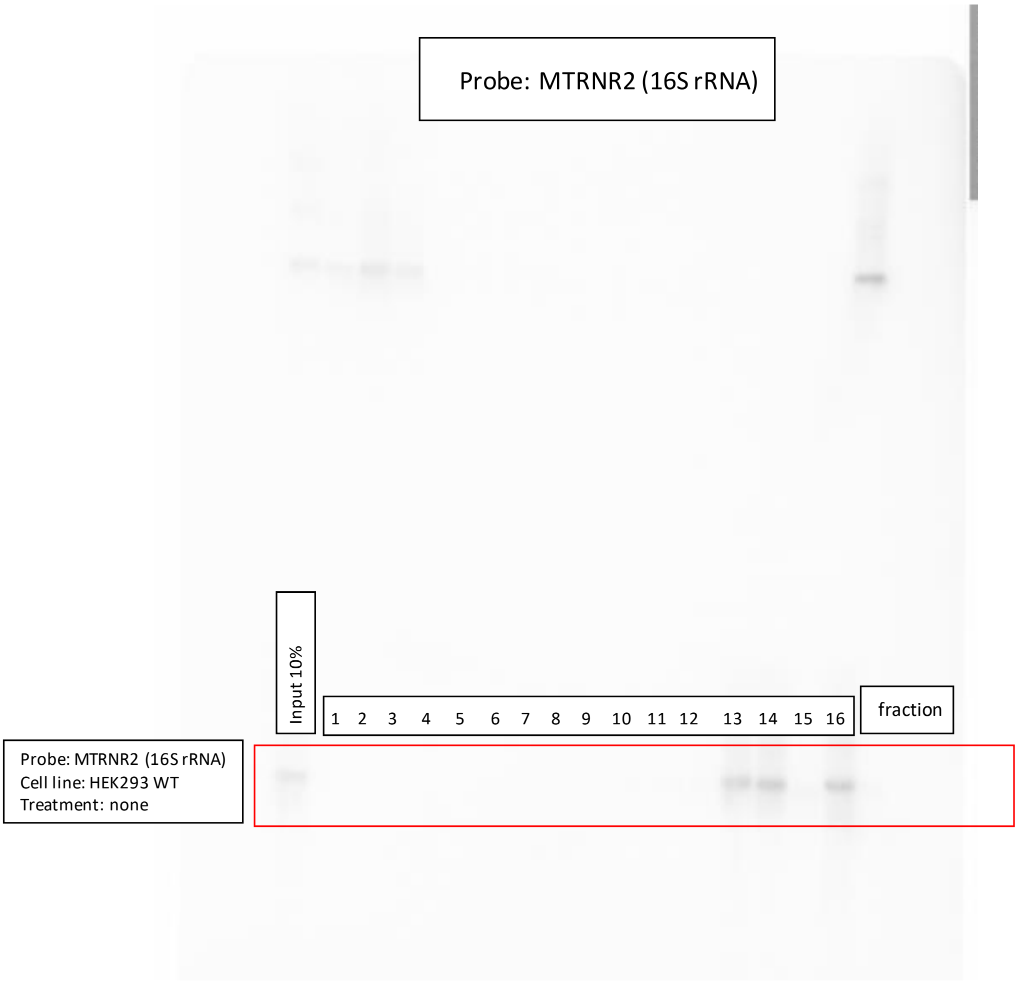

Source Data 1_related to Main Fig.4a
EL#398 WT Gradient + RNA isolation
Rotor: SW41 Ti
Gradient: Sucrose 5-30%
Speed: 158.000xg
Time: 15h

Probe: MTRNR1 (12S rRNA)

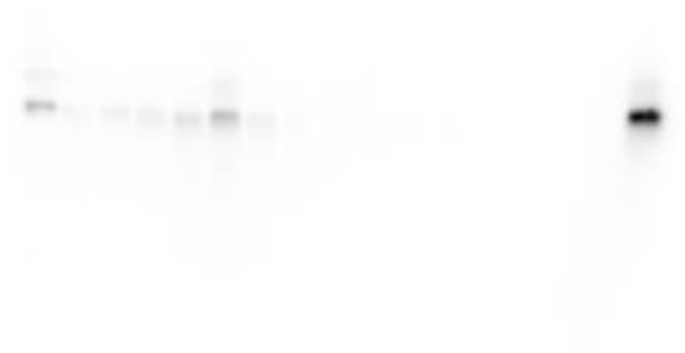

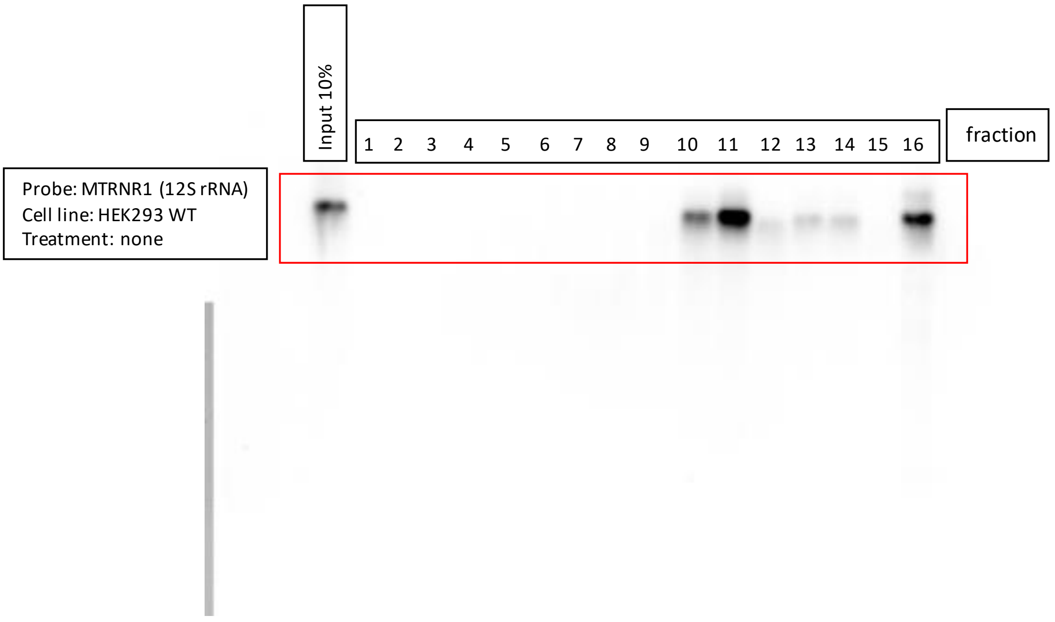

Source Data 1_related to Main Fig.4d
EL#450 bS1m-FLAG IP + EtBr treatment
Rotor: N/A
Gradient: N/A
Speed: N/A
Time: N/A

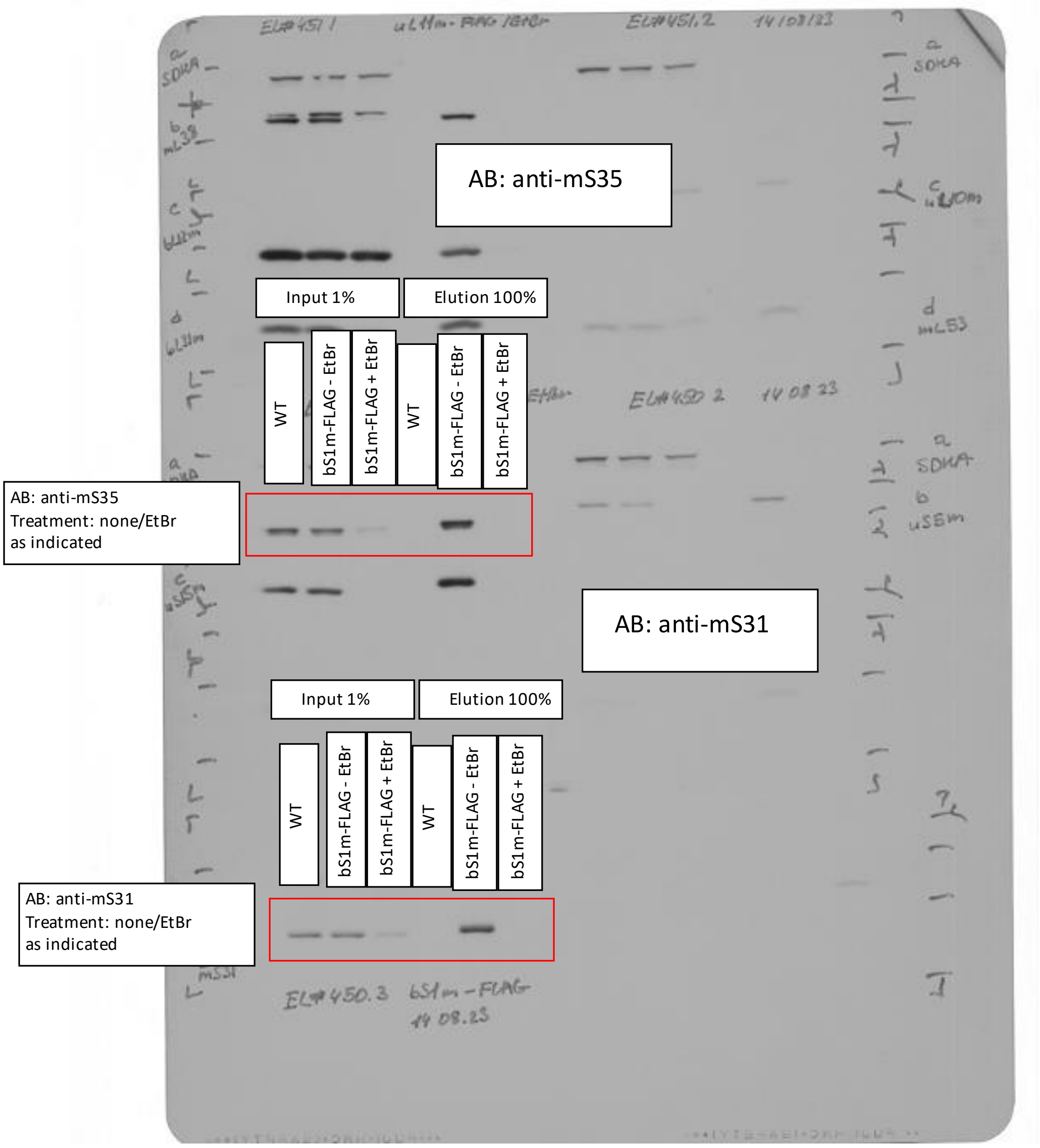

Source Data 1_related to Main Fig.4d
EL#450 bS1m-FLAG IP + EtBr treatment
Rotor: N/A
Gradient: N/A
Speed: N/A
Time: N/A

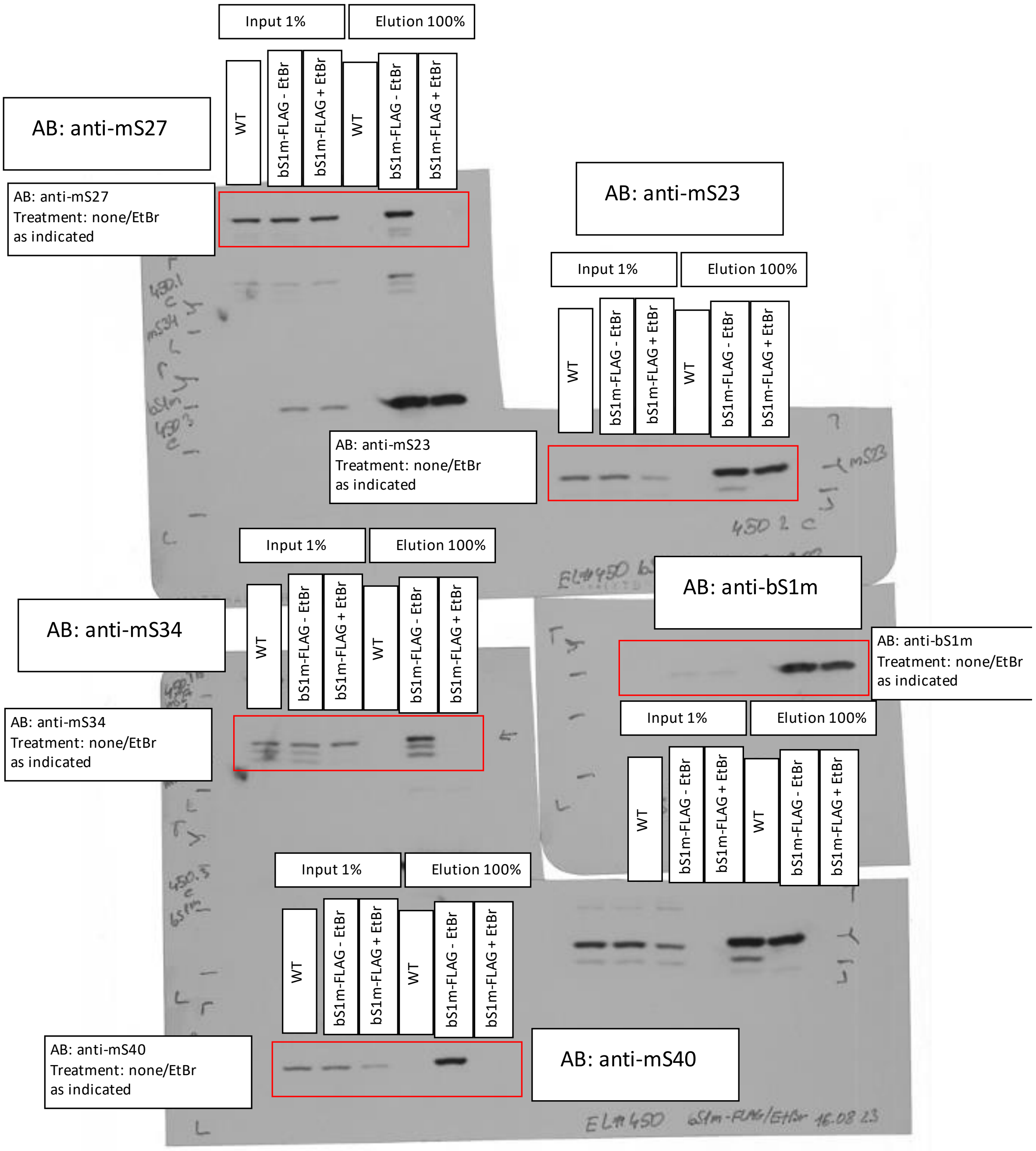

Source Data 1_related to Main Fig.4d
EL#450 bS1m-FLAG IP + EtBr treatment
Rotor: N/A
Gradient: N/A
Speed: N/A
Time: N/A

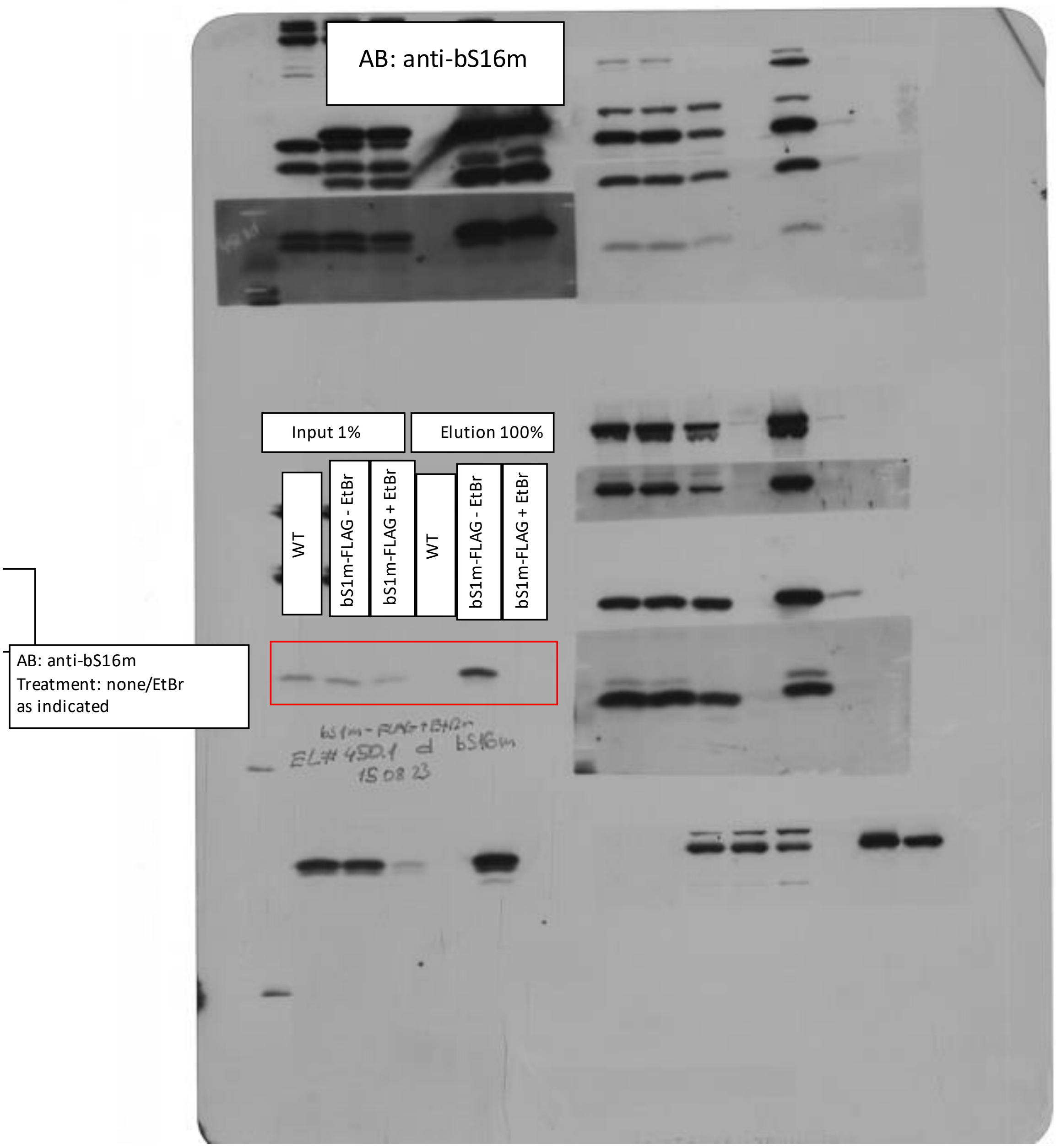

Source Data 1_related to Main Fig.4d
EL#450 bS1m-FLAG IP + EtBr treatment
Rotor: N/A
Gradient: N/A
Speed: N/A
Time: N/A

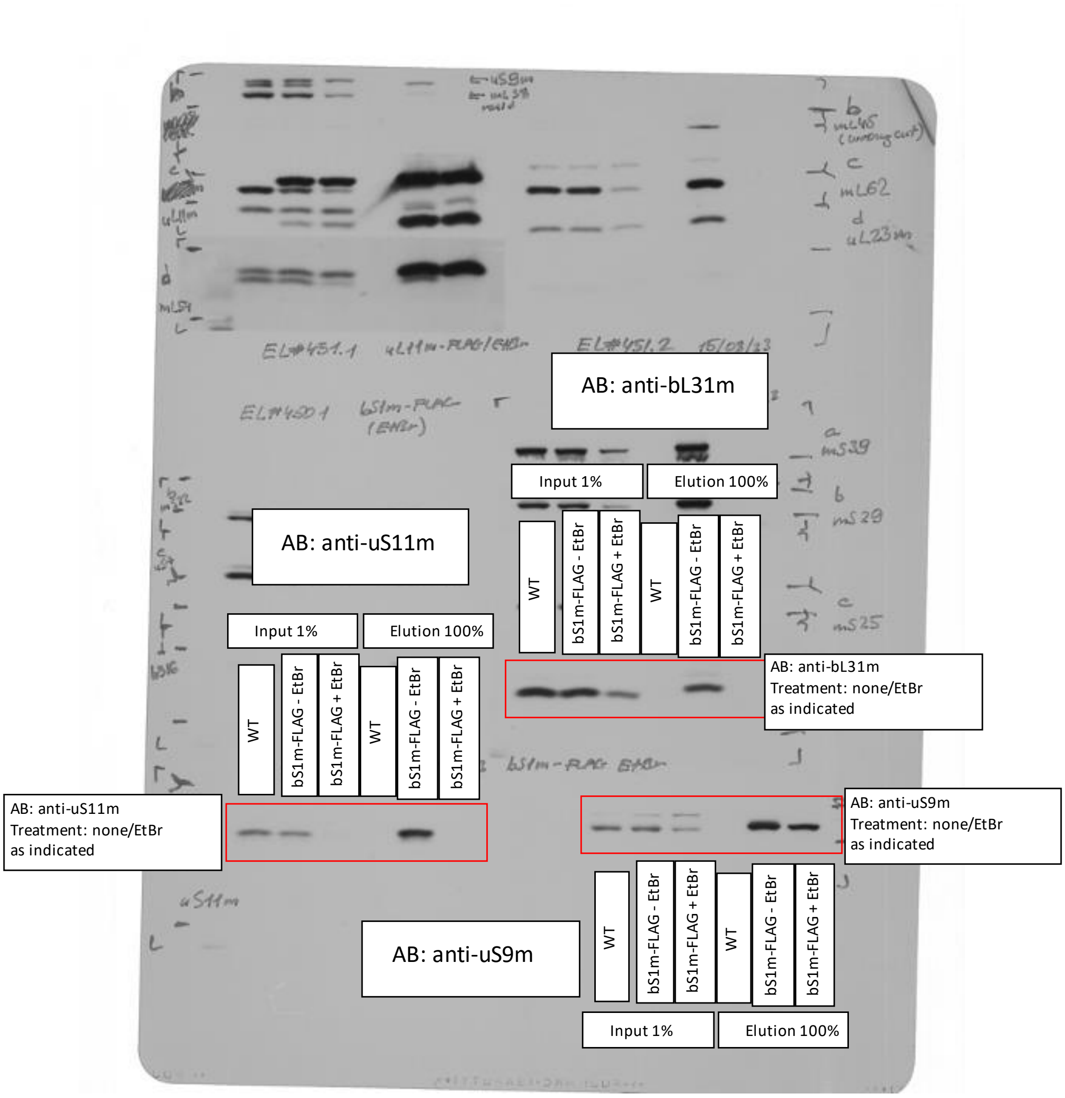

Source Data 1_related to Main Fig.4d
EL#450 bS1m-FLAG IP + EtBr treatment
Rotor: N/A
Gradient: N/A
Speed: N/A
Time: N/A

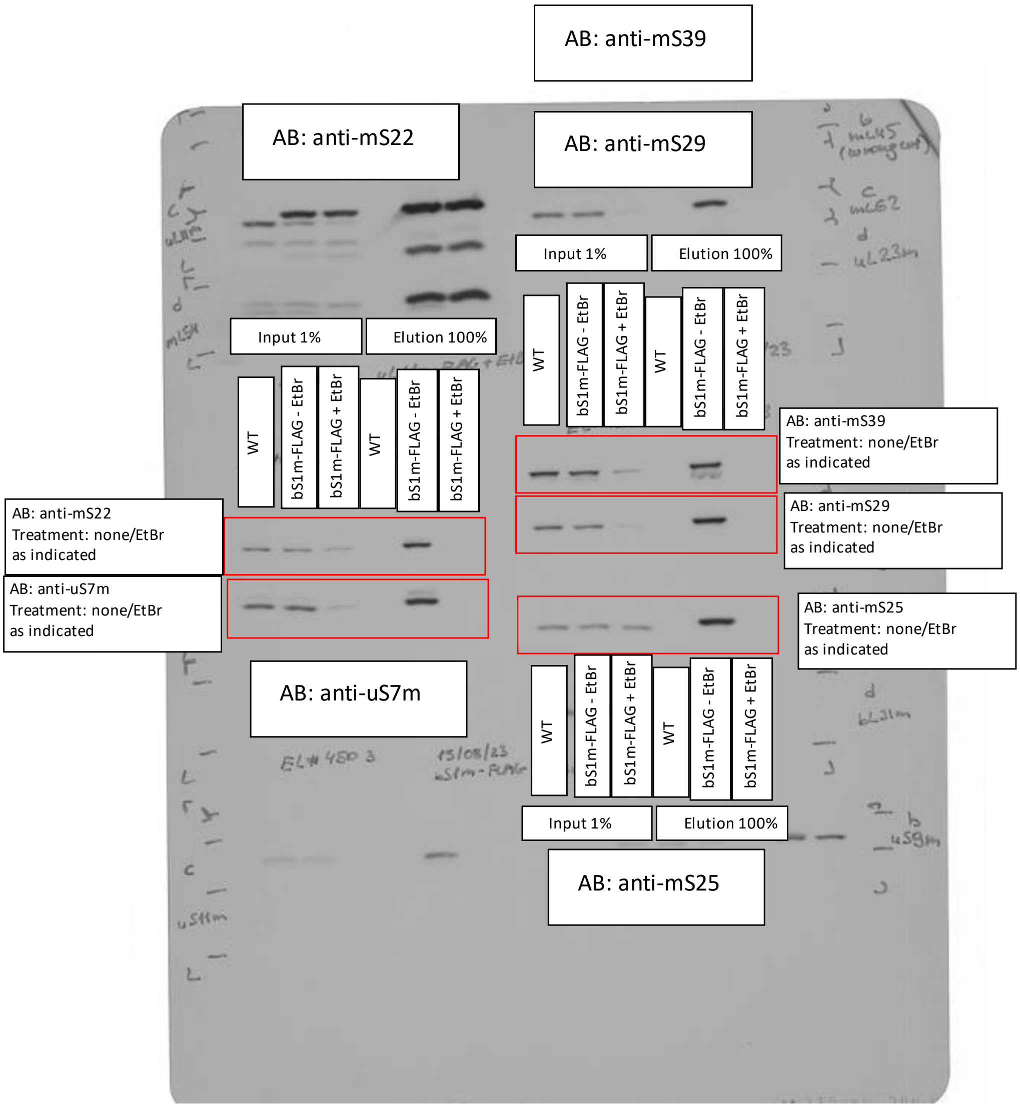

Source Data 1_related to Main Fig.4d
EL#450 bS1m-FLAG IP + EtBr treatment
Rotor: N/A
Gradient: N/A
Speed: N/A
Time: N/A

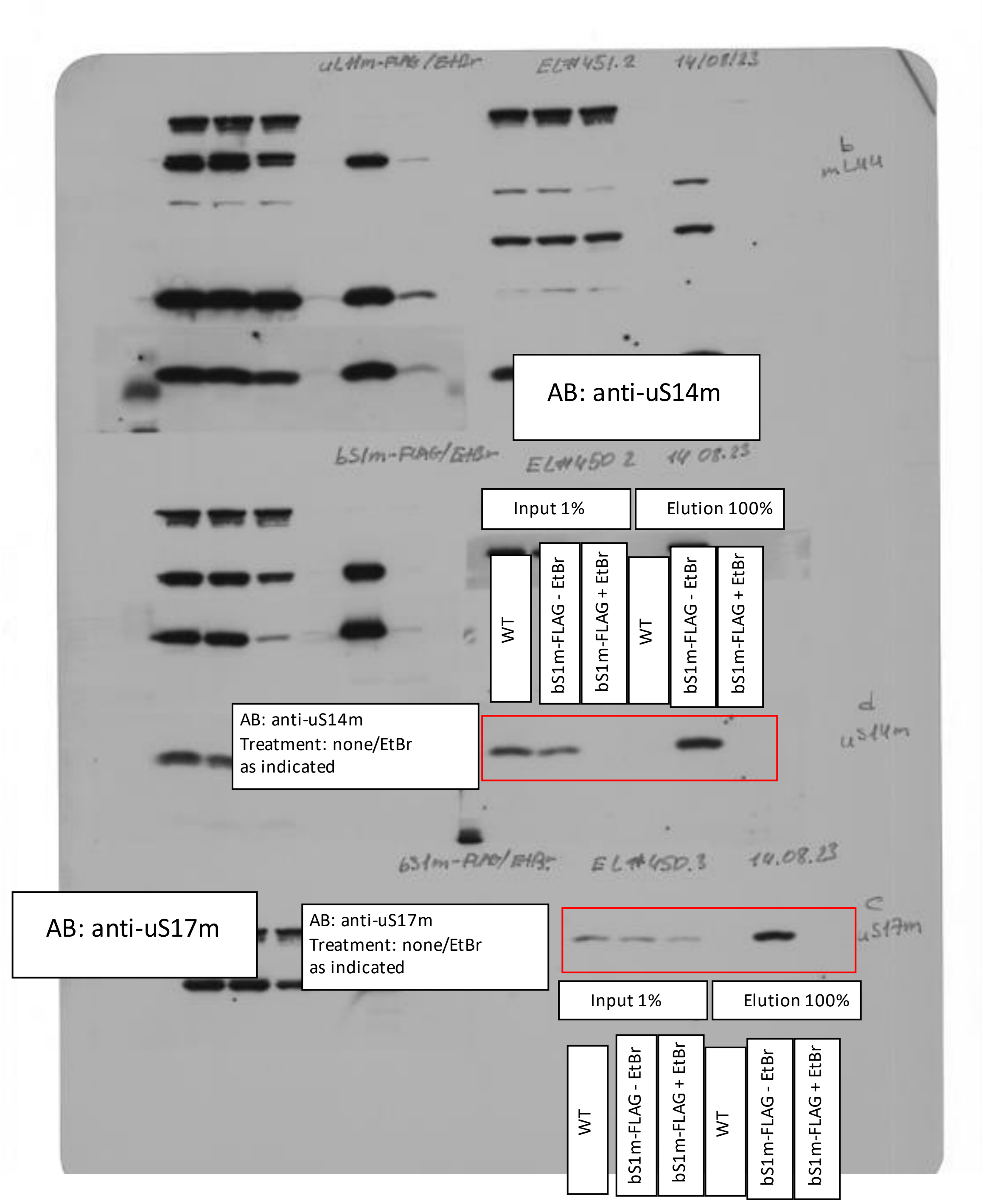

Source Data 1_related to Main Fig.4d
EL#450 bS1m-FLAG IP + EtBr treatment
Rotor: N/A
Gradient: N/A
Speed: N/A
Time: N/A

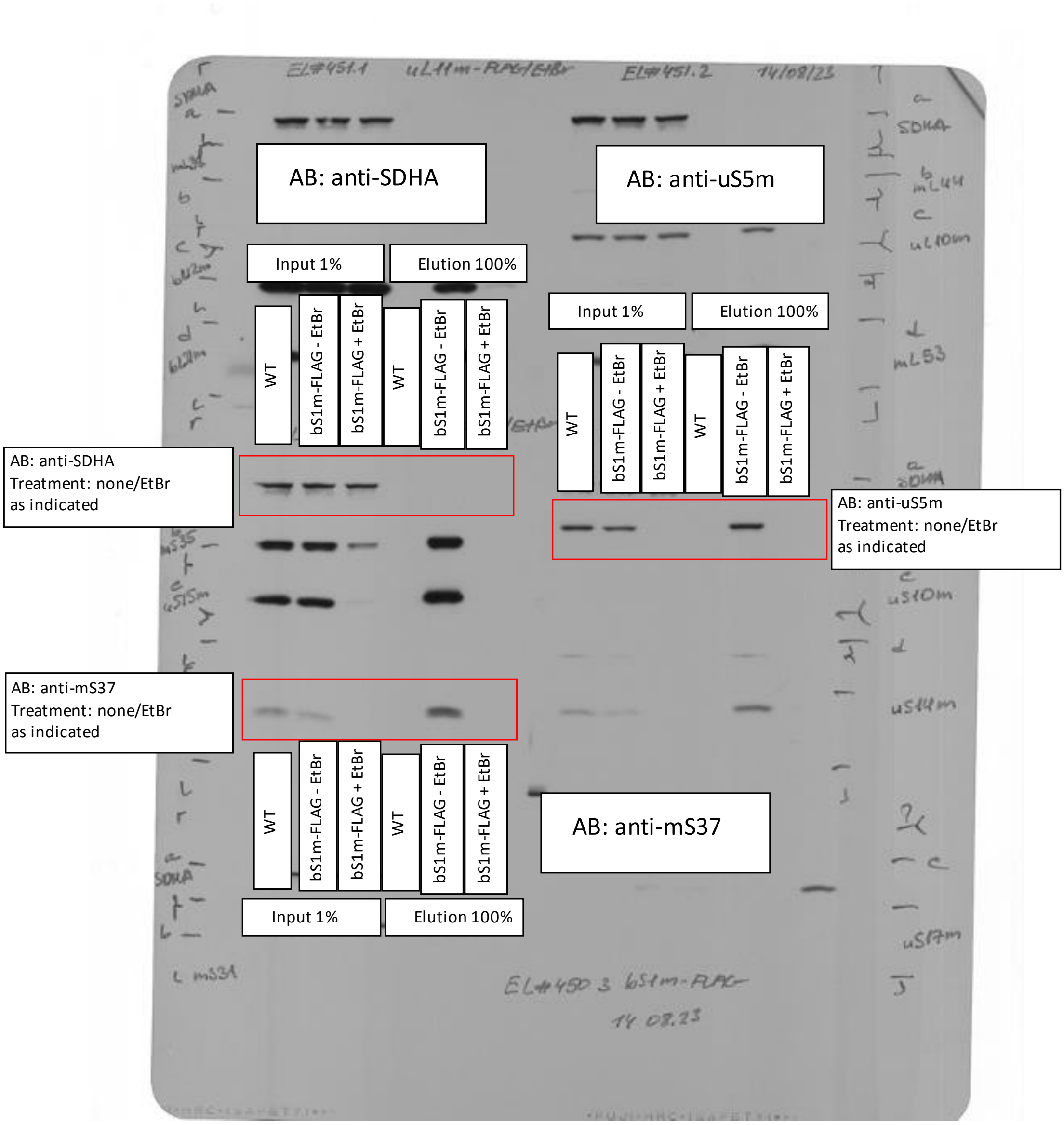

Source Data 1_related to Main Fig.4e
EL#445 mS27-FLAG IP + EtBr treatment
Rotor: N/A
Gradient: N/A
Speed: N/A
Time: N/A

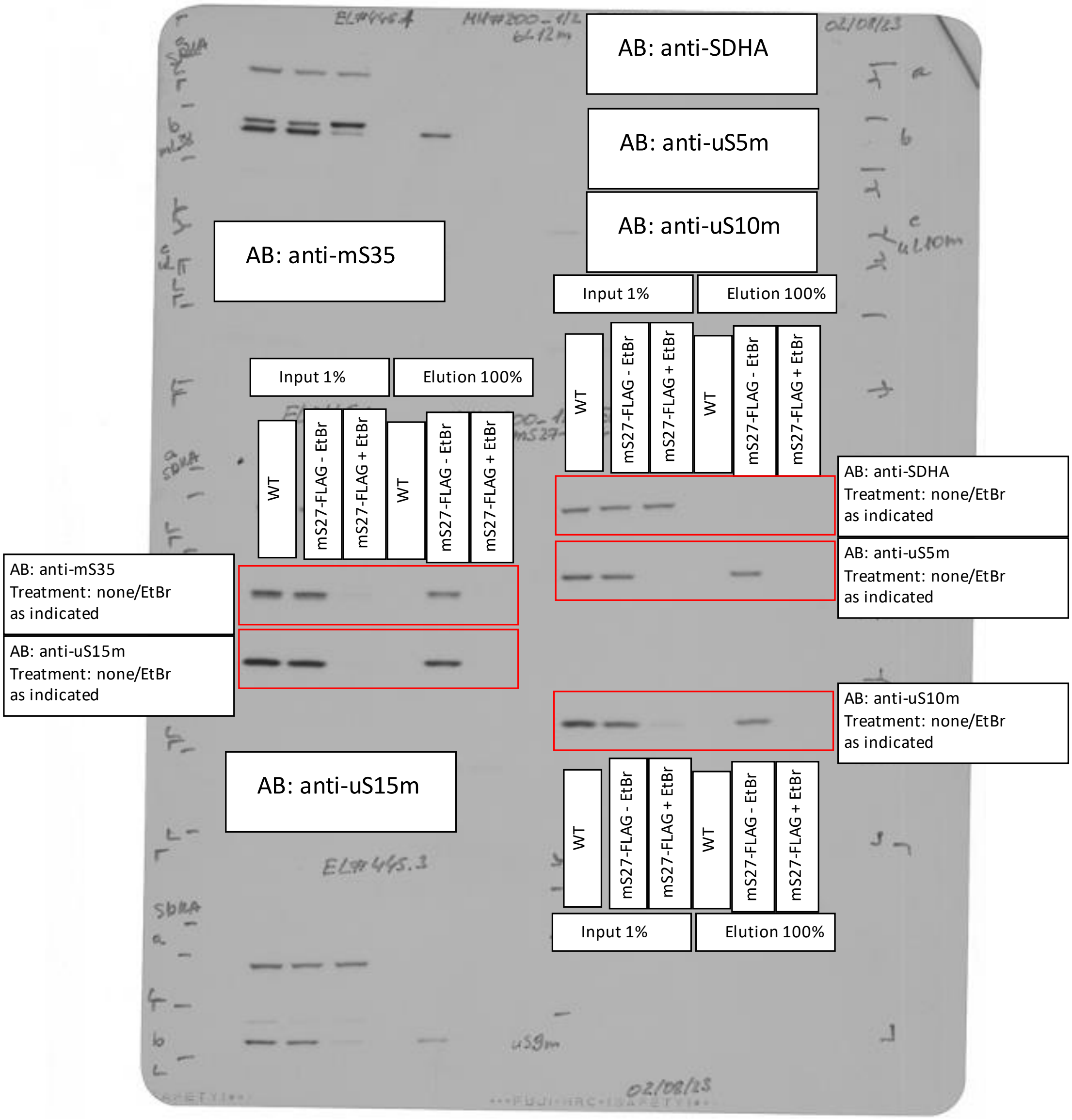

Source Data 1_related to Main Fig.4e
EL#445 mS27-FLAG IP + EtBr treatment
Rotor: N/A
Gradient: N/A
Speed: N/A
Time: N/A

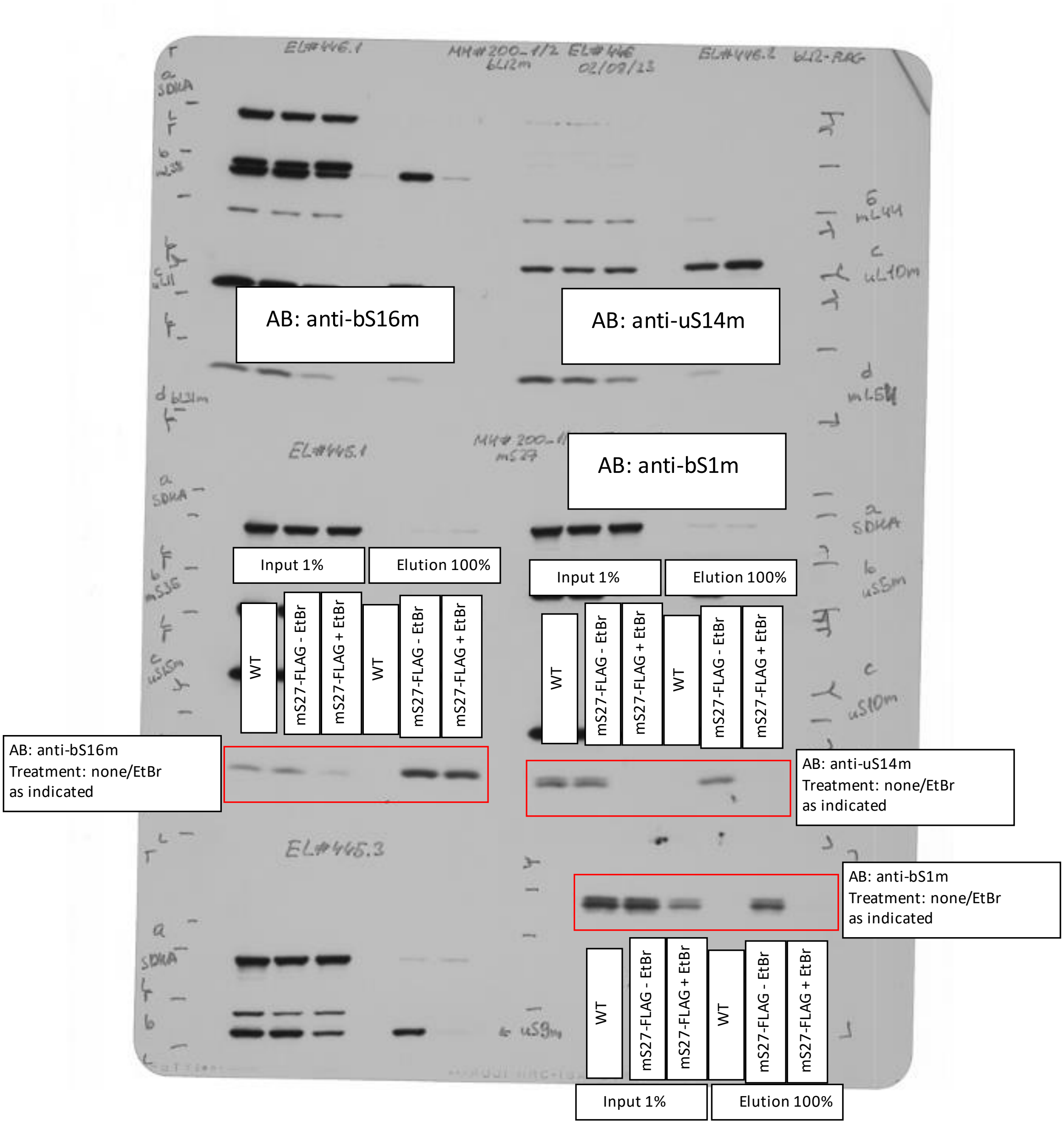

Source Data 1_related to Main Fig.4e
EL#445 mS27-FLAG IP + EtBr treatment
Rotor: N/A
Gradient: N/A
Speed: N/A
Time: N/A

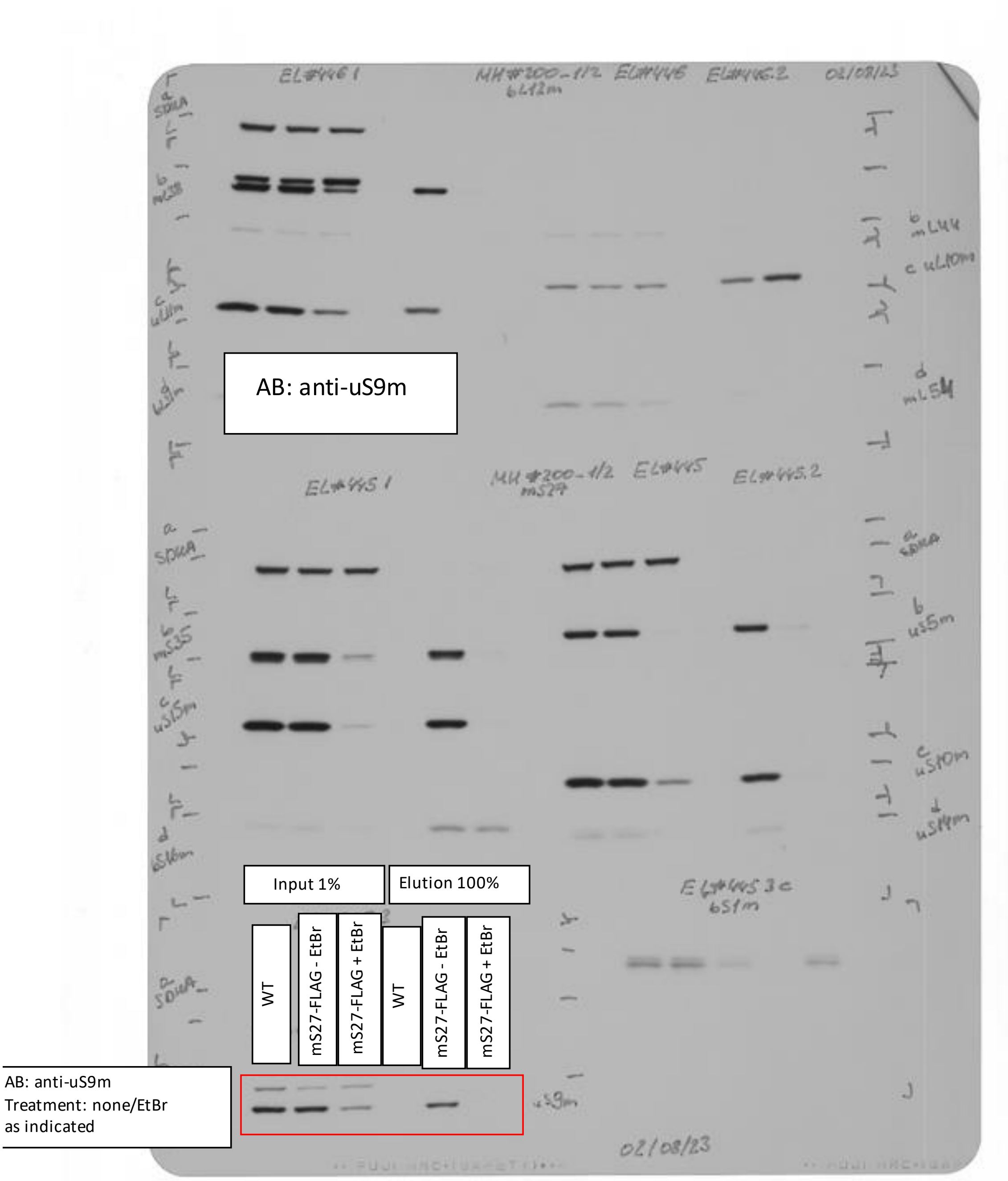

Source Data 1_related to Main Fig.4e
EL#445 mS27-FLAG IP + EtBr treatment
Rotor: N/A
Gradient: N/A
Speed: N/A
Time: N/A

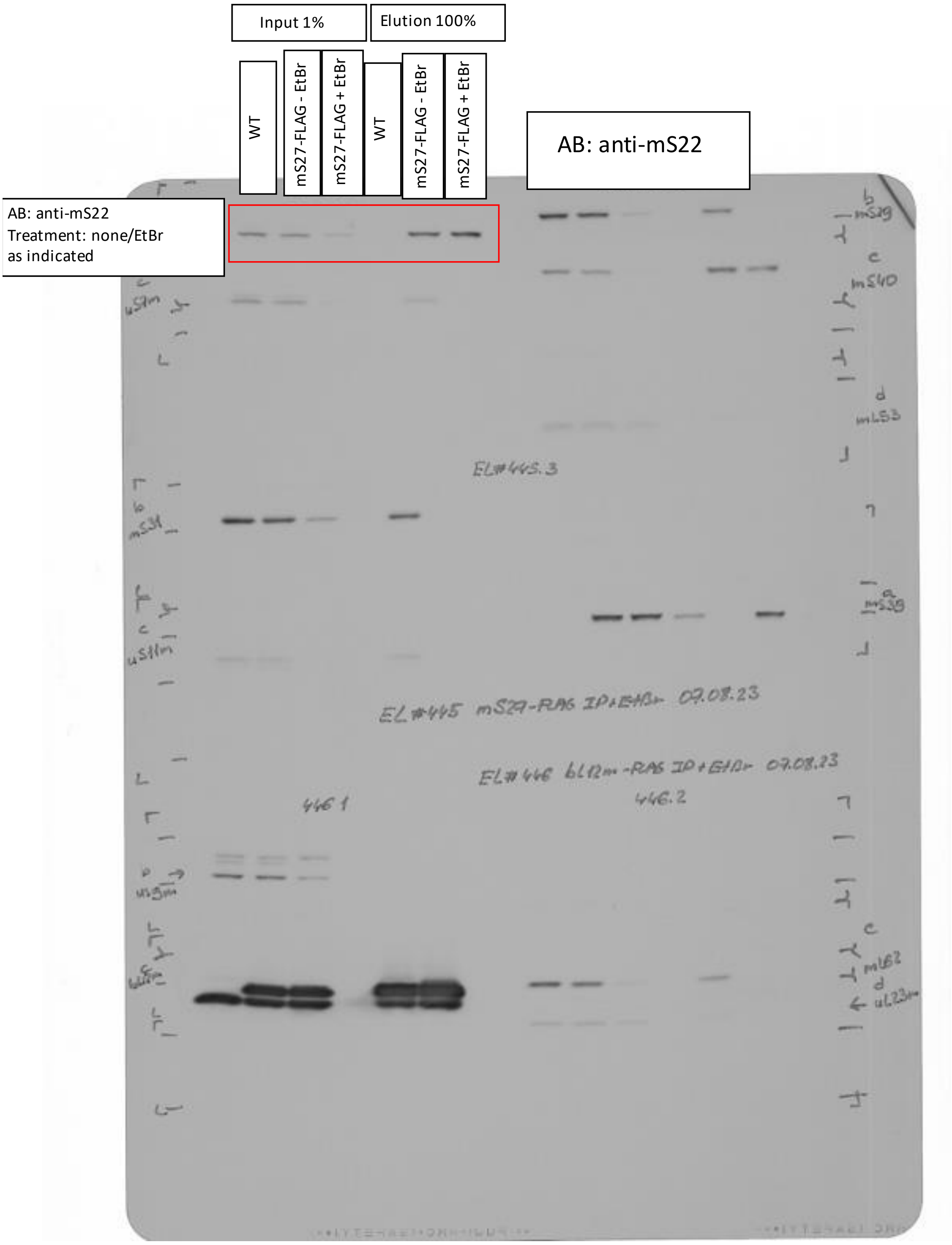

Source Data 1_related to Main Fig.4e
EL#445 mS27-FLAG IP + EtBr treatment
Rotor: N/A
Gradient: N/A
Speed: N/A
Time: N/A

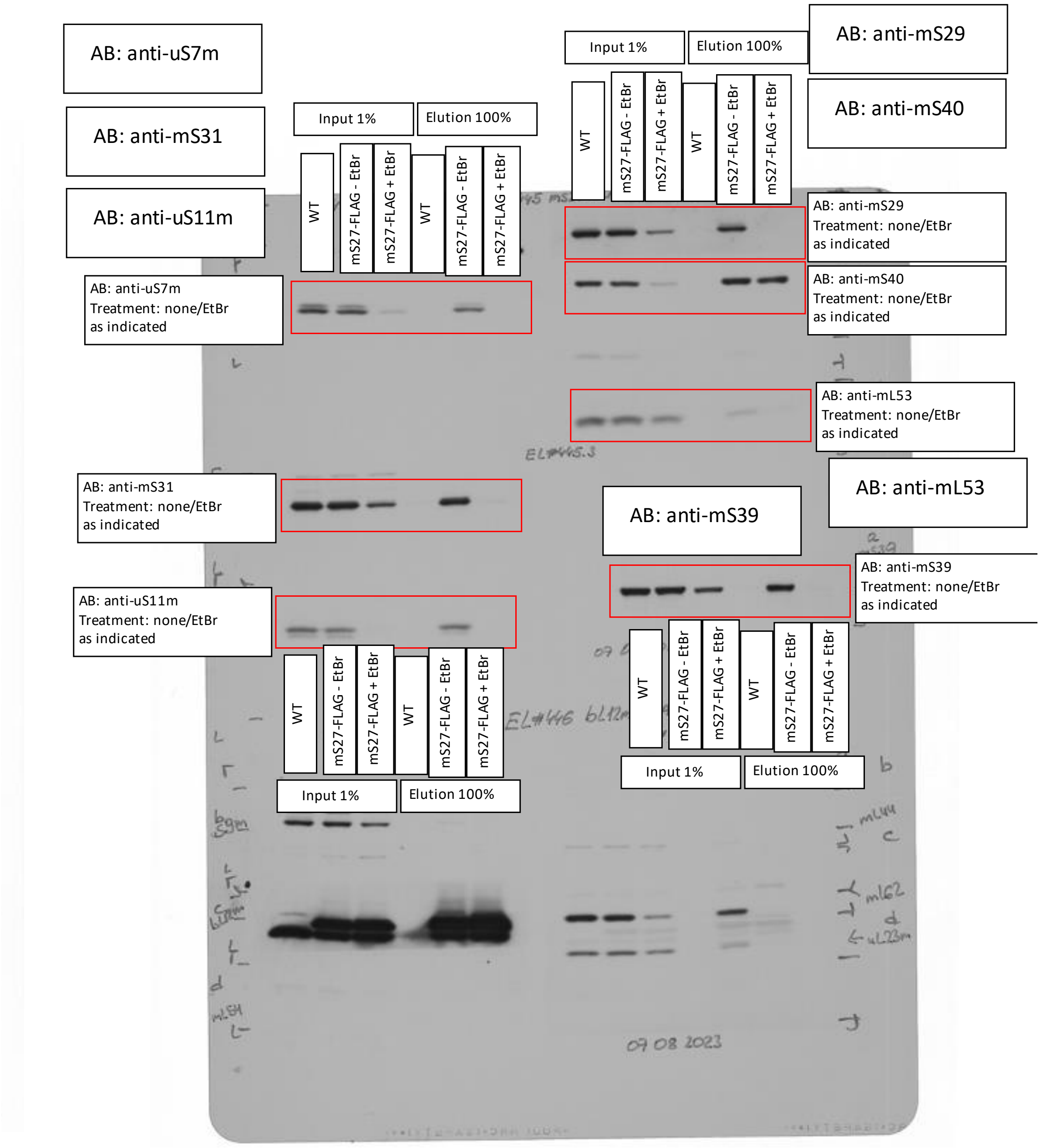

Source Data 1_related to Main Fig.4e
EL#445 mS27-FLAG IP + EtBr treatment
Rotor: N/A
Gradient: N/A
Speed: N/A
Time: N/A

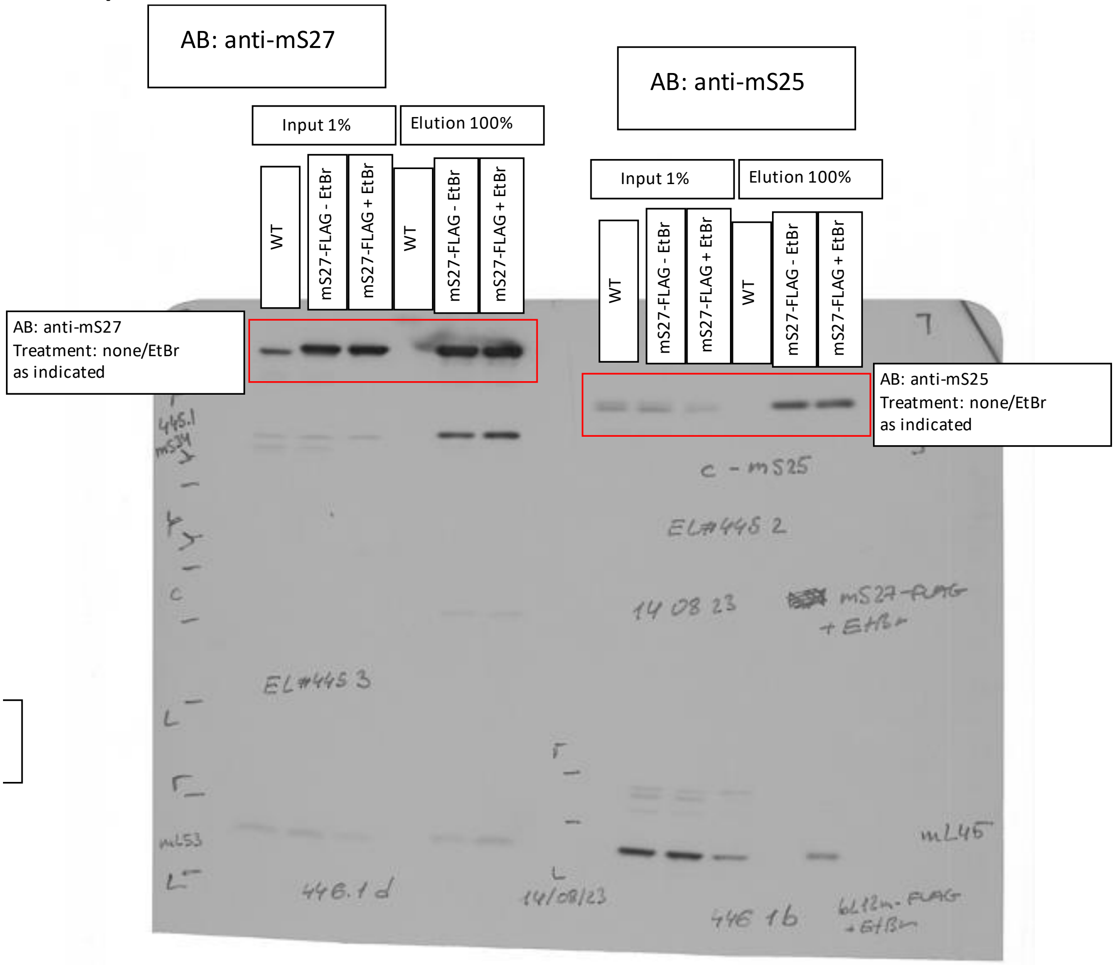

Source Data 1_related to Main Fig.4e
EL#445 mS27-FLAG IP + EtBr treatment
Rotor: N/A
Gradient: N/A
Speed: N/A
Time: N/A

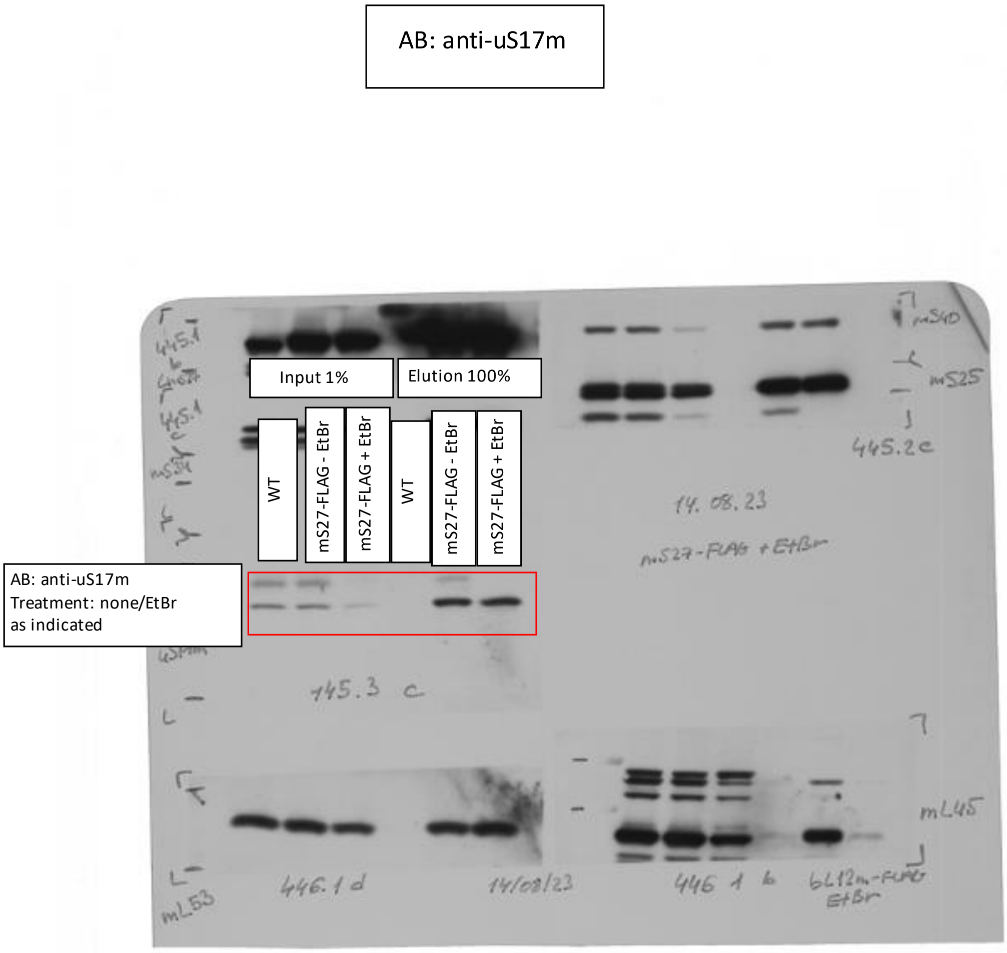

Source Data 1_related to Main Fig.4e
EL#445 mS27-FLAG IP + EtBr treatment
Rotor: N/A
Gradient: N/A
Speed: N/A
Time: N/A

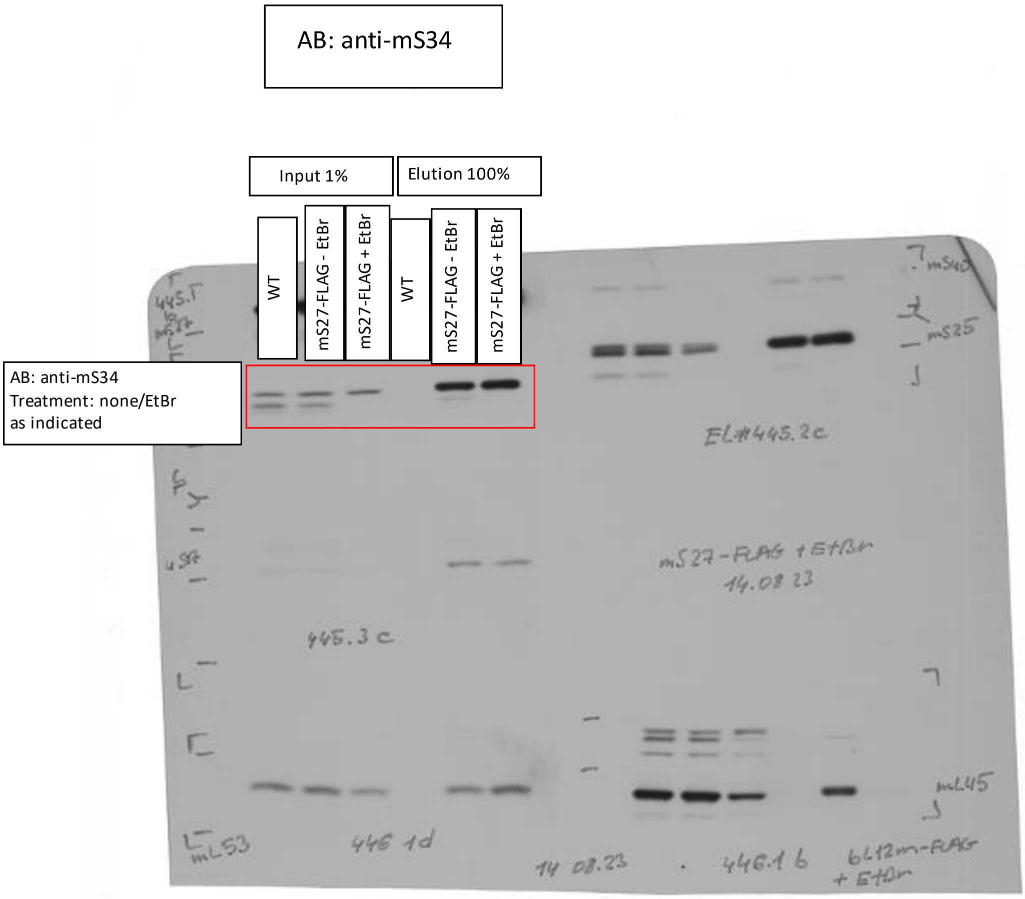

Source Data 1_related to Main Fig.4f
EL#378 mS22-FLAG IP + Gradient + EtBr treatment
Rotor: SW41 Ti
Gradient: Sucrose 5-30%
Speed: 158.000xg
Time: 15h

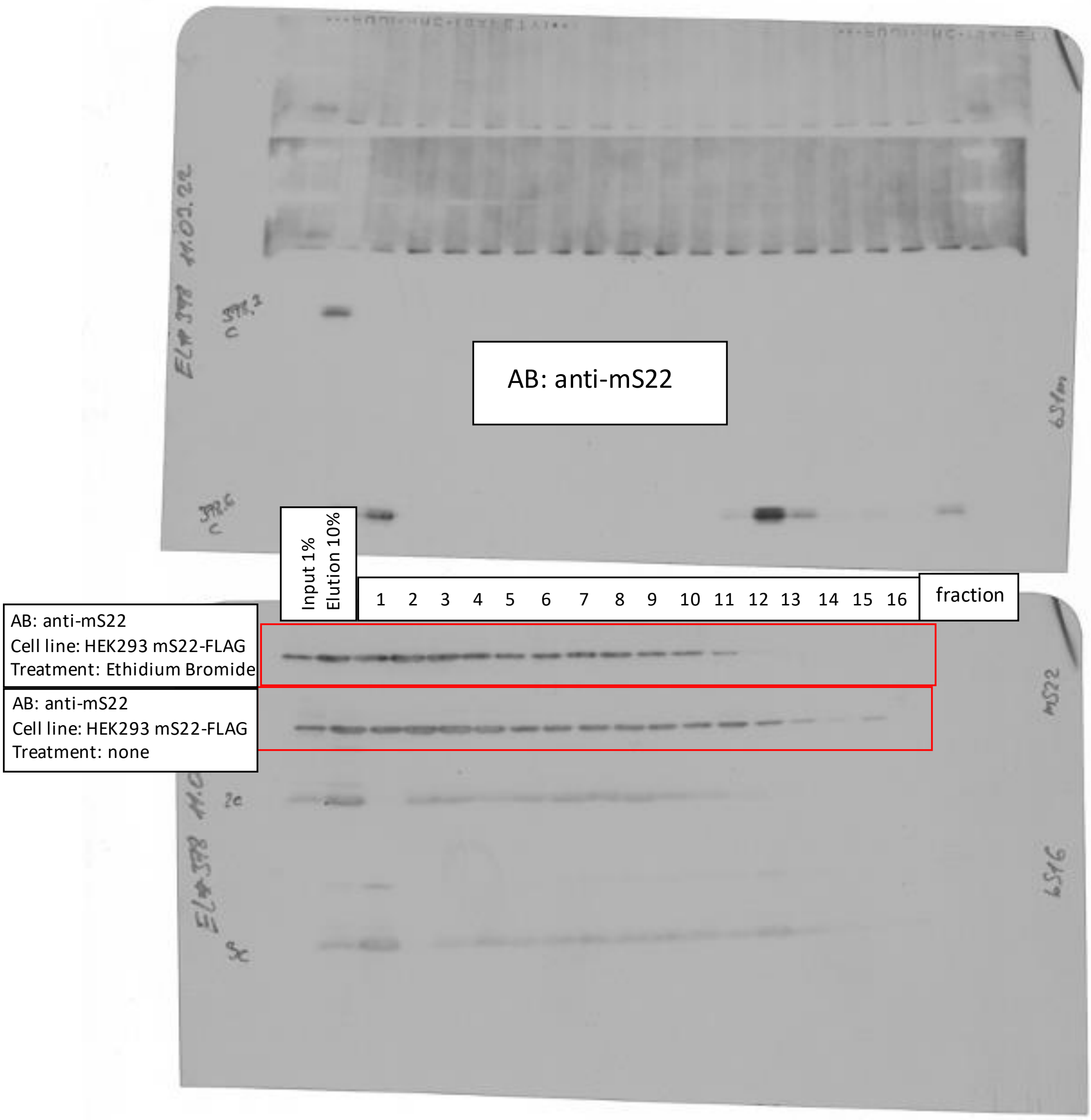

Source Data 1_related to Main Fig.4f
EL#378 mS22-FLAG IP + Gradient + EtBr treatment
Rotor: SW41 Ti
Gradient: Sucrose 5-30%
Speed: 158.000xg
Time: 15h

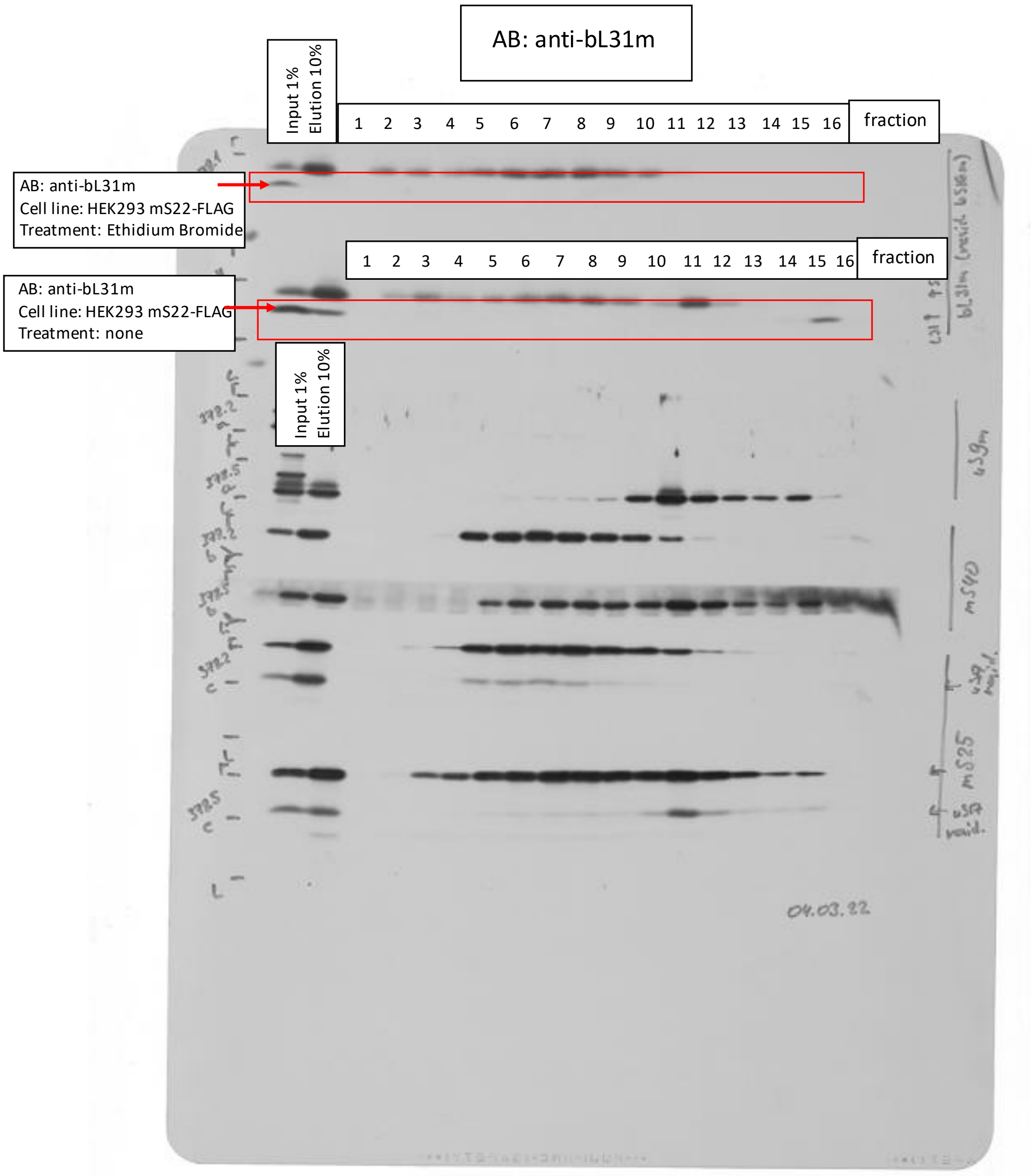

Source Data 1_related to Main Fig.4f
EL#378 mS22-FLAG IP + Gradient + EtBr treatment
Rotor: SW41 Ti
Gradient: Sucrose 5-30%
Speed: 158.000xg
Time: 15h

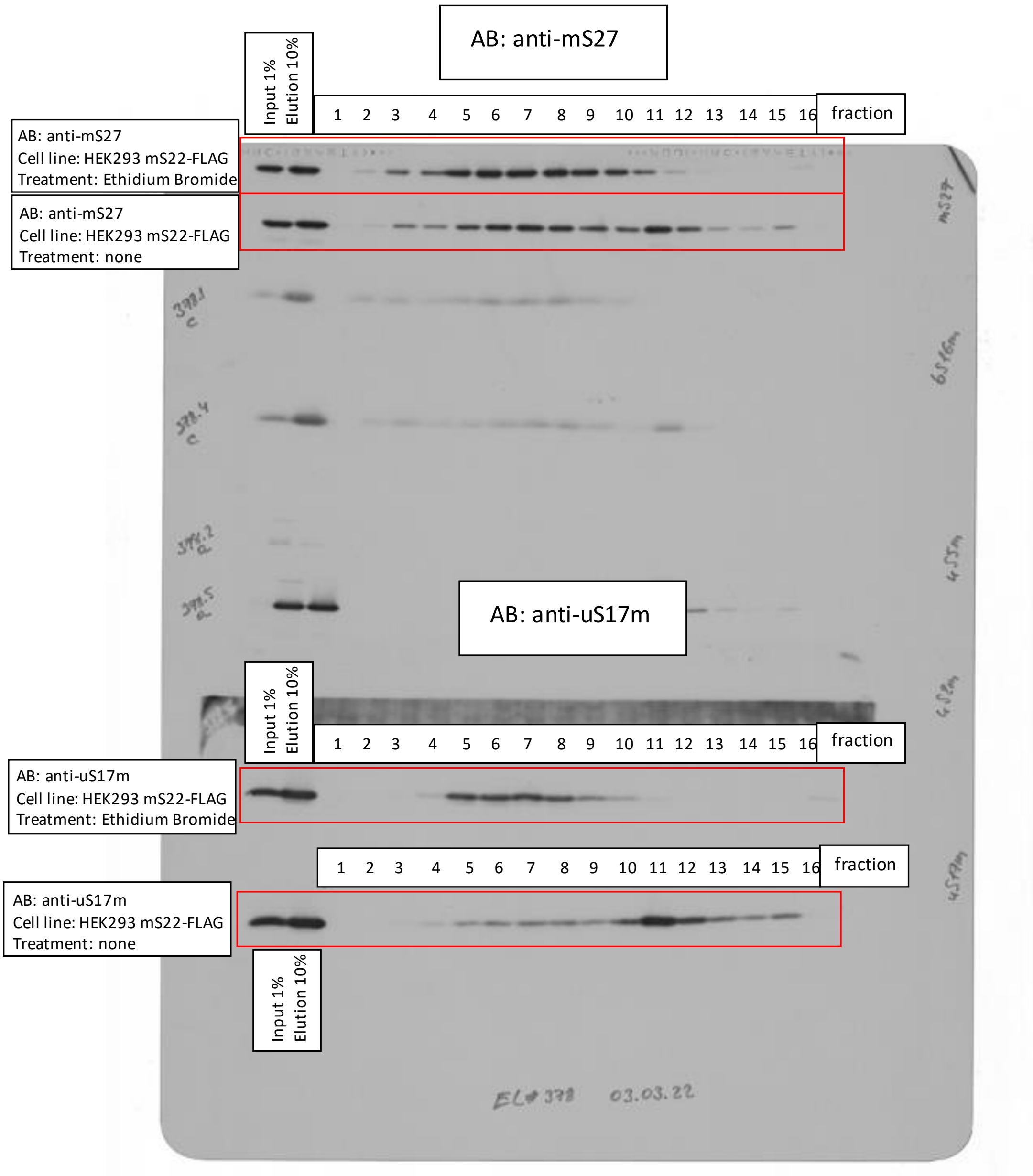

Source Data 1_related to Main Fig.4f
EL#378 mS22-FLAG IP + Gradient + EtBr treatment
Rotor: SW41 Ti
Gradient: Sucrose 5-30%
Speed: 158.000xg
Time: 15h

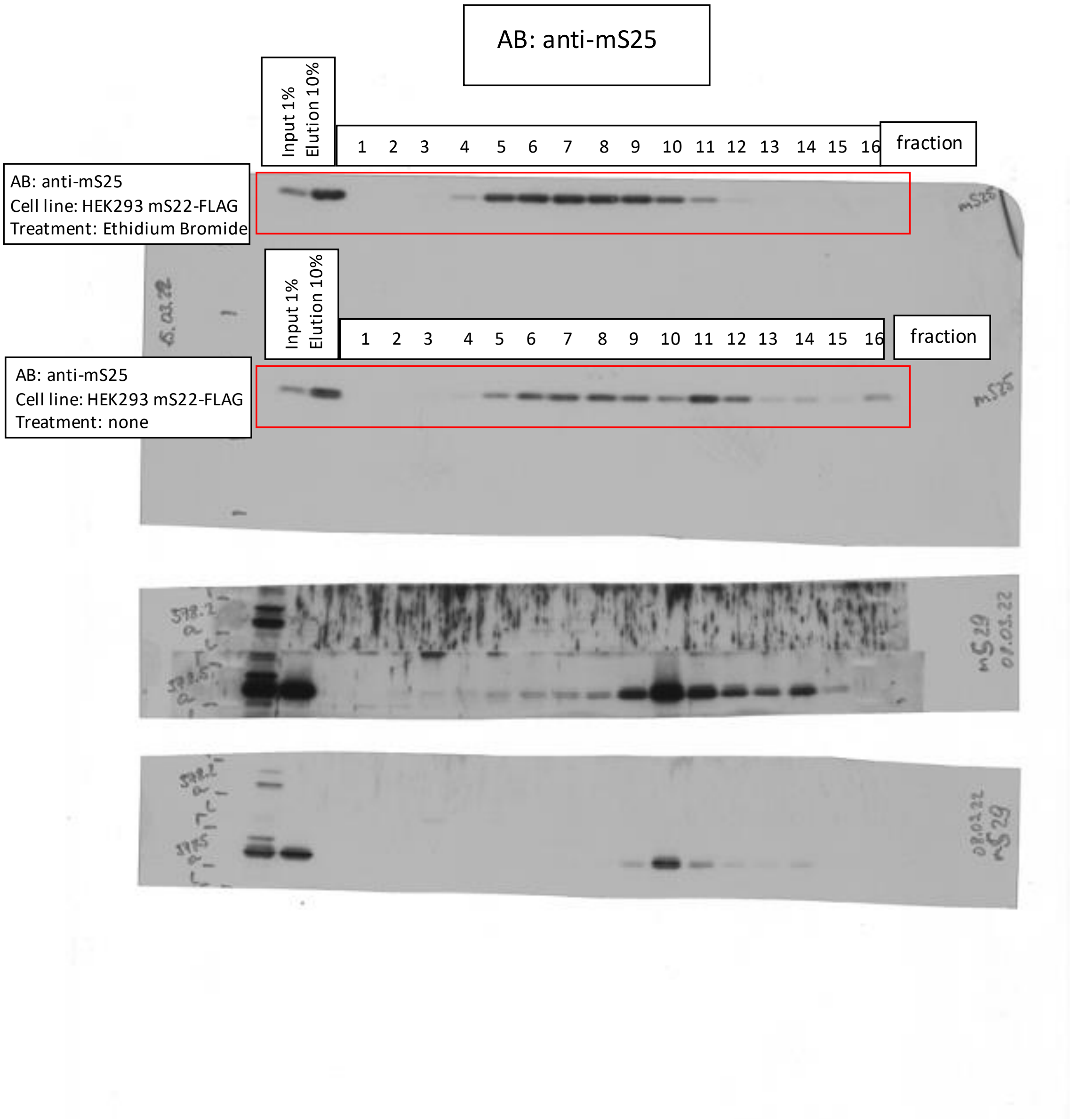

Source Data 1_related to Main Fig.4f
EL#378 mS22-FLAG IP + Gradient + EtBr treatment
Rotor: SW41 Ti
Gradient: Sucrose 5-30%
Speed: 158.000xg
Time: 15h

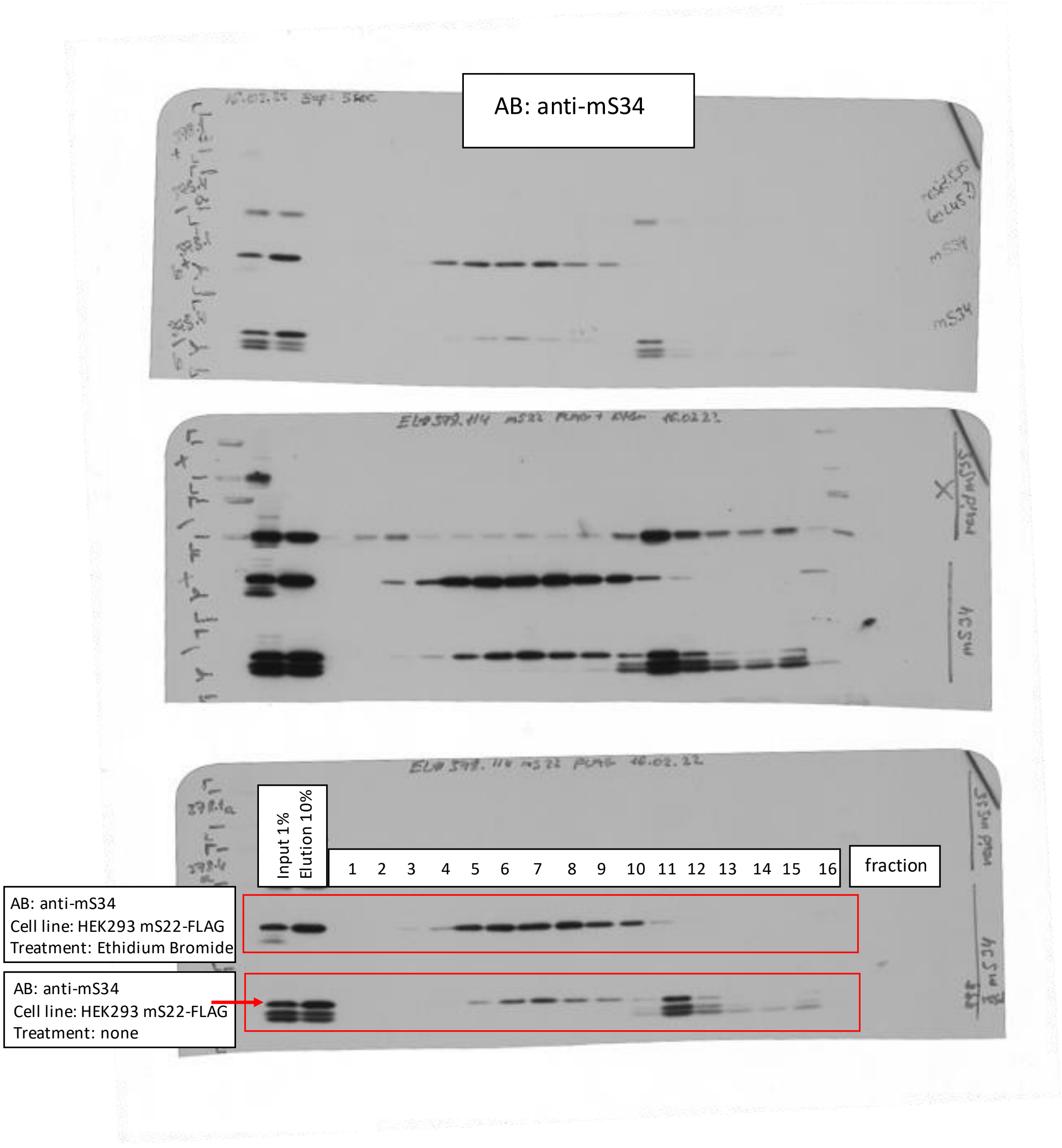

Source Data 1_related to Main Fig.4f
EL#378 mS22-FLAG IP + Gradient + EtBr treatment
Rotor: SW41 Ti
Gradient: Sucrose 5-30%
Speed: 158.000xg
Time: 15h

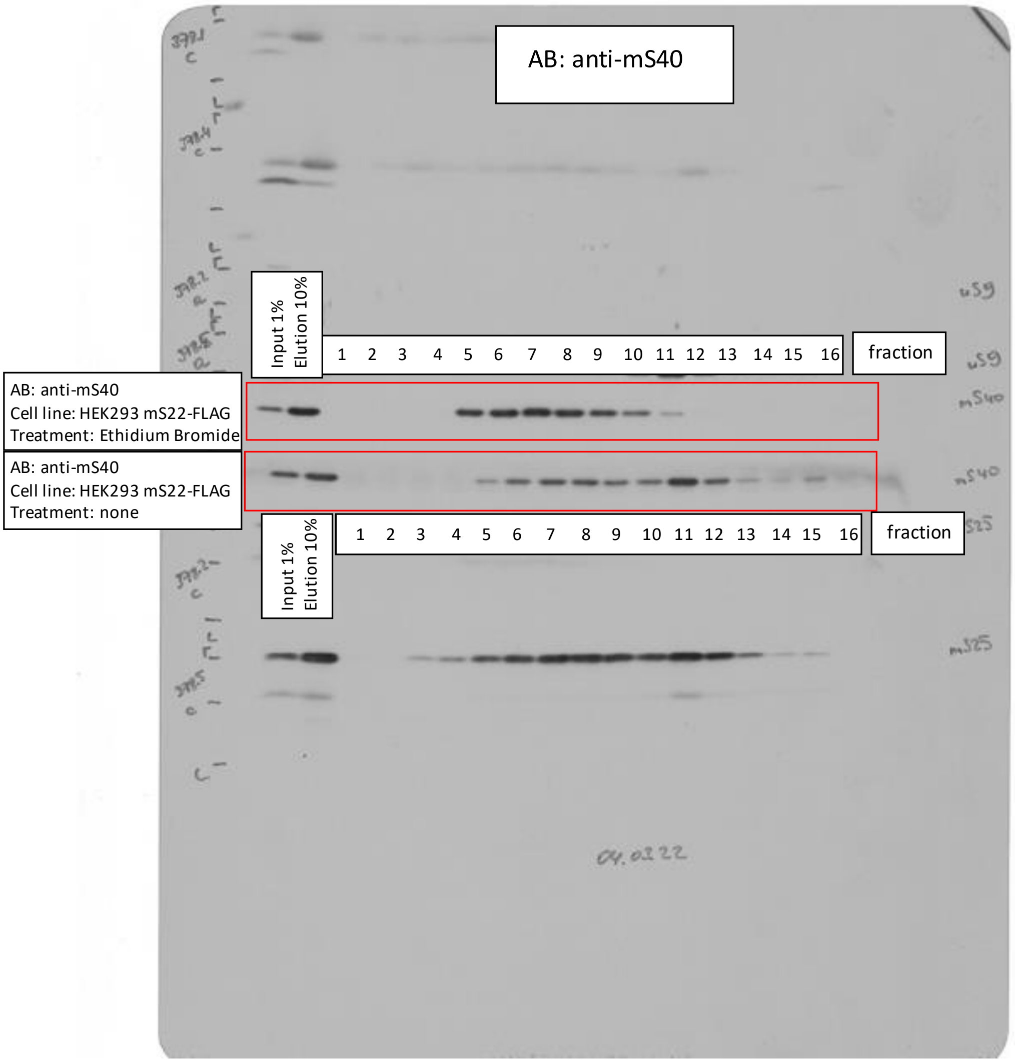

Source Data 1_related to Main Fig.4f
EL#378 mS22-FLAG IP + Gradient + EtBr treatment
Rotor: SW41 Ti
Gradient: Sucrose 5-30%
Speed: 158.000xg
Time: 15h

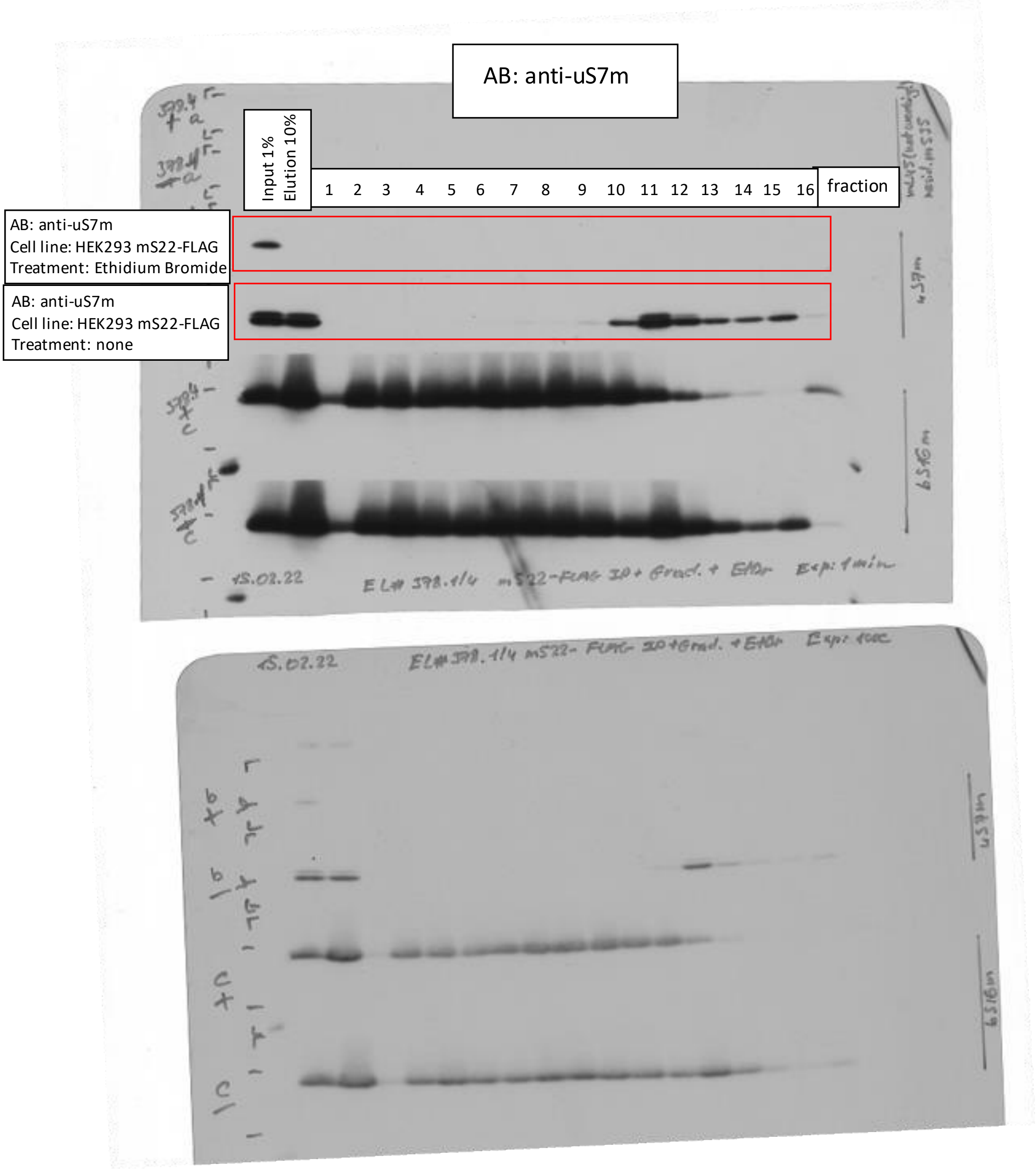

Supplement: Supplementary file 16 — Unprocessed blots. [file 41594_2024_1356_MOESM16_ESM.pdf]
