# Supplementary material for: A roadmap for ribosome assembly in human mitochondria
Source: Nat Struct Mol Biol. 2024 Jul 11;31(12):1898–908. doi: 10.1038/s41594-024-01356-w (PMC11638073; doi:10.1038/s41594-024-01356-w)

Source Data 2_related to Extended Data Fig.3a
EL#349 mS27-FLAG IP + Gradient
Rotor: SW41 Ti
Gradient: Sucrose 5-30%
Speed: 158.000xg
Time: 15h

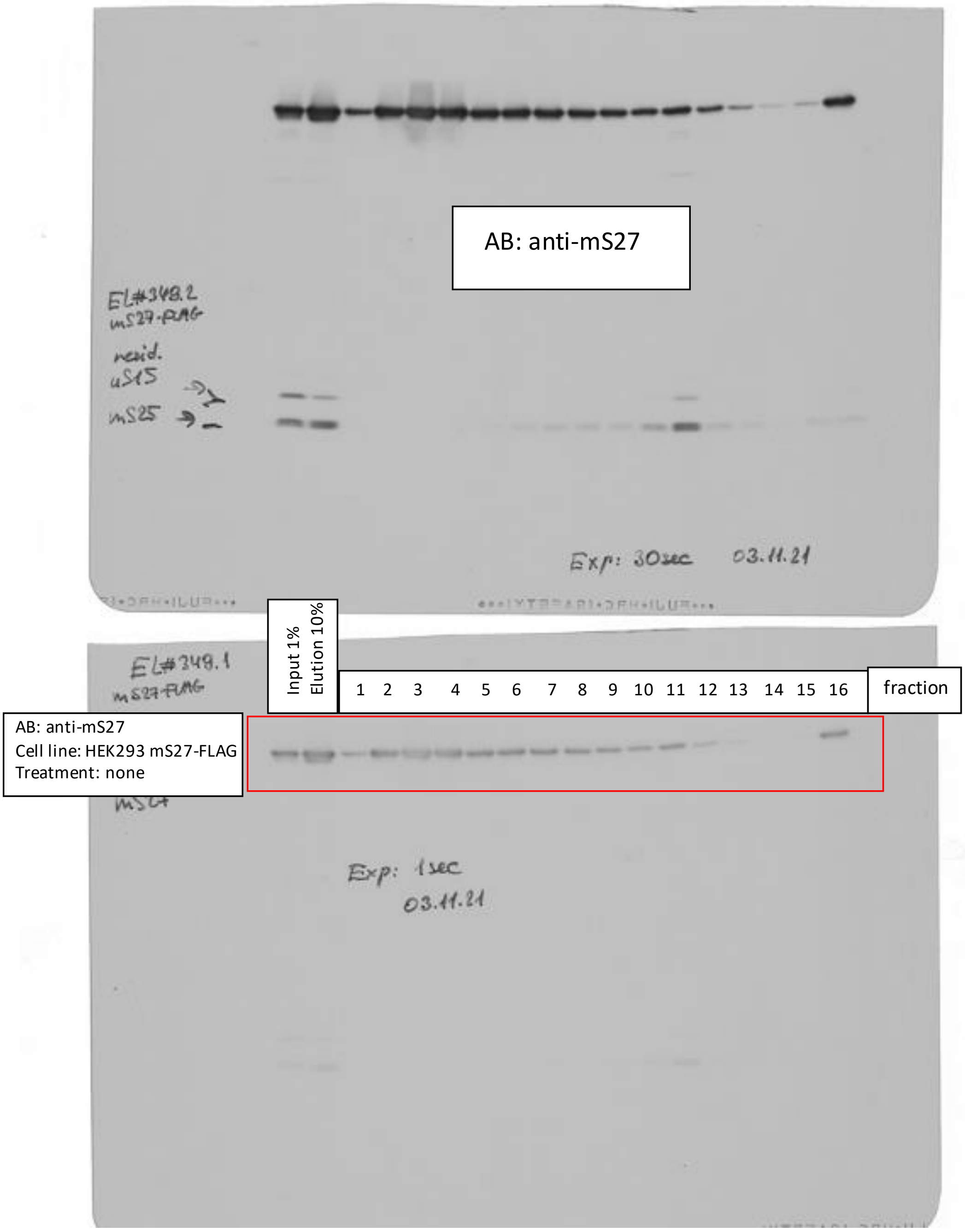

Source Data 2_related to Extended Data Fig.3a
EL#349 mS27-FLAG IP + Gradient
Rotor: SW41 Ti
Gradient: Sucrose 5-30%
Speed: 158.000xg
Time: 15h

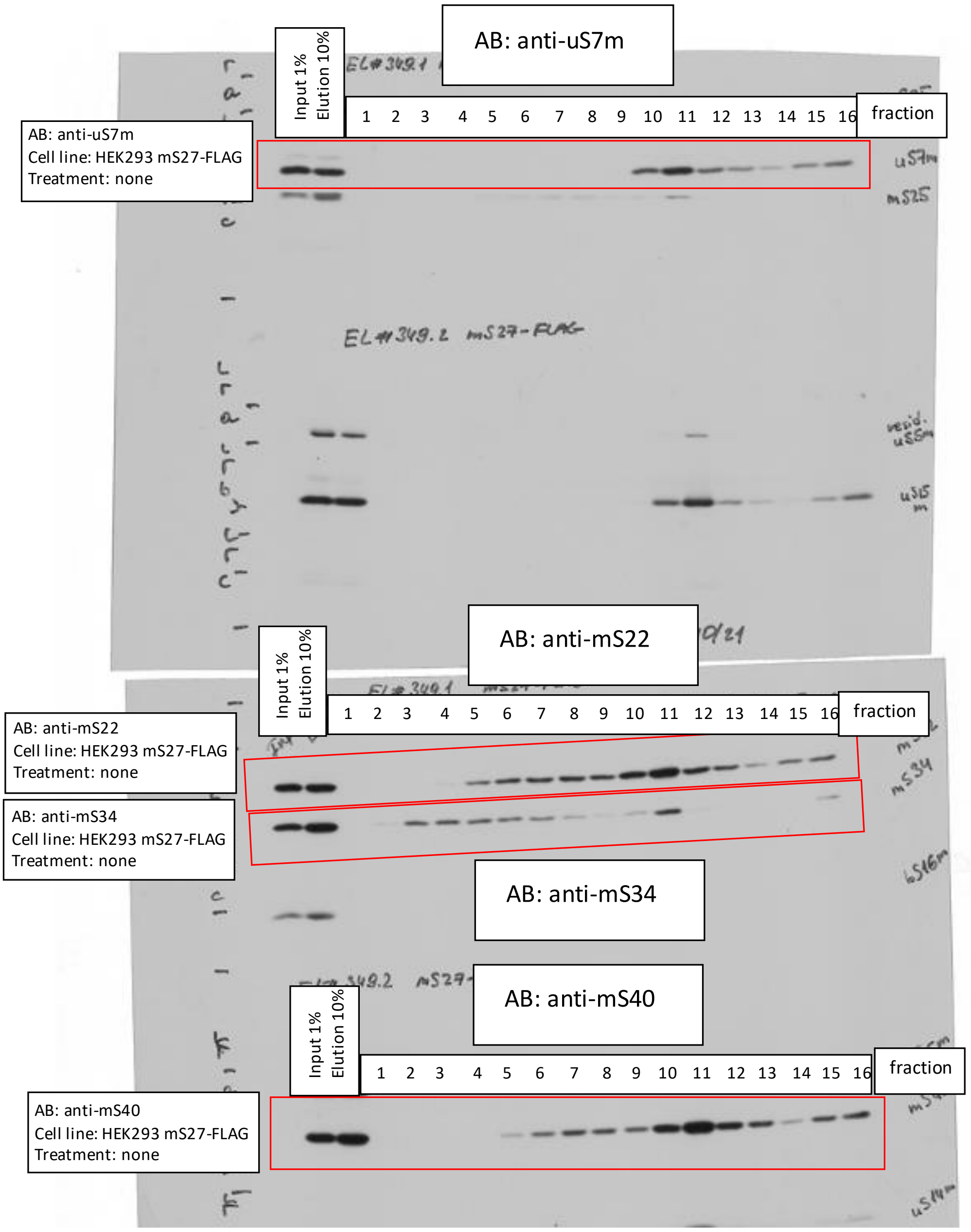

Source Data 2_related to Extended Data Fig.3a
EL#349 mS27-FLAG IP + Gradient
Rotor: SW41 Ti
Gradient: Sucrose 5-30%
Speed: 158.000xg
Time: 15h

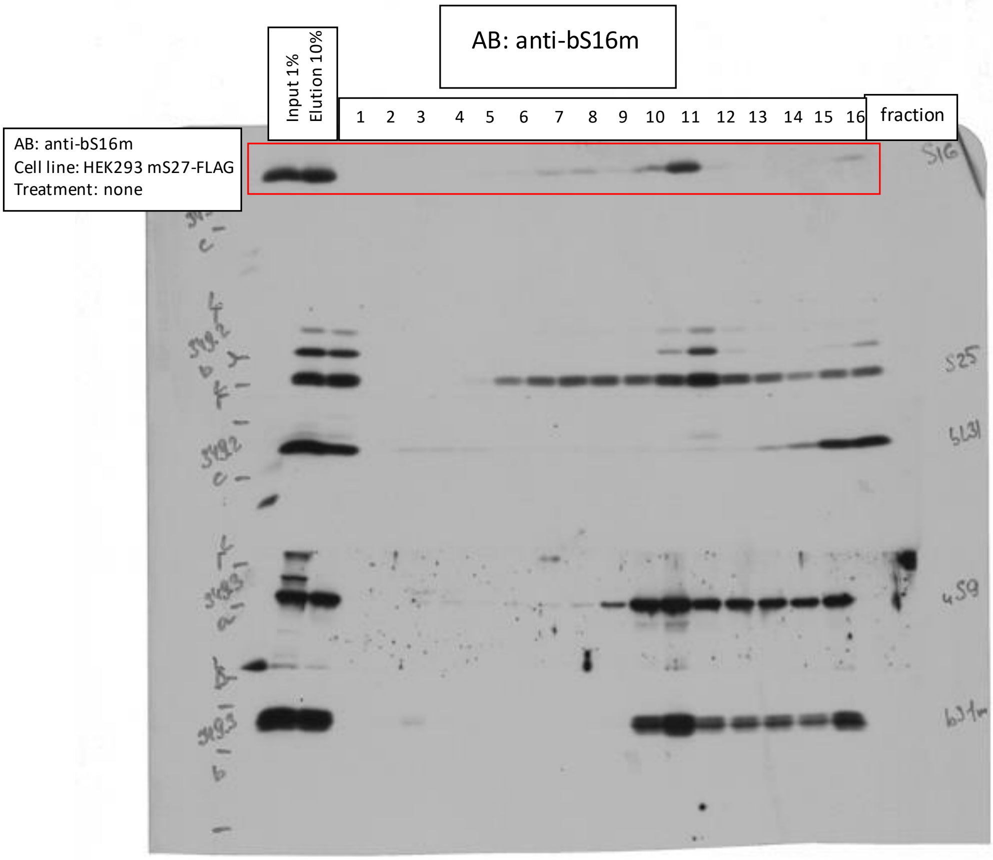

Source Data 2_related to Extended Data Fig.3a
EL#349 mS27-FLAG IP + Gradient
Rotor: SW41 Ti
Gradient: Sucrose 5-30%
Speed: 158.000xg
Time: 15h

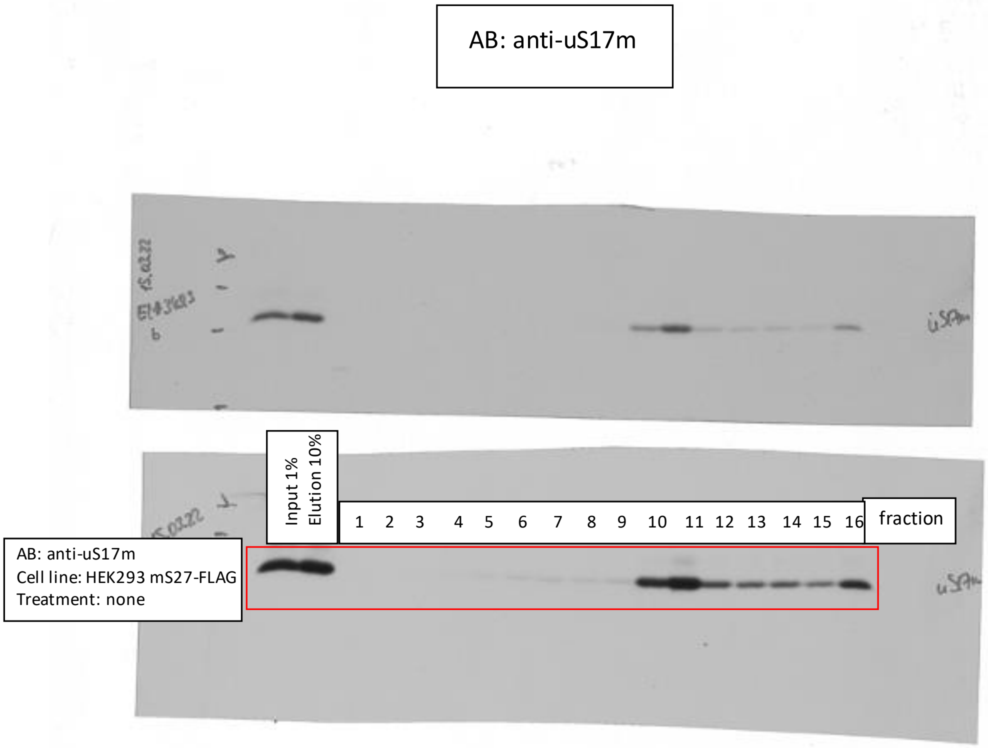

Source Data 2_related to Extended Data Fig.3a
EL#349 mS27-FLAG IP + Gradient
Rotor: SW41 Ti
Gradient: Sucrose 5-30%
Speed: 158.000xg
Time: 15h

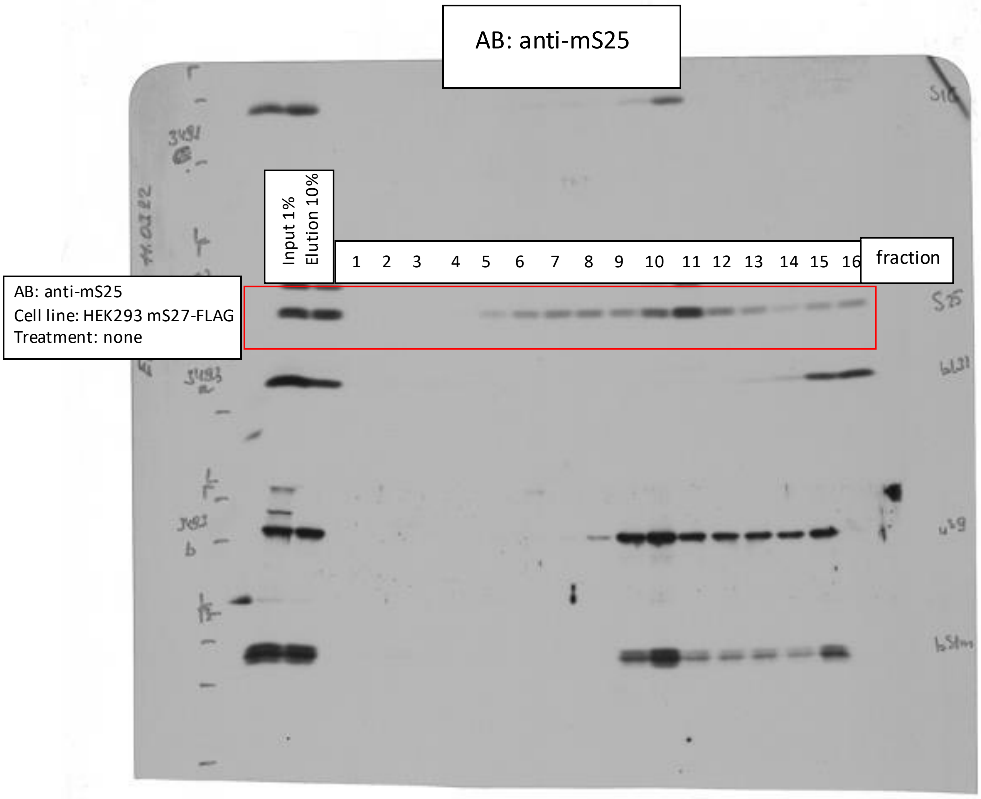

Source Data 2_related to Extended Data Fig.3a
EL#349 mS27-FLAG IP + Gradient
Rotor: SW41 Ti
Gradient: Sucrose 5-30%
Speed: 158.000xg
Time: 15h

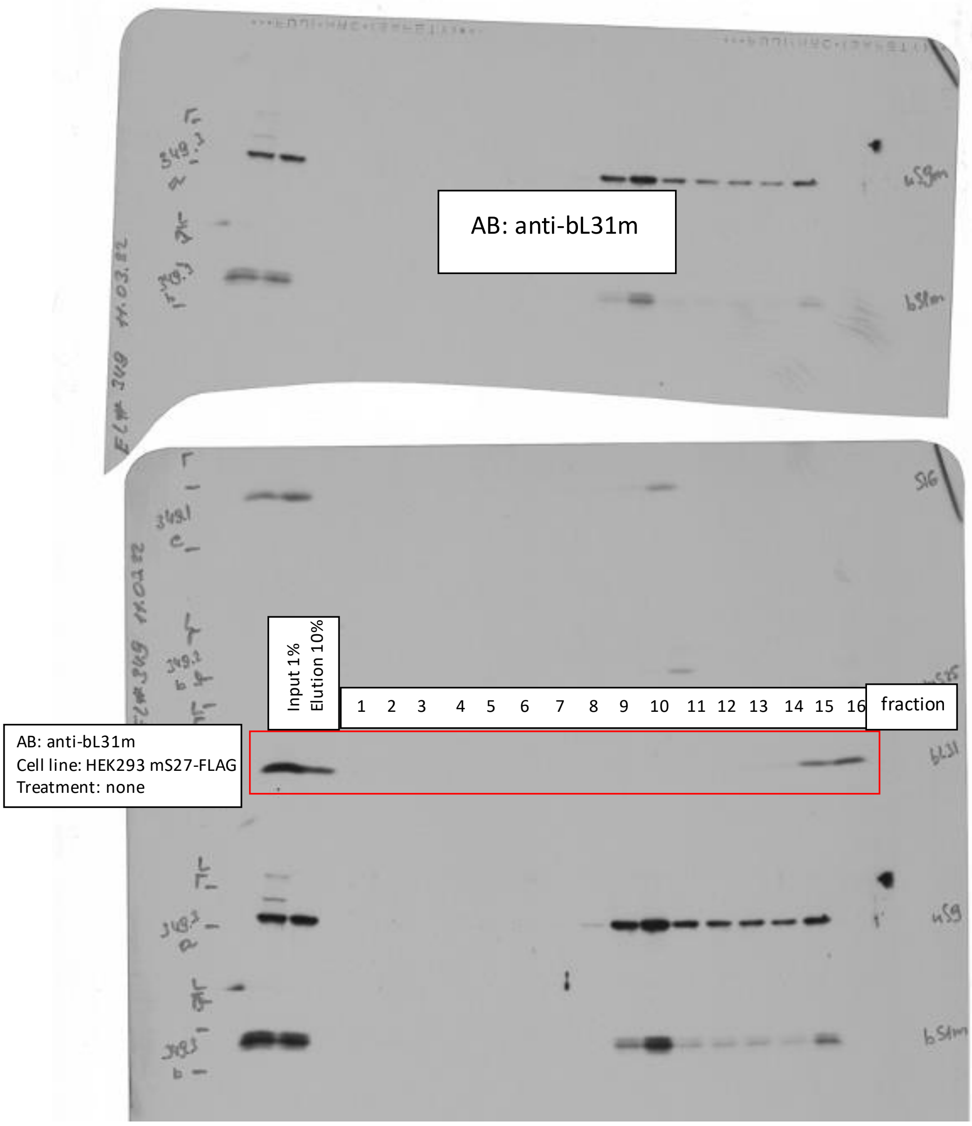

Source Data 2_related to Extended Data Fig.3b
EL#373 mS25-FLAG IP + Gradient
Rotor: SW41 Ti
Gradient: Sucrose 5-30%
Speed: 158.000xg
Time: 15h

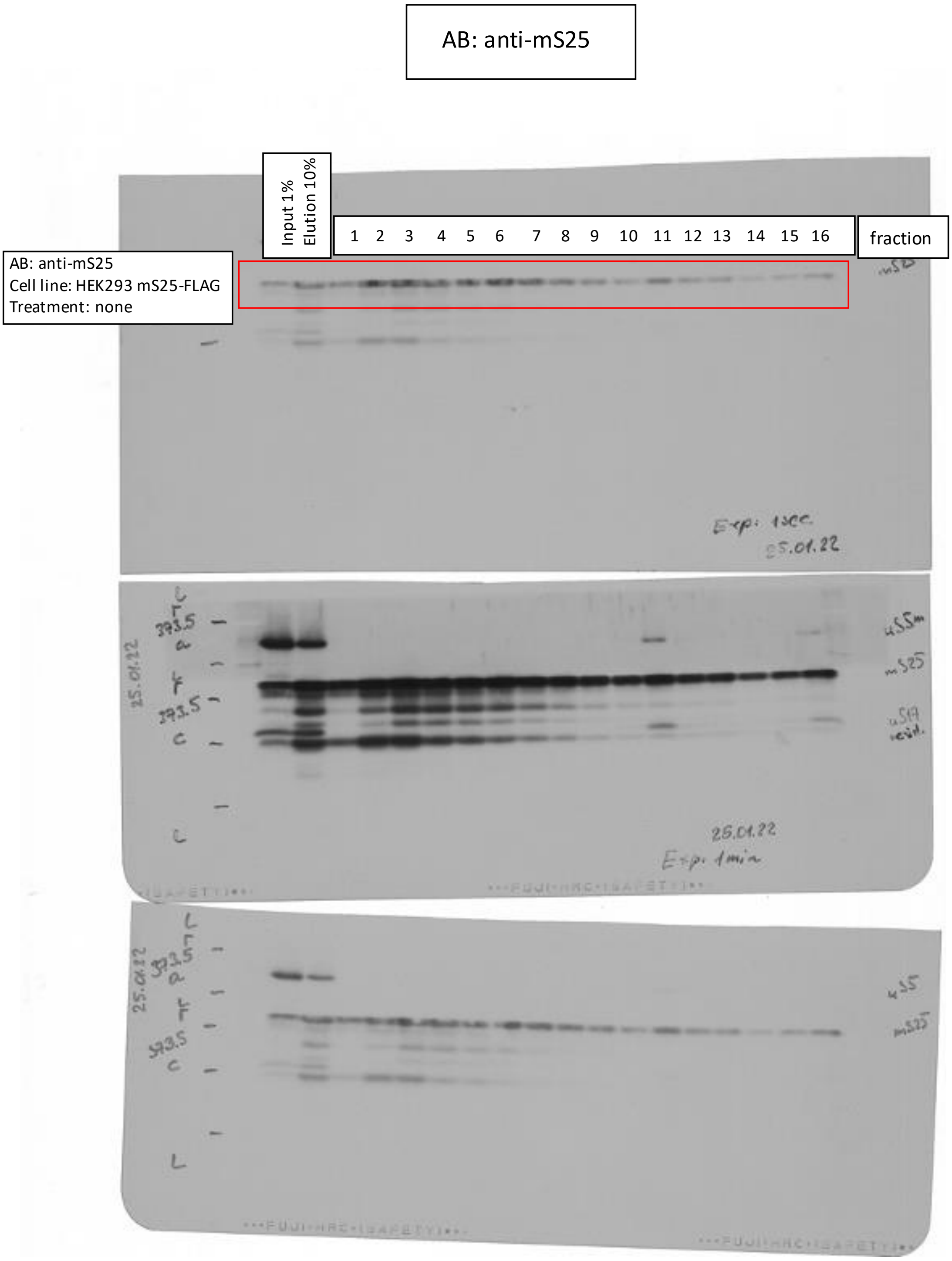

Source Data 2_related to Extended Data Fig.3b
EL#373 mS25-FLAG IP + Gradient
Rotor: SW41 Ti
Gradient: Sucrose 5-30%
Speed: 158.000xg
Time: 15h

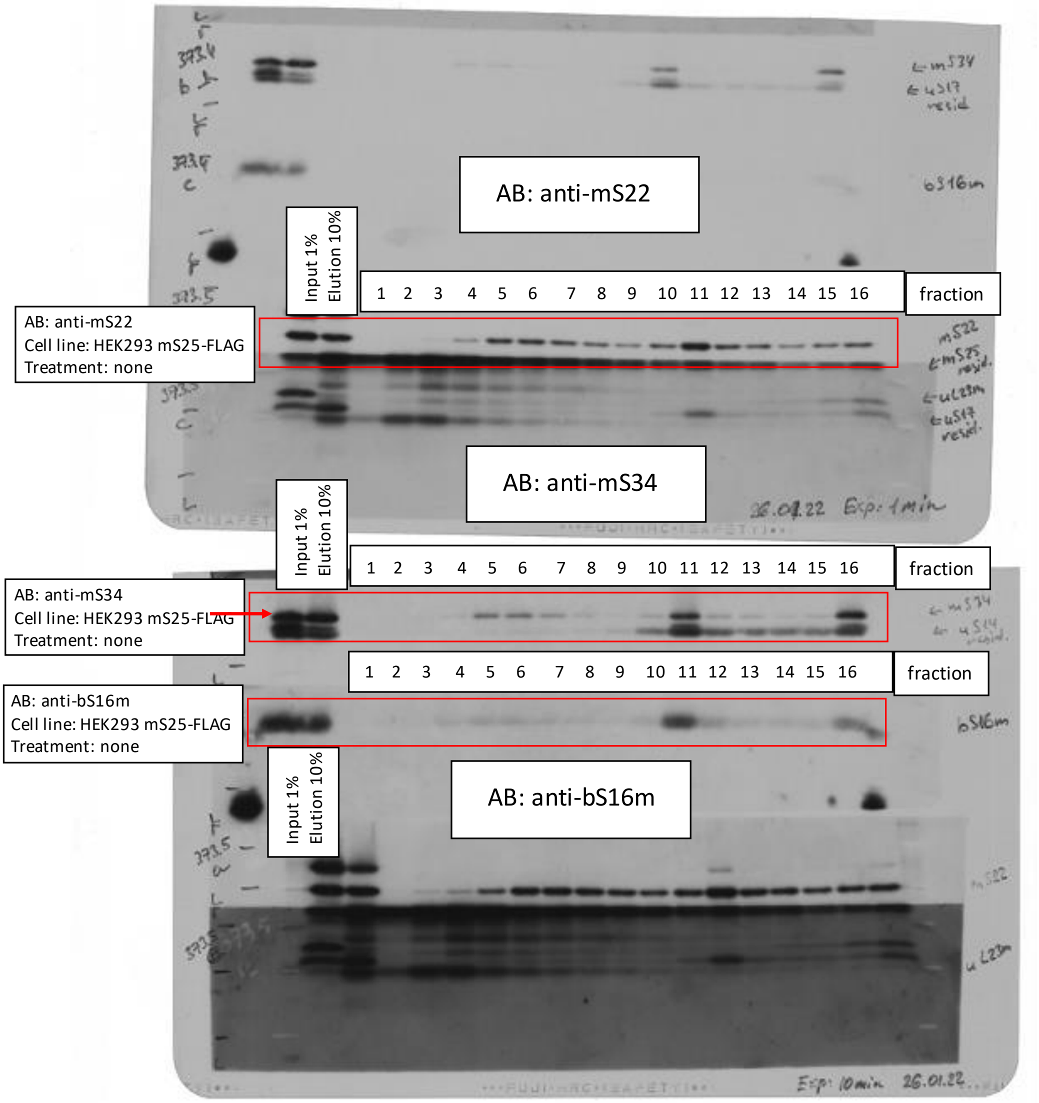

Source Data 2_related to Extended Data Fig.3b
EL#373 mS25-FLAG IP + Gradient
Rotor: SW41 Ti
Gradient: Sucrose 5-30%
Speed: 158.000xg
Time: 15h

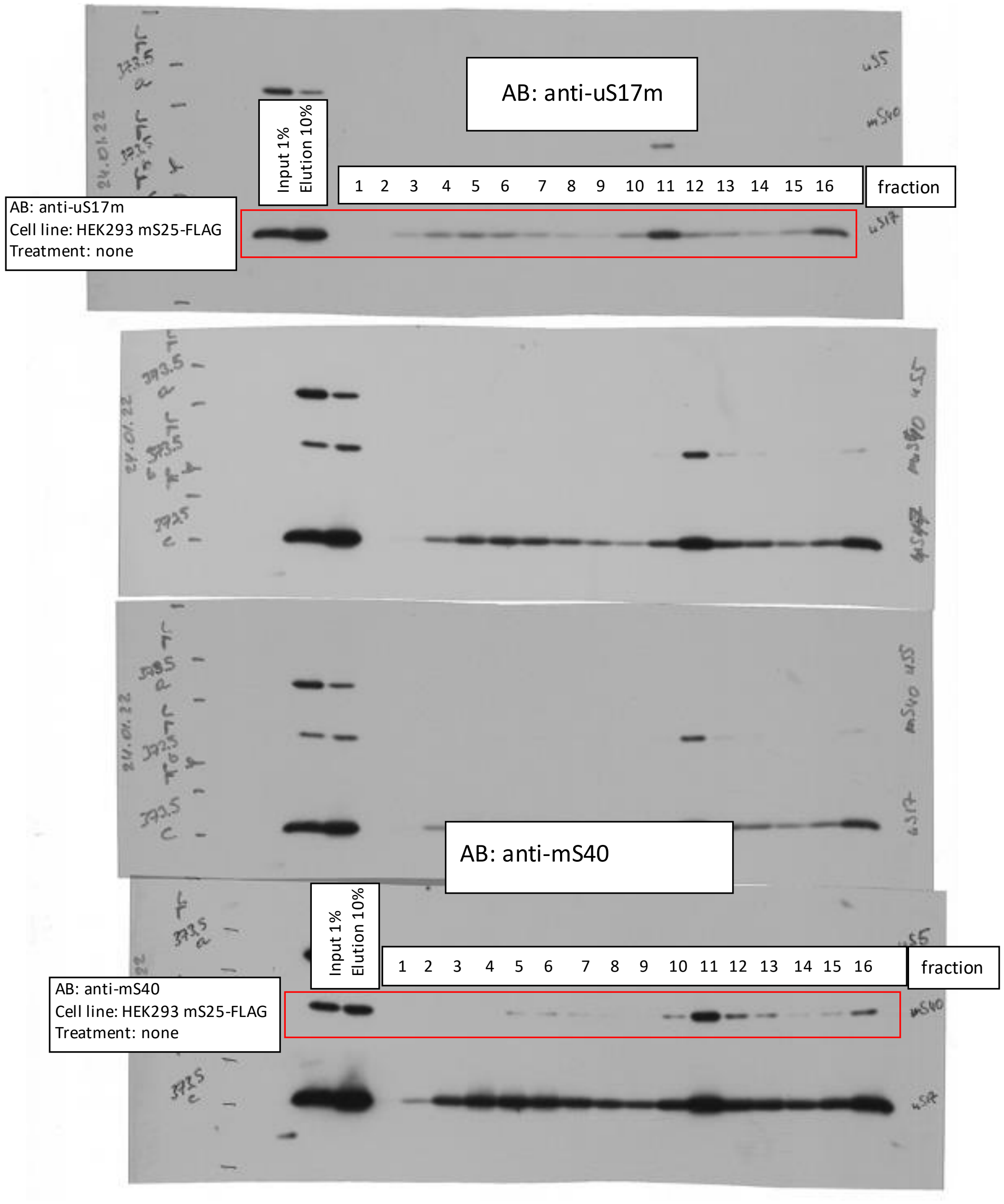

Source Data 2_related to Extended Data Fig.3b
EL#373 mS25-FLAG IP + Gradient
Rotor: SW41 Ti
Gradient: Sucrose 5-30%
Speed: 158.000xg
Time: 15h

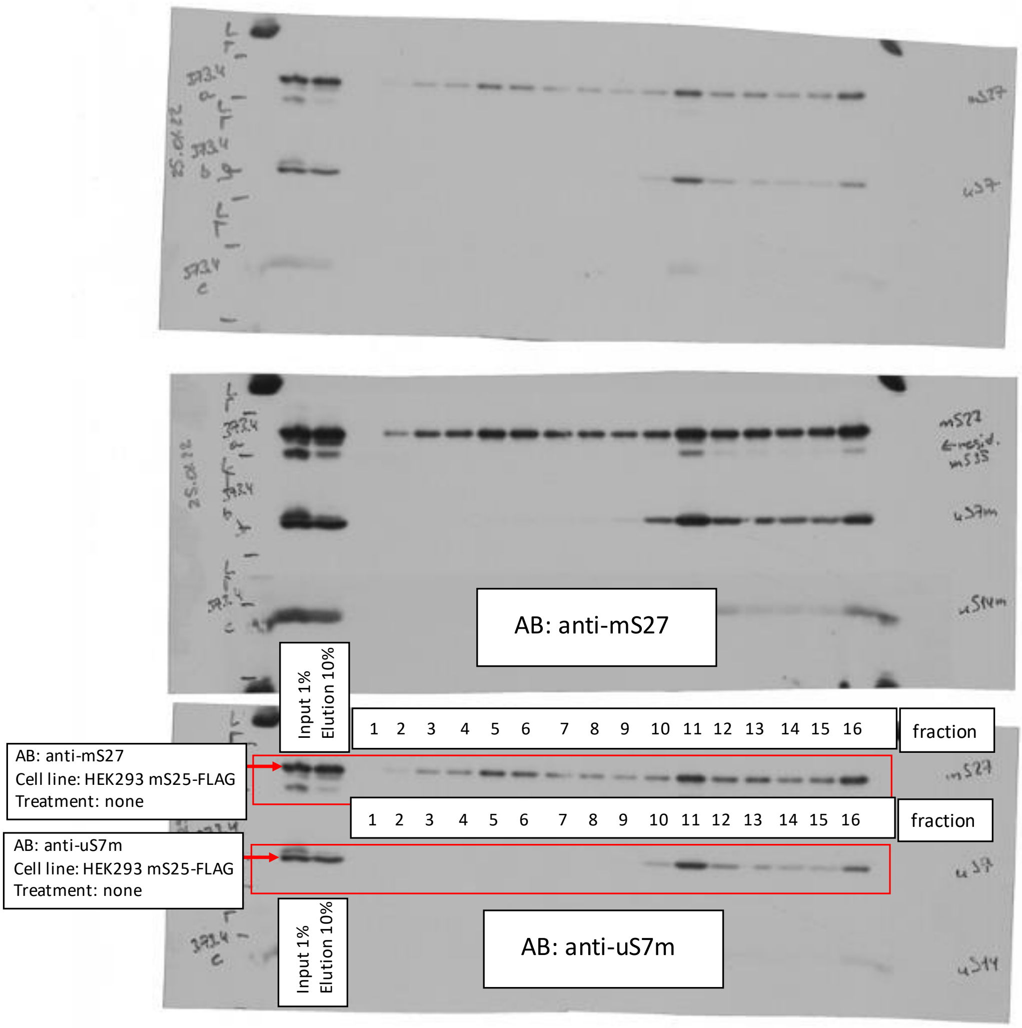

Source Data 2_related to Extended Data Fig.3b
EL#373 mS25-FLAG IP + Gradient
Rotor: SW41 Ti
Gradient: Sucrose 5-30%
Speed: 158.000xg
Time: 15h

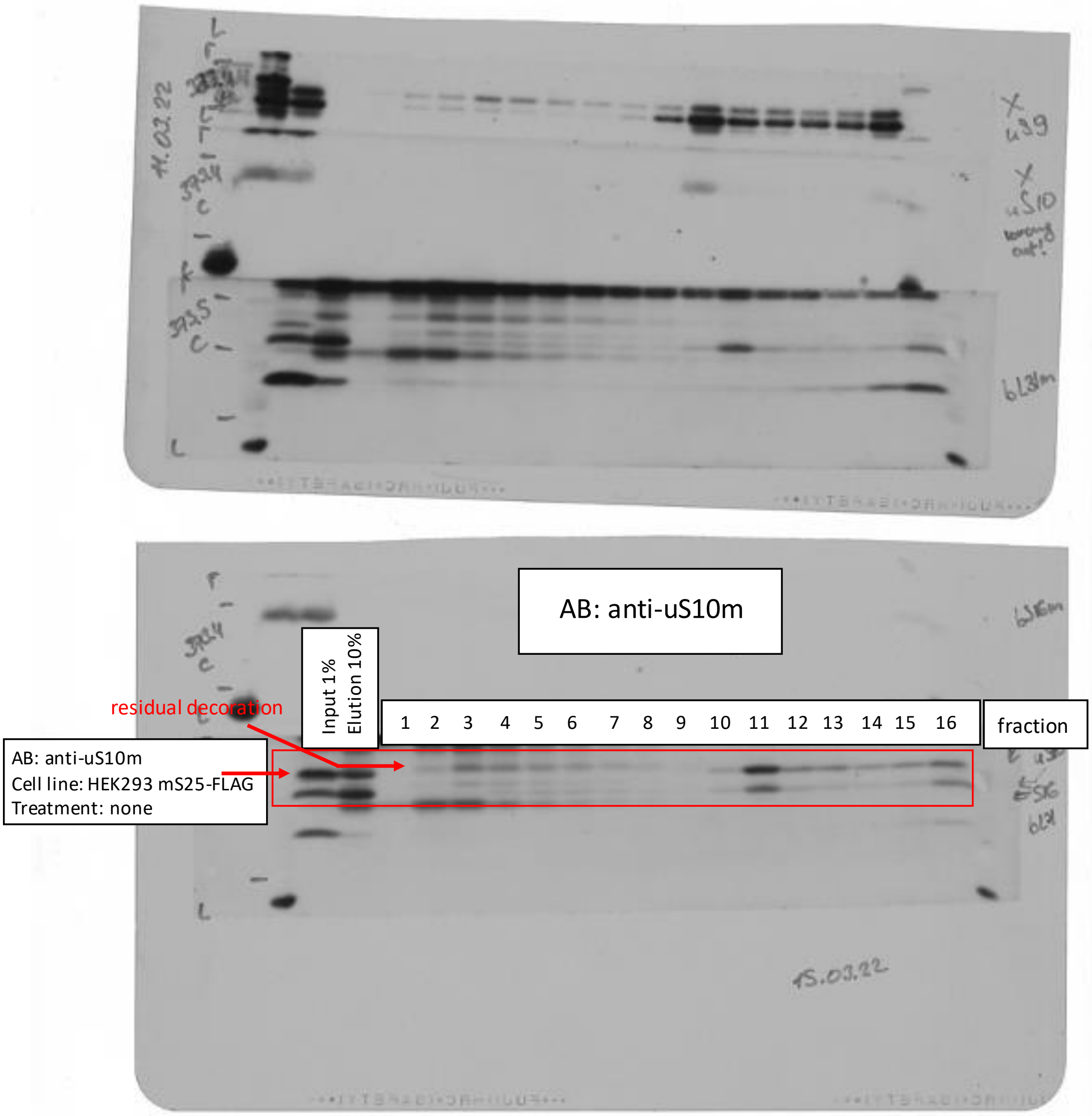

Source Data 2_related to Extended Data Fig.3b
EL#373 mS25-FLAG IP + Gradient
Rotor: SW41 Ti
Gradient: Sucrose 5-30%
Speed: 158.000xg
Time: 15h

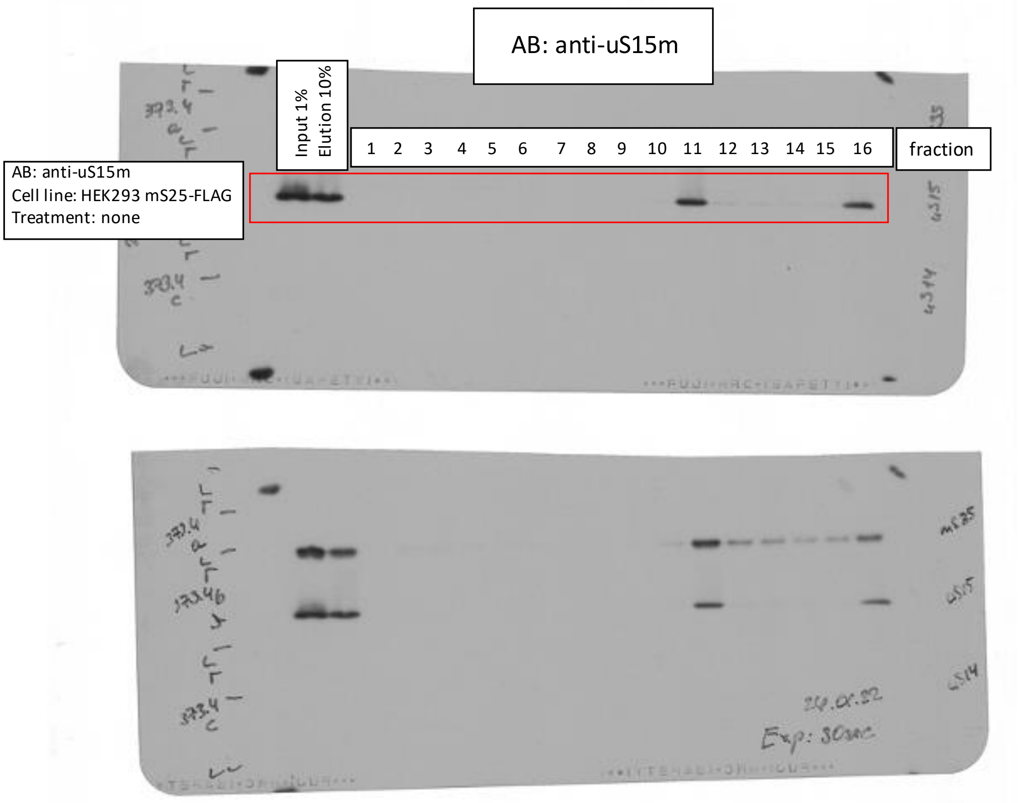

Source Data 2_related to Extended Data Fig.3b
EL#373 mS25-FLAG IP + Gradient
Rotor: SW41 Ti
Gradient: Sucrose 5-30%
Speed: 158.000xg
Time: 15h

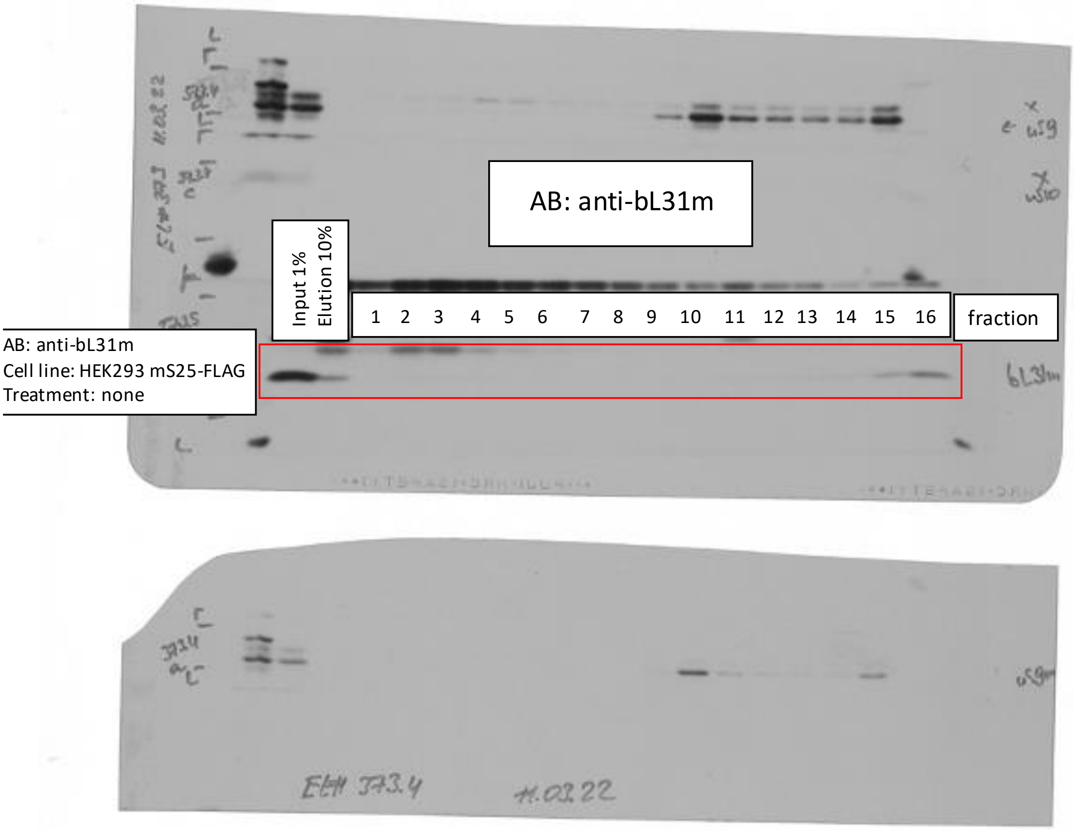

Source Data 2_related to Extended Data Fig.3b
EL#373 mS25-FLAG IP + Gradient
Rotor: SW41 Ti
Gradient: Sucrose 5-30%
Speed: 158.000xg
Time: 15h

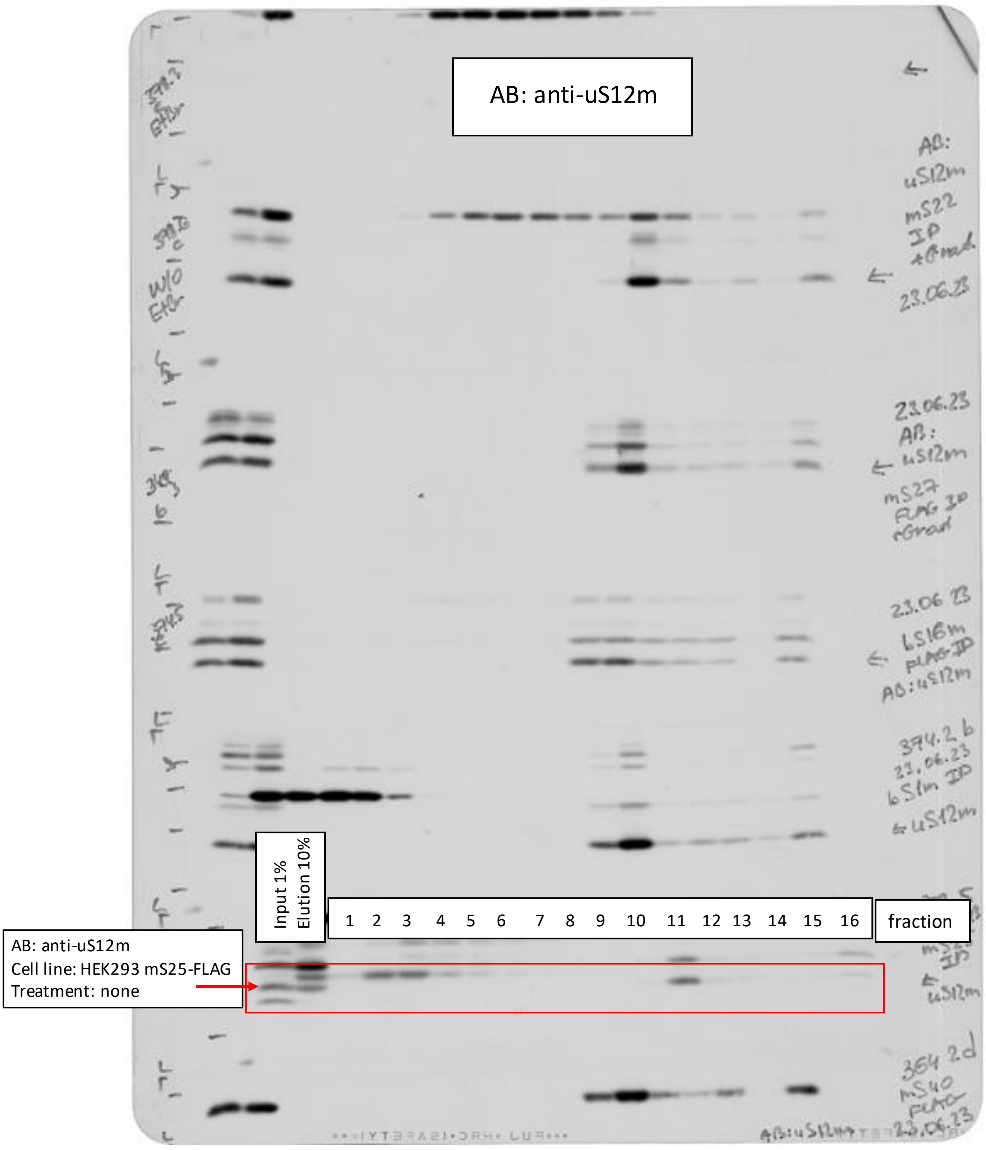

Source Data 2_related to Extended Data Fig.3c
EL#364 mS40-FLAG IP + Gradient
Rotor: SW41 Ti
Gradient: Sucrose 5-30%
Speed: 158.000xg
Time: 15h

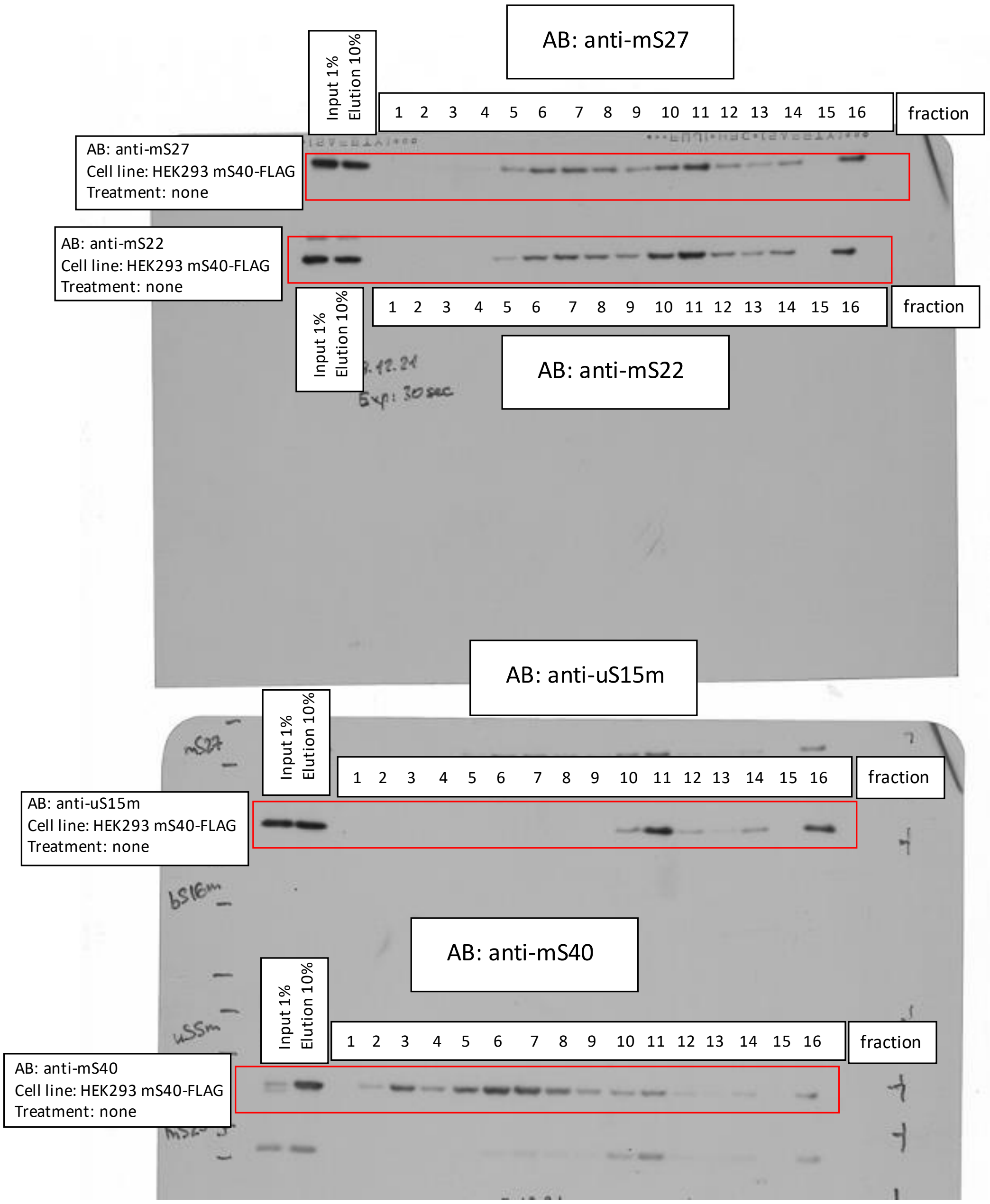

Source Data 2_related to Extended Data Fig.3c
EL#364 mS40-FLAG IP + Gradient
Rotor: SW41 Ti
Gradient: Sucrose 5-30%
Speed: 158.000xg
Time: 15h

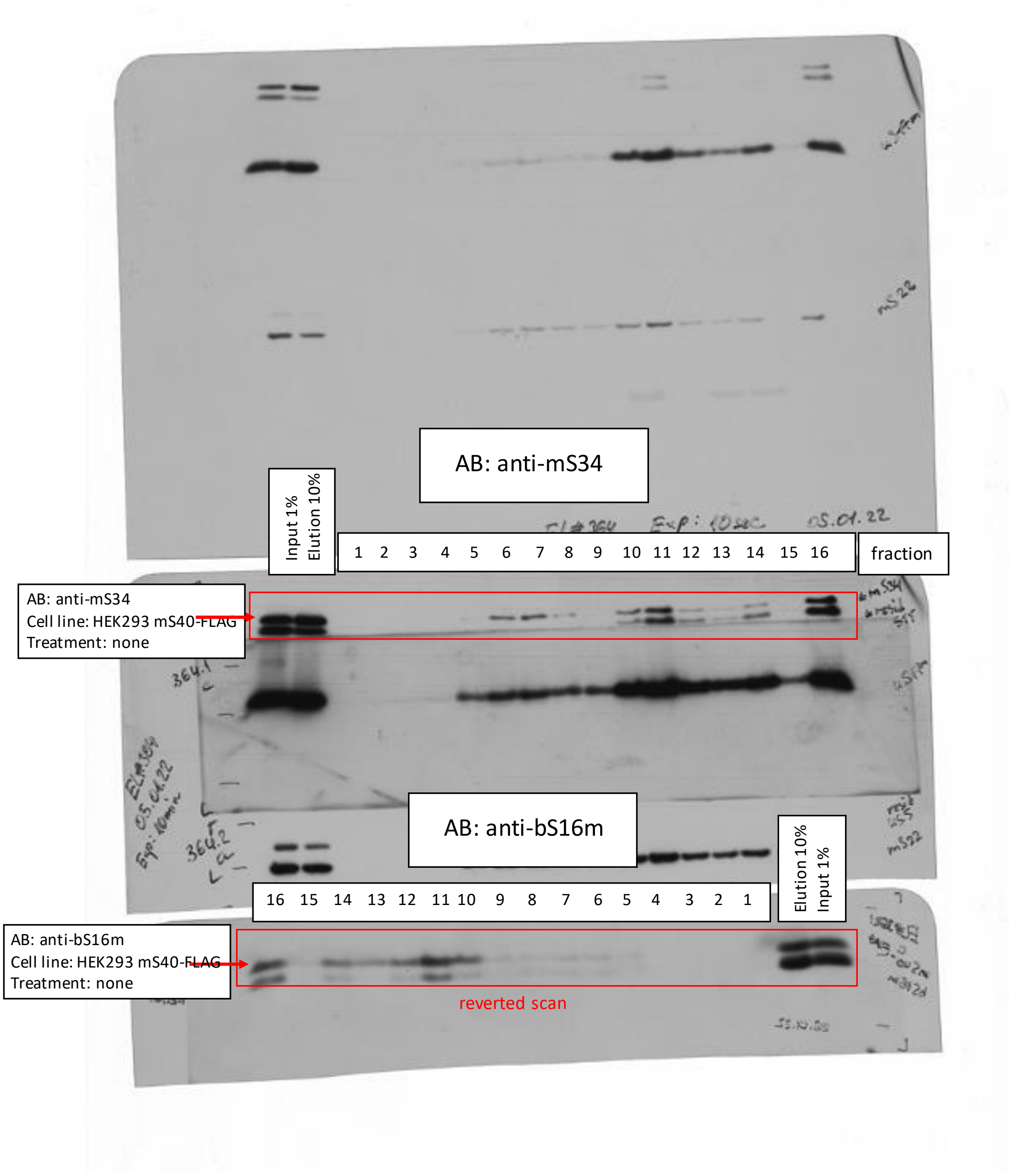

Source Data 2_related to Extended Data Fig.3c
EL#364 mS40-FLAG IP + Gradient
Rotor: SW41 Ti
Gradient: Sucrose 5-30%
Speed: 158.000xg
Time: 15h

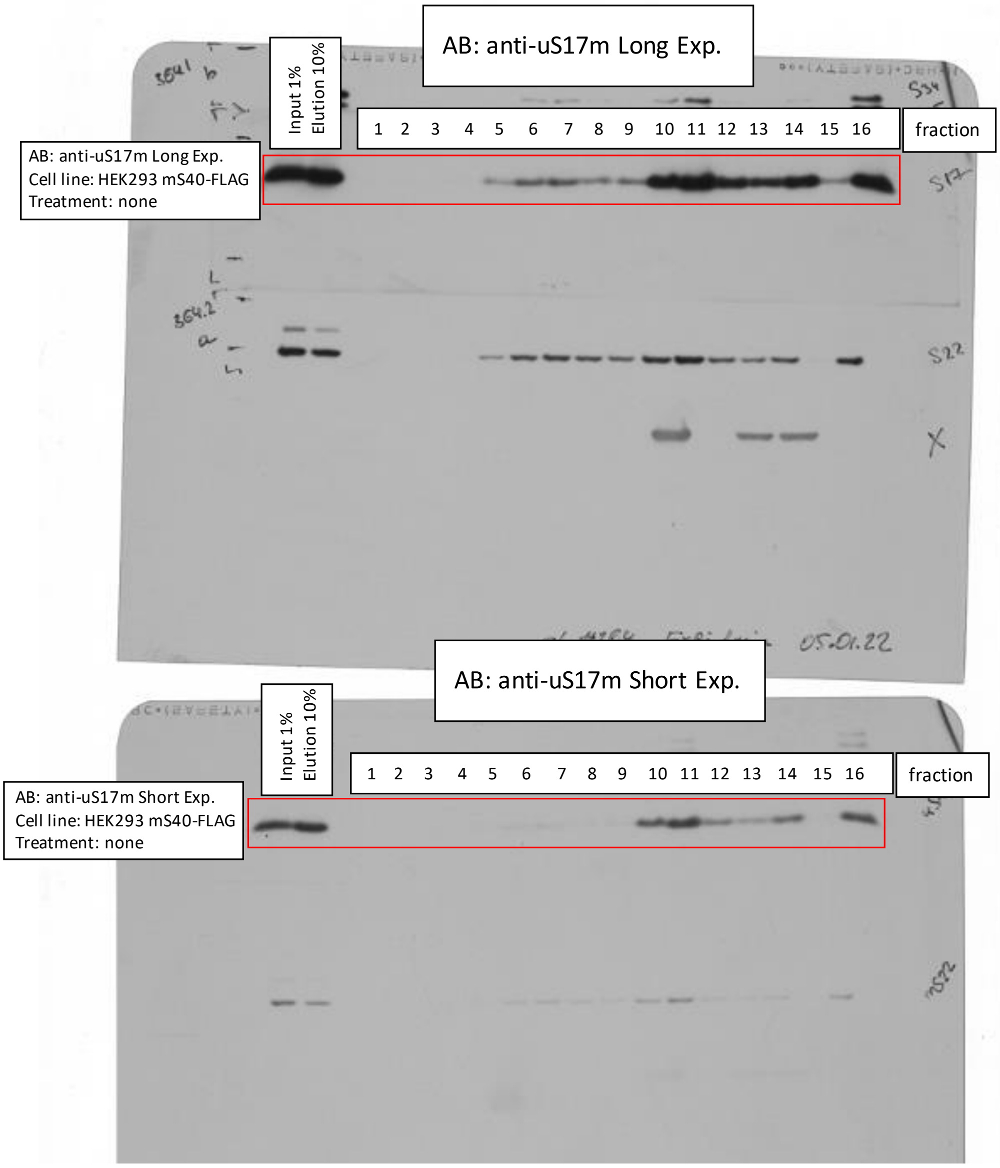

Source Data 2_related to Extended Data Fig.3c
EL#364 mS40-FLAG IP + Gradient
Rotor: SW41 Ti
Gradient: Sucrose 5-30%
Speed: 158.000xg
Time: 15h

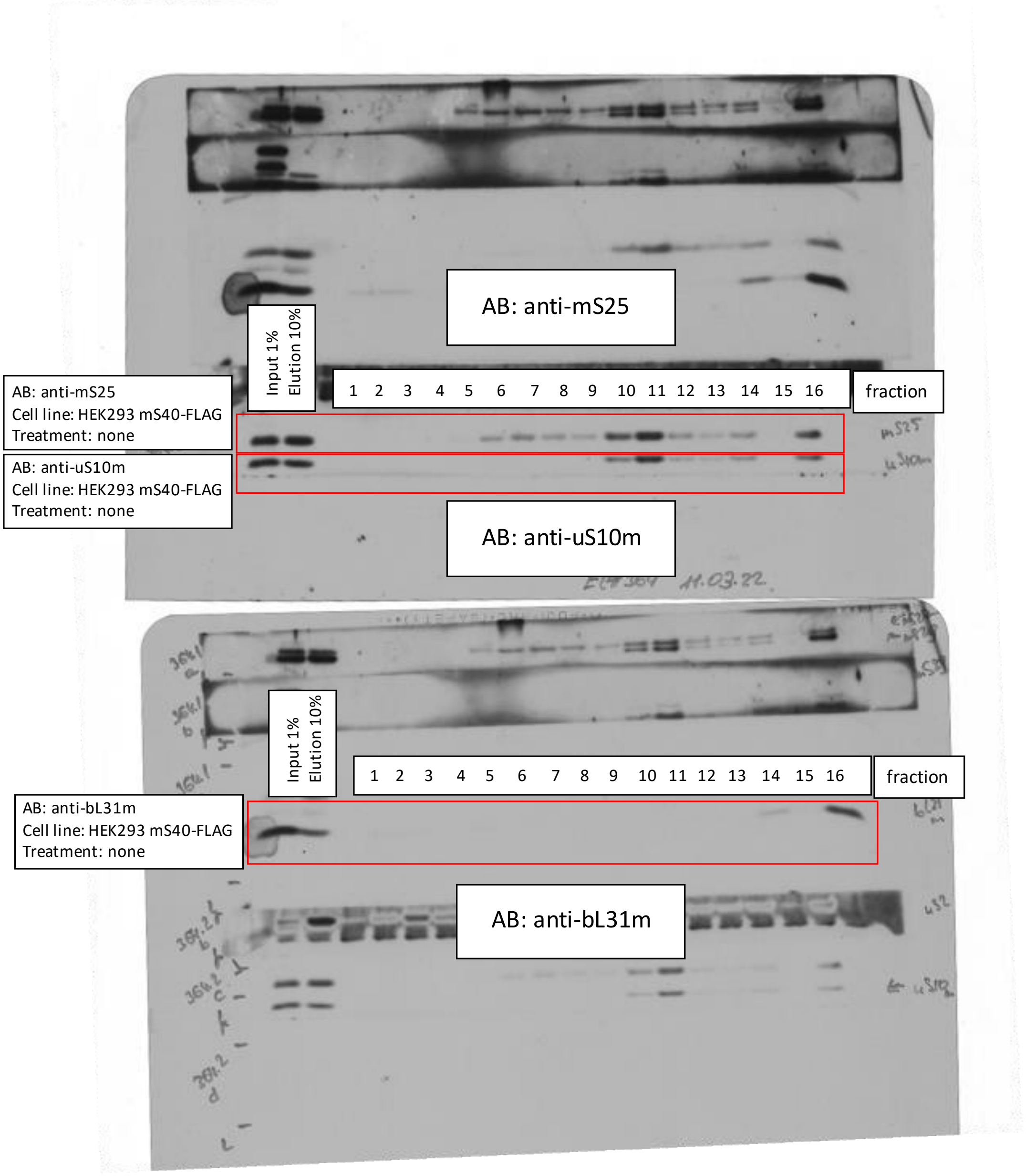

Source Data 2_related to Extended Data Fig.3c
EL#364 mS40-FLAG IP + Gradient
Rotor: SW41 Ti
Gradient: Sucrose 5-30%
Speed: 158.000xg
Time: 15h

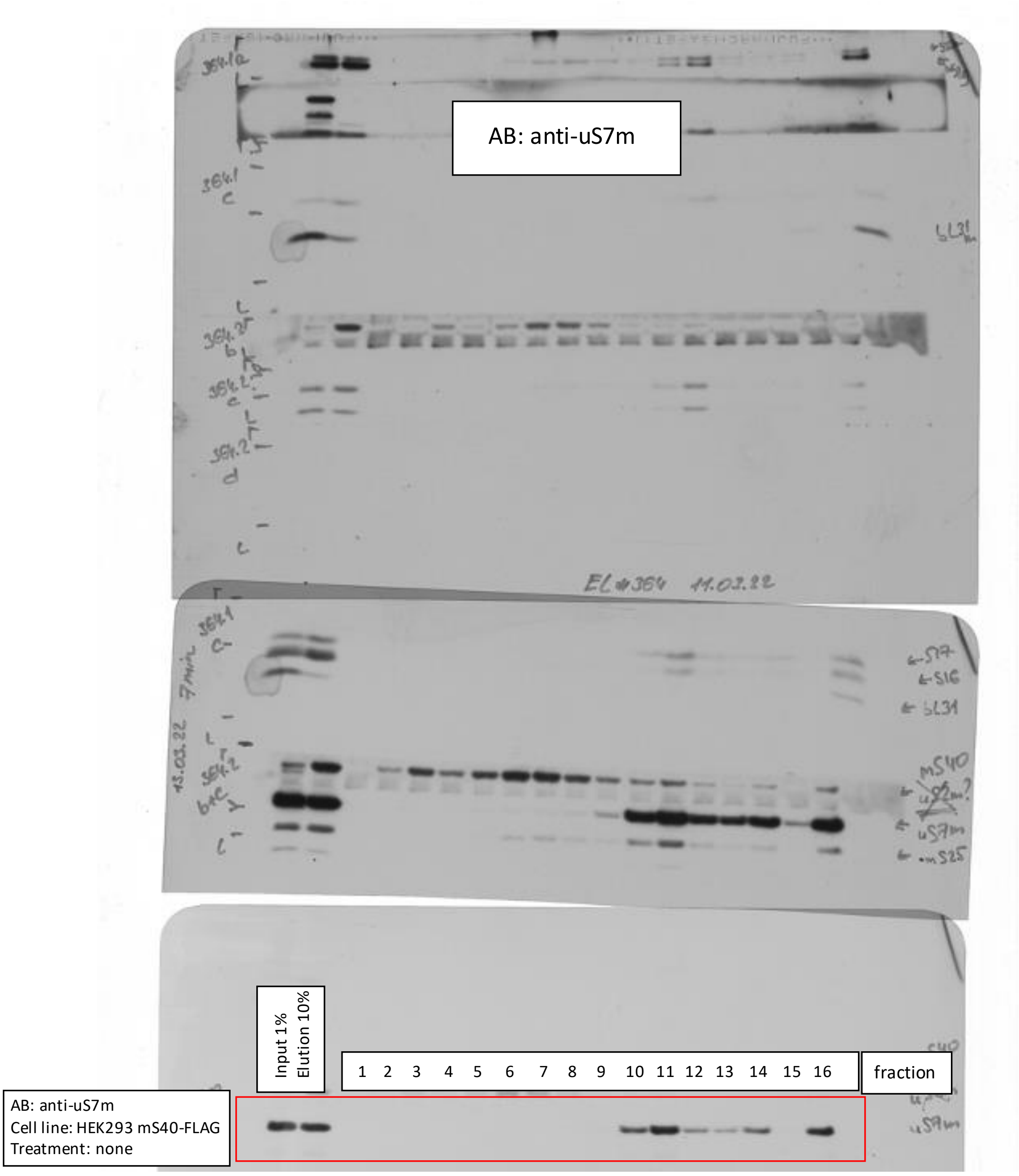

Source Data 2_related to Extended Data Fig.3c
EL#364 mS40-FLAG IP + Gradient
Rotor: SW41 Ti
Gradient: Sucrose 5-30%
Speed: 158.000xg
Time: 15h

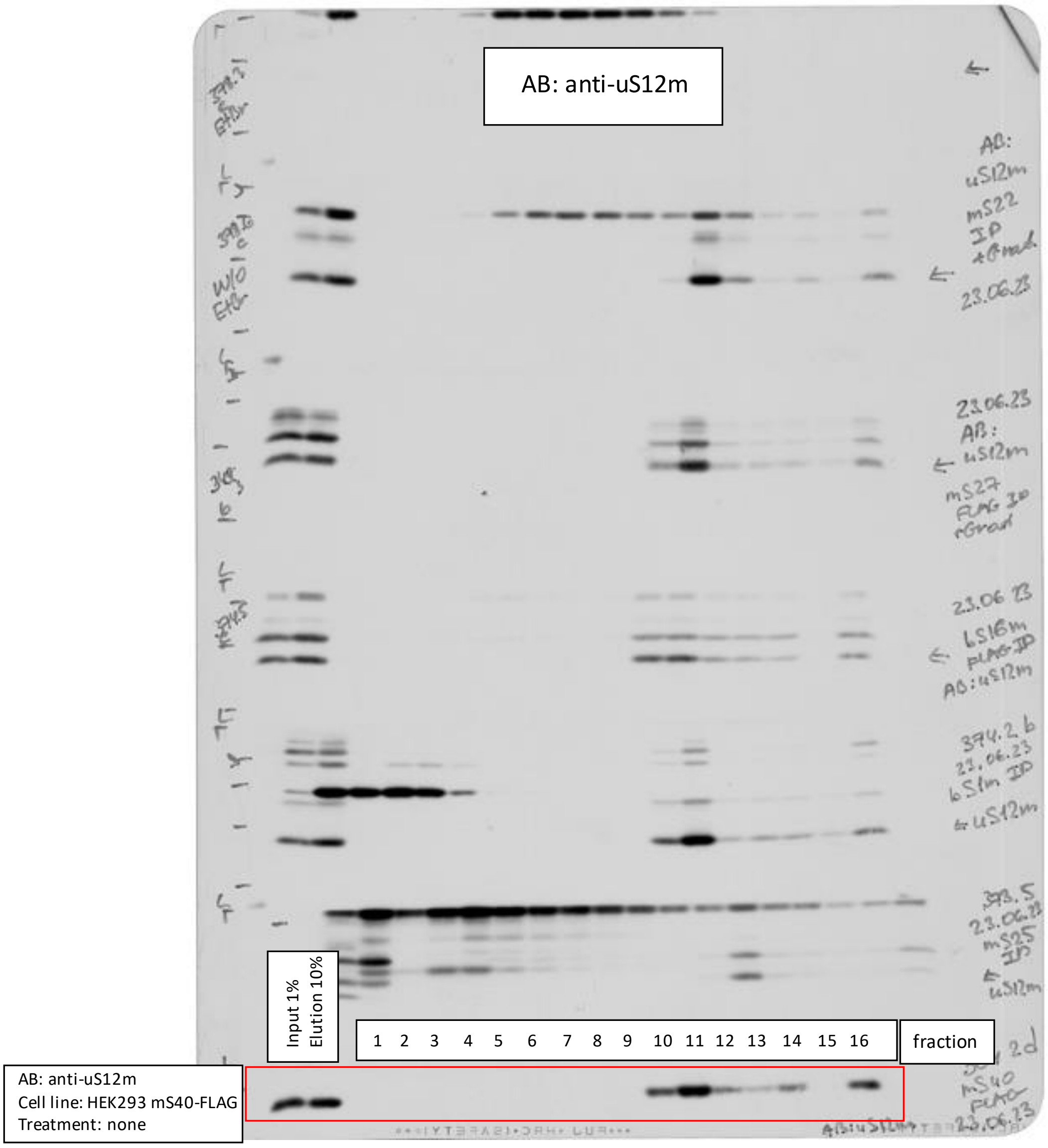

Source Data 2_related to Extended Data Fig.3d
AS#117 mS40 KO + Gradient
Rotor: SW41 Ti
Gradient: Sucrose 5-30%
Speed: 158.000xg
Time: 15h

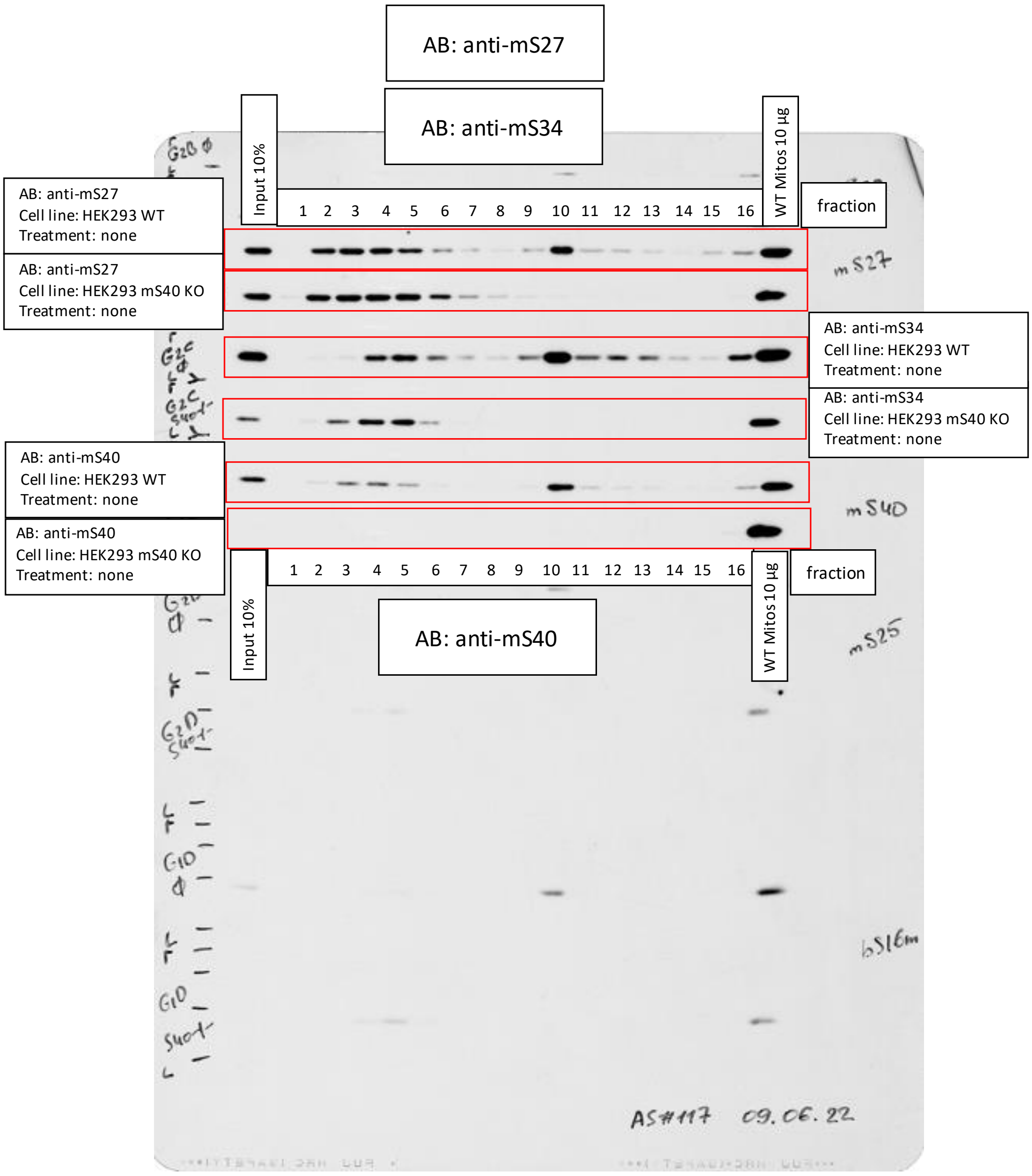

Source Data 2_related to Extended Data Fig.3d
AS#117 mS40 KO + Gradient
Rotor: SW41 Ti
Gradient: Sucrose 5-30%
Speed: 158.000xg
Time: 15h

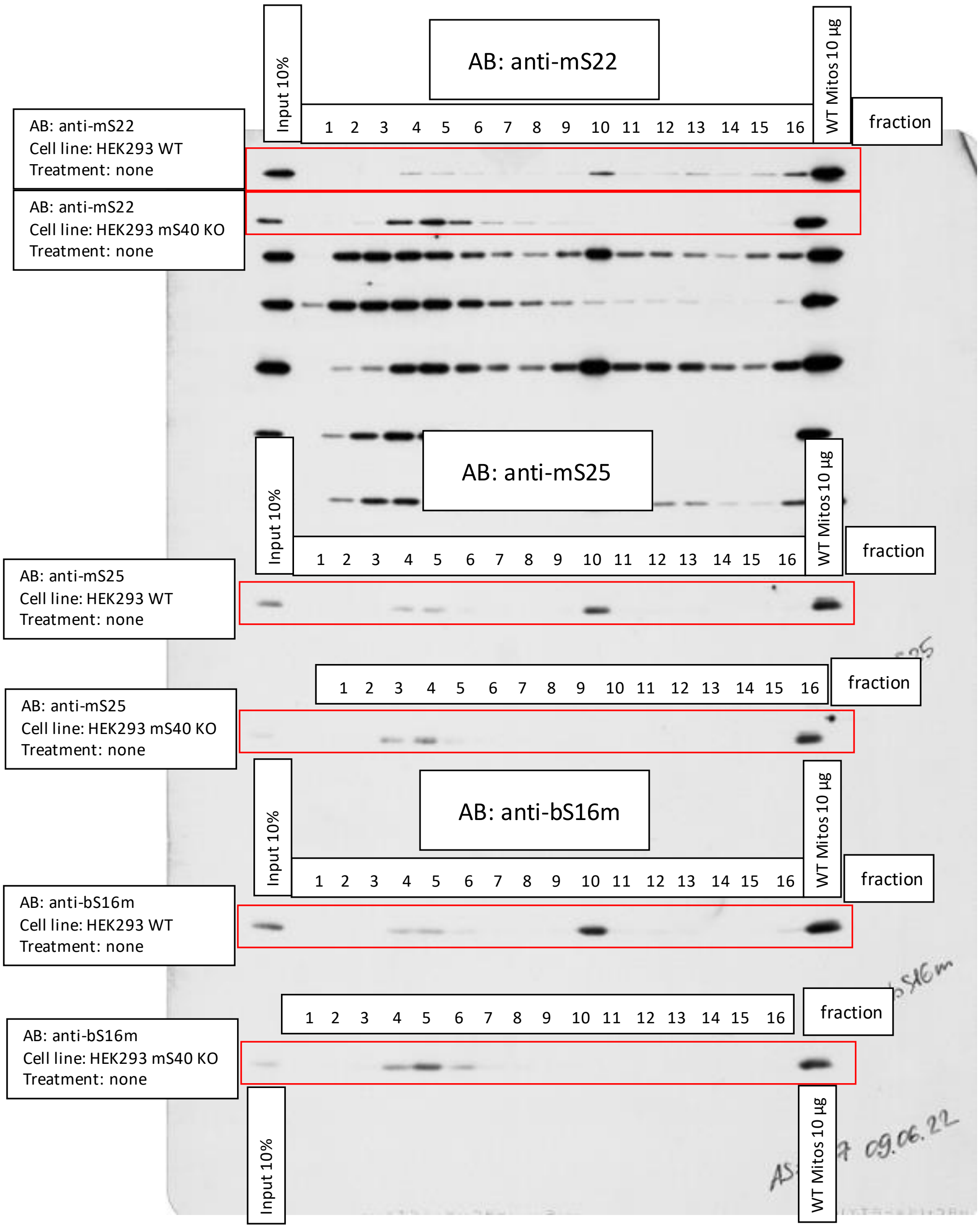

Source Data 2_related to Extended Data Fig.3d
AS#117 mS40 KO + Gradient
Rotor: SW41 Ti
Gradient: Sucrose 5-30%
Speed: 158.000xg
Time: 15h

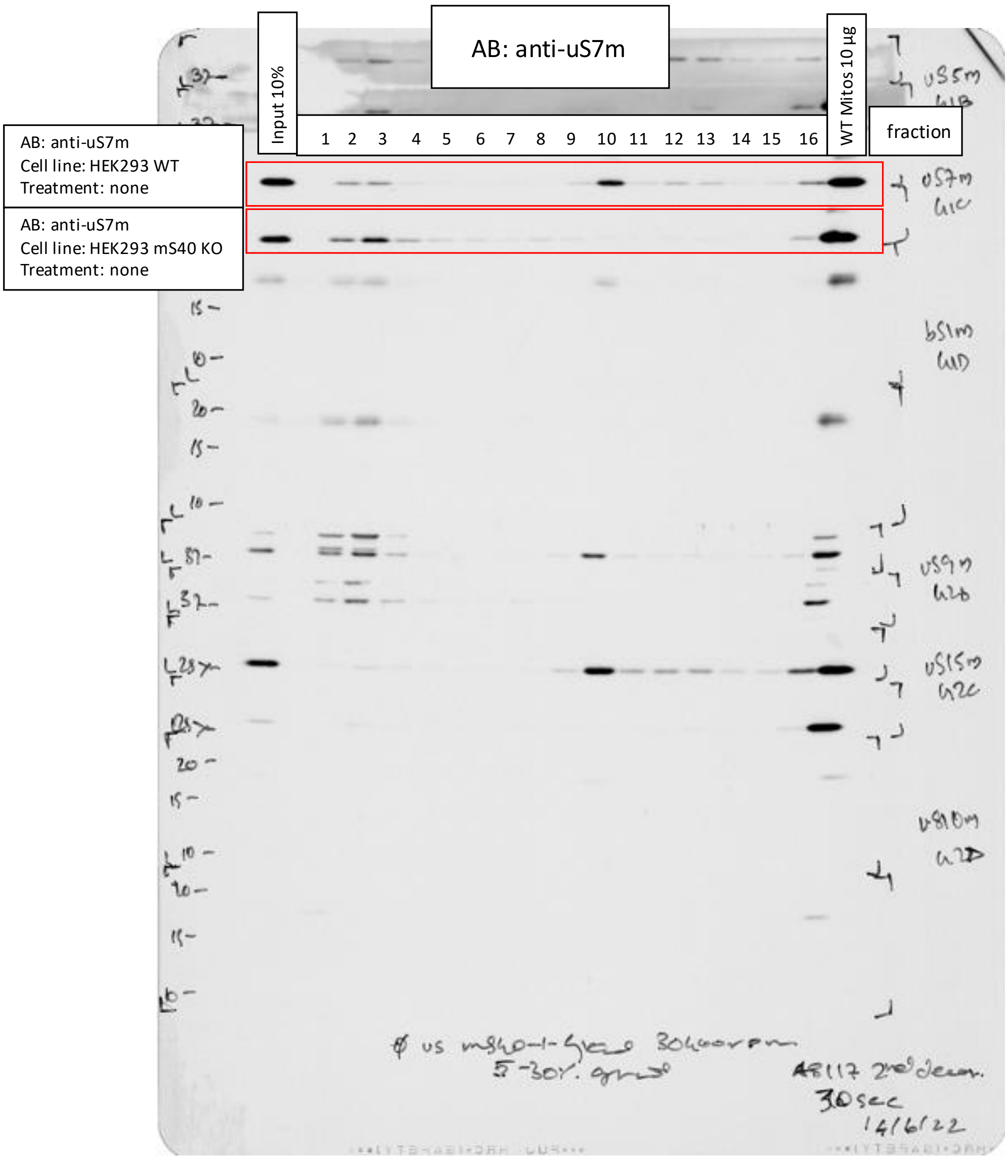

Source Data 2_related to Extended Data Fig.3d
AS#117 mS40 KO + Gradient
Rotor: SW41 Ti
Gradient: Sucrose 5-30%
Speed: 158.000xg
Time: 15h

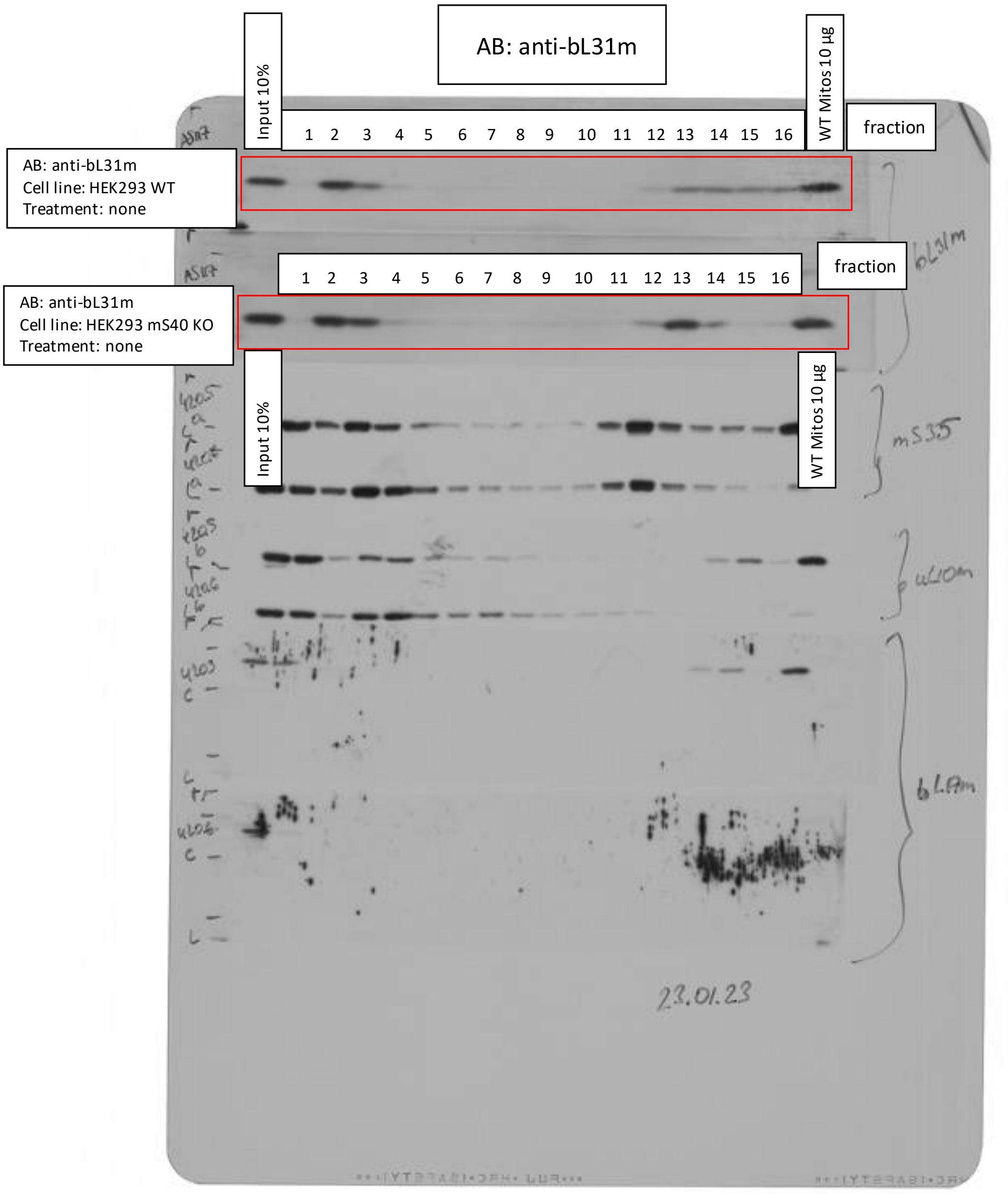

Source Data 2_related to Extended Data Fig.3e
EL#374.1-3 bS1m-FLAG IP + Gradient
Rotor: SW41 Ti
Gradient: Sucrose 5-30%
Speed: 158.000xg
Time: 15h

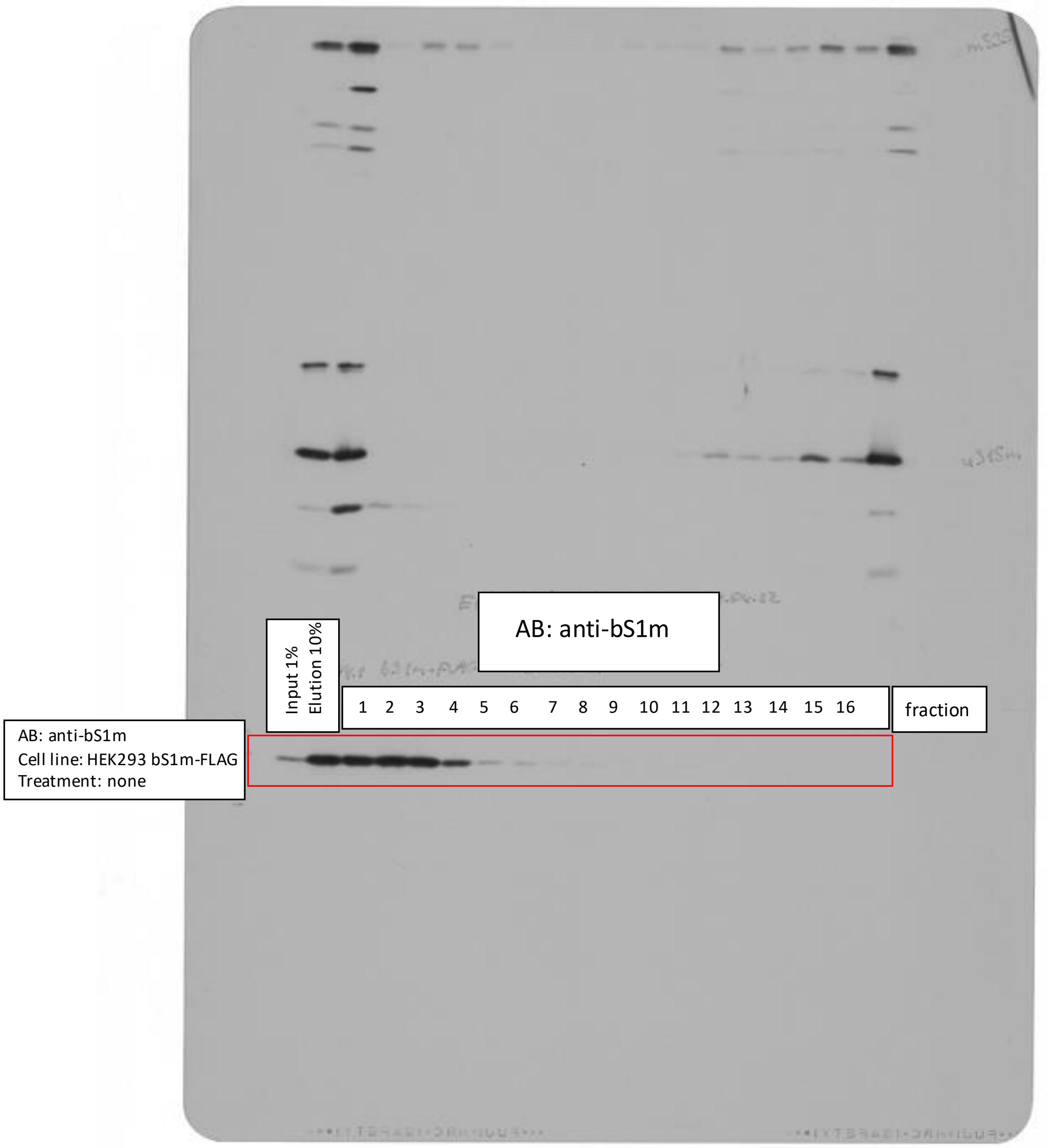

Source Data 2_related to Extended Data Fig.3e
EL#374.1-3 bS1m-FLAG IP + Gradient
Rotor: SW41 Ti
Gradient: Sucrose 5-30%
Speed: 158.000xg
Time: 15h

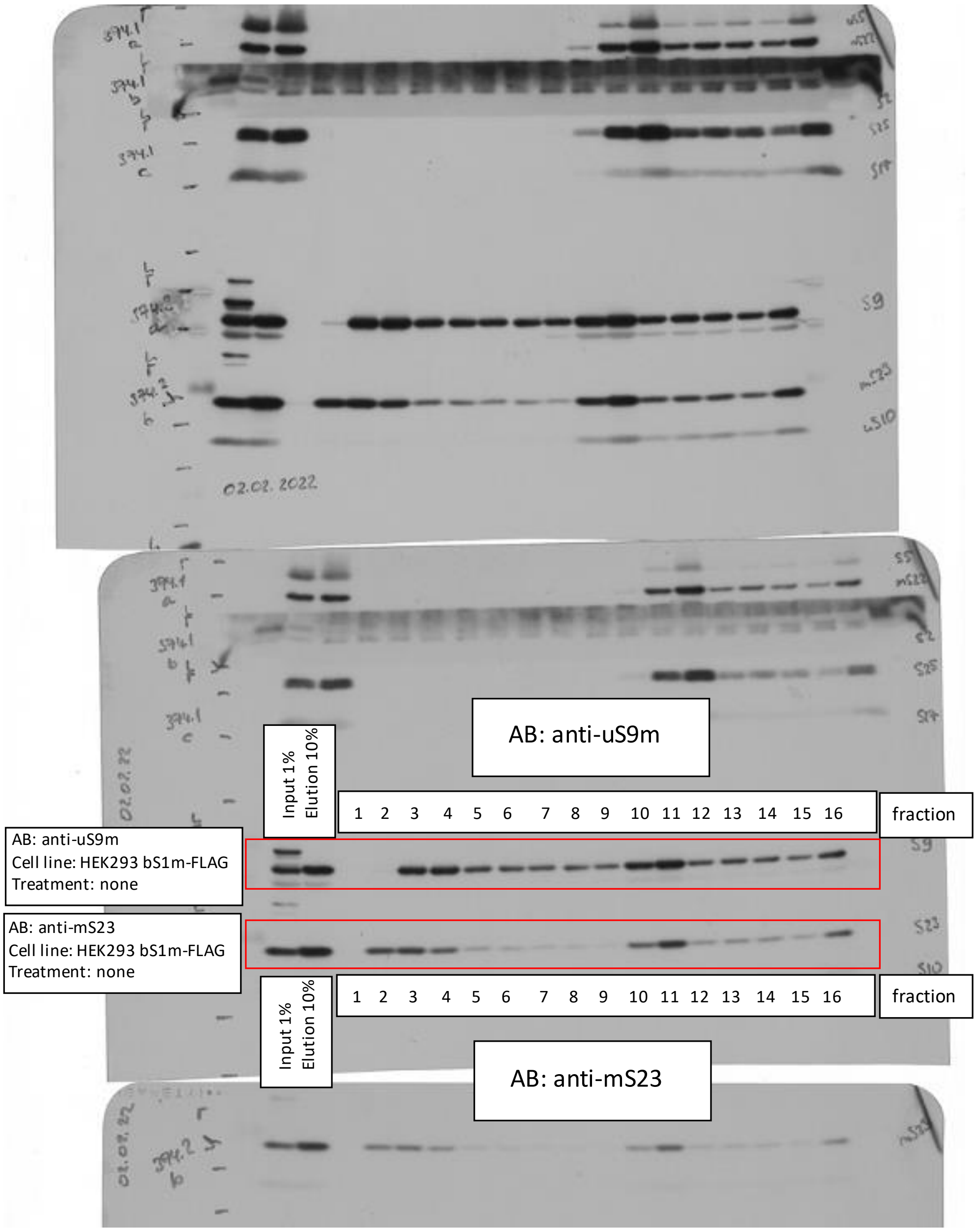

Source Data 2_related to Extended Data Fig.3e
EL#374.1-3 bS1m-FLAG IP + Gradient
Rotor: SW41 Ti
Gradient: Sucrose 5-30%
Speed: 158.000xg
Time: 15h

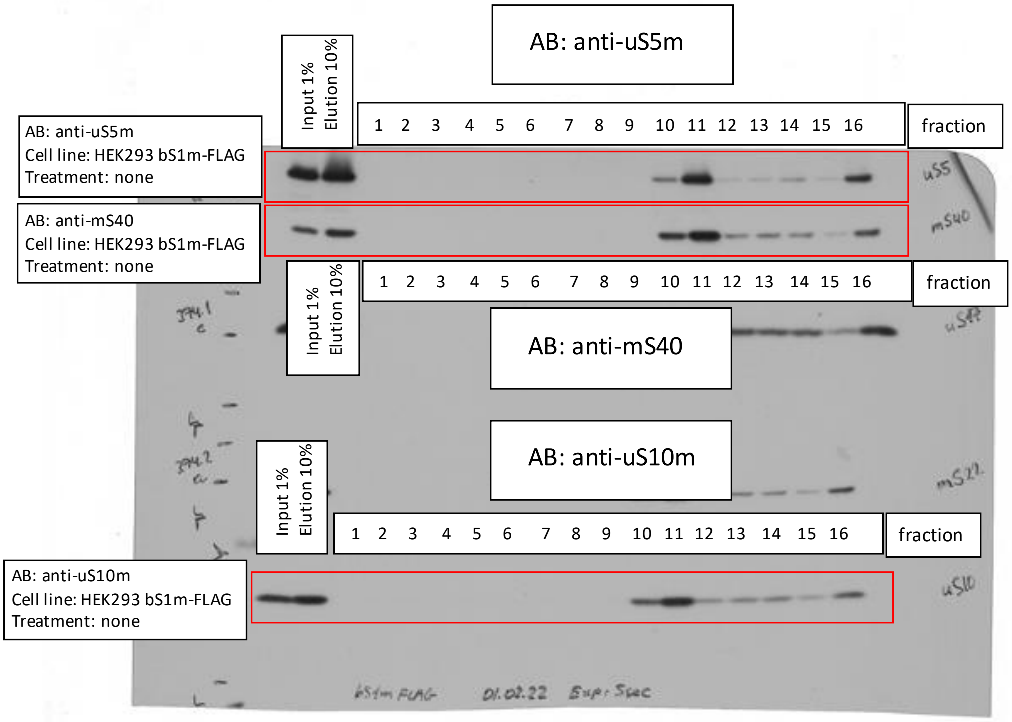

Source Data 2_related to Extended Data Fig.3e
EL#374.1-3 bS1m-FLAG IP + Gradient
Rotor: SW41 Ti
Gradient: Sucrose 5-30%
Speed: 158.000xg
Time: 15h

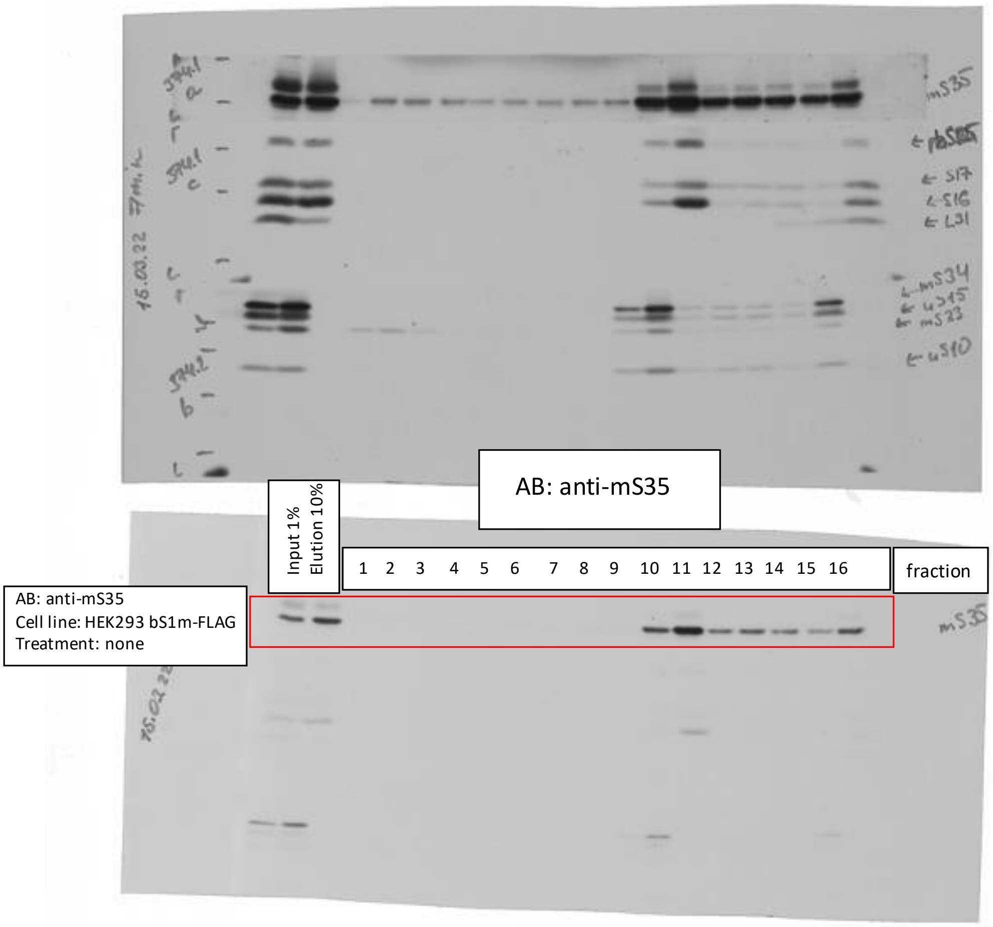

Source Data 2_related to Extended Data Fig.3e
EL#374.1-3 bS1m-FLAG IP + Gradient
Rotor: SW41 Ti
Gradient: Sucrose 5-30%
Speed: 158.000xg
Time: 15h

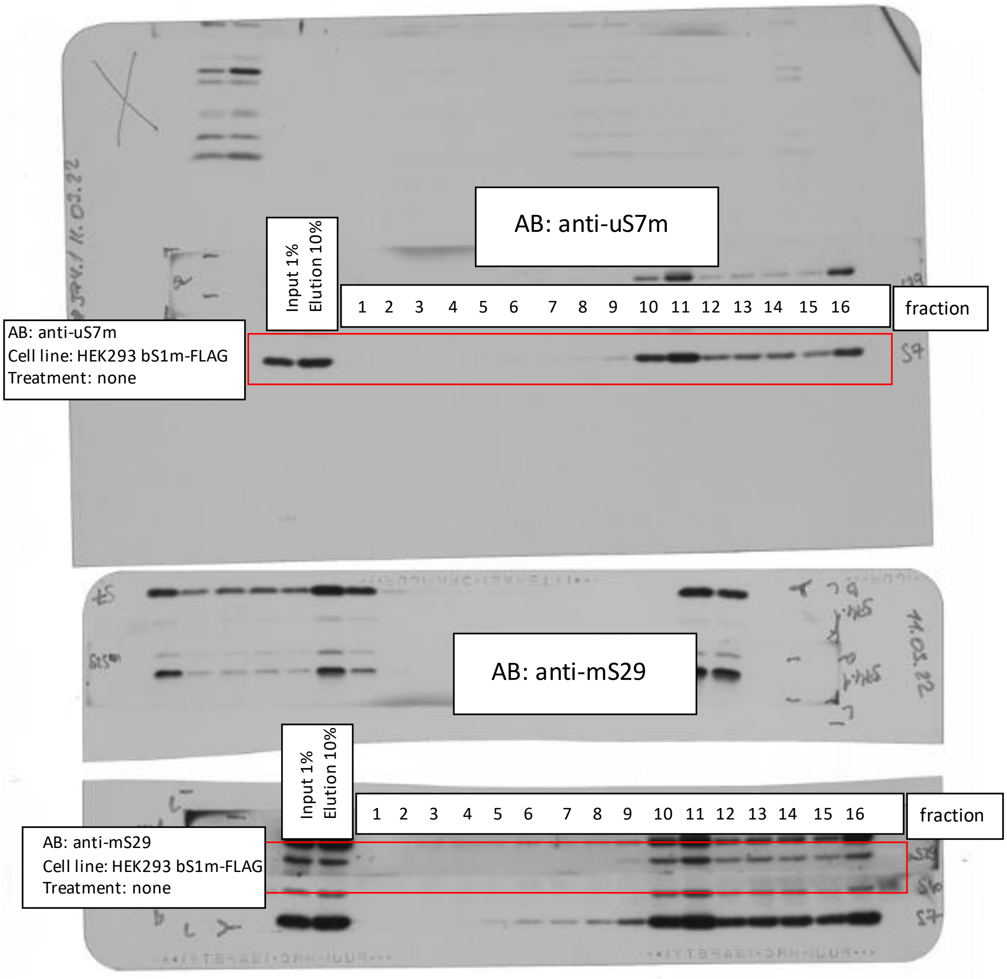

Source Data 2_related to Extended Data Fig.3e
EL#374.1-3 bS1m-FLAG IP + Gradient
Rotor: SW41 Ti
Gradient: Sucrose 5-30%
Speed: 158.000xg
Time: 15h

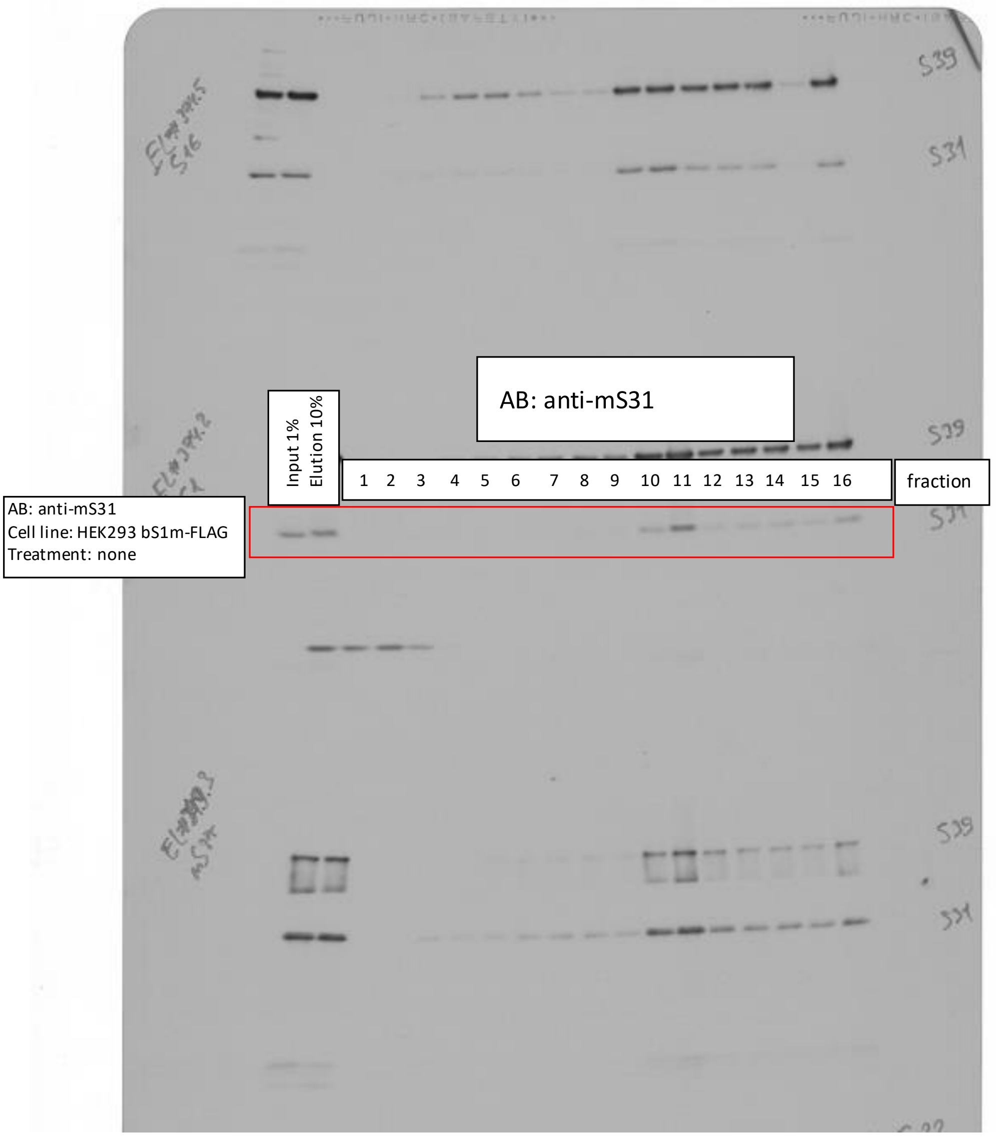

Source Data 2_related to Extended Data Fig.3e
EL#374.1-3 bS1m-FLAG IP + Gradient
Rotor: SW41 Ti
Gradient: Sucrose 5-30%
Speed: 158.000xg
Time: 15h

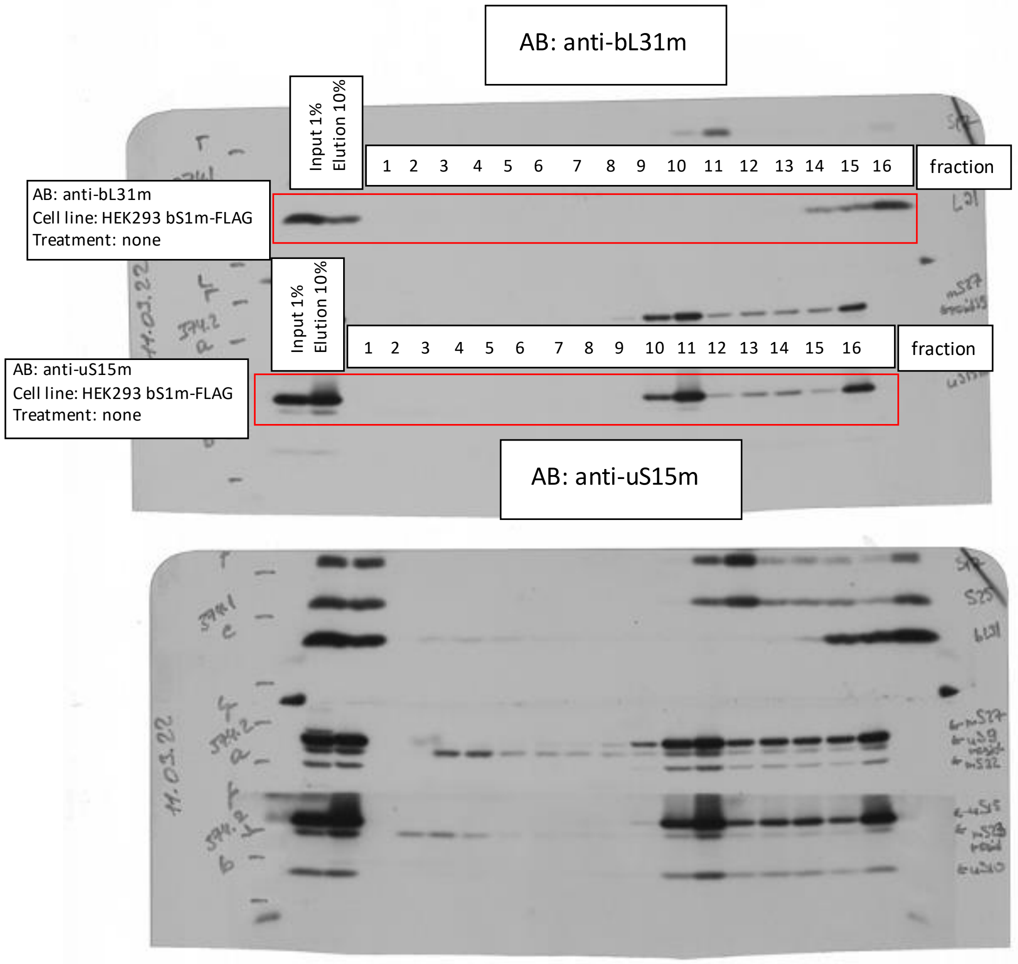

Source Data 2_related to Extended Data Fig.3f
EL#392.1-2 uS10m-FLAG IP + Gradient
Rotor: SW41 Ti
Gradient: Sucrose 5-30%
Speed: 158.000xg
Time: 15h

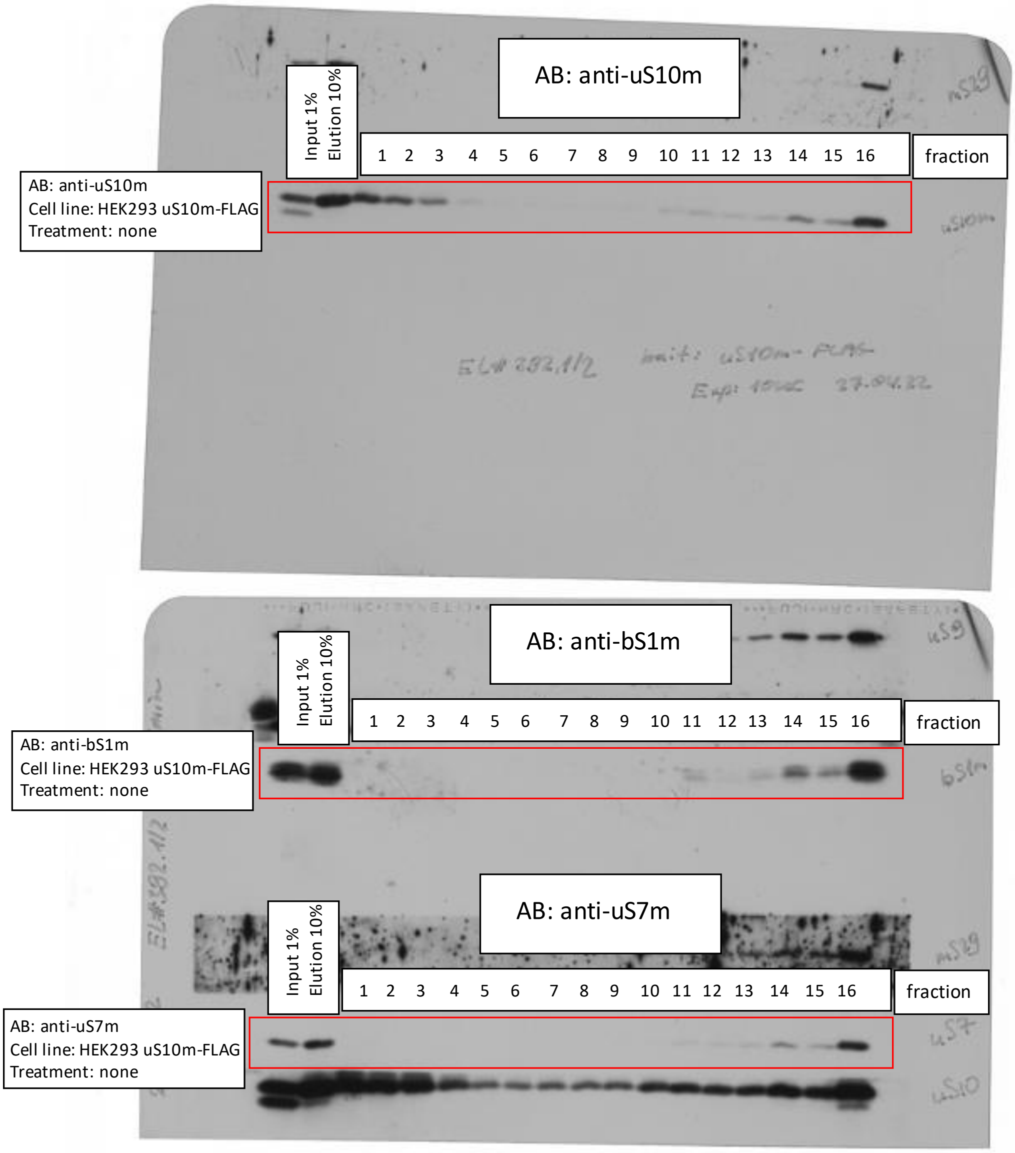

Source Data 2_related to Extended Data Fig.3f
EL#392.1-2 uS10m-FLAG IP + Gradient
Rotor: SW41 Ti
Gradient: Sucrose 5-30%
Speed: 158.000xg
Time: 15h

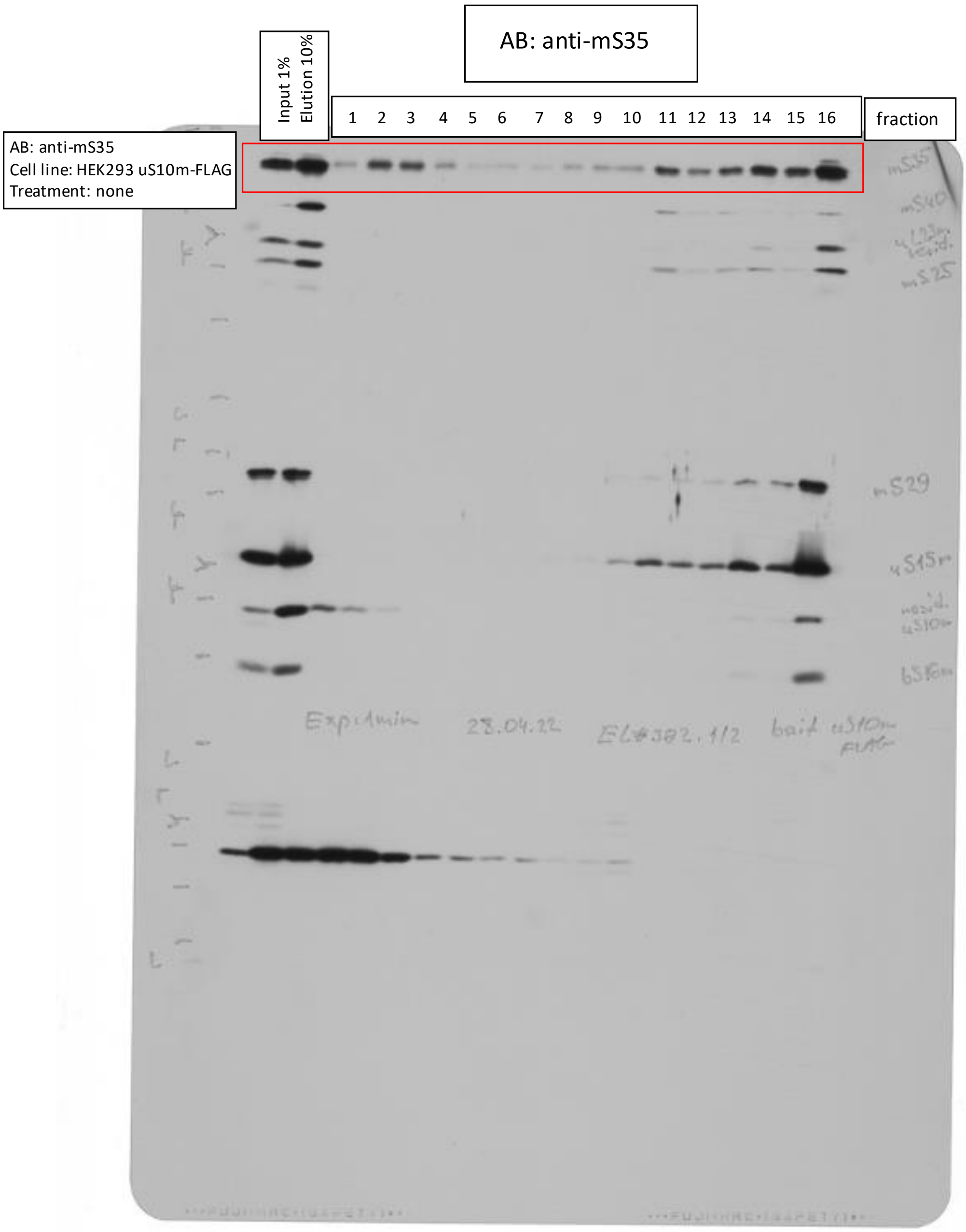

Source Data 2_related to Extended Data Fig.3f
EL#392.1-2 uS10m-FLAG IP + Gradient
Rotor: SW41 Ti
Gradient: Sucrose 5-30%
Speed: 158.000xg
Time: 15h

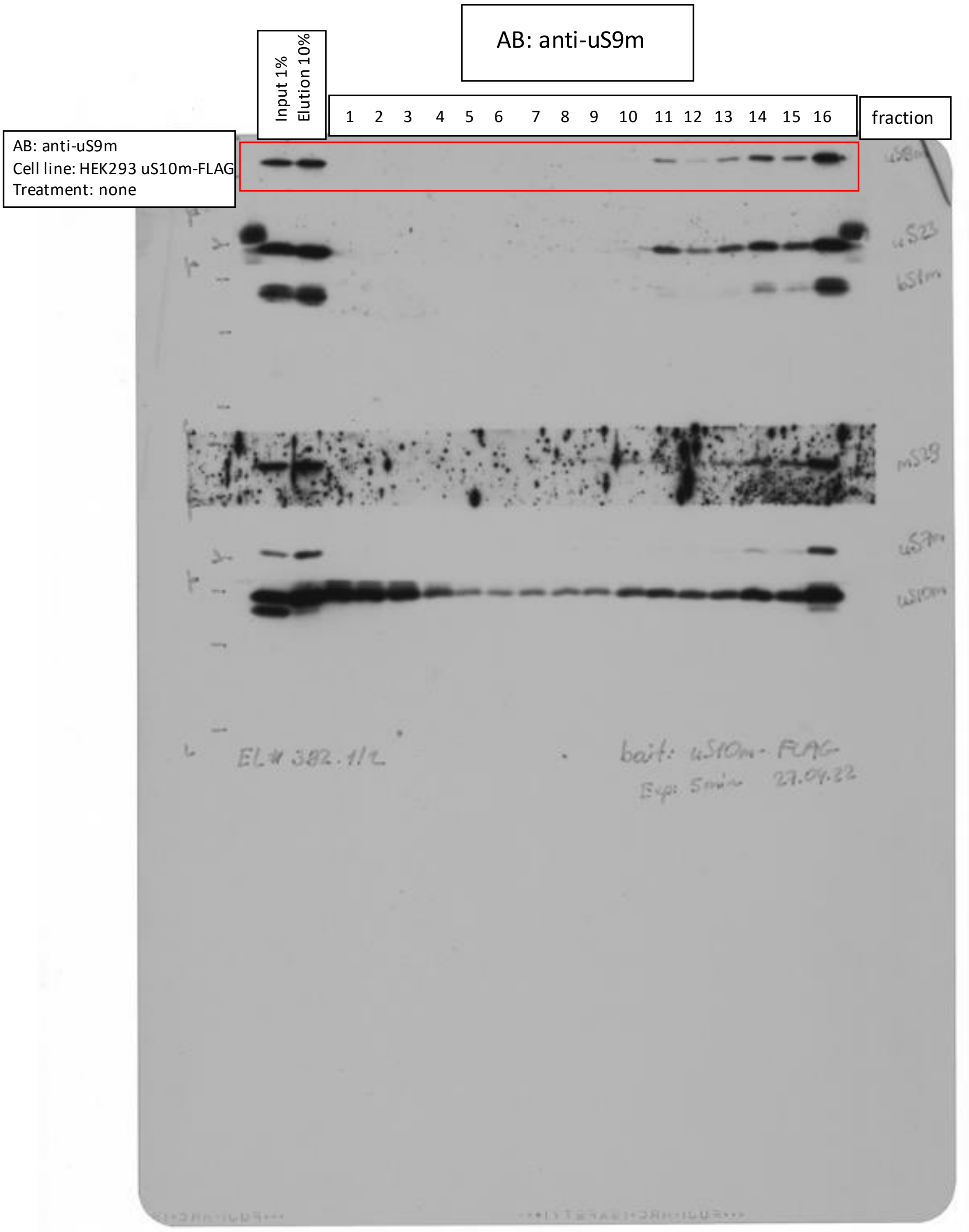

Source Data 2_related to Extended Data Fig.3f
EL#392.1-2 uS10m-FLAG IP + Gradient
Rotor: SW41 Ti
Gradient: Sucrose 5-30%
Speed: 158.000xg
Time: 15h

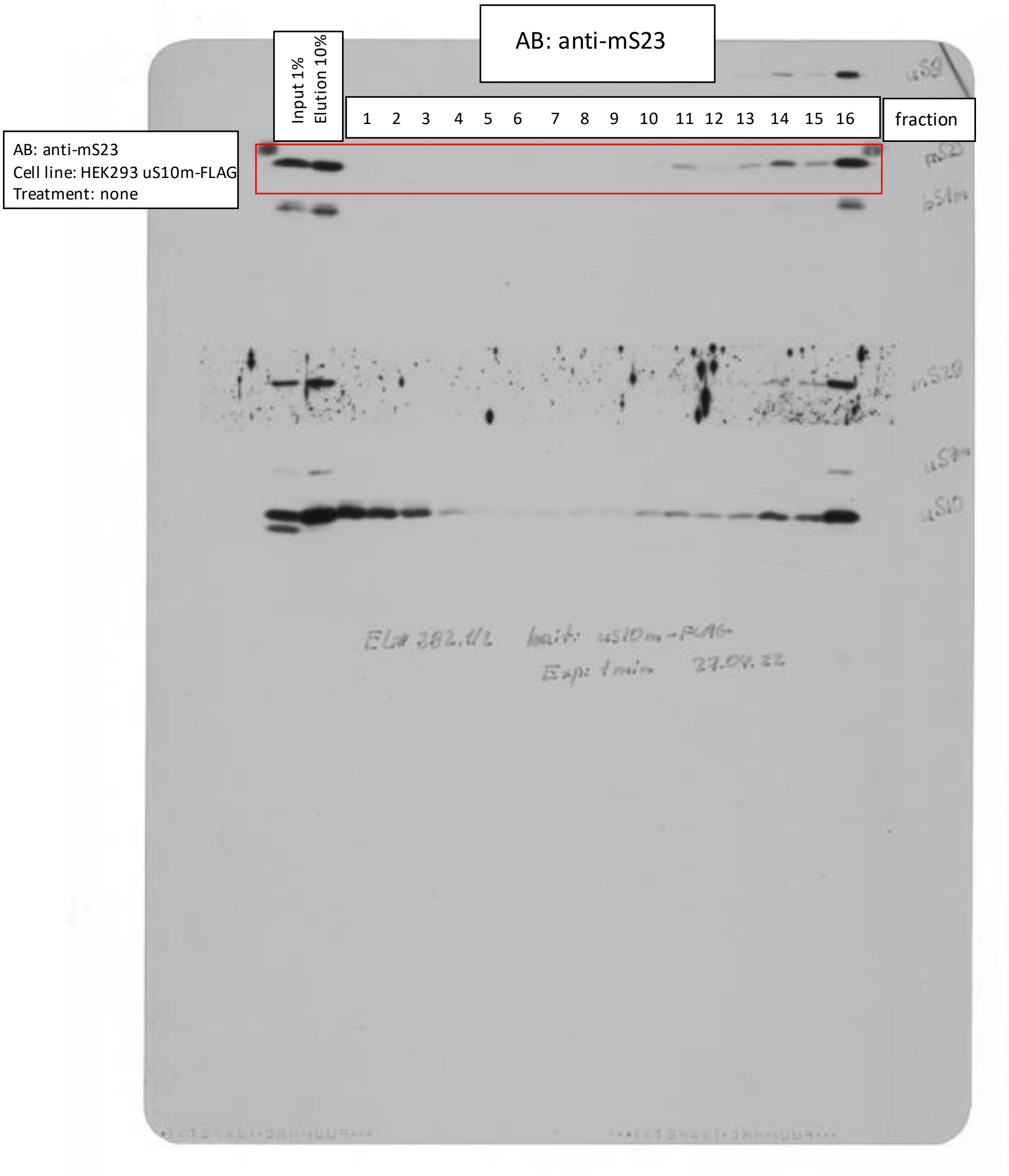

Source Data 2_related to Extended Data Fig.3f
EL#392.1-2 uS10m-FLAG IP + Gradient
Rotor: SW41 Ti
Gradient: Sucrose 5-30%
Speed: 158.000xg
Time: 15h

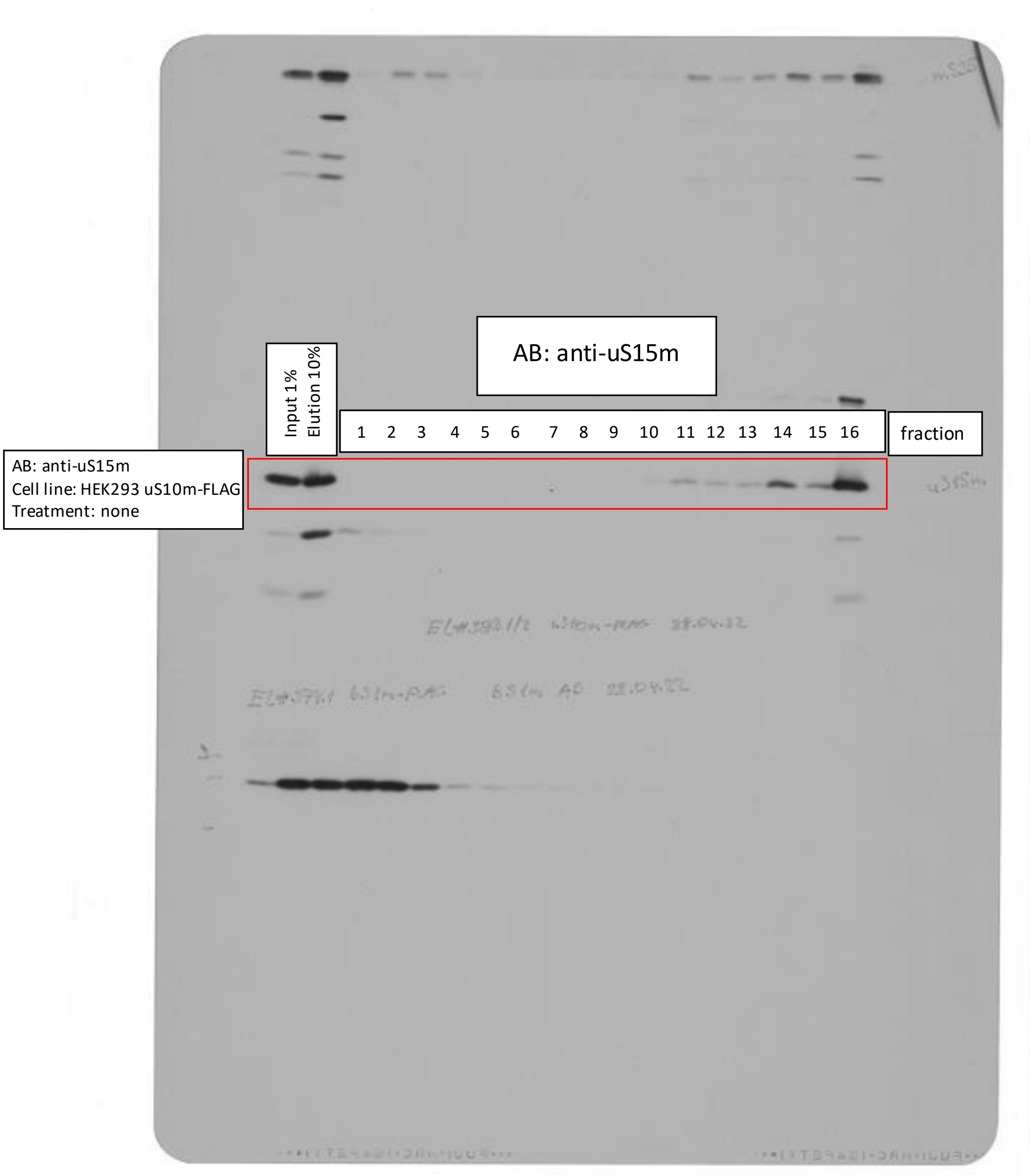

Source Data 2_related to Extended Data Fig.3f
EL#392.1-2 uS10m-FLAG IP + Gradient
Rotor: SW41 Ti
Gradient: Sucrose 5-30%
Speed: 158.000xg
Time: 15h

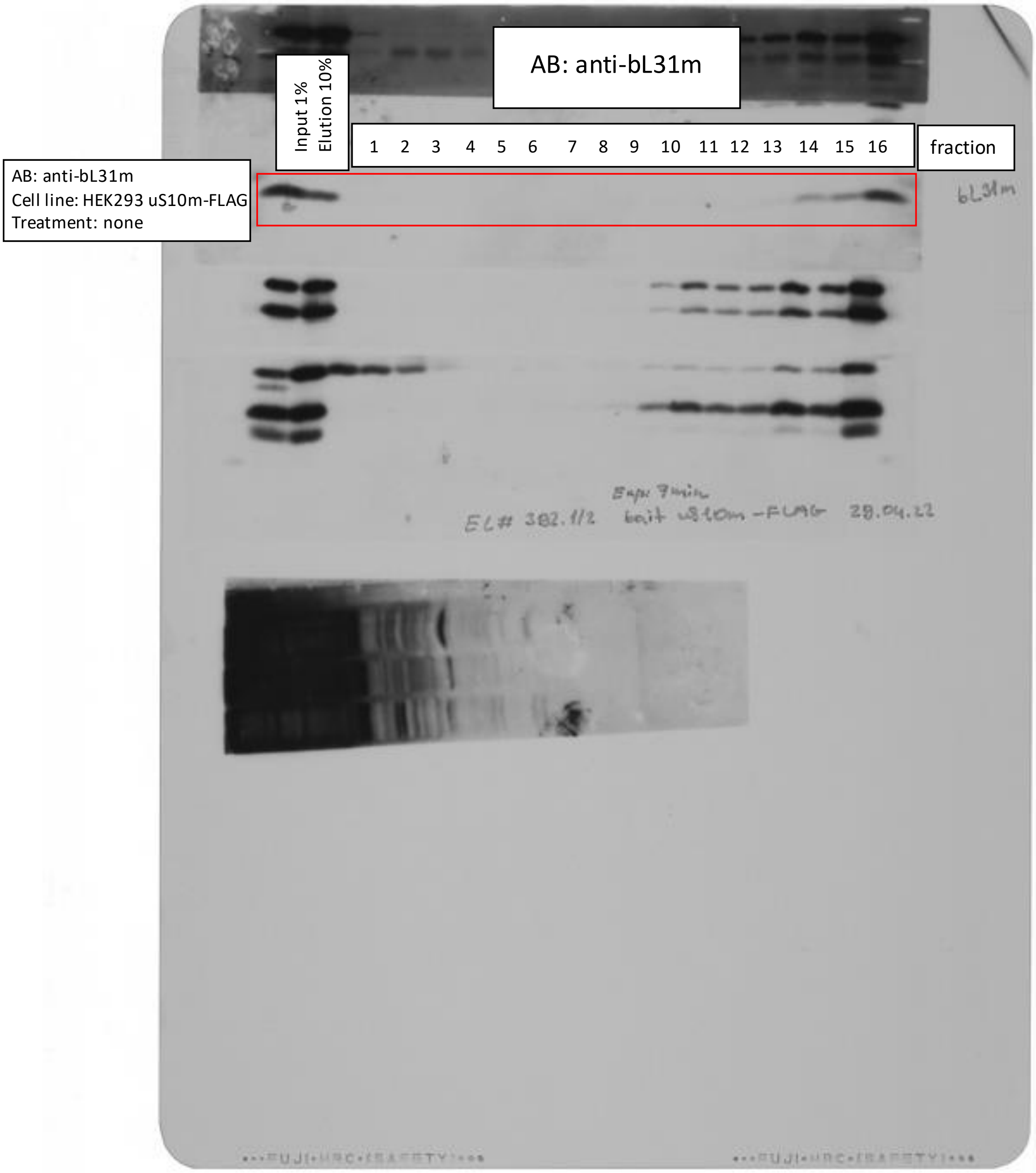

Source Data 2_related to Extended Data Fig.3f
EL#392.1-2 uS10m-FLAG IP + Gradient
Rotor: SW41 Ti
Gradient: Sucrose 5-30%
Speed: 158.000xg
Time: 15h

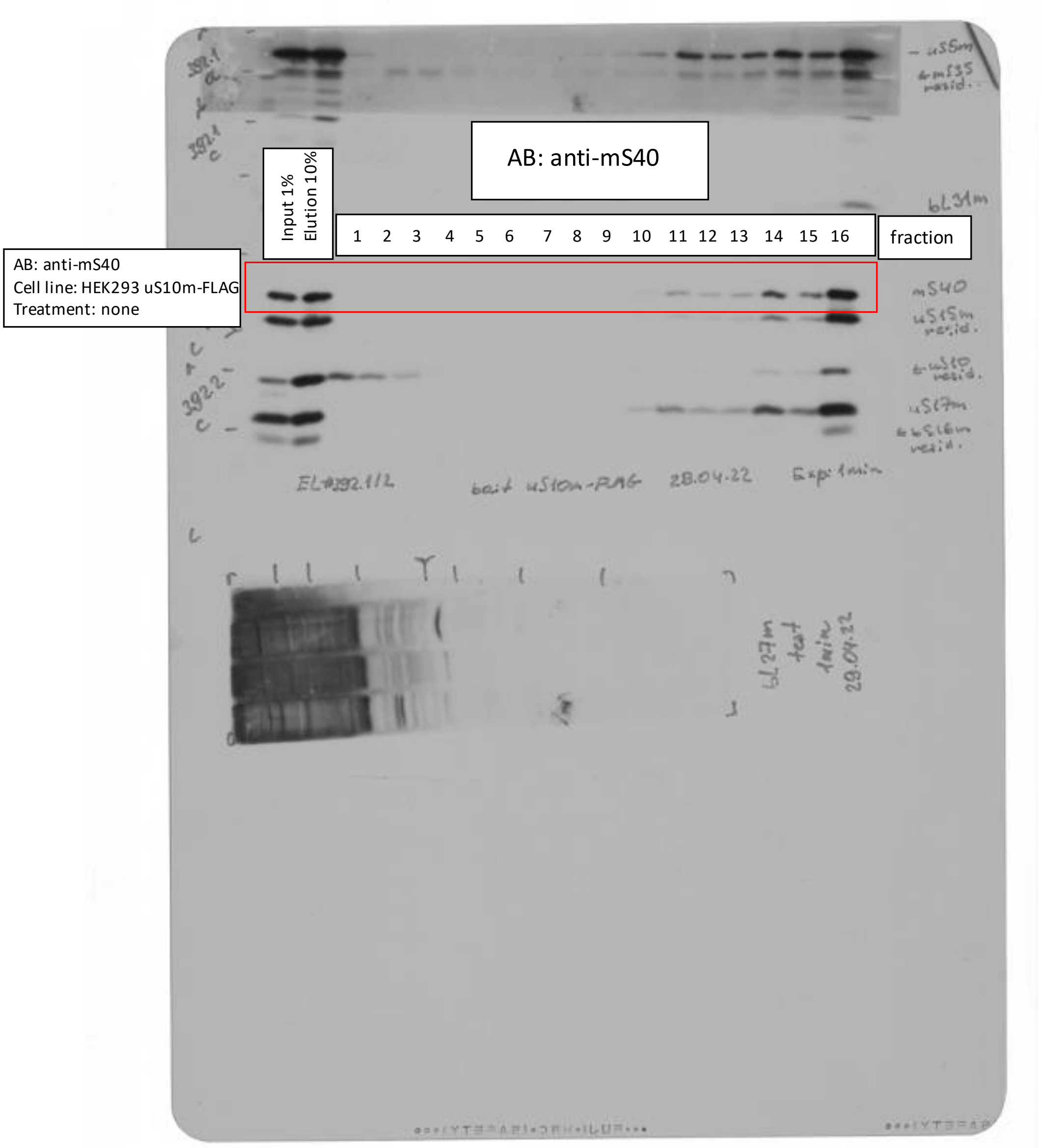

Source Data 2_related to Extended Data Fig.3f
EL#392.1-2 uS10m-FLAG IP + Gradient
Rotor: SW41 Ti
Gradient: Sucrose 5-30%
Speed: 158.000xg
Time: 15h

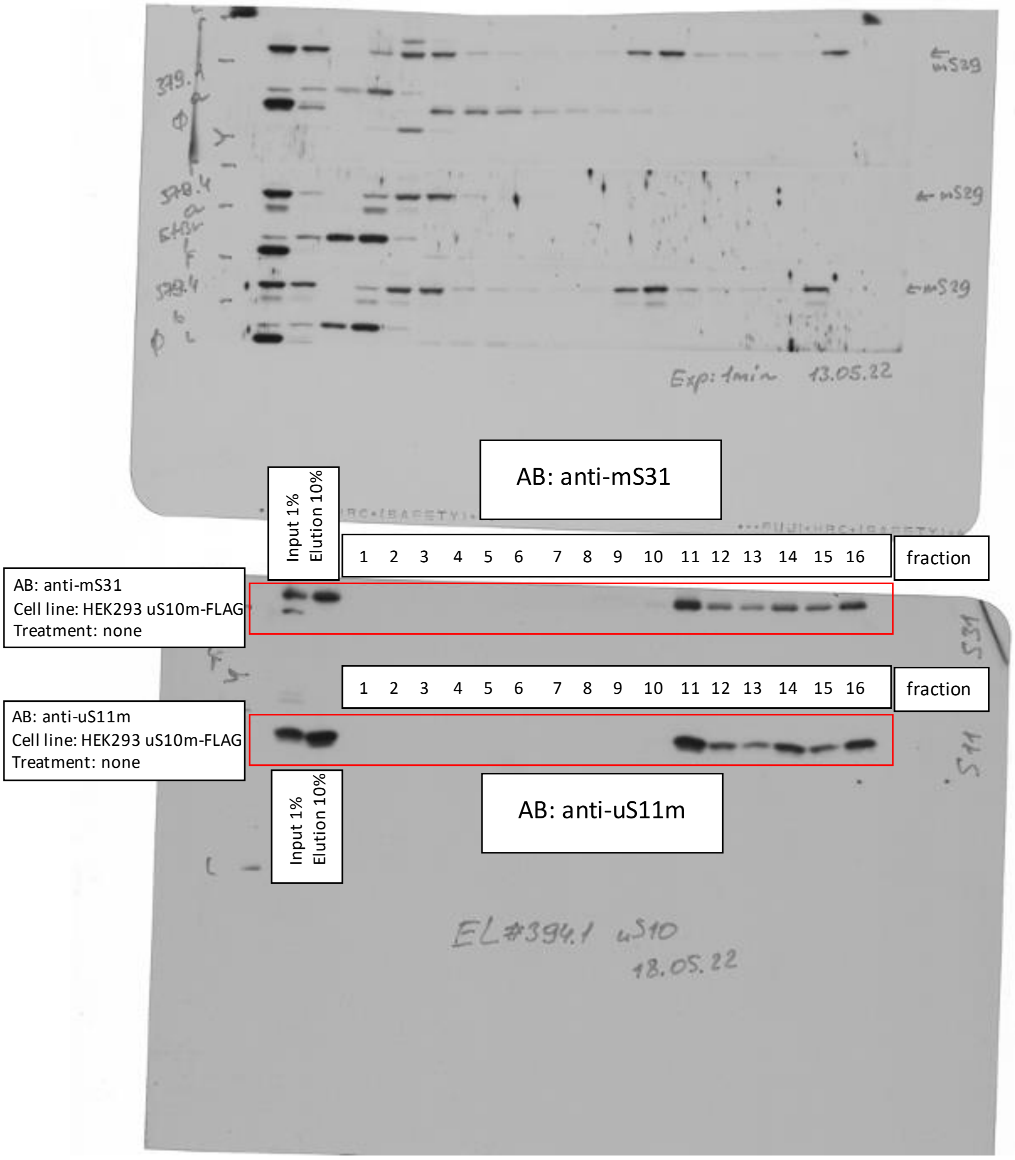

Source Data 2_related to Extended Data Fig.3f
EL#392.1-2 uS10m-FLAG IP + Gradient
Rotor: SW41 Ti
Gradient: Sucrose 5-30%
Speed: 158.000xg
Time: 15h

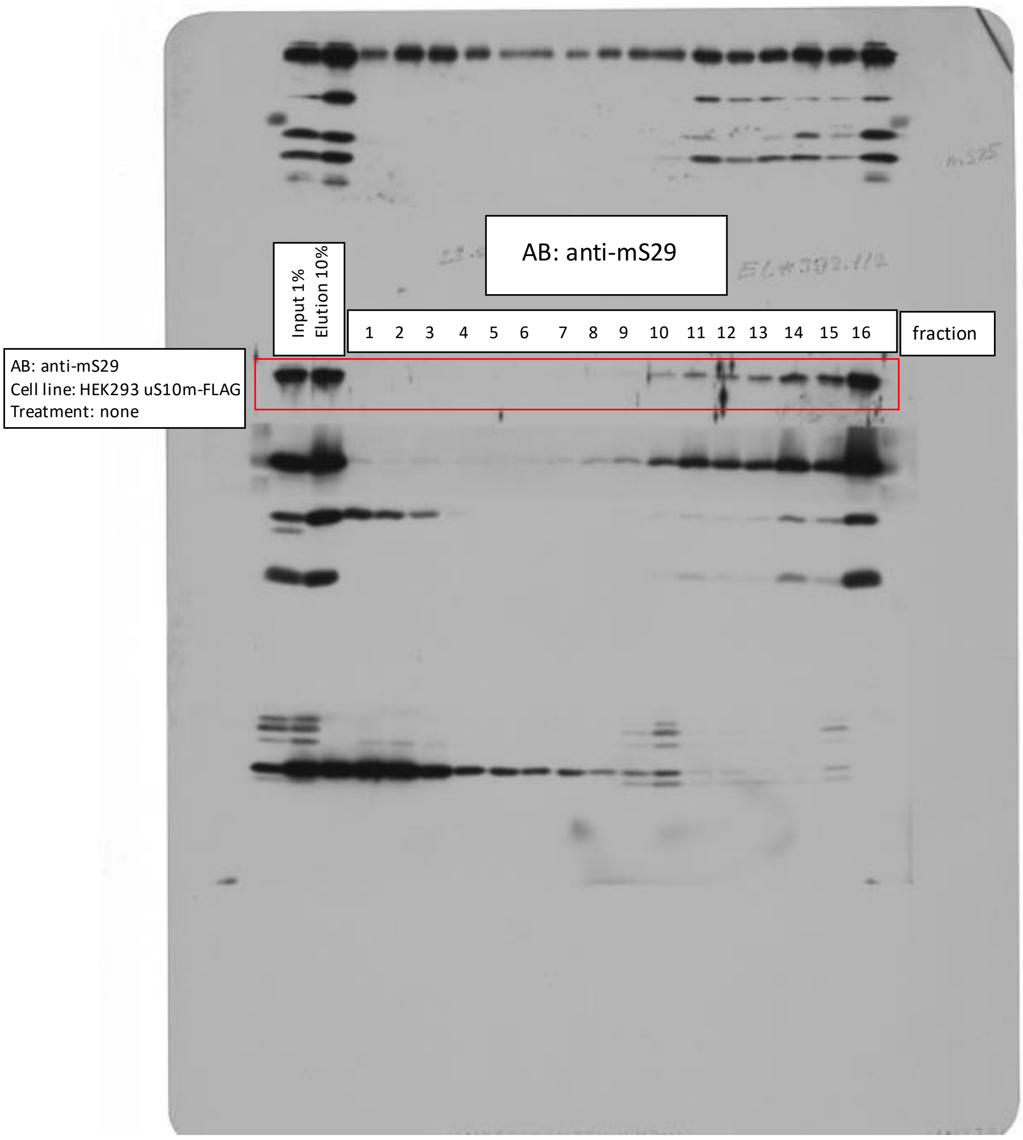

Source Data 2_related to Extended Data Fig.3f
EL#392.1-2 uS10m-FLAG IP + Gradient
Rotor: SW41 Ti
Gradient: Sucrose 5-30%
Speed: 158.000xg
Time: 15h

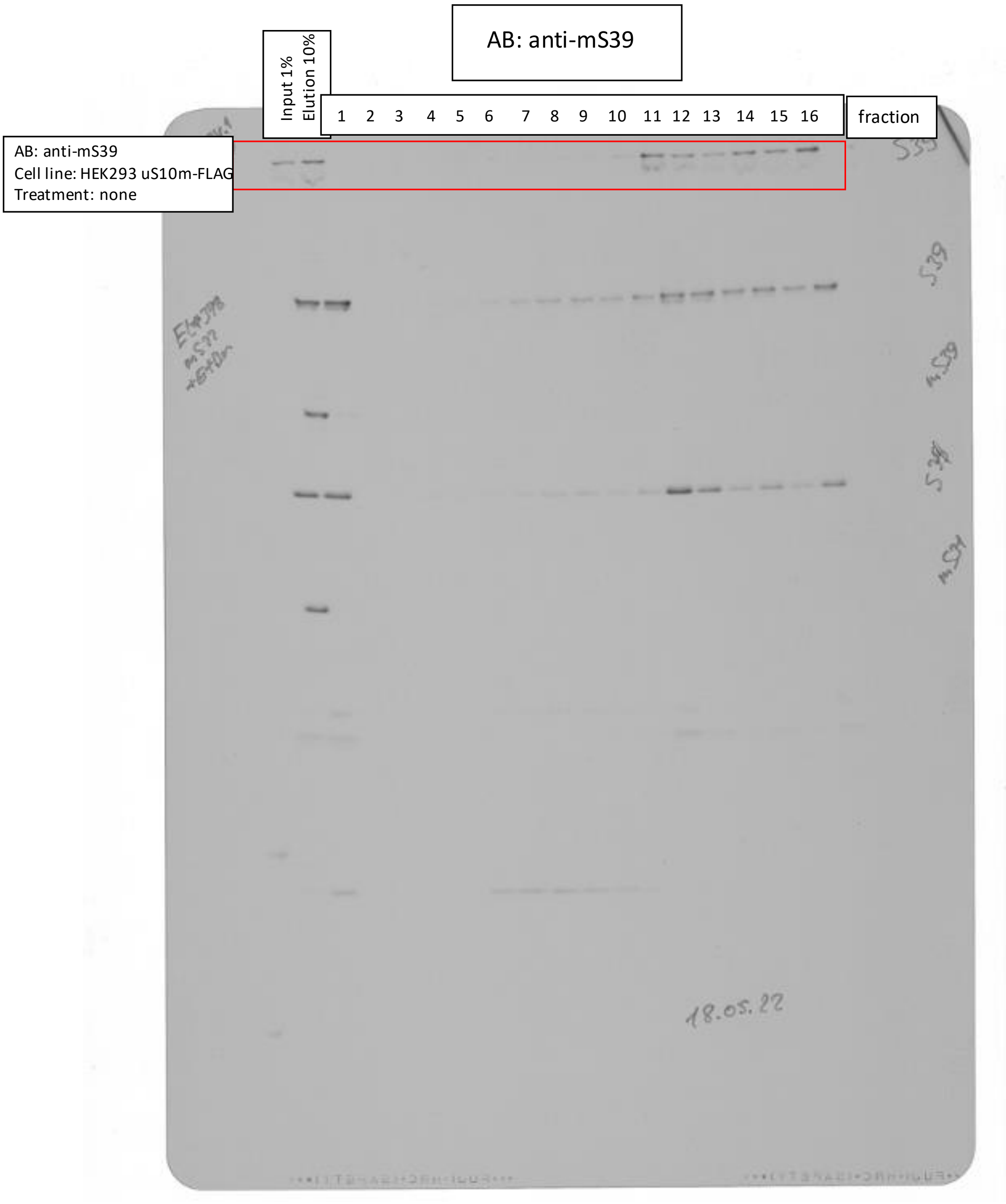

Source Data 2_related to Extended Data Fig.3g
AS#112 HEK293 WT vs. HEK293 uS7m KO
Rotor: SW41 Ti
Gradient: Sucrose 5-30%
Speed: 158.000xg
Time: 15h

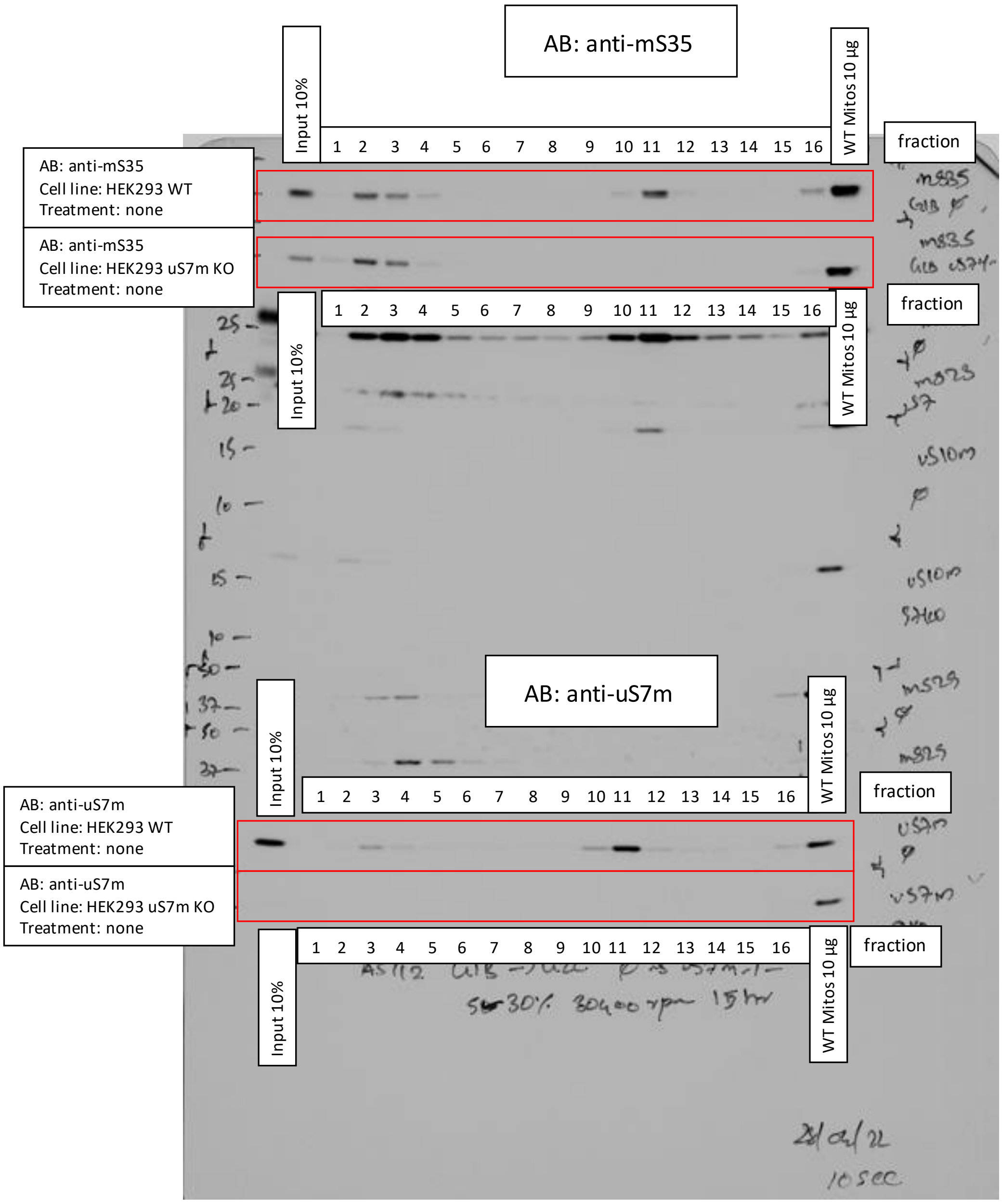

Source Data 2_related to Extended Data Fig.3g
AS#112 HEK293 WT vs. HEK293 uS7m KO
Rotor: SW41 Ti
Gradient: Sucrose 5-30%
Speed: 158.000xg
Time: 15h

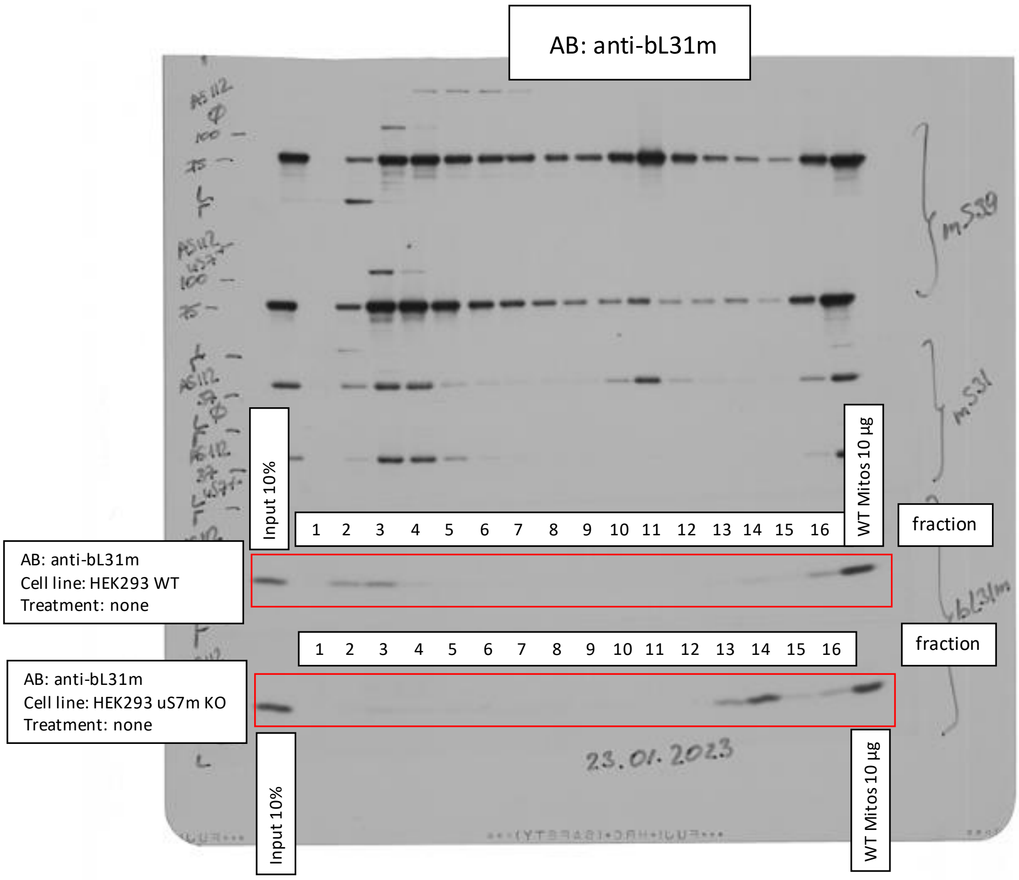

Source Data 2_related to Extended Data Fig.3g
AS#112 HEK293 WT vs. HEK293 uS7m KO
Rotor: SW41 Ti
Gradient: Sucrose 5-30%
Speed: 158.000xg
Time: 15h

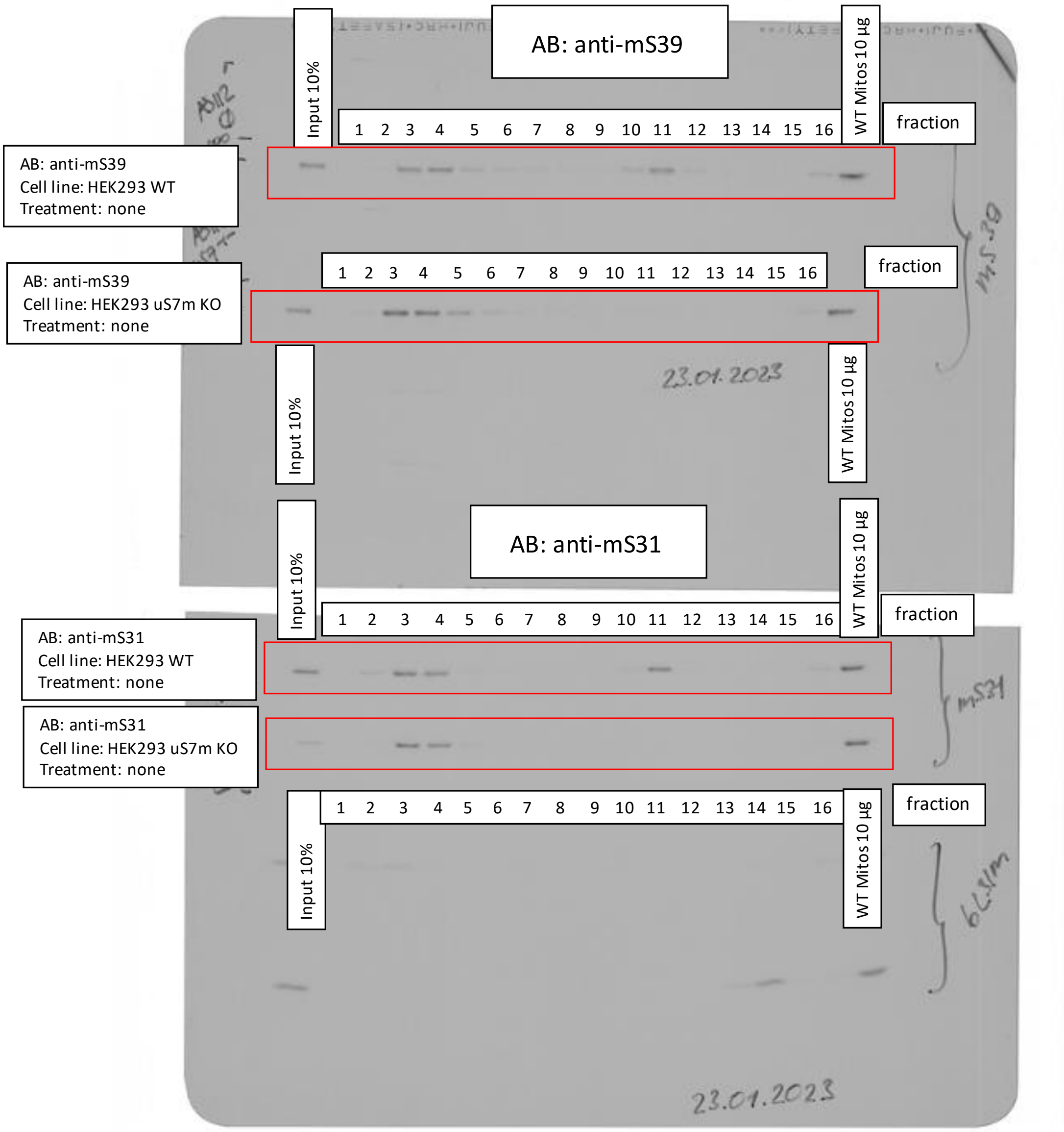

Source Data 2_related to Extended Data Fig.3g
AS#112 HEK293 WT vs. HEK293 uS7m KO
Rotor: SW41 Ti
Gradient: Sucrose 5-30%
Speed: 158.000xg
Time: 15h

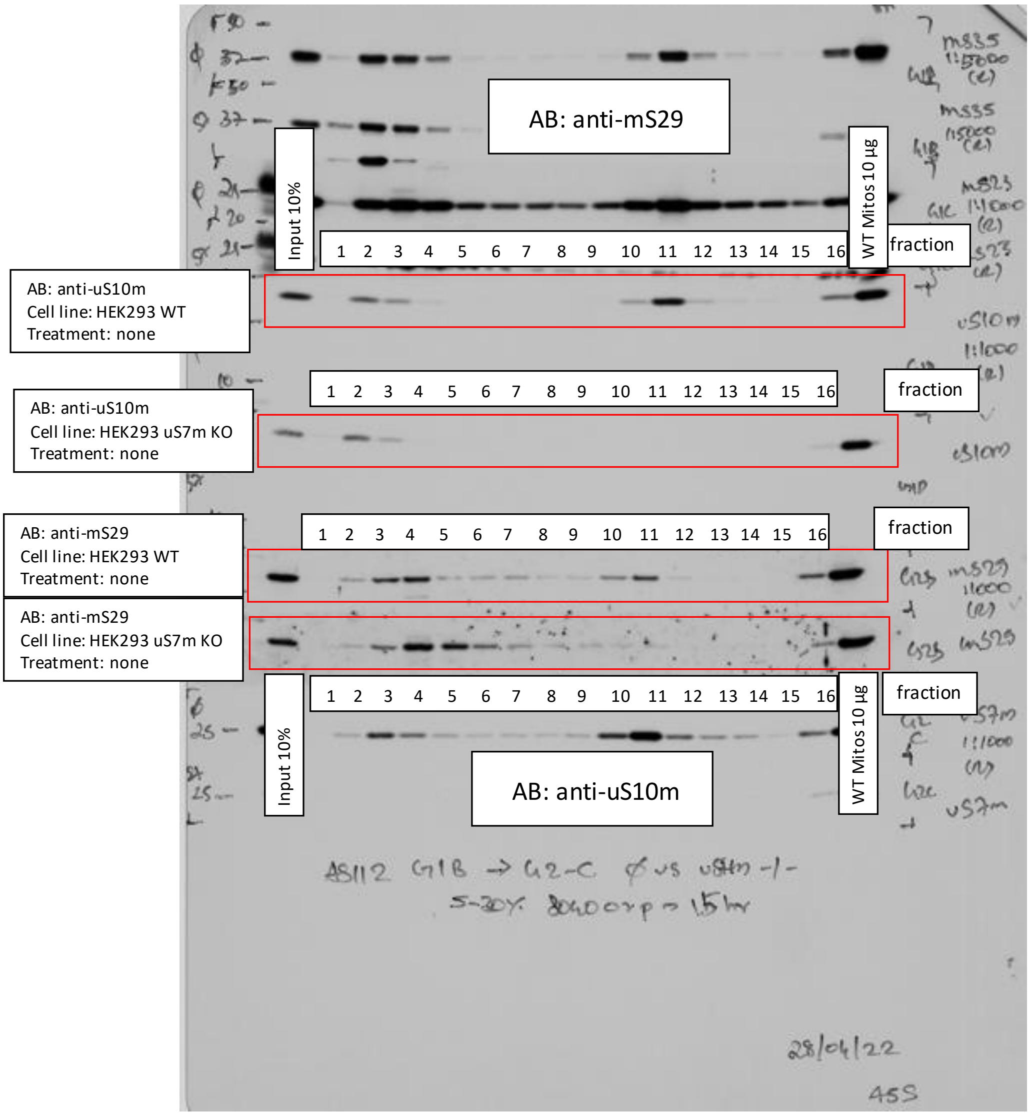

Source Data 2_related to Extended Data Fig.3g
AS#112 HEK293 WT vs. HEK293 uS7m KO
Rotor: SW41 Ti
Gradient: Sucrose 5-30%
Speed: 158.000xg
Time: 15h

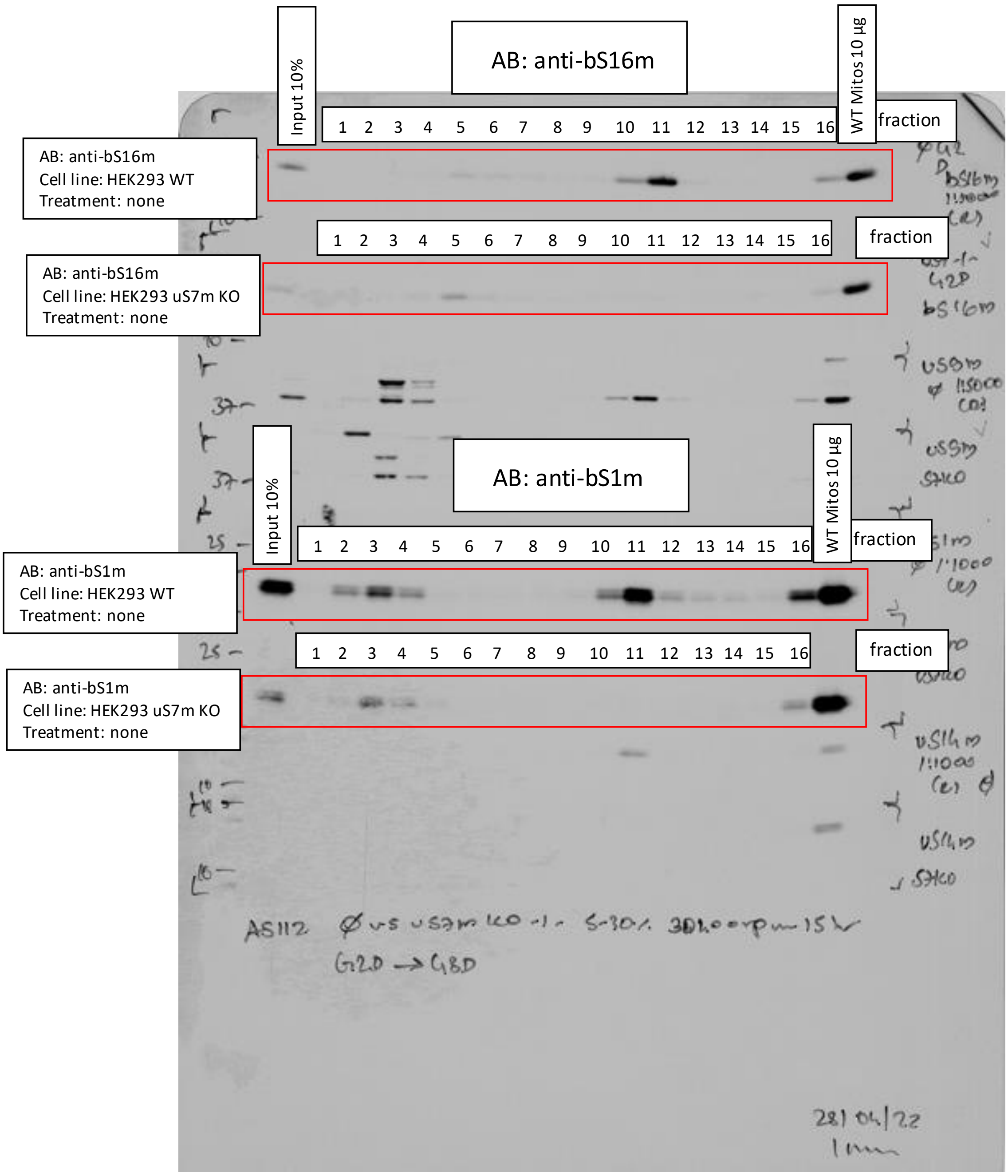

Source Data 2_related to Extended Data Fig.3g
AS#112 HEK293 WT vs. HEK293 uS7m KO
Rotor: SW41 Ti
Gradient: Sucrose 5-30%
Speed: 158.000xg
Time: 15h

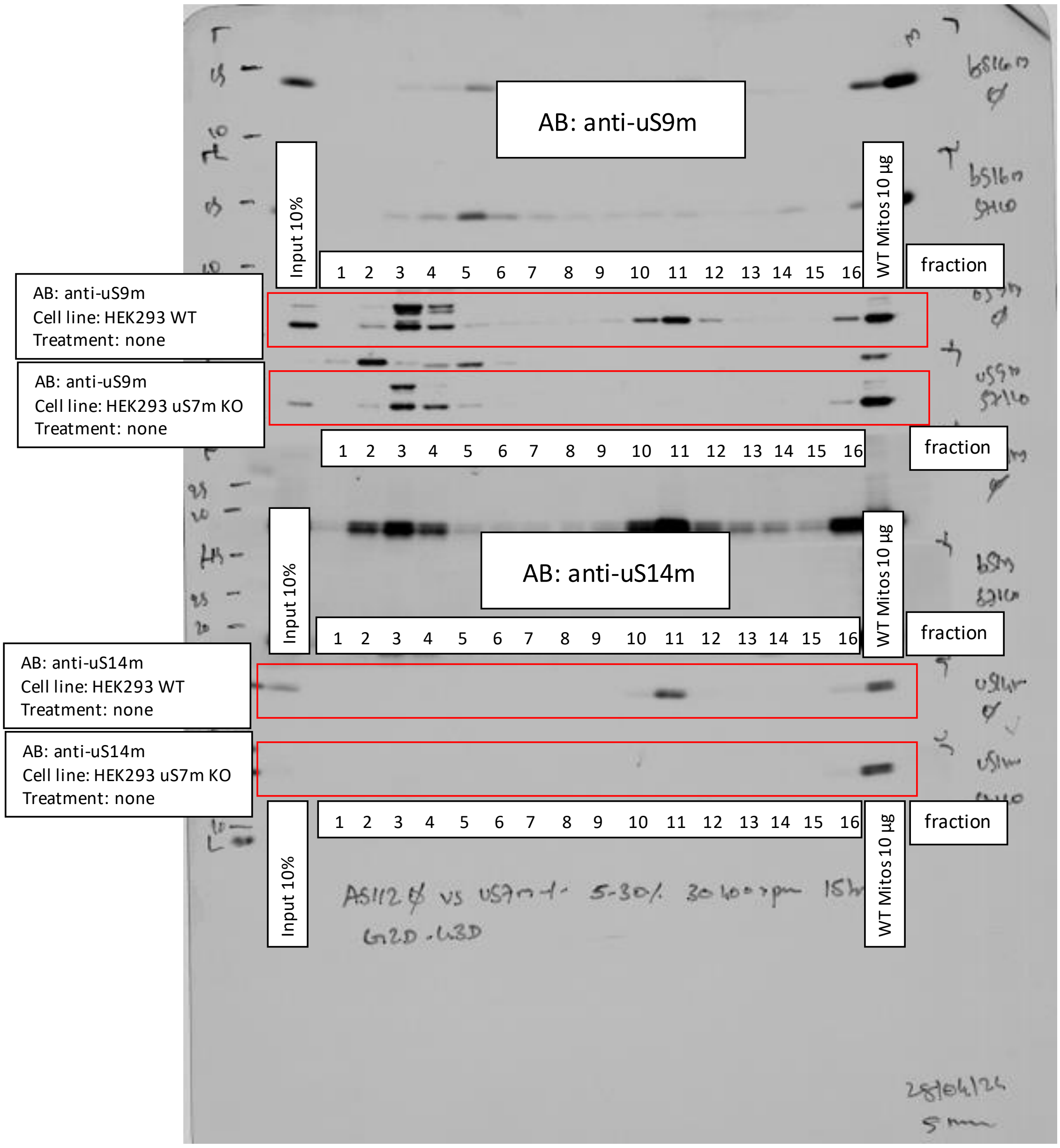

Supplement: Supplementary file 20 — Unprocessed blots. [file 41594_2024_1356_MOESM20_ESM.pdf]
