# Supplementary material for: A roadmap for ribosome assembly in human mitochondria
Source: Nat Struct Mol Biol. 2024 Jul 11;31(12):1898–908. doi: 10.1038/s41594-024-01356-w (PMC11638073; doi:10.1038/s41594-024-01356-w)

Source Data 3_related to Extended Data Fig.7a
EL#380 mL62-FLAG IP + Gradient + EtBr treatment
Rotor: SW41 Ti
Gradient: Sucrose 5-30%
Speed: 158.000xg
Time: 15h

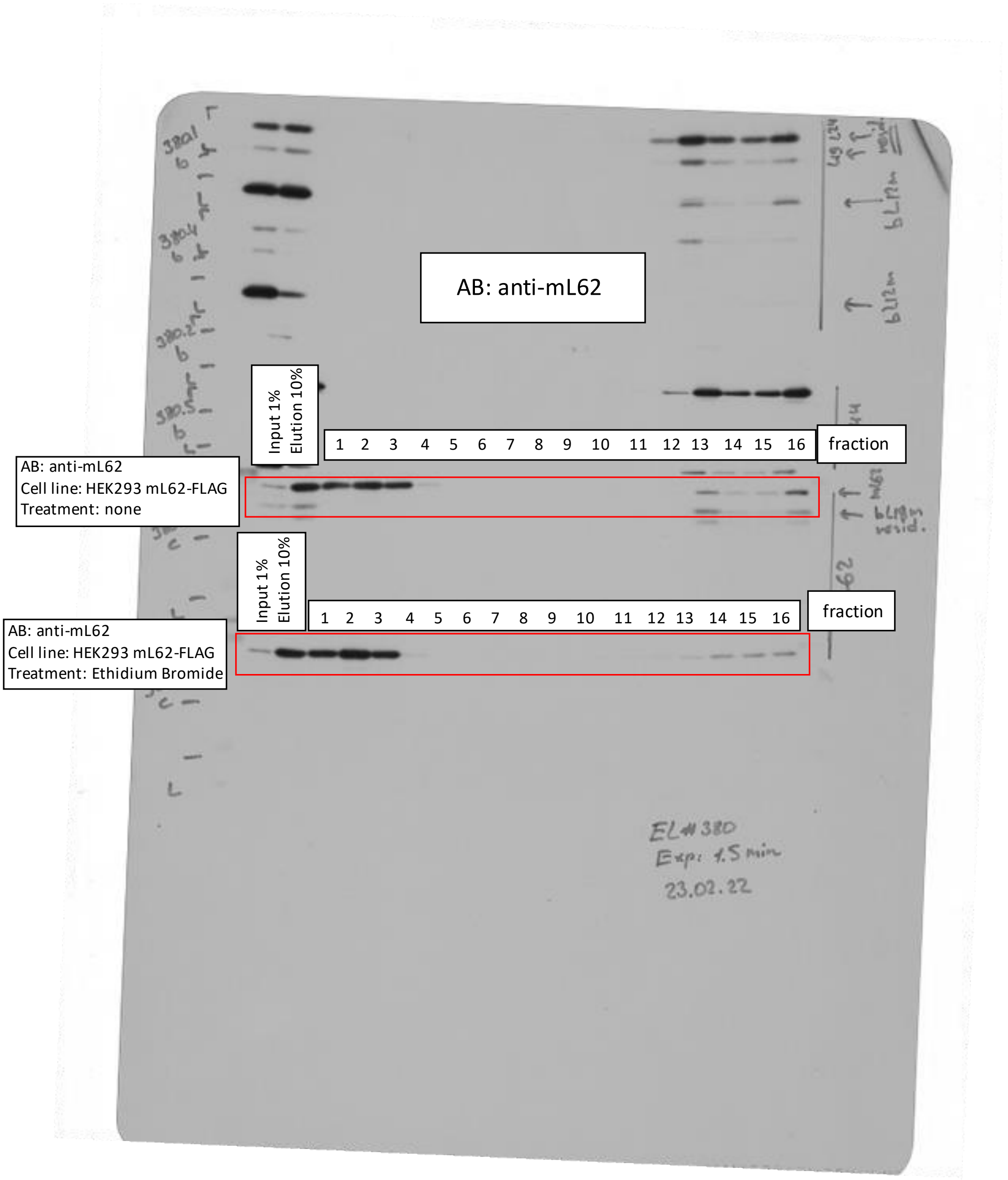

Source Data 3_related to Extended Data Fig.7a
EL#380 mL62-FLAG IP + Gradient + EtBr treatment
Rotor: SW41 Ti
Gradient: Sucrose 5-30%
Speed: 158.000xg
Time: 15h

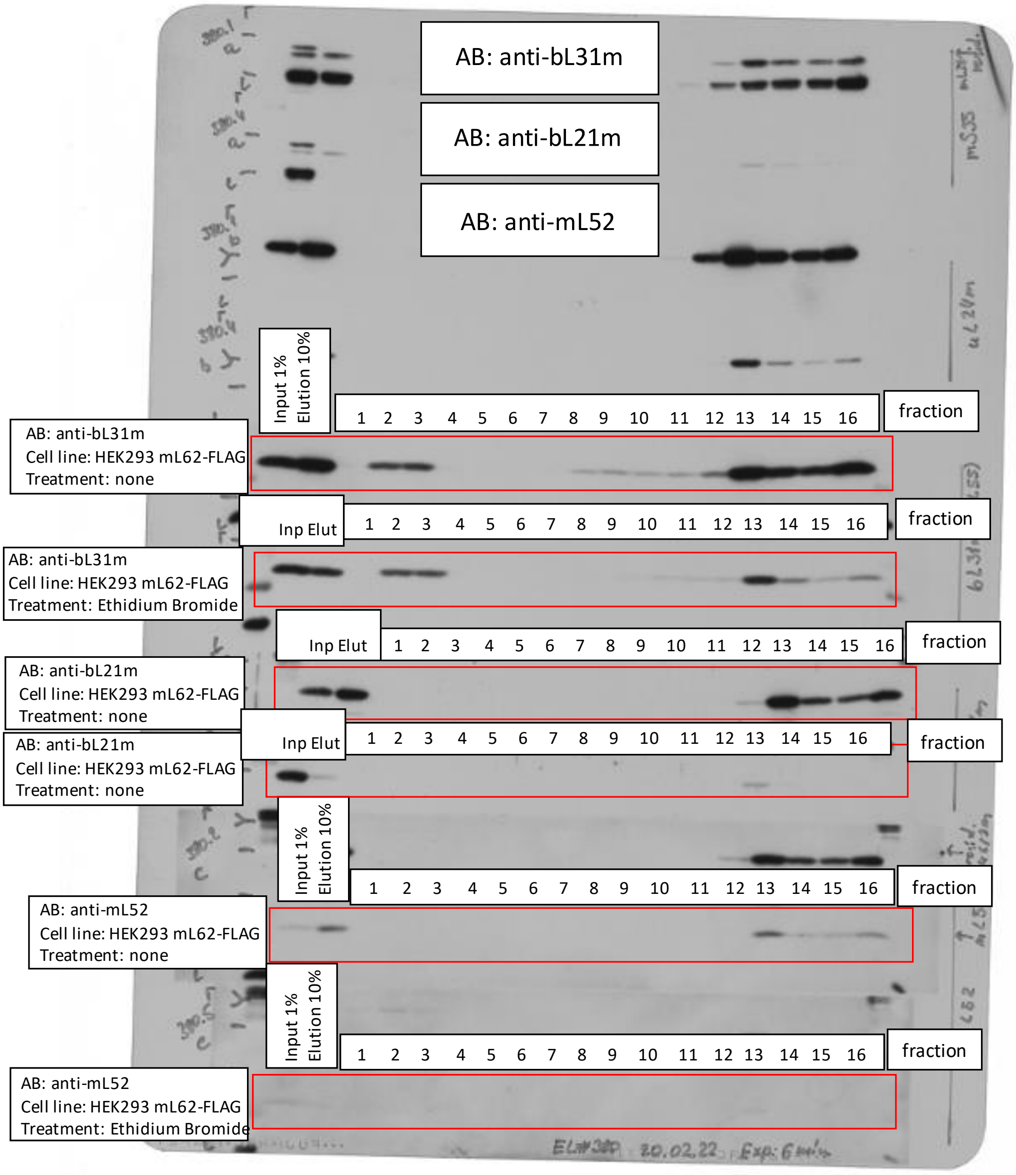

Source Data 3_related to Extended Data Fig.7a
EL#380 mL62-FLAG IP + Gradient + EtBr treatment
Rotor: SW41 Ti
Gradient: Sucrose 5-30%
Speed: 158.000xg
Time: 15h

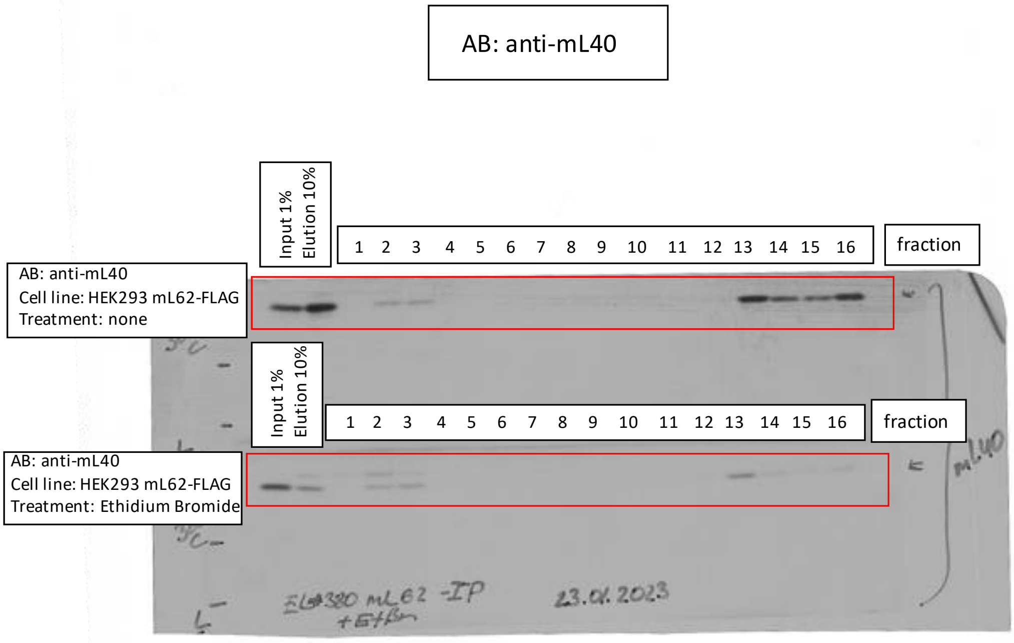

Source Data 3_related to Extended Data Fig.7a
EL#380 mL62-FLAG IP + Gradient + EtBr treatment
Rotor: SW41 Ti
Gradient: Sucrose 5-30%
Speed: 158.000xg
Time: 15h

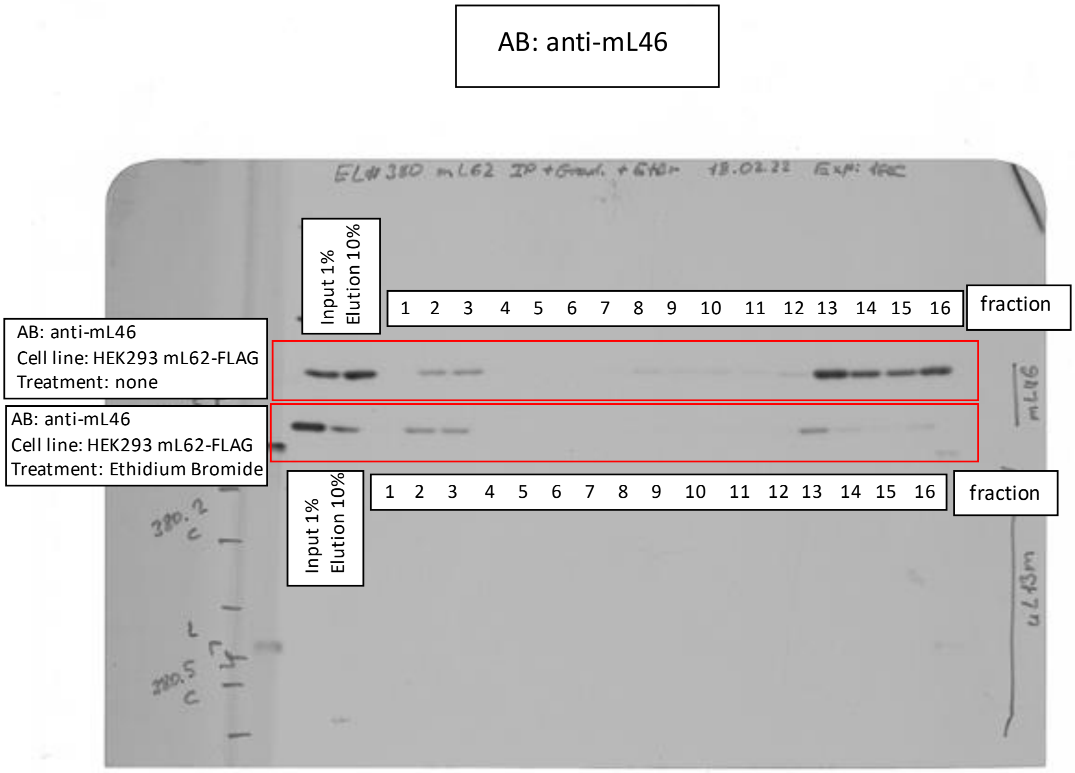

Source Data 3_related to Extended Data Fig.7a
EL#380 mL62-FLAG IP + Gradient + EtBr treatment
Rotor: SW41 Ti
Gradient: Sucrose 5-30%
Speed: 158.000xg
Time: 15h

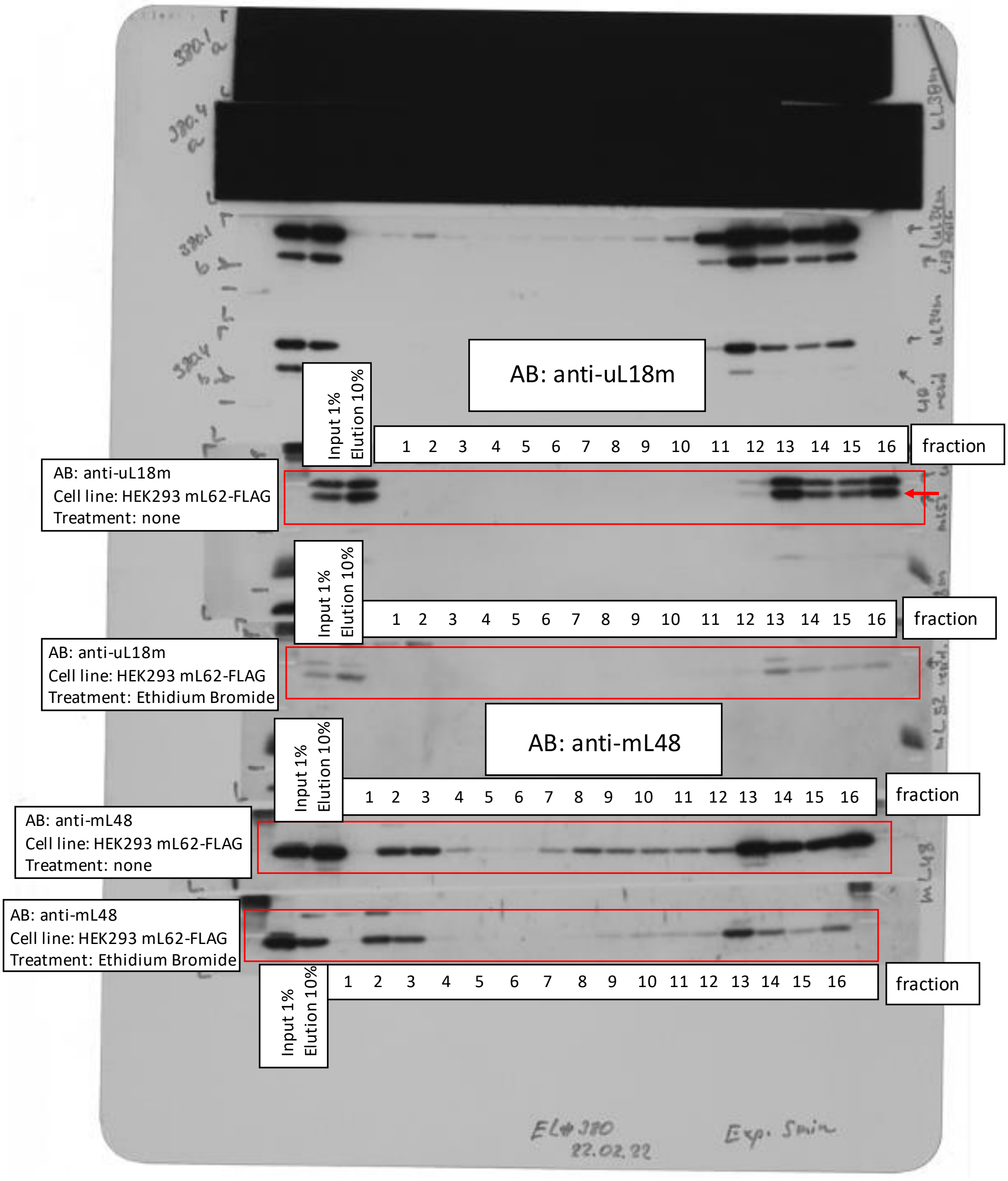

Source Data 3_related to Extended Data Fig.7a
EL#380 mL62-FLAG IP + Gradient + EtBr treatment
Rotor: SW41 Ti
Gradient: Sucrose 5-30%
Speed: 158.000xg
Time: 15h

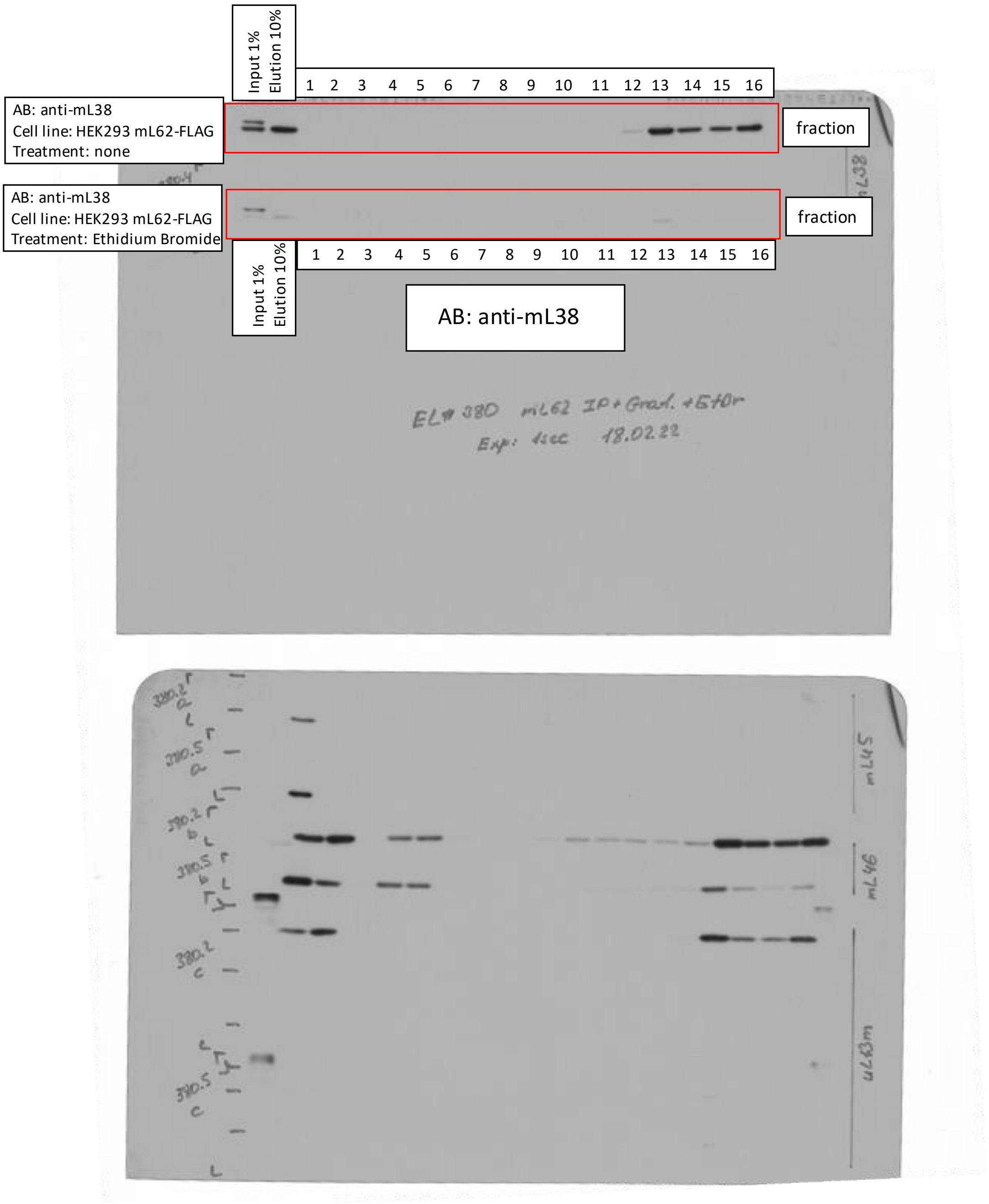

Source Data 3_related to Extended Data Fig.7a
EL#380 mL62-FLAG IP + Gradient + EtBr treatment
Rotor: SW41 Ti
Gradient: Sucrose 5-30%
Speed: 158.000xg
Time: 15h

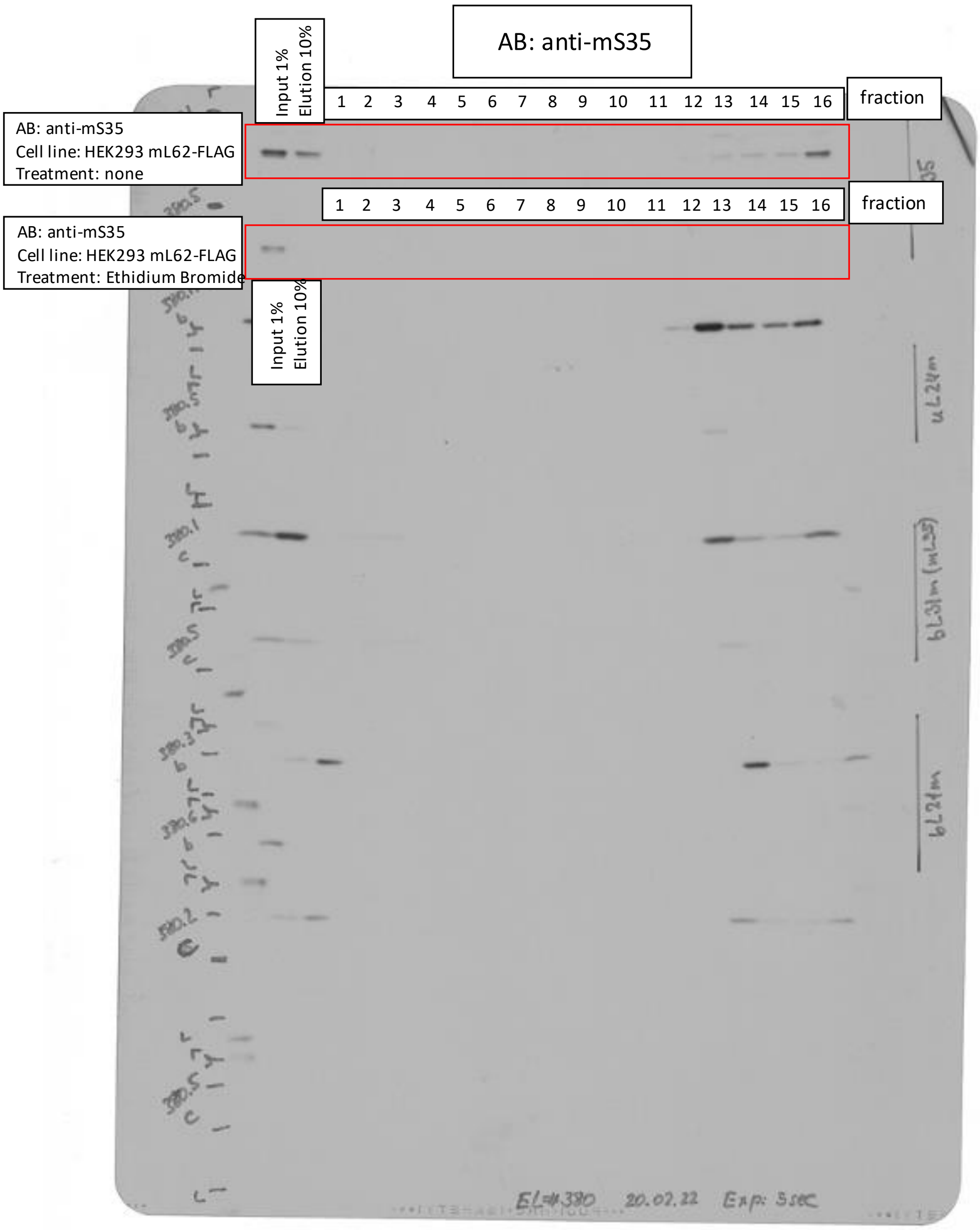

Source Data 3_related to Extended Data Fig.7b
EL#399.4-6 bL31m-FLAG IP + Gradient
Rotor: SW41 Ti
Gradient: Sucrose 5-30%
Speed: 158.000xg
Time: 15h

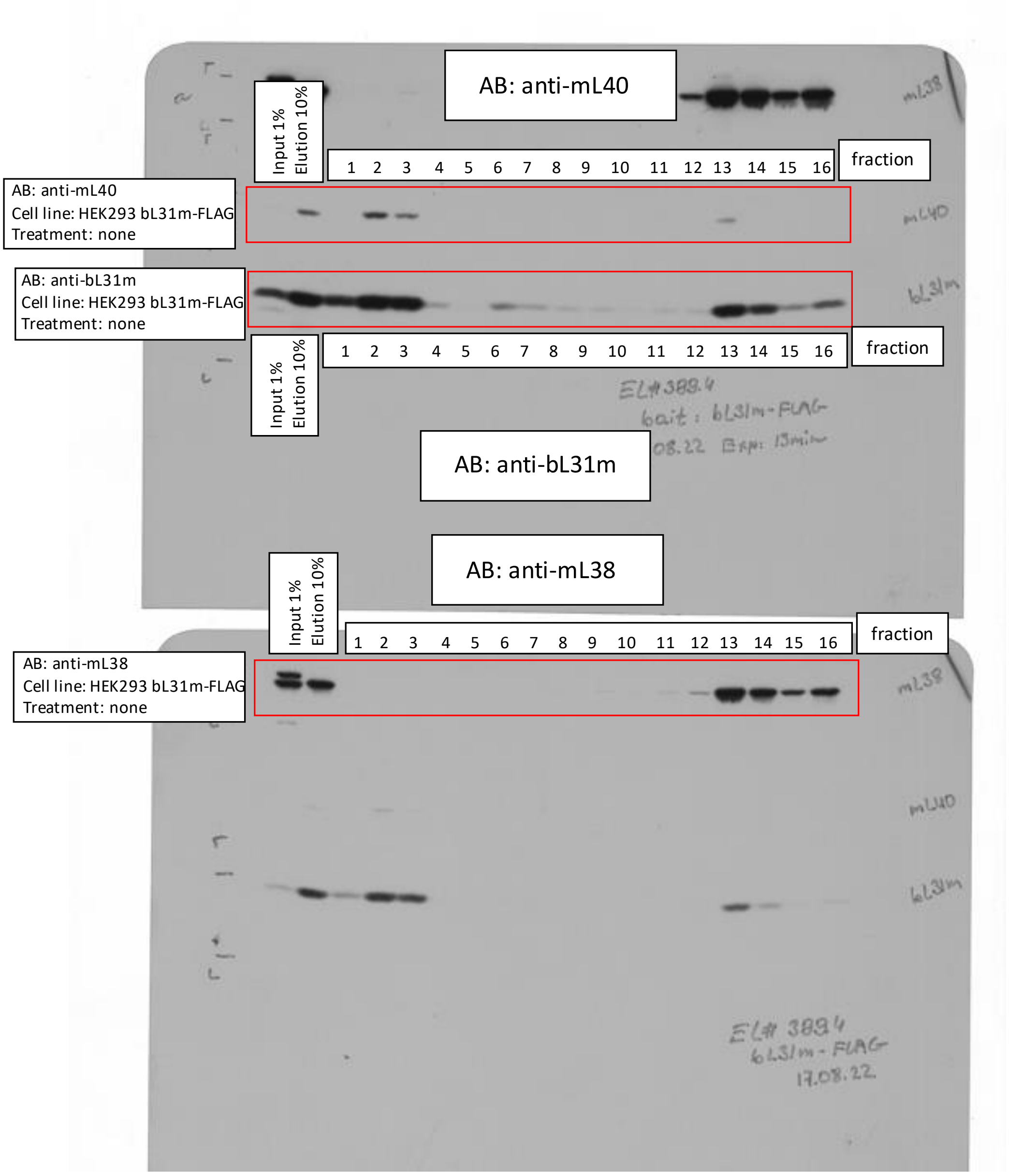

Source Data 3_related to Extended Data Fig.7b
EL#399.4-6 bL31m-FLAG IP + Gradient
Rotor: SW41 Ti
Gradient: Sucrose 5-30%
Speed: 158.000xg
Time: 15h

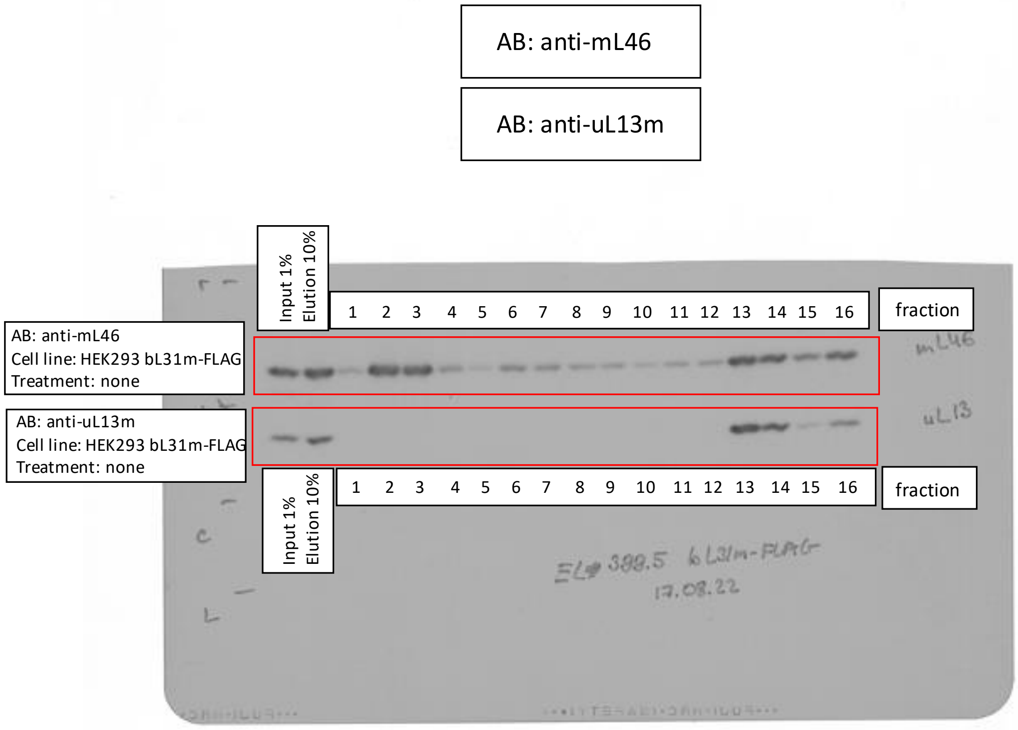

Source Data 3_related to Extended Data Fig.7b
EL#399.4-6 bL31m-FLAG IP + Gradient
Rotor: SW41 Ti
Gradient: Sucrose 5-30%
Speed: 158.000xg
Time: 15h

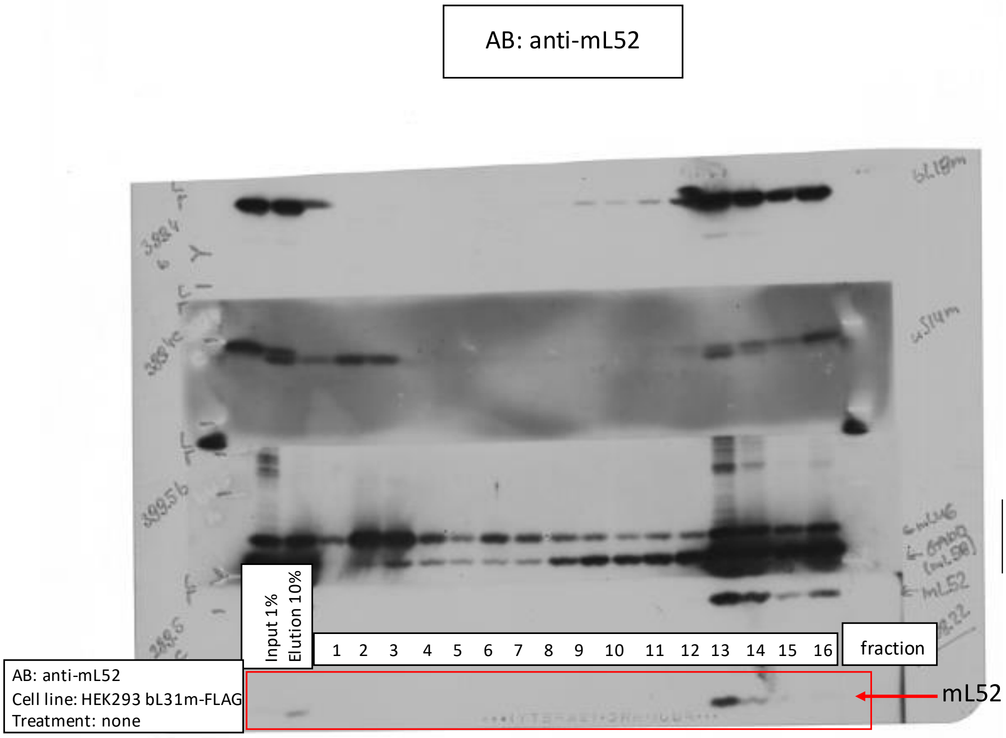

Source Data 3_related to Extended Data Fig.7b
EL#399.4-6 bL31m-FLAG IP + Gradient
Rotor: SW41 Ti
Gradient: Sucrose 5-30%
Speed: 158.000xg
Time: 15h

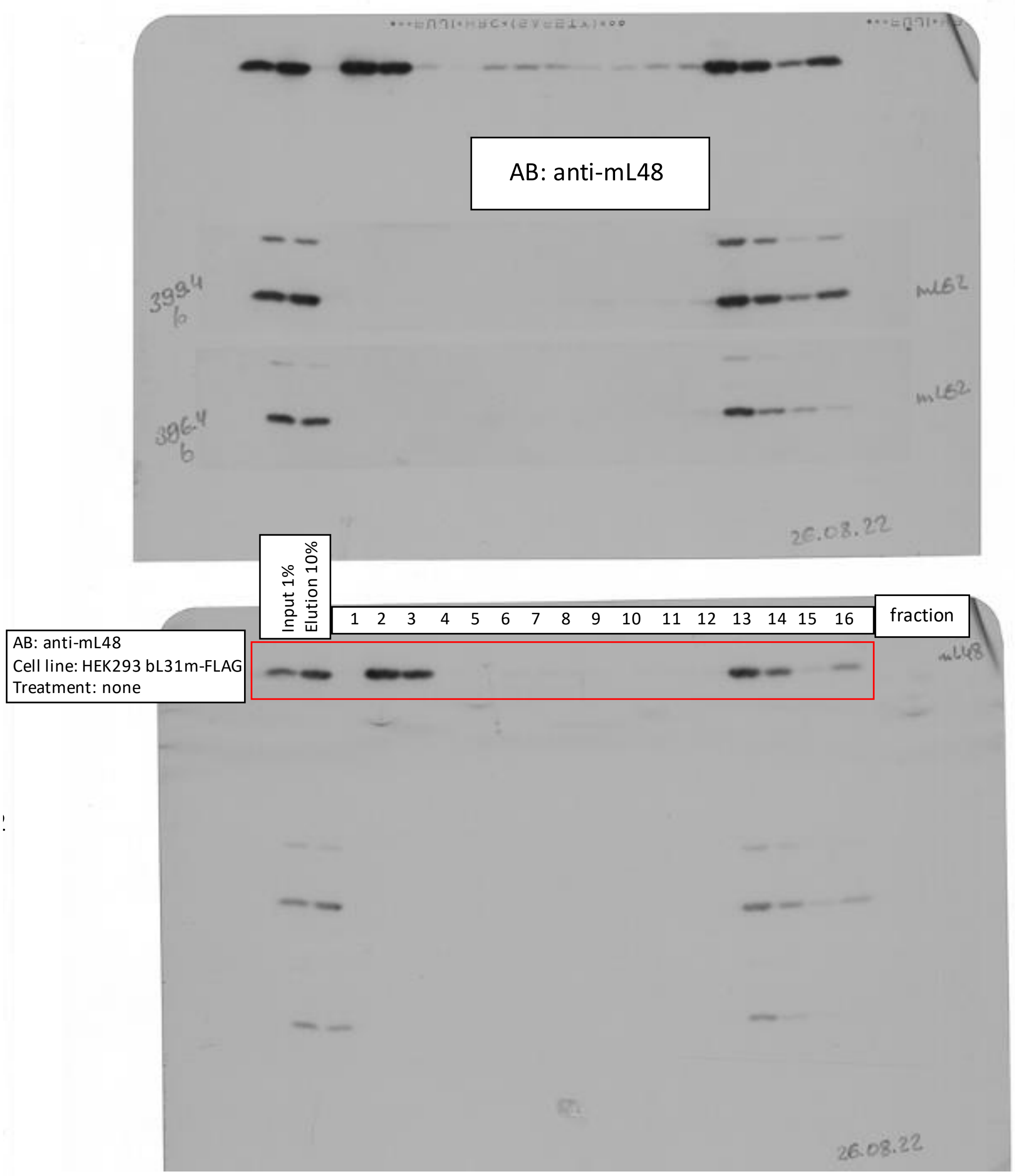

Source Data 3_related to Extended Data Fig.7b
EL#399.4-6 bL31m-FLAG IP + Gradient
Rotor: SW41 Ti
Gradient: Sucrose 5-30%
Speed: 158.000xg
Time: 15h

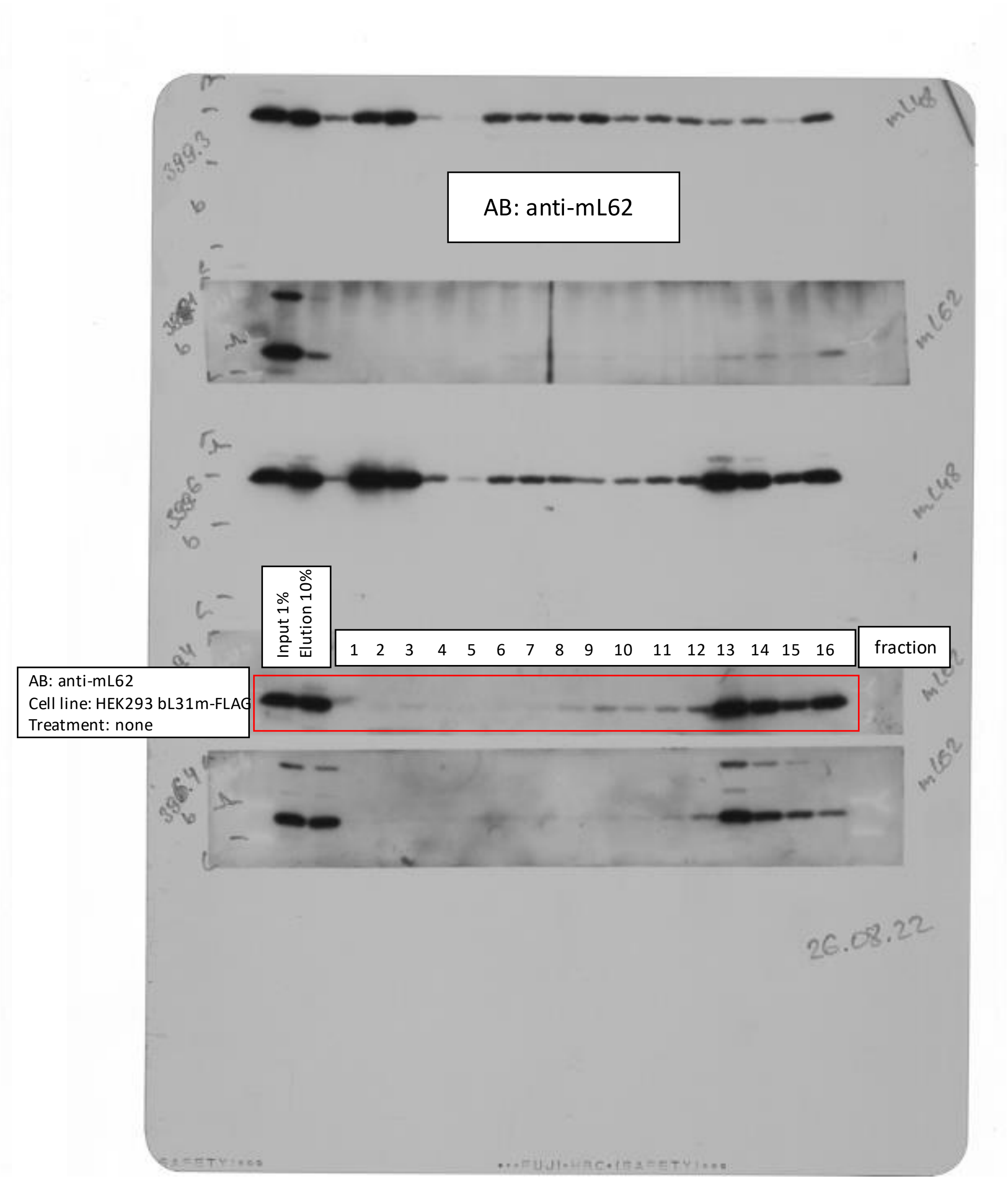

Source Data 3_related to Extended Data Fig.7b
EL#399.4-6 bL31m-FLAG IP + Gradient
Rotor: SW41 Ti
Gradient: Sucrose 5-30%
Speed: 158.000xg
Time: 15h

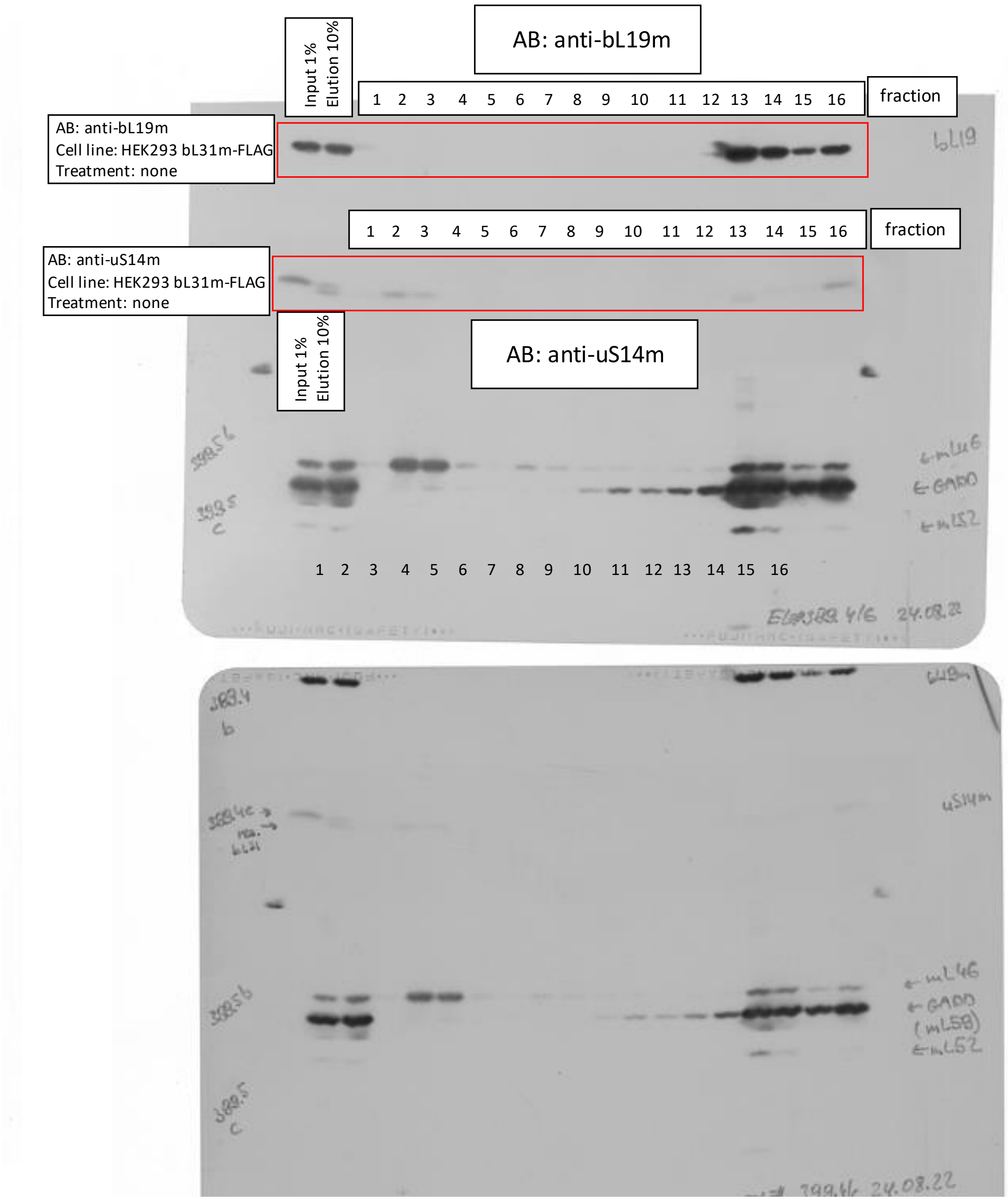

Source Data 3_related to Extended Data Fig.7b
EL#399.4-6 bL31m-FLAG IP + Gradient
Rotor: SW41 Ti
Gradient: Sucrose 5-30%
Speed: 158.000xg
Time: 15h

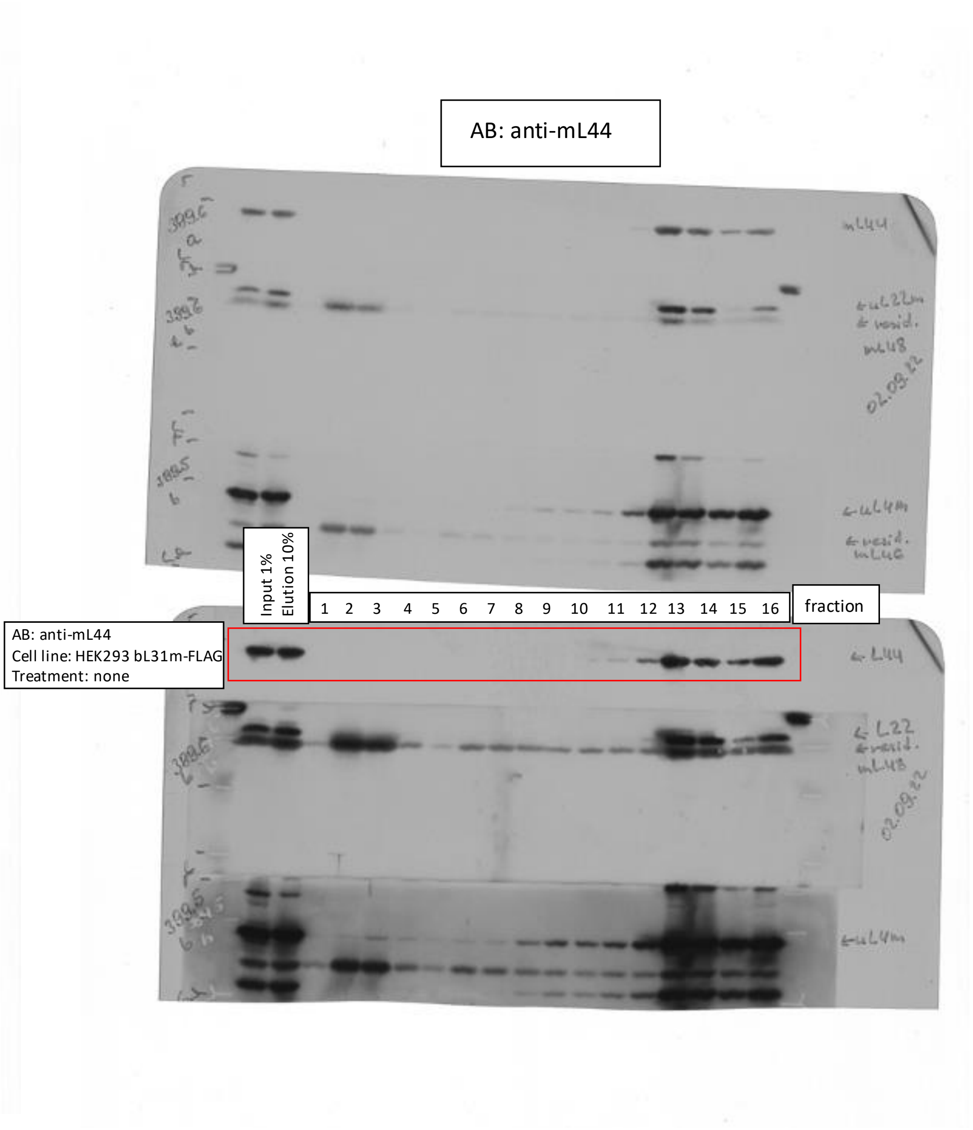

Source Data 3_related to Extended Data Fig.7b
EL#399.4-6 bL31m-FLAG IP + Gradient
Rotor: SW41 Ti
Gradient: Sucrose 5-30%
Speed: 158.000xg
Time: 15h

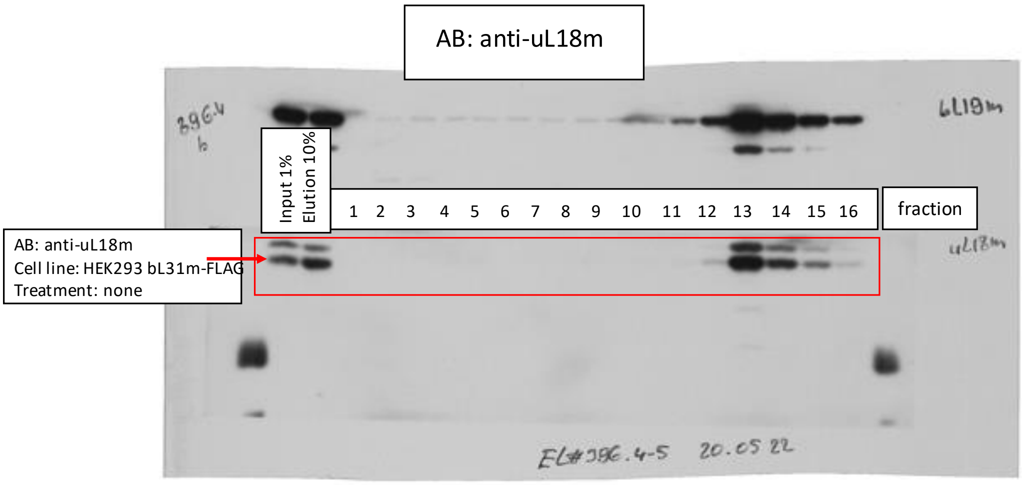

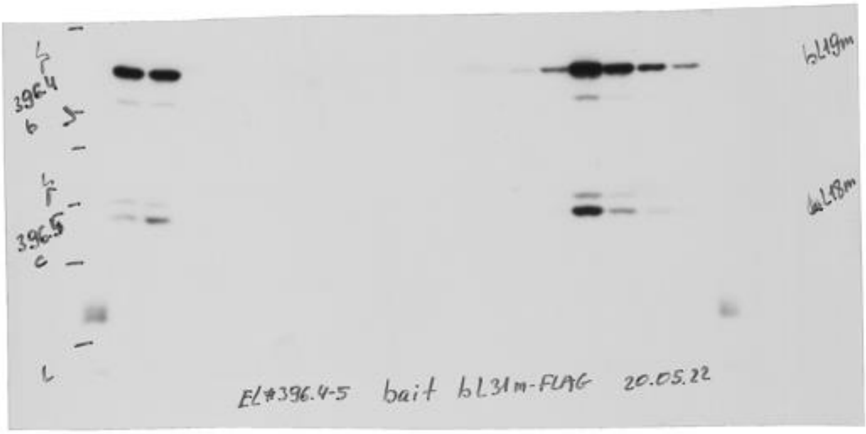

Source Data 3_related to Extended Data Fig.7c
EL#363 HEK293 WT + Ethidium Bromide treatment + Gradient
EL#379 HEK293 WT + Ethidium Bromide treatment + Gradient

Rotor: SW41 Ti
Gradient: Sucrose 5-30%
Speed: 158.000xg
Time: 15h

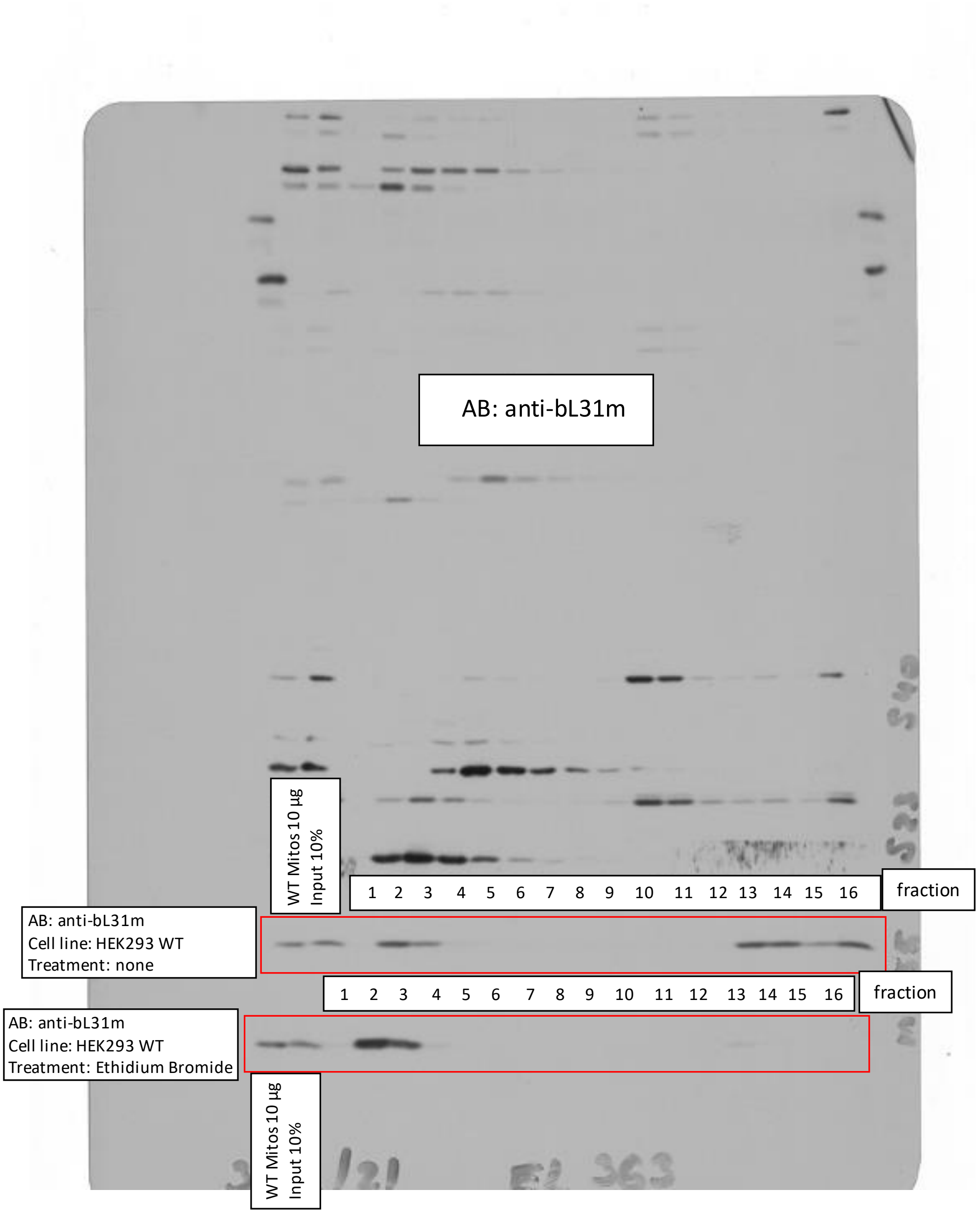

Source Data 3_related to Extended Data Fig.7c
EL#363 HEK293 WT + Ethidium Bromide treatment + Gradient
EL#379 HEK293 WT + Ethidium Bromide treatment + Gradient

Rotor: SW41 Ti
Gradient: Sucrose 5-30%
Speed: 158.000xg
Time: 15h

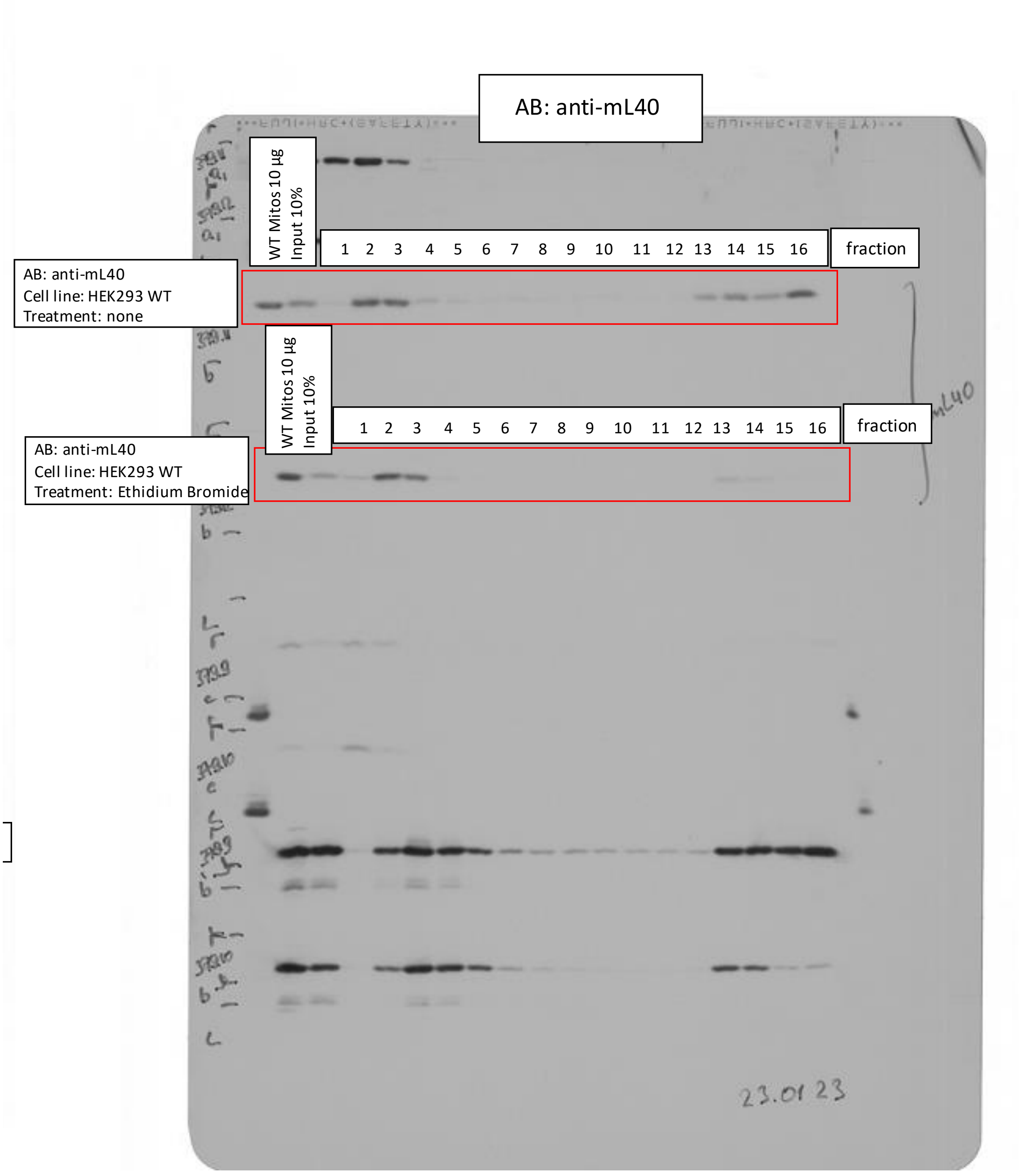

Source Data 3_related to Extended Data Fig.7c
EL#363 HEK293 WT + Ethidium Bromide treatment + Gradient
EL#379 HEK293 WT + Ethidium Bromide treatment + Gradient

Rotor: SW41 Ti
Gradient: Sucrose 5-30%
Speed: 158.000xg
Time: 15h

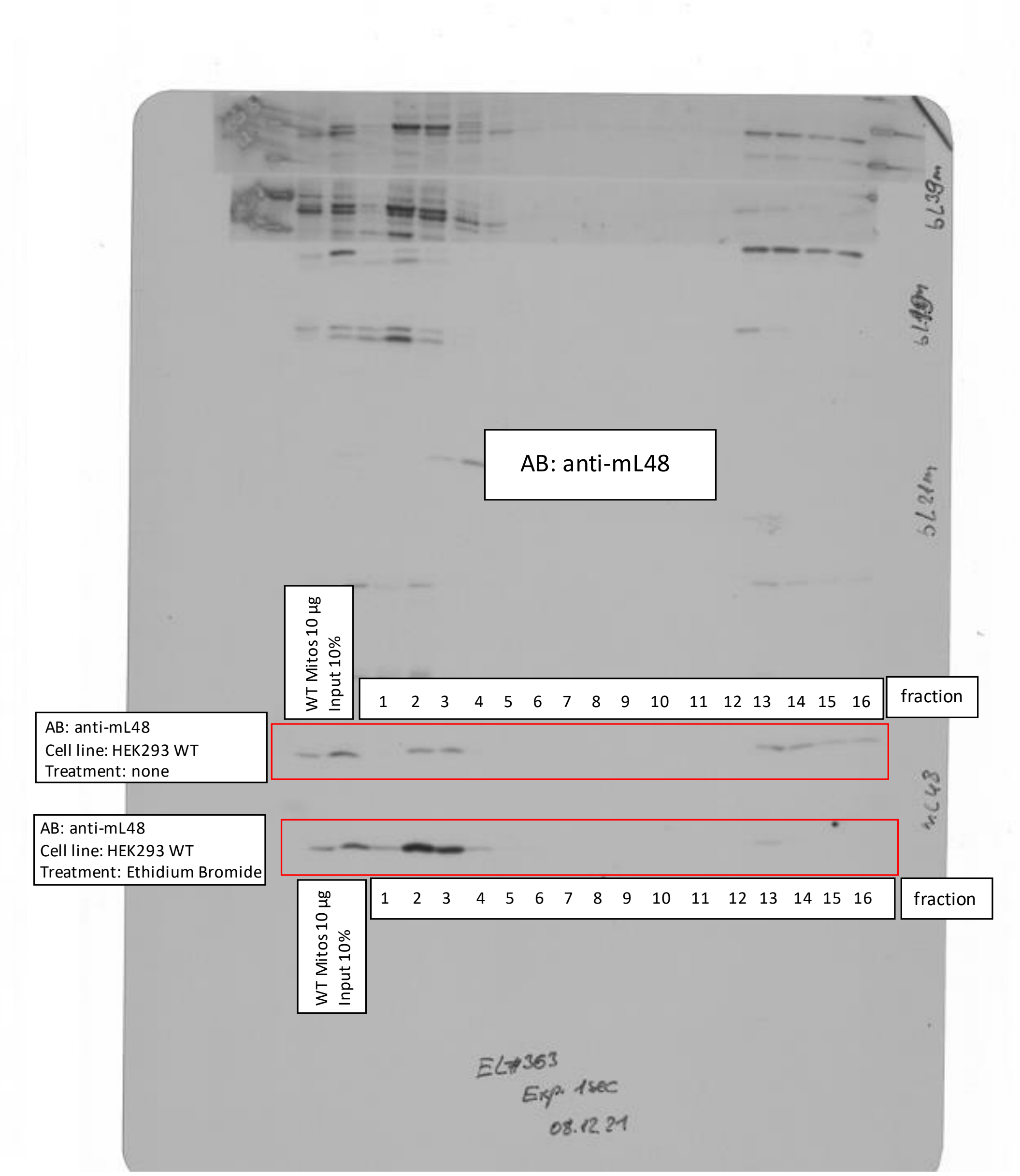

Source Data 3_related to Extended Data Fig.7c
EL#363 HEK293 WT + Ethidium Bromide treatment + Gradient
EL#379 HEK293 WT + Ethidium Bromide treatment + Gradient

Rotor: SW41 Ti
Gradient: Sucrose 5-30%
Speed: 158.000xg
Time: 15h

AB: anti-mL46

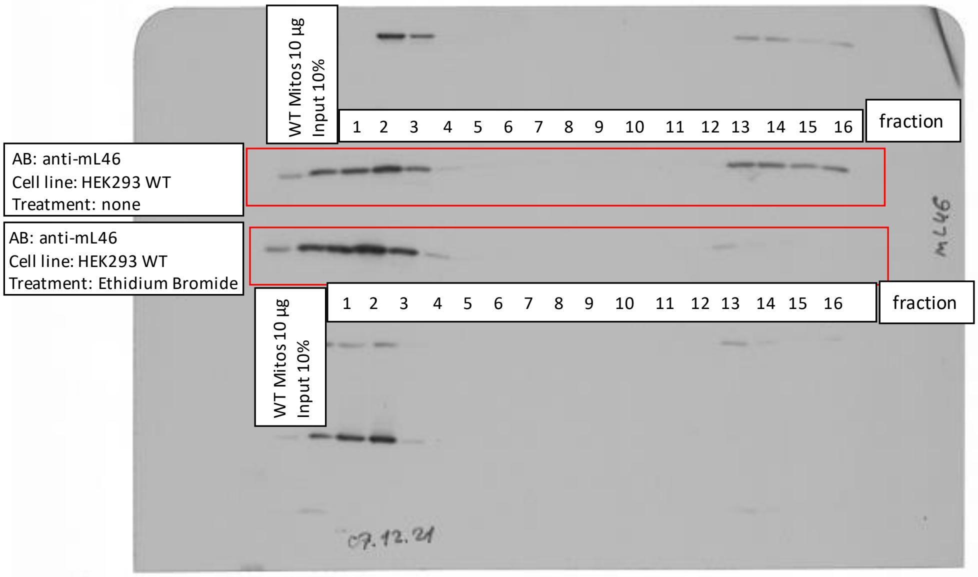

Source Data 3_related to Extended Data Fig.7c
EL#363 HEK293 WT + Ethidium Bromide treatment + Gradient
EL#379 HEK293 WT + Ethidium Bromide treatment + Gradient

Rotor: SW41 Ti
Gradient: Sucrose 5-30%
Speed: 158.000xg
Time: 15h

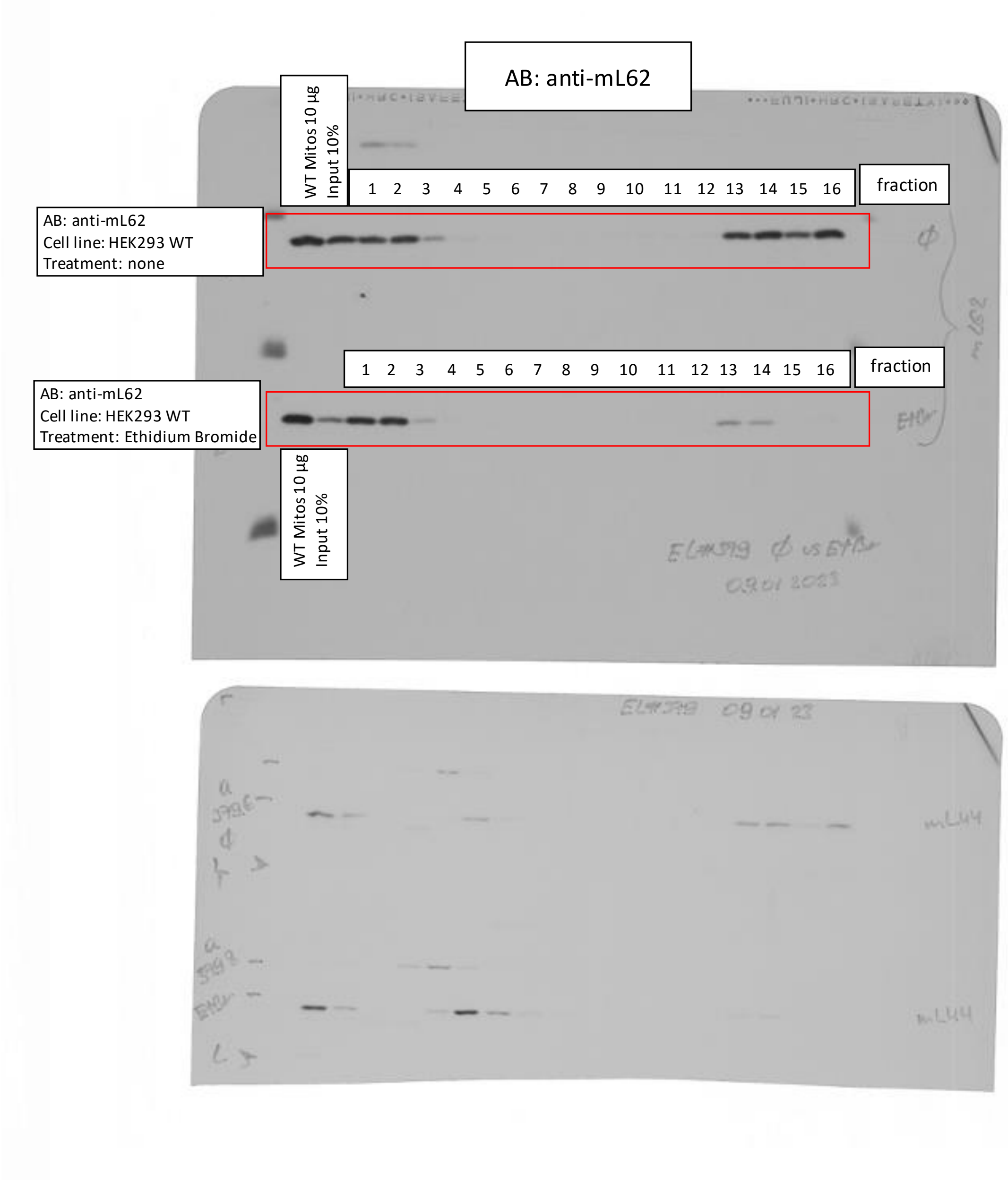

Source Data 3_related to Extended Data Fig.7c
EL#363 HEK293 WT + Ethidium Bromide treatment + Gradient
EL#379 HEK293 WT + Ethidium Bromide treatment + Gradient

Rotor: SW41 Ti
Gradient: Sucrose 5-30%
Speed: 158.000xg
Time: 15h

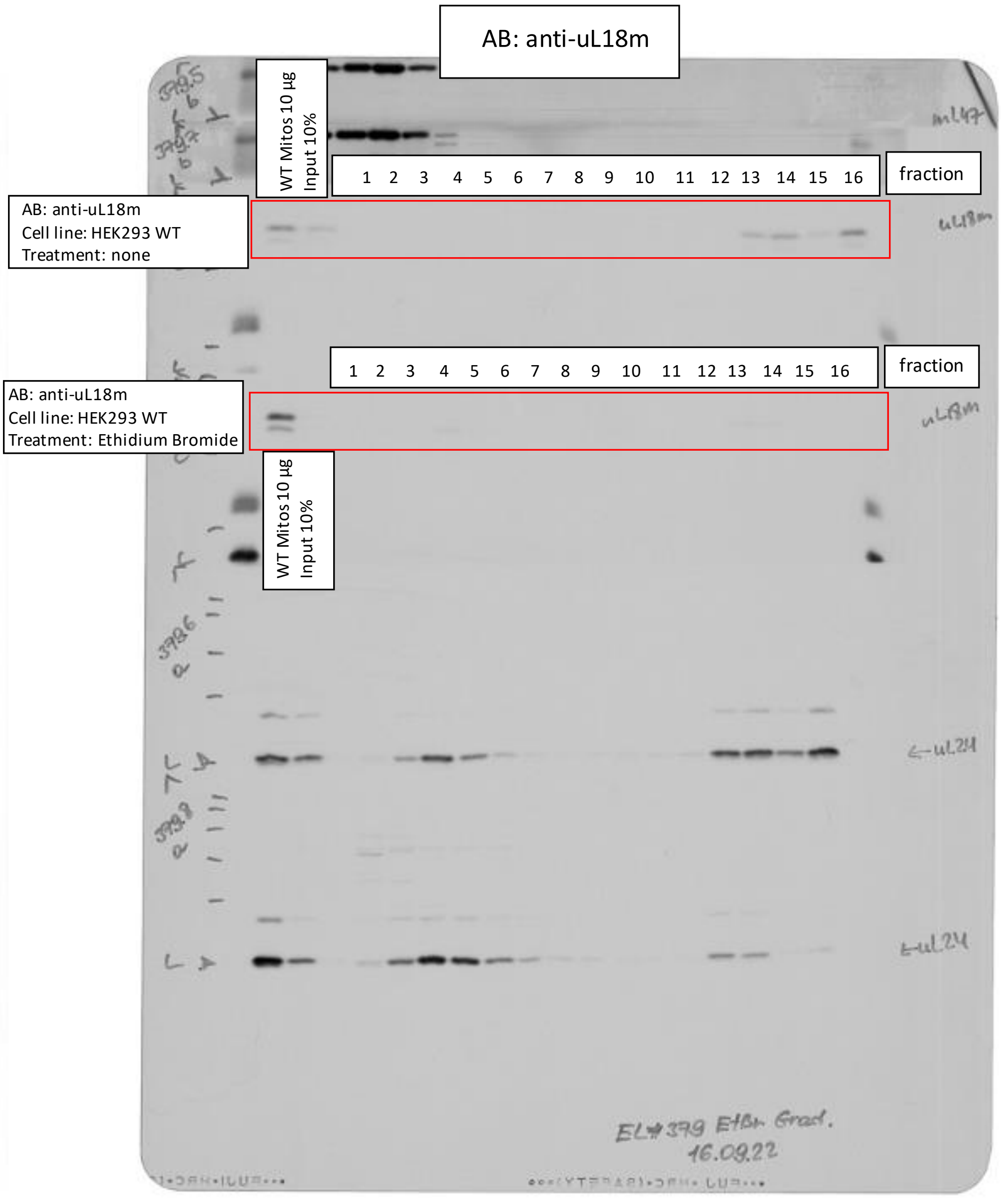

Source Data 3_related to Extended Data Fig.7c
EL#363 HEK293 WT + Ethidium Bromide treatment + Gradient
EL#379 HEK293 WT + Ethidium Bromide treatment + Gradient

Rotor: SW41 Ti
Gradient: Sucrose 5-30%
Speed: 158.000xg
Time: 15h

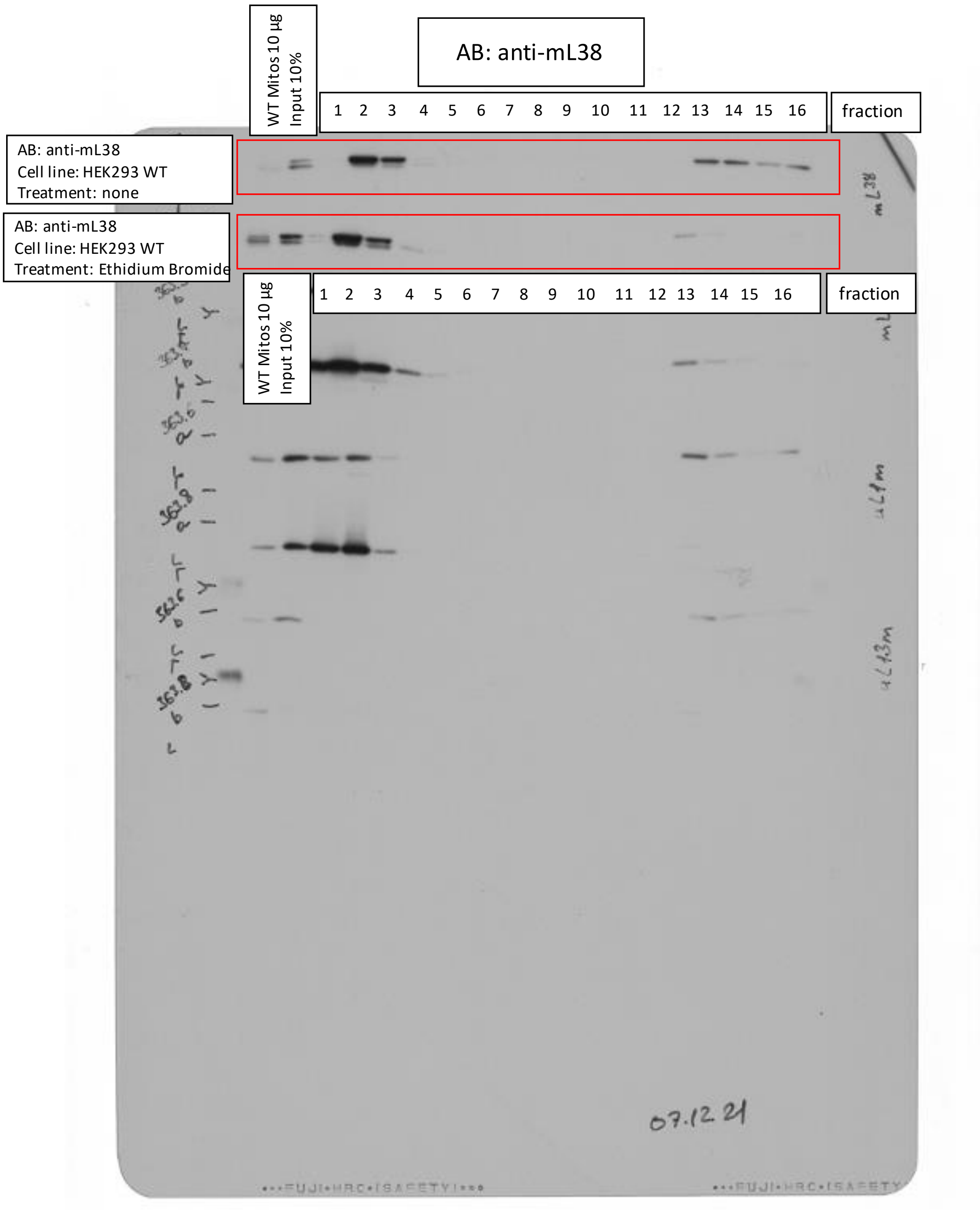

Source Data 3_related to Extended Data Fig.7d
VC#154 HEK293 WT vs. HEK293 mL62 KO
Rotor: SW41 Ti
Gradient: Sucrose 5-30%
Speed: 21500xg
Time: 15h

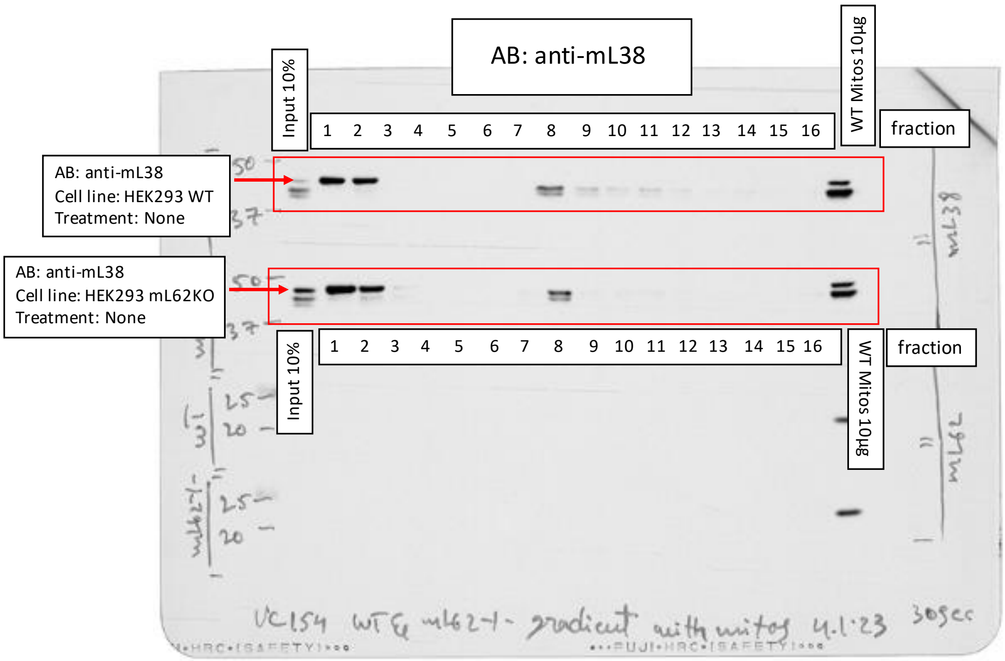

Source Data 3_related to Extended Data Fig.7d
VC#154 HEK293 WT vs. HEK293 mL62 KO
Rotor: SW41 Ti
Gradient: Sucrose 5-30%
Speed: 21500xg
Time: 15h

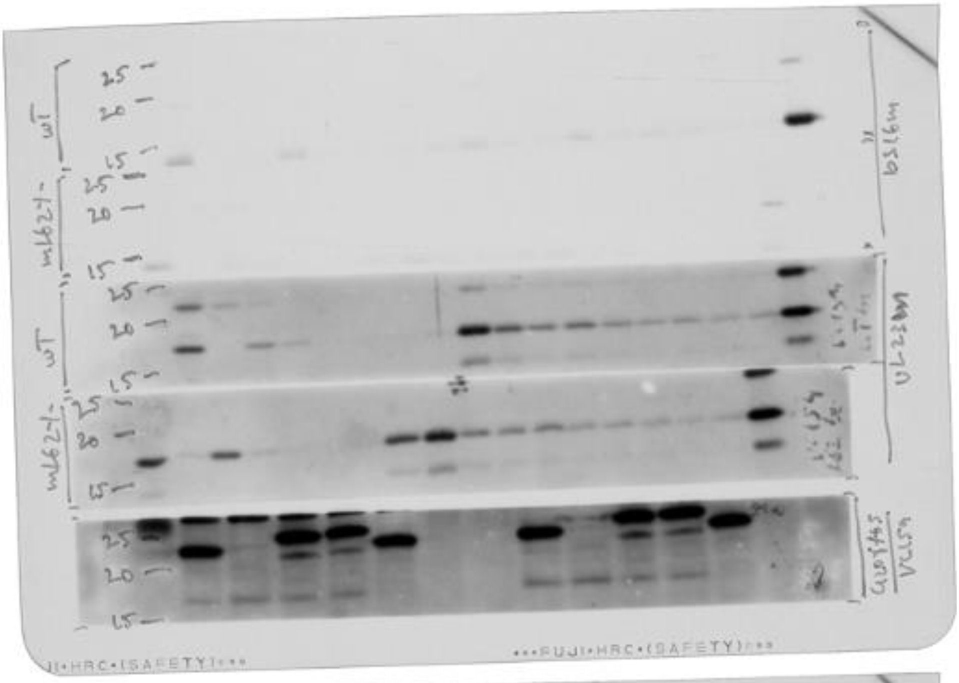

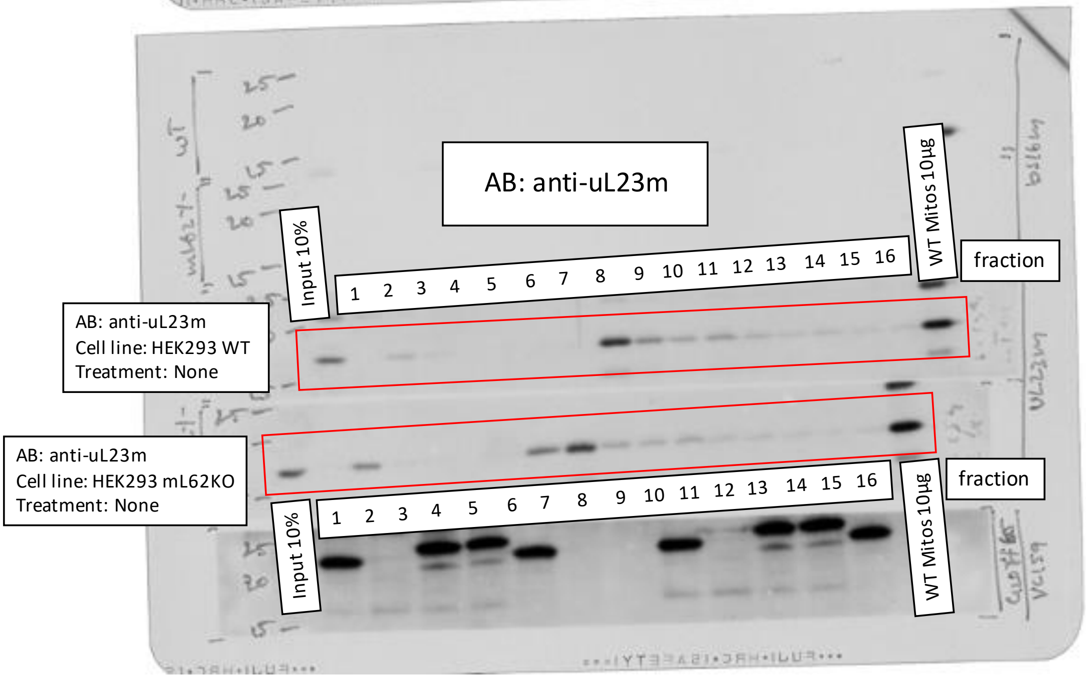

Source Data 3_related to Extended Data Fig.7d
VC#154 HEK293 WT vs. HEK293 mL62 KO
Rotor: SW41 Ti
Gradient: Sucrose 5-30%
Speed: 21500xg
Time: 15h

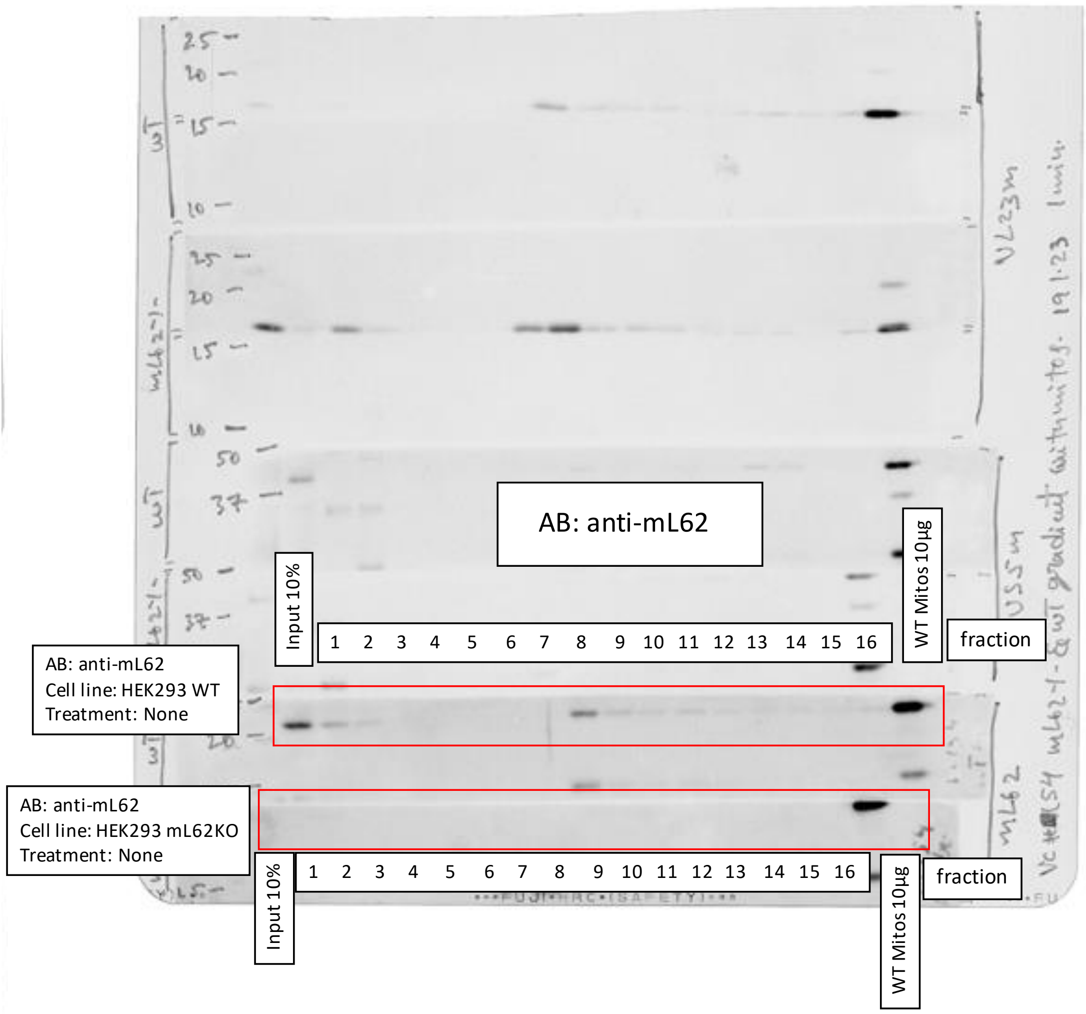

Source Data 3_related to Extended Data Fig.7d
VC#154 HEK293 WT vs. HEK293 mL62 KO
Rotor: SW41 Ti
Gradient: Sucrose 5-30%
Speed: 21500xg
Time: 15h

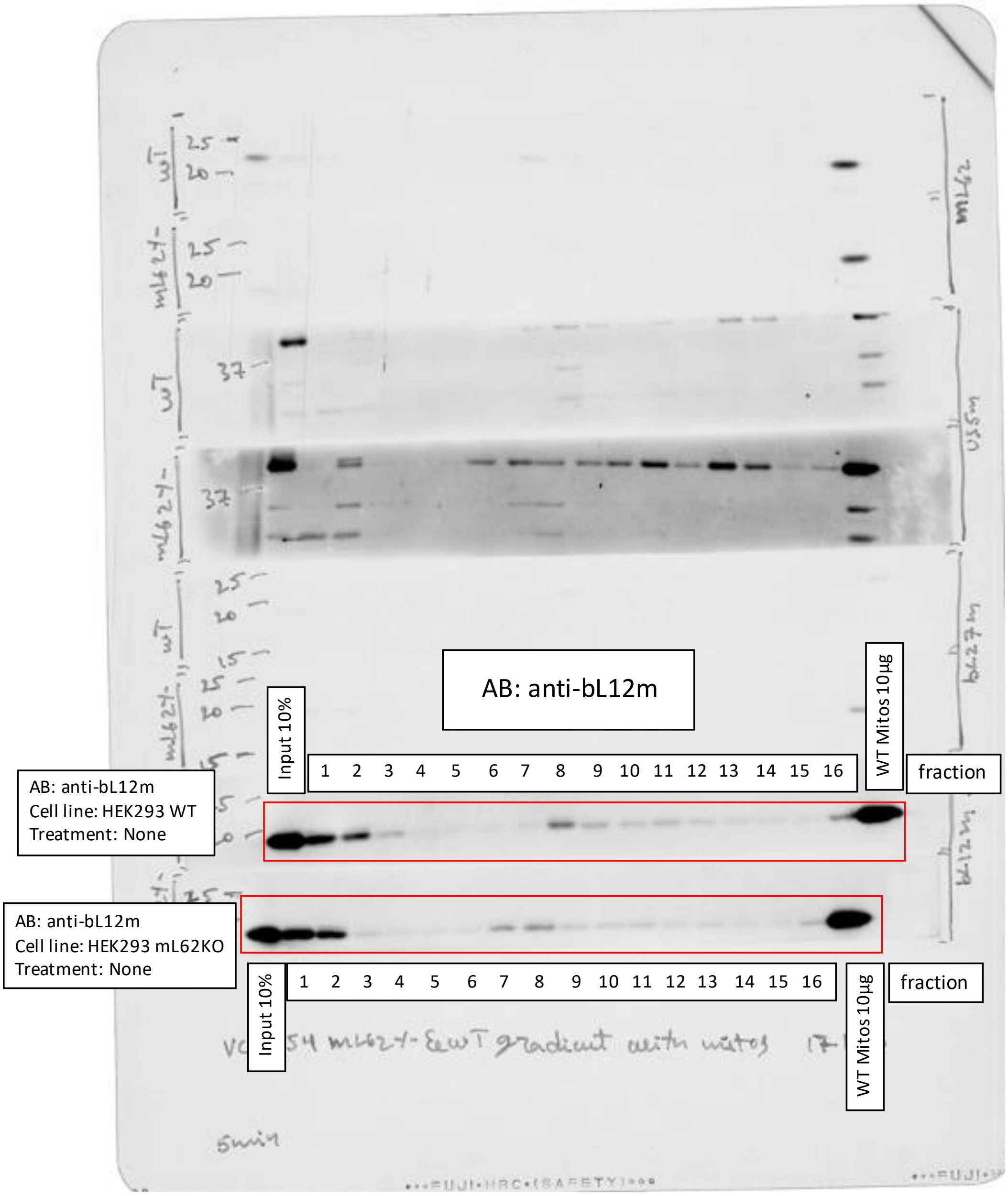

Source Data 3_related to Extended Data Fig.7d
VC#154 HEK293 WT vs. HEK293 mL62 KO
Rotor: SW41 Ti
Gradient: Sucrose 5-30%
Speed: 21500xg
Time: 15h

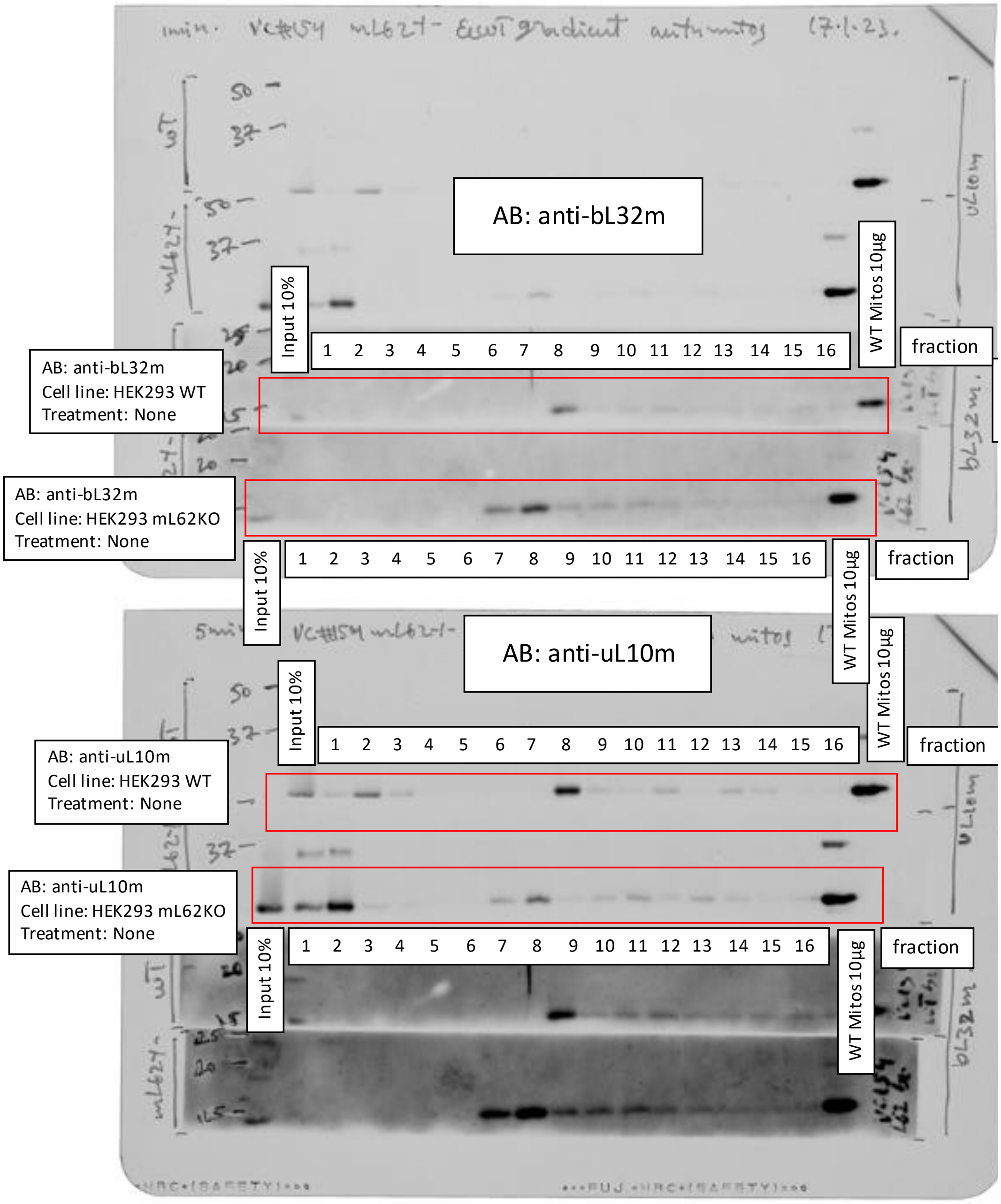

Source Data 3_related to Extended Data Fig.7d
VC#154 HEK293 WT vs. HEK293 mL62 KO
Rotor: SW41 Ti
Gradient: Sucrose 5-30%
Speed: 21500xg
Time: 15h

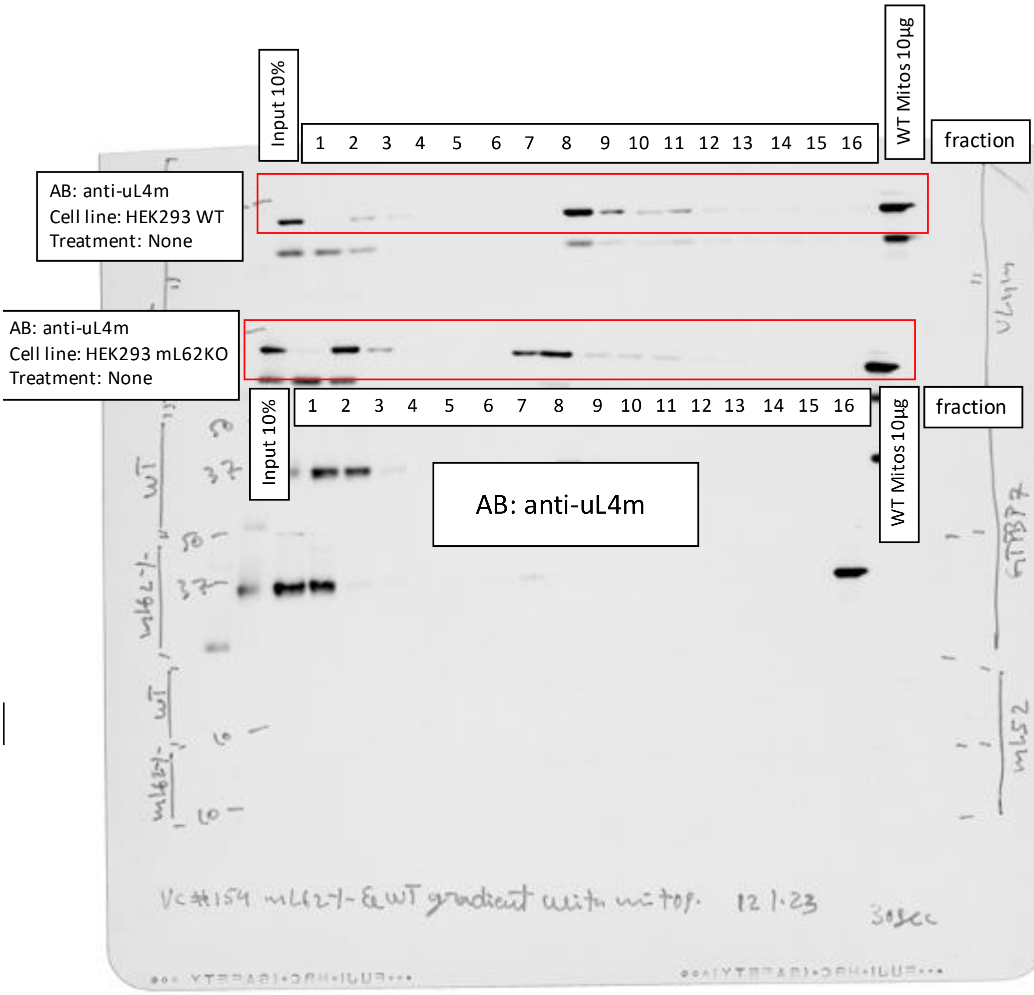

Source Data 3_related to Extended Data Fig.7d
VC#154 HEK293 WT vs. HEK293 mL62 KO
Rotor: SW41 Ti
Gradient: Sucrose 5-30%
Speed: 21500xg
Time: 15h

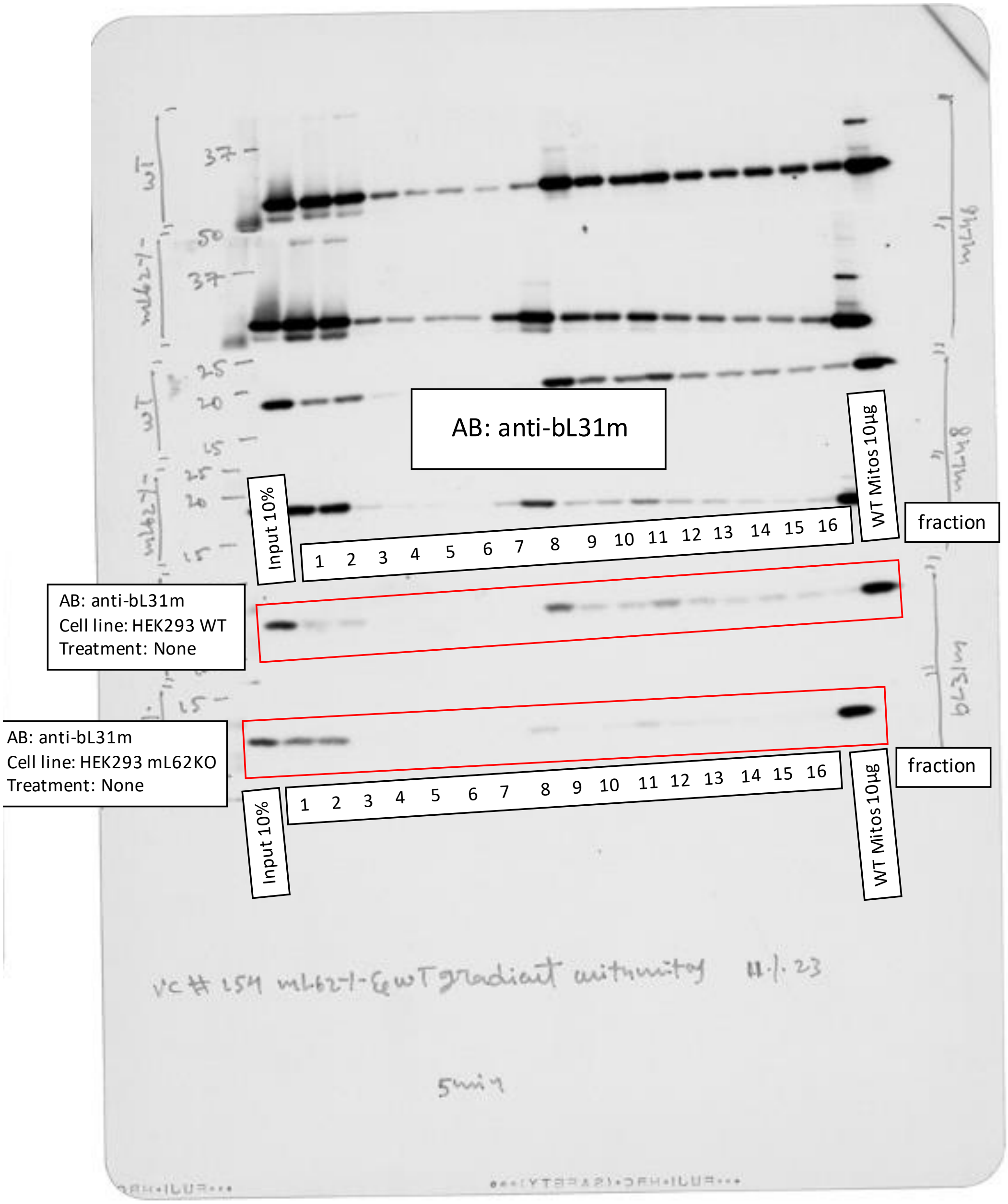

Source Data 3_related to Extended Data Fig.7d
VC#154 HEK293 WT vs. HEK293 mL62 KO
Rotor: SW41 Ti
Gradient: Sucrose 5-30%
Speed: 21500xg
Time: 15h

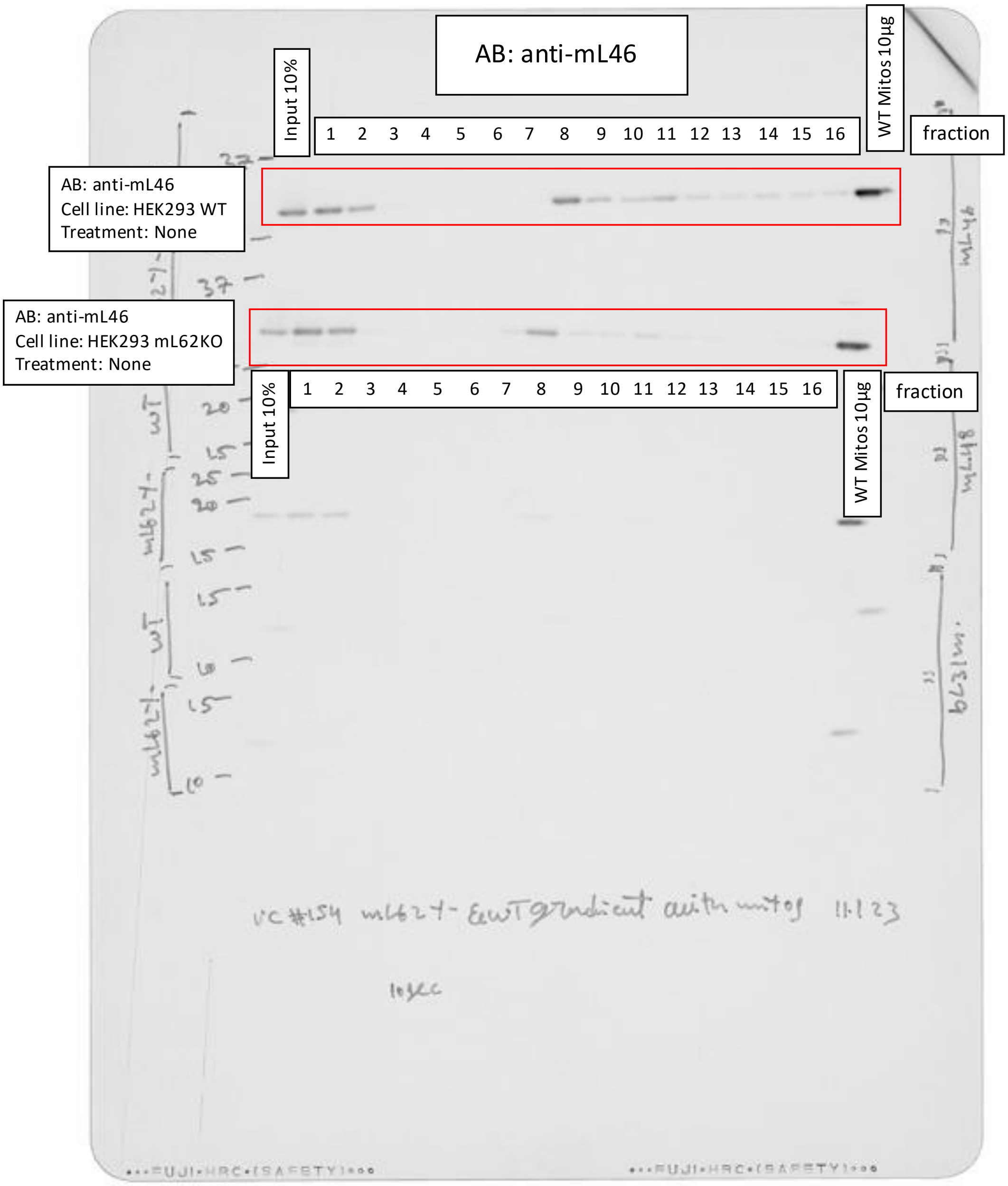

Source Data 3_related to Extended Data Fig.7d
VC#154 HEK293 WT vs. HEK293 mL62 KO
Rotor: SW41 Ti
Gradient: Sucrose 5-30%
Speed: 21500xg
Time: 15h

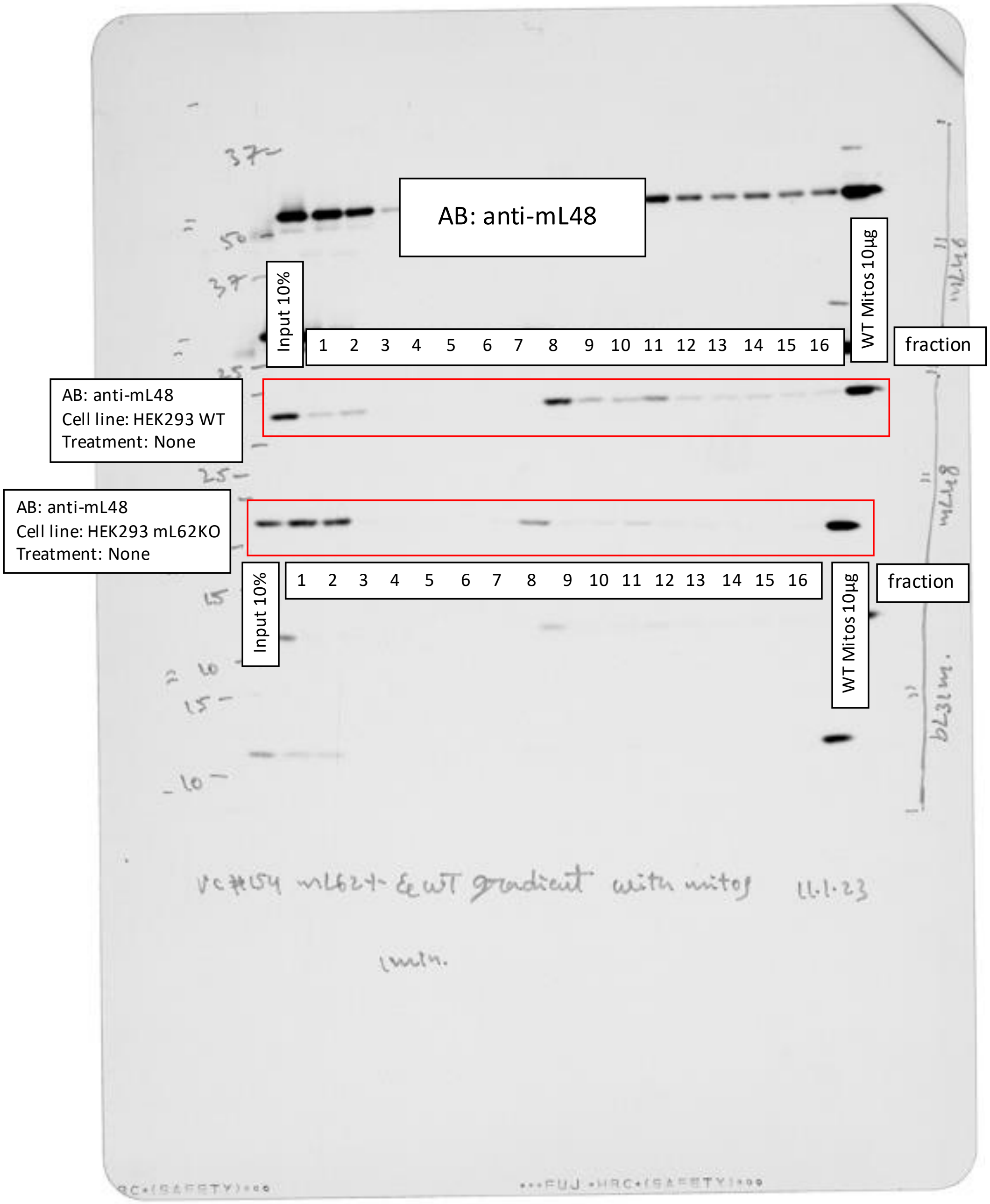

Source Data 3_related to Extended Data Fig.7d
VC#154 HEK293 WT vs. HEK293 mL62 KO
Rotor: SW41 Ti
Gradient: Sucrose 5-30%
Speed: 21500xg
Time: 15h

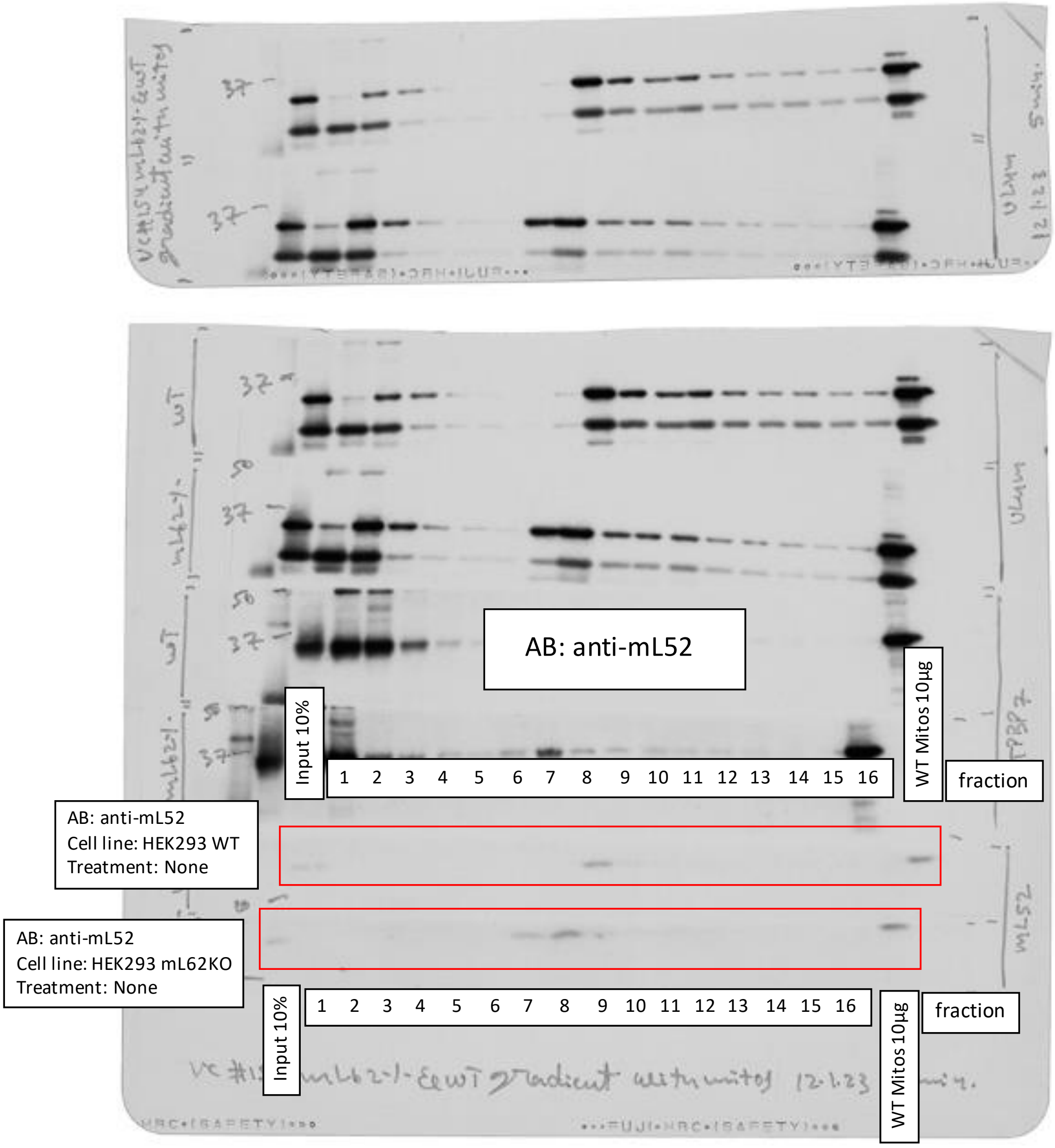

Source Data 3_related to Extended Data Fig.7d
VC#154 HEK293 WT vs. HEK293 mL62 KO
Rotor: SW41 Ti
Gradient: Sucrose 5-30%
Speed: 21500xg
Time: 15h

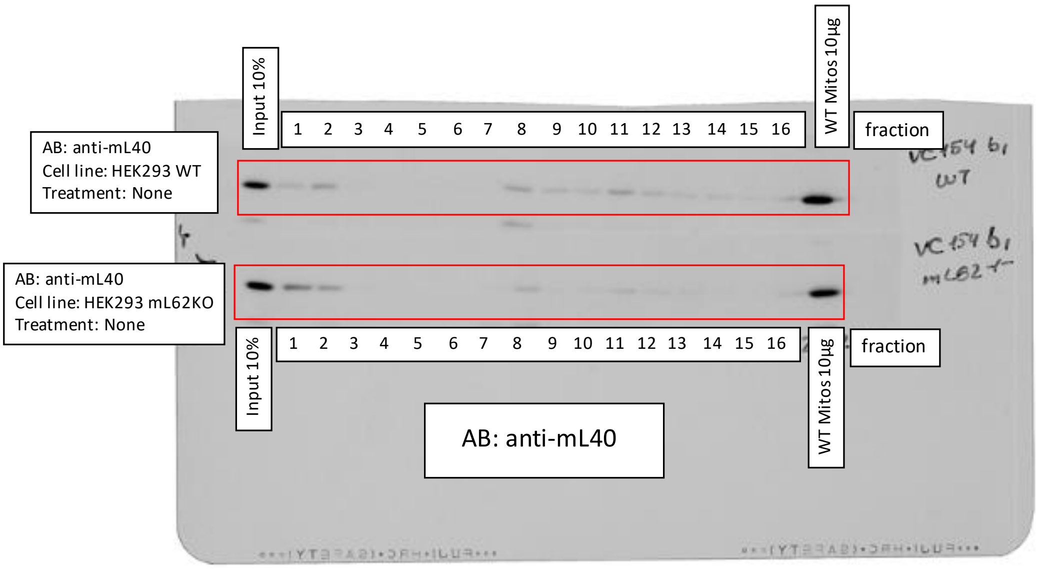

Source Data 3_related to Extended Data Fig.7d
VC#154 HEK293 WT vs. HEK293 mL62 KO
Rotor: SW41 Ti
Gradient: Sucrose 5-30%
Speed: 21500xg
Time: 15h

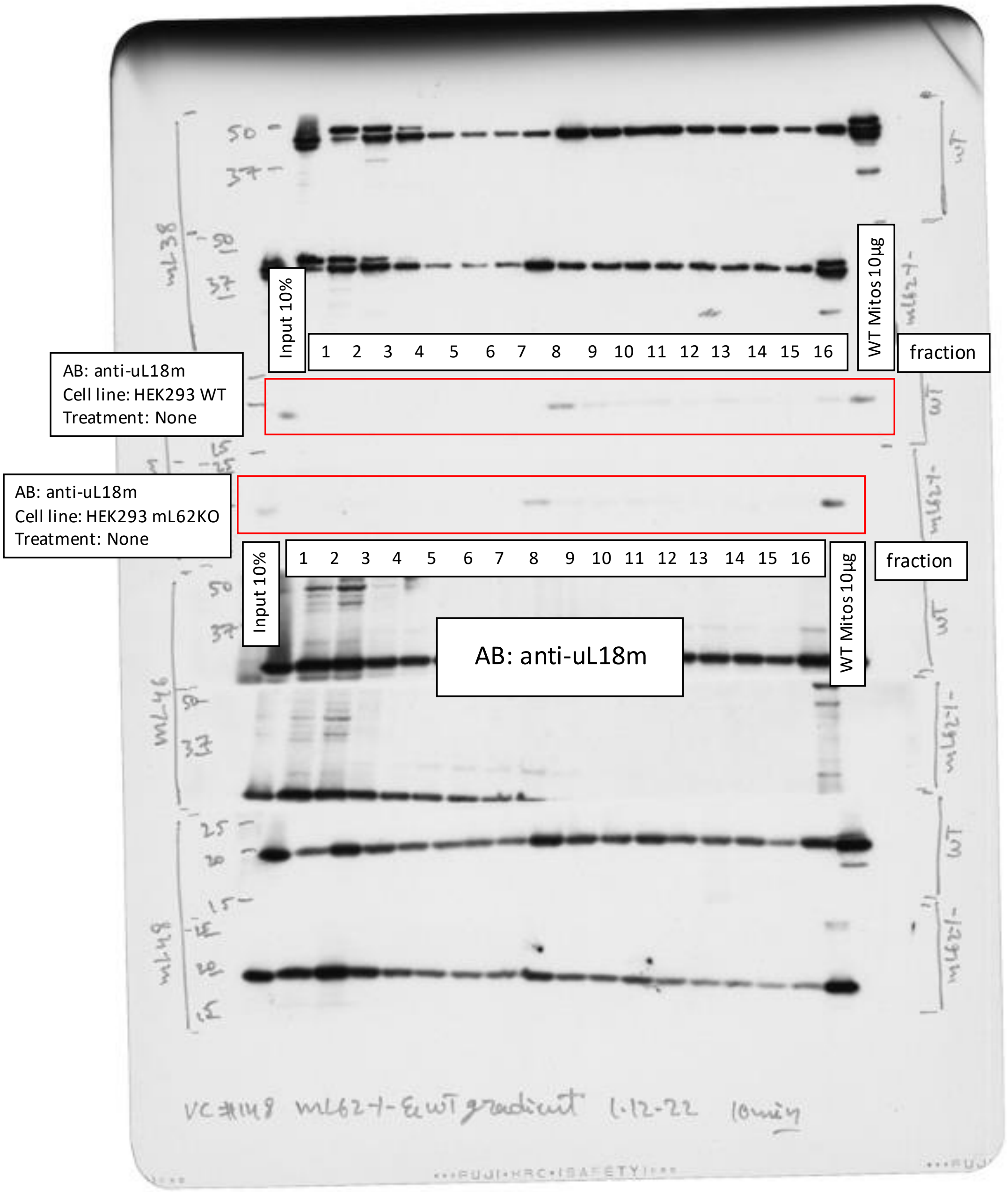

Source Data 3_related to Extended Data Fig.7e
VC#131 mL62KO and mL62KO +mL62FLAG 35S methionin labeling
Concentration of Radio activity: 200µCi/mL
Incubation Time: 1h

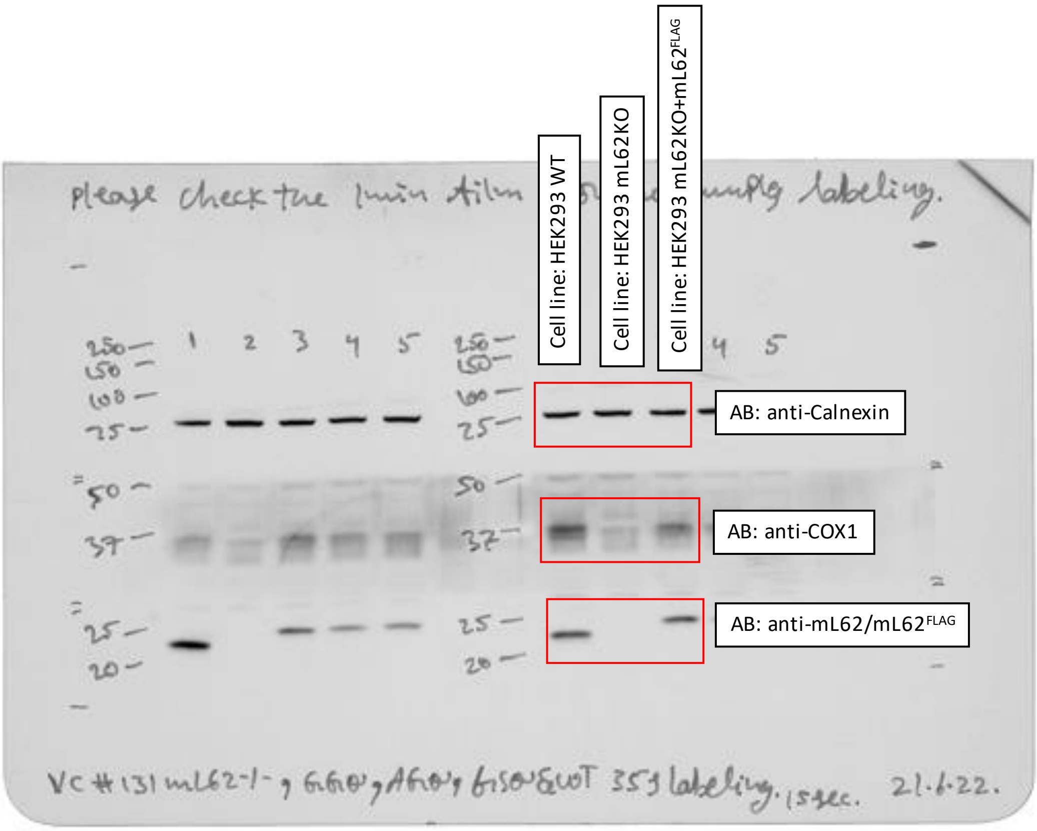

Source Data 3_related to Extended Data Fig.7e
VC#131 mL62KO and mL62KO +mL62FLAG 35S methionin labeling
Concentration of Radio activity: 200µCi/mL
Incubation Time: 1h

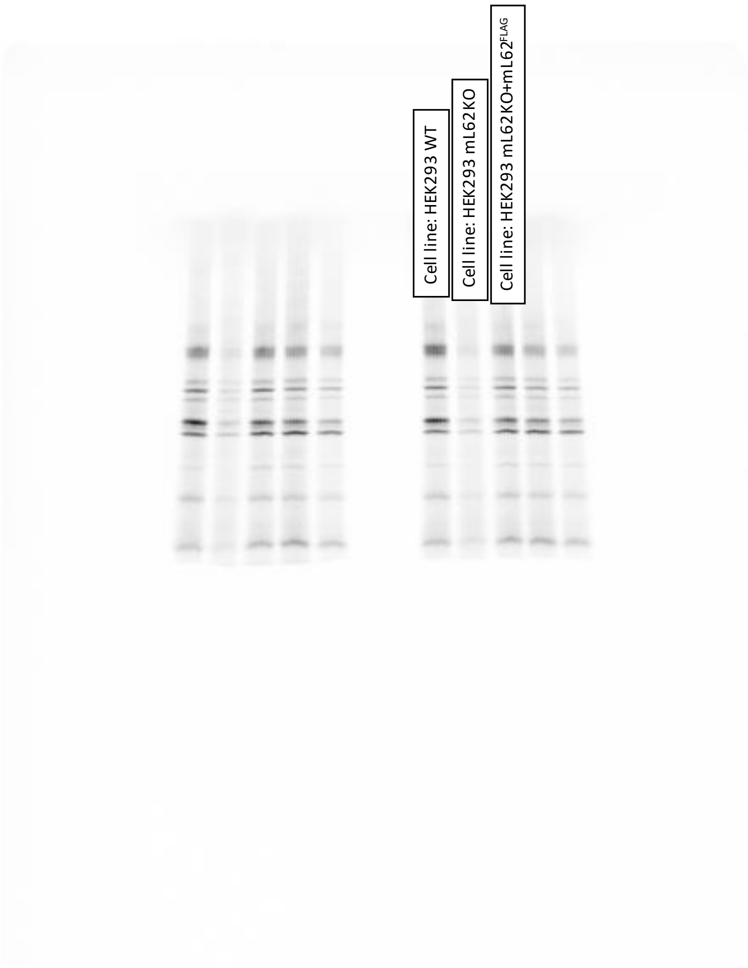

Supplement: Supplementary file 24 — Unprocessed blots. [file 41594_2024_1356_MOESM24_ESM.pdf]
