# Supplementary material for: A roadmap for ribosome assembly in human mitochondria
Source: Nat Struct Mol Biol. 2024 Jul 11;31(12):1898–908. doi: 10.1038/s41594-024-01356-w (PMC11638073; doi:10.1038/s41594-024-01356-w)

Source Data 4_related to Extended Data Fig.8a
EL#377.1-3 uL11m-FLAG IP + Gradient
Rotor: SW41 Ti
Gradient: Sucrose 5-30%
Speed: 158.000xg
Time: 15h

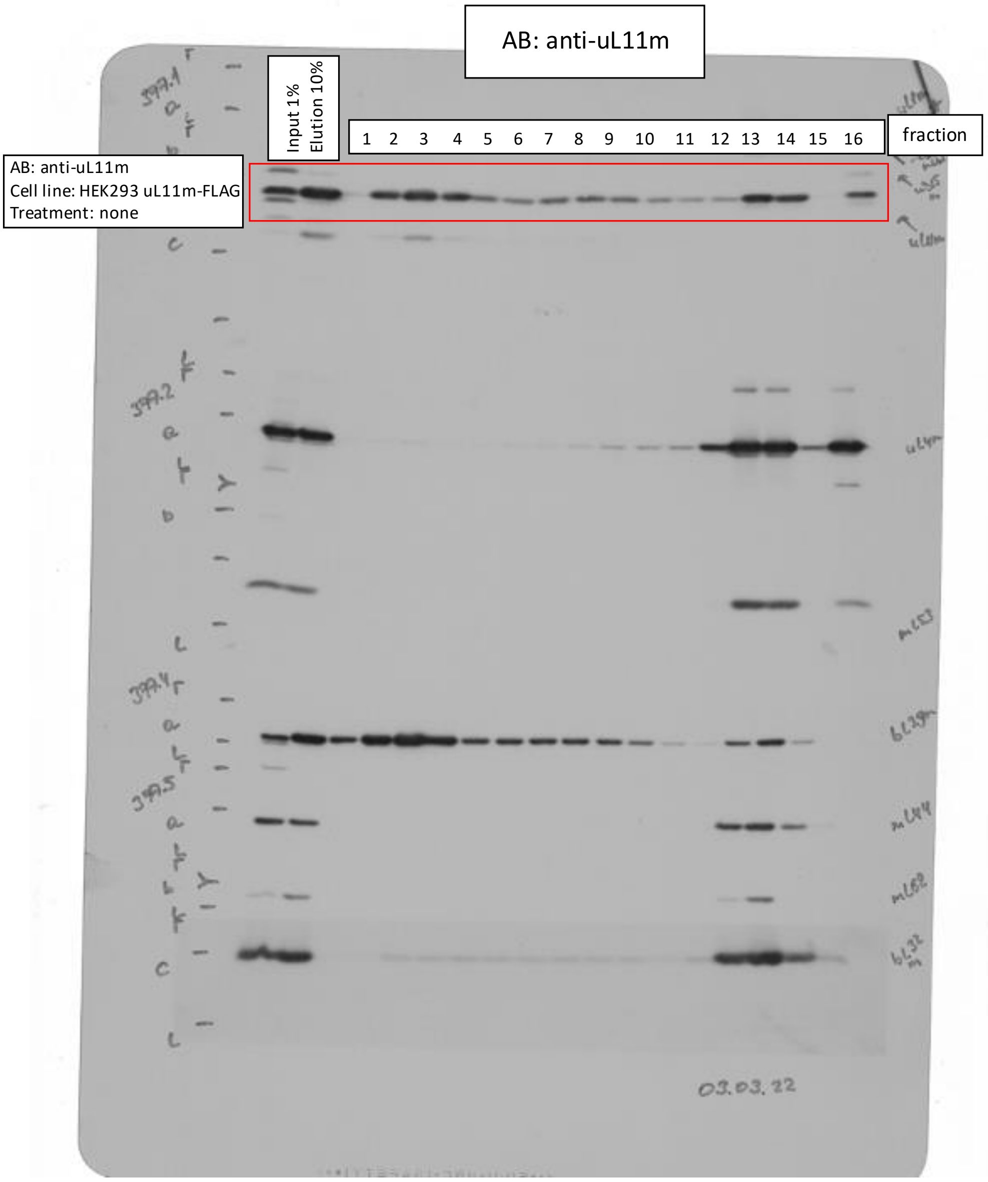

Source Data 4_related to Extended Data Fig.8a
EL#377.1-3 uL11m-FLAG IP + Gradient
Rotor: SW41 Ti
Gradient: Sucrose 5-30%
Speed: 158.000xg
Time: 15h

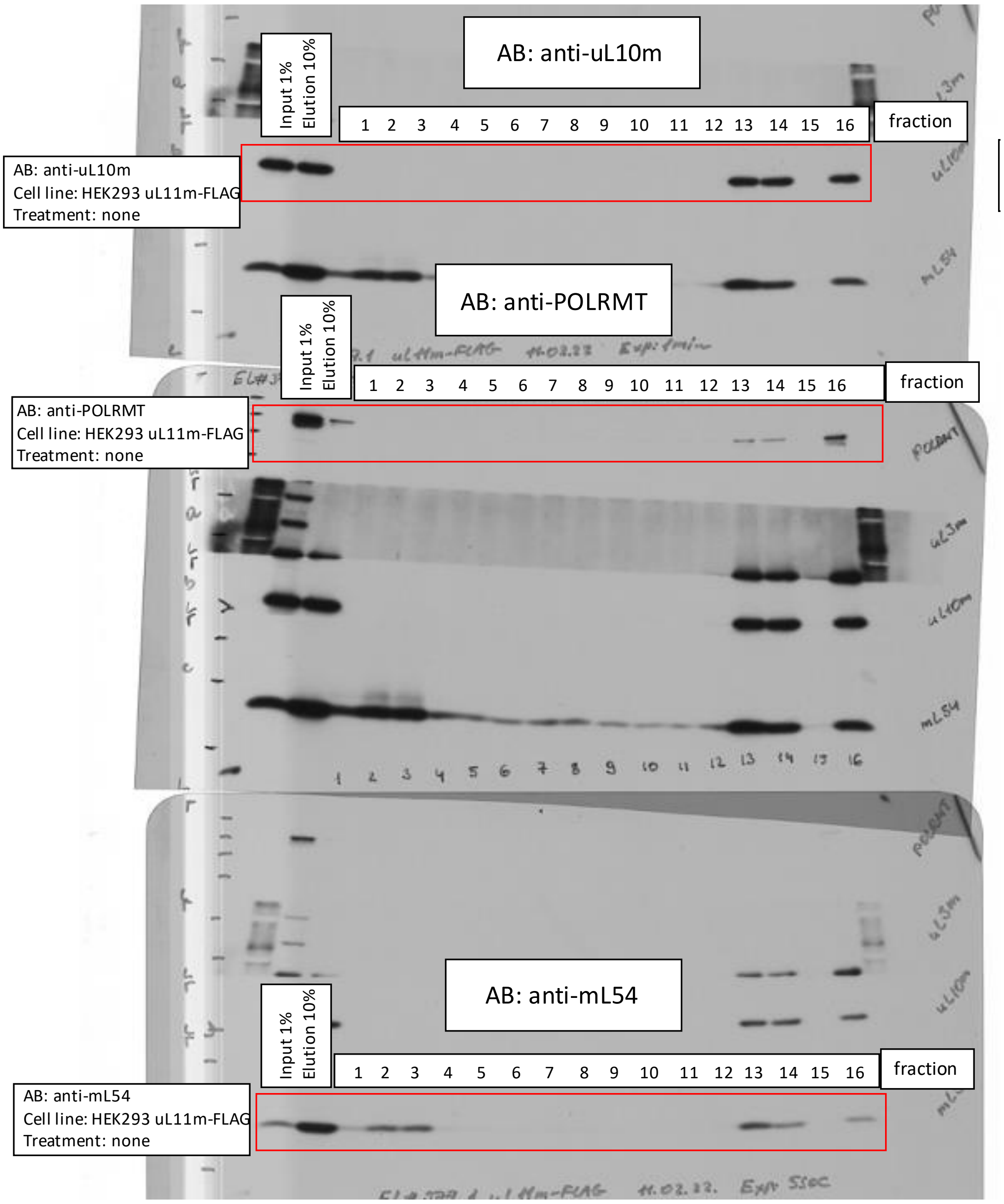

Source Data 4_related to Extended Data Fig.8a
EL#377.1-3 uL11m-FLAG IP + Gradient
Rotor: SW41 Ti
Gradient: Sucrose 5-30%
Speed: 158.000xg
Time: 15h

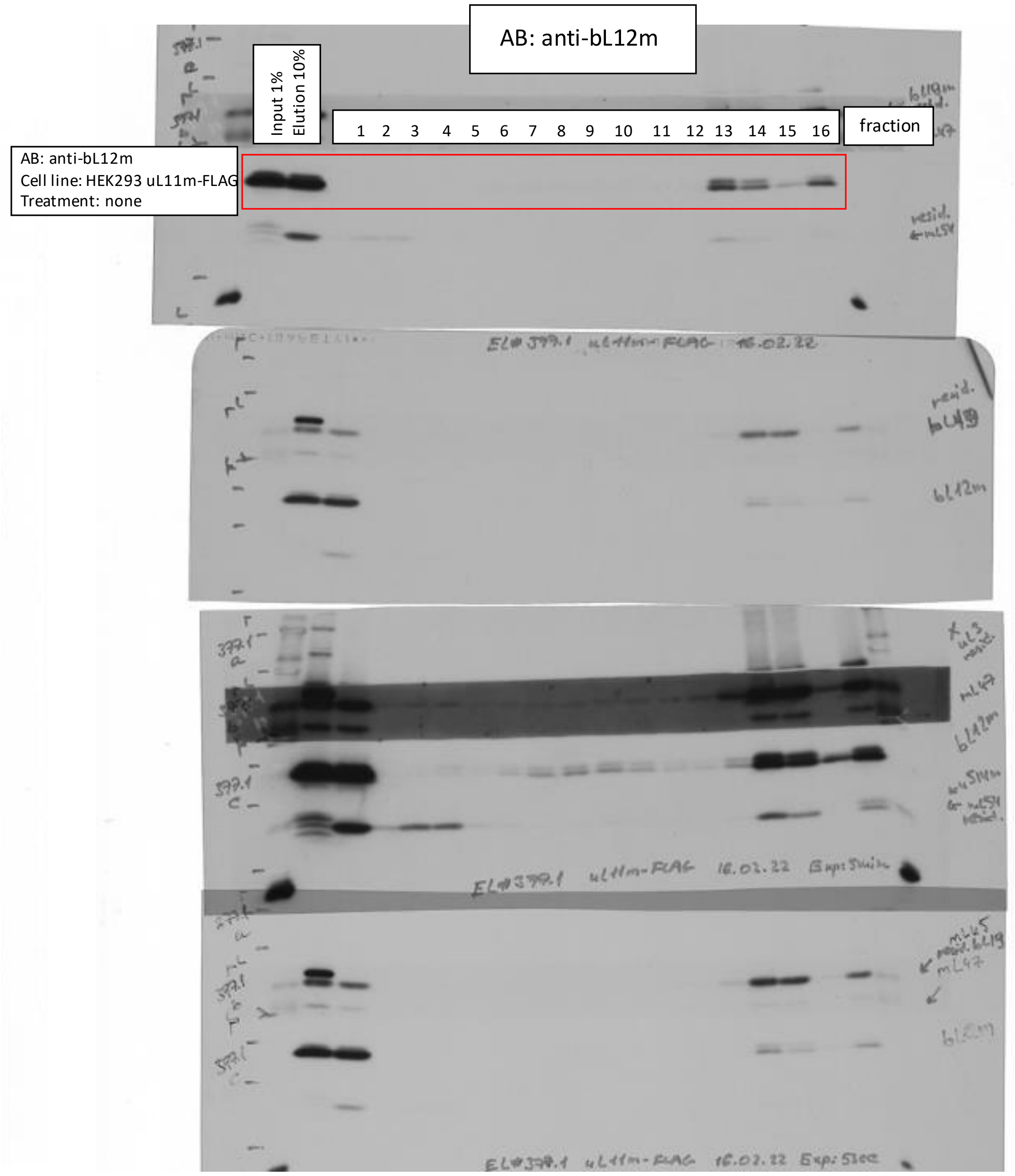

Source Data 4_related to Extended Data Fig.8a
EL#377.1-3 uL11m-FLAG IP + Gradient
Rotor: SW41 Ti
Gradient: Sucrose 5-30%
Speed: 158.000xg
Time: 15h

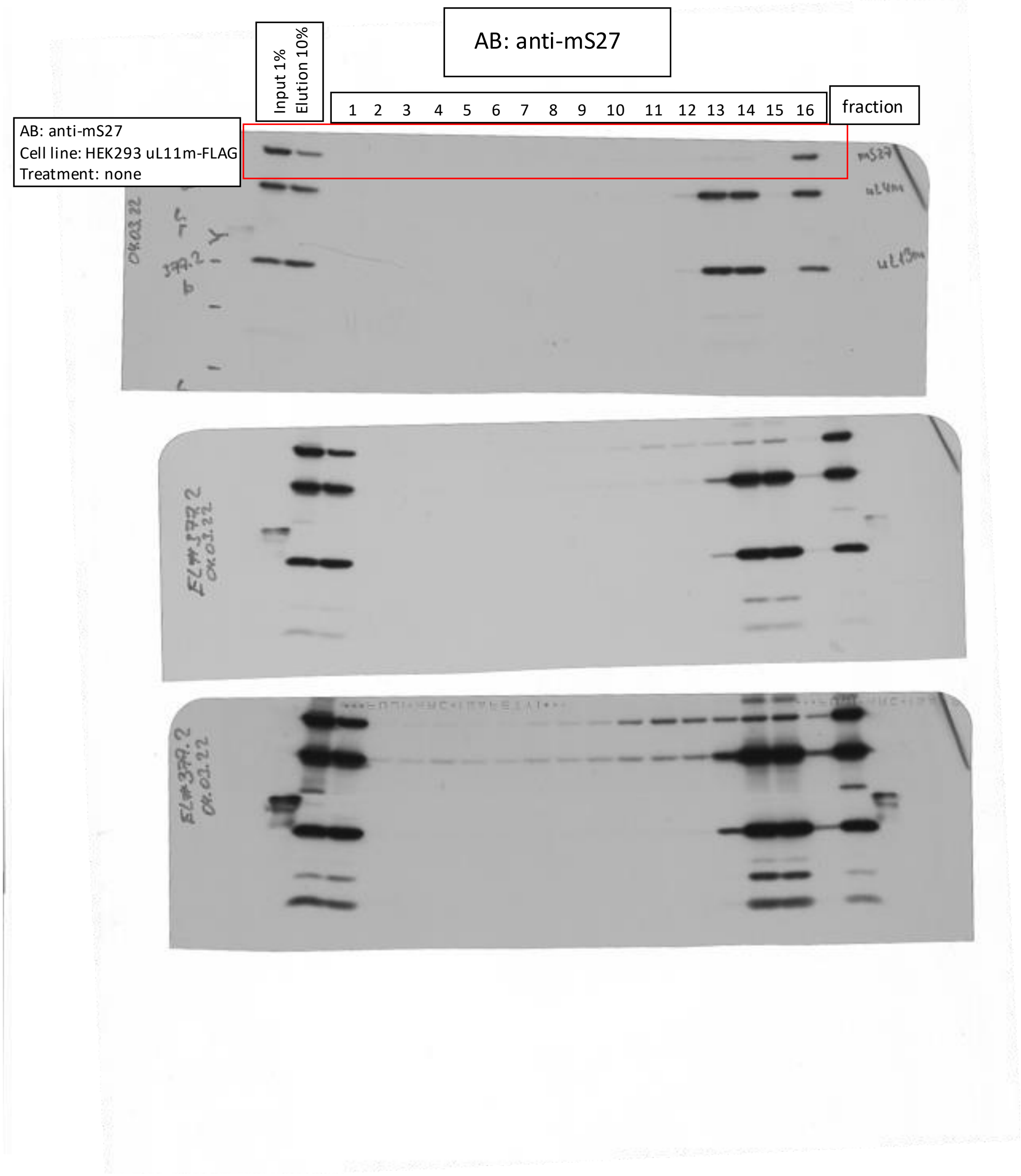

Source Data 4_related to Extended Data Fig.8a
EL#377.1-3 uL11m-FLAG IP + Gradient
Rotor: SW41 Ti
Gradient: Sucrose 5-30%
Speed: 158.000xg
Time: 15h

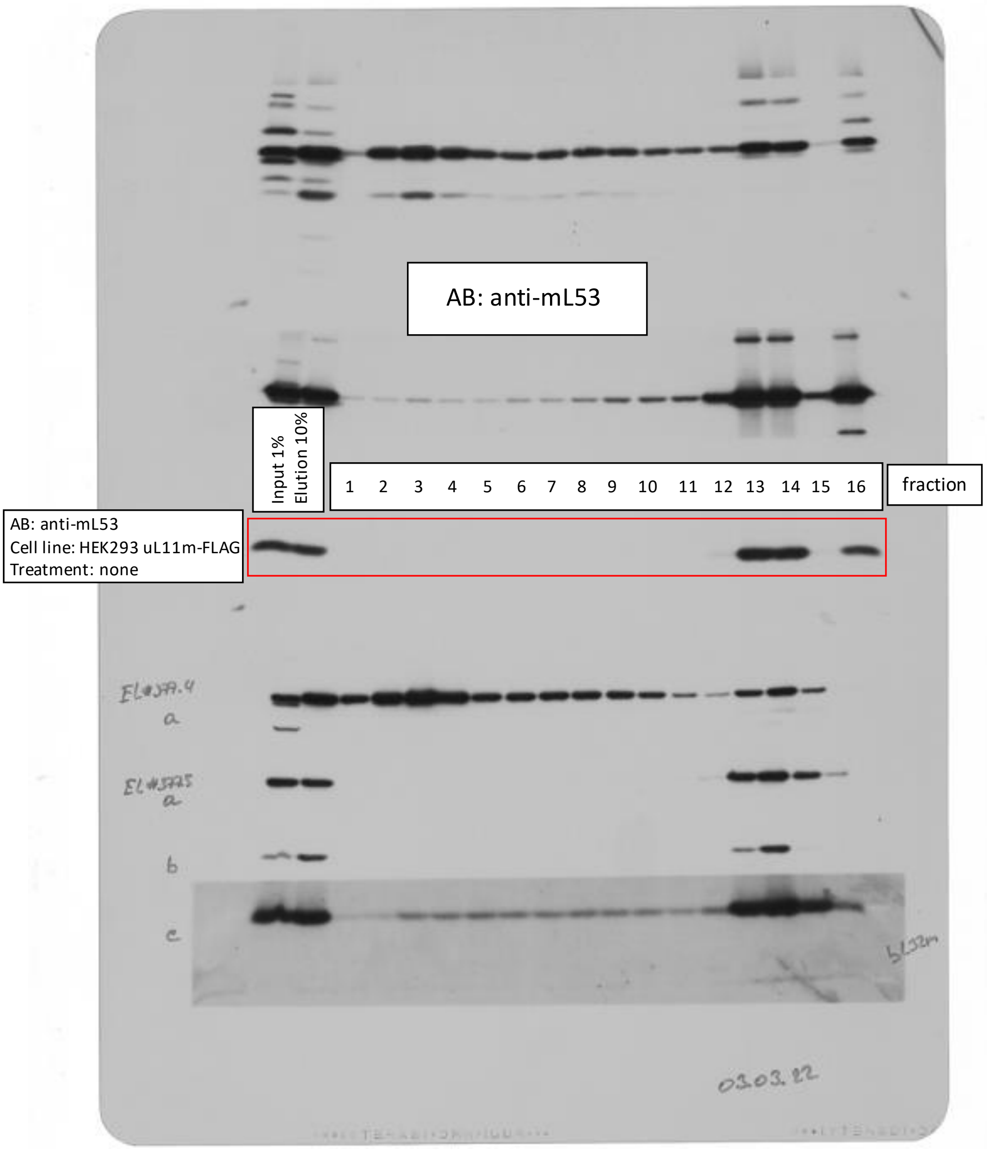

Source Data 4_related to Extended Data Fig.8a
EL#377.1-3 uL11m-FLAG IP + Gradient
Rotor: SW41 Ti
Gradient: Sucrose 5-30%
Speed: 158.000xg
Time: 15h

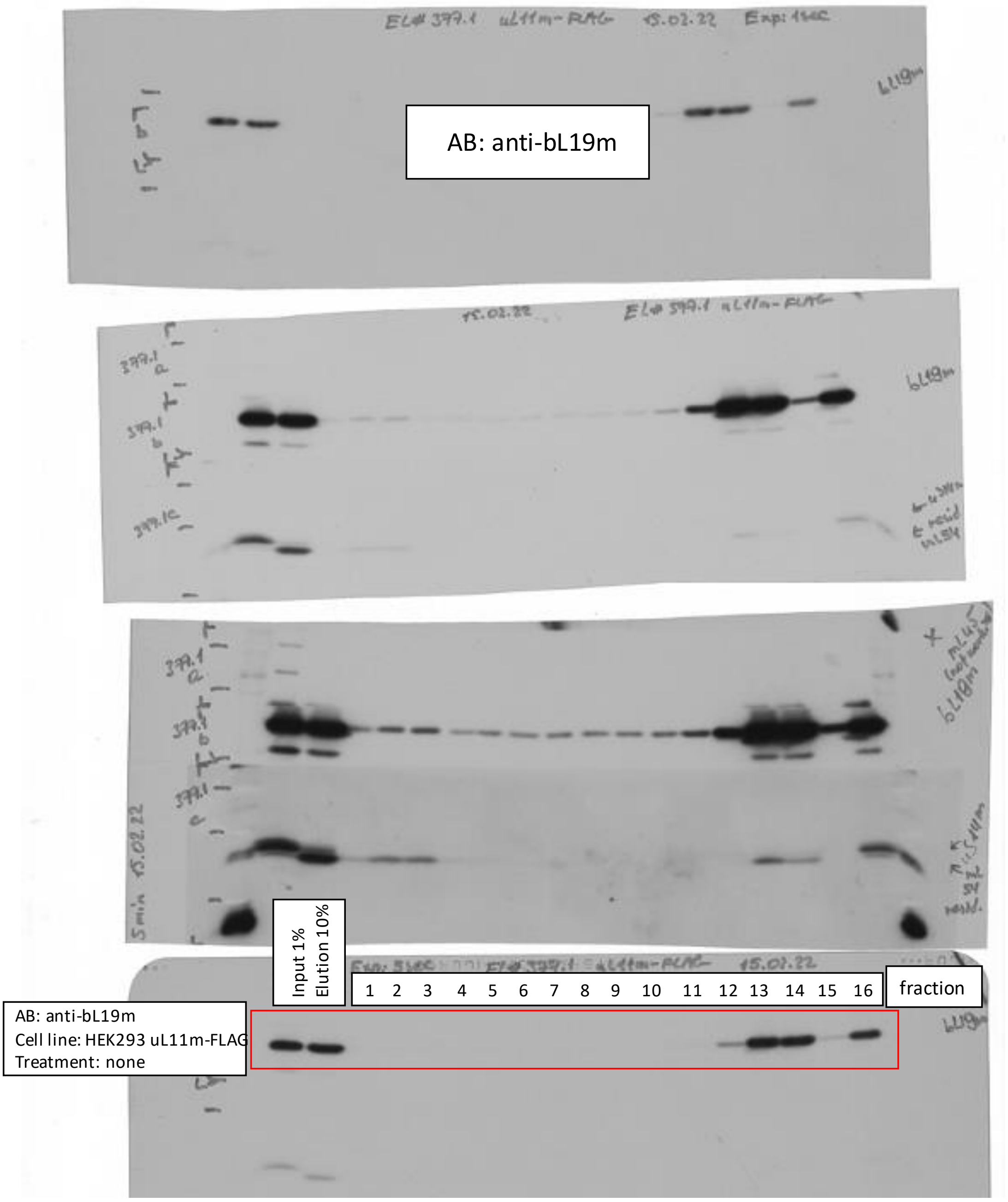

Source Data 4_related to Extended Data Fig.8b
EL#412.4-6 bL12m-FLAG IP + Gradient
Rotor: SW41 Ti
Gradient: Sucrose 5-30%
Speed: 158.000xg
Time: 15h

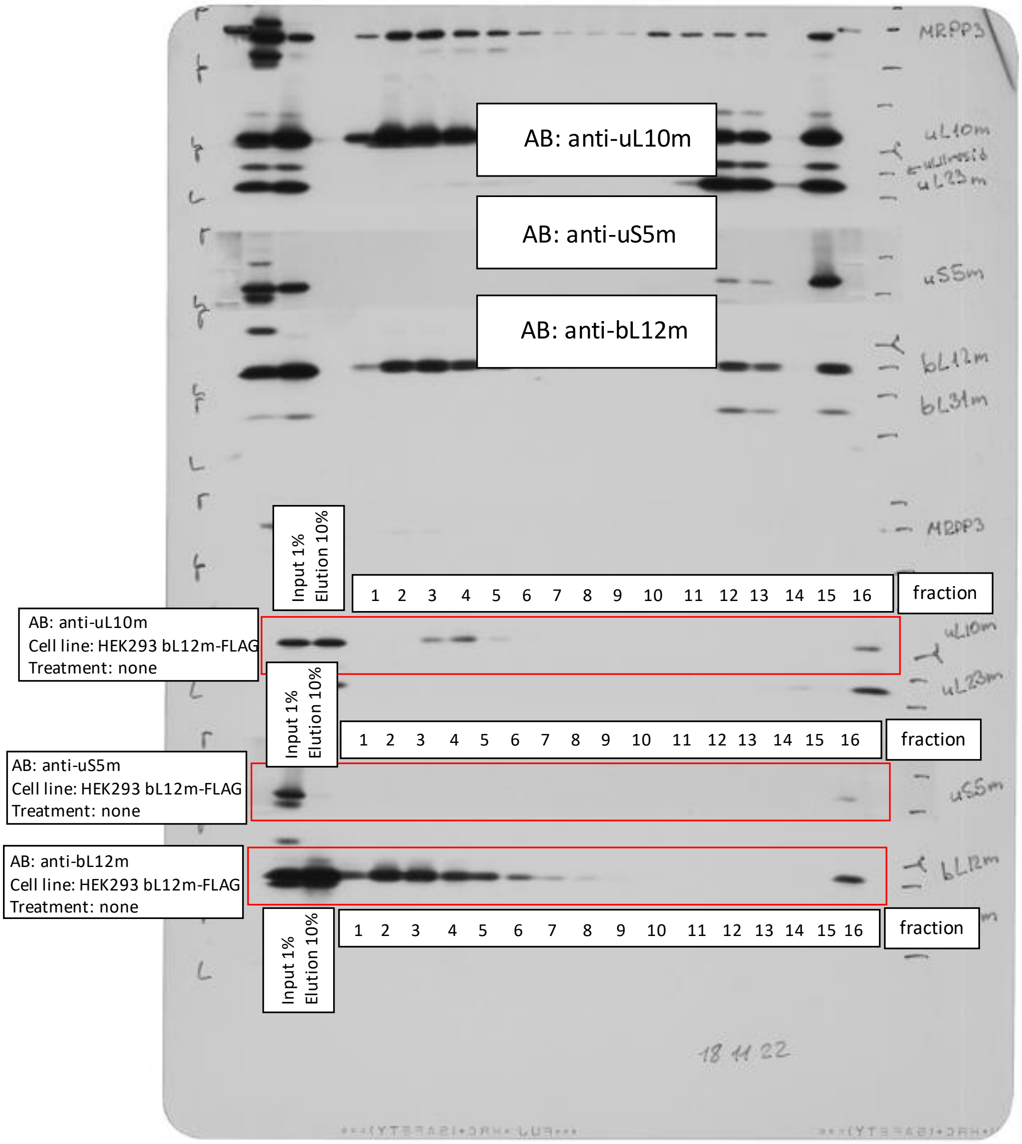

Source Data 4_related to Extended Data Fig.8b
EL#412.4-6 bL12m-FLAG IP + Gradient
Rotor: SW41 Ti
Gradient: Sucrose 5-30%
Speed: 158.000xg
Time: 15h

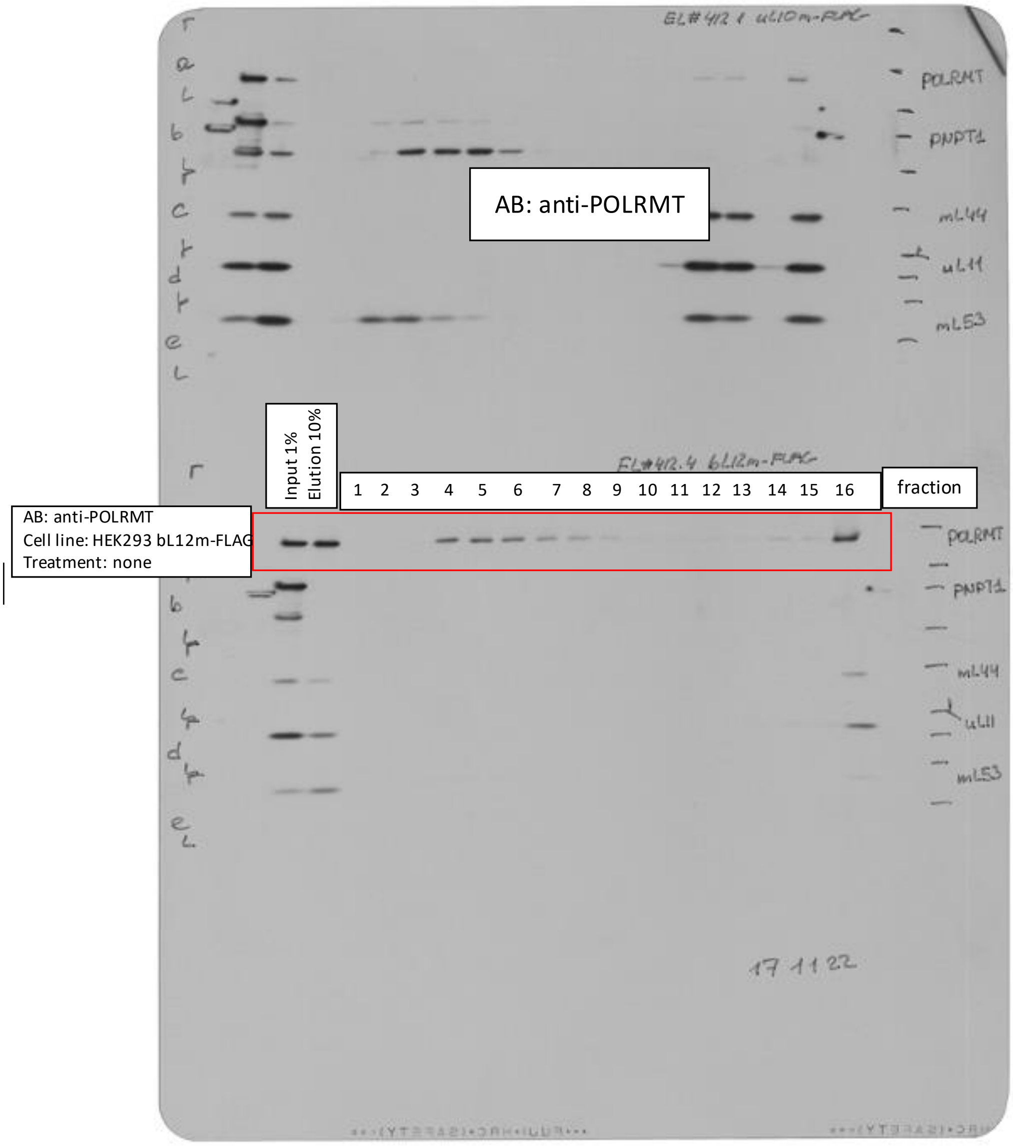

Source Data 4_related to Extended Data Fig.8b
EL#412.4-6 bL12m-FLAG IP + Gradient
Rotor: SW41 Ti
Gradient: Sucrose 5-30%
Speed: 158.000xg
Time: 15h

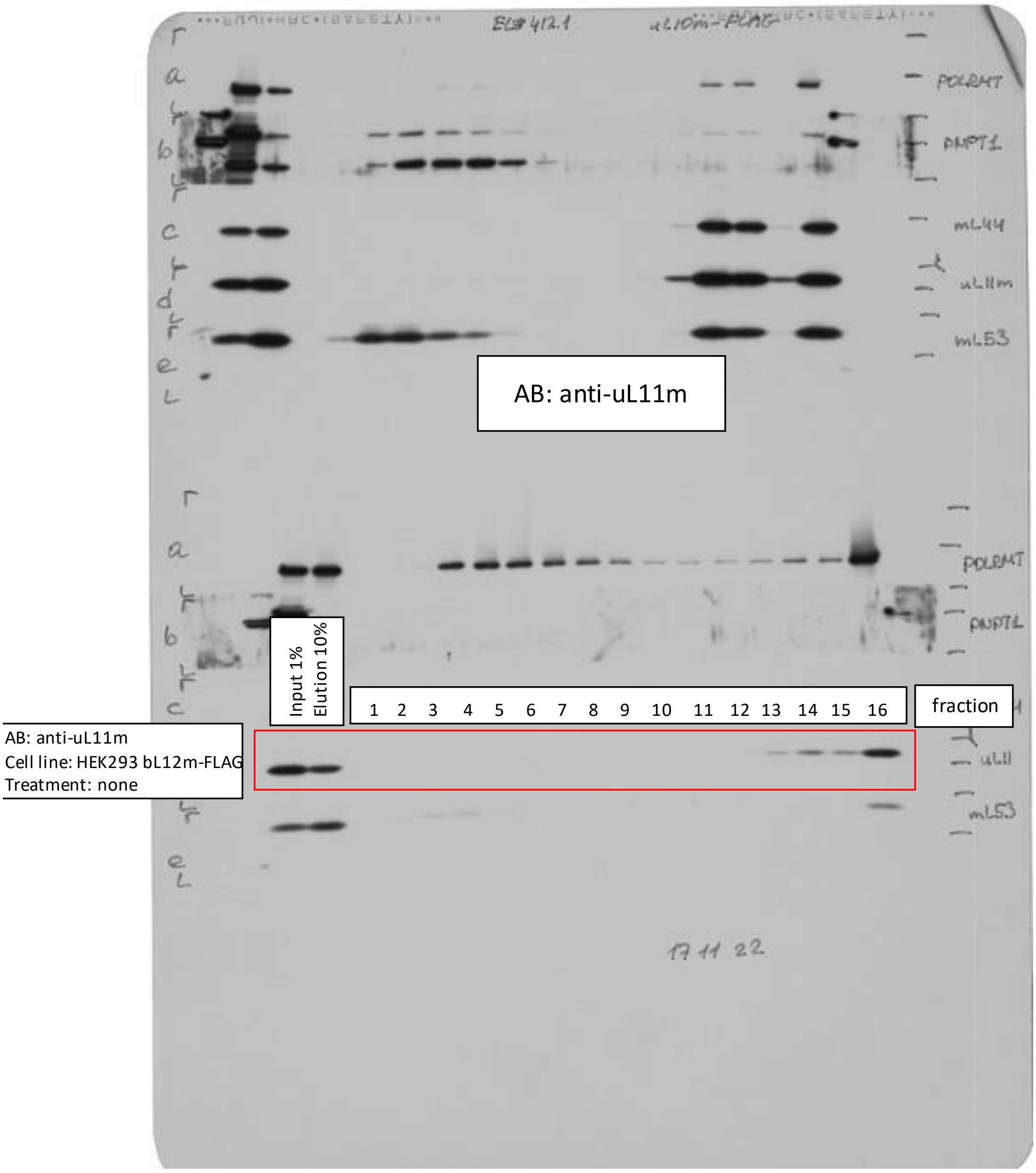

Source Data 4_related to Extended Data Fig.8b
EL#412.4-6 bL12m-FLAG IP + Gradient
Rotor: SW41 Ti
Gradient: Sucrose 5-30%
Speed: 158.000xg
Time: 15h

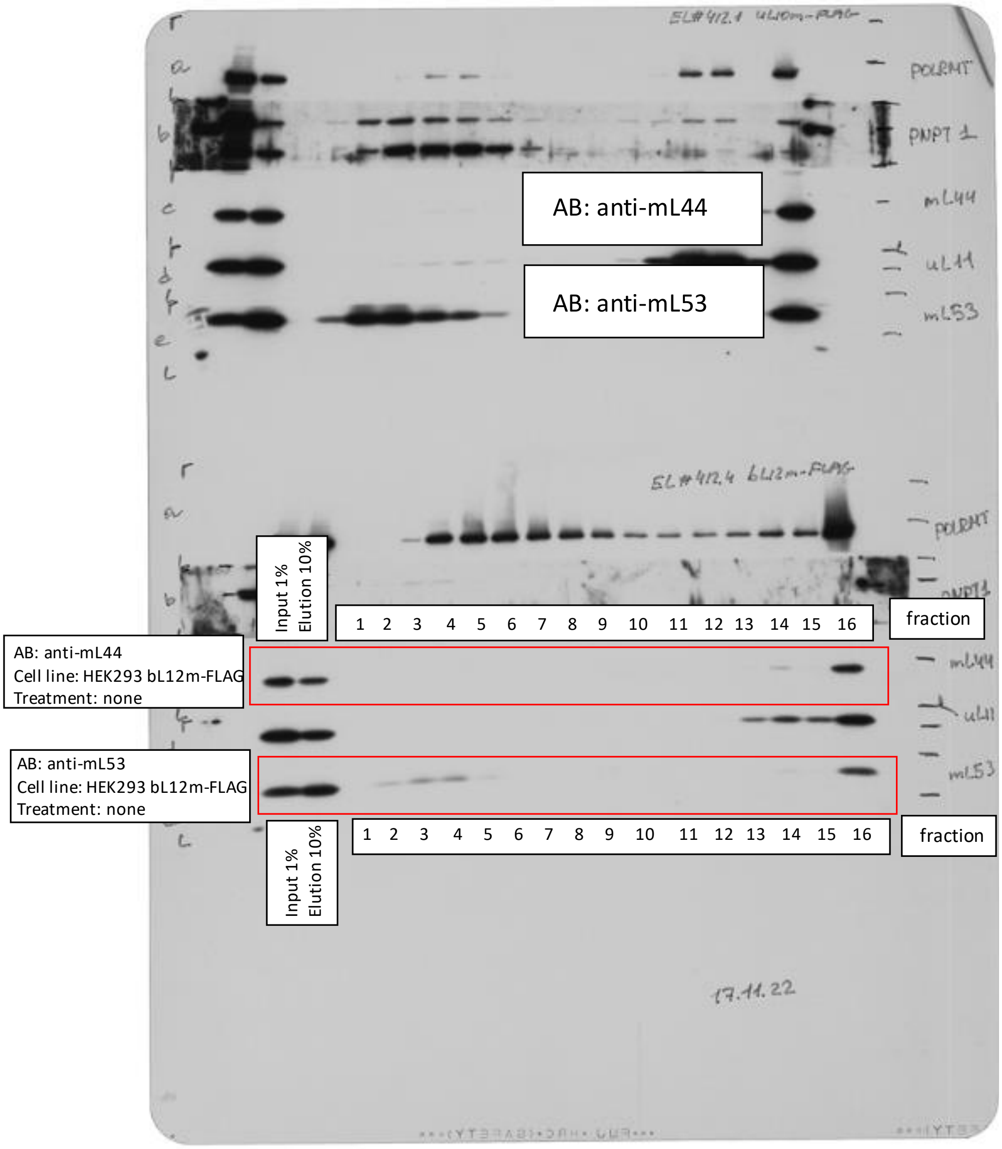

Source Data 4_related to Extended Data Fig.8b
EL#412.4-6 bL12m-FLAG IP + Gradient
Rotor: SW41 Ti
Gradient: Sucrose 5-30%
Speed: 158.000xg
Time: 15h

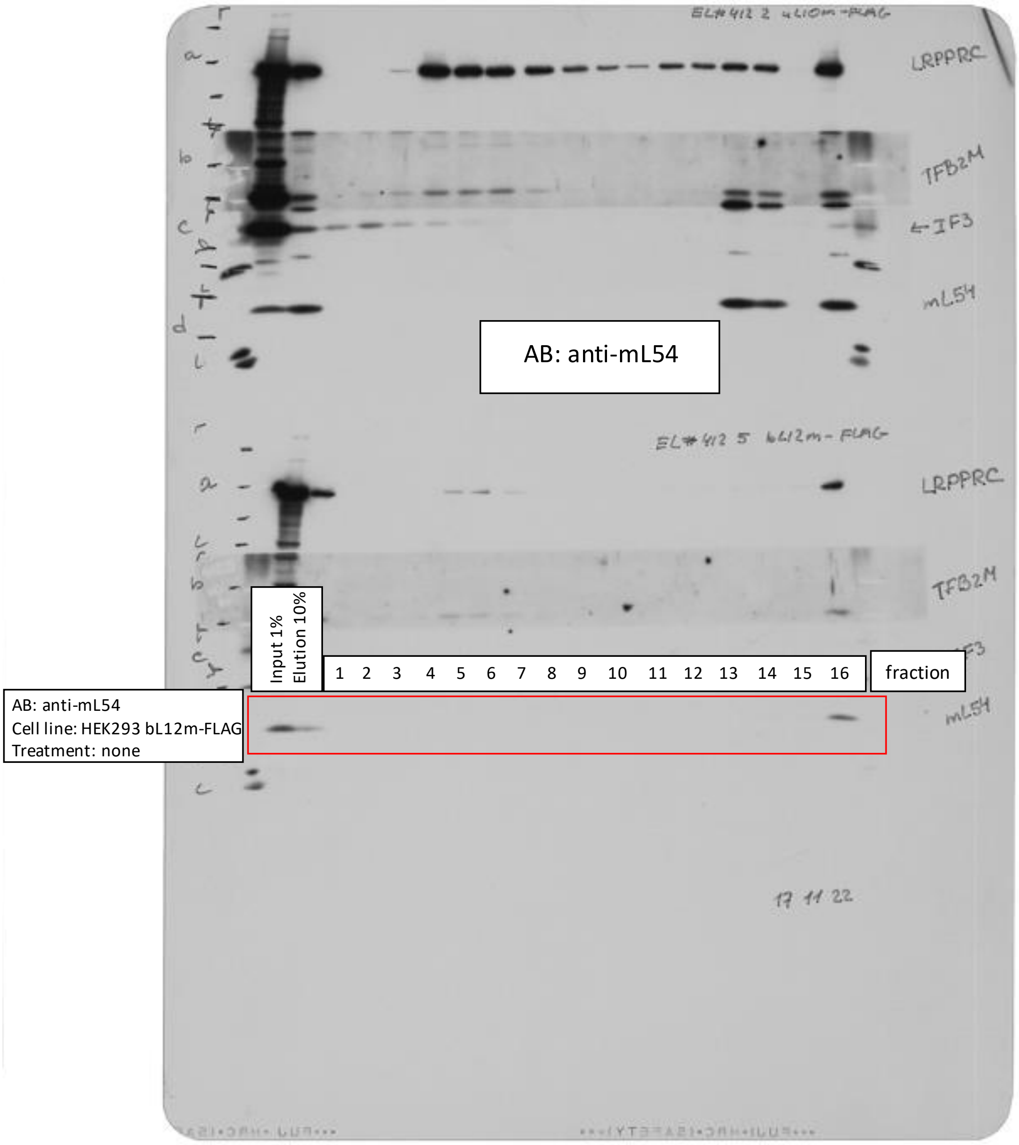

Source Data 4_related to Extended Data Fig.8c
EL#412.1-3 uL10m-FLAG IP + Gradient
Rotor: SW41 Ti
Gradient: Sucrose 5-30%
Speed: 158.000xg
Time: 15h

AB: anti-uL10m

AB: anti-uS5m

AB: anti-bL12m

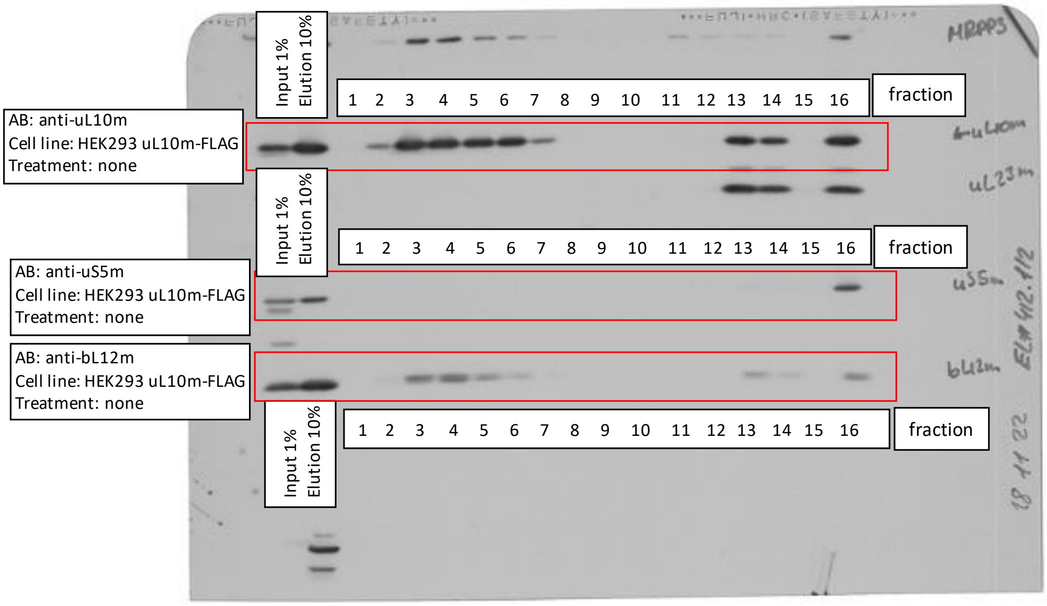

Source Data 4_related to Extended Data Fig.8c
EL#412.1-3 uL10m-FLAG IP + Gradient
Rotor: SW41 Ti
Gradient: Sucrose 5-30%
Speed: 158.000xg
Time: 15h

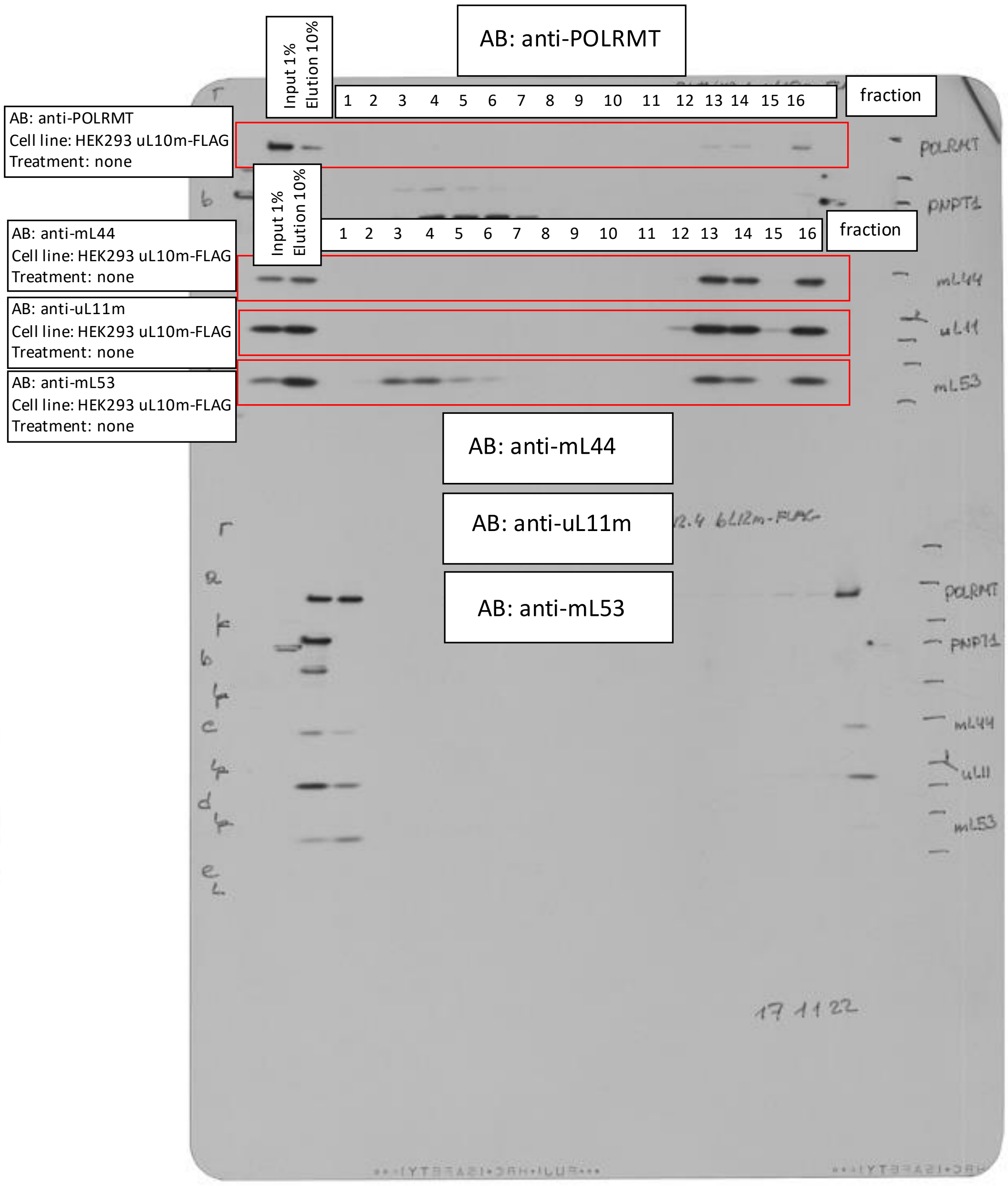

Source Data 4_related to Extended Data Fig.8c
EL#412.1-3 uL10m-FLAG IP + Gradient
Rotor: SW41 Ti
Gradient: Sucrose 5-30%
Speed: 158.000xg
Time: 15h

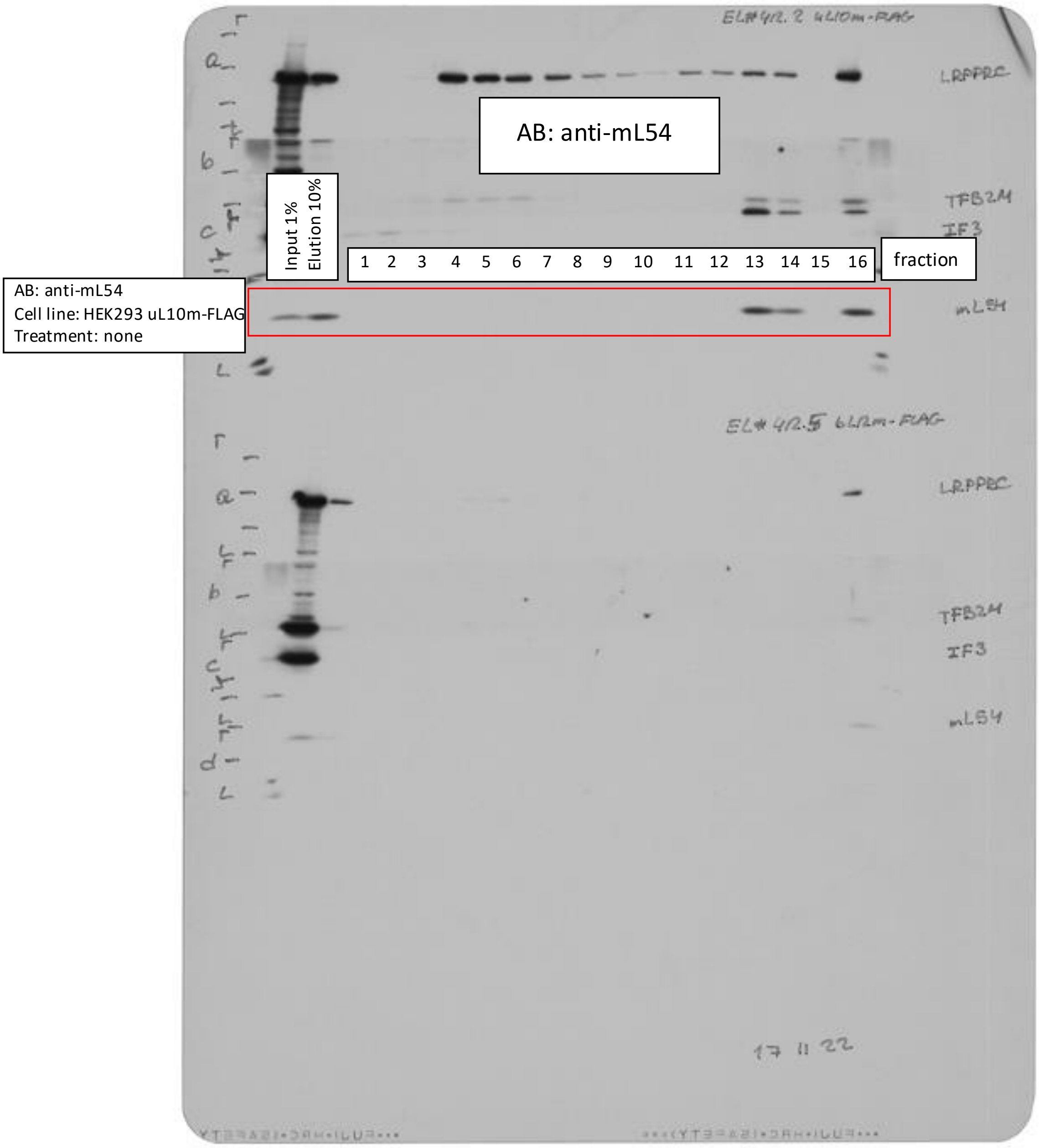

Source Data 4_related to Extended Data Fig.8e
EL#451 uL11m-FLAG IP + EtBr treatment
Rotor: N/A
Gradient: N/A
Speed: N/A
Time: N/A

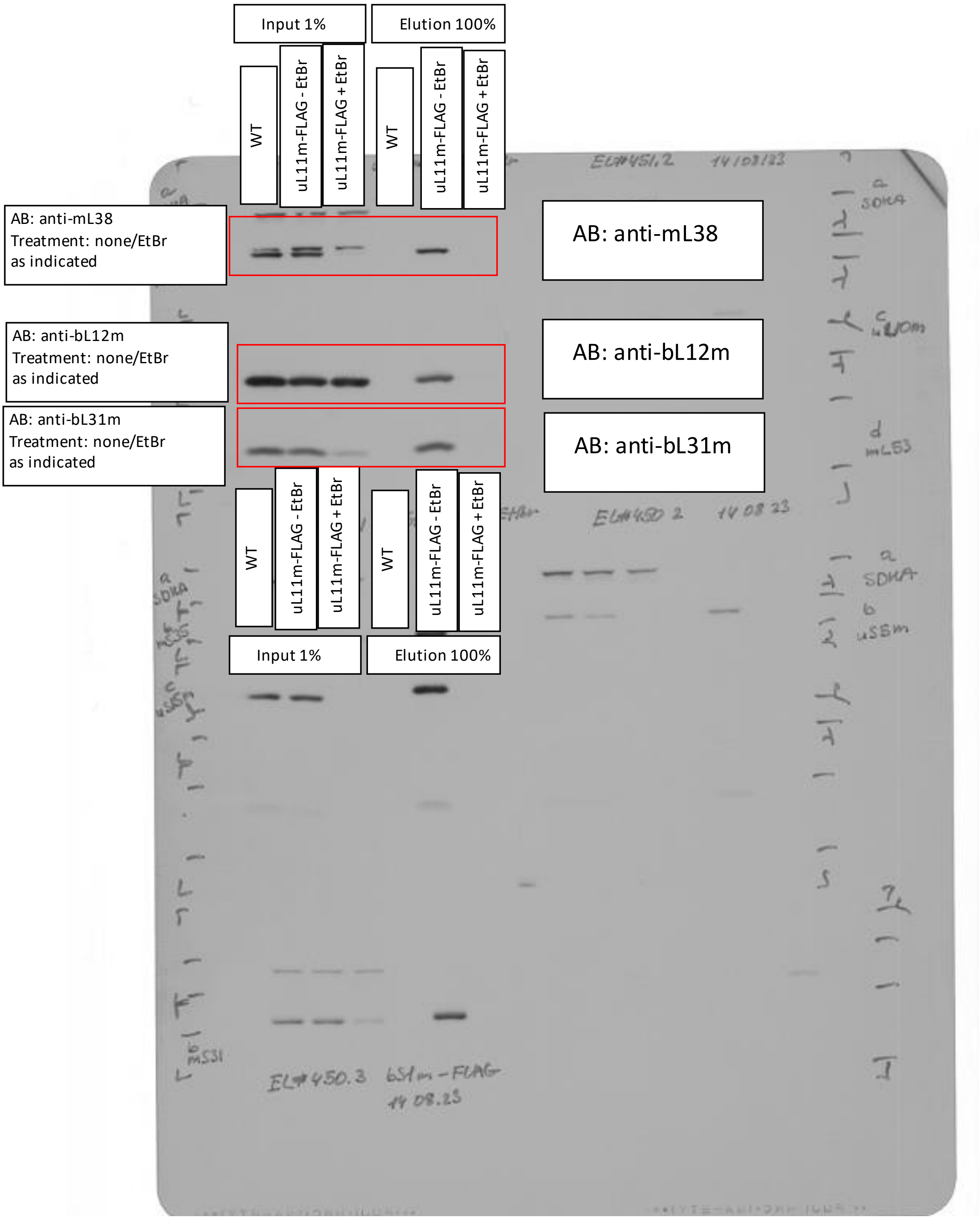

Source Data 4_related to Extended Data Fig.8e
EL#451 uL11m-FLAG IP + EtBr treatment
Rotor: N/A
Gradient: N/A
Speed: N/A
Time: N/A

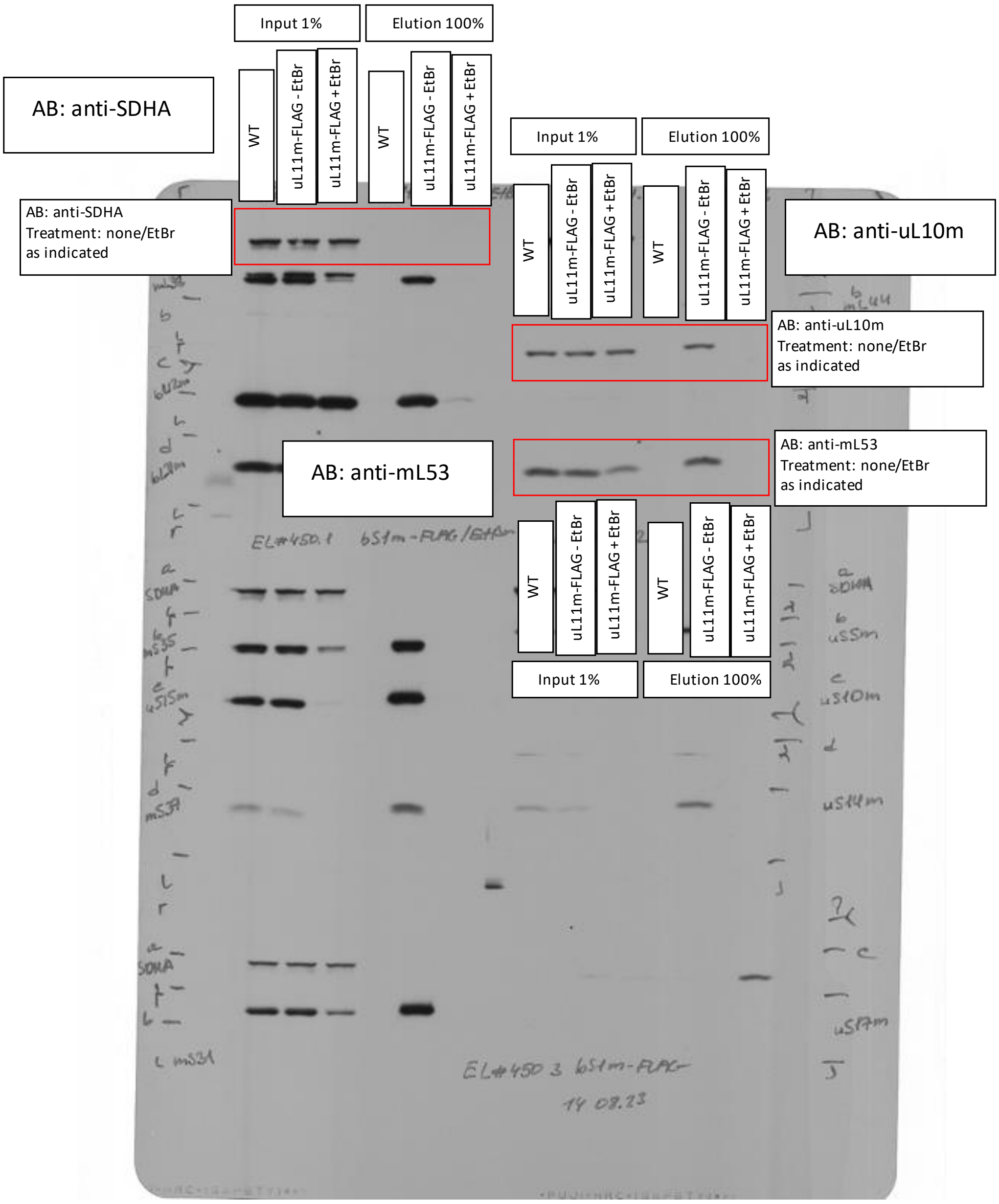

Source Data 4_related to Extended Data Fig.8e
EL#451 uL11m-FLAG IP + EtBr treatment
Rotor: N/A
Gradient: N/A
Speed: N/A
Time: N/A

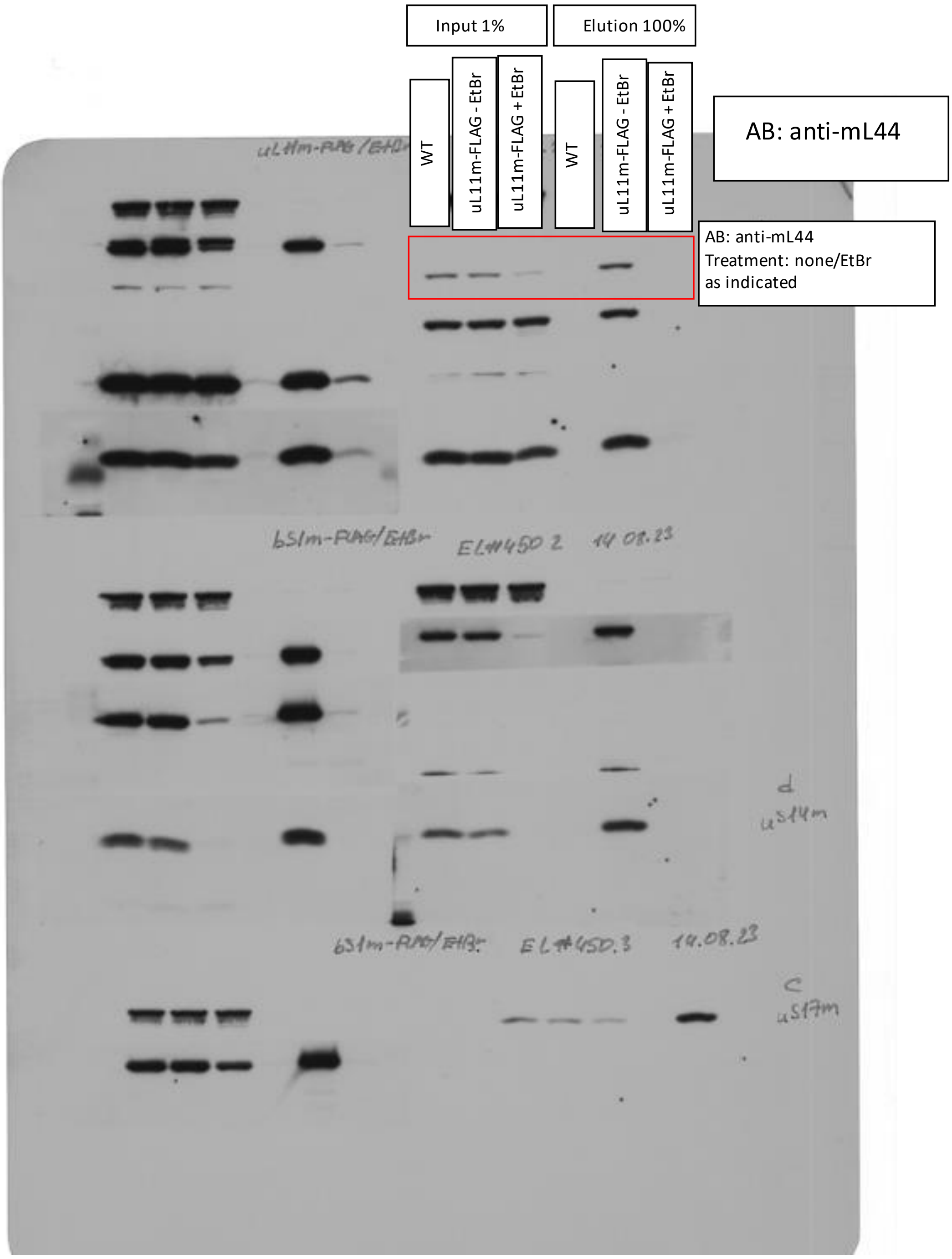

Source Data 4_related to Extended Data Fig.8e
EL#451 uL11m-FLAG IP + EtBr treatment
Rotor: N/A
Gradient: N/A
Speed: N/A
Time: N/A

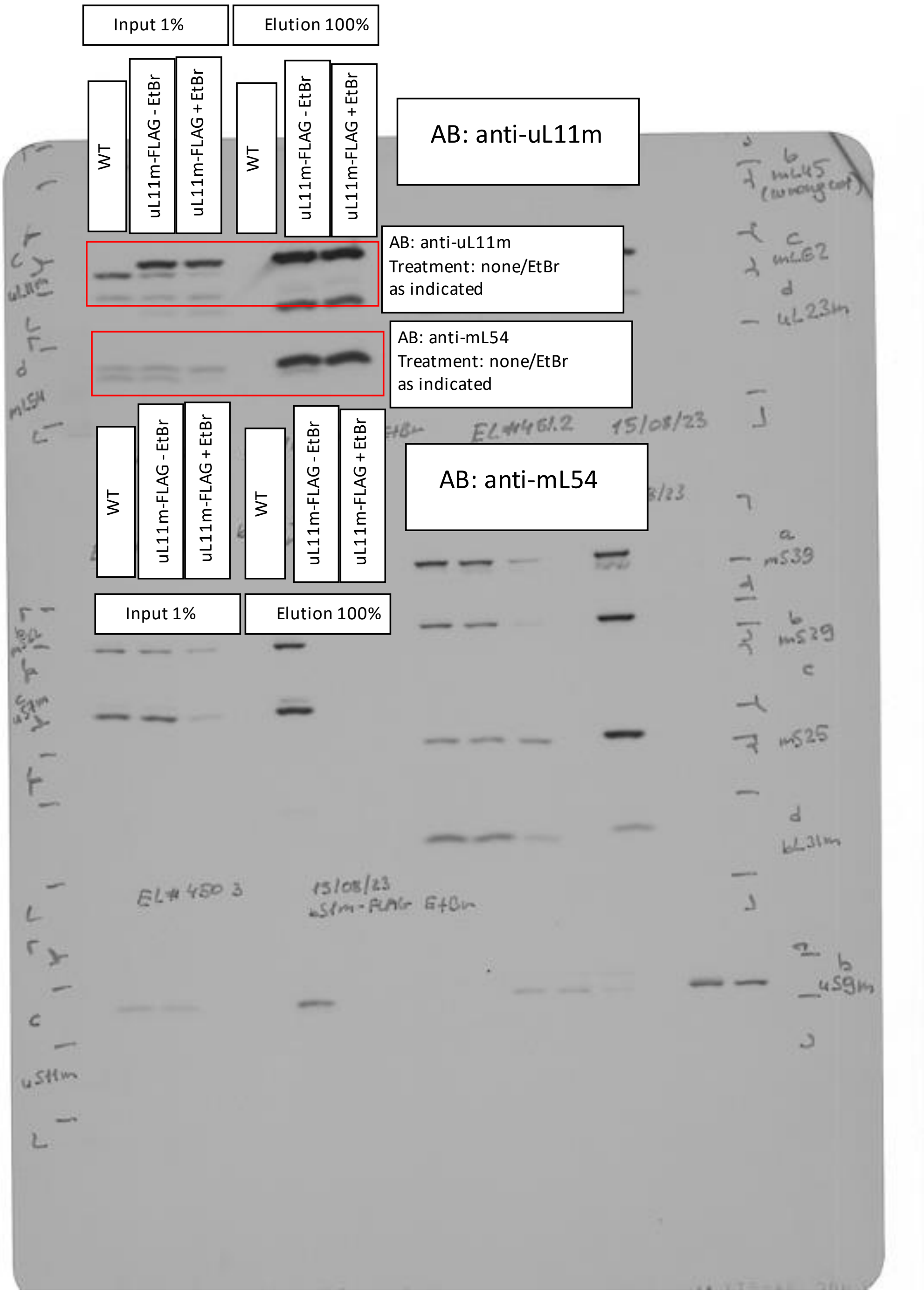

Source Data 4_related to Extended Data Fig.8e
EL#451 uL11m-FLAG IP + EtBr treatment
Rotor: N/A
Gradient: N/A
Speed: N/A
Time: N/A

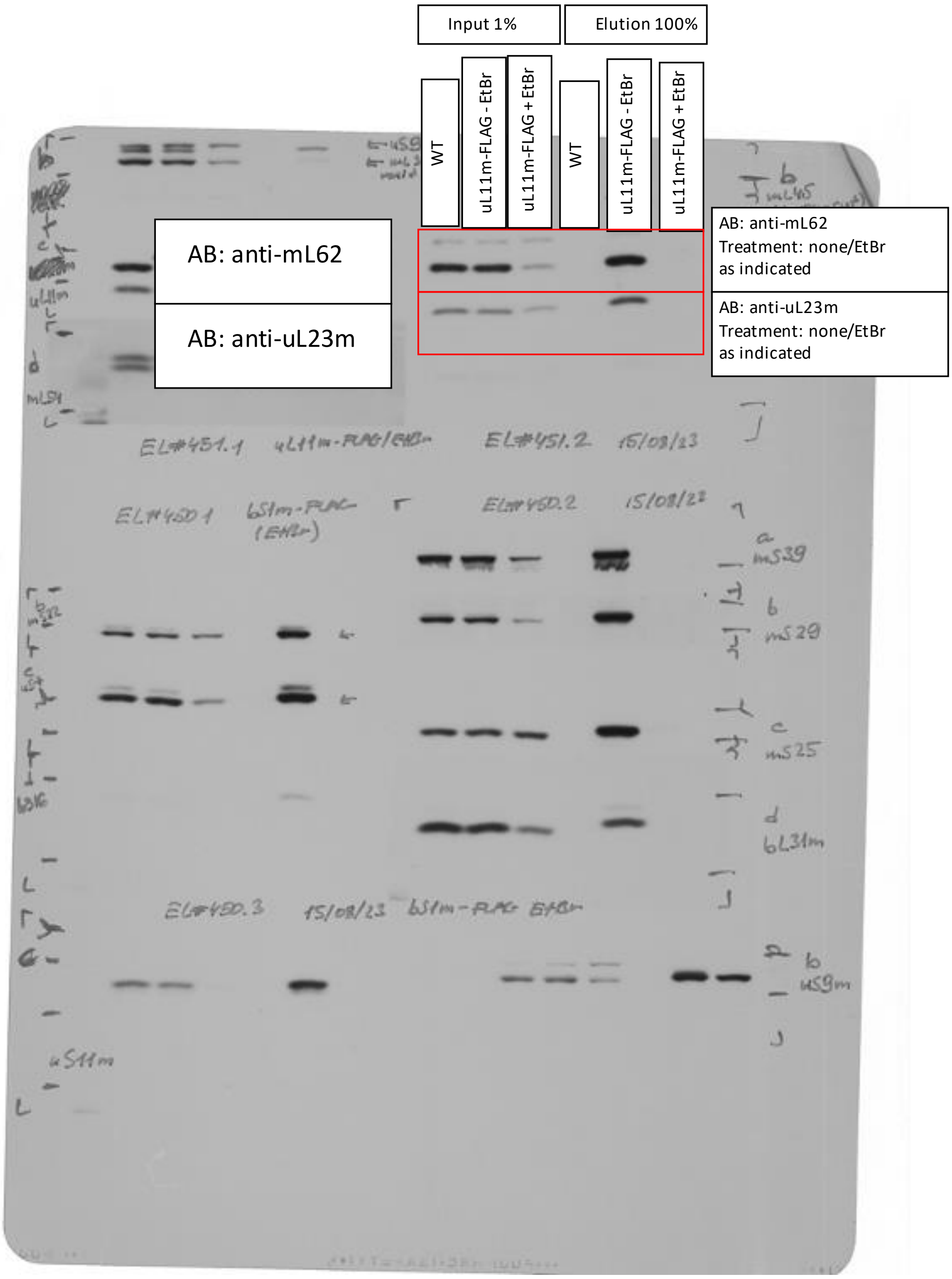

Source Data 4_related to Extended Data Fig.8e
EL#451 uL11m-FLAG IP + EtBr treatment
Rotor: N/A
Gradient: N/A
Speed: N/A
Time: N/A

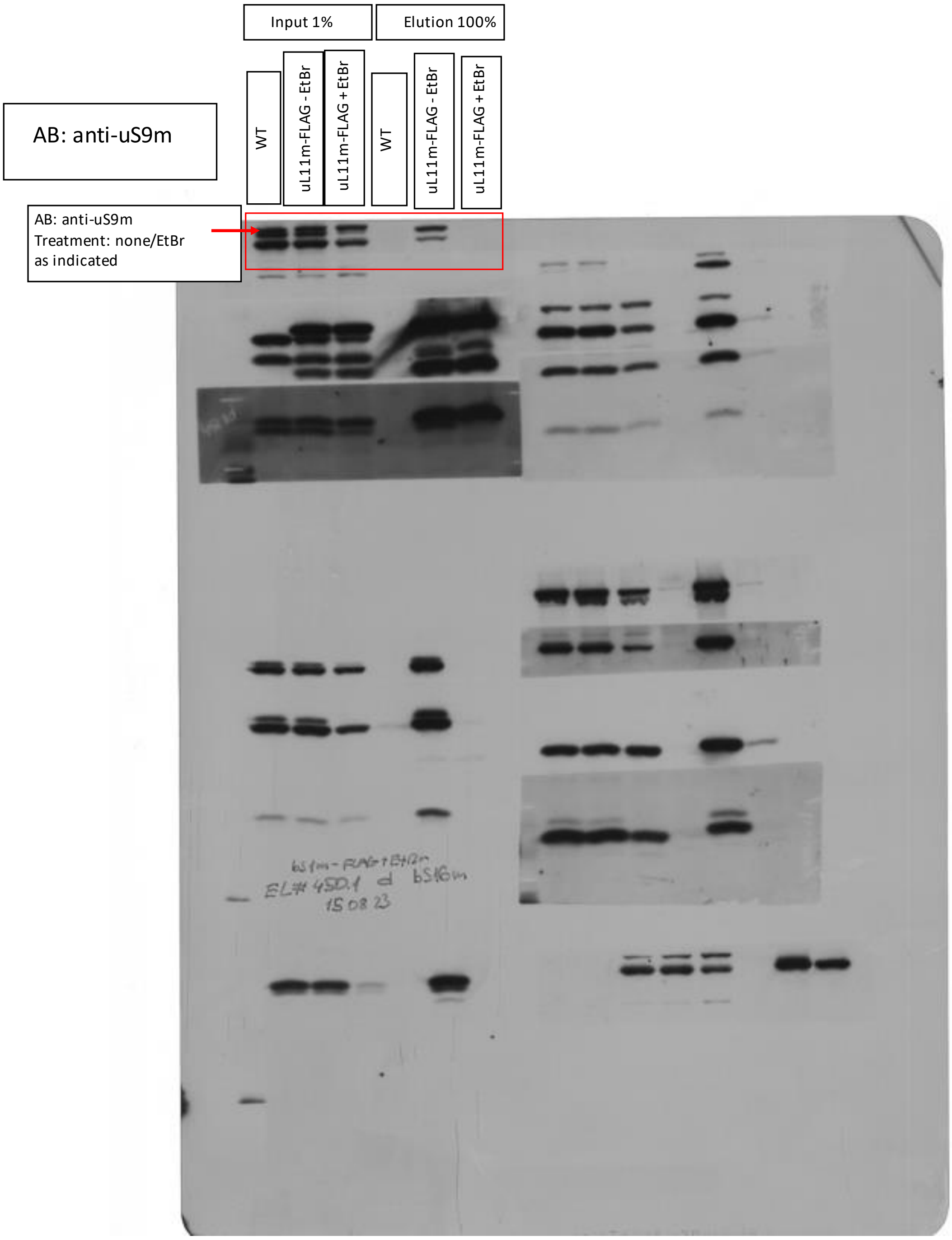

Source Data 4_related to Extended Data Fig.8d
EL#446/MH#200 bL12m-FLAG IP + EtBr treatment
Rotor: N/A
Gradient: N/A
Speed: N/A
Time: N/A

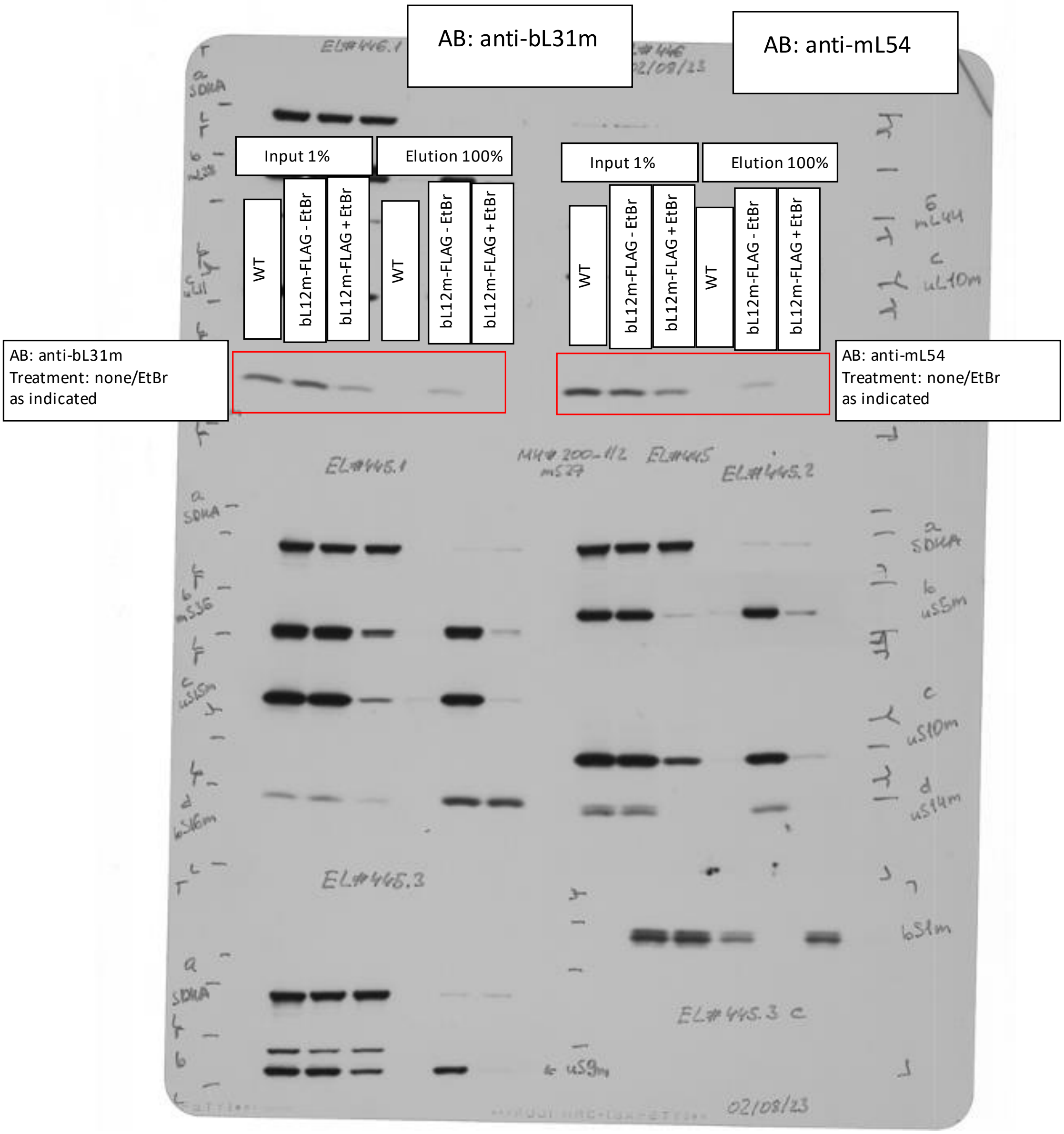

Source Data 4_related to Extended Data Fig.8d
EL#446/MH#200 bL12m-FLAG IP + EtBr treatment
Rotor: N/A
Gradient: N/A
Speed: N/A
Time: N/A

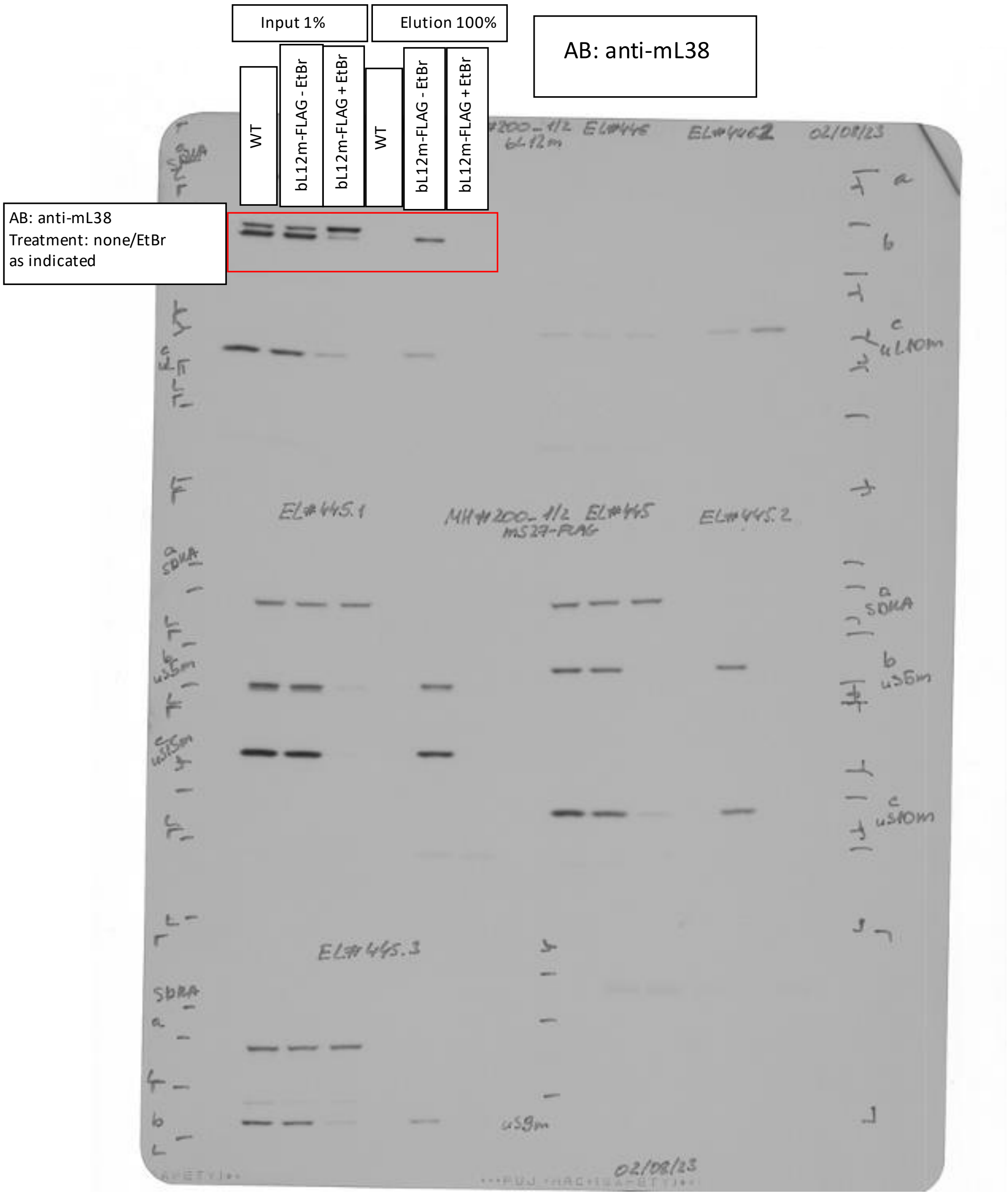

Source Data 4_related to Extended Data Fig.8d
EL#446/MH#200 bL12m-FLAG IP + EtBr treatment
Rotor: N/A
Gradient: N/A
Speed: N/A
Time: N/A

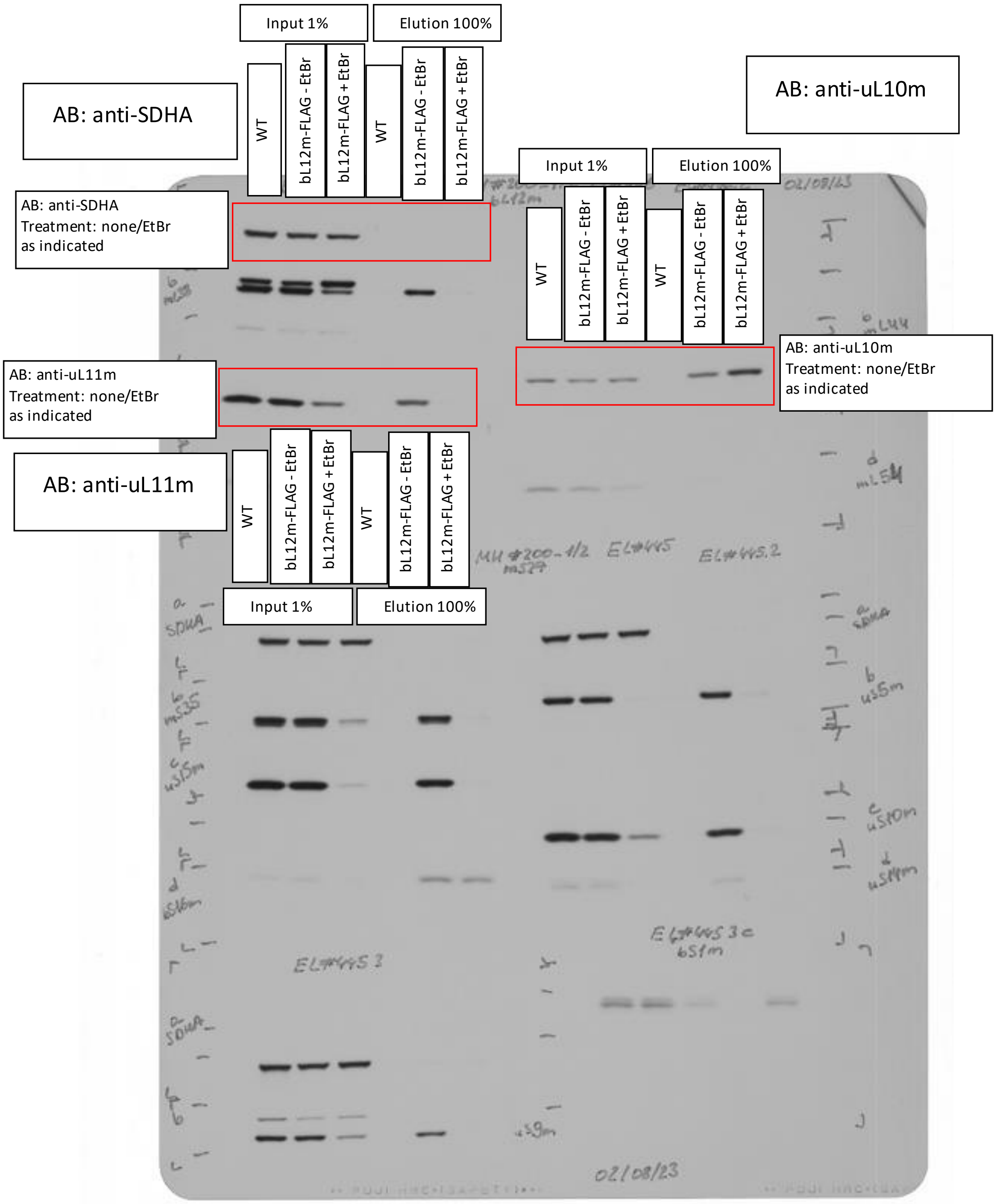

Source Data 4_related to Extended Data Fig.8d
EL#446/MH#200 bL12m-FLAG IP + EtBr treatment
Rotor: N/A
Gradient: N/A
Speed: N/A
Time: N/A

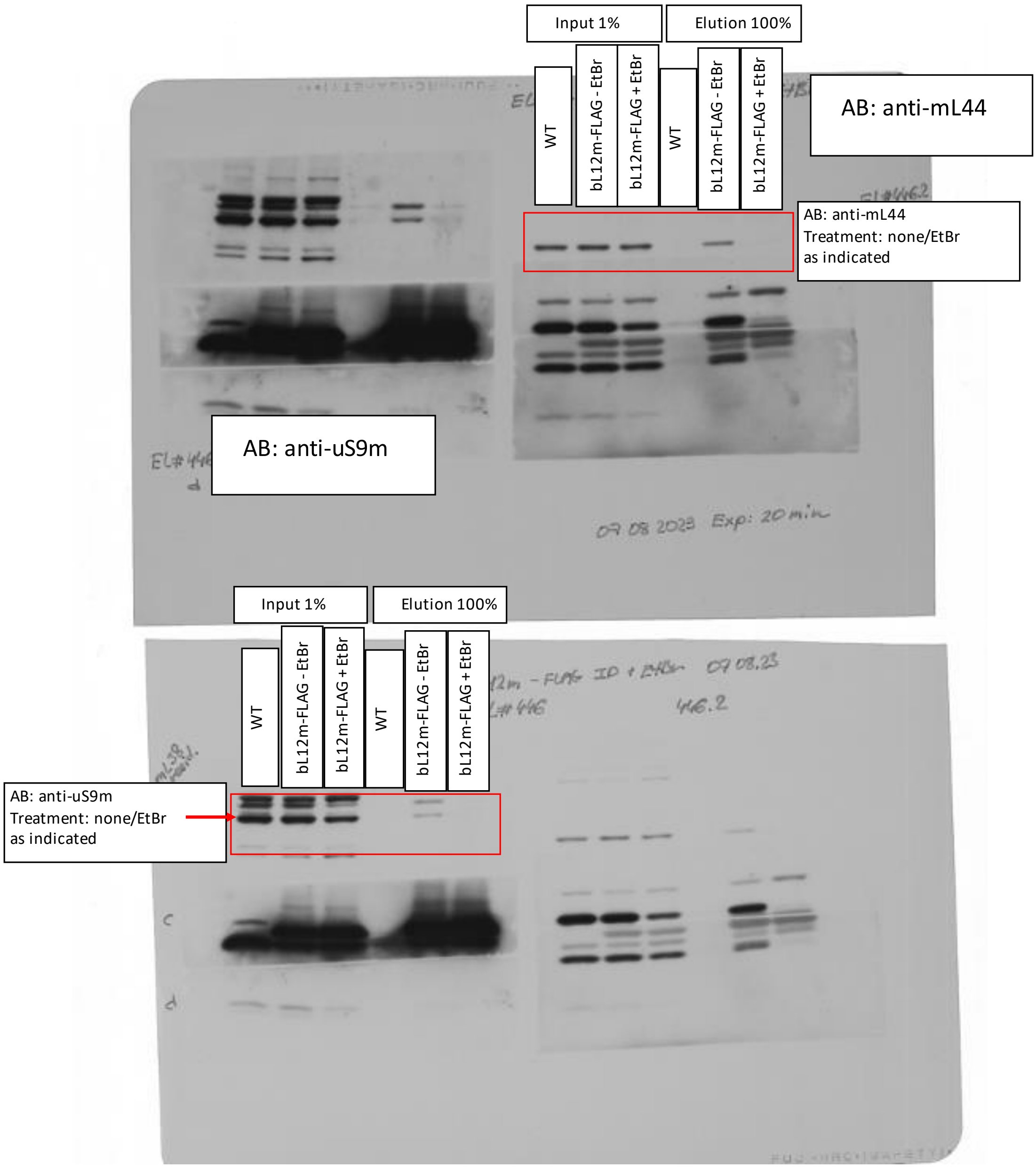

Source Data 4_related to Extended Data Fig.8d
EL#446/MH#200 bL12m-FLAG IP + EtBr treatment
Rotor: N/A
Gradient: N/A
Speed: N/A
Time: N/A

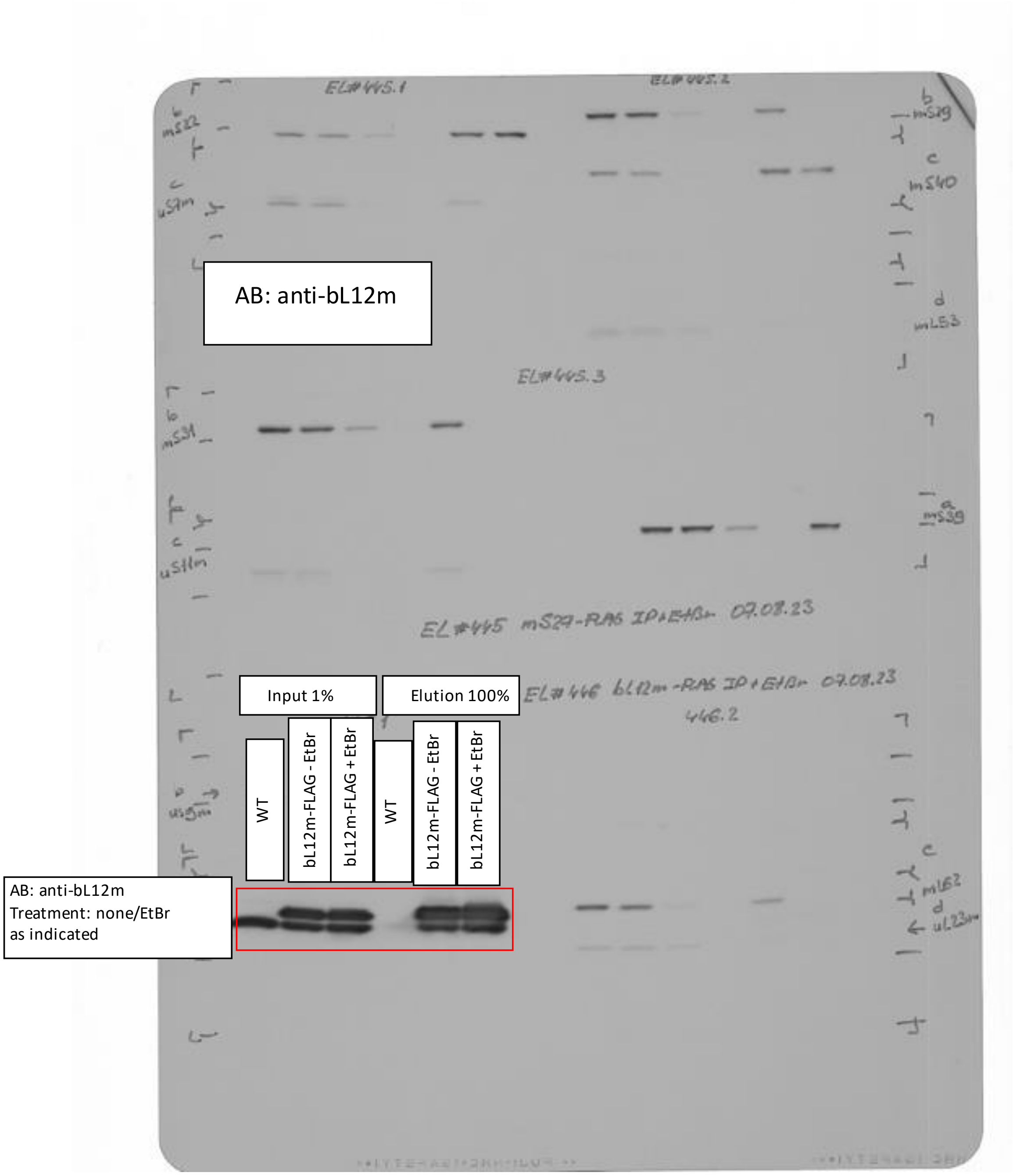

Source Data 4_related to Extended Data Fig.8d
EL#446/MH#200 bL12m-FLAG IP + EtBr treatment
Rotor: N/A
Gradient: N/A
Speed: N/A
Time: N/A

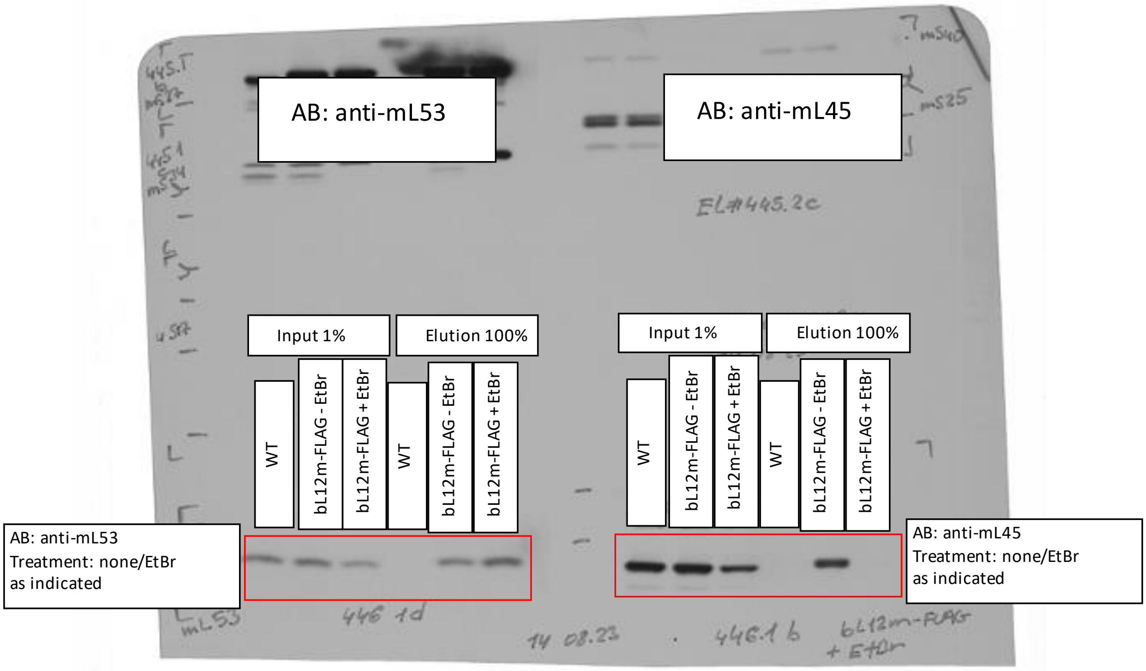

Source Data 4_related to Extended Data Fig.8d
EL#446/MH#200 bL12m-FLAG IP + EtBr treatment
Rotor: N/A
Gradient: N/A
Speed: N/A
Time: N/A

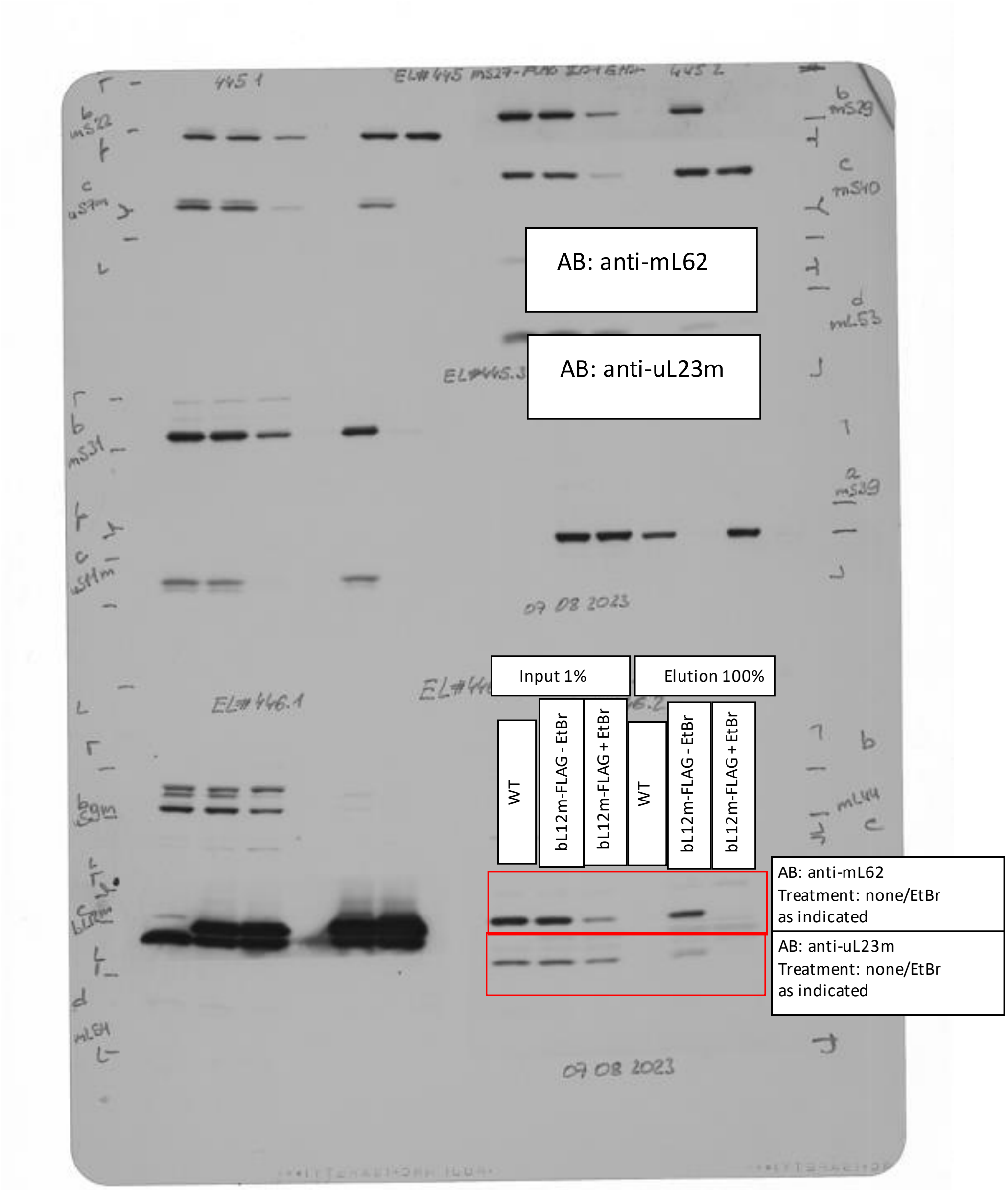

Source Data 4_related to Extended Data Fig.8f
EL#363 HEK293 WT + Ethidium Bromide treatment + Gradient
EL#379 HEK293 WT + Ethidium Bromide treatment + Gradient

Rotor: SW41 Ti
Gradient: Sucrose 5-30%
Speed: 158.000xg
Time: 15h

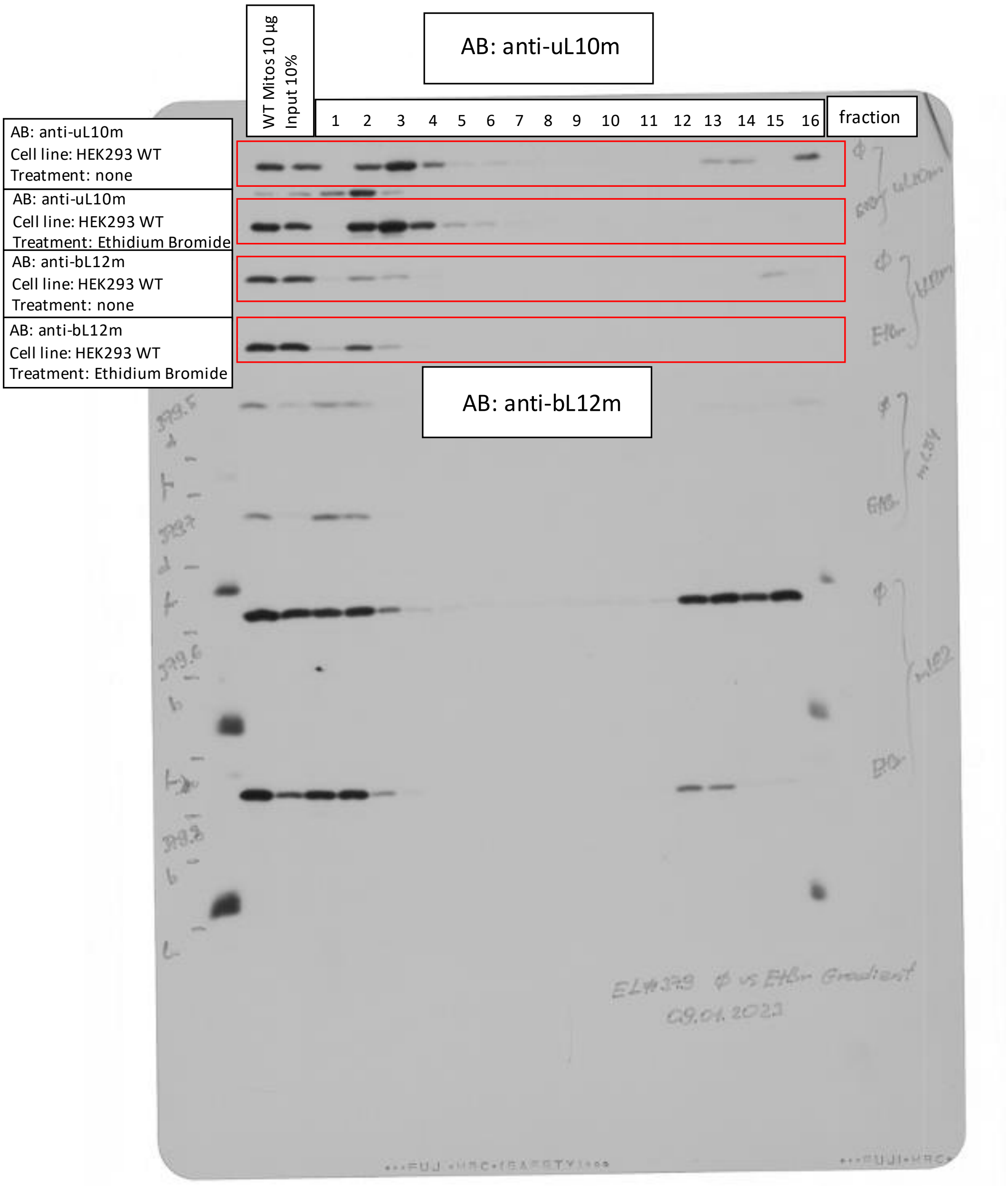

Source Data 4_related to Extended Data Fig.8f
EL#363 HEK293 WT + Ethidium Bromide treatment + Gradient
EL#379 HEK293 WT + Ethidium Bromide treatment + Gradient

Rotor: SW41 Ti
Gradient: Sucrose 5-30%
Speed: 158.000xg
Time: 15h

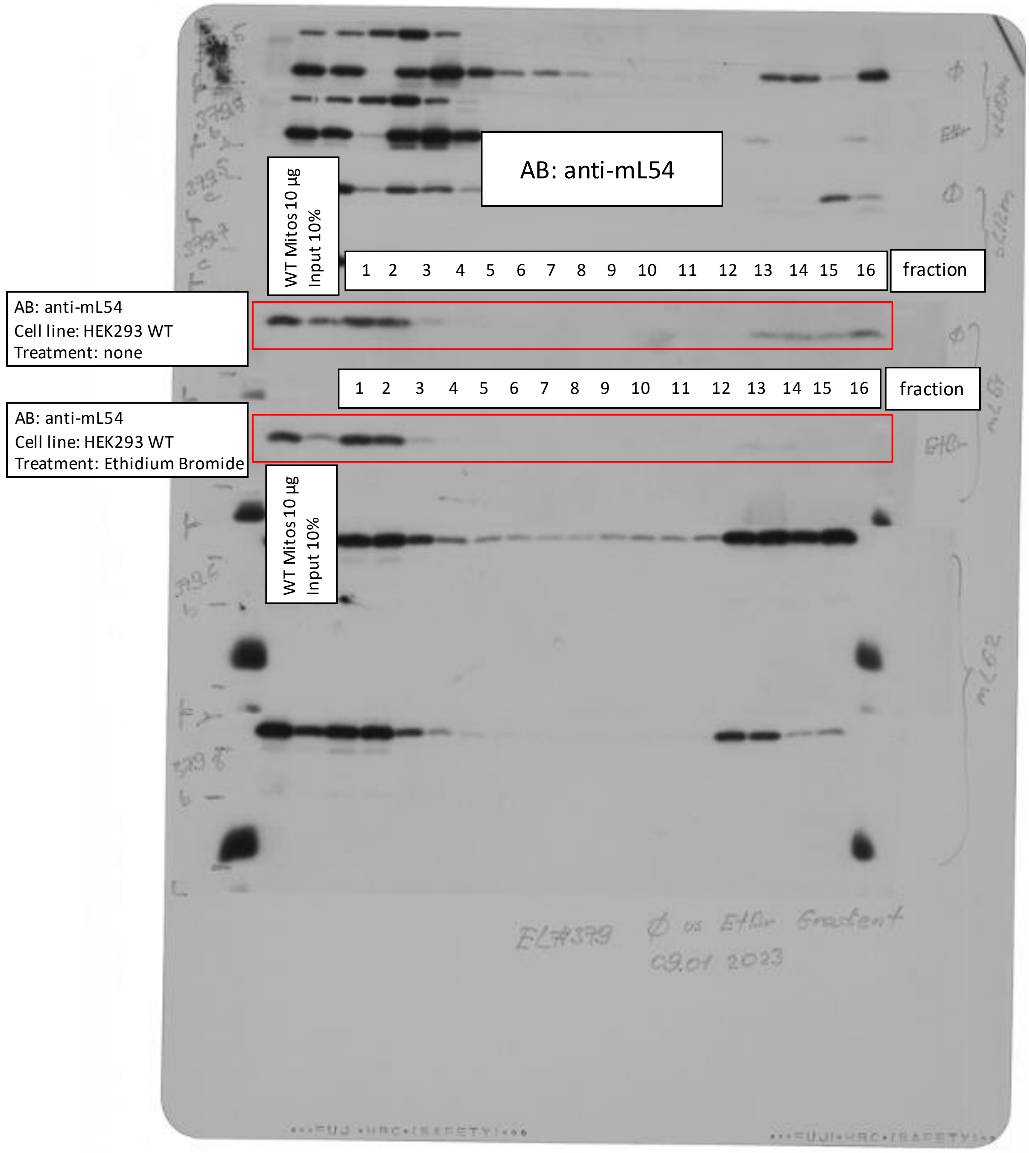

Source Data 4_related to Extended Data Fig.8f
EL#363 HEK293 WT + Ethidium Bromide treatment + Gradient
EL#379 HEK293 WT + Ethidium Bromide treatment + Gradient

Rotor: SW41 Ti
Gradient: Sucrose 5-30%
Speed: 158.000xg
Time: 15h

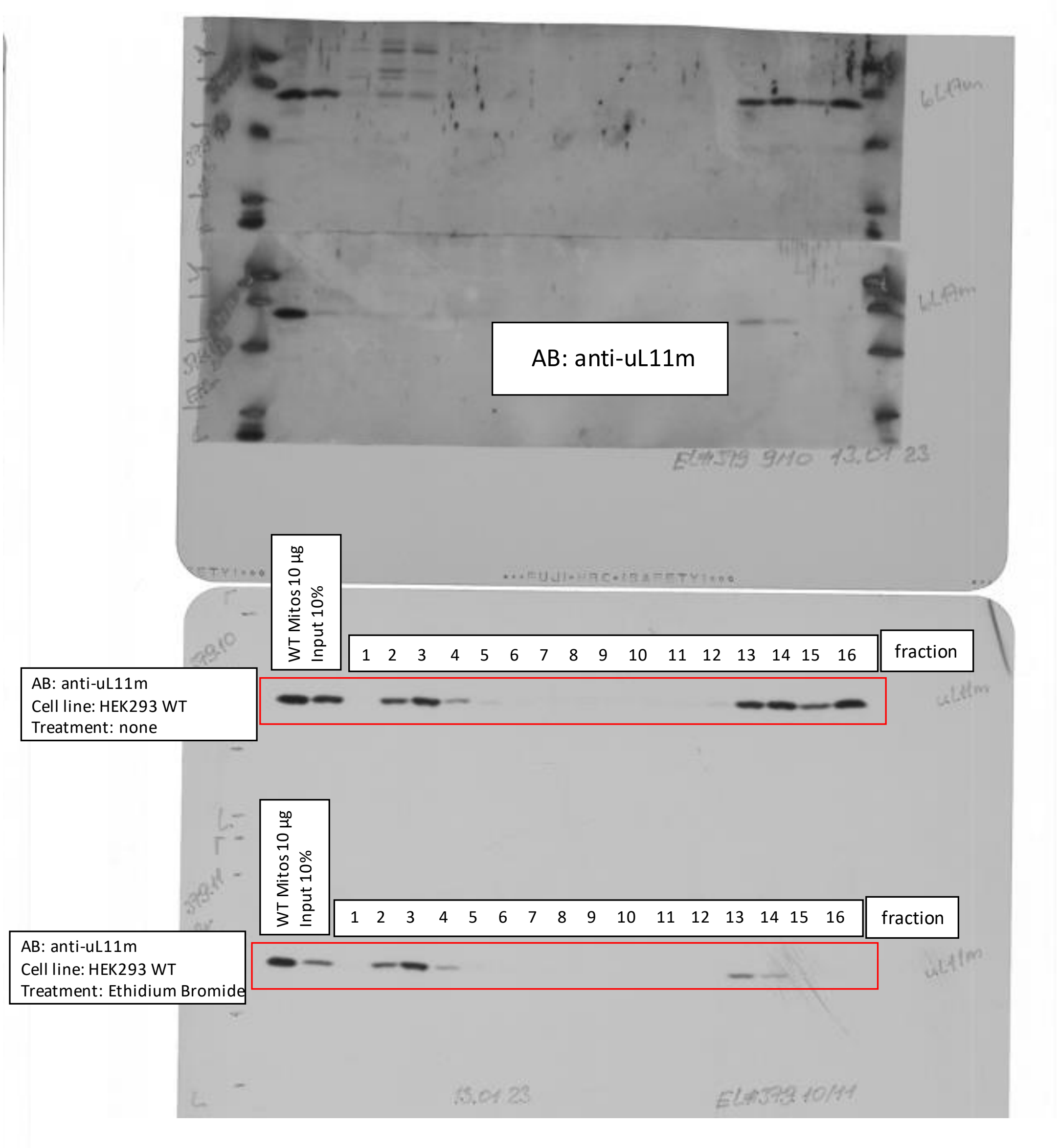

Source Data 4_related to Extended Data Fig.8f
EL#363 HEK293 WT + Ethidium Bromide treatment + Gradient
EL#379 HEK293 WT + Ethidium Bromide treatment + Gradient

Rotor: SW41 Ti
Gradient: Sucrose 5-30%
Speed: 158.000xg
Time: 15h

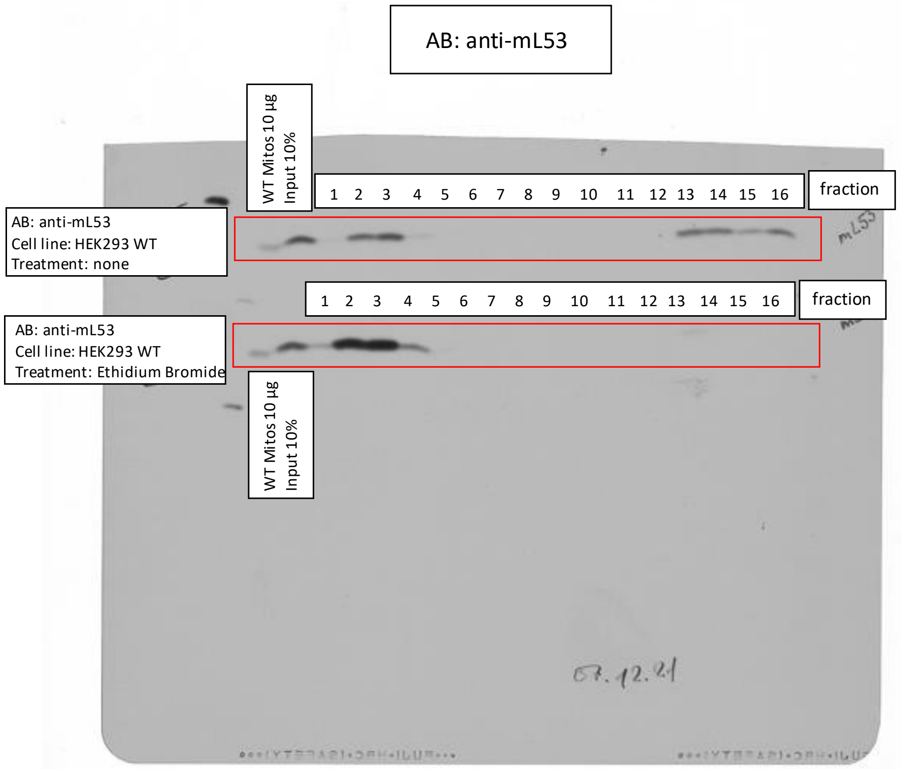

Supplement: Supplementary file 25 — Unprocessed blots. [file 41594_2024_1356_MOESM25_ESM.pdf]
