# Supplementary material for: A roadmap for ribosome assembly in human mitochondria
Source: Nat Struct Mol Biol. 2024 Jul 11;31(12):1898–908. doi: 10.1038/s41594-024-01356-w (PMC11638073; doi:10.1038/s41594-024-01356-w)

Source Data 5_related to Extended Data Fig.9a
EL#353 mL44-FLAG IP + Gradient
Rotor: SW41 Ti
Gradient: Sucrose 5-30%
Speed: 158.000xg
Time: 15h

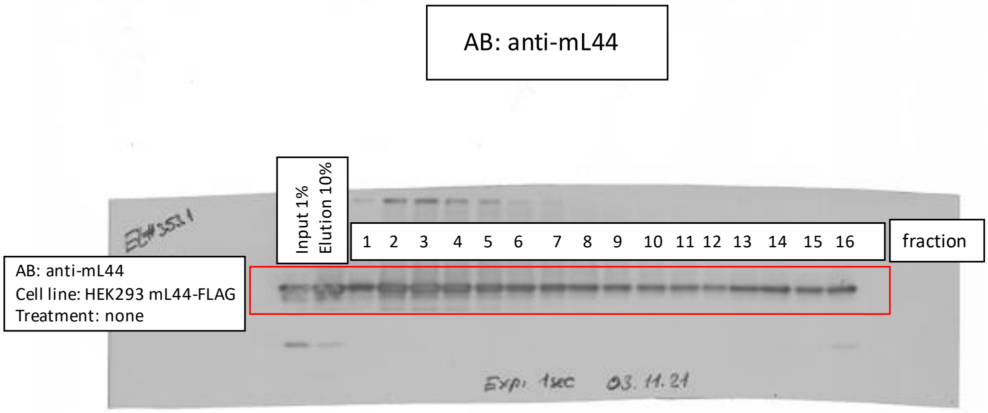

Source Data 5_related to Extended Data Fig.9a
EL#353 mL44-FLAG IP + Gradient
Rotor: SW41 Ti
Gradient: Sucrose 5-30%
Speed: 158.000xg
Time: 15h

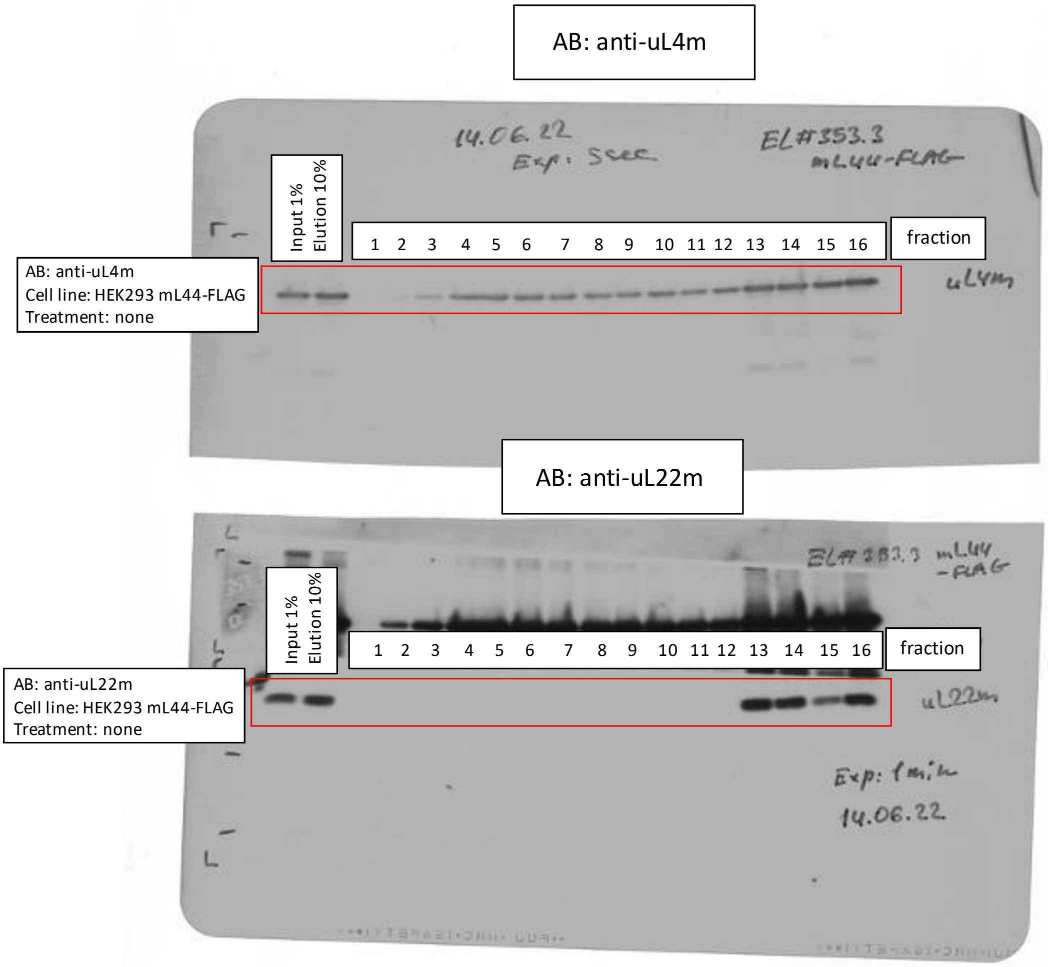

Source Data 5_related to Extended Data Fig.9a
EL#353 mL44-FLAG IP + Gradient
Rotor: SW41 Ti
Gradient: Sucrose 5-30%
Speed: 158.000xg
Time: 15h

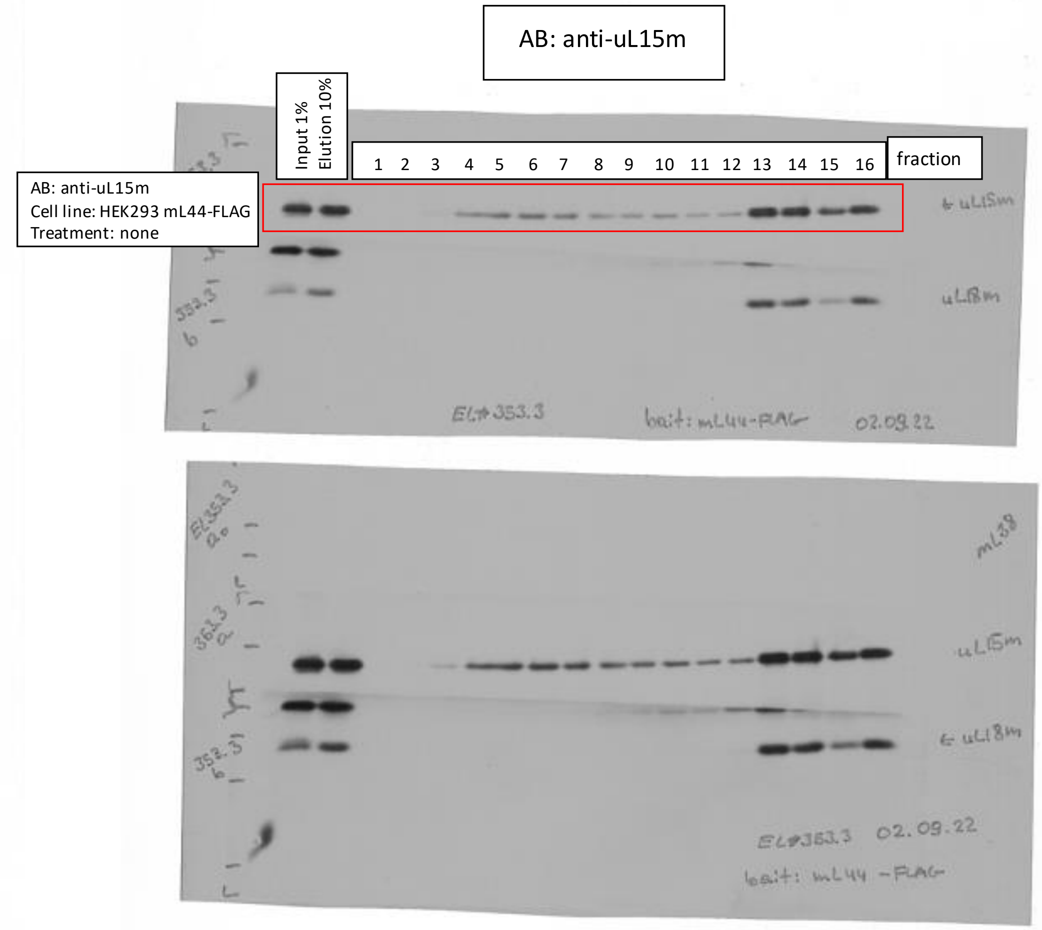

Source Data 5_related to Extended Data Fig.9a
EL#353 mL44-FLAG IP + Gradient
Rotor: SW41 Ti
Gradient: Sucrose 5-30%
Speed: 158.000xg
Time: 15h

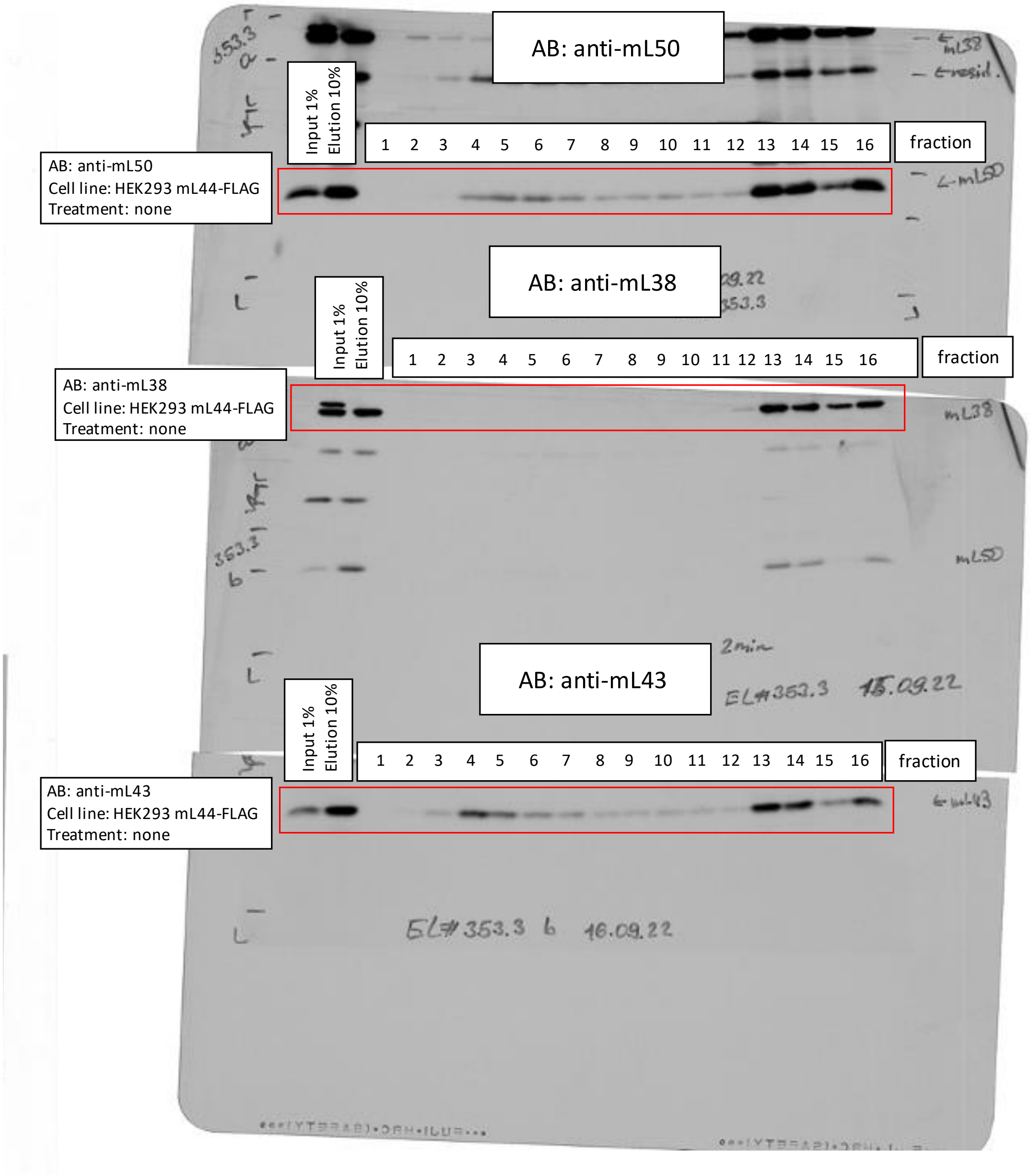

Source Data 5_related to Extended Data Fig.9a
EL#353 mL44-FLAG IP + Gradient
Rotor: SW41 Ti
Gradient: Sucrose 5-30%
Speed: 158.000xg
Time: 15h

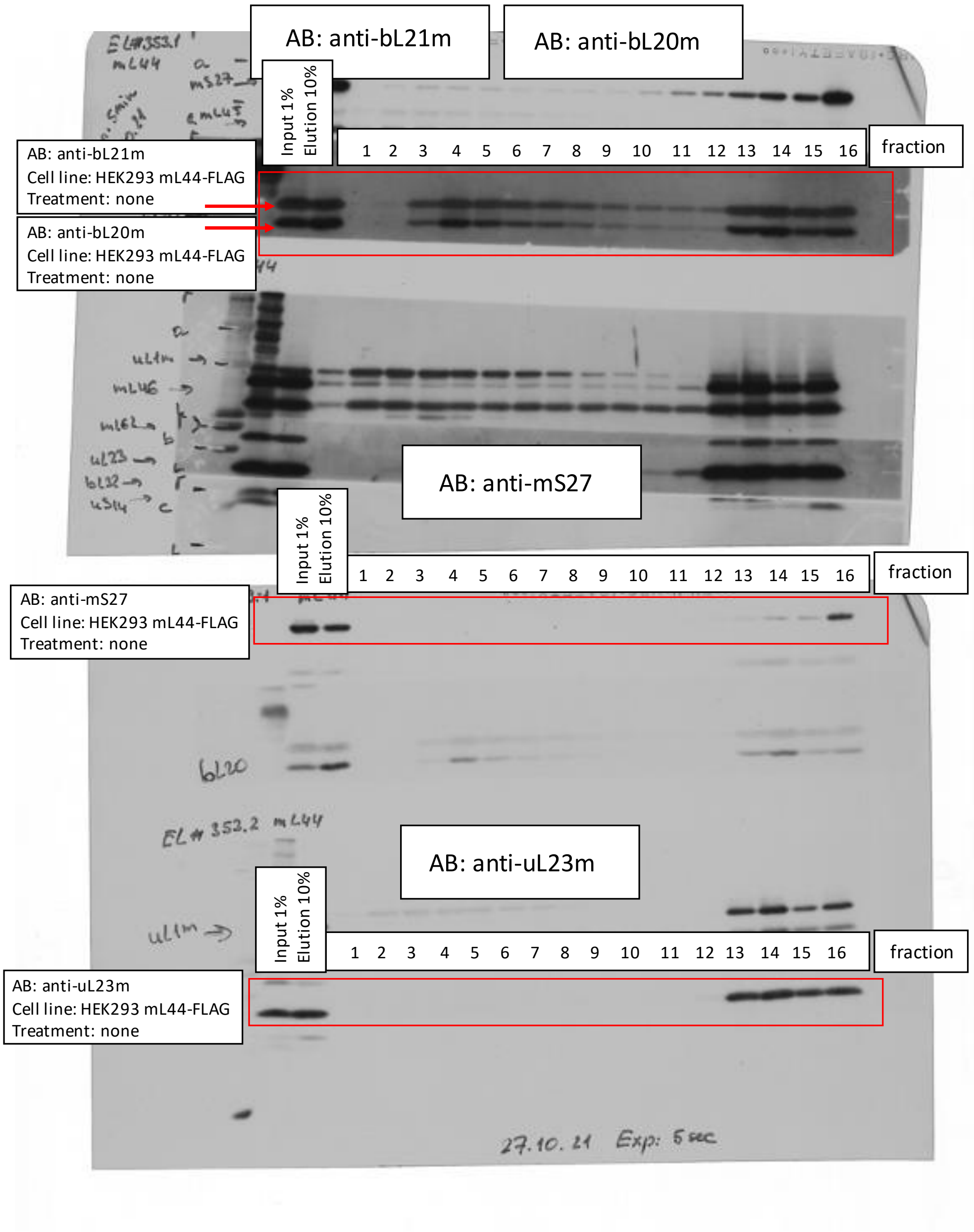

Source Data 5_related to Extended Data Fig.9a
EL#353 mL44-FLAG IP + Gradient
Rotor: SW41 Ti
Gradient: Sucrose 5-30%
Speed: 158.000xg
Time: 15h

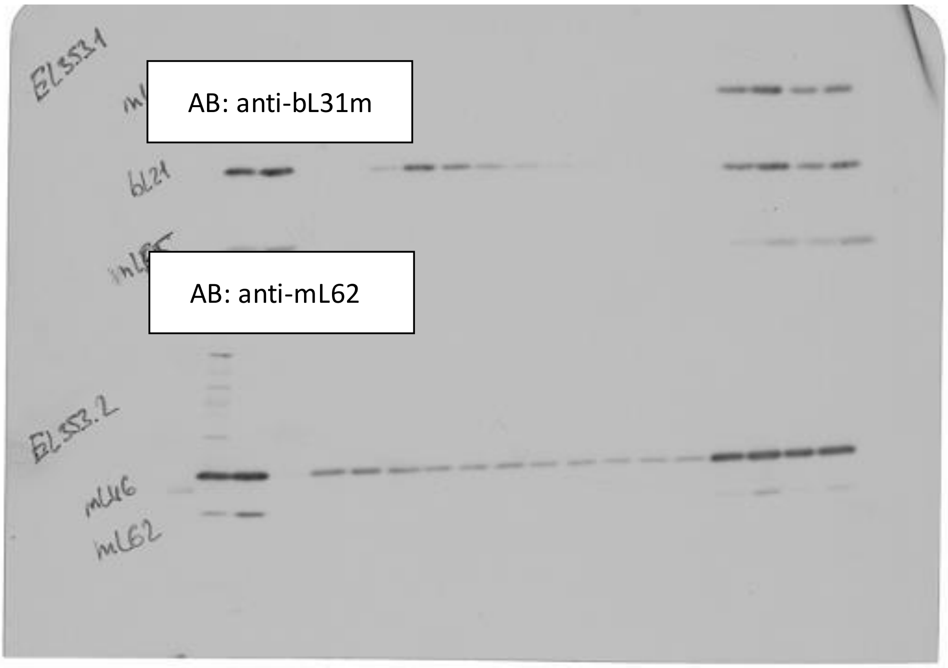

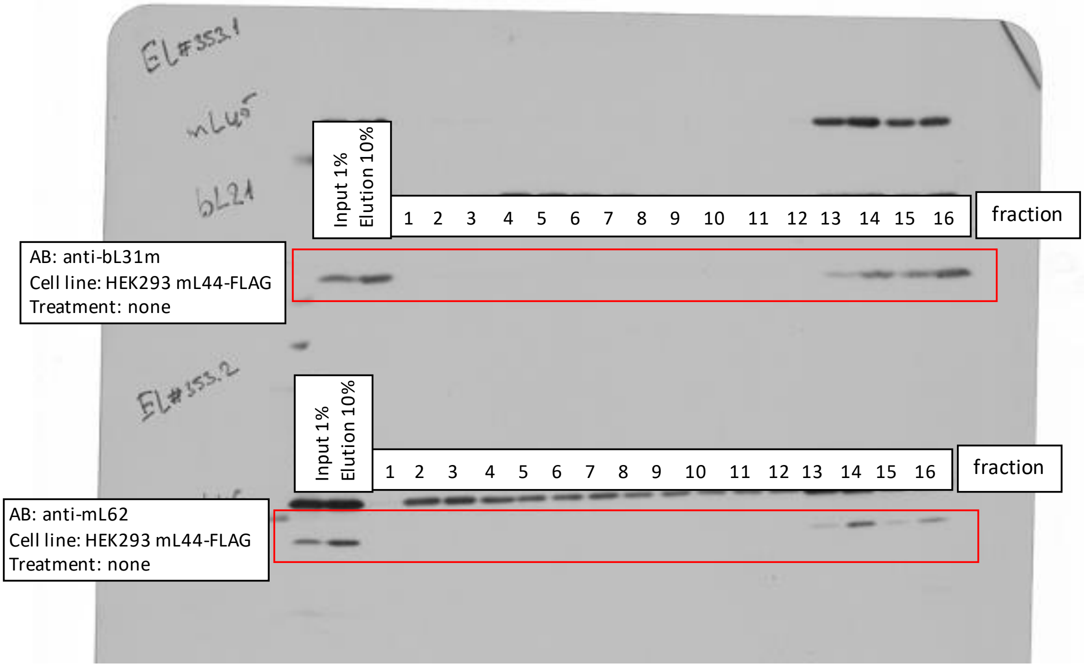

Source Data 5_related to Extended Data Fig.9b
EL#353 mL44-FLAG IP + Gradient
Rotor: SW41 Ti
Gradient: Sucrose 5-30%
Speed: 158.000xg
Time: 15h

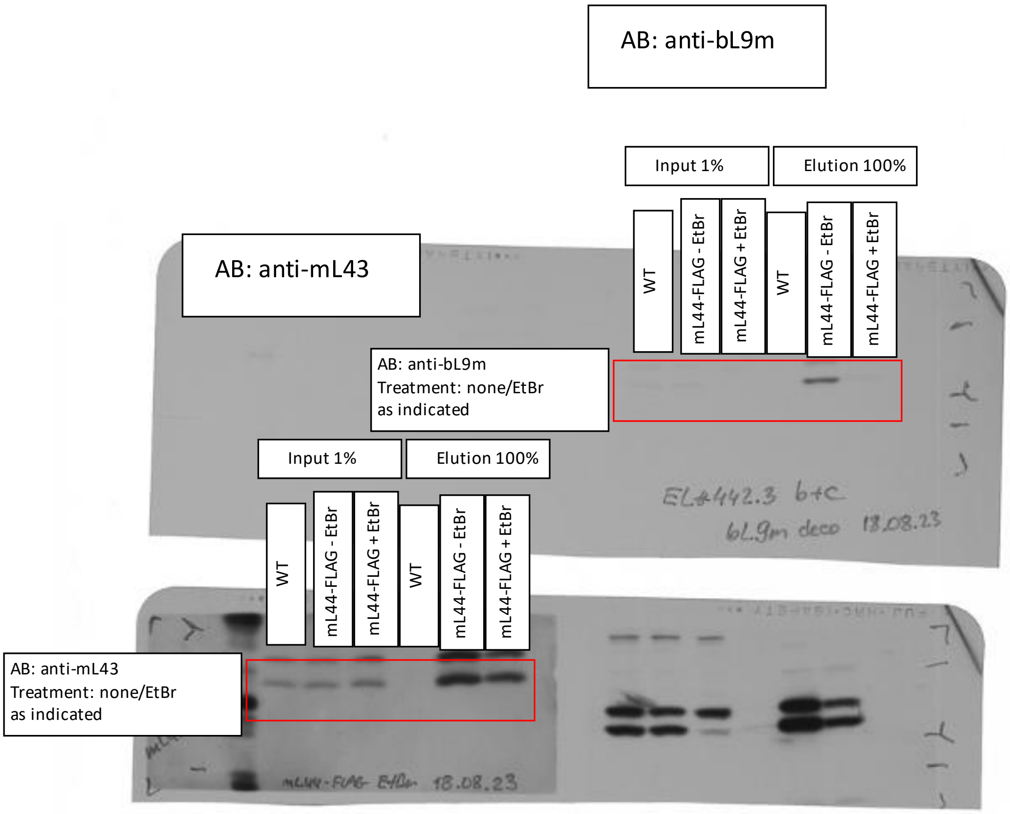

Source Data 5_related to Extended Data Fig.9b
EL#353 mL44-FLAG IP + Gradient
Rotor: SW41 Ti
Gradient: Sucrose 5-30%
Speed: 158.000xg
Time: 15h

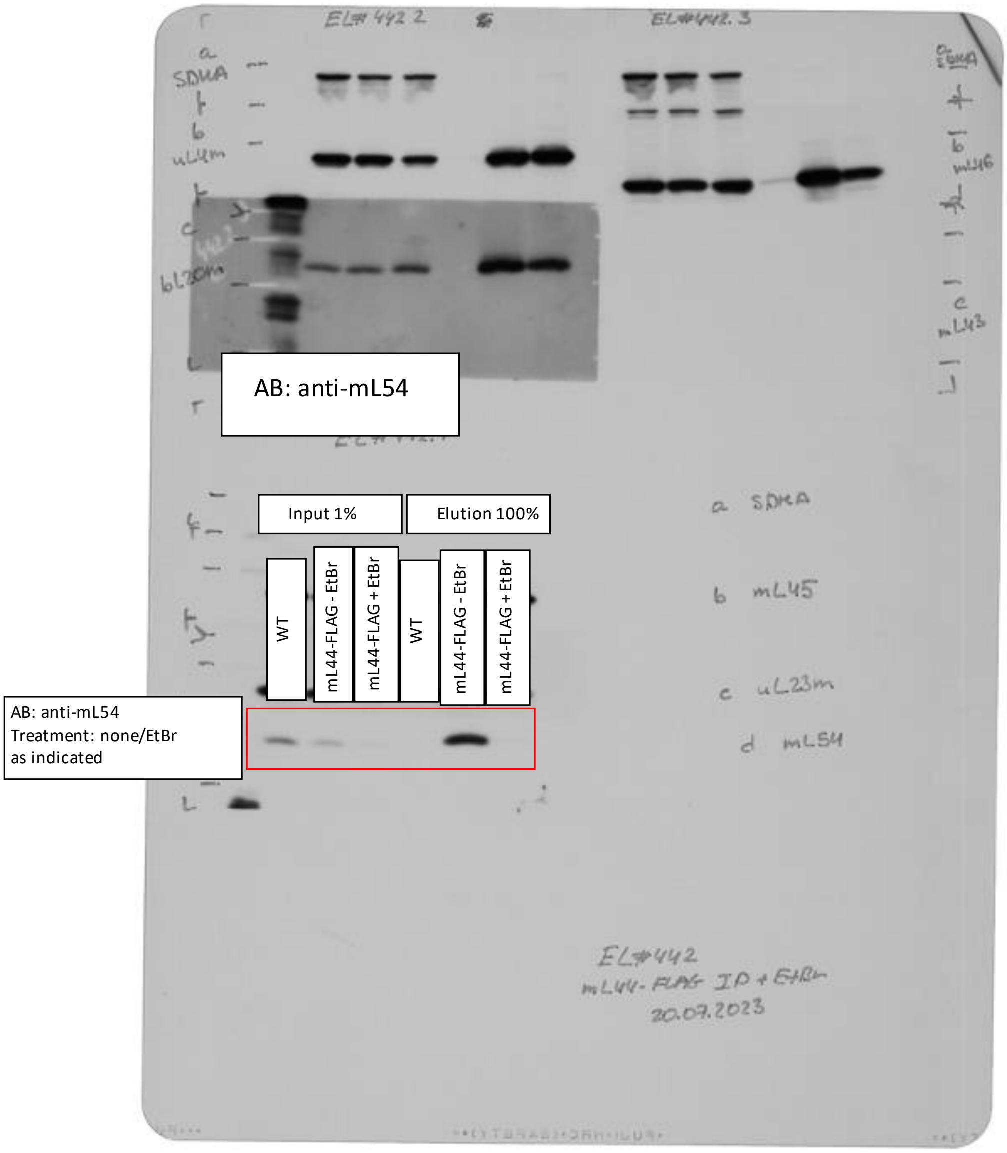

Source Data 5_related to Extended Data Fig.9b
EL#353 mL44-FLAG IP + Gradient
Rotor: SW41 Ti
Gradient: Sucrose 5-30%
Speed: 158.000xg
Time: 15h

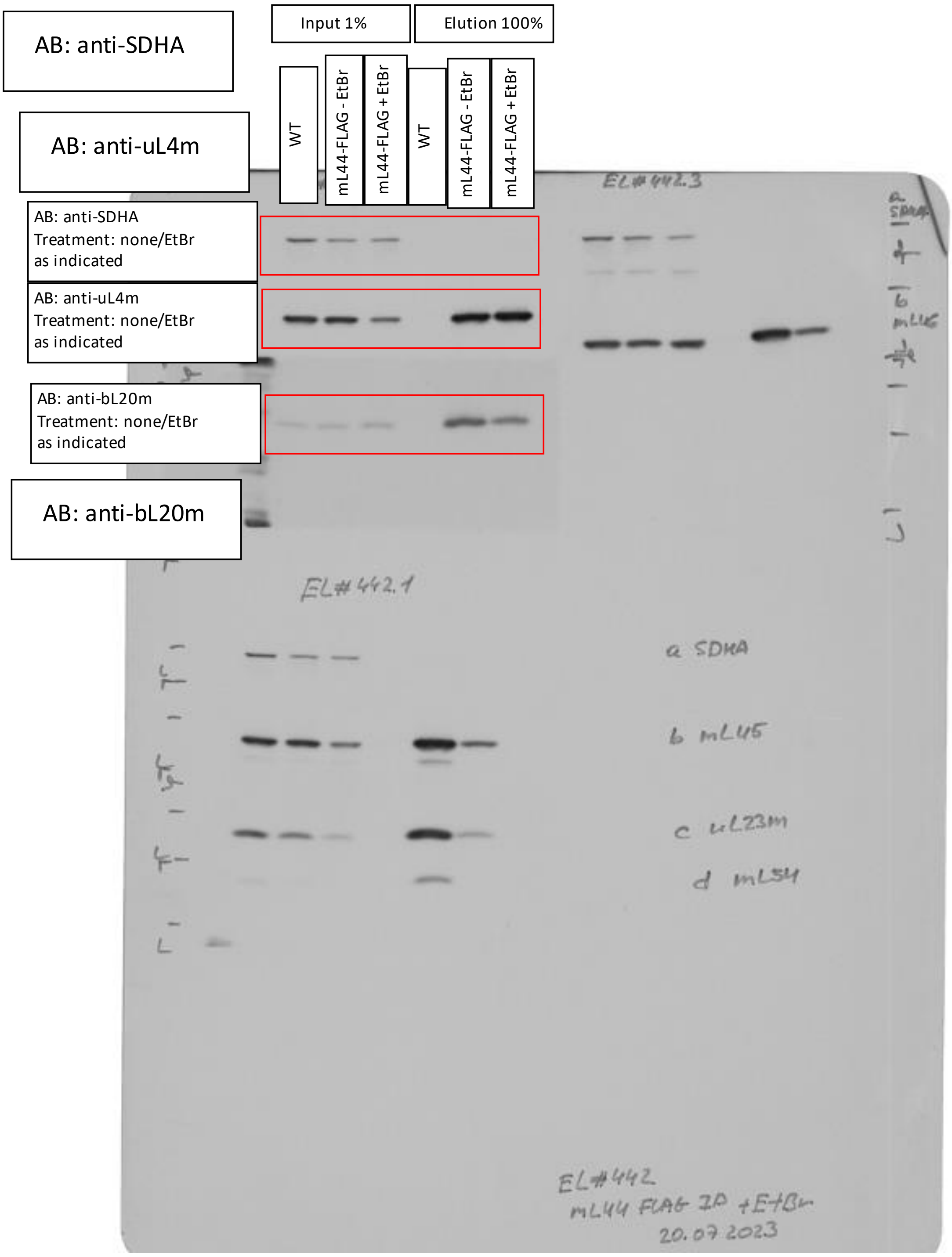

Source Data 5_related to Extended Data Fig.9b
EL#353 mL44-FLAG IP + Gradient
Rotor: SW41 Ti
Gradient: Sucrose 5-30%
Speed: 158.000xg
Time: 15h

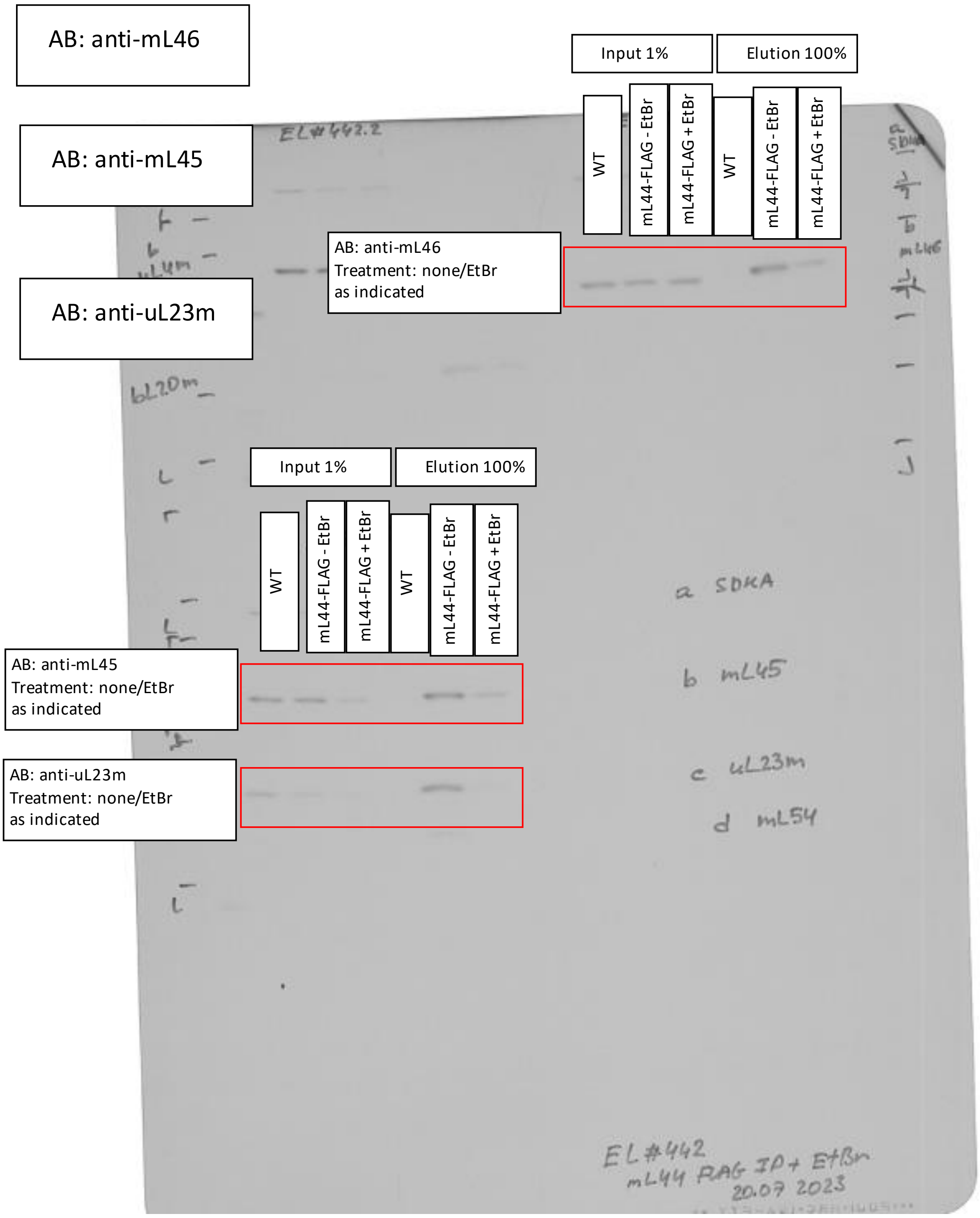

Source Data 5_related to Extended Data Fig.9b
EL#353 mL44-FLAG IP + Gradient
Rotor: SW41 Ti
Gradient: Sucrose 5-30%
Speed: 158.000xg
Time: 15h

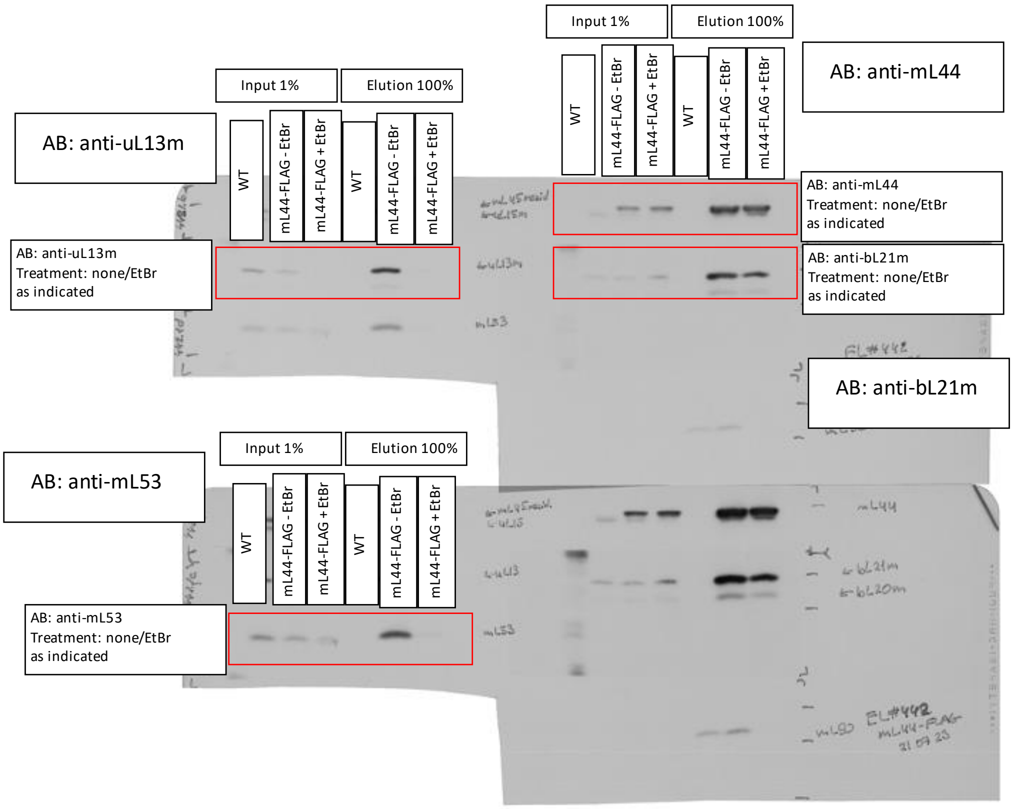

Source Data 5_related to Extended Data Fig.9b
EL#353 mL44-FLAG IP + Gradient
Rotor: SW41 Ti
Gradient: Sucrose 5-30%
Speed: 158.000xg
Time: 15h

AB: anti-mL50

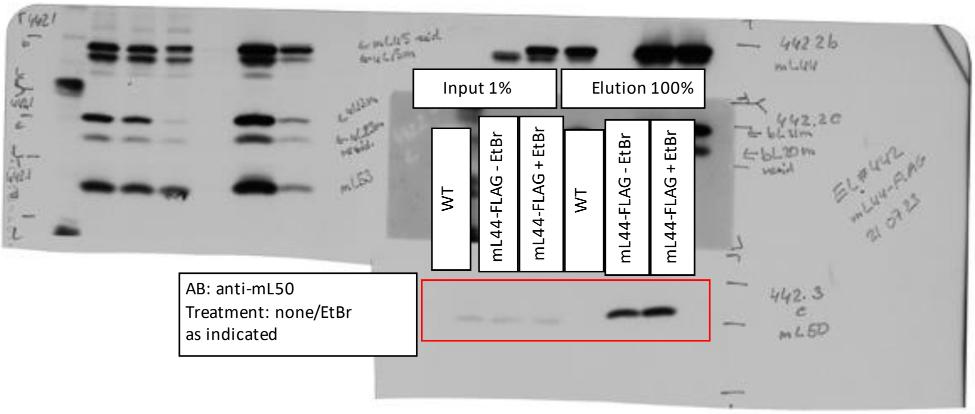

Source Data 5_related to Extended Data Fig.9b
EL#353 mL44-FLAG IP + Gradient
Rotor: SW41 Ti
Gradient: Sucrose 5-30%
Speed: 158.000xg
Time: 15h

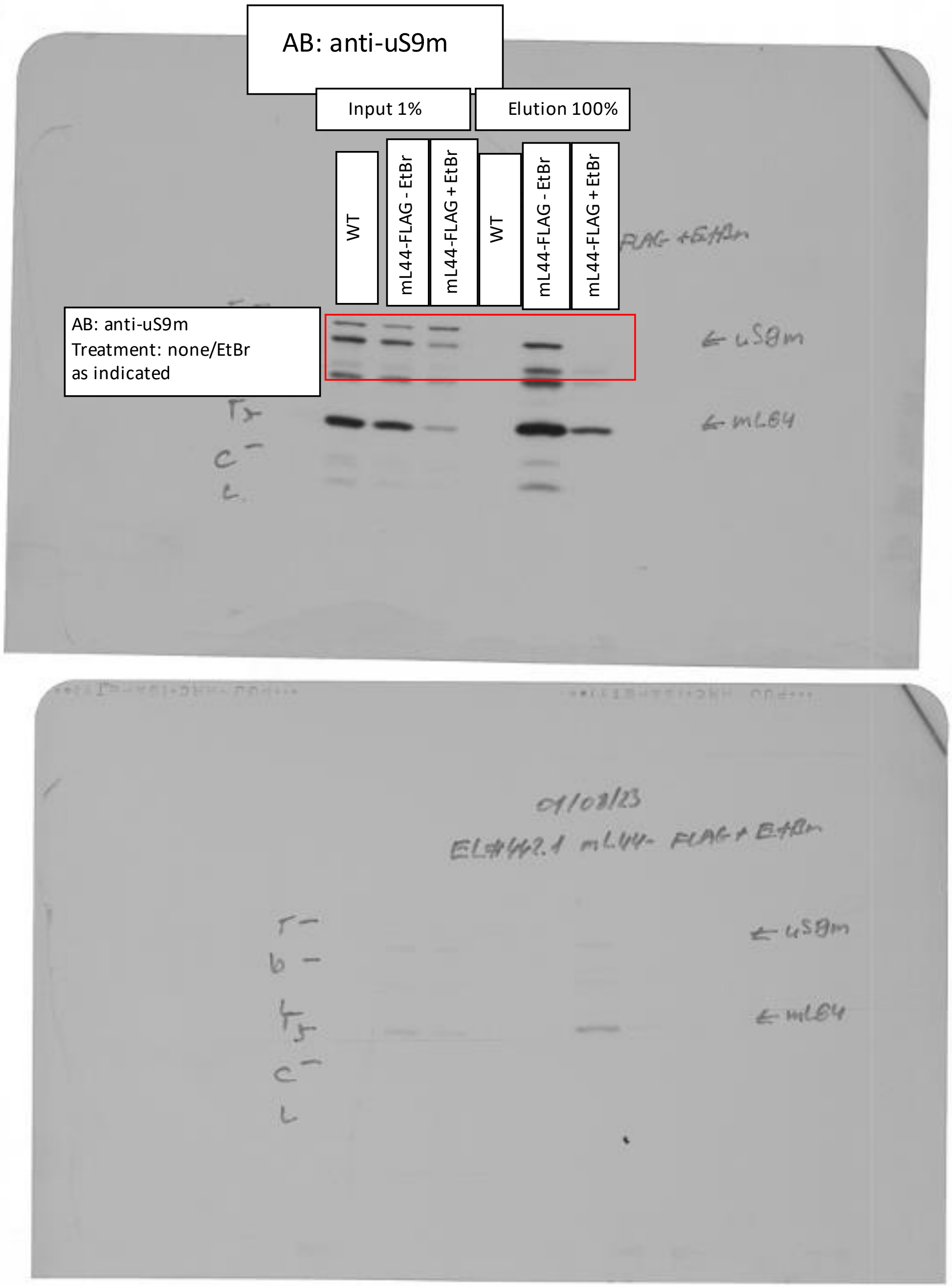

Source Data 5_related to Extended Data Fig.9c
EL#363 HEK293 WT + Ethidium Bromide treatment + Gradient
EL#379 HEK293 WT + Ethidium Bromide treatment + Gradient

Rotor: SW41 Ti
Gradient: Sucrose 5-30%
Speed: 158.000xg
Time: 15h

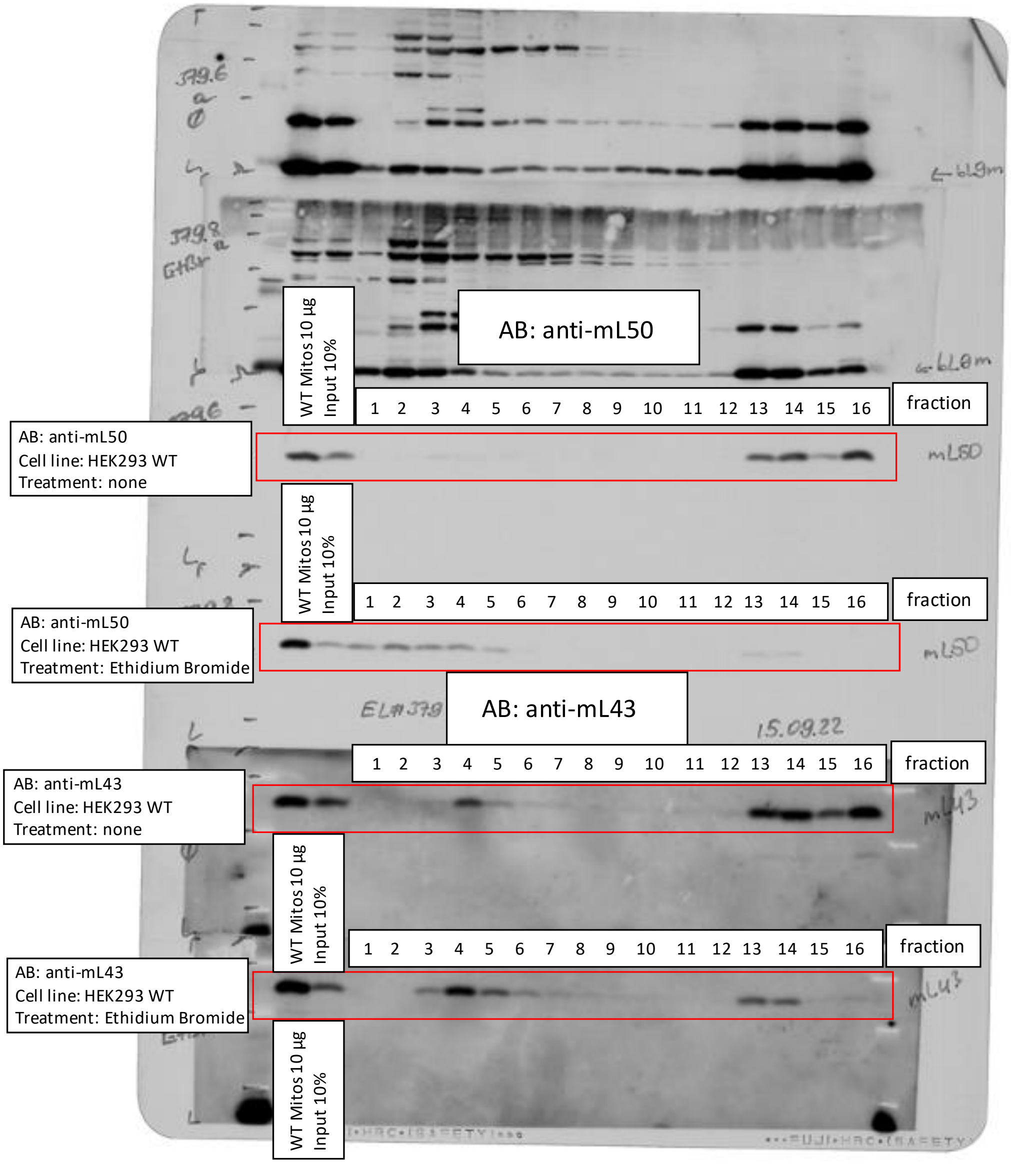

Source Data 5_related to Extended Data Fig.9c
EL#363 HEK293 WT + Ethidium Bromide treatment + Gradient
EL#379 HEK293 WT + Ethidium Bromide treatment + Gradient

Rotor: SW41 Ti
Gradient: Sucrose 5-30%
Speed: 158.000xg
Time: 15h

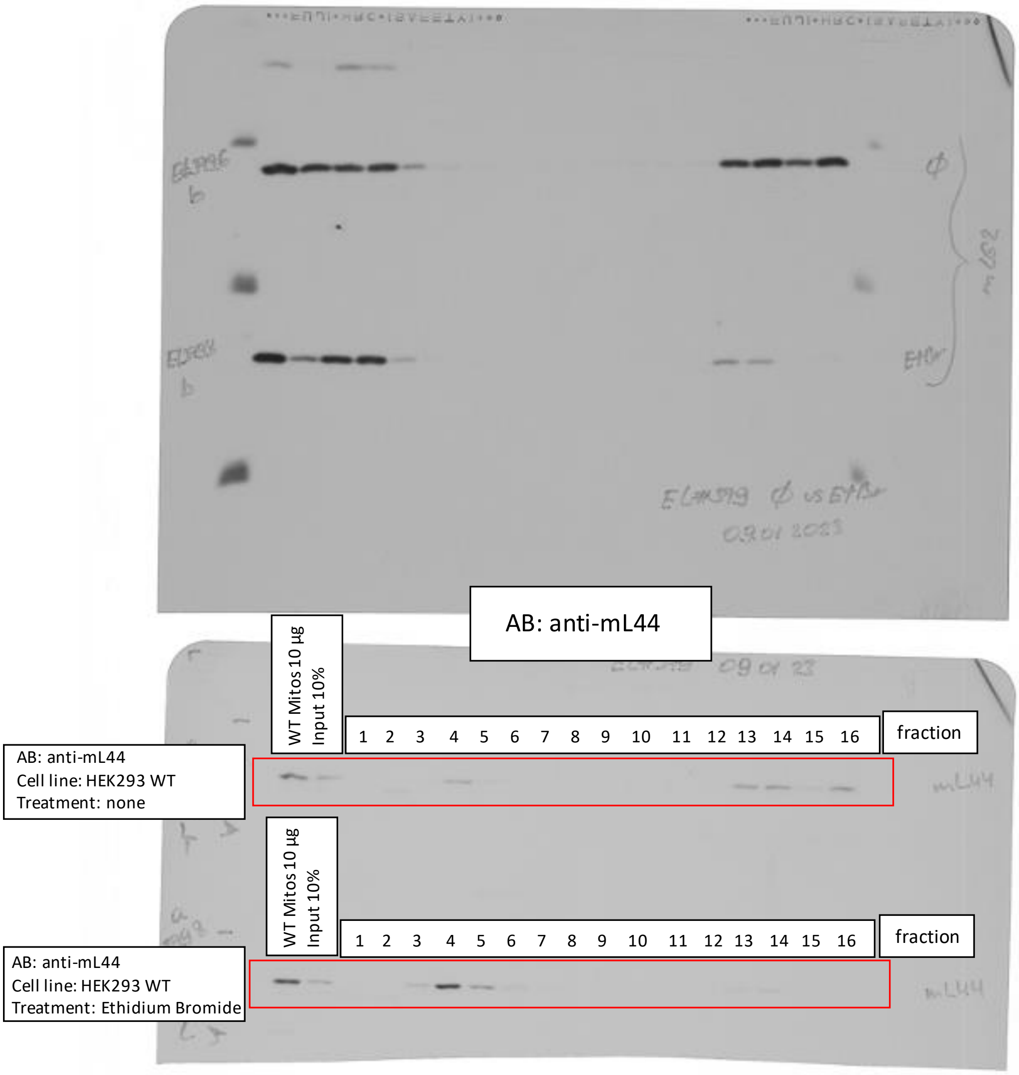

Source Data 5_related to Extended Data Fig.9c
EL#363 HEK293 WT + Ethidium Bromide treatment + Gradient
EL#379 HEK293 WT + Ethidium Bromide treatment + Gradient

Rotor: SW41 Ti
Gradient: Sucrose 5-30%
Speed: 158.000xg
Time: 15h

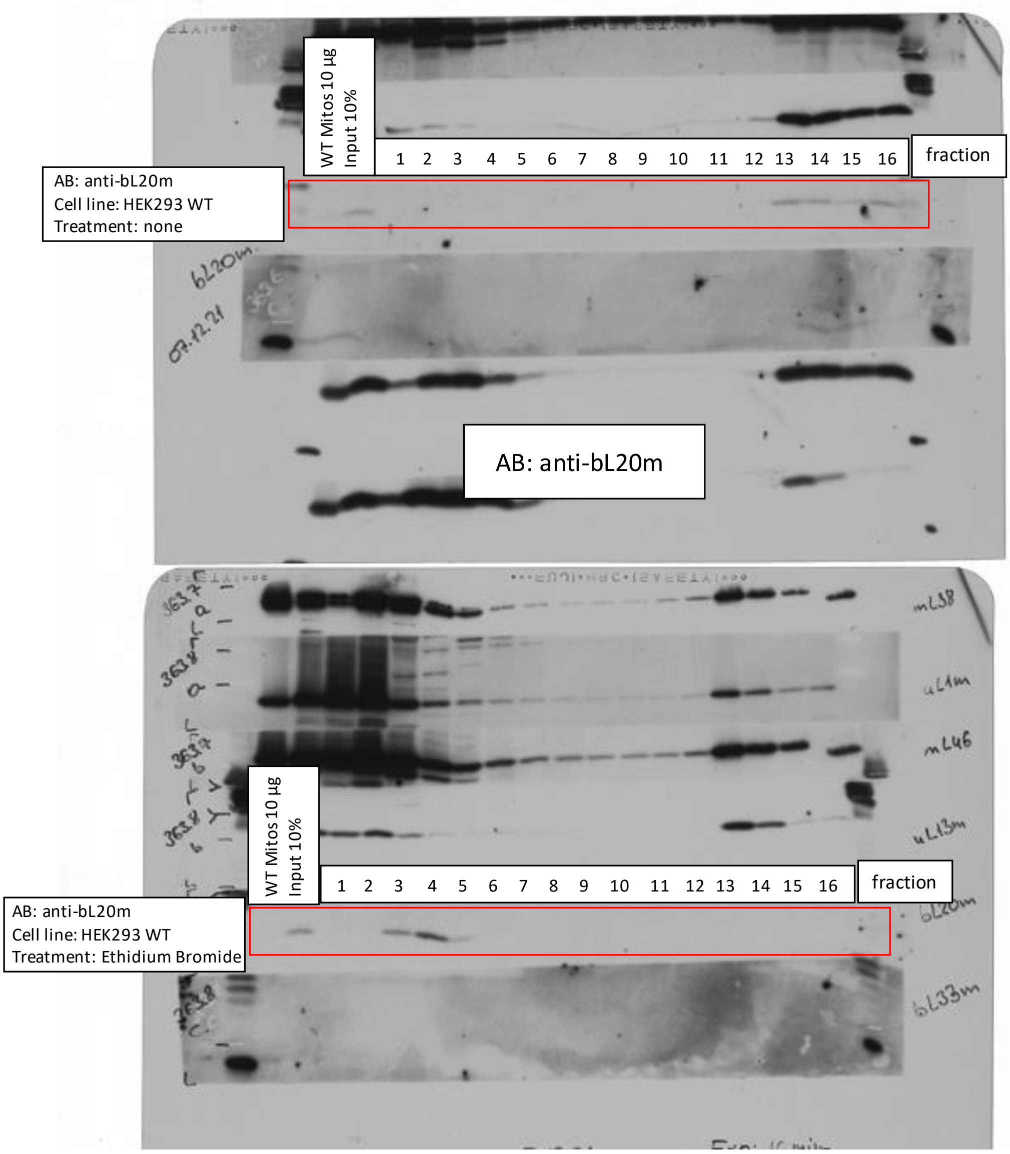

Source Data 5_related to Extended Data Fig.9c
EL#363 HEK293 WT + Ethidium Bromide treatment + Gradient
EL#379 HEK293 WT + Ethidium Bromide treatment + Gradient

Rotor: SW41 Ti
Gradient: Sucrose 5-30%
Speed: 158.000xg
Time: 15h

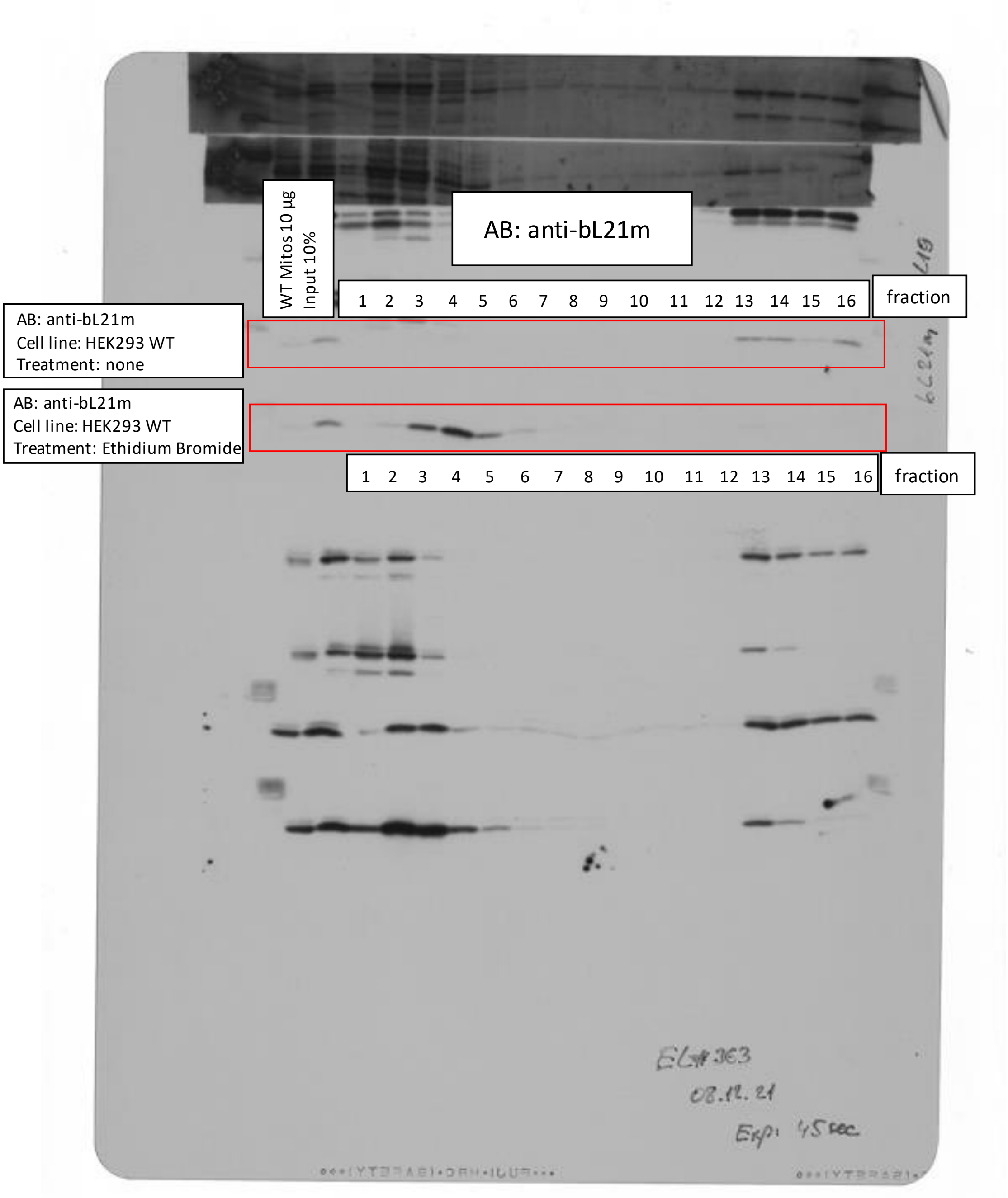

Source Data 5_related to Extended Data Fig.9c
EL#363 HEK293 WT + Ethidium Bromide treatment + Gradient
EL#379 HEK293 WT + Ethidium Bromide treatment + Gradient

Rotor: SW41 Ti
Gradient: Sucrose 5-30%
Speed: 158.000xg
Time: 15h

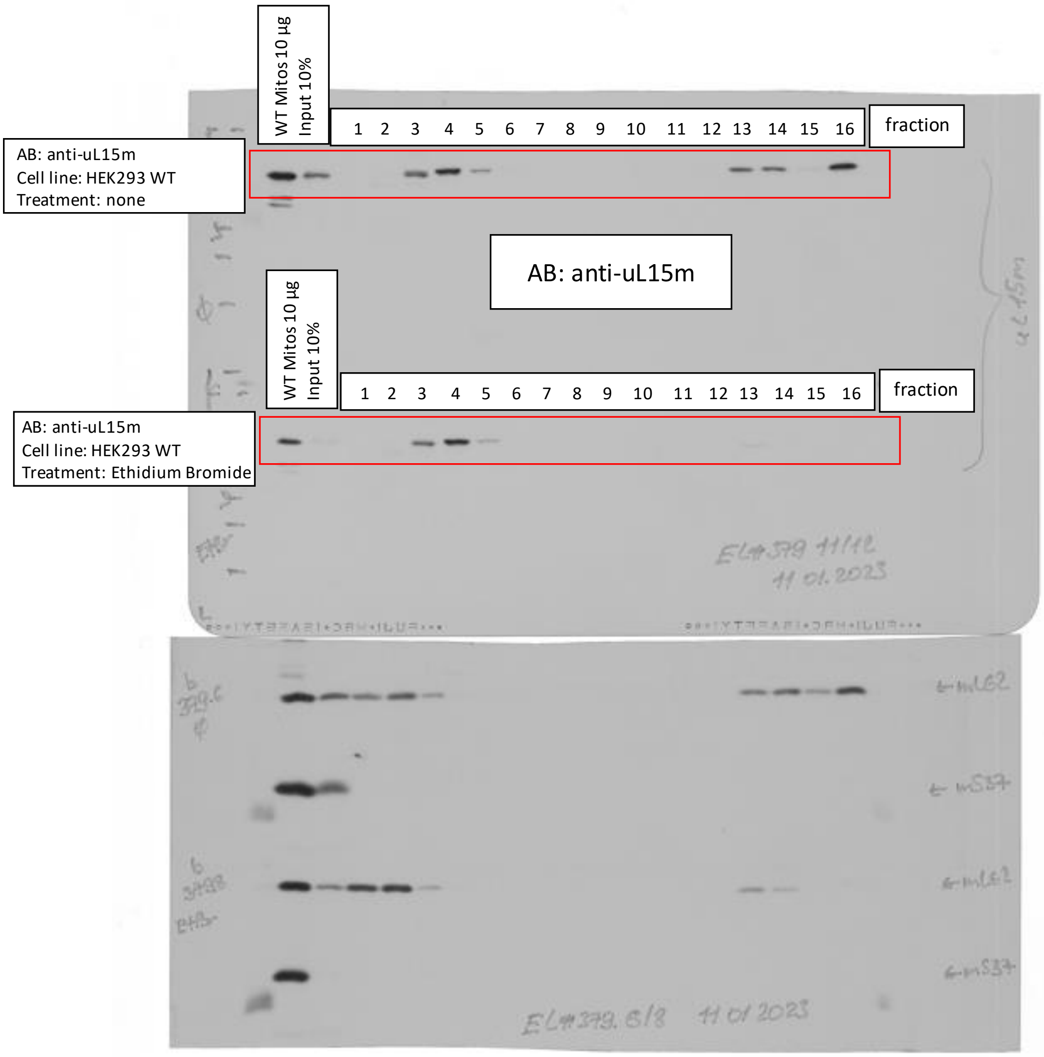

Source Data 5_related to Extended Data Fig.9c
EL#363 HEK293 WT + Ethidium Bromide treatment + Gradient
EL#379 HEK293 WT + Ethidium Bromide treatment + Gradient

Rotor: SW41 Ti
Gradient: Sucrose 5-30%
Speed: 158.000xg
Time: 15h

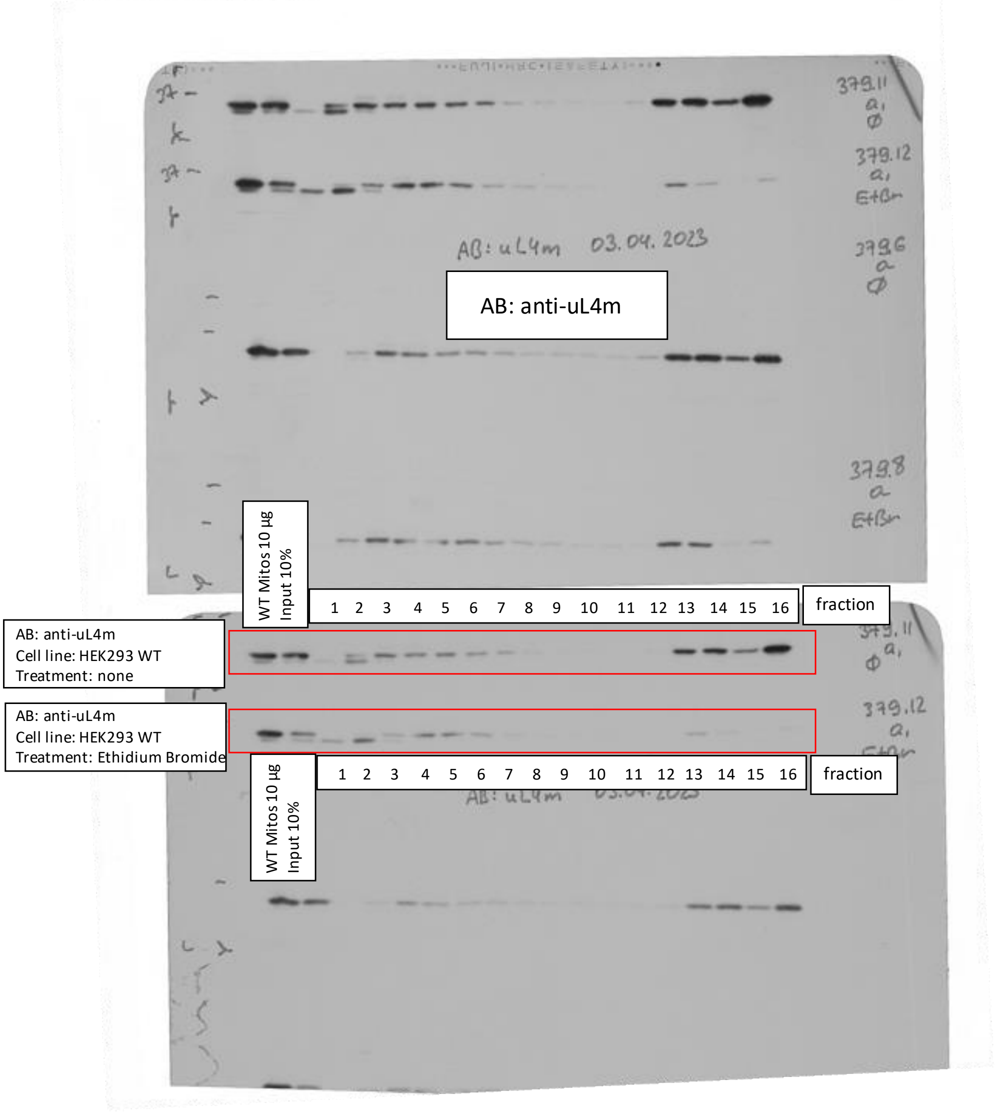

Source Data 5_related to Extended Data Fig.9d
EL#405 HEK293 WT vs. HEK293 mL44 KO + Gradient
EL#409 HEK293 WT vs. HEK293 uL4m KO and bL20m KO
Rotor: SW41 Ti
Gradient: Sucrose 5-30%
Speed: 158.000xg
Time: 15h

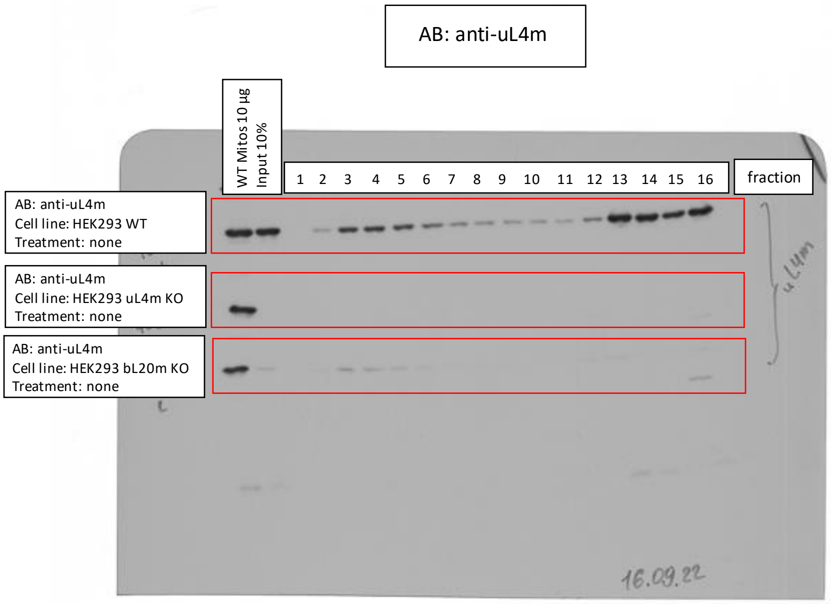

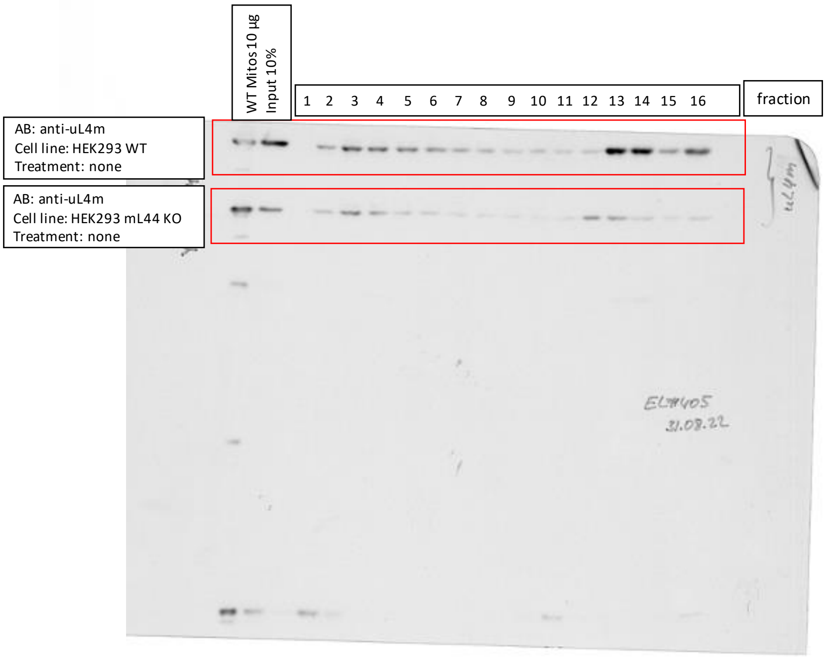

Source Data 5_related to Extended Data Fig.9d
EL#405 HEK293 WT vs. HEK293 mL44 KO + Gradient
EL#409 HEK293 WT vs. HEK293 uL4m KO and bL20m KO
Rotor: SW41 Ti
Gradient: Sucrose 5-30%
Speed: 158.000xg
Time: 15h

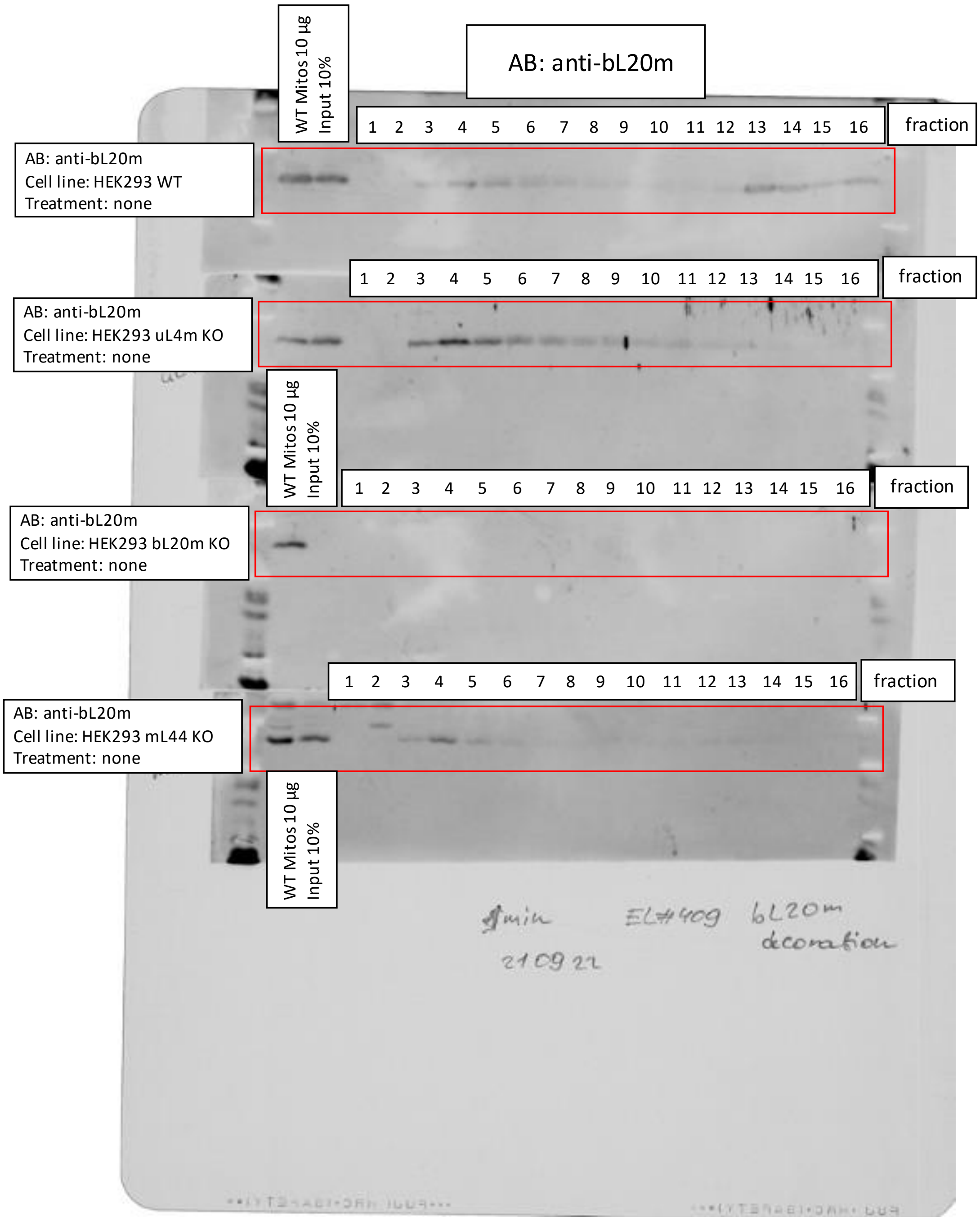

Source Data 5_related to Extended Data Fig.9d
EL#405 HEK293 WT vs. HEK293 mL44 KO + Gradient
EL#409 HEK293 WT vs. HEK293 uL4m KO and bL20m KO
Rotor: SW41 Ti
Gradient: Sucrose 5-30%
Speed: 158.000xg
Time: 15h

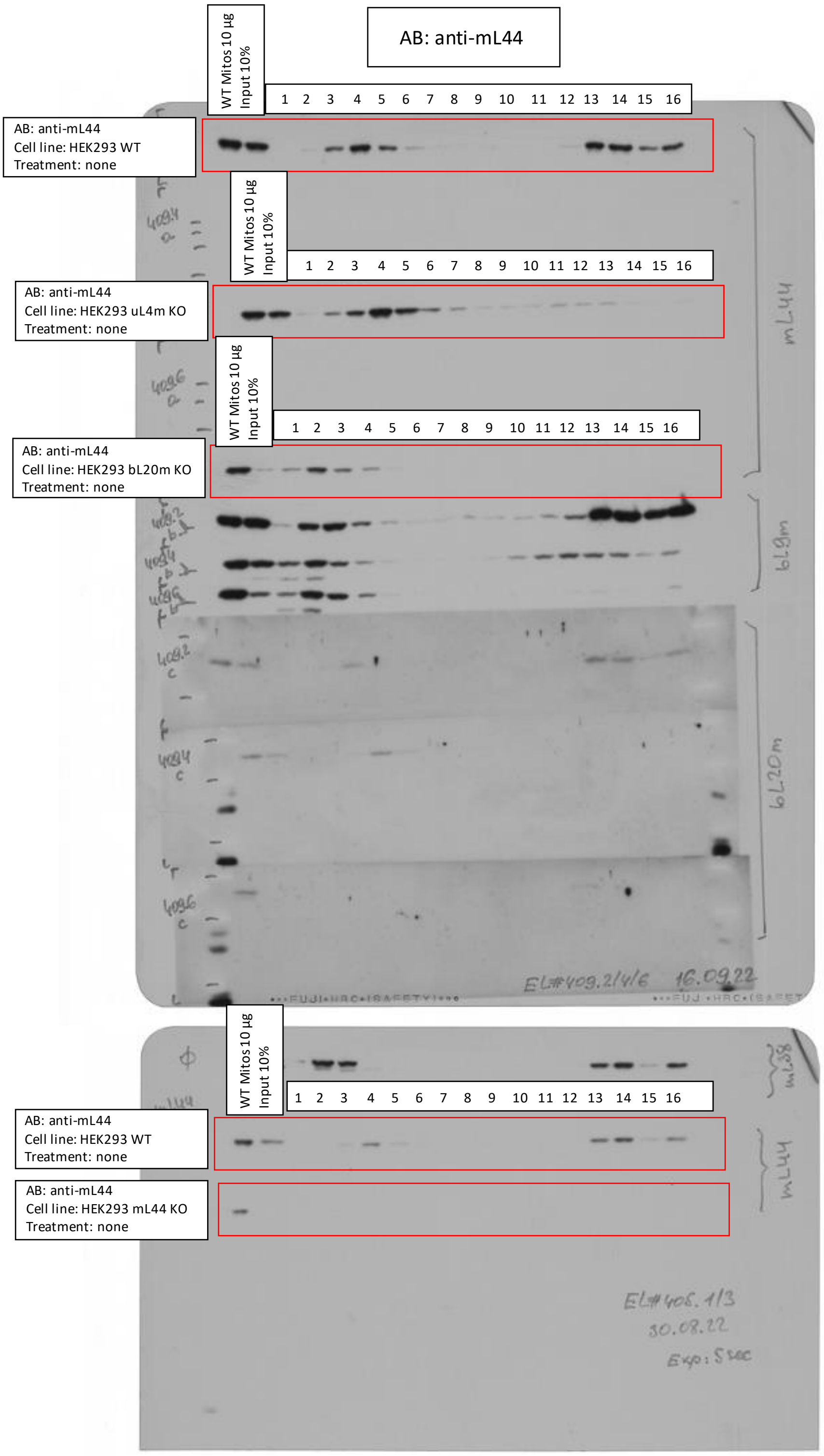

Source Data 5_related to Extended Data Fig.9d
EL#405 HEK293 WT vs. HEK293 mL44 KO + Gradient
EL#409 HEK293 WT vs. HEK293 uL4m KO and bL20m KO
Rotor: SW41 Ti
Gradient: Sucrose 5-30%
Speed: 158.000xg
Time: 15h

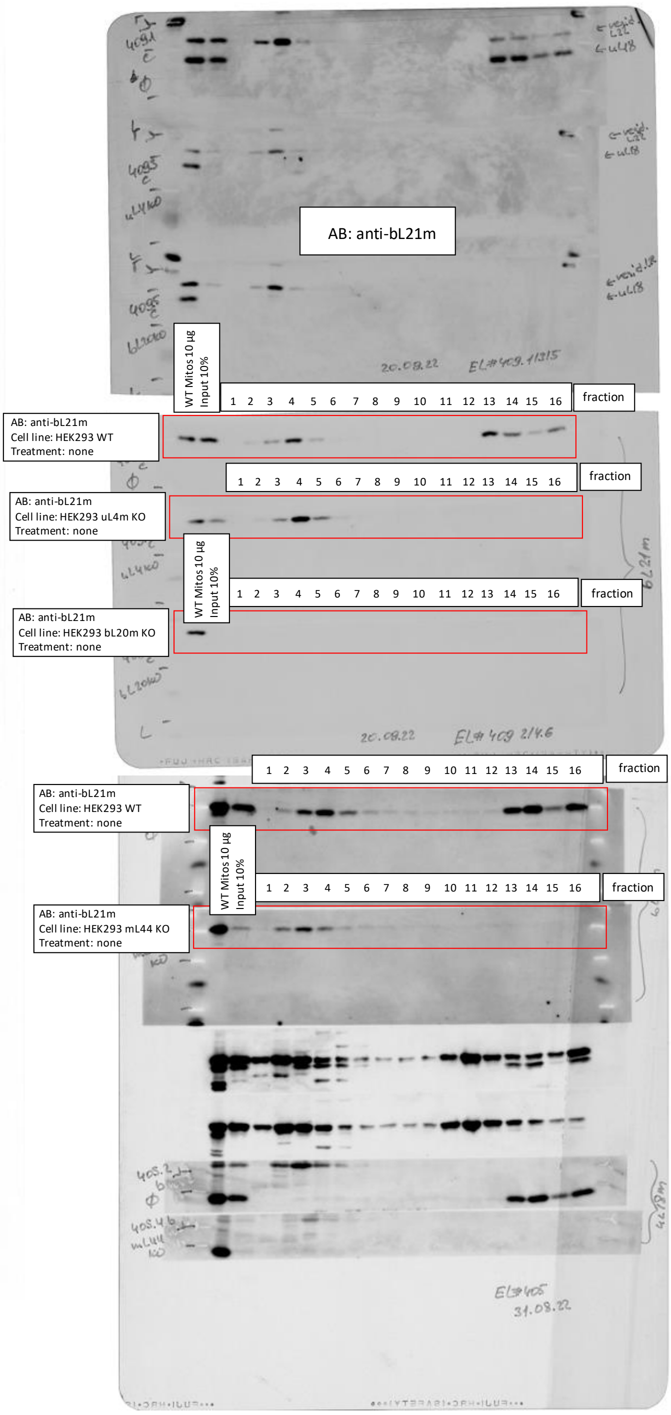

Source Data 5_related to Extended Data Fig.9d
EL#405 HEK293 WT vs. HEK293 mL44 KO + Gradient
EL#409 HEK293 WT vs. HEK293 uL4m KO and bL20m KO
Rotor: SW41 Ti
Gradient: Sucrose 5-30%
Speed: 158.000xg
Time: 15h

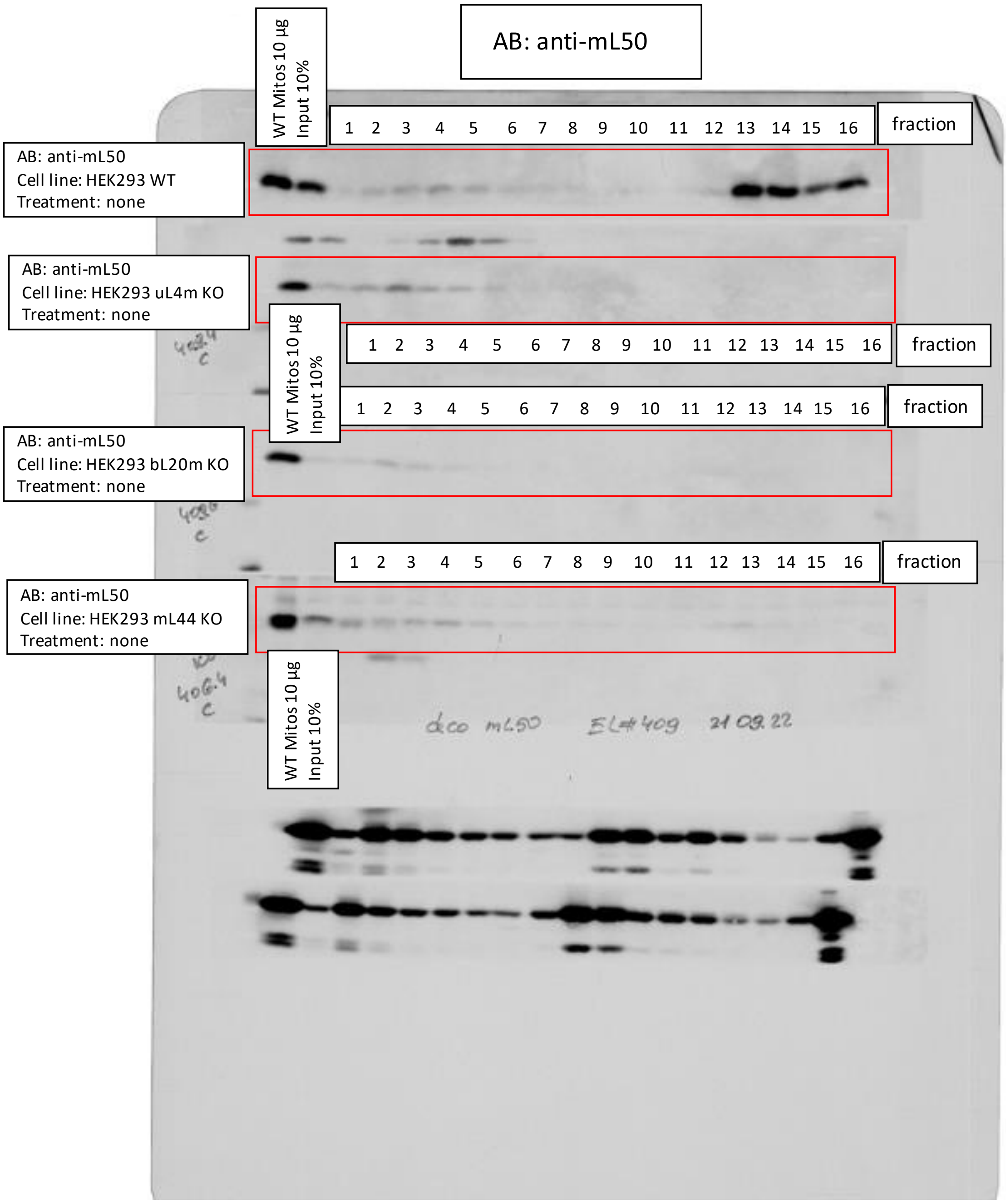

Source Data 5_related to Extended Data Fig.9d
EL#405 HEK293 WT vs. HEK293 mL44 KO + Gradient
EL#409 HEK293 WT vs. HEK293 uL4m KO and bL20m KO
Rotor: SW41 Ti
Gradient: Sucrose 5-30%
Speed: 158.000xg
Time: 15h

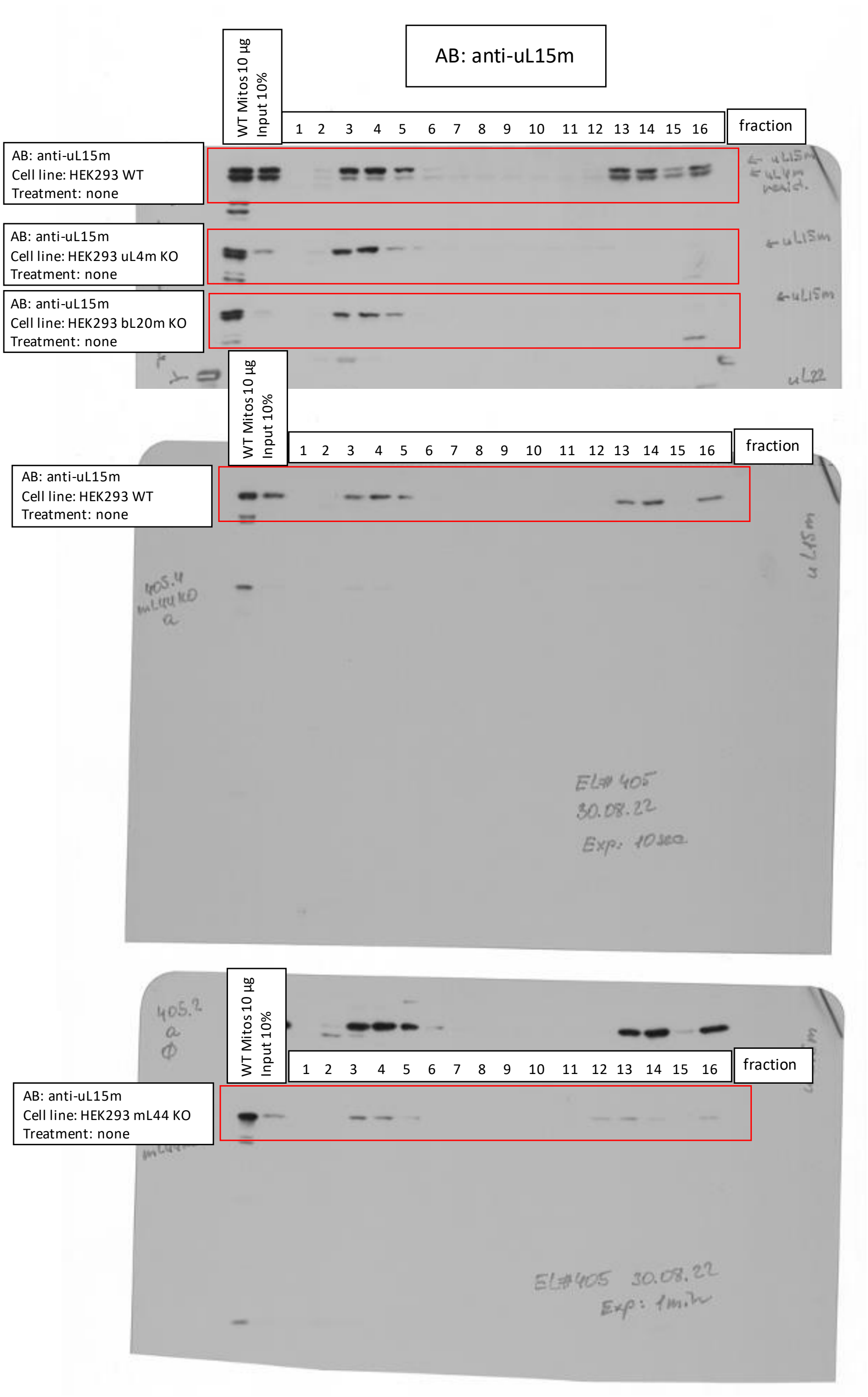

Source Data 5_related to Extended Data Fig.9d
EL#405 HEK293 WT vs. HEK293 mL44 KO + Gradient
EL#409 HEK293 WT vs. HEK293 uL4m KO and bL20m KO
Rotor: SW41 Ti
Gradient: Sucrose 5-30%
Speed: 158.000xg
Time: 15h

AB: anti-mL43

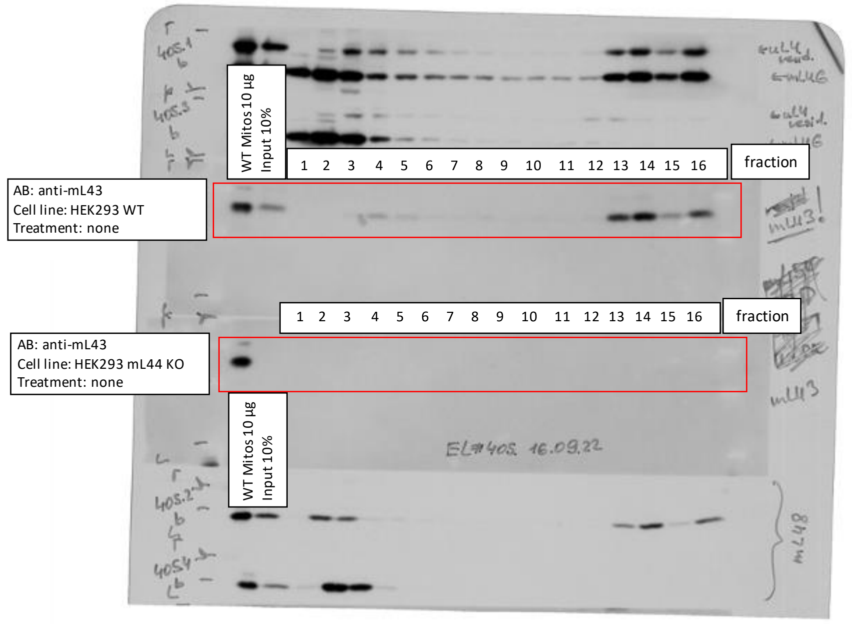

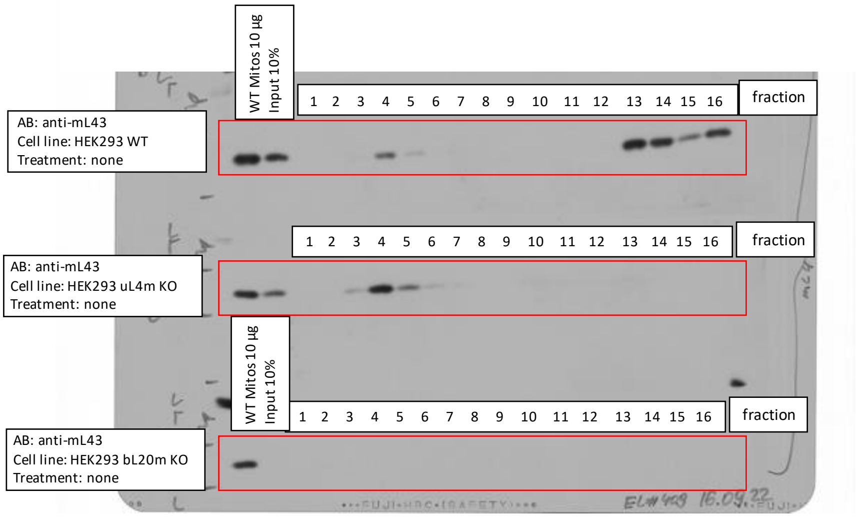

Source Data 5_related to Extended Data Fig.9d
EL#405 HEK293 WT vs. HEK293 mL44 KO + Gradient
EL#409 HEK293 WT vs. HEK293 uL4m KO and bL20m KO
Rotor: SW41 Ti
Gradient: Sucrose 5-30%
Speed: 158.000xg
Time: 15h

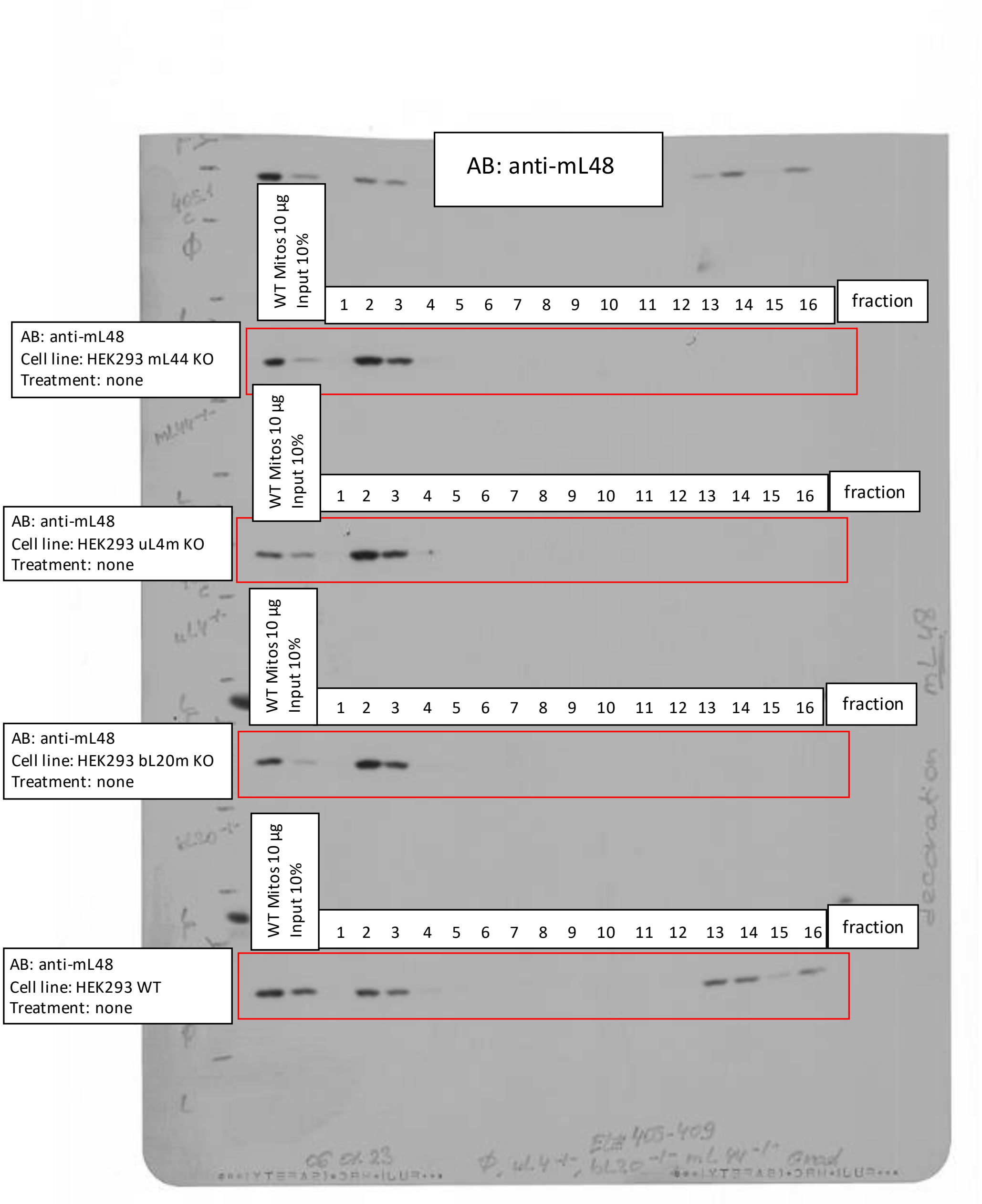

Source Data 5_related to Extended Data Fig.9d
EL#405 HEK293 WT vs. HEK293 mL44 KO + Gradient
EL#409 HEK293 WT vs. HEK293 uL4m KO and bL20m KO
Rotor: SW41 Ti
Gradient: Sucrose 5-30%
Speed: 158.000xg
Time: 15h

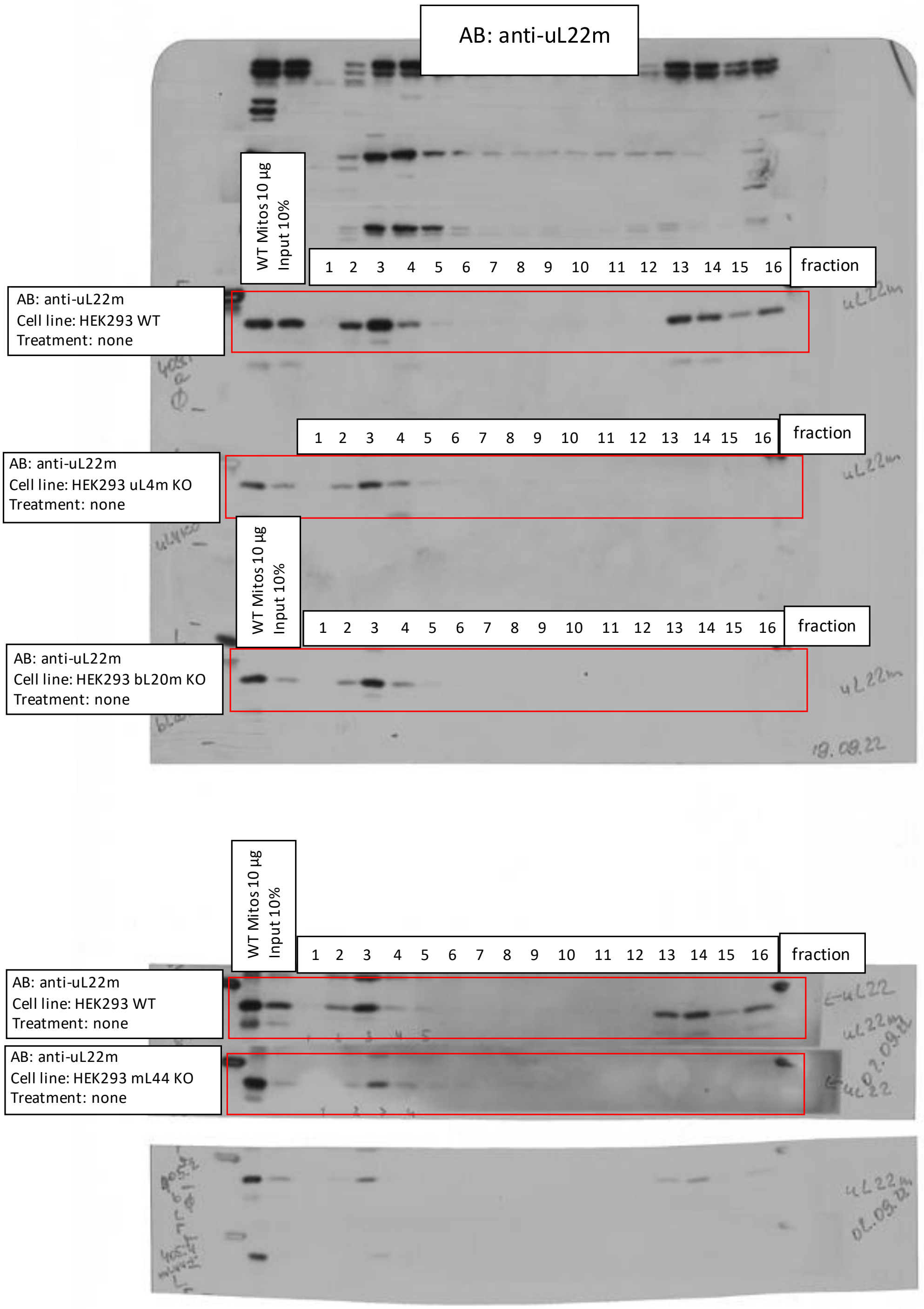

Source Data 5_related to Extended Data Fig.9d
EL#405 HEK293 WT vs. HEK293 mL44 KO + Gradient
EL#409 HEK293 WT vs. HEK293 uL4m KO and bL20m KO
Rotor: SW41 Ti
Gradient: Sucrose 5-30%
Speed: 158.000xg
Time: 15h

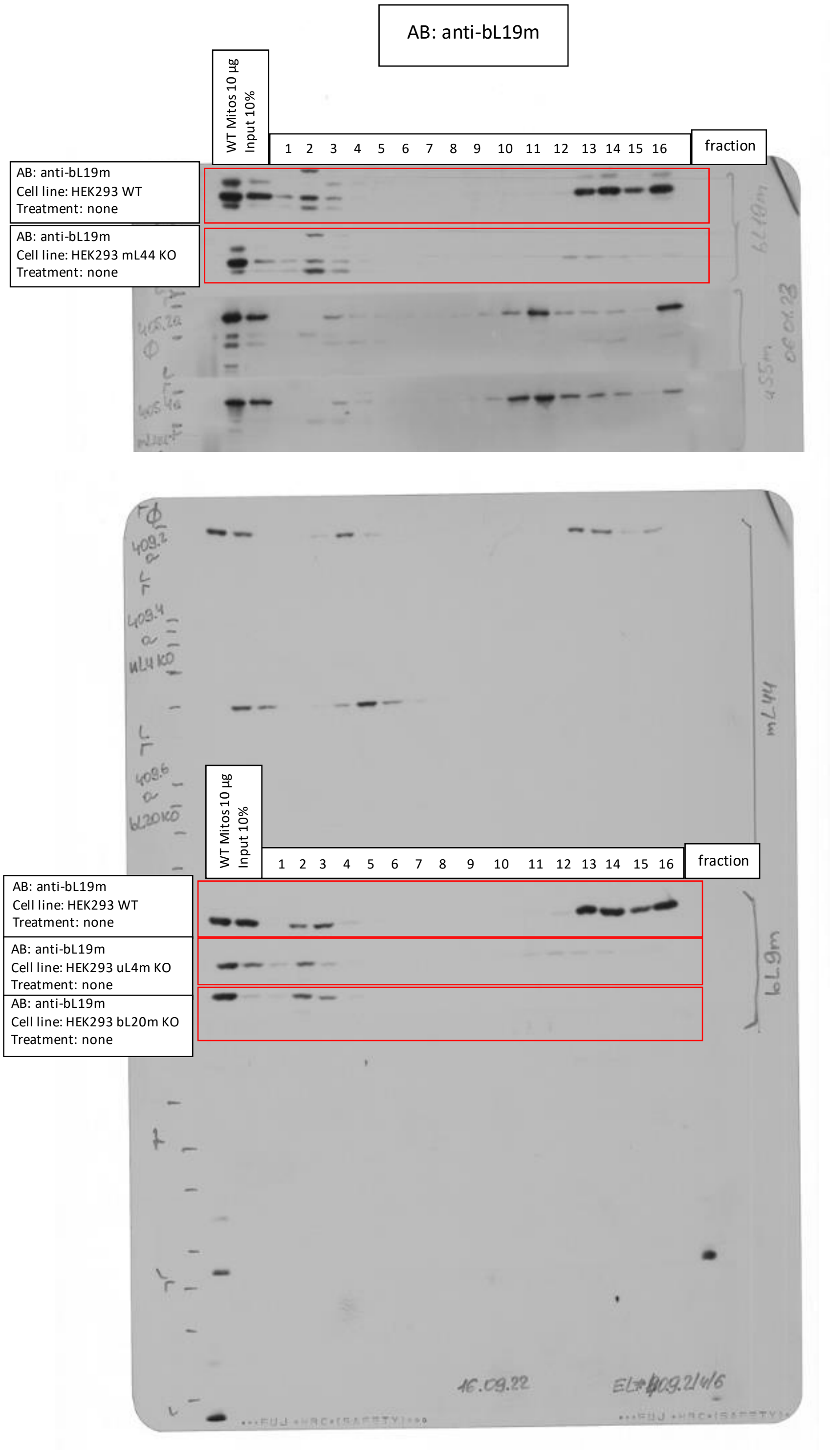

Source Data 5_related to Extended Data Fig.9d
EL#405 HEK293 WT vs. HEK293 mL44 KO + Gradient
EL#409 HEK293 WT vs. HEK293 uL4m KO and bL20m KO
Rotor: SW41 Ti
Gradient: Sucrose 5-30%
Speed: 158.000xg
Time: 15h

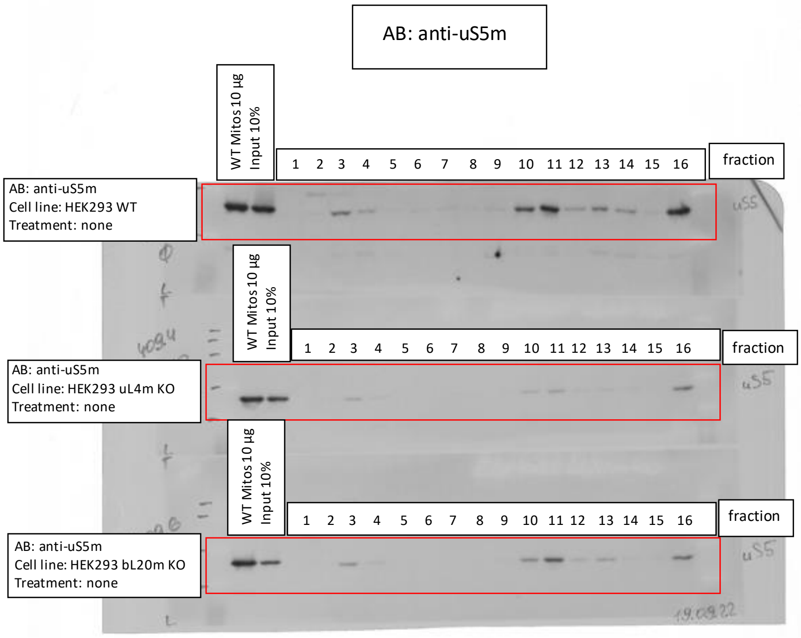

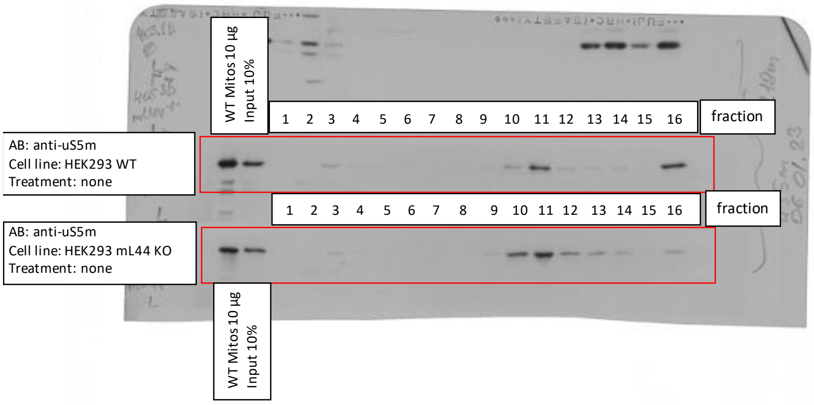

Supplement: Supplementary file 26 — Unprocessed blots. [file 41594_2024_1356_MOESM26_ESM.pdf]
