# Supplementary material for: A roadmap for ribosome assembly in human mitochondria
Source: Nat Struct Mol Biol. 2024 Jul 11;31(12):1898–908. doi: 10.1038/s41594-024-01356-w (PMC11638073; doi:10.1038/s41594-024-01356-w)

Source Data 6_related to Extended Data Fig.10a
EL#377.4-6 mL39-FLAG IP + Gradient
Rotor: SW41 Ti
Gradient: Sucrose 5-30%
Speed: 158.000xg
Time: 15h

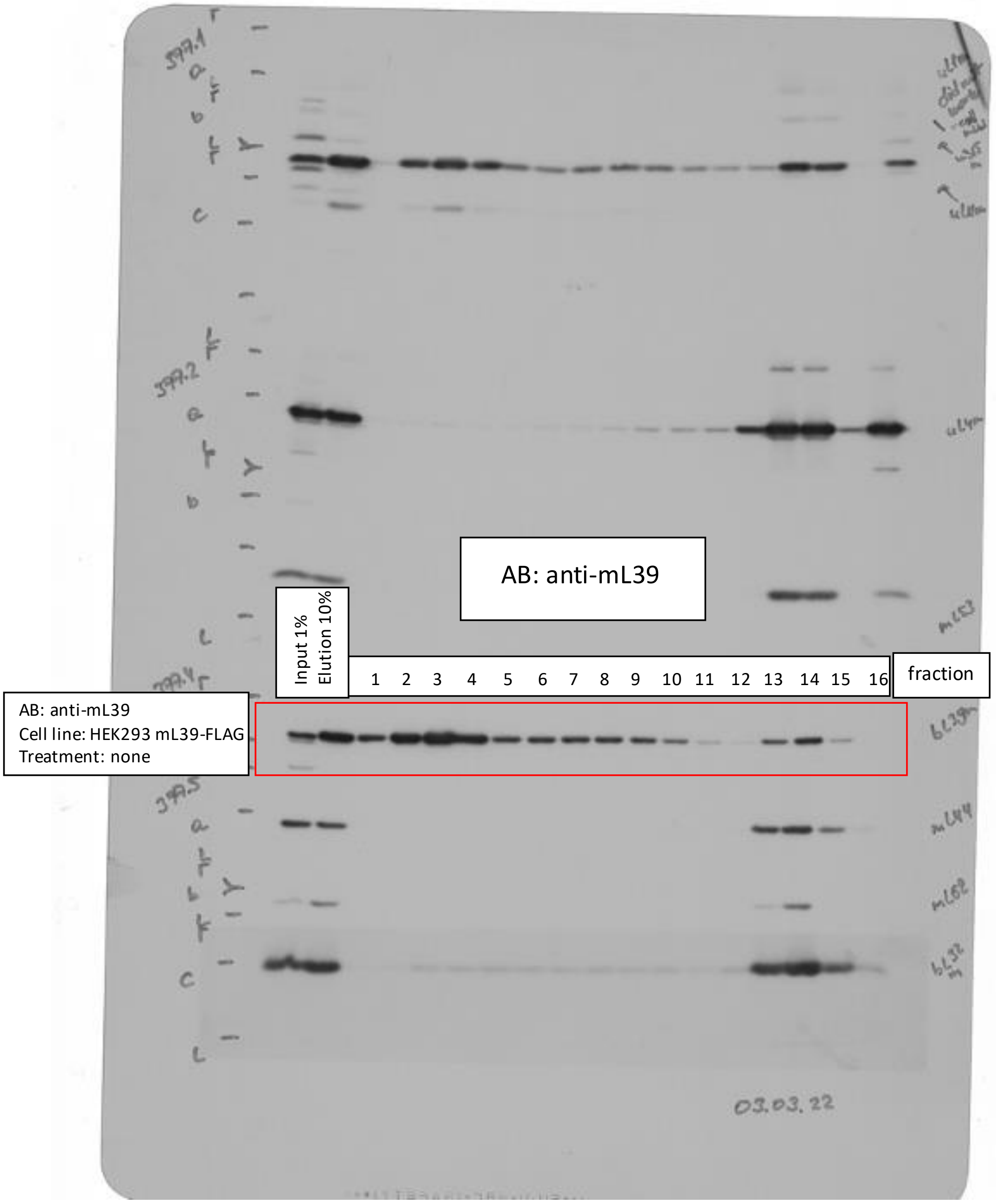

Source Data 6_related to Extended Data Fig.10a
EL#377.4-6 mL39-FLAG IP + Gradient
Rotor: SW41 Ti
Gradient: Sucrose 5-30%
Speed: 158.000xg
Time: 15h

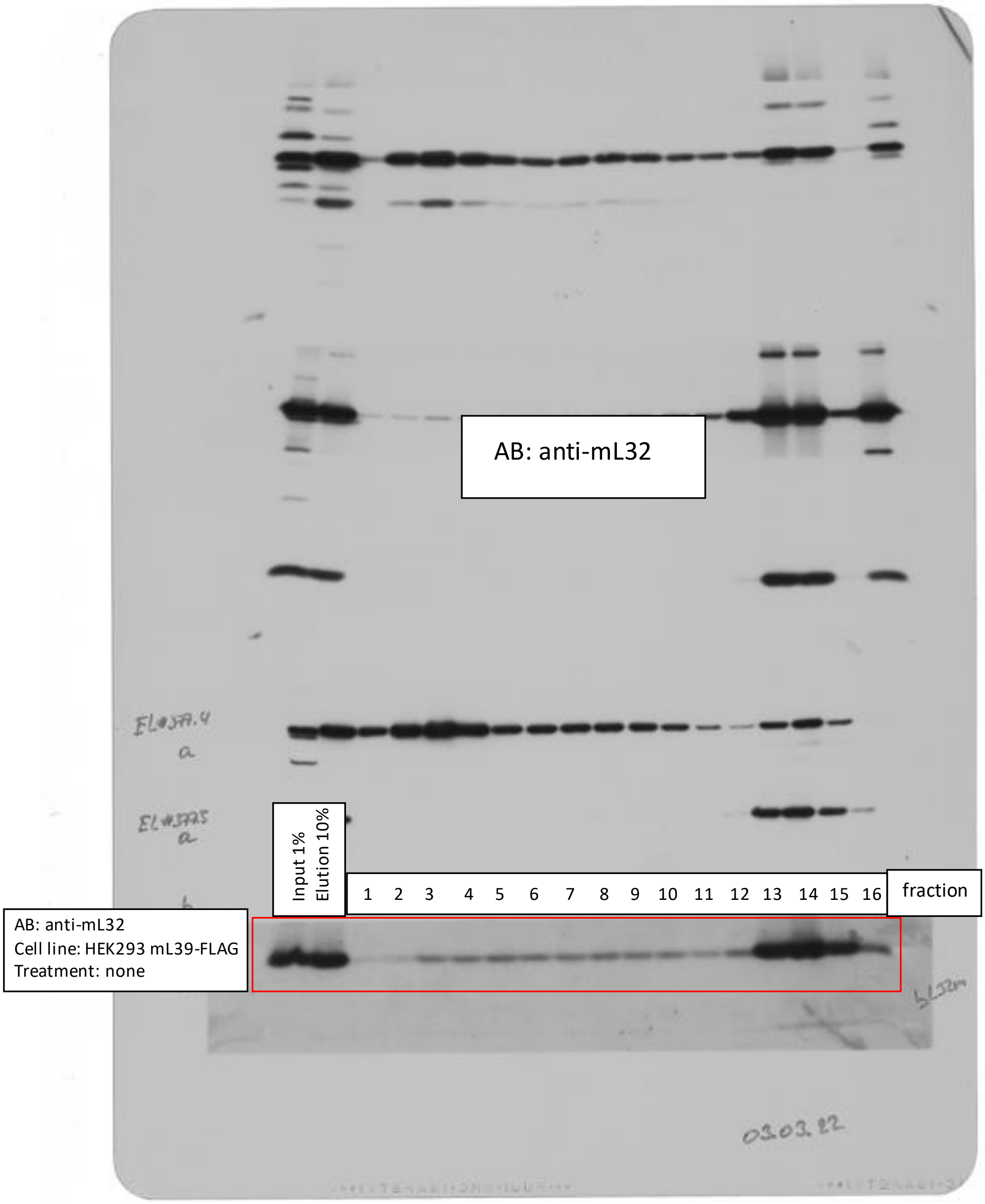

Source Data 6_related to Extended Data Fig.10a
EL#377.4-6 mL39-FLAG IP + Gradient
Rotor: SW41 Ti
Gradient: Sucrose 5-30%
Speed: 158.000xg
Time: 15h

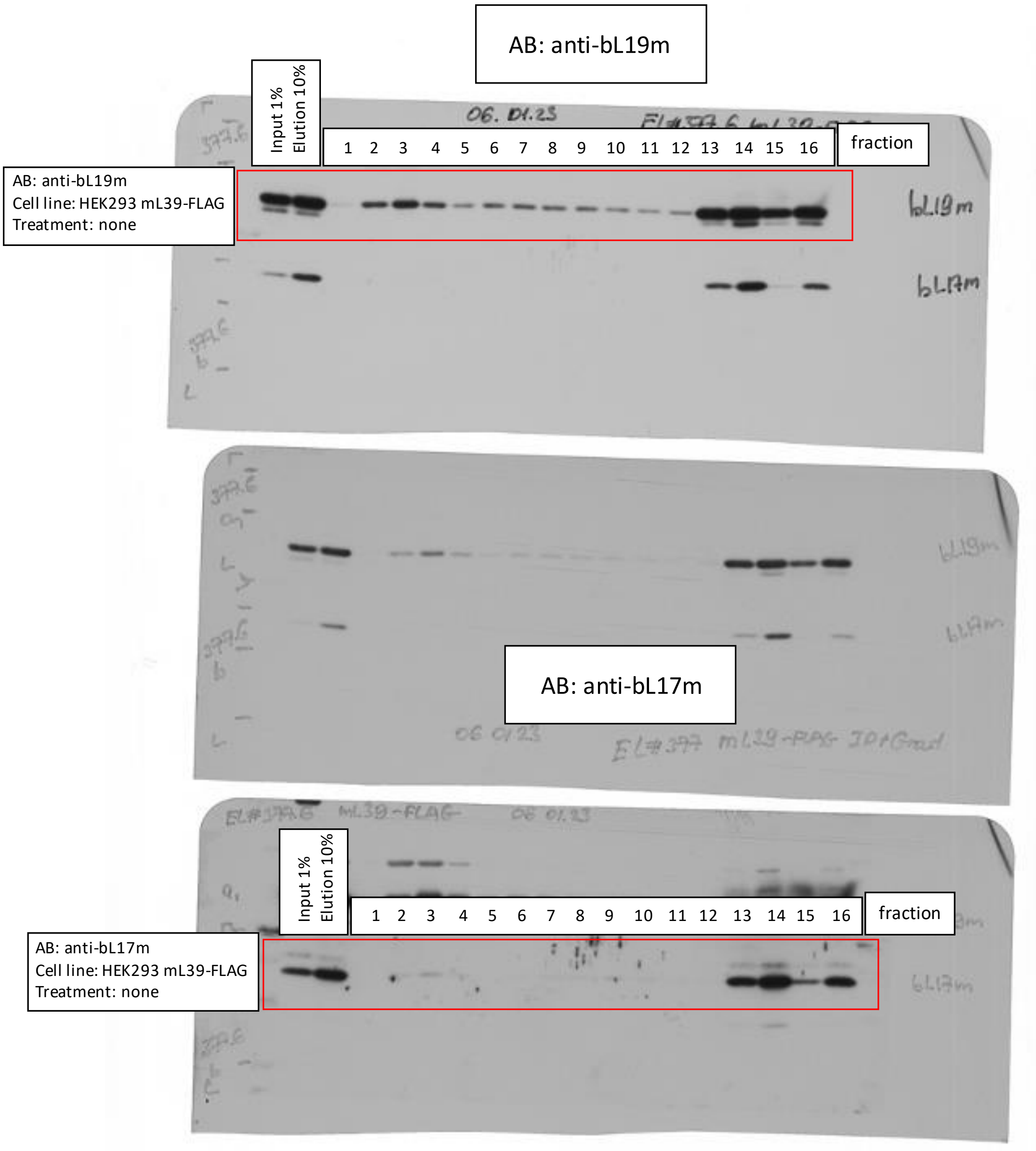

Source Data 6_related to Extended Data Fig.10a
EL#377.4-6 mL39-FLAG IP + Gradient
Rotor: SW41 Ti
Gradient: Sucrose 5-30%
Speed: 158.000xg
Time: 15h

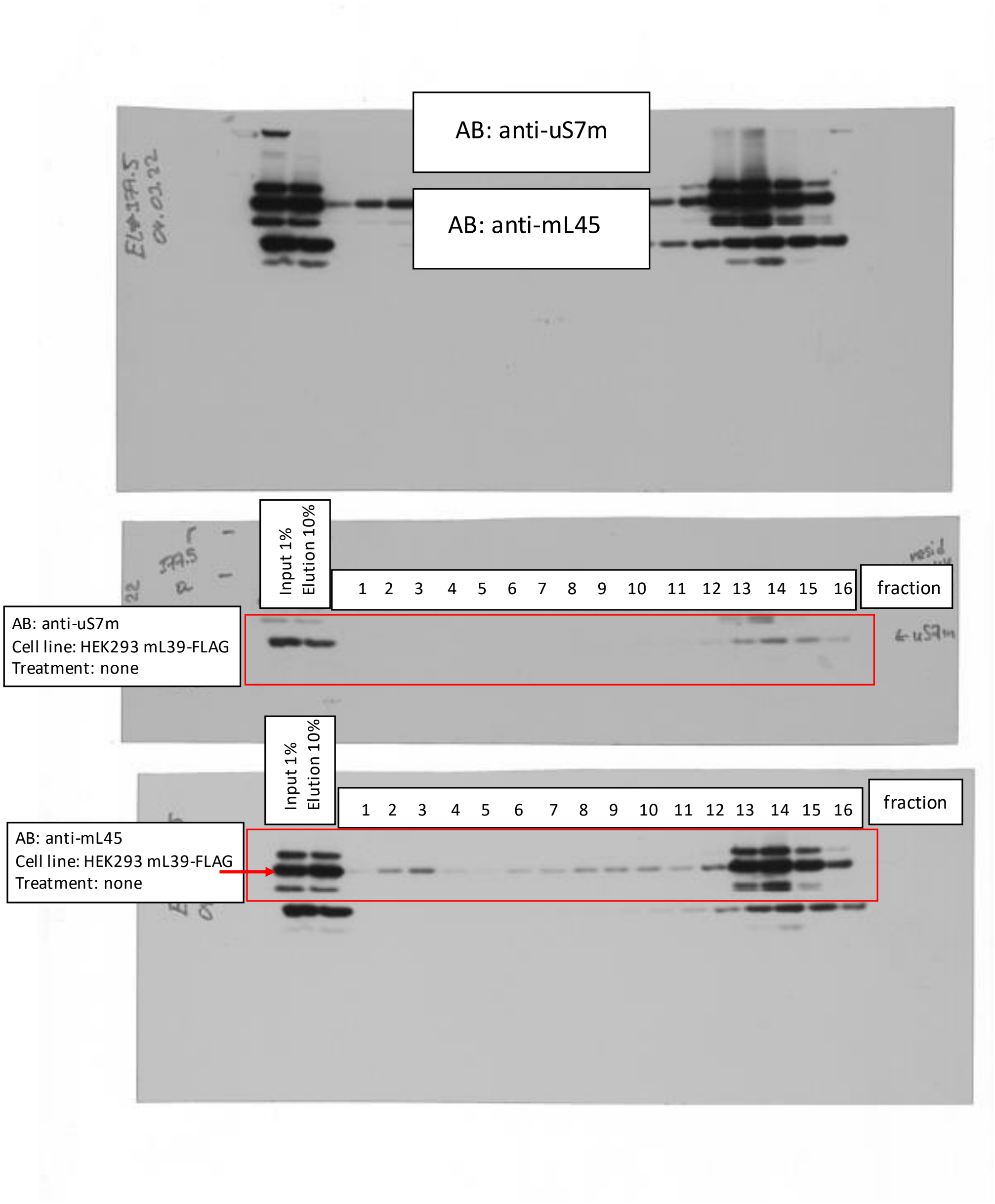

Source Data 6_related to Extended Data Fig.10a
EL#377.4-6 mL39-FLAG IP + Gradient
Rotor: SW41 Ti
Gradient: Sucrose 5-30%
Speed: 158.000xg
Time: 15h

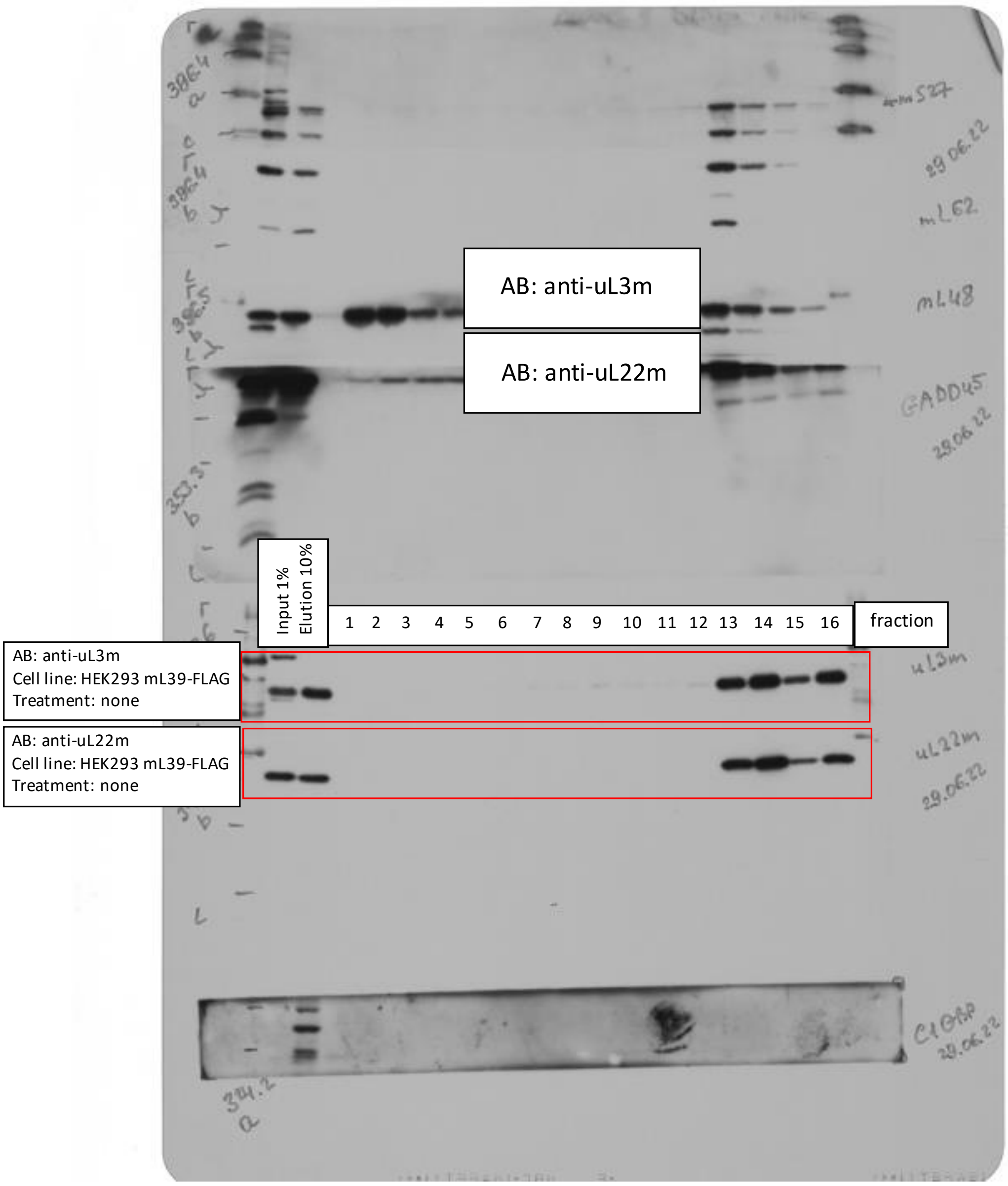

Source Data 6_related to Extended Data Fig.10a
EL#377.4-6 mL39-FLAG IP + Gradient
Rotor: SW41 Ti
Gradient: Sucrose 5-30%
Speed: 158.000xg
Time: 15h

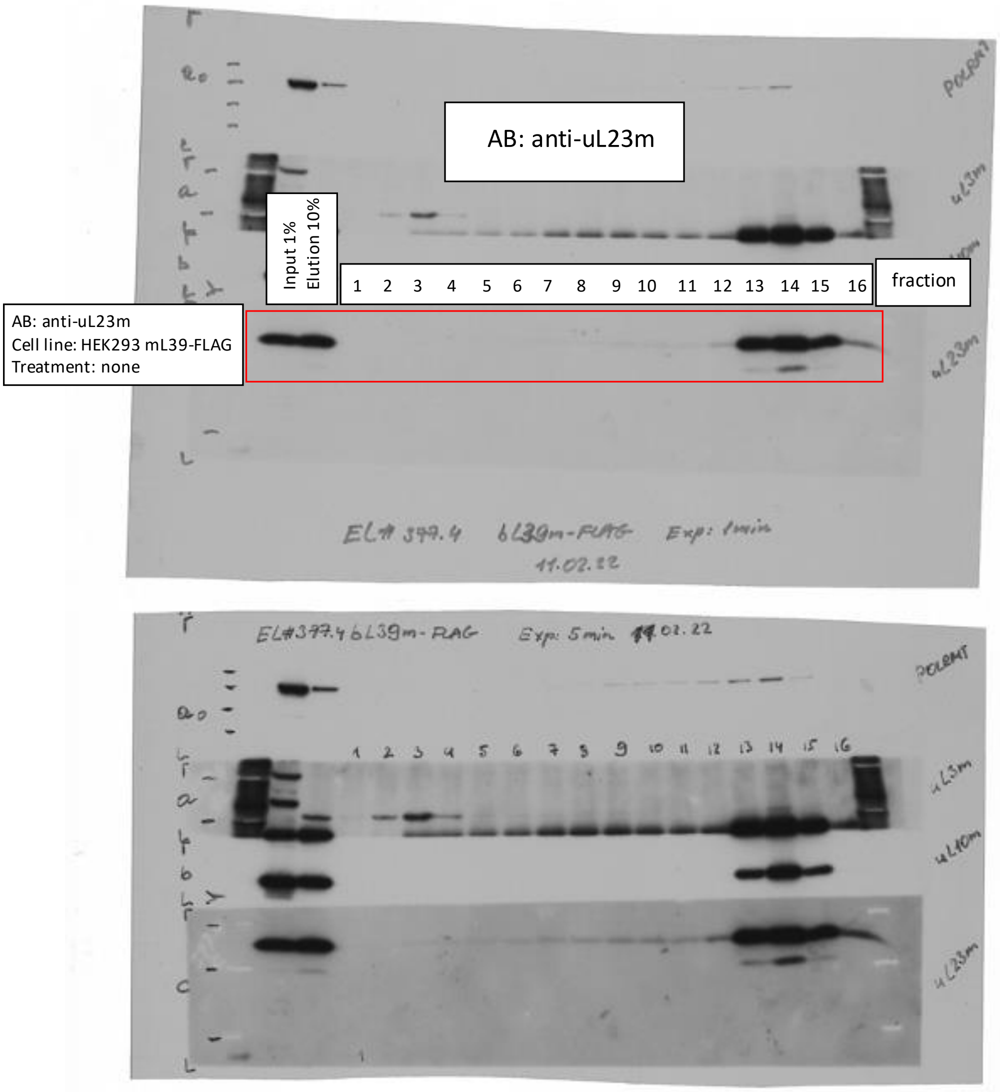

Source Data 6_related to Extended Data Fig.10a
EL#377.4-6 mL39-FLAG IP + Gradient
Rotor: SW41 Ti
Gradient: Sucrose 5-30%
Speed: 158.000xg
Time: 15h

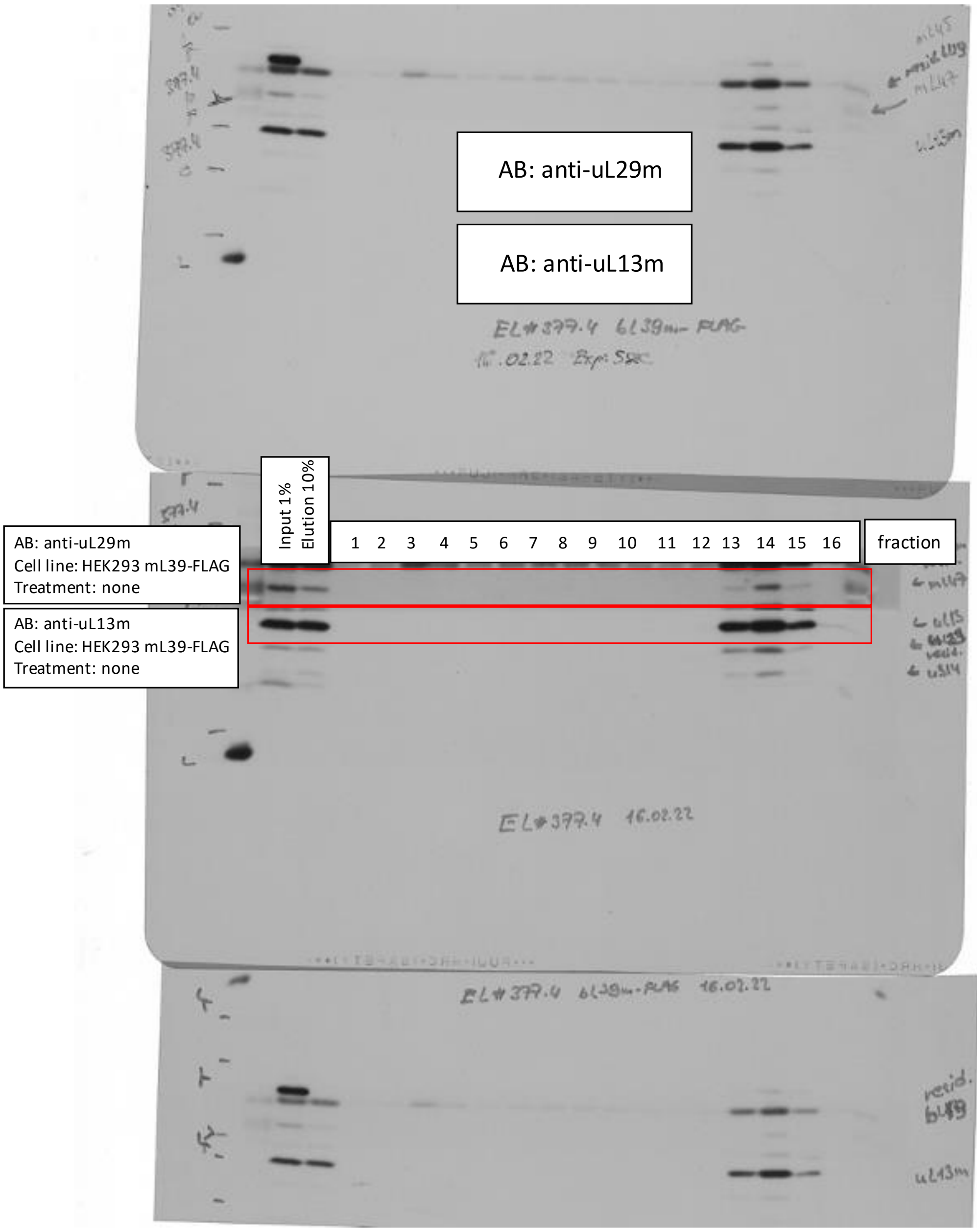

Source Data 6_related to Extended Data Fig.10a
EL#377.4-6 mL39-FLAG IP + Gradient
Rotor: SW41 Ti
Gradient: Sucrose 5-30%
Speed: 158.000xg
Time: 15h

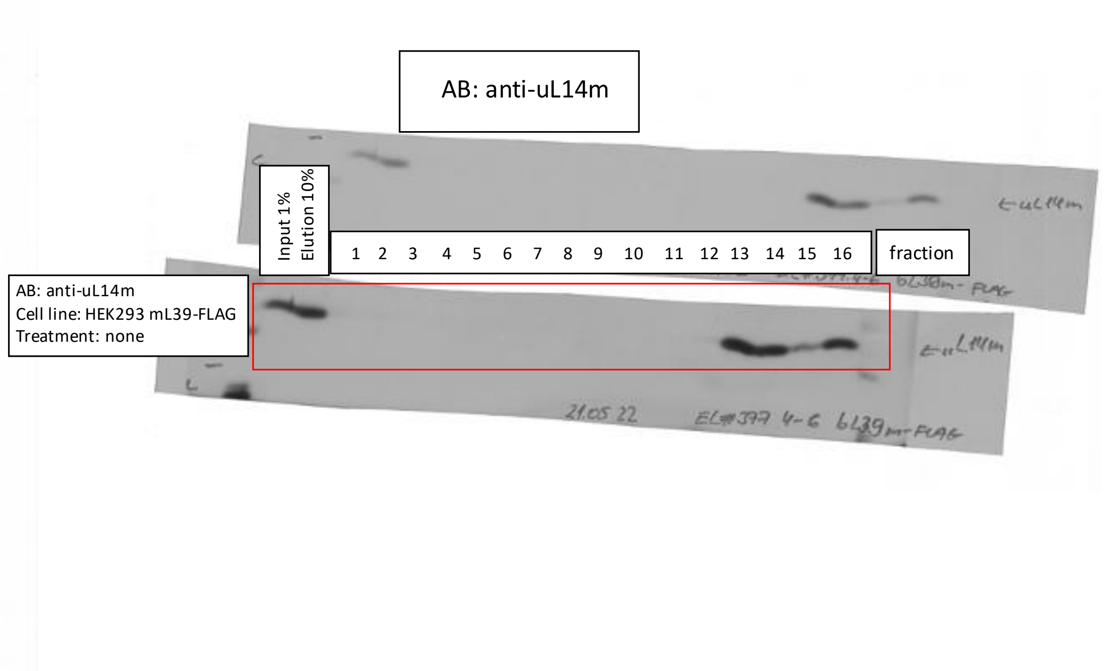

Source Data 6_related to Extended Data Fig.10b
EL#363 HEK293 WT + Ethidium Bromide treatment + Gradient
EL#379 HEK293 WT + Ethidium Bromide treatment + Gradient

Rotor: SW41 Ti
Gradient: Sucrose 5-30%
Speed: 158.000xg
Time: 15h

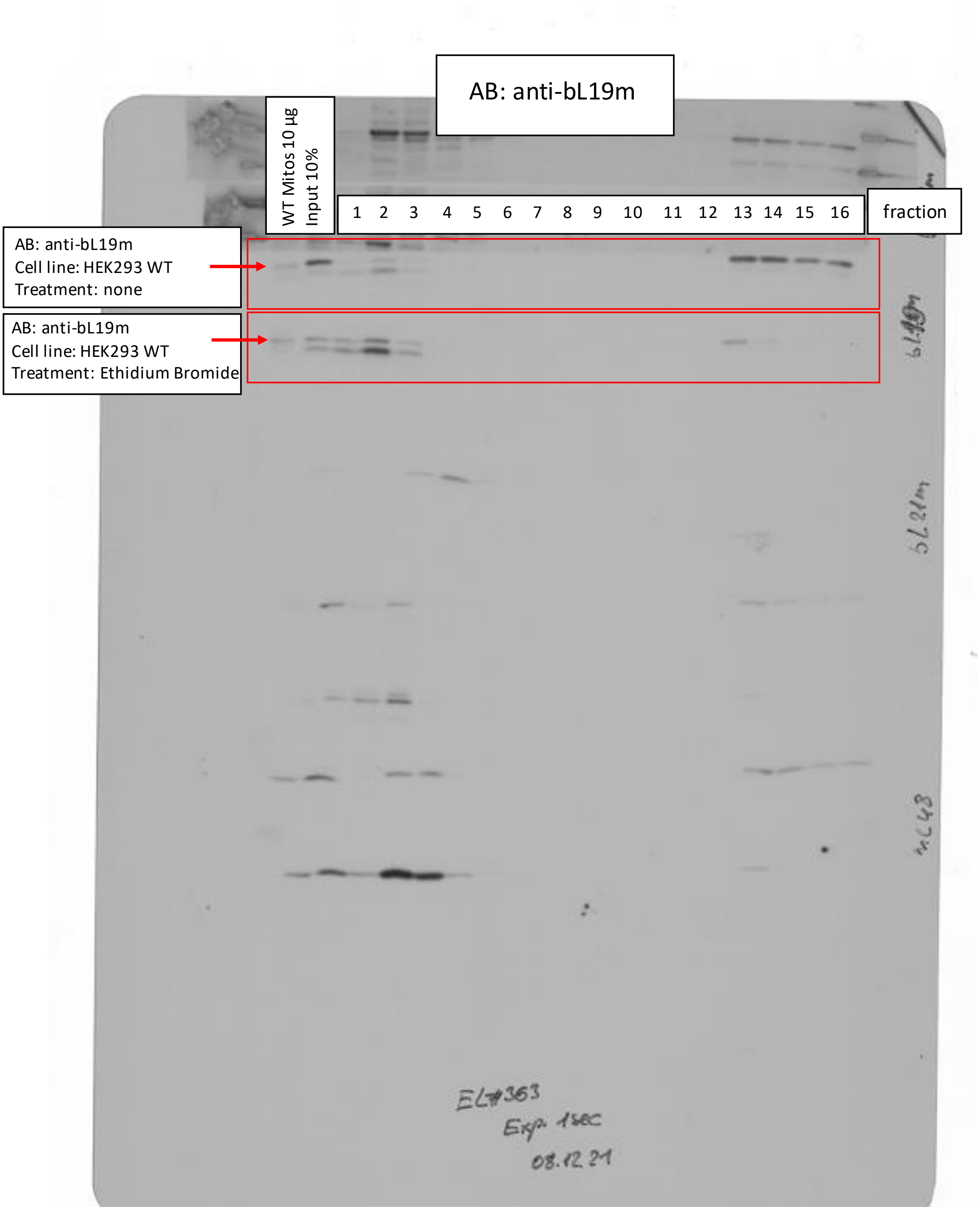

Source Data 6_related to Extended Data Fig.10b
EL#363 HEK293 WT + Ethidium Bromide treatment + Gradient
EL#379 HEK293 WT + Ethidium Bromide treatment + Gradient

Rotor: SW41 Ti
Gradient: Sucrose 5-30%
Speed: 158.000xg
Time: 15h

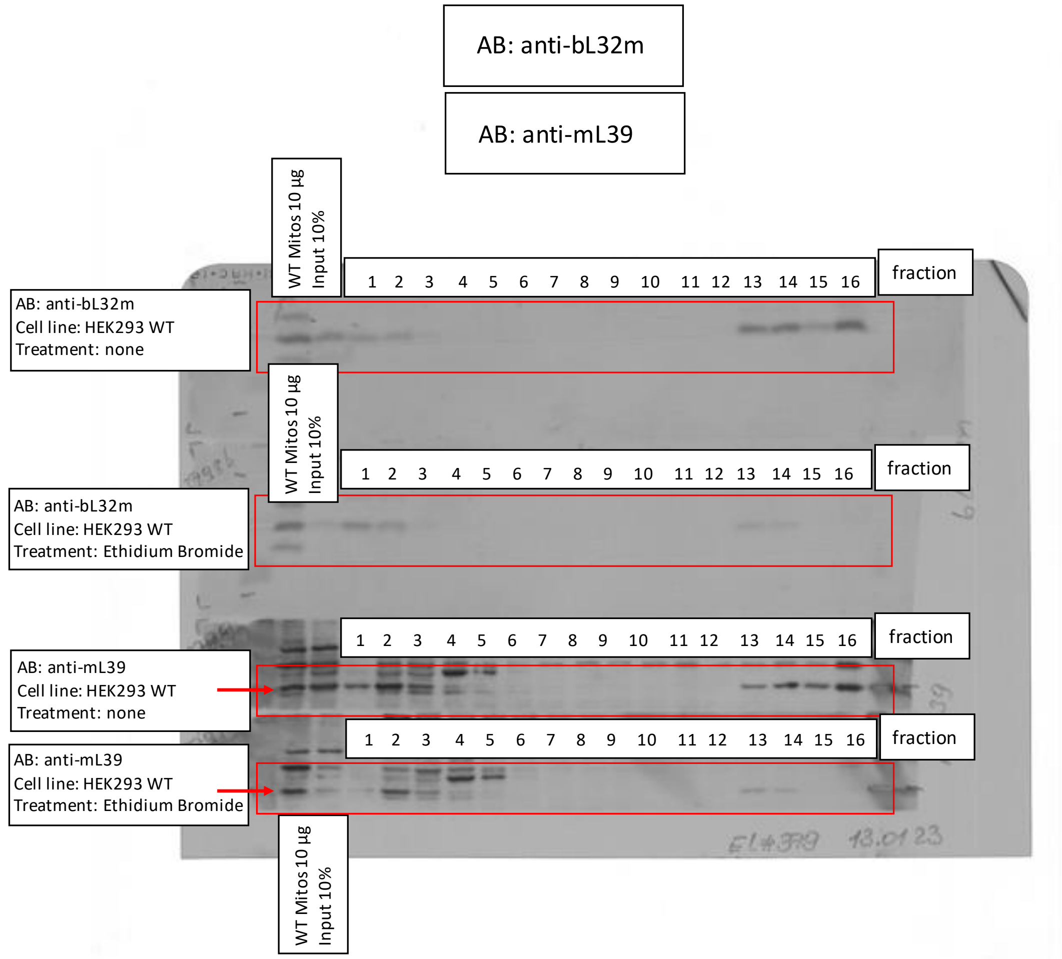

Source Data 6_related to Extended Data Fig.10b
EL#363 HEK293 WT + Ethidium Bromide treatment + Gradient
EL#379 HEK293 WT + Ethidium Bromide treatment + Gradient

Rotor: SW41 Ti
Gradient: Sucrose 5-30%
Speed: 158.000xg
Time: 15h

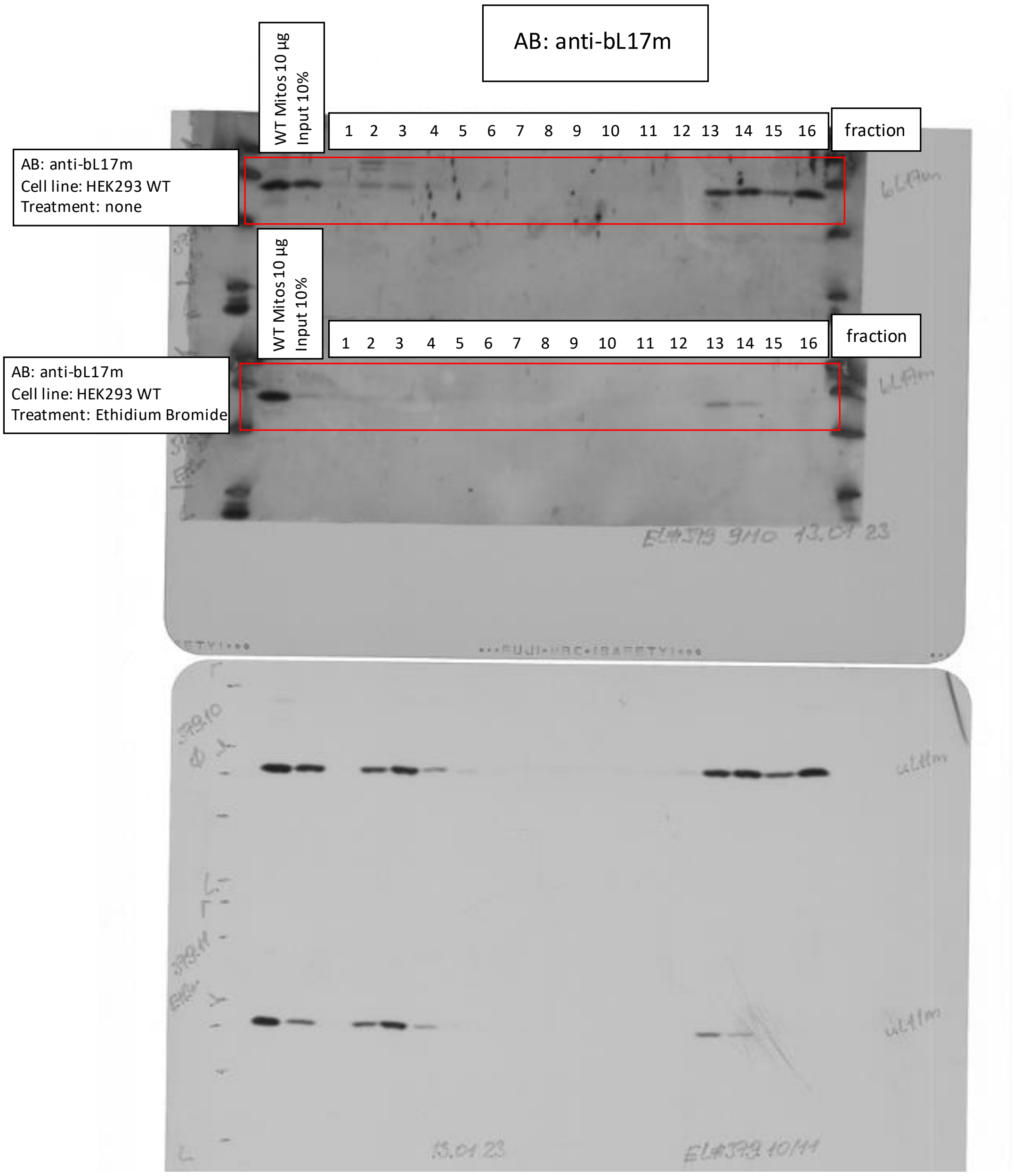

Source Data 6_related to Extended Data Fig.10b
EL#363 HEK293 WT + Ethidium Bromide treatment + Gradient
EL#379 HEK293 WT + Ethidium Bromide treatment + Gradient

Rotor: SW41 Ti
Gradient: Sucrose 5-30%
Speed: 158.000xg
Time: 15h

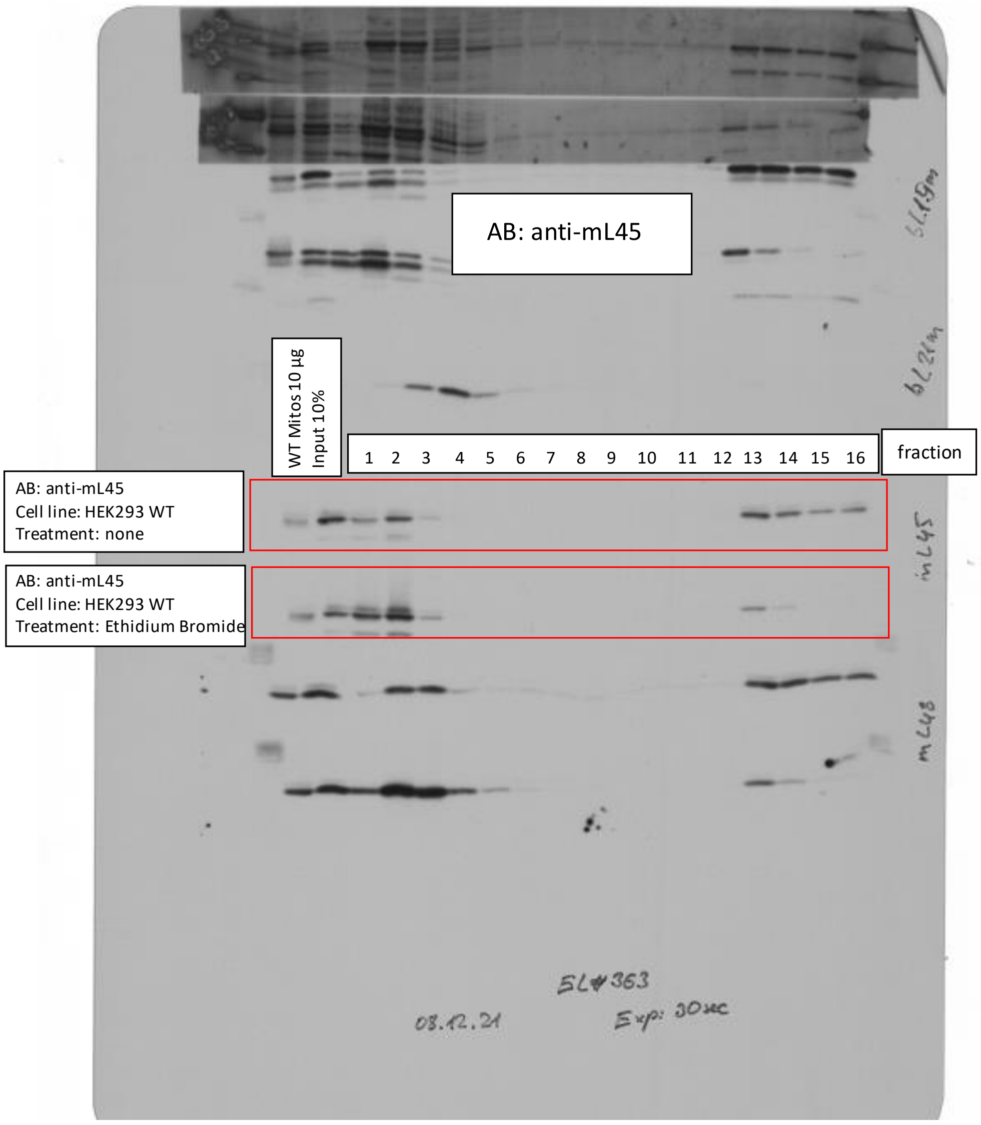

Source Data 6_related to Extended Data Fig.10c
EL#420 mL45 KO + Gradient
EL#406 mL45 KO + Gradient
Rotor: SW41 Ti
Gradient: Sucrose 5-30%
Speed: 158.000xg
Time: 15h

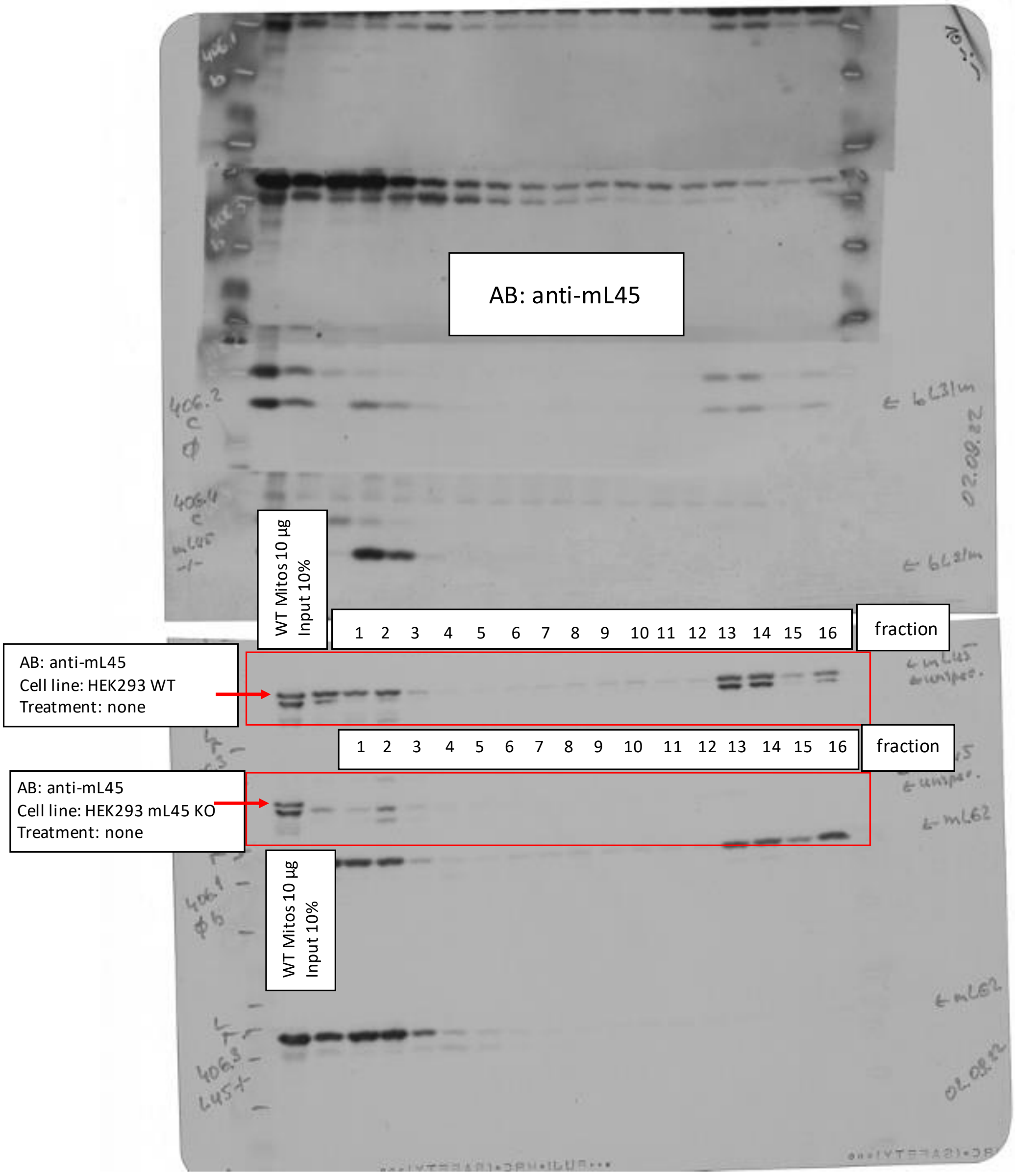

Source Data 6_related to Extended Data Fig.10c
EL#420 mL45 KO + Gradient
EL#406 mL45 KO + Gradient
Rotor: SW41 Ti
Gradient: Sucrose 5-30%
Speed: 158.000xg
Time: 15h

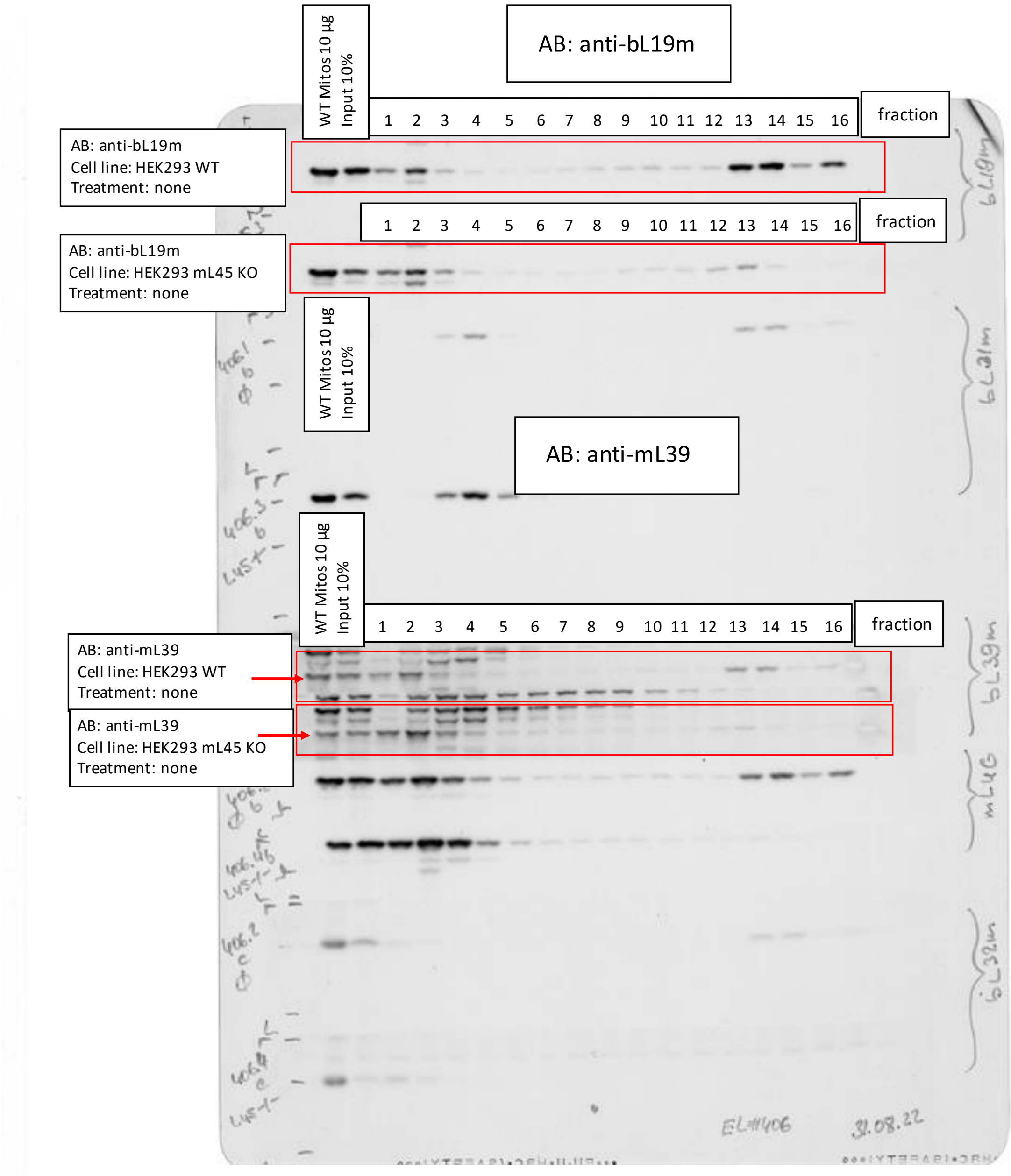

Source Data 6_related to Extended Data Fig.10c
EL#420 mL45 KO + Gradient
EL#406 mL45 KO + Gradient
Rotor: SW41 Ti
Gradient: Sucrose 5-30%
Speed: 158.000xg
Time: 15h

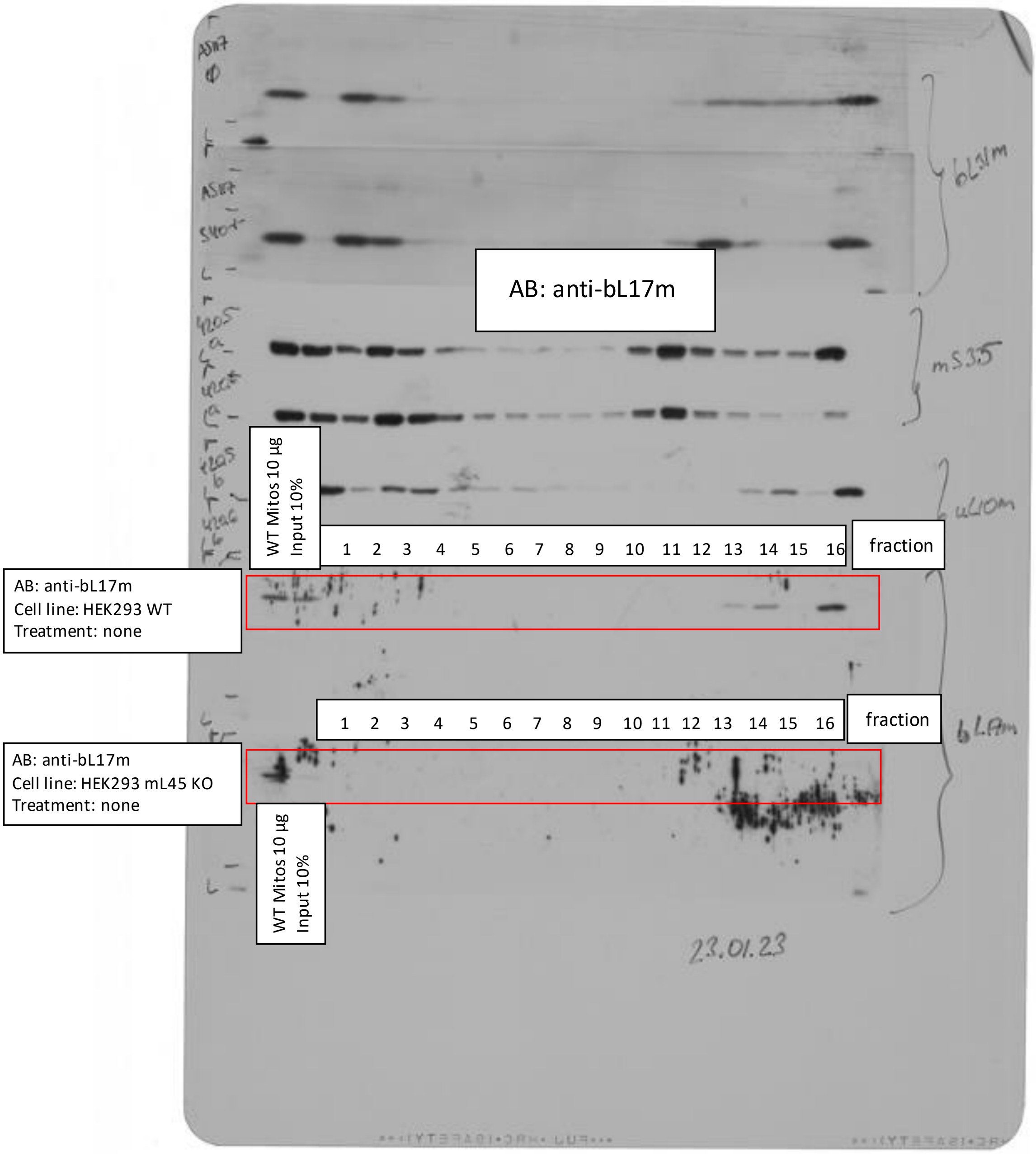

Source Data 6_related to Extended Data Fig.10c
EL#420 mL45 KO + Gradient
EL#406 mL45 KO + Gradient
Rotor: SW41 Ti
Gradient: Sucrose 5-30%
Speed: 158.000xg
Time: 15h

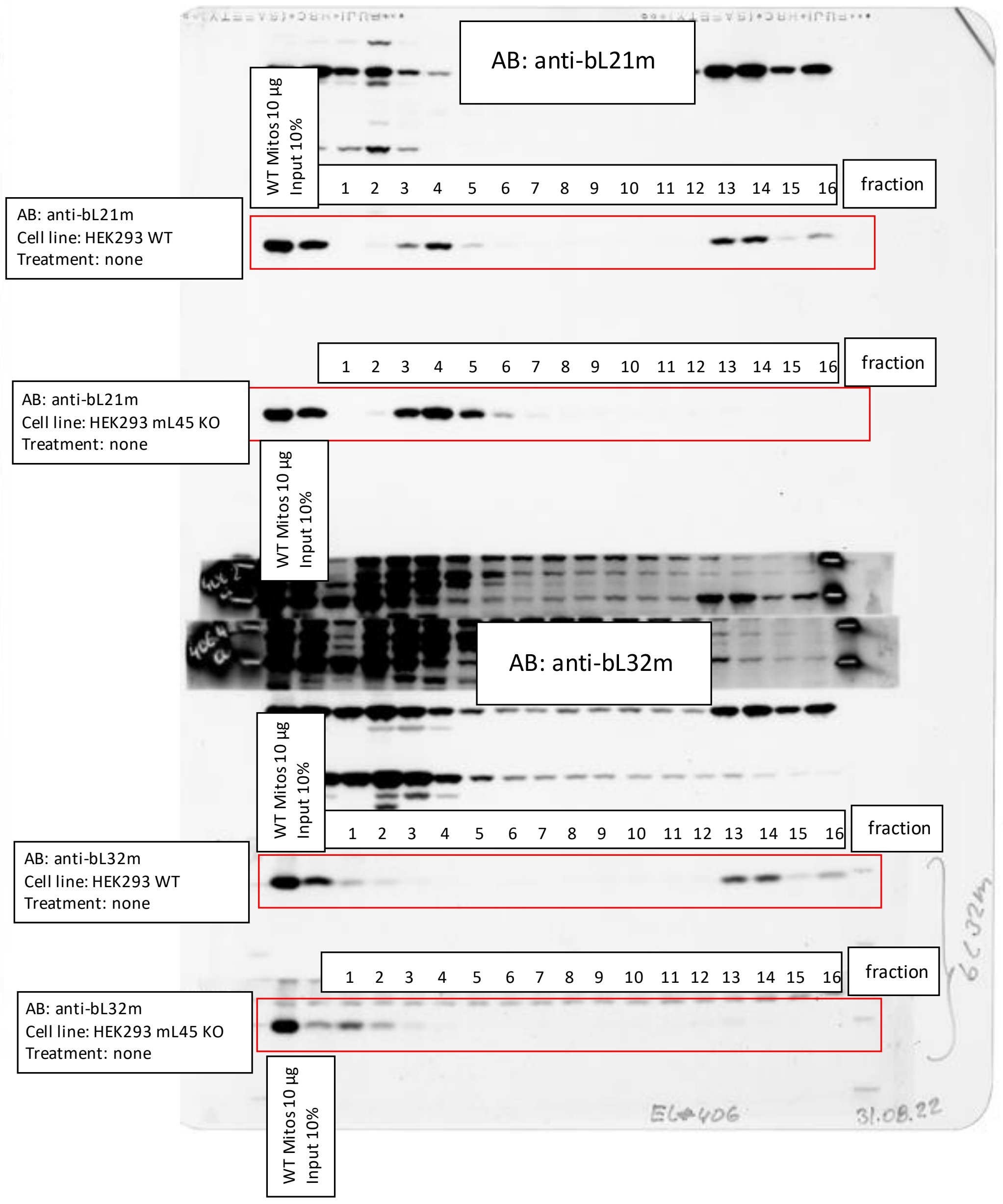

Source Data 6_related to Extended Data Fig.10c
EL#420 mL45 KO + Gradient
EL#406 mL45 KO + Gradient
Rotor: SW41 Ti
Gradient: Sucrose 5-30%
Speed: 158.000xg
Time: 15h

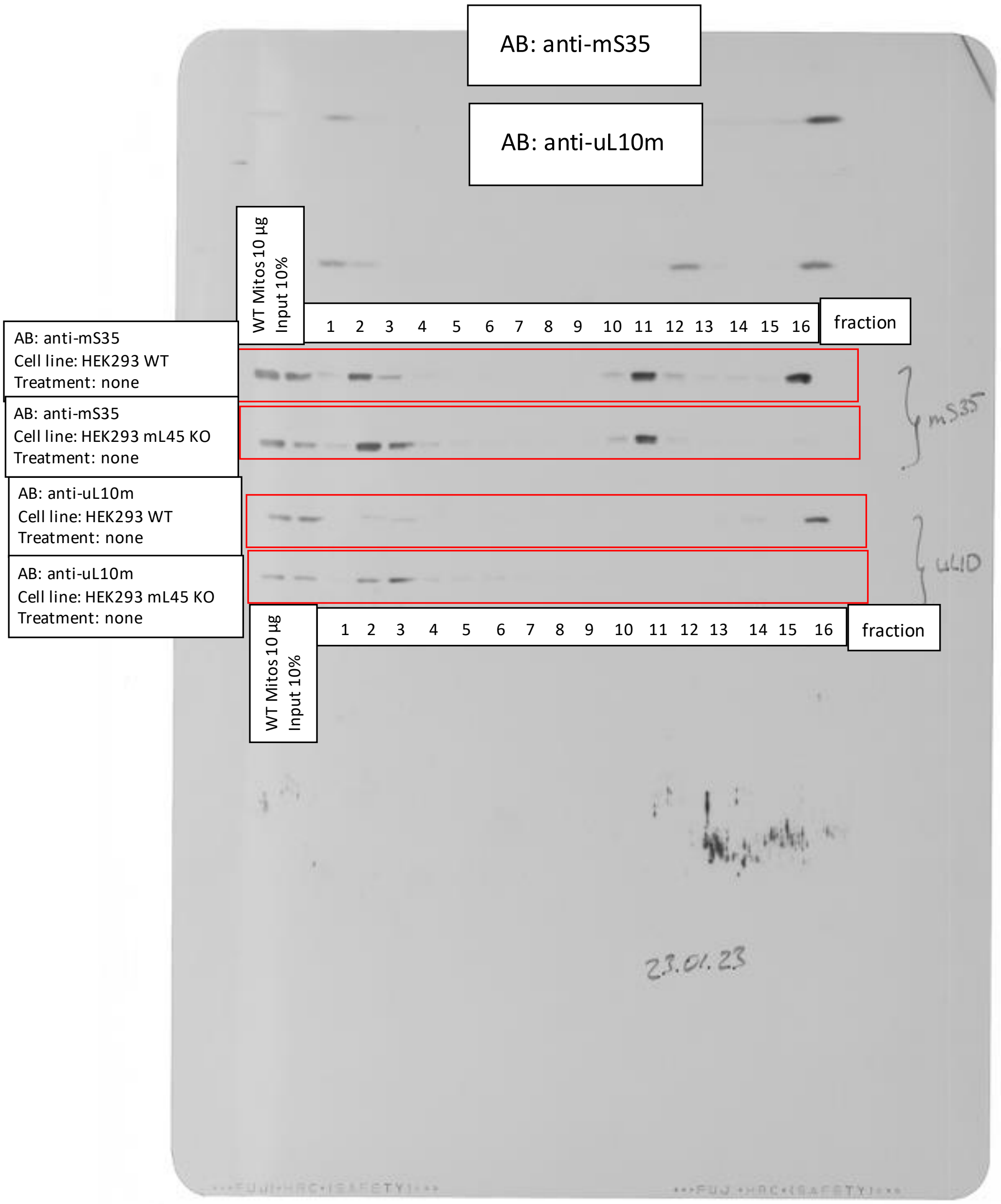

Source Data 6_related to Extended Data Fig.10c
EL#420 mL45 KO + Gradient
EL#406 mL45 KO + Gradient
Rotor: SW41 Ti
Gradient: Sucrose 5-30%
Speed: 158.000xg
Time: 15h

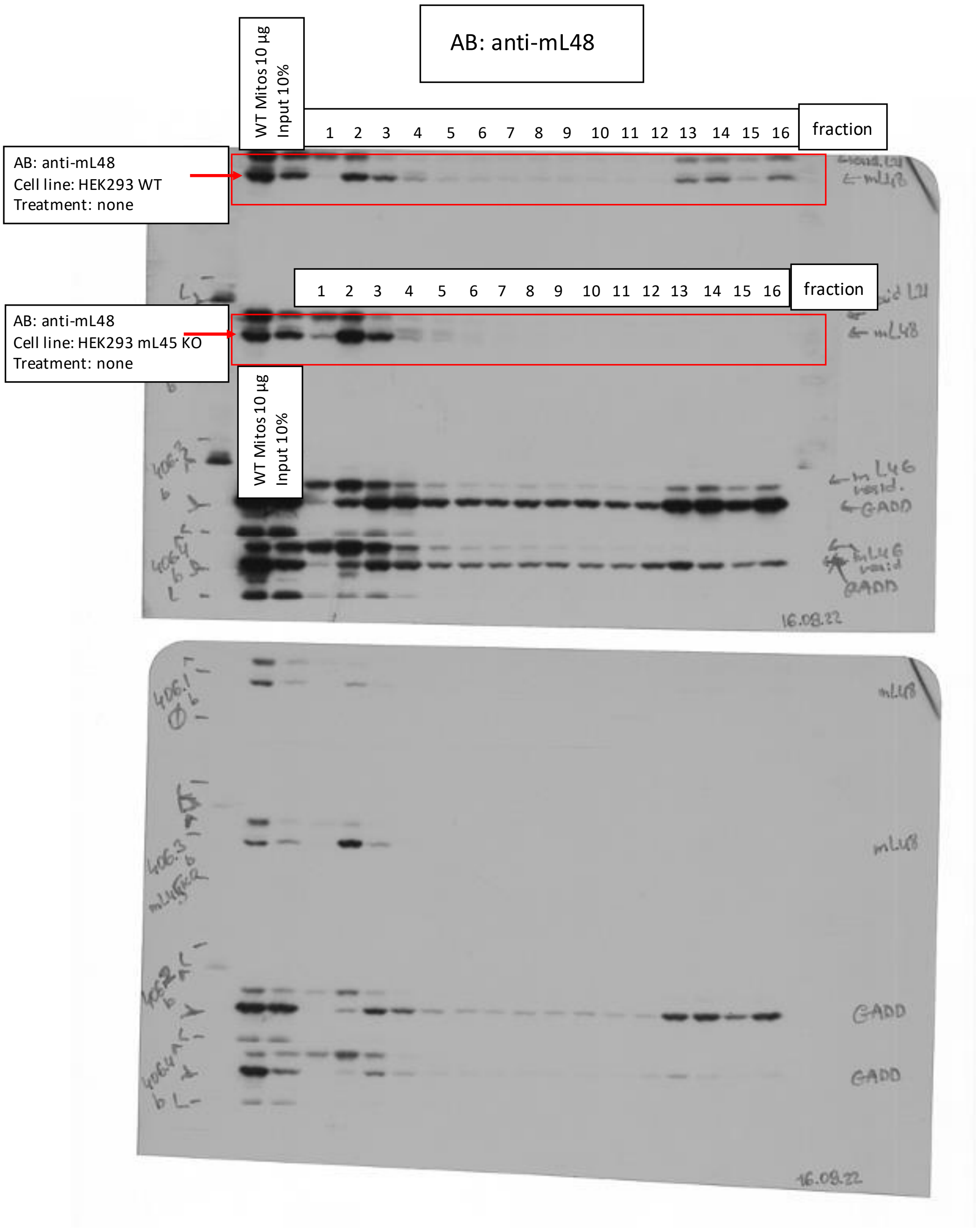

Source Data 6_related to Extended Data Fig.10c
EL#420 mL45 KO + Gradient
EL#406 mL45 KO + Gradient
Rotor: SW41 Ti
Gradient: Sucrose 5-30%
Speed: 158.000xg
Time: 15h

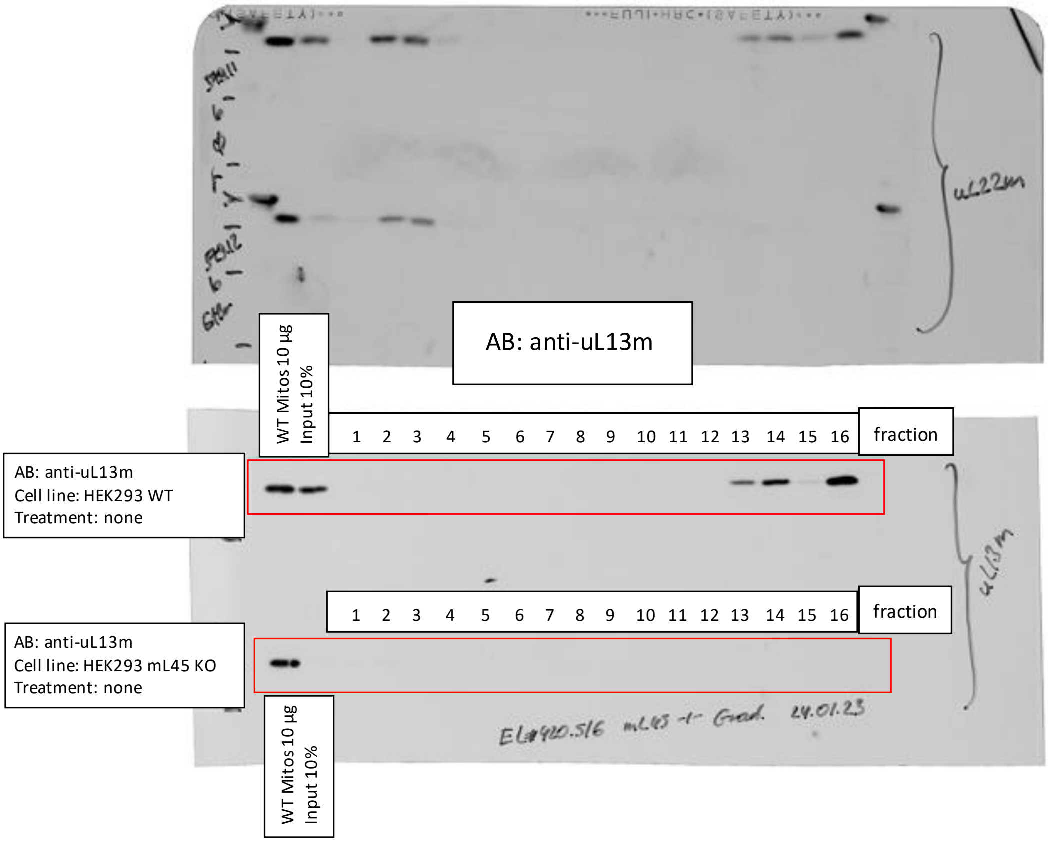

Source Data 6_related to Extended Data Fig.10d
EL#363 HEK293 WT + Ethidium Bromide treatment + Gradient
EL#379 HEK293 WT + Ethidium Bromide treatment + Gradient

Rotor: SW41 Ti
Gradient: Sucrose 5-30%
Speed: 158.000xg
Time: 15h

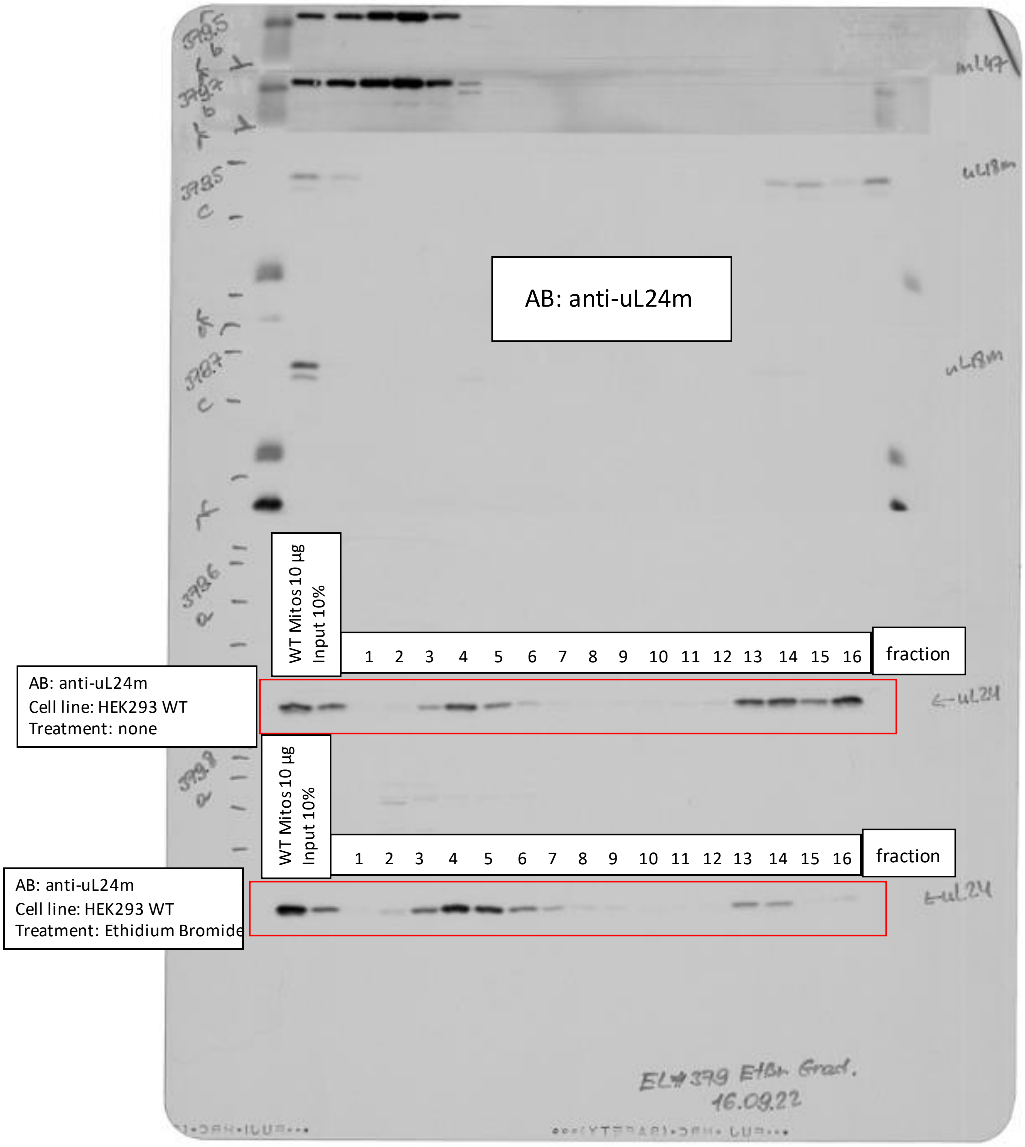

Source Data 6_related to Extended Data Fig.10d
EL#363 HEK293 WT + Ethidium Bromide treatment + Gradient
EL#379 HEK293 WT + Ethidium Bromide treatment + Gradient

Rotor: SW41 Ti
Gradient: Sucrose 5-30%
Speed: 158.000xg
Time: 15h

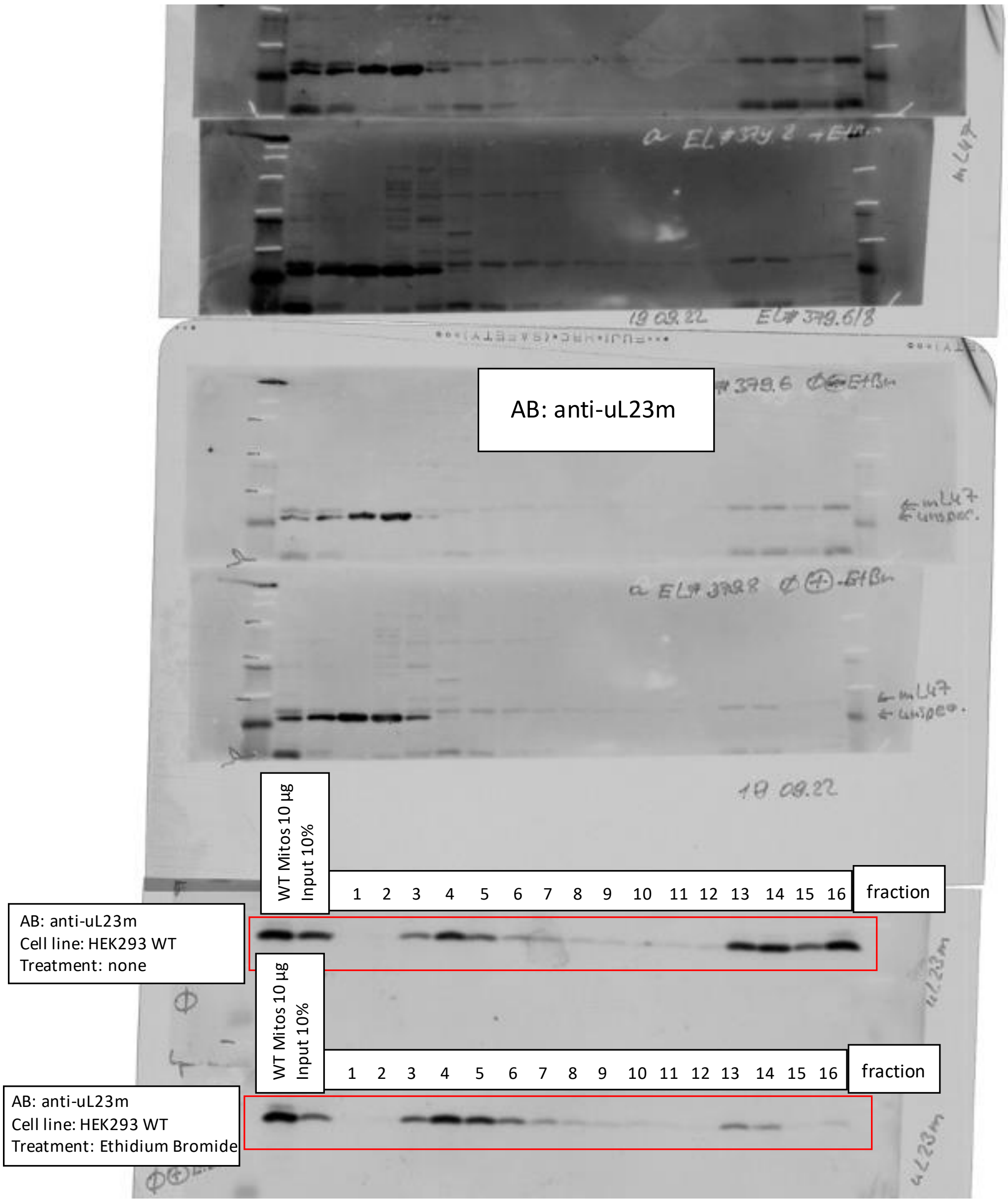

Source Data 6_related to Extended Data Fig.10d
EL#363 HEK293 WT + Ethidium Bromide treatment + Gradient
EL#379 HEK293 WT + Ethidium Bromide treatment + Gradient

Rotor: SW41 Ti
Gradient: Sucrose 5-30%
Speed: 158.000xg
Time: 15h

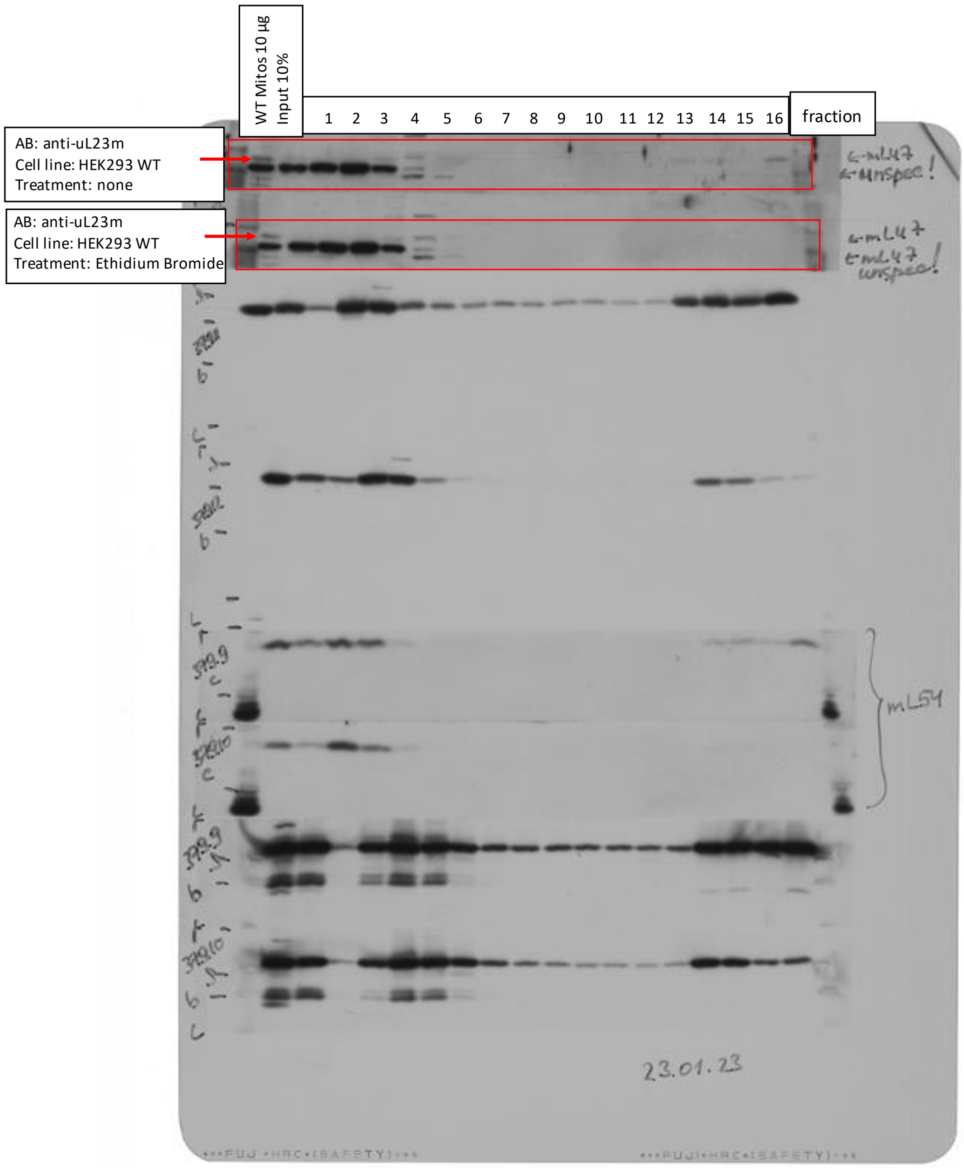

Source Data 6_related to Extended Data Fig.10e
EL#363 HEK293 WT + Ethidium Bromide treatment + Gradient
EL#379 HEK293 WT + Ethidium Bromide treatment + Gradient

Rotor: SW41 Ti
Gradient: Sucrose 5-30%
Speed: 158.000xg
Time: 15h

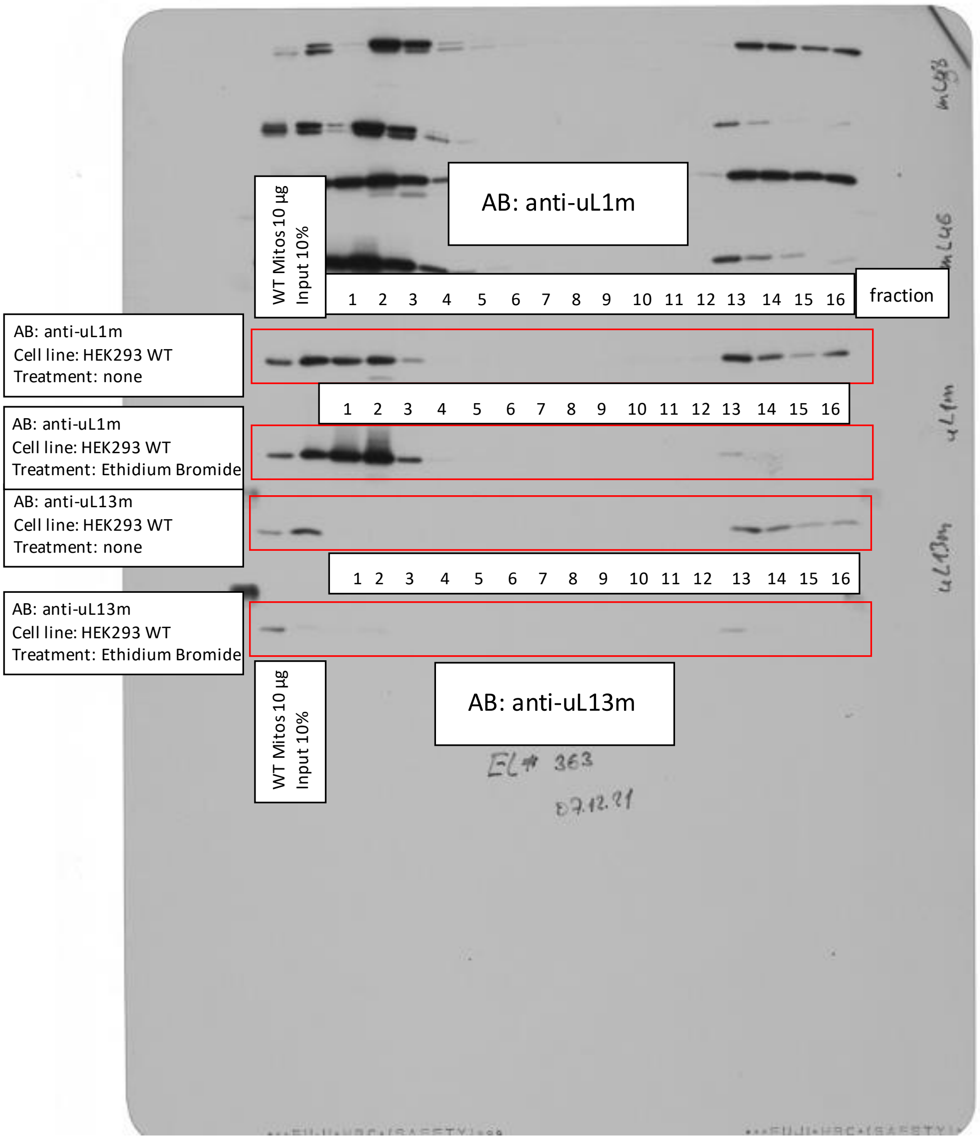

Source Data 6_related to Extended Data Fig.10e
EL#363 HEK293 WT + Ethidium Bromide treatment + Gradient
EL#379 HEK293 WT + Ethidium Bromide treatment + Gradient

Rotor: SW41 Ti
Gradient: Sucrose 5-30%
Speed: 158.000xg
Time: 15h

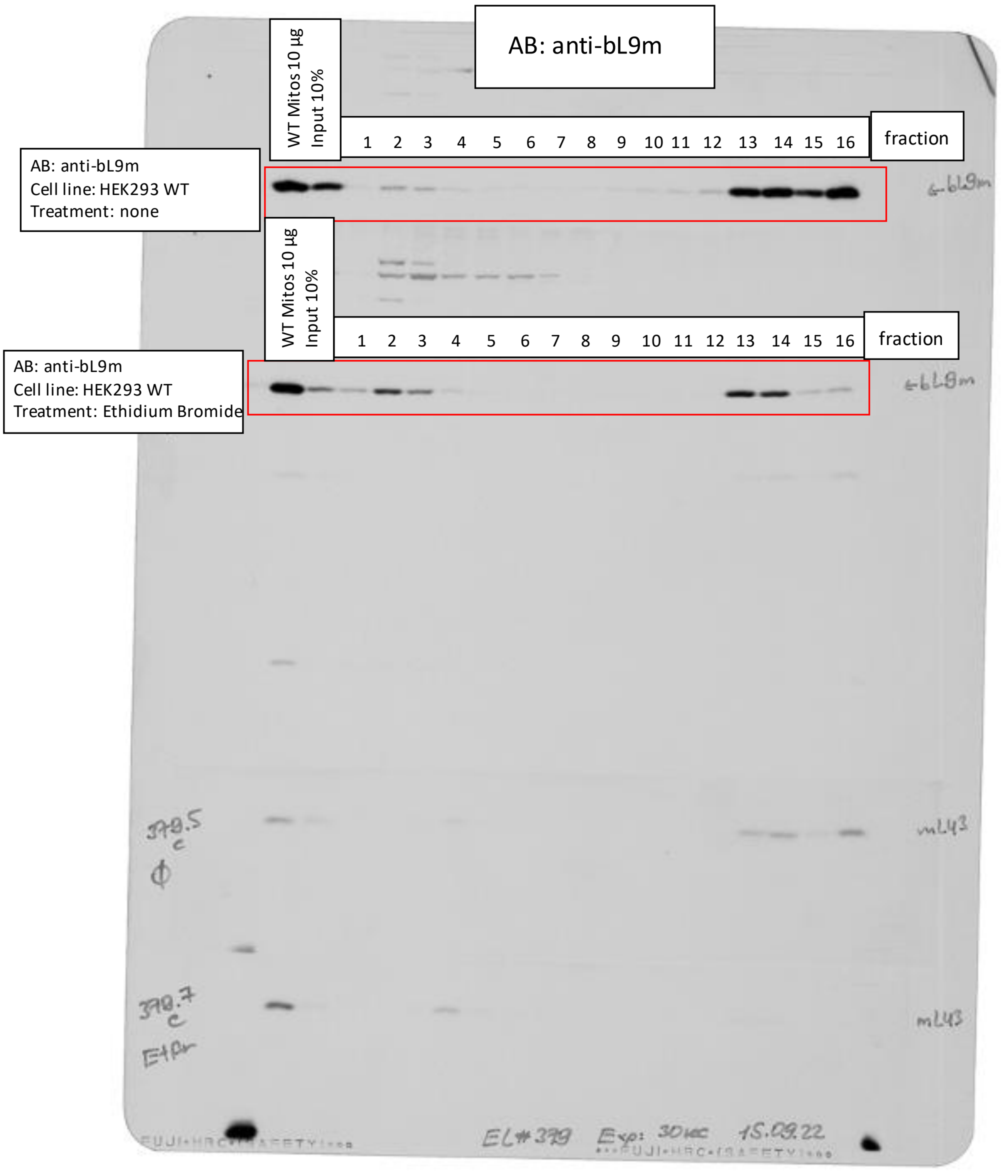

Source Data 6_related to Extended Data Fig.10e
EL#363 HEK293 WT + Ethidium Bromide treatment + Gradient
EL#379 HEK293 WT + Ethidium Bromide treatment + Gradient

Rotor: SW41 Ti
Gradient: Sucrose 5-30%
Speed: 158.000xg
Time: 15h

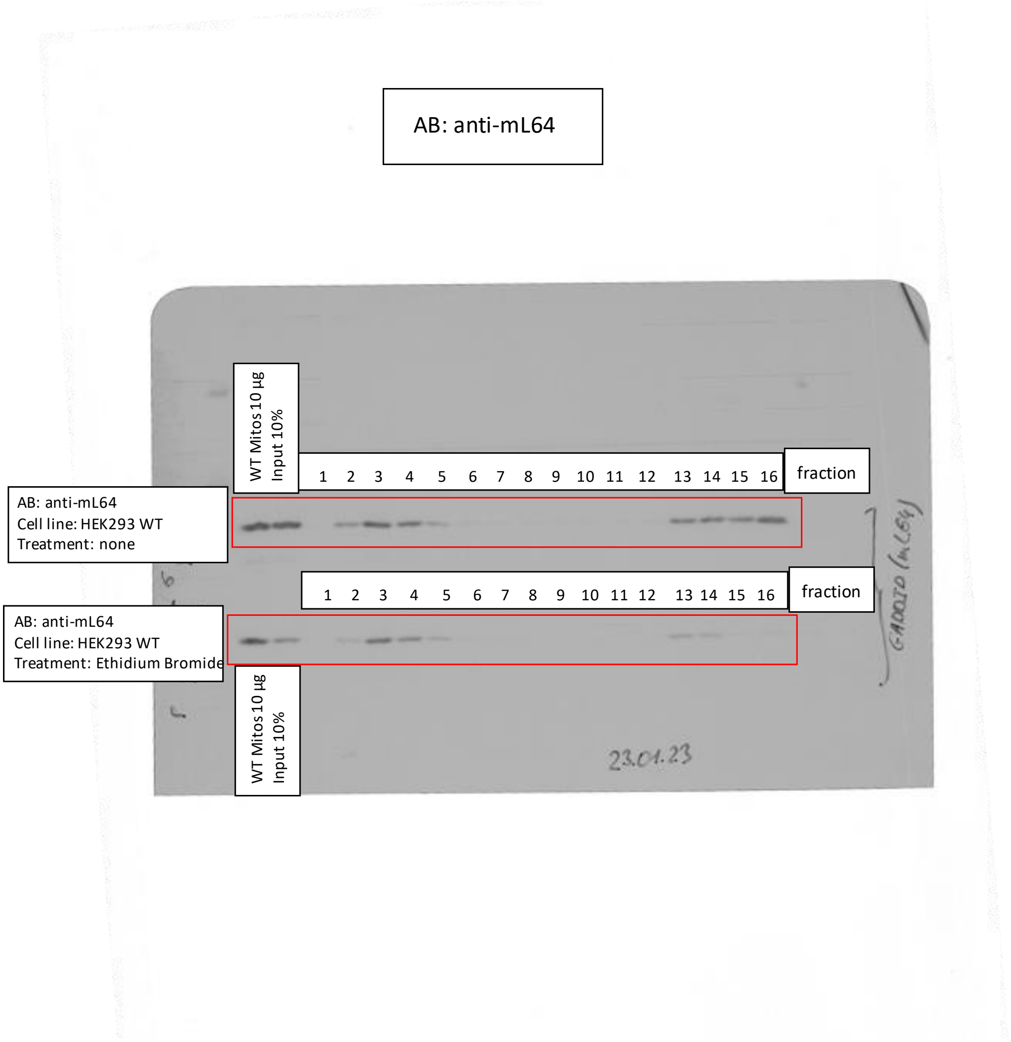

Source Data 6_related to Extended Data Fig.10e
EL#363 HEK293 WT + Ethidium Bromide treatment + Gradient
EL#379 HEK293 WT + Ethidium Bromide treatment + Gradient

Rotor: SW41 Ti
Gradient: Sucrose 5-30%
Speed: 158.000xg
Time: 15h

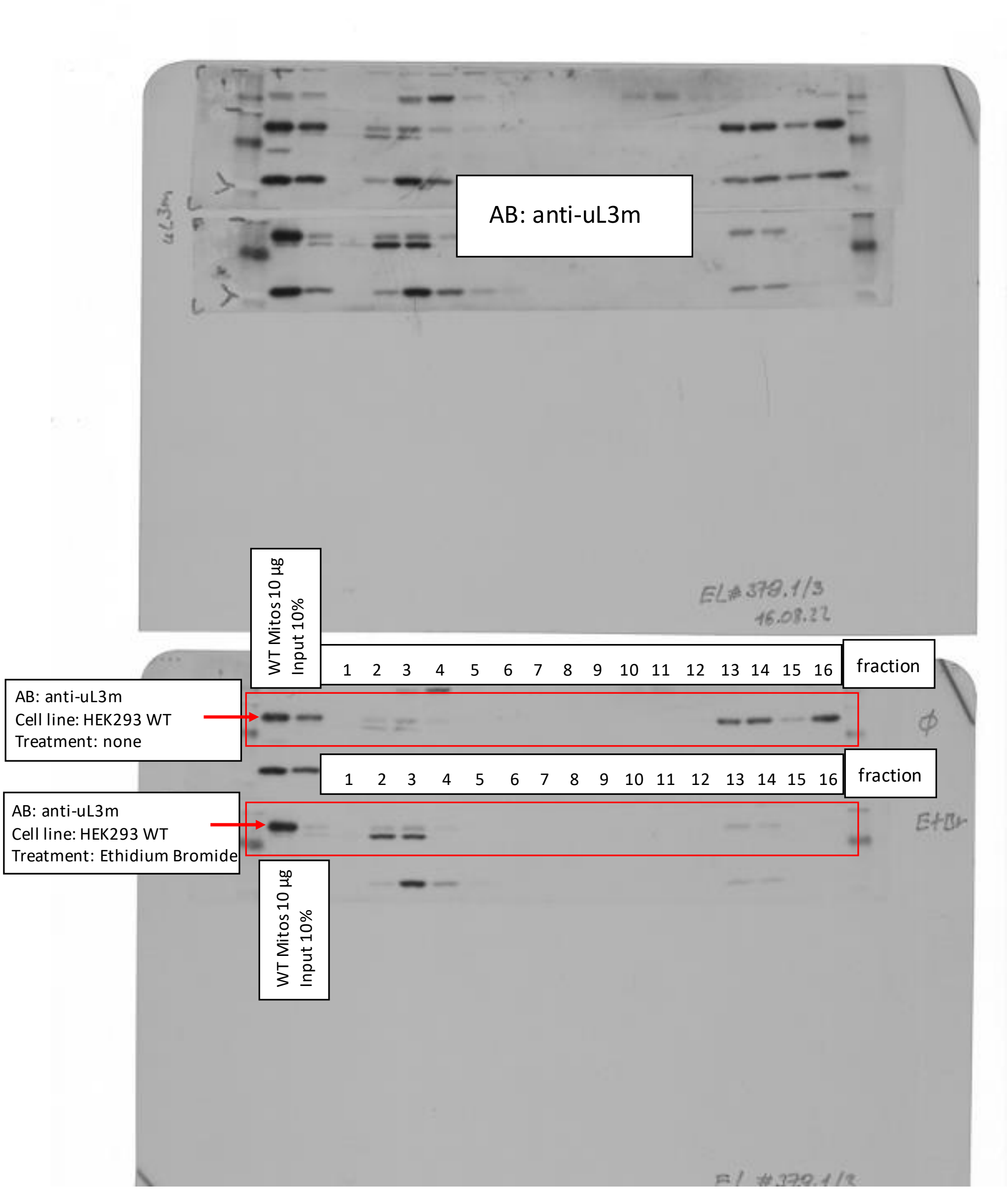

Source Data 6_related to Extended Data Fig.10e
EL#363 HEK293 WT + Ethidium Bromide treatment + Gradient
EL#379 HEK293 WT + Ethidium Bromide treatment + Gradient

Rotor: SW41 Ti
Gradient: Sucrose 5-30%
Speed: 158.000xg
Time: 15h

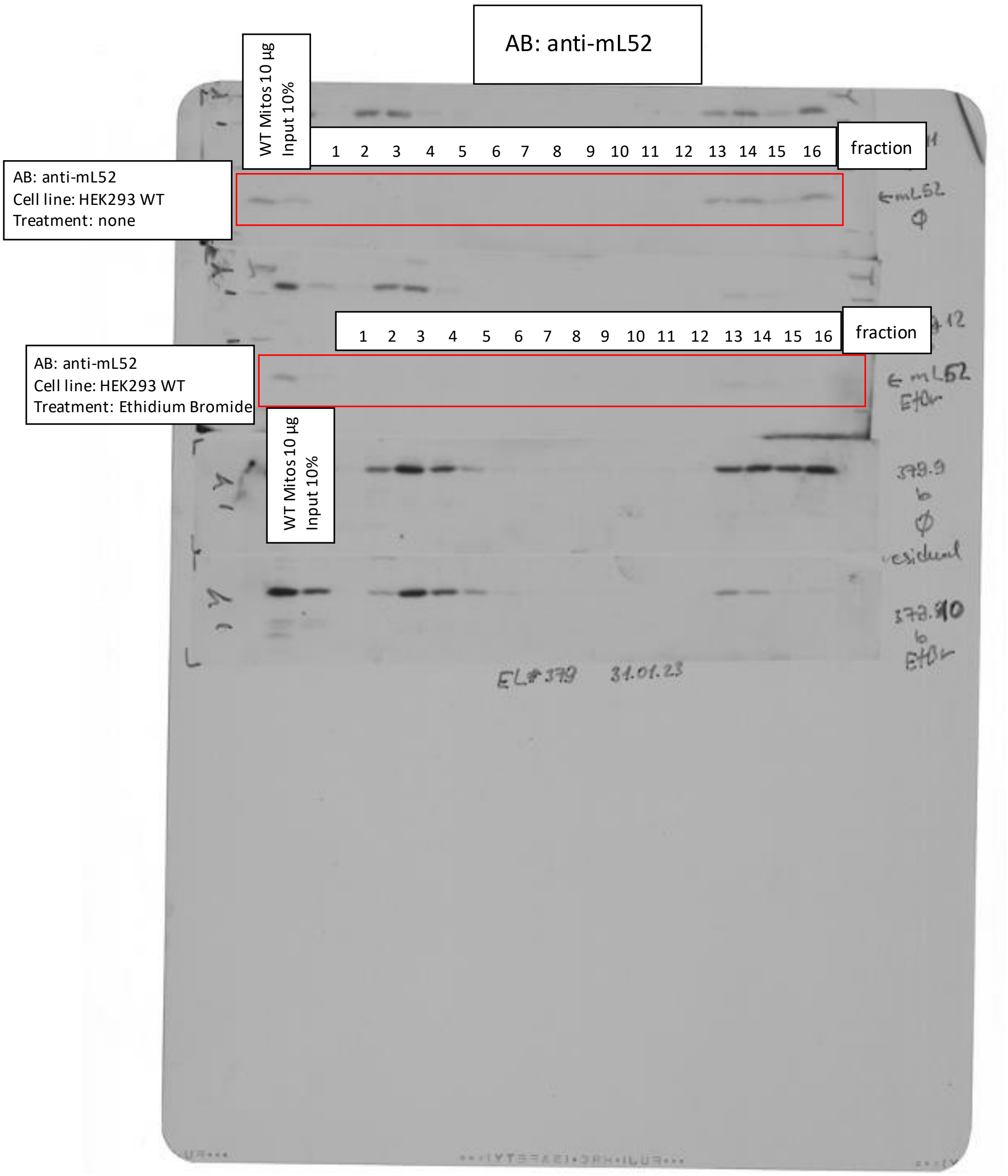

Source Data 6_related to Extended Data Fig.10e
EL#363 HEK293 WT + Ethidium Bromide treatment + Gradient
EL#379 HEK293 WT + Ethidium Bromide treatment + Gradient

Rotor: SW41 Ti
Gradient: Sucrose 5-30%
Speed: 158.000xg
Time: 15h

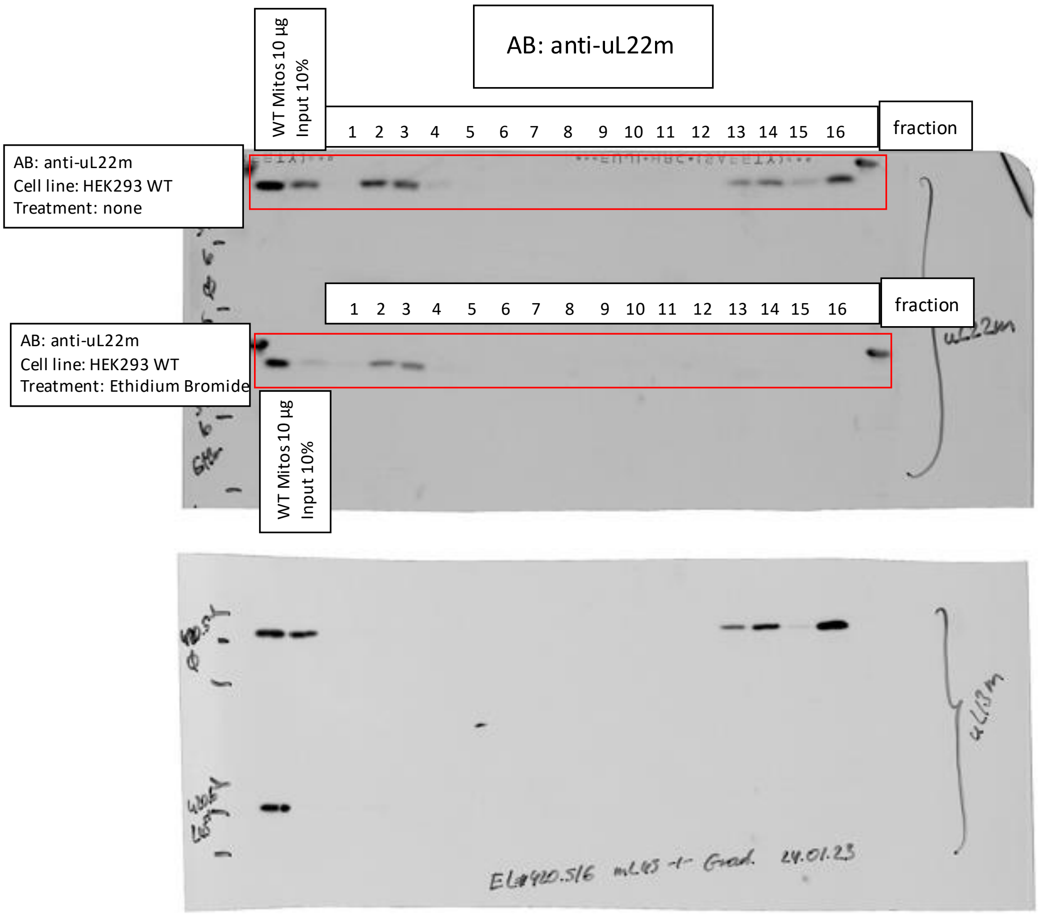

Supplement: Supplementary file 27 — Unprocessed blots. [file 41594_2024_1356_MOESM27_ESM.pdf]
